# Supplementary material for: Identification of Disease-Relevant, Sex-Based Proteomic Differences in iPSC-Derived Vascular Smooth Muscle Cells
Source: Int J Mol Sci. 2024 Dec 29;26(1):187. doi: 10.3390/ijms26010187 (PMC11719605; doi:10.3390/ijms26010187)
Supplement: Supplementary file 1 [file ijms-26-00187-s001.zip › ijms-3360661-supplementary.pdf]

Supplementary Figure S1

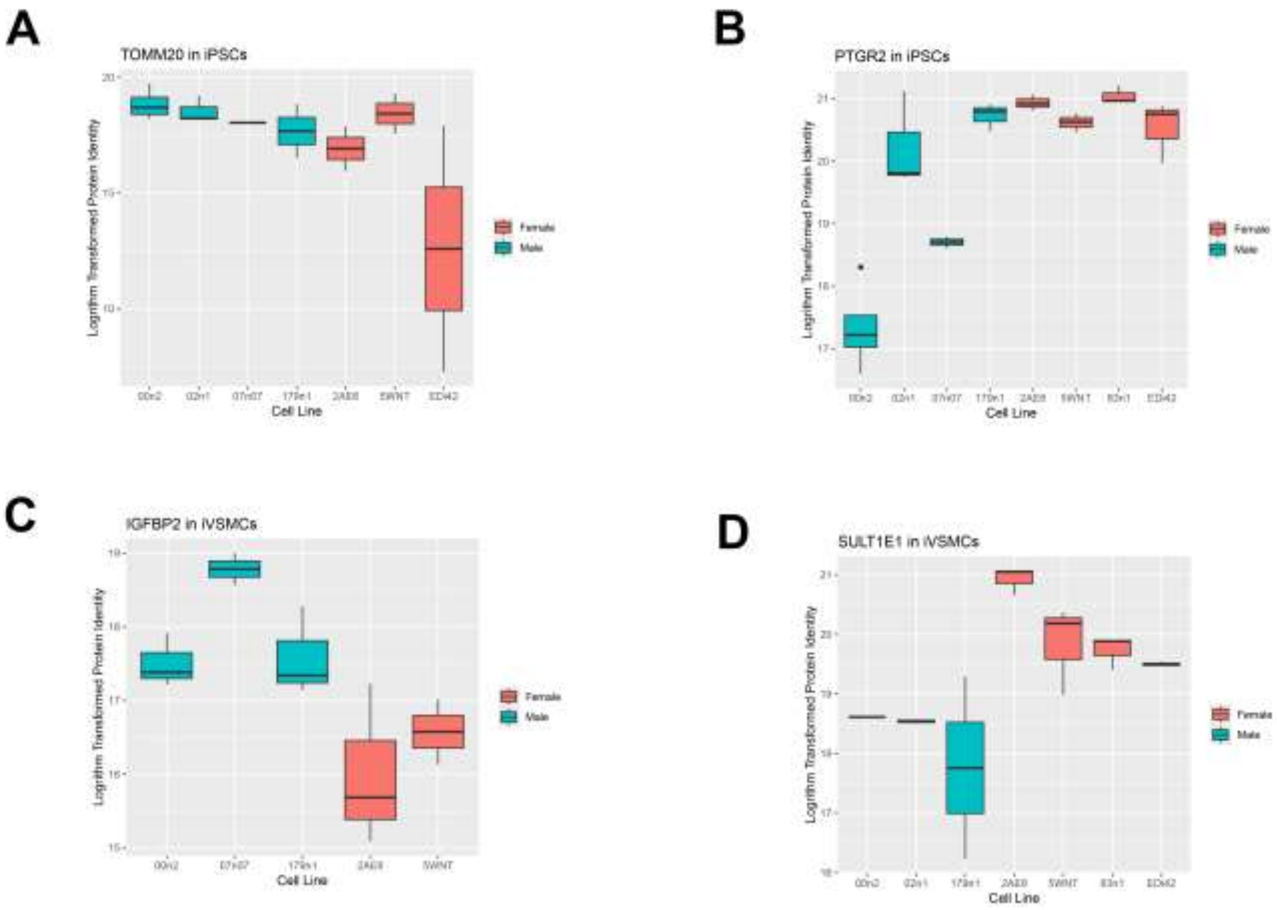

**Suppl. Table S1.** Characterization of the iPSC lines utilized in this study					
**Subject**	**Parent cell line**	**Disease condition**	**Sex**	**Age**	**Race**
CS0007iCTR-n07 (abbreviated as 07n07)	PBMC	Control	Male	60	Unknown
83iCTR_33n1 (abbreviated as 83n01)	Fibroblast	Control	Female	21	White
02iCTR_NTn1 (abbreviated as 02n1)	PBMC	Control	Male	51	White
EDi042-A	PBMC	Control	Female	79	White
5NWTiCTR-n3 (abbreviated as 5NWTn3)	PBMC	Control	Female	53	White
CS0179iCTR-n1 (abbreviated as 0179n1)	PBMC	Control	Male	57	Unknown
2AE8iCTR-n6 (abbreviated as 2AE9n6)	PBMC	Control	Female	50	White
CS00iCTR-n2 (abbreviated as 00n2)	Fibroblast	Control	Male	6	Unknown

* PBMC: Peripheral Blood Mononuclear Cell

Supplementary Table S2. Gene Set Enrichment Analysis for iPSCs vs iVSMCs. (Upregulation indicates upregulation in iPSCs)

GOTerm	Ontology Source	Group PValue	% Associated Genes	Nr. Genes	Associated Genes Found	Term Status
protein exit from endoplasmic reticulum	GO_BiologicalProcess-EBI-UniProt-GOA-ACAP-ARAP_13.05.2021_00h00	9.75E-06	28.57143	14	[AUP1, CD81, ERLEC1, FAF2, HSP90B1, LMAN1, SAR1A, SEC13, SEC16A, SEL1L, SURF4, TM9SF4, TMED9, YOD1]	Downregulation
homotypic cell-cell adhesion	GO_BiologicalProcess-EBI-UniProt-GOA-ACAP-ARAP_13.05.2021_00h00	5.17E-06	22.34043	21	[ABAT, ACTN1, ANK3, CXADR, DSP, F11R, FGG, FN1, HSPB1, ILK, LGALS1, METAP1, MYH9, MYL9, PKP2, PLPP3, PRKCA, RAP2B, RDX, TJP2, TLN1]	Downregulation
histone kinase activity	GO_BiologicalProcess-EBI-UniProt-GOA-ACAP-ARAP_13.05.2021_00h00	7.55E-06	50	8	[AURKA, AURKB, BAZ1B, CHEK1, DCAF1, PRKAA1, PRKCA, VRK1]	Upregulation
intracellular signal transduction	GO_BiologicalProcess-EBI-UniProt-GOA-ACAP-ARAP_13.05.2021_00h00	4.20E-06	9.648241	288	[ACTN4, AKT1, ANK2, ANTXR1, APAF1, APOE, ARAP1, ARAP3, ARF6, ARFGAP1, ARFGAP3, ARFGEF1, ARHGAP1, ARHGAP28, ARHGAP5, ARHGDIA, ARHGEF1, ARHGEF11, ARHGEF12, ARL2, ARL3, ARL6IP5, ATP2B4, ATR, AURKA, AURKB, BOP1, BRD4, BUB1B, C1QBP, CALR, CAMK2D, CAP1, CAP2, CARHSP1, CASP3, CASTOR2, CAT, CAV1, CBL, CCAR2, CCDC22, CCNB1, CD2AP, CD44, CD81, CDC42, CDC42BPA, CDC42BPB, CDC42EP1, CDC42EP3, CDH2, CDK5RAP3, CHD4, CHEK1, CHEK2, CLASP1, CNOT11, CNRIP1, COL1A2, COPS5, CSPG4, CTNNB1, CUL4A, CYFIP1, DAPK1, DAXX, DBNL, DDRGK1, DDX21, DDX39B, DDX5, DGKA, DHX33, DHX8, DICER1, DIPK2A, DLG1, DNMT1, DOCK7, DUSP3, ECT2, EEF1E1, EGFR, EHMT1, EIF3A, EPS8, EPS8L2, ERBIN, ERO1A, ETFA, F11R, FANCD2, FERMT2, FGG, FKBP8, FLNB, FLT1, FN1, G3BP1, G3BP2, GAB1, GARS1, GBF1, GDI1, GOLT1B, GPD1L, GPI, GSDME, GSN, GSTM2, HDAC2, HDAC4, HELLS,	No change

					HMOX1, HSP90AB1, HSPB1, HTT, ILK, IQGAP1, IQGAP3, IRF3, ITCH, ITGAV, ITGB1, KANK2, KAT7, KDM1A, KNTC1, LARS1, LGALS1, LIMS1, LIN28A, LMCD1, LRP1, LRRC59, LRRK1, MAGED1, MAP2K6, MAPK1, MAPK14, MAPK8, MASTL, MAVS, MBD3, MCU, MDC1, MEAK7, MLH1, MMAB, MSH2, MSH6, MTDH, MYADM, MYBBP1A, MYD88, MYDGF, MYO1C, MYO9B, NCAM1, NCKAP1, NDC80, NDRG1, NDRG2, NEDD4, NFKB1, NPM1, NQO2, NSUN2, NUCB2, OGT, OPTN, ORC3, P4HB, PABPN1, PAK1, PARP1, PASK, PDGFRB, PEA15, PI4KA, PIAS4, PIK3C2A, PIK3CA, PIP4K2B, PIP4K2C, PLCB3, PLCG1, PLK1, POLB, POLR1B, PPM1B, PPM1F, PPP1R9B, PRDX1, PRDX4, PRKAA1, PRKAB1, PRKACB, PRKACG, PRKAG1, PRKAR1A, PRKAR2A, PRKAR2B, PRKCA, PRKCSH, PRKDC, PRPF38B, PSMC6, PSMD1, PSMD2, PSMD9, PSME3, PTPMT1, PTPN1, PTPN2, PYCARD, RAB18, RAC3, RAI14, RALGPS2, RAP2A, RAP2B, RAP2C, RDX, RELA, RFC2, RFC3, RFC5, RHEB, RIT1, ROCK2, RPA1, RPA2, RPA3, RPF2, RPL5, RPS6KA1, RPS6KA3, RPS6KA4, RRAS, RRS1, RTKN, SEC13, SEH1L, SESN2, SETDB1, SIRT1, SIRT2, SLC44A2, SORBS3, SORD, SPAG9, SPTAN1, SPTBN1, SPTBN2, SRC, SRPK1, SSRP1, STK38, STYX, SUPT16H, TELO2, TF, TFG, TGM2, THBS1, TIMP3, TIPRL, TJP2, TKFC, TLK1, TNS1, TNS3, TPX2, TRAP1, TRIM28, TRIP13, TTI1, TTK, UFL1, USP15, USP47, USP7, YAP1, ZDHHC17, ZFP36L2, ZWILCH]	
melanosome	GO_CellularComponent-EBI-UniProt-GOA-ACAP-ARAP_13.0	4.87E-10	26.27119	31	[ANXA11, ANXA2, ANXA6, ATP1A1, CALU, CANX, CCT4, GSN, HSP90AB1, HSP90B1, HSPA5, ITGB1, MYO5A, NCAPG, P4HB, PDIA3, PDIA4, PDIA6, PPIB, PRDX1, PSMD2, RAB1A,	Downregulation

	5.2021_00h00				RAB2A, RAB32, RAB5B, RAB5C, RAN, RPN1, SEC22B, SLC2A1, TMED10]	
response to drug	GO_BiologicalProcess-EBI-UniProt-GOA-ACAP-ARAP_13.05.2021_00h00	2.41E-06	14.02715	62	[ABAT, ABCC1, ACSL1, ADAM10, AK4, ANTXR1, ANXA1, ARL6IP5, ATP1A1, ATR, CALR, CASP3, CAT, CCNB1, CDH3, CHEK2, COL1A1, CTNNB1, CTPS1, CYP2S1, DNMT3A, DNMT3B, DPYSL2, EEF2, EFTUD2, EGFR, FECH, GATA6, GCLM, HADHA, HDAC2, HDAC4, HMOX1, HSP90AB1, HSPA5, HSPD1, LGALS1, MAP1B, MAP2K6, MCM7, MGST1, MMAB, OXCT1, PFAS, PNP, POR, PPM1F, PPP1R9B, PRKAA1, PRKAR2B, QDPR, RAD51, RAP2A, RELA, S100A10, SLC1A3, SORD, SRC, SRR, THBS1, USP47, XRCC5]	No change
mitochondrial nucleoid	GO_CellularComponent-EBI-UniProt-GOA-ACAP-ARAP_13.05.2021_00h00	4.29E-06	30.43478	14	[DDX28, DHX30, ELAC2, FASTKD2, HADHA, HADHB, HSD17B10, HSPA9, LRPPRC, LRRC59, SLC25A5, SSBP1, TFB1M, TRMT10C]	Upregulation
identical protein binding	GO_MolecularFunction-EBI-UniProt-GOA-ACAP-ARAP_13.05.2021_00h00	4.01E-15	12.11832	254	[ABAT, ABCB10, ABCB7, ACACA, ACAT1, ACTN1, ACTN4, ADAM10, ADD2, ADSL, AKT1, ALCAM, ANXA1, ANXA2, ANXA4, ANXA6, APAF1, APEH, APOE, APPL2, ASL, ASNS, ATIC, ATL2, ATL3, BAG2, BAIAP2, BIN1, CACYBP, CALCOCO2, CAMK2D, CAP2, CARNMT1, CASTOR2, CAT, CAV1, CAVIN1, CBX5, CD2AP, CDC42, CDC42BPA, CDH2, CERCAM, CHAF1A, CHEK2, COL1A1, COL1A2, CPOX, CRLF3, CRMP1, CTPS1, CTSC, DAPK1, DARS2, DCTD, DCXR, DDX21, DDX39B, DECR1, DHPS, DHX8, DNMT3A, DPYSL2, DPYSL3, DRG1, DYNC1LI2, ECE1, ECT2, EEA1, EGFR, EHD1, EHD2, EMSY, EPM2AIP1, ESD, ESYT2, ETHE1, EXD2, F11R, FAM118B, FECH, FGG, FKBP8, FLAD1, FLNB, FN1, FSD1, FXR1, GALE, GARS1, GIPC1, GIPC2, GLA, GLMN, GLUD1, GLUL, GNPDA2, GOLGA2, GOPC, GOT2, GPD1L, GPHN, GSS, GSTM2, GSTM3, HACL1,	Downregulation

					HADHA, HCFC1, HDAC4, HMOX1, HOOK3, HSD17B4, HSP90AB1, HSPB1, HTRA1, HTT, IMPDH2, IRF3, JAM3, KCTD12, KEAP1, KIF5B, LDHB, LGALS1, LNPK, LRRC8A, LRRK1, LYAR, MAGED1, MAPK1, MATR3, MCM6, ME1, MESD, MGAT2, MGST1, MME, MPC2, MSH2, MSH3, MSH6, MSI1, MVD, MVK, MYD88, MYH9, MYO1C, MYO9B, NCL, NDC80, NDRG1, NECTIN2, NFKB1, NIF3L1, NLGN4X, NPM1, NQO2, NUDT16, NUP35, OPLAH, OPTN, OXCT1, P4HA1, PAICS, PARD3, PARP1, PC, PCYT1A, PDIA3, PDLIM4, PDXK, PFKL, PFKM, PFKP, PIP4K2B, PIP4K2C, PLK1, PM20D2, PNP, PON2, PRDX1, PRPSAP1, PSMC6, PSME3, PYCARD, PYCR1, QDPR, RAD51, RBPMS, RBPMS2, RELA, RPL22, S100A10, S100A11, SAMHD1, SARS1, SBDS, SCARB2, SCLY, SDF4, SEC13, SEPHS1, SEPTIN7, SH3GL3, SH3GLB1, SIL1, SIRT1, SKP2, SLC25A12, SLC2A1, SLC9A3R2, SLK, SNRNP200, SNX6, SNX9, SORD, SPAG9, SRM, SRR, SSBP1, STIM1, SUMF2, SUN2, TARS2, TELO2, TENM3, TFG, TGM2, THBS1, TKT, TMEM115, TMEM192, TMEM43, TNPO3, TOP2A, TPM1, TPM4, TPP2, TPST1, TRIP13, TRMT10C, UAP1, UGDH, UGP2, USP15, VAMP2, VIM, VPS25, WARS1, WRAP53, XPNPEP1, XRN2, YARS2, ZDHHC17]	
response to alkaloid	GO_BiologicalProcess-EBI-UniProt-GOA-ACAP-ARAP_13.05.2021_00h00	2.06E-05	19.04762	24	[ABAT, CASP3, DHX15, DNMT3A, DNMT3B, DPYSL2, EFTUD2, GOT2, GPI, GSTM2, HDAC2, HSP90AB1, HSPA5, HSPD1, MAP1LC3A, PEA15, PPP1R9B, PRKAA1, RAD51, RELA, SLC1A3, SRR, TGM2, TMED10]	No change
single-stranded RNA binding	GO_MolecularFunction-EBI-UniProt-GOA-ACAP-ARAP_13.05.2021_00h00	7.84E-06	22.47191	20	[AGO1, AGO2, ANXA1, CYFIP1, DAZAP1, DHX9, EIF4A3, EXOSC10, FXR1, HNRNPF, ILF3, L1TD1, MSI1, PNPT1, POLR2G, PUS1, RBMS2, RBPMS, SSB, TIA1]	Upregulation

cell-cell contact zone	GO_CellularComponent-EBI-UniProt-GOA-ACAP-ARAP_13.05.2021_00h00	6.77E-06	23.45679	19	[ACTN1, ANK2, ANK3, ATP1A1, CDH2, CTNNB1, DLG1, DSP, ITGB1, JAM3, NECTIN2, NOL6, PAK1, PIK3CA, PKP2, RAP2B, RAP2C, SLC2A1, TJP2]	Downregulation
negative regulation of protein kinase activity by regulation of protein phosphorylation	GO_BiologicalProcess-EBI-UniProt-GOA-ACAP-ARAP_13.05.2021_00h00	1.24E-05	66.66666	6	[ADAR, ADARB1, CDK5RAP3, CORO1C, NPM1, PPM1F]	Downregulation
mRNA binding	GO_MolecularFunction-EBI-UniProt-GOA-ACAP-ARAP_13.05.2021_00h00	3.06E-10	18.32797	57	[ACO1, ADARB1, AGO2, C1QBP, CALR, CARHSP1, CCT5, CELF1, CIRBP, CLUH, CSTF3, DAZAP1, DDX5, DDX6, DHX33, DHX9, EIF3A, EIF4A3, FXR1, G3BP1, G3BP2, GEMIN5, GNL3, HDLBP, HNRNPL, HNRNPLL, HNRNPM, IGF2BP3, ILF3, LIN28A, MYEF2, MYH10, NCL, NUDT16, PARN, PCBP2, PKM, PTBP2, RBM15, RBM25, RBM3, RBMS2, RBPMS, RBPMS2, RPL13A, RPL5, RPS2, SAMD4B, SERBP1, SF3B1, SSB, SUPT5H, TIA1, TUT1, UPF3B, XPO5, ZFP36L2]	Upregulation
perinuclear region of cytoplasm	GO_CellularComponent-EBI-UniProt-GOA-ACAP-ARAP_13.05.2021_00h00	4.49E-10	13.86393	108	[ACTN4, ADAM10, ALG2, ANXA2, ANXA4, ANXA6, ARFGEF1, ARHGAP1, AURKA, BPTF, BUB1B, CALCOCO2, CALR, CAPN2, CAV1, CBL, CCAR1, CD2AP, CDK7, CKAP4, COPS5, CTNNB1, CUL7, CYFIP1, DDX6, DICER1, DLG1, ECE1, EGFR, EHD1, EHD2, EHD4, EML1, FAT1, FBXW8, FKBP4, FXR1, GALNT1, GALNT2, GNB2, GSN, HMOX1, HSP90AB1, HSP90B1, HTT, ITGB1, KIF5B, KIRREL1, KRT18, LAMB1, LIMS1, LRPPRC, MAP1B, MAP1S, MCM3, MSN, MTDH, MTMR14, MTMR6, MYO9B, NDRG1, NDRG2, NEDD4, OPTN, OSBP, PDCL3, PDLIM4, PICALM, PIK3CA, PLEC, PPIB, PPM1F, PRKACB, PRKAR2B, PRKCA, PRKCSH, RAB14, RAC3, RAD51, RANBP2, RANGAP1, RHPN2, SBF1, SEC16A, SEC23A, SEC23B, SEC31A, SEPTIN2, SERBP1, SET, SIRT2, SLC1A3, SLC2A10, SLC5A3,	Downregulation

					SLIRP, SLK, SPAG9, SRC, TF, TGM2, TMEM192, TOLLIP, TWF1, UPF2, VAMP2, VAMP3, VPS53, YTHDC2]	
regulation of helicase activity	GO_BiologicalProcess-EBI-UniProt-GOA-ACAP-ARAP_13.05.2021_00h00	1.55E-05	53.84615	7	[MCM2, MCM7, MSH2, MSH3, MSH6, SIRT1, SSBP1]	Upregulation
oligosaccharyl transferase activity	GO_MolecularFunction-EBI-UniProt-GOA-ACAP-ARAP_13.05.2021_00h00	1.18E-06	85.71429	6	[ALG5, OSTC, PSMD2, RPN1, STT3A, STT3B]	Downregulation
NAD binding	GO_MolecularFunction-EBI-UniProt-GOA-ACAP-ARAP_13.05.2021_00h00	1.46E-06	26.86567	18	[ALDH2, BDH2, CTBP2, CYB5R3, GLUD1, GPD1L, HADHA, HIBADH, IDH2, ME1, NNT, PARP1, QDPR, SIRT1, SIRT2, SORD, UGDH, ZC3HAV1]	Downregulation
integrin binding	GO_MolecularFunction-EBI-UniProt-GOA-ACAP-ARAP_13.05.2021_00h00	1.42E-06	19.35484	30	[ACTN1, ACTN4, ADAM10, CALR, CD81, COL5A1, CXADR, EGFL6, EGFR, F11R, FERMT2, FN1, GLMN, ILK, ITGAV, ITGB1, JAM3, LAMB1, LAMC1, MFGE8, MYH9, P4HB, PLPP3, PRKCA, PTPN2, SRC, THBS1, TLN1, TLN2, UTRN]	Downregulation
structural constituent of cytoskeleton	GO_BiologicalProcess-EBI-UniProt-GOA-ACAP-ARAP_13.05.2021_00h00	8.68E-07	22.64151	24	[ACTBL2, ACTR2, ADD2, ADD3, ANK2, ANK3, CD2AP, DSP, EPB41, EPB41L3, ERBIN, KRT19, MSN, PLEC, SORBS3, SPTAN1, SPTBN1, SPTBN2, TLN1, TLN2, TPM1, TUBA4A, TUBB6, VIM]	Downregulation
response to epidermal growth factor	GO_BiologicalProcess-EBI-UniProt-GOA-ACAP-ARAP_13.05.2021_00h00	1.75E-06	29.09091	16	[AKT1, BAIAP2, CBL, COL1A1, DUSP3, EGFR, IQGAP1, MAPK1, MARS1, MCM7, NCL, PLCG1, PPP1R9B, PTPN12, SNX6, ZFP36L2]	Downregulation
nuclear envelope	GO_CellularComponent-EBI-UniProt-GOA-ACAP-ARAP_13.05.2021_00h00	9.14E-06	13.1579	65	[AAAS, ABCF1, ANXA11, ANXA4, APEH, BIN1, CACYBP, CALR, CBX5, CCND2, CSE1L, DCTN1, DHX37, EGFR, EMD, ERBIN, GARS1, GATA6, GTF3C3, IPO11, IPO5, IST1, LBR, LRPPRC, LRRC59, MATR3, MFSD10, MTDH, MTMR6, MYO1C, MYO1E, NDC1, NUCB2, NUP107, NUP133, NUP160, NUP210, NUP35, NUP50, PAK1,	No change

					PARP1, PCYT1A, PGRMC2, POLA1, RAN, RANBP2, RANGAP1, RBM15, RETSAT, RIF1, RRP12, SCRN1, SEC13, SEH1L, SEPHS1, SIRT1, SUN2, TEX2, TMEM43, TMEM97, TMX4, TNPO3, UTP18, WDR3, XPO7]	
transcription regulator activity	GO_BiologicalProcess-EBI-UniProt-GOA-ACAP-ARAP_13.05.2021_00h00	2.69E-10	4.447323	103	[AASS, ACTN1, ACTN4, AEBP2, AKT1, ANXA3, ANXA4, ATF7IP, BRD4, BRMS1, C1QBP, CAT, CAV1, CCAR1, CCAR2, CCDC22, CDK5RAP3, CEBPZ, COPS2, COPS5, CTBP2, CTCF, CTNNB1, CYFIP1, DAXX, DDRGK1, DDX54, DHX33, DHX9, DNMT3A, DNMT3B, ERBIN, FOXK1, G3BP2, GARS1, GATA6, GTF2I, HCFC1, HDAC2, HDAC4, HMOX1, HSD17B4, IRF2BPL, IRF3, ITCH, KAT7, KDM1A, KDM2A, KDM3B, KEAP1, LMCD1, LYAR, MAGED1, MAPK1, MAPK14, MAPK8, MAVS, MTA3, MTDH, MYBBP1A, MYD88, MYO1C, NFKB1, NIBAN2, NPM1, PATZ1, PAWR, PIAS4, PLPP3, PSIP1, PSMD9, PTGIS, PURA, PXDN, PYCARD, RAP2C, RBM15, RBPJ, RBPMS, RELA, RPS6KA4, SALL2, SIN3A, SIRT1, SMARCA4, SMARCC1, SMARCD1, SMARCD2, STXBP2, TAX1BP1, TDP2, TFB1M, TGFB1I1, TMF1, TRIM28, TRIP13, UFL1, USP7, WDR43, YAP1, ZMYND8, ZNF217, ZNF281]	No change
nuclear protein-containing complex	GO_CellularComponent-EBI-UniProt-GOA-ACAP-ARAP_13.05.2021_00h00	5.83E-18	14.34169	183	[AAAS, ACTBL2, ADAR, AEBP2, ANP32E, API5, ARFGEF1, BOP1, BPTF, BRMS1, BUB1B, CBX2, CBX5, CCNH, CDC73, CDK7, CHD4, CIRBP, COPS2, COPS5, COPS7A, CPSF2, CPSF3, CSTF1, CTNNB1, CTNNBL1, CUL7, CWC27, DCAF1, DDX20, DDX39B, DDX41, DDX5, DHX15, DHX8, DICER1, DOCK7, EFTUD2, EIF4A3, ERCC2, EXOSC10, EXOSC2, EXOSC5, EXOSC7, GARS1, GATAD2A, GEMIN4, GEMIN5, GINS3, GTF2E1, HCFC1, HDAC2, HDAC4, HEATR1, HNRNPF, HNRNPM, HSP90AB1, HSPA5, IGF2BP3, IMP3, INO80C, IPO5, JADE1, JARID2, KAT7, KDM3B, LAS1L, LIG3, LRWD1,	Upregulation

					LSM4, MBD3, MCM2, MCM3, MCM4, MCM5, MCM6, MCM7, MLH1, MPHOSPH10, MSH2, MSH3, MSH6, MTA3, MTREX, MYEF2, MYH9, MYO1C, MYO1E, NCL, NDC1, NOL11, NOL6, NOP14, NUP107, NUP133, NUP160, NUP210, NUP35, NUP50, NVL, OGT, ORC2, ORC3, ORC4, ORC5, PELP1, PES1, PHC1, PLCG1, POLA1, POLA2, POLD1, POLE, POLR1B, POLR2A, POLR2B, POLR2G, POLR3A, POLR3C, POLR3F, PRIM1, PRIM2, PRPF38A, PRPF38B, PRPF40A, PRPF8, PWP2, RAN, RANBP2, RANGAP1, RBBP5, RBM3, RELA, RHEB, RPA1, RPA2, RPA3, RPRD1A, RRP7A, SART3, SEC13, SEH1L, SF3A3, SF3B1, SIN3A, SIRT1, SIRT2, SMARCA4, SMARCA5, SMARCC1, SMARCD1, SMARCD2, SNRNP200, SNRPA1, SNRPD3, SRSF1, SSRP1, SUN2, SUPT16H, SUPT5H, SUPT6H, SYMPK, TBPL1, TELO2, TEX10, TRIM28, TSEN34, TTF2, TTI1, TUT1, UPF2, UPF3B, UTP18, UTP4, WDR18, WDR3, WDR36, WRAP53, XPO7, XRCC5, XRCC6, YAP1, ZNF217]	
endoplasmic reticulum protein-containing complex	GO_CellularComponent-EBI-UniProt-GOA-ACAP-ARAP_13.05.2021_00h00	1.19E-06	20.28986	28	[AUP1, CALR, FAF2, GET3, HLA-A, HSP90B1, HSPA5, NBAS, OSTC, P4HA1, P4HB, PDIA3, PDIA6, PIGS, PIGT, PPIB, PRKCSH, PSMD2, RPN1, SDF2L1, SEC61G, SEL1L, SPTLC2, SRPRA, SRPRB, SSR4, STT3A, STT3B]	Downregulation
exoribonuclease complex	GO_CellularComponent-EBI-UniProt-GOA-ACAP-ARAP_13.05.2021_00h00	9.86E-06	38.46154	10	[CARHSP1, EXOSC10, EXOSC2, EXOSC5, EXOSC7, GTPBP1, LAS1L, MTREX, NVL, PNPT1]	Upregulation
magnesium ion binding	GO_MolecularFunction-EBI-UniProt-GOA-ACAP-ARAP_13.05.2021_00h00	5.45E-08	18.29787	43	[ARL3, CDC42BPA, CDC42BPB, CLASP1, ENO2, ENOPH1, EXD2, FSCN1, GCLC, GLUL, GPI, GSS, HACL1, HMGCL, IDH2, ILVBL, ITPK1, MSH2, MSH6, MVK, NUDT16, PDXK, PGM3, PKM, PLK1, PLSCR3, PPM1B, PRKACB, PRKCA, PRPSAP1, RAN, RAP2A, RHEB, RP2, RPS6KA1,	No change

					RPS6KA3, RPS6KA4, SRPK1, SRR, STK38, TDP2, TKT, TOP2A]	
cytoplasm	GO_CellularComponent-EBI-UniProt-GOA-ACAP-ARAP_13.05.2021_00h00	7.12E-95	10.40486	1267	[AAAS, AASS, AATF, ABAT, ABCB10, ABCB7, ABCF1, ABHD10, ABHD12, ABHD14B, ABI1, ACAA1, ACACA, ACAD8, ACAD9, ACAT1, ACAT2, ACBD3, ACIN1, ACLY, ACO1, ACOT13, ACOT9, ACOX3, ACSL1, ACSL4, ACSS2, ACSS3, ACTBL2, ACTN1, ACTN4, ACTR10, ACTR1B, ACTR2, ADAM10, ADAR, ADARB1, ADD2, ADD3, ADPGK, ADSL, AGL, AGO1, AGO2, AGTPBP1, AIMP2, AK1, AK4, AKR7A2, AKT1, ALDH1L2, ALDH2, ALDH7A1, ALDOC, ALG11, ALG2, ALG5, ALG9, AMDHD2, AMPD2, ANK2, ANK3, ANKFY1, ANKRD28, ANKS1A, ANKZF1, ANO6, ANP32E, ANTXR1, ANXA1, ANXA11, ANXA2, ANXA3, ANXA4, ANXA5, ANXA6, AP1M1, AP1S1, AP2A1, AP2A2, AP2B1, AP2M1, AP2S1, AP3B1, APAF1, APEH, API5, APOBEC3C, APOE, APOOL, APPL2, APRT, ARAP1, ARAP3, ARCN1, ARF4, ARF6, ARFGAP1, ARFGAP3, ARFGEF1, ARHGAP1, ARHGAP28, ARHGAP5, ARHGDIA, ARHGEF1, ARHGEF11, ARHGEF12, ARL1, ARL2, ARL3, ARL6IP5, ARMC6, ARPC1A, ARSA, ARSB, ARVCF, ASAP1, ASL, ASNS, ASPH, ATF7IP, ATIC, ATL2, ATL3, ATP1A1, ATP2B1, ATP2B2, ATP2B4, ATP6V1A, ATR, AUP1, AURKA, AURKB, B3GLCT, B4GALT1, BAG1, BAG2, BAG3, BAIAP2, BCAT2, BCCIP, BDH2, BET1, BIN1, BIRC6, BLVRB, BPNT2, BPTF, BRAT1, BRMS1, BRWD1, BUB1B, BZW2, C1QBP, C1orf198, CACYBP, CALCOCO2, CALD1, CALR, CALU, CAMK1, CAMK2D, CANX, CAP1, CAP2, CAPN1, CAPN2, CAPNS1, CAPRIN1, CAPZA2, CARHSP1, CARNMT1, CASK, CASP3, CAST, CASTOR2, CAT, CAV1, CAVIN1, CBL, CBR1, CCAR1, CCAR2, CCDC22, CCNB1, CCND2, CCNY,	No change

					CCT2, CCT3, CCT4, CCT5, CCT6A, CCT7, CCT8, CD2AP, CD44, CDC123, CDC42, CDC42BPA, CDC42BPB, CDC42EP1, CDC42EP3, CDC73, CDCA8, CDH2, CDH3, CDK5RAP1, CDK5RAP3, CDK7, CDV3, CELF1, CEP170, CERCAM, CFAP20, CFAP298, CFL2, CHD1, CHD4, CHEK1, CHEK2, CHID1, CHPF, CHST14, CHTF18, CIRBP, CKAP4, CKAP5, CLASP1, CLUH, CMBL, CMPK1, CNDP2, CNN3, CNOT11, CNRIP1, COG1, COG6, COG7, COG8, COL1A1, COL1A2, COL4A1, COL4A2, COL5A1, COL5A2, COL6A1, COL6A2, COL6A3, COLGALT1, COMMD4, COPA, COPB1, COPB2, COPE, COPG1, COPG2, COPS2, COPS5, COPS7A, COPZ1, CORO1C, CPNE2, CPOX, CRLF3, CRMP1, CRTAP, CSDE1, CSE1L, CSPG4, CSRP2, CTBP2, CTNNB1, CTNNBL1, CTPS1, CTSC, CUL4B, CUL7, CXADR, CYB5R1, CYB5R3, CYFIP1, CYP2S1, CYP51A1, DAGLB, DAPK1, DARS2, DAXX, DAZAP1, DBNL, DCAF1, DCAF13, DCTD, DCTN1, DCTN4, DCXR, DDRGK1, DDX20, DDX21, DDX28, DDX31, DDX39B, DDX41, DDX42, DDX5, DDX54, DDX6, DECR1, DEGS1, DGKA, DHPS, DHRS7B, DHX30, DHX33, DHX37, DHX8, DHX9, DIAPH1, DICER1, DIPK2A, DLG1, DLGAP5, DNMT3A, DPH1, DPH2, DPH6, DPP3, DPYSL2, DPYSL3, DRG1, DSP, DSTN, DUSP3, DYNC1LI2, ECE1, ECHDC1, ECPAS, ECT2, EDEM3, EEA1, EEF1E1, EEF2, EFTUD2, EGFR, EHD1, EHD2, EHD4, EIF3A, EIF4A3, EIF5B, ELAC2, ELP1, ELP3, EMD, EML1, ENDOD1, ENO2, ENOPH1, EPB41, EPB41L3, EPB41L5, EPHB3, EPM2AIP1, EPN2, EPS8, EPS8L2, ERAP1, ERBIN, ERCC2, ERCC6L, ERGIC1, ERGIC2, ERLEC1, ERLIN2, ERO1A, ERP44, ESD, ESYT2, ETFA, ETHE1, ETNK1, EXD2, EXOG, EXOSC10, EXOSC2, EXOSC5,	

					EXOSC7, F11R, FAF2, FAHD1, FAM114A1, FAM120A, FANCD2, FANCI, FARP1, FASTKD2, FAT1, FBXO2, FBXO30, FBXW8, FDPS, FDXR, FECH, FERMT2, FGG, FH, FHL1, FKBP10, FKBP14, FKBP15, FKBP4, FKBP5, FKBP7, FKBP8, FKBP9, FLAD1, FLNB, FLNC, FLT1, FN1, FNBP1L, FNDC3A, FOXK1, FOXRED1, FSCN1, FSD1, FTH1, FUT11, FXR1, G3BP1, G3BP2, GAB1, GALE, GALK1, GALM, GALNT1, GALNT10, GALNT2, GARS1, GART, GBE1, GBF1, GCDH, GCLC, GCLM, GDAP1, GDI1, GEMIN4, GEMIN5, GET3, GET4, GFPT2, GIPC1, GIPC2, GLA, GLB1L3, GLCCI1, GLMN, GLS, GLT8D1, GLUD1, GLUL, GMPPA, GMPPB, GMPS, GNB2, GNE, GNG12, GNPDA2, GNS, GOLGA2, GOLGA3, GOLIM4, GOLT1B, GOPC, GOSR2, GOT2, GPC1, GPC3, GPC6, GPD1L, GPHN, GPI, GPSM1, GPX7, GPX8, GSDME, GSN, GSS, GSTK1, GSTM2, GSTM3, GTF2E1, GTF2I, GTF3C4, GTPBP1, GUF1, GYS1, H6PD, HACL1, HADHA, HADHB, HAGH, HAT1, HCFC1, HDAC2, HDAC4, HDHD5, HDLBP, HEATR1, HEATR5A, HERC2, HEXA, HIBADH, HK1, HK2, HLA-A, HMBS, HMGCL, HMMR, HMOX1, HMOX2, HNRNPF, HNRNPL, HNRNPM, HOOK3, HSD17B10, HSD17B4, HSDL2, HSP90AB1, HSP90B1, HSPA13, HSPA14, HSPA2, HSPA4, HSPA5, HSPA9, HSPB1, HSPD1, HSPE1, HSPH1, HTRA1, HTT, HUWE1, IDH2, IDI1, IGF2BP3, IKBIP, ILF2, ILF3, ILK, ILVBL, IMPDH1, IMPDH2, INCENP, IPO11, IPO4, IPO5, IQGAP1, IQGAP2, IQGAP3, IRF3, IST1, ITCH, ITGAV, ITGB1, ITPK1, JADE1, JAM3, JARID2, KANK2, KAT7, KDELR1, KDELR3, KEAP1, KIF11, KIF20A, KIF21A, KIF22, KIF23, KIF2C, KIF5B, KIRREL1, KLC1, KNTC1, KRT18, KRT19, KRT8, KTN1, L2HGDH, LAMB1,	

					LAMC1, LANCL2, LARS1, LARS2, LAS1L, LASP1, LBR, LCMT1, LDHB, LGALS1, LIG1, LIG3, LIMS1, LIN28A, LMAN1, LMCD1, LMF2, LNPEP, LNPK, LPCAT1, LPGAT1, LPP, LRATD2, LRBA, LRP1, LRPPRC, LRRC40, LRRC59, LRRC8A, LRRK1, LRWD1, LSG1, LSM4, LSS, LTBP1, LYAR, LYPLA2, MACF1, MAGED1, MAGED2, MAIP1, MAN1A1, MAN1A2, MANBA, MAP1B, MAP1LC3A, MAP1S, MAP2K6, MAP4, MAPK1, MAPK14, MAPK8, MARCHF5, MARS1, MASTL, MAVS, MBD3, MBNL1, MCC, MCCC2, MCM2, MCM3, MCM5, MCM7, MCMBP, MCU, MDN1, ME1, MEAK7, MEMO1, MESD, MEST, METAP1, METTL1, MEX3A, MFGE8, MGAT2, MGST1, MICAL1, MICAL3, MICOS13, MIPEP, MMAB, MME, MOCS2, MOGS, MON2, MOSPD2, MOXD1, MPC2, MPRIP, MRPS27, MRPS9, MSH6, MSI1, MSN, MTA3, MTAP, MTDH, MTHFD1, MTMR14, MTMR6, MTR, MVB12A, MVD, MVK, MYADM, MYBBP1A, MYD88, MYDGF, MYEF2, MYH10, MYH9, MYL9, MYO1C, MYO1E, MYO5A, MYO9B, NBAS, NCAM1, NCAPD2, NCAPG, NCKAP1, NCL, NDC1, NDC80, NDRG1, NDRG2, NECAP2, NEDD4, NEK7, NFKB1, NHLRC2, NIBAN1, NIBAN2, NIF3L1, NIT2, NLN, NMT2, NNT, NOL6, NOP56, NOP58, NPM1, NPM3, NQO2, NSDHL, NSF, NSFL1C, NSUN2, NT5C2, NTHL1, NUCB2, NUDCD1, NUDT16, NUP107, NUP133, NUP160, NUP210, NUP35, NUSAP1, NXN, OCLN, OGT, OPLAH, OPTN, ORC3, ORC4, ORC5, OSBP, OSTC, OSTF1, OTUD6B, OXCT1, P3H1, P3H3, P3H4, P4HA1, P4HA2, P4HB, P4HTM, PABPN1, PACS1, PAICS, PAK1, PALD1, PALLD, PAPSS2, PARD3, PARD6B, PARN, PARP1, PARVA, PASK, PAWR,	

					PAXBP1, PC, PCBP2, PCCA, PCCB, PCYT1A, PCYT2, PDCD11, PDCD2, PDCL3, PDGFRB, PDIA3, PDIA4, PDIA6, PDLIM2, PDLIM4, PDLIM5, PDLIM7, PDS5A, PDXDC1, PDXK, PDZRN3, PEA15, PELP1, PES1, PFAS, PFKL, PFKM, PFKP, PGAM5, PGM2L1, PGM3, PGRMC2, PHIP, PHPT1, PI4KA, PIAS4, PICALM, PIGS, PIGT, PIK3C2A, PIK3CA, PIK3R4, PIP4K2B, PIP4K2C, PITRM1, PKM, PKP2, PLCB3, PLCG1, PLD3, PLEC, PLEKHA5, PLEKHA7, PLIN3, PLK1, PLOD1, PLOD2, PLOD3, PLPP3, PLS3, PLSCR3, PM20D2, PNO1, PNP, PNPT1, POFUT1, POFUT2, POGLUT2, POGLUT3, POGZ, POLA1, POLA2, POLB, POLD1, POLR1B, POLR2A, POLR2G, POLR3A, POLR3C, POLR3F, PON2, POR, PPFIBP1, PPIB, PPIC, PPID, PPM1B, PPM1F, PPM1G, PPP1R18, PPP1R7, PPP1R9B, PPP2R5A, PRAF2, PRDX1, PRDX4, PREPL, PRKAA1, PRKAB1, PRKACB, PRKACG, PRKAG1, PRKAR1A, PRKAR2A, PRKAR2B, PRKCA, PRKCSH, PRKDC, PRMT3, PRORP, PRPF38B, PRPSAP1, PRRC1, PRUNE1, PSIP1, PSMC6, PSMD1, PSMD2, PSMD9, PSME3, PSPC1, PTCD1, PTCD3, PTGIS, PTPMT1, PTPN1, PTPN12, PTPN2, PURA, PUS1, PUS3, PXDN, PXK, PXMP2, PYCARD, PYCR1, PYCR2, PYCR3, QDPR, QRSL1, RAB14, RAB18, RAB1A, RAB23, RAB2A, RAB32, RAB5B, RAB5C, RAB8A, RABGAP1L, RABL6, RAC3, RAD51, RAI14, RALGPS2, RAN, RANBP2, RANGAP1, RAP1GDS1, RAP2A, RAP2B, RAP2C, RBM25, RBM3, RBMS2, RBP1, RBPJ, RBPMS, RBPMS2, RCC2, RCN1, RDH10, RDX, RELA, RETSAT, RFC1, RFTN1, RHEB, RHPN2, RIC1, RIC8A, RIF1, RIMKLB, RNF170, RNH1, ROCK2, RP2, RPL13A, RPL22, RPL27A, RPL4, RPL5, RPL7A, RPN1, RPS2,	

					RPS21, RPS6KA1, RPS6KA3, RPS6KA4, RPS8, RRBP1, RRP12, RRP7A, RTKN, RTTN, S100A10, S100A11, SAMD4B, SAR1A, SARM1, SARS1, SARS2, SART3, SBDS, SBF1, SCAMP2, SCARB2, SCFD1, SCFD2, SCLY, SCPEP1, SCRN1, SCYL1, SDF2L1, SDF4, SEC13, SEC16A, SEC22B, SEC23A, SEC23B, SEC24A, SEC24B, SEC24C, SEC24D, SEC31A, SEC61G, SEH1L, SEL1L, SELENBP1, SEPHS1, SEPTIN10, SEPTIN11, SEPTIN2, SEPTIN7, SEPTIN8, SERBP1, SERPINB6, SERPINB9, SERPINH1, SESN2, SET, SETDB1, SH3GL3, SH3GLB1, SHPK, SIL1, SIRT1, SIRT2, SKA3, SKP2, SLAIN2, SLC12A4, SLC1A3, SLC25A12, SLC25A24, SLC25A3, SLC25A32, SLC25A5, SLC2A1, SLC2A10, SLC33A1, SLC44A1, SLC44A2, SLC4A7, SLC5A3, SLIRP, SLK, SMAP2, SMARCA5, SMARCC1, SMC2, SMS, SNRPD3, SNTB2, SNX17, SNX3, SNX4, SNX6, SNX9, SORBS1, SORBS3, SORD, SPAG9, SPARC, SPATS2L, SPECC1L, SPON1, SPOUT1, SPR, SPTAN1, SPTBN1, SPTBN2, SPTLC2, SRC, SRM, SRP14, SRPK1, SRPRA, SRPRB, SRR, SRSF1, SRSF7, SSB, SSBP1, SSR1, SSR4, STAM2, STIM1, STK38, STT3A, STT3B, STX12, STX5, STXBP2, STYX, SUCLG1, SUMF2, SUN2, SURF4, SYMPK, TACC3, TAGLN, TAGLN2, TARS2, TAX1BP1, TBC1D17, TBC1D4, TBCEL, TBL2, TBPL1, TBRG4, TCP1, TDP1, TDP2, TELO2, TEX10, TEX2, TF, TFB1M, TFG, TGFB1I1, TGM2, THBS1, THUMPD3, TIA1, TIGAR, TIMM50, TIMMDC1, TIMP3, TIPRL, TJP2, TKFC, TKT, TLN1, TLN2, TM9SF4, TMED10, TMED2, TMED5, TMED7, TMED9, TMEM115, TMEM167A, TMEM168, TMEM192, TMEM214, TMEM43, TMEM97, TMF1, TMX3, TNPO3, TNS1, TNS3, TOLLIP, TOMM34,	

					TOP2A, TPD52L2, TPM1, TPM4, TPP2, TPST1, TPX2, TRAM1, TRAP1, TRIM71, TRIP12, TRMT10C, TRMT5, TSR1, TTC1, TTC28, TTF2, TTI1, TTK, TTLL12, TUBA4A, TUBB6, TUT1, TWF1, TXNDC5, TXNL1, TXNRD1, UAP1, UBA6, UBE2G1, UBE2H, UBE2O, UBR4, UBR5, UFL1, UGDH, UGGT1, UGGT2, UGP2, UNG, UPF2, UPF3B, USE1, USP15, USP19, USP47, USP5, USP7, USP9X, UTP15, UTP20, UTRN, VAMP2, VAMP3, VAT1, VIM, VLDLR, VPS25, VPS26A, VPS26B, VPS26C, VPS35, VPS35L, VPS36, VPS53, VRK1, WAPL, WARS1, WDHD1, WDR18, WDR37, WRAP53, XPNPEP1, XPO5, XPO7, XRCC5, XRCC6, XRN1, YAP1, YARS2, YIF1A, YKT6, YOD1, YRDC, YTHDC2, ZC3H15, ZC3HAV1, ZDHHC17, ZFP36L2, ZFYVE16, ZMYM2, ZMYND8, ZNF185, ZNF217, ZNF638, ZWILCH]	
endoplasmic reticulum lumen	GO_CellularComponent-EBI-UniProt-GOA-ACAP-ARAP_13.05.2021_00h00	1.91E-16	21.62162	72	[ADAM10, APOE, ARSA, ARSB, CALR, CALU, CANX, CDH2, CERCAM, CKAP4, COL1A1, COL1A2, COL4A1, COL4A2, COL5A1, COL5A2, COL6A1, COL6A2, COL6A3, COLGALT1, CRTAP, CTSC, EDEM3, ERAP1, ERLEC1, ERO1A, ERP44, ESD, FGG, FKBP10, FKBP14, FKBP7, FN1, GBF1, GPC3, GPX7, GPX8, H6PD, HSP90B1, HSPA5, KTN1, LAMB1, LAMC1, LGALS1, LTBP1, MAPK1, MFGE8, MYDGF, P3H1, P4HA1, P4HA2, P4HB, PDIA3, PDIA4, PDIA6, PLOD3, POGLUT2, POGLUT3, PPIB, PRKCSH, RCN1, SDF2L1, SERPINH1, SIL1, SPON1, SUMF2, TF, THBS1, TMEM43, TXNDC5, UGGT1, UGGT2]	Downregulation
rough endoplasmic reticulum	GO_CellularComponent-EBI-UniProt-GOA-ACAP-ARAP_13.05.2021_00h00	3.59E-06	22.82609	21	[ARSB, BAIAP2, CANX, CKAP4, GLUL, HSPD1, LIN28A, PKM, PLOD1, PLOD2, PLOD3, PSMD2, RAB14, RPL4, RPN1, RPS21, SEC61G, SRPRA, SRPRB, SSR4, TMEM97]	Downregulation
cytosol	GO_CellularCompone	7.29E-83	13.58443	761	[AAAS, AASS, ABCF1, ABHD10, ABHD14B,	No change

	nt-EBI-UniProt-GOA-ACAP-ARAP_13.05.2021_00h00				ABI1, ACAA1, ACACA, ACAT2, ACIN1, ACLY, ACO1, ACOT13, ACOX3, ACSS2, ACTN1, ACTN4, ACTR10, ACTR1B, ACTR2, ADARB1, ADD2, ADD3, ADSL, AGL, AGO1, AGO2, AGTPBP1, AIMP2, AK1, AKR7A2, AKT1, ALDH7A1, ALDOC, ALG2, AMDHD2, AMPD2, ANK2, ANK3, ANKFY1, ANKRD28, ANKS1A, ANO6, ANXA1, ANXA11, ANXA2, ANXA5, AP1M1, AP1S1, AP2A1, AP2A2, AP2B1, AP2M1, AP2S1, APAF1, APEH, APRT, ARAP1, ARAP3, ARCN1, ARF4, ARF6, ARFGAP1, ARFGAP3, ARFGEF1, ARHGAP1, ARHGAP28, ARHGAP5, ARHGDIA, ARHGEF1, ARHGEF11, ARHGEF12, ARL1, ARL2, ARMC6, ARPC1A, ASAP1, ASL, ASNS, ATF7IP, ATIC, ATP6V1A, AURKA, AURKB, BAG1, BAG2, BAG3, BAIAP2, BCCIP, BDH2, BIN1, BLVRB, BPNT2, BRWD1, BUB1B, C1QBP, C1orf198, CACYBP, CALCOCO2, CALD1, CALR, CAMK1, CAMK2D, CAPN1, CAPN2, CAPNS1, CAPRIN1, CAPZA2, CARHSP1, CARNMT1, CASK, CASP3, CAST, CASTOR2, CAT, CAVIN1, CBL, CBR1, CCDC22, CCNB1, CCND2, CCT2, CCT3, CCT4, CCT5, CCT6A, CCT7, CCT8, CD2AP, CD44, CDC42, CDC42EP3, CDC73, CDCA8, CDK5RAP1, CDK5RAP3, CDK7, CDV3, CEP170, CFAP298, CHEK1, CHPF, CHTF18, CKAP4, CKAP5, CLASP1, CMBL, CMPK1, CNDP2, CNN3, CNOT11, COMMD4, COPA, COPB1, COPB2, COPE, COPG1, COPG2, COPS2, COPS5, COPS7A, COPZ1, CPOX, CRLF3, CRMP1, CSDE1, CSE1L, CTBP2, CTNNB1, CTNNBL1, CTPS1, CUL4B, CUL7, CYB5R3, CYFIP1, DAXX, DAZAP1, DBNL, DCAF13, DCTD, DCTN1, DCTN4, DDX20, DDX21, DDX28, DDX41, DDX42, DDX5, DDX6, DECR1, DGKA, DHPS, DHX30, DHX33, DHX8, DHX9, DIAPH1, DICER1, DLG1, DLGAP5, DPH1, DPH2, DPH6, DPP3,	

					DPYSL2, DPYSL3, DRG1, DUSP3, DYNC1LI2, ECHDC1, ECT2, EEA1, EEF1E1, EEF2, EFTUD2, EHD2, EIF3A, EIF4A3, EIF5B, ELP1, ELP3, EMD, EML1, ENDOD1, ENO2, ENOPH1, EPB41, EPB41L3, EPB41L5, EPHB3, EPN2, EPS8L2, ERAP1, ERCC2, ERCC6L, ERLIN2, ESD, ETFA, ETNK1, EXOSC10, EXOSC2, EXOSC5, EXOSC7, FAHD1, FAM114A1, FAM120A, FANCD2, FANCI, FARP1, FBXO2, FBXO30, FBXW8, FDPS, FERMT2, FH, FHL1, FKBP4, FKBP5, FKBP8, FLAD1, FLNB, FLNC, FNBP1L, FNDC3A, FSCN1, FTH1, FXR1, G3BP1, G3BP2, GAB1, GALE, GALK1, GARS1, GART, GBE1, GBF1, GCLC, GCLM, GDAP1, GDI1, GEMIN4, GEMIN5, GET4, GFPT2, GIPC1, GLS, GLUL, GMPS, GNB2, GNE, GNPDA2, GOLGA3, GOLT1B, GOSR2, GPC1, GPD1L, GPHN, GPI, GPSM1, GSDME, GSN, GSS, GSTK1, GSTM2, GSTM3, GTF2E1, GTPBP1, GYS1, HACL1, HAGH, HDAC4, HDLBP, HEATR5A, HERC2, HEXA, HK1, HK2, HMBS, HMGCL, HMMR, HMOX1, HNRNPF, HOOK3, HSD17B4, HSP90AB1, HSP90B1, HSPA14, HSPA2, HSPA4, HSPA5, HSPB1, HSPD1, HSPH1, HTRA1, HTT, HUWE1, IDH2, IDI1, IGF2BP3, ILF3, ILK, IMPDH1, IMPDH2, INCENP, IPO11, IQGAP1, IQGAP2, IQGAP3, IRF3, IST1, ITCH, ITGAV, ITPK1, KAT7, KEAP1, KIF11, KIF21A, KIF22, KIF23, KIF2C, KIF5B, KLC1, KNTC1, KRT18, KRT19, KRT8, LANCL2, LARS1, LAS1L, LCMT1, LDHB, LGALS1, LIMS1, LIN28A, LMAN1, LNPEP, LPP, LRBA, LRRK1, LSG1, LSM4, LYPLA2, MAGED2, MAN1A1, MAP1B, MAP1LC3A, MAP1S, MAP2K6, MAP4, MAPK1, MAPK14, MAPK8, MARS1, MBNL1, MCC, MCCC2, MCM5, MCM7, MCMBP, MDN1, ME1, MEAK7, MEMO1, METAP1, METTL1,	

					MEX3A, MICAL1, MICAL3, MOCS2, MON2, MPRIP, MSH6, MSN, MTAP, MTHFD1, MTMR14, MTMR6, MTR, MVB12A, MVD, MVK, MYD88, MYDGF, MYH10, MYH9, MYL9, MYO1C, MYO1E, MYO5A, MYO9B, NBAS, NCAM1, NCAPD2, NCAPG, NCKAP1, NDC80, NDRG1, NDRG2, NEDD4, NFKB1, NHLRC2, NIBAN1, NIBAN2, NIT2, NMT2, NOP58, NPM1, NPM3, NQO2, NSF, NSFL1C, NT5C2, NUCB2, NUDCD1, NUP107, NUP133, NUP160, NUP35, NXN, OGT, OPLAH, OPTN, ORC4, ORC5, OSBP, P4HA2, P4HB, PACS1, PAICS, PAK1, PALD1, PALLD, PAPSS2, PARD3, PARD6B, PARN, PARVA, PASK, PAXBP1, PC, PCBP2, PCCA, PCCB, PCYT1A, PDCD11, PDCL3, PDIA6, PDLIM5, PDLIM7, PDS5A, PDXK, PEA15, PES1, PFAS, PFKL, PFKM, PFKP, PGM2L1, PGM3, PHIP, PHPT1, PI4KA, PICALM, PIK3C2A, PIK3CA, PIK3R4, PIP4K2B, PIP4K2C, PKM, PLCB3, PLCG1, PLEC, PLEKHA5, PLEKHA7, PLIN3, PLK1, PLS3, PLSCR3, PNO1, PNP, PNPT1, POGLUT2, POGZ, POLA1, POLA2, POLD1, POLR1B, POLR3A, POLR3C, POLR3F, POR, PPFIBP1, PPID, PPM1B, PPM1F, PPP2R5A, PRDX1, PRDX4, PREPL, PRKAA1, PRKAB1, PRKACB, PRKACG, PRKAG1, PRKAR1A, PRKAR2A, PRKAR2B, PRKCA, PRKDC, PRMT3, PRPF38B, PRUNE1, PSIP1, PSMC6, PSMD1, PSMD2, PSMD9, PSME3, PTCD3, PTPMT1, PTPN1, PTPN12, PTPN2, PUS3, PXDN, PXK, PXMP2, PYCARD, PYCR3, QDPR, RAB14, RAB18, RAB1A, RAB23, RAB2A, RAB32, RAB8A, RABL6, RAC3, RAI14, RAN, RANBP2, RANGAP1, RAP1GDS1, RAP2A, RAP2B, RAP2C, RBMS2, RBP1, RBPMS, RCC2, RELA, RHEB, RHPN2, RIC1, RIMKLB, RNH1, ROCK2, RPL13A, RPL22, RPL27A, RPL4,	

					RPL5, RPL7A, RPN1, RPS2, RPS21, RPS6KA1, RPS6KA3, RPS6KA4, RPS8, RRP12, RTKN, SAMD4B, SARM1, SARS1, SBDS, SBF1, SCFD1, SCLY, SCPEP1, SCYL1, SEC13, SEC16A, SEC23A, SEC23B, SEC24A, SEC24B, SEC24C, SEC24D, SEC31A, SEC61G, SEH1L, SELENBP1, SEPTIN7, SERBP1, SERPINB6, SERPINB9, SESN2, SET, SH3GLB1, SHPK, SIRT1, SIRT2, SKA3, SKP2, SLAIN2, SLC2A1, SLC44A1, SMARCA5, SMC2, SMS, SNRPD3, SNX17, SNX3, SNX6, SNX9, SORBS1, SORBS3, SORD, SPAG9, SPATS2L, SPR, SPTAN1, SPTBN1, SPTBN2, SRC, SRM, SRP14, SRPK1, SRR, STAM2, STK38, STX12, STX5, STXBP2, STYX, SURF4, SYMPK, TACC3, TAGLN2, TAX1BP1, TBC1D17, TBC1D4, TCP1, TELO2, TFG, TGFB1I1, TGM2, THUMPD3, TIA1, TIGAR, TIPRL, TJP2, TKFC, TKT, TLN1, TMED9, TMEM214, TMF1, TNS3, TOLLIP, TOMM34, TPM1, TPM4, TPP2, TPX2, TRIP12, TSR1, TTC1, TTF2, TUBA4A, TUT1, TWF1, TXNL1, TXNRD1, UAP1, UBA6, UBE2G1, UBE2H, UBE2O, UBR4, UBR5, UFL1, UGDH, UGP2, UPF2, UPF3B, USP15, USP19, USP47, USP5, USP7, USP9X, VAMP2, VAMP3, VIM, VPS25, VPS26A, VPS26B, VPS35, VPS36, VPS53, VRK1, WAPL, WARS1, WRAP53, XPNPEP1, XPO5, XRCC5, XRCC6, XRN1, YAP1, YARS2, YKT6, YOD1, ZC3H15, ZC3HAV1, ZFYVE16, ZMYM2, ZWILCH]	
rRNA transcription	GO_BiologicalProcess-EBI-UniProt-GOA-ACAP-ARAP_13.05.2021_00h00	1.36E-05	31.57895	12	[CAVIN1, GTF3C1, GTF3C2, GTF3C3, GTF3C4, MACROH2A1, MARS1, NCL, NOL11, NPM3, POLR1B, SMARCA4]	Upregulation
sterol biosynthetic process	GO_BiologicalProcess-EBI-UniProt-GOA-ACAP-ARAP_13.0	2.26E-05	23.28767	17	[ACAT2, ACLY, APOE, CYB5R1, CYB5R3, CYP51A1, ERLIN2, FDPS, IDI1, LBR, LSS, MVD, MVK, NSDHL, POR, PRKAA1, RAN]	Downregulation

	5.2021_00h00					
nuclear body	GO_CellularComponent-EBI-UniProt-GOA-ACAP-ARAP_13.05.2021_00h00	2.53E-06	11.93511	103	[ACIN1, ACTN4, ANTXR1, API5, ATF7IP, ATR, BAZ1B, BPNT2, CALCOCO2, CBX5, CCNL2, CHEK2, CKAP4, DAXX, DDX20, DDX39B, DDX42, DDX46, DHX15, DHX8, DHX9, DRG1, ECT2, EFTUD2, EHMT1, EIF4A3, EPB41, ERBIN, FAM118B, FANCD2, GATAD2A, GEMIN4, GEMIN5, GNL3, HDAC4, HNRNPM, HP1BP3, INCENP, JADE1, KIF22, LARS1, LSG1, MAPK14, MCM2, MDC1, MKI67, MMAB, MOCS2, MTDH, MTREX, MYO1C, MYO1E, NCAPG2, NDRG1, NOP58, NPM1, ORC3, PABPN1, PARN, PARP1, PHPT1, PIAS4, POLR1B, PRKAA1, PRKCA, PRPF40A, PRPF8, PSPC1, RAD51, RBM15, RBM25, RIF1, RP2, RPA1, RPA2, SART3, SBF1, SF3A3, SF3B1, SIRT1, SLTM, SNRPA1, SNRPD3, SRPK1, SRSF1, SRSF11, SRSF7, SYMPK, TDP2, TELO2, TIMM50, TKT, TOLLIP, TPP2, TRIP12, TUT1, UBE2O, USP7, WRAP53, YARS2, ZMYM2, ZNF217, ZNF638]	Upregulation
protein domain specific binding	GO_MolecularFunction-EBI-UniProt-GOA-ACAP-ARAP_13.05.2021_00h00	1.75E-05	11.92412	88	[AATF, ABI1, ADAM10, AP2A2, AP2M1, ARHGAP1, ARHGAP5, ARL1, ATP1A1, ATP2B1, ATP2B2, ATP2B4, BAIAP2, CACYBP, CBL, CCT6A, CD2AP, CDC42, CHAF1A, CHEK1, CTNNB1, CXADR, DBNL, DDX20, DDX5, DDX6, DICER1, DLG1, DPYSL3, EHD2, EHMT1, EPB41L5, F11R, FKBP8, FN1, GIPC1, GPI, HNRNPM, HSP90AB1, HSPA2, HSPA5, ILK, IQGAP1, IRF3, IST1, KEAP1, KIF21A, LBR, LSM4, MAVS, MDC1, MICAL1, MVB12A, MYD88, MYH9, NCAPG, NEDD4, NSF, OCLN, OSBP, OSTF1, PAWR, PLSCR3, PRKAR1A, PRKAR2A, PRKAR2B, PRKCA, PRKDC, PTPN12, PYCARD, RAP2B, RCC2, RELA, RFC1, SIRT1, SPON1, SRC, SRR, SRSF7, STYX, TGM2, TJP2, TLN1, TRIM28, USP47, VIM, WARS1, ZMYND8]	No change

lamellipodium	GO_CellularComponent-EBI-UniProt-GOA-ACAP-ARAP_13.05.2021_00h00	9.66E-08	18.46847	41	[ABI1, ACTR2, AKT1, ANTXR1, ARAP3, BAIAP2, CAPRIN1, CD44, CDC42BPA, CDC42BPB, CDH2, CORO1C, CSPG4, CTNNB1, CYFIP1, DBNL, DPYSL3, FAT1, FERMT2, FSCN1, GLMN, GSN, ILK, IQGAP2, ITGAV, ITGB1, MCC, MYH10, NCAPG, NCKAP1, P4HB, PAK1, PALLD, PARVA, PDLIM4, PIK3CA, PLCG1, PPP1R9B, PXDN, RAC3, RDX]	Downregulation
endocytic vesicle	GO_CellularComponent-EBI-UniProt-GOA-ACAP-ARAP_13.05.2021_00h00	2.65E-07	15.55555	56	[ANXA11, ANXA3, AP2A1, AP2A2, AP2B1, AP2M1, AP2S1, APOE, APPL2, ARF6, CALR, CAMK2D, CAV1, CD2AP, CDC42, ECPAS, EGFR, EHD1, EHD2, EHD4, EPN2, FLNB, GIPC1, GOLIM4, GSN, HEATR5A, HLA-A, HSP90B1, HSPH1, ITGAV, KIF5B, LRP1, MYO1C, MYO1E, OCLN, PDIA3, PICALM, PIK3R4, PRKCSH, RAB14, RAB23, RAB32, RAB5B, RAB5C, RAB8A, SCARB2, SEC22B, SNX3, SPARC, STAM2, STX12, TF, VAMP2, VAMP3, VIM, VPS26B]	Downregulation
filopodium	GO_CellularComponent-EBI-UniProt-GOA-ACAP-ARAP_13.05.2021_00h00	1.15E-05	20.17544	23	[ABI1, ANTXR1, AP2A1, ARF6, B4GALT1, BAIAP2, CDC42, CXADR, CYFIP1, FARP1, FAT1, FSCN1, FXR1, IQGAP2, ITGA6, ITGAV, ITGB1, MSN, MYO5A, PPP1R9B, RDX, TWF1, UTRN]	Downregulation
RNA 3'-end processing	GO_BiologicalProcess-EBI-UniProt-GOA-ACAP-ARAP_13.05.2021_00h00	3.68E-06	18.51852	30	[CCNB1, CDC73, CPSF2, CPSF3, CSTF1, CSTF3, DDX39B, EIF4A3, ELAC2, EXOSC10, EXOSC2, EXOSC5, EXOSC7, HSD17B10, LIN28A, PABPN1, PARN, PNPT1, PTCD1, RPRD1A, RPS21, SRSF1, SRSF11, SRSF7, SSB, SUPT5H, SYMPK, TRMT10C, TUT1, UPF3B]	Upregulation
actin cytoskeleton reorganization	GO_BiologicalProcess-EBI-UniProt-GOA-ACAP-ARAP_13.05.2021_00h00	2.91E-06	21.23894	24	[ANTXR1, ANXA1, ARAP1, ARHGDIA, BAIAP2, CD2AP, CDC42, CDC42BPA, CDC42BPB, CXADR, EPS8, F11R, GAB1, GSN, IQGAP2, MCU, MYH9, PAK1, PARVA, PDLIM4, PHPT1, PTK7, PTPN1, RAP2A]	Downregulation
nucleotide-activated protein kinase complex	GO_CellularComponent-EBI-UniProt-GOA-ACAP-ARAP_13.0	1.24E-05	66.66666	6	[PRKAA1, PRKAB1, PRKAG1, PRKAR1A, PRKAR2A, SESN2]	Downregulation

	5.2021_00h00					
response to wounding	GO_BiologicalProcess-EBI-UniProt-GOA-ACAP-ARAP_13.05.2021_00h00	2.14E-06	12.5	89	[ABAT, ACTN1, AK4, ANO6, ANXA1, ANXA2, ANXA5, ANXA6, AP3B1, APOE, ARF4, ARFGEF1, B4GALT1, BPTF, C1QBP, CAPZA2, CASK, CASP3, CAV1, CBX5, CD44, CDC42, CDH3, CLASP1, COL1A1, COL1A2, COL5A1, DGKA, DPYSL3, DSP, EGFR, EHD1, EHD2, F11R, FERMT2, FGG, FKBP10, FN1, GATA6, GLMN, GSN, HDAC2, HMOX1, HSPB1, ILK, ITGA5, ITGB1, ITPK1, JAM3, KDM1A, LAMC1, LGALS1, LMAN1, LNPK, LRP1, MACF1, MAP1B, MAPK1, MAPK14, MCAM, METAP1, MICAL1, MTR, MYH9, MYL9, OCLN, PAK1, PDGFRB, PIK3CA, PLEC, PLPP3, PRKACB, PRKACG, PRKAR1A, PRKAR2A, PRKAR2B, PRKCA, PTK7, RANGAP1, RAP2B, SARM1, SLC1A3, SPARC, SRC, THBS1, TLN1, TPM1, YAP1, ZFP36L2]	Downregulation
wound healing	GO_BiologicalProcess-EBI-UniProt-GOA-ACAP-ARAP_13.05.2021_00h00	2.14E-06	13.21739	76	[ABAT, ACTN1, AK4, ANO6, ANXA1, ANXA2, ANXA5, ANXA6, AP3B1, APOE, ARFGEF1, B4GALT1, C1QBP, CAPZA2, CASK, CASP3, CAV1, CBX5, CD44, CDC42, CDH3, CLASP1, COL1A1, COL1A2, COL5A1, DGKA, DSP, EGFR, EHD1, EHD2, F11R, FERMT2, FGG, FKBP10, FN1, GATA6, GLMN, GSN, HDAC2, HMOX1, HSPB1, ILK, ITGA5, ITGB1, ITPK1, KDM1A, LMAN1, LNPK, MACF1, MAPK1, MAPK14, MCAM, METAP1, MICAL1, MYH9, MYL9, OCLN, PAK1, PDGFRB, PIK3CA, PLEC, PLPP3, PRKACB, PRKACG, PRKAR1A, PRKAR2A, PRKAR2B, PRKCA, PTK7, RAP2B, SPARC, SRC, THBS1, TLN1, TPM1, YAP1]	Downregulation
double-stranded DNA binding	GO_MolecularFunction-EBI-UniProt-GOA-ACAP-ARAP_13.05.2021_00h00	3.97E-07	4.117315	73	[ACTN4, AGO1, AGO2, BPTF, CCAR1, CTCF, CTNNB1, DDRGK1, DHX33, DHX9, DNMT3A, EGFR, FOXK1, GARS1, GATA6, HDAC4, HNRNPL, HSD17B4, HSPD1, IRF3, KAT7, KIF2C, MACROH2A1, MAPK1, MAPK8, MCM2, MCM3, MCM4, MCM5, MCM6, MCM7,	Upregulation

					MLH1, MSH2, MSH3, MSH6, MYBBP1A, NFKB1, NIBAN2, NPM1, NTHL1, ORC2, ORC3, ORC4, ORC5, PARP1, PATZ1, POLA1, POLR2A, POLR3F, PRKDC, PSIP1, PSPC1, PURA, RAD51, RBBP5, RBPJ, RELA, RFC1, SALL2, SARS1, SIN3A, SIRT1, SMARCA4, TBPL1, TDP1, XRCC5, XRCC6, XRN2, YAP1, ZFP36L2, ZNF217, ZNF281, ZNF638]	
sequence-specific DNA binding	GO_MolecularFunction-EBI-UniProt-GOA-ACAP-ARAP_13.05.2021_00h00	3.97E-07	4.292085	77	[ACTN4, AGO1, AGO2, BPTF, CCAR1, CTCF, CTNNB1, DDRGK1, DHX33, DHX9, DNMT1, DNMT3A, FOXK1, GARS1, GATA6, GATAD2A, HDAC2, HDAC4, HNRNPL, HSD17B4, HSPD1, IRF3, KAT7, KDM1A, KDM2A, KIF2C, LRWD1, MACROH2A1, MAPK8, MBD3, MCM2, MCM3, MCM4, MCM5, MCM6, MCM7, MSH2, MTA3, MYBBP1A, NCL, NFKB1, NIBAN2, NPM1, ORC2, ORC3, ORC4, ORC5, PARP1, PATZ1, POLA1, POLR1B, POLR2A, PSPC1, PURA, RBBP5, RBPJ, RELA, RFC1, RPA1, RPA2, SALL2, SARS1, SIN3A, SIRT1, SLTM, SMARCA4, TBPL1, TELO2, UPF2, VRTN, XRCC5, XRCC6, XRN2, YAP1, ZFP36L2, ZNF217, ZNF281]	Upregulation
ubiquitin-like protein ligase binding	GO_MolecularFunction-EBI-UniProt-GOA-ACAP-ARAP_13.05.2021_00h00	7.19E-08	16.3142	54	[AUP1, AURKA, BAG1, BAG2, CACYBP, CALR, CCNB1, CCT2, CDK5RAP3, CHEK2, CUL4A, CUL4B, CUL7, DAXX, DDRGK1, EGFR, ERLIN2, FAF2, GPI, HERC2, HSP90AB1, HSPA5, HSPA9, HSPD1, ITCH, LRPPRC, MAP1LC3A, PATZ1, PCBP2, PIAS4, POLR1B, POLR2A, PRKACB, PRKAR1A, PRKAR2A, PRKAR2B, PRKCA, PSMD1, RANGAP1, RELA, RPA2, RPL5, SLC25A5, SNX9, SRC, TCP1, TOLLIP, TRIM28, UBE2G1, USP19, USP7, WRAP53, XRCC5, YOD1]	Upregulation
ubiquitin protein ligase binding	GO_MolecularFunction-EBI-UniProt-GOA-ACAP-ARAP_13.05.2021_00h00	7.19E-08	16.02564	50	[AUP1, AURKA, BAG1, BAG2, CACYBP, CALR, CCT2, CHEK2, CUL4A, CUL4B, CUL7, DAXX, EGFR, ERLIN2, FAF2, GPI, HERC2, HSP90AB1, HSPA5, HSPA9, HSPD1, LRPPRC, MAP1LC3A, PATZ1, PCBP2, PIAS4,	Upregulation

					POLR1B, POLR2A, PRKACB, PRKAR1A, PRKAR2A, PRKAR2B, PRKCA, PSMD1, RANGAP1, RELA, RPA2, RPL5, SLC25A5, SNX9, SRC, TCP1, TOLLIP, TRIM28, UBE2G1, USP19, USP7, WRAP53, XRCC5, YOD1]	
nucleoside monophosphate metabolic process	GO_BiologicalProcess-EBI-UniProt-GOA-ACAP-ARAP_13.05.2021_00h00	8.19E-06	23.17073	19	[ADSL, AK1, AK4, AMPD2, APRT, ATIC, CASK, CMPK1, DCTD, DLG1, GART, GMPS, IMPDH1, IMPDH2, NT5C2, PAICS, PFAS, SORD, TJP2]	No change
IMP metabolic process	GO_BiologicalProcess-EBI-UniProt-GOA-ACAP-ARAP_13.05.2021_00h00	8.19E-06	47.05882	8	[ADSL, AMPD2, ATIC, GART, NT5C2, PAICS, PFAS, SORD]	Upregulation
regulation of protein stability	GO_BiologicalProcess-EBI-UniProt-GOA-ACAP-ARAP_13.05.2021_00h00	6.20E-12	19.36508	61	[ANK2, ATF7IP, AURKA, BAG1, BAG2, BAG3, CALR, CASP3, CCAR2, CCNH, CCT2, CCT3, CCT4, CCT5, CCT6A, CCT7, CCT8, CD81, CDC73, CDK7, CHEK2, COG7, CPNE2, CRTAP, DDRGK1, GET4, GIPC1, GPI, GSN, HCFC1, HSP90AB1, HSPD1, HTT, LSS, MAPK1, NPM1, P3H1, PARVA, PDCL3, PLK1, PLPP3, PPIB, PRKDC, PYCARD, QRSL1, RPL5, SEC16A, SEL1L, SH3GLB1, SIRT1, SRC, STX12, TCP1, TELO2, TF, USP19, USP47, USP7, USP9X, UTP25, VPS35]	No change
protein stabilization	GO_BiologicalProcess-EBI-UniProt-GOA-ACAP-ARAP_13.05.2021_00h00	6.20E-12	18.4466	38	[ANK2, ATF7IP, BAG1, BAG2, BAG3, CALR, CCNH, CCT2, CCT3, CCT4, CCT5, CCT6A, CCT7, CCT8, CDK7, CHEK2, COG7, CPNE2, CRTAP, GPI, HCFC1, HSP90AB1, HSPD1, NPM1, P3H1, PARVA, PDCL3, PLPP3, PPIB, RPL5, SEC16A, SEL1L, STX12, TCP1, TELO2, USP19, USP7, USP9X]	Upregulation
cell adhesion molecule binding	GO_MolecularFunction-EBI-UniProt-GOA-ACAP-ARAP_13.05.2021_00h00	2.42E-38	24.61274	143	[ABI1, ACTN1, ACTN4, ADAM10, ANK3, ANXA1, ANXA2, ARHGAP1, ARVCF, ASAP1, ATIC, BAG3, BAIAP2, BCAT2, BZW2, CALD1, CALR, CAST, CBL, CCT8, CD2AP, CD81, CDC42EP1, CDH2, CDH3, CGN, CKAP5, CNN3, COBLL1, COL5A1, CTNNB1, CXADR, DBNL, DDX6, DLG1, DSP, EEF2,	Downregulation

					EGFL6, EGFR, EHD1, EHD4, EMD, EPCAM, EPN2, EPS8L2, ESYT2, F11R, FERMT2, FGG, FLNB, FN1, FNBP1L, FSCN1, GIPC1, GLMN, GOLGA2, GOLGA3, HCFC1, HDLBP, HSP90AB1, HSPA5, ILK, IQGAP1, IST1, ITGA6, ITGAV, ITGB1, JAM3, KIF5B, KIRREL1, KRT18, KTN1, LAMB1, LAMC1, LASP1, LRRC59, LYPLA2, MACF1, MFGE8, MPRIP, MSN, MYH9, NCAPG, NDRG1, NECTIN2, NIBAN2, NLGN4X, NOP56, P4HB, PAICS, PALLD, PARVA, PDLIM5, PDXDC1, PFKP, PI4KA, PICALM, PKM, PKP2, PLCB3, PLEC, PLIN3, PLPP3, PPFIBP1, PRDX1, PRKCA, PTK7, PTPN1, PTPN2, PTPRD, RAB1A, RAN, RANGAP1, RDX, RPL7A, RPS2, S100A11, SCYL1, SEPTIN2, SEPTIN7, SERBP1, SH3GLB1, SLC9A3R2, SLK, SNX9, SPTAN1, SPTBN1, SPTBN2, SRC, STK38, STX5, TAGLN2, TENM3, THBS1, TJP2, TLN1, TLN2, TWF1, UBFD1, UTRN, YKT6, ZC3H15, ZC3HAV1]	
cadherin binding	GO_MolecularFunction-EBI-UniProt-GOA-ACAP-ARAP_13.05.2021_00h00	2.42E-38	30.33708	108	[ABI1, ANK3, ANXA1, ANXA2, ARHGAP1, ARVCF, ASAP1, ATIC, BAG3, BAIAP2, BZW2, CALD1, CAST, CBL, CCT8, CD2AP, CDC42EP1, CDH2, CDH3, CGN, CKAP5, CNN3, COBLL1, CTNNB1, DBNL, DDX6, DLG1, EEF2, EGFR, EHD1, EHD4, EMD, EPCAM, EPN2, EPS8L2, ESYT2, F11R, FLNB, FNBP1L, FSCN1, GIPC1, GOLGA2, GOLGA3, HCFC1, HDLBP, HSP90AB1, HSPA5, IQGAP1, IST1, ITGA6, ITGB1, KIF5B, KRT18, KTN1, LASP1, LRRC59, LYPLA2, MACF1, MPRIP, MYH9, NCAPG, NDRG1, NIBAN2, NOP56, PAICS, PARVA, PDLIM5, PDXDC1, PFKP, PI4KA, PICALM, PKM, PKP2, PLCB3, PLEC, PLIN3, PPFIBP1, PRDX1, PTPN1, RAB1A, RAN, RANGAP1, RDX, RPL7A, RPS2, S100A11, SCYL1, SEPTIN2, SEPTIN7, SERBP1, SH3GLB1, SLC9A3R2, SLK, SNX9, SPTAN1, SPTBN1, SPTBN2, SRC, STK38,	Downregulation

					STX5, TAGLN2, TJP2, TLN1, TWF1, UBFD1, YKT6, ZC3H15, ZC3HAV1]	
GTPase binding	GO_MolecularFunction-EBI-UniProt-GOA-ACAP-ARAP_13.05.2021_00h00	1.81E-06	15.53398	48	[ANKFY1, ANXA2, ARHGAP1, ARHGDIA, CAV1, CDC42BPB, CORO1C, CSE1L, CYFIP1, DIAPH1, DOCK7, ECT2, EHD1, EPS8, FARP1, FNBP1L, GDI1, GNB2, IPO11, IPO4, IPO5, IQGAP1, IQGAP2, IQGAP3, MARCHF5, MICAL1, MICAL3, MYO1C, MYO1E, MYO5A, MYO9B, NCKAP1, NDRG1, OPTN, PAK1, PICALM, RAB8A, RABGAP1L, RANBP2, RANGAP1, RCC2, RIC1, ROCK2, RTKN, SPTBN1, TNPO3, XPO5, XPO7]	Downregulation
small GTPase binding	GO_MolecularFunction-EBI-UniProt-GOA-ACAP-ARAP_13.05.2021_00h00	1.81E-06	16	44	[ANKFY1, ANXA2, ARHGAP1, ARHGDIA, CAV1, CDC42BPB, CORO1C, CSE1L, CYFIP1, DIAPH1, DOCK7, ECT2, EHD1, EPS8, FARP1, GDI1, IPO11, IPO4, IPO5, IQGAP1, IQGAP2, IQGAP3, MICAL1, MICAL3, MYO1C, MYO1E, MYO5A, MYO9B, NCKAP1, NDRG1, OPTN, PAK1, PICALM, RAB8A, RABGAP1L, RANBP2, RANGAP1, RCC2, RIC1, ROCK2, RTKN, TNPO3, XPO5, XPO7]	Downregulation
'de novo' protein folding	GO_BiologicalProcess-EBI-UniProt-GOA-ACAP-ARAP_13.05.2021_00h00	5.60E-10	30.23256	13	[BAG1, CCT2, ERO1A, HSPA13, HSPA14, HSPA2, HSPA5, HSPA9, HSPD1, HSPE1, HSPH1, SDF2L1, UGGT1]	No change
chaperone-mediated protein folding	GO_BiologicalProcess-EBI-UniProt-GOA-ACAP-ARAP_13.05.2021_00h00	5.60E-10	31.88406	22	[BAG1, CCT2, CRTAP, ERO1A, FKBP11, FKBP4, FKBP5, HSPA13, HSPA14, HSPA2, HSPA5, HSPA9, HSPB1, HSPE1, HSPH1, P3H1, PDCL3, PDIA4, PPIB, PPID, SDF2L1, TRAP1]	No change
cytokinesis	GO_BiologicalProcess-EBI-UniProt-GOA-ACAP-ARAP_13.05.2021_00h00	6.42E-06	17.91908	31	[ACTR2, ANK3, ANXA11, ARL3, AURKA, AURKB, BIRC6, CDC42, CUL7, ECT2, FSD1, GIPC1, INCENP, IST1, KIF20A, KIF23, MYH9, NUSAP1, PIK3R4, PLK1, PRPF40A, ROCK2, RTKN, SEPTIN10, SEPTIN11, SEPTIN2, SEPTIN7, SEPTIN8, SH3GLB1, SNX9, SPTBN1]	No change
cytoskeleton-dependent cytokinesis	GO_BiologicalProcess-EBI-UniProt-	6.42E-06	21	21	[ACTR2, ANK3, ARL3, AURKB, CUL7, ECT2, INCENP, IST1, KIF20A, KIF23, NUSAP1, PLK1,	No change

	GOA-ACAP-ARAP_13.05.2021_00h00				ROCK2, RTKN, SEPTIN10, SEPTIN11, SEPTIN2, SEPTIN7, SEPTIN8, SNX9, SPTBN1]	
signal transduction by p53 class mediator	GO_BiologicalProcess-EBI-UniProt-GOA-ACAP-ARAP_13.05.2021_00h00	2.59E-06	15.2381	48	[AKT1, ANTXR1, ATR, AURKA, AURKB, BOP1, CCNB1, CD44, CDK5RAP3, CHD4, CHEK1, CHEK2, CNOT11, DAXX, DDX5, EEF1E1, EHMT1, HDAC2, KDM1A, MAP2K6, MAPK14, MBD3, MMAB, MSH2, MYBBP1A, NDRG1, NPM1, PIP4K2B, POLR1B, PRKAA1, PRKAB1, PRKAG1, PYCARD, RFC2, RFC3, RFC5, RPA1, RPA2, RPA3, RPF2, RPL5, RRS1, SESN2, SIRT1, SSRP1, SUPT16H, TPX2, USP7]	Upregulation
regulation of signal transduction by p53 class mediator	GO_BiologicalProcess-EBI-UniProt-GOA-ACAP-ARAP_13.05.2021_00h00	2.59E-06	18.14159	41	[AKT1, ANTXR1, ATR, AURKA, AURKB, BOP1, CD44, CDK5RAP3, CHD4, CHEK1, CHEK2, DAXX, DDX5, EEF1E1, EHMT1, HDAC2, KDM1A, MAP2K6, MAPK14, MBD3, MMAB, NPM1, PIP4K2B, POLR1B, PRKAA1, PRKAB1, PRKAG1, RFC2, RFC3, RFC5, RPA1, RPA2, RPA3, RPF2, RPL5, RRS1, SIRT1, SSRP1, SUPT16H, TPX2, USP7]	Upregulation
mitochondrion	GO_CellularComponent-EBI-UniProt-GOA-ACAP-ARAP_13.05.2021_00h00	3.14E-11	11.79164	206	[AASS, ABAT, ABCB10, ABCB7, ABHD10, ACAD8, ACAD9, ACAT1, ACAT2, ACBD3, ACO1, ACOT13, ACOT9, ACSL1, ACSL4, ACSS2, ACSS3, AGTPBP1, AK4, AKT1, ALDH1L2, ALDH2, ALDH7A1, ANK2, ANXA1, ANXA6, AP3B1, APOOL, ARL2, ARSB, BCAT2, BDH2, C1QBP, CAPN1, CAPN2, CAT, CAVIN1, CCAR2, CCNB1, CDK5RAP1, CHPF, CPOX, CYB5R1, CYB5R3, DARS2, DDX21, DDX28, DDX6, DECR1, DEGS1, DHX30, DLGAP5, ELAC2, ETFA, ETHE1, EXD2, EXOG, FAHD1, FASTKD2, FDXR, FECH, FH, FKBP10, FKBP4, FKBP8, FLAD1, FOXRED1, GARS1, GBF1, GCDH, GCLC, GDAP1, GLS, GLUD1, GLUL, GOT2, GSTK1, GTF3C4, GUF1, H6PD, HADHA, HADHB, HAGH, HAT1, HDHD5, HEATR1, HIBADH, HK1, HK2, HMGCL, HOOK3, HSD17B10, HSDL2,	No change

					HSP90AB1, HSPA13, HSPA4, HSPA5, HSPA9, HSPD1, HSPE1, IDH2, ILF3, IRF3, JARID2, KANK2, L2HGDH, LARS2, LDHB, LIG1, LIG3, LRPPRC, LRRC59, LRRK1, MAIP1, MAPK1, MAPK14, MAPK8, MARCHF5, MAVS, MCCC2, MCU, ME1, MGST1, MICOS13, MIPEP, MMAB, MPC2, MRPS27, MRPS9, MTHFD1, NFKB1, NIF3L1, NLN, NNT, NOL6, NSUN2, NTHL1, OGT, OXCT1, P4HA1, PC, PCCA, PCCB, PGAM5, PITRM1, PKM, PLSCR3, PNPT1, PON2, POR, PRKCA, PRORP, PTCD1, PTCD3, PTPMT1, PTPN1, PUS1, PXMP2, PYCARD, PYCR1, PYCR2, QRSL1, RAB32, RAD51, RANBP2, RAP1GDS1, SARM1, SARS2, SBDS, SESN2, SH3GLB1, SIRT1, SLC25A12, SLC25A24, SLC25A3, SLC25A32, SLC25A5, SLC44A1, SLC44A2, SLIRP, SORD, SPARC, SRC, SSBP1, SUCLG1, TARS2, TBRG4, TEX10, TFB1M, TGM2, TIGAR, TIMM50, TIMMDC1, TOMM34, TRAP1, TRMT10C, TRMT5, TXNRD1, UNG, USP15, VAT1, VPS35, YARS2, YKT6, YRDC, ZNF217]	
mitochondrial matrix	GO_CellularComponent-EBI-UniProt-GOA-ACAP-ARAP_13.05.2021_00h00	3.14E-11	16.23762	82	[AASS, ABAT, ABHD10, ACAD8, ACAT1, ACOT9, ACSS2, ACSS3, AK4, ALDH1L2, ALDH2, ALDH7A1, ARL2, BCAT2, C1QBP, CCAR2, CCNB1, CHPF, DARS2, DDX28, DECR1, DHX30, ELAC2, ETFA, ETHE1, EXD2, FAHD1, FASTKD2, FDXR, FECH, FH, FLAD1, GARS1, GCDH, GLS, GLUD1, GOT2, GSTK1, GUF1, HADHA, HADHB, HAGH, HIBADH, HMGCL, HSD17B10, HSPA9, HSPD1, HSPE1, IDH2, LARS2, LRPPRC, LRRC59, MAIP1, MCCC2, MIPEP, MMAB, MRPS27, MRPS9, OXCT1, PC, PCCA, PCCB, PITRM1, PNPT1, PRORP, PTCD1, PTPN1, PUS1, PYCR1, PYCR2, RAD51, SARS2, SLC25A5, SSBP1, SUCLG1, TARS2, TBRG4, TFB1M, TRAP1, TRMT10C, TRMT5, YARS2]	No change

response to ionizing radiation	GO_BiologicalProcess-EBI-UniProt-GOA-ACAP-ARAP_13.05.2021_00h00	1.75E-05	17.57576	29	[ANTXR1, ANXA1, ATR, BRAT1, CASP3, CBL, CCND2, CHEK2, DNMT3A, DNMT3B, ECT2, FANCD2, HSPA5, IKBIP, MAPK14, MMAB, MSH2, PARP1, POLB, PRKAA1, PRKDC, PRPF38B, RAD51, SIRT1, TELO2, TIGAR, XRCC5, XRCC6, YAP1]	Upregulation
response to gamma radiation	GO_BiologicalProcess-EBI-UniProt-GOA-ACAP-ARAP_13.05.2021_00h00	1.75E-05	27.11865	16	[ANTXR1, ATR, CBL, CHEK2, FANCD2, HSPA5, MMAB, PARP1, POLB, PRKAA1, PRKDC, RAD51, TIGAR, XRCC5, XRCC6, YAP1]	Upregulation
macroautophagy	GO_BiologicalProcess-EBI-UniProt-GOA-ACAP-ARAP_13.05.2021_00h00	9.59E-07	15.18625	53	[AKT1, ATP6V1A, AUP1, BAG3, CALCOCO2, CAPN1, CAPNS1, CASP3, DDRGK1, DYNC1LI2, GPSM1, HMOX1, HTT, HUWE1, MAP1LC3A, MAPK8, MTMR14, MVB12A, NEDD4, NSFL1C, OPTN, PGAM5, PIK3CA, PIK3R4, PIP4K2B, PIP4K2C, PRKAA1, PRKAB1, PRKAG1, PRKCA, RAB1A, RAB23, RHEB, SCFD1, SEC22B, SESN2, SH3GLB1, SIRT1, SLC25A5, SNX6, SRC, STAM2, STX12, SUPT5H, TIGAR, UFL1, VIM, VPS25, VPS26A, VPS26B, VPS35, VPS36, YOD1]	Downregulation
regulation of macroautophagy	GO_BiologicalProcess-EBI-UniProt-GOA-ACAP-ARAP_13.05.2021_00h00	9.59E-07	20.2454	33	[AKT1, ATP6V1A, BAG3, CALCOCO2, CAPN1, CAPNS1, CASP3, GPSM1, HMOX1, HTT, MAPK8, NEDD4, OPTN, PIK3CA, PIP4K2B, PIP4K2C, PRKAA1, PRKAB1, PRKAG1, PRKCA, RHEB, SCFD1, SEC22B, SESN2, SH3GLB1, SIRT1, SLC25A5, SNX6, SUPT5H, TIGAR, VPS26A, VPS26B, VPS35]	Downregulation
leading edge membrane	GO_CellularComponent-EBI-UniProt-GOA-ACAP-ARAP_13.05.2021_00h00	1.21E-11	17.41294	35	[ANTXR1, APPL2, ARF4, ATP2B1, ATP2B2, ATP2B4, CD44, CDC42, CORO1C, CSPG4, DIAPH1, EGFR, EPB41L3, EPB41L5, EPS8, EPS8L2, FERMT2, GLMN, ITGA5, ITGAV, ITGB1, MACF1, MTMR6, MYO1C, MYO1E, NCKAP1, PAK1, PLCG1, PPP1R9B, SHISA8, SPTBN1, SRC, TLN1, TPM1, TWF1]	Downregulation
ruffle	GO_CellularComponent-EBI-UniProt-	1.21E-11	23.23232	46	[ACTN1, ANXA2, APPL2, ARAP3, ARF4, ARF6, BAIAP2, CD2AP, CORO1C, CSPG4,	Downregulation

	GOA-ACAP-ARAP_13.05.2021_00h00				CYFIP1, DBNL, DIAPH1, EGFR, EPB41L5, EPS8, EPS8L2, FSCN1, GLMN, GSN, IQGAP1, ITGA5, ITGAV, ITGB1, MACF1, MTMR14, MTMR6, MYADM, MYH9, MYO1C, MYO1E, MYO5A, NCAPG, NCKAP1, PAK1, PALLD, PDLIM7, PLCG1, PPP1R9B, S100A11, SNX9, SRC, TLN1, TLN2, TPM1, TWF1]	
ruffle membrane	GO_CellularComponent-EBI-UniProt-GOA-ACAP-ARAP_13.05.2021_00h00	1.21E-11	19.82759	23	[APPL2, ARF4, CORO1C, DIAPH1, EGFR, EPB41L5, EPS8, EPS8L2, GLMN, ITGA5, ITGAV, ITGB1, MACF1, MTMR6, MYO1C, MYO1E, PAK1, PLCG1, PPP1R9B, SRC, TLN1, TPM1, TWF1]	Downregulation
receptor catabolic process	GO_BiologicalProcess-EBI-UniProt-GOA-ACAP-ARAP_13.05.2021_00h00	3.06E-09	41.66667	15	[ANXA2, AP2A1, AP2A2, AP2B1, AP2M1, AP2S1, APOE, CAPN1, ITCH, MVB12A, NEDD4, PIK3R4, PTPN1, SH3GLB1, VLDLR]	Downregulation
low-density lipoprotein particle receptor catabolic process	GO_BiologicalProcess-EBI-UniProt-GOA-ACAP-ARAP_13.05.2021_00h00	3.06E-09	57.14286	8	[ANXA2, AP2A1, AP2A2, AP2B1, AP2M1, AP2S1, APOE, VLDLR]	Downregulation
clathrin coat of endocytic vesicle	GO_CellularComponent-EBI-UniProt-GOA-ACAP-ARAP_13.05.2021_00h00	3.06E-09	63.63636	7	[AP2A1, AP2A2, AP2B1, AP2M1, AP2S1, EPN2, PICALM]	Downregulation
membrane-bounded organelle	GO_CellularComponent-EBI-UniProt-GOA-ACAP-ARAP_13.05.2021_00h00	3.20E-97	9.913825	1323	[AAAS, AASS, AATF, ABAT, ABCB10, ABCB7, ABCC1, ABCF1, ABHD10, ABHD12, ABHD14B, ABI1, ACAA1, ACACA, ACAD8, ACAD9, ACAT1, ACAT2, ACBD3, ACIN1, ACLY, ACO1, ACOT13, ACOT9, ACOX3, ACSL1, ACSL4, ACSS2, ACSS3, ACTBL2, ACTN1, ACTN4, ACTR10, ACTR1B, ACTR2, ADAM10, ADAR, ADARB1, ADD2, ADD3, ADPGK, ADSL, AEBP2, AGL, AGO1, AGO2, AGTPBP1, AIMP2, AK1, AK4, AKR7A2, AKT1, ALCAM, ALDH1L2, ALDH2, ALDH7A1, ALDOC, ALG11, ALG2, ALG5, ALG9, ALPL, AMDHD2, ANK2, ANK3, ANKFY1, ANKRD28,	No change

					ANKS1A, ANO6, ANP32E, ANTXR1, ANXA1, ANXA11, ANXA2, ANXA3, ANXA4, ANXA5, ANXA6, AP1M1, AP1S1, AP2A1, AP2A2, AP2B1, AP2M1, AP2S1, AP3B1, APAF1, APEH, API5, APOBEC3C, APOE, APOOL, APPL2, APRT, ARAP1, ARCN1, ARF4, ARF6, ARFGAP1, ARFGAP3, ARFGEF1, ARHGAP1, ARHGAP5, ARHGDIA, ARHGEF12, ARL1, ARL2, ARL3, ARL6IP5, ARPC1A, ARSA, ARSB, ARVCF, ASL, ASPH, ATF7IP, ATIC, ATL2, ATL3, ATP1A1, ATP2B1, ATP2B2, ATP2B4, ATP6V1A, ATR, AUP1, AURKA, AURKB, B3GLCT, B4GALT1, BAG1, BAG3, BAIAP2, BAZ1B, BCAT2, BCCIP, BDH2, BET1, BIN1, BIRC6, BLVRB, BMS1, BOP1, BPNT2, BPTF, BRAT1, BRD3, BRD4, BRMS1, BRWD1, BUB1B, C1QBP, CACYBP, CALCOCO2, CALR, CALU, CAMK1, CAMK2D, CANX, CAP1, CAPN1, CAPN2, CAPNS1, CAPZA2, CARNMT1, CASK, CASP3, CAST, CAT, CAV1, CAVIN1, CBL, CBR1, CBX2, CBX5, CCAR1, CCAR2, CCDC22, CCNB1, CCND2, CCNH, CCNL2, CCNY, CCT2, CCT3, CCT4, CCT5, CCT6A, CCT7, CCT8, CD2AP, CD44, CD81, CDC42, CDC42BPA, CDC42BPB, CDC73, CDCA8, CDH2, CDK5RAP1, CDK5RAP3, CDK7, CEBPZ, CELF1, CEP170, CERCAM, CFAP20, CFAP298, CFL2, CHAF1A, CHD1, CHD4, CHEK1, CHEK2, CHID1, CHPF, CHST14, CHTF18, CIRBP, CKAP4, CKAP5, CLASP1, CMBL, CMPK1, CNDP2, CNOT11, COBLL1, COG1, COG6, COG7, COG8, COL1A1, COL1A2, COL4A1, COL4A2, COL5A1, COL5A2, COL6A1, COL6A2, COL6A3, COLGALT1, COMMD4, COPA, COPB1, COPB2, COPE, COPG1, COPG2, COPS2, COPS5, COPS7A, COPZ1, CORO1C, CPD, CPNE2, CPOX, CPSF2, CPSF3, CRLF3, CRMP1,	

					CRTAP, CSDE1, CSE1L, CSPG4, CSRP2, CSTF1, CSTF3, CTBP2, CTCF, CTNNB1, CTNNBL1, CTSC, CUL4A, CUL4B, CUL7, CWC27, CXADR, CYB5R1, CYB5R3, CYFIP1, CYP2S1, CYP51A1, DAGLB, DAPK1, DARS2, DAXX, DAZAP1, DBNL, DCAF1, DCAF13, DCTN1, DCTN4, DCXR, DDRGK1, DDX10, DDX18, DDX20, DDX21, DDX24, DDX28, DDX31, DDX39B, DDX41, DDX42, DDX46, DDX47, DDX49, DDX5, DDX52, DDX54, DDX6, DECR1, DEGS1, DHRS7B, DHX15, DHX30, DHX33, DHX37, DHX8, DHX9, DIAPH1, DICER1, DIPK2A, DLG1, DLGAP5, DNMT1, DNMT3A, DNMT3B, DNTTIP2, DOCK7, DPH1, DPH6, DPP3, DPPA4, DPYSL2, DPYSL3, DRG1, DSP, DSTN, DUSP3, DYNC1LI2, ECE1, ECPAS, ECT2, EDEM3, EDRF1, EEA1, EEF1E1, EEF2, EFTUD2, EGFR, EHD1, EHD2, EHD4, EHMT1, EIF3A, EIF4A3, EIF5B, ELAC2, ELP1, ELP3, EMD, EMSY, ENDOD1, ENO2, ENOPH1, EPB41, EPB41L5, EPCAM, EPM2AIP1, EPN2, EPS8, EPS8L2, ERAP1, ERBIN, ERCC2, ERGIC1, ERGIC2, ERLEC1, ERLIN2, ERO1A, ERP44, ESD, ESF1, ESYT2, ETFA, ETHE1, EXD2, EXOG, EXOSC10, EXOSC2, EXOSC5, EXOSC7, F11R, FAF2, FAHD1, FAM114A1, FAM118B, FAM120A, FANCD2, FANCI, FASTKD2, FAT1, FBXO2, FBXW8, FDPS, FDXR, FECH, FERMT2, FGG, FH, FHL1, FKBP10, FKBP14, FKBP15, FKBP4, FKBP5, FKBP7, FKBP8, FKBP9, FLAD1, FLNB, FLT1, FN1, FNBP1L, FNDC3A, FOXK1, FOXRED1, FSCN1, FSD1, FTH1, FTSJ3, FUT11, FXR1, G3BP1, GALK1, GALM, GALNT1, GALNT10, GALNT2, GARS1, GART, GATA6, GATAD2A, GATD1, GBE1, GBF1, GCDH, GCLC, GDAP1, GDI1, GEMIN4, GEMIN5, GET3, GET4, GINS3, GIPC1, GIPC2, GLA, GLB1L3, GLS, GLT8D1, GLUD1, GLUL,	

					GMPPA, GNB2, GNG12, GNL2, GNL3, GNPDA2, GNS, GOLGA2, GOLGA3, GOLIM4, GOLT1B, GOPC, GOSR2, GOT2, GPC1, GPC3, GPC6, GPD1L, GPI, GPSM1, GPX7, GPX8, GSN, GSS, GSTK1, GSTM2, GSTM3, GTF2E1, GTF2I, GTF3C1, GTF3C2, GTF3C3, GTF3C4, GUF1, H6PD, HACL1, HADHA, HADHB, HAGH, HAT1, HCFC1, HDAC2, HDAC4, HDHD5, HDLBP, HEATR1, HEATR5A, HELLS, HERC2, HEXA, HIBADH, HK1, HK2, HLA-A, HMGCL, HMOX1, HMOX2, HNRNPF, HNRNPL, HNRNPLL, HNRNPM, HOOK3, HP1BP3, HSD17B10, HSD17B4, HSDL2, HSP90AB1, HSP90B1, HSPA13, HSPA14, HSPA2, HSPA4, HSPA5, HSPA9, HSPB1, HSPB11, HSPD1, HSPE1, HSPH1, HTRA1, HTT, HUWE1, IDH2, IDI1, IGF2BP3, IKBIP, ILF2, ILF3, ILK, ILVBL, IMP3, IMPDH1, IMPDH2, INCENP, INO80C, IPO11, IPO4, IPO5, IQGAP1, IQGAP2, IRF2BPL, IRF3, IST1, ITCH, ITGAV, ITGB1, IWS1, JADE1, JAM3, JARID2, KANK2, KAT7, KDELR1, KDELR3, KDM1A, KDM2A, KDM3B, KEAP1, KIF11, KIF20A, KIF22, KIF23, KIF2C, KIF5B, KLC1, KNTC1, KRT18, KRT19, KRT8, KTN1, L1TD1, L2HGDH, LAMB1, LAMC1, LANCL2, LARS1, LARS2, LAS1L, LBR, LCMT1, LDHB, LGALS1, LIG1, LIG3, LIN28A, LMAN1, LMCD1, LMF2, LNPEP, LNPK, LPCAT1, LPGAT1, LPP, LRBA, LRP1, LRPPRC, LRRC40, LRRC57, LRRC59, LRRC8A, LRRK1, LRWD1, LSG1, LSM4, LSS, LTBP1, LYAR, LYPLA2, MACF1, MACROH2A1, MAGED1, MAGED2, MAIP1, MAN1A1, MAN1A2, MANBA, MAP1B, MAP1LC3A, MAP1S, MAP2K6, MAP4, MAPK1, MAPK14, MAPK8, MARCHF5, MARS1, MASTL, MATR3, MAVS, MBD3, MBLAC2, MBNL1, MCAM, MCC, MCCC2,	

					MCM2, MCM3, MCM4, MCM5, MCM6, MCM7, MCMBP, MCU, MDC1, MDN1, ME1, MEAK7, MEMO1, MESD, MEST, METTL1, MEX3A, MFGE8, MFSD10, MGAT2, MGST1, MICAL3, MICOS13, MIPEP, MKI67, MLH1, MMAB, MME, MOCS2, MOGS, MON2, MOSPD2, MOXD1, MPC2, MPHOSPH10, MRPS27, MRPS9, MSH2, MSH3, MSH6, MSI1, MSN, MTA3, MTAP, MTDH, MTHFD1, MTMR6, MTREX, MVB12A, MVD, MVK, MYBBP1A, MYD88, MYDGF, MYEF2, MYH10, MYH9, MYO1C, MYO1E, MYO5A, NBAS, NCAM1, NCAPD2, NCAPG, NCAPG2, NCKAP1, NCL, NDC1, NDC80, NDRG1, NDRG2, NECAP2, NECTIN2, NEDD4, NEK7, NFKB1, NHLRC2, NIBAN1, NIBAN2, NID1, NIF3L1, NIT2, NLE1, NLN, NMT2, NNT, NOL10, NOL11, NOL6, NOL9, NOP14, NOP56, NOP58, NOP9, NPM1, NPM3, NQO2, NSDHL, NSF, NSFL1C, NSUN2, NSUN5, NTHL1, NUCB2, NUDCD1, NUDT16, NUP107, NUP133, NUP160, NUP210, NUP35, NUP50, NUSAP1, NVL, NXN, OCLN, OGT, OPTN, ORC2, ORC3, ORC4, ORC5, OSBP, OSTC, OSTF1, OXCT1, P3H1, P3H3, P3H4, P4HA1, P4HA2, P4HB, P4HTM, PABPN1, PACS1, PAICS, PAK1, PALLD, PARD6B, PARN, PARP1, PARVA, PASK, PATZ1, PAWR, PAXBP1, PC, PCBP2, PCCA, PCCB, PCOLCE, PCYT1A, PCYT2, PDCD11, PDCD2, PDCL3, PDGFRB, PDIA3, PDIA4, PDIA6, PDLIM2, PDLIM4, PDLIM7, PDS5A, PDXDC1, PDXK, PEA15, PELP1, PES1, PFAS, PFKL, PFKM, PFKP, PGAM5, PGRMC2, PHC1, PHIP, PHPT1, PI4KA, PIAS4, PICALM, PIGS, PIGT, PIK3C2A, PIK3R4, PIP4K2B, PIP4K2C, PITRM1, PKM, PKP2, PLCB3, PLCG1, PLD3, PLEC, PLEKHA5, PLEKHA7, PLIN3, PLK1,	

					PLOD1, PLOD2, PLOD3, PLPP3, PLSCR3, PLXNB2, PM20D2, PNO1, PNP, PNPT1, POFUT1, POFUT2, POGLUT2, POGLUT3, POGZ, POLA1, POLA2, POLB, POLD1, POLE, POLR1B, POLR2A, POLR2B, POLR2G, POLR3A, POLR3C, POLR3F, PON2, POR, PPIB, PPIC, PPID, PPM1B, PPM1F, PPM1G, PPP1R7, PPP1R9B, PPP2R5A, PRAF2, PRDX1, PRDX4, PREPL, PRIM1, PRIM2, PRKAA1, PRKAB1, PRKACB, PRKACG, PRKAG1, PRKAR1A, PRKAR2A, PRKAR2B, PRKCA, PRKCSH, PRKDC, PRORP, PRPF38A, PRPF38B, PRPF40A, PRPF8, PRRC1, PRUNE1, PSIP1, PSMC6, PSMD1, PSMD2, PSMD9, PSME3, PSPC1, PTBP2, PTCD1, PTCD3, PTGIS, PTPMT1, PTPN1, PTPN12, PTPN2, PTPRD, PURA, PUS1, PUS3, PUS7, PWP2, PXDN, PXMP2, PYCARD, PYCR1, PYCR2, QDPR, QRSL1, RAB14, RAB18, RAB1A, RAB23, RAB2A, RAB32, RAB5B, RAB5C, RAB8A, RABGAP1L, RABL6, RAC3, RAD51, RAD54L2, RAI14, RAN, RANBP2, RANGAP1, RAP1GDS1, RAP2A, RAP2B, RAP2C, RBBP5, RBM15, RBM25, RBM26, RBM3, RBM6, RBMS2, RBP1, RBPJ, RBPMS, RCC2, RCN1, RDH10, RDX, RELA, RETSAT, RFC1, RFC2, RFC3, RFC5, RFTN1, RHEB, RIC1, RIF1, RIOX1, RNF170, RNH1, RNPEP, ROCK2, RP2, RPA1, RPA2, RPA3, RPF2, RPL13A, RPL22, RPL27A, RPL4, RPL5, RPL7A, RPN1, RPRD1A, RPS2, RPS21, RPS6KA1, RPS6KA3, RPS6KA4, RPS8, RRAS, RRBP1, RRP12, RRP7A, RRS1, RTCA, RTTN, S100A10, S100A11, SAAL1, SALL2, SAMD4B, SAMHD1, SAP30BP, SAR1A, SARM1, SARS1, SARS2, SART3, SBDS, SBF1, SCAMP2, SCARB2, SCFD1, SCFD2, SCLY, SCPEP1, SCRN1, SCYL1, SDAD1, SDF2L1, SDF4, SEC13, SEC16A, SEC22B, SEC23A, SEC23B, SEC24A, SEC24B,	

					SEC24C, SEC24D, SEC31A, SEC61G, SEH1L, SEL1L, SELENBP1, SEPHS1, SEPTIN2, SEPTIN7, SEPTIN8, SERBP1, SERPINB6, SERPINB9, SERPINH1, SESN2, SET, SETD7, SETDB1, SF3A3, SF3B1, SH3GL3, SH3GLB1, SIL1, SIN3A, SIRT1, SIRT2, SKP2, SLC12A4, SLC1A3, SLC25A12, SLC25A24, SLC25A3, SLC25A32, SLC25A5, SLC2A1, SLC33A1, SLC44A1, SLC44A2, SLC4A7, SLC7A6, SLC9A3R2, SLIRP, SLK, SLTM, SMARCA4, SMARCA5, SMARCAD1, SMARCC1, SMARCD1, SMARCD2, SMC2, SMPDL3B, SMS, SNRNP200, SNRPA1, SNRPD3, SNTB2, SNX17, SNX3, SNX4, SNX6, SNX9, SORBS1, SORBS3, SORD, SPAG9, SPARC, SPATS2L, SPON1, SPR, SPTAN1, SPTBN1, SPTLC2, SRC, SRP14, SRPK1, SRPRA, SRPRB, SRSF1, SRSF11, SRSF7, SSB, SSBP1, SSR1, SSR4, SSRP1, STAM2, STIM1, STK38, STT3A, STT3B, STX12, STX5, STXBP2, STYX, SUCLG1, SUMF2, SUN2, SUPT16H, SUPT5H, SUPT6H, SURF4, SYMPK, TACC3, TAGLN2, TARS2, TAX1BP1, TBC1D17, TBC1D4, TBL2, TBL3, TBPL1, TBRG4, TCP1, TDP1, TDP2, TELO2, TEX10, TEX2, TF, TFB1M, TFG, TGFB1I1, TGM2, THBS1, THUMPD3, TIA1, TIGAR, TIMM50, TIMMDC1, TIMP3, TJP2, TKFC, TKT, TLK1, TLN1, TM9SF4, TMA16, TMED10, TMED2, TMED5, TMED7, TMED9, TMEM115, TMEM167A, TMEM168, TMEM192, TMEM214, TMEM43, TMEM97, TMF1, TMX3, TMX4, TNPO3, TOLLIP, TOMM34, TOP2A, TPM4, TPP2, TPST1, TPX2, TRAM1, TRAP1, TRIM28, TRIM33, TRIP12, TRIP13, TRMT1, TRMT10C, TRMT1L, TRMT5, TSEN34, TSR1, TTC1, TTC38, TTF2, TTI1, TTK, TTLL12, TUBA4A, TUBB6, TUT1, TWF1, TXNDC5, TXNL1, TXNRD1, UAP1, UBA6, UBE2G1, UBE2H,	

					UBE2O, UBN2, UBR4, UBR5, UFL1, UGDH, UGGT1, UGGT2, UGP2, UNG, UPF2, UPF3B, USE1, USP15, USP19, USP47, USP5, USP7, USP9X, UTP15, UTP18, UTP20, UTP25, UTP4, UTRN, VAMP2, VAMP3, VAT1, VIM, VLDLR, VPS25, VPS26A, VPS26B, VPS26C, VPS35, VPS35L, VPS36, VPS53, VRK1, VWA5A, WAPL, WARS1, WDHD1, WDR18, WDR3, WDR36, WDR37, WDR43, WRAP53, XPNPEP1, XPO5, XPO7, XRCC5, XRCC6, XRN1, XRN2, YAP1, YARS2, YIF1A, YKT6, YRDC, YTHDC2, ZC3H15, ZC3H7B, ZC3HAV1, ZDHHC17, ZFP36L2, ZFYVE16, ZMYM2, ZMYM3, ZMYND8, ZNF217, ZNF281, ZNF462, ZNF532, ZNF638]	
intracellular organelle	GO_CellularComponent-EBI-UniProt-GOA-ACAP-ARAP_13.05.2021_00h00	3.20E-97	9.88024	1320	[AAAS, AASS, AATF, ABAT, ABCB10, ABCB7, ABCF1, ABHD10, ABHD12, ABHD14B, ABI1, ACAA1, ACACA, ACAD8, ACAD9, ACAT1, ACAT2, ACBD3, ACIN1, ACLY, ACO1, ACOT13, ACOT9, ACOX3, ACSL1, ACSL4, ACSS2, ACSS3, ACTBL2, ACTN1, ACTN4, ACTR10, ACTR1B, ACTR2, ADAM10, ADAR, ADARB1, ADD2, ADD3, ADPGK, AEBP2, AGL, AGO1, AGO2, AGTPBP1, AIMP2, AK1, AK4, AKR7A2, AKT1, ALDH1L2, ALDH2, ALDH7A1, ALDOC, ALG11, ALG2, ALG5, ALG9, AMDHD2, ANK2, ANK3, ANKFY1, ANKRD28, ANKS1A, ANO6, ANP32E, ANTXR1, ANXA1, ANXA11, ANXA2, ANXA3, ANXA4, ANXA6, AP1M1, AP1S1, AP2A1, AP2A2, AP2B1, AP2M1, AP2S1, AP3B1, APAF1, APEH, API5, APOBEC3C, APOE, APOOL, APPL2, APRT, ARAP1, ARAP3, ARCN1, ARF4, ARF6, ARFGAP1, ARFGAP3, ARFGEF1, ARHGAP1, ARHGAP5, ARHGDIA, ARL1, ARL2, ARL3, ARL6IP5, ARPC1A, ARSA, ARSB, ARVCF, ASAP1, ASPH, ATF7IP, ATL2, ATL3, ATP1A1, ATP2B1, ATP2B2, ATP2B4, ATP6V1A, ATR,	No change

					AUP1, AURKA, AURKB, B3GLCT, B4GALT1, BAG1, BAG2, BAG3, BAIAP2, BAZ1B, BCAT2, BCCIP, BDH2, BET1, BIN1, BIRC6, BLVRB, BMS1, BOP1, BPNT2, BPTF, BRAT1, BRD3, BRD4, BRMS1, BRWD1, BUB1B, C1QBP, CACYBP, CALCOCO2, CALD1, CALR, CALU, CAMK1, CAMK2D, CANX, CAP1, CAPN1, CAPN2, CAPRIN1, CAPZA2, CARHSP1, CARNMT1, CASK, CASP3, CAST, CAT, CAV1, CAVIN1, CBL, CBX2, CBX5, CCAR1, CCAR2, CCDC22, CCNB1, CCND2, CCNH, CCNL2, CCNY, CCT2, CCT3, CCT4, CCT5, CCT6A, CCT7, CCT8, CD2AP, CD44, CDC42, CDC42BPA, CDC42BPB, CDC42EP1, CDC42EP3, CDC73, CDCA8, CDH2, CDK5RAP1, CDK5RAP3, CDK7, CEBPZ, CELF1, CEP170, CERCAM, CFAP20, CFAP298, CFL2, CGN, CHAF1A, CHD1, CHD4, CHEK1, CHEK2, CHID1, CHPF, CHST14, CHTF18, CIRBP, CKAP4, CKAP5, CLASP1, CMPK1, CNDP2, CNN3, CNOT11, COG1, COG6, COG7, COG8, COL1A1, COL1A2, COL4A1, COL4A2, COL5A1, COL5A2, COL6A1, COL6A2, COL6A3, COLGALT1, COMMD4, COPA, COPB1, COPB2, COPE, COPG1, COPG2, COPS2, COPS5, COPS7A, COPZ1, CORO1C, CPNE2, CPOX, CPSF2, CPSF3, CRLF3, CRMP1, CRTAP, CSDE1, CSE1L, CSPG4, CSRP2, CSTF1, CSTF3, CTBP2, CTCF, CTNNB1, CTNNBL1, CTSC, CUL4A, CUL4B, CUL7, CWC27, CXADR, CYB5R1, CYB5R3, CYFIP1, CYP2S1, CYP51A1, DAGLB, DAPK1, DARS2, DAXX, DAZAP1, DBNL, DCAF1, DCAF13, DCTN1, DCTN4, DCXR, DDRGK1, DDX10, DDX18, DDX20, DDX21, DDX24, DDX28, DDX31, DDX39B, DDX41, DDX42, DDX46, DDX47, DDX49, DDX5, DDX52, DDX54, DDX6, DECR1, DEGS1, DHRS7B, DHX15, DHX30, DHX33, DHX37,	

					DHX8, DHX9, DIAPH1, DICER1, DIPK2A, DLG1, DLGAP5, DNMT1, DNMT3A, DNMT3B, DNTTIP2, DOCK7, DPH1, DPH6, DPPA4, DPYSL2, DPYSL3, DRG1, DSP, DSTN, DUSP3, DYNC1LI2, ECE1, ECPAS, ECT2, EDEM3, EDRF1, EEA1, EEF1E1, EEF2, EFTUD2, EGFR, EHD1, EHD2, EHD4, EHMT1, EIF3A, EIF4A3, EIF5B, ELAC2, ELP1, ELP3, EMD, EML1, EMSY, ENOPH1, EPB41, EPB41L3, EPB41L5, EPM2AIP1, EPN2, ERAP1, ERBIN, ERCC2, ERCC6L, ERGIC1, ERGIC2, ERLEC1, ERLIN2, ERO1A, ERP44, ESD, ESF1, ESYT2, ETFA, ETHE1, EXD2, EXOG, EXOSC10, EXOSC2, EXOSC5, EXOSC7, F11R, FAF2, FAHD1, FAM114A1, FAM118B, FAM120A, FANCD2, FANCI, FARP1, FASTKD2, FAT1, FBXO2, FBXW8, FDPS, FDXR, FECH, FERMT2, FGG, FH, FHL1, FKBP10, FKBP14, FKBP15, FKBP4, FKBP5, FKBP7, FKBP8, FKBP9, FLAD1, FLNB, FLNC, FLT1, FN1, FNBP1L, FNDC3A, FOXK1, FOXRED1, FSCN1, FSD1, FTH1, FTSJ3, FUT11, FXR1, G3BP1, G3BP2, GALNT1, GALNT10, GALNT2, GARS1, GATA6, GATAD2A, GBF1, GCDH, GCLC, GDAP1, GDI1, GEMIN4, GEMIN5, GET3, GET4, GINS3, GIPC1, GLA, GLB1L3, GLS, GLT8D1, GLUD1, GLUL, GNB2, GNG12, GNL2, GNL3, GNPDA2, GNS, GOLGA2, GOLGA3, GOLIM4, GOLT1B, GOPC, GOSR2, GOT2, GPC1, GPC3, GPC6, GPHN, GPI, GPSM1, GPX7, GPX8, GSN, GSTK1, GSTM2, GSTM3, GTF2E1, GTF2I, GTF3C1, GTF3C2, GTF3C3, GTF3C4, GUF1, H6PD, HACL1, HADHA, HADHB, HAGH, HAT1, HCFC1, HDAC2, HDAC4, HDHD5, HDLBP, HEATR1, HEATR5A, HELLS, HERC2, HEXA, HIBADH, HK1, HK2, HLA-A, HMGCL, HMMR, HMOX1, HMOX2, HNRNPF, HNRNPL,	

					HNRNPLL, HNRNPM, HOOK3, HP1BP3, HSD17B10, HSD17B4, HSDL2, HSP90AB1, HSP90B1, HSPA13, HSPA14, HSPA2, HSPA4, HSPA5, HSPA9, HSPB1, HSPB11, HSPD1, HSPE1, HSPH1, HTT, HUWE1, IDH2, IDI1, IGF2BP3, IKBIP, ILF2, ILF3, ILK, ILVBL, IMP3, IMPDH1, IMPDH2, INCENP, INO80C, IPO11, IPO4, IPO5, IQGAP1, IQGAP2, IRF2BPL, IRF3, IST1, ITCH, ITGAV, ITGB1, IWS1, JADE1, JAM3, JARID2, KANK2, KAT7, KDELR1, KDELR3, KDM1A, KDM2A, KDM3B, KEAP1, KIF11, KIF20A, KIF21A, KIF22, KIF23, KIF2C, KIF5B, KLC1, KNTC1, KRT18, KRT19, KRT8, KTN1, L1TD1, L2HGDH, LAMB1, LAMC1, LANCL2, LARS1, LARS2, LAS1L, LASP1, LBR, LCMT1, LDHB, LGALS1, LIG1, LIG3, LIN28A, LMAN1, LMCD1, LMF2, LNPEP, LNPK, LPCAT1, LPGAT1, LPP, LRBA, LRP1, LRPPRC, LRRC40, LRRC59, LRRC8A, LRRK1, LRWD1, LSG1, LSM4, LSS, LTBP1, LYAR, LYPLA2, MACF1, MACROH2A1, MAGED1, MAGED2, MAIP1, MAN1A1, MAN1A2, MANBA, MAP1B, MAP1LC3A, MAP1S, MAP2K6, MAP4, MAPK1, MAPK14, MAPK8, MARCHF5, MARS1, MASTL, MATR3, MAVS, MBD3, MBNL1, MCAM, MCC, MCCC2, MCM2, MCM3, MCM4, MCM5, MCM6, MCM7, MCMBP, MCU, MDC1, MDN1, ME1, MEAK7, MEMO1, MESD, MEST, METAP1, METTL1, MEX3A, MFGE8, MFSD10, MGAT2, MGST1, MICAL1, MICAL3, MICOS13, MIPEP, MKI67, MLH1, MMAB, MME, MOCS2, MOGS, MON2, MOSPD2, MOXD1, MPC2, MPHOSPH10, MPRIP, MRPS27, MRPS9, MSH2, MSH3, MSH6, MSI1, MSN, MTA3, MTAP, MTDH, MTHFD1, MTMR6, MTREX, MVB12A, MVD, MVK, MYADM, MYBBP1A, MYD88, MYDGF,	

					MYEF2, MYH10, MYH9, MYL9, MYO1C, MYO1E, MYO5A, MYO9B, NBAS, NCAM1, NCAPD2, NCAPG, NCAPG2, NCKAP1, NCL, NDC1, NDC80, NDRG1, NDRG2, NECAP2, NEDD4, NEK7, NFKB1, NHLRC2, NIBAN2, NIF3L1, NIT2, NLE1, NLN, NMT2, NNT, NOL10, NOL11, NOL6, NOL9, NOP14, NOP56, NOP58, NOP9, NPM1, NPM3, NQO2, NSDHL, NSF, NSFL1C, NSUN2, NSUN5, NTHL1, NUCB2, NUDCD1, NUDT16, NUP107, NUP133, NUP160, NUP210, NUP35, NUP50, NUSAP1, NVL, NXN, OCLN, OGT, OPTN, ORC2, ORC3, ORC4, ORC5, OSBP, OSTC, OSTF1, OXCT1, P3H1, P3H3, P3H4, P4HA1, P4HA2, P4HB, P4HTM, PABPN1, PACS1, PAK1, PALLD, PARD3, PARD6B, PARN, PARP1, PARVA, PASK, PATZ1, PAWR, PAXBP1, PC, PCBP2, PCCA, PCCB, PCYT1A, PCYT2, PDCD11, PDCD2, PDCL3, PDGFRB, PDIA3, PDIA4, PDIA6, PDLIM2, PDLIM4, PDLIM5, PDLIM7, PDS5A, PDXDC1, PDXK, PEA15, PELP1, PES1, PFKL, PFKM, PFKP, PGAM5, PGRMC2, PHC1, PHIP, PHPT1, PI4KA, PIAS4, PICALM, PIGS, PIGT, PIK3C2A, PIK3R4, PIP4K2B, PIP4K2C, PITRM1, PKM, PKP2, PLCB3, PLCG1, PLD3, PLEC, PLEKHA5, PLEKHA7, PLIN3, PLK1, PLOD1, PLOD2, PLOD3, PLPP3, PLS3, PLSCR3, PM20D2, PNO1, PNP, PNPT1, POFUT1, POFUT2, POGLUT2, POGLUT3, POGZ, POLA1, POLA2, POLB, POLD1, POLE, POLR1B, POLR2A, POLR2B, POLR2G, POLR3A, POLR3C, POLR3F, PON2, POR, PPIB, PPIC, PPID, PPM1B, PPM1F, PPM1G, PPP1R18, PPP1R7, PPP1R9B, PPP2R5A, PRAF2, PRDX1, PRDX4, PREPL, PRIM1, PRIM2, PRKAA1, PRKAB1, PRKACB, PRKACG, PRKAG1, PRKAR1A, PRKAR2A, PRKAR2B, PRKCA,	

					PRKCSH, PRKDC, PRORP, PRPF38A, PRPF38B, PRPF40A, PRPF8, PRRC1, PRUNE1, PSIP1, PSMC6, PSMD1, PSMD2, PSMD9, PSME3, PSPC1, PTBP2, PTCD1, PTCD3, PTGIS, PTPMT1, PTPN1, PTPN12, PTPN2, PURA, PUS1, PUS3, PUS7, PWP2, PXDN, PXK, PXMP2, PYCARD, PYCR1, PYCR2, PYCR3, QRSL1, RAB14, RAB18, RAB1A, RAB23, RAB2A, RAB32, RAB5B, RAB5C, RAB8A, RABGAP1L, RABL6, RAC3, RAD51, RAD54L2, RAI14, RAN, RANBP2, RANGAP1, RAP1GDS1, RAP2A, RAP2B, RAP2C, RBBP5, RBM15, RBM25, RBM26, RBM3, RBM6, RBMS2, RBP1, RBPJ, RBPMS, RCC2, RCN1, RDH10, RDX, RELA, RETSAT, RFC1, RFC2, RFC3, RFC5, RFTN1, RHEB, RIC1, RIF1, RIOX1, RNF170, RNH1, ROCK2, RP2, RPA1, RPA2, RPA3, RPF2, RPL13A, RPL22, RPL27A, RPL4, RPL5, RPL7A, RPN1, RPRD1A, RPS2, RPS21, RPS6KA1, RPS6KA3, RPS6KA4, RPS8, RRBP1, RRP12, RRP7A, RRS1, RTCA, RTKN, RTTN, S100A10, S100A11, SAAL1, SALL2, SAMD4B, SAMHD1, SAP30BP, SAR1A, SARM1, SARS1, SARS2, SART3, SBDS, SBF1, SCAMP2, SCARB2, SCFD1, SCFD2, SCLY, SCRN1, SCYL1, SDAD1, SDF2L1, SDF4, SEC13, SEC16A, SEC22B, SEC23A, SEC23B, SEC24A, SEC24B, SEC24C, SEC24D, SEC31A, SEC61G, SEH1L, SEL1L, SELENBP1, SEPHS1, SEPTIN10, SEPTIN11, SEPTIN2, SEPTIN7, SEPTIN8, SERBP1, SERPINB6, SERPINB9, SERPINH1, SESN2, SESTD1, SET, SETD7, SETDB1, SF3A3, SF3B1, SH3GL3, SH3GLB1, SIL1, SIN3A, SIRT1, SIRT2, SKA3, SKP2, SLAIN2, SLC12A4, SLC1A3, SLC25A12, SLC25A24, SLC25A3, SLC25A32, SLC25A5, SLC2A1, SLC33A1, SLC44A1, SLC44A2, SLC4A7, SLC7A6, SLC9A3R2,	

					SLIRP, SLTM, SMARCA4, SMARCA5, SMARCAD1, SMARCC1, SMARCD1, SMARCD2, SMC2, SNRNP200, SNRPA1, SNRPD3, SNTB2, SNX17, SNX3, SNX4, SNX6, SNX9, SORBS1, SORBS3, SORD, SPAG9, SPARC, SPATS2L, SPECC1L, SPON1, SPOUT1, SPR, SPTAN1, SPTBN1, SPTBN2, SPTLC2, SRC, SRP14, SRPK1, SRPRA, SRPRB, SRSF1, SRSF11, SRSF7, SSB, SSBP1, SSR1, SSR4, SSRP1, STAM2, STIM1, STK38, STT3A, STT3B, STX12, STX5, STXBP2, STYX, SUCLG1, SUMF2, SUN2, SUPT16H, SUPT5H, SUPT6H, SURF4, SYMPK, TACC3, TARS2, TBC1D17, TBCEL, TBL2, TBL3, TBPL1, TBRG4, TCP1, TDP1, TDP2, TELO2, TEX10, TEX2, TF, TFB1M, TFG, TGFB1I1, TGM2, THBS1, THUMPD3, TIA1, TIGAR, TIMM50, TIMMDC1, TIMP3, TJP2, TKFC, TKT, TLK1, TLN1, TLN2, TM9SF4, TMA16, TMED10, TMED2, TMED5, TMED7, TMED9, TMEM115, TMEM167A, TMEM168, TMEM192, TMEM214, TMEM43, TMEM97, TMF1, TMX3, TMX4, TNPO3, TNS1, TOLLIP, TOMM34, TOP2A, TPM1, TPM4, TPP2, TPST1, TPX2, TRAM1, TRAP1, TRIM28, TRIM33, TRIM71, TRIP12, TRIP13, TRMT1, TRMT10C, TRMT1L, TRMT5, TSEN34, TSR1, TTC1, TTC28, TTF2, TTI1, TTK, TTLL12, TUBA4A, TUBB6, TUT1, TWF1, TXNDC5, TXNL1, TXNRD1, UAP1, UBA6, UBE2H, UBE2O, UBN2, UBR4, UBR5, UFL1, UGDH, UGGT1, UGGT2, UGP2, UNG, UPF2, UPF3B, USE1, USP15, USP19, USP47, USP5, USP7, USP9X, UTP15, UTP18, UTP20, UTP25, UTP4, UTRN, VAMP2, VAMP3, VAT1, VIM, VLDLR, VPS25, VPS26A, VPS26B, VPS26C, VPS35, VPS35L, VPS36, VPS53, VRK1, VRTN, VWA5A, WAPL, WARS1, WDHD1, WDR18, WDR3, WDR36, WDR37, WDR43, WRAP53, XPO5, XPO7,	

					XRCC5, XRCC6, XRN1, XRN2, YAP1, YARS2, YIF1A, YKT6, YRDC, YTHDC2, ZC3H15, ZC3H7B, ZC3HAV1, ZDHHC17, ZFP36L2, ZFYVE16, ZMYM2, ZMYM3, ZMYND8, ZNF185, ZNF217, ZNF281, ZNF462, ZNF532, ZNF638, ZWILCH]	
intracellular membrane-bounded organelle	GO_CellularComponent-EBI-UniProt-GOA-ACAP-ARAP_13.05.2021_00h00	3.20E-97	10.11136	1244	[AAAS, AASS, AATF, ABAT, ABCB10, ABCB7, ABCF1, ABHD10, ABHD12, ABHD14B, ABI1, ACAA1, ACACA, ACAD8, ACAD9, ACAT1, ACAT2, ACBD3, ACIN1, ACLY, ACO1, ACOT13, ACOT9, ACOX3, ACSL1, ACSL4, ACSS2, ACSS3, ACTBL2, ACTN1, ACTN4, ACTR10, ACTR1B, ACTR2, ADAM10, ADAR, ADARB1, ADD2, ADD3, ADPGK, AEBP2, AGL, AGO1, AGO2, AGTPBP1, AIMP2, AK4, AKR7A2, AKT1, ALDH1L2, ALDH2, ALDH7A1, ALDOC, ALG11, ALG2, ALG5, ALG9, AMDHD2, ANK2, ANK3, ANKFY1, ANKRD28, ANKS1A, ANO6, ANP32E, ANTXR1, ANXA1, ANXA11, ANXA2, ANXA3, ANXA4, ANXA6, AP1M1, AP1S1, AP2A1, AP2A2, AP2B1, AP2M1, AP2S1, AP3B1, APAF1, APEH, API5, APOBEC3C, APOE, APOOL, APPL2, APRT, ARAP1, ARCN1, ARF4, ARF6, ARFGAP1, ARFGAP3, ARFGEF1, ARHGAP1, ARHGAP5, ARHGDIA, ARL1, ARL2, ARL3, ARL6IP5, ARPC1A, ARSA, ARSB, ARVCF, ASPH, ATF7IP, ATL2, ATL3, ATP1A1, ATP2B1, ATP2B2, ATP2B4, ATP6V1A, ATR, AUP1, AURKA, AURKB, B3GLCT, B4GALT1, BAG1, BAG3, BAIAP2, BAZ1B, BCAT2, BCCIP, BDH2, BET1, BIN1, BIRC6, BLVRB, BMS1, BOP1, BPNT2, BPTF, BRAT1, BRD3, BRD4, BRMS1, BRWD1, BUB1B, C1QBP, CACYBP, CALCOCO2, CALR, CALU, CAMK1, CAMK2D, CANX, CAP1, CAPN1, CAPN2, CARNMT1, CASK, CASP3, CAST, CAT, CAV1, CAVIN1, CBL, CBX2, CBX5, CCAR1, CCAR2, CCDC22, CCNB1, CCND2, CCNH,	No change

					CCNL2, CCNY, CCT2, CCT4, CCT8, CD2AP, CD44, CDC42, CDC73, CDCA8, CDH2, CDK5RAP1, CDK5RAP3, CDK7, CEBPZ, CELF1, CERCAM, CFAP20, CFAP298, CFL2, CHAF1A, CHD1, CHD4, CHEK1, CHEK2, CHID1, CHPF, CHST14, CHTF18, CIRBP, CKAP4, CKAP5, CLASP1, CMPK1, CNDP2, CNOT11, COG1, COG6, COG7, COG8, COL1A1, COL1A2, COL4A1, COL4A2, COL5A1, COL5A2, COL6A1, COL6A2, COL6A3, COLGALT1, COMMD4, COPA, COPB1, COPB2, COPE, COPG1, COPG2, COPS2, COPS5, COPS7A, COPZ1, CORO1C, CPNE2, CPOX, CPSF2, CPSF3, CRLF3, CRMP1, CRTAP, CSDE1, CSE1L, CSPG4, CSRP2, CSTF1, CSTF3, CTBP2, CTCF, CTNNB1, CTNNBL1, CTSC, CUL4A, CUL4B, CUL7, CWC27, CXADR, CYB5R1, CYB5R3, CYFIP1, CYP2S1, CYP51A1, DAGLB, DAPK1, DARS2, DAXX, DAZAP1, DBNL, DCAF1, DCAF13, DCTN1, DCTN4, DCXR, DDRGK1, DDX10, DDX18, DDX20, DDX21, DDX24, DDX28, DDX31, DDX39B, DDX41, DDX42, DDX46, DDX47, DDX49, DDX5, DDX52, DDX54, DDX6, DECR1, DEGS1, DHRS7B, DHX15, DHX30, DHX33, DHX37, DHX8, DHX9, DIAPH1, DICER1, DIPK2A, DLG1, DLGAP5, DNMT1, DNMT3A, DNMT3B, DNTTIP2, DOCK7, DPH1, DPH6, DPPA4, DPYSL3, DRG1, DSP, DUSP3, DYNC1LI2, ECE1, ECPAS, ECT2, EDEM3, EDRF1, EEA1, EEF1E1, EEF2, EFTUD2, EGFR, EHD1, EHD2, EHD4, EHMT1, EIF3A, EIF4A3, EIF5B, ELAC2, ELP1, ELP3, EMD, EMSY, ENOPH1, EPB41, EPB41L5, EPM2AIP1, EPN2, ERAP1, ERBIN, ERCC2, ERGIC1, ERGIC2, ERLEC1, ERLIN2, ERO1A, ERP44, ESD, ESF1, ESYT2, ETFA, ETHE1, EXD2, EXOG, EXOSC10, EXOSC2, EXOSC5, EXOSC7, F11R, FAF2, FAHD1,	

					FAM114A1, FAM118B, FAM120A, FANCD2, FANCI, FASTKD2, FAT1, FBXO2, FBXW8, FDPS, FDXR, FECH, FERMT2, FGG, FH, FHL1, FKBP10, FKBP14, FKBP15, FKBP4, FKBP5, FKBP7, FKBP8, FKBP9, FLAD1, FLNB, FLT1, FN1, FNBP1L, FNDC3A, FOXK1, FOXRED1, FSCN1, FSD1, FTH1, FTSJ3, FUT11, FXR1, G3BP1, GALNT1, GALNT10, GALNT2, GARS1, GATA6, GATAD2A, GBF1, GCDH, GCLC, GDAP1, GDI1, GEMIN4, GEMIN5, GET3, GET4, GINS3, GIPC1, GLA, GLB1L3, GLS, GLT8D1, GLUD1, GLUL, GNB2, GNL2, GNL3, GNPDA2, GNS, GOLGA2, GOLGA3, GOLIM4, GOLT1B, GOPC, GOSR2, GOT2, GPC1, GPC3, GPC6, GPI, GPSM1, GPX7, GPX8, GSN, GSTK1, GSTM2, GSTM3, GTF2E1, GTF2I, GTF3C1, GTF3C2, GTF3C3, GTF3C4, GUF1, H6PD, HACL1, HADHA, HADHB, HAGH, HAT1, HCFC1, HDAC2, HDAC4, HDHD5, HDLBP, HEATR1, HEATR5A, HELLS, HERC2, HEXA, HIBADH, HK1, HK2, HLA-A, HMGCL, HMOX1, HMOX2, HNRNPF, HNRNPL, HNRNPLL, HNRNPM, HOOK3, HP1BP3, HSD17B10, HSD17B4, HSDL2, HSP90AB1, HSP90B1, HSPA13, HSPA14, HSPA2, HSPA4, HSPA5, HSPA9, HSPB1, HSPD1, HSPE1, HSPH1, HTT, HUWE1, IDH2, IDI1, IGF2BP3, IKBIP, ILF2, ILF3, ILK, ILVBL, IMP3, IMPDH1, IMPDH2, INCENP, INO80C, IPO11, IPO4, IPO5, IQGAP1, IQGAP2, IRF2BPL, IRF3, IST1, ITCH, ITGAV, ITGB1, IWS1, JADE1, JAM3, JARID2, KANK2, KAT7, KDELR1, KDELR3, KDM1A, KDM2A, KDM3B, KEAP1, KIF11, KIF20A, KIF22, KIF23, KIF2C, KIF5B, KLC1, KNTC1, KRT18, KRT8, KTN1, L1TD1, L2HGDH, LAMB1, LAMC1, LANCL2, LARS1, LARS2, LAS1L, LBR, LCMT1, LDHB, LGALS1, LIG1, LIG3, LIN28A, LMAN1,	

					LMCD1, LMF2, LNPEP, LNPK, LPCAT1, LPGAT1, LPP, LRBA, LRP1, LRPPRC, LRRC40, LRRC59, LRRC8A, LRRK1, LRWD1, LSG1, LSM4, LSS, LTBP1, LYAR, LYPLA2, MACF1, MACROH2A1, MAGED1, MAGED2, MAIP1, MAN1A1, MAN1A2, MANBA, MAP1LC3A, MAP1S, MAP2K6, MAPK1, MAPK14, MAPK8, MARCHF5, MARS1, MASTL, MATR3, MAVS, MBD3, MBNL1, MCAM, MCC, MCCC2, MCM2, MCM3, MCM4, MCM5, MCM6, MCM7, MCMBP, MCU, MDC1, MDN1, ME1, MEAK7, MEMO1, MESD, MEST, METTL1, MEX3A, MFGE8, MFSD10, MGAT2, MGST1, MICAL3, MICOS13, MIPEP, MKI67, MLH1, MMAB, MME, MOCS2, MOGS, MON2, MOSPD2, MOXD1, MPC2, MPHOSPH10, MRPS27, MRPS9, MSH2, MSH3, MSH6, MSI1, MSN, MTA3, MTAP, MTDH, MTHFD1, MTMR6, MTREX, MVB12A, MVD, MVK, MYBBP1A, MYD88, MYDGF, MYEF2, MYH10, MYH9, MYO1C, MYO1E, MYO5A, NBAS, NCAM1, NCAPD2, NCAPG, NCAPG2, NCL, NDC1, NDC80, NDRG1, NDRG2, NECAP2, NEDD4, NEK7, NFKB1, NHLRC2, NIBAN2, NIF3L1, NIT2, NLE1, NLN, NMT2, NNT, NOL10, NOL11, NOL6, NOL9, NOP14, NOP56, NOP58, NOP9, NPM1, NPM3, NQO2, NSDHL, NSF, NSFL1C, NSUN2, NSUN5, NTHL1, NUCB2, NUDCD1, NUDT16, NUP107, NUP133, NUP160, NUP210, NUP35, NUP50, NUSAP1, NVL, NXN, OCLN, OGT, OPTN, ORC2, ORC3, ORC4, ORC5, OSBP, OSTC, OSTF1, OXCT1, P3H1, P3H3, P3H4, P4HA1, P4HA2, P4HB, P4HTM, PABPN1, PACS1, PAK1, PALLD, PARD6B, PARN, PARP1, PARVA, PASK, PATZ1, PAWR, PAXBP1, PC, PCBP2, PCCA, PCCB, PCYT1A, PCYT2, PDCD11, PDCD2,	

					PDCL3, PDGFRB, PDIA3, PDIA4, PDIA6, PDLIM2, PDLIM4, PDLIM7, PDS5A, PDXDC1, PDXK, PEA15, PELP1, PES1, PFKL, PFKM, PFKP, PGAM5, PGRMC2, PHC1, PHIP, PHPT1, PI4KA, PIAS4, PICALM, PIGS, PIGT, PIK3C2A, PIK3R4, PIP4K2B, PIP4K2C, PITRM1, PKM, PKP2, PLCB3, PLCG1, PLD3, PLEKHA5, PLEKHA7, PLIN3, PLK1, PLOD1, PLOD2, PLOD3, PLPP3, PLSCR3, PM20D2, PNO1, PNP, PNPT1, POFUT1, POFUT2, POGLUT2, POGLUT3, POGZ, POLA1, POLA2, POLB, POLD1, POLE, POLR1B, POLR2A, POLR2B, POLR2G, POLR3A, POLR3C, POLR3F, PON2, POR, PPIB, PPIC, PPID, PPM1B, PPM1F, PPM1G, PPP1R7, PPP1R9B, PPP2R5A, PRAF2, PRDX1, PRDX4, PREPL, PRIM1, PRIM2, PRKAA1, PRKAB1, PRKACB, PRKACG, PRKAG1, PRKCA, PRKCSH, PRKDC, PRORP, PRPF38A, PRPF38B, PRPF40A, PRPF8, PRRC1, PRUNE1, PSIP1, PSMC6, PSMD1, PSMD2, PSMD9, PSME3, PSPC1, PTBP2, PTCD1, PTCD3, PTGIS, PTPMT1, PTPN1, PTPN12, PTPN2, PURA, PUS1, PUS3, PUS7, PWP2, PXDN, PXMP2, PYCARD, PYCR1, PYCR2, QRSL1, RAB14, RAB18, RAB1A, RAB23, RAB2A, RAB32, RAB5B, RAB5C, RAB8A, RABGAP1L, RABL6, RAC3, RAD51, RAD54L2, RAI14, RAN, RANBP2, RANGAP1, RAP1GDS1, RAP2A, RAP2B, RAP2C, RBBP5, RBM15, RBM25, RBM26, RBM3, RBM6, RBMS2, RBP1, RBPJ, RBPMS, RCC2, RCN1, RDH10, RELA, RETSAT, RFC1, RFC2, RFC3, RFC5, RFTN1, RHEB, RIC1, RIF1, RIOX1, RNF170, RNH1, ROCK2, RP2, RPA1, RPA2, RPA3, RPF2, RPL13A, RPL22, RPL27A, RPL4, RPL5, RPL7A, RPN1, RPRD1A, RPS2, RPS21, RPS6KA1, RPS6KA3, RPS6KA4, RPS8, RRBP1, RRP12, RRP7A, RRS1, RTCA, S100A10, S100A11,	

					SAAL1, SALL2, SAMD4B, SAMHD1, SAP30BP, SAR1A, SARM1, SARS1, SARS2, SART3, SBDS, SBF1, SCAMP2, SCARB2, SCFD1, SCFD2, SCLY, SCRN1, SCYL1, SDAD1, SDF2L1, SDF4, SEC13, SEC16A, SEC22B, SEC23A, SEC23B, SEC24A, SEC24B, SEC24C, SEC24D, SEC31A, SEC61G, SEH1L, SEL1L, SELENBP1, SEPHS1, SEPTIN2, SEPTIN7, SEPTIN8, SERBP1, SERPINB6, SERPINB9, SERPINH1, SESN2, SET, SETD7, SETDB1, SF3A3, SF3B1, SH3GL3, SH3GLB1, SIL1, SIN3A, SIRT1, SIRT2, SKP2, SLC12A4, SLC1A3, SLC25A12, SLC25A24, SLC25A3, SLC25A32, SLC25A5, SLC2A1, SLC33A1, SLC44A1, SLC44A2, SLC4A7, SLC7A6, SLC9A3R2, SLIRP, SLTM, SMARCA4, SMARCA5, SMARCAD1, SMARCC1, SMARCD1, SMARCD2, SMC2, SNRNP200, SNRPA1, SNRPD3, SNTB2, SNX17, SNX3, SNX4, SNX6, SNX9, SORBS1, SORBS3, SORD, SPAG9, SPARC, SPATS2L, SPON1, SPR, SPTAN1, SPTBN1, SPTLC2, SRC, SRP14, SRPK1, SRPRA, SRPRB, SRSF1, SRSF11, SRSF7, SSB, SSBP1, SSR1, SSR4, SSRP1, STAM2, STIM1, STK38, STT3A, STT3B, STX12, STX5, STXBP2, STYX, SUCLG1, SUMF2, SUN2, SUPT16H, SUPT5H, SUPT6H, SURF4, SYMPK, TACC3, TARS2, TBC1D17, TBL2, TBL3, TBPL1, TBRG4, TCP1, TDP1, TDP2, TELO2, TEX10, TEX2, TF, TFB1M, TFG, TGFB1I1, TGM2, THBS1, THUMPD3, TIA1, TIGAR, TIMM50, TIMMDC1, TIMP3, TJP2, TKFC, TKT, TLK1, TM9SF4, TMA16, TMED10, TMED2, TMED5, TMED7, TMED9, TMEM115, TMEM167A, TMEM168, TMEM192, TMEM214, TMEM43, TMEM97, TMF1, TMX3, TMX4, TNPO3, TOLLIP, TOMM34, TOP2A, TPP2, TPST1, TPX2, TRAM1, TRAP1, TRIM28, TRIM33, TRIP12, TRIP13, TRMT1,	

					TRMT10C, TRMT1L, TRMT5, TSEN34, TSR1, TTC1, TTF2, TTI1, TTK, TTLL12, TUBB6, TUT1, TWF1, TXNDC5, TXNL1, TXNRD1, UAP1, UBA6, UBE2H, UBE2O, UBN2, UBR4, UBR5, UFL1, UGDH, UGGT1, UGGT2, UGP2, UNG, UPF2, UPF3B, USE1, USP15, USP19, USP47, USP5, USP7, USP9X, UTP15, UTP18, UTP20, UTP25, UTP4, UTRN, VAMP2, VAMP3, VAT1, VIM, VLDLR, VPS25, VPS26A, VPS26B, VPS26C, VPS35, VPS35L, VPS36, VPS53, VRK1, VWA5A, WAPL, WARS1, WDHD1, WDR18, WDR3, WDR36, WDR37, WDR43, WRAP53, XPO5, XPO7, XRCC5, XRCC6, XRN1, XRN2, YAP1, YARS2, YIF1A, YKT6, YRDC, YTHDC2, ZC3H15, ZC3H7B, ZC3HAV1, ZDHHC17, ZFP36L2, ZFYVE16, ZMYM2, ZMYM3, ZMYND8, ZNF217, ZNF281, ZNF462, ZNF532, ZNF638]	
cell adhesion mediator activity	GO_BiologicalProcess-EBI-UniProt-GOA-ACAP-ARAP_13.05.2021_00h00	1.21E-05	25.39683	16	[ANXA1, ANXA2, BAIAP2, BCAT2, CDC42EP1, CNN3, CXADR, DSP, EPCAM, ITGB1, JAM3, KRT18, PALLD, PDLIM5, PKP2, S100A11]	Downregulation
cell-cell adhesion mediator activity	GO_BiologicalProcess-EBI-UniProt-GOA-ACAP-ARAP_13.05.2021_00h00	1.21E-05	26.92308	14	[ANXA1, ANXA2, BAIAP2, CDC42EP1, CNN3, CXADR, DSP, EPCAM, JAM3, KRT18, PALLD, PDLIM5, PKP2, S100A11]	Downregulation
cadherin binding involved in cell-cell adhesion	GO_BiologicalProcess-EBI-UniProt-GOA-ACAP-ARAP_13.05.2021_00h00	1.21E-05	50	9	[ANXA1, ANXA2, BAIAP2, CDC42EP1, CNN3, EPCAM, KRT18, PDLIM5, S100A11]	Downregulation
polymeric cytoskeletal fiber	GO_CellularComponent-EBI-UniProt-GOA-ACAP-ARAP_13.05.2021_00h00	7.44E-17	12.9771	102	[ACTBL2, ACTN1, AK1, ANXA1, ARL3, AURKA, AURKB, BAG2, BAIAP2, CCT2, CCT3, CCT4, CCT5, CCT6A, CCT7, CCT8, CD2AP, CDK5RAP3, CEP170, CFAP20, CKAP5, CLASP1, DBNL, DCTN1, DCXR, DDX6, DLG1, DPYSL2, DPYSL3, DSP, DYNC1LI2, EIF3A, EMD,	No change

					EML1, FKBP4, FSD1, GNG12, GOLGA2, HOOK3, HSPH1, INCENP, IQGAP1, JAM3, KEAP1, KIF11, KIF20A, KIF21A, KIF22, KIF23, KIF2C, KIF5B, KLC1, KNTC1, KRT18, KRT19, KRT8, LRPPRC, MACF1, MAP1B, MAP1LC3A, MAP1S, MAP4, METAP1, MICAL1, MYO1C, MYO1E, MYO5A, MYO9B, NCKAP1, NDRG1, NEK7, NUSAP1, PAK1, PALLD, PAWR, PDLIM2, PDLIM4, PDLIM5, PDLIM7, PKP2, PLEC, PLK1, PLS3, RCC2, SARM1, SIRT2, SKA3, SLAIN2, SNTB2, SPECC1L, SRC, SRPRB, STIM1, TCP1, TMEM214, TPM1, TPM4, TPX2, TUBA4A, TUBB6, TWF1, VIM]	
actin cytoskeleton	GO_CellularComponent-EBI-UniProt-GOA-ACAP-ARAP_13.05.2021_00h00	7.44E-17	18.65672	100	[ACACA, ACTBL2, ACTN1, ACTN4, ACTR10, ACTR1B, ACTR2, ADD2, ALG2, ANXA1, ARPC1A, ASAP1, BAG3, BAIAP2, BIN1, CALD1, CAP1, CAPN2, CAPZA2, CASK, CD2AP, CDC42BPA, CDC42BPB, CDC42EP3, CDH2, CFL2, CGN, CNN3, CORO1C, DAPK1, DBNL, DCTN4, DHX9, DPYSL3, DSTN, FERMT2, FKBP15, FLNB, FLT1, FSCN1, GNG12, GSN, HDAC4, ILF3, ILK, IQGAP1, IQGAP2, JAM3, KEAP1, KNTC1, KRT19, LANCL2, LASP1, LPP, MACF1, MICAL1, MPRIP, MYADM, MYH10, MYH9, MYL9, MYO1C, MYO1E, MYO5A, MYO9B, NCAPG, NCKAP1, NDC1, NPM3, PAK1, PALLD, PARVA, PAWR, PDLIM2, PDLIM4, PDLIM5, PDLIM7, PLS3, PPP1R9B, PTPN12, PXDN, RDX, RTKN, SEPTIN11, SEPTIN2, SEPTIN7, SLC2A1, SNX9, SORBS1, SPECC1L, SPTAN1, SPTBN1, SPTBN2, SRC, TLN2, TPM1, TPM4, TWF1, UTRN, ZNF185]	Downregulation
actin filament	GO_CellularComponent-EBI-UniProt-GOA-ACAP-ARAP_13.05.2021_00h00	7.44E-17	22.22222	28	[ACTBL2, ACTN1, ANXA1, CD2AP, DBNL, DPYSL3, GNG12, IQGAP1, JAM3, KEAP1, MYO1C, MYO1E, MYO5A, MYO9B, NCKAP1, PAK1, PALLD, PAWR, PDLIM2, PDLIM4, PDLIM5,	Downregulation

					PDLIM7, PLS3, SPECC1L, SRC, TPM1, TPM4, TWF1]	
enzyme binding	GO_MolecularFunction-EBI-UniProt-GOA-ACAP-ARAP_13.05.2021_00h00	4.86E-25	13.63208	289	[AATF, ABAT, ACAT1, ACIN1, ACTBL2, ADAM10, ADD2, AGO1, AGO2, AKT1, ANK2, ANKFY1, ANXA2, AP2A1, AP2A2, AP3B1, ARF6, ARHGAP1, ARHGDIA, ATP1A1, ATP2B4, AUP1, AURKA, AURKB, BAG1, BAG2, BIN1, BRD4, BRMS1, C1QBP, CACYBP, CALR, CAP1, CAP2, CAPN2, CARHSP1, CASP3, CAT, CAV1, CBL, CCAR2, CCNB1, CCND2, CCNY, CCT2, CDC42, CDC42BPB, CDC73, CDH2, CDK5RAP1, CDK5RAP3, CHD4, CHEK2, COL1A1, COL1A2, COPS5, CORO1C, CSE1L, CSPG4, CTBP2, CTNNB1, CTNNBL1, CTSC, CUL4A, CUL4B, CUL7, CYFIP1, DAXX, DCTN1, DDRGK1, DDX20, DDX5, DHX9, DIAPH1, DLG1, DNMT3B, DOCK7, DPYSL2, DSP, DUSP3, ECT2, EEF2, EGFR, EHD1, EIF3A, EPS8, ERLIN2, FAF2, FANCD2, FANCI, FARP1, FERMT2, FN1, FNBP1L, GARS1, GATA6, GCLM, GDI1, GLMN, GNB2, GOLGA2, GOT2, GPI, GSN, GSTM2, GSTM3, GYS1, HDAC2, HDAC4, HERC2, HMOX1, HSP90AB1, HSPA2, HSPA5, HSPA9, HSPB1, HSPD1, HTT, ILK, IPO11, IPO4, IPO5, IQGAP1, IQGAP2, IQGAP3, ITCH, ITGAV, ITGB1, KDM1A, KIF11, KIF20A, KIF5B, LIMS1, LRBA, LRP1, LRPPRC, MACROH2A1, MAP1LC3A, MAP2K6, MAPK1, MAPK14, MAPK8, MARCHF5, MASTL, MAVS, MCM2, MCM7, MICAL1, MICAL3, MLH1, MSH2, MSH3, MSH6, MSN, MTA3, MYO1C, MYO1E, MYO5A, MYO9B, NCKAP1, NCL, NDRG1, NEDD4, NOP14, NOP56, NOP58, NPM1, NSF, NSFL1C, OPTN, ORC3, P4HB, PABPN1, PAK1, PARD3, PARD6B, PARN, PARP1, PATZ1, PAWR, PCBP2, PCCA, PDCD2, PDGFRB, PDLIM4, PDLIM5, PFKL, PFKM, PIAS4, PICALM, PKP2, PLCG1, PLK1, POLA1,	No change

					POLB, POLD1, POLR1B, POLR2A, POR, PPIB, PPP1R18, PPP1R9B, PPP2R5A, PRKAB1, PRKACB, PRKAG1, PRKAR1A, PRKAR2A, PRKAR2B, PRKCA, PRKCSH, PRKDC, PSMD1, PTPN1, PTPN2, PUS7, PXDN, PYCARD, RAB8A, RABGAP1L, RAC3, RAD51, RANBP2, RANGAP1, RCC2, RDX, RELA, RFC2, RFC5, RHEB, RIC1, ROCK2, RPA2, RPL5, RPRD1A, RPS2, RPS6KA3, RTKN, SARS1, SART3, SCARB2, SERPINB6, SERPINB9, SIRT1, SIRT2, SLC12A4, SLC25A5, SLC2A1, SLC9A3R2, SMARCA4, SNRPD3, SNX3, SNX9, SPAG9, SPTBN1, SRC, SRSF1, STIM1, STK38, SUPT5H, TAX1BP1, TBL2, TCP1, TELO2, TIMP3, TJP2, TNPO3, TOLLIP, TOP2A, TPX2, TRAP1, TRIM28, TTC28, TUBA4A, TUT1, UBE2G1, UFL1, USP19, USP7, UTRN, WARS1, WDR43, WRAP53, XPO5, XPO7, XRCC5, YOD1, YTHDC2, ZMYM2]	
kinase binding	GO_MolecularFunction-EBI-UniProt-GOA-ACAP-ARAP_13.05.2021_00h00	4.86E-25	14.72081	116	[AATF, ACTBL2, ADAM10, ADD2, AKT1, ANK2, AP2A1, AP2A2, ARHGDIA, ATP1A1, ATP2B4, AURKA, AURKB, C1QBP, CAV1, CBL, CCNB1, CCND2, CCNY, CDC42, CDH2, CDK5RAP1, CDK5RAP3, CHEK2, CSPG4, CTBP2, CTNNB1, DAXX, DCTN1, DLG1, DPYSL2, DSP, DUSP3, EEF2, EGFR, EIF3A, FERMT2, GARS1, GATA6, GOLGA2, GSN, GYS1, HDAC4, HSP90AB1, HSPB1, HTT, ILK, IQGAP1, ITGAV, ITGB1, KIF11, KIF20A, KIF5B, LIMS1, LRBA, MACROH2A1, MAP2K6, MAPK1, MAPK14, MAVS, MICAL1, MSH2, MSN, NPM1, NSF, ORC3, PAK1, PARD6B, PARN, PARP1, PDGFRB, PDLIM5, PFKL, PFKM, PKP2, PLCG1, PLK1, POLA1, POLR2A, PPP1R9B, PPP2R5A, PRKAB1, PRKAG1, PRKAR1A, PRKAR2A, PRKAR2B, PRKCA, PRKCSH, PTPN1, PTPN2, RAB8A, RAC3, RCC2, RELA, RHEB, RPS6KA3, SIRT1, SLC12A4, SLC2A1, SPAG9, SRC,	Downregulation

					SRSF1, STK38, TAX1BP1, TBL2, TELO2, TJP2, TOLLIP, TOP2A, TPX2, TRAP1, TTC28, TUBA4A, UFL1, UTRN, WARS1]	
protein kinase binding	GO_MolecularFunction-EBI-UniProt-GOA-ACAP-ARAP_13.05.2021_00h00	4.86E-25	14.66855	104	[AATF, ACTBL2, ADAM10, ADD2, AKT1, ANK2, AP2A1, AP2A2, ARHGDIA, ATP1A1, ATP2B4, AURKA, C1QBP, CAV1, CBL, CCNB1, CCND2, CCNY, CDC42, CDH2, CDK5RAP1, CDK5RAP3, CHEK2, CSPG4, CTBP2, CTNNB1, DAXX, DCTN1, DLG1, DPYSL2, DSP, DUSP3, EEF2, EGFR, EIF3A, FERMT2, GARS1, GATA6, GOLGA2, GYS1, HDAC4, HSP90AB1, HSPB1, ILK, IQGAP1, ITGAV, ITGB1, KIF11, KIF20A, KIF5B, LIMS1, LRBA, MACROH2A1, MAP2K6, MAPK1, MAPK14, MAVS, MICAL1, MSH2, MSN, NPM1, NSF, ORC3, PAK1, PARD6B, PARN, PARP1, PDGFRB, PDLIM5, PKP2, PLCG1, PLK1, POLA1, PRKAB1, PRKAG1, PRKAR1A, PRKAR2A, PRKAR2B, PRKCA, PRKCSH, PTPN1, PTPN2, RAB8A, RAC3, RCC2, RELA, RHEB, RPS6KA3, SIRT1, SLC12A4, SPAG9, SRC, SRSF1, STK38, TBL2, TELO2, TJP2, TOP2A, TPX2, TRAP1, TUBA4A, UFL1, UTRN, WARS1]	Downregulation
cell cortex	GO_CellularComponent-EBI-UniProt-GOA-ACAP-ARAP_13.05.2021_00h00	1.69E-19	19.14894	63	[ACTR2, ADD3, ANXA2, ARF6, ASPH, CALD1, CAP1, CAPN2, CAPZA2, CAV1, CDH2, CLASP1, CORO1C, CTBP2, CTNNB1, DBNL, DCTN1, DCTN4, DSTN, ECT2, EMD, EPB41, EPS8, FERMT2, FLNB, FNBP1L, FSCN1, GIPC1, GPSM1, GSN, ITCH, KRT19, LANCL2, LASP1, MICAL3, MYADM, MYH10, MYH9, MYL9, MYO9B, NCL, NEDD4, PARD3, PARD6B, PPP1R9B, PXDN, RAC3, RAI14, RDX, RTKN, SEPTIN10, SEPTIN11, SEPTIN2, SEPTIN7, SEPTIN8, SLC2A1, SNX9, SPTAN1, SPTBN1, SPTBN2, STIM1, TPM4, UTRN]	Downregulation
cortical cytoskeleton	GO_CellularComponent-EBI-UniProt-GOA-ACAP-ARAP_13.0	1.69E-19	22.0339	26	[ACTR2, CALD1, CAP1, CAPN2, CAPZA2, CDH2, CLASP1, DBNL, DSTN, EPB41, GSN, KRT19, LANCL2, LASP1, MYADM, MYH9, PPP1R9B, RDX, RTKN,	Downregulation

	5.2021_00h00				SLC2A1, SNX9, SPTAN1, SPTBN1, SPTBN2, TPM4, UTRN]	
cortical actin cytoskeleton	GO_CellularComponent-EBI-UniProt-GOA-ACAP-ARAP_13.05.2021_00h00	1.69E-19	25.88235	22	[ACTR2, CALD1, CAP1, CAPN2, CDH2, DBNL, DSTN, GSN, KRT19, LANCL2, LASP1, MYADM, MYH9, PPP1R9B, RDX, RTKN, SLC2A1, SNX9, SPTAN1, SPTBN1, SPTBN2, UTRN]	Downregulation
ribonucleoprotein complex biogenesis	GO_BiologicalProcess-EBI-UniProt-GOA-ACAP-ARAP_13.05.2021_00h00	3.00E-30	23.3871	116	[AATF, AGO1, AGO2, ANTXR1, ATR, BMS1, BOP1, C1QBP, CDC73, CELF1, CUL4A, CUL4B, DCAF13, DDX10, DDX18, DDX20, DDX21, DDX28, DDX31, DDX39B, DDX47, DDX49, DDX52, DDX54, DHX30, DHX37, DHX9, DICER1, EFL1, EIF3A, EIF4A3, ERCC2, ESF1, EXOSC10, EXOSC2, EXOSC5, EXOSC7, FASTKD2, FTSJ3, GEMIN4, GEMIN5, GLUL, GNL2, HEATR1, HSP90AB1, IGF2BP3, IMP3, LAS1L, LSG1, LSM4, LYAR, MDN1, MMAB, MPHOSPH10, MTREX, MYBBP1A, NEDD4, NLE1, NOL10, NOL11, NOL6, NOL9, NOP14, NOP56, NOP58, NOP9, NPM1, NPM3, NSUN5, NUDT16, NVL, PDCD11, PELP1, PES1, PRKDC, PRPF8, PSIP1, PWP2, RAN, RPF2, RPL13A, RPL22, RPL27A, RPL4, RPL5, RPL7A, RPS2, RPS21, RPS8, RRP12, RRP7A, RRS1, SART3, SBDS, SDAD1, SF3A3, SF3B1, SNRNP200, SNRPD3, SRPK1, SRSF1, TBL3, TEX10, TFB1M, TSR1, UTP15, UTP18, UTP20, UTP25, UTP4, WDR18, WDR3, WDR36, WDR43, XRCC5, XRN2]	Upregulation
ribonucleoprotein complex subunit organization	GO_BiologicalProcess-EBI-UniProt-GOA-ACAP-ARAP_13.05.2021_00h00	3.00E-30	20	42	[AGO1, AGO2, ANTXR1, ATR, BOP1, CDC73, CELF1, DDX20, DDX28, DDX39B, DHX30, DHX8, DHX9, DICER1, EIF3A, FASTKD2, GEMIN4, GEMIN5, HSP90AB1, KIF5B, KLC1, LSM4, MDN1, MMAB, NLE1, PRKDC, PRPF8, PSIP1, PWP2, RPF2, RPL13A, RPL5, RRP7A, RRS1, SART3, SF3A3, SF3B1, SNRNP200, SNRPD3, SRPK1, SRSF1, XRCC5]	Upregulation
ribosomal large subunit biogenesis	GO_BiologicalProcess-EBI-UniProt-GOA-	3.00E-30	25.33333	19	[BOP1, DDX18, DDX28, DHX30, FASTKD2, FTSJ3, LAS1L, MDN1, NEDD4, NLE1, NOL9, NPM1, NVL, PES1,	Upregulation

	ACAP-ARAP_13.05.2021_00h00				RPF2, RPL5, RPL7A, RRS1, SDAD1]	
ribonucleoprotein complex assembly	GO_BiologicalProcess-EBI-UniProt-GOA-ACAP-ARAP_13.05.2021_00h00	3.00E-30	19.21182	39	[AGO1, AGO2, ANTXR1, ATR, BOP1, CDC73, CELF1, DDX20, DDX28, DDX39B, DHX30, DHX9, DICER1, EIF3A, FASTKD2, GEMIN4, GEMIN5, HSP90AB1, LSM4, MDN1, MMAB, NLE1, PRKDC, PRPF8, PSIP1, PWP2, RPF2, RPL13A, RPL5, RRP7A, RRS1, SART3, SF3A3, SF3B1, SNRNP200, SNRPD3, SRPK1, SRSF1, XRCC5]	Upregulation
ribosome assembly	GO_BiologicalProcess-EBI-UniProt-GOA-ACAP-ARAP_13.05.2021_00h00	3.00E-30	26.86567	18	[BOP1, C1QBP, DDX28, DHX30, DHX37, EFL1, FASTKD2, MDN1, NLE1, NPM1, PRKDC, PWP2, RPF2, RPL5, RRP7A, RRS1, SBDS, XRCC5]	Upregulation
programmed cell death	GO_BiologicalProcess-EBI-UniProt-GOA-ACAP-ARAP_13.05.2021_00h00	7.56E-07	10.35422	228	[AATF, ACIN1, ACTN1, ACTN4, ADAM10, ADAR, ADARB1, AIMP2, AKT1, ANO6, ANP32E, ANXA1, ANXA4, ANXA5, ANXA6, APAF1, API5, APOE, ARF4, ARF6, ARHGDIA, ARHGEF1, ARHGEF11, ARHGEF12, ARL6IP5, ASNS, AURKA, AURKB, B4GALT1, BAG1, BAG3, BIN1, BIRC6, BRAT1, BRMS1, BUB1B, C1QBP, CALR, CAMK2D, CAPN1, CAPN2, CAPNS1, CASP3, CAST, CAT, CAV1, CBL, CCAR1, CCAR2, CCND2, CD44, CDC42, CDC73, CDK5RAP3, CHEK1, CHEK2, CNRIP1, CNTFR, COPS5, CTNNB1, CTNNBL1, CTSC, CUL4A, CYFIP1, DAPK1, DAXX, DDRGK1, DDX20, DDX41, DDX42, DDX47, DDX5, DHX9, DICER1, DIPK2A, DNMT1, DNMT3A, DSP, ECT2, EEF1E1, EGFR, EPB41L3, EPCAM, ERCC2, ERO1A, ETFA, EXOG, FASTKD2, FGG, FKBP8, FXR1, GATA6, GBE1, GCLC, GCLM, GLMN, GPI, GSDME, GSN, HDAC2, HDAC4, HELLS, HK2, HMOX1, HSP90AB1, HSP90B1, HSPA5, HSPA9, HSPB1, HSPD1, HSPE1, HTT, ILK, IRF3, ITCH, ITGA5, ITGA6, ITGAV, ITGB1, ITPK1, JADE1, KANK2, KDELR1, KDM1A, KRT18, KRT19, KRT8, LGALS1, LRP1,	No change

					MAGED1, MAP1S, MAP2K6, MAPK1, MAPK14, MAPK8, MCM2, MCM7, MFSD10, MICAL1, MLH1, MSH2, MSH6, MTDH, MYBBP1A, MYD88, MYDGF, NCKAP1, NDRG1, NFKB1, NIBAN2, NLE1, NNT, NPM1, NQO2, OGT, P4HB, PAK1, PARP1, PAWR, PDCD2, PDCL3, PDGFRB, PDIA3, PEA15, PGAM5, PHIP, PIAS4, PIGT, PIK3CA, PKM, PKP2, PLCG1, PLK1, PLSCR3, POLB, POLR2G, POR, PPID, PPM1F, PRKAA1, PRKCA, PRKDC, PRPF38B, PSME3, PTGIS, PTPMT1, PTPN1, PTPN2, PYCARD, RAI14, RBM25, RELA, RPS6KA1, RPS6KA3, RTKN, SAP30BP, SARM1, SERBP1, SERPINB9, SH3GLB1, SIN3A, SIRT1, SIRT2, SLC25A5, SLK, SLTM, SNX6, SORD, SRC, TAX1BP1, TCP1, TGM2, THBS1, TIA1, TIGAR, TIMP3, TMEM214, TMF1, TOP2A, TPX2, TRAP1, TXNDC5, UBE2O, UFL1, UNG, USP47, VPS35, YAP1]	
regulation of cell death	GO_BiologicalProcess-EBI-UniProt-GOA-ACAP-ARAP_13.05.2021_00h00	7.56E-07	10.37249	181	[AATF, ACTN1, ACTN4, ADAM10, ADAR, AKT1, ANO6, ANP32E, ANXA1, ANXA4, ANXA5, AP2B1, APAF1, API5, APOE, ARF4, ARHGDIA, ARHGEF1, ARHGEF11, ARHGEF12, ARL6IP5, ASNS, AURKA, AURKB, B4GALT1, BAG1, BAG3, BIN1, BIRC6, BRMS1, C1QBP, CACYBP, CALR, CAMK2D, CAPN2, CASP3, CAST, CAT, CAV1, CBL, CCAR2, CCND2, CD44, CDC42, CDC73, CELF1, CHEK2, CNTFR, COPS5, CTNNB1, CTNNBL1, CTSC, CYFIP1, DAPK1, DAXX, DDRGK1, DDX20, DDX42, DIPK2A, DNMT1, DNMT3A, ECT2, EEF1E1, EFNB2, EGFR, EPCAM, ETFA, FERMT2, FGG, FKBP8, GATA6, GBE1, GCLC, GCLM, GLMN, GPI, GSDME, GSN, HDAC2, HDAC4, HELLS, HMOX1, HSP90AB1, HSP90B1, HSPA5, HSPA9, HSPB1, HSPD1, HTT, ILK, IRF3, ITCH, ITGA5, ITGA6, ITGAV, ITGB1, KANK2, KDM1A, KRT18,	No change

					LGALS1, LRP1, MAGED1, MAP2K6, MAPK1, MAPK8, MEAK7, MICAL1, MSH2, MTDH, MYBBP1A, MYD88, MYDGF, NDRG1, NFKB1, NIBAN2, NLE1, NNT, NPM1, NQO2, OGT, P4HB, PARP1, PAWR, PDCD2, PDGFRB, PDIA3, PEA15, PHIP, PIAS4, PICALM, PIK3CA, PLCG1, PLK1, PLSCR3, POR, PPID, PRKAA1, PRKCA, PRKDC, PRPF38B, PSME3, PTGIS, PTPMT1, PTPN1, PTPN2, PYCARD, PYCR1, RBM25, RELA, RPS6KA1, RPS6KA3, RTKN, SAP30BP, SARM1, SERBP1, SERPINB9, SIN3A, SIRT1, SIRT2, SLC25A24, SLC25A5, SLK, SNX6, SRC, TAX1BP1, TCP1, TGM2, THBS1, TIA1, TIGAR, TIMP3, TMF1, TOP2A, TRAP1, TXNDC5, UBE2O, UFL1, UNG, USP47, VPS35, YAP1]	
apoptotic process	GO_BiologicalProcess-EBI-UniProt-GOA-ACAP-ARAP_13.05.2021_00h00	7.56E-07	10.74056	219	[AATF, ACIN1, ACTN1, ACTN4, ADAM10, ADAR, ADARB1, AIMP2, AKT1, ANO6, ANP32E, ANXA1, ANXA4, ANXA5, ANXA6, APAF1, API5, APOE, ARF4, ARF6, ARHGDIA, ARHGEF1, ARHGEF11, ARHGEF12, ARL6IP5, ASNS, AURKA, AURKB, B4GALT1, BAG1, BAG3, BIN1, BIRC6, BRAT1, BRMS1, BUB1B, C1QBP, CALR, CAMK2D, CAPN2, CASP3, CAST, CAT, CAV1, CBL, CCAR1, CCAR2, CCND2, CD44, CDC42, CDC73, CDK5RAP3, CHEK1, CHEK2, CNRIP1, CNTFR, COPS5, CTNNB1, CTNNBL1, CTSC, CUL4A, CYFIP1, DAPK1, DAXX, DDRGK1, DDX20, DDX41, DDX42, DDX47, DDX5, DICER1, DIPK2A, DNMT1, DNMT3A, ECT2, EEF1E1, EGFR, EPB41L3, EPCAM, ERCC2, ERO1A, ETFA, EXOG, FASTKD2, FGG, FKBP8, FXR1, GATA6, GBE1, GCLC, GCLM, GLMN, GPI, GSDME, GSN, HDAC2, HDAC4, HELLS, HK2, HMOX1, HSP90AB1, HSP90B1, HSPA5, HSPA9, HSPB1, HSPD1, HSPE1, HTT, ILK, IRF3, ITCH, ITGA5, ITGA6, ITGAV, ITGB1,	No change

					JADE1, KANK2, KDELR1, KDM1A, KRT18, KRT8, LGALS1, LRP1, MAGED1, MAP1S, MAP2K6, MAPK1, MAPK14, MAPK8, MCM2, MCM7, MFSD10, MICAL1, MLH1, MSH2, MSH6, MTDH, MYBBP1A, MYD88, MYDGF, NCKAP1, NDRG1, NFKB1, NIBAN2, NLE1, NNT, NPM1, NQO2, OGT, P4HB, PAK1, PARP1, PAWR, PDCD2, PDCL3, PDGFRB, PDIA3, PEA15, PHIP, PIAS4, PIGT, PIK3CA, PLCG1, PLK1, PLSCR3, POLB, POLR2G, POR, PPID, PPM1F, PRKAA1, PRKCA, PRKDC, PRPF38B, PSME3, PTGIS, PTPMT1, PTPN1, PTPN2, PYCARD, RAI14, RBM25, RELA, RPS6KA1, RPS6KA3, RTKN, SAP30BP, SARM1, SERBP1, SERPINB9, SH3GLB1, SIN3A, SIRT1, SIRT2, SLC25A5, SLK, SLTM, SNX6, SORD, SRC, TAX1BP1, TCP1, TGM2, THBS1, TIA1, TIGAR, TIMP3, TMEM214, TMF1, TOP2A, TPX2, TRAP1, TXNDC5, UBE2O, UFL1, UNG, USP47, VPS35, YAP1]	
regulation of programmed cell death	GO_BiologicalProcess-EBI-UniProt-GOA-ACAP-ARAP_13.05.2021_00h00	7.56E-07	10.5625	169	[AATF, ACTN1, ACTN4, ADAM10, ADAR, AKT1, ANO6, ANP32E, ANXA1, ANXA4, ANXA5, APAF1, API5, APOE, ARF4, ARHGDIA, ARHGEF1, ARHGEF11, ARHGEF12, ARL6IP5, ASNS, AURKA, AURKB, B4GALT1, BAG1, BAG3, BIN1, BIRC6, BRMS1, C1QBP, CALR, CAMK2D, CAPN2, CASP3, CAST, CAT, CAV1, CBL, CCAR2, CCND2, CD44, CDC42, CDC73, CHEK2, CNTFR, COPS5, CTNNB1, CTNNBL1, CTSC, CYFIP1, DAPK1, DAXX, DDRGK1, DDX20, DDX42, DIPK2A, DNMT1, ECT2, EEF1E1, EGFR, EPCAM, ETFA, FGG, FKBP8, GATA6, GBE1, GCLC, GCLM, GLMN, GPI, GSDME, GSN, HDAC2, HDAC4, HELLS, HMOX1, HSP90AB1, HSP90B1, HSPA5, HSPA9, HSPB1, HSPD1, HTT, ILK, IRF3, ITCH, ITGA5, ITGA6, ITGAV, ITGB1, KANK2, KDM1A, KRT18, LGALS1, LRP1,	No change

					MAGED1, MAP2K6, MAPK1, MAPK8, MICAL1, MSH2, MTDH, MYBBP1A, MYD88, MYDGF, NDRG1, NFKB1, NIBAN2, NLE1, NNT, NPM1, NQO2, OGT, P4HB, PARP1, PAWR, PDCD2, PDGFRB, PDIA3, PEA15, PHIP, PIAS4, PIK3CA, PLCG1, PLK1, PLSCR3, POR, PPID, PRKAA1, PRKCA, PRKDC, PRPF38B, PSME3, PTGIS, PTPMT1, PTPN1, PTPN2, PYCARD, RBM25, RELA, RPS6KA1, RPS6KA3, RTKN, SARM1, SERBP1, SERPINB9, SIN3A, SIRT1, SIRT2, SLC25A5, SLK, SNX6, SRC, TAX1BP1, TCP1, TGM2, THBS1, TIA1, TIGAR, TIMP3, TMF1, TOP2A, TRAP1, TXNDC5, UBE2O, UFL1, UNG, USP47, YAP1]	
regulation of apoptotic process	GO_BiologicalProcess-EBI-UniProt-GOA-ACAP-ARAP_13.05.2021_00h00	7.56E-07	10.72576	167	[AATF, ACTN1, ACTN4, ADAM10, ADAR, AKT1, ANO6, ANP32E, ANXA1, ANXA4, ANXA5, APAF1, API5, APOE, ARF4, ARHGDIA, ARHGEF1, ARHGEF11, ARHGEF12, ARL6IP5, ASNS, AURKA, AURKB, B4GALT1, BAG1, BAG3, BIN1, BIRC6, BRMS1, C1QBP, CALR, CAMK2D, CAPN2, CASP3, CAST, CAT, CAV1, CBL, CCAR2, CCND2, CD44, CDC42, CDC73, CHEK2, CNTFR, COPS5, CTNNB1, CTNNBL1, CTSC, CYFIP1, DAPK1, DAXX, DDRGK1, DDX20, DDX42, DIPK2A, DNMT1, ECT2, EEF1E1, EGFR, EPCAM, ETFA, FGG, FKBP8, GATA6, GBE1, GCLC, GCLM, GLMN, GPI, GSDME, GSN, HDAC2, HDAC4, HELLS, HMOX1, HSP90AB1, HSP90B1, HSPA5, HSPA9, HSPB1, HSPD1, HTT, ILK, IRF3, ITCH, ITGA5, ITGA6, ITGAV, ITGB1, KDM1A, KRT18, LGALS1, LRP1, MAGED1, MAP2K6, MAPK1, MAPK8, MICAL1, MSH2, MTDH, MYBBP1A, MYD88, MYDGF, NDRG1, NFKB1, NIBAN2, NLE1, NNT, NPM1, NQO2, P4HB, PARP1, PAWR, PDCD2, PDGFRB, PDIA3, PEA15, PHIP, PIAS4, PIK3CA, PLCG1, PLK1, PLSCR3, POR, PPID, PRKAA1, PRKCA,	No change

					PRKDC, PRPF38B, PSME3, PTGIS, PTPMT1, PTPN1, PTPN2, PYCARD, RBM25, RELA, RPS6KA1, RPS6KA3, RTKN, SARM1, SERBP1, SERPINB9, SIN3A, SIRT1, SIRT2, SLC25A5, SLK, SNX6, SRC, TAX1BP1, TCP1, TGM2, THBS1, TIA1, TIGAR, TIMP3, TMF1, TOP2A, TRAP1, TXNDC5, UBE2O, UFL1, UNG, USP47, YAP1]	
catalytic activity, acting on a tRNA	GO_MolecularFunction-EBI-UniProt-GOA-ACAP-ARAP_13.05.2021_00h00	1.30E-26	18.93939	25	[AIMP2, DARS2, ELAC2, GARS1, LARS1, LARS2, MARS1, METTL1, NSUN2, PRORP, PUS1, PUS3, QRSL1, SARS1, SARS2, TARS2, THUMPD3, TRMT1, TRMT10C, TRMT1L, TRMT5, TRPT1, TSEN34, WARS1, YARS2]	Upregulation
tRNA binding	GO_MolecularFunction-EBI-UniProt-GOA-ACAP-ARAP_13.05.2021_00h00	1.30E-26	26.0274	19	[DARS2, ELP1, ELP3, HSD17B10, MARS1, METTL1, MRPS27, NSUN2, PTCD1, PUS1, SARS1, SARS2, SSB, TRMT1, TRMT10C, TRMT1L, XPO5, YARS2, YRDC]	Upregulation
mitochondrial RNA metabolic process	GO_BiologicalProcess-EBI-UniProt-GOA-ACAP-ARAP_13.05.2021_00h00	1.30E-26	37.5	18	[CDK5RAP1, DARS2, ELAC2, GARS1, HSD17B10, LRPPRC, PNPT1, PRKAA1, PRORP, PUS1, SARS2, SLIRP, TARS2, TBRG4, TFB1M, TRMT10C, TRMT5, YARS2]	Upregulation
ncRNA metabolic process	GO_BiologicalProcess-EBI-UniProt-GOA-ACAP-ARAP_13.05.2021_00h00	1.30E-26	21.54399	120	[AGO1, AGO2, AIMP2, BMS1, BOP1, CDK5RAP1, DARS2, DCAF13, DDX10, DDX18, DDX21, DDX47, DDX49, DDX52, DDX54, DHX37, DICER1, EEF1E1, EIF4A3, ELAC2, ELP1, ELP3, ERCC2, ESF1, EXOSC10, EXOSC2, EXOSC5, EXOSC7, FTSJ3, GARS1, GEMIN4, HEATR1, HSD17B10, IGF2BP3, IMP3, LARS1, LARS2, LAS1L, LIN28A, LYAR, MARS1, MDN1, METTL1, MPHOSPH10, MTREX, NEDD4, NFKB1, NOL10, NOL11, NOL6, NOL9, NOP14, NOP56, NOP58, NOP9, NPM3, NSUN2, NSUN5, NUDT16, NVL, PARN, PDCD11, PELP1, PES1, PNPT1, PRKDC, PRORP, PTCD1, PUS1, PUS3, PUS7, PWP2, QRSL1, RAN, RELA, RPF2, RPL13A, RPL22, RPL27A, RPL4, RPL5, RPL7A, RPS2, RPS21,	Upregulation

					RPS8, RRP12, RRP7A, RRS1, SARS1, SARS2, SBDS, SSB, TARS2, TBL3, TEX10, TFB1M, THUMPD3, TRIM71, TRMT1, TRMT10C, TRMT1L, TRMT5, TRPT1, TSEN34, TSR1, TUT1, UTP15, UTP18, UTP20, UTP25, UTP4, WARS1, WDR18, WDR3, WDR36, WDR43, XPO5, XRN1, XRN2, YARS2]	
mitochondrial RNA processing	GO_BiologicalProcess-EBI-UniProt-GOA-ACAP-ARAP_13.05.2021_00h00	1.30E-26	50	9	[CDK5RAP1, ELAC2, HSD17B10, PNPT1, PRORP, PUS1, TBRG4, TRMT10C, TRMT5]	Upregulation
tRNA metabolic process	GO_BiologicalProcess-EBI-UniProt-GOA-ACAP-ARAP_13.05.2021_00h00	1.30E-26	18.46154	36	[AIMP2, CDK5RAP1, DARS2, EEF1E1, ELAC2, ELP1, ELP3, EXOSC10, EXOSC2, EXOSC7, GARS1, HSD17B10, LARS1, LARS2, MARS1, METTL1, NSUN2, PRORP, PTCD1, PUS1, PUS3, PUS7, QRSL1, SARS1, SARS2, SSB, TARS2, THUMPD3, TRMT1, TRMT10C, TRMT1L, TRMT5, TRPT1, TSEN34, WARS1, YARS2]	Upregulation
hydrolase activity, acting on acid anhydrides, in phosphorus-containing anhydrides	GO_MolecularFunction-EBI-UniProt-GOA-ACAP-ARAP_13.05.2021_00h00	3.23E-08	11.24595	139	[ABCB10, ALPL, ARAP1, ARAP3, ARF6, ARFGAP1, ARFGAP3, ARFGEF1, ARHGAP1, ARHGAP28, ARHGAP5, ARHGDIA, ARHGEF1, ARHGEF11, ARHGEF12, ARL1, ARL2, ARL3, ASAP1, ATL2, ATL3, ATP2B1, BAG1, BAG2, BAG3, BMS1, CDC42, CORO1C, DHX9, DOCK7, DRG1, ECT2, EEF2, EFL1, EFTUD2, EIF5B, EPHB3, EPS8L2, ERCC2, ETFA, F11R, FARP1, FERMT2, GARS1, GBF1, GDI1, GMPS, GNB2, GNL2, GPSM1, GTPBP1, GUF1, HERC2, HSPH1, IPO5, IQGAP1, IQGAP2, IQGAP3, ITGA6, ITGB1, KIF11, KIF20A, KIF21A, KIF22, KIF23, KIF2C, KIF5B, KLC1, LARS1, LIMS1, LSG1, MYH10, MYH9, MYO1C, MYO1E, MYO5A, MYO9B, NDRG1, NUCB2, NUDT16, PGAM5, PICALM, PLXNB2, PRPF38B, PRUNE1, PYCARD, RAB14, RAB18, RAB1A, RAB23, RAB2A, RAB32, RAB5B, RAB5C, RAB8A, RABGAP1L, RAC3, RALGPS2, RAN,	Downregulation

					RANBP2, RANGAP1, RAP1GDS1, RAP2A, RAP2B, RAP2C, RCC2, RDX, RHEB, RIC1, RIC8A, RIT1, RP2, RRAS, RTKN, S100A10, SAR1A, SBF1, SEC23A, SEC23B, SEPTIN10, SEPTIN11, SEPTIN2, SEPTIN7, SEPTIN8, SESN2, SIL1, SMAP2, SMARCA5, SNX9, SRPRA, TBC1D17, TBC1D22B, TBC1D4, TGM2, TMED2, TSR1, TUBA4A, TUBB6, ZC3H15]	
pyrophosphatase activity	GO_MolecularFunction-EBI-UniProt-GOA-ACAP-ARAP_13.05.2021_00h00	3.23E-08	11.21113	137	[ABCB10, ALPL, ARAP1, ARAP3, ARF6, ARFGAP1, ARFGAP3, ARFGEF1, ARHGAP1, ARHGAP28, ARHGAP5, ARHGDIA, ARHGEF1, ARHGEF11, ARHGEF12, ARL1, ARL2, ARL3, ASAP1, ATL2, ATL3, ATP2B1, BAG1, BAG2, BAG3, BMS1, CDC42, CORO1C, DHX9, DOCK7, DRG1, ECT2, EEF2, EFL1, EFTUD2, EIF5B, EPHB3, EPS8L2, ETFA, F11R, FARP1, FERMT2, GARS1, GBF1, GDI1, GMPS, GNB2, GNL2, GPSM1, GTPBP1, GUF1, HERC2, HSPH1, IPO5, IQGAP1, IQGAP2, IQGAP3, ITGA6, ITGB1, KIF11, KIF20A, KIF21A, KIF22, KIF23, KIF2C, KIF5B, KLC1, LARS1, LIMS1, LSG1, MYH10, MYH9, MYO1C, MYO1E, MYO5A, MYO9B, NDRG1, NUCB2, NUDT16, PGAM5, PICALM, PLXNB2, PRPF38B, PRUNE1, PYCARD, RAB14, RAB18, RAB1A, RAB23, RAB2A, RAB32, RAB5B, RAB5C, RAB8A, RABGAP1L, RAC3, RALGPS2, RAN, RANBP2, RANGAP1, RAP1GDS1, RAP2A, RAP2B, RAP2C, RCC2, RDX, RHEB, RIC1, RIC8A, RIT1, RP2, RRAS, RTKN, S100A10, SAR1A, SBF1, SEC23A, SEC23B, SEPTIN10, SEPTIN11, SEPTIN2, SEPTIN7, SEPTIN8, SESN2, SIL1, SMAP2, SNX9, SRPRA, TBC1D17, TBC1D22B, TBC1D4, TGM2, TMED2, TSR1, TUBA4A, TUBB6, ZC3H15]	Downregulation
nucleoside-triphosphatase activity	GO_MolecularFunction-EBI-UniProt-GOA-ACAP-	3.23E-08	11.33047	132	[ABCB10, ARAP1, ARAP3, ARF6, ARFGAP1, ARFGAP3, ARFGEF1, ARHGAP1, ARHGAP28, ARHGAP5, ARHGDIA, ARHGEF1,	Downregulation

	ARAP_13.05.2021_00h00				ARHGEF11, ARHGEF12, ARL1, ARL2, ARL3, ASAP1, ATL2, ATL3, ATP2B1, BAG1, BAG2, BAG3, BMS1, CDC42, CORO1C, DHX9, DOCK7, DRG1, ECT2, EEF2, EFL1, EFTUD2, EIF5B, EPHB3, EPS8L2, ETFA, F11R, FARP1, FERMT2, GBF1, GDI1, GNB2, GNL2, GPSM1, GTPBP1, GUF1, HERC2, HSPH1, IPO5, IQGAP1, IQGAP2, IQGAP3, ITGA6, ITGB1, KIF11, KIF20A, KIF21A, KIF22, KIF23, KIF2C, KIF5B, KLC1, LARS1, LIMS1, LSG1, MYH10, MYH9, MYO1C, MYO1E, MYO5A, MYO9B, NDRG1, NUCB2, PGAM5, PICALM, PLXNB2, PRPF38B, PYCARD, RAB14, RAB18, RAB1A, RAB23, RAB2A, RAB32, RAB5B, RAB5C, RAB8A, RABGAP1L, RAC3, RALGPS2, RAN, RANBP2, RANGAP1, RAP1GDS1, RAP2A, RAP2B, RAP2C, RCC2, RDX, RHEB, RIC1, RIC8A, RIT1, RP2, RRAS, RTKN, S100A10, SAR1A, SBF1, SEC23A, SEC23B, SEPTIN10, SEPTIN11, SEPTIN2, SEPTIN7, SEPTIN8, SESN2, SIL1, SMAP2, SNX9, SRPRA, TBC1D17, TBC1D22B, TBC1D4, TGM2, TMED2, TSR1, TUBA4A, TUBB6, ZC3H15]	
guanyl ribonucleotide binding	GO_MolecularFunction-EBI-UniProt-GOA-ACAP-ARAP_13.05.2021_00h00	3.23E-08	13.56185	91	[AK4, ANXA6, ARF4, ARF6, ARFGEF1, ARHGAP28, ARHGAP5, ARHGDIA, ARHGEF1, ARHGEF11, ARHGEF12, ARL1, ARL2, ARL3, ATL2, ATL3, BMS1, CDC42, DAPK1, DOCK7, DRG1, ECT2, EEF2, EFL1, EFTUD2, EHD1, EHD2, EHD4, EIF5B, EPS8L2, ETFA, FARP1, GBF1, GDI1, GLUD1, GMPPB, GNL2, GNL3, GPSM1, GTPBP1, GUF1, HERC2, HSP90AB1, LANCL2, LRRK1, LSG1, NDRG1, NUCB2, NUDT16, PIP4K2B, PRPF38B, RAB14, RAB18, RAB1A, RAB23, RAB2A, RAB32, RAB5B, RAB5C, RAB8A, RABL6, RAC3, RALGPS2, RAN, RAP2A, RAP2B, RAP2C, RCC2, RHEB, RIC1, RIC8A, RIT1, RP2, RRAS, RTKN, SAMHD1, SAR1A, SBF1, SEPHS1, SEPTIN10, SEPTIN11, SEPTIN2, SEPTIN7,	Downregulation

					SEPTIN8, SESN2, SRPRA, SRPRB, TGM2, TSR1, TUBA4A, TUBB6]	
GTP binding	GO_MolecularFunction-EBI-UniProt-GOA-ACAP-ARAP_13.05.2021_00h00	3.23E-08	13.7931	88	[AK4, ANXA6, ARF4, ARF6, ARFGEF1, ARHGAP28, ARHGAP5, ARHGEF1, ARHGEF11, ARHGEF12, ARL1, ARL2, ARL3, ATL2, ATL3, BMS1, CDC42, DAPK1, DOCK7, DRG1, ECT2, EEF2, EFL1, EFTUD2, EHD1, EHD2, EHD4, EIF5B, EPS8L2, ETFA, FARP1, GBF1, GLUD1, GMPPB, GNL2, GNL3, GPSM1, GTPBP1, GUF1, HERC2, HSP90AB1, LANCL2, LRRK1, LSG1, NDRG1, NUCB2, NUDT16, PIP4K2B, PRPF38B, RAB14, RAB18, RAB1A, RAB23, RAB2A, RAB32, RAB5B, RAB5C, RAB8A, RABL6, RAC3, RALGPS2, RAN, RAP2A, RAP2B, RAP2C, RCC2, RHEB, RIC1, RIC8A, RIT1, RP2, RRAS, RTKN, SAMHD1, SAR1A, SBF1, SEPHS1, SEPTIN10, SEPTIN11, SEPTIN2, SEPTIN7, SEPTIN8, SRPRA, SRPRB, TGM2, TSR1, TUBA4A, TUBB6]	Downregulation
GTPase activity	GO_MolecularFunction-EBI-UniProt-GOA-ACAP-ARAP_13.05.2021_00h00	3.23E-08	11.50259	111	[ARAP1, ARAP3, ARF6, ARFGAP1, ARFGAP3, ARFGEF1, ARHGAP1, ARHGAP28, ARHGAP5, ARHGDIA, ARHGEF1, ARHGEF11, ARHGEF12, ARL1, ARL2, ARL3, ASAP1, ATL2, ATL3, BMS1, CDC42, CORO1C, DOCK7, DRG1, ECT2, EEF2, EFL1, EFTUD2, EIF5B, EPHB3, EPS8L2, ETFA, F11R, FARP1, FERMT2, GBF1, GDI1, GNB2, GNL2, GPSM1, GTPBP1, GUF1, HERC2, IPO5, IQGAP1, IQGAP2, IQGAP3, ITGA6, ITGB1, LARS1, LIMS1, LSG1, MYO9B, NDRG1, NUCB2, PGAM5, PICALM, PLXNB2, PRPF38B, PYCARD, RAB14, RAB18, RAB1A, RAB23, RAB2A, RAB32, RAB5B, RAB5C, RAB8A, RABGAP1L, RAC3, RALGPS2, RAN, RANBP2, RANGAP1, RAP1GDS1, RAP2A, RAP2B, RAP2C, RCC2, RDX, RHEB, RIC1, RIC8A, RIT1, RP2, RRAS, RTKN, S100A10, SAR1A, SBF1, SEC23A, SEC23B, SEPTIN10, SEPTIN11, SEPTIN2, SEPTIN7, SEPTIN8, SESN2, SMAP2, SNX9, SRPRA,	Downregulation

					TBC1D17, TBC1D22B, TBC1D4, TGM2, TMED2, TSR1, TUBA4A, TUBB6, ZC3H15]	
response to temperature stimulus	GO_BiologicalProcess-EBI-UniProt-GOA-ACAP-ARAP_13.05.2021_00h00	1.82E-11	18	45	[AAAS, AKT1, ANTXR1, APPL2, ATP2B1, ATR, BAG1, BAG2, BAG3, CAMK2D, CCAR2, CIRBP, DAXX, ERO1A, FKBP4, GCLC, HDAC2, HMOX1, HSP90AB1, HSPA2, HSPD1, HSPH1, MAPK1, MMAB, NDC1, NUP107, NUP133, NUP160, NUP210, NUP35, NUP50, PDCL3, POLR1B, PRKAA1, PRKCA, PSIP1, RANBP2, RPA1, RPA2, RPA3, SEC13, SEH1L, SIRT1, TGFB1I1, THBS1]	Upregulation
response to heat	GO_BiologicalProcess-EBI-UniProt-GOA-ACAP-ARAP_13.05.2021_00h00	1.82E-11	22.59887	40	[AAAS, AKT1, ANTXR1, ATR, BAG1, BAG2, BAG3, CAMK2D, CCAR2, DAXX, FKBP4, GCLC, HDAC2, HMOX1, HSP90AB1, HSPA2, HSPD1, HSPH1, MAPK1, MMAB, NDC1, NUP107, NUP133, NUP160, NUP210, NUP35, NUP50, PDCL3, POLR1B, PRKCA, PSIP1, RANBP2, RPA1, RPA2, RPA3, SEC13, SEH1L, SIRT1, TGFB1I1, THBS1]	Upregulation
cellular response to heat	GO_BiologicalProcess-EBI-UniProt-GOA-ACAP-ARAP_13.05.2021_00h00	1.82E-11	26.51515	35	[AAAS, ANTXR1, ATR, BAG1, BAG2, BAG3, CAMK2D, CCAR2, DAXX, FKBP4, HDAC2, HMOX1, HSP90AB1, HSPD1, HSPH1, MAPK1, MMAB, NDC1, NUP107, NUP133, NUP160, NUP210, NUP35, NUP50, PDCL3, POLR1B, PRKCA, RANBP2, RPA1, RPA2, RPA3, SEC13, SEH1L, SIRT1, THBS1]	Upregulation
regulation of cellular response to heat	GO_BiologicalProcess-EBI-UniProt-GOA-ACAP-ARAP_13.05.2021_00h00	1.82E-11	32.18391	28	[AAAS, ANTXR1, ATR, BAG1, BAG2, BAG3, CAMK2D, CCAR2, FKBP4, HSP90AB1, HSPH1, MAPK1, MMAB, NDC1, NUP107, NUP133, NUP160, NUP210, NUP35, NUP50, POLR1B, RANBP2, RPA1, RPA2, RPA3, SEC13, SEH1L, SIRT1]	Upregulation
mRNA export from nucleus	GO_BiologicalProcess-EBI-UniProt-GOA-ACAP-ARAP_13.05.2021_00h00	1.82E-11	21.73913	25	[AAAS, CPSF2, CPSF3, DDX39B, EIF4A3, IWS1, NDC1, NSUN2, NUP107, NUP133, NUP160, NUP210, NUP35, NUP50, PABPN1, RANBP2, SEC13, SEH1L, SRSF1, SRSF11, SRSF7, SUPT6H, SYMPK, UPF2, UPF3B]	Upregulation
ncRNA export from nucleus	GO_BiologicalProcess-EBI-UniProt-	1.82E-11	37.5	15	[AAAS, NDC1, NOL6, NPM1, NUP107, NUP133, NUP160, NUP210, NUP35,	Upregulation

	GOA-ACAP-ARAP_13.05.2021_00h00				NUP50, RAN, RANBP2, SEC13, SEH1L, SSB]	
nucleus organization	GO_BiologicalProcess-EBI-UniProt-GOA-ACAP-ARAP_13.05.2021_00h00	1.14E-13	20.39474	31	[BIN1, CCNB1, CHEK1, DCTN1, EMD, FAM118B, IST1, LBR, NDC1, NECTIN2, NSFL1C, NUP107, NUP133, NUP160, NUP35, PLK1, POLR1B, PRKCA, RAN, RANGAP1, SEC13, SEH1L, SERBP1, SIRT2, SRPK1, SUN2, TBPL1, TMEM43, TMF1, VRK1, WRAP53]	Upregulation
membrane assembly	GO_BiologicalProcess-EBI-UniProt-GOA-ACAP-ARAP_13.05.2021_00h00	1.14E-13	28.26087	26	[ANK3, ANXA2, CAV1, CCNB1, CDH2, EMD, IST1, LBR, NDC1, NLGN4X, NSFL1C, NUP107, NUP133, NUP160, NUP35, PICALM, PTPRD, RAN, RANGAP1, RFTN1, S100A10, SEC13, SEH1L, SIRT2, SPTBN1, VRK1]	No change
mitotic nuclear division	GO_BiologicalProcess-EBI-UniProt-GOA-ACAP-ARAP_13.05.2021_00h00	1.14E-13	16.0221	58	[AAAS, AURKA, AURKB, BCCIP, BUB1B, CCNB1, CDC42, CDCA8, CHEK1, CHEK2, CLASP1, CUL7, DLGAP5, DRG1, EMD, EPS8, GOLGA2, INCENP, IST1, KIF11, KIF22, KIF23, KIF2C, KNTC1, LBR, MACROH2A1, MKI67, NCAPD2, NCAPG, NCAPG2, NDC1, NDC80, NDRG1, NSFL1C, NUP107, NUP133, NUP160, NUP35, NUSAP1, PDGFRB, PDS5A, PHIP, PLK1, POGZ, PPP1R9B, RAN, RANGAP1, RRS1, SEC13, SEH1L, SET, SIRT2, SMC2, TACC3, TPX2, TRIP13, TTK, VRK1]	Upregulation
nuclear envelope organization	GO_BiologicalProcess-EBI-UniProt-GOA-ACAP-ARAP_13.05.2021_00h00	1.14E-13	25	21	[CCNB1, DCTN1, EMD, IST1, LBR, NDC1, NSFL1C, NUP107, NUP133, NUP160, NUP35, PLK1, PRKCA, RAN, RANGAP1, SEC13, SEH1L, SIRT2, SUN2, TMEM43, VRK1]	No change
nuclear membrane reassembly	GO_BiologicalProcess-EBI-UniProt-GOA-ACAP-ARAP_13.05.2021_00h00	1.14E-13	28.57143	16	[CCNB1, EMD, IST1, LBR, NDC1, NSFL1C, NUP107, NUP133, NUP160, NUP35, RAN, RANGAP1, SEC13, SEH1L, SIRT2, VRK1]	Upregulation
response to topologically incorrect protein	GO_BiologicalProcess-EBI-UniProt-GOA-ACAP-ARAP_13.0	7.32E-15	24.41315	52	[ABCB10, ARFGAP1, ARFGAP3, AUP1, BAG3, CALR, CANX, CDK5RAP3, COPS5, CUL7, DAXX, DCTN1, DDRGK1, EDEM3, ERO1A, ERP44, FAF2, FKBP14, GOSR2,	Downregulation

	5.2021_00h00				HSP90AB1, HSP90B1, HSPA13, HSPA14, HSPA2, HSPA4, HSPA5, HSPA9, HSPB1, HSPD1, HSPE1, HSPH1, KDELR3, MYDGF, OPTN, PDIA6, PTPN1, PTPN2, SEC31A, SERPINH1, SRPRA, SRPRB, SSR1, STT3B, TBL2, THBS1, TLN1, TMED2, UFL1, UGGT1, UGGT2, YIF1A, YOD1]	
response to endoplasmic reticulum stress	GO_BiologicalProcess-EBI-UniProt-GOA-ACAP-ARAP_13.05.2021_00h00	7.32E-15	18.42105	56	[ANKZF1, ARFGAP1, ARFGAP3, AUP1, CALR, CANX, CAV1, CDK5RAP3, COPS5, CUL7, DCTN1, DDRGK1, ECPAS, EDEM3, EEF2, ERLEC1, ERLIN2, ERO1A, ERP44, FAF2, FBXO2, FKBP14, GET4, GOSR2, HSP90B1, HSPA5, KDELR3, MAN1A1, MYDGF, NIBAN1, P4HB, PDIA3, PDIA4, PDIA6, PSMC6, PTPN1, PTPN2, SEC16A, SEC31A, SEL1L, SESN2, SIRT1, SRPRA, SRPRB, SSR1, STT3B, TBL2, THBS1, TLN1, TMED2, UFL1, UGGT1, UGGT2, USP19, YIF1A, YOD1]	Downregulation
response to unfolded protein	GO_BiologicalProcess-EBI-UniProt-GOA-ACAP-ARAP_13.05.2021_00h00	7.32E-15	25.65445	49	[ABCB10, ARFGAP1, ARFGAP3, BAG3, CALR, CANX, CDK5RAP3, COPS5, CUL7, DAXX, DCTN1, DDRGK1, EDEM3, ERO1A, ERP44, FAF2, FKBP14, GOSR2, HSP90AB1, HSP90B1, HSPA13, HSPA14, HSPA2, HSPA4, HSPA5, HSPA9, HSPB1, HSPD1, HSPE1, HSPH1, KDELR3, MYDGF, OPTN, PDIA6, PTPN1, PTPN2, SEC31A, SERPINH1, SRPRA, SRPRB, SSR1, STT3B, TBL2, THBS1, TLN1, TMED2, UFL1, YIF1A, YOD1]	Downregulation
cellular response to topologically incorrect protein	GO_BiologicalProcess-EBI-UniProt-GOA-ACAP-ARAP_13.05.2021_00h00	7.32E-15	23.29545	41	[ABCB10, ARFGAP1, ARFGAP3, AUP1, BAG3, CALR, CANX, CDK5RAP3, COPS5, CUL7, DAXX, DCTN1, DDRGK1, ERO1A, FKBP14, GOSR2, HSP90B1, HSPA13, HSPA14, HSPA2, HSPA5, HSPA9, HSPD1, KDELR3, MYDGF, OPTN, PDIA6, PTPN1, PTPN2, SEC31A, SRPRA, SRPRB, SSR1, TBL2, TLN1, TMED2, UFL1, UGGT1, UGGT2, YIF1A, YOD1]	Downregulation
endoplasmic reticulum unfolded protein response	GO_BiologicalProcess-EBI-UniProt-GOA-ACAP-ARAP_13.0	7.32E-15	21.80451	29	[ARFGAP1, ARFGAP3, CALR, CANX, CDK5RAP3, COPS5, CUL7, DCTN1, DDRGK1, ERO1A, FKBP14, GOSR2, HSP90B1, HSPA5, KDELR3,	Downregulation

	5.2021_00h00				MYDGF, PDIA6, PTPN1, PTPN2, SEC31A, SRPRA, SRPRB, SSR1, TBL2, TLN1, TMED2, UFL1, YIF1A, YOD1]	
cellular response to unfolded protein	GO_BiologicalProcess-EBI-UniProt-GOA-ACAP-ARAP_13.05.2021_00h00	7.32E-15	24.35897	38	[ABCB10, ARFGAP1, ARFGAP3, BAG3, CALR, CANX, CDK5RAP3, COPS5, CUL7, DAXX, DCTN1, DDRGK1, ERO1A, FKBP14, GOSR2, HSP90B1, HSPA13, HSPA14, HSPA2, HSPA5, HSPA9, HSPD1, KDELR3, MYDGF, OPTN, PDIA6, PTPN1, PTPN2, SEC31A, SRPRA, SRPRB, SSR1, TBL2, TLN1, TMED2, UFL1, YIF1A, YOD1]	Downregulation
IRE1-mediated unfolded protein response	GO_BiologicalProcess-EBI-UniProt-GOA-ACAP-ARAP_13.05.2021_00h00	7.32E-15	28.57143	20	[ARFGAP1, ARFGAP3, COPS5, CUL7, DCTN1, DDRGK1, FKBP14, GOSR2, HSPA5, KDELR3, MYDGF, PDIA6, PTPN1, SEC31A, SRPRA, SRPRB, SSR1, TLN1, UFL1, YIF1A]	Downregulation
monosaccharide binding	GO_MolecularFunction-EBI-UniProt-GOA-ACAP-ARAP_13.05.2021_00h00	5.71E-11	23.52941	20	[GALK1, GPI, GYS1, HK1, HK2, HOOK3, LMAN1, MANBA, P3H1, P3H3, P4HA1, P4HA2, P4HTM, PFKL, PFKM, PFKP, PLOD1, PLOD2, PLOD3, UGP2]	Downregulation
extracellular matrix organization	GO_BiologicalProcess-EBI-UniProt-GOA-ACAP-ARAP_13.05.2021_00h00	5.71E-11	13.93258	62	[ADAM10, ANTXR1, ANXA2, B4GALT1, CAPN1, CAPN2, CAPNS1, CAV1, CD44, CLASP1, COL1A1, COL1A2, COL4A1, COL4A2, COL5A1, COL5A2, COL6A1, COL6A2, COL6A3, COLGALT1, CRTAP, EGFL6, ERCC2, ERO1A, F11R, FBN2, FGG, FKBP10, FN1, FSCN1, GLMN, GSN, HTRA1, ITGA5, ITGA6, ITGAV, ITGB1, JAM3, LAMB1, LAMC1, LRP1, MYO1E, NCAPG, NID1, P3H1, P3H4, P4HA1, P4HA2, P4HB, PLEC, PLOD1, PLOD2, PLOD3, PPIB, PRDX4, PXDN, RIC1, RIC8A, SERPINH1, SLC2A10, SPARC, THBS1]	Downregulation
collagen fibril organization	GO_BiologicalProcess-EBI-UniProt-GOA-ACAP-ARAP_13.05.2021_00h00	5.71E-11	23.63636	26	[ANXA2, COL1A1, COL1A2, COL4A1, COL4A2, COL5A1, COL5A2, COL6A1, COL6A2, COL6A3, COLGALT1, CRTAP, FKBP10, ITGA6, P3H1, P3H4, P4HA1, P4HA2, P4HB, PLEC, PLOD1, PLOD2, PLOD3, PPIB, PXDN, SERPINH1]	Downregulation

protein hydroxylation	GO_BiologicalProcess-EBI-UniProt-GOA-ACAP-ARAP_13.05.2021_00h00	5.71E-11	55.55556	15	[ASPH, CRTAP, ERO1A, FKBP10, P3H1, P3H3, P3H4, P4HA1, P4HA2, P4HB, P4HTM, PLOD1, PLOD2, PLOD3, PRDX4]	Downregulation
peptidyl-proline modification	GO_BiologicalProcess-EBI-UniProt-GOA-ACAP-ARAP_13.05.2021_00h00	5.71E-11	34.32836	23	[CRTAP, CWC27, ERO1A, FKBP10, FKBP11, FKBP14, FKBP15, FKBP4, FKBP5, FKBP7, FKBP8, FKBP9, P3H1, P3H3, P4HA1, P4HA2, P4HB, P4HTM, PPIB, PPIC, PPID, PRDX4, RANBP2]	Downregulation
peptidyl-lysine hydroxylation	GO_BiologicalProcess-EBI-UniProt-GOA-ACAP-ARAP_13.05.2021_00h00	5.71E-11	75	6	[FKBP10, P3H3, P3H4, PLOD1, PLOD2, PLOD3]	Downregulation
peptidyl-proline hydroxylation	GO_BiologicalProcess-EBI-UniProt-GOA-ACAP-ARAP_13.05.2021_00h00	5.71E-11	56.25	9	[CRTAP, ERO1A, P3H1, P3H3, P4HA1, P4HA2, P4HB, P4HTM, PRDX4]	Downregulation
protein peptidyl-prolyl isomerization	GO_BiologicalProcess-EBI-UniProt-GOA-ACAP-ARAP_13.05.2021_00h00	5.71E-11	28	14	[CWC27, FKBP10, FKBP11, FKBP14, FKBP15, FKBP4, FKBP5, FKBP7, FKBP8, FKBP9, PPIB, PPIC, PPID, RANBP2]	No change
ATPase, acting on DNA	GO_MolecularFunction-EBI-UniProt-GOA-ACAP-ARAP_13.05.2021_00h00	3.38E-28	30.73171	63	[ANXA1, BPTF, CDK7, CHD1, CHD4, CHTF18, DDX10, DDX18, DDX20, DDX21, DDX24, DDX28, DDX31, DDX39B, DDX41, DDX42, DDX46, DDX47, DDX49, DDX5, DDX52, DDX54, DDX6, DHX15, DHX30, DHX33, DHX37, DHX57, DHX8, DHX9, DICER1, EIF4A3, ERCC2, ERCC6L, G3BP1, HELLS, MCM2, MCM3, MCM4, MCM5, MCM6, MCM7, MSH2, MSH3, MSH6, MTREX, RAD51, RAD54L2, RFC1, RFC2, RFC3, RFC5, SIRT1, SMARCA4, SMARCA5, SMARCAD1, SNRNP200, SSBP1, TOP2A, TTF2, XRCC5, XRCC6, YTHDC2]	Upregulation
helicase activity	GO_MolecularFunction-EBI-UniProt-GOA-ACAP-ARAP_13.0	3.38E-28	32.77778	59	[ANXA1, CHD1, CHD4, CHTF18, DDX10, DDX18, DDX20, DDX21, DDX24, DDX28, DDX31, DDX39B, DDX41, DDX42, DDX46, DDX47, DDX49, DDX5, DDX52, DDX54, DDX6, DHX15,	Upregulation

	5.2021_00 h00				DHX30, DHX33, DHX37, DHX57, DHX8, DHX9, DICER1, EIF4A3, ERCC2, ERCC6L, G3BP1, HELLS, MCM2, MCM3, MCM4, MCM5, MCM6, MCM7, MSH2, MSH3, MSH6, MTREX, RAD51, RAD54L2, RFC2, RFC3, RFC5, SIRT1, SMARCA4, SMARCA5, SMARCAD1, SNRNP200, SSBP1, TTF2, XRCC5, XRCC6, YTHDC2]	
RNA helicase activity	GO_MolecularFunction-EBI-UniProt-GOA-ACAP-ARAP_13.05.2021_00h00	3.38E-28	36.70886	29	[DDX10, DDX18, DDX20, DDX21, DDX24, DDX28, DDX31, DDX39B, DDX41, DDX42, DDX46, DDX47, DDX49, DDX5, DDX52, DDX54, DDX6, DHX15, DHX30, DHX33, DHX37, DHX57, DHX8, DHX9, EIF4A3, G3BP1, MTREX, SNRNP200, YTHDC2]	Upregulation
chromosome organization	GO_BiologicalProcess-EBI-UniProt-GOA-ACAP-ARAP_13.05.2021_00h00	3.38E-28	14.41441	192	[AASS, ACIN1, AEBP2, ANKRD28, ANP32E, ANTXR1, ANXA1, ATF7IP, ATR, AURKA, AURKB, BAZ1B, BPTF, BRD3, BRD4, BRMS1, BRWD1, BUB1B, CAMK1, CAMK2D, CBX2, CCNB1, CCT2, CCT3, CCT4, CCT5, CCT6A, CCT7, CCT8, CDC73, CDCA8, CHAF1A, CHD1, CHD4, CHEK1, CHTF18, COPS2, COPS5, COPS7A, CTCF, CTNNB1, CUL4A, CUL4B, DAXX, DCAF1, DHX9, DLGAP5, DNMT1, DNMT3A, DNMT3B, EHMT1, EMSY, ERCC2, ERCC6L, EXOSC10, FANCD2, G3BP1, GNL3, GTF3C4, HAT1, HCFC1, HDAC2, HDAC4, HELLS, HP1BP3, HSP90AB1, HSPA2, HUWE1, INCENP, INO80C, IPO4, IWS1, JADE1, JARID2, KAT7, KDM1A, KDM2A, KDM3B, KIF22, KIF23, KIF2C, KNTC1, LRWD1, MACROH2A1, MAP1S, MAPK1, MAPK8, MBD3, MCM2, MCM3, MCM4, MCM5, MCM6, MCM7, MCMBP, MKI67, MLH1, MMAB, MSH2, MSH3, MSH6, MTA3, NCAPD2, NCAPG, NCAPG2, NDC1, NDC80, NEK7, NPM1, NPM3, NUP107, NUP133, NUSAP1, OGT, P3H4, PAK1, PARN, PARP1, PAXBP1, PDS5A, PHC1, PLK1, POGZ, POLA1, POLA2, POLD1, POLR1B, PPM1F, PRIM1, PRIM2, PRKAA1, PRKCA, PRKDC, PURA,	Upregulation

					RAD51, RAD54L2, RAN, RBBP5, RELA, RFC1, RFC2, RFC3, RFC5, RIF1, RIOX1, RPA1, RPA2, RPA3, RPS6KA4, RRS1, SART3, SEH1L, SET, SETD7, SETDB1, SIN3A, SIRT1, SIRT2, SMARCA4, SMARCA5, SMARCAD1, SMARCC1, SMARCD1, SMARCD2, SMC2, SRC, SRPK1, SSBP1, SSRP1, SUPT16H, SUPT6H, TACC3, TCP1, TELO2, TLK1, TOP2A, TRIM28, TRIP12, TRIP13, TTK, TTLL12, UBR5, UFL1, USP15, USP7, VRK1, WAPL, WRAP53, XRCC5, XRCC6, XRN1, ZNF462]	
protein-DNA complex subunit organization	GO_BiologicalProcess-EBI-UniProt-GOA-ACAP-ARAP_13.05.2021_00h00	3.38E-28	14.20613	51	[ANP32E, ATF7IP, BPTF, CCNH, CDK7, CHAF1A, CTCF, CUL4A, CUL4B, DAXX, DLGAP5, ERCC2, GTF2E1, HAT1, HELLS, HP1BP3, IPO4, MACROH2A1, MCM2, MCM3, MCM4, MCM5, MCM6, MCM7, NPM1, ORC2, ORC3, ORC4, ORC5, PARP1, POGZ, POLR1B, PSMC6, PSMD1, PSMD2, PSMD9, PSME3, RAD51, RPA1, RPA2, RPA3, SART3, SET, SMARCA4, SMARCA5, SMARCC1, SMARCD1, SMARCD2, SUPT16H, SUPT6H, ZFP36L2]	Upregulation
DNA conformation change	GO_BiologicalProcess-EBI-UniProt-GOA-ACAP-ARAP_13.05.2021_00h00	3.38E-28	15.95745	60	[ACIN1, ANXA1, ATF7IP, CCNB1, CHAF1A, CHD1, CHD4, CHTF18, CTCF, CUL4A, CUL4B, DAXX, DHX9, DNMT1, DNMT3A, ERCC2, ERCC6L, G3BP1, HAT1, HELLS, HP1BP3, INCENP, IPO4, MACROH2A1, MBD3, MCM2, MCM3, MCM4, MCM5, MCM6, MCM7, NCAPD2, NCAPG, NCAPG2, NPM1, NUSAP1, PARP1, PLK1, PURA, RAD51, RAD54L2, RFC2, RFC3, RFC5, RPA1, SART3, SET, SETDB1, SIRT1, SIRT2, SMARCA5, SMARCAD1, SMC2, SRPK1, SSBP1, TOP2A, TRIM28, WAPL, XRCC5, XRCC6]	Upregulation
nuclear DNA replication	GO_BiologicalProcess-EBI-UniProt-GOA-ACAP-ARAP_13.05.2021_00h00	3.38E-28	31.57895	24	[CHTF18, GINS3, LIG1, MCM2, MCM3, MCM4, MCM5, MCM6, MCM7, ORC3, POLA1, POLA2, POLD1, POLR1B, PRIM1, PRIM2, RAD51, RFC1, RFC2, RFC3, RFC5, RPA1, RPA2, RPA3]	Upregulation

DNA unwinding involved in DNA replication	GO_BiologicalProcess-EBI-UniProt-GOA-ACAP-ARAP_13.05.2021_00h00	3.38E-28	47.05882	8	[MCM2, MCM4, MCM6, MCM7, PURA, RAD51, RPA1, SSBP1]	Upregulation
DNA duplex unwinding	GO_BiologicalProcess-EBI-UniProt-GOA-ACAP-ARAP_13.05.2021_00h00	3.38E-28	25.21739	29	[ANXA1, CHD1, CHD4, CHTF18, CUL4A, CUL4B, DHX9, ERCC2, ERCC6L, G3BP1, MCM2, MCM3, MCM4, MCM5, MCM6, MCM7, PARP1, PURA, RAD51, RAD54L2, RFC2, RFC3, RFC5, RPA1, SMARCAD1, SSBP1, TOP2A, XRCC5, XRCC6]	Upregulation
anion binding	GO_MolecularFunction-EBI-UniProt-GOA-ACAP-ARAP_13.05.2021_00h00	6.26E-51	14.68455	405	[ABAT, ABCB10, ABCB7, ABCC1, ABCF1, ACACA, ACAD8, ACAD9, ACAT1, ACLY, ACOX3, ACSL1, ACSL4, ACSS2, ACSS3, ACTBL2, ACTR1B, ACTR2, AK1, AK4, AKT1, ANTXR1, ANXA2, ANXA6, APAF1, APRT, ARAP1, ARAP3, ARF4, ARF6, ARFGEF1, ARHGAP28, ARHGAP5, ARHGDIA, ARHGEF1, ARHGEF11, ARHGEF12, ARL1, ARL2, ARL3, ASAP1, ASNS, ATL2, ATL3, ATP1A1, ATP2B1, ATP2B2, ATP2B4, ATP6V1A, ATR, AURKA, AURKB, BAZ1B, BMS1, BUB1B, CAMK1, CAMK2D, CASK, CASTOR2, CCT2, CCT3, CCT4, CCT5, CCT6A, CCT7, CCT8, CDC42, CDC42BPA, CDC42BPB, CDK7, CHD1, CHD4, CHEK1, CHEK2, CHST14, CHTF18, CLASP1, CMPK1, CTPS1, CTSC, CYB5R1, CYB5R3, DAPK1, DARS2, DCAF1, DDX10, DDX18, DDX20, DDX21, DDX24, DDX28, DDX31, DDX39B, DDX41, DDX42, DDX46, DDX47, DDX49, DDX5, DDX52, DDX54, DDX6, DECR1, DGKA, DHX15, DHX30, DHX33, DHX37, DHX57, DHX8, DHX9, DICER1, DOCK7, DPH6, DRG1, DYNC1LI2, ECT2, EEF2, EFL1, EFTUD2, EGFR, EHD1, EHD2, EHD4, EIF4A3, EIF5B, EPHB3, EPS8L2, ERCC2, ERCC6L, ETFA, ETNK1, FARP1, FERMT2, FLAD1, FLT1, G3BP1, GALK1, GARS1, GART, GBF1, GCDH, GCLC, GDI1, GET3, GLUD1, GLUL, GMPPB, GMPS, GNE, GNG12, GNL2, GNL3, GOLT1B, GOT2, GPHN,	No change

					GPI, GPSM1, GSDME, GSN, GSS, GSTM2, GSTM3, GTPBP1, GUF1, HACL1, HADHA, HELLS, HERC2, HK1, HK2, HMGCL, HOOK3, HSP90AB1, HSP90B1, HSPA13, HSPA14, HSPA2, HSPA4, HSPA5, HSPA9, HSPD1, HSPE1, HSPH1, ILK, ILVBL, IQGAP1, IQGAP2, ITPK1, KDM1A, KIF11, KIF20A, KIF21A, KIF22, KIF23, KIF2C, KIF5B, LANCL2, LARS1, LARS2, LBR, LIG1, LIG3, LRRK1, LSG1, MAP2K6, MAPK1, MAPK14, MAPK8, MARS1, MASTL, MCCC2, MCM2, MCM3, MCM4, MCM5, MCM6, MCM7, MDN1, ME1, MGST1, MICAL1, MICAL3, MKI67, MLH1, MMAB, MME, MSH2, MSH3, MSH6, MTHFD1, MTREX, MVD, MVK, MYH10, MYH9, MYO1C, MYO1E, MYO5A, MYO9B, NCAPG, NDRG1, NEK7, NLGN4X, NOL9, NQO2, NSF, NUCB2, NUDT16, NVL, OGT, OPLAH, ORC4, ORC5, P3H1, P3H3, P4HA1, P4HA2, P4HTM, PAICS, PAK1, PAPSS2, PARD3, PASK, PC, PCCA, PCCB, PDGFRB, PDXDC1, PDXK, PFAS, PFKL, PFKM, PFKP, PI4KA, PICALM, PIK3C2A, PIK3CA, PIK3R4, PIP4K2B, PIP4K2C, PKM, PLEKHA5, PLK1, PLOD1, PLOD2, PLOD3, PNP, POR, PRKAA1, PRKACB, PRKACG, PRKAG1, PRKAR1A, PRKAR2A, PRKAR2B, PRKCA, PRKDC, PRPF38B, PSMC6, PTK7, PXK, QDPR, QRSL1, RAB14, RAB18, RAB1A, RAB23, RAB2A, RAB32, RAB5B, RAB5C, RAB8A, RABL6, RAC3, RAD51, RAD54L2, RALGPS2, RAN, RAP2A, RAP2B, RAP2C, RCC2, RELA, RFC1, RFC2, RFC5, RHEB, RIC1, RIC8A, RIMKLB, RIT1, ROCK2, RP2, RPS6KA1, RPS6KA3, RPS6KA4, RRAS, RTCA, RTKN, SAMHD1, SAR1A, SARS1, SARS2, SBDS, SBF1, SCYL1, SEPHS1, SEPTIN10, SEPTIN11, SEPTIN2, SEPTIN7, SEPTIN8, SESN2, SESTD1, SHPK, SIRT1, SIRT2, SLC1A3, SLK,	

					SMARCA4, SMARCA5, SMARCAD1, SMC2, SNRNP200, SNX3, SORD, SPTLC2, SRC, SRPK1, SRPRA, SRPRB, SRR, STK38, TARS2, TCP1, TELO2, TGM2, TKFC, TKT, TLK1, TOP2A, TRAP1, TRIP13, TSR1, TTF2, TTK, TTLL12, TUBA4A, TUBB6, TUT1, TWF1, TXNRD1, UBA6, UBE2G1, UBE2H, UBE2O, VRK1, WARS1, XRCC5, XRCC6, YARS2, YTHDC2, ZC3HAV1, ZFYVE16]	
nucleotide binding	GO_MolecularFunction-EBI-UniProt-GOA-ACAP-ARAP_13.05.2021_00h00	6.26E-51	15.66121	392	[ABCB10, ABCB7, ABCC1, ABCF1, ACACA, ACAD8, ACAD9, ACAT1, ACBD3, ACLY, ACOX3, ACSL1, ACSL4, ACSS2, ACSS3, ACTBL2, ACTR1B, ACTR2, AK1, AK4, AKT1, ALDH2, ANTXR1, ANXA6, APAF1, APRT, ARF4, ARF6, ARFGEF1, ARHGAP28, ARHGAP5, ARHGDIA, ARHGEF1, ARHGEF11, ARHGEF12, ARL1, ARL2, ARL3, ASNS, ATL2, ATL3, ATP1A1, ATP2B1, ATP2B2, ATP2B4, ATP6V1A, ATR, AURKA, AURKB, BAG1, BAG2, BAG3, BAZ1B, BDH2, BMS1, BUB1B, CAMK1, CAMK2D, CASK, CAT, CCT2, CCT3, CCT4, CCT5, CCT6A, CCT7, CCT8, CDC42, CDC42BPA, CDC42BPB, CDK7, CHD1, CHD4, CHEK1, CHEK2, CHTF18, CLASP1, CMPK1, CTBP2, CTPS1, CYB5R1, CYB5R3, DAPK1, DARS2, DCAF1, DDX10, DDX18, DDX20, DDX21, DDX24, DDX28, DDX31, DDX39B, DDX41, DDX42, DDX46, DDX47, DDX49, DDX5, DDX52, DDX54, DDX6, DECR1, DGKA, DHX15, DHX30, DHX33, DHX37, DHX57, DHX8, DHX9, DICER1, DNMT1, DOCK7, DPH6, DRG1, DYNC1LI2, ECT2, EEF2, EFL1, EFTUD2, EGFR, EHD1, EHD2, EHD4, EIF4A3, EIF5B, EPHB3, EPS8L2, ERCC2, ERCC6L, ETFA, ETNK1, EXOSC10, FAM114A2, FARP1, FLAD1, FLT1, G3BP1, GALK1, GARS1, GART, GBF1, GCDH, GCLC, GDI1, GET3, GLUD1, GLUL, GMPPB, GMPS, GNE, GNL2, GNL3, GPD1L, GPHN,	No change

					GPI, GPSM1, GSS, GTPBP1, GUF1, H6PD, HACL1, HADHA, HELLS, HERC2, HIBADH, HK1, HK2, HMGCL, HOOK3, HSP90AB1, HSP90B1, HSPA13, HSPA14, HSPA2, HSPA4, HSPA5, HSPA9, HSPD1, HSPE1, HSPH1, IDH2, ILK, ILVBL, IMPDH1, IMPDH2, ITPK1, KDM1A, KIF11, KIF20A, KIF21A, KIF22, KIF23, KIF2C, KIF5B, LANCL2, LARS1, LARS2, LBR, LIG1, LIG3, LRRK1, LRWD1, LSG1, MAP2K6, MAPK1, MAPK14, MAPK8, MARS1, MASTL, MBD3, MCCC2, MCM2, MCM3, MCM4, MCM5, MCM6, MCM7, MDN1, ME1, MICAL1, MICAL3, MKI67, MLH1, MMAB, MOCS2, MSH2, MSH3, MSH6, MTHFD1, MTREX, MVD, MVK, MYH10, MYH9, MYO1C, MYO1E, MYO5A, MYO9B, NDRG1, NEK7, NNT, NOL9, NQO2, NSF, NT5C2, NUCB2, NUDT16, NVL, OPLAH, ORC4, ORC5, PAICS, PAK1, PAPSS2, PARP1, PASK, PC, PCCA, PCCB, PDGFRB, PDXK, PFAS, PFKL, PFKM, PFKP, PI4KA, PIK3C2A, PIK3CA, PIK3R4, PIP4K2B, PIP4K2C, PKM, PLK1, POLA1, POLD1, POLE, POR, PRKAA1, PRKACB, PRKACG, PRKAG1, PRKAR1A, PRKAR2A, PRKAR2B, PRKCA, PRKDC, PRPF38B, PSMC6, PTK7, PXK, QDPR, QRSL1, RAB14, RAB18, RAB1A, RAB23, RAB2A, RAB32, RAB5B, RAB5C, RAB8A, RABL6, RAC3, RAD51, RAD54L2, RALGPS2, RAN, RAP2A, RAP2B, RAP2C, RCC2, RFC1, RFC2, RFC5, RHEB, RIC1, RIC8A, RIMKLB, RIT1, ROCK2, RP2, RPS6KA1, RPS6KA3, RPS6KA4, RRAS, RTCA, RTKN, SAMHD1, SAR1A, SARS1, SARS2, SBF1, SCYL1, SEPHS1, SEPTIN10, SEPTIN11, SEPTIN2, SEPTIN7, SEPTIN8, SESN2, SHPK, SIL1, SIRT1, SIRT2, SLK, SMARCA4, SMARCA5, SMARCAD1, SMC2, SNRNP200, SORD, SPR, SRC, SRPK1, SRPRA, SRPRB, SRR, STK38, SUCLG1, TARS2, TCP1, TELO2, TGM2, TKFC,	

					TLK1, TOP2A, TRAP1, TRIP13, TSR1, TTF2, TTK, TTLL12, TUBA4A, TUBB6, TUT1, TWF1, TXNRD1, UBA6, UBE2G1, UBE2H, UBE2O, UGDH, UGP2, VRK1, WARS1, XRCC5, XRCC6, YARS2, YTHDC2, ZC3HAV1]	
ribonucleotide binding	GO_MolecularFunction-EBI-UniProt-GOA-ACAP-ARAP_13.05.2021_00h00	6.26E-51	15.44281	347	[ABCB10, ABCB7, ABCC1, ABCF1, ACACA, ACAD9, ACAT1, ACBD3, ACLY, ACSL1, ACSL4, ACSS2, ACSS3, ACTBL2, ACTR1B, ACTR2, AK1, AK4, AKT1, ANTXR1, ANXA6, APAF1, APRT, ARF4, ARF6, ARFGEF1, ARHGAP28, ARHGAP5, ARHGDIA, ARHGEF1, ARHGEF11, ARHGEF12, ARL1, ARL2, ARL3, ASNS, ATL2, ATL3, ATP1A1, ATP2B1, ATP2B2, ATP2B4, ATP6V1A, ATR, AURKA, AURKB, BAZ1B, BMS1, BUB1B, CAMK1, CAMK2D, CASK, CCT2, CCT3, CCT4, CCT5, CCT6A, CCT7, CCT8, CDC42, CDC42BPA, CDC42BPB, CDK7, CHD1, CHD4, CHEK1, CHEK2, CHTF18, CLASP1, CMPK1, CTPS1, CYB5R3, DAPK1, DARS2, DCAF1, DDX10, DDX18, DDX20, DDX21, DDX24, DDX28, DDX31, DDX39B, DDX41, DDX42, DDX46, DDX47, DDX49, DDX5, DDX52, DDX54, DDX6, DGKA, DHX15, DHX30, DHX33, DHX37, DHX57, DHX8, DHX9, DICER1, DOCK7, DPH6, DRG1, DYNC1LI2, ECT2, EEF2, EFL1, EFTUD2, EGFR, EHD1, EHD2, EHD4, EIF4A3, EIF5B, EPHB3, EPS8L2, ERCC2, ERCC6L, ETFA, ETNK1, FARP1, FLAD1, FLT1, G3BP1, GALK1, GARS1, GART, GBF1, GCDH, GCLC, GDI1, GET3, GLUD1, GLUL, GMPPB, GMPS, GNE, GNL2, GNL3, GPHN, GPI, GPSM1, GSS, GTPBP1, GUF1, HACL1, HADHA, HELLS, HERC2, HK1, HK2, HMGCL, HOOK3, HSP90AB1, HSP90B1, HSPA13, HSPA14, HSPA2, HSPA4, HSPA5, HSPA9, HSPD1, HSPE1, HSPH1, ILK, ITPK1, KIF11, KIF20A, KIF21A, KIF22, KIF23, KIF2C, KIF5B, LANCL2, LARS1, LARS2, LIG1, LIG3, LRRK1, LSG1,	No change

					MAP2K6, MAPK1, MAPK14, MAPK8, MARS1, MASTL, MCCC2, MCM2, MCM3, MCM4, MCM5, MCM6, MCM7, MDN1, ME1, MKI67, MLH1, MMAB, MSH2, MSH3, MSH6, MTHFD1, MTREX, MVD, MVK, MYH10, MYH9, MYO1C, MYO1E, MYO5A, MYO9B, NDRG1, NEK7, NOL9, NSF, NUCB2, NUDT16, NVL, OPLAH, ORC4, ORC5, PAICS, PAK1, PAPSS2, PASK, PC, PCCA, PCCB, PDGFRB, PDXK, PFAS, PFKL, PFKM, PFKP, PI4KA, PIK3C2A, PIK3CA, PIK3R4, PIP4K2B, PIP4K2C, PKM, PLK1, POR, PRKAA1, PRKACB, PRKACG, PRKAG1, PRKAR1A, PRKAR2A, PRKAR2B, PRKCA, PRKDC, PRPF38B, PSMC6, PTK7, PXK, QRSL1, RAB14, RAB18, RAB1A, RAB23, RAB2A, RAB32, RAB5B, RAB5C, RAB8A, RABL6, RAC3, RAD51, RAD54L2, RALGPS2, RAN, RAP2A, RAP2B, RAP2C, RCC2, RFC1, RFC2, RFC5, RHEB, RIC1, RIC8A, RIMKLB, RIT1, ROCK2, RP2, RPS6KA1, RPS6KA3, RPS6KA4, RRAS, RTCA, RTKN, SAMHD1, SAR1A, SARS1, SARS2, SBF1, SCYL1, SEPHS1, SEPTIN10, SEPTIN11, SEPTIN2, SEPTIN7, SEPTIN8, SESN2, SHPK, SLK, SMARCA4, SMARCA5, SMARCAD1, SMC2, SNRNP200, SRC, SRPK1, SRPRA, SRPRB, SRR, STK38, TARS2, TCP1, TELO2, TGM2, TKFC, TLK1, TOP2A, TRAP1, TRIP13, TSR1, TTF2, TTK, TTLL12, TUBA4A, TUBB6, TUT1, TWF1, UBA6, UBE2G1, UBE2H, UBE2O, UGP2, VRK1, WARS1, XRCC5, XRCC6, YARS2, YTHDC2]	
purine ribonucleoside triphosphate binding	GO_MolecularFunction-EBI-UniProt-GOA-ACAP-ARAP_13.05.2021_00h00	6.26E-51	15.40616	330	[ABCB10, ABCB7, ABCC1, ABCF1, ACACA, ACLY, ACSL1, ACSL4, ACSS2, ACSS3, ACTBL2, ACTR1B, ACTR2, AK1, AK4, AKT1, ANTXR1, ANXA6, APAF1, ARF4, ARF6, ARFGEF1, ARHGAP28, ARHGAP5, ARHGEF1, ARHGEF11, ARHGEF12, ARL1, ARL2, ARL3, ASNS, ATL2, ATL3, ATP1A1, ATP2B1,	No change

					ATP2B2, ATP2B4, ATP6V1A, ATR, AURKA, AURKB, BAZ1B, BMS1, BUB1B, CAMK1, CAMK2D, CASK, CCT2, CCT3, CCT4, CCT5, CCT6A, CCT7, CCT8, CDC42, CDC42BPA, CDC42BPB, CDK7, CHD1, CHD4, CHEK1, CHEK2, CHTF18, CLASP1, CMPK1, CTPS1, DAPK1, DARS2, DCAF1, DDX10, DDX18, DDX20, DDX21, DDX24, DDX28, DDX31, DDX39B, DDX41, DDX42, DDX46, DDX47, DDX49, DDX5, DDX52, DDX54, DDX6, DGKA, DHX15, DHX30, DHX33, DHX37, DHX57, DHX8, DHX9, DICER1, DOCK7, DPH6, DRG1, DYNC1LI2, ECT2, EEF2, EFL1, EFTUD2, EGFR, EHD1, EHD2, EHD4, EIF4A3, EIF5B, EPHB3, EPS8L2, ERCC2, ERCC6L, ETFA, ETNK1, FARP1, FLAD1, FLT1, G3BP1, GALK1, GARS1, GART, GBF1, GCLC, GET3, GLUD1, GLUL, GMPPB, GMPS, GNE, GNL2, GNL3, GPHN, GPI, GPSM1, GSS, GTPBP1, GUF1, HACL1, HELLS, HERC2, HK1, HK2, HOOK3, HSP90AB1, HSP90B1, HSPA13, HSPA14, HSPA2, HSPA4, HSPA5, HSPA9, HSPD1, HSPE1, HSPH1, ILK, ITPK1, KIF11, KIF20A, KIF21A, KIF22, KIF23, KIF2C, KIF5B, LANCL2, LARS1, LARS2, LIG1, LIG3, LRRK1, LSG1, MAP2K6, MAPK1, MAPK14, MAPK8, MARS1, MASTL, MCCC2, MCM2, MCM3, MCM4, MCM5, MCM6, MCM7, MDN1, MKI67, MLH1, MMAB, MSH2, MSH3, MSH6, MTHFD1, MTREX, MVD, MVK, MYH10, MYH9, MYO1C, MYO1E, MYO5A, MYO9B, NDRG1, NEK7, NOL9, NSF, NUCB2, NUDT16, NVL, OPLAH, ORC4, ORC5, PAICS, PAK1, PAPSS2, PASK, PC, PCCA, PCCB, PDGFRB, PDXK, PFAS, PFKL, PFKM, PFKP, PI4KA, PIK3C2A, PIK3CA, PIK3R4, PIP4K2B, PIP4K2C, PKM, PLK1, PRKAA1, PRKACB, PRKACG, PRKAG1, PRKCA, PRKDC, PRPF38B, PSMC6, PTK7, PXK, QRSL1, RAB14,	

					RAB18, RAB1A, RAB23, RAB2A, RAB32, RAB5B, RAB5C, RAB8A, RABL6, RAC3, RAD51, RAD54L2, RALGPS2, RAN, RAP2A, RAP2B, RAP2C, RCC2, RFC1, RFC2, RFC5, RHEB, RIC1, RIC8A, RIMKLB, RIT1, ROCK2, RP2, RPS6KA1, RPS6KA3, RPS6KA4, RRAS, RTCA, RTKN, SAMHD1, SAR1A, SARS1, SARS2, SBF1, SCYL1, SEPHS1, SEPTIN10, SEPTIN11, SEPTIN2, SEPTIN7, SEPTIN8, SHPK, SLK, SMARCA4, SMARCA5, SMARCAD1, SMC2, SNRNP200, SRC, SRPK1, SRPRA, SRPRB, SRR, STK38, TARS2, TCP1, TELO2, TGM2, TKFC, TLK1, TOP2A, TRAP1, TRIP13, TSR1, TTF2, TTK, TTLL12, TUBA4A, TUBB6, TUT1, TWF1, UBA6, UBE2G1, UBE2H, UBE2O, VRK1, WARS1, XRCC5, XRCC6, YARS2, YTHDC2]	
purine nucleotide binding	GO_MolecularFunction-EBI-UniProt-GOA-ACAP-ARAP_13.05.2021_00h00	6.26E-51	15.66265	351	[ABCB10, ABCB7, ABCC1, ABCF1, ACACA, ACAD9, ACAT1, ACBD3, ACLY, ACSL1, ACSL4, ACSS2, ACSS3, ACTBL2, ACTR1B, ACTR2, AK1, AK4, AKT1, ANTXR1, ANXA6, APAF1, APRT, ARF4, ARF6, ARFGEF1, ARHGAP28, ARHGAP5, ARHGDIA, ARHGEF1, ARHGEF11, ARHGEF12, ARL1, ARL2, ARL3, ASNS, ATL2, ATL3, ATP1A1, ATP2B1, ATP2B2, ATP2B4, ATP6V1A, ATR, AURKA, AURKB, BAG1, BAG2, BAG3, BAZ1B, BMS1, BUB1B, CAMK1, CAMK2D, CASK, CCT2, CCT3, CCT4, CCT5, CCT6A, CCT7, CCT8, CDC42, CDC42BPA, CDC42BPB, CDK7, CHD1, CHD4, CHEK1, CHEK2, CHTF18, CLASP1, CMPK1, CTPS1, CYB5R3, DAPK1, DARS2, DCAF1, DDX10, DDX18, DDX20, DDX21, DDX24, DDX28, DDX31, DDX39B, DDX41, DDX42, DDX46, DDX47, DDX49, DDX5, DDX52, DDX54, DDX6, DGKA, DHX15, DHX30, DHX33, DHX37, DHX57, DHX8, DHX9, DICER1, DOCK7, DPH6, DRG1, DYNC1LI2, ECT2, EEF2, EFL1, EFTUD2, EGFR, EHD1, EHD2, EHD4, EIF4A3,	No change

					EIF5B, EPHB3, EPS8L2, ERCC2, ERCC6L, ETFA, ETNK1, FAM114A2, FARP1, FLAD1, FLT1, G3BP1, GALK1, GARS1, GART, GBF1, GCDH, GCLC, GDI1, GET3, GLUD1, GLUL, GMPPB, GMPS, GNE, GNL2, GNL3, GPHN, GPI, GPSM1, GSS, GTPBP1, GUF1, HACL1, HADHA, HELLS, HERC2, HK1, HK2, HMGCL, HOOK3, HSP90AB1, HSP90B1, HSPA13, HSPA14, HSPA2, HSPA4, HSPA5, HSPA9, HSPD1, HSPE1, HSPH1, ILK, ITPK1, KIF11, KIF20A, KIF21A, KIF22, KIF23, KIF2C, KIF5B, LANCL2, LARS1, LARS2, LIG1, LIG3, LRRK1, LSG1, MAP2K6, MAPK1, MAPK14, MAPK8, MARS1, MASTL, MCCC2, MCM2, MCM3, MCM4, MCM5, MCM6, MCM7, MDN1, ME1, MKI67, MLH1, MMAB, MSH2, MSH3, MSH6, MTHFD1, MTREX, MVD, MVK, MYH10, MYH9, MYO1C, MYO1E, MYO5A, MYO9B, NDRG1, NEK7, NOL9, NSF, NUCB2, NUDT16, NVL, OPLAH, ORC4, ORC5, PAICS, PAK1, PAPSS2, PASK, PC, PCCA, PCCB, PDGFRB, PDXK, PFAS, PFKL, PFKM, PFKP, PI4KA, PIK3C2A, PIK3CA, PIK3R4, PIP4K2B, PIP4K2C, PKM, PLK1, POLA1, PRKAA1, PRKACB, PRKACG, PRKAG1, PRKAR1A, PRKAR2A, PRKAR2B, PRKCA, PRKDC, PRPF38B, PSMC6, PTK7, PXK, QRSL1, RAB14, RAB18, RAB1A, RAB23, RAB2A, RAB32, RAB5B, RAB5C, RAB8A, RABL6, RAC3, RAD51, RAD54L2, RALGPS2, RAN, RAP2A, RAP2B, RAP2C, RCC2, RFC1, RFC2, RFC5, RHEB, RIC1, RIC8A, RIMKLB, RIT1, ROCK2, RP2, RPS6KA1, RPS6KA3, RPS6KA4, RRAS, RTCA, RTKN, SAMHD1, SAR1A, SARS1, SARS2, SBF1, SCYL1, SEPHS1, SEPTIN10, SEPTIN11, SEPTIN2, SEPTIN7, SEPTIN8, SESN2, SHPK, SIL1, SLK, SMARCA4, SMARCA5, SMARCAD1, SMC2, SNRNP200, SRC, SRPK1, SRPRA, SRPRB,	

					SRR, STK38, TARS2, TCP1, TELO2, TGM2, TKFC, TLK1, TOP2A, TRAP1, TRIP13, TSR1, TTF2, TTK, TTLL12, TUBA4A, TUBB6, TUT1, TWF1, UBA6, UBE2G1, UBE2H, UBE2O, VRK1, WARS1, XRCC5, XRCC6, YARS2, YTHDC2]	
purine ribonucleotide binding	GO_MolecularFunction-EBI-UniProt-GOA-ACAP-ARAP_13.05.2021_00h00	6.26E-51	15.49865	345	[ABCB10, ABCB7, ABCC1, ABCF1, ACACA, ACAD9, ACAT1, ACBD3, ACLY, ACSL1, ACSL4, ACSS2, ACSS3, ACTBL2, ACTR1B, ACTR2, AK1, AK4, AKT1, ANTXR1, ANXA6, APAF1, APRT, ARF4, ARF6, ARFGEF1, ARHGAP28, ARHGAP5, ARHGDIA, ARHGEF1, ARHGEF11, ARHGEF12, ARL1, ARL2, ARL3, ASNS, ATL2, ATL3, ATP1A1, ATP2B1, ATP2B2, ATP2B4, ATP6V1A, ATR, AURKA, AURKB, BAZ1B, BMS1, BUB1B, CAMK1, CAMK2D, CASK, CCT2, CCT3, CCT4, CCT5, CCT6A, CCT7, CCT8, CDC42, CDC42BPA, CDC42BPB, CDK7, CHD1, CHD4, CHEK1, CHEK2, CHTF18, CLASP1, CMPK1, CTPS1, CYB5R3, DAPK1, DARS2, DCAF1, DDX10, DDX18, DDX20, DDX21, DDX24, DDX28, DDX31, DDX39B, DDX41, DDX42, DDX46, DDX47, DDX49, DDX5, DDX52, DDX54, DDX6, DGKA, DHX15, DHX30, DHX33, DHX37, DHX57, DHX8, DHX9, DICER1, DOCK7, DPH6, DRG1, DYNC1LI2, ECT2, EEF2, EFL1, EFTUD2, EGFR, EHD1, EHD2, EHD4, EIF4A3, EIF5B, EPHB3, EPS8L2, ERCC2, ERCC6L, ETFA, ETNK1, FARP1, FLAD1, FLT1, G3BP1, GALK1, GARS1, GART, GBF1, GCDH, GCLC, GDI1, GET3, GLUD1, GLUL, GMPPB, GMPS, GNE, GNL2, GNL3, GPHN, GPI, GPSM1, GSS, GTPBP1, GUF1, HACL1, HADHA, HELLS, HERC2, HK1, HK2, HMGCL, HOOK3, HSP90AB1, HSP90B1, HSPA13, HSPA14, HSPA2, HSPA4, HSPA5, HSPA9, HSPD1, HSPE1, HSPH1, ILK, ITPK1, KIF11, KIF20A, KIF21A, KIF22, KIF23, KIF2C, KIF5B, LANCL2, LARS1, LARS2, LIG1, LIG3, LRRK1, LSG1,	No change

					MAP2K6, MAPK1, MAPK14, MAPK8, MARS1, MASTL, MCCC2, MCM2, MCM3, MCM4, MCM5, MCM6, MCM7, MDN1, ME1, MKI67, MLH1, MMAB, MSH2, MSH3, MSH6, MTHFD1, MTREX, MVD, MVK, MYH10, MYH9, MYO1C, MYO1E, MYO5A, MYO9B, NDRG1, NEK7, NOL9, NSF, NUCB2, NUDT16, NVL, OPLAH, ORC4, ORC5, PAICS, PAK1, PAPSS2, PASK, PC, PCCA, PCCB, PDGFRB, PDXK, PFAS, PFKL, PFKM, PFKP, PI4KA, PIK3C2A, PIK3CA, PIK3R4, PIP4K2B, PIP4K2C, PKM, PLK1, PRKAA1, PRKACB, PRKACG, PRKAG1, PRKAR1A, PRKAR2A, PRKAR2B, PRKCA, PRKDC, PRPF38B, PSMC6, PTK7, PXK, QRSL1, RAB14, RAB18, RAB1A, RAB23, RAB2A, RAB32, RAB5B, RAB5C, RAB8A, RABL6, RAC3, RAD51, RAD54L2, RALGPS2, RAN, RAP2A, RAP2B, RAP2C, RCC2, RFC1, RFC2, RFC5, RHEB, RIC1, RIC8A, RIMKLB, RIT1, ROCK2, RP2, RPS6KA1, RPS6KA3, RPS6KA4, RRAS, RTCA, RTKN, SAMHD1, SAR1A, SARS1, SARS2, SBF1, SCYL1, SEPHS1, SEPTIN10, SEPTIN11, SEPTIN2, SEPTIN7, SEPTIN8, SESN2, SHPK, SLK, SMARCA4, SMARCA5, SMARCAD1, SMC2, SNRNP200, SRC, SRPK1, SRPRA, SRPRB, SRR, STK38, TARS2, TCP1, TELO2, TGM2, TKFC, TLK1, TOP2A, TRAP1, TRIP13, TSR1, TTF2, TTK, TTLL12, TUBA4A, TUBB6, TUT1, TWF1, UBA6, UBE2G1, UBE2H, UBE2O, VRK1, WARS1, XRCC5, XRCC6, YARS2, YTHDC2]	
adenyl nucleotide binding	GO_MolecularFunction-EBI-UniProt-GOA-ACAP-ARAP_13.05.2021_00h00	6.26E-51	16.55426	270	[ABCB10, ABCB7, ABCC1, ABCF1, ACACA, ACAD9, ACAT1, ACBD3, ACLY, ACSL1, ACSL4, ACSS2, ACSS3, ACTBL2, ACTR1B, ACTR2, AK1, AK4, AKT1, ANTXR1, APAF1, APRT, ASNS, ATP1A1, ATP2B1, ATP2B2, ATP2B4, ATP6V1A, ATR, AURKA, AURKB, BAG1, BAG2, BAG3, BAZ1B, BMS1,	Upregulation

					BUB1B, CAMK1, CAMK2D, CASK, CCT2, CCT3, CCT4, CCT5, CCT6A, CCT7, CCT8, CDC42BPA, CDC42BPB, CDK7, CHD1, CHD4, CHEK1, CHEK2, CHTF18, CLASP1, CMPK1, CTPS1, CYB5R3, DAPK1, DARS2, DCAF1, DDX10, DDX18, DDX20, DDX21, DDX24, DDX28, DDX31, DDX39B, DDX41, DDX42, DDX46, DDX47, DDX49, DDX5, DDX52, DDX54, DDX6, DGKA, DHX15, DHX30, DHX33, DHX37, DHX57, DHX8, DHX9, DICER1, DPH6, DYNC1LI2, EGFR, EHD1, EHD2, EHD4, EIF4A3, EPHB3, ERCC2, ERCC6L, ETNK1, FLAD1, FLT1, G3BP1, GALK1, GARS1, GART, GCDH, GCLC, GET3, GLUD1, GLUL, GMPS, GNE, GPHN, GPI, GSS, HACL1, HADHA, HELLS, HK1, HK2, HMGCL, HOOK3, HSP90AB1, HSP90B1, HSPA13, HSPA14, HSPA2, HSPA4, HSPA5, HSPA9, HSPD1, HSPE1, HSPH1, ILK, ITPK1, KIF11, KIF20A, KIF21A, KIF22, KIF23, KIF2C, KIF5B, LANCL2, LARS1, LARS2, LIG1, LIG3, LRRK1, MAP2K6, MAPK1, MAPK14, MAPK8, MARS1, MASTL, MCCC2, MCM2, MCM3, MCM4, MCM5, MCM6, MCM7, MDN1, ME1, MKI67, MLH1, MMAB, MSH2, MSH3, MSH6, MTHFD1, MTREX, MVD, MVK, MYH10, MYH9, MYO1C, MYO1E, MYO5A, MYO9B, NEK7, NOL9, NSF, NVL, OPLAH, ORC4, ORC5, PAICS, PAK1, PAPSS2, PASK, PC, PCCA, PCCB, PDGFRB, PDXK, PFAS, PFKL, PFKM, PFKP, PI4KA, PIK3C2A, PIK3CA, PIK3R4, PIP4K2B, PIP4K2C, PKM, PLK1, PRKAA1, PRKACB, PRKACG, PRKAG1, PRKAR1A, PRKAR2A, PRKAR2B, PRKCA, PRKDC, PSMC6, PTK7, PXK, QRSL1, RAD51, RAD54L2, RFC1, RFC2, RFC5, RIMKLB, ROCK2, RPS6KA1, RPS6KA3, RPS6KA4, RTCA, SARS1, SARS2, SCYL1, SEPHS1, SHPK, SIL1, SLK, SMARCA4, SMARCA5, SMARCAD1, SMC2,	

					SNRNP200, SRC, SRPK1, SRR, STK38, TARS2, TCP1, TELO2, TKFC, TLK1, TOP2A, TRAP1, TRIP13, TTF2, TTK, TTLL12, TUT1, TWF1, UBA6, UBE2G1, UBE2H, UBE2O, VRK1, WARS1, XRCC5, XRCC6, YARS2, YTHDC2]	
adenyl ribonucleotide binding	GO_MolecularFunction-EBI-UniProt-GOA-ACAP-ARAP_13.05.2021_00h00	6.26E-51	16.44005	266	[ABCB10, ABCB7, ABCC1, ABCF1, ACACA, ACAD9, ACAT1, ACBD3, ACLY, ACSL1, ACSL4, ACSS2, ACSS3, ACTBL2, ACTR1B, ACTR2, AK1, AK4, AKT1, ANTXR1, APAF1, APRT, ASNS, ATP1A1, ATP2B1, ATP2B2, ATP2B4, ATP6V1A, ATR, AURKA, AURKB, BAZ1B, BMS1, BUB1B, CAMK1, CAMK2D, CASK, CCT2, CCT3, CCT4, CCT5, CCT6A, CCT7, CCT8, CDC42BPA, CDC42BPB, CDK7, CHD1, CHD4, CHEK1, CHEK2, CHTF18, CLASP1, CMPK1, CTPS1, CYB5R3, DAPK1, DARS2, DCAF1, DDX10, DDX18, DDX20, DDX21, DDX24, DDX28, DDX31, DDX39B, DDX41, DDX42, DDX46, DDX47, DDX49, DDX5, DDX52, DDX54, DDX6, DGKA, DHX15, DHX30, DHX33, DHX37, DHX57, DHX8, DHX9, DICER1, DPH6, DYNC1LI2, EGFR, EHD1, EHD2, EHD4, EIF4A3, EPHB3, ERCC2, ERCC6L, ETNK1, FLAD1, FLT1, G3BP1, GALK1, GARS1, GART, GCDH, GCLC, GET3, GLUD1, GLUL, GMPS, GNE, GPHN, GPI, GSS, HACL1, HADHA, HELLS, HK1, HK2, HMGCL, HOOK3, HSP90AB1, HSP90B1, HSPA13, HSPA14, HSPA2, HSPA4, HSPA5, HSPA9, HSPD1, HSPE1, HSPH1, ILK, ITPK1, KIF11, KIF20A, KIF21A, KIF22, KIF23, KIF2C, KIF5B, LANCL2, LARS1, LARS2, LIG1, LIG3, LRRK1, MAP2K6, MAPK1, MAPK14, MAPK8, MARS1, MASTL, MCCC2, MCM2, MCM3, MCM4, MCM5, MCM6, MCM7, MDN1, ME1, MKI67, MLH1, MMAB, MSH2, MSH3, MSH6, MTHFD1, MTREX, MVD, MVK, MYH10, MYH9, MYO1C, MYO1E, MYO5A, MYO9B, NEK7, NOL9, NSF, NVL, OPLAH,	Upregulation

					ORC4, ORC5, PAICS, PAK1, PAPSS2, PASK, PC, PCCA, PCCB, PDGFRB, PDXK, PFAS, PFKL, PFKM, PFKP, PI4KA, PIK3C2A, PIK3CA, PIK3R4, PIP4K2B, PIP4K2C, PKM, PLK1, PRKAA1, PRKACB, PRKACG, PRKAG1, PRKAR1A, PRKAR2A, PRKAR2B, PRKCA, PRKDC, PSMC6, PTK7, PXK, QRSL1, RAD51, RAD54L2, RFC1, RFC2, RFC5, RIMKLB, ROCK2, RPS6KA1, RPS6KA3, RPS6KA4, RTCA, SARS1, SARS2, SCYL1, SEPHS1, SHPK, SLK, SMARCA4, SMARCA5, SMARCAD1, SMC2, SNRNP200, SRC, SRPK1, SRR, STK38, TARS2, TCP1, TELO2, TKFC, TLK1, TOP2A, TRAP1, TRIP13, TTF2, TTK, TTLL12, TUT1, TWF1, UBA6, UBE2G1, UBE2H, UBE2O, VRK1, WARS1, XRCC5, XRCC6, YARS2, YTHDC2]	
ATP binding	GO_MolecularFunction-EBI-UniProt-GOA-ACAP-ARAP_13.05.2021_00h00	6.26E-51	16.36598	254	[ABCB10, ABCB7, ABCC1, ABCF1, ACACA, ACLY, ACSL1, ACSL4, ACSS2, ACSS3, ACTBL2, ACTR1B, ACTR2, AK1, AK4, AKT1, ANTXR1, APAF1, ASNS, ATP1A1, ATP2B1, ATP2B2, ATP2B4, ATP6V1A, ATR, AURKA, AURKB, BAZ1B, BMS1, BUB1B, CAMK1, CAMK2D, CASK, CCT2, CCT3, CCT4, CCT5, CCT6A, CCT7, CCT8, CDC42BPA, CDC42BPB, CDK7, CHD1, CHD4, CHEK1, CHEK2, CHTF18, CLASP1, CMPK1, CTPS1, DAPK1, DARS2, DCAF1, DDX10, DDX18, DDX20, DDX21, DDX24, DDX28, DDX31, DDX39B, DDX41, DDX42, DDX46, DDX47, DDX49, DDX5, DDX52, DDX54, DDX6, DGKA, DHX15, DHX30, DHX33, DHX37, DHX57, DHX8, DHX9, DICER1, DPH6, DYNC1LI2, EGFR, EHD1, EHD2, EHD4, EIF4A3, EPHB3, ERCC2, ERCC6L, ETNK1, FLAD1, FLT1, G3BP1, GALK1, GARS1, GART, GCLC, GET3, GLUD1, GLUL, GMPS, GNE, GPHN, GPI, GSS, HACL1, HELLS, HK1, HK2, HOOK3, HSP90AB1, HSP90B1, HSPA13, HSPA14, HSPA2, HSPA4, HSPA5, HSPA9, HSPD1, HSPE1, HSPH1, ILK, ITPK1, KIF11, KIF20A, KIF21A,	Upregulation

					KIF22, KIF23, KIF2C, KIF5B, LANCL2, LARS1, LARS2, LIG1, LIG3, LRRK1, MAP2K6, MAPK1, MAPK14, MAPK8, MARS1, MASTL, MCCC2, MCM2, MCM3, MCM4, MCM5, MCM6, MCM7, MDN1, MKI67, MLH1, MMAB, MSH2, MSH3, MSH6, MTHFD1, MTREX, MVD, MVK, MYH10, MYH9, MYO1C, MYO1E, MYO5A, MYO9B, NEK7, NOL9, NSF, NVL, OPLAH, ORC4, ORC5, PAICS, PAK1, PAPSS2, PASK, PC, PCCA, PCCB, PDGFRB, PDXK, PFAS, PFKL, PFKM, PFKP, PI4KA, PIK3C2A, PIK3CA, PIK3R4, PIP4K2B, PIP4K2C, PKM, PLK1, PRKAA1, PRKACB, PRKACG, PRKAG1, PRKCA, PRKDC, PSMC6, PTK7, PXK, QRSL1, RAD51, RAD54L2, RFC1, RFC2, RFC5, RIMKLB, ROCK2, RPS6KA1, RPS6KA3, RPS6KA4, RTCA, SARS1, SARS2, SCYL1, SEPHS1, SHPK, SLK, SMARCA4, SMARCA5, SMARCAD1, SMC2, SNRNP200, SRC, SRPK1, SRR, STK38, TARS2, TCP1, TELO2, TKFC, TLK1, TOP2A, TRAP1, TRIP13, TTF2, TTK, TTLL12, TUT1, TWF1, UBA6, UBE2G1, UBE2H, UBE2O, VRK1, WARS1, XRCC5, XRCC6, YARS2, YTHDC2]	
cellular component organization	GO_BiologicalProcess-EBI-UniProt-GOA-ACAP-ARAP_13.05.2021_00h00	8.27E-44	10.57455	727	[AAAS, AASS, ABCB7, ABCC1, ABI1, ACACA, ACAD9, ACIN1, ACOT13, ACSL4, ACTBL2, ACTN1, ACTR2, ADAM10, ADARB1, ADD2, ADD3, AEBP2, AGO1, AGO2, AGTPBP1, AIMP2, AKT1, ALCAM, ANK2, ANK3, ANKFY1, ANKRD28, ANO6, ANP32E, ANTXR1, ANXA1, ANXA2, ANXA6, AP1M1, AP2A1, AP2A2, AP2B1, AP2M1, AP2S1, AP3B1, APEH, APOE, APOOL, APPL2, ARAP1, ARAP3, ARF4, ARF6, ARFGAP1, ARFGAP3, ARFGEF1, ARHGAP28, ARHGDIA, ARHGEF11, ARL1, ARL2, ARL3, ARPC1A, ARSB, ARVCF, ASAP1, ATF7IP, ATL2, ATL3, ATP6V1A, ATR, AUP1, AURKA, AURKB, B4GALT1, BAG3, BAIAP2, BAZ1B, BCCIP, BET1, BIN1, BOP1,	No change

					BPTF, BRAT1, BRD3, BRD4, BRMS1, BRWD1, BUB1B, C1QBP, CALCOCO2, CALD1, CALR, CAMK1, CAMK2D, CAP1, CAP2, CAPN1, CAPN2, CAPNS1, CAPRIN1, CAPZA2, CASP3, CAST, CAV1, CBL, CBX2, CCAR2, CCNB1, CCNH, CCT2, CCT3, CCT4, CCT5, CCT6A, CCT7, CCT8, CD2AP, CD44, CDC123, CDC42, CDC42BPA, CDC42BPB, CDC42EP1, CDC42EP3, CDC73, CDCA8, CDH2, CDH3, CDK5RAP3, CDK7, CELF1, CFAP20, CFAP298, CFL2, CHAF1A, CHD1, CHD4, CHEK1, CHEK2, CHTF18, CIRBP, CKAP5, CLASP1, CLUH, CNN1, CNN3, COG1, COG7, COL1A1, COL1A2, COL4A1, COL4A2, COL5A1, COL5A2, COL6A1, COL6A2, COL6A3, COLGALT1, COPS2, COPS5, COPS7A, CORO1C, CRLF3, CRMP1, CRTAP, CSDE1, CSPG4, CSRP2, CTBP2, CTCF, CTNNB1, CTSC, CUL4A, CUL4B, CUL7, CXADR, CYFIP1, DAXX, DBNL, DCAF1, DCTN1, DDRGK1, DDX20, DDX28, DDX39B, DDX49, DDX6, DEGS1, DHX30, DHX33, DHX37, DHX8, DHX9, DIAPH1, DICER1, DLG1, DLGAP5, DNMT1, DNMT3A, DNMT3B, DOCK7, DPYSL2, DPYSL3, DRG1, DSP, DSTN, DUSP3, DYNC1LI2, ECPAS, ECT2, EEA1, EFL1, EFNB2, EGFL6, EGFR, EHD1, EHD2, EHD4, EHMT1, EIF3A, EMD, EML1, EMSY, ENO2, EPB41, EPB41L3, EPB41L5, EPHB3, EPN2, EPS8, EPS8L2, ERBIN, ERCC2, ERCC6L, ERO1A, ESYT2, EXOG, EXOSC10, EXOSC2, F11R, FAM118B, FANCD2, FARP1, FASTKD2, FAT1, FBN2, FBXW8, FERMT2, FGG, FHL1, FKBP10, FKBP4, FLNB, FLNC, FN1, FNBP1L, FOXRED1, FSCN1, FSD1, FXR1, G3BP1, G3BP2, GAB1, GARS1, GBF1, GCLC, GCLM, GDAP1, GDI1, GEMIN4, GEMIN5, GET3, GET4, GLMN, GLS, GNL3, GOLGA2, GOSR2, GPC1, GPC3,	

					GPC6, GPHN, GSN, GTF2E1, GTF3C4, HACL1, HAT1, HCFC1, HDAC2, HDAC4, HELLS, HK2, HMGCL, HMOX1, HOOK3, HP1BP3, HSD17B10, HSP90AB1, HSPA2, HSPA4, HSPA5, HSPA9, HSPB11, HSPD1, HTRA1, HTT, HUWE1, ILK, INCENP, INO80C, IPO4, IQGAP1, IQGAP2, IQGAP3, IST1, ITCH, ITGA5, ITGA6, ITGAV, ITGB1, IWS1, JADE1, JAM3, JARID2, KAT7, KCTD12, KDM1A, KDM2A, KDM3B, KIF11, KIF20A, KIF22, KIF23, KIF2C, KIF5B, KLC1, KNTC1, KRT18, KRT19, KRT8, LAMB1, LAMC1, LARS1, LBR, LGALS1, LIG3, LIMS1, LMAN1, LNPK, LRP1, LRRC8A, LRWD1, LSM4, LYPLA2, MACF1, MACROH2A1, MAIP1, MAP1B, MAP1LC3A, MAP1S, MAP4, MAPK1, MAPK14, MAPK8, MARCHF5, MAVS, MBD3, MCM2, MCM3, MCM4, MCM5, MCM6, MCM7, MCMBP, MCU, MDN1, MESD, METAP1, MFGE8, MICAL1, MICAL3, MICOS13, MIPEP, MKI67, MLH1, MMAB, MPRIP, MRPS27, MRPS9, MSH2, MSH3, MSH6, MSN, MTA3, MTDH, MTR, MVB12A, MYADM, MYD88, MYH10, MYH9, MYO1C, MYO1E, MYO5A, NCAM1, NCAPD2, NCAPG, NCAPG2, NCKAP1, NDC1, NDC80, NDRG1, NECTIN2, NEDD4, NEK7, NIBAN2, NID1, NLE1, NLGN4X, NOL6, NPM1, NPM3, NRP2, NSF, NSFL1C, NUP107, NUP133, NUP160, NUP210, NUP35, NUSAP1, OCLN, OGT, OPTN, ORC2, ORC3, ORC4, ORC5, OSBP, OTUD6B, P3H1, P3H4, P4HA1, P4HA2, P4HB, PAK1, PALLD, PARD3, PARD6B, PARN, PARP1, PARVA, PAWR, PAXBP1, PDCL3, PDGFRB, PDLIM2, PDLIM4, PDLIM5, PDLIM7, PDS5A, PDZRN3, PHC1, PHIP, PHPT1, PI4KA, PICALM, PIK3C2A, PIK3CA, PIP4K2B, PIP4K2C, PITRM1, PKP2, PLCG1, PLEC, PLEKHA7, PLK1,	

					PLOD1, PLOD2, PLOD3, PLS3, PLSCR3, PLXNB2, PNPT1, POGZ, POLA1, POLA2, POLD1, POLR1B, PPFIBP1, PPIB, PPID, PPM1F, PPP1R7, PPP1R9B, PRDX4, PRIM1, PRIM2, PRKAA1, PRKACB, PRKACG, PRKAR1A, PRKAR2B, PRKCA, PRKCSH, PRKDC, PRPF38B, PRPF40A, PRPF8, PRUNE1, PSIP1, PSMC6, PSMD1, PSMD2, PSMD9, PSME3, PSMG3, PTCD3, PTK7, PTPN1, PTPRD, PURA, PWP2, PXDN, PXMP2, PYCARD, RAB14, RAB18, RAB1A, RAB23, RAB2A, RAB32, RAB5B, RAB5C, RAB8A, RAC3, RAD51, RAD54L2, RAN, RANBP2, RANGAP1, RAP1GDS1, RAP2A, RAP2B, RAP2C, RBBP5, RCC2, RDX, RELA, RFC1, RFC2, RFC3, RFC5, RFTN1, RIC1, RIC8A, RIF1, RIOX1, ROCK2, RP2, RPA1, RPA2, RPA3, RPF2, RPL13A, RPL5, RPS6KA1, RPS6KA3, RPS6KA4, RRP7A, RRS1, RTKN, RTTN, S100A10, SAMHD1, SAR1A, SARM1, SART3, SBDS, SCARB2, SCFD1, SDAD1, SEC13, SEC16A, SEC22B, SEC23A, SEC23B, SEC24A, SEC24B, SEC24C, SEC24D, SEC31A, SEC61G, SEH1L, SEPTIN11, SEPTIN2, SEPTIN7, SEPTIN8, SERBP1, SERPINH1, SESN2, SET, SETD7, SETDB1, SF3A3, SF3B1, SH3GL3, SH3GLB1, SIN3A, SIRT1, SIRT2, SKA3, SLAIN2, SLC12A4, SLC25A5, SLC2A1, SLC2A10, SLC9A3R2, SLIRP, SLK, SMARCA4, SMARCA5, SMARCAD1, SMARCC1, SMARCD1, SMARCD2, SMC2, SNRNP200, SNRPD3, SNX17, SNX3, SNX9, SORBS1, SORBS3, SORD, SPAG9, SPARC, SPECC1L, SPTAN1, SPTBN1, SPTBN2, SRC, SRPK1, SRSF1, SSBP1, SSRP1, STAM2, STX12, STX5, SUN2, SUPT16H, SUPT6H, SURF4, TACC3, TBC1D17, TBC1D22B, TBC1D4, TBCEL, TBPL1, TCP1, TELO2, TENM3, TF, TFB1M, TFG, THBS1, TIGAR, TIMM50, TIMMDC1, TJP2, TLK1, TLN1, TLN2, TM9SF4,	

					TMED10, TMED2, TMED5, TMED7, TMED9, TMEM43, TMEM97, TMF1, TOMM34, TOP2A, TPM1, TPM4, TPX2, TRAM1, TRIM28, TRIP12, TRIP13, TTK, TTLL12, TUBA4A, TUBB6, TWF1, UBA6, UBR5, UFL1, UGDH, USE1, USP15, USP47, USP7, USP9X, UTRN, VAMP2, VAMP3, VAT1, VIM, VLDLR, VPS25, VPS35, VPS36, VRK1, WAPL, WARS1, WRAP53, XRCC5, XRCC6, XRN1, YAP1, YKT6, ZDHHC17, ZFP36L2, ZFYVE16, ZMYM3, ZMYND8, ZNF462, ZWILCH]	
cellular component biogenesis	GO_BiologicalProcess-EBI-UniProt-GOA-ACAP-ARAP_13.05.2021_00h00	8.27E-44	12.92135	460	[AAAS, AATF, ABCB7, ACACA, ACAD9, ACOT13, ACTN1, ACTR2, ADAM10, ADD2, ADD3, AGO1, AGO2, AIMP2, ANK2, ANK3, ANKRD28, ANO6, ANTXR1, ANXA2, AP2B1, AP2M1, AP2S1, APOE, APPL2, ARAP1, ARF4, ARF6, ARFGEF1, ARHGAP28, ARL2, ARL3, ARPC1A, ARVCF, ASAP1, ATF7IP, ATL2, ATL3, ATR, AUP1, AURKA, AURKB, BAG3, BAIAP2, BCCIP, BET1, BIN1, BMS1, BOP1, C1QBP, CALD1, CALR, CAPZA2, CAV1, CCNB1, CCNH, CCT3, CCT8, CD2AP, CDC123, CDC42, CDC42EP1, CDC42EP3, CDC73, CDH2, CDK7, CELF1, CFAP20, CFAP298, CFL2, CHAF1A, CHEK2, CIRBP, CKAP5, CLASP1, COL1A2, COPS5, COPS7A, CORO1C, CSDE1, CSPG4, CSRP2, CTCF, CTNNB1, CTSC, CUL4A, CUL4B, CYFIP1, DAXX, DBNL, DCAF13, DCTN1, DDX10, DDX18, DDX20, DDX21, DDX28, DDX31, DDX39B, DDX47, DDX49, DDX52, DDX54, DDX6, DHX30, DHX33, DHX37, DHX9, DIAPH1, DICER1, DLG1, DLGAP5, DNMT1, DNMT3A, DPYSL3, DRG1, DUSP3, ECPAS, ECT2, EFL1, EFNB2, EHD1, EHD2, EHD4, EIF3A, EIF4A3, EMD, EPB41, EPB41L3, EPB41L5, EPHB3, EPS8, EPS8L2, ERCC2, ESF1, EXOSC10, EXOSC2, EXOSC5, EXOSC7, F11R,	No change

					FARP1, FASTKD2, FERMT2, FGG, FKBP10, FKBP4, FLNC, FN1, FNBP1L, FOXRED1, FSCN1, FTSJ3, FXR1, G3BP1, G3BP2, GARS1, GBF1, GEMIN4, GEMIN5, GLS, GLUL, GNL2, GOLGA2, GOSR2, GPC1, GPC6, GSN, HAT1, HCFC1, HDAC4, HEATR1, HELLS, HP1BP3, HSD17B10, HSP90AB1, HSPA2, HSPA4, HSPA5, HSPA9, HSPB11, HSPD1, HTT, IGF2BP3, ILK, IMP3, INCENP, IPO4, IQGAP1, IQGAP2, IST1, ITGA6, ITGB1, JAM3, KCTD12, KIF11, KIF23, KNTC1, KRT19, KRT8, LAMB1, LAMC1, LAS1L, LBR, LIMS1, LMAN1, LRP1, LRRC8A, LSG1, LSM4, LYAR, MACF1, MACROH2A1, MAP1B, MAP1LC3A, MAP4, MBD3, MCM2, MCM3, MCM4, MCM5, MCM6, MCM7, MCU, MDN1, MICAL1, MIPEP, MLH1, MMAB, MPHOSPH10, MSN, MTDH, MTREX, MVB12A, MYADM, MYBBP1A, MYD88, MYO1C, MYO1E, NCAPG, NCKAP1, NDC1, NDRG1, NECTIN2, NEDD4, NEK7, NLE1, NLGN4X, NOL10, NOL11, NOL6, NOL9, NOP14, NOP56, NOP58, NOP9, NPM1, NPM3, NSF, NSFL1C, NSUN5, NUDT16, NUP107, NUP133, NUP160, NUP210, NUP35, NVL, OCLN, ORC2, ORC3, ORC4, ORC5, OTUD6B, P3H4, P4HB, PAK1, PARD3, PARD6B, PARP1, PARVA, PAWR, PDCD11, PDGFRB, PDLIM5, PELP1, PES1, PHIP, PICALM, PIK3C2A, PIK3CA, PIP4K2B, PIP4K2C, PKP2, PLEC, PLEKHA7, PLK1, PLOD3, PLS3, PLXNB2, PNPT1, POGZ, POLR1B, PPID, PPM1F, PPP1R9B, PRKAA1, PRKACB, PRKACG, PRKAR1A, PRKAR2B, PRKCA, PRKCSH, PRKDC, PRPF8, PRUNE1, PSIP1, PSMC6, PSMD1, PSMD2, PSMD9, PSME3, PSMG3, PTPRD, PWP2, PXDN, PXMP2, PYCARD, RAB14, RAB1A, RAB23, RAB32, RAB8A, RAC3, RAD51, RAN, RANGAP1, RAP1GDS1, RAP2A,	

					RAP2B, RAP2C, RBBP5, RCC2, RDX, RFTN1, ROCK2, RP2, RPA1, RPA2, RPA3, RPF2, RPL13A, RPL22, RPL27A, RPL4, RPL5, RPL7A, RPS2, RPS21, RPS8, RRP12, RRP7A, RRS1, RTKN, RTTN, S100A10, SAMHD1, SAR1A, SART3, SBDS, SCFD1, SDAD1, SEC13, SEC16A, SEC22B, SEC23A, SEC24A, SEC24B, SEC24C, SEC24D, SEC31A, SEH1L, SEPTIN2, SEPTIN7, SEPTIN8, SET, SETDB1, SF3A3, SF3B1, SH3GLB1, SIRT1, SIRT2, SLAIN2, SLC2A1, SLC9A3R2, SLK, SMARCA4, SMARCA5, SNRNP200, SNRPD3, SNX9, SORBS1, SORBS3, SORD, SPTAN1, SPTBN1, SPTBN2, SRC, SRPK1, SRSF1, SSBP1, STAM2, STX12, STX5, TBC1D17, TBC1D22B, TBCEL, TBL3, TBPL1, TCP1, TEX10, TFB1M, TFG, THBS1, TIMMDC1, TLN1, TLN2, TM9SF4, TMED10, TMED2, TMF1, TPM1, TPX2, TRIM28, TRIP13, TSR1, TUBA4A, TWF1, UGDH, UTP15, UTP18, UTP20, UTP25, UTP4, VAMP2, VAMP3, VPS25, VPS35, VPS36, VRK1, WARS1, WDR18, WDR3, WDR36, WDR43, WRAP53, XRCC5, XRN2, YAP1, ZFP36L2, ZMYND8]	
regulation of cellular component biogenesis	GO_BiologicalProcess-EBI-UniProt-GOA-ACAP-ARAP_13.05.2021_00h00	8.27E-44	12.38185	131	[ABCB7, ACTR2, ADD2, ADD3, ANTXR1, APOE, ARAP1, ARF6, ARFGEF1, ARHGAP28, ARL2, ARL3, ARPC1A, ASAP1, ATF7IP, ATR, BAIAP2, BIN1, CAPZA2, CAV1, CDC42, CDC42EP1, CDC42EP3, CKAP5, CLASP1, CORO1C, CUL4A, CUL4B, CYFIP1, DBNL, DCTN1, DHX33, DLG1, DNMT1, DPYSL3, DRG1, DUSP3, ECT2, EPB41L5, EPHB3, EPS8, EPS8L2, ERCC2, F11R, FARP1, FERMT2, FKBP4, FNBP1L, FSCN1, FXR1, G3BP1, G3BP2, GPC6, GSN, HCFC1, HDAC4, HSPA2, HSPA5, HTT, IQGAP1, IQGAP2, JAM3, LIMS1, LRP1, MACF1, MACROH2A1, MAP1B, MAP4, MMAB, MSN, MYADM, MYD88, MYO1C, MYO1E, NCAPG, NCKAP1,	Downregulation

					NDRG1, NPM1, NUDT16, OCLN, PAK1, PARP1, PDLIM5, PIK3CA, PIP4K2B, PIP4K2C, PLK1, POLR1B, PPM1F, PRKAA1, PRKCA, PRKCSH, PRUNE1, PSMC6, PTPRD, PXDN, PYCARD, RCC2, RDX, ROCK2, RPA1, RPA2, RPA3, RPL13A, S100A10, SAR1A, SCFD1, SEC22B, SEPTIN7, SEPTIN8, SETDB1, SH3GLB1, SLAIN2, SLK, SNX9, SORBS3, SPTAN1, SPTBN1, SPTBN2, SRC, TBC1D17, TBC1D22B, THBS1, TPM1, TRIM28, TWF1, VPS35, WARS1, XRCC5, YAP1, ZMYND8]	
organelle organization	GO_BiologicalProcess-EBI-UniProt-GOA-ACAP-ARAP_13.05.2021_00h00	8.27E-44	12.40672	532	[AAAS, AASS, ABI1, ACAD9, ACIN1, ACTBL2, ACTN1, ACTR2, ADD2, ADD3, AEBP2, AGTPBP1, AKT1, ANK2, ANK3, ANKFY1, ANKRD28, ANP32E, ANTXR1, ANXA1, ANXA2, AP1M1, AP2M1, AP3B1, APOE, APOOL, ARAP1, ARAP3, ARF4, ARF6, ARFGAP3, ARFGEF1, ARHGAP28, ARHGDIA, ARHGEF11, ARL1, ARL2, ARL3, ARPC1A, ARSB, ASAP1, ATF7IP, ATL2, ATL3, ATP6V1A, ATR, AUP1, AURKA, AURKB, BAG3, BAIAP2, BAZ1B, BCCIP, BET1, BIN1, BOP1, BPTF, BRD3, BRD4, BRMS1, BRWD1, BUB1B, C1QBP, CALCOCO2, CALD1, CALR, CAMK1, CAMK2D, CAP1, CAP2, CAPN2, CAPZA2, CAV1, CBX2, CCAR2, CCNB1, CCT2, CCT3, CCT4, CCT5, CCT6A, CCT7, CCT8, CD2AP, CDC42, CDC42BPA, CDC42BPB, CDC42EP1, CDC42EP3, CDC73, CDCA8, CDH2, CFAP20, CFAP298, CFL2, CHAF1A, CHD1, CHD4, CHEK1, CHEK2, CHTF18, CIRBP, CKAP5, CLASP1, CLUH, CNN1, CNN3, COG1, COG7, COPS2, COPS5, COPS7A, CORO1C, CRMP1, CSDE1, CSRP2, CTCF, CTNNB1, CTSC, CUL4A, CUL4B, CUL7, CXADR, CYFIP1, DAXX, DBNL, DCAF1, DCTN1, DDRGK1, DDX28, DDX6, DHX30, DHX37, DHX9, DIAPH1, DLG1, DLGAP5, DNMT1, DNMT3A, DNMT3B, DOCK7,	No change

					DPYSL2, DPYSL3, DRG1,<br>DSP, DSTN, DYNC1LI2,<br>ECT2, EEA1, EFL1,<br>EHD1, EHD2, EHD4,<br>EHMT1, EMD, EML1,<br>EMSY, ENO2, EPB41,<br>EPB41L3, EPB41L5,<br>EPS8, ERBIN, ERCC2,<br>ERCC6L, ESYT2,<br>EXOSC10, F11R,<br>FAM118B, FANCD2,<br>FARP1, FASTKD2, FAT1,<br>FBXW8, FERMT2,<br>FKBP4, FLNB, FNBP1L,<br>FOXRED1, FSCN1, FSD1,<br>G3BP1, G3BP2, GAB1,<br>GBF1, GCLC, GCLM,<br>GDAP1, GET3, GET4,<br>GNL3, GOLGA2, GOSR2,<br>GSN, GTF3C4, HACL1,<br>HAT1, HCFC1, HDAC2,<br>HDAC4, HELLS, HK2,<br>HMGCL, HMOX1,<br>HOOK3, HP1BP3,<br>HSD17B10, HSP90AB1,<br>HSPA2, HSPA4, HSPA9,<br>HSPB11, HSPD1, HTT,<br>HUWE1, ILK, INCENP,<br>INO80C, IPO4, IQGAP1,<br>IQGAP2, IQGAP3, IST1,<br>ITGB1, IWS1, JADE1,<br>JAM3, JARID2, KAT7,<br>KDM1A, KDM2A,<br>KDM3B, KIF11, KIF20A,<br>KIF22, KIF23, KIF2C,<br>KIF5B, KLC1, KNTC1,<br>KRT18, KRT19, KRT8,<br>LBR, LIG3, LMAN1,<br>LNPK, LRP1, LRWD1,<br>LSM4, MACF1,<br>MACROH2A1, MAIP1,<br>MAP1B, MAP1LC3A,<br>MAP1S, MAP4, MAPK1,<br>MAPK8, MARCHF5,<br>MAVS, MBD3, MCM2,<br>MCM3, MCM4, MCM5,<br>MCM6, MCM7,<br>MCMBP, MCU, MDN1,<br>METAP1, MICAL1,<br>MICAL3, MICOS13,<br>MIPEP, MKI67, MLH1,<br>MMAB, MPRIP, MSH2,<br>MSH3, MSH6, MSN,<br>MTA3, MVB12A,<br>MYADM, MYH10,<br>MYH9, MYO1C, MYO1E,<br>MYO5A, NCAPD2,<br>NCAPG, NCAPG2,<br>NCKAP1, NDC1, NDC80,<br>NDRG1, NECTIN2,<br>NEK7, NLE1, NOL6,<br>NPM1, NPM3, NSF,<br>NSFL1C, NUP107,<br>NUP133, NUP160,<br>NUP35, NUSAP1, OCLN,<br>OGT, OPTN, OSBP,<br>P3H4, PAK1, PALLD,<br>PARD3, PARD6B, PARN,<br>PARP1, PARVA, PAWR,<br>PAXBP1, PDCL3,<br>PDGFRB, PDLIM2,<br>PDLIM4, PDLIM5,<br>PDLIM7, PDS5A, PHC1,<br>PHIP, PHPT1, PICALM,<br>PIK3CA, PIP4K2B,	

					PIP4K2C, PITRM1, PKP2, PLEC, PLK1, PLS3, PLSCR3, PNPT1, POGZ, POLA1, POLA2, POLD1, POLR1B, PPID, PPM1F, PPP1R9B, PRIM1, PRIM2, PRKAA1, PRKAR1A, PRKAR2B, PRKCA, PRKDC, PRPF40A, PRUNE1, PTK7, PTPN1, PTPRD, PURA, PWP2, PXDN, PXMP2, PYCARD, RAB14, RAB18, RAB1A, RAB23, RAB2A, RAB32, RAB8A, RAC3, RAD51, RAD54L2, RAN, RANBP2, RANGAP1, RAP1GDS1, RAP2A, RBBP5, RCC2, RDX, RELA, RFC1, RFC2, RFC3, RFC5, RIF1, RIOX1, ROCK2, RP2, RPA1, RPA2, RPA3, RPF2, RPL5, RPS6KA4, RRP7A, RRS1, RTKN, RTTN, S100A10, SAR1A, SART3, SBDS, SCARB2, SCFD1, SDAD1, SEC13, SEC16A, SEC22B, SEC23A, SEC23B, SEC24A, SEC24B, SEC24C, SEC24D, SEC31A, SEC61G, SEH1L, SEPTIN2, SEPTIN7, SEPTIN8, SERBP1, SESN2, SET, SETD7, SETDB1, SH3GLB1, SIN3A, SIRT1, SIRT2, SKA3, SLAIN2, SLC25A5, SLIRP, SLK, SMARCA4, SMARCA5, SMARCAD1, SMARCC1, SMARCD1, SMARCD2, SMC2, SNX3, SNX9, SORBS1, SORBS3, SPECC1L, SPTAN1, SPTBN1, SPTBN2, SRC, SRPK1, SSBP1, SSRP1, STAM2, STX12, STX5, SUN2, SUPT16H, SUPT6H, SURF4, TACC3, TBC1D17, TBC1D22B, TBC1D4, TBCEL, TBPL1, TCP1, TELO2, TF, TFB1M, TFG, TIGAR, TIMM50, TIMMDC1, TLK1, TLN1, TLN2, TMED10, TMED2, TMED5, TMED7, TMED9, TMEM43, TMF1, TOMM34, TOP2A, TPM1, TPM4, TPX2, TRAM1, TRIM28, TRIP12, TRIP13, TTK, TTLL12, TUBA4A, TUBB6, TWF1, UBR5, UFL1, USP15, USP7, VAMP2, VAMP3, VAT1, VIM, VPS25, VPS35, VPS36, VRK1, WAPL, WRAP53, XRCC5, XRCC6, XRN1, YAP1, ZFYVE16, ZMYM3,	

					ZMYND8, ZNF462, ZWILCH]	
cellular component assembly	GO_BiologicalProcess-EBI-UniProt-GOA-ACAP-ARAP_13.05.2021_00h00	8.27E-44	11.94394	392	[AAAS, ABCB7, ACACA, ACAD9, ACOT13, ACTN1, ACTR2, ADAM10, ADD2, ADD3, AGO1, AGO2, AIMP2, ANK2, ANK3, ANKRD28, ANO6, ANTXR1, ANXA2, AP2B1, AP2M1, AP2S1, APOE, APPL2, ARAP1, ARF4, ARF6, ARFGEF1, ARHGAP28, ARL2, ARL3, ARPC1A, ARVCF, ASAP1, ATF7IP, ATL2, ATL3, ATR, AUP1, AURKA, AURKB, BAG3, BAIAP2, BCCIP, BET1, BIN1, BOP1, C1QBP, CALD1, CALR, CAPZA2, CAV1, CCNB1, CCNH, CCT3, CCT8, CD2AP, CDC123, CDC42, CDC42EP1, CDC42EP3, CDC73, CDH2, CDK7, CELF1, CFAP20, CFAP298, CFL2, CHAF1A, CHEK2, CIRBP, CKAP5, CLASP1, COL1A2, COPS7A, CORO1C, CSDE1, CSPG4, CSRP2, CTCF, CTNNB1, CTSC, CUL4A, CUL4B, CYFIP1, DAXX, DBNL, DCTN1, DDX20, DDX28, DDX39B, DDX6, DHX30, DHX33, DHX37, DHX9, DIAPH1, DICER1, DLG1, DLGAP5, DNMT1, DNMT3A, DPYSL3, DRG1, DUSP3, ECPAS, ECT2, EFL1, EFNB2, EHD1, EHD2, EHD4, EIF3A, EMD, EPB41, EPB41L3, EPB41L5, EPHB3, EPS8, EPS8L2, ERCC2, F11R, FARP1, FASTKD2, FERMT2, FGG, FKBP10, FKBP4, FLNC, FN1, FNBP1L, FOXRED1, FSCN1, FXR1, G3BP1, G3BP2, GARS1, GBF1, GEMIN4, GEMIN5, GLS, GOLGA2, GOSR2, GPC1, GPC6, GSN, HAT1, HCFC1, HDAC4, HELLS, HP1BP3, HSD17B10, HSP90AB1, HSPA2, HSPA4, HSPA5, HSPA9, HSPB11, HSPD1, HTT, ILK, INCENP, IPO4, IQGAP1, IQGAP2, IST1, ITGA6, ITGB1, JAM3, KCTD12, KIF11, KIF23, KNTC1, KRT19, KRT8, LAMB1, LAMC1, LBR, LIMS1, LMAN1, LRP1, LRRC8A, LSM4, MACF1, MACROH2A1, MAP1B, MAP1LC3A, MAP4, MBD3, MCM2, MCM3, MCM4, MCM5, MCM6, MCM7, MCU, MDN1, MICAL1, MIPEP, MLH1, MMAB, MSN, MTDH, MVB12A, MYADM,	No change

					MYD88, MYO1C, MYO1E, NCAPG, NCKAP1, NDC1, NDRG1, NECTIN2, NEK7, NLE1, NLGN4X, NOL6, NPM1, NSF, NSFL1C, NUP107, NUP133, NUP160, NUP210, NUP35, OCLN, ORC2, ORC3, ORC4, ORC5, OTUD6B, P3H4, P4HB, PAK1, PARD3, PARD6B, PARP1, PARVA, PAWR, PDGFRB, PDLIM5, PHIP, PICALM, PIK3C2A, PIK3CA, PIP4K2B, PIP4K2C, PKP2, PLEC, PLEKHA7, PLK1, PLOD3, PLS3, PLXNB2, PNPT1, POGZ, POLR1B, PPID, PPM1F, PPP1R9B, PRKAA1, PRKACB, PRKACG, PRKAR1A, PRKAR2B, PRKCA, PRKCSH, PRKDC, PRPF8, PRUNE1, PSIP1, PSMC6, PSMD1, PSMD2, PSMD9, PSME3, PSMG3, PTPRD, PWP2, PXDN, PXMP2, PYCARD, RAB14, RAB1A, RAB23, RAB32, RAB8A, RAC3, RAD51, RAN, RANGAP1, RAP1GDS1, RAP2A, RAP2B, RAP2C, RBBP5, RCC2, RDX, RFTN1, ROCK2, RP2, RPA1, RPA2, RPA3, RPF2, RPL13A, RPL5, RRP7A, RRS1, RTKN, RTTN, S100A10, SAMHD1, SAR1A, SART3, SBDS, SCFD1, SEC13, SEC16A, SEC22B, SEC23A, SEC24A, SEC24B, SEC24C, SEC24D, SEC31A, SEH1L, SEPTIN2, SEPTIN7, SEPTIN8, SET, SETDB1, SF3A3, SF3B1, SH3GLB1, SIRT1, SIRT2, SLAIN2, SLC2A1, SLC9A3R2, SLK, SMARCA4, SMARCA5, SNRNP200, SNRPD3, SNX9, SORBS1, SORBS3, SORD, SPTAN1, SPTBN1, SPTBN2, SRC, SRPK1, SRSF1, SSBP1, STAM2, STX12, STX5, TBC1D17, TBC1D22B, TBCEL, TBPL1, TCP1, TFG, THBS1, TIMMDC1, TLN1, TLN2, TM9SF4, TMED10, TMED2, TMF1, TPM1, TPX2, TRIM28, TRIP13, TUBA4A, TWF1, UGDH, VAMP2, VAMP3, VPS25, VPS35, VPS36, VRK1, WARS1, WRAP53, XRCC5, YAP1, ZFP36L2, ZMYND8]	

protein-containing complex subunit organization	GO_BiologicalProcess-EBI-UniProt-GOA-ACAP-ARAP_13.05.2021_00h00	8.27E-44	12.6177	268	[ACACA, ACAD9, ACOT13, ACTR2, ADAM10, ADD2, ADD3, AGO1, AGO2, AIMP2, ANKRD28, ANO6, ANP32E, ANTXR1, AP2B1, AP2M1, AP2S1, APEH, APOE, APPL2, ARF6, ARFGEF1, ARHGAP28, ARL2, ARL3, ARPC1A, ASAP1, ATF7IP, ATL2, ATL3, ATR, BAIAP2, BET1, BIN1, BOP1, BPTF, CALCOCO2, CALR, CAPZA2, CAV1, CCNB1, CCNH, CCT3, CCT8, CD2AP, CDC123, CDC42, CDC42EP1, CDC42EP3, CDC73, CDK7, CELF1, CFL2, CHAF1A, CKAP5, CLASP1, COL1A2, COPS7A, CTCF, CTNNB1, CTSC, CUL4A, CUL4B, CYFIP1, DAXX, DBNL, DCTN1, DDX20, DDX28, DDX39B, DHX30, DHX33, DHX8, DHX9, DIAPH1, DICER1, DLG1, DLGAP5, DRG1, DSTN, ECPAS, ECT2, EHD1, EHD4, EIF3A, EPB41, EPS8, ERCC2, FASTKD2, FERMT2, FGG, FKBP4, FN1, FOXRED1, FSCN1, G3BP2, GARS1, GEMIN4, GEMIN5, GLS, GOLGA2, GOSR2, GSN, GTF2E1, HAT1, HCFC1, HELLS, HP1BP3, HSD17B10, HSP90AB1, HSPA4, HSPA5, HSPD1, IPO4, IQGAP2, IST1, JAM3, KCTD12, KIF2C, KIF5B, KLC1, KNTC1, LAMC1, LMAN1, LRRC8A, LSM4, MACROH2A1, MAP1B, MAP1LC3A, MAP1S, MCM2, MCM3, MCM4, MCM5, MCM6, MCM7, MCU, MDN1, METAP1, MICAL1, MICAL3, MIPEP, MMAB, MRPS27, MRPS9, MSN, MYADM, MYD88, MYO1C, MYO1E, NCAPG, NCKAP1, NDC1, NDRG1, NLE1, NPM1, NSF, NUP107, NUP133, NUP210, NUP35, OCLN, ORC2, ORC3, ORC4, ORC5, OTUD6B, P4HB, PAK1, PARD3, PARD6B, PARP1, PICALM, PIK3C2A, PIK3CA, PLEKHA7, PNPT1, POGZ, POLR1B, PPID, PPP1R9B, PRKACB, PRKACG, PRKCA, PRKDC, PRPF8, PRUNE1, PSIP1, PSMC6, PSMD1, PSMD2, PSMD9,	No change

					PSME3, PSMG3, PTCD3, PWP2, PXDN, PYCARD, RAB1A, RAD51, RAP1GDS1, RBBP5, RDX, RPA1, RPA2, RPA3, RPF2, RPL13A, RPL5, RRP7A, RRS1, SAMHD1, SAR1A, SART3, SCFD1, SEC13, SEC16A, SEC22B, SEC23A, SEC24A, SEC24B, SEC24C, SEC24D, SEC31A, SEH1L, SEPTIN8, SET, SF3A3, SF3B1, SH3GLB1, SLAIN2, SLC2A1, SLC9A3R2, SMARCA4, SMARCA5, SMARCC1, SMARCD1, SMARCD2, SNRNP200, SNRPD3, SNX9, SPTAN1, SPTBN1, SPTBN2, SRC, SRPK1, SRSF1, SSBP1, STX5, SUPT16H, SUPT6H, TBCEL, TCP1, TFG, TIMMDC1, TLN1, TM9SF4, TMED10, TMED2, TPX2, TWF1, UGDH, VAMP2, VAMP3, WARS1, XRCC5, YAP1, ZFP36L2]	
regulation of protein-containing complex assembly	GO_BiologicalProcess-EBI-UniProt-GOA-ACAP-ARAP_13.05.2021_00h00	8.27E-44	15.17672	73	[ACTR2, ADD2, ADD3, ANTXR1, APOE, ARF6, ARFGEF1, ARHGAP28, ARL2, ARL3, ARPC1A, ASAP1, ATF7IP, ATR, BAIAP2, BIN1, CAPZA2, CDC42, CDC42EP1, CDC42EP3, CKAP5, CLASP1, CUL4A, CUL4B, CYFIP1, DBNL, DCTN1, DHX33, DLG1, DRG1, EPS8, ERCC2, FERMT2, FKBP4, FSCN1, GSN, HCFC1, HSPA5, IQGAP2, JAM3, MAP1B, MMAB, MSN, MYADM, MYD88, MYO1C, MYO1E, NCAPG, NCKAP1, NDRG1, OCLN, PAK1, PARP1, POLR1B, PRUNE1, PSMC6, PYCARD, RDX, RPA1, RPA2, RPA3, RPL13A, SAR1A, SEPTIN8, SH3GLB1, SLAIN2, SNX9, SPTAN1, SPTBN1, SPTBN2, SRC, TWF1, WARS1]	Downregulation
protein-containing complex assembly	GO_BiologicalProcess-EBI-UniProt-GOA-ACAP-ARAP_13.05.2021_00h00	8.27E-44	12.98844	236	[ACACA, ACAD9, ACOT13, ACTR2, ADAM10, ADD2, ADD3, AGO1, AGO2, AIMP2, ANKRD28, ANO6, ANTXR1, AP2B1, AP2M1, AP2S1, APOE, APPL2, ARF6, ARFGEF1, ARHGAP28, ARL2, ARL3, ARPC1A, ASAP1, ATF7IP, ATL2, ATL3, ATR, BAIAP2, BET1, BIN1, BOP1, CALR, CAPZA2, CCNB1, CCNH, CCT3, CCT8, CD2AP, CDC123, CDC42, CDC42EP1, CDC42EP3,	No change

					CDC73, CDK7, CELF1, CHAF1A, CKAP5, CLASP1, COL1A2, COPS7A, CTNNB1, CTSC, CUL4A, CUL4B, CYFIP1, DAXX, DBNL, DCTN1, DDX20, DDX28, DDX39B, DHX30, DHX33, DHX9, DIAPH1, DICER1, DLG1, DLGAP5, DRG1, ECPAS, ECT2, EHD1, EHD4, EIF3A, EPB41, EPS8, ERCC2, FASTKD2, FERMT2, FGG, FKBP4, FN1, FOXRED1, FSCN1, G3BP2, GARS1, GEMIN4, GEMIN5, GLS, GOLGA2, GOSR2, GSN, HAT1, HCFC1, HELLS, HP1BP3, HSD17B10, HSP90AB1, HSPA4, HSPA5, HSPD1, IPO4, IQGAP2, JAM3, KCTD12, KNTC1, LAMC1, LMAN1, LRRC8A, LSM4, MACROH2A1, MAP1B, MCM2, MCM3, MCM4, MCM5, MCM6, MCM7, MCU, MDN1, MIPEP, MMAB, MSN, MYADM, MYD88, MYO1C, MYO1E, NCAPG, NCKAP1, NDC1, NDRG1, NLE1, NPM1, NSF, NUP107, NUP210, OCLN, ORC2, ORC3, ORC4, ORC5, OTUD6B, P4HB, PAK1, PARD3, PARD6B, PARP1, PICALM, PIK3C2A, PLEKHA7, PNPT1, POGZ, POLR1B, PPID, PRKACB, PRKACG, PRKCA, PRKDC, PRPF8, PRUNE1, PSIP1, PSMC6, PSMD1, PSMD2, PSMD9, PSME3, PSMG3, PWP2, PXDN, PYCARD, RAB1A, RAD51, RAP1GDS1, RBBP5, RDX, RPA1, RPA2, RPA3, RPF2, RPL13A, RPL5, RRP7A, RRS1, SAMHD1, SAR1A, SART3, SCFD1, SEC13, SEC16A, SEC22B, SEC23A, SEC24A, SEC24B, SEC24C, SEC24D, SEC31A, SEPTIN8, SET, SF3A3, SF3B1, SH3GLB1, SLAIN2, SLC2A1, SLC9A3R2, SMARCA4, SMARCA5, SNRNP200, SNRPD3, SNX9, SPTAN1, SPTBN1, SPTBN2, SRC, SRPK1, SRSF1, SSBP1, STX5, TBCEL, TCP1, TFG, TIMMDC1, TLN1, TM9SF4, TMED10, TMED2, TPX2, TWF1, UGDH, VAMP2, VAMP3, WARS1, XRCC5, YAP1, ZFP36L2]	

cellular protein-containing complex assembly	GO_BiologicalProcess-EBI-UniProt-GOA-ACAP-ARAP_13.05.2021_00h00	8.27E-44	13	156	[ACAD9, ACTR2, ADD2, ADD3, AGO1, AGO2, ANTXR1, AP2B1, AP2M1, AP2S1, ARF6, ARFGEF1, ARHGAP28, ARL2, ARL3, ARPC1A, ATF7IP, ATR, BAIAP2, BIN1, BOP1, CALR, CAPZA2, CCNH, CDC42EP1, CDC42EP3, CDC73, CDK7, CELF1, CHAF1A, CKAP5, CLASP1, COPS7A, CUL4A, CUL4B, CYFIP1, DAXX, DBNL, DCTN1, DDX20, DDX28, DDX39B, DHX30, DHX33, DHX9, DIAPH1, DICER1, DLG1, DLGAP5, DRG1, ECPAS, EIF3A, EPS8, ERCC2, FASTKD2, FGG, FKBP4, FOXRED1, GARS1, GEMIN4, GEMIN5, GOLGA2, GSN, HAT1, HELLS, HP1BP3, HSP90AB1, HSPA4, HSPD1, IPO4, IQGAP2, LSM4, MACROH2A1, MAP1B, MCM2, MCM3, MCM4, MCM5, MCM6, MCM7, MDN1, MMAB, MYADM, MYD88, MYO1C, MYO1E, NCAPG, NCKAP1, NDC1, NDRG1, NLE1, NPM1, NUP107, OCLN, ORC2, ORC3, ORC4, ORC5, OTUD6B, PAK1, PARP1, PICALM, PIK3C2A, POGZ, POLR1B, PRKDC, PRPF8, PRUNE1, PSIP1, PSMC6, PSMD1, PSMD2, PSMD9, PSME3, PSMG3, PWP2, PXDN, PYCARD, RAD51, RAP1GDS1, RDX, RPA1, RPA2, RPA3, RPF2, RPL13A, RPL5, RRP7A, RRS1, SART3, SEPTIN8, SET, SF3A3, SF3B1, SLAIN2, SMARCA4, SMARCA5, SNRNP200, SNRPD3, SNX9, SPTAN1, SPTBN1, SPTBN2, SRC, SRPK1, SRSF1, TBCEL, TCP1, TIMMDC1, TM9SF4, TPX2, TWF1, VAMP2, VAMP3, XRCC5, ZFP36L2]	Upregulation
regulation of protein polymerization	GO_BiologicalProcess-EBI-UniProt-GOA-ACAP-ARAP_13.05.2021_00h00	8.27E-44	17.2	43	[ACTR2, ADD2, ADD3, ARF6, ARFGEF1, ARHGAP28, ARL2, ARL3, ARPC1A, BAIAP2, BIN1, CAPZA2, CDC42EP1, CDC42EP3, CKAP5, CLASP1, CYFIP1, DBNL, DCTN1, DLG1, DRG1, EPS8, FKBP4, GSN, IQGAP2, MAP1B, MYADM, MYO1C, MYO1E, NCAPG, NCKAP1, NDRG1, OCLN, PAK1, PRUNE1, PYCARD, RDX, SLAIN2,	Downregulation

					SNX9, SPTAN1, SPTBN1, SPTBN2, TWF1]	
protein polymerization	GO_BiologicalProcess-EBI-UniProt-GOA-ACAP-ARAP_13.05.2021_00h00	8.27E-44	14.41718	47	[ACTR2, ADD2, ADD3, ARF6, ARFGEF1, ARHGAP28, ARL2, ARL3, ARPC1A, BAIAP2, BIN1, CAPZA2, CDC42EP1, CDC42EP3, CKAP5, CLASP1, CYFIP1, DBNL, DCTN1, DIAPH1, DLG1, DRG1, EPS8, FGG, FKBP4, GOLGA2, GSN, IQGAP2, MAP1B, MYADM, MYO1C, MYO1E, NCAPG, NCKAP1, NDRG1, OCLN, PAK1, PRUNE1, PYCARD, RDX, SLAIN2, SNX9, SPTAN1, SPTBN1, SPTBN2, TPX2, TWF1]	Downregulation
localization within membrane	GO_BiologicalProcess-EBI-UniProt-GOA-ACAP-ARAP_13.05.2021_00h00	2.06E-40	13.4901	109	[ADAM10, AKT1, ANK2, ANK3, ANXA2, AP2B1, AP2M1, AP3B1, APOE, ARF6, ARHGAP1, ARL3, ARL6IP5, ATP2B4, CAV1, CCDC22, CD81, CDH2, DLG1, EGFR, EHD1, EHD2, EHD4, EMD, EPB41L3, F11R, FERMT2, GDAP1, GDI1, GET3, GET4, GOPC, GPC1, GPC3, GPC6, GPHN, GSN, HMOX1, HSPA4, HSPA5, ITGB1, KIF5B, KRT18, LRP1, MACF1, MAIP1, MAPK8, MESD, MYADM, MYO1C, MYO1E, MYO5A, NDC1, NSF, OPTN, PACS1, PAK1, PARD3, PICALM, PKP2, PPP1R9B, PPP2R5A, PREPL, PXMP2, RAB14, RAB32, RAB8A, RAP2A, RDX, RFTN1, ROCK2, RPL13A, RPL22, RPL27A, RPL4, RPL5, RPL7A, RPS2, RPS21, RPS8, S100A10, SEC16A, SEC23A, SEC61G, SH3GLB1, SIL1, SLC5A3, SLC9A3R2, SNX17, SNX3, SNX4, SORBS1, SPTBN1, SRP14, SRPRA, SSR1, STX5, TCAF1, TM9SF4, TMED2, TRAM1, VAMP2, VAMP3, VPS26A, VPS26C, VPS35, VPS35L, VPS53, ZMYND8]	Downregulation
cellular macromolecule localization	GO_BiologicalProcess-EBI-UniProt-GOA-ACAP-ARAP_13.05.2021_00h00	2.06E-40	15.22419	292	[ADAM10, AKT1, ANK2, ANK3, ANTXR1, ANXA2, AP1M1, AP1S1, AP2A1, AP2A2, AP2B1, AP2M1, AP2S1, AP3B1, APOE, APPL2, ARCN1, ARF4, ARF6, ARFGAP3, ARL1, ARL2, ARL3, ARL6IP5, ATP2B4, ATR, AUP1, AURKA, AURKB, BAG3, BUB1B, CALR, CAMK1, CAV1, CCT2, CCT3, CCT4, CCT5, CCT6A, CCT7, CCT8, CD2AP,	Downregulation

					CD81, CDC42, CDH2, CDK5RAP3, COG7, COL1A1, COPA, COPB1, COPB2, COPG1, COPG2, COPZ1, CSE1L, CTCF, CTNNB1, DDRGK1, DHX9, DIAPH1, DLG1, ECT2, EGFR, EHD1, EHD2, EHD4, EMD, EPB41, EPB41L3, ERBIN, ERLEC1, EXOSC10, EXOSC2, F11R, FAF2, FERMT2, GBF1, GDAP1, GDI1, GET3, GET4, GIPC1, GLUL, GNL3, GOPC, GOSR2, GPC1, GPC3, GPC6, GPD1L, GPHN, GSN, HACL1, HERC2, HK1, HK2, HMOX1, HOOK3, HSP90AB1, HSP90B1, HSPA4, HSPA5, HSPA9, HSPB1, HSPD1, HUWE1, IPO11, IPO4, IPO5, IQGAP1, ITGB1, JAM3, KAT7, KDELR1, KDELR3, KIF5B, KRT18, LMAN1, LRP1, LRWD1, MACF1, MACROH2A1, MAGED1, MAIP1, MAN1A1, MAPK1, MAPK14, MAPK8, MARCHF5, MAVS, MEAK7, MESD, METAP1, MIPEP, MMAB, MSH2, MSN, MYADM, MYO1C, MYO1E, MYO5A, NDC1, NDC80, NEDD4, NPM1, NSF, NSFL1C, NUP107, NUP133, NUP35, NUP50, NVL, OCLN, OPTN, PACS1, PAK1, PARD3, PARP1, PHIP, PICALM, PIK3R4, PITRM1, PKP2, PLK1, POLR1B, PPM1F, PPP1R9B, PPP2R5A, PREPL, PRKAA1, PRKCA, PTPN1, PXMP2, RAB14, RAB18, RAB1A, RAB23, RAB32, RAB5B, RAB5C, RAB8A, RABGAP1L, RAN, RANBP2, RANGAP1, RAP2A, RCC2, RDX, RFTN1, RIC1, ROCK2, RPA1, RPA2, RPF2, RPL13A, RPL22, RPL27A, RPL4, RPL5, RPL7A, RPS2, RPS21, RPS8, RRS1, S100A10, SAR1A, SCARB2, SCFD1, SCFD2, SCYL1, SEC13, SEC16A, SEC23A, SEC23B, SEC24A, SEC24B, SEC24C, SEC24D, SEC31A, SEC61G, SEH1L, SEL1L, SEPTIN10, SEPTIN11, SEPTIN2, SEPTIN7, SEPTIN8, SESN2, SH3GLB1, SIL1, SIN3A, SLC5A3, SLC9A3R2, SNX17, SNX6, SNX9, SORBS1, SPTBN1, SRC,	

					SRP14, SRPRA, SRPRB, SSB, SSR1, STAM2, STX12, STX5, STXBP2, STYX, SUN2, SURF4, TBC1D17, TBC1D22B, TBC1D4, TCAF1, TCP1, TIA1, TIMM50, TLK1, TM9SF4, TMED10, TMED2, TMED5, TMED7, TMED9, TNPO3, TOLLIP, TOMM34, TRAM1, TRIM28, TTK, TWF1, UBR5, UTP25, VAMP2, VAMP3, VPS25, VPS26A, VPS26B, VPS26C, VPS35, VPS36, WAPL, WRAP53, XPO5, XPO7, XRCC5, YAP1, YOD1, ZFYVE16, ZMYND8, ZWILCH]	
endosomal transport	GO_BiologicalProcess-EBI-UniProt-GOA-ACAP-ARAP_13.05.2021_00h00	2.06E-40	17.57813	45	[ANKFY1, AP3B1, ARF6, ARHGAP1, ARL1, CCDC22, CORO1C, DCTN1, EEA1, EHD1, EHD2, EHD4, GBF1, GOSR2, HEATR5A, LMAN1, MVB12A, PICALM, PREPL, RAB14, RAB8A, RIC1, SNX17, SNX3, SNX4, SNX6, SNX9, STAM2, STX5, SURF4, TBC1D17, TMED9, UBE2O, USP7, VAMP3, VPS25, VPS26A, VPS26B, VPS26C, VPS35, VPS35L, VPS36, VPS53, YKT6, ZFYVE16]	Downregulation
cellular protein localization	GO_BiologicalProcess-EBI-UniProt-GOA-ACAP-ARAP_13.05.2021_00h00	2.06E-40	15.2231	290	[ADAM10, AKT1, ANK2, ANK3, ANTXR1, ANXA2, AP1M1, AP1S1, AP2A1, AP2A2, AP2B1, AP2M1, AP2S1, AP3B1, APOE, APPL2, ARCN1, ARF4, ARF6, ARFGAP3, ARL1, ARL2, ARL3, ARL6IP5, ATP2B4, ATR, AUP1, AURKA, AURKB, BAG3, BUB1B, CALR, CAMK1, CAV1, CCT2, CCT3, CCT4, CCT5, CCT6A, CCT7, CCT8, CD2AP, CD81, CDC42, CDH2, CDK5RAP3, COG7, COL1A1, COPA, COPB1, COPB2, COPG1, COPG2, COPZ1, CSE1L, CTCF, CTNNB1, DDRGK1, DHX9, DIAPH1, DLG1, ECT2, EGFR, EHD1, EHD2, EHD4, EMD, EPB41, EPB41L3, ERBIN, ERLEC1, F11R, FAF2, FERMT2, GBF1, GDAP1, GDI1, GET3, GET4, GIPC1, GLUL, GNL3, GOPC, GOSR2, GPC1, GPC3, GPC6, GPD1L, GPHN, GSN, HACL1, HERC2, HK1, HK2, HMOX1, HOOK3, HSP90AB1, HSP90B1, HSPA4, HSPA5, HSPA9, HSPB1, HSPD1, HUWE1,	Downregulation

					IPO11, IPO4, IPO5, IQGAP1, ITGB1, JAM3, KAT7, KDELR1, KDELR3, KIF5B, KRT18, LMAN1, LRP1, LRWD1, MACF1, MACROH2A1, MAGED1, MAIP1, MAN1A1, MAPK1, MAPK14, MAPK8, MARCHF5, MAVS, MEAK7, MESD, METAP1, MIPEP, MMAB, MSH2, MSN, MYADM, MYO1C, MYO1E, MYO5A, NDC1, NDC80, NEDD4, NPM1, NSF, NSFL1C, NUP107, NUP133, NUP35, NUP50, NVL, OCLN, OPTN, PACS1, PAK1, PARD3, PARP1, PHIP, PICALM, PIK3R4, PITRM1, PKP2, PLK1, POLR1B, PPM1F, PPP1R9B, PPP2R5A, PREPL, PRKAA1, PRKCA, PTPN1, PXMP2, RAB14, RAB18, RAB1A, RAB23, RAB32, RAB5B, RAB5C, RAB8A, RABGAP1L, RAN, RANBP2, RANGAP1, RAP2A, RCC2, RDX, RFTN1, RIC1, ROCK2, RPA1, RPA2, RPF2, RPL13A, RPL22, RPL27A, RPL4, RPL5, RPL7A, RPS2, RPS21, RPS8, RRS1, S100A10, SAR1A, SCARB2, SCFD1, SCFD2, SCYL1, SEC13, SEC16A, SEC23A, SEC23B, SEC24A, SEC24B, SEC24C, SEC24D, SEC31A, SEC61G, SEH1L, SEL1L, SEPTIN10, SEPTIN11, SEPTIN2, SEPTIN7, SEPTIN8, SESN2, SH3GLB1, SIL1, SIN3A, SLC5A3, SLC9A3R2, SNX17, SNX6, SNX9, SORBS1, SPTBN1, SRC, SRP14, SRPRA, SRPRB, SSB, SSR1, STAM2, STX12, STX5, STXBP2, STYX, SUN2, SURF4, TBC1D17, TBC1D22B, TBC1D4, TCAF1, TCP1, TIA1, TIMM50, TLK1, TM9SF4, TMED10, TMED2, TMED5, TMED7, TMED9, TNPO3, TOLLIP, TOMM34, TRAM1, TRIM28, TTK, TWF1, UBR5, UTP25, VAMP2, VAMP3, VPS25, VPS26A, VPS26B, VPS26C, VPS35, VPS36, WAPL, WRAP53, XPO5, XPO7, XRCC5, YAP1, YOD1, ZFYVE16, ZMYND8, ZWILCH]	
Golgi vesicle transport	GO_BiologicalProcess-	2.06E-40	22.91667	99	[ACBD3, ACTR10, ANK2, ANK3, ANKFY1,	Downregulation

	EBI-UniProt-GOA-ACAP-ARAP_13.05.2021_00h00				ANKRD28, AP1M1, AP1S1, AP2A1, ARCN1, ARF4, ARFGAP1, ARFGAP3, ARL1, ARL3, ATL2, ATL3, BET1, CAPZA2, CCDC22, COG1, COG6, COG7, COG8, COPA, COPB1, COPB2, COPE, COPG1, COPG2, COPZ1, CTSC, DCTN1, DCTN4, DYNC1LI2, ERGIC1, ERGIC2, FTH1, GBF1, GOLGA2, GOPC, GOSR2, HTT, KDELR1, KDELR3, KIF11, KIF22, KIF23, KIF2C, KLC1, KRT18, LMAN1, MACF1, MON2, MYO5A, NBAS, NSF, OPTN, PLPP3, PREPL, RAB14, RAB1A, RAB2A, RAB8A, RP2, SAR1A, SCAMP2, SCFD1, SCYL1, SEC13, SEC16A, SEC22B, SEC23A, SEC23B, SEC24A, SEC24B, SEC24C, SEC24D, SEC31A, SNX3, SPTAN1, SPTBN1, SPTBN2, STX5, SURF4, TFG, TMED10, TMED2, TMED5, TMED7, TMED9, TMEM115, TXNDC5, USE1, VAMP2, VAMP3, VPS35L, YIF1A, YKT6]	
vesicle-mediated transport to the plasma membrane	GO_BiologicalProcess-EBI-UniProt-GOA-ACAP-ARAP_13.05.2021_00h00	2.06E-40	19.58042	28	[ANK3, ARF6, ARHGAP1, ARL3, CCDC22, EHD1, EHD2, EHD4, GOPC, KRT18, MACF1, NSF, OPTN, PREPL, RAB14, RAB8A, SEC16A, SNX17, SNX3, SNX4, SPTBN1, VAMP2, VAMP3, VPS26A, VPS26C, VPS35, VPS35L, VPS53]	Downregulation
cytosolic transport	GO_BiologicalProcess-EBI-UniProt-GOA-ACAP-ARAP_13.05.2021_00h00	2.06E-40	18.68132	34	[ACTR2, ANKFY1, AP2A1, ARL1, DCTN1, EEA1, GBF1, GOSR2, HEATR5A, HOOK3, KIF5B, LMAN1, MAPK1, MON2, MSN, PREPL, RAB14, RDX, RIC1, SNX3, SNX6, SRC, STX5, SURF4, TBC1D17, TMED9, UBE2O, USP7, VAMP3, VPS26A, VPS26B, VPS35, VPS53, YKT6]	Downregulation
protein localization to membrane	GO_BiologicalProcess-EBI-UniProt-GOA-ACAP-ARAP_13.05.2021_00h00	2.06E-40	13.63636	99	[ADAM10, AKT1, ANK2, ANK3, ANXA2, AP2B1, AP2M1, AP3B1, APOE, ARF6, ARL3, ARL6IP5, ATP2B4, CAV1, CD81, CDH2, DLG1, EGFR, EHD1, EHD2, EHD4, EMD, EPB41L3, F11R, FERMT2, GDAP1, GDI1, GET3, GET4, GOPC, GPC1, GPC3, GPC6, GPHN, GSN, HMOX1, HSPA4, HSPA5, ITGB1, KIF5B, KRT18, LRP1, MACF1, MAIP1, MAPK8, MESD,	Downregulation

					MYADM, MYO1C, MYO1E, MYO5A, NDC1, NSF, OPTN, PACS1, PAK1, PARD3, PICALM, PKP2, PPP1R9B, PPP2R5A, PREPL, PXMP2, RAB32, RAB8A, RAP2A, RDX, RFTN1, ROCK2, RPL13A, RPL22, RPL27A, RPL4, RPL5, RPL7A, RPS2, RPS21, RPS8, S100A10, SEC16A, SEC23A, SEC61G, SH3GLB1, SIL1, SLC5A3, SLC9A3R2, SORBS1, SPTBN1, SRP14, SRPRA, SSR1, STX5, TCAF1, TM9SF4, TMED2, TRAM1, VAMP2, VAMP3, VPS35, ZMYND8]	
post-Golgi vesicle-mediated transport	GO_BiologicalProcess-EBI-UniProt-GOA-ACAP-ARAP_13.05.2021_00h00	2.06E-40	19.46309	29	[ACBD3, ANK3, ANKFY1, AP1M1, AP1S1, AP2A1, ARL3, CCDC22, FTH1, GBF1, GOPC, GOSR2, KRT18, MACF1, MON2, MYO5A, NSF, OPTN, PREPL, RAB14, RP2, SCAMP2, SCFD1, SEC16A, SPTBN1, TXNDC5, VAMP2, VAMP3, VPS35L]	Downregulation
retrograde transport, endosome to Golgi	GO_BiologicalProcess-EBI-UniProt-GOA-ACAP-ARAP_13.05.2021_00h00	2.06E-40	22.77228	23	[ANKFY1, ARL1, DCTN1, GBF1, GOSR2, HEATR5A, LMAN1, PREPL, RIC1, SNX3, SNX6, STX5, SURF4, TBC1D17, TMED9, UBE2O, USP7, VAMP3, VPS26A, VPS26B, VPS35, VPS53, YKT6]	Downregulation
protein localization to cell periphery	GO_BiologicalProcess-EBI-UniProt-GOA-ACAP-ARAP_13.05.2021_00h00	2.06E-40	15.22989	53	[ADAM10, AKT1, ANK2, ANK3, ANXA2, AP2M1, ARF6, ARL3, ARL6IP5, ATP2B4, CAV1, CD81, CDH2, DLG1, EGFR, EHD1, EHD2, EHD4, EPB41, EPB41L3, F11R, GOPC, GPC6, ITGB1, KIF5B, KRT18, LRP1, MACF1, MYADM, MYO5A, NSF, OPTN, PACS1, PICALM, PKP2, PLK1, PPP1R9B, PPP2R5A, PREPL, RAB8A, RAP2A, RDX, ROCK2, S100A10, SEC16A, SEC23A, SLC9A3R2, SORBS1, SPTBN1, TMED2, VAMP2, VAMP3, VPS35]	Downregulation
protein localization to plasma membrane	GO_BiologicalProcess-EBI-UniProt-GOA-ACAP-ARAP_13.05.2021_00h00	2.06E-40	15.9322	47	[AKT1, ANK2, ANK3, ANXA2, AP2M1, ARF6, ARL6IP5, ATP2B4, CD81, CDH2, DLG1, EGFR, EHD1, EHD2, EHD4, EPB41L3, F11R, GOPC, ITGB1, KIF5B, KRT18, LRP1, MACF1, MYADM, MYO5A, NSF, OPTN, PACS1, PICALM, PKP2, PPP1R9B, PPP2R5A, PREPL, RAB8A, RAP2A, RDX,	Downregulation

					ROCK2, S100A10, SEC16A, SEC23A, SLC9A3R2, SORBS1, SPTBN1, TMED2, VAMP2, VAMP3, VPS35]	
response to organic substance	GO_BiologicalProcess-EBI-UniProt-GOA-ACAP-ARAP_13.05.2021_00h00	3.72E-16	10.98528	388	[ABAT, ABCB10, ABCC1, ACACA, ACAT1, ACSL1, ACSL4, ACTN4, ACTR2, ADAM10, ADAR, AGL, AKT1, ALPL, ANK2, ANKZF1, ANXA1, ANXA2, ANXA3, APAF1, APOE, APPL2, APRT, ARF6, ARFGAP1, ARFGAP3, ARHGDIA, ARL6IP5, ARSA, ARSB, ASNS, ATIC, ATP1A1, ATP2B1, ATP2B4, ATP6V1A, AUP1, BAG3, BAIAP2, BCAT2, BPTF, BRWD1, CACYBP, CALCOCO2, CALR, CAMK2D, CANX, CAPN2, CASK, CASP3, CASTOR2, CAT, CAV1, CBL, CCNB1, CD44, CD81, CDC42, CDC73, CDK5RAP3, CGN, CHEK2, CNRIP1, CNTFR, COL1A1, COL1A2, COL4A1, COL4A2, COL5A2, COL6A1, COL6A2, COPS5, CTBP2, CTNNB1, CTSC, CUL7, CYFIP1, DAPK1, DAXX, DCTN1, DDRGK1, DDX18, DDX21, DDX5, DDX54, DHX15, DHX8, DHX9, DIAPH1, DNMT1, DNMT3A, DNMT3B, DPYSL2, DPYSL3, DUSP3, DYNC1LI2, ECPAS, EDEM3, EEF2, EFTUD2, EGFR, EHD1, EHD4, EIF4A3, EMD, EPB41L5, EPM2AIP1, EPN2, EPS8, ERBIN, ERLEC1, ERLIN2, ERO1A, ERP44, ESD, ETFA, F11R, FAF2, FBN2, FBXO2, FECH, FERMT2, FGG, FKBP14, FKBP8, FLNB, FLT1, FN1, FSCN1, GAB1, GARS1, GART, GATA6, GCLC, GCLM, GDAP1, GET4, GFPT2, GIPC1, GLUL, GNG12, GOLT1B, GOSR2, GOT2, GPC1, GPC3, GPI, GSDME, GSN, GSS, GSTM2, GSTM3, H6PD, HADHA, HCFC1, HDAC2, HDAC4, HELLS, HK2, HLA-A, HMGCL, HMOX1, HNRNPF, HNRNPM, HSP90AB1, HSP90B1, HSPA13, HSPA14, HSPA2, HSPA4, HSPA5, HSPA9, HSPB1, HSPD1, HSPE1, HSPH1, HTRA1, ILK, IMPDH2, IPO5, IQGAP1, IQGAP3, IRF3, ITGA5, ITGA6, ITGB1,	Downregulation

					JARID2, KANK2, KAT7, KDELR3, KDM1A, KEAP1, KIF5B, KRT18, KRT19, KRT8, LANCL2, LARS1, LGALS1, LIMS1, LIN28A, LRP1, LTBP1, MAN1A1, MAP1B, MAP1LC3A, MAP2K6, MAPK1, MAPK14, MAPK8, MARS1, MAVS, MBD3, MCM2, MCM7, ME1, MEAK7, MGST1, MME, MPC2, MSN, MTAP, MTDH, MYD88, MYDGF, MYO1C, MYO1E, MYO5A, NCAM1, NCL, NEDD4, NFKB1, NIBAN2, NPM1, NRP2, NUP35, OCLN, OGT, OPTN, OSBP, OXCT1, P4HB, PABPN1, PAK1, PARD3, PARP1, PDGFRB, PDIA3, PDIA6, PEA15, PELP1, PFKL, PFKP, PGRMC2, PHIP, PHPT1, PIAS4, PIK3C2A, PIK3CA, PIP4K2B, PIP4K2C, PKM, PLCG1, PLOD3, PLSCR3, PNPT1, POLB, POLR2A, POLR2B, POLR2G, POR, PPP1R9B, PRKAA1, PRKACB, PRKACG, PRKAR1A, PRKAR2A, PRKAR2B, PRKCA, PRKDC, PRPF8, PSMC6, PSMD1, PSMD2, PSMD9, PSME3, PTGIS, PTK7, PTPN1, PTPN12, PTPN2, PXDN, PYCARD, QDPR, RAB14, RAB8A, RAD51, RANBP2, RANGAP1, RBBP5, RBM15, RBPJ, RBPMS2, RDX, RELA, RFC3, RIF1, ROCK2, RPL13A, RPS6KA3, RPS6KA4, SAMHD1, SARM1, SDF4, SEC31A, SEL1L, SERPINB9, SERPINH1, SESN2, SETD7, SETDB1, SHPK, SIN3A, SIRT1, SIRT2, SKP2, SLC1A3, SLC25A5, SLC2A1, SLC2A10, SLC33A1, SMARCA4, SMARCA5, SMARCC1, SMARCD1, SNRPA1, SNX6, SORBS1, SORD, SPARC, SRC, SRM, SRPRA, SRPRB, SRR, SRSF7, SSR1, STT3B, TAX1BP1, TBC1D4, TBL2, TCP1, TGFB1I1, TGM2, THBS1, TIA1, TIMP3, TLN1, TMED10, TMED2, TMF1, TOLLIP, TRIM33, TRIM71, TTLL12, UBE2O, UBR5, UFL1, UGGT1, UGGT2, USP15, USP19, USP9X, VAMP2, VAMP3, VIM, VPS26B, VPS35, XRCC5, XRN1, YAP1, YIF1A, YOD1, YTHDC2, ZC3H15,	

					ZDHHC17, ZFP36L2, ZFYVE16]	
cellular response to chemical stimulus	GO_BiologicalProcess-EBI-UniProt-GOA-ACAP-ARAP_13.05.2021_00h00	3.72E-16	10.6383	375	[ABCB10, ABCC1, ABTB2, ACAA1, ACACA, ACSL1, ACTN4, ACTR2, ADAM10, ADAR, AK4, AKT1, ALPL, ANK3, ANKZF1, ANO6, ANXA1, ANXA2, APAF1, APOE, APPL2, APRT, ARF6, ARFGAP1, ARFGAP3, ARHGDIA, ARL6IP5, ASNS, ATIC, ATP1A1, ATP2B1, ATP2B4, ATP6V1A, AUP1, BAG3, BAIAP2, BCAT2, BPTF, BRWD1, C1QBP, CACYBP, CALR, CAMK2D, CANX, CAPN2, CASK, CASP3, CASTOR2, CAT, CAV1, CBL, CCNB1, CD44, CDC42, CDC73, CDK5RAP3, CGN, CHEK2, CMBL, CNRIP1, CNTFR, COL1A1, COL1A2, COL4A1, COL4A2, COL5A2, COL6A1, COPS5, CPNE2, CTBP2, CTNNB1, CUL7, CXADR, CYB5R3, CYFIP1, CYP2S1, DAPK1, DAXX, DCTN1, DDRGK1, DDX18, DDX5, DDX54, DHX15, DHX8, DHX9, DIAPH1, DNMT1, DNMT3A, DNMT3B, DPYSL3, DUSP3, DYNC1LI2, ECT2, EEF2, EFTUD2, EGFR, EHD1, EHD4, EIF4A3, EMD, EPB41L5, EPN2, EPS8, ERBIN, ERO1A, ESD, ETFA, F11R, FBN2, FECH, FERMT2, FGG, FKBP14, FKBP8, FLNB, FLT1, FN1, FSCN1, GAB1, GARS1, GATA6, GBF1, GCLC, GCLM, GDAP1, GFPT2, GIPC1, GOLT1B, GOSR2, GPC1, GPC3, GPI, GPX7, GPX8, GSDME, GSN, GSTK1, GSTM2, GSTM3, HCFC1, HDAC2, HDAC4, HELLS, HK2, HLA-A, HMOX1, HNRNPF, HNRNPM, HP1BP3, HSP90AB1, HSP90B1, HSPA13, HSPA14, HSPA2, HSPA5, HSPA9, HSPB1, HSPD1, HTRA1, ILK, IMPDH2, IPO5, IQGAP1, IQGAP3, IRF3, ITGA5, ITGA6, ITGB1, JAM3, JARID2, KANK2, KDELR3, KDM1A, KDM3B, KEAP1, KIF5B, KRT18, KRT8, LANCL2, LARS1, LGALS1, LIMS1, LIN28A, LRP1, LTBP1, MAP1B, MAP1LC3A, MAP2K6, MAPK1, MAPK14, MAPK8, MARS1, MAVS, MCM2, MCM7, MCU,	Downregulation

					MEAK7, MGST1, MME, MOSPD2, MPC2, MSN, MTAP, MTDH, MTR, MYD88, MYDGF, MYO1C, MYO1E, MYO5A, NCAM1, NCL, NDRG1, NEDD4, NFKB1, NIBAN2, NNT, NPM1, NQO2, NRP2, NUP35, NXN, OCLN, OGT, OPTN, OSBP, OXCT1, P4HB, PABPN1, PAK1, PARD3, PARP1, PARVA, PAWR, PDGFRB, PDIA3, PDIA6, PELP1, PFKP, PGRMC2, PHIP, PHPT1, PIAS4, PIK3C2A, PIK3CA, PIP4K2B, PIP4K2C, PKM, PLCG1, PLOD3, PLSCR3, PNPT1, POLR2A, POLR2B, POLR2G, POR, PPIB, PPM1F, PPP1R9B, PRDX1, PRDX4, PRKAA1, PRKACB, PRKACG, PRKAR1A, PRKAR2A, PRKAR2B, PRKCA, PRKDC, PRPF38B, PRPF8, PSMC6, PSMD1, PSMD2, PSMD9, PSME3, PTGIS, PTK7, PTPN1, PTPN12, PTPN2, PXDN, PYCARD, PYCR1, PYCR2, QDPR, RAB14, RAB8A, RAD51, RANGAP1, RAP2A, RBM15, RBPJ, RBPMS2, RDX, RELA, RIF1, ROCK2, RPL13A, RPS6KA4, SAMHD1, SBDS, SEC31A, SERPINB6, SERPINB9, SESN2, SHPK, SIN3A, SIRT1, SIRT2, SKP2, SLC1A3, SLC25A24, SLC25A5, SLC2A1, SLC2A10, SLC33A1, SMARCA4, SMARCA5, SMARCC1, SMARCD1, SNRPA1, SNX6, SORBS1, SORD, SRC, SRM, SRPRA, SRPRB, SRSF7, SSR1, TAX1BP1, TBC1D4, TBL2, TCP1, TF, TGFB1I1, TGM2, THBS1, TIA1, TIGAR, TLN1, TMED2, TMF1, TOLLIP, TPM1, TRAP1, TRIM33, TRIM71, TTLL12, TXNRD1, UBE2O, UBR5, UFL1, UGGT1, UGGT2, USP15, USP19, USP9X, VAMP2, VAMP3, VIM, VPS26B, VPS35, XRCC5, XRCC6, XRN1, YAP1, YIF1A, YOD1, ZC3H15, ZDHHC17, ZFP36L2, ZFYVE16]	
cellular response to endogenous stimulus	GO_BiologicalProcess-EBI-UniProt-GOA-	3.72E-16	11.26574	170	[ABCC1, ACACA, ACSL1, ACTR2, AKT1, ANXA1, APAF1, APPL2, APRT, ARF6, ASNS, ATP1A1, ATP2B1, ATP2B4,	Downregulation

	ACAP-ARAP_13.05.2021_00h00				ATP6V1A, BAIAP2, BPTF, CALR, CAPN2, CASP3, CASTOR2, CAV1, CBL, CD44, CGN, COL1A1, COL1A2, COL4A1, COL4A2, COL5A2, COL6A1, CTNNB1, CYFIP1, DAXX, DDRGK1, DDX5, DDX54, DHX15, DHX8, DIAPH1, DNMT1, DNMT3A, DNMT3B, DUSP3, DYNC1LI2, EEF2, EGFR, EHD1, EIF4A3, EPB41L5, F11R, FBN2, FECH, FERMT2, FKBP8, GAB1, GARS1, GATA6, GCLC, GCLM, GIPC1, GOLT1B, GPC1, GPC3, GPI, GSTM2, HDAC2, HDAC4, HNRNPF, HNRNPM, HSP90AB1, HSPA5, HTRA1, ILK, IPO5, IQGAP1, ITGB1, KANK2, KDM1A, LANCL2, LARS1, LIMS1, LRP1, LTBP1, MAP1B, MAPK1, MARS1, MCM7, MYO1C, MYO1E, MYO5A, NCL, NEDD4, NFKB1, OGT, PAK1, PARD3, PARP1, PDGFRB, PELP1, PGRMC2, PHIP, PIK3C2A, PIK3CA, PIP4K2B, PIP4K2C, PKM, PLCG1, PLOD3, POLR2A, POLR2B, POLR2G, POR, PPP1R9B, PRKAA1, PRKACB, PRKACG, PRKAR1A, PRKAR2A, PRKAR2B, PRKCA, PRKDC, PTPN1, PTPN12, PTPN2, PXDN, RAB14, RAB8A, RAD51, RANGAP1, RBPJ, RBPMS2, RDX, RELA, ROCK2, SERPINB9, SESN2, SIN3A, SIRT1, SIRT2, SKP2, SLC1A3, SLC2A10, SLC33A1, SMARCA4, SMARCC1, SNX6, SORBS1, SRC, TBC1D4, TGFB1I1, TGM2, THBS1, TIA1, TIGAR, TMF1, TRIM33, TRIM71, UBE2O, UBR5, UFL1, USP15, USP9X, VAMP2, VIM, VPS35, XRN1, YAP1, ZFP36L2, ZFYVE16]	
response to nitrogen compound	GO_BiologicalProcess-EBI-UniProt-GOA-ACAP-ARAP_13.05.2021_00h00	3.72E-16	12.1732	149	[ABAT, ABCC1, ACTR2, AKT1, ANKZF1, ANXA1, APOE, APPL2, APRT, ASNS, ATP2B1, ATP2B4, ATP6V1A, AUP1, BAIAP2, CACYBP, CAPN2, CASP3, CASTOR2, CAT, CAV1, CD81, COL1A1, COL1A2, COL4A1, COL5A2, COL6A1, CTNNB1, CYFIP1, DAXX, DDX21, DHX15, DHX8, DHX9, DIAPH1, DNMT1,	Downregulation

					DNMT3A, DNMT3B, DPYSL2, ECPAS, EDEM3, EEF2, EFTUD2, EGFR, EPM2AIP1, ERBIN, ERLEC1, ERLIN2, FAF2, FBXO2, GAB1, GCLC, GCLM, GET4, GOLT1B, GOT2, GPI, GSN, GSS, GSTM2, GSTM3, HADHA, HDAC2, HSP90AB1, HSP90B1, HSPA5, HSPD1, IPO5, IQGAP1, IRF3, ITGB1, KAT7, KDM1A, LARS1, LRP1, MAN1A1, MAP1B, MAP1LC3A, MAPK1, MAPK14, MARS1, MAVS, MEAK7, MGST1, MTR, MYD88, MYO1C, MYO1E, MYO5A, NFKB1, NPM1, OGT, PAK1, PARP1, PEA15, PHIP, PIK3C2A, PIK3CA, PIP4K2B, PIP4K2C, PKM, PNPT1, POR, PPP1R9B, PRKAA1, PRKACB, PRKACG, PRKAR1A, PRKAR2A, PRKAR2B, PRKCA, PRKDC, PSMC6, PTPN1, PTPN2, PXDN, QDPR, RAB8A, RAD51, RANBP2, RANGAP1, RELA, ROCK2, SEL1L, SESN2, SIN3A, SIRT1, SIRT2, SLC1A3, SLC2A1, SMARCC1, SNX6, SORBS1, SPARC, SRC, SRR, STT3B, TBC1D4, TGM2, TMED10, UFL1, UGGT1, UGGT2, USP19, VAMP2, VIM, VPS35, XRN1, YOD1]	
response to oxygen-containing compound	GO_BiologicalProcess-EBI-UniProt-GOA-ACAP-ARAP_13.05.2021_00h00	3.72E-16	10.27884	188	[ABAT, ABCC1, ACACA, ACSL1, ACTR2, AKT1, ALPL, ANKZF1, ANXA1, APOE, APPL2, APRT, ARSA, ASNS, ATP1A1, ATP2B1, ATP2B4, ATP6V1A, BAIAP2, CACYBP, CALR, CAPN2, CASP3, CASTOR2, CAT, CAV1, CBL, CCNB1, CDC73, CHEK2, COL1A1, COL1A2, COL4A1, COL5A2, COL6A1, COL6A2, CTNNB1, CYFIP1, DAPK1, DDX18, DHX15, DHX8, DNMT1, DNMT3A, DNMT3B, DPYSL2, ECT2, EDEM3, EEF2, EFTUD2, EGFR, EPM2AIP1, EPS8, ERBIN, ESD, FECH, GAB1, GATA6, GCLC, GCLM, GDAP1, GLUL, GNG12, GOLT1B, GOT2, GPI, GSN, GSS, H6PD, HADHA, HDAC2, HMGCL, HMOX1, HSP90AB1, HSPA5, HSPD1, IPO5, IQGAP1, IRF3, KANK2, KAT7, KDM1A, KIF5B, KRT18, KRT8, LANCL2, LARS1,	Downregulation

					LGALS1, LIN28A, LRP1, MAN1A1, MAP1B, MAP1LC3A, MAP2K6, MAPK1, MAPK14, MAPK8, MARS1, MBD3, ME1, MEAK7, MGST1, MPC2, MSN, MTAP, MTDH, MTR, MYD88, MYO1C, MYO1E, MYO5A, NFKB1, OGT, OSBP, OXCT1, PABPN1, PAK1, PARP1, PAWR, PDGFRB, PFKL, PHIP, PHPT1, PIK3C2A, PIK3CA, PIP4K2B, PIP4K2C, PKM, PLSCR3, PNPT1, POLB, POR, PPP1R9B, PRDX1, PRKAA1, PRKACB, PRKACG, PRKAR1A, PRKAR2A, PRKAR2B, PRKCA, PRKDC, PRPF38B, PRPF8, PTK7, PTPN1, PTPN2, PXDN, PYCARD, PYCR1, QDPR, RAB8A, RAD51, RANGAP1, RELA, ROCK2, RPS6KA3, SARM1, SDF4, SESN2, SETD7, SETDB1, SHPK, SIN3A, SIRT1, SIRT2, SLC1A3, SLC2A1, SMARCA4, SMARCC1, SMARCD1, SNX6, SORBS1, SPARC, SRC, SRR, TBC1D4, TGM2, THBS1, TPM1, TRAP1, UFL1, VAMP2, VIM, VPS35, XRCC5, XRN1, YAP1]	
response to hormone	GO_BiologicalProcess-EBI-UniProt-GOA-ACAP-ARAP_13.05.2021_00h00	3.72E-16	11.38371	116	[ACACA, ACAT1, ACSL1, AGL, AKT1, ALPL, ANXA1, ANXA2, ANXA3, APPL2, APRT, ARSA, ARSB, ASNS, ATP1A1, ATP2B1, ATP6V1A, BAIAP2, CACYBP, CALR, CASP3, CAT, CAV1, COL1A1, CYFIP1, DAXX, DDRGK1, DDX5, DDX54, DNMT3B, EGFR, EPM2AIP1, FECH, GAB1, GATA6, GCLC, GCLM, GOLT1B, GOT2, GPI, GSTM3, HADHA, HDAC4, HMOX1, HSPD1, IQGAP1, KANK2, KDM1A, KRT19, LANCL2, MAP1B, MAPK1, MARS1, ME1, MEAK7, MYO1C, MYO1E, MYO5A, NEDD4, NFKB1, OGT, OXCT1, PAK1, PARD3, PARP1, PDGFRB, PELP1, PGRMC2, PHIP, PIK3C2A, PIK3CA, PIP4K2B, PIP4K2C, PKM, PLOD3, PNPT1, POR, PPP1R9B, PRKAA1, PRKACB, PRKACG, PRKAR1A, PRKAR2A, PRKAR2B, PRKCA, PRKDC, PTPN1, PTPN2, PXDN, QDPR, RAB8A,	Downregulation

					RANGAP1, RBBP5, RDX, RELA, ROCK2, SERPINB9, SESN2, SIRT1, SKP2, SLC2A1, SMARCA4, SMARCC1, SORBS1, SORD, SPARC, SRC, TBC1D4, THBS1, TIMP3, TMF1, UBR5, UFL1, VAMP2, YAP1, ZFP36L2]	
response to organonitrogen compound	GO_BiologicalProcess-EBI-UniProt-GOA-ACAP-ARAP_13.05.2021_00h00	3.72E-16	12.41197	141	[ABAT, ABCC1, ACTR2, AKT1, ANKZF1, ANXA1, APOE, APPL2, APRT, ASNS, ATP2B1, ATP2B4, ATP6V1A, AUP1, BAIAP2, CACYBP, CAPN2, CASP3, CASTOR2, CAT, CAV1, CD81, COL1A1, COL1A2, COL4A1, COL5A2, COL6A1, CTNNB1, CYFIP1, DHX15, DHX8, DIAPH1, DNMT1, DNMT3A, DNMT3B, DPYSL2, ECPAS, EDEM3, EEF2, EFTUD2, EGFR, EPM2AIP1, ERBIN, ERLEC1, ERLIN2, FAF2, FBXO2, GAB1, GCLC, GCLM, GET4, GOLT1B, GOT2, GPI, GSN, GSS, GSTM2, HADHA, HDAC2, HSP90AB1, HSP90B1, HSPA5, HSPD1, IPO5, IQGAP1, ITGB1, KAT7, KDM1A, LARS1, LRP1, MAN1A1, MAP1B, MAP1LC3A, MAPK1, MAPK14, MARS1, MEAK7, MGST1, MYD88, MYO1C, MYO1E, MYO5A, NFKB1, OGT, PAK1, PARP1, PEA15, PHIP, PIK3C2A, PIK3CA, PIP4K2B, PIP4K2C, PKM, PNPT1, POR, PPP1R9B, PRKAA1, PRKACB, PRKACG, PRKAR1A, PRKAR2A, PRKAR2B, PRKCA, PRKDC, PSMC6, PTPN1, PTPN2, PXDN, QDPR, RAB8A, RAD51, RANBP2, RANGAP1, RELA, ROCK2, SEL1L, SESN2, SIN3A, SIRT1, SIRT2, SLC1A3, SLC2A1, SMARCC1, SNX6, SORBS1, SPARC, SRC, SRR, STT3B, TBC1D4, TGM2, TMED10, UFL1, UGGT1, UGGT2, USP19, VAMP2, VIM, VPS35, XRN1, YOD1]	Downregulation
response to growth factor	GO_BiologicalProcess-EBI-UniProt-GOA-ACAP-ARAP_13.05.2021_00h00	3.72E-16	13.64221	106	[AKT1, ANXA1, ANXA3, APAF1, APPL2, ARF6, ATP2B4, BAIAP2, BPTF, CASK, CAT, CAV1, CBL, CD44, CGN, COL1A1, COL1A2, COL4A2, CTNNB1, CYFIP1, DDX5, DUSP3, DYNC1LI2, EEF2, EGFR, EHD1, EHD4, EIF4A3, EMD, EPB41L5, EPN2, F11R,	Downregulation

					FBN2, FERMT2, FKBP8, FLT1, GAB1, GARS1, GATA6, GCLC, GCLM, GIPC1, GOLT1B, GPC1, GPC3, GPI, HDAC2, HNRNPF, HNRNPM, HSP90AB1, HSPA5, HSPB1, HTRA1, ILK, IQGAP1, ITGA5, ITGB1, LIMS1, LTBP1, MAP1B, MAPK1, MAPK14, MARS1, MCM7, MYO1C, MYO1E, NCL, NEDD4, NIBAN2, NRP2, PARD3, PARP1, PDGFRB, PIK3CA, PLCG1, POLR2A, POLR2B, POLR2G, PPP1R9B, PTPN1, PTPN12, PXDN, RAB14, RBPJ, RBPMS2, RDX, RELA, ROCK2, SIRT1, SIRT2, SLC2A10, SLC33A1, SNX6, SORD, SRC, TGFB1I1, THBS1, TIA1, TRIM33, TRIM71, UBE2O, USP15, USP9X, ZDHHC17, ZFP36L2, ZFYVE16]	
cellular response to organic substance	GO_BiologicalProcess-EBI-UniProt-GOA-ACAP-ARAP_13.05.2021_00h00	3.72E-16	11.02253	318	[ABCB10, ABCC1, ACACA, ACSL1, ACTN4, ACTR2, ADAR, AKT1, ALPL, ANXA1, ANXA2, APAF1, APPL2, APRT, ARF6, ARFGAP1, ARFGAP3, ARHGDIA, ARL6IP5, ASNS, ATIC, ATP1A1, ATP2B1, ATP2B4, ATP6V1A, AUP1, BAG3, BAIAP2, BCAT2, BPTF, BRWD1, CACYBP, CALR, CAMK2D, CANX, CAPN2, CASK, CASP3, CASTOR2, CAT, CAV1, CBL, CCNB1, CD44, CDC42, CDC73, CDK5RAP3, CGN, CHEK2, CNTFR, COL1A1, COL1A2, COL4A1, COL4A2, COL5A2, COL6A1, COPS5, CTBP2, CTNNB1, CUL7, CYFIP1, DAPK1, DAXX, DCTN1, DDRGK1, DDX18, DDX5, DDX54, DHX15, DHX8, DHX9, DIAPH1, DNMT1, DNMT3A, DNMT3B, DPYSL3, DUSP3, DYNC1LI2, EEF2, EGFR, EHD1, EHD4, EIF4A3, EMD, EPB41L5, EPN2, EPS8, ERBIN, ERO1A, ETFA, F11R, FBN2, FECH, FERMT2, FGG, FKBP14, FKBP8, FLNB, FLT1, FN1, FSCN1, GAB1, GARS1, GATA6, GCLC, GCLM, GDAP1, GFPT2, GIPC1, GOLT1B, GOSR2, GPC1, GPC3, GPI, GSDME, GSN, GSTM2, HCFC1, HDAC2, HDAC4, HELLS, HK2, HLA-A, HMOX1,	No change

					HNRNPF, HNRNPM, HSP90AB1, HSP90B1, HSPA13, HSPA14, HSPA2, HSPA5, HSPA9, HSPB1, HSPD1, HTRA1, ILK, IMPDH2, IPO5, IQGAP1, IQGAP3, IRF3, ITGA5, ITGA6, ITGB1, JARID2, KANK2, KDELR3, KDM1A, KEAP1, KIF5B, KRT18, KRT8, LANCL2, LARS1, LGALS1, LIMS1, LIN28A, LRP1, LTBP1, MAP1B, MAP2K6, MAPK1, MAPK14, MAPK8, MARS1, MAVS, MCM2, MCM7, MGST1, MME, MPC2, MSN, MTAP, MTDH, MYD88, MYDGF, MYO1C, MYO1E, MYO5A, NCAM1, NCL, NEDD4, NFKB1, NIBAN2, NPM1, NRP2, NUP35, OCLN, OGT, OPTN, OSBP, OXCT1, P4HB, PABPN1, PAK1, PARD3, PARP1, PDGFRB, PDIA3, PDIA6, PELP1, PFKP, PGRMC2, PHIP, PHPT1, PIAS4, PIK3C2A, PIK3CA, PIP4K2B, PIP4K2C, PKM, PLCG1, PLOD3, PLSCR3, PNPT1, POLR2A, POLR2B, POLR2G, POR, PPP1R9B, PRKAA1, PRKACB, PRKACG, PRKAR1A, PRKAR2A, PRKAR2B, PRKCA, PRKDC, PRPF8, PSMC6, PSMD1, PSMD2, PSMD9, PSME3, PTGIS, PTK7, PTPN1, PTPN12, PTPN2, PXDN, PYCARD, RAB14, RAB8A, RAD51, RANGAP1, RBM15, RBPJ, RBPMS2, RDX, RELA, RIF1, ROCK2, RPL13A, RPS6KA4, SAMHD1, SEC31A, SERPINB9, SESN2, SHPK, SIN3A, SIRT1, SIRT2, SKP2, SLC1A3, SLC25A5, SLC2A10, SLC33A1, SMARCA4, SMARCA5, SMARCC1, SMARCD1, SNRPA1, SNX6, SORBS1, SORD, SRC, SRM, SRPRA, SRPRB, SRSF7, SSR1, TAX1BP1, TBC1D4, TBL2, TCP1, TGFB1I1, TGM2, THBS1, TIA1, TLN1, TMED2, TMF1, TOLLIP, TRIM33, TRIM71, TTLL12, UBE2O, UBR5, UFL1, UGGT1, UGGT2, USP15, USP9X, VAMP2, VAMP3, VIM, VPS26B, VPS35, XRCC5, XRN1, YAP1, YIF1A, YOD1, ZC3H15, ZDHHC17, ZFP36L2, ZFYVE16]	

cellular response to nitrogen compound	GO_BiologicalProcess-EBI-UniProt-GOA-ACAP-ARAP_13.05.2021_00h00	3.72E-16	11.89543	91	[ABCC1, ACTR2, AKT1, APPL2, APRT, ATP2B1, ATP2B4, ATP6V1A, BAIAP2, CAPN2, CASP3, CASTOR2, CAV1, COL1A1, COL1A2, COL4A1, COL5A2, COL6A1, CTNNB1, CYFIP1, DAXX, DHX15, DHX8, DHX9, DIAPH1, DNMT1, DNMT3A, GAB1, GCLC, GCLM, GOLT1B, GSTM2, HDAC2, HSPA5, IPO5, IRF3, KDM1A, LARS1, LRP1, MAP1B, MAPK1, MARS1, MAVS, MTR, MYO1C, MYO1E, MYO5A, NFKB1, NPM1, OGT, PAK1, PARP1, PHIP, PIK3C2A, PIK3CA, PIP4K2B, PIP4K2C, PKM, POR, PPP1R9B, PRKAA1, PRKACB, PRKACG, PRKAR1A, PRKAR2A, PRKAR2B, PRKCA, PRKDC, PTPN1, PTPN2, PXDN, RAB8A, RAD51, RANGAP1, RELA, ROCK2, SESN2, SIN3A, SIRT1, SIRT2, SLC1A3, SMARCC1, SNX6, SORBS1, SRC, TBC1D4, TGM2, VAMP2, VIM, VPS35, XRN1]	Downregulation
cellular response to organonitrogen compound	GO_BiologicalProcess-EBI-UniProt-GOA-ACAP-ARAP_13.05.2021_00h00	3.72E-16	12.09104	85	[ABCC1, ACTR2, AKT1, APPL2, APRT, ATP2B1, ATP2B4, ATP6V1A, BAIAP2, CAPN2, CASP3, CASTOR2, CAV1, COL1A1, COL1A2, COL4A1, COL5A2, COL6A1, CTNNB1, CYFIP1, DHX15, DHX8, DIAPH1, DNMT1, DNMT3A, GAB1, GCLC, GCLM, GOLT1B, GSTM2, HDAC2, HSPA5, IPO5, KDM1A, LARS1, LRP1, MAP1B, MAPK1, MARS1, MYO1C, MYO1E, MYO5A, NFKB1, OGT, PAK1, PARP1, PHIP, PIK3C2A, PIK3CA, PIP4K2B, PIP4K2C, PKM, POR, PPP1R9B, PRKAA1, PRKACB, PRKACG, PRKAR1A, PRKAR2A, PRKAR2B, PRKCA, PRKDC, PTPN1, PTPN2, PXDN, RAB8A, RAD51, RANGAP1, RELA, ROCK2, SESN2, SIN3A, SIRT1, SIRT2, SLC1A3, SMARCC1, SNX6, SORBS1, SRC, TBC1D4, TGM2, VAMP2, VIM, VPS35, XRN1]	Downregulation
cellular response to growth factor stimulus	GO_BiologicalProcess-EBI-UniProt-GOA-	3.72E-16	13.95973	104	[AKT1, ANXA1, APAF1, APPL2, ARF6, ATP2B4, BAIAP2, BPTF, CASK, CAT, CAV1, CBL, CD44, CGN, COL1A1, COL1A2,	Downregulation

	ACAP-ARAP_13.05.2021_00h00				COL4A2, CTNNB1, CYFIP1, DDX5, DUSP3, DYNC1LI2, EEF2, EGFR, EHD1, EHD4, EIF4A3, EMD, EPB41L5, EPN2, F11R, FBN2, FERMT2, FKBP8, FLT1, GAB1, GARS1, GATA6, GCLC, GCLM, GIPC1, GOLT1B, GPC1, GPC3, GPI, HDAC2, HNRNPF, HNRNPM, HSP90AB1, HSPA5, HSPB1, HTRA1, ILK, IQGAP1, ITGA5, ITGB1, LIMS1, LTBP1, MAP1B, MAPK1, MAPK14, MARS1, MCM7, MYO1C, MYO1E, NCL, NEDD4, NIBAN2, NRP2, PARD3, PARP1, PDGFRB, PIK3CA, PLCG1, POLR2A, POLR2B, POLR2G, PPP1R9B, PTPN1, PTPN12, PXDN, RAB14, RBPJ, RBPMS2, RDX, RELA, SIRT1, SIRT2, SLC2A10, SLC33A1, SNX6, SORD, SRC, TGFB1I1, THBS1, TIA1, TRIM33, TRIM71, UBE2O, USP15, USP9X, ZDHHC17, ZFP36L2, ZFYVE16]	
cell cycle process	GO_BiologicalProcess-EBI-UniProt-GOA-ACAP-ARAP_13.05.2021_00h00	5.48E-33	13.61111	196	[AAAS, ACTR2, AKT1, ANK3, ANTXR1, ANXA1, ANXA11, APAF1, APPL2, ARAP1, ARL2, ARL3, ATP2B4, ATR, AURKA, AURKB, BCCIP, BIRC6, BUB1B, CALR, CBX5, CCAR2, CCNB1, CCND2, CCNH, CCNY, CDC42, CDC73, CDCA8, CDK5RAP3, CDK7, CHEK1, CHEK2, CHTF18, CKAP5, CLASP1, CNOT11, CRLF3, CTCF, CTNNB1, CUL4A, CUL4B, CUL7, DCTN1, DDRGK1, DDX39B, DLG1, DLGAP5, DRG1, DYNC1LI2, ECT2, EGFR, EHMT1, EMD, EML1, EPS8, ERCC2, ERCC6L, FANCD2, FHL1, FSD1, GINS3, GIPC1, GOLGA2, HMMR, HSPA2, HTT, INCENP, IQGAP3, IST1, ITGB1, KANK2, KIF11, KIF20A, KIF22, KIF23, KIF2C, KNTC1, LBR, LCMT1, LIG1, MACROH2A1, MAPK14, MASTL, MCM2, MCM3, MCM4, MCM5, MCM6, MCM7, MCMBP, MDC1, MKI67, MLH1, MMAB, MSH2, MSH3, MSH6, MTA3, MYH9, NCAPD2, NCAPG, NCAPG2, NDC1, NDC80, NDRG1, NEK7, NPM1, NSFL1C, NSUN2, NUDT16, NUP107, NUP133,	Upregulation

					NUP160, NUP35, NUSAP1, OPTN, ORC3, P3H4, PARD6B, PDGFRB, PDS5A, PHC1, PHIP, PIK3R4, PLK1, POGZ, POLA1, POLA2, POLD1, POLE, POLR1B, PPP1R9B, PRIM1, PRIM2, PRKAR1A, PRKAR2B, PRKCA, PRKDC, PRPF40A, PSMC6, PSMD1, PSMD2, PSMD9, PSME3, RAD51, RAN, RANBP2, RANGAP1, RCC2, RDX, RFC1, RFC2, RFC3, RFC5, ROCK2, RPA1, RPA2, RPA3, RRS1, RTKN, RTTN, SBDS, SEC13, SEH1L, SEPTIN10, SEPTIN11, SEPTIN2, SEPTIN7, SEPTIN8, SET, SH3GLB1, SIN3A, SIRT1, SIRT2, SKP2, SMARCAD1, SMC2, SNX9, SPTBN1, SUN2, TACC3, TIPRL, TOP2A, TPX2, TRIM71, TRIP13, TTK, TUBA4A, USP19, USP47, VRK1, WAPL, YTHDC2, ZFP36L2, ZWILCH]	
macromolecule modification	GO_BiologicalProcess-EBI-UniProt-GOA-ACAP-ARAP_13.05.2021_00h00	5.48E-33	10.01356	443	[AAAS, AASS, ABHD10, ABHD12, ABI1, ACSL1, ADAM10, ADAR, ADARB1, AGTPBP1, AIMP2, AKT1, ALG11, ALG2, ALG5, ALG9, ANTXR1, ANXA1, ANXA2, AP3B1, APOBEC3C, APOE, ARF4, ARFGEF1, ASPH, ATF7IP, ATP2B4, ATR, AUP1, AURKA, AURKB, B3GLCT, B4GALT1, BAG2, BAZ1B, BCCIP, BIRC6, BRAT1, BRD4, BRMS1, BUB1B, CALU, CAMK1, CAMK2D, CASK, CASP3, CAV1, CBL, CCAR2, CCDC22, CCNB1, CCND2, CCNH, CCNL2, CCNY, CD44, CD81, CDC42, CDC42BPA, CDC42BPB, CDC73, CDH2, CDK5RAP1, CDK5RAP3, CDK7, CFAP20, CHD1, CHEK1, CHEK2, CKAP4, CLASP1, COG7, COPS2, COPS5, COPS7A, CORO1C, CRTAP, CSPG4, CTCF, CTNNB1, CUL4A, CUL4B, CUL7, CWC27, DAPK1, DAXX, DBNL, DCAF1, DCAF13, DDRGK1, DHPS, DIPK2A, DLG1, DNMT1, DNMT3A, DNMT3B, DOCK7, DPH1, DPH2, DPH6, DSP, DUSP3, ECT2, EDEM3, EGFR, EHD4, EHMT1, ELP1, ELP3, EPHB3, ERO1A, ETFA, EXOSC5, FBXO2,	No change

					FBXO30, FBXW8, FERMT2, FGG, FKBP10, FKBP11, FKBP14, FKBP15, FKBP4, FKBP5, FKBP7, FKBP8, FKBP9, FLT1, FN1, FOXK1, FTSJ3, FUT11, FXR1, GALNT1, GALNT10, GALNT2, GARS1, GATAD2A, GCLC, GFPT2, GLMN, GLUL, GNL3, GOLGA2, GPC3, GPD1L, GPHN, GPI, GTF3C4, GXYLT1, HAT1, HCFC1, HDAC2, HDAC4, HELLS, HERC2, HLA-A, HMBS, HSD17B10, HSP90AB1, HSP90B1, HSPA2, HSPA5, HSPB1, HTT, HUWE1, ILF3, ILK, INCENP, INO80C, IPO5, IQGAP1, IQGAP3, IRF2BPL, ITCH, ITGA5, IWS1, JADE1, JARID2, KAT7, KDM1A, KDM2A, KDM3B, KEAP1, KTN1, LAMB1, LAMC1, LCMT1, LGALS1, LMAN1, LNPEP, LRRC40, LRRK1, LTBP1, LYPLA2, MACROH2A1, MAGED1, MAN1A1, MAN1A2, MANBA, MAP2K6, MAPK1, MAPK14, MAPK8, MARCHF5, MASTL, MAVS, MBD3, MCM2, METAP1, METTL1, MFGE8, MGAT2, MICAL1, MMAB, MOGS, MTA3, MTMR14, MTMR6, MVD, MYADM, MYDGF, MYO1C, NDC1, NEDD4, NEK7, NIBAN1, NIBAN2, NMT2, NNT, NPM1, NRP2, NSUN2, NSUN5, NTHL1, NUDT16, NUP107, NUP133, NUP160, NUP210, NUP35, NUP50, NXN, OCLN, OGT, ORC3, OSBP, OSTC, OTUD6B, P3H1, P3H3, P3H4, P4HA1, P4HA2, P4HB, P4HTM, PAK1, PALD1, PARD3, PARN, PARP1, PARVA, PASK, PAXBP1, PDGFRB, PDIA6, PDZRN3, PEA15, PGAM5, PGM3, PHC1, PHIP, PHPT1, PIAS4, PIGS, PIGT, PIK3CA, PIK3R4, PLK1, PLOD1, PLOD2, PLOD3, PLPP3, PLXNB2, POFUT1, POFUT2, POGLUT2, POGLUT3, POLB, POR, PPIB, PPIC, PPID, PPM1B, PPM1F, PPM1G, PPP1R7, PPP1R9B, PPP2R5A, PPP4R1, PRDX4, PRKAA1, PRKAB1, PRKACB, PRKACG, PRKAG1, PRKAR1A,	

					PRKAR2A, PRKAR2B, PRKCA, PRKCSH, PRKDC, PRMT3, PRORP, PRRC1, PSMC6, PSMD1, PSMD2, PSMD9, PSME3, PTK7, PTPMT1, PTPN1, PTPN12, PTPN2, PTPRD, PUS1, PUS3, PUS7, PXK, PYCARD, RAB1A, RAB2A, RAB8A, RAD51, RANBP2, RANGAP1, RAP2A, RAP2B, RAP2C, RBBP5, RBM15, RBPMS, RCN1, RELA, RIF1, RIMKLB, RIOX1, RNF170, ROCK2, RPL5, RPN1, RPRD1A, RPS2, RPS6KA1, RPS6KA3, RPS6KA4, SART3, SBF1, SCYL1, SDF2L1, SEH1L, SEL1L, SEPHS1, SESN2, SET, SETD7, SETDB1, SIN3A, SIRT1, SIRT2, SKP2, SLK, SMARCAD1, SNX3, SNX6, SNX9, SORD, SPAG9, SPTBN1, SRC, SRPK1, SSB, STAM2, STK38, STT3A, STT3B, STYX, SUMF2, SUPT6H, SYMPK, TELO2, TF, TFB1M, TGM2, THBS1, THUMPD3, TIMM50, TIPRL, TLK1, TMED2, TMX3, TOLLIP, TPP2, TPST1, TPX2, TRIM28, TRIM33, TRIM71, TRIP12, TRMT1, TRMT10C, TRMT1L, TRMT5, TRPT1, TTK, TTLL12, TWF1, UBA6, UBE2G1, UBE2H, UBE2O, UBR4, UBR5, UFL1, UGGT1, UGGT2, UNG, USP15, USP19, USP47, USP5, USP7, USP9X, VLDLR, VPS25, VRK1, WARS1, XRCC5, XRCC6, YOD1, ZC3HAV1, ZDHHC17]	
chromosomal region	GO_CellularComponent-EBI-UniProt-GOA-ACAP-ARAP_13.05.2021_00h00	5.48E-33	21.98391	82	[ANTXR1, ATR, AURKB, BAZ1B, BUB1B, CBX5, CCNB1, CDC73, CDCA8, CHEK1, CHEK2, CKAP5, CLASP1, CTCF, DAXX, DCTN1, DCTN4, DNMT1, DNMT3A, DYNC1LI2, ERCC6L, HAT1, HELLS, INCENP, KAT7, KDM1A, KIF22, KIF2C, KNTC1, LRWD1, MACROH2A1, MCM2, MCM3, MCM4, MCM5, MCM6, MCM7, MMAB, MSH2, NCAPD2, NCAPG, NDC80, NUP107, NUP133, NUP160, ORC2, ORC3, ORC4, ORC5, PARP1, PDS5A, PLK1, POLD1, POLR1B, POLR2B, PPP2R5A, PRKDC, PURA, RAD51, RANGAP1, RCC2, RIF1, RPA1, RPA2, SEC13,	Upregulation

					SEH1L, SEPTIN2, SEPTIN7, SIN3A, SIRT2, SKA3, SPOUT1, SSB, SUN2, TELO2, TOP2A, TTK, WAPL, WRAP53, XRCC5, XRCC6, ZWILCH]	
chromosome	GO_CellularComponent-EBI-UniProt-GOA-ACAP-ARAP_13.05.2021_00h00	5.48E-33	10.34301	196	[ACTR2, ADD3, AEBP2, ANP32E, ANTXR1, ARPC1A, ATR, AURKB, BAZ1B, BMS1, BOP1, BPTF, BRD4, BRMS1, BUB1B, CAPN2, CBX2, CBX5, CCAR2, CCNB1, CCND2, CDC73, CDCA8, CHAF1A, CHD4, CHEK1, CHEK2, CHTF18, CKAP5, CLASP1, COPS5, CTCF, CTNNB1, DAXX, DCTN1, DCTN4, DDX18, DDX21, DDX6, DHX9, DNMT1, DNMT3A, DNTTIP2, DYNC1LI2, EHMT1, ERCC6L, EXD2, EXOSC10, EXOSC5, FANCD2, FH, FOXK1, FTSJ3, GARS1, GATA6, GATAD2A, GET4, GINS3, GNL3, HAT1, HDAC2, HELLS, HP1BP3, HSD17B4, HSPA2, INCENP, INO80C, IPO4, IRF3, IST1, JADE1, KAT7, KDM1A, KDM3B, KIF22, KIF2C, KNTC1, LRPPRC, LRWD1, MACROH2A1, MAGED1, MBD3, MCM2, MCM3, MCM4, MCM5, MCM6, MCM7, MDC1, MKI67, MLH1, MMAB, MPHOSPH10, MSH2, MSH6, MTA3, NCAPD2, NCAPG, NCAPG2, NCL, NDC80, NEDD4, NFKB1, NOL6, NSFL1C, NUP107, NUP133, NUP160, NUSAP1, ORC2, ORC3, ORC4, ORC5, P3H4, PAK1, PARP1, PAWR, PDS5A, PELP1, PES1, PLK1, POGZ, POLA1, POLA2, POLD1, POLE, POLR1B, POLR2A, POLR2B, PPP2R5A, PRIM1, PRIM2, PRKDC, PSIP1, PURA, RAD51, RAN, RANGAP1, RBPJ, RCC2, RELA, RFC1, RFC2, RFC3, RFC5, RIF1, RPA1, RPA2, RPA3, RPF2, RRS1, SAMHD1, SEC13, SEH1L, SEPTIN2, SEPTIN7, SET, SETD7, SETDB1, SIN3A, SIRT1, SIRT2, SKA3, SMARCA4, SMARCA5, SMARCAD1, SMARCC1, SMARCD1, SMARCD2, SMC2, SPOUT1, SRPK1, SSB, SSRP1, SUN2, SUPT16H, SUPT6H, TCP1, TELO2, TGM2, TOP2A, TRIM28, TRIP13, TTI1, TTK, UFL1, USP7, UTP4, VRTN, WAPL, WDHD1,	Upregulation

					WDR43, WRAP53, XRCC5, XRCC6, ZWILCH]	
DNA metabolic process	GO_BiologicalProcess-EBI-UniProt-GOA-ACAP-ARAP_13.05.2021_00h00	5.48E-33	15.18987	156	[ACTR2, AKT1, ANKRD28, ANTXR1, ANXA3, APOBEC3C, ATF7IP, ATR, AURKB, BAZ1B, BCCIP, CASP3, CCNH, CCT2, CCT3, CCT4, CCT5, CCT6A, CCT7, CCT8, CDK7, CHAF1A, CHEK1, CHEK2, CHTF18, COPS2, COPS5, COPS7A, CTCF, CTNNB1, CUL4A, CUL4B, DAXX, DCAF1, DDX21, DDX39B, DHX9, DICER1, DNMT1, DNMT3A, DNMT3B, EGFR, EHMT1, EMSY, ERCC2, EXD2, EXOG, EXOSC10, EXOSC5, FANCD2, FANCI, FH, FSCN1, GATAD2A, GINS3, GNL3, GTF2I, HELLS, HERC2, HSP90AB1, HSPD1, HUWE1, INO80C, KAT7, KDM2A, KIF22, LIG1, LIG3, LRWD1, MAP1S, MAPK1, MAPK8, MBD3, MCM2, MCM3, MCM4, MCM5, MCM6, MCM7, MDC1, MLH1, MMAB, MSH2, MSH3, MSH6, NEK7, NIBAN2, NPM1, NTHL1, NVL, ORC2, ORC3, ORC4, ORC5, PARN, PARP1, PDGFRB, PDS5A, PIAS4, PIK3CA, PLD3, POLA1, POLA2, POLB, POLD1, POLE, POLR1B, POLR2A, POLR2B, POLR2G, PRIM1, PRIM2, PRKDC, PURA, RAD51, RAN, RFC1, RFC2, RFC3, RFC5, RIF1, RPA1, RPA2, RPA3, SAMHD1, SESN2, SIRT1, SMARCA5, SMARCAD1, SRC, SSRP1, SUPT16H, SUPT6H, TCP1, TDP1, TDP2, TELO2, TIGAR, TOP2A, TRIM28, TRIP12, TRIP13, UBR5, UFL1, UNG, USP47, USP7, USP9X, VRTN, WDHD1, WDR18, WRAP53, XRCC5, XRCC6, XRN1, XRN2]	Upregulation
regulation of chromatin organization	GO_BiologicalProcess-EBI-UniProt-GOA-ACAP-ARAP_13.05.2021_00h00	5.48E-33	16.08696	37	[AASS, ATF7IP, BRD4, CAMK1, CAMK2D, CCNB1, CHEK1, CTCF, CTNNB1, DAXX, DNMT1, DNMT3B, IWS1, JARID2, KAT7, KDM1A, MACROH2A1, MAP1S, MAPK8, MKI67, OGT, PAXBP1, RIF1, RPS6KA4, SART3, SET, SETD7, SETDB1, SIN3A, SIRT1, SSRP1, SUPT6H, TLK1, TRIM28, TRIP12, TTLL12, UBR5]	Upregulation

cellular protein modification process	GO_BiologicalProcess-EBI-UniProt-GOA-ACAP-ARAP_13.05.2021_00h00	5.48E-33	9.878019	413	[AAAS, AASS, ABHD10, ABHD12, ABI1, ACSL1, ADAM10, ADAR, ADARB1, AGTPBP1, AIMP2, AKT1, ALG11, ALG2, ALG5, ALG9, ANTXR1, ANXA1, ANXA2, AP3B1, APOE, ARF4, ARFGEF1, ASPH, ATP2B4, ATR, AUP1, AURKA, AURKB, B3GLCT, B4GALT1, BAG2, BAZ1B, BCCIP, BIRC6, BRAT1, BRD4, BRMS1, BUB1B, CALU, CAMK1, CAMK2D, CASK, CASP3, CAV1, CBL, CCAR2, CCDC22, CCNB1, CCND2, CCNH, CCNL2, CCNY, CD44, CD81, CDC42, CDC42BPA, CDC42BPB, CDC73, CDH2, CDK5RAP1, CDK5RAP3, CDK7, CFAP20, CHEK1, CHEK2, CKAP4, CLASP1, COG7, COPS2, COPS5, COPS7A, CORO1C, CRTAP, CSPG4, CTCF, CTNNB1, CUL4A, CUL4B, CUL7, CWC27, DAPK1, DAXX, DBNL, DCAF1, DCAF13, DDRGK1, DHPS, DIPK2A, DLG1, DNMT1, DNMT3B, DOCK7, DPH1, DPH2, DPH6, DSP, DUSP3, ECT2, EDEM3, EGFR, EHD4, EHMT1, ELP3, EPHB3, ERO1A, ETFA, FBXO2, FBXO30, FBXW8, FERMT2, FGG, FKBP10, FKBP11, FKBP14, FKBP15, FKBP4, FKBP5, FKBP7, FKBP8, FKBP9, FLT1, FN1, FOXK1, FUT11, FXR1, GALNT1, GALNT10, GALNT2, GARS1, GCLC, GFPT2, GLMN, GLUL, GNL3, GOLGA2, GPC3, GPD1L, GPHN, GPI, GTF3C4, GXYLT1, HAT1, HCFC1, HDAC2, HDAC4, HERC2, HLA-A, HMBS, HSP90AB1, HSP90B1, HSPA2, HSPA5, HSPB1, HTT, HUWE1, ILF3, ILK, INCENP, INO80C, IPO5, IQGAP1, IQGAP3, IRF2BPL, ITCH, ITGA5, IWS1, JADE1, JARID2, KAT7, KDM1A, KDM2A, KDM3B, KEAP1, KTN1, LAMB1, LAMC1, LCMT1, LGALS1, LMAN1, LNPEP, LRRC40, LRRK1, LTBP1, LYPLA2, MACROH2A1, MAGED1, MAN1A1, MAN1A2, MANBA, MAP2K6, MAPK1, MAPK14, MAPK8, MARCHF5, MASTL, MAVS, MBD3, MCM2,	No change

					METAP1, MFGE8, MGAT2, MICAL1, MMAB, MOGS, MTA3, MTMR14, MTMR6, MVD, MYADM, MYDGF, MYO1C, NDC1, NEDD4, NEK7, NIBAN1, NMT2, NNT, NPM1, NRP2, NUP107, NUP133, NUP160, NUP210, NUP35, NUP50, NXN, OCLN, OGT, ORC3, OSBP, OSTC, OTUD6B, P3H1, P3H3, P3H4, P4HA1, P4HA2, P4HB, P4HTM, PAK1, PALD1, PARD3, PARP1, PARVA, PASK, PAXBP1, PDGFRB, PDIA6, PDZRN3, PEA15, PGAM5, PGM3, PHC1, PHIP, PHPT1, PIAS4, PIGS, PIGT, PIK3CA, PIK3R4, PLK1, PLOD1, PLOD2, PLOD3, PLPP3, PLXNB2, POFUT1, POFUT2, POGLUT2, POGLUT3, POLB, POR, PPIB, PPIC, PPID, PPM1B, PPM1F, PPM1G, PPP1R7, PPP1R9B, PPP2R5A, PPP4R1, PRDX4, PRKAA1, PRKAB1, PRKACB, PRKACG, PRKAG1, PRKAR1A, PRKAR2A, PRKAR2B, PRKCA, PRKCSH, PRKDC, PRMT3, PRRC1, PSMC6, PSMD1, PSMD2, PSMD9, PSME3, PTK7, PTPMT1, PTPN1, PTPN12, PTPN2, PTPRD, PXK, PYCARD, RAB1A, RAB2A, RAB8A, RAD51, RANBP2, RANGAP1, RAP2A, RAP2B, RAP2C, RBBP5, RBPMS, RCN1, RELA, RIF1, RIMKLB, RIOX1, RNF170, ROCK2, RPL5, RPN1, RPRD1A, RPS2, RPS6KA1, RPS6KA3, RPS6KA4, SART3, SBF1, SCYL1, SDF2L1, SEH1L, SEL1L, SEPHS1, SESN2, SET, SETD7, SETDB1, SIN3A, SIRT1, SIRT2, SKP2, SLK, SMARCAD1, SNX3, SNX6, SNX9, SORD, SPAG9, SPTBN1, SRC, SRPK1, STAM2, STK38, STT3A, STT3B, STYX, SUMF2, SUPT6H, SYMPK, TELO2, TF, TGM2, THBS1, TIMM50, TIPRL, TLK1, TMED2, TMX3, TOLLIP, TPP2, TPST1, TPX2, TRIM28, TRIM33, TRIM71, TRIP12, TRPT1, TTK, TTLL12, TWF1, UBA6, UBE2G1, UBE2H, UBE2O, UBR4, UBR5, UFL1, UGGT1, UGGT2, USP15, USP19, USP47,	

					USP5, USP7, USP9X, VLDLR, VPS25, VRK1, WARS1, XRCC5, XRCC6, YOD1, ZC3HAV1, ZDHHC17]	
regulation of chromosome organization	GO_BiologicalProcess-EBI-UniProt-GOA-ACAP-ARAP_13.05.2021_00h00	5.48E-33	17.35016	55	[ANTXR1, ATF7IP, ATR, AURKB, BRD4, BUB1B, CCNB1, CCT2, CCT3, CCT4, CCT5, CCT6A, CCT7, CCT8, CTCF, CTNNB1, DAXX, DLGAP5, DNMT1, DNMT3B, EXOSC10, GNL3, JARID2, KAT7, KNTC1, MACROH2A1, MAPK1, MCM2, MCM7, MMAB, NDC80, NEK7, OGT, PARN, PARP1, PAXBP1, PLK1, RIF1, RPS6KA4, SART3, SETDB1, SIN3A, SIRT1, SRC, SSBP1, TACC3, TCP1, TOP2A, TRIM28, TRIP13, TTK, USP7, WAPL, XRCC5, XRN1]	Upregulation
covalent chromatin modification	GO_BiologicalProcess-EBI-UniProt-GOA-ACAP-ARAP_13.05.2021_00h00	5.48E-33	13.24111	67	[AASS, ATF7IP, AURKA, AURKB, BAZ1B, BRD4, BRMS1, CAMK1, CAMK2D, CCNB1, CDC73, CHD1, CHEK1, CTCF, CTNNB1, CUL4B, DCAF1, DNMT1, DNMT3A, DNMT3B, EHMT1, GTF3C4, HAT1, HCFC1, HDAC2, HDAC4, HELLS, HUWE1, INCENP, IWS1, JADE1, JARID2, KAT7, KDM1A, KDM2A, KDM3B, MACROH2A1, MAPK8, MBD3, MTA3, OGT, PAXBP1, PHC1, PPM1F, PRKAA1, PRKCA, RBBP5, RIF1, RIOX1, RPS6KA4, SART3, SET, SETD7, SETDB1, SIN3A, SIRT1, SIRT2, SMARCAD1, SUPT6H, TRIM28, TRIP12, TTLL12, UBR5, UFL1, USP15, USP7, VRK1]	Upregulation
positive regulation of chromosome organization	GO_BiologicalProcess-EBI-UniProt-GOA-ACAP-ARAP_13.05.2021_00h00	5.48E-33	18.04878	37	[ANTXR1, ATF7IP, ATR, AURKB, BRD4, CCNB1, CCT2, CCT3, CCT4, CCT5, CCT6A, CCT7, CCT8, CTNNB1, DAXX, DNMT1, DNMT3B, GNL3, JARID2, KAT7, MACROH2A1, MAPK1, MMAB, NEK7, OGT, PARN, PAXBP1, RIF1, RPS6KA4, SART3, SETDB1, SIN3A, SIRT1, SSBP1, TCP1, TRIM28, XRCC5]	Upregulation
peptidyl-amino acid modification	GO_BiologicalProcess-EBI-UniProt-GOA-ACAP-ARAP_13.05.2021_00h00	5.48E-33	12.44541	171	[AAAS, ABI1, AGTPBP1, AKT1, ALG5, ANTXR1, ASPH, ATP2B4, ATR, AURKA, AURKB, BAZ1B, BRD4, CAMK1, CAMK2D, CAV1, CBL, CCNB1, CD44, CD81, CDC42BPA, CDC42BPB, CFAP20, CHEK1, CHEK2, CLASP1, CRTAP, CSPG4,	No change

					CTCF, CTNNB1, CWC27, DCAF1, DHPS, DNMT1, DNMT3B, DOCK7, DPH1, DPH2, DPH6, EGFR, EHD4, EHMT1, EPHB3, ERO1A, FKBP10, FKBP11, FKBP14, FKBP15, FKBP4, FKBP5, FKBP7, FKBP8, FKBP9, FLT1, GALNT1, GALNT2, GNL3, GPD1L, GPI, GTF3C4, HAT1, HCFC1, HDAC2, HDAC4, HMBS, HSP90AB1, ILK, IQGAP1, ITGA5, IWS1, JADE1, JARID2, KAT7, KDM1A, LMAN1, LRRK1, MACROH2A1, MAP2K6, MAPK1, MAPK14, MAPK8, MASTL, MBD3, METAP1, MGAT2, MMAB, NDC1, NMT2, NRP2, NUP107, NUP133, NUP160, NUP210, NUP35, NUP50, OGT, OSBP, OSTC, P3H1, P3H3, P3H4, P4HA1, P4HA2, P4HB, P4HTM, PAK1, PARD3, PARP1, PDGFRB, PIAS4, PIK3CA, PLK1, PLOD1, PLOD2, PLOD3, POGLUT2, POGLUT3, POR, PPIB, PPIC, PPID, PPM1F, PRDX4, PRKAA1, PRKCA, PRKDC, PRMT3, PSMD2, PTPN1, PTPN2, RANBP2, RANGAP1, RAP2B, RAP2C, RBBP5, RELA, RIF1, ROCK2, RPN1, RPS6KA1, RPS6KA3, RPS6KA4, SCYL1, SEH1L, SET, SETD7, SETDB1, SIN3A, SIRT1, SIRT2, SNX6, SRC, STK38, STT3A, STT3B, SUPT6H, TELO2, TGM2, TLK1, TMX3, TOLLIP, TPST1, TRIM28, TTK, TTLL12, TWF1, UGGT1, UGGT2, VPS25, VRK1, ZDHHC17]	
regulation of histone modification	GO_BiologicalProcess-EBI-UniProt-GOA-ACAP-ARAP_13.05.2021_00h00	5.48E-33	17.90123	29	[AASS, BRD4, CAMK1, CAMK2D, CCNB1, CHEK1, CTCF, CTNNB1, DNMT1, DNMT3B, IWS1, JARID2, KAT7, KDM1A, MACROH2A1, MAPK8, OGT, PAXBP1, RIF1, RPS6KA4, SART3, SET, SETD7, SIN3A, SIRT1, SUPT6H, TRIP12, TTLL12, UBR5]	Upregulation
peptidyl-lysine modification	GO_BiologicalProcess-EBI-UniProt-GOA-ACAP-ARAP_13.05.2021_00h00	5.48E-33	13.61502	58	[AAAS, BRD4, CAMK1, CHEK1, CTCF, CTNNB1, DHPS, DNMT1, DNMT3B, EHMT1, FKBP10, GNL3, GTF3C4, HAT1, HCFC1, HDAC2, HDAC4, IWS1, JADE1, JARID2, KAT7, KDM1A, MACROH2A1, MBD3, NDC1, NUP107, NUP133, NUP160,	Upregulation

					NUP210, NUP35, NUP50, OGT, P3H3, P3H4, PIAS4, PLOD1, PLOD2, PLOD3, POR, PRKAA1, RANBP2, RANGAP1, RBBP5, RELA, RIF1, RPS6KA4, SEH1L, SET, SETD7, SETDB1, SIN3A, SIRT1, SIRT2, SUPT6H, TGM2, TOLLIP, TRIM28, TTLL12]	
regulation of histone methylation	GO_BiologicalProcess-EBI-UniProt-GOA-ACAP-ARAP_13.05.2021_00h00	5.48E-33	23.61111	17	[AASS, BRD4, CTCF, CTNNB1, DNMT1, DNMT3B, IWS1, JARID2, KDM1A, MACROH2A1, OGT, PAXBP1, RIF1, SETD7, SIRT1, SUPT6H, TTLL12]	Upregulation
ligase activity, forming carbon-nitrogen bonds	GO_MolecularFunction-EBI-UniProt-GOA-ACAP-ARAP_13.05.2021_00h00	2.07E-22	26.31579	15	[ASNS, CTPS1, DPH6, GART, GCLC, GCLM, GLUL, GMPS, GSS, MTHFD1, PAICS, PFAS, QRSL1, RIMKLB, TTLL12]	Upregulation
small molecule catabolic process	GO_BiologicalProcess-EBI-UniProt-GOA-ACAP-ARAP_13.05.2021_00h00	2.07E-22	15.20833	73	[AASS, ABAT, ACAA1, ACAD8, ACAT1, ACAT2, ACOX3, AKT1, ALDH1L2, ALDH2, ALDH7A1, ALDOC, AMDHD2, APOBEC3C, APOE, ATP2B4, BCAT2, BDH2, BPNT2, CARNMT1, DCXR, DECR1, ECHDC1, ENO2, ESD, ETFA, FOXK1, GALE, GALK1, GALM, GCDH, GLS, GLUD1, GLUL, GNPDA2, GOLT1B, GOT2, GPI, HACL1, HADHA, HADHB, HAGH, HIBADH, HK1, HK2, HMGCL, HOOK3, HSD17B10, HSD17B4, ILVBL, MCCC2, MTAP, NQO2, NUDT16, OXCT1, PCCA, PCCB, PFKL, PFKM, PFKP, PGM2L1, PKM, PM20D2, PNP, QDPR, SBDS, SCLY, SESN2, SLC25A12, SNX17, SORD, TIGAR, TKFC]	Downregulation
small molecule biosynthetic process	GO_BiologicalProcess-EBI-UniProt-GOA-ACAP-ARAP_13.05.2021_00h00	2.07E-22	13.36088	97	[AASS, ABAT, ACACA, ACAT1, ACAT2, ACLY, ACSS2, ACSS3, ADSL, ALDOC, ANXA1, APOE, APRT, ASL, ASNS, ATP2B4, BCAT2, BDH2, CBR1, CYB5R3, CYP51A1, DAGLB, DEGS1, DHX8, ENO2, ENOPH1, ERLIN2, FDPS, FDXR, GLS, GLUD1, GLUL, GOLT1B, GOT2, GPI, GSTM2, H6PD, HMGCL, HSD17B10, HSD17B4, IDI1, ILVBL, LBR, LPGAT1, LSS, MOXD1, MTAP,	Downregulation

					MTHFD1, MTR, MVD, MVK, NFKB1, NLN, NSDHL, NT5C2, NTHL1, NUDT16, OGT, OSBP, PC, PCCB, PDXK, PLOD2, PLOD3, PNP, POR, PRKAA1, PRKAB1, PRKAG1, PTGIS, PTPN2, PYCR1, PYCR2, PYCR3, QDPR, RAN, RANBP2, RBP1, RDH10, SAMHD1, SARM1, SBDS, SEPHS1, SESN2, SIRT1, SLC1A3, SLC25A12, SORD, SPR, SPTLC2, SRR, TELO2, TKT, UGDH, UGP2, UNG, USP7]	
organic acid biosynthetic process	GO_BiologicalProcess-EBI-UniProt-GOA-ACAP-ARAP_13.05.2021_00h00	2.07E-22	14.32665	50	[AASS, ABAT, ACACA, ACLY, ACSS2, ADSL, ANXA1, ASL, ASNS, ATP2B4, BCAT2, CBR1, DAGLB, DEGS1, ENOPH1, ERLIN2, GLS, GLUD1, GLUL, GOLT1B, GOT2, GSTM2, HSD17B10, HSD17B4, ILVBL, LPGAT1, MTAP, MTHFD1, MTR, OSBP, PCCB, PLOD2, PLOD3, PRKAA1, PRKAB1, PRKAG1, PTGIS, PYCR1, PYCR2, PYCR3, RBP1, RDH10, SBDS, SEPHS1, SIRT1, SLC1A3, SLC25A12, SRR, UGDH, UGP2]	Downregulation
organic acid catabolic process	GO_BiologicalProcess-EBI-UniProt-GOA-ACAP-ARAP_13.05.2021_00h00	2.07E-22	14.98258	43	[AASS, ABAT, ACAA1, ACAD8, ACAT1, ACAT2, ACOX3, AKT1, ALDH1L2, ALDH7A1, AMDHD2, ATP2B4, BCAT2, BDH2, CARNMT1, DCXR, DECR1, ECHDC1, ETFA, GCDH, GLS, GLUD1, GLUL, GNPDA2, GOLT1B, GOT2, HACL1, HADHA, HADHB, HIBADH, HMGCL, HSD17B10, HSD17B4, ILVBL, MCCC2, PCCA, PCCB, PM20D2, QDPR, SBDS, SCLY, SESN2, SORD]	Downregulation
oxoacid metabolic process	GO_BiologicalProcess-EBI-UniProt-GOA-ACAP-ARAP_13.05.2021_00h00	2.07E-22	14.87676	169	[AAAS, AASS, ABAT, ABCC1, ABHD10, ABHD14B, ACAA1, ACACA, ACAD8, ACAD9, ACAT1, ACAT2, ACLY, ACO1, ACOX3, ACSL1, ACSL4, ACSS2, ADPGK, ADSL, AIMP2, AKT1, ALDH1L2, ALDH7A1, ALDOC, AMDHD2, ANXA1, APPL2, ASL, ASNS, ATIC, ATP2B4, BCAT2, BDH2, BPNT2, CARNMT1, CAV1, CBR1, CTPS1, CYB5R3, CYP2S1, DAGLB, DARS2, DCXR, DECR1, DEGS1, ECHDC1, EEF1E1, ENO2, ENOPH1, ERLIN2, ETFA, FH, FOXK1, GALK1, GARS1, GART, GCDH, GCLC, GCLM, GFPT2,	No change

					GLS, GLUD1, GLUL, GMPS, GNE, GNPDA2, GOLT1B, GOT2, GPI, GSS, GSTM2, HACL1, HADHA, HADHB, HAGH, HDAC4, HIBADH, HK1, HK2, HMGCL, HOOK3, HSD17B10, HSD17B4, IDH2, ILVBL, L2HGDH, LARS1, LARS2, LDHB, LPGAT1, LYPLA2, MAPK14, MARS1, MCCC2, ME1, MPC2, MTAP, MTHFD1, MTR, NDC1, NIT2, NUP107, NUP133, NUP160, NUP210, NUP35, NUP50, OGT, OSBP, PAPSS2, PC, PCCA, PCCB, PDXDC1, PFAS, PFKL, PFKM, PFKP, PKM, PLOD2, PLOD3, PM20D2, PON2, POR, PRKAA1, PRKAB1, PRKAG1, PRKAR2B, PRUNE1, PSMC6, PSMD1, PSMD2, PSMD9, PSME3, PTGIS, PYCR1, PYCR2, PYCR3, QDPR, QRSL1, RANBP2, RBP1, RDH10, RIMKLB, SARS1, SARS2, SBDS, SCLY, SCPEP1, SEC13, SEH1L, SEPHS1, SESN2, SIRT1, SLC1A3, SLC25A12, SLC25A32, SLC2A1, SMS, SORD, SRR, TARS2, TIGAR, TPST1, UGDH, UGP2, WARS1, YARS2]	
nucleobase-containing small molecule metabolic process	GO_BiologicalProcess-EBI-UniProt-GOA-ACAP-ARAP_13.05.2021_00h00	2.07E-22	16.14815	109	[AAAS, AASS, ABHD14B, ACACA, ACAT1, ACLY, ACOT13, ACOT9, ACSL1, ACSL4, ACSS2, ADPGK, ADSL, AK1, AK4, ALDOC, AMDHD2, AMPD2, APOBEC3C, APRT, ATIC, ATP6V1A, BPNT2, CASK, CMPK1, CRMP1, CTPS1, DCTD, DLG1, DPYSL2, DPYSL3, EFL1, ENO2, FLAD1, FOXK1, GALK1, GARS1, GART, GCDH, GFPT2, GMPPB, GMPS, GNE, GNPDA2, GPI, GTPBP1, HDAC4, HK1, HK2, HMGCL, HOOK3, HSD17B4, IDH2, IMPDH1, IMPDH2, KDM1A, MAPK1, MCCC2, ME1, MPC2, MTAP, MTHFD1, MVD, MVK, NDC1, NT5C2, NTHL1, NUDT16, NUP107, NUP133, NUP160, NUP210, NUP35, NUP50, OGT, PAICS, PAPSS2, PARP1, PFAS, PFKL, PFKM, PFKP, PGM3, PKM, PNP, PRKAA1, PRKAG1, PRPSAP1, PTGIS, RAB23, RAN, RANBP2, SAMHD1, SARM1, SEC13, SEH1L,	No change

					SLC25A12, SORD, TBPL1, TIGAR, TJP2, TPST1, TXNRD1, UAP1, UGDH, UGGT1, UGGT2, UGP2, UNG]	
cellular amino acid metabolic process	GO_BiologicalProcess-EBI-UniProt-GOA-ACAP-ARAP_13.05.2021_00h00	2.07E-22	18.0593	67	[AASS, ABAT, ACAD8, ACAT1, ADSL, AIMP2, ALDH7A1, ASL, ASNS, ATP2B4, BCAT2, CARNMT1, CTPS1, DARS2, EEF1E1, ENOPH1, GARS1, GART, GCDH, GCLC, GCLM, GFPT2, GLS, GLUD1, GLUL, GMPS, GOLT1B, GOT2, GSS, HIBADH, HMGCL, HSD17B10, ILVBL, LARS1, LARS2, MARS1, MCCC2, MTAP, MTHFD1, MTR, NIT2, PFAS, PLOD2, PLOD3, PSMC6, PSMD1, PSMD2, PSMD9, PSME3, PYCR1, PYCR2, PYCR3, QDPR, QRSL1, RIMKLB, SARS1, SARS2, SBDS, SCLY, SEPHS1, SLC1A3, SLC25A12, SMS, SRR, TARS2, WARS1, YARS2]	No change
carboxylic acid metabolic process	GO_BiologicalProcess-EBI-UniProt-GOA-ACAP-ARAP_13.05.2021_00h00	2.07E-22	14.86854	164	[AAAS, AASS, ABAT, ABCC1, ABHD10, ACAA1, ACACA, ACAD8, ACAD9, ACAT1, ACAT2, ACLY, ACO1, ACOX3, ACSL1, ACSL4, ACSS2, ADPGK, ADSL, AIMP2, AKT1, ALDH1L2, ALDH7A1, ALDOC, AMDHD2, ANXA1, APPL2, ASL, ASNS, ATIC, ATP2B4, BCAT2, BDH2, CARNMT1, CAV1, CBR1, CTPS1, CYB5R3, CYP2S1, DAGLB, DARS2, DCXR, DECR1, DEGS1, ECHDC1, EEF1E1, ENO2, ENOPH1, ERLIN2, ETFA, FH, FOXK1, GALK1, GARS1, GART, GCDH, GCLC, GCLM, GFPT2, GLS, GLUD1, GLUL, GMPS, GNE, GNPDA2, GOLT1B, GOT2, GPI, GSS, GSTM2, HACL1, HADHA, HADHB, HAGH, HDAC4, HIBADH, HK1, HK2, HMGCL, HOOK3, HSD17B10, HSD17B4, IDH2, ILVBL, L2HGDH, LARS1, LARS2, LDHB, LPGAT1, LYPLA2, MAPK14, MARS1, MCCC2, ME1, MPC2, MTAP, MTHFD1, MTR, NDC1, NIT2, NUP107, NUP133, NUP160, NUP210, NUP35, NUP50, OGT, OSBP, PC, PCCA, PCCB, PDXDC1, PFAS, PFKL, PFKM, PFKP, PKM, PLOD2, PLOD3, PM20D2, PON2, POR, PRKAA1, PRKAB1, PRKAG1, PRKAR2B,	No change

					PSMC6, PSMD1, PSMD2, PSMD9, PSME3, PTGIS, PYCR1, PYCR2, PYCR3, QDPR, QRSL1, RANBP2, RBP1, RDH10, RIMKLB, SARS1, SARS2, SBDS, SCLY, SCPEP1, SEC13, SEH1L, SEPHS1, SESN2, SIRT1, SLC1A3, SLC25A12, SLC25A32, SLC2A1, SMS, SORD, SRR, TARS2, TIGAR, UGDH, UGP2, WARS1, YARS2]	
alpha-amino acid metabolic process	GO_BiologicalProcess-EBI-UniProt-GOA-ACAP-ARAP_13.05.2021_00h00	2.07E-22	18.10345	42	[AASS, ACAT1, ADSL, ALDH7A1, ASL, ASNS, ATP2B4, BCAT2, CTPS1, ENOPH1, GART, GCDH, GCLC, GCLM, GFPT2, GLS, GLUD1, GLUL, GMPS, GOLT1B, GOT2, HSD17B10, ILVBL, MTAP, MTHFD1, MTR, NIT2, PFAS, PLOD2, PLOD3, PYCR1, PYCR2, PYCR3, QDPR, RIMKLB, SARS1, SBDS, SCLY, SEPHS1, SLC25A12, SMS, SRR]	No change
cellular amino acid biosynthetic process	GO_BiologicalProcess-EBI-UniProt-GOA-ACAP-ARAP_13.05.2021_00h00	2.07E-22	32.87671	24	[AASS, ABAT, ADSL, ASL, ASNS, BCAT2, ENOPH1, GLS, GLUD1, GLUL, GOLT1B, GOT2, ILVBL, MTAP, MTHFD1, MTR, PLOD2, PLOD3, PYCR1, PYCR2, PYCR3, SLC1A3, SLC25A12, SRR]	Downregulation
carboxylic acid biosynthetic process	GO_BiologicalProcess-EBI-UniProt-GOA-ACAP-ARAP_13.05.2021_00h00	2.07E-22	14.28571	48	[AASS, ABAT, ACACA, ACLY, ACSS2, ADSL, ANXA1, ASL, ASNS, BCAT2, CBR1, DAGLB, DEGS1, ENOPH1, ERLIN2, GLS, GLUD1, GLUL, GOLT1B, GOT2, GSTM2, HSD17B10, HSD17B4, ILVBL, LPGAT1, MTAP, MTHFD1, MTR, OSBP, PCCB, PLOD2, PLOD3, PRKAA1, PRKAB1, PRKAG1, PTGIS, PYCR1, PYCR2, PYCR3, RBP1, RDH10, SBDS, SIRT1, SLC1A3, SLC25A12, SRR, UGDH, UGP2]	Downregulation
carboxylic acid catabolic process	GO_BiologicalProcess-EBI-UniProt-GOA-ACAP-ARAP_13.05.2021_00h00	2.07E-22	15.18519	41	[AASS, ABAT, ACAA1, ACAD8, ACAT1, ACAT2, ACOX3, AKT1, ALDH1L2, ALDH7A1, AMDHD2, ATP2B4, BDH2, CARNMT1, DCXR, DECR1, ECHDC1, ETFA, GCDH, GLS, GLUD1, GLUL, GNPDA2, GOLT1B, GOT2, HACL1, HADHA, HADHB, HIBADH, HMGCL, HSD17B10, HSD17B4, ILVBL, MCCC2, PCCA, PCCB, PM20D2, QDPR, SBDS, SESN2, SORD]	Downregulation
monocarboxylic acid metabolic process	GO_BiologicalProcess-EBI-	2.07E-22	12.93952	92	[AAAS, ABAT, ABHD10, ACAA1, ACACA, ACAD9, ACAT1, ACAT2, ACLY,	No change

	UniProt-GOA-ACAP-ARAP_13.05.2021_00h00				ACOX3, ACSL1, ACSL4, ACSS2, ADPGK, AKT1, ALDOC, ANXA1, APPL2, BDH2, CAV1, CBR1, CYP2S1, DAGLB, DCXR, DECR1, DEGS1, ECHDC1, ENO2, ERLIN2, ETFA, FOXK1, GALK1, GCDH, GOT2, GPI, GSTM2, HACL1, HADHA, HADHB, HAGH, HDAC4, HK1, HK2, HOOK3, HSD17B10, HSD17B4, IDH2, ILVBL, LDHB, LPGAT1, LYPLA2, MAPK14, MCCC2, ME1, MPC2, NDC1, NUP107, NUP133, NUP160, NUP210, NUP35, NUP50, OGT, OSBP, PC, PCCA, PCCB, PFKL, PFKM, PFKP, PKM, PON2, POR, PRKAA1, PRKAB1, PRKAG1, PRKAR2B, PTGIS, RANBP2, RBP1, RDH10, SBDS, SCPEP1, SEC13, SEH1L, SESN2, SIRT1, SLC1A3, SLC25A12, SORD, SRR, TIGAR]	
glutamine family amino acid metabolic process	GO_BiologicalProcess-EBI-UniProt-GOA-ACAP-ARAP_13.05.2021_00h00	2.07E-22	21.875	21	[ADSL, ASL, ASNS, ATP2B4, CTPS1, GCLC, GCLM, GFPT2, GLS, GLUD1, GLUL, GMPS, GOLT1B, GOT2, NIT2, PFAS, PYCR1, PYCR2, PYCR3, RIMKLB, SLC25A12]	No change
aspartate family amino acid metabolic process	GO_BiologicalProcess-EBI-UniProt-GOA-ACAP-ARAP_13.05.2021_00h00	2.07E-22	24.61539	16	[AASS, ALDH7A1, ASNS, ENOPH1, GCDH, GOLT1B, GOT2, MTAP, MTHFD1, MTR, NIT2, PLOD2, PLOD3, SBDS, SLC25A12, SMS]	No change
alpha-amino acid biosynthetic process	GO_BiologicalProcess-EBI-UniProt-GOA-ACAP-ARAP_13.05.2021_00h00	2.07E-22	30.76923	24	[AASS, ADSL, ASL, ASNS, ATP2B4, ENOPH1, GLS, GLUD1, GLUL, GOLT1B, GOT2, ILVBL, MTAP, MTHFD1, MTR, PLOD2, PLOD3, PYCR1, PYCR2, PYCR3, SBDS, SEPHS1, SLC25A12, SRR]	Downregulation
aspartate family amino acid biosynthetic process	GO_BiologicalProcess-EBI-UniProt-GOA-ACAP-ARAP_13.05.2021_00h00	2.07E-22	41.66667	10	[AASS, ASNS, ENOPH1, GOLT1B, GOT2, MTAP, MTHFD1, MTR, PLOD2, PLOD3]	No change
glutamine family amino acid biosynthetic process	GO_BiologicalProcess-EBI-UniProt-GOA-ACAP-ARAP_13.05.2021_00h00	2.07E-22	47.36842	9	[ADSL, ASL, GLS, GLUD1, GLUL, PYCR1, PYCR2, PYCR3, SLC25A12]	Downregulation

anatomical structure morphogenesis	GO_BiologicalProcess-EBI-UniProt-GOA-ACAP-ARAP_13.05.2021_00h00	2.04E-13	9.71489	276	[ABI1, ACTBL2, ACTN1, ACTN4, ACTR2, ADAM10, ADARB1, AGO1, AGO2, AGTPBP1, AKT1, ALCAM, ALPL, ANK2, ANK3, ANTXR1, ANXA1, ANXA2, ANXA3, ANXA6, AP2A1, AP2A2, AP2B1, AP2M1, AP2S1, AP3B1, APOE, ARAP1, ARHGDIA, ATP2B4, AURKA, B4GALT1, BAIAP2, BPNT2, BRWD1, C1QBP, CALD1, CALR, CAMK1, CAP1, CAP2, CAPN1, CAPN2, CAPRIN1, CASP3, CAV1, CD44, CD81, CDC42, CDC42EP1, CDC42EP3, CDC73, CDH2, CFL2, CLASP1, CNRIP1, COL1A1, COL1A2, COL4A1, COL4A2, COL5A1, COL5A2, COL6A1, CORO1C, CRMP1, CSPG4, CSRP2, CTNNB1, CUL7, CYFIP1, DBNL, DIAPH1, DICER1, DLG1, DOCK7, DPYSL2, DSP, ECE1, ECT2, EFNB2, EGFR, EHD1, EHD2, EIF4A3, EPB41, EPB41L3, EPB41L5, EPHB3, EPN2, EPS8, ERAP1, ERCC2, F11R, FARP1, FAT1, FBN2, FBXW8, FERMT2, FGG, FHL1, FKBP10, FLNB, FLT1, FN1, GAB1, GARS1, GATA6, GDI1, GLMN, GLUL, GPC1, GPC3, GPC6, GPI, HDAC2, HK2, HMOX1, HSP90AB1, HSPA5, HSPB1, HTRA1, HUWE1, IGF2BP3, ILK, IQGAP1, IST1, ITGA5, ITGA6, ITGAV, ITGB1, JAM3, KIF5B, KRT18, KRT19, KRT8, LAMB1, LAMC1, LIG1, LIMS1, LNPK, LRP1, LYPLA2, MACF1, MACROH2A1, MAGED1, MAP1B, MAP1S, MAPK1, MAPK14, MARCHF5, MBNL1, MCAM, MCU, METAP1, MFGE8, MSN, MTDH, MTHFD1, MYADM, MYDGF, MYH10, MYH9, MYO1E, NCAM1, NCKAP1, NCL, NECTIN2, NEDD4, NIBAN2, NLE1, NOL6, NRP2, P4HB, PAK1, PALLD, PARD3, PARD6B, PARVA, PDCL3, PDGFRB, PDLIM5, PDLIM7, PHIP, PICALM, PIK3C2A, PIK3CA, PKM, PKP2, PLCG1, PLOD3, PLXNB2, PNPT1, POFUT1, POFUT2, POLB, POLR1B, POR, PRKACB, PRKAR1A, PRKCA,	Downregulation

					PRKDC, PRPF40A, PSMC6, PSMD1, PSMD2, PSMD9, PSME3, PTGIS, PTK7, PTPRD, PXDN, PXMP2, RAB1A, RAB23, RAB8A, RAC3, RAP2A, RBM15, RBPJ, RBPMS2, RCC2, RDH10, RDX, RELA, RIC8A, RNH1, ROCK2, RP2, RPS6KA1, RRAS, RRP7A, S100A10, SARM1, SARS1, SART3, SCFD1, SEC24B, SEPTIN7, SERPINH1, SIN3A, SIRT1, SLC1A3, SLIRP, SMARCC1, SORD, SPAG9, SPARC, SPTAN1, SPTBN1, SPTBN2, SRC, SSBP1, STIM1, SUPT6H, TBPL1, TENM3, TGFB1I1, TGM2, THBS1, TMED2, TMF1, TPM1, TRIM28, TRIM71, TXNRD1, UGDH, USP9X, VAMP3, VAT1, VLDLR, VPS35, WARS1, YAP1, ZDHHC17, ZMYM3, ZNF281]	
cell-substrate adhesion	GO_BiologicalProcess-EBI-UniProt-GOA-ACAP-ARAP_13.05.2021_00h00	2.04E-13	13.91076	53	[ACTN1, ACTN4, ANTXR1, ARL2, BCAT2, C1QBP, CALR, CASK, CD44, CDC42, CLASP1, COL1A1, CORO1C, CTNNB1, DUSP3, EGFL6, EPB41L5, EPHB3, FERMT2, FGG, FN1, ILK, IQGAP1, ITGA5, ITGA6, ITGAV, ITGB1, JAM3, LAMB1, LAMC1, LGALS1, LIMS1, LRP1, MACF1, MYADM, NID1, P4HB, PARVA, PPM1F, PXDN, RAB1A, RAC3, RCC2, ROCK2, RRAS, S100A10, SLK, SORBS1, SORBS3, SRC, THBS1, UTRN, VAMP3]	Downregulation
anchoring junction	GO_CellularComponent-EBI-UniProt-GOA-ACAP-ARAP_13.05.2021_00h00	2.04E-13	19.59687	175	[ACTN1, ACTN4, ACTR2, ADAM10, ADD3, AKT1, ALCAM, ANK2, ANK3, ANXA1, ANXA2, ANXA5, ANXA6, ARF6, ARL2, ARVCF, ATP1A1, B4GALT1, BAIAP2, CALR, CAP1, CAPN1, CAPN2, CASK, CAT, CAV1, CBL, CD2AP, CD44, CD81, CDC42, CDC42BPA, CDC42BPB, CDC42EP1, CDH2, CDH3, CGN, CLASP1, CNN1, CNN3, CORO1C, CSPG4, CSRP2, CTNNB1, CXADR, CYFIP1, DCTN4, DDX6, DLG1, DOCK7, DSP, ECT2, EFNB2, EGFR, EPB41L3, EPB41L5, EPCAM, ERBIN, F11R, FAT1, FERMT2, FHL1, FLNB, FLNC, FLT1, FOCAD, FSCN1, G3BP1, GAB1, GLMN, GNB2, GSN, HSP90B1, HSPA5,	Downregulation

					HSPA9, HSPB1, ILF3, ILK, IQGAP1, IQGAP3, ITGA5, ITGA6, ITGAV, ITGB1, JAM3, KIF23, KIRREL1, KRT18, KRT8, LASP1, LIMS1, LPP, LRP1, MAPK1, MCAM, MDC1, MIPEP, MME, MPRIP, MRC2, MSN, MTDH, MYADM, MYH9, MYO1E, NCKAP1, NDRG1, NECTIN2, NIBAN2, NOL6, NPM1, OCLN, ORC3, P4HB, PAK1, PALLD, PARD3, PARD6B, PARVA, PCBP2, PDGFRB, PDIA3, PDLIM2, PDLIM4, PDLIM5, PDLIM7, PI4KA, PIK3CA, PKP2, PLCG1, PLEC, PLEKHA7, PLPP3, PPFIBP1, PPIB, PPP1R9B, PRKAR2A, PRUNE1, PTK7, PTPN12, PXDN, PXMP2, RAP2B, RAP2C, RDX, RPL13A, RPL22, RPL4, RPL5, RPL7A, RPS2, RPS8, RRAS, S100A11, SCARB2, SIRT2, SLC2A1, SLC9A3R2, SNTB2, SORBS1, SORBS3, SPECC1L, SRC, SYMPK, TGFB1I1, TGM2, TJP2, TLN1, TLN2, TNS1, TNS3, TPM4, TWF1, VIM, ZNF185]	
cell morphogenesis	GO_BiologicalProcess-EBI-UniProt-GOA-ACAP-ARAP_13.05.2021_00h00	2.04E-13	12.13768	134	[ABI1, ACTBL2, ACTN1, ACTN4, ACTR2, ADAM10, ADARB1, ALCAM, ANK3, ANTXR1, ANXA1, AP3B1, APOE, ARAP1, ARHGDIA, AURKA, BAIAP2, BRWD1, C1QBP, CALR, CAP1, CAP2, CAPRIN1, CASP3, CD44, CDC42, CDC42EP1, CDC42EP3, CDH2, CORO1C, CRMP1, CTNNB1, CUL7, CYFIP1, DBNL, DIAPH1, DICER1, DLG1, DOCK7, DPYSL2, ECT2, EFNB2, EGFR, EPB41, EPB41L3, EPB41L5, EPHB3, EPS8, F11R, FARP1, FAT1, FBXW8, FERMT2, FGG, FLNB, FN1, GAB1, GDI1, GPC1, HSP90AB1, ILK, IQGAP1, IST1, ITGAV, ITGB1, KIF5B, LAMB1, LAMC1, LIMS1, LRP1, LYPLA2, MACF1, MAP1B, MAP1S, MAPK1, MAPK14, METAP1, MSN, MYADM, MYH10, MYH9, NCAM1, NCKAP1, NEDD4, NIBAN2, NRP2, P4HB, PAK1, PALLD, PARD3, PARD6B, PARVA, PDLIM5, PDLIM7, PHIP, PICALM, PIK3CA, PLCG1, PLOD3, PLXNB2,	Downregulation

					PRKCA, PRKDC, PRPF40A, PTK7, PTPRD, PXDN, RAB1A, RAB8A, RAC3, RAP2A, RCC2, RDX, RP2, S100A10, SARM1, SART3, SCFD1, SEC24B, SEPTIN7, SIN3A, SLC1A3, SPAG9, SPARC, SPTAN1, SPTBN1, SPTBN2, SRC, TPM1, USP9X, VAMP3, VLDLR, YAP1, ZDHHC17, ZMYM3]	
regulation of anatomical structure morphogenesis	GO_BiologicalProcess-EBI-UniProt-GOA-ACAP-ARAP_13.05.2021_00h00	2.04E-13	11.77052	119	[ACTN4, ACTR2, ADAM10, AGO1, AGO2, ANXA1, ANXA3, AP2A1, AP2A2, AP2B1, AP2M1, AP2S1, ARAP1, ARHGDIA, ATP2B4, AURKA, BAIAP2, BRWD1, C1QBP, CALR, CAPRIN1, CD44, CDC42, CDC42EP1, CDC42EP3, CDH2, CLASP1, COL4A2, CORO1C, CTNNB1, CUL7, CYFIP1, DBNL, DIAPH1, DLG1, EPB41, EPB41L3, EPHB3, EPN2, EPS8, ERAP1, F11R, FBXW8, FERMT2, FGG, FLT1, FN1, GAB1, GARS1, GATA6, GDI1, GLUL, GPC3, GPC6, HK2, HMOX1, HSPB1, HUWE1, ILK, IST1, ITGA5, ITGB1, LIMS1, LRP1, MACF1, MAGED1, MAP1B, MARCHF5, MCU, MSN, MTDH, MYADM, MYDGF, MYH10, MYH9, NEDD4, NIBAN2, P4HB, PAK1, PARVA, PDLIM5, PHIP, PKM, PLCG1, PLXNB2, PRKCA, PRKDC, PRPF40A, PSMC6, PSMD1, PSMD2, PSMD9, PSME3, PTGIS, PTK7, PTPRD, RAC3, RAP2A, RCC2, RDX, RNH1, ROCK2, RRAS, S100A10, SARM1, SARS1, SEC24B, SEPTIN7, SIRT1, SPAG9, SPARC, SRC, STIM1, THBS1, TPM1, VAT1, VPS35, WARS1, ZMYM3]	Downregulation
system development	GO_BiologicalProcess-EBI-UniProt-GOA-ACAP-ARAP_13.05.2021_00h00	2.04E-13	8.955224	462	[ABAT, ABCB10, ABI1, ACAT1, ACIN1, ACSL4, ACTBL2, ACTN1, ACTR2, ADAM10, ADAR, ADARB1, ADD2, AGO1, AGO2, AGTPBP1, AIMP2, AK4, AKT1, ALCAM, ALPL, ANK2, ANK3, ANKS1A, ANO6, ANTXR1, ANXA1, ANXA2, ANXA3, ANXA6, AP2A1, AP2A2, AP2B1, AP2M1, AP2S1, AP3B1, APAF1, APOE, APPL2, APRT, ARCN1, ARF4, ARF6, ARFGEF1, ARHGAP5, ARHGDIA,	Downregulation

					ARL3, ARMC6, ARSA, ARSB, ASAP1, ASNS, ATIC, ATP2B1, ATP2B2, ATP2B4, AURKA, B4GALT1, BAG3, BAIAP2, BIN1, BIRC6, BPNT2, BPTF, BZW2, CACYBP, CALD1, CALR, CAMK1, CAPN1, CAPNS1, CAPRIN1, CASP3, CAT, CAV1, CBL, CCNB1, CD44, CD81, CDC42, CDC73, CDH2, CDH3, CDK5RAP1, CDK5RAP3, CELF1, CFL2, CLASP1, CNRIP1, CNTFR, COL1A1, COL1A2, COL4A1, COL4A2, COL5A1, COL5A2, COL6A3, COPS2, CORO1C, CRMP1, CSDE1, CSPG4, CTNNB1, CTNNBL1, CTSC, CUL4A, CUL4B, CUL7, CXADR, CYFIP1, CYP51A1, DAGLB, DAZAP1, DBNL, DCAF1, DCTN1, DDRGK1, DDX39B, DDX5, DDX6, DEGS1, DHX30, DHX37, DICER1, DIPK2A, DLG1, DNMT1, DNMT3A, DNMT3B, DOCK7, DPPA4, DPYSL2, DPYSL3, DSP, ECE1, ECT2, EEF2, EFNB2, EGFR, EHD1, EIF4A3, ELP3, EMD, EML1, EPB41L3, EPB41L5, EPCAM, EPHB3, EPN2, ERAP1, ERCC2, ERO1A, FARP1, FAT1, FBN2, FBXW8, FERMT2, FHL1, FKBP10, FKBP4, FKBP8, FLNB, FLT1, FN1, FNDC3A, FOXK1, FXR1, GAB1, GARS1, GART, GATA6, GDI1, GLMN, GLUD1, GLUL, GNG12, GOT2, GPC1, GPC3, GPC6, GPSM1, GSDME, GSN, GSS, GSTM3, GYS1, HDAC2, HDAC4, HELLS, HK2, HMGCL, HMOX1, HOOK3, HSD17B4, HSP90AB1, HSPA5, HSPA9, HSPB1, HSPB11, HSPD1, HTRA1, IDH2, IGF2BP3, ILK, IMPDH2, IQGAP1, IQGAP3, IRF2BPL, IST1, ITCH, ITGA5, ITGA6, ITGAV, ITGB1, ITPK1, JAM3, JARID2, KANK2, KAT7, KDELR1, KDM1A, KIF5B, KRT18, KRT19, KRT8, LAMB1, LAMC1, LBR, LGALS1, LIG1, LIG3, LIMS1, LIN28A, LNPK, LPCAT1, LRP1, LRRC8A, LRRK1, LYAR, LYPLA2, MACF1, MACROH2A1, MAGED1, MAN1A2, MAP1B, MAP1S, MAP2K6,	

					MAP4, MAPK1, MAPK14, MATR3, MBD3, MBNL1, MCAM, MCM2, MCM7, METAP1, MFGE8, MGST1, MLH1, MME, MSH2, MSH3, MSH6, MSI1, MSN, MTDH, MTHFD1, MTR, MYDGF, MYEF2, MYH9, MYL9, MYO1E, NCAM1, NCAPG, NCAPG2, NCKAP1, NCL, NDRG1, NDRG2, NEDD4, NIBAN2, NID1, NIF3L1, NLE1, NLGN4X, NLN, NOL6, NRP2, NSDHL, NSUN2, NSUN5, NUP107, NUP133, NUP160, NXN, OXCT1, P3H1, P4HTM, PAK1, PALLD, PAPSS2, PARD3, PARD6B, PARP1, PARVA, PATZ1, PAXBP1, PDCD2, PDCL3, PDGFRB, PDLIM2, PDLIM4, PDLIM5, PDLIM7, PGM3, PGRMC2, PHIP, PICALM, PIGT, PIK3C2A, PIK3CA, PKM, PKP2, PLCG1, PLEKHA5, PLOD3, PLS3, PLXNB2, PNPT1, POFUT1, POFUT2, POLB, POLE, POLR1B, POR, PPIB, PPP1R9B, PRDX1, PRDX4, PRKAA1, PRKACB, PRKACG, PRKAR1A, PRKCA, PRKCSH, PRKDC, PRUNE1, PSMC6, PSMD1, PSMD2, PSMD9, PSME3, PTGIS, PTK7, PTPN2, PTPRD, PURA, PUS7, PXMP2, QDPR, RAB18, RAB23, RAB8A, RAC3, RAP2A, RBBP5, RBM15, RBPJ, RBPMS2, RDH10, RELA, RHEB, RIC8A, RIF1, RNH1, ROCK2, RPL22, RPS6KA1, RPS6KA3, RRAS, SALL2, SAMHD1, SARM1, SARS1, SART3, SBDS, SCARB2, SCYL1, SDF4, SEC16A, SEC24B, SEPTIN2, SERPINH1, SH3GL3, SIN3A, SIRT1, SIRT2, SLC1A3, SLC25A12, SLC25A5, SLC2A1, SLC2A10, SLC4A7, SLC5A3, SMARCA4, SMARCC1, SMARCD1, SNX17, SNX3, SORD, SPAG9, SPARC, SPTAN1, SPTBN1, SPTBN2, SPTLC2, SRC, SRR, SRSF1, STIM1, SUN2, SUPT6H, TACC3, TAGLN, TDP2, TENM3, TF, TGFB1I1, TGM2, THBS1, TIGAR, TMED10, TMED2, TMF1, TOP2A,	

					TPM1, TRIM28, TRIM71, TWF1, UBA6, UFL1, UGDH, UGP2, UNG, UPF2, USP19, USP9X, UTP25, UTRN, VIM, VLDLR, VPS35, WARS1, XRCC5, XRCC6, XRN2, YAP1, ZDHHC17, ZFP36L2, ZMYND8]	
cell-cell junction	GO_CellularComponent-EBI-UniProt-GOA-ACAP-ARAP_13.05.2021_00h00	2.04E-13	16.85824	88	[ACTN1, ADAM10, ADD3, AKT1, ANK2, ANK3, ANXA1, ANXA2, ARVCF, ATP1A1, B4GALT1, BAIAP2, CASK, CD2AP, CDC42, CDC42BPA, CDC42BPB, CDC42EP1, CDH2, CDH3, CGN, CNN3, CTNNB1, CXADR, DDX6, DLG1, DSP, ECT2, EFNB2, EPB41L3, EPB41L5, EPCAM, F11R, FAT1, FERMT2, FSCN1, GAB1, ILF3, ILK, IQGAP1, IQGAP3, ITGA5, ITGA6, ITGB1, JAM3, KIRREL1, KRT18, KRT8, LIMS1, MIPEP, MTDH, MYADM, MYH9, MYO1E, NDRG1, NECTIN2, NIBAN2, NOL6, OCLN, ORC3, PAK1, PARD3, PARD6B, PDLIM2, PDLIM4, PDLIM5, PDLIM7, PIK3CA, PKP2, PLCG1, PLEKHA7, PLPP3, PPP1R9B, PTK7, PXDN, PXMP2, RAP2B, RAP2C, RDX, S100A11, SIRT2, SLC2A1, SORBS1, SPECC1L, SYMPK, TJP2, TLN1, TWF1]	Downregulation
cell-substrate junction	GO_CellularComponent-EBI-UniProt-GOA-ACAP-ARAP_13.05.2021_00h00	2.04E-13	25.43478	117	[ACTN1, ACTN4, ACTR2, ADAM10, ALCAM, ANXA1, ANXA5, ANXA6, ARF6, ARL2, CALR, CAP1, CAPN1, CAPN2, CASK, CAT, CAV1, CBL, CD44, CD81, CDC42, CDC42EP1, CDH2, CLASP1, CNN1, CNN3, CORO1C, CSPG4, CSRP2, CTNNB1, CYFIP1, DCTN4, DOCK7, EFNB2, EGFR, EPB41L5, ERBIN, FAT1, FERMT2, FHL1, FLNB, FLNC, FLT1, FOCAD, G3BP1, GLMN, GNB2, GSN, HSP90B1, HSPA5, HSPA9, HSPB1, ILK, IQGAP1, ITGA5, ITGA6, ITGAV, ITGB1, KIF23, LASP1, LIMS1, LPP, LRP1, MAPK1, MCAM, MDC1, MME, MPRIP, MRC2, MSN, MYH9, NCKAP1, NECTIN2, NOL6, NPM1, P4HB, PAK1, PALLD, PARVA, PCBP2, PDGFRB, PDIA3, PDLIM7, PI4KA, PLEC, PPFIBP1, PPIB, PRKAR2A, PRUNE1,	Downregulation

					PTK7, PTPN12, PXDN, RDX, RPL13A, RPL22, RPL4, RPL5, RPL7A, RPS2, RPS8, RRAS, SCARB2, SLC9A3R2, SNTB2, SORBS1, SORBS3, SRC, TGFB1I1, TGM2, TLN1, TLN2, TNS1, TNS3, TPM4, TWF1, VIM, ZNF185]	
cell junction organization	GO_BiologicalProcess-EBI-UniProt-GOA-ACAP-ARAP_13.05.2021_00h00	2.04E-13	12.69634	97	[ACTBL2, ACTN1, ACTR2, ADAM10, ADD2, ANK2, ANK3, APOE, ARF4, ARF6, ARL2, ARVCF, BAIAP2, CAMK1, CAPRIN1, CAST, CAV1, CDC42, CDH2, CDH3, CLASP1, COL4A1, CORO1C, CTBP2, CTNNB1, CXADR, CYFIP1, DBNL, DCTN1, DLG1, DPYSL2, DSP, DUSP3, ECT2, EFNB2, EPB41L3, EPB41L5, EPHB3, F11R, FARP1, FERMT2, FLNC, FN1, FSCN1, GPC6, GPHN, ILK, IQGAP1, ITGA6, JAM3, LAMC1, LIMS1, LRP1, MACF1, MAP1B, MAPK14, MESD, MTDH, MYADM, MYH10, MYO1C, MYO1E, NDRG1, NECTIN2, NEDD4, NLGN4X, NRP2, OCLN, PARD3, PARD6B, PDLIM5, PDZRN3, PKP2, PLEC, PLEKHA7, PLXNB2, PPFIBP1, PPM1F, PRKCA, PTPRD, RCC2, RDX, ROCK2, S100A10, SEPTIN11, SLK, SORBS1, SPARC, SPTBN2, SRC, THBS1, TJP2, TLN1, TLN2, UTRN, VPS35, ZMYND8]	Downregulation
cell morphogenesis involved in differentiation	GO_BiologicalProcess-EBI-UniProt-GOA-ACAP-ARAP_13.05.2021_00h00	2.04E-13	12.37374	98	[ABI1, ACTBL2, ACTN1, ACTN4, ACTR2, ADAM10, ADARB1, ALCAM, ANK3, ANTXR1, APOE, ARHGDIA, BAIAP2, C1QBP, CALR, CAPRIN1, CASP3, CDC42, CDH2, CORO1C, CRMP1, CTNNB1, CUL7, CYFIP1, DBNL, DOCK7, DPYSL2, EFNB2, EPB41L5, EPHB3, FARP1, FAT1, FBXW8, FERMT2, FGG, FLNB, FN1, GAB1, GDI1, GPC1, HSP90AB1, ILK, IST1, ITGAV, ITGB1, KIF5B, LAMB1, LAMC1, LIMS1, LRP1, LYPLA2, MACF1, MAP1B, MAP1S, MAPK1, METAP1, MYADM, MYH9, NCAM1, NEDD4, NIBAN2, NRP2, P4HB, PAK1, PALLD, PARD3, PARD6B, PARVA, PDLIM5, PDLIM7, PICALM, PIK3CA,	Downregulation

					PLCG1, PLOD3, PLXNB2, PRKCA, PRKDC, PTK7, PTPRD, PXDN, RAB1A, RAB8A, RAC3, RAP2A, RCC2, S100A10, SARM1, SEC24B, SIN3A, SLC1A3, SPTAN1, SPTBN1, SPTBN2, SRC, USP9X, VAMP3, VLDLR, ZDHHC17]	
regulation of cell morphogenesis	GO_BiologicalProcess-EBI-UniProt-GOA-ACAP-ARAP_13.05.2021_00h00	2.04E-13	15.90214	52	[ACTN4, ACTR2, ANXA1, ARAP1, ARHGDIA, BAIAP2, BRWD1, C1QBP, CALR, CAPRIN1, CD44, CDC42, CDC42EP1, CDC42EP3, CORO1C, CUL7, CYFIP1, DBNL, DIAPH1, DLG1, EPB41, EPB41L3, EPS8, F11R, FBXW8, FERMT2, FGG, FN1, ILK, LIMS1, MACF1, MSN, MYADM, MYH10, MYH9, P4HB, PARVA, PHIP, PLXNB2, PRKDC, PRPF40A, PTPRD, RAC3, RCC2, RDX, S100A10, SEPTIN7, SPAG9, SPARC, SRC, TPM1, ZMYM3]	Downregulation
adherens junction	GO_CellularComponent-EBI-UniProt-GOA-ACAP-ARAP_13.05.2021_00h00	2.04E-13	21.11111	38	[ADAM10, ANXA1, ANXA2, ARVCF, BAIAP2, CDC42EP1, CDH2, CDH3, CNN3, CTNNB1, CXADR, DDX6, DLG1, EFNB2, EPB41L5, FERMT2, ILF3, ITGA6, KRT18, MYH9, MYO1E, NDRG1, NECTIN2, NIBAN2, PARD3, PDLIM2, PDLIM4, PDLIM5, PDLIM7, PKP2, PLEKHA7, PLPP3, PPP1R9B, RDX, S100A11, SORBS1, TJP2, TLN1]	Downregulation
focal adhesion	GO_CellularComponent-EBI-UniProt-GOA-ACAP-ARAP_13.05.2021_00h00	2.04E-13	25.44248	115	[ACTN1, ACTN4, ACTR2, ADAM10, ALCAM, ANXA1, ANXA5, ANXA6, ARF6, ARL2, CALR, CAP1, CAPN1, CAPN2, CASK, CAT, CAV1, CBL, CD44, CD81, CDC42, CDC42EP1, CDH2, CLASP1, CNN1, CNN3, CORO1C, CSPG4, CSRP2, CTNNB1, CYFIP1, DCTN4, DOCK7, EFNB2, EGFR, EPB41L5, FAT1, FERMT2, FHL1, FLNB, FLNC, FLT1, FOCAD, G3BP1, GLMN, GNB2, GSN, HSP90B1, HSPA5, HSPA9, HSPB1, ILK, IQGAP1, ITGA5, ITGA6, ITGAV, ITGB1, KIF23, LASP1, LIMS1, LPP, LRP1, MAPK1, MCAM, MDC1, MME, MPRIP, MRC2, MSN, MYH9, NCKAP1, NECTIN2, NPM1, P4HB, PAK1, PALLD, PARVA, PCBP2, PDGFRB, PDIA3, PDLIM7, PI4KA, PLEC, PPFIBP1, PPIB,	Downregulation

					PRKAR2A, PRUNE1, PTK7, PTPN12, PXDN, RDX, RPL13A, RPL22, RPL4, RPL5, RPL7A, RPS2, RPS8, RRAS, SCARB2, SLC9A3R2, SNTB2, SORBS1, SORBS3, SRC, TGFB1I1, TGM2, TLN1, TLN2, TNS1, TNS3, TPM4, TWF1, VIM, ZNF185]	
positive regulation of cell development	GO_BiologicalProcess-EBI-UniProt-GOA-ACAP-ARAP_13.05.2021_00h00	2.04E-13	14.60317	46	[ACTR2, ARHGDIA, AURKA, BAIAP2, BIN1, C1QBP, CALR, CAPRIN1, CDC42, CTNNB1, CUL7, CYFIP1, DBNL, DICER1, EGFR, F11R, FBXW8, FERMT2, FGG, FN1, GDI1, HDAC2, ILK, IST1, KDM1A, LIMS1, LRP1, MACF1, MAP1B, MME, MYADM, NIBAN2, P4HB, PAK1, PLXNB2, PRKDC, PTPRD, RAC3, RBM15, RELA, RHEB, S100A10, SIRT2, TGM2, UFL1, XRCC5]	Downregulation
cell junction assembly	GO_BiologicalProcess-EBI-UniProt-GOA-ACAP-ARAP_13.05.2021_00h00	2.04E-13	13.20346	61	[ACTN1, ADD2, ANK2, ARF6, ARL2, ARVCF, CAV1, CDC42, CDH2, CLASP1, CORO1C, CTNNB1, DBNL, DLG1, DUSP3, ECT2, EFNB2, EPB41L3, EPB41L5, EPHB3, F11R, FARP1, FERMT2, FLNC, FN1, FSCN1, GPC6, ILK, IQGAP1, ITGA6, JAM3, LAMC1, LIMS1, LRP1, MACF1, MAP1B, MTDH, MYO1C, MYO1E, NLGN4X, OCLN, PARD3, PARD6B, PDLIM5, PKP2, PLEC, PLXNB2, PPM1F, PRKCA, PTPRD, RCC2, ROCK2, S100A10, SLK, SORBS1, SPTBN2, SRC, THBS1, TLN1, TLN2, VPS35]	Downregulation
substrate adhesion-dependent cell spreading	GO_BiologicalProcess-EBI-UniProt-GOA-ACAP-ARAP_13.05.2021_00h00	2.04E-13	21.55172	25	[ACTN4, ANTXR1, C1QBP, CALR, CDC42, CORO1C, EPHB3, FERMT2, FGG, FN1, ILK, ITGAV, LAMB1, LAMC1, LIMS1, MYADM, P4HB, PARVA, PXDN, RAB1A, RAC3, RCC2, S100A10, SRC, VAMP3]	Downregulation
cell-cell junction organization	GO_BiologicalProcess-EBI-UniProt-GOA-ACAP-ARAP_13.05.2021_00h00	2.04E-13	16.08696	37	[ADAM10, ANK2, ARL2, ARVCF, CAV1, CDC42, CDH2, CDH3, CTNNB1, CXADR, DLG1, DSP, ECT2, EPB41L3, F11R, FERMT2, FSCN1, JAM3, LIMS1, MTDH, MYADM, MYO1C, MYO1E, NECTIN2, NLGN4X, OCLN, PARD3, PARD6B, PKP2, PLEKHA7, PRKCA, RDX, ROCK2, SRC, TJP2, TLN1, TLN2]	Downregulation
cell-substrate junction organization	GO_BiologicalProcess-EBI-UniProt-	2.04E-13	20.51282	24	[ACTN1, ARF6, CLASP1, CORO1C, DUSP3, EPB41L5, FERMT2, FN1, IQGAP1, ITGA6, LAMC1,	Downregulation

	GOA-ACAP-ARAP_13.05.2021_00h00				LIMS1, LRP1, MACF1, PLEC, PPM1F, RCC2, ROCK2, S100A10, SLK, SORBS1, SRC, THBS1, TLN1]	
regulation of cell morphogenesis involved in differentiation	GO_BiologicalProcess-EBI-UniProt-GOA-ACAP-ARAP_13.05.2021_00h00	2.04E-13	22	22	[ACTN4, ACTR2, BAIAP2, C1QBP, CALR, CAPRIN1, CDC42, CORO1C, CUL7, DBNL, FBXW8, FERMT2, FGG, ILK, LIMS1, MYADM, P4HB, PRKDC, PTPRD, RAC3, RCC2, S100A10]	Downregulation
positive regulation of cell morphogenesis involved in differentiation	GO_BiologicalProcess-EBI-UniProt-GOA-ACAP-ARAP_13.05.2021_00h00	2.04E-13	22.61905	19	[ACTR2, BAIAP2, C1QBP, CALR, CAPRIN1, CDC42, CUL7, DBNL, FBXW8, FERMT2, FGG, ILK, LIMS1, MYADM, P4HB, PRKDC, PTPRD, RAC3, S100A10]	Downregulation
RNA localization	GO_BiologicalProcess-EBI-UniProt-GOA-ACAP-ARAP_13.05.2021_00h00	1.37E-26	22.22222	54	[AAAS, ANTXR1, ATR, CCT2, CCT3, CCT4, CCT5, CCT6A, CCT7, CCT8, CKAP5, CPSF2, CPSF3, DDX39B, DHX9, EIF4A3, EXOSC10, EXOSC2, G3BP2, IGF2BP3, IWS1, LRPPRC, MMAB, MYO1C, MYO1E, NDC1, NOL6, NOP58, NPM1, NSUN2, NUP107, NUP133, NUP160, NUP210, NUP35, NUP50, PABPN1, PARN, PNPT1, RAN, RANBP2, SEC13, SEH1L, SRSF1, SRSF11, SRSF7, SSB, SUPT6H, SYMPK, TCP1, UPF2, UPF3B, WRAP53, XPO5]	Upregulation
establishment of RNA localization	GO_BiologicalProcess-EBI-UniProt-GOA-ACAP-ARAP_13.05.2021_00h00	1.37E-26	19.80676	41	[AAAS, ANTXR1, ATR, CKAP5, CPSF2, CPSF3, DDX39B, DHX9, EIF4A3, G3BP2, IGF2BP3, IWS1, LRPPRC, MMAB, MYO1C, MYO1E, NDC1, NOL6, NPM1, NSUN2, NUP107, NUP133, NUP160, NUP210, NUP35, NUP50, PABPN1, PNPT1, RAN, RANBP2, SEC13, SEH1L, SRSF1, SRSF11, SRSF7, SSB, SUPT6H, SYMPK, UPF2, UPF3B, XPO5]	Upregulation
nuclear transport	GO_BiologicalProcess-EBI-UniProt-GOA-ACAP-ARAP_13.05.2021_00h00	1.37E-26	18.08219	66	[AAAS, AKT1, APPL2, BAG3, CALR, CAMK1, CPSF2, CPSF3, CSE1L, DDX20, DDX39B, DHX9, ECT2, EIF4A3, EMD, GEMIN4, GEMIN5, HSP90AB1, HSPA9, IPO11, IPO4, IPO5, IWS1, LSG1, MAPK1, MAPK14, MAVS, NDC1, NEDD4, NOL6, NOP9, NPM1, NSUN2, NUP107, NUP133, NUP160, NUP210, NUP35, NUP50, PABPN1, PRKAG1, PRKCA, PSIP1, RAB23,	Upregulation

					RAN, RANBP2, RANGAP1, RRS1, SDAD1, SEC13, SEH1L, SNRPD3, SRSF1, SRSF11, SRSF7, SSB, STYX, SUPT6H, SYMPK, TNPO3, TRIM28, UBR5, UPF2, UPF3B, XPO5, XPO7]	
nucleobase-containing compound transport	GO_BiologicalProcess-EBI-UniProt-GOA-ACAP-ARAP_13.05.2021_00h00	1.37E-26	16.41221	43	[AAAS, CKAP5, CPSF2, CPSF3, DDX39B, DHX9, EIF4A3, G3BP2, IGF2BP3, IWS1, LRPPRC, LRRC8A, MYO1C, MYO1E, NDC1, NOL6, NPM1, NSUN2, NUP107, NUP133, NUP160, NUP210, NUP35, NUP50, PABPN1, PNPT1, RAN, RANBP2, SEC13, SEH1L, SLC25A24, SLC25A32, SLC25A5, SLC33A1, SRSF1, SRSF11, SRSF7, SSB, SUPT6H, SYMPK, UPF2, UPF3B, XPO5]	Upregulation
RNA transport	GO_BiologicalProcess-EBI-UniProt-GOA-ACAP-ARAP_13.05.2021_00h00	1.37E-26	18.90547	38	[AAAS, CKAP5, CPSF2, CPSF3, DDX39B, DHX9, EIF4A3, G3BP2, IGF2BP3, IWS1, LRPPRC, MYO1C, MYO1E, NDC1, NOL6, NPM1, NSUN2, NUP107, NUP133, NUP160, NUP210, NUP35, NUP50, PABPN1, PNPT1, RAN, RANBP2, SEC13, SEH1L, SRSF1, SRSF11, SRSF7, SSB, SUPT6H, SYMPK, UPF2, UPF3B, XPO5]	Upregulation
ribonucleoprotein complex export from nucleus	GO_BiologicalProcess-EBI-UniProt-GOA-ACAP-ARAP_13.05.2021_00h00	1.37E-26	24.81203	33	[AAAS, CPSF2, CPSF3, DDX39B, EIF4A3, IWS1, LSG1, NDC1, NOL6, NOP9, NPM1, NSUN2, NUP107, NUP133, NUP160, NUP210, NUP35, NUP50, PABPN1, RAN, RANBP2, RRS1, SDAD1, SEC13, SEH1L, SRSF1, SRSF11, SRSF7, SSB, SUPT6H, SYMPK, UPF2, UPF3B]	Upregulation
protein localization to nucleus	GO_BiologicalProcess-EBI-UniProt-GOA-ACAP-ARAP_13.05.2021_00h00	1.37E-26	17.82178	54	[AKT1, APPL2, ARL2, BAG3, CALR, CCT2, CCT3, CCT4, CCT5, CCT6A, CCT7, CCT8, CD2AP, CDK5RAP3, COL1A1, CSE1L, ECT2, FERMT2, GLUL, HSP90AB1, IPO11, IPO4, IPO5, KAT7, MAGED1, MAPK1, MAPK14, MAVS, NPM1, NUP107, NUP133, NUP35, NUP50, NVL, PARP1, PLK1, RAB23, RAN, RANBP2, RANGAP1, RPF2, RRS1, SEC13, SESN2, SIN3A, SRC, SUN2, TCP1, TNPO3, TRIM28, UBR5, UTP25, WRAP53, YAP1]	Upregulation
mRNA transport	GO_BiologicalProcess-EBI-	1.37E-26	18.3432	31	[AAAS, CPSF2, CPSF3, DDX39B, DHX9, EIF4A3, G3BP2, IGF2BP3, IWS1,	Upregulation

	UniProt-GOA-ACAP-ARAP_13.05.2021_00h00				LRPPRC, MYO1C, MYO1E, NDC1, NSUN2, NUP107, NUP133, NUP160, NUP210, NUP35, NUP50, PABPN1, RANBP2, SEC13, SEH1L, SRSF1, SRSF11, SRSF7, SUPT6H, SYMPK, UPF2, UPF3B]	
RNA export from nucleus	GO_BiologicalProcess-EBI-UniProt-GOA-ACAP-ARAP_13.05.2021_00h00	1.37E-26	23.30827	31	[AAAS, CPSF2, CPSF3, DDX39B, DHX9, EIF4A3, IWS1, NDC1, NOL6, NPM1, NSUN2, NUP107, NUP133, NUP160, NUP210, NUP35, NUP50, PABPN1, RAN, RANBP2, SEC13, SEH1L, SRSF1, SRSF11, SRSF7, SSB, SUPT6H, SYMPK, UPF2, UPF3B, XPO5]	Upregulation
nuclear export	GO_BiologicalProcess-EBI-UniProt-GOA-ACAP-ARAP_13.05.2021_00h00	1.37E-26	21.63462	45	[AAAS, BAG3, CALR, CAMK1, CPSF2, CPSF3, CSE1L, DDX39B, DHX9, EIF4A3, EMD, HSPA9, IWS1, LSG1, NDC1, NOL6, NOP9, NPM1, NSUN2, NUP107, NUP133, NUP160, NUP210, NUP35, NUP50, PABPN1, PRKCA, RAN, RANBP2, RANGAP1, RRS1, SDAD1, SEC13, SEH1L, SRSF1, SRSF11, SRSF7, SSB, STYX, SUPT6H, SYMPK, UPF2, UPF3B, XPO5, XPO7]	Upregulation
pyruvate metabolic process	GO_BiologicalProcess-EBI-UniProt-GOA-ACAP-ARAP_13.05.2021_00h00	1.37E-26	20.11173	36	[AAAS, ADPGK, ALDOC, ENO2, FOXK1, GALK1, GPI, HAGH, HDAC4, HK1, HK2, HOOK3, LDHB, ME1, MPC2, NDC1, NUP107, NUP133, NUP160, NUP210, NUP35, NUP50, OGT, PC, PFKL, PFKM, PFKP, PKM, PRKAA1, PRKAG1, RANBP2, SBDS, SEC13, SEH1L, SRR, TIGAR]	No change
nucleoside diphosphate phosphorylation	GO_BiologicalProcess-EBI-UniProt-GOA-ACAP-ARAP_13.05.2021_00h00	1.37E-26	21.05263	32	[AAAS, ADPGK, AK1, AK4, ALDOC, CMPK1, ENO2, FOXK1, GALK1, GPI, HDAC4, HK1, HK2, HOOK3, NDC1, NUP107, NUP133, NUP160, NUP210, NUP35, NUP50, OGT, PFKL, PFKM, PFKP, PKM, PRKAA1, PRKAG1, RANBP2, SEC13, SEH1L, TIGAR]	No change
ribonucleoside diphosphate metabolic process	GO_BiologicalProcess-EBI-UniProt-GOA-ACAP-ARAP_13.05.2021_00h00	1.37E-26	22.78481	36	[AAAS, ADPGK, AK1, AK4, ALDOC, CASK, CMPK1, DLG1, ENO2, FOXK1, GALK1, GPI, HDAC4, HK1, HK2, HOOK3, NDC1, NUDT16, NUP107, NUP133, NUP160, NUP210, NUP35, NUP50, OGT, PFKL, PFKM, PFKP, PKM, PRKAA1, PRKAG1,	No change

					RANBP2, SEC13, SEH1L, TIGAR, TJP2]	
glycolytic process	GO_BiologicalProcess-EBI-UniProt-GOA-ACAP-ARAP_13.05.2021_00h00	1.37E-26	22.1374	29	[AAAS, ADPGK, ALDOC, ENO2, FOXK1, GALK1, GPI, HDAC4, HK1, HK2, HOOK3, NDC1, NUP107, NUP133, NUP160, NUP210, NUP35, NUP50, OGT, PFKL, PFKM, PFKP, PKM, PRKAA1, PRKAG1, RANBP2, SEC13, SEH1L, TIGAR]	No change
ADP metabolic process	GO_BiologicalProcess-EBI-UniProt-GOA-ACAP-ARAP_13.05.2021_00h00	1.37E-26	22.14286	31	[AAAS, ADPGK, AK1, AK4, ALDOC, ENO2, FOXK1, GALK1, GPI, HDAC4, HK1, HK2, HOOK3, NDC1, NUP107, NUP133, NUP160, NUP210, NUP35, NUP50, OGT, PFKL, PFKM, PFKP, PKM, PRKAA1, PRKAG1, RANBP2, SEC13, SEH1L, TIGAR]	No change
mitotic cell cycle	GO_BiologicalProcess-EBI-UniProt-GOA-ACAP-ARAP_13.05.2021_00h00	1.51E-13	13.79621	153	[AAAS, AATF, AKT1, ANK3, ANXA1, APPL2, ARL3, ASNS, AURKA, AURKB, BCCIP, BUB1B, CCNB1, CCND2, CCNH, CCNY, CDC42, CDC73, CDCA8, CDK5RAP3, CDK7, CHEK1, CHEK2, CKAP5, CLASP1, CNOT11, CRLF3, CTNNB1, CUL4A, CUL4B, CUL7, CYFIP1, DCTN1, DLG1, DLGAP5, DNMT3A, DRG1, DUSP3, DYNC1LI2, ECT2, EGFR, EMD, EML1, EPS8, ERCC2, ERCC6L, FANCD2, FHL1, FSD1, GBF1, GINS3, GOLGA2, HMMR, HSPA2, HTT, INCENP, IQGAP1, IQGAP3, IST1, ITGB1, KANK2, KIF11, KIF20A, KIF22, KIF23, KIF2C, KNTC1, LBR, LCMT1, LIG1, MACROH2A1, MASTL, MCM2, MCM3, MCM4, MCM6, MCM7, MDC1, MKI67, MSH2, MTA3, NCAPD2, NCAPG, NCAPG2, NDC1, NDC80, NDRG1, NEK7, NLE1, NSFL1C, NUP107, NUP133, NUP160, NUP35, NUSAP1, OPTN, PDGFRB, PDS5A, PHIP, PLK1, POGZ, POLA1, POLE, POLR1B, PPP1R9B, PRKAR2B, PRKCA, PRKDC, PSMC6, PSMD1, PSMD2, PSMD9, PSME3, RAD51, RAN, RANBP2, RANGAP1, RCC2, RDX, ROCK2, RPA2, RPA3, RRS1, RTKN, SBDS, SEC13, SEH1L, SET, SIN3A, SIRT1, SIRT2, SKA3, SKP2, SMC2,	Upregulation

					SNX9, SPTBN1, SUN2, TACC3, TPX2, TRIM71, TRIP13, TTC28, TTK, TTLL12, TUBA4A, TUBB6, USP47, VRK1, WAPL, WDHD1, YTHDC2, ZFP36L2, ZWILCH]	
cell cycle phase transition	GO_BiologicalProcess-EBI-UniProt-GOA-ACAP-ARAP_13.05.2021_00h00	1.51E-13	12.02346	82	[AKT1, ANTXR1, ANXA1, APPL2, ATP2B4, ATR, AURKA, AURKB, BUB1B, CCAR2, CCNB1, CCND2, CCNH, CCNY, CDC73, CDK5RAP3, CDK7, CHEK1, CHEK2, CKAP5, CLASP1, CNOT11, CRLF3, CUL4A, CUL4B, DCTN1, DDRGK1, DDX39B, DLG1, DLGAP5, EGFR, EPS8, ERCC2, FANCD2, FHL1, HMMR, HSPA2, IQGAP3, ITGB1, KANK2, KNTC1, LCMT1, MACROH2A1, MAPK14, MASTL, MDC1, MMAB, MSH2, MTA3, NDC80, NPM1, NSUN2, OPTN, PLK1, POLA1, POLE, POLR1B, PPP1R9B, PRKAR2B, PRKCA, PRKDC, PSMC6, PSMD1, PSMD2, PSMD9, PSME3, RCC2, RDX, RPA2, SIN3A, SIRT2, SKP2, TACC3, TIPRL, TPX2, TRIM71, TRIP13, TTK, TUBA4A, USP47, ZFP36L2, ZWILCH]	Upregulation
regulation of cell cycle	GO_BiologicalProcess-EBI-UniProt-GOA-ACAP-ARAP_13.05.2021_00h00	1.51E-13	11.9281	146	[AATF, ADAM10, ADARB1, AKT1, ANTXR1, ANXA1, APAF1, APPL2, ASNS, ATP2B4, ATR, AURKA, AURKB, BCCIP, BIN1, BIRC6, BOP1, BUB1B, CALR, CASP3, CBX5, CCAR2, CCNB1, CCND2, CCNH, CCNL2, CCNY, CDC123, CDC42, CDC73, CDK5RAP1, CDK5RAP3, CDK7, CELF1, CHEK1, CHEK2, CKAP5, CLASP1, CNOT11, COPS5, CRLF3, CTCF, CTNNB1, CUL4A, CUL7, CYFIP1, DCTN1, DDRGK1, DDX39B, DLG1, DLGAP5, DRG1, DUSP3, ECT2, EGFR, EHMT1, ERCC2, ETFA, FANCD2, FHL1, FSD1, GATA6, GBF1, GIPC1, GLMN, HCFC1, HMMR, HSP90AB1, HSPA2, INCENP, IPO5, IQGAP1, ITGB1, KANK2, KIF11, KIF20A, KIF23, KNTC1, MACROH2A1, MAP2K6, MAPK14, MASTL, MCM2, MDC1, MKI67, MMAB, MSH2, MTA3, MYBBP1A, NDC80, NDRG1, NEK7, NLE1, NPM1, NSFL1C, NSUN2, NUDT16, PDGFRB,	Upregulation

					PES1, PHC1, PHIP, PIK3R4, PLK1, PNPT1, POLR1B, PPM1G, PPP1R9B, PRKACB, PRKAR1A, PRKAR2B, PRKCA, PRKDC, PRPF40A, PSMC6, PSMD1, PSMD2, PSMD9, PSME3, RAD51, RCC2, RDX, ROCK2, RPA2, RPA3, SH3GLB1, SIN3A, SIRT1, SIRT2, SKP2, SRC, TACC3, TBRG4, THBS1, TIPRL, TPX2, TRIP13, TTC28, TTK, TTLL12, TUBA4A, USP19, USP47, WAPL, YTHDC2, ZFP36L2, ZWILCH]	
mitotic cell cycle process	GO_BiologicalProcess-EBI-UniProt-GOA-ACAP-ARAP_13.05.2021_00h00	1.51E-13	14.03141	134	[AAAS, AKT1, ANK3, ANXA1, APPL2, ARL3, AURKA, AURKB, BCCIP, BUB1B, CCNB1, CCND2, CCNH, CCNY, CDC42, CDC73, CDCA8, CDK5RAP3, CDK7, CHEK1, CHEK2, CKAP5, CLASP1, CNOT11, CRLF3, CUL4A, CUL4B, CUL7, DCTN1, DLG1, DLGAP5, DRG1, DYNC1LI2, ECT2, EGFR, EMD, EML1, EPS8, ERCC2, ERCC6L, FANCD2, FHL1, FSD1, GINS3, GOLGA2, HMMR, HSPA2, HTT, INCENP, IQGAP3, IST1, ITGB1, KANK2, KIF11, KIF20A, KIF22, KIF23, KIF2C, KNTC1, LBR, LCMT1, LIG1, MACROH2A1, MASTL, MCM2, MCM3, MCM4, MCM6, MCM7, MDC1, MKI67, MSH2, MTA3, NCAPD2, NCAPG, NCAPG2, NDC1, NDC80, NDRG1, NSFL1C, NUP107, NUP133, NUP160, NUP35, NUSAP1, OPTN, PDGFRB, PDS5A, PHIP, PLK1, POGZ, POLA1, POLE, POLR1B, PPP1R9B, PRKAR2B, PRKCA, PRKDC, PSMC6, PSMD1, PSMD2, PSMD9, PSME3, RAD51, RAN, RANBP2, RANGAP1, RCC2, RDX, ROCK2, RPA2, RRS1, RTKN, SBDS, SEC13, SEH1L, SET, SIN3A, SIRT2, SKP2, SMC2, SNX9, SPTBN1, SUN2, TACC3, TPX2, TRIM71, TRIP13, TTK, TUBA4A, USP47, VRK1, ZFP36L2, ZWILCH]	Upregulation
regulation of cell cycle process	GO_BiologicalProcess-EBI-UniProt-GOA-	1.51E-13	12.12121	100	[AKT1, ANTXR1, ANXA1, APAF1, APPL2, ATP2B4, ATR, AURKA, AURKB, BIRC6, BUB1B, CALR, CBX5, CCAR2, CCNB1,	Upregulation

	ACAP-ARAP_13.05.2021_00h00				CCND2, CDC42, CDC73, CDK5RAP3, CHEK1, CHEK2, CKAP5, CLASP1, CNOT11, CTCF, CTNNB1, CUL4A, CUL7, DCTN1, DDRGK1, DDX39B, DLG1, DLGAP5, DRG1, ECT2, EGFR, EHMT1, ERCC2, FANCD2, FHL1, FSD1, GIPC1, HMMR, HSPA2, INCENP, KANK2, KIF11, KIF20A, KIF23, KNTC1, MACROH2A1, MAPK14, MDC1, MKI67, MMAB, MSH2, MTA3, NDC80, NDRG1, NPM1, NSFL1C, NSUN2, NUDT16, PDGFRB, PHC1, PHIP, PIK3R4, PLK1, POLR1B, PPP1R9B, PRKAR1A, PRKAR2B, PRKCA, PRKDC, PRPF40A, PSMC6, PSMD1, PSMD2, PSMD9, PSME3, RAD51, RCC2, RDX, ROCK2, RPA2, SH3GLB1, SIN3A, SIRT1, SIRT2, TACC3, TIPRL, TPX2, TRIP13, TTK, TUBA4A, USP19, USP47, WAPL, ZFP36L2, ZWILCH]	
spindle organization	GO_BiologicalProcess-EBI-UniProt-GOA-ACAP-ARAP_13.05.2021_00h00	1.51E-13	15.90106	45	[AAAS, AURKA, AURKB, BCCIP, BUB1B, CCNB1, CDCA8, CHEK2, CKAP5, CLASP1, DCTN1, DLG1, DLGAP5, DRG1, DYNC1LI2, EML1, ERCC6L, FSD1, GOLGA2, INCENP, KIF11, KIF23, KIF2C, KNTC1, MLH1, MYH9, NDC80, NDRG1, NEK7, NUP107, NUP133, NUP160, PLK1, RAN, RANBP2, RANGAP1, RCC2, SBDS, SEC13, SEH1L, SUN2, TACC3, TPX2, TTK, ZWILCH]	Upregulation
chromosome, centromeric region	GO_CellularComponent-EBI-UniProt-GOA-ACAP-ARAP_13.05.2021_00h00	1.51E-13	22.48804	47	[AURKB, BAZ1B, BUB1B, CBX5, CCNB1, CDCA8, CKAP5, CLASP1, CTCF, DAXX, DCTN1, DCTN4, DNMT1, DNMT3A, DYNC1LI2, ERCC6L, HELLS, INCENP, KAT7, KIF22, KIF2C, KNTC1, LRWD1, MACROH2A1, NCAPD2, NCAPG, NDC80, NUP107, NUP133, NUP160, ORC2, PDS5A, PLK1, PPP2R5A, RANGAP1, RCC2, SEC13, SEH1L, SEPTIN2, SEPTIN7, SIN3A, SKA3, SPOUT1, TOP2A, TTK, WAPL, ZWILCH]	Upregulation
chromosome, telomeric region	GO_CellularComponent-EBI-UniProt-GOA-ACAP-	1.51E-13	22.0339	39	[ANTXR1, ATR, CBX5, CDC73, CHEK1, CHEK2, HAT1, KDM1A, LRWD1, MACROH2A1, MCM2, MCM3, MCM4, MCM5, MCM6, MCM7, MMAB,	Upregulation

	ARAP_13.05.2021_00h00				MSH2, ORC2, ORC3, ORC4, ORC5, PARP1, POLD1, POLR1B, POLR2B, PRKDC, PURA, RAD51, RIF1, RPA1, RPA2, SIRT2, SSB, SUN2, TELO2, WRAP53, XRCC5, XRCC6]	
organelle fission	GO_BiologicalProcess-EBI-UniProt-GOA-ACAP-ARAP_13.05.2021_00h00	1.51E-13	13.02817	74	[AAAS, ACTR2, AP3B1, AURKA, AURKB, BCCIP, BUB1B, CALR, CCNB1, CDC42, CDCA8, CHEK1, CHEK2, CLASP1, CORO1C, CUL7, DLGAP5, DRG1, EMD, EPS8, FANCD2, GDAP1, GOLGA2, HSPA2, INCENP, IST1, KIF11, KIF22, KIF23, KIF2C, KNTC1, LBR, MACROH2A1, MARCHF5, MCU, MKI67, MLH1, MSH3, NCAPD2, NCAPG, NCAPG2, NDC1, NDC80, NDRG1, NSFL1C, NUP107, NUP133, NUP160, NUP35, NUSAP1, P3H4, PDGFRB, PDS5A, PHIP, PLK1, POGZ, PPP1R9B, PRKAR1A, RAD51, RAN, RANGAP1, RRS1, SEC13, SEH1L, SET, SIRT2, SMC2, TACC3, TOP2A, TPX2, TRIP13, TTK, VPS35, VRK1]	Upregulation
microtubule cytoskeleton organization involved in mitosis	GO_BiologicalProcess-EBI-UniProt-GOA-ACAP-ARAP_13.05.2021_00h00	1.51E-13	20.45455	45	[AAAS, AURKA, AURKB, BCCIP, BUB1B, CCNB1, CDCA8, CHEK2, CKAP5, CLASP1, DCTN1, DLGAP5, DRG1, DYNC1LI2, EML1, ERCC6L, FSD1, GOLGA2, HTT, INCENP, ITGB1, KIF11, KIF23, KIF2C, KNTC1, NDC80, NDRG1, NSFL1C, NUP107, NUP133, NUP160, NUSAP1, PLK1, RAN, RANBP2, RANGAP1, RCC2, SBDS, SEC13, SEH1L, SUN2, TACC3, TPX2, TTK, ZWILCH]	Upregulation
mitotic spindle organization	GO_BiologicalProcess-EBI-UniProt-GOA-ACAP-ARAP_13.05.2021_00h00	1.51E-13	20.5	41	[AAAS, AURKA, AURKB, BCCIP, BUB1B, CCNB1, CDCA8, CHEK2, CKAP5, CLASP1, DCTN1, DLGAP5, DRG1, DYNC1LI2, EML1, ERCC6L, FSD1, GOLGA2, INCENP, KIF11, KIF23, KIF2C, KNTC1, NDC80, NDRG1, NUP107, NUP133, NUP160, PLK1, RAN, RANBP2, RANGAP1, RCC2, SBDS, SEC13, SEH1L, SUN2, TACC3, TPX2, TTK, ZWILCH]	Upregulation
nuclear division	GO_BiologicalProcess-EBI-UniProt-GOA-	1.51E-13	13.10212	68	[AAAS, ACTR2, AURKA, AURKB, BCCIP, BUB1B, CALR, CCNB1, CDC42, CDCA8, CHEK1, CHEK2, CLASP1, CUL7, DLGAP5,	Upregulation

	ACAP-ARAP_13.05.2021_00h00				DRG1, EMD, EPS8, FANCD2, GOLGA2, HSPA2, INCENP, IST1, KIF11, KIF22, KIF23, KIF2C, KNTC1, LBR, MACROH2A1, MKI67, MLH1, MSH3, NCAPD2, NCAPG, NCAPG2, NDC1, NDC80, NDRG1, NSFL1C, NUP107, NUP133, NUP160, NUP35, NUSAP1, P3H4, PDGFRB, PDS5A, PHIP, PLK1, POGZ, PPP1R9B, PRKAR1A, RAD51, RAN, RANGAP1, RRS1, SEC13, SEH1L, SET, SIRT2, SMC2, TACC3, TOP2A, TPX2, TRIP13, TTK, VRK1]	
condensed chromosome	GO_CellularComponent-EBI-UniProt-GOA-ACAP-ARAP_13.05.2021_00h00	1.51E-13	20.07722	52	[ADD3, AURKB, BAZ1B, BUB1B, CCNB1, CHEK1, CKAP5, CLASP1, CTCF, DCTN1, DCTN4, DYNC1LI2, ERCC6L, FANCD2, HSPA2, INCENP, KIF22, KIF2C, KNTC1, LRPPRC, LRWD1, MACROH2A1, MKI67, MLH1, NCAPD2, NCAPG, NDC80, NOL6, NUP107, NUP133, NUP160, ORC2, P3H4, PLK1, RAD51, RANGAP1, RIF1, RRS1, SEC13, SEH1L, SEPTIN2, SEPTIN7, SIN3A, SKA3, SMARCA5, SMC2, SPOUT1, SUN2, TOP2A, TTK, WAPL, ZWILCH]	Upregulation
spindle	GO_CellularComponent-EBI-UniProt-GOA-ACAP-ARAP_13.05.2021_00h00	1.51E-13	17.33668	69	[AAAS, ACOT13, AKT1, ANXA11, ARL3, AURKA, AURKB, BCCIP, BIRC6, BUB1B, CCAR2, CCNB1, CDC42, CDCA8, CEP170, CKAP5, CLASP1, CRMP1, CTNNB1, DCTN1, DCTN4, DIAPH1, DLGAP5, ECT2, EMD, EML1, EPB41, ERCC2, GOLGA2, HSPA2, HSPB1, INCENP, KIF11, KIF20A, KIF22, KIF23, KIF2C, KNTC1, MAP1S, MAPK1, MAPK14, MICAL3, MYH9, NCAPG, NEK7, NPM1, NSFL1C, NSUN2, NUSAP1, PLK1, PYCR3, RANGAP1, RCC2, RIF1, SBDS, SEPTIN2, SEPTIN7, SIRT2, SKA3, SLC25A5, SPECC1L, SPOUT1, TACC3, TPX2, TTC28, TTK, TTLL12, VRK1, WAPL]	Upregulation
microtubule cytoskeleton	GO_CellularComponent-EBI-UniProt-GOA-ACAP-ARAP_13.05.2021_00h00	1.51E-13	12.61993	171	[AAAS, ACOT13, ACTR10, ACTR1B, AKT1, ANXA11, ARL2, ARL3, AURKA, AURKB, BAG2, BAIAP2, BCCIP, BIRC6, BUB1B, CCAR2, CCNB1, CCT2, CCT3, CCT4, CCT5, CCT6A, CCT7, CCT8, CD2AP, CDC42,	No change

					CDCA8, CDK5RAP3, CEP170, CFAP20, CHD4, CHEK1, CKAP5, CLASP1, CRMP1, CTNNB1, CTNNBL1, CTSC, CUL7, CYFIP1, DCAF13, DCTN1, DCTN4, DCXR, DHX9, DIAPH1, DLG1, DLGAP5, DPYSL2, DYNC1LI2, ECPAS, ECT2, EHD2, EIF3A, EMD, EML1, EPB41, ERCC2, FKBP4, FSD1, GOLGA2, HERC2, HK2, HMMR, HOOK3, HSPA2, HSPB1, HSPB11, HSPH1, HTT, INCENP, IQGAP1, IST1, JADE1, KEAP1, KIF11, KIF20A, KIF21A, KIF22, KIF23, KIF2C, KIF5B, KLC1, KNTC1, KRT18, LRPPRC, LRWD1, MACF1, MAP1B, MAP1LC3A, MAP1S, MAP4, MAPK1, MAPK14, MASTL, MCM3, METAP1, MICAL3, MVB12A, MYH9, NCAPG, NDC80, NDRG1, NEK7, NIT2, NPM1, NSFL1C, NSUN2, NUSAP1, ORC2, PEA15, PHIP, PIK3R4, PLEKHA7, PLK1, PPP2R5A, PRKACB, PRKAR1A, PRKAR2A, PRKAR2B, PRKCA, PXDN, PXK, PYCR3, RAB23, RAB8A, RABL6, RAD51, RAN, RANGAP1, RCC2, RIF1, ROCK2, RP2, RTTN, SARM1, SBDS, SCYL1, SEPTIN10, SEPTIN11, SEPTIN2, SEPTIN7, SEPTIN8, SIRT2, SKA3, SLAIN2, SLC25A5, SNTB2, SNX4, SORBS1, SPAG9, SPECC1L, SPOUT1, SPTAN1, SRPRB, STIM1, TACC3, TCP1, TMEM214, TOP2A, TPX2, TTC28, TTK, TTLL12, TUBA4A, TUBB6, UBR4, UPF3B, VIM, VRK1, WAPL]	
kinetochore	GO_CellularComponent-EBI-UniProt-GOA-ACAP-ARAP_13.05.2021_00h00	1.51E-13	21.12676	30	[AURKB, BUB1B, CCNB1, CKAP5, CLASP1, DCTN1, DCTN4, DYNC1LI2, ERCC6L, INCENP, KIF22, KIF2C, KNTC1, LRWD1, NDC80, NUP107, NUP133, NUP160, ORC2, PLK1, RANGAP1, SEC13, SEH1L, SEPTIN2, SEPTIN7, SIN3A, SKA3, SPOUT1, TTK, ZWILCH]	Upregulation
condensed chromosome, centromeric region	GO_CellularComponent-EBI-UniProt-GOA-ACAP-ARAP_13.0	1.51E-13	21.05263	32	[AURKB, BUB1B, CCNB1, CKAP5, CLASP1, DCTN1, DCTN4, DYNC1LI2, ERCC6L, INCENP, KIF22, KIF2C, KNTC1, LRWD1, NCAPD2, NCAPG, NDC80, NUP107,	Upregulation

	5.2021_00h00				NUP133, NUP160, ORC2, PLK1, RANGAP1, SEC13, SEH1L, SEPTIN2, SEPTIN7, SIN3A, SKA3, SPOUT1, TTK, ZWILCH]	
nuclear chromosome	GO_CellularComponent-EBI-UniProt-GOA-ACAP-ARAP_13.05.2021_00h00	1.51E-13	19.83806	49	[ADD3, ANP32E, BAZ1B, BRMS1, CHEK1, DHX9, GINS3, HDAC2, HSPA2, INCENP, INO80C, LRPPRC, LRWD1, MACROH2A1, MCM2, MCM3, MCM4, MCM5, MCM6, MCM7, MLH1, NCAPD2, NOL6, ORC2, ORC3, ORC4, ORC5, P3H4, PLK1, POLA1, POLA2, POLD1, POLE, POLR1B, PRIM1, PRIM2, RAD51, RPA1, RPA2, RPA3, RRS1, SIN3A, SMARCA5, SMARCAD1, SMC2, SUN2, TOP2A, WAPL, WDHD1]	Upregulation
mitotic spindle	GO_CellularComponent-EBI-UniProt-GOA-ACAP-ARAP_13.05.2021_00h00	1.51E-13	17.68293	29	[AAAS, AURKA, AURKB, BCCIP, CDC42, CEP170, CLASP1, DCTN1, DIAPH1, ECT2, EML1, EPB41, GOLGA2, KIF11, KIF22, KIF23, MAPK1, NCAPG, NUSAP1, PLK1, PYCR3, RANGAP1, RCC2, SIRT2, SKA3, SPOUT1, TACC3, TPX2, WAPL]	Upregulation
glycolytic process through glucose-6-phosphate	GO_BiologicalProcess-EBI-UniProt-GOA-ACAP-ARAP_13.05.2021_00h00	2.72E-42	34.21053	13	[ADPGK, ALDOC, ENO2, FOXK1, GALK1, GPI, HK1, HK2, HOOK3, PFKL, PFKM, PFKP, PKM]	No change
carbohydrate metabolic process	GO_BiologicalProcess-EBI-UniProt-GOA-ACAP-ARAP_13.05.2021_00h00	2.72E-42	14.76608	101	[AAAS, ABHD10, ADPGK, AGL, AKR7A2, AKT1, ALDH2, ALDOC, AMDHD2, AP2A1, B3GLCT, B4GALT1, BPNT2, BRAT1, CHID1, CHST14, CYB5R3, DCXR, DHX8, EDEM3, ENO2, EPM2AIP1, FOXK1, GALE, GALK1, GALM, GBE1, GCLC, GLA, GLB1L3, GNE, GNPDA2, GOLT1B, GOT2, GPD1L, GPI, GYS1, H6PD, HDAC4, HEXA, HK1, HK2, HOOK3, IDH2, ITPK1, LANCL2, LCMT1, LDHB, MAN1A1, MAN1A2, MANBA, MAPK14, ME1, MGAT2, MOGS, NDC1, NFKB1, NLN, NUP107, NUP133, NUP160, NUP210, NUP35, NUP50, OGT, PASK, PC, PFKL, PFKM, PFKP, PGM2L1, PGM3, PIK3CA, PKM, POFUT1, POFUT2, PRKAA1, PRKAG1, PTPN2, RANBP2, SBDS, SCARB2, SEC13, SEH1L, SESN2, SHPK, SIRT1, SLC25A12,	No change

					SLC2A1, SLC5A3, SORBS1, SORD, SRC, TELO2, TIGAR, TKFC, TKT, TPD52L2, UGDH, UGP2, USP7]	
generation of precursor metabolites and energy	GO_BiologicalProcess-EBI-UniProt-GOA-ACAP-ARAP_13.05.2021_00h00	2.72E-42	13.33333	78	[AAAS, ACAT1, ACO1, ACSS2, ACSS3, ADPGK, ADSL, AGL, AK4, AKR7A2, AKT1, ALDH2, ALDOC, ASPH, BDH2, CAT, CCNB1, DEGS1, ENO2, EPM2AIP1, ETFA, FAHD1, FDXR, FECH, FH, FOXK1, GALK1, GBE1, GFPT2, GPI, GYS1, H6PD, HDAC4, HK1, HK2, HMGCL, HOOK3, IDH2, ME1, MSH2, MYBBP1A, NDC1, NNT, NQO2, NUP107, NUP133, NUP160, NUP210, NUP35, NUP50, OGT, OXCT1, P4HA2, PARD3, PASK, PFKL, PFKM, PFKP, PGM2L1, PIK3CA, PKM, PNPT1, POR, PRKAA1, PRKAG1, QDPR, RANBP2, SEC13, SEH1L, SHPK, SLC25A12, SORBS1, SORD, SUCLG1, TIGAR, TKT, TRAP1, UGP2]	No change
phosphorus metabolic process	GO_BiologicalProcess-EBI-UniProt-GOA-ACAP-ARAP_13.05.2021_00h00	2.72E-42	11.07492	340	[AAAS, AASS, ABHD12, ABHD14B, ABI1, ACACA, ACAT1, ACLY, ACOT13, ACOT9, ACSL1, ACSL4, ACSS2, ADAM10, ADAR, ADARB1, ADPGK, ADSL, AK1, AK4, AKT1, ALDOC, ALPL, AMDHD2, AMPD2, ANP32E, ANTXR1, ANXA2, APOE, APRT, ATIC, ATP1A1, ATP2B4, ATP6V1A, ATR, AURKA, AURKB, BAZ1B, BCCIP, BIRC6, BPNT2, BRAT1, BRD4, BUB1B, CAMK1, CAMK2D, CAPN2, CASK, CASP3, CAV1, CBL, CCNB1, CCND2, CCNH, CCNL2, CCNY, CD44, CD81, CDC42, CDC42BPA, CDC42BPB, CDK5RAP1, CDK5RAP3, CDK7, CHEK1, CHEK2, CLASP1, CMPK1, COPS2, CORO1C, CSPG4, CTPS1, DAPK1, DAXX, DBNL, DCAF1, DCTD, DCXR, DDRGK1, DGKA, DHX8, DIPK2A, DLG1, DOCK7, DUSP3, ECT2, EFL1, EGFR, EHD4, ELP3, ENO2, ENOPH1, EPHB3, ETFA, ETNK1, FDPS, FERMT2, FKBP8, FLAD1, FLT1, FN1, FOXK1, FXR1, GALK1, GARS1, GART, GATA6, GCDH, GFPT2, GLMN, GMPS, GNE, GNPDA2, GPD1L, GPHN, GPI, GTPBP1, H6PD, HADHA, HADHB,	No change

					HDAC2, HDAC4, HDHD5, HK1, HK2, HMGCL, HOOK3, HSD17B4, HSP90AB1, HSPA2, HSPB1, HTT, IDH2, IDI1, ILF3, ILK, IMPDH1, IMPDH2, INCENP, IPO5, IQGAP1, IQGAP3, ITGA5, ITGA6, ITPK1, LPCAT1, LPGAT1, LRRC40, LRRK1, LTBP1, MACROH2A1, MAGED1, MAP2K6, MAPK1, MAPK14, MAPK8, MASTL, MAVS, MCCC2, MCM2, MCM7, ME1, MICAL1, MMAB, MOCS2, MPC2, MTHFD1, MTMR14, MTMR6, MVD, MVK, MYADM, MYDGF, NDC1, NEK7, NIBAN1, NNT, NOL9, NPM1, NRP2, NT5C2, NT5DC1, NT5DC3, NTHL1, NUDT16, NUP107, NUP133, NUP160, NUP210, NUP35, NUP50, OCLN, OGT, ORC3, OSBP, PAICS, PAK1, PALD1, PAPSS2, PARD3, PARP1, PARVA, PASK, PCYT1A, PCYT2, PDGFRB, PDXK, PEA15, PFAS, PFKL, PFKM, PFKP, PGAM5, PGM2L1, PGM3, PHIP, PHPT1, PI4KA, PIGS, PIGT, PIK3C2A, PIK3CA, PIK3R4, PIP4K2B, PIP4K2C, PKM, PLCB3, PLCG1, PLK1, PLPP3, PLSCR3, PLXNB2, PNP, POLB, PPM1B, PPM1F, PPM1G, PPP1R7, PPP1R9B, PPP2R5A, PPP4R1, PRDX4, PRKAA1, PRKAB1, PRKACB, PRKACG, PRKAG1, PRKAR1A, PRKAR2A, PRKAR2B, PRKCA, PRKCSH, PRKDC, PRPSAP1, PRRC1, PRUNE1, PTGIS, PTK7, PTPMT1, PTPN1, PTPN12, PTPN2, PTPRD, PXK, PYCARD, RAB14, RAB23, RAD51, RAN, RANBP2, RAP2A, RAP2B, RAP2C, RBPMS, ROCK2, RPRD1A, RPS6KA1, RPS6KA3, RPS6KA4, SAMHD1, SARM1, SBF1, SCYL1, SEC13, SEH1L, SEPHS1, SESN2, SET, SHPK, SIRT1, SIRT2, SKP2, SLC25A12, SLC44A1, SLC44A2, SLK, SMPDL3B, SNX6, SNX9, SORD, SPAG9, SPTBN1, SPTLC2, SRC, SRPK1, STK38, STYX, SYMPK, TBPL1, TELO2, TF, THBS1, TIGAR, TIMM50,	

					TIPRL, TJP2, TKFC, TKT, TLK1, TMED2, TOLLIP, TPST1, TPX2, TRIM28, TRPT1, TTK, TWF1, UAP1, UGDH, UGGT1, UGGT2, UGP2, UNG, USP15, VLDLR, VPS25, VRK1, WARS1, XRCC5, XRCC6]	
glycosyltransferase activity	GO_MolecularFunction-EBI-UniProt-GOA-ACAP-ARAP_13.05.2021_00h00	2.72E-42	14.61039	45	[AGL, ALG11, ALG2, ALG5, ALG9, APRT, B3GLCT, B4GALT1, CERCAM, CHPF, COLGALT1, EPM2AIP1, FUT11, GALNT1, GALNT10, GALNT2, GBE1, GLT8D1, GXYLT1, GYS1, HEXA, MGAT2, MTAP, OGT, OSTC, PARP1, PLOD1, PLOD2, PLOD3, PNP, POFUT1, POFUT2, POGLUT2, POGLUT3, PSMD2, RPN1, SDF2L1, SIRT1, SIRT2, STT3A, STT3B, TRPT1, UGGT1, UGGT2, ZC3HAV1]	Downregulation
carbohydrate derivative metabolic process	GO_BiologicalProcess-EBI-UniProt-GOA-ACAP-ARAP_13.05.2021_00h00	2.72E-42	13.80991	170	[AAAS, AASS, AATF, ABHD10, ABHD14B, ACACA, ACAT1, ACLY, ACOT13, ACOT9, ACSL1, ACSL4, ACSS2, ADPGK, ADSL, AGO2, AK1, AK4, AKR7A2, AKT1, ALDOC, ALG11, ALG2, ALG5, ALG9, AMDHD2, AMPD2, AP2A1, APOBEC3C, APRT, ARFGEF1, ARSA, ARSB, ATIC, ATP6V1A, B3GLCT, B4GALT1, BPNT2, CASK, CD44, CHPF, CHST14, CMPK1, COG7, CSPG4, CTNNB1, CTPS1, DCTD, DCXR, DLG1, EDEM3, EFL1, ENO2, ERP44, ESYT2, FBXO2, FOXK1, FUT11, GALK1, GALNT1, GALNT10, GALNT2, GART, GCDH, GFPT2, GLA, GMPPB, GMPS, GNE, GNPDA2, GNS, GOLGA2, GPC1, GPC3, GPC6, GPD1L, GPI, GTPBP1, GXYLT1, H6PD, HDAC4, HEXA, HK1, HK2, HMGCL, HMMR, HOOK3, HSD17B4, IMPDH1, IMPDH2, LMAN1, MAN1A1, MAN1A2, MANBA, MCCC2, MGAT2, MOGS, MPC2, MTAP, MVD, MVK, NDC1, NFKB1, NT5C2, NTHL1, NUDT16, NUP107, NUP133, NUP160, NUP210, NUP35, NUP50, OGT, OSTC, PAICS, PAPSS2, PARP1, PAWR, PDGFRB, PFAS, PFKL, PFKM, PFKP, PGM2L1, PGM3, PIGS, PIGT, PKM, PLOD1,	Downregulation

					PLOD2, PLOD3, PNP, POFUT1, POFUT2, POGLUT2, POGLUT3, PRKAA1, PRKAG1, PRKCSH, PRPSAP1, PSMD2, RAB1A, RAB23, RAN, RANBP2, RPN1, SAMHD1, SDF2L1, SEC13, SEH1L, SEL1L, SHPK, SLC25A12, SLC2A10, SORD, STT3A, STT3B, SUMF2, TBPL1, TIGAR, TJP2, TKFC, TKT, TPST1, UAP1, UGDH, UGGT1, UGGT2, UGP2, UNG]	
monosaccharide metabolic process	GO_BiologicalProcess-EBI-UniProt-GOA-ACAP-ARAP_13.05.2021_00h00	2.72E-42	16.34615	51	[AKT1, ALDOC, B3GLCT, B4GALT1, BRAT1, CYB5R3, DCXR, ENO2, EPM2AIP1, FOXK1, GALE, GALK1, GALM, GCLC, GOLT1B, GOT2, GPI, H6PD, HK1, HK2, HOOK3, LCMT1, MAPK14, NLN, OGT, PASK, PC, PFKL, PFKM, PFKP, PGM2L1, PIK3CA, PKM, POFUT1, POFUT2, PRKAA1, PTPN2, RANBP2, SBDS, SESN2, SIRT1, SLC25A12, SLC2A1, SORBS1, SORD, SRC, TELO2, TIGAR, TKFC, TKT, USP7]	No change
organophosphate metabolic process	GO_BiologicalProcess-EBI-UniProt-GOA-ACAP-ARAP_13.05.2021_00h00	2.72E-42	12.93706	148	[AAAS, AASS, ABHD12, ABHD14B, ACACA, ACAT1, ACLY, ACOT13, ACOT9, ACSL1, ACSL4, ACSS2, ADPGK, ADSL, AK1, AK4, ALDOC, AMPD2, APRT, ATIC, ATP6V1A, BPNT2, CAPN2, CASK, CD81, CDC42, CHEK2, CMPK1, CTPS1, DCTD, DCXR, DGKA, DHX8, DLG1, EFL1, ENO2, ETNK1, FDPS, FLAD1, FLT1, FOXK1, GALK1, GARS1, GART, GATA6, GCDH, GFPT2, GMPS, GPD1L, GPHN, GPI, GTPBP1, H6PD, HADHA, HADHB, HDAC4, HDHD5, HK1, HK2, HMGCL, HOOK3, HSD17B4, IDH2, IDI1, IMPDH1, IMPDH2, ITPK1, LPCAT1, LPGAT1, MAPK1, MCCC2, ME1, MOCS2, MPC2, MTHFD1, MTMR14, MTMR6, MVD, MVK, NDC1, NT5C2, NTHL1, NUDT16, NUP107, NUP133, NUP160, NUP210, NUP35, NUP50, OGT, PAICS, PAPSS2, PARP1, PCYT1A, PCYT2, PDGFRB, PDXK, PFAS, PFKL, PFKM, PFKP, PGM2L1, PGM3, PI4KA, PIGS, PIGT, PIK3C2A, PIK3CA, PIK3R4, PIP4K2B, PIP4K2C, PKM,	No change

					PLCB3, PLCG1, PLPP3, PLSCR3, PNP, PRKAA1, PRKAG1, PRKCSH, PRPSAP1, PTGIS, PTPMT1, RAB14, RAB23, RAN, RANBP2, SAMHD1, SARM1, SBF1, SEC13, SEH1L, SHPK, SLC25A12, SLC44A1, SLC44A2, SMPDL3B, SORD, SPTLC2, SRC, TBPL1, TIGAR, TJP2, TKFC, TKT, TPST1, UGP2, UNG]	
phosphate-containing compound metabolic process	GO_BiologicalProcess-EBI-UniProt-GOA-ACAP-ARAP_13.05.2021_00h00	2.72E-42	10.97601	334	[AAAS, AASS, ABHD12, ABHD14B, ABI1, ACACA, ACAT1, ACLY, ACOT13, ACOT9, ACSL1, ACSL4, ACSS2, ADAM10, ADAR, ADARB1, ADPGK, ADSL, AK1, AK4, AKT1, ALDOC, ALPL, AMPD2, ANP32E, ANTXR1, ANXA2, APOE, APRT, ATIC, ATP1A1, ATP2B4, ATP6V1A, ATR, AURKA, AURKB, BAZ1B, BCCIP, BIRC6, BPNT2, BRAT1, BRD4, BUB1B, CAMK1, CAMK2D, CAPN2, CASK, CASP3, CAV1, CBL, CCNB1, CCND2, CCNH, CCNL2, CCNY, CD44, CD81, CDC42, CDC42BPA, CDC42BPB, CDK5RAP1, CDK5RAP3, CDK7, CHEK1, CHEK2, CLASP1, CMPK1, COPS2, CORO1C, CSPG4, CTPS1, DAPK1, DAXX, DBNL, DCAF1, DCTD, DCXR, DDRGK1, DGKA, DHX8, DIPK2A, DLG1, DOCK7, DUSP3, ECT2, EFL1, EGFR, EHD4, ELP3, ENO2, ENOPH1, EPHB3, ETFA, ETNK1, FDPS, FERMT2, FKBP8, FLAD1, FLT1, FN1, FOXK1, FXR1, GALK1, GARS1, GART, GATA6, GCDH, GFPT2, GLMN, GMPS, GNE, GPD1L, GPHN, GPI, GTPBP1, H6PD, HADHA, HADHB, HDAC2, HDAC4, HDHD5, HK1, HK2, HMGCL, HOOK3, HSD17B4, HSP90AB1, HSPA2, HSPB1, HTT, IDH2, IDI1, ILF3, ILK, IMPDH1, IMPDH2, INCENP, IPO5, IQGAP1, IQGAP3, ITGA5, ITGA6, ITPK1, LPCAT1, LPGAT1, LRRC40, LRRK1, LTBP1, MACROH2A1, MAGED1, MAP2K6, MAPK1, MAPK14, MAPK8, MASTL, MAVS, MCCC2, MCM2, MCM7, ME1, MICAL1, MMAB, MOCS2, MPC2, MTHFD1, MTMR14, MTMR6, MVD, MVK,	No change

					MYADM, MYDGF, NDC1, NEK7, NIBAN1, NNT, NOL9, NPM1, NRP2, NT5C2, NT5DC1, NT5DC3, NTHL1, NUDT16, NUP107, NUP133, NUP160, NUP210, NUP35, NUP50, OCLN, OGT, ORC3, OSBP, PAICS, PAK1, PALD1, PAPSS2, PARD3, PARP1, PARVA, PASK, PCYT1A, PCYT2, PDGFRB, PDXK, PEA15, PFAS, PFKL, PFKM, PFKP, PGAM5, PGM2L1, PGM3, PHIP, PHPT1, PI4KA, PIGS, PIGT, PIK3C2A, PIK3CA, PIK3R4, PIP4K2B, PIP4K2C, PKM, PLCB3, PLCG1, PLK1, PLPP3, PLSCR3, PLXNB2, PNP, POLB, PPM1B, PPM1F, PPM1G, PPP1R7, PPP1R9B, PPP2R5A, PPP4R1, PRDX4, PRKAA1, PRKAB1, PRKACB, PRKACG, PRKAG1, PRKAR1A, PRKAR2A, PRKAR2B, PRKCA, PRKCSH, PRKDC, PRPSAP1, PRRC1, PRUNE1, PTGIS, PTK7, PTPMT1, PTPN1, PTPN12, PTPN2, PTPRD, PXK, PYCARD, RAB14, RAB23, RAD51, RAN, RANBP2, RAP2A, RAP2B, RAP2C, RBPMS, ROCK2, RPRD1A, RPS6KA1, RPS6KA3, RPS6KA4, SAMHD1, SARM1, SBF1, SCYL1, SEC13, SEH1L, SEPHS1, SESN2, SET, SHPK, SIRT1, SIRT2, SKP2, SLC25A12, SLC44A1, SLC44A2, SLK, SMPDL3B, SNX6, SNX9, SORD, SPAG9, SPTBN1, SPTLC2, SRC, SRPK1, STK38, STYX, SYMPK, TBPL1, TELO2, TF, THBS1, TIGAR, TIMM50, TIPRL, TJP2, TKFC, TKT, TLK1, TMED2, TOLLIP, TPST1, TPX2, TRIM28, TRPT1, TTK, TWF1, UGP2, UNG, USP15, VLDLR, VPS25, VRK1, WARS1, XRCC5, XRCC6]	
carbohydrate catabolic process	GO_BiologicalProcess-EBI-UniProt-GOA-ACAP-ARAP_13.05.2021_00h00	2.72E-42	17.72727	39	[AAAS, ABHD10, ADPGK, AGL, ALDOC, ENO2, FOXK1, GALE, GALK1, GALM, GPI, HDAC4, HK1, HK2, HOOK3, MANBA, NDC1, NUP107, NUP133, NUP160, NUP210, NUP35, NUP50, OGT, PFKL, PFKM, PFKP, PGM2L1, PKM, PRKAA1, PRKAG1, RANBP2, SCARB2, SEC13, SEH1L,	No change

					SLC25A12, SORD, TIGAR, TKFC]	
hexosyltransferase activity	GO_MolecularFunction-EBI-UniProt-GOA-ACAP-ARAP_13.05.2021_00h00	2.72E-42	15.9292	36	[AGL, ALG11, ALG2, ALG5, ALG9, B3GLCT, B4GALT1, CERCAM, CHPF, COLGALT1, EPM2AIP1, FUT11, GALNT1, GALNT10, GALNT2, GBE1, GYS1, HEXA, MGAT2, MTAP, OGT, OSTC, PLOD1, PLOD2, PLOD3, POFUT1, POFUT2, POGLUT2, POGLUT3, PSMD2, RPN1, SDF2L1, STT3A, STT3B, UGGT1, UGGT2]	Downregulation
purine-containing compound metabolic process	GO_BiologicalProcess-EBI-UniProt-GOA-ACAP-ARAP_13.05.2021_00h00	2.72E-42	15.84158	80	[AAAS, AASS, ABHD14B, ACACA, ACAT1, ACLY, ACOT13, ACOT9, ACSL1, ACSL4, ACSS2, ADPGK, ADSL, AK1, AK4, ALDOC, AMPD2, APRT, ATIC, ATP6V1A, BPNT2, CASK, DLG1, EFL1, ENO2, FOXK1, GALK1, GART, GCDH, GMPS, GPI, GTPBP1, HDAC4, HK1, HK2, HMGCL, HOOK3, HSD17B4, IMPDH1, IMPDH2, KDM1A, MCCC2, MPC2, MTAP, MTHFD1, MVD, MVK, NDC1, NT5C2, NUDT16, NUP107, NUP133, NUP160, NUP210, NUP35, NUP50, OGT, PAICS, PAPSS2, PARP1, PFAS, PFKL, PFKM, PFKP, PKM, PNP, PRKAA1, PRKAG1, PRPSAP1, RAB23, RAN, RANBP2, SAMHD1, SEC13, SEH1L, SLC25A12, SORD, TIGAR, TJP2, TPST1]	No change
carbohydrate derivative biosynthetic process	GO_BiologicalProcess-EBI-UniProt-GOA-ACAP-ARAP_13.05.2021_00h00	2.72E-42	13.44307	98	[AATF, ACACA, ACAT1, ACLY, ACSL1, ACSL4, ACSS2, ADSL, AGO2, AK1, AK4, ALG11, ALG2, ALG5, ALG9, AMDHD2, AMPD2, AP2A1, APRT, ARFGEF1, ATIC, ATP6V1A, B3GLCT, B4GALT1, CHPF, CHST14, CMPK1, COG7, CSPG4, CTNNB1, CTPS1, DCTD, DCXR, EDEM3, FUT11, GALNT1, GALNT10, GALNT2, GART, GCDH, GFPT2, GMPPB, GMPS, GNE, GNPDA2, GOLGA2, GPC1, GPC3, GPC6, GXYLT1, HEXA, IMPDH1, IMPDH2, LMAN1, MAN1A1, MAN1A2, MGAT2, MOGS, MPC2, MTAP, MVD, NFKB1, OGT, OSTC, PAICS, PAPSS2, PARP1, PAWR, PDGFRB, PFAS, PGM3, PIGS, PIGT, PLOD1, PLOD2, PLOD3, PNP, POFUT1,	Downregulation

					POFUT2, POGLUT2, POGLUT3, PRKAA1, PRPSAP1, PSMD2, RPN1, SDF2L1, SLC25A12, SLC2A10, SORD, STT3A, STT3B, TBPL1, TKT, UAP1, UGDH, UGGT1, UGGT2, UGP2]	
organonitrogen compound biosynthetic process	GO_BiologicalProcess-EBI-UniProt-GOA-ACAP-ARAP_13.05.2021_00h00	2.72E-42	13.82979	260	[AASS, AATF, ABAT, ABCB10, ABCB7, ABCF1, ACACA, ACAT1, ACLY, ACO1, ACSL1, ACSL4, ACSS2, ADSL, AGO1, AGO2, AIMP2, AK1, AK4, AKT1, ALDH7A1, ALG11, ALG2, ALG5, ALG9, AMPD2, AP2A1, APEH, APOE, APRT, ARFGEF1, ASL, ASNS, ATIC, ATP2B4, ATP6V1A, B3GLCT, B4GALT1, BCAT2, BDH2, C1QBP, CALR, CAPN2, CAPRIN1, CDC123, CDK5RAP1, CELF1, CHEK2, CHPF, CHST14, CIRBP, CMPK1, CNDP2, CNOT11, COG7, COPS5, CPOX, CSDE1, CSPG4, CTNNB1, CTPS1, CYFIP1, DAPK1, DARS2, DCTD, DDX39B, DDX6, DEGS1, DHPS, DHX33, DHX9, DPH1, DPH2, DPH6, DRG1, EDEM3, EEF1E1, EEF2, EFL1, EGFR, EIF3A, EIF4A3, EIF5B, ENOPH1, ESD, EXOSC2, EXOSC5, EXOSC7, FASTKD2, FECH, FLAD1, FUT11, FXR1, GALNT1, GALNT10, GALNT2, GARS1, GART, GCDH, GCLC, GCLM, GEMIN5, GFPT2, GLS, GLUD1, GLUL, GMPS, GOLGA2, GOLT1B, GOT2, GPC1, GPC3, GPC6, GPHN, GSS, GSTK1, GSTM2, GSTM3, GTPBP1, GUF1, GXYLT1, HAGH, HEXA, HMBS, HSPB1, IDH2, IGF2BP3, ILF3, ILVBL, IMPDH1, IMPDH2, LARS1, LARS2, LIN28A, LMAN1, LPCAT1, LRPPRC, LSM4, MAN1A1, MAN1A2, MAPK1, MARS1, METAP1, MGAT2, MGST1, MLH1, MOCS2, MOGS, MOXD1, MPC2, MRPS27, MRPS9, MTAP, MTHFD1, MTR, MVD, NCL, NDRG1, NFKB1, NIBAN1, NMT2, NPM1, NSUN5, OGT, OPLAH, OSTC, PAICS, PAPSS2, PARN, PARP1, PASK, PAWR, PCYT1A, PDGFRB, PDXK, PFAS, PGM3, PIGS, PIGT, PKM, PLOD1, PLOD2,	No change

					PLOD3, PLPP3, PLXNB2, PNP, PNPT1, POFUT1, POFUT2, POGLUT2, POGLUT3, POLR2G, PPM1B, PRKAA1, PRKCSH, PRKDC, PRPSAP1, PSMD2, PTCD3, PTGIS, PURA, PUS7, PYCR1, PYCR2, PYCR3, QDPR, QRSL1, RBM3, ROCK2, RPL13A, RPL22, RPL27A, RPL4, RPL5, RPL7A, RPN1, RPS2, RPS21, RPS6KA1, RPS6KA3, RPS8, RRBP1, SAMD4B, SARS1, SARS2, SBDS, SDF2L1, SEPHS1, SESN2, SLC1A3, SLC25A12, SLC2A10, SLC44A1, SLC44A2, SMS, SPR, SPTLC2, SRM, SRR, STT3A, STT3B, TARS2, TBPL1, THBS1, TIA1, TMED2, TRAP1, TRIM71, TRMT10C, UGDH, UGGT1, UGGT2, UPF3B, VIM, WARS1, XRN1, YARS2, ZC3H15, ZDHHC17, ZFP36L2]	
glycoprotein metabolic process	GO_BiologicalProcess-EBI-UniProt-GOA-ACAP-ARAP_13.05.2021_00h00	2.72E-42	13.45708	58	[AATF, AGO2, ALG11, ALG2, ALG5, ALG9, ARFGEF1, ARSB, B3GLCT, B4GALT1, BPNT2, CHPF, CHST14, COG7, CSPG4, CTNNB1, EDEM3, ERP44, FBXO2, FUT11, GALNT1, GALNT10, GALNT2, GFPT2, GOLGA2, GPC1, GXYLT1, HEXA, LMAN1, MAN1A1, MAN1A2, MANBA, MGAT2, MOGS, MVD, OGT, OSTC, PAWR, PGM3, PLOD1, PLOD2, PLOD3, POFUT1, POFUT2, POGLUT2, POGLUT3, PRKCSH, PSMD2, RAB1A, RPN1, SDF2L1, SEL1L, SLC2A10, STT3A, STT3B, UGDH, UGGT1, UGGT2]	Downregulation
hexose metabolic process	GO_BiologicalProcess-EBI-UniProt-GOA-ACAP-ARAP_13.05.2021_00h00	2.72E-42	16.49123	47	[AKT1, ALDOC, B3GLCT, B4GALT1, BRAT1, DCXR, ENO2, EPM2AIP1, FOXK1, GALE, GALK1, GALM, GOLT1B, GOT2, GPI, H6PD, HK1, HK2, HOOK3, LCMT1, MAPK14, NLN, OGT, PASK, PC, PFKL, PFKM, PFKP, PGM2L1, PIK3CA, PKM, POFUT1, POFUT2, PRKAA1, PTPN2, RANBP2, SBDS, SESN2, SIRT1, SLC25A12, SORBS1, SORD, SRC, TELO2, TIGAR, TKFC, USP7]	No change
ribose phosphate metabolic process	GO_BiologicalProcess-EBI-UniProt-GOA-	2.72E-42	15.8215	78	[AAAS, AASS, ABHD14B, ACACA, ACAT1, ACLY, ACOT13, ACOT9, ACSL1, ACSL4, ACSS2, ADPGK, ADSL, AK1, AK4, ALDOC,	No change

	ACAP-ARAP_13.05.2021_00h00				AMPD2, APRT, ATIC, ATP6V1A, BPNT2, CASK, CMPK1, CTPS1, DLG1, EFL1, ENO2, FOXK1, GALK1, GART, GCDH, GMPS, GPI, GTPBP1, HDAC4, HK1, HK2, HMGCL, HOOK3, HSD17B4, IMPDH1, IMPDH2, MCCC2, MPC2, MVD, MVK, NDC1, NT5C2, NUDT16, NUP107, NUP133, NUP160, NUP210, NUP35, NUP50, OGT, PAICS, PAPSS2, PARP1, PFAS, PFKL, PFKM, PFKP, PKM, PRKAA1, PRKAG1, PRPSAP1, RAB23, RAN, RANBP2, SEC13, SEH1L, SLC25A12, SORD, TIGAR, TJP2, TKT, TPST1]	
organophosphate biosynthetic process	GO_BiologicalProcess-EBI-UniProt-GOA-ACAP-ARAP_13.05.2021_00h00	2.72E-42	11.92146	85	[ACACA, ACAT1, ACLY, ACSL1, ACSL4, ACSS2, ADSL, AK1, AK4, AMPD2, APRT, ATIC, ATP6V1A, BPNT2, CAPN2, CASK, CD81, CDC42, CHEK2, CMPK1, CTPS1, DCTD, DCXR, DGKA, DHX8, DLG1, ETNK1, FDPS, FLAD1, FLT1, GARS1, GART, GCDH, GMPS, GPD1L, GPHN, HDHD5, IDH2, IDI1, IMPDH1, IMPDH2, LPCAT1, LPGAT1, MAPK1, ME1, MOCS2, MPC2, MTHFD1, MTMR14, MTMR6, MVD, MVK, PAICS, PAPSS2, PARP1, PCYT1A, PCYT2, PDGFRB, PDXK, PFAS, PI4KA, PIGS, PIGT, PIK3C2A, PIK3CA, PIK3R4, PIP4K2B, PIP4K2C, PLSCR3, PNP, PRKCSH, PRPSAP1, PTGIS, PTPMT1, RAB14, SBF1, SLC25A12, SLC44A1, SLC44A2, SORD, SPTLC2, SRC, TBPL1, TJP2, TKT]	No change
glucose metabolic process	GO_BiologicalProcess-EBI-UniProt-GOA-ACAP-ARAP_13.05.2021_00h00	2.72E-42	16.39344	40	[AKT1, ALDOC, BRAT1, DCXR, ENO2, EPM2AIP1, FOXK1, GALM, GOLT1B, GOT2, GPI, H6PD, HK1, HK2, HOOK3, LCMT1, MAPK14, NLN, OGT, PASK, PC, PFKL, PFKM, PFKP, PGM2L1, PIK3CA, PKM, PRKAA1, PTPN2, RANBP2, SBDS, SESN2, SIRT1, SLC25A12, SORBS1, SORD, SRC, TELO2, TIGAR, USP7]	No change
hexose catabolic process	GO_BiologicalProcess-EBI-UniProt-GOA-	2.72E-42	28.125	18	[ALDOC, ENO2, FOXK1, GALE, GALK1, GALM, GPI, HK1, HK2, HOOK3, PFKL, PFKM, PFKP,	No change

	ACAP-ARAP_13.05.2021_00h00				PGM2L1, PKM, SLC25A12, TIGAR, TKFC]	
nucleotide metabolic process	GO_BiologicalProcess-EBI-UniProt-GOA-ACAP-ARAP_13.05.2021_00h00	2.72E-42	15.66265	91	[AAAS, AASS, ABHD14B, ACACA, ACAT1, ACLY, ACOT13, ACOT9, ACSL1, ACSL4, ACSS2, ADPGK, ADSL, AK1, AK4, ALDOC, AMPD2, APRT, ATIC, ATP6V1A, BPNT2, CASK, CMPK1, CTPS1, DCTD, DLG1, EFL1, ENO2, FLAD1, FOXK1, GALK1, GARS1, GART, GCDH, GMPS, GPI, GTPBP1, HDAC4, HK1, HK2, HMGCL, HOOK3, HSD17B4, IDH2, IMPDH1, IMPDH2, MAPK1, MCCC2, ME1, MPC2, MTHFD1, MVD, MVK, NDC1, NT5C2, NTHL1, NUDT16, NUP107, NUP133, NUP160, NUP210, NUP35, NUP50, OGT, PAICS, PAPSS2, PARP1, PFAS, PFKL, PFKM, PFKP, PKM, PNP, PRKAA1, PRKAG1, PRPSAP1, PTGIS, RAB23, RAN, RANBP2, SAMHD1, SARM1, SEC13, SEH1L, SLC25A12, SORD, TBPL1, TIGAR, TJP2, TPST1, UNG]	No change
glucose catabolic process	GO_BiologicalProcess-EBI-UniProt-GOA-ACAP-ARAP_13.05.2021_00h00	2.72E-42	29.54545	13	[ALDOC, ENO2, FOXK1, GPI, HK1, HK2, HOOK3, PFKL, PFKM, PFKP, PKM, SLC25A12, TIGAR]	No change
purine nucleotide metabolic process	GO_BiologicalProcess-EBI-UniProt-GOA-ACAP-ARAP_13.05.2021_00h00	2.72E-42	16.1157	78	[AAAS, AASS, ABHD14B, ACACA, ACAT1, ACLY, ACOT13, ACOT9, ACSL1, ACSL4, ACSS2, ADPGK, ADSL, AK1, AK4, ALDOC, AMPD2, APRT, ATIC, ATP6V1A, BPNT2, CASK, DLG1, EFL1, ENO2, FOXK1, GALK1, GART, GCDH, GMPS, GPI, GTPBP1, HDAC4, HK1, HK2, HMGCL, HOOK3, HSD17B4, IMPDH1, IMPDH2, MCCC2, MPC2, MTHFD1, MVD, MVK, NDC1, NT5C2, NUDT16, NUP107, NUP133, NUP160, NUP210, NUP35, NUP50, OGT, PAICS, PAPSS2, PARP1, PFAS, PFKL, PFKM, PFKP, PKM, PNP, PRKAA1, PRKAG1, PRPSAP1, RAB23, RAN, RANBP2, SAMHD1, SEC13, SEH1L, SLC25A12,	No change

					SORD, TIGAR, TJP2, TPST1]	
ribonucleotide metabolic process	GO_BiologicalProcess-EBI-UniProt-GOA-ACAP-ARAP_13.05.2021_00h00	2.72E-42	15.83333	76	[AAAS, AASS, ABHD14B, ACACA, ACAT1, ACLY, ACOT13, ACOT9, ACSL1, ACSL4, ACSS2, ADPGK, ADSL, AK1, AK4, ALDOC, AMPD2, APRT, ATIC, ATP6V1A, BPNT2, CASK, CMPK1, CTPS1, DLG1, EFL1, ENO2, FOXK1, GALK1, GART, GCDH, GMPS, GPI, GTPBP1, HDAC4, HK1, HK2, HMGCL, HOOK3, HSD17B4, IMPDH1, IMPDH2, MCCC2, MPC2, MVD, MVK, NDC1, NT5C2, NUDT16, NUP107, NUP133, NUP160, NUP210, NUP35, NUP50, OGT, PAICS, PAPSS2, PARP1, PFAS, PFKL, PFKM, PFKP, PKM, PRKAA1, PRKAG1, RAB23, RAN, RANBP2, SEC13, SEH1L, SLC25A12, SORD, TIGAR, TJP2, TPST1]	No change
purine ribonucleotide metabolic process	GO_BiologicalProcess-EBI-UniProt-GOA-ACAP-ARAP_13.05.2021_00h00	2.72E-42	15.94828	74	[AAAS, AASS, ABHD14B, ACACA, ACAT1, ACLY, ACOT13, ACOT9, ACSL1, ACSL4, ACSS2, ADPGK, ADSL, AK1, AK4, ALDOC, AMPD2, APRT, ATIC, ATP6V1A, BPNT2, CASK, DLG1, EFL1, ENO2, FOXK1, GALK1, GART, GCDH, GMPS, GPI, GTPBP1, HDAC4, HK1, HK2, HMGCL, HOOK3, HSD17B4, IMPDH1, IMPDH2, MCCC2, MPC2, MVD, MVK, NDC1, NT5C2, NUDT16, NUP107, NUP133, NUP160, NUP210, NUP35, NUP50, OGT, PAICS, PAPSS2, PARP1, PFAS, PFKL, PFKM, PFKP, PKM, PRKAA1, PRKAG1, RAB23, RAN, RANBP2, SEC13, SEH1L, SLC25A12, SORD, TIGAR, TJP2, TPST1]	No change
intrinsic component of membrane	GO_CellularComponent-EBI-UniProt-GOA-ACAP-ARAP_13.05.2021_00h00	0.079766	4.96348	299	[ABCB10, ABCB7, ABCC1, ABHD12, ACSL1, ACSL4, ADAM10, ADPGK, ALCAM, ALG11, ALG2, ALG5, ALG9, ALPL, ANO6, ANTXR1, APMAP, APOOL, ARF4, ARHGAP1, ARL6IP5, ARSA, ASPH, ATL2, ATL3, ATP1A1, ATP2B1, ATP2B2, ATP2B4, AUP1, B3GLCT, B4GALT1, BCAT2, BET1, BPNT2, CALR, CANX, CAPZA2, CASK, CAV1, CD44, CD81, CDH2, CDH3, CEP170, CHEK2, CHPF, CHST14, CKAP4, CNTFR, CPD, CPOX, CRLF3, CSPG4, CXADR, CYB5R1,	Downregulation

					CYP2S1, CYP51A1, DAGLB, DAXX, DEGS1, DHRS7B, DHX15, DHX8, ECE1, ECHDC1, EEF1E1, EFNB2, EGFR, EHMT1, EMD, EPB41, EPB41L3, EPCAM, EPHB3, EPS8, ERAP1, ERGIC1, ERGIC2, ERLIN2, ESYT2, ETFA, EXD2, EXOG, F11R, FAT1, FDPS, FKBP11, FKBP8, FLNB, FLT1, FNDC3A, FNDC3B, FOCAD, FOXRED1, FTH1, FUT11, GALNT1, GALNT10, GALNT2, GARS1, GDAP1, GLMN, GLT8D1, GNL2, GOLIM4, GOLT1B, GOSR2, GPC1, GPC3, GPC6, GPHN, GPX8, GSDME, GTF3C3, GXYLT1, HDAC2, HLA-A, HMOX2, HSPA2, HSPA5, HSPA9, HSPD1, IKBIP, ILVBL, ITGA5, ITGA6, ITGAV, ITGB1, JAM3, KDELR1, KDELR3, KIRREL1, KTN1, L2HGDH, LBR, LMAN1, LMF2, LNPEP, LNPK, LPCAT1, LPGAT1, LRBA, LRP1, LRRC59, LRRC8A, MACF1, MAN1A1, MAN1A2, MARCHF5, MAVS, MCAM, MCU, MEMO1, MEST, MFSD10, MGAT2, MGST1, MICOS13, MIPEP, MME, MOGS, MOSPD2, MOXD1, MPC2, MRC2, MTDH, MYADM, NBAS, NCAM1, NCKAP1, NDC1, NDRG2, NECTIN2, NLGN4X, NNT, NPM3, NRP2, NSDHL, NUP210, NUP50, OCLN, ORC3, OSTC, P4HTM, PDGFRB, PDIA3, PGAM5, PGRMC2, PICALM, PIGS, PIGT, PKP2, PLD3, PLEKHA7, PLPP3, PLSCR3, PLXNB2, PON2, POR, PPP1R7, PRAF2, PRKCA, PRMT3, PSMD2, PTBP2, PTGIS, PTK7, PTPN1, PTPN2, PTPRD, PXMP2, RABGAP1L, RDH10, RNF170, RPN1, RRBP1, RRP12, RTTN, SBF1, SCAMP2, SCARB2, SCFD1, SEC22B, SEC61G, SEL1L, SERPINB6, SESTD1, SHISA8, SLC12A4, SLC1A3, SLC25A12, SLC25A24, SLC25A3, SLC25A32, SLC25A5, SLC2A1, SLC2A10, SLC33A1, SLC44A1, SLC44A2, SLC4A7, SLC5A3, SLC7A3, SLC7A6, SMPDL3B,	

					SPAG9, SPTLC2, SRPRB, SSR1, SSR4, STIM1, STT3A, STT3B, STX12, STX5, STXBP2, SUN2, SURF4, TBL2, TENM3, TEX2, TGM2, TIMM50, TIMMDC1, TM9SF4, TMED10, TMED2, TMED5, TMED7, TMED9, TMEM115, TMEM167A, TMEM168, TMEM192, TMEM214, TMEM41A, TMEM43, TMEM97, TMX3, TMX4, TOMM34, TPM1, TPST1, TRAM1, UBR4, USE1, USP19, UTP18, VAMP2, VAMP3, VAT1, VLDLR, VPS26B, VPS35L, VRK1, YIF1A, YKT6, ZDHHC17]	
antigen processing and presentation of peptide or polysaccharide antigen via MHC class II	GO_BiologicalProcess-EBI-UniProt-GOA-ACAP-ARAP_13.05.2021_00h00	0.079766	26.6055	29	[ACTR10, ACTR1B, AP1M1, AP1S1, AP2A1, AP2A2, AP2B1, AP2M1, AP2S1, CANX, CAPZA2, DCTN1, DCTN4, DYNC1LI2, KIF11, KIF22, KIF23, KIF2C, KLC1, PYCARD, SEC13, SEC23A, SEC24A, SEC24B, SEC24C, SEC24D, SEC31A, SPTBN2, THBS1]	Downregulation
vesicle-mediated transport	GO_BiologicalProcess-EBI-UniProt-GOA-ACAP-ARAP_13.05.2021_00h00	0.079766	13.84682	320	[ABI1, ACAA1, ACBD3, ACLY, ACTN1, ACTN4, ACTR10, ACTR1B, ACTR2, ADAM10, AGL, ALDOC, ANK2, ANK3, ANKFY1, ANKRD28, ANO6, ANXA1, ANXA11, ANXA2, ANXA3, ANXA5, AP1M1, AP1S1, AP2A1, AP2A2, AP2B1, AP2M1, AP2S1, AP3B1, APAF1, APEH, APOE, APOOL, APPL2, APRT, ARAP3, ARCN1, ARF4, ARF6, ARFGAP1, ARFGAP3, ARFGEF1, ARHGAP1, ARL1, ARL3, ARPC1A, ARSA, ARSB, ATL2, ATL3, B4GALT1, BAIAP2, BET1, BIN1, CALR, CANX, CAP1, CAPN1, CAPZA2, CASK, CAT, CAV1, CBL, CCDC22, CCT2, CCT8, CD2AP, CD44, CD81, CDC42, CDH2, CHID1, CKAP4, CLASP1, COG1, COG6, COG7, COG8, COPA, COPB1, COPB2, COPE, COPG1, COPG2, COPS5, COPZ1, CORO1C, CTBP2, CTNNB1, CTSC, CYB5R1, CYB5R3, CYFIP1, DBNL, DCTN1, DCTN4, DEGS1, DIAPH1, DPYSL2, DSP, DYNC1LI2, EEA1, EEF2, EGFR, EHD1, EHD2, EHD4, ENDOD1, EPN2, ERGIC1, ERGIC2, ERP44, ESYT2, FAF2, FGG, FKBP15, FN1, FNBP1L,	Downregulation

					FTH1, GBF1, GDI1, GIPC1, GLA, GNS, GOLGA2, GOLT1B, GOPC, GOSR2, GPC3, GPI, GSN, HEATR5A, HMOX1, HMOX2, HOOK3, HSP90AB1, HSP90B1, HSPA14, HSPA2, HSPH1, HTT, HUWE1, ILF2, IMPDH1, IMPDH2, IQGAP1, IQGAP2, IST1, ITGAV, ITGB1, KDELR1, KDELR3, KIF11, KIF22, KIF23, KIF2C, KIF5B, KLC1, KRT18, LMAN1, LPCAT1, LRP1, LYAR, MACF1, MAGED2, MANBA, MAPK1, MAPK14, MESD, MFGE8, MGST1, MICAL1, MICAL3, MME, MON2, MOSPD2, MRC2, MSN, MTMR6, MVB12A, MYD88, MYH9, MYO1C, MYO1E, MYO5A, NBAS, NCKAP1, NECAP2, NEDD4, NFKB1, NHLRC2, NIT2, NLGN4X, NSF, OPTN, ORC3, OSTF1, PAK1, PDLIM7, PDXK, PFKL, PHIP, PICALM, PIK3C2A, PIK3CA, PIK3R4, PIP4K2B, PKM, PLCG1, PLIN3, PLPP3, PNP, PRDX4, PREPL, PRKAA1, PRKCSH, PSMD1, PSMD2, PTPN1, PXDN, PYCARD, RAB14, RAB18, RAB1A, RAB2A, RAB32, RAB5B, RAB5C, RAB8A, RABGAP1L, RAP2B, RAP2C, RDX, RIC1, RP2, S100A10, S100A11, SAR1A, SCAMP2, SCARB2, SCFD1, SCFD2, SCRN1, SCYL1, SDF4, SEC13, SEC16A, SEC22B, SEC23A, SEC23B, SEC24A, SEC24B, SEC24C, SEC24D, SEC31A, SEPTIN2, SEPTIN8, SERPINB6, SH3GL3, SLC44A2, SNX17, SNX3, SNX4, SNX6, SNX9, SPAG9, SPARC, SPTAN1, SPTBN1, SPTBN2, SRC, SRP14, STAM2, STX12, STX5, STXBP2, SURF4, TAGLN2, TBC1D17, TBC1D4, TF, TFG, TGM2, THBS1, TIMP3, TLN1, TM9SF4, TMED10, TMED2, TMED5, TMED7, TMED9, TMEM115, TMEM167A, TMX3, TOLLIP, TUBA4A, TXNDC5, UBE2O, UBR4, USE1, USP7, VAMP2, VAMP3, VAT1, VLDLR, VPS25, VPS26A,	

					VPS26B, VPS26C, VPS35, VPS35L, VPS36, VPS53, XRCC5, XRCC6, YIF1A, YKT6, ZFYVE16]	
antigen processing and presentation of exogenous antigen	GO_BiologicalProcess-EBI-UniProt-GOA-ACAP-ARAP_13.05.2021_00h00	0.079766	20.51282	40	[ACTR10, ACTR1B, AP1M1, AP1S1, AP2A1, AP2A2, AP2B1, AP2M1, AP2S1, AP3B1, CALR, CANX, CAPZA2, DCTN1, DCTN4, DYNC1LI2, HLA-A, ITGAV, KIF11, KIF22, KIF23, KIF2C, KLC1, LNPEP, PDIA3, PSMC6, PSMD1, PSMD2, PSMD9, PSME3, SEC13, SEC22B, SEC23A, SEC24A, SEC24B, SEC24C, SEC24D, SEC31A, SPTBN2, VAMP3]	Downregulation
organelle subcompartment	GO_CellularComponent-EBI-UniProt-GOA-ACAP-ARAP_13.05.2021_00h00	0.079766	12.01949	222	[ABHD12, ACBD3, ACSL1, ACSL4, ADAM10, ADPGK, ALG11, ALG2, ALG5, ALG9, AP1M1, AP1S1, ARAP1, ARCN1, ARFGAP1, ARFGAP3, ARFGEF1, ARL1, ARL3, ARL6IP5, ASPH, ATL2, ATL3, AUP1, B3GLCT, B4GALT1, BET1, BIRC6, BPNT2, CALR, CALU, CAMK2D, CANX, CAV1, CDC42, CHEK2, CHID1, CHPF, CHST14, CKAP4, COG1, COG6, COG7, COG8, COPA, COPB1, COPB2, COPE, COPG1, COPG2, COPZ1, CYB5R1, CYB5R3, CYP2S1, CYP51A1, DBNL, DDRGK1, DEGS1, DHRS7B, DLG1, ECPAS, EGFR, EMD, EPM2AIP1, ERAP1, ERGIC1, ERGIC2, ERLIN2, ERO1A, ERP44, ESYT2, FAF2, FKBP8, FNDC3A, FUT11, GALNT1, GALNT10, GALNT2, GBF1, GOLGA2, GOLGA3, GOLIM4, GOLT1B, GOPC, GOSR2, GPSM1, HLA-A, HMOX1, HMOX2, HSP90B1, HSPA5, HSPD1, HUWE1, IKBIP, ILVBL, KDELR1, KDELR3, KTN1, LBR, LMAN1, LMF2, LNPK, LPCAT1, LPGAT1, LRRC59, LSS, LYPLA2, MAN1A1, MAN1A2, MARCHF5, MEST, MGAT2, MGST1, MME, MOGS, MOSPD2, MOXD1, MTDH, NBAS, NCAM1, NSDHL, NSF, NSFL1C, NUP210, OPTN, OSBP, OSTC, P4HTM, PCYT1A, PCYT2, PDIA3, PDIA6, PHIP, PIGS, PIGT, PIK3C2A, PIP4K2B, PLD3, PLOD1, PLOD2, PLOD3, PLPP3, PNPT1,	Downregulation

					POFUT2, POR, PREPL, PRKCSH, PSMD2, PTGIS, PTPN1, PYCARD, RAB14, RAB18, RAB1A, RAB2A, RAB32, RAB8A, RDH10, RETSAT, RHEB, RIC1, RNF170, RPN1, RRBP1, SBF1, SCAMP2, SCARB2, SCFD1, SDF2L1, SEC13, SEC16A, SEC22B, SEC23A, SEC23B, SEC24A, SEC24B, SEC24C, SEC24D, SEC31A, SEC61G, SEL1L, SH3GLB1, SLC2A1, SLC33A1, SNX9, SPTLC2, SRPRA, SRPRB, SSR1, SSR4, STIM1, STT3A, STT3B, STX12, STX5, SURF4, TBL2, TEX2, TKT, TMED10, TMED2, TMED5, TMED7, TMED9, TMEM115, TMEM167A, TMEM214, TMEM97, TMF1, TMX3, TPST1, TRAM1, UFL1, USE1, USP19, VAMP2, VAMP3, VPS53, VRK1, YIF1A, YKT6, ZDHHC17]	
nuclear outer membrane-endoplasmic reticulum membrane network	GO_CellularComponent-EBI-UniProt-GOA-ACAP-ARAP_13.05.2021_00h00	0.079766	13.1579	160	[ABHD12, ACSL1, ACSL4, ADPGK, ALG11, ALG2, ALG5, ALG9, ARCN1, ARL6IP5, ASPH, ATL2, ATL3, AUP1, B3GLCT, BET1, CALR, CALU, CAMK2D, CANX, CAV1, CDC42, CHEK2, CKAP4, COPA, COPB1, COPB2, COPE, COPG1, COPG2, COPZ1, CYB5R1, CYB5R3, CYP2S1, CYP51A1, DDRGK1, DEGS1, DHRS7B, DLG1, EGFR, EMD, EPM2AIP1, ERAP1, ERGIC1, ERGIC2, ERLIN2, ERO1A, ERP44, ESYT2, FAF2, FKBP8, GALNT1, GALNT2, GOSR2, GPSM1, HLA-A, HMOX1, HMOX2, HSP90B1, HSPA5, IKBIP, ILVBL, KDELR1, KDELR3, KTN1, LBR, LMAN1, LMF2, LNPK, LPCAT1, LPGAT1, LRPPRC, LRRC59, LSS, MARCHF5, MEST, MGST1, MOGS, MOSPD2, MOXD1, MTDH, NBAS, NSDHL, NUP210, OSBP, OSTC, P4HTM, PCYT1A, PCYT2, PDIA3, PDIA6, PIGS, PIGT, PIP4K2B, PLD3, PLOD1, PLOD2, PLOD3, PLPP3, PNPT1, POFUT2, POR, PRKCSH, PSMD2, PTGIS, PTPN1, RAB14, RAB18, RAB2A, RANBP2, RDH10, RETSAT, RHEB, RNF170, RPN1, RRBP1, SBF1, SCARB2, SCFD1, SDF2L1, SEC13, SEC16A,	Downregulation

					SEC22B, SEC23A, SEC23B, SEC24A, SEC24B, SEC24C, SEC24D, SEC31A, SEC61G, SEL1L, SLC33A1, SPTLC2, SRPRA, SRPRB, SSR1, SSR4, STIM1, STT3A, STT3B, STX5, SURF4, TBL2, TEX2, TKT, TMED10, TMED2, TMED5, TMED7, TMED9, TMEM214, TMEM97, TMX3, TNPO3, TRAM1, UFL1, USE1, USP19, YIF1A]	
antigen processing and presentation of peptide antigen	GO_BiologicalProcess-EBI-UniProt-GOA-ACAP-ARAP_13.05.2021_00h00	0.079766	20.19704	41	[ACTR10, ACTR1B, AP1M1, AP1S1, AP2A1, AP2A2, AP2B1, AP2M1, AP2S1, CALR, CANX, CAPZA2, DCTN1, DCTN4, DYNC1LI2, ERAP1, HLA-A, ITGAV, KIF11, KIF22, KIF23, KIF2C, KLC1, LNPEP, PDIA3, PSMC6, PSMD1, PSMD2, PSMD9, PSME3, PYCARD, SEC13, SEC22B, SEC23A, SEC24A, SEC24B, SEC24C, SEC24D, SEC31A, SPTBN2, VAMP3]	Downregulation
organelle localization	GO_BiologicalProcess-EBI-UniProt-GOA-ACAP-ARAP_13.05.2021_00h00	0.079766	14.78261	102	[ACTN4, ACTR10, ACTR2, AGTPBP1, ANKRD28, AP3B1, ARCN1, ARFGAP3, AURKB, BET1, BIN1, BRAT1, CBL, CCNB1, CDC42, CDCA8, CDH2, CDH3, CKAP5, CLASP1, CLUH, COPG1, COPG2, CTBP2, CTNNB1, CTSC, DCTN1, DLG1, DLGAP5, DOCK7, DPYSL2, ESYT2, FNBP1L, GBF1, GOLGA2, GOSR2, HMOX1, HOOK3, HTT, INCENP, ITGB1, KIF22, KIF2C, KIF5B, LMAN1, LRPPRC, LSG1, MAP1B, MAP1S, MLH1, MYH9, MYO1C, MYO1E, MYO5A, NDC80, NECTIN2, NOP9, NPM1, NSF, NSFL1C, NUSAP1, ORC3, PARD3, PHIP, PICALM, PLK1, PRKAR2B, PRKCA, RAB1A, RAB8A, RAN, RRS1, SAR1A, SCFD1, SCFD2, SDAD1, SEC13, SEC16A, SEC22B, SEC23A, SEC24A, SEC24B, SEC24C, SEC24D, SEC31A, SEH1L, SNX4, SNX6, SPAG9, SPOUT1, STX12, STX5, STXBP2, SUN2, TFG, TMED10, TMED2, TMED9, TUBA4A, VAMP2, VAMP3, YKT6]	No change
integral component of membrane	GO_CellularComponent-EBI-	0.079766	4.961637	291	[ABCB10, ABCB7, ABCC1, ABHD12, ACSL1, ACSL4, ADAM10,	Downregulation

	UniProt-GOA-ACAP-ARAP_13.05.2021_00h00				ADPGK, ALCAM, ALG11, ALG2, ALG5, ALG9, ANO6, ANTXR1, APMAP, APOOL, ARF4, ARHGAP1, ARL6IP5, ARSA, ASPH, ATL2, ATL3, ATP1A1, ATP2B1, ATP2B2, ATP2B4, AUP1, B3GLCT, B4GALT1, BCAT2, BET1, BPNT2, CALR, CANX, CAPZA2, CASK, CAV1, CD44, CD81, CDH2, CDH3, CEP170, CHEK2, CHPF, CHST14, CKAP4, CNTFR, CPD, CPOX, CRLF3, CSPG4, CXADR, CYB5R1, CYP2S1, CYP51A1, DAGLB, DAXX, DEGS1, DHRS7B, DHX15, DHX8, ECE1, ECHDC1, EEF1E1, EFNB2, EGFR, EHMT1, EMD, EPB41, EPB41L3, EPCAM, EPHB3, EPS8, ERAP1, ERGIC1, ERGIC2, ERLIN2, ESYT2, ETFA, EXD2, EXOG, F11R, FAT1, FDPS, FKBP11, FKBP8, FLNB, FLT1, FNDC3A, FNDC3B, FOCAD, FOXRED1, FTH1, FUT11, GALNT1, GALNT10, GALNT2, GARS1, GDAP1, GLMN, GLT8D1, GNL2, GOLIM4, GOLT1B, GOSR2, GPX8, GSDME, GTF3C3, GXYLT1, HDAC2, HLA-A, HMOX2, HSPA2, HSPA5, HSPA9, HSPD1, IKBIP, ILVBL, ITGA5, ITGA6, ITGAV, ITGB1, JAM3, KDELR1, KDELR3, KIRREL1, KTN1, L2HGDH, LBR, LMAN1, LMF2, LNPEP, LNPK, LPCAT1, LPGAT1, LRBA, LRP1, LRRC59, LRRC8A, MACF1, MAN1A1, MAN1A2, MARCHF5, MAVS, MCAM, MCU, MEMO1, MEST, MFSD10, MGAT2, MGST1, MICOS13, MIPEP, MME, MOGS, MOSPD2, MOXD1, MPC2, MRC2, MTDH, MYADM, NBAS, NCAM1, NCKAP1, NDC1, NDRG2, NECTIN2, NLGN4X, NNT, NPM3, NRP2, NSDHL, NUP210, NUP50, OCLN, ORC3, OSTC, P4HTM, PDGFRB, PDIA3, PGAM5, PGRMC2, PIGS, PIGT, PKP2, PLD3, PLEKHA7, PLPP3, PLSCR3, PLXNB2, PON2, POR, PPP1R7, PRAF2, PRKCA, PRMT3, PSMD2, PTBP2, PTGIS, PTK7, PTPN1, PTPN2, PTPRD, PXMP2, RABGAP1L, RDH10,	

					RNF170, RPN1, RRBP1, RRP12, RTTN, SBF1, SCAMP2, SCARB2, SCFD1, SEC22B, SEC61G, SEL1L, SERPINB6, SESTD1, SHISA8, SLC12A4, SLC1A3, SLC25A12, SLC25A24, SLC25A3, SLC25A32, SLC25A5, SLC2A1, SLC2A10, SLC33A1, SLC44A1, SLC44A2, SLC4A7, SLC5A3, SLC7A3, SLC7A6, SPAG9, SPTLC2, SRPRB, SSR1, SSR4, STIM1, STT3A, STT3B, STX12, STX5, STXBP2, SUN2, SURF4, TBL2, TENM3, TEX2, TIMM50, TIMMDC1, TM9SF4, TMED10, TMED2, TMED5, TMED7, TMED9, TMEM115, TMEM167A, TMEM168, TMEM192, TMEM214, TMEM41A, TMEM43, TMEM97, TMX3, TMX4, TOMM34, TPM1, TPST1, TRAM1, UBR4, USE1, USP19, UTP18, VAMP2, VAMP3, VAT1, VLDLR, VPS26B, VPS35L, VRK1, YIF1A, YKT6, ZDHHC17]	
membrane coat	GO_CellularComponent-EBI-UniProt-GOA-ACAP-ARAP_13.05.2021_00h00	0.079766	31.63265	31	[AP1M1, AP1S1, AP2A1, AP2A2, AP2B1, AP2M1, AP2S1, AP3B1, ARCN1, COPA, COPB1, COPB2, COPE, COPG1, COPG2, COPZ1, DIPK2A, EPN2, NECAP2, PICALM, SAR1A, SCYL1, SEC13, SEC23A, SEC23B, SEC24A, SEC24B, SEC24C, SEC24D, SEC31A, TMED7]	Downregulation
organelle membrane	GO_CellularComponent-EBI-UniProt-GOA-ACAP-ARAP_13.05.2021_00h00	0.079766	10.5083	399	[AAAS, ABCB10, ABCB7, ABHD12, ACAD9, ACBD3, ACSL1, ACSL4, ADAM10, ADPGK, ALG11, ALG2, ALG5, ALG9, ANKFY1, ANO6, ANTXR1, ANXA1, ANXA2, ANXA3, ANXA4, ANXA6, AP1M1, AP1S1, AP2A1, AP2A2, AP2B1, AP2M1, AP2S1, AP3B1, APEH, APOE, APOOL, APPL2, ARAP1, ARCN1, ARF6, ARFGAP1, ARFGAP3, ARFGEF1, ARHGAP1, ARL1, ARL3, ARL6IP5, ASPH, ATL2, ATL3, ATP1A1, ATP2B1, ATP6V1A, AUP1, B3GLCT, B4GALT1, BET1, BPNT2, CALCOCO2, CALR, CALU, CAMK2D, CANX, CASK, CAT, CAV1, CCND2, CD44, CDC42, CHEK2, CHPF, CHST14, CKAP4, COG1, COG6, COG7, COG8, COL6A1,	Downregulation

					COPA, COPB1, COPB2, COPE, COPG1, COPG2, COPZ1, CORO1C, CTSC, CYB5R1, CYB5R3, CYP2S1, CYP51A1, DAGLB, DBNL, DDRGK1, DEGS1, DHRS7B, DHX37, DIAPH1, DIPK2A, DLG1, DSP, ECE1, EEA1, EGFR, EHD1, EHD2, EHD4, EMD, EPM2AIP1, EPN2, ERAP1, ERBIN, ERGIC1, ERGIC2, ERLIN2, ERO1A, ERP44, ESYT2, EXD2, EXOG, FAF2, FDXR, FECH, FKBP8, FNDC3A, FOXRED1, FUT11, GALNT1, GALNT10, GALNT2, GARS1, GATA6, GBF1, GDAP1, GIPC1, GNB2, GOLGA2, GOLGA3, GOLIM4, GOLT1B, GOPC, GOSR2, GOT2, GPI, GPSM1, GTF3C3, GUF1, HADHA, HADHB, HK1, HK2, HLA-A, HMOX1, HMOX2, HSD17B4, HSP90AB1, HSP90B1, HSPA5, HSPA9, HSPD1, HTT, HUWE1, IKBIP, ILVBL, IMPDH2, IQGAP1, IQGAP2, ITCH, ITGAV, ITGB1, KDELR1, KDELR3, KTN1, L2HGDH, LBR, LDHB, LMAN1, LMF2, LNPEP, LNPK, LPCAT1, LPGAT1, LRP1, LRPPRC, LRRC59, LRRC8A, LSS, MAIP1, MAN1A1, MAN1A2, MANBA, MAP1LC3A, MARCHF5, MATR3, MAVS, MCU, MEAK7, MEST, MFGE8, MFSD10, MGAT2, MGST1, MICOS13, MME, MOGS, MON2, MOSPD2, MOXD1, MPC2, MRPS27, MRPS9, MTDH, MVB12A, MYD88, MYO1C, MYO1E, NBAS, NCAM1, NDC1, NDRG1, NECAP2, NNT, NSDHL, NSF, NUP107, NUP133, NUP210, NUP35, NUP50, OCLN, OGT, OPTN, OSBP, OSTC, P4HTM, PAK1, PCYT1A, PCYT2, PDIA3, PDIA6, PDLIM4, PGAM5, PHIP, PI4KA, PICALM, PIGS, PIGT, PIK3R4, PIP4K2B, PLD3, PLIN3, PLOD1, PLOD2, PLOD3, PLPP3, PLSCR3, PNPT1, POFUT2, POR, PRAF2, PRKCA, PRKCSH, PSMD2, PTCD3, PTGIS, PTPMT1, PTPN1, PXMP2, PYCARD, RAB14, RAB18, RAB1A, RAB23, RAB2A, RAB32, RAB5B, RAB5C, RAB8A,	

					RANBP2, RANGAP1, RAP2A, RAP2B, RAP2C, RBM15, RCC2, RDH10, RETSAT, RHEB, RIC1, RIF1, RNF170, RPN1, RRBP1, RRP12, SAR1A, SARM1, SBF1, SCAMP2, SCARB2, SCFD1, SCRN1, SCYL1, SDF2L1, SEC13, SEC16A, SEC22B, SEC23A, SEC23B, SEC24A, SEC24B, SEC24C, SEC24D, SEC31A, SEC61G, SEH1L, SEL1L, SEPHS1, SEPTIN2, SEPTIN8, SERPINB6, SH3GL3, SH3GLB1, SIRT1, SLC12A4, SLC25A12, SLC25A24, SLC25A3, SLC25A32, SLC25A5, SLC2A1, SLC33A1, SLC44A1, SLC44A2, SNTB2, SNX17, SNX3, SNX4, SNX6, SNX9, SORD, SPAG9, SPARC, SPTLC2, SRC, SRPRA, SRPRB, SSR1, SSR4, STAM2, STIM1, STT3A, STT3B, STX12, STX5, SUN2, SURF4, TBL2, TEX2, TF, TIGAR, TIMM50, TIMMDC1, TKT, TMED10, TMED2, TMED5, TMED7, TMED9, TMEM115, TMEM167A, TMEM192, TMEM214, TMEM43, TMEM97, TMF1, TMX3, TMX4, TOMM34, TPST1, TRAM1, TRAP1, TTC1, UBR4, UFL1, USE1, USP19, UTP18, VAMP2, VAMP3, VAT1, VLDLR, VPS25, VPS26A, VPS35, VPS35L, VPS36, VPS53, WDR3, YIF1A, YKT6, ZDHHC17, ZFYVE16]	
establishment of organelle localization	GO_BiologicalProcess-EBI-UniProt-GOA-ACAP-ARAP_13.05.2021_00h00	0.079766	18.33689	86	[ACTN4, ACTR10, ACTR2, AGTPBP1, ANKRD28, AP3B1, ARFGAP3, AURKB, BET1, CBL, CCNB1, CDC42, CDCA8, CDH3, CLASP1, COPG1, COPG2, CTNNB1, CTSC, DCTN1, DLG1, DLGAP5, DOCK7, DPYSL2, FNBP1L, GBF1, GOLGA2, GOSR2, HMOX1, HOOK3, HTT, INCENP, ITGB1, KIF22, KIF2C, KIF5B, LMAN1, LRPPRC, LSG1, MAP1B, MAP1S, MLH1, MYH9, MYO1C, MYO1E, MYO5A, NDC80, NECTIN2, NOP9, NPM1, NSF, NSFL1C, NUSAP1, ORC3, PARD3, PHIP, PICALM, PLK1, RAB1A, RAN, RRS1, SAR1A, SCFD1, SDAD1, SEC13, SEC16A, SEC22B,	No change

					SEC23A, SEC24A, SEC24B, SEC24C, SEC24D, SEC31A, SEH1L, SNX4, SNX6, STX5, STXBP2, SUN2, TFG, TMED10, TMED2, TMED9, VAMP2, VAMP3, YKT6]	
antigen processing and presentation of peptide antigen via MHC class I	GO_BiologicalProcess-EBI-UniProt-GOA-ACAP-ARAP_13.05.2021_00h00	0.079766	20.38835	21	[CALR, CANX, ERAP1, HLA-A, ITGAV, LNPEP, PDIA3, PSMC6, PSMD1, PSMD2, PSMD9, PSME3, SEC13, SEC22B, SEC23A, SEC24A, SEC24B, SEC24C, SEC24D, SEC31A, VAMP3]	Downregulation
endoplasmic reticulum	GO_CellularComponent-EBI-UniProt-GOA-ACAP-ARAP_13.05.2021_00h00	0.079766	13.56256	284	[ABHD12, ABI1, ACSL1, ACSL4, ADAM10, ADPGK, AGL, ALG11, ALG2, ALG5, ALG9, ANK3, APOE, ARCN1, ARHGAP5, ARL6IP5, ARSA, ARSB, ASPH, ATL2, ATL3, ATP1A1, AUP1, B3GLCT, BAIAP2, BET1, CALR, CALU, CAMK2D, CANX, CAPN2, CAST, CAT, CAV1, CAVIN1, CDC42, CDH2, CERCAM, CHEK2, CKAP4, COL1A1, COL1A2, COL4A1, COL4A2, COL5A1, COL5A2, COL6A1, COL6A2, COL6A3, COLGALT1, COPA, COPB1, COPB2, COPE, COPG1, COPG2, COPZ1, CRTAP, CTSC, CYB5R1, CYB5R3, CYP2S1, CYP51A1, DDRGK1, DEGS1, DHRS7B, DLG1, ECPAS, EDEM3, EGFR, EHD4, EMD, EPM2AIP1, ERAP1, ERGIC1, ERGIC2, ERLEC1, ERLIN2, ERO1A, ERP44, ESD, ESYT2, FAF2, FBXO2, FGG, FKBP10, FKBP14, FKBP7, FKBP8, FKBP9, FN1, GALNT1, GALNT2, GBF1, GET3, GLUD1, GLUL, GOLT1B, GOSR2, GPC3, GPSM1, GPX7, GPX8, GSTM2, H6PD, HADHB, HK2, HLA-A, HMOX1, HMOX2, HSP90B1, HSPA13, HSPA5, HSPD1, HTT, IKBIP, ILVBL, KDELR1, KDELR3, KEAP1, KTN1, LAMB1, LAMC1, LARS1, LBR, LGALS1, LIN28A, LMAN1, LMF2, LNPK, LPCAT1, LPGAT1, LRBA, LRRC59, LSG1, LSS, LTBP1, MAN1A1, MAN1A2, MAPK1, MARCHF5, MESD, MEST, MFGE8, MGST1, MIPEP, MOGS, MOSPD2, MOXD1, MTDH, MTMR6, MYDGF, MYO5A, NBAS,	Downregulation

					NSDHL, NUCB2, NUP210, OSBP, OSTC, P3H1, P3H3, P3H4, P4HA1, P4HA2, P4HB, P4HTM, PCYT1A, PCYT2, PDCL3, PDIA3, PDIA4, PDIA6, PDXDC1, PGRMC2, PIGS, PIGT, PIP4K2B, PIP4K2C, PKM, PLD3, PLOD1, PLOD2, PLOD3, PLPP3, PNPT1, POFUT1, POFUT2, POGLUT2, POGLUT3, POR, PPIB, PRDX4, PRKCA, PRKCSH, PSMD2, PTGIS, PTPN1, PTPN2, PXDN, PYCARD, RAB14, RAB18, RAB1A, RAB2A, RAB32, RAP1GDS1, RCN1, RDH10, RETSAT, RHEB, RNF170, RPL27A, RPL4, RPL5, RPN1, RPS21, RPS8, RRBP1, S100A10, SAR1A, SBF1, SCARB2, SCFD1, SDF2L1, SDF4, SEC13, SEC16A, SEC22B, SEC23A, SEC23B, SEC24A, SEC24B, SEC24C, SEC24D, SEC31A, SEC61G, SEL1L, SERPINH1, SET, SIL1, SLC33A1, SORD, SPON1, SPTLC2, SRPK1, SRPRA, SRPRB, SSR1, SSR4, STIM1, STT3A, STT3B, STX5, SUMF2, SURF4, TBL2, TEX2, TF, TFG, TGM2, THBS1, TKT, TMED10, TMED2, TMED5, TMED7, TMED9, TMEM192, TMEM214, TMEM43, TMEM97, TMF1, TMX3, TRAM1, TXNDC5, UFL1, UGGT1, UGGT2, USE1, USP19, UTP15, YIF1A, YKT6, YTHDC2]	
endoplasmic reticulum-Golgi intermediate compartment	GO_CellularComponent-EBI-UniProt-GOA-ACAP-ARAP_13.05.2021_00h00	0.079766	30.28169	43	[BET1, CALR, COPB1, COPG1, COPG2, CTSC, DICER1, ECPAS, ERGIC1, ERGIC2, ERP44, FN1, GALNT1, GBF1, GOLGA2, GOSR2, HSPA5, IST1, KDELR1, LMAN1, MAN1A1, MTMR6, MYDGF, NUCB2, P4HB, PDIA6, PLPP3, PTPN2, RAB2A, SCYL1, SEC22B, SERPINH1, STX5, SURF4, TMED10, TMED2, TMED5, TMED7, TMED9, UGGT1, UGGT2, YIF1A, YKT6]	Downregulation
Golgi apparatus	GO_CellularComponent-EBI-UniProt-GOA-ACAP-ARAP_13.05.2021_00h00	0.079766	11.44902	192	[AATF, ACBD3, ADAM10, AKR7A2, ANK3, ANTXR1, AP1M1, AP1S1, AP2A1, AP2A2, AP3B1, APOE, ARAP1, ARCN1, ARF4, ARF6, ARFGAP1, ARFGAP3, ARFGEF1, ARL1, ARL2, ARL3, ARSB, ATP1A1,	Downregulation

					ATR, B4GALT1, BAIAP2, BET1, BIRC6, BPNT2, CALR, CALU, CAPN2, CAT, CAV1, CBL, CD44, CDC42, CHEK2, CHID1, CHPF, CHST14, CLASP1, COG1, COG6, COG7, COG8, COL1A1, COPA, COPB1, COPB2, COPE, COPG1, COPG2, COPZ1, CSDE1, CSPG4, CTSC, CUL7, DBNL, DDX31, DDX54, DIPK2A, DLG1, ECPAS, EGFR, ERGIC1, ERGIC2, FAM114A1, FBXW8, FNDC3A, FUT11, GALNT1, GALNT10, GALNT2, GBF1, GDI1, GLA, GLT8D1, GOLGA2, GOLGA3, GOLIM4, GOLT1B, GOPC, GOSR2, GPC1, GPC3, GPC6, GPSM1, HLA-A, HOOK3, HSPD1, HTT, HUWE1, JAM3, KDELR1, KDELR3, KIF20A, LMAN1, LPCAT1, LRBA, LRP1, LYPLA2, MACF1, MAN1A1, MAN1A2, MAPK1, MGAT2, MMAB, MME, MSH6, MVB12A, MYDGF, NCAM1, NDRG2, NEDD4, NMT2, NSF, NSFL1C, NUCB2, OPTN, ORC3, OSBP, PACS1, PDGFRB, PDXDC1, PHIP, PICALM, PIK3C2A, PLD3, PLIN3, PLOD3, PLPP3, POFUT2, PREPL, PRKCSH, PRRC1, PYCARD, RAB14, RAB18, RAB1A, RAB2A, RAB32, RAB8A, RABGAP1L, RHEB, RIC1, RP2, SAR1A, SCAMP2, SCARB2, SCFD1, SCLY, SCYL1, SDF4, SEC16A, SEC22B, SEC23A, SERPINB9, SH3GLB1, SLC2A1, SLC33A1, SNTB2, SNX17, SNX9, STX12, STX5, SURF4, TCP1, TM9SF4, TMED10, TMED2, TMED5, TMED7, TMED9, TMEM115, TMEM167A, TMEM192, TMEM214, TMEM43, TMF1, TPST1, USE1, VAMP2, VAMP3, VPS53, VRK1, WAPL, YIF1A, YKT6, ZDHHC17, ZMYND8]	
vesicle targeting	GO_BiologicalProcess-EBI-UniProt-GOA-ACAP-ARAP_13.05.2021_00h00	0.079766	28.43137	29	[ANKRD28, ARFGAP3, BET1, CLASP1, CTSC, GBF1, GOLGA2, GOSR2, LMAN1, NSF, PHIP, RAB1A, SAR1A, SCFD1, SEC13, SEC16A, SEC22B, SEC23A, SEC24A, SEC24B, SEC24C, SEC24D, SEC31A, STX5,	Downregulation

					TFG, TMED10, TMED2, TMED9, YKT6]	
endomembrane system organization	GO_BiologicalProcess-EBI-UniProt-GOA-ACAP-ARAP_13.05.2021_00h00	0.079766	16.95205	99	[AKT1, ANK2, ANK3, ANO6, ANXA6, AP3B1, ARFGEF1, ARL1, ASAP1, ATL2, ATL3, BAIAP2, BET1, BIN1, CAV1, CCNB1, CDC42, CLASP1, COG1, COG7, COL5A1, CORO1C, CUL7, DCTN1, DEGS1, EEA1, EHD2, EMD, EPB41L3, ESYT2, FBXW8, FNBP1L, GBF1, GET3, GET4, GOLGA2, GOSR2, GSN, HMOX1, HOOK3, HTT, HUWE1, IST1, LBR, LMAN1, LNPK, MAPK1, MVB12A, MYH9, MYO5A, NDC1, NDRG1, NECTIN2, NSF, NSFL1C, NUP107, NUP133, NUP160, NUP35, OPTN, OSBP, PLK1, PLSCR3, PPP1R7, PRKCA, RAB18, RAB1A, RAB2A, RAN, RANGAP1, SCARB2, SEC13, SEC16A, SEC22B, SEC31A, SEC61G, SEH1L, SH3GLB1, SIRT2, SNX3, SNX9, SPTBN1, STAM2, STX5, SUN2, SURF4, TBPL1, TMED10, TMED2, TMED5, TMED7, TMED9, TMEM43, TMF1, TRAM1, VAMP2, VPS25, VPS36, VRK1]	Downregulation
vesicle cargo loading	GO_BiologicalProcess-EBI-UniProt-GOA-ACAP-ARAP_13.05.2021_00h00	0.079766	43.33333	13	[PICALM, RAB1A, SAR1A, SEC13, SEC23A, SEC23B, SEC24A, SEC24B, SEC24C, SEC24D, SEC31A, TMED10, TMED2]	Downregulation
vesicle localization	GO_BiologicalProcess-EBI-UniProt-GOA-ACAP-ARAP_13.05.2021_00h00	0.079766	17.69547	43	[ACTN4, ANKRD28, AP3B1, ARFGAP3, BET1, CDH2, CDH3, CLASP1, CTNNB1, CTSC, DPYSL2, FNBP1L, GBF1, GOLGA2, GOSR2, HTT, KIF5B, LMAN1, MYO1C, MYO1E, MYO5A, NSF, PARD3, PHIP, PICALM, RAB1A, SAR1A, SCFD1, SEC13, SEC16A, SEC22B, SEC23A, SEC24A, SEC24B, SEC24C, SEC24D, SEC31A, STX5, TFG, TMED10, TMED2, TMED9, YKT6]	Downregulation
membrane organization	GO_BiologicalProcess-EBI-UniProt-GOA-ACAP-ARAP_13.05.2021_00h00	0.079766	13.13321	140	[ABCC1, ACTR2, AKT1, ANK2, ANK3, ANKFY1, ANKRD28, ANO6, ANXA1, ANXA2, ANXA6, AP2A1, AP2A2, AP2B1, AP2M1, AP2S1, AP3B1, APOE, APOOL, APPL2, ARFGAP3, ARPC1A, ASAP1, ATL2, AURKB, BAIAP2, BET1, BIN1, CALR, CAV1, CBL, CCNB1, CDC42, CDH2,	Downregulation

					COL5A1, CORO1C, CTSC, DCTN1, DEGS1, DLG1, EEA1, EGFR, EHD2, EMD, EPB41L3, EPN2, FNBP1L, GBF1, GCLC, GET3, GET4, GOLGA2, GOSR2, GPHN, GSN, HK2, HMOX1, HSPA4, HSPA9, HUWE1, IST1, KIF20A, KIF5B, LBR, LMAN1, MAIP1, MAPK8, MESD, MFGE8, MICOS13, MYADM, MYH9, NDC1, NDRG1, NECTIN2, NLGN4X, NSF, NSFL1C, NUP107, NUP133, NUP160, NUP35, OSBP, PI4KA, PICALM, PIK3C2A, PIP4K2B, PLK1, PLSCR3, PPP1R7, PRKCA, PTPRD, RAB1A, RAB8A, RAN, RANGAP1, RFTN1, S100A10, SAR1A, SCARB2, SCFD1, SEC13, SEC16A, SEC22B, SEC23A, SEC23B, SEC24A, SEC24B, SEC24C, SEC24D, SEC31A, SEC61G, SEH1L, SEPTIN8, SH3GLB1, SIRT2, SLC25A5, SNX3, SNX9, SPTBN1, STAM2, STX12, STX5, SUN2, TBC1D4, TF, TFG, THBS1, TIMM50, TMED10, TMED2, TMED9, TMEM43, TRAM1, USE1, VAMP2, VAMP3, VRK1, YKT6, ZMYND8]	
endoplasmic reticulum exit site	GO_CellularComponent-EBI-UniProt-GOA-ACAP-ARAP_13.05.2021_00h00	0.079766	37.14286	13	[HLA-A, PLPP3, SAR1A, SEC16A, SEC23A, SEC23B, SEC24A, SEC24B, SEC24C, SEC24D, SEC31A, TFG, TMED5]	Downregulation
cytoplasmic vesicle	GO_CellularComponent-EBI-UniProt-GOA-ACAP-ARAP_13.05.2021_00h00	0.079766	12.15794	311	[ACAA1, ACLY, ACTN1, ACTN4, ACTR10, ACTR1B, ACTR2, ADAM10, ADD2, AGL, ALDOC, ANK2, ANKFY1, ANO6, ANP32E, ANTXR1, ANXA1, ANXA11, ANXA2, ANXA3, ANXA6, AP1M1, AP1S1, AP2A1, AP2A2, AP2B1, AP2M1, AP2S1, AP3B1, APAF1, APEH, APOE, APOOL, APPL2, APRT, ARAP1, ARCN1, ARF6, ARHGAP1, ARSA, ARSB, ATP1A1, ATP2B1, ATP6V1A, AUP1, B4GALT1, BAIAP2, BET1, BIN1, BIRC6, CALCOCO2, CALR, CALU, CAMK2D, CANX, CAP1, CAPN1, CAT, CAV1, CCDC22, CCT2, CCT4, CCT8, CD2AP,	Downregulation

					CD44, CDC42, CHID1, CKAP4, COL1A1, COPA, COPB1, COPB2, COPE, COPG1, COPG2, COPS5, COPZ1, CORO1C, CTSC, CXADR, CYB5R1, CYB5R3, CYFIP1, DBNL, DEGS1, DIAPH1, DIPK2A, DPYSL3, DSP, DYNC1LI2, ECE1, ECPAS, EEA1, EEF2, EGFR, EHD1, EHD2, EHD4, EPN2, ERGIC1, ERGIC2, ERP44, ESD, F11R, FAF2, FGG, FKBP15, FLNB, FLT1, FN1, FNBP1L, FNDC3A, FTH1, GARS1, GIPC1, GLA, GNS, GOLGA2, GOLIM4, GOPC, GOSR2, GPC1, GPI, GSN, HEATR5A, HEXA, HLA-A, HMOX2, HOOK3, HSP90AB1, HSP90B1, HSPA5, HSPD1, HSPH1, HTT, HUWE1, ILF2, IMPDH1, IMPDH2, IQGAP1, IQGAP2, IST1, ITCH, ITGAV, ITGB1, KDELR1, KDELR3, KIF5B, KLC1, LMAN1, LNPEP, LPCAT1, LRP1, LSM4, MAGED2, MAN1A1, MANBA, MAP1LC3A, MAPK1, MAPK14, MFGE8, MGST1, MME, MON2, MOSPD2, MOXD1, MVB12A, MYD88, MYH9, MYO1C, MYO1E, MYO5A, NCAPG, NDRG1, NECAP2, NFKB1, NHLRC2, NIT2, NPM3, OCLN, OPTN, OSTF1, P4HB, PACS1, PDGFRB, PDIA3, PDIA4, PDIA6, PDLIM4, PDXK, PFKL, PHIP, PI4KA, PICALM, PIGT, PIK3C2A, PIK3R4, PKM, PLD3, PLIN3, PNP, PPIB, PRAF2, PRDX1, PRDX4, PRKCA, PRKCSH, PSMD1, PSMD2, PTPN1, PXDN, PYCARD, RAB14, RAB18, RAB1A, RAB23, RAB2A, RAB32, RAB5B, RAB5C, RAB8A, RABGAP1L, RAC3, RAN, RAP2A, RAP2B, RAP2C, RCC2, RFTN1, RPN1, S100A11, SAR1A, SCAMP2, SCARB2, SCFD1, SCFD2, SCYL1, SDF4, SEC13, SEC16A, SEC22B, SEC23A, SEC23B, SEC24A, SEC24B, SEC24C, SEC24D, SEC31A, SEPTIN2, SEPTIN8, SERPINB6, SH3GL3, SH3GLB1, SLC1A3, SLC2A1, SLC44A2, SLC4A7, SLIRP, SNTB2, SNX17, SNX3, SNX4, SNX6, SNX9, SPAG9,	

					SPARC, SPTAN1, SRC, SRP14, STAM2, STX12, STX5, STXBP2, SUN2, SURF4, TBC1D17, TCP1, TF, THBS1, TIMP3, TM9SF4, TMED10, TMED2, TMED5, TMED7, TMED9, TMEM168, TMEM192, TMX3, TOLLIP, TXNDC5, UBR4, USE1, VAMP2, VAMP3, VAT1, VIM, VPS25, VPS26A, VPS26B, VPS26C, VPS35, VPS35L, VPS36, VPS53, XRCC5, XRCC6, YIF1A, YKT6, ZDHHC17, ZFYVE16]	
vesicle membrane	GO_CellularComponent-EBI-UniProt-GOA-ACAP-ARAP_13.05.2021_00h00	0.079766	11.93317	150	[ADAM10, ANKFY1, ANO6, ANTXR1, ANXA1, ANXA2, ANXA3, ANXA4, ANXA6, AP1M1, AP1S1, AP2A1, AP2A2, AP2B1, AP2M1, AP2S1, AP3B1, APOE, APPL2, ARCN1, ARF6, ARHGAP1, ATP2B1, B4GALT1, CALR, CAMK2D, CAV1, CD44, CKAP4, COPA, COPB1, COPB2, COPE, COPG1, COPG2, COPZ1, CORO1C, CYB5R1, DBNL, DEGS1, DIAPH1, DIPK2A, DSP, ECE1, EEA1, EGFR, EHD1, EHD2, EHD4, EPN2, FNDC3A, GIPC1, GOLIM4, GOPC, GOSR2, HLA-A, HMOX2, HTT, IQGAP1, IQGAP2, ITCH, ITGAV, ITGB1, KDELR1, KDELR3, LMAN1, LPCAT1, LRP1, MANBA, MFGE8, MGST1, MME, MON2, MOSPD2, MOXD1, MVB12A, MYD88, MYO1C, MYO1E, NDRG1, NECAP2, OPTN, PDIA3, PDLIM4, PI4KA, PICALM, PIK3R4, PLD3, PLIN3, PRAF2, PRKCSH, RAB14, RAB18, RAB1A, RAB23, RAB32, RAB5B, RAB5C, RAB8A, RAP2A, RAP2B, RAP2C, RCC2, SAR1A, SCAMP2, SCARB2, SCYL1, SEC13, SEC16A, SEC22B, SEC23A, SEC23B, SEC24A, SEC24B, SEC24C, SEC24D, SEC31A, SEPTIN8, SERPINB6, SH3GL3, SLC44A2, SNTB2, SNX17, SNX3, SNX4, SNX6, SNX9, SPARC, STAM2, STX12, STX5, SUN2, SURF4, TF, TMED10, TMED2, TMED7, TMX3, UBR4, VAMP2, VAMP3, VPS25, VPS26A, VPS35, VPS35L, VPS36, VPS53,	Downregulation

					YKT6, ZDHHC17, ZFYVE16]	
bounding membrane of organelle	GO_CellularComponent-EBI-UniProt-GOA-ACAP-ARAP_13.05.2021_00h00	0.079766	11.04754	251	[ACBD3, ACSL1, ACSL4, ADAM10, ANKFY1, ANO6, ANTXR1, ANXA1, ANXA2, ANXA3, ANXA6, AP1M1, AP1S1, AP2A1, AP2A2, AP2B1, AP2M1, AP2S1, AP3B1, APOE, APOOL, APPL2, ARAP1, ARCN1, ARF6, ARFGAP1, ARFGAP3, ARFGEF1, ARHGAP1, ARL1, ARL3, ASPH, ATP2B1, ATP6V1A, B4GALT1, BET1, BPNT2, CALCOCO2, CALR, CAMK2D, CASK, CAT, CAV1, CD44, CDC42, CHPF, CHST14, CKAP4, COG1, COG6, COG7, COG8, COL6A1, COPA, COPB1, COPB2, COPE, COPG1, COPG2, COPZ1, CORO1C, CTSC, CYB5R1, CYB5R3, DAGLB, DBNL, DEGS1, DHRS7B, DIAPH1, DIPK2A, DSP, ECE1, EEA1, EGFR, EHD1, EHD2, EHD4, EMD, EPN2, ERGIC1, ERGIC2, EXD2, FNDC3A, FUT11, GALNT1, GALNT10, GALNT2, GBF1, GDAP1, GNB2, GOLGA2, GOLGA3, GOLIM4, GOLT1B, GOPC, GOSR2, GPI, GPSM1, HADHB, HK1, HK2, HLA-A, HMOX2, HSD17B4, HSP90AB1, HSPA9, HUWE1, IMPDH2, IQGAP1, IQGAP2, ITCH, ITGAV, ITGB1, KDELR1, KDELR3, LMAN1, LNPEP, LPCAT1, LRP1, LRPPRC, LRRC8A, MAN1A1, MAN1A2, MANBA, MAP1LC3A, MARCHF5, MAVS, MEAK7, MFGE8, MGAT2, MGST1, MICOS13, MME, MON2, MOSPD2, MOXD1, MVB12A, MYD88, NCAM1, NDRG1, NECAP2, NSF, OCLN, OPTN, OSBP, PDIA3, PDLIM4, PGAM5, PHIP, PI4KA, PICALM, PIK3R4, PLD3, PLIN3, PLOD1, PLOD2, PLPP3, PRAF2, PRKCSH, PXMP2, PYCARD, RAB14, RAB18, RAB1A, RAB23, RAB2A, RAB32, RAB5B, RAB5C, RAB8A, RAP2A, RAP2B, RAP2C, RCC2, RETSAT, RHEB, RIC1, SAR1A, SARM1, SCAMP2, SCARB2, SCFD1, SCYL1, SEC13, SEC16A, SEC22B, SEC23A, SEC23B, SEC24A, SEC24B,	Downregulation

					SEC24C, SEC24D, SEC31A, SEC61G, SEH1L, SEPTIN2, SEPTIN8, SERPINB6, SH3GL3, SH3GLB1, SLC12A4, SLC2A1, SLC33A1, SLC44A1, SLC44A2, SNTB2, SNX3, SNX4, SNX6, SPAG9, SPARC, SRPRA, SRPRB, SSR4, STAM2, STIM1, STX12, STX5, SUN2, SURF4, TF, TIGAR, TMED10, TMED2, TMED5, TMED7, TMED9, TMEM115, TMEM167A, TMEM192, TMEM97, TMF1, TMX3, TOMM34, TPST1, TTC1, UBR4, VAMP2, VAMP3, VAT1, VLDLR, VPS25, VPS26A, VPS35, VPS35L, VPS36, VPS53, YIF1A, YKT6, ZDHHC17, ZFYVE16]	
Golgi apparatus subcompartment	GO_CellularComponent-EBI-UniProt-GOA-ACAP-ARAP_13.05.2021_00h00	0.079766	12.18962	108	[ACBD3, ADAM10, AP1M1, AP1S1, ARAP1, ARCN1, ARFGAP1, ARFGAP3, ARFGEF1, ARL1, ARL3, B4GALT1, BET1, BIRC6, BPNT2, CAV1, CDC42, CHID1, CHPF, CHST14, COG1, COG6, COG7, COG8, COPA, COPB1, COPB2, COPE, COPG1, COPG2, COPZ1, DBNL, ECPAS, EGFR, ERGIC1, FNDC3A, FUT11, GALNT1, GALNT10, GALNT2, GBF1, GOLGA2, GOLGA3, GOLIM4, GOLT1B, GOPC, GOSR2, GPSM1, HLA-A, HSPD1, HUWE1, KDELR1, KDELR3, LMAN1, LPCAT1, LYPLA2, MAN1A1, MAN1A2, MGAT2, MME, NCAM1, NSF, NSFL1C, OPTN, OSBP, PHIP, PIK3C2A, PLD3, PLOD3, PLPP3, PREPL, PRKCSH, PYCARD, RAB14, RAB1A, RAB2A, RAB32, RAB8A, RHEB, RIC1, SCAMP2, SCARB2, SCFD1, SEC16A, SEC22B, SEC23A, SH3GLB1, SLC2A1, SLC33A1, SNX9, STX12, STX5, SURF4, TMED10, TMED2, TMED7, TMED9, TMEM115, TMEM167A, TMF1, TPST1, VAMP2, VAMP3, VPS53, VRK1, YIF1A, YKT6, ZDHHC17]	Downregulation
endoplasmic reticulum subcompartment	GO_CellularComponent-EBI-UniProt-GOA-ACAP-ARAP_13.0	0.079766	13.0217	156	[ABHD12, ACSL1, ACSL4, ADPGK, ALG11, ALG2, ALG5, ALG9, ARCN1, ARL6IP5, ASPH, ATL2, ATL3, AUP1, B3GLCT, BET1, CALR, CALU, CAMK2D, CANX,	Downregulation

	5.2021_00h00				CAV1, CDC42, CHEK2, CKAP4, COPA, COPB1, COPB2, COPE, COPG1, COPG2, COPZ1, CYB5R1, CYB5R3, CYP2S1, CYP51A1, DDRGK1, DEGS1, DHRS7B, DLG1, EGFR, EMD, EPM2AIP1, ERAP1, ERGIC1, ERGIC2, ERLIN2, ERO1A, ERP44, ESYT2, FAF2, FKBP8, GALNT1, GALNT2, GOSR2, GPSM1, HLA-A, HMOX1, HMOX2, HSP90B1, HSPA5, IKBIP, ILVBL, KDELR1, KDELR3, KTN1, LBR, LMAN1, LMF2, LNPK, LPCAT1, LPGAT1, LRRC59, LSS, MARCHF5, MEST, MGST1, MOGS, MOSPD2, MOXD1, MTDH, NBAS, NSDHL, NUP210, OSBP, OSTC, P4HTM, PCYT1A, PCYT2, PDIA3, PDIA6, PIGS, PIGT, PIP4K2B, PLD3, PLOD1, PLOD2, PLOD3, PLPP3, PNPT1, POFUT2, POR, PRKCSH, PSMD2, PTGIS, PTPN1, RAB18, RAB2A, RDH10, RETSAT, RHEB, RNF170, RPN1, RRBP1, SBF1, SCARB2, SCFD1, SDF2L1, SEC13, SEC16A, SEC22B, SEC23A, SEC23B, SEC24A, SEC24B, SEC24C, SEC24D, SEC31A, SEC61G, SEL1L, SLC33A1, SPTLC2, SRPRA, SRPRB, SSR1, SSR4, STIM1, STT3A, STT3B, STX5, SURF4, TBL2, TEX2, TKT, TMED10, TMED2, TMED5, TMED7, TMED9, TMEM214, TMEM97, TMX3, TRAM1, UFL1, USE1, USP19, YIF1A]	
vesicle budding from membrane	GO_BiologicalProcess-EBI-UniProt-GOA-ACAP-ARAP_13.05.2021_00h00	0.079766	29.31035	34	[ANKRD28, ANXA2, AP2M1, AP3B1, ARFGAP3, BET1, CTSC, FNBP1L, GBF1, GOLGA2, GOSR2, LMAN1, NSF, PICALM, RAB1A, S100A10, SAR1A, SCFD1, SEC13, SEC16A, SEC22B, SEC23A, SEC23B, SEC24A, SEC24B, SEC24C, SEC24D, SEC31A, SNX3, STX5, TFG, TMED10, TMED2, TMED9]	Downregulation
transport vesicle	GO_CellularComponent-EBI-UniProt-GOA-ACAP-ARAP_13.0	0.079766	13.30275	58	[ADAM10, AP1S1, AP2A1, AP2A2, ARCN1, ATP2B1, BET1, BIN1, COPA, COPB1, COPB2, COPE, COPG1, COPG2, COPS5, COPZ1, DPYSL3, GIPC1, GOLIM4, GOPC,	Downregulation

	5.2021_00h00				GOSR2, HLA-A, KDELR1, KDELR3, LMAN1, MME, PICALM, PLIN3, PXDN, RAB14, RAB1A, RAB8A, SAR1A, SCAMP2, SEC13, SEC16A, SEC22B, SEC23A, SEC23B, SEC24A, SEC24B, SEC24C, SEC24D, SEC31A, SEPTIN2, SEPTIN8, SNTB2, STX12, STX5, SURF4, TMED10, TMED2, TMED7, TMED9, TMEM168, VAMP2, VAMP3, YKT6]	
endoplasmic reticulum to Golgi vesicle-mediated transport	GO_BiologicalProcess-EBI-UniProt-GOA-ACAP-ARAP_13.05.2021_00h00	0.079766	29.01786	65	[ACTR10, ANK2, ANK3, ANKRD28, ARCN1, ARF4, ARFGAP1, ARFGAP3, ATL2, ATL3, BET1, CAPZA2, COG1, COG6, COG7, COG8, COPA, COPB1, COPB2, COPE, COPG1, COPG2, COPZ1, CTSC, DCTN1, DCTN4, DYNC1LI2, ERGIC1, ERGIC2, GBF1, GOLGA2, GOPC, GOSR2, KDELR1, KDELR3, LMAN1, NSF, RAB1A, RAB2A, SAR1A, SCFD1, SEC13, SEC16A, SEC22B, SEC23A, SEC23B, SEC24A, SEC24B, SEC24C, SEC24D, SEC31A, SPTAN1, SPTBN1, SPTBN2, STX5, TFG, TMED10, TMED2, TMED5, TMED7, TMED9, TMEM115, USE1, YIF1A, YKT6]	Downregulation
retrograde vesicle-mediated transport, Golgi to endoplasmic reticulum	GO_BiologicalProcess-EBI-UniProt-GOA-ACAP-ARAP_13.05.2021_00h00	0.079766	38.94737	37	[ARCN1, ARF4, ARFGAP1, ARFGAP3, COG7, COPA, COPB1, COPB2, COPE, COPG1, COPG2, COPZ1, ERGIC1, ERGIC2, GBF1, HTT, KDELR1, KDELR3, KIF11, KIF22, KIF23, KIF2C, KLC1, NBAS, NSF, PLPP3, RAB1A, SCFD1, SCYL1, SEC22B, SURF4, TMED10, TMED2, TMED7, TMED9, TMEM115, USE1]	Downregulation
intra-Golgi vesicle-mediated transport	GO_BiologicalProcess-EBI-UniProt-GOA-ACAP-ARAP_13.05.2021_00h00	0.079766	33.33333	12	[COG1, COG6, COG8, COPA, COPB1, COPB2, COPE, COPG1, COPG2, COPZ1, GOSR2, NSF]	Downregulation
Golgi organization	GO_BiologicalProcess-EBI-UniProt-GOA-ACAP-ARAP_13.05.2021_00h00	0.079766	20.4969	33	[ARFGEF1, ARL1, ATL2, ATL3, BET1, CDC42, CLASP1, COG1, COG7, CUL7, FBXW8, GBF1, GOLGA2, GOSR2, HTT, HUWE1, LMAN1, MAPK1, MYO5A, NSFL1C, OPTN, RAB1A, RAB2A, SEC16A, SEC22B, STX5, SURF4, TMED10, TMED2,	Downregulation

					TMED5, TMED7, TMED9, VRK1]	
vesicle organization	GO_BiologicalProcess-EBI-UniProt-GOA-ACAP-ARAP_13.05.2021_00h00	0.079766	18.25843	65	[ANKFY1, ANKRD28, ANXA1, ANXA2, AP1M1, AP2M1, AP3B1, ARFGAP3, BET1, CALR, CAV1, CD2AP, CORO1C, CTSC, EEA1, FNBP1L, GBF1, GOLGA2, GOSR2, HOOK3, IST1, KIF5B, LMAN1, MVB12A, NECTIN2, NSF, OSBP, PARD3, PICALM, PIP4K2B, RAB14, RAB1A, RAB32, RAB8A, S100A10, SAR1A, SCARB2, SCFD1, SEC13, SEC16A, SEC22B, SEC23A, SEC23B, SEC24A, SEC24B, SEC24C, SEC24D, SEC31A, SEPTIN8, SNX3, STAM2, STX12, STX5, TBC1D4, TBPL1, TFG, TMED10, TMED2, TMED9, TMF1, VAMP2, VAMP3, VPS25, VPS36, ZFYVE16]	Downregulation
endoplasmic reticulum-Golgi intermediate compartment membrane	GO_CellularComponent-EBI-UniProt-GOA-ACAP-ARAP_13.05.2021_00h00	0.079766	26.19048	22	[BET1, CALR, CTSC, ERGIC1, ERGIC2, GALNT1, GOLGA2, GOSR2, KDELR1, LMAN1, PLPP3, RAB2A, SEC22B, STX5, SURF4, TMED10, TMED2, TMED5, TMED7, TMED9, YIF1A, YKT6]	Downregulation
vesicle targeting, to, from or within Golgi	GO_BiologicalProcess-EBI-UniProt-GOA-ACAP-ARAP_13.05.2021_00h00	0.079766	33.33333	26	[ANKRD28, ARFGAP3, BET1, CTSC, GBF1, GOLGA2, GOSR2, LMAN1, NSF, RAB1A, SAR1A, SCFD1, SEC13, SEC16A, SEC22B, SEC23A, SEC24A, SEC24B, SEC24C, SEC24D, SEC31A, STX5, TFG, TMED10, TMED2, TMED9]	Downregulation
Golgi-associated vesicle	GO_CellularComponent-EBI-UniProt-GOA-ACAP-ARAP_13.05.2021_00h00	0.079766	29.89691	29	[ADAM10, AP1S1, AP2A1, AP2A2, ARCN1, COPA, COPB1, COPB2, COPE, COPG1, COPG2, COPZ1, DIPK2A, GOPC, KDELR1, KDELR3, PACS1, PI4KA, RAB14, RAB8A, SCFD1, SCYL1, SEC22B, TMED10, TMED2, TMED7, TMED9, USE1, ZDHHC17]	Downregulation
coated vesicle	GO_CellularComponent-EBI-UniProt-GOA-ACAP-ARAP_13.05.2021_00h00	0.079766	21.76656	69	[ADAM10, AP1M1, AP1S1, AP2A1, AP2A2, AP2B1, AP2M1, AP2S1, AP3B1, APOE, ARCN1, COPA, COPB1, COPB2, COPE, COPG1, COPG2, COPZ1, CTSC, DBNL, DIPK2A, ECE1, ECPAS, EGFR, EPN2, ERGIC1, ERGIC2, GOLGA2, GOPC, GOSR2, HLA-A, HSPD1, KDELR1, KDELR3, LMAN1, LRP1, MYO1E, NECAP2, PACS1, PICALM,	Downregulation

					PIK3C2A, RAB14, RAB8A, SAR1A, SCARB2, SCYL1, SEC13, SEC16A, SEC22B, SEC23A, SEC23B, SEC24A, SEC24B, SEC24C, SEC24D, SEC31A, SNX3, SNX9, STX5, TF, TMED10, TMED2, TMED5, TMED7, TMED9, USE1, VAMP2, VAMP3, YIF1A]	
Golgi membrane	GO_CellularComponent-EBI-UniProt-GOA-ACAP-ARAP_13.05.2021_00h00	0.079766	12.10526	92	[ACBD3, ADAM10, AP1M1, AP1S1, ARAP1, ARCN1, ARFGAP1, ARFGAP3, ARFGEF1, ARL1, ARL3, B4GALT1, BET1, BPNT2, CAV1, CDC42, CHPF, CHST14, COG1, COG6, COG7, COG8, COPA, COPB1, COPB2, COPE, COPG1, COPG2, COPZ1, DBNL, EGFR, ERGIC1, FNDC3A, FUT11, GALNT1, GALNT10, GALNT2, GBF1, GOLGA2, GOLGA3, GOLIM4, GOLT1B, GOPC, GOSR2, GPSM1, HLA-A, HUWE1, KDELR1, KDELR3, LMAN1, LPCAT1, MAN1A1, MAN1A2, MGAT2, NCAM1, OPTN, OSBP, PLD3, PLPP3, PRKCSH, PYCARD, RAB14, RAB1A, RAB2A, RAB8A, RHEB, RIC1, SCAMP2, SCARB2, SCFD1, SEC16A, SEC22B, SEC23A, SH3GLB1, SLC2A1, SLC33A1, STX12, STX5, SURF4, TMED10, TMED2, TMED7, TMED9, TMEM115, TMEM167A, TMF1, TPST1, VAMP3, VPS53, YIF1A, YKT6, ZDHHC17]	Downregulation
cytoplasmic vesicle membrane	GO_CellularComponent-EBI-UniProt-GOA-ACAP-ARAP_13.05.2021_00h00	0.079766	12.48514	105	[ADAM10, ANO6, ANXA1, ANXA3, AP1M1, AP1S1, AP2A1, AP2A2, AP2B1, AP2M1, AP2S1, AP3B1, APOE, APPL2, ARCN1, ATP2B1, B4GALT1, CALR, CAMK2D, CAV1, CD44, CKAP4, COPA, COPB1, COPB2, COPE, COPG1, COPG2, COPZ1, CYB5R1, DBNL, DEGS1, DIAPH1, DIPK2A, DSP, EGFR, EPN2, GOSR2, HLA-A, HMOX2, HTT, IQGAP1, IQGAP2, ITGAV, KDELR1, KDELR3, LMAN1, LPCAT1, LRP1, MANBA, MFGE8, MGST1, MME, MOSPD2, MOXD1, MYO1C, MYO1E, NECAP2, PICALM, PIK3R4, PRKCSH, RAB14, RAB18, RAB1A,	Downregulation

					RAB23, RAB32, RAB5B, RAB5C, RAB8A, RAP2B, RAP2C, SAR1A, SCARB2, SCYL1, SEC13, SEC16A, SEC22B, SEC23A, SEC23B, SEC24A, SEC24B, SEC24C, SEC24D, SEC31A, SEPTIN8, SERPINB6, SLC44A2, SNTB2, SNX17, SNX9, SPARC, STX12, STX5, SURF4, TF, TMED10, TMED2, TMED7, TMX3, UBR4, VAMP2, VAMP3, VPS35L, YKT6, ZDHHC17]	
COPII-coated vesicle budding	GO_BiologicalProcess-EBI-UniProt-GOA-ACAP-ARAP_13.05.2021_00h00	0.079766	31.16883	24	[ANKRD28, BET1, CTSC, GOLGA2, GOSR2, LMAN1, NSF, RAB1A, SAR1A, SCFD1, SEC13, SEC16A, SEC22B, SEC23A, SEC23B, SEC24A, SEC24B, SEC24C, SEC24D, SEC31A, STX5, TFG, TMED10, TMED2]	Downregulation
vesicle coating	GO_BiologicalProcess-EBI-UniProt-GOA-ACAP-ARAP_13.05.2021_00h00	0.079766	35.13514	26	[ANKRD28, ARFGAP3, BET1, CTSC, GBF1, GOLGA2, GOSR2, LMAN1, NSF, RAB1A, SAR1A, SCFD1, SEC13, SEC16A, SEC22B, SEC23A, SEC24A, SEC24B, SEC24C, SEC24D, SEC31A, STX5, TFG, TMED10, TMED2, TMED9]	Downregulation
COPII-coated vesicle cargo loading	GO_BiologicalProcess-EBI-UniProt-GOA-ACAP-ARAP_13.05.2021_00h00	0.079766	62.5	10	[RAB1A, SAR1A, SEC13, SEC23A, SEC23B, SEC24A, SEC24B, SEC24C, SEC24D, SEC31A]	Downregulation
COPII-coated ER to Golgi transport vesicle	GO_CellularComponent-EBI-UniProt-GOA-ACAP-ARAP_13.05.2021_00h00	0.079766	26.26263	26	[CTSC, ECPAS, ERGIC1, ERGIC2, GOLGA2, GOSR2, HLA-A, LMAN1, SAR1A, SEC13, SEC16A, SEC22B, SEC23A, SEC23B, SEC24A, SEC24B, SEC24C, SEC24D, SEC31A, STX5, TMED10, TMED2, TMED5, TMED7, TMED9, YIF1A]	Downregulation
COPI-coated vesicle	GO_CellularComponent-EBI-UniProt-GOA-ACAP-ARAP_13.05.2021_00h00	0.079766	56.25	18	[ARCN1, COPA, COPB1, COPB2, COPE, COPG1, COPG2, COPZ1, DIPK2A, KDELR1, KDELR3, PACS1, SCYL1, SEC22B, TMED10, TMED2, TMED7, USE1]	Downregulation
Golgi-associated vesicle membrane	GO_CellularComponent-EBI-UniProt-GOA-ACAP-ARAP_13.0	0.079766	37.03704	20	[AP1S1, AP2A1, AP2A2, ARCN1, COPA, COPB1, COPB2, COPE, COPG1, COPG2, COPZ1, DIPK2A, GOPC, KDELR1, KDELR3, PI4KA, SCYL1, TMED2, TMED7, ZDHHC17]	Downregulation

	5.2021_00h00					
vesicle coat	GO_CellularComponent-EBI-UniProt-GOA-ACAP-ARAP_13.05.2021_00h00	0.079766	51.78571	29	[AP1S1, AP2A1, AP2A2, AP2B1, AP2M1, AP2S1, ARCN1, COPA, COPB1, COPB2, COPE, COPG1, COPG2, COPZ1, DIPK2A, EPN2, NECAP2, PICALM, SAR1A, SCYL1, SEC13, SEC23A, SEC23B, SEC24A, SEC24B, SEC24C, SEC24D, SEC31A, TMED7]	Downregulation
coated vesicle membrane	GO_CellularComponent-EBI-UniProt-GOA-ACAP-ARAP_13.05.2021_00h00	0.079766	24.61539	48	[AP1M1, AP1S1, AP2A1, AP2A2, AP2B1, AP2M1, AP2S1, AP3B1, APOE, ARCN1, COPA, COPB1, COPB2, COPE, COPG1, COPG2, COPZ1, DBNL, DIPK2A, EGFR, EPN2, GOSR2, HLA-A, KDELR1, KDELR3, LMAN1, NECAP2, PICALM, SAR1A, SCARB2, SCYL1, SEC13, SEC16A, SEC22B, SEC23A, SEC23B, SEC24A, SEC24B, SEC24C, SEC24D, SEC31A, STX5, TF, TMED10, TMED2, TMED7, VAMP2, VAMP3]	Downregulation
ER to Golgi transport vesicle membrane	GO_CellularComponent-EBI-UniProt-GOA-ACAP-ARAP_13.05.2021_00h00	0.079766	26.86567	18	[GOSR2, HLA-A, LMAN1, SAR1A, SEC13, SEC16A, SEC22B, SEC23A, SEC23B, SEC24A, SEC24B, SEC24C, SEC24D, SEC31A, STX5, TMED10, TMED2, TMED7]	Downregulation
COPI vesicle coat	GO_CellularComponent-EBI-UniProt-GOA-ACAP-ARAP_13.05.2021_00h00	0.079766	78.57143	11	[ARCN1, COPA, COPB1, COPB2, COPE, COPG1, COPG2, COPZ1, DIPK2A, SCYL1, TMED7]	Downregulation
COPII vesicle coat	GO_CellularComponent-EBI-UniProt-GOA-ACAP-ARAP_13.05.2021_00h00	0.079766	66.66666	10	[SAR1A, SEC13, SEC23A, SEC23B, SEC24A, SEC24B, SEC24C, SEC24D, SEC31A, TMED7]	Downregulation
COPI-coated vesicle membrane	GO_CellularComponent-EBI-UniProt-GOA-ACAP-ARAP_13.05.2021_00h00	0.079766	73.68421	14	[ARCN1, COPA, COPB1, COPB2, COPE, COPG1, COPG2, COPZ1, DIPK2A, KDELR1, KDELR3, SCYL1, TMED2, TMED7]	Downregulation
cytoskeletal protein binding	GO_MolecularFunction-EBI-UniProt-GOA-ACAP-ARAP_13.0	3.10E-35	15.85014	165	[AATF, ABI1, ACTN1, ACTN4, ACTR2, ADD2, ADD3, AGTPBP1, ALDOC, ANK2, ANK3, ANTXR1, ANXA2, ANXA6, APOE, ARFGEF1, ARL3,	Downregulation

	5.2021_00h00				ARPC1A, ATP1A1, B4GALT1, BAG2, BIN1, CACYBP, CALD1, CAMK2D, CAP1, CAP2, CAPN2, CAPZA2, CCT5, CDC42EP3, CFL2, CGN, CKAP5, CLASP1, CNN1, CNN3, COBLL1, CORO1C, CRMP1, CSRP2, CYFIP1, DBNL, DCTN1, DIAPH1, DLG1, DLGAP5, DPYSL2, DPYSL3, DRG1, DSTN, DUSP3, EEF2, EGFR, EMD, EML1, EPB41, EPB41L3, EPB41L5, EPS8, EPS8L2, FARP1, FERMT2, FKBP15, FKBP4, FLNB, FLNC, FSCN1, FSD1, GIPC1, GOLGA2, GSN, HOOK3, HSP90AB1, HSPA2, HSPH1, HTT, IQGAP1, IQGAP2, IQGAP3, ITGB1, KIF11, KIF20A, KIF21A, KIF22, KIF23, KIF2C, KIF5B, KIRREL1, KTN1, LASP1, LRPPRC, MACF1, MAP1B, MAP1LC3A, MAP1S, MAP4, METAP1, MICAL1, MICAL3, MPRIP, MSN, MYH10, MYH9, MYL9, MYO1C, MYO1E, MYO5A, MYO9B, NCAPG, NDRG1, NFKB1, NOL6, NUSAP1, P4HB, PAK1, PALLD, PARVA, PAWR, PDLIM2, PDLIM4, PDLIM5, PDLIM7, PICALM, PLEC, PLK1, PLS3, PPP1R18, PPP1R9B, PRKAA1, PRUNE1, PXDN, PXK, PYCARD, RAB14, RAB8A, RAI14, RCC2, RDX, RELA, ROCK2, SBDS, SNTB2, SNX6, SORBS1, SORBS3, SPAG9, SPTAN1, SPTBN1, SPTBN2, STIM1, SUN2, TAGLN, TBCEL, TLN1, TLN2, TNS1, TPM1, TPM4, TPX2, TTLL12, TWF1, UTRN, VAMP2, ZNF185]	
non-membrane-bounded organelle	GO_CellularComponent-EBI-UniProt-GOA-ACAP-ARAP_13.05.2021_00h00	3.10E-35	11.66604	619	[AAAS, AATF, ABCF1, ABHD14B, ABI1, ACACA, ACIN1, ACOT13, ACSL4, ACTBL2, ACTN1, ACTN4, ACTR10, ACTR1B, ACTR2, ADAR, ADARB1, ADD2, ADD3, AEBP2, AGO1, AGO2, AGTPBP1, AK1, AKT1, ALDOC, ALG2, ANK2, ANK3, ANP32E, ANTXR1, ANXA1, ANXA11, ANXA2, APOBEC3C, ARAP3, ARFGEF1, ARHGDIA, ARL2, ARL3, ARL6IP5, ARPC1A, ASAP1, ATP2B4, ATR, AUP1, AURKA, AURKB,	Upregulation

					BAG2, BAG3, BAIAP2, BAZ1B, BCCIP, BIN1, BIRC6, BMS1, BOP1, BPTF, BRD4, BRMS1, BRWD1, BUB1B, C1QBP, CALCOCO2, CALD1, CANX, CAP1, CAPN2, CAPRIN1, CAPZA2, CARHSP1, CASK, CAV1, CBX2, CBX5, CCAR2, CCNB1, CCND2, CCT2, CCT3, CCT4, CCT5, CCT6A, CCT7, CCT8, CD2AP, CDC42, CDC42BPA, CDC42BPB, CDC42EP1, CDC42EP3, CDC73, CDCA8, CDH2, CDK5RAP3, CDK7, CELF1, CEP170, CFAP20, CFAP298, CFL2, CGN, CHAF1A, CHD4, CHEK1, CHEK2, CHTF18, CIRBP, CKAP4, CKAP5, CLASP1, CMPK1, CNN3, COG7, COPS5, CORO1C, CRMP1, CSDE1, CSRP2, CTCF, CTNNB1, CTNNBL1, CTSC, CUL7, CYB5R3, CYFIP1, DAPK1, DAXX, DBNL, DCAF1, DCAF13, DCTN1, DCTN4, DCXR, DDRGK1, DDX18, DDX20, DDX21, DDX24, DDX28, DDX31, DDX46, DDX47, DDX5, DDX52, DDX54, DDX6, DHX15, DHX30, DHX33, DHX37, DHX9, DIAPH1, DLG1, DLGAP5, DNMT1, DNMT3A, DNTTIP2, DPH6, DPYSL2, DPYSL3, DSP, DSTN, DYNC1LI2, ECPAS, ECT2, EEF1E1, EEF2, EHD1, EHD2, EHMT1, EIF3A, EIF4A3, ELAC2, ELP3, EMD, EML1, EPB41, EPB41L3, EPB41L5, ERCC2, ERCC6L, ERGIC2, ESF1, EXD2, EXOSC10, EXOSC2, EXOSC5, EXOSC7, FAF2, FANCD2, FARP1, FASTKD2, FERMT2, FH, FKBP15, FKBP4, FLNB, FLNC, FLT1, FNBP1L, FOXK1, FSCN1, FSD1, FTSJ3, FXR1, G3BP1, G3BP2, GARS1, GATA6, GATAD2A, GBF1, GEMIN4, GET3, GET4, GINS3, GNG12, GNL2, GNL3, GOLGA2, GOLGA3, GPHN, GSN, GTF3C1, GTF3C3, HADHA, HADHB, HAT1, HDAC2, HDAC4, HEATR1, HELLS, HERC2, HK2, HMMR, HMOX1, HNRNPL, HNRNPM, HOOK3, HP1BP3, HSD17B10, HSD17B4, HSP90AB1, HSPA14, HSPA2, HSPA9, HSPB1,	

					HSPB11, HSPH1, HTT, IGF2BP3, ILF2, ILF3, ILK, IMP3, INCENP, INO80C, IPO4, IPO5, IQGAP1, IQGAP2, IRF3, IST1, JADE1, JAM3, KAT7, KDM1A, KDM3B, KEAP1, KIF11, KIF20A, KIF21A, KIF22, KIF23, KIF2C, KIF5B, KLC1, KNTC1, KRT18, KRT19, KRT8, LANCL2, LAS1L, LASP1, LIN28A, LMAN1, LPCAT1, LPP, LRP1, LRPPRC, LRRC59, LRWD1, LSM4, LSS, LYAR, MACF1, MACROH2A1, MAGED1, MAGED2, MAP1B, MAP1LC3A, MAP1S, MAP2K6, MAP4, MAPK1, MAPK14, MARS1, MASTL, MBD3, MBNL1, MCC, MCM2, MCM3, MCM4, MCM5, MCM6, MCM7, MDC1, MDN1, MEAK7, METAP1, METTL1, MEX3A, MICAL1, MICAL3, MKI67, MLH1, MMAB, MPHOSPH10, MPRIP, MRPS27, MRPS9, MSH2, MSH6, MSN, MTA3, MTDH, MTREX, MVB12A, MYADM, MYBBP1A, MYH10, MYH9, MYL9, MYO1C, MYO1E, MYO5A, MYO9B, NCAPD2, NCAPG, NCAPG2, NCKAP1, NCL, NDC1, NDC80, NDRG1, NEDD4, NEK7, NFKB1, NIT2, NLE1, NOL10, NOL11, NOL6, NOL9, NOP14, NOP56, NOP58, NOP9, NPM1, NPM3, NSDHL, NSFL1C, NSUN2, NSUN5, NUDT16, NUP107, NUP133, NUP160, NUSAP1, NVL, ORC2, ORC3, ORC4, ORC5, OSBP, P3H4, P4HB, PAK1, PALLD, PARD3, PARN, PARP1, PARVA, PAWR, PDCD11, PDLIM2, PDLIM4, PDLIM5, PDLIM7, PDS5A, PEA15, PELP1, PES1, PHIP, PIK3R4, PKP2, PLEC, PLEKHA7, PLIN3, PLK1, PLS3, PNO1, PNPT1, POGZ, POLA1, POLA2, POLD1, POLE, POLR1B, POLR2A, POLR2B, POLR2G, PPID, PPM1B, PPP1R18, PPP1R9B, PPP2R5A, PREPL, PRIM1, PRIM2, PRKACB, PRKAR1A, PRKAR2A, PRKAR2B, PRKCA, PRKDC, PSIP1, PSMD1, PSMD2, PSPC1, PTCD3, PTPN12, PURA,	

					PWP2, PXDN, PXK, PYCARD, PYCR3, RAB18, RAB23, RAB5C, RAB8A, RABL6, RAC3, RAD51, RAI14, RAN, RANGAP1, RBBP5, RBM3, RBP1, RBPJ, RBPMS, RCC2, RDH10, RDX, RELA, RFC1, RFC2, RFC3, RFC5, RIF1, RIOX1, ROCK2, RP2, RPA1, RPA2, RPA3, RPF2, RPL13A, RPL22, RPL27A, RPL4, RPL5, RPL7A, RPS2, RPS21, RPS6KA3, RPS8, RRBP1, RRP12, RRP7A, RRS1, RTKN, RTTN, SAMD4B, SAMHD1, SAP30BP, SARM1, SBDS, SCYL1, SDAD1, SEC13, SEH1L, SELENBP1, SEPTIN10, SEPTIN11, SEPTIN2, SEPTIN7, SEPTIN8, SESTD1, SET, SETD7, SETDB1, SH3GL3, SIN3A, SIRT1, SIRT2, SKA3, SKP2, SLAIN2, SLC25A5, SLC2A1, SMARCA4, SMARCA5, SMARCAD1, SMARCC1, SMARCD1, SMARCD2, SMC2, SNTB2, SNX4, SNX9, SORBS1, SORBS3, SPAG9, SPATS2L, SPECC1L, SPOUT1, SPTAN1, SPTBN1, SPTBN2, SRC, SRPK1, SRPRB, SSB, SSBP1, SSRP1, STIM1, STXBP2, SUN2, SUPT16H, SUPT6H, SYMPK, TACC3, TBCEL, TBL3, TCP1, TDP2, TELO2, TEX10, TFB1M, TGFB1I1, TGM2, THUMPD3, TIA1, TLN1, TLN2, TMA16, TMEM214, TNS1, TOP2A, TPM1, TPM4, TPX2, TRIM28, TRIM71, TRIP13, TRMT10C, TSEN34, TSR1, TTC28, TTI1, TTK, TTLL12, TUBA4A, TUBB6, TUT1, TWF1, TXNRD1, UBR4, UFL1, UPF2, UPF3B, USP7, UTP15, UTP18, UTP20, UTP25, UTP4, UTRN, VIM, VRK1, VRTN, WAPL, WDHD1, WDR18, WDR3, WDR36, WDR43, WRAP53, XRCC5, XRCC6, XRN1, XRN2, YTHDC2, ZNF185, ZWILCH]	
positive regulation of cellular process	GO_BiologicalProcess-EBI-UniProt-GOA-ACAP-ARAP_13.0	3.10E-35	9.758569	578	[AATF, ABAT, ABCB10, ABCB7, ABHD14B, ABI1, ACACA, ACIN1, ACLY, ACSL1, ACSL4, ACTN1, ACTN4, ACTR2, ADAM10, AGO1, AGO2, AGTPBP1, AIMP2, AKT1, ANK2, ANK3, ANKFY1,	No change

	5.2021_00 h00				ANO6, ANTXR1, ANXA1, ANXA2, ANXA3, AP2A1, AP2B1, AP2M1, AP3B1, APAF1, APOE, APPL2, ARAP1, ARF4, ARF6, ARFGEF1, ARHGDIA, ARHGEF1, ARHGEF11, ARHGEF12, ARL2, ARL3, ARL6IP5, ARPC1A, ARSB, ASAP1, ASNS, ASPH, ATF7IP, ATL3, ATP2B4, ATR, AURKA, AURKB, B4GALT1, BAG2, BAG3, BAIAP2, BIN1, BIRC6, BPTF, BRAT1, BRD4, BRMS1, C1QBP, CACYBP, CALCOCO2, CALR, CAMK1, CAMK2D, CAPN1, CAPN2, CAPNS1, CAPRIN1, CASK, CASP3, CAT, CAV1, CAVIN1, CBL, CBR1, CCAR1, CCAR2, CCDC22, CCNB1, CCND2, CCNY, CCT2, CCT3, CCT4, CCT5, CCT6A, CCT7, CCT8, CD2AP, CD44, CD81, CDC123, CDC42, CDC42EP1, CDC42EP3, CDC73, CDH2, CDH3, CDK5RAP1, CDK5RAP3, CDK7, CEBPZ, CELF1, CFAP20, CFL2, CHEK1, CHEK2, CHTF18, CIRBP, CKAP5, CLASP1, CNOT11, CNTFR, COL1A1, COLGALT1, COPS5, CORO1C, CRLF3, CSPG4, CTBP2, CTCF, CTNNB1, CTNNBL1, CTSC, CUL4A, CUL4B, CUL7, CYFIP1, DAGLB, DAPK1, DAXX, DAZAP1, DBNL, DCTN1, DCXR, DDRGK1, DDX20, DDX21, DDX39B, DDX49, DDX5, DDX6, DHPS, DHX33, DHX8, DHX9, DIAPH1, DICER1, DIPK2A, DLG1, DLGAP5, DNMT1, DNMT3A, DNMT3B, DOCK7, DPYSL2, DPYSL3, DRG1, DSTN, DUSP3, ECE1, ECT2, EDRF1, EEF1E1, EEF2, EFNB2, EGFL6, EGFR, EHD1, EHD2, EHD4, EIF4A3, ELP3, EMD, EPB41, EPB41L5, EPCAM, EPHB3, EPM2AIP1, EPN2, EPS8, EPS8L2, ERBIN, ERCC2, ETFA, EXOSC2, EXOSC5, EXOSC7, F11R, FASTKD2, FBN2, FBXW8, FERMT2, FGG, FKBP8, FLNB, FLT1, FN1, FNBP1L, FOXK1, FSCN1, FXR1, G3BP1, G3BP2, GAB1, GARS1, GATA6, GCLC, GDI1, GIPC1, GLMN, GLUD1, GLUL, GNL3, GOLGA2,	

					GOLT1B, GPC1, GPC3, GPD1L, GPI, GPSM1, GSDME, GSN, GSTM2, GTF2I, GTPBP1, GUF1, HCFC1, HDAC2, HDAC4, HEATR1, HLA-A, HMOX1, HNRNPLL, HSD17B4, HSP90AB1, HSPA2, HSPA5, HSPB1, HSPD1, HSPE1, HSPH1, HTRA1, HTT, HUWE1, ILF2, ILF3, ILK, INCENP, IPO5, IQGAP1, IQGAP2, IQGAP3, IRF2BPL, IRF3, IST1, ITCH, ITGA5, ITGA6, ITGAV, ITGB1, JAM3, JARID2, KAT7, KDM1A, KEAP1, KIF23, KIF5B, LAMB1, LAMC1, LANCL2, LARS1, LGALS1, LIMS1, LIN28A, LMAN1, LMCD1, LNPK, LPGAT1, LRP1, LRRC59, LRRC8A, LRRK1, LSM4, LYAR, MACF1, MACROH2A1, MAGED1, MAP1B, MAP2K6, MAPK1, MAPK14, MAPK8, MARCHF5, MARS1, MAVS, MCAM, MCU, MEAK7, MESD, METAP1, MFGE8, MIPEP, MLH1, MMAB, MME, MOSPD2, MPC2, MRPS27, MSH2, MSN, MTA3, MTDH, MVD, MYADM, MYBBP1A, MYD88, MYDGF, MYH10, MYH9, MYO1C, MYO1E, NCAPG, NCKAP1, NCL, NDC80, NDRG1, NECTIN2, NEDD4, NEK7, NFKB1, NIBAN1, NIBAN2, NID1, NIF3L1, NLE1, NNT, NOL11, NPM1, NQO2, NRP2, NSF, NSFL1C, NSUN5, NUDT16, NVL, OCLN, OGT, OPTN, ORC3, OSBP, OXCT1, P3H1, P4HB, PAK1, PARD3, PARN, PARP1, PASK, PATZ1, PAWR, PAXBP1, PCOLCE, PDCD2, PDGFRB, PDIA3, PDLIM7, PEA15, PELP1, PFKM, PHIP, PHPT1, PIAS4, PICALM, PIK3C2A, PIK3CA, PIK3R4, PIP4K2B, PIP4K2C, PKM, PLCG1, PLK1, PLPP3, PLXNB2, PNPT1, POFUT2, POGZ, POLR1B, POLR2A, POLR2G, POR, PPID, PPM1B, PPM1F, PPP1R9B, PPP2R5A, PRKAA1, PRKACB, PRKACG, PRKAG1, PRKAR1A, PRKAR2A, PRKAR2B, PRKCA, PRKCSH, PRKDC, PRPF38B, PRRC1, PSMC6, PSMD1, PSMD2, PSMD9,	

					PSME3, PTGIS, PTK7, PTPN1, PTPN2, PTPRD, PURA, PXDN, PYCARD, RAB1A, RAC3, RAD51, RAN, RANBP2, RAP2A, RAP2B, RAP2C, RBM15, RBM3, RBPJ, RBPMS, RBPMS2, RCC2, RDH10, RDX, RELA, RFC1, RFC2, RFC3, RFC5, RHEB, RIF1, ROCK2, RPA1, RPA2, RPA3, RPL5, RPS2, RPS6KA1, RPS6KA3, RPS6KA4, RRAS, S100A10, S100A11, SAAL1, SALL2, SAMD4B, SAP30BP, SAR1A, SARM1, SART3, SCARB2, SEC13, SEC16A, SEH1L, SEPTIN7, SESN2, SETD7, SETDB1, SH3GL3, SH3GLB1, SIN3A, SIRT1, SIRT2, SKP2, SLAIN2, SLC1A3, SLC25A12, SLC25A5, SLC2A10, SLC44A2, SLC5A3, SMARCA4, SMARCA5, SMARCC1, SMARCD1, SMARCD2, SNX3, SNX4, SNX9, SORBS1, SORBS3, SORD, SPAG9, SPARC, SPON1, SPTBN1, SRC, SRSF1, SSBP1, SSR1, STIM1, STXBP2, SUN2, SUPT16H, SUPT5H, SUPT6H, SURF4, SYMPK, TBRG4, TCAF1, TCP1, TELO2, TENM3, TF, TFG, TGFB1I1, TGM2, THBS1, TIA1, TIGAR, TIMP3, TM9SF4, TMED10, TMED9, TMF1, TOLLIP, TOP2A, TPM1, TPX2, TRIM28, TRIM71, TRMT10C, TTK, TWF1, UBE2O, UBR5, UFL1, UPF3B, USP15, USP19, USP47, USP5, USP7, USP9X, UTP15, UTRN, VAMP2, VAMP3, VIM, VLDLR, VPS35, WAPL, WARS1, WDR43, WRAP53, XPO5, XRCC5, XRCC6, YAP1, YTHDC2, ZC3HAV1, ZDHHC17, ZFP36L2, ZMYND8, ZNF281, ZNF462]	
regulation of anatomical structure size	GO_BiologicalProcess-EBI-UniProt-GOA-ACAP-ARAP_13.05.2021_00h00	3.10E-35	13.24864	73	[ACTR2, ADD2, ADD3, AKT1, ANO6, AP2M1, APOE, ARF6, ARFGEF1, ARHGAP28, ARPC1A, ATP2B1, BAIAP2, BIN1, CAPZA2, CAV1, CDC42EP1, CDC42EP3, CFL2, CYFIP1, DBNL, DHX15, DHX8, DLG1, DPYSL2, DSTN, ECE1, EGFR, EPS8, FGG, FLNB, FN1, GCLC, GCLM, GDI1, GSN, HP1BP3, HSP90AB1, ILK, IQGAP2, IQGAP3, IST1, KDM1A, LARS1, LRP1, LRRC8A, MACF1, MAP1B, MSN,	Downregulation

					MYADM, MYO1C, MYO1E, NCAPG, NCKAP1, NPM1, NPM3, PAK1, PICALM, PIK3C2A, PIK3CA, PLOD3, PYCARD, RAP1GDS1, RDX, ROCK2, SCPEP1, SIN3A, SLC12A4, SNX9, SPTAN1, SPTBN1, SPTBN2, TWF1]	
supramolecular polymer	GO_CellularComponent-EBI-UniProt-GOA-ACAP-ARAP_13.05.2021_00h00	3.10E-35	13.51351	140	[ACTBL2, ACTN1, AK1, ANK2, ANK3, ANXA1, ARL3, ATP2B4, AURKA, AURKB, BAG2, BAG3, BAIAP2, BIN1, CALD1, CCT2, CCT3, CCT4, CCT5, CCT6A, CCT7, CCT8, CD2AP, CDK5RAP3, CEP170, CFAP20, CFL2, CKAP5, CLASP1, COL1A1, COL1A2, COL4A1, COL4A2, COL5A1, COL5A2, COL6A1, COL6A3, CORO1C, CSRP2, CTNNB1, DBNL, DCTN1, DCTN4, DCXR, DDX6, DLG1, DPYSL2, DPYSL3, DSP, DYNC1LI2, EIF3A, EMD, EML1, FBN2, FERMT2, FKBP4, FLNB, FLNC, FSD1, FXR1, GNG12, GOLGA2, HDAC4, HOOK3, HSPB1, HSPH1, ILK, INCENP, IQGAP1, JAM3, KEAP1, KIF11, KIF20A, KIF21A, KIF22, KIF23, KIF2C, KIF5B, KLC1, KNTC1, KRT18, KRT19, KRT8, LMAN1, LRPPRC, LTBP1, MACF1, MAP1B, MAP1LC3A, MAP1S, MAP4, METAP1, MICAL1, MYH10, MYH9, MYL9, MYO1C, MYO1E, MYO5A, MYO9B, NCKAP1, NDRG1, NEK7, NOL6, NUSAP1, PAK1, PALLD, PARVA, PAWR, PDLIM2, PDLIM4, PDLIM5, PDLIM7, PKP2, PLEC, PLK1, PLS3, PPP2R5A, RCC2, SARM1, SIRT2, SKA3, SLAIN2, SLC2A1, SNTB2, SPECC1L, SPTBN1, SRC, SRPRB, STIM1, STXBP2, TCP1, TMEM214, TPM1, TPM4, TPX2, TUBA4A, TUBB6, TWF1, VIM]	Downregulation
regulation of actin filament-based process	GO_BiologicalProcess-EBI-UniProt-GOA-ACAP-ARAP_13.05.2021_00h00	3.10E-35	15.47619	65	[ABRACL, ACTR2, ADD2, ADD3, ANK2, ARAP1, ARF6, ARFGEF1, ARHGAP28, ARHGDIA, ARPC1A, ATP1A1, BAIAP2, BIN1, CAMK2D, CAPZA2, CAV1, CD2AP, CDC42, CDC42EP1, CDC42EP3, CFL2, CLASP1, CYFIP1, DBNL, DLG1, DSP, DSTN, ECT2, EPS8, F11R, FERMT2,	Downregulation

					FSCN1, GSN, ILK, IQGAP1, IQGAP2, IQGAP3, JAM3, LRP1, MYADM, MYH9, MYO1C, MYO1E, NCAPG, NCKAP1, PAK1, PDGFRB, PHPT1, PIK3CA, PKP2, PPM1F, PXDN, PYCARD, RAC3, RDX, ROCK2, S100A10, SNX9, SORBS3, SPTAN1, SPTBN1, SPTBN2, TPM1, TWF1]	
regulation of cellular component organization	GO_BiologicalProcess-EBI-UniProt-GOA-ACAP-ARAP_13.05.2021_00h00	3.10E-35	12.51476	318	[AASS, ABCB7, ACTR2, ADAM10, ADD2, ADD3, AKT1, ANKFY1, ANO6, ANTXR1, ANXA1, ANXA2, AP2A1, AP2B1, AP2M1, AP2S1, APOE, APPL2, ARAP1, ARF4, ARF6, ARFGAP1, ARFGAP3, ARFGEF1, ARHGAP28, ARHGDIA, ARHGEF11, ARL2, ARL3, ARPC1A, ARSB, ASAP1, ATF7IP, ATL3, ATR, AURKA, AURKB, BAIAP2, BIN1, BRAT1, BRD4, BUB1B, CALCOCO2, CALR, CAMK1, CAMK2D, CAPN2, CAPRIN1, CAPZA2, CAV1, CBL, CCAR2, CCNB1, CCT2, CCT3, CCT4, CCT5, CCT6A, CCT7, CCT8, CD2AP, CD44, CDC42, CDC42EP1, CDC42EP3, CDC73, CDH2, CFL2, CHEK1, CKAP5, CLASP1, COL5A1, COLGALT1, CORO1C, CRLF3, CRMP1, CTCF, CTNNB1, CUL4A, CUL4B, CUL7, CYFIP1, DAXX, DBNL, DCTN1, DDX39B, DDX49, DHX33, DIAPH1, DLG1, DLGAP5, DNMT1, DNMT3B, DPYSL2, DPYSL3, DRG1, DSTN, DUSP3, ECT2, EFNB2, EGFR, EHD1, EHD2, EHD4, ENO2, EPB41L3, EPB41L5, EPHB3, EPS8, EPS8L2, ERCC2, EXOSC10, EXOSC2, F11R, FARP1, FBXW8, FERMT2, FHL1, FKBP4, FN1, FNBP1L, FSCN1, FSD1, FXR1, G3BP1, G3BP2, GCLC, GDI1, GLMN, GNL3, GPC3, GPC6, GSN, HCFC1, HDAC2, HDAC4, HSPA2, HSPA5, HTT, HUWE1, ILK, IQGAP1, IQGAP2, IQGAP3, IST1, ITCH, ITGA6, ITGAV, IWS1, JADE1, JAM3, JARID2, KAT7, KDM1A, KIF5B, KNTC1, LGALS1, LIMS1, LMAN1, LNPK, LRP1, MACF1, MACROH2A1, MAP1B, MAP1S, MAP4, MAPK1,	No change

					MAPK14, MAPK8, MARCHF5, MAVS, MCM2, MCM7, MCU, METAP1, MKI67, MMAB, MSN, MYADM, MYD88, MYH9, MYO1C, MYO1E, MYO5A, NCAPG, NCKAP1, NDC80, NDRG1, NEDD4, NEK7, NPM1, NRP2, OCLN, OGT, OSBP, P3H1, PAK1, PARN, PARP1, PAXBP1, PDGFRB, PDLIM5, PHIP, PHPT1, PICALM, PIK3CA, PIP4K2B, PIP4K2C, PLK1, PLSCR3, PLXNB2, POLR1B, PPM1F, PPP1R9B, PRKAA1, PRKAR1A, PRKCA, PRKCSH, PRPF38B, PRUNE1, PSMC6, PTK7, PTPN1, PTPRD, PXDN, PXMP2, PYCARD, RAB5B, RAB5C, RAC3, RAP2A, RCC2, RDX, RIC1, RIF1, ROCK2, RPA1, RPA2, RPA3, RPL13A, RPS6KA1, RPS6KA3, RPS6KA4, S100A10, SAR1A, SARM1, SART3, SCARB2, SCFD1, SEC22B, SEPTIN11, SEPTIN7, SEPTIN8, SESN2, SET, SETD7, SETDB1, SH3GL3, SH3GLB1, SIN3A, SIRT1, SIRT2, SKA3, SLAIN2, SLC25A5, SLC2A10, SLK, SMARCA4, SNX17, SNX3, SNX9, SORBS3, SPAG9, SPARC, SPTAN1, SPTBN1, SPTBN2, SRC, SSBP1, SSRP1, STX5, SUPT6H, SURF4, TACC3, TBC1D17, TBC1D22B, TBC1D4, TCP1, TENM3, TF, THBS1, TIGAR, TLK1, TMED9, TMEM97, TOP2A, TPM1, TPX2, TRIM28, TRIP12, TRIP13, TTK, TTLL12, TWF1, UBR5, USP47, USP7, VAT1, VIM, VLDLR, VPS35, WAPL, WARS1, XRCC5, XRN1, YAP1, ZFYVE16, ZMYND8]	
actin binding	GO_MolecularFunction-EBI-UniProt-GOA-ACAP-ARAP_13.05.2021_00h00	3.10E-35	18.97655	89	[ACTN1, ACTN4, ACTR2, ADD2, ADD3, ANTXR1, ANXA6, ARPC1A, BIN1, CALD1, CAP1, CAP2, CAPZA2, CFL2, CGN, CNN1, CNN3, COBLL1, CORO1C, CRMP1, CYFIP1, DBNL, DIAPH1, DSTN, EEF2, EGFR, EMD, EPB41, EPB41L3, EPS8, EPS8L2, FERMT2, FKBP15, FLNB, FLNC, FSCN1, GIPC1, GSN, IQGAP1, IQGAP2, IQGAP3, ITGB1, LASP1, LRPPRC, MACF1,	Downregulation

					MAP1B, MAP1S, METAP1, MICAL1, MICAL3, MPRIP, MSN, MYH10, MYH9, MYO1C, MYO1E, MYO5A, MYO9B, NCAPG, NOL6, P4HB, PALLD, PARVA, PAWR, PDLIM2, PDLIM4, PDLIM5, PDLIM7, PLEC, PLS3, PPP1R18, PPP1R9B, PXK, RAI14, RDX, SNTB2, SORBS1, SPTAN1, SPTBN1, SPTBN2, TAGLN, TLN1, TLN2, TNS1, TPM1, TPM4, TWF1, UTRN, ZNF185]	
cellular component disassembly	GO_BiologicalProcess-EBI-UniProt-GOA-ACAP-ARAP_13.05.2021_00h00	3.10E-35	12.89256	78	[ACIN1, ADAM10, ADD2, ADD3, APEH, ARF6, CALCOCO2, CAPN1, CAPN2, CAPNS1, CAPZA2, CASP3, CAV1, CCNB1, CD44, CDK5RAP3, CFL2, CKAP5, CLASP1, CTNNB1, DCTN1, DDRGK1, DHX8, DICER1, DSTN, DUSP3, EPS8, EXOG, FSCN1, GBF1, GLMN, GOLGA2, GSN, HK2, HSPA2, HTRA1, HTT, HUWE1, IST1, KIF2C, KIF5B, KLC1, LAMC1, LRP1, MAP1B, MAP1LC3A, MAP1S, METAP1, MICAL1, MICAL3, MRPS27, MRPS9, NCAPG, NSF, OPTN, PIK3CA, PLK1, PPP1R9B, PRKCA, PTCD3, RDX, SET, SLC25A5, SMARCA4, SMARCC1, SMARCD1, SMARCD2, SPTAN1, SPTBN1, SPTBN2, STX5, SUPT16H, TIGAR, TOP2A, TPX2, TWF1, UFL1, VRK1]	No change
regulation of cellular component size	GO_BiologicalProcess-EBI-UniProt-GOA-ACAP-ARAP_13.05.2021_00h00	3.10E-35	14.25061	58	[ACTR2, ADD2, ADD3, ANO6, AP2M1, APOE, ARF6, ARFGEF1, ARHGAP28, ARPC1A, BAIAP2, BIN1, CAPZA2, CDC42EP1, CDC42EP3, CFL2, CYFIP1, DBNL, DLG1, DPYSL2, DSTN, EPS8, FLNB, FN1, GDI1, GSN, HP1BP3, HSP90AB1, ILK, IQGAP2, IQGAP3, IST1, KDM1A, LARS1, LRP1, LRRC8A, MACF1, MAP1B, MSN, MYADM, MYO1C, MYO1E, NCAPG, NCKAP1, NPM1, NPM3, PAK1, PICALM, PIK3CA, PYCARD, RDX, SIN3A, SLC12A4, SNX9, SPTAN1, SPTBN1, SPTBN2, TWF1]	Downregulation
positive regulation of cellular	GO_BiologicalProcess-EBI-	3.10E-35	14.0625	81	[ACTR2, ANTXR1, ARAP1, ARF6, ARL2, ARL3, ARPC1A, ASAP1,	Downregulation

component biogenesis	UniProt-GOA-ACAP-ARAP_13.05.2021_00h00				ATF7IP, ATR, BAIAP2, BIN1, CAV1, CDC42, CDC42EP1, CDC42EP3, CKAP5, CLASP1, CUL4A, CUL4B, CYFIP1, DCTN1, DHX33, DLG1, DNMT1, DPYSL3, DRG1, EPB41L5, EPHB3, EPS8, EPS8L2, ERCC2, FERMT2, FNBP1L, FSCN1, G3BP1, G3BP2, GSN, HDAC4, HTT, IQGAP1, IQGAP2, LIMS1, MAP1B, MMAB, MSN, MYD88, MYO1C, MYO1E, NCAPG, NCKAP1, NDRG1, OCLN, PAK1, PARP1, PIK3CA, PIP4K2B, PIP4K2C, POLR1B, PPM1F, PRKCA, PSMC6, PTPRD, PXDN, PYCARD, ROCK2, RPA1, RPA2, RPA3, S100A10, SEPTIN7, SETDB1, SH3GLB1, SLAIN2, SNX9, SORBS3, SRC, TPM1, TRIM28, WARS1, ZMYND8]	
actin filament binding	GO_MolecularFunction-EBI-UniProt-GOA-ACAP-ARAP_13.05.2021_00h00	3.10E-35	20.88889	47	[ACTN1, ACTN4, ACTR2, ADD2, ADD3, ANTXR1, ANXA6, ARPC1A, BIN1, CAPZA2, CFL2, CORO1C, CRMP1, CYFIP1, DBNL, DSTN, EEF2, EGFR, FERMT2, FSCN1, GSN, IQGAP1, IQGAP2, IQGAP3, LASP1, LRPPRC, MACF1, MAP1S, MICAL1, MPRIP, MYH10, MYH9, MYO1C, MYO1E, MYO5A, NCAPG, NOL6, PLS3, PPP1R9B, TAGLN, TLN1, TLN2, TPM1, TPM4, TWF1, UTRN, ZNF185]	Downregulation
positive regulation of cellular component organization	GO_BiologicalProcess-EBI-UniProt-GOA-ACAP-ARAP_13.05.2021_00h00	3.10E-35	14.06629	174	[ABCB7, ACTR2, ANKFY1, ANO6, ANTXR1, ANXA1, ANXA2, AP2A1, AP2B1, AP2M1, APOE, APPL2, ARAP1, ARF6, ARHGDIA, ARL2, ARL3, ARPC1A, ARSB, ASAP1, ATF7IP, ATL3, ATR, AURKA, AURKB, BAIAP2, BIN1, BRD4, CALCOCO2, CAMK1, CAPN2, CAPRIN1, CAV1, CBL, CCNB1, CCT2, CCT3, CCT4, CCT5, CCT6A, CCT7, CCT8, CDC42, CDC42EP1, CDC42EP3, CFL2, CKAP5, CLASP1, COLGALT1, CORO1C, CTNNB1, CUL4A, CUL4B, CUL7, CYFIP1, DAXX, DBNL, DCTN1, DHX33, DLG1, DLGAP5, DNMT1, DNMT3B, DPYSL3, DRG1, DSTN, DUSP3, EHD1, EHD2, EPB41L5, EPHB3, EPS8, EPS8L2, ERCC2, FBXW8,	No change

					FERMT2, FN1, FNBP1L, FSCN1, G3BP1, G3BP2, GDI1, GNL3, GPC3, GSN, HDAC4, HSPA5, HTT, ILK, IQGAP1, IQGAP2, IST1, ITGA6, JARID2, KAT7, KDM1A, KIF5B, LIMS1, LMAN1, LNPK, LRP1, MACF1, MACROH2A1, MAP1B, MAPK1, MAPK14, MAPK8, MARCHF5, MCU, MMAB, MSN, MYD88, MYO1C, MYO1E, NCAPG, NCKAP1, NDRG1, NEK7, OCLN, OGT, OSBP, P3H1, PAK1, PARN, PARP1, PAXBP1, PDGFRB, PHIP, PIK3CA, PIP4K2B, PIP4K2C, PLXNB2, POLR1B, PPM1F, PSMC6, PTK7, PTPRD, PXDN, PYCARD, RIF1, ROCK2, RPA1, RPA2, RPA3, RPS6KA4, S100A10, SART3, SCARB2, SEPTIN7, SETDB1, SH3GLB1, SIN3A, SIRT1, SIRT2, SLAIN2, SLC25A5, SNX3, SNX9, SORBS3, SRC, SSBP1, SURF4, TCP1, TENM3, TF, TMED9, TPM1, TRIM28, TWF1, VLDLR, VPS35, WARS1, XRCC5, ZMYND8]	
supramolecular fiber organization	GO_BiologicalProcess-EBI-UniProt-GOA-ACAP-ARAP_13.05.2021_00h00	3.10E-35	14.41441	128	[ABI1, ACTN1, ACTR2, ADAM10, ADD2, ADD3, ANXA2, APOE, ARAP1, ARF6, ARFGEF1, ARHGAP28, ARL2, ARL3, ARPC1A, BAIAP2, BIN1, CALD1, CAPZA2, CD2AP, CDC42, CDC42EP1, CDC42EP3, CFL2, CKAP5, CLASP1, COL1A1, COL1A2, COL4A1, COL4A2, COL5A1, COL5A2, COL6A1, COL6A2, COL6A3, COLGALT1, CORO1C, CRTAP, CSRP2, CYFIP1, DBNL, DCTN1, DIAPH1, DLG1, DPYSL3, DRG1, DSP, DSTN, EPS8, F11R, FAT1, FERMT2, FKBP10, FKBP4, FSCN1, FSD1, GOLGA2, GSN, HOOK3, HSP90AB1, ILK, IQGAP2, ITGA6, ITGB1, KIF2C, KRT19, KRT8, MAP1B, MAP1S, MCU, METAP1, MFGE8, MICAL1, MICAL3, MPRIP, MYADM, MYO1C, MYO1E, MYO5A, NCAPG, NCKAP1, NDRG1, NOL6, OCLN, P3H1, P3H4, P4HA1, P4HA2, P4HB, PAK1, PAWR, PDGFRB, PIK3CA, PKP2, PLEC, PLOD1, PLOD2, PLOD3,	Downregulation

					PLS3, PPIB, PPM1F, PPP1R9B, PRKAR1A, PRUNE1, PXDN, PYCARD, RAC3, RAP1GDS1, RDX, ROCK2, S100A10, SERPINH1, SLAIN2, SLK, SNX9, SORBS1, SORBS3, SPTAN1, SPTBN1, SPTBN2, SRC, TF, TPM1, TPM4, TPX2, TWF1, USP9X, VIM]	
supramolecular fiber	GO_CellularComponent-EBI-UniProt-GOA-ACAP-ARAP_13.05.2021_00h00	3.10E-35	13.42413	138	[ACTBL2, ACTN1, AK1, ANK2, ANK3, ANXA1, ARL3, ATP2B4, AURKA, AURKB, BAG2, BAG3, BAIAP2, BIN1, CALD1, CCT2, CCT3, CCT4, CCT5, CCT6A, CCT7, CCT8, CD2AP, CDK5RAP3, CEP170, CFAP20, CFL2, CKAP5, CLASP1, COL1A1, COL1A2, COL5A1, COL5A2, COL6A1, COL6A3, CORO1C, CSRP2, CTNNB1, DBNL, DCTN1, DCTN4, DCXR, DDX6, DLG1, DPYSL2, DPYSL3, DSP, DYNC1LI2, EIF3A, EMD, EML1, FBN2, FERMT2, FKBP4, FLNB, FLNC, FSD1, FXR1, GNG12, GOLGA2, HDAC4, HOOK3, HSPB1, HSPH1, ILK, INCENP, IQGAP1, JAM3, KEAP1, KIF11, KIF20A, KIF21A, KIF22, KIF23, KIF2C, KIF5B, KLC1, KNTC1, KRT18, KRT19, KRT8, LMAN1, LRPPRC, LTBP1, MACF1, MAP1B, MAP1LC3A, MAP1S, MAP4, METAP1, MICAL1, MYH10, MYH9, MYL9, MYO1C, MYO1E, MYO5A, MYO9B, NCKAP1, NDRG1, NEK7, NOL6, NUSAP1, PAK1, PALLD, PARVA, PAWR, PDLIM2, PDLIM4, PDLIM5, PDLIM7, PKP2, PLEC, PLK1, PLS3, PPP2R5A, RCC2, SARM1, SIRT2, SKA3, SLAIN2, SLC2A1, SNTB2, SPECC1L, SPTBN1, SRC, SRPRB, STIM1, STXBP2, TCP1, TMEM214, TPM1, TPM4, TPX2, TUBA4A, TUBB6, TWF1, VIM]	Downregulation
microtubule organizing center	GO_CellularComponent-EBI-UniProt-GOA-ACAP-ARAP_13.05.2021_00h00	3.10E-35	11.76471	98	[AAAS, ACTR1B, AKT1, ARL2, ARL3, AURKA, AURKB, BCCIP, BIRC6, BUB1B, CCNB1, CCT4, CCT5, CCT8, CD2AP, CDC42, CDK5RAP3, CEP170, CFAP20, CHD4, CHEK1, CKAP5, CLASP1, CRMP1, CTNNB1, CTNNBL1, CTSC, CUL7, DCAF13, DCTN1, DCTN4, DHX9, DIAPH1,	No change

					DLGAP5, DYNC1LI2, ECPAS, EMD, FSD1, HERC2, HK2, HMMR, HOOK3, HSPB11, HTT, IST1, JADE1, KEAP1, KIF2C, KIF5B, KRT18, LRWD1, MAPK1, MASTL, MCM3, MVB12A, NCAPG, NDC80, NDRG1, NEK7, NIT2, NPM1, NSFL1C, ORC2, PHIP, PLEKHA7, PLK1, PPP2R5A, PRKACB, PRKAR1A, PRKAR2A, PRKAR2B, PRKCA, PXK, RAB23, RAB8A, RABL6, RAD51, RAN, ROCK2, RP2, RTTN, SCYL1, SIRT2, SKA3, SLAIN2, SORBS1, SPAG9, SPECC1L, SPOUT1, TACC3, TCP1, TOP2A, TTC28, TTLL12, UBR4, UPF3B, VIM, WAPL]	
actin cytoskeleton organization	GO_BiologicalProcess-EBI-UniProt-GOA-ACAP-ARAP_13.05.2021_00h00	3.10E-35	15.89674	117	[ABI1, ACTBL2, ACTN1, ACTR2, ADD2, ADD3, ANTXR1, ANXA1, ARAP1, ARF6, ARFGEF1, ARHGAP28, ARHGDIA, ARHGEF11, ARPC1A, BAIAP2, BIN1, CALD1, CALR, CAP1, CAPZA2, CD2AP, CDC42, CDC42BPA, CDC42BPB, CDC42EP1, CDC42EP3, CFL2, CLASP1, CNN1, CNN3, CORO1C, CSRP2, CXADR, CYFIP1, DBNL, DIAPH1, DLG1, DPYSL3, DSTN, ECT2, EHD2, EPB41, EPB41L3, EPB41L5, EPS8, F11R, FARP1, FAT1, FERMT2, FLNB, FSCN1, GAB1, GSN, ILK, IQGAP1, IQGAP2, IQGAP3, ITGB1, JAM3, KRT19, KRT8, LRP1, MCU, MICAL1, MICAL3, MPRIP, MYADM, MYH10, MYH9, MYO1C, MYO1E, MYO5A, NCAPG, NCKAP1, NOL6, PAK1, PALLD, PARVA, PAWR, PDCL3, PDGFRB, PDLIM2, PDLIM4, PDLIM5, PDLIM7, PHPT1, PIK3CA, PLS3, PPM1F, PPP1R9B, PRKAR1A, PTK7, PTPN1, PXDN, PYCARD, RAC3, RAP1GDS1, RAP2A, RDX, ROCK2, RTKN, S100A10, SDAD1, SNX9, SORBS1, SORBS3, SPECC1L, SPTAN1, SPTBN1, SPTBN2, SRC, TF, TLN1, TPM1, TPM4, TWF1]	Downregulation
actin filament bundle	GO_CellularComponent-EBI-UniProt-GOA-	3.10E-35	29.26829	24	[ACTN1, BAG3, DCTN4, FERMT2, FLNB, FSCN1, ILK, LPP, MYH10, MYH9, MYL9, MYO1C, MYO1E, PALLD, PDLIM2,	Downregulation

	ACAP-ARAP_13.05.2021_00h00				PDLIM4, PDLIM5, PDLIM7, PLS3, PXDN, SEPTIN11, SEPTIN7, SORBS1, TPM1]	
regulation of organelle organization	GO_BiologicalProcess-EBI-UniProt-GOA-ACAP-ARAP_13.05.2021_00h00	3.10E-35	14.20932	186	[ACTR2, ADD2, ADD3, AKT1, ANTXR1, ANXA1, ANXA2, ARAP1, ARF6, ARFGEF1, ARHGAP28, ARHGDIA, ARL2, ARL3, ARPC1A, ASAP1, ATF7IP, ATL3, ATR, AURKA, AURKB, BAIAP2, BIN1, BRD4, BUB1B, CALCOCO2, CALR, CAPN2, CAPZA2, CCNB1, CCT2, CCT3, CCT4, CCT5, CCT6A, CCT7, CCT8, CD2AP, CDC42, CDC42EP1, CDC42EP3, CDH2, CFL2, CHEK1, CKAP5, CLASP1, CTCF, CTNNB1, CUL7, CYFIP1, DAXX, DBNL, DCTN1, DIAPH1, DLG1, DLGAP5, DNMT1, DNMT3B, DRG1, DSTN, ECT2, ENO2, EPS8, EXOSC10, F11R, FERMT2, FKBP4, FSCN1, FSD1, G3BP1, G3BP2, GCLC, GNL3, GSN, HTT, HUWE1, ILK, IQGAP1, IQGAP2, IQGAP3, JAM3, JARID2, KAT7, KIF5B, KNTC1, LMAN1, LNPK, LRP1, MACROH2A1, MAP1B, MAP1S, MAP4, MAPK1, MAPK8, MARCHF5, MAVS, MCM2, MCM7, MCU, METAP1, MKI67, MMAB, MSN, MYADM, MYO1C, MYO1E, MYO5A, NCAPG, NCKAP1, NDC80, NDRG1, NEK7, NPM1, OCLN, OGT, OSBP, PAK1, PARN, PARP1, PAXBP1, PDGFRB, PHIP, PHPT1, PIK3CA, PIP4K2B, PIP4K2C, PLK1, PLSCR3, PPM1F, PRKAA1, PRKAR1A, PRUNE1, PTPRD, PXDN, PYCARD, RAC3, RDX, RIF1, ROCK2, RPS6KA4, S100A10, SAR1A, SART3, SCARB2, SCFD1, SEC22B, SEPTIN7, SETDB1, SH3GLB1, SIN3A, SIRT1, SIRT2, SKA3, SLAIN2, SLC25A5, SNX9, SORBS3, SPTAN1, SPTBN1, SPTBN2, SRC, SSBP1, STX5, SURF4, TACC3, TBC1D17, TBC1D22B, TBC1D4, TCP1, TIGAR, TMED9, TOP2A, TPM1, TPX2, TRIM28, TRIP13, TTK, TWF1, USP7, VAT1, VPS35, WAPL, XRCC5, XRN1, YAP1, ZMYND8]	No change
actomyosin	GO_CellularCompone	3.10E-35	31.3253	26	[ACTN1, BAG3, CDC42BPA, CDC42BPB,	Downregulation

	nt-EBI-UniProt-GOA-ACAP-ARAP_13.05.2021_00h00				DCTN4, FERMT2, FLNB, FSCN1, HDAC4, ILK, LPP, MYH10, MYH9, MYL9, MYO1C, MYO1E, PALLD, PDLIM2, PDLIM4, PDLIM5, PDLIM7, PXDN, SEPTIN11, SEPTIN7, SORBS1, TPM1]	
regulation of supramolecular fiber organization	GO_BiologicalProcess-EBI-UniProt-GOA-ACAP-ARAP_13.05.2021_00h00	3.10E-35	15.21197	61	[ACTR2, ADD2, ADD3, APOE, ARAP1, ARF6, ARFGEF1, ARHGAP28, ARL2, ARL3, ARPC1A, BAIAP2, BIN1, CAPZA2, CDC42, CDC42EP1, CDC42EP3, CFL2, CKAP5, CLASP1, COLGALT1, CYFIP1, DBNL, DCTN1, DLG1, DRG1, DSTN, EPS8, F11R, FERMT2, FKBP4, GSN, IQGAP2, MAP1B, MAP1S, METAP1, MYADM, MYO1C, MYO1E, NCAPG, NCKAP1, NDRG1, OCLN, PAK1, PIK3CA, PPM1F, PRUNE1, PXDN, PYCARD, RDX, ROCK2, S100A10, SLAIN2, SNX9, SORBS3, SPTAN1, SPTBN1, SPTBN2, TPM1, TPX2, TWF1]	Downregulation
cytoskeleton	GO_CellularComponent-EBI-UniProt-GOA-ACAP-ARAP_13.05.2021_00h00	3.10E-35	12.25248	297	[AAAS, ABI1, ACACA, ACOT13, ACTBL2, ACTN1, ACTN4, ACTR10, ACTR1B, ACTR2, ADD2, ADD3, AK1, AKT1, ALDOC, ALG2, ANK2, ANK3, ANXA1, ANXA11, ARAP3, ARHGDIA, ARL2, ARL3, ARL6IP5, ARPC1A, ASAP1, AURKA, AURKB, BAG2, BAG3, BAIAP2, BCCIP, BIN1, BIRC6, BUB1B, CALCOCO2, CALD1, CAP1, CAPN2, CAPZA2, CASK, CCAR2, CCNB1, CCT2, CCT3, CCT4, CCT5, CCT6A, CCT7, CCT8, CD2AP, CDC42, CDC42BPA, CDC42BPB, CDC42EP1, CDC42EP3, CDCA8, CDH2, CDK5RAP3, CEP170, CFAP20, CFAP298, CFL2, CGN, CHD4, CHEK1, CKAP4, CKAP5, CLASP1, CNN3, CORO1C, CRMP1, CTNNB1, CTNNBL1, CTSC, CUL7, CYFIP1, DAPK1, DBNL, DCAF13, DCTN1, DCTN4, DCXR, DDX20, DDX6, DHX9, DIAPH1, DLG1, DLGAP5, DPYSL2, DPYSL3, DSP, DSTN, DYNC1LI2, ECPAS, ECT2, EHD2, EIF3A, EMD, EML1, EPB41, EPB41L3, EPB41L5, ERCC2, EXD2, FARP1, FERMT2,	Downregulation

					FKBP15, FKBP4, FLNB, FLNC, FLT1, FNBP1L, FSCN1, FSD1, GNG12, GOLGA2, GPHN, GSN, HDAC4, HERC2, HK2, HMMR, HOOK3, HSPA2, HSPB1, HSPB11, HSPH1, HTT, ILF3, ILK, INCENP, IQGAP1, IQGAP2, IST1, JADE1, JAM3, KEAP1, KIF11, KIF20A, KIF21A, KIF22, KIF23, KIF2C, KIF5B, KLC1, KNTC1, KRT18, KRT19, KRT8, LANCL2, LASP1, LPP, LRP1, LRPPRC, LRWD1, MACF1, MAP1B, MAP1LC3A, MAP1S, MAP2K6, MAP4, MAPK1, MAPK14, MASTL, MCM3, MDN1, METAP1, MICAL1, MICAL3, MPRIP, MSN, MVB12A, MYADM, MYH10, MYH9, MYL9, MYO1C, MYO1E, MYO5A, MYO9B, NCAPG, NCKAP1, NDC1, NDC80, NDRG1, NEK7, NIT2, NOL9, NPM1, NPM3, NSFL1C, NSUN2, NUSAP1, ORC2, P4HB, PAK1, PALLD, PARD3, PARVA, PAWR, PDLIM2, PDLIM4, PDLIM5, PDLIM7, PEA15, PHIP, PIK3R4, PKP2, PLEC, PLEKHA7, PLK1, PLS3, PPP1R18, PPP1R9B, PPP2R5A, PREPL, PRKACB, PRKAR1A, PRKAR2A, PRKAR2B, PRKCA, PTPN12, PXDN, PXK, PYCR3, RAB23, RAB8A, RABL6, RAC3, RAD51, RAI14, RAN, RANGAP1, RCC2, RDX, RIF1, ROCK2, RP2, RTKN, RTTN, SAP30BP, SARM1, SBDS, SCYL1, SEPTIN10, SEPTIN11, SEPTIN2, SEPTIN7, SEPTIN8, SESTD1, SIRT2, SKA3, SLAIN2, SLC25A5, SLC2A1, SNTB2, SNX4, SNX9, SORBS1, SORBS3, SPAG9, SPECC1L, SPOUT1, SPTAN1, SPTBN1, SPTBN2, SRC, SRPRB, STIM1, SYMPK, TACC3, TBCEL, TCP1, TGFB1I1, TLN1, TLN2, TMEM214, TNS1, TOP2A, TPM1, TPM4, TPX2, TTC28, TTK, TTLL12, TUBA4A, TUBB6, TWF1, UBR4, UPF3B, UTRN, VIM, VRK1, WAPL, ZNF185]	
cytoskeleton organization	GO_BiologicalProcess-EBI-UniProt-GOA-	3.10E-35	13.67742	212	[AAAS, ABI1, ACTBL2, ACTN1, ACTR2, ADD2, ADD3, ANK2, ANK3, ANTXR1, ANXA1, APOE, ARAP1, ARAP3, ARF6,	Downregulation

	ACAP-ARAP_13.05.2021_00h00				ARFGEF1, ARHGAP28, ARHGDIA, ARHGEF11, ARL2, ARL3, ARPC1A, AURKA, AURKB, BAIAP2, BCCIP, BIN1, BRWD1, BUB1B, CALD1, CALR, CAP1, CAP2, CAPN2, CAPZA2, CCNB1, CD2AP, CDC42, CDC42BPA, CDC42BPB, CDC42EP1, CDC42EP3, CDCA8, CFL2, CHEK1, CHEK2, CKAP5, CLASP1, CNN1, CNN3, CORO1C, CRMP1, CSRP2, CTNNB1, CUL7, CXADR, CYFIP1, DBNL, DCTN1, DIAPH1, DLG1, DLGAP5, DOCK7, DPYSL2, DPYSL3, DRG1, DSP, DSTN, DYNC1LI2, ECT2, EHD2, EML1, EPB41, EPB41L3, EPB41L5, EPS8, ERBIN, ERCC6L, F11R, FARP1, FAT1, FERMT2, FKBP4, FLNB, FSCN1, FSD1, GAB1, GOLGA2, GSN, HOOK3, HTT, ILK, INCENP, IQGAP1, IQGAP2, IQGAP3, ITGB1, JAM3, KIF11, KIF20A, KIF23, KIF2C, KNTC1, KRT18, KRT19, KRT8, LRP1, MACF1, MAP1B, MAP1S, MAP4, MAPK1, MCU, METAP1, MICAL1, MICAL3, MLH1, MPRIP, MSN, MYADM, MYH10, MYH9, MYO1C, MYO1E, MYO5A, NCAPG, NCKAP1, NDC80, NDRG1, NECTIN2, NEK7, NOL6, NPM1, NSFL1C, NUP107, NUP133, NUP160, NUSAP1, OCLN, PAK1, PALLD, PARD3, PARD6B, PARVA, PAWR, PDCL3, PDGFRB, PDLIM2, PDLIM4, PDLIM5, PDLIM7, PHIP, PHPT1, PIK3CA, PKP2, PLEC, PLK1, PLS3, PPM1F, PPP1R9B, PRKAA1, PRKAR1A, PRPF40A, PRUNE1, PTK7, PTPN1, PXDN, PYCARD, RAC3, RAN, RANBP2, RANGAP1, RAP1GDS1, RAP2A, RCC2, RDX, ROCK2, RTKN, RTTN, S100A10, SBDS, SDAD1, SEC13, SEH1L, SIRT1, SKA3, SLAIN2, SLK, SNX9, SORBS1, SORBS3, SPECC1L, SPTAN1, SPTBN1, SPTBN2, SRC, SUN2, TACC3, TBCEL, TF, TLN1, TLN2, TPM1, TPM4, TPX2, TTK, TUBA4A, TUBB6, TWF1, VIM, ZMYM3, ZWILCH]	

positive regulation of organelle organization	GO_BiologicalProcess-EBI-UniProt-GOA-ACAP-ARAP_13.05.2021_00h00	3.10E-35	16.08498	106	[ACTR2, ANTXR1, ANXA1, ANXA2, ARF6, ARL2, ARL3, ARPC1A, ASAP1, ATF7IP, ATL3, ATR, AURKA, AURKB, BAIAP2, BIN1, BRD4, CALCOCO2, CCNB1, CCT2, CCT3, CCT4, CCT5, CCT6A, CCT7, CCT8, CDC42, CDC42EP1, CDC42EP3, CFL2, CKAP5, CLASP1, CTNNB1, CYFIP1, DAXX, DCTN1, DLG1, DLGAP5, DNMT1, DNMT3B, DRG1, DSTN, FERMT2, FSCN1, G3BP1, G3BP2, GNL3, GSN, HTT, IQGAP2, JARID2, KAT7, KIF5B, LMAN1, LNPK, MACROH2A1, MAP1B, MAPK1, MAPK8, MARCHF5, MCU, MMAB, MSN, MYO1C, MYO1E, NCAPG, NCKAP1, NDRG1, NEK7, OCLN, OGT, OSBP, PAK1, PARN, PAXBP1, PDGFRB, PHIP, PIP4K2B, PIP4K2C, PPM1F, PXDN, PYCARD, RIF1, ROCK2, RPS6KA4, S100A10, SART3, SEPTIN7, SETDB1, SH3GLB1, SIN3A, SIRT1, SIRT2, SLAIN2, SLC25A5, SNX9, SORBS3, SRC, SSBP1, SURF4, TCP1, TMED9, TPM1, TRIM28, VPS35, XRCC5]	No change
myofibril	GO_CellularComponent-EBI-UniProt-GOA-ACAP-ARAP_13.05.2021_00h00	3.10E-35	16.1157	39	[ACTN1, ANK2, ANK3, ATP2B4, BAG3, BIN1, CALD1, CFL2, CORO1C, CSRP2, CTNNB1, DCTN4, FERMT2, FLNB, FLNC, FXR1, HDAC4, HSPB1, ILK, KRT19, KRT8, LMAN1, MYL9, NOL6, PAK1, PALLD, PARVA, PDLIM2, PDLIM4, PDLIM5, PDLIM7, PLEC, PPP2R5A, SLC2A1, SPTBN1, STXBP2, TPM1, TPM4, TWF1]	Downregulation
positive regulation of supramolecular fiber organization	GO_BiologicalProcess-EBI-UniProt-GOA-ACAP-ARAP_13.05.2021_00h00	3.10E-35	17.48879	39	[ACTR2, APOE, ARF6, ARL2, ARL3, ARPC1A, BAIAP2, BIN1, CDC42, CDC42EP1, CDC42EP3, CFL2, CKAP5, CLASP1, COLGALT1, CYFIP1, DCTN1, DLG1, DRG1, DSTN, FERMT2, GSN, IQGAP2, MAP1B, MYO1C, MYO1E, NCKAP1, NDRG1, OCLN, PAK1, PPM1F, PXDN, PYCARD, ROCK2, S100A10, SLAIN2, SNX9, SORBS3, TPM1]	Downregulation
I band	GO_CellularComponent-EBI-UniProt-GOA-	3.10E-35	18.24324	27	[ACTN1, ANK2, ANK3, ATP2B4, BAG3, BIN1, CFL2, CSRP2, CTNNB1, FERMT2, FLNB, FLNC, HSPB1, KRT19, KRT8,	Downregulation

	ACAP-ARAP_13.05.2021_00h00				MYL9, NOL6, PAK1, PALLD, PARVA, PDLIM2, PDLIM4, PDLIM5, PDLIM7, PPP2R5A, SLC2A1, STXBP2]	
actin filament organization	GO_BiologicalProcess-EBI-UniProt-GOA-ACAP-ARAP_13.05.2021_00h00	3.10E-35	14.96746	69	[ABI1, ACTN1, ACTR2, ADD2, ADD3, ARAP1, ARF6, ARFGEF1, ARHGAP28, ARPC1A, BAIAP2, BIN1, CALD1, CAPZA2, CD2AP, CDC42, CDC42EP1, CDC42EP3, CFL2, CLASP1, CORO1C, CYFIP1, DBNL, DIAPH1, DLG1, DPYSL3, DSTN, EPS8, F11R, FAT1, FERMT2, FSCN1, GSN, IQGAP2, ITGB1, MCU, MICAL1, MICAL3, MPRIP, MYADM, MYO1C, MYO1E, MYO5A, NCAPG, NCKAP1, NOL6, PAK1, PAWR, PIK3CA, PLS3, PPM1F, PPP1R9B, PXDN, PYCARD, RAC3, RDX, ROCK2, S100A10, SNX9, SORBS1, SORBS3, SPTAN1, SPTBN1, SPTBN2, SRC, TF, TPM1, TPM4, TWF1]	Downregulation
actomyosin structure organization	GO_BiologicalProcess-EBI-UniProt-GOA-ACAP-ARAP_13.05.2021_00h00	3.10E-35	16.83168	34	[ARAP1, ARHGAP28, CDC42, CDC42BPA, CDC42BPB, CFL2, CLASP1, CNN1, CNN3, CSRP2, ECT2, EPB41, EPB41L3, EPB41L5, F11R, FERMT2, ITGB1, KRT19, KRT8, MYH10, MYH9, NOL6, PAK1, PDGFRB, PPM1F, PRKAR1A, PXDN, ROCK2, RTKN, S100A10, SORBS1, SORBS3, SRC, TPM1]	Downregulation
positive regulation of protein-containing complex assembly	GO_BiologicalProcess-EBI-UniProt-GOA-ACAP-ARAP_13.05.2021_00h00	3.10E-35	17.25352	49	[ACTR2, ANTXR1, ARF6, ARL2, ARL3, ARPC1A, ASAP1, ATR, BAIAP2, BIN1, CDC42EP1, CDC42EP3, CKAP5, CLASP1, CUL4A, CUL4B, CYFIP1, DCTN1, DHX33, DLG1, DRG1, ERCC2, FERMT2, FSCN1, GSN, IQGAP2, MAP1B, MMAB, MSN, MYD88, MYO1C, MYO1E, NCAPG, NCKAP1, NDRG1, OCLN, PAK1, PARP1, POLR1B, PSMC6, PYCARD, RPA1, RPA2, RPA3, SH3GLB1, SLAIN2, SNX9, SRC, WARS1]	No change
regulation of actin cytoskeleton organization	GO_BiologicalProcess-EBI-UniProt-GOA-ACAP-ARAP_13.05.2021_00h00	3.10E-35	15.07937	57	[ACTR2, ADD2, ADD3, ARAP1, ARF6, ARFGEF1, ARHGAP28, ARHGDIA, ARPC1A, BAIAP2, BIN1, CAPZA2, CD2AP, CDC42, CDC42EP1, CDC42EP3, CFL2, CLASP1, CYFIP1, DBNL, DLG1, DSTN, ECT2, EPS8, F11R, FERMT2,	Downregulation

					FSCN1, GSN, ILK, IQGAP1, IQGAP2, IQGAP3, JAM3, LRP1, MYADM, MYO1C, MYO1E, NCAPG, NCKAP1, PAK1, PDGFRB, PHPT1, PIK3CA, PPM1F, PXDN, PYCARD, RAC3, RDX, ROCK2, S100A10, SNX9, SORBS3, SPTAN1, SPTBN1, SPTBN2, TPM1, TWF1]	
regulation of cytoskeleton organization	GO_BiologicalProcess-EBI-UniProt-GOA-ACAP-ARAP_13.05.2021_00h00	3.10E-35	14.38849	80	[ACTR2, ADD2, ADD3, ARAP1, ARF6, ARFGEF1, ARHGAP28, ARHGDIA, ARL2, ARL3, ARPC1A, BAIAP2, BIN1, CAPN2, CAPZA2, CD2AP, CDC42, CDC42EP1, CDC42EP3, CFL2, CKAP5, CLASP1, CYFIP1, DBNL, DCTN1, DIAPH1, DLG1, DRG1, DSTN, ECT2, EPS8, F11R, FERMT2, FKBP4, FSCN1, FSD1, GSN, ILK, IQGAP1, IQGAP2, IQGAP3, JAM3, LRP1, MAP1B, MAP1S, MAPK1, METAP1, MYADM, MYO1C, MYO1E, NCAPG, NCKAP1, NDRG1, NPM1, OCLN, PAK1, PDGFRB, PHPT1, PIK3CA, PLK1, PPM1F, PRKAA1, PRUNE1, PXDN, PYCARD, RAC3, RDX, ROCK2, S100A10, SKA3, SLAIN2, SNX9, SORBS3, SPTAN1, SPTBN1, SPTBN2, TACC3, TPM1, TPX2, TWF1]	Downregulation
stress fiber	GO_CellularComponent-EBI-UniProt-GOA-ACAP-ARAP_13.05.2021_00h00	3.10E-35	31.08108	23	[ACTN1, BAG3, DCTN4, FERMT2, FLNB, FSCN1, ILK, LPP, MYH10, MYH9, MYL9, MYO1C, MYO1E, PALLD, PDLIM2, PDLIM4, PDLIM5, PDLIM7, PXDN, SEPTIN11, SEPTIN7, SORBS1, TPM1]	Downregulation
actin polymerization or depolymerization	GO_BiologicalProcess-EBI-UniProt-GOA-ACAP-ARAP_13.05.2021_00h00	3.10E-35	16.37931	38	[ABI1, ACTR2, ADD2, ADD3, ARF6, ARFGEF1, ARHGAP28, ARPC1A, BAIAP2, BIN1, CAPZA2, CDC42EP1, CDC42EP3, CFL2, CYFIP1, DBNL, DIAPH1, DLG1, DSTN, EPS8, GSN, IQGAP2, MICAL1, MICAL3, MYADM, MYO1C, MYO1E, NCAPG, NCKAP1, PIK3CA, PPP1R9B, PYCARD, RDX, SNX9, SPTAN1, SPTBN1, SPTBN2, TWF1]	Downregulation
microtubule	GO_CellularComponent-EBI-UniProt-GOA-ACAP-ARAP_13.0	3.10E-35	14.73923	65	[ARL3, AURKA, AURKB, BAG2, BAIAP2, CCT2, CCT3, CCT4, CCT5, CCT6A, CCT7, CCT8, CDK5RAP3, CEP170, CFAP20, CKAP5, CLASP1, DCTN1, DCXR,	No change

	5.2021_00 h00				DLG1, DPYSL2, DYNC1LI2, EIF3A, EMD, EML1, FKBP4, FSD1, GOLGA2, HOOK3, HSPH1, INCENP, IQGAP1, KIF11, KIF20A, KIF21A, KIF22, KIF23, KIF2C, KIF5B, KLC1, KNTC1, LRPPRC, MACF1, MAP1B, MAP1LC3A, MAP1S, MAP4, METAP1, NDRG1, NEK7, NUSAP1, PLK1, RCC2, SARM1, SIRT2, SKA3, SLAIN2, SNTB2, SRPRB, STIM1, TCP1, TMEM214, TPX2, TUBA4A, TUBB6]	
regulation of actin filament organization	GO_BiologicalProcess-EBI-UniProt-GOA-ACAP-ARAP_13.05.2021_00h00	3.10E-35	15.46392	45	[ACTR2, ADD2, ADD3, ARAP1, ARF6, ARFGEF1, ARHGAP28, ARPC1A, BAIAP2, BIN1, CAPZA2, CDC42, CDC42EP1, CDC42EP3, CFL2, CLASP1, CYFIP1, DBNL, DLG1, DSTN, EPS8, F11R, FERMT2, GSN, IQGAP2, MYADM, MYO1C, MYO1E, NCAPG, NCKAP1, PAK1, PIK3CA, PPM1F, PXDN, PYCARD, RDX, ROCK2, S100A10, SNX9, SORBS3, SPTAN1, SPTBN1, SPTBN2, TPM1, TWF1]	Downregulation
protein depolymerization	GO_BiologicalProcess-EBI-UniProt-GOA-ACAP-ARAP_13.05.2021_00h00	3.10E-35	19.35484	24	[ADD2, ADD3, CAPZA2, CFL2, CKAP5, CLASP1, DSTN, EPS8, GSN, KIF2C, MAP1B, MAP1S, METAP1, MICAL1, MICAL3, NCAPG, PIK3CA, PPP1R9B, RDX, SPTAN1, SPTBN1, SPTBN2, TPX2, TWF1]	Downregulation
regulation of actin polymerization or depolymerization	GO_BiologicalProcess-EBI-UniProt-GOA-ACAP-ARAP_13.05.2021_00h00	3.10E-35	16.58291	33	[ACTR2, ADD2, ADD3, ARF6, ARFGEF1, ARHGAP28, ARPC1A, BAIAP2, BIN1, CAPZA2, CDC42EP1, CDC42EP3, CFL2, CYFIP1, DBNL, DLG1, DSTN, EPS8, GSN, IQGAP2, MYADM, MYO1C, MYO1E, NCAPG, NCKAP1, PIK3CA, PYCARD, RDX, SNX9, SPTAN1, SPTBN1, SPTBN2, TWF1]	Downregulation
actin filament depolymerization	GO_BiologicalProcess-EBI-UniProt-GOA-ACAP-ARAP_13.05.2021_00h00	3.10E-35	26.5625	17	[ADD2, ADD3, CAPZA2, CFL2, DSTN, EPS8, GSN, MICAL1, MICAL3, NCAPG, PIK3CA, PPP1R9B, RDX, SPTAN1, SPTBN1, SPTBN2, TWF1]	Downregulation
positive regulation of biological process	GO_BiologicalProcess-EBI-UniProt-GOA-ACAP-ARAP_13.0	1.49E-64	9.443173	619	[AATF, ABAT, ABCB10, ABCB7, ABCC1, ABHD14B, ABI1, ACACA, ACIN1, ACLY, ACSL1, ACSL4, ACTN1, ACTN4, ACTR2, ADAM10, ADAR, ADARB1, AGO1, AGO2, AGTPBP1, AIMP2, AKT1,	No change

	5.2021_00h00				ANK2, ANK3, ANKFY1, ANO6, ANTXR1, ANXA1, ANXA2, ANXA3, AP2A1, AP2B1, AP2M1, AP3B1, APAF1, APOE, APPL2, ARAP1, ARF4, ARF6, ARFGEF1, ARHGDIA, ARHGEF1, ARHGEF11, ARHGEF12, ARL2, ARL3, ARL6IP5, ARPC1A, ARSB, ASAP1, ASNS, ASPH, ATF7IP, ATL3, ATP1A1, ATP2B1, ATP2B4, ATR, AURKA, AURKB, B4GALT1, BAG2, BAG3, BAIAP2, BAZ1B, BIN1, BIRC6, BPTF, BRAT1, BRD4, BRMS1, C1QBP, CACYBP, CALCOCO2, CALR, CAMK1, CAMK2D, CAPN1, CAPN2, CAPNS1, CAPRIN1, CASK, CASP3, CAT, CAV1, CAVIN1, CBL, CBR1, CCAR1, CCAR2, CCDC22, CCNB1, CCND2, CCNY, CCT2, CCT3, CCT4, CCT5, CCT6A, CCT7, CCT8, CD2AP, CD44, CD81, CDC123, CDC42, CDC42EP1, CDC42EP3, CDC73, CDH2, CDH3, CDK5RAP1, CDK5RAP3, CDK7, CEBPZ, CELF1, CFAP20, CFL2, CHEK1, CHEK2, CHTF18, CIRBP, CKAP5, CLASP1, CNOT11, CNTFR, COL1A1, COLGALT1, COPS5, CORO1C, CPNE2, CRLF3, CSPG4, CTBP2, CTCF, CTNNB1, CTNNBL1, CTSC, CUL4A, CUL4B, CUL7, CYFIP1, DAGLB, DAPK1, DAXX, DAZAP1, DBNL, DCTN1, DCXR, DDRGK1, DDX20, DDX21, DDX39B, DDX41, DDX49, DDX5, DDX6, DECR1, DHPS, DHX15, DHX33, DHX37, DHX8, DHX9, DIAPH1, DICER1, DIPK2A, DLG1, DLGAP5, DNMT1, DNMT3A, DNMT3B, DOCK7, DPYSL2, DPYSL3, DRG1, DSTN, DUSP3, ECE1, ECT2, EDRF1, EEF1E1, EEF2, EFNB2, EGFL6, EGFR, EHD1, EHD2, EHD4, EHMT1, EIF4A3, ELP3, EMD, EPB41, EPB41L5, EPCAM, EPHB3, EPM2AIP1, EPN2, EPS8, EPS8L2, ERAP1, ERBIN, ERCC2, ETFA, EXOSC2, EXOSC5, EXOSC7, F11R, FASTKD2, FBN2, FBXW8, FERMT2, FGG, FH, FHL1, FKBP8, FLNB, FLT1, FN1, FNBP1L, FOXK1, FSCN1, FXR1,	

					G3BP1, G3BP2, GAB1, GARS1, GATA6, GCLC, GDI1, GIPC1, GLMN, GLUD1, GLUL, GNL3, GOLGA2, GOLT1B, GPC1, GPC3, GPD1L, GPI, GPSM1, GSDME, GSN, GSTM2, GTF2I, GTPBP1, GUF1, HADHA, HCFC1, HDAC2, HDAC4, HEATR1, HK1, HK2, HLA-A, HMOX1, HNRNPLL, HSD17B4, HSP90AB1, HSPA2, HSPA5, HSPB1, HSPD1, HSPE1, HSPH1, HTRA1, HTT, HUWE1, ILF2, ILF3, ILK, INCENP, IPO5, IQGAP1, IQGAP2, IQGAP3, IRF2BPL, IRF3, IST1, ITCH, ITGA5, ITGA6, ITGAV, ITGB1, JAM3, JARID2, KAT7, KDM1A, KEAP1, KIF23, KIF5B, LAMB1, LAMC1, LANCL2, LARS1, LGALS1, LIMS1, LIN28A, LMAN1, LMCD1, LNPK, LPCAT1, LPGAT1, LRP1, LRRC59, LRRC8A, LRRK1, LSM4, LYAR, MACF1, MACROH2A1, MAGED1, MAP1B, MAP2K6, MAPK1, MAPK14, MAPK8, MARCHF5, MARS1, MATR3, MAVS, MCAM, MCU, MEAK7, MESD, METAP1, MFGE8, MIPEP, MLH1, MMAB, MME, MOSPD2, MPC2, MRPS27, MSH2, MSN, MTA3, MTDH, MVD, MYADM, MYBBP1A, MYD88, MYDGF, MYH10, MYH9, MYO1C, MYO1E, NBAS, NCAPG, NCKAP1, NCL, NDC80, NDRG1, NECTIN2, NEDD4, NEK7, NFKB1, NIBAN1, NIBAN2, NID1, NIF3L1, NLE1, NNT, NOL11, NPM1, NQO2, NRP2, NSF, NSFL1C, NSUN5, NUDT16, NVL, OCLN, OGT, OPTN, ORC3, OSBP, OXCT1, P3H1, P4HB, PAK1, PARD3, PARN, PARP1, PASK, PATZ1, PAWR, PAXBP1, PC, PCOLCE, PDCD2, PDCL3, PDGFRB, PDIA3, PDLIM7, PEA15, PELP1, PFKM, PHIP, PHPT1, PIAS4, PICALM, PIK3C2A, PIK3CA, PIK3R4, PIP4K2B, PIP4K2C, PKM, PKP2, PLCG1, PLIN3, PLK1, PLPP3, PLXNB2, PNP, PNPT1, POFUT2, POGZ, POLR1B, POLR2A, POLR2B, POLR2G, POLR3A, POLR3C,	

					POLR3F, POR, PPIB, PPID, PPM1B, PPM1F, PPP1R9B, PPP2R5A, PRKAA1, PRKAB1, PRKACB, PRKACG, PRKAG1, PRKAR1A, PRKAR2A, PRKAR2B, PRKCA, PRKCSH, PRKDC, PRPF38B, PRRC1, PSMC6, PSMD1, PSMD2, PSMD9, PSME3, PSPC1, PTGIS, PTK7, PTPN1, PTPN2, PTPRD, PURA, PXDN, PYCARD, RAB1A, RAC3, RAD51, RAN, RANBP2, RAP2A, RAP2B, RAP2C, RBM15, RBM3, RBPJ, RBPMS, RBPMS2, RCC2, RDH10, RDX, RELA, RFC1, RFC2, RFC3, RFC5, RFTN1, RHEB, RIF1, ROCK2, RPA1, RPA2, RPA3, RPL5, RPS2, RPS6KA1, RPS6KA3, RPS6KA4, RRAS, S100A10, S100A11, SAAL1, SALL2, SAMD4B, SAP30BP, SAR1A, SARM1, SART3, SCARB2, SEC13, SEC16A, SEC22B, SEH1L, SEPTIN7, SERPINB9, SESN2, SETD7, SETDB1, SF3B1, SH3GL3, SH3GLB1, SIN3A, SIRT1, SIRT2, SKP2, SLAIN2, SLC1A3, SLC25A12, SLC25A5, SLC2A10, SLC44A2, SLC5A3, SMARCA4, SMARCA5, SMARCC1, SMARCD1, SMARCD2, SNX3, SNX4, SNX9, SORBS1, SORBS3, SORD, SPAG9, SPARC, SPON1, SPTBN1, SRC, SRPK1, SRSF1, SSBP1, SSR1, STIM1, STX5, STXBP2, SUN2, SUPT16H, SUPT5H, SUPT6H, SURF4, SYMPK, TBRG4, TCAF1, TCP1, TELO2, TENM3, TF, TFG, TGFB1I1, TGM2, THBS1, TIA1, TIGAR, TIMP3, TM9SF4, TMED10, TMED2, TMED9, TMF1, TOLLIP, TOP2A, TPM1, TPX2, TRIM28, TRIM71, TRMT10C, TTK, TWF1, UBE2O, UBR5, UFL1, UPF3B, USP15, USP19, USP47, USP5, USP7, USP9X, UTP15, UTP25, UTRN, VAMP2, VAMP3, VIM, VLDLR, VPS35, WAPL, WARS1, WDR43, WRAP53, XPO5, XRCC5, XRCC6, YAP1, YTHDC2, ZC3HAV1, ZDHHC17, ZFP36L2, ZMYND8, ZNF281, ZNF462]	
negative regulation of biological process	GO_BiologicalProcess-	1.49E-64	9.354505	542	[AAAS, AASS, AATF, ABAT, ABCB7, ABI1,	No change

	EBI-UniProt-GOA-ACAP-ARAP_13.05.2021_00h00				ACOT13, ACTN1, ACTN4, ADAM10, ADAR, ADARB1, ADD2, ADD3, AEBP2, AGO1, AGO2, AGTPBP1, AIMP2, AKT1, ANK2, ANK3, ANTXR1, ANXA1, ANXA2, ANXA4, ANXA5, ANXA6, AP2A1, AP2B1, AP2M1, APAF1, API5, APOBEC3C, APOE, APPL2, ARAP1, ARF4, ARF6, ARFGEF1, ARHGAP1, ARHGAP28, ARHGDIA, ARL2, ARL6IP5, ASAP1, ASNS, ATF7IP, ATP1A1, ATP2B1, ATP2B4, ATR, AURKA, AURKB, B4GALT1, BAG1, BAG2, BAG3, BIN1, BIRC6, BRMS1, BUB1B, C1QBP, CACYBP, CALR, CAMK2D, CAPRIN1, CAPZA2, CARHSP1, CASK, CASP3, CAST, CASTOR2, CAT, CAV1, CBL, CBX2, CBX5, CCAR1, CCAR2, CCDC22, CCNB1, CCND2, CD2AP, CD44, CDC42, CDC73, CDH2, CDH3, CDK5RAP1, CDK5RAP3, CELF1, CHEK1, CHEK2, CHID1, CIRBP, CLASP1, CNN1, CNOT11, CNTFR, COL1A1, COL4A2, COL5A1, COL5A2, COL6A3, COPS2, COPS5, CORO1C, CRLF3, CRMP1, CRTAP, CSDE1, CTBP2, CTCF, CTNNB1, CTSC, CUL4A, CYFIP1, CYP51A1, DAPK1, DAXX, DCAF1, DDRGK1, DDX20, DDX39B, DDX5, DDX54, DDX6, DHX15, DHX9, DIAPH1, DICER1, DIPK2A, DLG1, DNMT1, DNMT3A, DNMT3B, DOCK7, DPYSL3, DUSP3, EEF1E1, EFNB2, EGFR, EHMT1, EIF3A, EIF4A3, EMD, EPB41L5, EPCAM, EPN2, EPS8, ERBIN, ERLEC1, ERLIN2, ERO1A, EXOSC10, EXOSC2, EXOSC5, EXOSC7, F11R, FANCD2, FASTKD2, FBN2, FBXO2, FERMT2, FGG, FHL1, FKBP4, FKBP8, FLNB, FLT1, FN1, FOXK1, FTH1, FXR1, G3BP1, GARS1, GATA6, GATAD2A, GBE1, GCLC, GCLM, GDI1, GIPC1, GLA, GLMN, GOLGA2, GOLT1B, GOPC, GPC1, GPC3, GPD1L, GPI, GPSM1, GSDME, GSN, GSTM2, GTPBP1, HADHA, HAT1, HCFC1, HDAC2, HDAC4, HELLS,	

					HK2, HLA-A, HMOX1, HNRNPM, HOOK3, HSP90AB1, HSP90B1, HSPA2, HSPA5, HSPA9, HSPB1, HSPD1, HTRA1, HTT, HUWE1, IDH2, IGF2BP3, ILF3, ILK, IPO5, IQGAP3, IRF2BPL, IRF3, ITCH, ITGA5, ITGA6, ITGAV, ITGB1, JADE1, JAM3, JARID2, KANK2, KAT7, KDM1A, KDM2A, KNTC1, KRT18, LANCL2, LGALS1, LIG3, LIMS1, LIN28A, LMAN1, LMCD1, LNPEP, LPCAT1, LRP1, LRPPRC, LRRK1, LSM4, LTBP1, LYAR, MACROH2A1, MAGED1, MAP1B, MAP2K6, MAP4, MAPK1, MAPK14, MAPK8, MARCHF5, MASTL, MAVS, MBD3, MCC, MCM2, MCM7, MDC1, MEAK7, METAP1, MICAL1, MLH1, MMAB, MSH2, MSH3, MSH6, MTA3, MTDH, MVK, MYADM, MYBBP1A, MYD88, MYDGF, MYEF2, MYH9, MYO1C, NBAS, NCAPG, NCL, NDC1, NDC80, NDRG1, NDRG2, NEDD4, NFKB1, NIBAN1, NIBAN2, NIF3L1, NLE1, NLGN4X, NNT, NPM1, NSFL1C, NSUN2, NUCB2, NUDT16, NUP107, NUP133, NUP160, NUP210, NUP35, NUP50, NXN, OCLN, OGT, OPTN, ORC2, P3H1, P3H3, PAK1, PARD3, PARN, PARP1, PARVA, PASK, PATZ1, PAWR, PC, PCBP2, PDCL3, PDGFRB, PDS5A, PEA15, PFKL, PGAM5, PHC1, PHIP, PHPT1, PIAS4, PICALM, PIK3CA, PIP4K2B, PIP4K2C, PKP2, PLCG1, PLK1, PLPP3, PLXNB2, PNPT1, POLR1B, POLR2A, POLR2B, POLR2G, POR, PPID, PPM1B, PPM1F, PPP1R9B, PPP2R5A, PRDX4, PRKAA1, PRKACB, PRKACG, PRKAR1A, PRKAR2A, PRKAR2B, PRKCA, PRKDC, PRMT3, PSMC6, PSMD1, PSMD2, PSMD9, PSME3, PSPC1, PTBP2, PTGIS, PTPN1, PTPN12, PTPN2, PTPRD, PURA, PUS7, PXDN, PXK, PXMP2, PYCARD, PYCR1, RAB23, RAD51, RAN, RANBP2, RANGAP1, RAP2A, RAP2B, RAP2C, RBM15, RBPJ, RBPMS2, RCC2,	

					RDX, RELA, RFC1, RHEB, RIC1, RIF1, RIOX1, RNH1, ROCK2, RPA2, RPL13A, RPL22, RPL27A, RPL4, RPL5, RPL7A, RPS2, RPS21, RPS6KA1, RPS6KA3, RPS6KA4, RPS8, RRAS, S100A11, SALL2, SAMD4B, SAMHD1, SARM1, SARS1, SCFD1, SEC13, SEC22B, SEH1L, SERBP1, SERPINB6, SERPINB9, SERPINH1, SESN2, SESTD1, SET, SETDB1, SH3GL3, SIN3A, SIRT1, SIRT2, SLC25A12, SLC25A5, SLC2A10, SLIRP, SMARCA4, SMARCA5, SMARCC1, SMPDL3B, SNX3, SNX6, SORBS3, SORD, SPAG9, SPARC, SPON1, SPOUT1, SPTAN1, SPTBN1, SPTBN2, SRC, SRPK1, SRSF7, SSB, STAM2, STK38, STYX, SUPT5H, SUPT6H, TAX1BP1, TBC1D4, TBRG4, TCAF1, TDP2, TELO2, TGFB1I1, THBS1, TIA1, TIGAR, TIMP3, TIPRL, TKFC, TMED10, TMED2, TMEM115, TMF1, TOP2A, TPM1, TPX2, TRAP1, TRIM28, TRIM33, TRIM71, TRIP12, TRIP13, TTK, TTLL12, TUT1, TWF1, TXNDC5, UBE2O, UBR5, UFL1, UNG, UPF2, UPF3B, USP15, USP19, USP47, USP7, USP9X, UTP20, VAMP3, VAT1, VIM, VPS25, VPS35, WAPL, WARS1, XPO5, XRCC5, XRCC6, XRN1, XRN2, YAP1, YOD1, YTHDC2, ZC3H7B, ZC3HAV1, ZFP36L2, ZMYND8, ZNF217, ZNF281, ZWILCH]	
regulation of metabolic process	GO_BiologicalProcess-EBI-UniProt-GOA-ACAP-ARAP_13.05.2021_00h00	1.49E-64	8.769725	628	[AAAS, AASS, AATF, ABAT, ABCB10, ABCB7, ABCF1, ABHD14B, ABI1, ACACA, ACLY, ACO1, ACOT13, ACSL1, ACTN1, ACTN4, ACTR2, ADAM10, ADAR, ADARB1, AEBP2, AGO1, AGO2, AGTPBP1, AIMP2, AK4, AKT1, ANK2, ANK3, ANTXR1, ANXA1, ANXA2, ANXA3, ANXA4, AP2A1, AP2M1, AP3B1, APAF1, APOE, APPL2, ARAP1, ARF4, ARFGEF1, ARHGEF11, ARL6IP5, ASPH, ATF7IP, ATP1A1, ATP2B1, ATP2B4, ATP6V1A, ATR, AURKA, AURKB, BAG2, BAG3, BAZ1B, BCCIP, BIN1, BIRC6, BPTF,	No change

					BRAT1, BRD3, BRD4, BRMS1, BRWD1, C1QBP, CACYBP, CALCOCO2, CALR, CAMK1, CAMK2D, CAPN1, CAPN2, CAPNS1, CAPRIN1, CARHSP1, CASK, CASP3, CAST, CAT, CAV1, CBL, CBR1, CBX2, CBX5, CCAR1, CCAR2, CCDC22, CCNB1, CCND2, CCNH, CCNL2, CCNY, CCT2, CCT3, CCT4, CCT5, CCT6A, CCT7, CCT8, CD2AP, CD44, CD81, CDC123, CDC42, CDC73, CDH3, CDK5RAP1, CDK5RAP3, CDK7, CEBPZ, CELF1, CHD1, CHD4, CHEK1, CHEK2, CHID1, CHTF18, CIRBP, CNOT11, CNRIP1, COL1A1, COL6A3, COPS2, COPS5, CORO1C, CRLF3, CRTAP, CSDE1, CSPG4, CTBP2, CTCF, CTNNB1, CTSC, CUL4A, CYFIP1, CYP51A1, DAGLB, DAPK1, DAXX, DAZAP1, DBNL, DCAF1, DCXR, DDRGK1, DDX20, DDX21, DDX39B, DDX41, DDX49, DDX5, DDX54, DDX6, DECR1, DHX33, DHX8, DHX9, DICER1, DIPK2A, DLG1, DLGAP5, DNMT1, DNMT3A, DNMT3B, DOCK7, DPH1, DPH2, DPH6, DUSP3, ECE1, ECT2, EDRF1, EEF2, EFL1, EGFR, EHD4, EHMT1, EIF3A, EIF4A3, EIF5B, ELP3, EMSY, EPB41L5, EPCAM, EPHB3, EPM2AIP1, ERBIN, ERCC2, ERLIN2, ETFA, EXOSC10, EXOSC2, EXOSC5, EXOSC7, F11R, FASTKD2, FBXO2, FBXW8, FDPS, FERMT2, FH, FKBP8, FLNB, FLT1, FN1, FOXK1, FXR1, G3BP1, G3BP2, GARS1, GATA6, GATAD2A, GCLC, GEMIN5, GIPC1, GLA, GLMN, GNL3, GOLGA2, GOLT1B, GPC3, GPD1L, GPI, GPSM1, GSN, GTF2I, GTPBP1, GUF1, H6PD, HADHA, HAT1, HCFC1, HDAC2, HDAC4, HEATR1, HELLS, HK1, HK2, HLA-A, HMOX1, HNRNPF, HNRNPL, HNRNPLL, HNRNPM, HP1BP3, HSD17B4, HSP90AB1, HSPA2, HSPA5, HSPB1, HSPD1, HSPE1, HSPH1, HTT, HUWE1, IDI1, IGF2BP3,	

					ILF2, ILF3, ILK, INCENP, IPO5, IQGAP1, IQGAP3, IRF2BPL, IRF3, IST1, ITCH, ITGA5, ITGA6, ITGAV, ITGB1, IWS1, JARID2, KANK2, KAT7, KDM1A, KDM2A, KDM3B, KEAP1, LANCL2, LCMT1, LIG3, LIMS1, LIN28A, LMCD1, LNPEP, LPCAT1, LPGAT1, LRP1, LRPPRC, LRRK1, LSM4, LSS, LTBP1, LYAR, MACROH2A1, MAGED1, MAP2K6, MAPK1, MAPK14, MAPK8, MARS1, MASTL, MATR3, MAVS, MBD3, MBNL1, MCM2, MCM7, ME1, METAP1, MICAL1, MLH1, MMAB, MRPS27, MSH2, MSH3, MSH6, MSN, MTA3, MTDH, MVD, MVK, MYADM, MYBBP1A, MYD88, MYDGF, MYEF2, MYH9, MYO1C, MYO1E, NBAS, NCL, NDC1, NDRG2, NEDD4, NEK7, NFKB1, NIBAN1, NIBAN2, NIF3L1, NLE1, NLN, NNT, NOL11, NPM1, NQO2, NSF, NSUN2, NSUN5, NUDT16, NUP107, NUP133, NUP160, NUP210, NUP35, NUP50, NVL, NXN, OCLN, OGT, OPTN, ORC2, ORC3, ORC5, OSBP, P3H1, PABPN1, PAK1, PARD3, PARN, PARP1, PARVA, PASK, PATZ1, PAWR, PAXBP1, PC, PCBP2, PCOLCE, PDCD2, PDCL3, PDGFRB, PDS5A, PEA15, PELP1, PFKM, PGAM5, PHC1, PHIP, PIAS4, PICALM, PIK3C2A, PIK3CA, PIK3R4, PIP4K2B, PIP4K2C, PKM, PLD3, PLK1, PLPP3, PLXNB2, PM20D2, PNP, PNPT1, POFUT1, POFUT2, POGZ, POLA1, POLR1B, POLR2A, POLR2B, POLR2G, POLR3A, POLR3C, POLR3F, POR, PPIB, PPID, PPM1B, PPM1F, PPP1R7, PPP1R9B, PPP2R5A, PPP4R1, PRKAA1, PRKAB1, PRKACB, PRKACG, PRKAG1, PRKAR1A, PRKAR2A, PRKAR2B, PRKCA, PRKCSH, PRKDC, PRMT3, PRRC1, PSIP1, PSMC6, PSMD1, PSMD2, PSMD9, PSME3, PSPC1, PTBP2, PTCD3, PTGIS, PTPN1, PTPN2, PURA, PUS7,	

					PXDN, PYCARD, RAB1A, RAB8A, RAD51, RAN, RANBP2, RAP2A, RAP2B, RAP2C, RBM15, RBM25, RBM3, RBPJ, RBPMS, RDH10, RDX, RELA, RFC1, RFC2, RFC3, RFC5, RFTN1, RHEB, RIC1, RIF1, RIOX1, RNH1, ROCK2, RPA2, RPL13A, RPL22, RPL27A, RPL4, RPL5, RPL7A, RPRD1A, RPS2, RPS21, RPS6KA1, RPS6KA3, RPS6KA4, RPS8, S100A11, SALL2, SAMD4B, SAP30BP, SARS1, SART3, SCARB2, SCFD1, SEC13, SEC22B, SEH1L, SERBP1, SERPINB6, SERPINB9, SERPINH1, SESN2, SET, SETD7, SETDB1, SF3B1, SH3GLB1, SIN3A, SIRT1, SIRT2, SKP2, SLC25A12, SLC25A5, SLC2A10, SLC5A3, SLIRP, SLTM, SMARCA4, SMARCA5, SMARCAD1, SMARCC1, SMARCD1, SMARCD2, SNX3, SNX6, SNX9, SORBS1, SORBS3, SORD, SPAG9, SPON1, SPOUT1, SPTBN1, SRC, SRPK1, SRSF1, SRSF7, SSB, SSBP1, STK38, STX5, STXBP2, STYX, SUPT16H, SUPT5H, SUPT6H, SYMPK, TAX1BP1, TBRG4, TCP1, TDP2, TELO2, TF, TFB1M, TGFB1I1, THBS1, TIA1, TIGAR, TIMP3, TIPRL, TMED10, TMED2, TMF1, TOLLIP, TOP2A, TPX2, TRAP1, TRIM28, TRIM33, TRIM71, TRIP12, TRIP13, TRMT10C, TRPT1, TTK, TTLL12, TUT1, TWF1, TXNRD1, UBE2O, UBR5, UFL1, UPF2, UPF3B, USP19, USP47, USP5, USP7, USP9X, UTP15, UTP4, VAMP3, VIM, VLDLR, VPS25, VPS26A, VPS26B, VPS35, VRTN, WAPL, WARS1, WDR18, WDR43, WRAP53, XPO5, XRCC5, XRCC6, XRN1, XRN2, YAP1, ZC3H7B, ZC3HAV1, ZFP36L2, ZMYND8, ZNF217, ZNF281, ZNF462]	
nucleic acid binding	GO_MolecularFunction-EBI-UniProt-GOA-ACAP-ARAP_13.05.2021_00h00	1.49E-64	10.94682	459	[AATF, ABCF1, ACIN1, ACO1, ACTN4, ADAR, ADARB1, AEBP2, AGO1, AGO2, ANTXR1, ANXA1, ANXA11, ANXA2, APEH, API5, APOBEC3C, ARCN1, ARHGEF1, ATR, BCCIP, BMS1, BOP1, BPTF, C1QBP, CALR,	Upregulation

					CANX, CAPRIN1, CARHSP1, CAST, CAVIN1, CBX2, CCAR1, CCAR2, CCT3, CCT4, CCT5, CCT6A, CEBPZ, CELF1, CFAP20, CHD1, CHD4, CHTF18, CIRBP, CKAP4, CLUH, COPS5, CPSF2, CPSF3, CRLF3, CSDE1, CSTF1, CSTF3, CTCF, CTNNB1, CUL4B, CYFIP1, DARS2, DAZAP1, DCAF13, DDRGK1, DDX10, DDX18, DDX20, DDX21, DDX24, DDX28, DDX31, DDX39B, DDX41, DDX42, DDX46, DDX47, DDX49, DDX5, DDX52, DDX54, DDX6, DHX15, DHX30, DHX33, DHX37, DHX57, DHX8, DHX9, DIAPH1, DICER1, DNMT1, DNMT3A, DNMT3B, DNTTIP2, DSP, EEF2, EFL1, EFTUD2, EGFR, EHD2, EHD4, EIF3A, EIF4A3, EIF5B, ELAC2, ELP1, ELP3, ENDOD1, ERCC2, ERCC6L, ESF1, EXD2, EXOG, EXOSC10, EXOSC2, EXOSC5, EXOSC7, FAM120A, FANCI, FASTKD2, FDPS, FKBP4, FLNB, FNDC3A, FNDC3B, FOXK1, FSCN1, FTSJ3, FXR1, G3BP1, G3BP2, GARS1, GATA6, GATAD2A, GEMIN5, GNL2, GNL3, GOT2, GTF2I, GTF3C1, GTF3C2, GTF3C3, GTF3C4, GTPBP1, HADHB, HDAC2, HDAC4, HDLBP, HEATR1, HLA-A, HMOX1, HNRNPF, HNRNPL, HNRNPLL, HNRNPM, HP1BP3, HSD17B10, HSD17B4, HSP90AB1, HSP90B1, HSPA9, HSPB1, HSPD1, HSPE1, HUWE1, IGF2BP3, ILF2, ILF3, IMP3, IMPDH1, IMPDH2, IPO5, IRF3, JARID2, KAT7, KCTD12, KDM1A, KDM2A, KDM3B, KIF22, KIF2C, KRT18, KTN1, L1TD1, LAS1L, LBR, LGALS1, LIG1, LIG3, LIN28A, LRP1, LRPPRC, LRRC59, LRWD1, LSM4, LYAR, MACF1, MACROH2A1, MAP1S, MAP4, MAPK1, MAPK8, MARS1, MATR3, MBD3, MBNL1, MCM2, MCM3, MCM4, MCM5, MCM6, MCM7, METTL1, MEX3A, MKI67, MLH1, MMAB, MPHOSPH10, MRPS27, MRPS9, MSH2, MSH3, MSH6, MSI1, MTA3,	

					MTDH, MTREX, MYBBP1A, MYEF2, MYH10, MYH9, MYO5A, NCL, NEDD4, NFKB1, NIBAN2, NOL10, NOL11, NOL6, NOL9, NOP14, NOP56, NOP58, NOP9, NPM1, NPM3, NSUN2, NSUN5, NTHL1, NUCB2, NUDT16, NUP35, NUSAP1, NVL, ORC2, ORC3, ORC4, ORC5, P4HB, PABPN1, PARN, PARP1, PATZ1, PAXBP1, PCBP2, PDCD11, PDCD2, PDIA3, PDIA4, PELP1, PES1, PHC1, PIAS4, PKM, PLEC, PNO1, PNPT1, POGZ, POLA1, POLA2, POLB, POLD1, POLE, POLR1B, POLR2A, POLR2B, POLR2G, POLR3A, POLR3C, POLR3F, PPIB, PRDX1, PRIM2, PRKDC, PRPF38A, PRPF38B, PRPF40A, PRPF8, PSIP1, PSMD2, PSPC1, PTBP2, PTCD1, PTCD3, PTPN1, PURA, PUS1, PUS3, PUS7, PWP2, RAD51, RAD54L2, RAN, RANBP2, RANGAP1, RBBP5, RBM15, RBM25, RBM26, RBM3, RBM6, RBMS2, RBPJ, RBPMS, RBPMS2, RCC2, RDX, RELA, RFC1, RFC2, RFC3, RFC5, ROCK2, RPA1, RPA2, RPA3, RPF2, RPL13A, RPL22, RPL27A, RPL4, RPL5, RPL7A, RPN1, RPS2, RPS21, RPS8, RRBP1, RRP12, RRP7A, RRS1, RTCA, SALL2, SAMD4B, SAMHD1, SARS1, SARS2, SART3, SBDS, SCYL1, SERBP1, SERPINH1, SET, SETDB1, SF3A3, SF3B1, SIN3A, SIRT1, SIRT2, SLC25A5, SLIRP, SLTM, SMARCA4, SMARCA5, SMARCAD1, SMARCC1, SMARCD2, SMC2, SNRNP200, SNRPA1, SNRPD3, SNTB2, SPATS2L, SPOUT1, SPTBN1, SRP14, SRPK1, SRPRA, SRSF1, SRSF11, SRSF7, SSB, SSBP1, SSRP1, SUCLG1, SUPT16H, SUPT5H, SUPT6H, TBL2, TBL3, TBPL1, TBRG4, TCP1, TDP1, TDP2, TELO2, TFB1M, THUMPD3, TIA1, TIMM50, TMF1, TNS1, TOP2A, TPD52L2, TRAP1, TRIM28, TRIM33, TRIM71, TRMT1, TRMT10C, TRMT1L, TSEN34, TSR1,	

					TTF2, TUT1, UBE2O, UBFD1, UBR5, UNG, UPF2, UPF3B, UTP15, UTP18, UTP20, UTP25, UTP4, VRTN, WDHD1, WDR3, WDR36, WDR43, WRAP53, XPO5, XRCC5, XRCC6, XRN1, XRN2, YAP1, YARS2, YRDC, YTHDC2, ZC3H15, ZC3H7B, ZC3HAV1, ZFP36L2, ZMYM3, ZNF217, ZNF281, ZNF462, ZNF532, ZNF638]	
cellular aromatic compound metabolic process	GO_BiologicalProcess-EBI-UniProt-GOA-ACAP-ARAP_13.05.2021_00h00	1.49E-64	10.20442	624	[AAAS, AASS, AATF, ABAT, ABCB10, ABCB7, ABCC1, ABHD14B, ACAA1, ACACA, ACAT1, ACIN1, ACLY, ACOT13, ACOT9, ACSL1, ACSL4, ACSS2, ACTN1, ACTN4, ACTR2, ADAR, ADARB1, ADPGK, ADSL, AEBP2, AGO1, AGO2, AIMP2, AK1, AK4, AKR7A2, AKT1, ALDH1L2, ALDOC, AMDHD2, AMPD2, ANKRD28, ANTXR1, ANXA3, ANXA4, AP3B1, APOBEC3C, APOE, APRT, ARF4, ARHGEF11, ATF7IP, ATIC, ATP2B4, ATP6V1A, ATR, AURKB, BAG3, BAZ1B, BCCIP, BDH2, BLVRB, BMS1, BOP1, BPNT2, BPTF, BRD3, BRD4, BRMS1, BRWD1, C1QBP, CALR, CAMK1, CAMK2D, CARHSP1, CASK, CASP3, CAT, CAV1, CAVIN1, CBX2, CBX5, CCAR1, CCAR2, CCDC22, CCNB1, CCNH, CCNL2, CCT2, CCT3, CCT4, CCT5, CCT6A, CCT7, CCT8, CD81, CDC73, CDH3, CDK5RAP1, CDK5RAP3, CDK7, CEBPZ, CELF1, CHAF1A, CHD1, CHD4, CHEK1, CHEK2, CHTF18, CIRBP, CMPK1, CNOT11, COL1A1, COL4A2, COPS2, COPS5, COPS7A, CPOX, CPSF2, CPSF3, CRLF3, CRMP1, CSDE1, CSTF1, CSTF3, CTBP2, CTCF, CTNNB1, CTNNBL1, CTPS1, CUL4A, CUL4B, CWC27, CYFIP1, DARS2, DAXX, DAZAP1, DCAF1, DCAF13, DCTD, DDRGK1, DDX10, DDX18, DDX20, DDX21, DDX24, DDX39B, DDX41, DDX42, DDX46, DDX47, DDX49, DDX5, DDX52, DDX54, DDX6, DHX15, DHX33, DHX37, DHX8, DHX9, DICER1, DLG1, DLGAP5, DNMT1, DNMT3A, DNMT3B,	Upregulation

					DPYSL2, DPYSL3, DRG1, EDRF1, EEF1E1, EFL1, EFTUD2, EGFR, EHMT1, EIF4A3, ELAC2, ELP1, ELP3, EMSY, ENDOD1, ENO2, EPCAM, ERBIN, ERCC2, ESF1, EXD2, EXOG, EXOSC10, EXOSC2, EXOSC5, EXOSC7, FANCD2, FANCI, FASTKD2, FECH, FH, FLAD1, FOXK1, FSCN1, FTSJ3, FXR1, G3BP1, G3BP2, GALK1, GARS1, GART, GATA6, GATAD2A, GCDH, GCLC, GEMIN4, GEMIN5, GFPT2, GINS3, GIPC1, GMPPB, GMPS, GNE, GNL3, GNPDA2, GOT2, GPI, GSTM2, GSTM3, GTF2E1, GTF2I, GTF3C1, GTF3C2, GTF3C3, GTF3C4, GTPBP1, HAT1, HCFC1, HDAC2, HDAC4, HEATR1, HELLS, HERC2, HK1, HK2, HMBS, HMGCL, HMOX1, HMOX2, HNRNPF, HNRNPL, HNRNPLL, HNRNPM, HOOK3, HP1BP3, HSD17B10, HSD17B4, HSP90AB1, HSPA5, HSPB1, HSPD1, HUWE1, IDH2, IGF2BP3, ILF2, ILF3, ILK, IMP3, IMPDH1, IMPDH2, INO80C, IRF2BPL, IRF3, ITCH, ITGA6, IWS1, JARID2, KANK2, KAT7, KDM1A, KDM2A, KDM3B, KEAP1, KIF22, LANCL2, LARS1, LARS2, LAS1L, LIG1, LIG3, LIMS1, LIN28A, LMCD1, LRPPRC, LRWD1, LSM4, LYAR, MACROH2A1, MAGED1, MAP1S, MAPK1, MAPK14, MAPK8, MARS1, MAVS, MBD3, MBNL1, MCCC2, MCM2, MCM3, MCM4, MCM5, MCM6, MCM7, MDC1, MDN1, ME1, METTL1, MLH1, MMAB, MOXD1, MPC2, MPHOSPH10, MSH2, MSH3, MSH6, MTA3, MTAP, MTDH, MTHFD1, MTR, MTREX, MVD, MVK, MYBBP1A, MYD88, MYDGF, MYEF2, MYO1C, NBAS, NCAPG2, NCL, NDC1, NDRG1, NEDD4, NEK7, NFKB1, NIBAN2, NIF3L1, NOL10, NOL11, NOL6, NOL9, NOP14, NOP56, NOP58, NOP9, NPM1, NPM3, NSUN2, NSUN5, NT5C2, NTHL1, NUDT16, NUP107, NUP133, NUP160, NUP210, NUP35, NUP50, NVL, OGT,	

					ORC2, ORC3, ORC4, ORC5, PABPN1, PAICS, PAPSS2, PARD3, PARN, PARP1, PATZ1, PAWR, PAXBP1, PCBP2, PDCD11, PDGFRB, PDS5A, PDXK, PELP1, PES1, PFAS, PFKL, PFKM, PFKP, PGM3, PHC1, PHIP, PIAS4, PICALM, PIK3CA, PKM, PLD3, PLK1, PLPP3, PM20D2, PNP, PNPT1, POFUT1, POGZ, POLA1, POLA2, POLB, POLD1, POLE, POLR1B, POLR2A, POLR2B, POLR2G, POLR3A, POLR3C, POLR3F, PON2, PPID, PPM1F, PPP1R9B, PRIM1, PRIM2, PRKAA1, PRKAG1, PRKAR1A, PRKCA, PRKDC, PRORP, PRPF38A, PRPF38B, PRPF40A, PRPF8, PRPSAP1, PSIP1, PSMC6, PSMD1, PSMD2, PSMD9, PSME3, PSPC1, PTBP2, PTCD1, PTGIS, PTPN2, PURA, PUS1, PUS3, PUS7, PWP2, PXDN, PYCARD, QDPR, QRSL1, RAB23, RAD51, RAN, RANBP2, RAP2C, RBM15, RBM25, RBM26, RBM3, RBM6, RBMS2, RBPJ, RBPMS, RELA, RFC1, RFC2, RFC3, RFC5, RIF1, RIOX1, RNH1, ROCK2, RPA1, RPA2, RPA3, RPF2, RPL13A, RPL22, RPL27A, RPL4, RPL5, RPL7A, RPRD1A, RPS2, RPS21, RPS6KA1, RPS6KA3, RPS6KA4, RPS8, RRP12, RRP7A, RRS1, RTCA, SALL2, SAMD4B, SAMHD1, SAP30BP, SARM1, SARS1, SARS2, SART3, SBDS, SEC13, SEH1L, SERBP1, SESN2, SET, SETD7, SETDB1, SF3A3, SF3B1, SIN3A, SIRT1, SIRT2, SLC25A12, SLC25A32, SLIRP, SLTM, SMARCA4, SMARCA5, SMARCAD1, SMARCC1, SMARCD1, SMARCD2, SNRNP200, SNRPA1, SNRPD3, SNX6, SORBS3, SORD, SPOUT1, SPR, SRC, SRPK1, SRSF1, SRSF11, SRSF7, SSB, SSRP1, STXBP2, SUPT16H, SUPT5H, SUPT6H, SYMPK, TARS2, TAX1BP1, TBL3, TBPL1, TBRG4, TCP1, TDP1, TDP2, TELO2, TEX10, TF, TFB1M, TGFB1I1, THUMPD3, TIA1, TIGAR, TJP2,	

					TMF1, TOP2A, TPST1, TRIM28, TRIM33, TRIM71, TRIP12, TRIP13, TRMT1, TRMT10C, TRMT1L, TRMT5, TRPT1, TSEN34, TSR1, TTF2, TUT1, TXNRD1, UAP1, UBR5, UFL1, UGDH, UGGT1, UGGT2, UGP2, UNG, UPF2, UPF3B, USP47, USP7, USP9X, UTP15, UTP18, UTP20, UTP25, UTP4, VIM, VRTN, WARS1, WDHD1, WDR18, WDR3, WDR36, WDR43, WRAP53, XPO5, XRCC5, XRCC6, XRN1, XRN2, YAP1, YARS2, ZC3H7B, ZC3HAV1, ZFP36L2, ZMYND8, ZNF217, ZNF281, ZNF462, ZNF638]	
negative regulation of metabolic process	GO_BiologicalProcess-EBI-UniProt-GOA-ACAP-ARAP_13.05.2021_00h00	1.49E-64	10.25335	344	[AAAS, AASS, AATF, ABCB7, ACOT13, ADAM10, ADAR, ADARB1, AEBP2, AGO1, AGO2, AKT1, ANTXR1, ANXA1, ANXA2, ANXA4, AP2A1, APOE, APPL2, ATF7IP, ATP1A1, ATP2B1, ATP2B4, ATR, AURKA, AURKB, BAG2, BAG3, BIN1, BIRC6, BRMS1, C1QBP, CALR, CAPRIN1, CARHSP1, CASP3, CAST, CAV1, CBL, CBX2, CBX5, CCAR1, CCAR2, CCNB1, CD2AP, CD44, CDC73, CDH3, CDK5RAP1, CDK5RAP3, CELF1, CHID1, CIRBP, CNOT11, COL6A3, COPS2, CORO1C, CRTAP, CSDE1, CTBP2, CTCF, CTNNB1, CYP51A1, DAPK1, DAXX, DCAF1, DDRGK1, DDX20, DDX5, DDX54, DDX6, DHX9, DICER1, DLG1, DNMT1, DNMT3A, DNMT3B, DOCK7, DUSP3, EGFR, EHMT1, EIF4A3, ERBIN, ERLIN2, EXOSC10, EXOSC2, EXOSC5, EXOSC7, FASTKD2, FKBP8, FN1, FOXK1, FXR1, GARS1, GATA6, GATAD2A, GCLC, GIPC1, GLA, GLMN, GOLGA2, GOLT1B, GPC3, GPD1L, GPI, GTPBP1, HAT1, HCFC1, HDAC2, HDAC4, HELLS, HK2, HMOX1, HNRNPM, HSP90AB1, HSPB1, HSPD1, IGF2BP3, ILF3, ILK, IPO5, IQGAP3, IRF2BPL, IRF3, ITCH, ITGAV, JARID2, KANK2, KAT7, KDM1A, KDM2A, LANCL2, LIG3, LIMS1, LIN28A, LMCD1, LNPEP,	Upregulation

					LPCAT1, LRP1, LRPPRC, LRRK1, LSM4, LYAR, MACROH2A1, MAGED1, MAP2K6, MAPK14, MASTL, MAVS, MBD3, METAP1, MICAL1, MLH1, MMAB, MSH2, MSH3, MSH6, MTA3, MTDH, MYADM, MYBBP1A, MYD88, MYEF2, MYO1C, NBAS, NCL, NDC1, NDRG2, NEDD4, NFKB1, NIBAN1, NIBAN2, NIF3L1, NLE1, NNT, NPM1, NSUN2, NUDT16, NUP107, NUP133, NUP160, NUP210, NUP35, NUP50, NXN, OCLN, OGT, OPTN, ORC2, P3H1, PARD3, PARN, PARP1, PARVA, PASK, PATZ1, PAWR, PC, PCBP2, PDS5A, PGAM5, PHC1, PIAS4, PICALM, PIK3CA, PIP4K2B, PIP4K2C, PLK1, PLPP3, PNPT1, POLR2A, POLR2B, POLR2G, POR, PPID, PPM1B, PPM1F, PPP1R9B, PPP2R5A, PRKAA1, PRKAR1A, PRKAR2A, PRKAR2B, PRKCA, PRKDC, PRMT3, PSMC6, PSMD1, PSMD2, PSMD9, PSME3, PSPC1, PTBP2, PTGIS, PTPN1, PTPN2, PURA, PUS7, PYCARD, RAN, RANBP2, RBM15, RBPJ, RELA, RFC1, RHEB, RIC1, RIF1, RIOX1, RNH1, ROCK2, RPL13A, RPL22, RPL27A, RPL4, RPL5, RPL7A, RPS2, RPS21, RPS6KA1, RPS6KA3, RPS6KA4, RPS8, S100A11, SALL2, SAMD4B, SARS1, SCFD1, SEC13, SEC22B, SEH1L, SERBP1, SERPINB6, SERPINB9, SERPINH1, SESN2, SET, SETDB1, SIN3A, SIRT1, SIRT2, SLC25A12, SLC2A10, SLIRP, SMARCA4, SMARCA5, SMARCC1, SNX3, SNX6, SORBS3, SORD, SPAG9, SPON1, SPOUT1, SRC, SRSF7, SSB, STK38, STYX, SUPT5H, SUPT6H, TAX1BP1, TBRG4, TDP2, TELO2, THBS1, TIA1, TIGAR, TIMP3, TIPRL, TMED10, TMED2, TMF1, TRAP1, TRIM28, TRIM33, TRIM71, TRIP12, TUT1, UBE2O, UBR5, UFL1, UPF2, UPF3B, USP19, USP47, USP7, USP9X, VIM, VPS25, WAPL, WARS1,	

					XPO5, XRCC5, XRCC6, XRN1, XRN2, YAP1, ZC3H7B, ZC3HAV1, ZFP36L2, ZMYND8, ZNF217, ZNF281]	
positive regulation of metabolic process	GO_BiologicalProcess-EBI-UniProt-GOA-ACAP-ARAP_13.05.2021_00h00	1.49E-64	9.989909	396	[AATF, ABAT, ABCB10, ABCB7, ABHD14B, ABI1, ACACA, ACLY, ACSL1, ACTN1, ACTN4, ACTR2, ADAR, AGO1, AGO2, AGTPBP1, AIMP2, AKT1, ANK2, ANK3, ANTXR1, ANXA1, ANXA2, ANXA3, AP2M1, AP3B1, APAF1, APOE, APPL2, ARAP1, ARF4, ARFGEF1, ARHGEF11, ARL6IP5, ASPH, ATF7IP, ATP2B4, ATR, AURKA, AURKB, BAG2, BAG3, BAZ1B, BPTF, BRAT1, BRD4, BRMS1, C1QBP, CACYBP, CALCOCO2, CALR, CAMK1, CAPN2, CASK, CASP3, CAV1, CBR1, CCAR1, CCAR2, CCDC22, CCNB1, CCND2, CCNY, CCT2, CCT3, CCT4, CCT5, CCT6A, CCT7, CCT8, CD44, CD81, CDC123, CDC42, CDC73, CDH3, CDK5RAP1, CDK5RAP3, CDK7, CEBPZ, CELF1, CHEK1, CHEK2, CHTF18, CIRBP, CNOT11, COL1A1, COPS5, CRLF3, CSPG4, CTBP2, CTCF, CTNNB1, CTSC, CYFIP1, DAGLB, DAPK1, DAXX, DAZAP1, DBNL, DCXR, DDRGK1, DDX21, DDX39B, DDX41, DDX5, DDX6, DECR1, DHX33, DHX8, DHX9, DIPK2A, DLG1, DLGAP5, DNMT1, DNMT3B, DOCK7, ECE1, ECT2, EDRF1, EEF2, EGFR, EHD4, EHMT1, EIF4A3, EPCAM, EPHB3, EPM2AIP1, ERCC2, ETFA, EXOSC2, EXOSC5, EXOSC7, FASTKD2, FBXW8, FERMT2, FH, FLNB, FLT1, FN1, FOXK1, FXR1, G3BP1, GARS1, GATA6, GCLC, GIPC1, GLMN, GNL3, GOLGA2, GPC3, GPI, GPSM1, GSN, GTF2I, GTPBP1, GUF1, HADHA, HCFC1, HDAC2, HDAC4, HEATR1, HK1, HLA-A, HMOX1, HNRNPLL, HSD17B4, HSP90AB1, HSPA2, HSPA5, HSPB1, HSPD1, HSPE1, HSPH1, HTT, HUWE1, ILF2, ILF3, ILK, INCENP, IQGAP1, IQGAP3, IRF2BPL, IRF3, IST1, ITCH, ITGA5, ITGA6, JARID2, KAT7, KDM1A, KEAP1, LIMS1, LIN28A, LPCAT1, LPGAT1, LRP1, LRRK1,	No change

					LSM4, LYAR, MACROH2A1, MAGED1, MAP2K6, MAPK1, MAPK14, MAPK8, MARS1, MAVS, MLH1, MMAB, MRPS27, MSH2, MSN, MTA3, MTDH, MYBBP1A, MYD88, MYDGF, MYH9, MYO1C, MYO1E, NBAS, NCL, NEDD4, NEK7, NFKB1, NIBAN1, NIBAN2, NIF3L1, NNT, NOL11, NPM1, NQO2, NSF, NSUN5, NVL, OCLN, OGT, OPTN, ORC3, OSBP, PAK1, PARD3, PARN, PARP1, PASK, PATZ1, PAWR, PAXBP1, PCOLCE, PDCD2, PDCL3, PDGFRB, PEA15, PELP1, PFKM, PHIP, PIAS4, PICALM, PIK3C2A, PIK3CA, PIK3R4, PIP4K2B, PIP4K2C, PKM, PLK1, PLXNB2, PNP, PNPT1, POGZ, POLR1B, POLR2A, POLR2G, POLR3A, POLR3C, POLR3F, POR, PPM1F, PPP2R5A, PRKAA1, PRKAB1, PRKACB, PRKACG, PRKAG1, PRKAR1A, PRKAR2A, PRKAR2B, PRKCA, PRKCSH, PRKDC, PRRC1, PSMC6, PSMD9, PSME3, PTPN1, PTPN2, PYCARD, RAB1A, RAD51, RANBP2, RAP2A, RAP2B, RAP2C, RBM15, RBM3, RBPJ, RBPMS, RDH10, RDX, RELA, RFC1, RFC2, RFC3, RFC5, RFTN1, RIF1, ROCK2, RPL5, RPS2, RPS6KA1, RPS6KA3, RPS6KA4, SALL2, SAMD4B, SART3, SEC22B, SERPINB9, SESN2, SETD7, SETDB1, SF3B1, SH3GLB1, SIN3A, SIRT1, SIRT2, SKP2, SLC25A12, SLC25A5, SLC2A10, SLC5A3, SMARCA4, SMARCA5, SMARCC1, SMARCD1, SMARCD2, SNX9, SORBS1, SORD, SPAG9, SPON1, SPTBN1, SRC, SRSF1, SSBP1, STX5, STXBP2, SUPT16H, SUPT5H, SUPT6H, SYMPK, TCP1, TELO2, TF, TGFB1I1, THBS1, TIGAR, TMED10, TMED2, TMF1, TOLLIP, TOP2A, TPX2, TRIM28, TRIM71, TRMT10C, TTK, UFL1, UPF3B, USP5, USP7, USP9X, UTP15, VAMP3, VIM, VLDLR, VPS35, WARS1, WDR43, WRAP53, XRCC5,	

					XRCC6, YAP1, ZC3HAV1, ZFP36L2, ZNF281, ZNF462]	
transferase activity, transferring phosphorus-containing groups	GO_MolecularFunction-EBI-UniProt-GOA-ACAP-ARAP_13.05.2021_00h00	1.49E-64	11.58676	203	[ABI1, ACSL1, ADAR, ADARB1, ADPGK, AK1, AK4, AKT1, ANTXR1, APOE, ATP2B4, ATR, AURKA, AURKB, BAZ1B, BCCIP, BUB1B, CAMK1, CAMK2D, CASK, CASP3, CAV1, CBL, CCNB1, CCND2, CCNH, CCNL2, CCNY, CCT2, CCT4, CD44, CD81, CDC42, CDC42BPA, CDC42BPB, CDK5RAP1, CDK5RAP3, CDK7, CHEK1, CHEK2, CHTF18, CLASP1, CMPK1, CORO1C, CSPG4, CTNNB1, DAPK1, DAXX, DBNL, DCAF1, DGKA, DIPK2A, DLG1, DUSP3, ECT2, EGFR, ELP3, EPHB3, ETFA, ETNK1, FERMT2, FLAD1, FLT1, GALK1, GMPPA, GMPPB, GNE, GPHN, GPI, GYS1, HK1, HK2, HOOK3, HSP90AB1, HSPB1, HTT, ILK, INCENP, IPO5, IQGAP1, IQGAP3, ITPK1, LRRK1, LTBP1, MACROH2A1, MAGED1, MAP2K6, MAPK1, MAPK14, MAPK8, MASTL, MCM2, MMAB, MVK, NEK7, NOL9, NPM1, NRP2, NVL, ORC3, PABPN1, PAK1, PAPSS2, PARN, PARVA, PASK, PCYT1A, PCYT2, PDGFRB, PDXK, PEA15, PFKL, PFKM, PFKP, PGM2L1, PI4KA, PIK3C2A, PIK3CA, PIK3R4, PIP4K2B, PIP4K2C, PKM, PLK1, PNPT1, POLA1, POLB, POLD1, POLE, POLR1B, POLR2A, POLR2B, POLR2G, POLR3A, POLR3C, POLR3F, PPM1F, PPP1R9B, PPP2R5A, PRIM1, PRKAA1, PRKAB1, PRKACB, PRKACG, PRKAG1, PRKAR1A, PRKAR2A, PRKAR2B, PRKCA, PRKDC, PRPSAP1, PRRC1, PTK7, PTPN1, PTPN2, PXK, PYCARD, RANBP2, RAP2B, RAP2C, RFC2, RFC3, RFC5, ROCK2, RPS6KA1, RPS6KA3, RPS6KA4, SCYL1, SEPHS1, SESN2, SHPK, SIRT1, SKP2, SLK, SNX6, SNX9, SPAG9, SRC, SRPK1, STK38, TCP1, TELO2, TF, THBS1, TIGAR, TJP2, TKFC, TLK1, TPX2, TRIM28, TRPT1, TTK, TUT1,	No change

					TWF1, UAP1, UGP2, VLDLR, VPS25, VRK1, WARS1, WRAP53, XRCC5, XRCC6, YRDC]	
cellular nitrogen compound metabolic process	GO_BiologicalProcess-EBI-UniProt-GOA-ACAP-ARAP_13.05.2021_00h00	1.49E-64	10.2491	683	[AAAS, AASS, AATF, ABAT, ABCB10, ABCB7, ABCC1, ABCF1, ABHD14B, ACACA, ACAT1, ACIN1, ACLY, ACO1, ACOT13, ACOT9, ACSL1, ACSL4, ACSS2, ACTN1, ACTN4, ACTR2, ADAM10, ADAR, ADARB1, ADPGK, ADSL, AEBP2, AGO1, AGO2, AIMP2, AK1, AK4, AKT1, ALDH1L2, ALDH7A1, ALDOC, AMDHD2, AMPD2, ANKRD28, ANTXR1, ANXA3, ANXA4, AP3B1, APEH, APOBEC3C, APOE, APRT, ARF4, ARHGEF11, ARL6IP5, ASL, ASNS, ATF7IP, ATIC, ATP2B4, ATP6V1A, ATR, AURKB, BAG3, BAZ1B, BCCIP, BDH2, BIN1, BLVRB, BMS1, BOP1, BPNT2, BPTF, BRD3, BRD4, BRMS1, BRWD1, C1QBP, CALR, CAMK1, CAMK2D, CAPRIN1, CARHSP1, CARNMT1, CASK, CASP3, CAT, CAV1, CAVIN1, CBX2, CBX5, CCAR1, CCAR2, CCDC22, CCNB1, CCNH, CCNL2, CCT2, CCT3, CCT4, CCT5, CCT6A, CCT7, CCT8, CD81, CDC123, CDC73, CDK5RAP1, CDK5RAP3, CDK7, CEBPZ, CELF1, CHAF1A, CHD1, CHD4, CHEK1, CHEK2, CHTF18, CIRBP, CMPK1, CNDP2, CNOT11, COL1A1, COL4A2, COPS2, COPS5, COPS7A, CPD, CPOX, CPSF2, CPSF3, CPZ, CRLF3, CRMP1, CSDE1, CSTF1, CSTF3, CTBP2, CTCF, CTNNB1, CTNNBL1, CTPS1, CUL4A, CUL4B, CWC27, CYB5R3, CYFIP1, DAPK1, DARS2, DAXX, DAZAP1, DCAF1, DCAF13, DCTD, DDRGK1, DDX10, DDX18, DDX20, DDX21, DDX24, DDX39B, DDX41, DDX42, DDX46, DDX47, DDX49, DDX5, DDX52, DDX54, DDX6, DEGS1, DHPS, DHX15, DHX33, DHX37, DHX8, DHX9, DICER1, DLG1, DLGAP5, DNMT1, DNMT3A, DNMT3B, DPH1, DPH2, DPH6, DPYSL2, DPYSL3, DRG1, ECE1, EDRF1, EEF1E1, EEF2, EFL1, EFTUD2, EGFR, EHMT1, EIF3A,	Upregulation

					EIF4A3, EIF5B, ELAC2, ELP1, ELP3, EMSY, ENDOD1, ENO2, EPCAM, ERAP1, ERBIN, ERCC2, ESF1, ETHE1, EXD2, EXOG, EXOSC10, EXOSC2, EXOSC5, EXOSC7, FANCD2, FANCI, FASTKD2, FECH, FH, FLAD1, FOXK1, FSCN1, FTSJ3, FXR1, G3BP1, G3BP2, GALK1, GARS1, GART, GATA6, GATAD2A, GCDH, GCLC, GCLM, GDAP1, GEMIN4, GEMIN5, GFPT2, GINS3, GLA, GMPPB, GMPS, GNE, GNL3, GNPDA2, GPI, GSS, GSTK1, GSTM2, GSTM3, GTF2E1, GTF2I, GTF3C1, GTF3C2, GTF3C3, GTF3C4, GTPBP1, GUF1, HAGH, HAT1, HCFC1, HDAC2, HDAC4, HEATR1, HELLS, HERC2, HEXA, HK1, HK2, HMBS, HMGCL, HMOX1, HMOX2, HNRNPF, HNRNPL, HNRNPLL, HNRNPM, HOOK3, HP1BP3, HSD17B10, HSD17B4, HSP90AB1, HSPA5, HSPB1, HSPD1, HUWE1, IDH2, IGF2BP3, ILF2, ILF3, ILK, IMP3, IMPDH1, IMPDH2, INO80C, IRF2BPL, IRF3, ITCH, ITGA6, IWS1, JARID2, KANK2, KAT7, KDM1A, KDM2A, KDM3B, KEAP1, KIF22, LANCL2, LARS1, LARS2, LAS1L, LIG1, LIG3, LIMS1, LIN28A, LMCD1, LNPEP, LRPPRC, LRWD1, LSM4, LYAR, MACROH2A1, MAGED1, MAP1S, MAPK1, MAPK14, MAPK8, MARS1, MAVS, MBD3, MBNL1, MCCC2, MCM2, MCM3, MCM4, MCM5, MCM6, MCM7, MDC1, MDN1, ME1, METAP1, METTL1, MIPEP, MLH1, MMAB, MME, MOXD1, MPC2, MPHOSPH10, MRPS27, MRPS9, MSH2, MSH3, MSH6, MTA3, MTAP, MTDH, MTHFD1, MTREX, MVD, MVK, MYBBP1A, MYD88, MYDGF, MYEF2, MYO1C, NBAS, NCAPG2, NCL, NDC1, NDRG1, NEDD4, NEK7, NFKB1, NIBAN1, NIBAN2, NIF3L1, NLN, NOL10, NOL11, NOL6, NOL9, NOP14, NOP56, NOP58, NOP9, NPM1, NPM3, NSUN2, NSUN5,	

					NT5C2, NTHL1, NUDT16, NUP107, NUP133, NUP160, NUP210, NUP35, NUP50, NVL, OGT, OPLAH, ORC2, ORC3, ORC4, ORC5, PABPN1, PAICS, PAPSS2, PARD3, PARN, PARP1, PASK, PATZ1, PAWR, PAXBP1, PC, PCBP2, PCCA, PCCB, PDCD11, PDGFRB, PDS5A, PDXK, PELP1, PES1, PFAS, PFKL, PFKM, PFKP, PGM3, PHC1, PHIP, PIAS4, PICALM, PIK3CA, PKM, PLD3, PLK1, PLPP3, PLXNB2, PM20D2, PNP, PNPT1, POFUT1, POGZ, POLA1, POLA2, POLB, POLD1, POLE, POLR1B, POLR2A, POLR2B, POLR2G, POLR3A, POLR3C, POLR3F, POR, PPID, PPM1F, PPP1R9B, PRIM1, PRIM2, PRKAA1, PRKAG1, PRKAR1A, PRKCA, PRKCSH, PRKDC, PRORP, PRPF38A, PRPF38B, PRPF40A, PRPF8, PRPSAP1, PSIP1, PSMC6, PSMD1, PSMD2, PSMD9, PSME3, PSPC1, PTBP2, PTCD1, PTCD3, PTGIS, PTPN2, PURA, PUS1, PUS3, PUS7, PWP2, PXDN, PYCARD, QDPR, QRSL1, RAB23, RAD51, RAN, RANBP2, RAP2C, RBM15, RBM25, RBM26, RBM3, RBM6, RBMS2, RBPJ, RBPMS, RELA, RFC1, RFC2, RFC3, RFC5, RIF1, RIOX1, RNH1, ROCK2, RPA1, RPA2, RPA3, RPF2, RPL13A, RPL22, RPL27A, RPL4, RPL5, RPL7A, RPRD1A, RPS2, RPS21, RPS6KA1, RPS6KA3, RPS6KA4, RPS8, RRBP1, RRP12, RRP7A, RRS1, RTCA, SALL2, SAMD4B, SAMHD1, SAP30BP, SARM1, SARS1, SARS2, SART3, SBDS, SEC13, SEH1L, SERBP1, SESN2, SET, SETD7, SETDB1, SF3A3, SF3B1, SIN3A, SIRT1, SIRT2, SLC25A12, SLC25A32, SLC44A1, SLIRP, SLTM, SMARCA4, SMARCA5, SMARCAD1, SMARCC1, SMARCD1, SMARCD2, SMPDL3B, SMS, SNRNP200, SNRPA1, SNRPD3, SNX6, SORBS3, SORD, SPON1, SPOUT1, SPR, SPTLC2, SRC, SRM, SRPK1, SRSF1, SRSF11, SRSF7, SSB, SSRP1,	

					STXBP2, SUPT16H, SUPT5H, SUPT6H, SYMPK, TARS2, TAX1BP1, TBL3, TBPL1, TBRG4, TCP1, TDP1, TDP2, TELO2, TEX10, TF, TFB1M, TGFB1I1, THBS1, THUMPD3, TIA1, TIGAR, TJP2, TMED10, TMED2, TMF1, TOP2A, TPST1, TRAP1, TRIM28, TRIM33, TRIM71, TRIP12, TRIP13, TRMT1, TRMT10C, TRMT1L, TRMT5, TRPT1, TSEN34, TSR1, TTF2, TUT1, TXNRD1, UAP1, UBR5, UFL1, UGDH, UGGT1, UGGT2, UGP2, UNG, UPF2, UPF3B, USP47, USP7, USP9X, UTP15, UTP18, UTP20, UTP25, UTP4, VIM, VRTN, WARS1, WDHD1, WDR18, WDR3, WDR36, WDR43, WRAP53, XPNPEP1, XPO5, XRCC5, XRCC6, XRN1, XRN2, YAP1, YARS2, ZC3H15, ZC3H7B, ZC3HAV1, ZFP36L2, ZMYND8, ZNF217, ZNF281, ZNF462, ZNF638]	
macromolecule metabolic process	GO_BiologicalProcess-EBI-UniProt-GOA-ACAP-ARAP_13.05.2021_00h00	1.49E-64	9.251687	905	[AAAS, AASS, AATF, ABCB10, ABCF1, ABHD10, ABHD12, ABHD14B, ABI1, ACACA, ACIN1, ACO1, ACSL1, ACTN1, ACTN4, ACTR2, ADAM10, ADAR, ADARB1, AEBP2, AGL, AGO1, AGO2, AGTPBP1, AIMP2, AKT1, ALG11, ALG2, ALG5, ALG9, ANK2, ANK3, ANKRD28, ANKZF1, ANTXR1, ANXA1, ANXA2, ANXA3, ANXA4, AP2A1, AP2A2, AP2B1, AP2M1, AP2S1, AP3B1, APAF1, APEH, APOBEC3C, APOE, APPL2, ARAP1, ARF4, ARFGEF1, ARHGEF11, ARL6IP5, ARSB, ASPH, ATF7IP, ATP2B1, ATP2B4, ATR, AUP1, AURKA, AURKB, B3GLCT, B4GALT1, BAG2, BAG3, BAZ1B, BCCIP, BIN1, BIRC6, BMS1, BOP1, BPNT2, BPTF, BRAT1, BRD3, BRD4, BRMS1, BRWD1, BUB1B, C1QBP, CACYBP, CALR, CALU, CAMK1, CAMK2D, CAPN1, CAPN2, CAPNS1, CAPRIN1, CARHSP1, CASK, CASP3, CAST, CAT, CAV1, CAVIN1, CBL, CBX2, CBX5, CCAR1, CCAR2, CCDC22, CCNB1,	No change

					CCND2, CCNH, CCNL2,<br>CCNY, CCT2, CCT3,<br>CCT4, CCT5, CCT6A,<br>CCT7, CCT8, CD2AP,<br>CD44, CD81, CDC123,<br>CDC42, CDC42BPA,<br>CDC42BPB, CDC73,<br>CDH2, CDH3,<br>CDK5RAP1, CDK5RAP3,<br>CDK7, CEBPZ, CELF1,<br>CFAP20, CHAF1A,<br>CHD1, CHD4, CHEK1,<br>CHEK2, CHID1, CHPF,<br>CHST14, CHTF18,<br>CIRBP, CKAP4, CLASP1,<br>CNDP2, CNOT11,<br>CNRIP1, COG7, COL1A1,<br>COL4A2, COL5A1,<br>COL6A3, COPS2, COPS5,<br>COPS7A, CORO1C, CPD,<br>CPOX, CPSF2, CPSF3,<br>CPZ, CRLF3, CRTAP,<br>CSDE1, CSPG4, CSTF1,<br>CSTF3, CTBP2, CTCF,<br>CTNNB1, CTNNBL1,<br>CTSC, CUL4A, CUL4B,<br>CUL7, CWC27, CYFIP1,<br>CYP51A1, DAPK1,<br>DARS2, DAXX, DAZAP1,<br>DBNL, DCAF1, DCAF13,<br>DDRGK1, DDX10,<br>DDX18, DDX20, DDX21,<br>DDX24, DDX39B,<br>DDX41, DDX42, DDX46,<br>DDX47, DDX49, DDX5,<br>DDX52, DDX54, DDX6,<br>DHPS, DHX15, DHX33,<br>DHX37, DHX8, DHX9,<br>DICER1, DIPK2A, DLG1,<br>DLGAP5, DNMT1,<br>DNMT3A, DNMT3B,<br>DOCK7, DPH1, DPH2,<br>DPH6, DPP3, DRG1,<br>DSP, DUSP3, ECE1,<br>ECPAS, ECT2, EDEM3,<br>EDRF1, EEF1E1, EEF2,<br>EFL1, EFTUD2, EGFR,<br>EHD4, EHMT1, EIF3A,<br>EIF4A3, EIF5B, ELAC2,<br>ELP1, ELP3, EMSY,<br>ENDOD1, EPB41L5,<br>EPCAM, EPHB3,<br>EPM2AIP1, ERAP1,<br>ERBIN, ERCC2, ERLEC1,<br>ERLIN2, ERO1A, ERP44,<br>ESF1, ETFA, EXD2,<br>EXOG, EXOSC10,<br>EXOSC2, EXOSC5,<br>EXOSC7, F11R, FAF2,<br>FANCD2, FANCI,<br>FASTKD2, FBXO2,<br>FBXO30, FBXW8, FDXR,<br>FERMT2, FGG, FH,<br>FKBP10, FKBP11,<br>FKBP14, FKBP15,<br>FKBP4, FKBP5, FKBP7,<br>FKBP8, FKBP9, FLT1,<br>FN1, FOXK1, FSCN1,<br>FTSJ3, FUT11, FXR1,<br>G3BP1, G3BP2,<br>GALNT1, GALNT10,<br>GALNT2, GARS1,<br>GATA6, GATAD2A,<br>GBE1, GCLC, GEMIN4,<br>GEMIN5, GET4, GFPT2,	

					GINS3, GIPC1, GLMN, GLUL, GNL3, GNS, GOLGA2, GPC1, GPC3, GPC6, GPD1L, GPHN, GPI, GSN, GTF2E1, GTF2I, GTF3C1, GTF3C2, GTF3C3, GTF3C4, GTPBP1, GUF1, GXYLT1, GYS1, HAT1, HCFC1, HDAC2, HDAC4, HEATR1, HELLS, HERC2, HEXA, HK1, HLA-A, HMBS, HMMR, HMOX1, HNRNPF, HNRNPL, HNRNPLL, HNRNPM, HP1BP3, HSD17B10, HSD17B4, HSP90AB1, HSP90B1, HSPA2, HSPA5, HSPB1, HSPD1, HSPE1, HSPH1, HTRA1, HTT, HUWE1, IGF2BP3, ILF2, ILF3, ILK, IMP3, INCENP, INO80C, IPO5, IQGAP1, IQGAP3, IRF2BPL, IRF3, IST1, ITCH, ITGA5, ITGA6, ITGAV, ITGB1, IWS1, JADE1, JARID2, KANK2, KAT7, KDM1A, KDM2A, KDM3B, KEAP1, KIF22, KTN1, L2HGDH, LAMB1, LAMC1, LANCL2, LARS1, LARS2, LAS1L, LCMT1, LGALS1, LIG1, LIG3, LIMS1, LIN28A, LMAN1, LMCD1, LMF2, LNPEP, LPCAT1, LRP1, LRPPRC, LRRC40, LRRK1, LRWD1, LSM4, LTBP1, LYAR, LYPLA2, MACROH2A1, MAGED1, MAN1A1, MAN1A2, MANBA, MAP1S, MAP2K6, MAPK1, MAPK14, MAPK8, MARCHF5, MARS1, MASTL, MATR3, MAVS, MBD3, MBNL1, MCM2, MCM3, MCM4, MCM5, MCM6, MCM7, MCMBP, MDC1, MDN1, METAP1, METTL1, MFGE8, MGAT2, MICAL1, MIPEP, MLH1, MMAB, MME, MOCS2, MOGS, MPHOSPH10, MRPS27, MRPS9, MSH2, MSH3, MSH6, MSN, MTA3, MTDH, MTMR14, MTMR6, MTREX, MVB12A, MVD, MYADM, MYBBP1A, MYD88, MYDGF, MYEF2, MYH9, MYO1C, MYO1E, NBAS, NCAPG2, NCL, NDC1, NDRG1, NDRG2, NEDD4, NEK7, NFKB1, NIBAN1, NIBAN2, NIF3L1, NLE1, NLN, NMT2, NNT, NOL10, NOL11, NOL6, NOL9, NOP14, NOP56, NOP58, NOP9, NPM1, NPM3, NRP2, NSF, NSFL1C,	

					NSUN2, NSUN5, NTHL1, NUDT16, NUP107, NUP133, NUP160, NUP210, NUP35, NUP50, NVL, NXN, OCLN, OGT, OPTN, ORC2, ORC3, ORC4, ORC5, OSBP, OSTC, OTUD6B, P3H1, P3H3, P3H4, P4HA1, P4HA2, P4HB, P4HTM, PABPN1, PAK1, PALD1, PARD3, PARN, PARP1, PARVA, PASK, PATZ1, PAWR, PAXBP1, PC, PCBP2, PCOLCE, PDCD11, PDCD2, PDCL3, PDGFRB, PDIA3, PDIA6, PDS5A, PDZRN3, PEA15, PELP1, PES1, PFKM, PGAM5, PGM2L1, PGM3, PHC1, PHIP, PHPT1, PIAS4, PICALM, PIGS, PIGT, PIK3CA, PIK3R4, PITRM1, PKM, PLD3, PLK1, PLOD1, PLOD2, PLOD3, PLPP3, PLXNB2, PM20D2, PNP, PNPT1, POFUT1, POFUT2, POGLUT2, POGLUT3, POGZ, POLA1, POLA2, POLB, POLD1, POLE, POLR1B, POLR2A, POLR2B, POLR2G, POLR3A, POLR3C, POLR3F, POR, PPIB, PPIC, PPID, PPM1B, PPM1F, PPM1G, PPP1R7, PPP1R9B, PPP2R5A, PPP4R1, PRDX4, PREPL, PRIM1, PRIM2, PRKAA1, PRKAB1, PRKACB, PRKACG, PRKAG1, PRKAR1A, PRKAR2A, PRKAR2B, PRKCA, PRKCSH, PRKDC, PRMT3, PRORP, PRPF38A, PRPF38B, PRPF40A, PRPF8, PRRC1, PSIP1, PSMC6, PSMD1, PSMD2, PSMD9, PSME3, PSPC1, PTBP2, PTCD1, PTCD3, PTGIS, PTK7, PTPMT1, PTPN1, PTPN12, PTPN2, PTPRD, PURA, PUS1, PUS3, PUS7, PWP2, PXDN, PXK, PYCARD, QRSL1, RAB1A, RAB2A, RAB8A, RAD51, RAN, RANBP2, RANGAP1, RAP2A, RAP2B, RAP2C, RBBP5, RBM15, RBM25, RBM26, RBM3, RBM6, RBMS2, RBPJ, RBPMS, RCN1, RDX, RELA, RFC1, RFC2, RFC3, RFC5, RFTN1, RIC1, RIF1, RIMKLB, RIOX1, RNF170, RNH1, RNPEP, ROCK2, RPA1, RPA2, RPA3, RPF2, RPL13A, RPL22, RPL27A, RPL4, RPL5, RPL7A, RPN1,	

					RPRD1A, RPS2, RPS21, RPS6KA1, RPS6KA3, RPS6KA4, RPS8, RRBP1, RRP12, RRP7A, RRS1, RTCA, S100A11, SALL2, SAMD4B, SAMHD1, SAP30BP, SARS1, SARS2, SART3, SBDS, SBF1, SCARB2, SCPEP1, SCRN1, SCRN3, SCYL1, SDF2L1, SEC13, SEC22B, SEH1L, SEL1L, SEPHS1, SERBP1, SERPINB6, SERPINB9, SERPINH1, SESN2, SET, SETD7, SETDB1, SF3A3, SF3B1, SH3GLB1, SIN3A, SIRT1, SIRT2, SKP2, SLC2A10, SLIRP, SLK, SLTM, SMARCA4, SMARCA5, SMARCAD1, SMARCC1, SMARCD1, SMARCD2, SNRNP200, SNRPA1, SNRPD3, SNX3, SNX6, SNX9, SORBS1, SORBS3, SORD, SPAG9, SPON1, SPOUT1, SPTBN1, SRC, SRPK1, SRSF1, SRSF11, SRSF7, SSB, SSBP1, SSRP1, STAM2, STK38, STT3A, STT3B, STX5, STXBP2, STYX, SUMF2, SUPT16H, SUPT5H, SUPT6H, SYMPK, TARS2, TAX1BP1, TBL3, TBPL1, TBRG4, TCP1, TDP1, TDP2, TELO2, TEX10, TF, TFB1M, TGFB1I1, TGM2, THBS1, THUMPD3, TIA1, TIGAR, TIMM50, TIMP3, TIPRL, TLK1, TMED10, TMED2, TMF1, TMX3, TOLLIP, TOP2A, TPP2, TPST1, TPX2, TRAP1, TRIM28, TRIM33, TRIM71, TRIP12, TRIP13, TRMT1, TRMT10C, TRMT1L, TRMT5, TRPT1, TSEN34, TSR1, TTF2, TTK, TTLL12, TUT1, TWF1, UBA6, UBE2G1, UBE2H, UBE2O, UBR4, UBR5, UFL1, UGDH, UGGT1, UGGT2, UGP2, UNG, UPF2, UPF3B, USE1, USP15, USP19, USP47, USP5, USP7, USP9X, UTP15, UTP18, UTP20, UTP25, UTP4, VAMP3, VIM, VLDLR, VPS25, VPS35, VPS36, VRK1, VRTN, WAPL, WARS1, WDHD1, WDR18, WDR3, WDR36, WDR43, WRAP53, XPNPEP1, XPO5, XRCC5, XRCC6, XRN1, XRN2, YAP1, YARS2, YOD1, ZC3H15, ZC3H7B, ZC3HAV1, ZDHHC17, ZFP36L2, ZMYND8, ZNF217, ZNF281, ZNF462, ZNF638]	

positive regulation of molecular function	GO_BiologicalProcess-EBI-UniProt-GOA-ACAP-ARAP_13.05.2021_00h00	1.49E-64	10.34483	177	[ABI1, ACSL1, ACTN4, ADD2, AIMP2, AKT1, ANK2, ANK3, ANTXR1, ANXA2, ANXA3, APAF1, APOE, ARAP1, ARAP3, ARF4, ARL1, ARL6IP5, ASAP1, ASPH, ATP2B4, AURKB, CAMK1, CAT, CAV1, CCNB1, CCND2, CCNY, CCT2, CCT4, CD44, CD81, CDC42, CDH3, CDK5RAP1, CHTF18, COPS5, CORO1C, CSPG4, CTBP2, CTNNB1, CTSC, DAPK1, DAXX, DBNL, DDRGK1, DHX33, DHX9, DIPK2A, DLG1, DOCK7, ECT2, EGFR, EPB41, EPB41L5, EPHB3, EPM2AIP1, ERCC2, ETFA, F11R, FERMT2, FLT1, FN1, GCLM, GOLGA2, GPSM1, GSN, GSTM2, HDAC2, HDAC4, HSP90AB1, HSPA2, HSPD1, HSPE1, HTT, ILK, INCENP, IQGAP1, IQGAP3, ITGA6, ITGB1, KDM1A, KIF5B, LARS1, LIMS1, LRP1, MAGED1, MAP2K6, MAPK1, MAPK14, MAPK8, MAVS, MSH2, MSH3, MSH6, MTDH, MYD88, NEK7, NFKB1, NIBAN2, NPM1, NVL, ORC3, PABPN1, PAK1, PARN, PARP1, PCOLCE, PDCD2, PDGFRB, PEA15, PICALM, PIK3CA, PIK3R4, PLCG1, PLK1, PLPP3, POR, PPM1F, PRKAA1, PRKACB, PRKACG, PRKAG1, PRKAR1A, PRKAR2A, PRKAR2B, PRKCA, PRPF38B, PRRC1, PSME3, PTPN1, PYCARD, RAB1A, RABGAP1L, RAN, RANBP2, RANGAP1, RAP1GDS1, RCC2, RELA, RFC2, RFC3, RFC5, RIC1, ROCK2, RPS2, RPS6KA4, S100A10, SIRT1, SIRT2, SLC5A3, SMARCA4, SNX9, SORD, SPAG9, SPON1, SRC, SSBP1, STIM1, TBC1D17, TBC1D22B, TBC1D4, TCAF1, TCP1, TELO2, TF, TGM2, THBS1, TIGAR, TPM1, TPX2, TRIM28, VLDLR, WRAP53, XRCC5, XRCC6, ZC3H15]	No change
cellular biosynthetic process	GO_BiologicalProcess-EBI-UniProt-GOA-ACAP-ARAP_13.0	1.49E-64	9.584715	607	[AASS, AATF, ABAT, ABCB10, ABCB7, ABCF1, ABHD14B, ACACA, ACAT1, ACLY, ACO1, ACSL1, ACSL4, ACSS2, ACSS3, ACTN1, ACTN4, ACTR2, ADSL, AEBP2, AGL, AGO1, AGO2,	Upregulation

	5.2021_00 h00				AIMP2, AK1, AK4, AKT1, ALDH7A1, ALG11, ALG2, ALG5, ALG9, AMDHD2, AMPD2, ANKRD28, ANTXR1, ANXA1, ANXA3, ANXA4, AP3B1, APEH, APOE, APRT, ARF4, ARFGEF1, ARHGEF11, ASL, ASNS, ATF7IP, ATIC, ATP1A1, ATP2B4, ATP6V1A, ATR, AURKB, B3GLCT, B4GALT1, BAG3, BAZ1B, BCAT2, BDH2, BPNT2, BPTF, BRD3, BRD4, BRMS1, BRWD1, C1QBP, CACYBP, CALR, CAMK1, CAMK2D, CAPN2, CAPRIN1, CASK, CAT, CAV1, CAVIN1, CBR1, CBX2, CBX5, CCAR1, CCAR2, CCDC22, CCNB1, CCNH, CCNL2, CCT2, CCT3, CCT4, CCT5, CCT6A, CCT7, CCT8, CD81, CDC123, CDC42, CDC73, CDH3, CDK5RAP1, CDK5RAP3, CDK7, CEBPZ, CELF1, CHAF1A, CHD1, CHD4, CHEK1, CHEK2, CHPF, CHST14, CHTF18, CIRBP, CMPK1, CNDP2, CNOT11, COG7, COL1A1, COL4A2, COPS2, COPS5, CPOX, CPSF2, CPSF3, CRLF3, CSDE1, CSPG4, CSTF1, CSTF3, CTBP2, CTCF, CTNNB1, CTPS1, CYB5R3, CYFIP1, DAGLB, DAPK1, DARS2, DAXX, DCAF1, DCTD, DDRGK1, DDX20, DDX21, DDX39B, DDX5, DDX54, DDX6, DEGS1, DGKA, DHPS, DHRS7B, DHX33, DHX8, DHX9, DICER1, DLG1, DLGAP5, DNMT1, DNMT3A, DNMT3B, DPH1, DPH2, DPH6, DRG1, EDEM3, EDRF1, EEF1E1, EEF2, EFL1, EGFR, EHMT1, EIF3A, EIF4A3, EIF5B, ELP3, EMSY, ENOPH1, EPCAM, EPM2AIP1, ERBIN, ERCC2, ERLIN2, ESD, ETNK1, EXD2, EXOSC10, EXOSC2, EXOSC5, EXOSC7, FASTKD2, FDPS, FDXR, FECH, FLAD1, FLT1, FOXK1, FUT11, FXR1, G3BP2, GALNT1, GALNT10, GALNT2, GARS1, GART, GATA6, GATAD2A, GBE1, GCDH, GCLC, GCLM, GEMIN5, GFPT2, GINS3, GIPC1, GLA, GLS, GLUD1, GLUL, GMPPB, GMPS, GNL3, GNPDA2, GOLGA2, GOLT1B, GOT2, GPD1L, GPHN, GPI, GSS, GSTK1,	

					GSTM2, GSTM3, GTF2E1, GTF2I, GTF3C1, GTF3C2, GTF3C3, GTF3C4, GTPBP1, GUF1, GXYLT1, GYS1, H6PD, HAGH, HAT1, HCFC1, HDAC2, HDAC4, HDHD5, HEATR1, HELLS, HMBS, HMGCL, HMOX1, HP1BP3, HSD17B10, HSD17B4, HSP90AB1, HSPA5, HSPB1, HSPD1, IDH2, IDI1, IGF2BP3, ILF2, ILF3, ILK, ILVBL, IMPDH1, IMPDH2, IRF2BPL, IRF3, ITCH, ITGA6, IWS1, JARID2, KANK2, KAT7, KDM1A, KDM2A, KDM3B, KEAP1, LANCL2, LARS1, LARS2, LIG1, LIG3, LIMS1, LIN28A, LMAN1, LMCD1, LPCAT1, LPGAT1, LRPPRC, LRWD1, LSM4, LSS, LYAR, MACROH2A1, MAGED1, MAN1A1, MAN1A2, MAPK1, MAPK14, MAPK8, MARS1, MAVS, MBD3, MCM2, MCM3, MCM4, MCM5, MCM6, MCM7, MCMBP, ME1, METAP1, MGAT2, MGST1, MLH1, MMAB, MOCS2, MOGS, MOXD1, MPC2, MRPS27, MRPS9, MSH3, MTA3, MTAP, MTDH, MTHFD1, MTMR14, MTMR6, MTR, MVD, MVK, MYBBP1A, MYD88, MYDGF, MYEF2, MYO1C, NCAPG2, NCL, NDRG1, NEDD4, NEK7, NFKB1, NIBAN1, NIBAN2, NIF3L1, NMT2, NOL11, NPM1, NPM3, NSUN5, NT5C2, NTHL1, NUDT16, NUP107, NUP35, NVL, OGT, OPLAH, ORC2, ORC3, ORC4, ORC5, OSBP, OSTC, PABPN1, PAICS, PAPSS2, PARD3, PARN, PARP1, PASK, PATZ1, PAWR, PAXBP1, PCCB, PCYT1A, PCYT2, PDGFRB, PDS5A, PDXK, PELP1, PFAS, PFKM, PGM2L1, PGM3, PHC1, PHIP, PI4KA, PIAS4, PICALM, PIGS, PIGT, PIK3C2A, PIK3CA, PIK3R4, PIP4K2B, PIP4K2C, PKM, PLK1, PLOD1, PLOD2, PLOD3, PLPP3, PLSCR3, PLXNB2, PNP, PNPT1, POFUT1, POFUT2, POGLUT2, POGLUT3, POGZ, POLA1, POLA2, POLB, POLD1, POLE, POLR1B, POLR2A, POLR2B,	

					POLR2G, POLR3A, POLR3C, POLR3F, POR, PPID, PPM1B, PPM1F, PRIM1, PRIM2, PRKAA1, PRKAB1, PRKAG1, PRKAR1A, PRKCSH, PRKDC, PRPSAP1, PSIP1, PSMC6, PSMD1, PSMD2, PSMD9, PSME3, PSPC1, PTCD3, PTGIS, PTPMT1, PTPN2, PURA, PUS7, PXDN, PYCARD, PYCR1, PYCR2, PYCR3, QDPR, QRSL1, RAB14, RAD51, RAP2C, RBM15, RBM3, RBP1, RBPJ, RBPMS, RDH10, RELA, RFC1, RFC2, RFC3, RFC5, RIF1, RIOX1, ROCK2, RPA1, RPA2, RPA3, RPL13A, RPL22, RPL27A, RPL4, RPL5, RPL7A, RPN1, RPRD1A, RPS2, RPS21, RPS6KA1, RPS6KA3, RPS6KA4, RPS8, RRBP1, S100A11, SALL2, SAMD4B, SAMHD1, SAP30BP, SARM1, SARS1, SARS2, SBDS, SBF1, SDF2L1, SEPHS1, SESN2, SET, SETD7, SETDB1, SIN3A, SIRT1, SIRT2, SLC1A3, SLC25A12, SLC2A1, SLC2A10, SLC44A1, SLC44A2, SLTM, SMARCA4, SMARCA5, SMARCC1, SMARCD1, SMARCD2, SMS, SNRPD3, SNX6, SORBS1, SORBS3, SORD, SPR, SPTLC2, SRC, SRM, SRR, SSBP1, SSRP1, STT3A, STT3B, STXBP2, SUPT16H, SUPT5H, SUPT6H, SYMPK, TARS2, TAX1BP1, TBPL1, TCP1, TDP2, TELO2, TF, TFB1M, TGFB1I1, THBS1, TIA1, TJP2, TKT, TMED2, TMF1, TOP2A, TRAP1, TRIM28, TRIM33, TRIM71, TRIP13, TRMT10C, TTF2, UAP1, UFL1, UGDH, UGGT1, UGGT2, UGP2, UNG, UPF3B, USP47, USP7, USP9X, UTP15, UTP4, VIM, VRTN, WAPL, WARS1, WDHD1, WDR18, WDR43, WRAP53, XRCC5, XRCC6, XRN1, XRN2, YAP1, YARS2, ZC3H15, ZDHHC17, ZFP36L2, ZMYND8, ZNF217, ZNF281, ZNF462]	
heterocycle metabolic process	GO_BiologicalProcess-EBI-UniProt-GOA-ACAP-	1.49E-64	10.29824	625	[AAAS, AASS, AATF, ABCB10, ABCB7, ABCC1, ABHD14B, ACACA, ACAT1, ACIN1, ACLY, ACOT13, ACOT9, ACSL1, ACSL4, ACSS2,	Upregulation

	ARAP_13.05.2021_00h00				ACTN1, ACTN4, ACTR2, ADAR, ADARB1, ADPGK, ADSL, AEBP2, AGO1, AGO2, AIMP2, AK1, AK4, AKR7A2, AKT1, ALDH1L2, ALDOC, AMDHD2, AMPD2, ANKRD28, ANTXR1, ANXA3, ANXA4, AP3B1, APOBEC3C, APOE, APRT, ARF4, ARHGEF11, ATF7IP, ATIC, ATP2B4, ATP6V1A, ATR, AURKB, BAG3, BAZ1B, BCCIP, BDH2, BLVRB, BMS1, BOP1, BPNT2, BPTF, BRD3, BRD4, BRMS1, BRWD1, C1QBP, CALR, CAMK1, CAMK2D, CARHSP1, CASK, CASP3, CAT, CAV1, CAVIN1, CBX2, CBX5, CCAR1, CCAR2, CCDC22, CCNB1, CCNH, CCNL2, CCT2, CCT3, CCT4, CCT5, CCT6A, CCT7, CCT8, CD81, CDC73, CDK5RAP1, CDK5RAP3, CDK7, CEBPZ, CELF1, CHAF1A, CHD1, CHD4, CHEK1, CHEK2, CHTF18, CIRBP, CMPK1, CNOT11, COL1A1, COL4A2, COPS2, COPS5, COPS7A, CPOX, CPSF2, CPSF3, CRLF3, CRMP1, CSDE1, CSTF1, CSTF3, CTBP2, CTCF, CTNNB1, CTNNBL1, CTPS1, CUL4A, CUL4B, CWC27, CYFIP1, DARS2, DAXX, DAZAP1, DCAF1, DCAF13, DCTD, DDRGK1, DDX10, DDX18, DDX20, DDX21, DDX24, DDX39B, DDX41, DDX42, DDX46, DDX47, DDX49, DDX5, DDX52, DDX54, DDX6, DHX15, DHX33, DHX37, DHX8, DHX9, DICER1, DLG1, DLGAP5, DNMT1, DNMT3A, DNMT3B, DPYSL2, DPYSL3, DRG1, EDRF1, EEF1E1, EFL1, EFTUD2, EGFR, EHMT1, EIF4A3, ELAC2, ELP1, ELP3, EMSY, ENDOD1, ENO2, EPCAM, ERBIN, ERCC2, ESF1, EXD2, EXOG, EXOSC10, EXOSC2, EXOSC5, EXOSC7, FANCD2, FANCI, FASTKD2, FECH, FH, FLAD1, FOXK1, FSCN1, FTSJ3, FXR1, G3BP1, G3BP2, GALK1, GARS1, GART, GATA6, GATAD2A, GCDH, GCLC, GEMIN4, GEMIN5, GFPT2, GINS3, GMPPB, GMPS, GNE, GNL3, GNPDA2, GPHN, GPI, GTF2E1, GTF2I, GTF3C1, GTF3C2, GTF3C3,	

					GTF3C4, GTPBP1, HAT1,<br>HCFC1, HDAC2, HDAC4,<br>HEATR1, HELLS, HERC2,<br>HK1, HK2, HMBS,<br>HMGCL, HMOX1,<br>HMOX2, HNRNPF,<br>HNRNPL, HNRNPLL,<br>HNRNPM, HOOK3,<br>HP1BP3, HSD17B10,<br>HSD17B4, HSP90AB1,<br>HSPA5, HSPB1, HSPD1,<br>HUWE1, IDH2, IGF2BP3,<br>ILF2, ILF3, ILK, IMP3,<br>IMPDH1, IMPDH2,<br>INO80C, IRF2BPL, IRF3,<br>ITCH, ITGA6, IWS1,<br>JARID2, KANK2, KAT7,<br>KDM1A, KDM2A,<br>KDM3B, KEAP1, KIF22,<br>LANCL2, LARS1, LARS2,<br>LAS1L, LIG1, LIG3,<br>LIMS1, LIN28A, LMCD1,<br>LRPPRC, LRWD1, LSM4,<br>LYAR, MACROH2A1,<br>MAGED1, MAP1S,<br>MAPK1, MAPK14,<br>MAPK8, MARS1, MAVS,<br>MBD3, MBNL1, MCCC2,<br>MCM2, MCM3, MCM4,<br>MCM5, MCM6, MCM7,<br>MDC1, MDN1, ME1,<br>METTL1, MGST1, MLH1,<br>MMAB, MME, MOCS2,<br>MPC2, MPHOSPH10,<br>MSH2, MSH3, MSH6,<br>MTA3, MTAP, MTDH,<br>MTHFD1, MTR, MTREX,<br>MVD, MVK, MYBBP1A,<br>MYD88, MYDGF,<br>MYEF2, MYO1C, NBAS,<br>NCAPG2, NCL, NDC1,<br>NDRG1, NEDD4, NEK7,<br>NFKB1, NIBAN2,<br>NIF3L1, NOL10, NOL11,<br>NOL6, NOL9, NOP14,<br>NOP56, NOP58, NOP9,<br>NPM1, NPM3, NSUN2,<br>NSUN5, NT5C2, NTHL1,<br>NUDT16, NUP107,<br>NUP133, NUP160,<br>NUP210, NUP35,<br>NUP50, NVL, OGT,<br>ORC2, ORC3, ORC4,<br>ORC5, PABPN1, PAICS,<br>PAPSS2, PARD3, PARN,<br>PARP1, PATZ1, PAWR,<br>PAXBP1, PC, PCBP2,<br>PCCA, PCCB, PDCD11,<br>PDGFRB, PDS5A, PDXK,<br>PELP1, PES1, PFAS,<br>PFKL, PFKM, PFKP,<br>PGM3, PHC1, PHIP,<br>PIAS4, PICALM, PIK3CA,<br>PKM, PLD3, PLK1,<br>PLPP3, PM20D2, PNP,<br>PNPT1, POFUT1, POGZ,<br>POLA1, POLA2, POLB,<br>POLD1, POLE, POLR1B,<br>POLR2A, POLR2B,<br>POLR2G, POLR3A,<br>POLR3C, POLR3F, PPID,<br>PPM1F, PPP1R9B,<br>PRIM1, PRIM2, PRKAA1,<br>PRKAG1, PRKAR1A,<br>PRKCA, PRKDC, PRORP,	

					PRPF38A, PRPF38B, PRPF40A, PRPF8, PRPSAP1, PSIP1, PSMC6, PSMD1, PSMD2, PSMD9, PSME3, PSPC1, PTBP2, PTCD1, PTGIS, PTPN2, PURA, PUS1, PUS3, PUS7, PWP2, PXDN, PYCARD, PYCR1, PYCR2, PYCR3, QDPR, QRSL1, RAB23, RAD51, RAN, RANBP2, RAP2C, RBM15, RBM25, RBM26, RBM3, RBM6, RBMS2, RBPJ, RBPMS, RELA, RFC1, RFC2, RFC3, RFC5, RIF1, RIOX1, RNH1, ROCK2, RPA1, RPA2, RPA3, RPF2, RPL13A, RPL22, RPL27A, RPL4, RPL5, RPL7A, RPRD1A, RPS2, RPS21, RPS6KA1, RPS6KA3, RPS6KA4, RPS8, RRP12, RRP7A, RRS1, RTCA, SALL2, SAMD4B, SAMHD1, SAP30BP, SARM1, SARS1, SARS2, SART3, SBDS, SEC13, SEH1L, SERBP1, SESN2, SET, SETD7, SETDB1, SF3A3, SF3B1, SIN3A, SIRT1, SIRT2, SLC25A12, SLC25A32, SLIRP, SLTM, SMARCA4, SMARCA5, SMARCAD1, SMARCC1, SMARCD1, SMARCD2, SNRNP200, SNRPA1, SNRPD3, SNX6, SORBS3, SORD, SPOUT1, SPR, SRC, SRPK1, SRSF1, SRSF11, SRSF7, SSB, SSRP1, STXBP2, SUPT16H, SUPT5H, SUPT6H, SYMPK, TARS2, TAX1BP1, TBL3, TBPL1, TBRG4, TCP1, TDP1, TDP2, TELO2, TEX10, TF, TFB1M, TGFB1I1, THUMPD3, TIA1, TIGAR, TJP2, TMF1, TOP2A, TPST1, TRIM28, TRIM33, TRIM71, TRIP12, TRIP13, TRMT1, TRMT10C, TRMT1L, TRMT5, TRPT1, TSEN34, TSR1, TTF2, TUT1, TXNRD1, UAP1, UBR5, UFL1, UGDH, UGGT1, UGGT2, UGP2, UNG, UPF2, UPF3B, USP47, USP7, USP9X, UTP15, UTP18, UTP20, UTP25, UTP4, VIM, VRTN, WARS1, WDHD1, WDR18, WDR3, WDR36, WDR43, WRAP53, XPO5, XRCC5, XRCC6, XRN1, XRN2, YAP1, YARS2, ZC3H7B, ZC3HAV1, ZFP36L2, ZMYND8, ZNF217,	

					ZNF281, ZNF462, ZNF638]	
negative regulation of cellular process	GO_BiologicalProcess-EBI-UniProt-GOA-ACAP-ARAP_13.05.2021_00h00	1.49E-64	9.269717	476	[AASS, AATF, ABAT, ABCB7, ABI1, ACTN1, ACTN4, ADAM10, ADAR, ADARB1, ADD2, ADD3, AEBP2, AGO1, AGO2, AGTPBP1, AIMP2, AKT1, ANK2, ANK3, ANTXR1, ANXA1, ANXA2, ANXA4, ANXA5, ANXA6, AP2B1, AP2M1, APAF1, API5, APOBEC3C, APOE, APPL2, ARAP1, ARF4, ARF6, ARFGEF1, ARHGAP1, ARHGAP28, ARHGDIA, ARL6IP5, ASNS, ATF7IP, ATP1A1, ATP2B4, ATR, AURKA, AURKB, B4GALT1, BAG1, BAG2, BAG3, BIN1, BIRC6, BRMS1, BUB1B, C1QBP, CACYBP, CALR, CAMK2D, CAPRIN1, CAPZA2, CASK, CASP3, CAST, CASTOR2, CAT, CAV1, CBL, CBX2, CBX5, CCAR1, CCAR2, CCDC22, CCNB1, CCND2, CD2AP, CD44, CDC42, CDC73, CDH2, CDK5RAP1, CDK5RAP3, CELF1, CHEK1, CHEK2, CIRBP, CLASP1, CNN1, CNOT11, CNTFR, COL1A1, COL5A1, COL5A2, COL6A3, COPS2, COPS5, CORO1C, CRLF3, CRMP1, CRTAP, CTBP2, CTCF, CTNNB1, CTSC, CUL4A, CYFIP1, CYP51A1, DAPK1, DAXX, DCAF1, DDRGK1, DDX20, DDX39B, DDX5, DDX54, DDX6, DHX9, DIAPH1, DICER1, DIPK2A, DLG1, DNMT1, DNMT3A, DNMT3B, DPYSL3, DUSP3, EEF1E1, EFNB2, EGFR, EHMT1, EIF3A, EIF4A3, EMD, EPB41L5, EPCAM, EPN2, EPS8, ERBIN, ERLEC1, ERLIN2, ERO1A, EXOSC10, EXOSC2, EXOSC5, EXOSC7, F11R, FANCD2, FBN2, FBXO2, FERMT2, FGG, FHL1, FKBP4, FKBP8, FLT1, FOXK1, FTH1, FXR1, G3BP1, GARS1, GATA6, GATAD2A, GBE1, GCLC, GCLM, GDI1, GIPC1, GLA, GLMN, GOLGA2, GOPC, GPC1, GPC3, GPD1L, GPI, GSDME, GSN, GSTM2, HADHA, HAT1, HCFC1, HDAC2, HDAC4, HELLS, HK2, HLA-A, HMOX1, HOOK3, HSP90AB1,	No change

					HSP90B1, HSPA2, HSPA5, HSPA9, HSPB1, HSPD1, HTRA1, HTT, HUWE1, IDH2, IGF2BP3, ILF3, ILK, IPO5, IRF2BPL, IRF3, ITCH, ITGA5, ITGA6, ITGAV, ITGB1, JADE1, JAM3, JARID2, KANK2, KAT7, KDM1A, KDM2A, KNTC1, KRT18, LANCL2, LGALS1, LIG3, LIMS1, LIN28A, LMAN1, LMCD1, LPCAT1, LRP1, LRPPRC, LRRK1, LSM4, LTBP1, LYAR, MACROH2A1, MAGED1, MAP1B, MAP4, MAPK1, MAPK14, MAPK8, MARCHF5, MASTL, MBD3, MCC, MCM2, MCM7, MDC1, MEAK7, METAP1, MICAL1, MLH1, MMAB, MSH2, MSH3, MSH6, MTA3, MTDH, MYADM, MYBBP1A, MYD88, MYDGF, MYH9, MYO1C, NBAS, NCAPG, NCL, NDC80, NDRG1, NDRG2, NEDD4, NFKB1, NIBAN1, NIBAN2, NIF3L1, NLE1, NLGN4X, NNT, NPM1, NSFL1C, NSUN2, NUDT16, NXN, OCLN, OGT, OPTN, ORC2, P3H1, P3H3, PAK1, PARD3, PARN, PARP1, PARVA, PASK, PATZ1, PAWR, PDCL3, PDGFRB, PDS5A, PEA15, PFKL, PHC1, PHIP, PHPT1, PIAS4, PICALM, PIK3CA, PIP4K2B, PIP4K2C, PKP2, PLCG1, PLK1, PLPP3, PLXNB2, PNPT1, POLR1B, POLR2A, POLR2B, POLR2G, POR, PPID, PPM1B, PPM1F, PPP1R9B, PPP2R5A, PRDX4, PRKAA1, PRKACB, PRKAR1A, PRKAR2A, PRKAR2B, PRKCA, PRKDC, PRMT3, PSMC6, PSMD1, PSMD2, PSMD9, PSME3, PSPC1, PTBP2, PTGIS, PTPN1, PTPN12, PTPN2, PTPRD, PURA, PUS7, PXDN, PXMP2, PYCARD, PYCR1, RAB23, RAD51, RANGAP1, RAP2A, RAP2B, RAP2C, RBM15, RBPJ, RBPMS2, RCC2, RDX, RELA, RFC1, RIC1, RIF1, RIOX1, ROCK2, RPA2, RPL13A, RPL5, RPS6KA1, RPS6KA3, RRAS, S100A11, SALL2, SAMD4B, SAMHD1, SARM1, SARS1, SCFD1, SEC22B, SERPINB6, SERPINB9, SERPINH1, SESN2, SESTD1, SET,	

					SETDB1, SH3GL3, SIN3A, SIRT1, SIRT2, SLC25A12, SLC25A5, SLC2A10, SLIRP, SMARCA4, SMARCA5, SMARCC1, SMPDL3B, SNX3, SNX6, SORBS3, SORD, SPAG9, SPARC, SPON1, SPTAN1, SPTBN1, SPTBN2, SRC, SRSF7, STAM2, STK38, STYX, SUPT5H, SUPT6H, TAX1BP1, TBC1D4, TCAF1, TDP2, TELO2, TGFB1I1, THBS1, TIA1, TIGAR, TIMP3, TIPRL, TKFC, TMED10, TMED2, TMEM115, TMF1, TOP2A, TPM1, TPX2, TRAP1, TRIM28, TRIM33, TRIM71, TRIP12, TRIP13, TTK, TTLL12, TWF1, TXNDC5, UBE2O, UBR5, UFL1, UNG, USP19, USP47, USP7, USP9X, UTP20, VAMP3, VAT1, VIM, VPS25, VPS35, WAPL, WARS1, XRCC5, XRCC6, XRN1, YAP1, YOD1, YTHDC2, ZFP36L2, ZMYND8, ZNF217, ZNF281, ZWILCH]	
regulation of catalytic activity	GO_BiologicalProcess-EBI-UniProt-GOA-ACAP-ARAP_13.05.2021_00h00	1.49E-64	10.08567	259	[ABI1, ACSL1, ADAR, ADARB1, AIMP2, AKT1, ANP32E, ANTXR1, ANXA1, ANXA2, ANXA3, ANXA4, ANXA5, AP3B1, APAF1, APOE, ARAP1, ARAP3, ARF4, ARFGAP1, ARFGAP3, ARFGEF1, ARHGAP1, ARHGAP28, ARHGAP5, ARHGDIA, ARHGEF1, ARHGEF11, ARHGEF12, ARL1, ARL2, ARL6IP5, ASAP1, ASPH, ATP2B4, AUP1, AURKB, BAG1, BAG2, BAG3, BCCIP, BIN1, BIRC6, CAMK1, CAMK2D, CAP1, CAP2, CAPN1, CASP3, CAST, CAV1, CBL, CCAR2, CCNB1, CCND2, CCNH, CCNL2, CCNY, CCT2, CCT4, CD44, CD81, CDC42, CDH3, CDK5RAP1, CDK5RAP3, CDK7, CHTF18, COL6A3, COPS2, CORO1C, CPNE2, CSPG4, CTNNB1, CTSC, DAPK1, DAXX, DBNL, DDRGK1, DHX9, DIPK2A, DLG1, DOCK7, DUSP3, ECT2, EGFR, ELP3, EPHB3, EPM2AIP1, EPS8L2, ETFA, F11R, FARP1, FERMT2, FLT1, FN1, GBF1, GCLM, GDI1, GLA, GLMN, GOLGA2, GPC3, GPI, GPSM1, GSN, HERC2, HSP90AB1, HSPB1, HSPD1, HSPE1, HSPH1,	Downregulation

					HTT, ILK, INCENP, IPO5, IQGAP1, IQGAP2, IQGAP3, ITGA6, ITGB1, LARS1, LIMS1, LRP1, MACROH2A1, MAGED1, MAP2K6, MAPK1, MAPK14, MAPK8, MASTL, MCM2, MCM7, MICAL1, MSH2, MSH3, MSH6, MYO9B, NEK7, NFKB1, NLE1, NPM1, NUCB2, NVL, ORC3, PABPN1, PAK1, PARN, PARP1, PARVA, PCOLCE, PDCD2, PDGFRB, PEA15, PGAM5, PHPT1, PICALM, PIK3CA, PIK3R4, PIP4K2B, PIP4K2C, PLCG1, PLK1, PLXNB2, POR, PPM1F, PPP1R7, PPP1R9B, PPP2R5A, PPP4R1, PRKAA1, PRKAB1, PRKACB, PRKACG, PRKAG1, PRKAR1A, PRKAR2A, PRKAR2B, PRKCA, PRPF38B, PRPSAP1, PRRC1, PSMD1, PSMD2, PSME3, PTPN1, PTPN2, PYCARD, RAB1A, RABGAP1L, RALGPS2, RANBP2, RANGAP1, RAP1GDS1, RAP2B, RAP2C, RCC2, RDX, RFC2, RFC3, RFC5, RIC1, RIC8A, RNH1, ROCK2, RP2, RPL5, RPS2, RPS6KA1, RPS6KA3, RTKN, S100A10, SBF1, SCARB2, SEC23A, SEC23B, SERPINB6, SERPINB9, SERPINH1, SESN2, SET, SIL1, SIRT1, SLC5A3, SMAP2, SNX6, SNX9, SORD, SPAG9, SPR, SRC, SSBP1, STIM1, STK38, STYX, TBC1D17, TBC1D22B, TBC1D4, TCP1, TELO2, TF, TGM2, THBS1, TIGAR, TIMP3, TIPRL, TMED10, TMED2, TPX2, TRPT1, UBE2O, USP47, VLDLR, VPS25, WARS1, WRAP53, XRCC5, XRCC6, ZC3H15]	
organic cyclic compound metabolic process	GO_BiologicalProcess-EBI-UniProt-GOA-ACAP-ARAP_13.05.2021_00h00	1.49E-64	10.23066	652	[AAAS, AASS, AATF, ABAT, ABCB10, ABCB7, ABCC1, ABHD14B, ACAA1, ACACA, ACAT1, ACAT2, ACBD3, ACIN1, ACLY, ACOT13, ACOT9, ACSL1, ACSL4, ACSS2, ACTN1, ACTN4, ACTR2, ADAR, ADARB1, ADPGK, ADSL, AEBP2, AGO1, AGO2, AIMP2, AK1, AK4, AKR7A2, AKT1, ALDH1L2, ALDOC, AMDHD2, AMPD2, ANKRD28, ANTXR1, ANXA3, ANXA4, AP3B1, APOBEC3C, APOE, APRT, ARF4, ARHGEF11,	Upregulation

					ATF7IP, ATIC, ATP1A1, ATP2B4, ATP6V1A, ATR, AURKB, BAG3, BAZ1B, BCCIP, BDH2, BLVRB, BMS1, BOP1, BPNT2, BPTF, BRD3, BRD4, BRMS1, BRWD1, C1QBP, CALR, CAMK1, CAMK2D, CARHSP1, CASK, CASP3, CAT, CAV1, CAVIN1, CBX2, CBX5, CCAR1, CCAR2, CCDC22, CCNB1, CCNH, CCNL2, CCT2, CCT3, CCT4, CCT5, CCT6A, CCT7, CCT8, CD81, CDC73, CDH3, CDK5RAP1, CDK5RAP3, CDK7, CEBPZ, CELF1, CHAF1A, CHD1, CHD4, CHEK1, CHEK2, CHTF18, CIRBP, CMPK1, CNOT11, COL1A1, COL4A2, COPS2, COPS5, COPS7A, CPOX, CPSF2, CPSF3, CRLF3, CRMP1, CSDE1, CSTF1, CSTF3, CTBP2, CTCF, CTNNB1, CTNNBL1, CTPS1, CUL4A, CUL4B, CWC27, CYB5R1, CYB5R3, CYFIP1, CYP51A1, DARS2, DAXX, DAZAP1, DCAF1, DCAF13, DCTD, DDRGK1, DDX10, DDX18, DDX20, DDX21, DDX24, DDX39B, DDX41, DDX42, DDX46, DDX47, DDX49, DDX5, DDX52, DDX54, DDX6, DHX15, DHX33, DHX37, DHX8, DHX9, DICER1, DLG1, DLGAP5, DNMT1, DNMT3A, DNMT3B, DPYSL2, DPYSL3, DRG1, EDRF1, EEF1E1, EFL1, EFTUD2, EGFR, EHMT1, EIF4A3, ELAC2, ELP1, ELP3, EMSY, ENDOD1, ENO2, EPCAM, ERBIN, ERCC2, ERLIN2, ESF1, EXD2, EXOG, EXOSC10, EXOSC2, EXOSC5, EXOSC7, FANCD2, FANCI, FASTKD2, FDPS, FDXR, FECH, FH, FLAD1, FOXK1, FSCN1, FTSJ3, FXR1, G3BP1, G3BP2, GALK1, GARS1, GART, GATA6, GATAD2A, GCDH, GCLC, GEMIN4, GEMIN5, GFPT2, GINS3, GIPC1, GMPPB, GMPS, GNE, GNL3, GNPDA2, GOT2, GPHN, GPI, GSTM2, GSTM3, GTF2E1, GTF2I, GTF3C1, GTF3C2, GTF3C3, GTF3C4, GTPBP1, H6PD, HAT1, HCFC1, HDAC2, HDAC4, HDLBP, HEATR1, HELLS, HERC2, HK1, HK2, HMBS, HMGCL, HMOX1, HMOX2, HNRNPF,	

					HNRNPL, HNRNPLL, HNRNPM, HOOK3, HP1BP3, HSD17B10, HSD17B4, HSP90AB1, HSPA5, HSPB1, HSPD1, HUWE1, IDH2, IDI1, IGF2BP3, ILF2, ILF3, ILK, IMP3, IMPDH1, IMPDH2, INO80C, IRF2BPL, IRF3, ITCH, ITGA6, IWS1, JARID2, KANK2, KAT7, KDM1A, KDM2A, KDM3B, KEAP1, KIF22, LANCL2, LARS1, LARS2, LAS1L, LBR, LIG1, LIG3, LIMS1, LIN28A, LMCD1, LRPPRC, LRWD1, LSM4, LSS, LYAR, MACROH2A1, MAGED1, MAP1S, MAPK1, MAPK14, MAPK8, MARS1, MAVS, MBD3, MBNL1, MCCC2, MCM2, MCM3, MCM4, MCM5, MCM6, MCM7, MDC1, MDN1, ME1, METTL1, MGST1, MLH1, MMAB, MME, MOCS2, MOXD1, MPC2, MPHOSPH10, MSH2, MSH3, MSH6, MTA3, MTAP, MTDH, MTHFD1, MTR, MTREX, MVD, MVK, MYBBP1A, MYD88, MYDGF, MYEF2, MYO1C, NBAS, NCAPG2, NCL, NDC1, NDRG1, NEDD4, NEK7, NFKB1, NIBAN2, NIF3L1, NOL10, NOL11, NOL6, NOL9, NOP14, NOP56, NOP58, NOP9, NPM1, NPM3, NSDHL, NSUN2, NSUN5, NT5C2, NTHL1, NUDT16, NUP107, NUP133, NUP160, NUP210, NUP35, NUP50, NVL, OGT, ORC2, ORC3, ORC4, ORC5, OSBP, PABPN1, PAICS, PAPSS2, PARD3, PARN, PARP1, PATZ1, PAWR, PAXBP1, PC, PCBP2, PCCA, PCCB, PDCD11, PDGFRB, PDS5A, PDXK, PELP1, PES1, PFAS, PFKL, PFKM, PFKP, PGM3, PHC1, PHIP, PIAS4, PICALM, PIK3CA, PKM, PLD3, PLK1, PLPP3, PM20D2, PNP, PNPT1, POFUT1, POGZ, POLA1, POLA2, POLB, POLD1, POLE, POLR1B, POLR2A, POLR2B, POLR2G, POLR3A, POLR3C, POLR3F, POR, PPID, PPM1F, PPP1R9B, PRIM1, PRIM2, PRKAA1, PRKAG1, PRKAR1A, PRKCA, PRKDC, PRORP, PRPF38A, PRPF38B, PRPF40A, PRPF8,	

					PRPSAP1, PSIP1, PSMC6, PSMD1, PSMD2, PSMD9, PSME3, PSPC1, PTBP2, PTCD1, PTGIS, PTPN2, PURA, PUS1, PUS3, PUS7, PWP2, PXDN, PYCARD, PYCR1, PYCR2, PYCR3, QDPR, QRSL1, RAB23, RAD51, RAN, RANBP2, RAP2C, RBM15, RBM25, RBM26, RBM3, RBM6, RBMS2, RBPJ, RBPMS, RELA, RFC1, RFC2, RFC3, RFC5, RIF1, RIOX1, RNH1, ROCK2, RPA1, RPA2, RPA3, RPF2, RPL13A, RPL22, RPL27A, RPL4, RPL5, RPL7A, RPRD1A, RPS2, RPS21, RPS6KA1, RPS6KA3, RPS6KA4, RPS8, RRP12, RRP7A, RRS1, RTCA, SALL2, SAMD4B, SAMHD1, SAP30BP, SARM1, SARS1, SARS2, SART3, SBDS, SEC13, SEH1L, SERBP1, SESN2, SET, SETD7, SETDB1, SF3A3, SF3B1, SIN3A, SIRT1, SIRT2, SLC25A12, SLC25A32, SLIRP, SLTM, SMARCA4, SMARCA5, SMARCAD1, SMARCC1, SMARCD1, SMARCD2, SNRNP200, SNRPA1, SNRPD3, SNX17, SNX6, SORBS3, SORD, SPOUT1, SPR, SRC, SRPK1, SRSF1, SRSF11, SRSF7, SSB, SSRP1, STXBP2, SUPT16H, SUPT5H, SUPT6H, SYMPK, TARS2, TAX1BP1, TBL3, TBPL1, TBRG4, TCP1, TDP1, TDP2, TELO2, TEX10, TF, TFB1M, TGFB1I1, THUMPD3, TIA1, TIGAR, TJP2, TMF1, TOP2A, TPST1, TRIM28, TRIM33, TRIM71, TRIP12, TRIP13, TRMT1, TRMT10C, TRMT1L, TRMT5, TRPT1, TSEN34, TSR1, TTF2, TUT1, TXNRD1, UAP1, UBR5, UFL1, UGDH, UGGT1, UGGT2, UGP2, UNG, UPF2, UPF3B, USP47, USP7, USP9X, UTP15, UTP18, UTP20, UTP25, UTP4, VIM, VLDLR, VRTN, WARS1, WDHD1, WDR18, WDR3, WDR36, WDR43, WRAP53, XPO5, XRCC5, XRCC6, XRN1, XRN2, YAP1, YARS2, ZC3H7B, ZC3HAV1, ZFP36L2, ZMYND8, ZNF217, ZNF281, ZNF462, ZNF638]	

organonitrogen compound metabolic process	GO_BiologicalProcess-EBI-UniProt-GOA-ACAP-ARAP_13.05.2021_00h00	1.49E-64	10.38346	723	[AAAS, AASS, AATF, ABAT, ABCB10, ABCB7, ABCC1, ABCF1, ABHD10, ABHD12, ABHD14B, ABI1, ACACA, ACAD8, ACAT1, ACLY, ACO1, ACOT13, ACOT9, ACSL1, ACSL4, ACSS2, ADAM10, ADAR, ADARB1, ADPGK, ADSL, AGO1, AGO2, AGTPBP1, AIMP2, AK1, AK4, AKT1, ALDH1L2, ALDH7A1, ALDOC, ALG11, ALG2, ALG5, ALG9, AMDHD2, AMPD2, ANKZF1, ANTXR1, ANXA1, ANXA2, AP2A1, AP2A2, AP2B1, AP2M1, AP2S1, AP3B1, APAF1, APEH, APOBEC3C, APOE, APRT, ARF4, ARFGEF1, ARL6IP5, ARSA, ARSB, ASL, ASNS, ASPH, ATIC, ATP2B4, ATP6V1A, ATR, AUP1, AURKA, AURKB, B3GLCT, B4GALT1, BAG2, BAG3, BAZ1B, BCAT2, BCCIP, BDH2, BIN1, BIRC6, BLVRB, BPNT2, BRAT1, BRD4, BRMS1, BUB1B, C1QBP, CALR, CALU, CAMK1, CAMK2D, CAPN1, CAPN2, CAPNS1, CAPRIN1, CARNMT1, CASK, CASP3, CAST, CAT, CAV1, CBL, CCAR2, CCDC22, CCNB1, CCND2, CCNH, CCNL2, CCNY, CD2AP, CD44, CD81, CDC123, CDC42, CDC42BPA, CDC42BPB, CDC73, CDH2, CDK5RAP1, CDK5RAP3, CDK7, CELF1, CFAP20, CHEK1, CHEK2, CHPF, CHST14, CIRBP, CKAP4, CLASP1, CMPK1, CNDP2, CNOT11, COG7, COL6A3, COPS2, COPS5, COPS7A, CORO1C, CPD, CPOX, CPZ, CRMP1, CRTAP, CSDE1, CSPG4, CTCF, CTNNB1, CTPS1, CTSC, CUL4A, CUL4B, CUL7, CWC27, CYFIP1, CYP51A1, DAPK1, DARS2, DAXX, DBNL, DCAF1, DCAF13, DCTD, DDRGK1, DDX39B, DDX6, DEGS1, DHPS, DHX33, DHX9, DIPK2A, DLG1, DNMT1, DNMT3B, DOCK7, DPH1, DPH2, DPH6, DPP3, DPYSL2, DPYSL3, DRG1, DSP, DUSP3, ECE1, ECPAS, ECT2, EDEM3, EEF1E1, EEF2, EFL1, EGFR, EHD4, EHMT1, EIF3A, EIF4A3, EIF5B, ELP3, ENO2, ENOPH1, EPHB3, ERAP1, ERLEC1, ERLIN2,	No change

					ERO1A, ERP44, ESD, ESYT2, ETFA, ETHE1, EXOSC2, EXOSC5, EXOSC7, FAF2, FASTKD2, FBXO2, FBXO30, FBXW8, FDXR, FECH, FERMT2, FGG, FKBP10, FKBP11, FKBP14, FKBP15, FKBP4, FKBP5, FKBP7, FKBP8, FKBP9, FLAD1, FLT1, FN1, FOXK1, FUT11, FXR1, GALK1, GALNT1, GALNT10, GALNT2, GARS1, GART, GATA6, GCDH, GCLC, GCLM, GDAP1, GEMIN5, GET4, GFPT2, GIPC1, GLA, GLMN, GLS, GLUD1, GLUL, GMPS, GNE, GNL3, GNPDA2, GNS, GOLGA2, GOLT1B, GOT2, GPC1, GPC3, GPC6, GPD1L, GPHN, GPI, GSN, GSS, GSTK1, GSTM2, GSTM3, GTF3C4, GTPBP1, GUF1, GXYLT1, HAGH, HAT1, HCFC1, HDAC2, HDAC4, HERC2, HEXA, HIBADH, HK1, HK2, HLA-A, HMBS, HMGCL, HMMR, HMOX1, HMOX2, HOOK3, HSD17B10, HSD17B4, HSP90AB1, HSP90B1, HSPA2, HSPA5, HSPB1, HSPD1, HSPE1, HTRA1, HTT, HUWE1, IDH2, IGF2BP3, ILF3, ILK, ILVBL, IMPDH1, IMPDH2, INCENP, INO80C, IPO5, IQGAP1, IQGAP3, IRF2BPL, IST1, ITCH, ITGA5, ITGAV, IWS1, JADE1, JARID2, KAT7, KDM1A, KDM2A, KDM3B, KEAP1, KTN1, L2HGDH, LAMB1, LAMC1, LARS1, LARS2, LCMT1, LGALS1, LIN28A, LMAN1, LMCD1, LMF2, LNPEP, LPCAT1, LRP1, LRPPRC, LRRC40, LRRK1, LSM4, LTBP1, LYPLA2, MACROH2A1, MAGED1, MAN1A1, MAN1A2, MANBA, MAP2K6, MAPK1, MAPK14, MAPK8, MARCHF5, MARS1, MASTL, MAVS, MBD3, MCCC2, MCM2, METAP1, MFGE8, MGAT2, MGST1, MICAL1, MIPEP, MLH1, MMAB, MME, MOCS2, MOGS, MOXD1, MPC2, MRPS27, MRPS9, MSN, MTA3, MTAP, MTHFD1, MTMR14, MTMR6, MTR, MVB12A, MVD, MVK, MYADM, MYDGF, MYH9, MYO1C, NCL, NDC1, NDRG1, NEDD4,	

					NEK7, NFKB1, NIBAN1, NIT2, NLE1, NLN, NMT2, NNT, NPM1, NRP2, NSF, NSFL1C, NSUN5, NT5C2, NTHL1, NUDT16, NUP107, NUP133, NUP160, NUP210, NUP35, NUP50, NXN, OCLN, OGT, OPLAH, ORC3, OSBP, OSTC, OTUD6B, P3H1, P3H3, P3H4, P4HA1, P4HA2, P4HB, P4HTM, PAICS, PAK1, PALD1, PAPSS2, PARD3, PARN, PARP1, PARVA, PASK, PAWR, PAXBP1, PC, PCBP2, PCCA, PCCB, PCOLCE, PCYT1A, PDCD2, PDGFRB, PDIA3, PDIA6, PDXDC1, PDXK, PDZRN3, PEA15, PFAS, PFKL, PFKM, PFKP, PGAM5, PGM3, PHC1, PHIP, PHPT1, PIAS4, PICALM, PIGS, PIGT, PIK3CA, PIK3R4, PITRM1, PKM, PLK1, PLOD1, PLOD2, PLOD3, PLPP3, PLXNB2, PM20D2, PNP, PNPT1, POFUT1, POFUT2, POGLUT2, POGLUT3, POLB, POLR2G, POR, PPIB, PPIC, PPID, PPM1B, PPM1F, PPM1G, PPP1R7, PPP1R9B, PPP2R5A, PPP4R1, PRDX4, PREPL, PRKAA1, PRKAB1, PRKACB, PRKACG, PRKAG1, PRKAR1A, PRKAR2A, PRKAR2B, PRKCA, PRKCSH, PRKDC, PRMT3, PRPF8, PRPSAP1, PRRC1, PSMC6, PSMD1, PSMD2, PSMD9, PSME3, PTCD3, PTGIS, PTK7, PTPMT1, PTPN1, PTPN12, PTPN2, PTPRD, PURA, PUS7, PXK, PYCARD, PYCR1, PYCR2, PYCR3, QDPR, QRSL1, RAB1A, RAB23, RAB2A, RAB8A, RAD51, RAN, RANBP2, RANGAP1, RAP2A, RAP2B, RAP2C, RBBP5, RBM3, RBPMS, RCN1, RDX, RELA, RIC1, RIF1, RIMKLB, RIOX1, RNF170, RNPEP, ROCK2, RPL13A, RPL22, RPL27A, RPL4, RPL5, RPL7A, RPN1, RPRD1A, RPS2, RPS21, RPS6KA1, RPS6KA3, RPS6KA4, RPS8, RRBP1, SAMD4B, SAMHD1, SARM1, SARS1, SARS2, SART3, SBDS, SBF1, SCLY, SCPEP1, SCRN1, SCRN3, SCYL1, SDF2L1, SEC13, SEC22B, SEH1L, SEL1L, SEPHS1, SERPINB6,	

					SERPINB9, SERPINH1, SESN2, SET, SETD7, SETDB1, SIN3A, SIRT1, SIRT2, SKP2, SLC1A3, SLC25A12, SLC25A32, SLC2A10, SLC44A1, SLC44A2, SLK, SMARCAD1, SMARCC1, SMPDL3B, SMS, SNX3, SNX6, SNX9, SORD, SPAG9, SPON1, SPR, SPTBN1, SPTLC2, SRC, SRM, SRPK1, SRR, STAM2, STK38, STT3A, STT3B, STX5, STYX, SUMF2, SUPT6H, SYMPK, TARS2, TBPL1, TELO2, TEX2, TF, TGFB1I1, TGM2, THBS1, TIA1, TIGAR, TIMM50, TIMP3, TIPRL, TJP2, TLK1, TMED10, TMED2, TMF1, TMX3, TOLLIP, TPP2, TPST1, TPX2, TRAP1, TRIM28, TRIM33, TRIM71, TRIP12, TRMT10C, TRPT1, TTK, TTLL12, TWF1, UBA6, UBE2G1, UBE2H, UBE2O, UBR4, UBR5, UFL1, UGDH, UGGT1, UGGT2, UNG, UPF3B, USE1, USP15, USP19, USP47, USP5, USP7, USP9X, UTP25, VIM, VLDLR, VPS25, VPS35, VPS36, VRK1, WARS1, XPNPEP1, XRCC5, XRCC6, XRN1, YARS2, YOD1, ZC3H15, ZC3HAV1, ZDHHC17, ZFP36L2]	
organic substance biosynthetic process	GO_BiologicalProcess-EBI-UniProt-GOA-ACAP-ARAP_13.05.2021_00h00	1.49E-64	9.769757	628	[AASS, AATF, ABAT, ABCB10, ABCB7, ABCF1, ABHD14B, ACACA, ACAT1, ACAT2, ACBD3, ACLY, ACO1, ACSL1, ACSL4, ACSS2, ACSS3, ACTN1, ACTN4, ACTR2, ADSL, AEBP2, AGL, AGO1, AGO2, AIMP2, AK1, AK4, AKT1, ALDH7A1, ALDOC, ALG11, ALG2, ALG5, ALG9, AMDHD2, AMPD2, ANKRD28, ANTXR1, ANXA1, ANXA3, ANXA4, AP2A1, AP3B1, APEH, APOE, APRT, ARF4, ARFGEF1, ARHGEF11, ASL, ASNS, ATF7IP, ATIC, ATP1A1, ATP2B4, ATP6V1A, ATR, AURKB, B3GLCT, B4GALT1, BAG3, BAZ1B, BCAT2, BDH2, BPNT2, BPTF, BRD3, BRD4, BRMS1, BRWD1, C1QBP, CACYBP, CALR, CAMK1, CAMK2D, CAPN2, CAPRIN1, CASK, CAT, CAV1, CAVIN1, CBR1, CBX2, CBX5, CCAR1, CCAR2, CCDC22, CCNB1, CCNH,	No change

					CCNL2, CCT2, CCT3, CCT4, CCT5, CCT6A, CCT7, CCT8, CD81, CDC123, CDC42, CDC73, CDH3, CDK5RAP1, CDK5RAP3, CDK7, CEBPZ, CELF1, CHAF1A, CHD1, CHD4, CHEK1, CHEK2, CHPF, CHST14, CHTF18, CIRBP, CMPK1, CNDP2, CNOT11, COG7, COL1A1, COL4A2, COL5A1, COPS2, COPS5, CPOX, CPSF2, CPSF3, CRLF3, CSDE1, CSPG4, CSTF1, CSTF3, CTBP2, CTCF, CTNNB1, CTPS1, CYB5R1, CYB5R3, CYFIP1, CYP51A1, DAGLB, DAPK1, DARS2, DAXX, DCAF1, DCTD, DCXR, DDRGK1, DDX20, DDX21, DDX39B, DDX5, DDX54, DDX6, DEGS1, DGKA, DHPS, DHRS7B, DHX33, DHX8, DHX9, DICER1, DLG1, DLGAP5, DNMT1, DNMT3A, DNMT3B, DPH1, DPH2, DPH6, DRG1, EDEM3, EDRF1, EEF1E1, EEF2, EFL1, EGFR, EHMT1, EIF3A, EIF4A3, EIF5B, ELP3, EMSY, ENO2, ENOPH1, EPCAM, EPM2AIP1, ERBIN, ERCC2, ERLIN2, ESD, ETNK1, EXD2, EXOSC10, EXOSC2, EXOSC5, EXOSC7, FASTKD2, FDPS, FDXR, FECH, FLAD1, FLT1, FOXK1, FUT11, FXR1, G3BP2, GALNT1, GALNT10, GALNT2, GARS1, GART, GATA6, GATAD2A, GBE1, GCDH, GCLC, GCLM, GEMIN5, GFPT2, GINS3, GIPC1, GLS, GLUD1, GLUL, GMPPB, GMPS, GNE, GNL3, GNPDA2, GOLGA2, GOLT1B, GOT2, GPC1, GPC3, GPC6, GPD1L, GPHN, GPI, GSS, GSTK1, GSTM2, GSTM3, GTF2E1, GTF2I, GTF3C1, GTF3C2, GTF3C3, GTF3C4, GTPBP1, GUF1, GXYLT1, GYS1, H6PD, HAGH, HAT1, HCFC1, HDAC2, HDAC4, HDHD5, HEATR1, HELLS, HEXA, HMBS, HMGCL, HMOX1, HP1BP3, HSD17B10, HSD17B4, HSP90AB1, HSPA5, HSPB1, HSPD1, HSPH1, IDH2, IDI1, IGF2BP3, ILF2, ILF3, ILK, ILVBL, IMPDH1, IMPDH2, IRF2BPL, IRF3, ITCH, ITGA6, IWS1, JARID2, KANK2, KAT7, KDM1A, KDM2A,	

					KDM3B, KEAP1, LANCL2, LARS1, LARS2, LBR, LIG1, LIG3, LIMS1, LIN28A, LMAN1, LMCD1, LPCAT1, LPGAT1, LRPPRC, LRWD1, LSM4, LSS, LYAR, MACROH2A1, MAGED1, MAN1A1, MAN1A2, MAP2K6, MAPK1, MAPK14, MAPK8, MARS1, MAVS, MBD3, MCM2, MCM3, MCM4, MCM5, MCM6, MCM7, MCMBP, ME1, METAP1, MGAT2, MGST1, MLH1, MMAB, MOCS2, MOGS, MOXD1, MPC2, MRPS27, MRPS9, MSH3, MTA3, MTAP, MTDH, MTHFD1, MTMR14, MTMR6, MTR, MVD, MVK, MYBBP1A, MYD88, MYDGF, MYEF2, MYO1C, NCAPG2, NCL, NDRG1, NEDD4, NEK7, NFKB1, NIBAN1, NIBAN2, NIF3L1, NLN, NMT2, NOL11, NPM1, NPM3, NSDHL, NSUN5, NT5C2, NTHL1, NUDT16, NUP107, NUP35, NVL, OGT, OPLAH, ORC2, ORC3, ORC4, ORC5, OSBP, OSTC, PABPN1, PAICS, PAPSS2, PARD3, PARN, PARP1, PASK, PATZ1, PAWR, PAXBP1, PC, PCCB, PCYT1A, PCYT2, PDGFRB, PDS5A, PDXK, PELP1, PFAS, PFKM, PGM2L1, PGM3, PHC1, PHIP, PI4KA, PIAS4, PICALM, PIGS, PIGT, PIK3C2A, PIK3CA, PIK3R4, PIP4K2B, PIP4K2C, PKM, PLK1, PLOD1, PLOD2, PLOD3, PLPP3, PLSCR3, PLXNB2, PNP, PNPT1, POFUT1, POFUT2, POGLUT2, POGLUT3, POGZ, POLA1, POLA2, POLB, POLD1, POLE, POLR1B, POLR2A, POLR2B, POLR2G, POLR3A, POLR3C, POLR3F, POR, PPID, PPM1B, PPM1F, PRIM1, PRIM2, PRKAA1, PRKAB1, PRKAG1, PRKAR1A, PRKCSH, PRKDC, PRPSAP1, PSIP1, PSMC6, PSMD1, PSMD2, PSMD9, PSME3, PSPC1, PTCD3, PTGIS, PTPMT1, PTPN2, PURA, PUS7, PXDN, PYCARD, PYCR1, PYCR2, PYCR3, QDPR, QRSL1, RAB14, RAD51, RAN, RANBP2, RAP2C, RBM15, RBM3, RBP1,	

					RBPJ, RBPMS, RDH10, RELA, RFC1, RFC2, RFC3, RFC5, RIF1, RIOX1, ROCK2, RPA1, RPA2, RPA3, RPL13A, RPL22, RPL27A, RPL4, RPL5, RPL7A, RPN1, RPRD1A, RPS2, RPS21, RPS6KA1, RPS6KA3, RPS6KA4, RPS8, RRBP1, S100A11, SALL2, SAMD4B, SAMHD1, SAP30BP, SARM1, SARS1, SARS2, SBDS, SBF1, SDF2L1, SEPHS1, SESN2, SET, SETD7, SETDB1, SIN3A, SIRT1, SIRT2, SLC1A3, SLC25A12, SLC2A1, SLC2A10, SLC44A1, SLC44A2, SLTM, SMARCA4, SMARCA5, SMARCC1, SMARCD1, SMARCD2, SMS, SNRPD3, SNX6, SORBS1, SORBS3, SORD, SPR, SPTLC2, SRC, SRM, SRR, SSBP1, SSRP1, STT3A, STT3B, STXBP2, SUPT16H, SUPT5H, SUPT6H, SYMPK, TARS2, TAX1BP1, TBPL1, TCP1, TDP2, TELO2, TF, TFB1M, TGFB1I1, THBS1, TIA1, TJP2, TKT, TMED2, TMF1, TOP2A, TRAP1, TRIM28, TRIM33, TRIM71, TRIP13, TRMT10C, TTF2, UAP1, UFL1, UGDH, UGGT1, UGGT2, UGP2, UNG, UPF3B, USP47, USP7, USP9X, UTP15, UTP4, VIM, VRTN, WAPL, WARS1, WDHD1, WDR18, WDR43, WRAP53, XRCC5, XRCC6, XRN1, XRN2, YAP1, YARS2, ZC3H15, ZDHHC17, ZFP36L2, ZMYND8, ZNF217, ZNF281, ZNF462]	
nucleobase-containing compound metabolic process	GO_BiologicalProcess-EBI-UniProt-GOA-ACAP-ARAP_13.05.2021_00h00	1.49E-64	10.16834	598	[AAAS, AASS, AATF, ABHD14B, ACACA, ACAT1, ACIN1, ACLY, ACOT13, ACOT9, ACSL1, ACSL4, ACSS2, ACTN1, ACTN4, ACTR2, ADAR, ADARB1, ADPGK, ADSL, AEBP2, AGO1, AGO2, AIMP2, AK1, AK4, AKT1, ALDOC, AMDHD2, AMPD2, ANKRD28, ANTXR1, ANXA3, ANXA4, AP3B1, APOBEC3C, APOE, APRT, ARF4, ARHGEF11, ATF7IP, ATIC, ATP2B4, ATP6V1A, ATR, AURKB, BAG3, BAZ1B, BCCIP, BMS1, BOP1, BPNT2, BPTF, BRD3, BRD4, BRMS1, BRWD1, C1QBP, CALR, CAMK1,	Upregulation

					CAMK2D, CARHSP1, CASK, CASP3, CAT, CAV1, CAVIN1, CBX2, CBX5, CCAR1, CCAR2, CCDC22, CCNB1, CCNH, CCNL2, CCT2, CCT3, CCT4, CCT5, CCT6A, CCT7, CCT8, CD81, CDC73, CDK5RAP1, CDK5RAP3, CDK7, CEBPZ, CELF1, CHAF1A, CHD1, CHD4, CHEK1, CHEK2, CHTF18, CIRBP, CMPK1, CNOT11, COL1A1, COL4A2, COPS2, COPS5, COPS7A, CPSF2, CPSF3, CRLF3, CRMP1, CSDE1, CSTF1, CSTF3, CTBP2, CTCF, CTNNB1, CTNNBL1, CTPS1, CUL4A, CUL4B, CWC27, CYFIP1, DARS2, DAXX, DAZAP1, DCAF1, DCAF13, DCTD, DDRGK1, DDX10, DDX18, DDX20, DDX21, DDX24, DDX39B, DDX41, DDX42, DDX46, DDX47, DDX49, DDX5, DDX52, DDX54, DDX6, DHX15, DHX33, DHX37, DHX8, DHX9, DICER1, DLG1, DLGAP5, DNMT1, DNMT3A, DNMT3B, DPYSL2, DPYSL3, DRG1, EDRF1, EEF1E1, EFL1, EFTUD2, EGFR, EHMT1, EIF4A3, ELAC2, ELP1, ELP3, EMSY, ENDOD1, ENO2, EPCAM, ERBIN, ERCC2, ESF1, EXD2, EXOG, EXOSC10, EXOSC2, EXOSC5, EXOSC7, FANCD2, FANCI, FASTKD2, FH, FLAD1, FOXK1, FSCN1, FTSJ3, FXR1, G3BP1, G3BP2, GALK1, GARS1, GART, GATA6, GATAD2A, GCDH, GCLC, GEMIN4, GEMIN5, GFPT2, GINS3, GMPPB, GMPS, GNE, GNL3, GNPDA2, GPI, GTF2E1, GTF2I, GTF3C1, GTF3C2, GTF3C3, GTF3C4, GTPBP1, HAT1, HCFC1, HDAC2, HDAC4, HEATR1, HELLS, HERC2, HK1, HK2, HMGCL, HMOX1, HNRNPF, HNRNPL, HNRNPLL, HNRNPM, HOOK3, HP1BP3, HSD17B10, HSD17B4, HSP90AB1, HSPA5, HSPB1, HSPD1, HUWE1, IDH2, IGF2BP3, ILF2, ILF3, ILK, IMP3, IMPDH1, IMPDH2, INO80C, IRF2BPL, IRF3, ITCH, ITGA6, IWS1, JARID2, KANK2, KAT7, KDM1A, KDM2A, KDM3B, KEAP1, KIF22, LANCL2, LARS1, LARS2,	

					LAS1L, LIG1, LIG3, LIMS1, LIN28A, LMCD1, LRPPRC, LRWD1, LSM4, LYAR, MACROH2A1, MAGED1, MAP1S, MAPK1, MAPK14, MAPK8, MARS1, MAVS, MBD3, MBNL1, MCCC2, MCM2, MCM3, MCM4, MCM5, MCM6, MCM7, MDC1, MDN1, ME1, METTL1, MLH1, MMAB, MPC2, MPHOSPH10, MSH2, MSH3, MSH6, MTA3, MTAP, MTDH, MTHFD1, MTREX, MVD, MVK, MYBBP1A, MYD88, MYDGF, MYEF2, MYO1C, NBAS, NCAPG2, NCL, NDC1, NDRG1, NEDD4, NEK7, NFKB1, NIBAN2, NIF3L1, NOL10, NOL11, NOL6, NOL9, NOP14, NOP56, NOP58, NOP9, NPM1, NPM3, NSUN2, NSUN5, NT5C2, NTHL1, NUDT16, NUP107, NUP133, NUP160, NUP210, NUP35, NUP50, NVL, OGT, ORC2, ORC3, ORC4, ORC5, PABPN1, PAICS, PAPSS2, PARD3, PARN, PARP1, PATZ1, PAWR, PAXBP1, PCBP2, PDCD11, PDGFRB, PDS5A, PELP1, PES1, PFAS, PFKL, PFKM, PFKP, PGM3, PHC1, PHIP, PIAS4, PICALM, PIK3CA, PKM, PLD3, PLK1, PLPP3, PNP, PNPT1, POFUT1, POGZ, POLA1, POLA2, POLB, POLD1, POLE, POLR1B, POLR2A, POLR2B, POLR2G, POLR3A, POLR3C, POLR3F, PPID, PPM1F, PPP1R9B, PRIM1, PRIM2, PRKAA1, PRKAG1, PRKAR1A, PRKCA, PRKDC, PRORP, PRPF38A, PRPF38B, PRPF40A, PRPF8, PRPSAP1, PSIP1, PSMC6, PSMD1, PSMD2, PSMD9, PSME3, PSPC1, PTBP2, PTCD1, PTGIS, PTPN2, PURA, PUS1, PUS3, PUS7, PWP2, PXDN, PYCARD, QRSL1, RAB23, RAD51, RAN, RANBP2, RAP2C, RBM15, RBM25, RBM26, RBM3, RBM6, RBMS2, RBPJ, RBPMS, RELA, RFC1, RFC2, RFC3, RFC5, RIF1, RIOX1, RNH1, ROCK2, RPA1, RPA2, RPA3, RPF2, RPL13A, RPL22, RPL27A, RPL4, RPL5, RPL7A, RPRD1A, RPS2, RPS21, RPS6KA1,	

					RPS6KA3, RPS6KA4, RPS8, RRP12, RRP7A, RRS1, RTCA, SALL2, SAMD4B, SAMHD1, SAP30BP, SARM1, SARS1, SARS2, SART3, SBDS, SEC13, SEH1L, SERBP1, SESN2, SET, SETD7, SETDB1, SF3A3, SF3B1, SIN3A, SIRT1, SIRT2, SLC25A12, SLIRP, SLTM, SMARCA4, SMARCA5, SMARCAD1, SMARCC1, SMARCD1, SMARCD2, SNRNP200, SNRPA1, SNRPD3, SNX6, SORBS3, SORD, SPOUT1, SRC, SRPK1, SRSF1, SRSF11, SRSF7, SSB, SSRP1, STXBP2, SUPT16H, SUPT5H, SUPT6H, SYMPK, TARS2, TAX1BP1, TBL3, TBPL1, TBRG4, TCP1, TDP1, TDP2, TELO2, TEX10, TF, TFB1M, TGFB1I1, THUMPD3, TIA1, TIGAR, TJP2, TMF1, TOP2A, TPST1, TRIM28, TRIM33, TRIM71, TRIP12, TRIP13, TRMT1, TRMT10C, TRMT1L, TRMT5, TRPT1, TSEN34, TSR1, TTF2, TUT1, TXNRD1, UAP1, UBR5, UFL1, UGDH, UGGT1, UGGT2, UGP2, UNG, UPF2, UPF3B, USP47, USP7, USP9X, UTP15, UTP18, UTP20, UTP25, UTP4, VIM, VRTN, WARS1, WDHD1, WDR18, WDR3, WDR36, WDR43, WRAP53, XPO5, XRCC5, XRCC6, XRN1, XRN2, YAP1, YARS2, ZC3H7B, ZC3HAV1, ZFP36L2, ZMYND8, ZNF217, ZNF281, ZNF462, ZNF638]	
protein metabolic process	GO_BiologicalProcess-EBI-UniProt-GOA-ACAP-ARAP_13.05.2021_00h00	1.49E-64	9.936006	590	[AAAS, AASS, AATF, ABCB10, ABCF1, ABHD10, ABHD12, ABI1, ACACA, ACO1, ACSL1, ADAM10, ADAR, ADARB1, AGO1, AGO2, AGTPBP1, AIMP2, AKT1, ALG11, ALG2, ALG5, ALG9, ANKZF1, ANTXR1, ANXA1, ANXA2, AP2A1, AP2A2, AP2B1, AP2M1, AP2S1, AP3B1, APAF1, APEH, APOE, ARF4, ARFGEF1, ARL6IP5, ARSB, ASPH, ATP2B4, ATR, AUP1, AURKA, AURKB, B3GLCT, B4GALT1, BAG2, BAG3, BAZ1B, BCCIP, BIN1, BIRC6, BPNT2, BRAT1, BRD4, BRMS1, BUB1B, C1QBP, CALR, CALU, CAMK1, CAMK2D,	No change

					CAPN1, CAPN2, CAPNS1, CAPRIN1, CASK, CASP3, CAST, CAT, CAV1, CBL, CCAR2, CCDC22, CCNB1, CCND2, CCNH, CCNL2, CCNY, CD2AP, CD44, CD81, CDC123, CDC42, CDC42BPA, CDC42BPB, CDC73, CDH2, CDK5RAP1, CDK5RAP3, CDK7, CELF1, CFAP20, CHEK1, CHEK2, CHPF, CHST14, CIRBP, CKAP4, CLASP1, CNDP2, CNOT11, COG7, COL6A3, COPS2, COPS5, COPS7A, CORO1C, CPD, CPOX, CPZ, CRTAP, CSDE1, CSPG4, CTCF, CTNNB1, CTSC, CUL4A, CUL4B, CUL7, CWC27, CYFIP1, CYP51A1, DAPK1, DARS2, DAXX, DBNL, DCAF1, DCAF13, DDRGK1, DDX39B, DDX6, DHPS, DHX33, DHX9, DIPK2A, DLG1, DNMT1, DNMT3B, DOCK7, DPH1, DPH2, DPH6, DPP3, DRG1, DSP, DUSP3, ECE1, ECPAS, ECT2, EDEM3, EEF1E1, EEF2, EFL1, EGFR, EHD4, EHMT1, EIF3A, EIF4A3, EIF5B, ELP3, EPHB3, ERAP1, ERLEC1, ERLIN2, ERO1A, ERP44, ETFA, EXOSC2, EXOSC5, EXOSC7, FAF2, FASTKD2, FBXO2, FBXO30, FBXW8, FDXR, FERMT2, FGG, FKBP10, FKBP11, FKBP14, FKBP15, FKBP4, FKBP5, FKBP7, FKBP8, FKBP9, FLT1, FN1, FOXK1, FUT11, FXR1, GALNT1, GALNT10, GALNT2, GARS1, GATA6, GCLC, GEMIN5, GET4, GFPT2, GIPC1, GLMN, GLUL, GNL3, GOLGA2, GPC1, GPC3, GPD1L, GPHN, GPI, GSN, GTF3C4, GTPBP1, GUF1, GXYLT1, HAT1, HCFC1, HDAC2, HDAC4, HERC2, HEXA, HLA-A, HMBS, HSP90AB1, HSP90B1, HSPA2, HSPA5, HSPB1, HSPD1, HSPE1, HTRA1, HTT, HUWE1, IGF2BP3, ILF3, ILK, INCENP, INO80C, IPO5, IQGAP1, IQGAP3, IRF2BPL, IST1, ITCH, ITGA5, ITGAV, IWS1, JADE1, JARID2, KAT7, KDM1A, KDM2A, KDM3B, KEAP1, KTN1, L2HGDH, LAMB1, LAMC1, LARS1, LARS2, LCMT1, LGALS1, LIN28A, LMAN1,	

					LMCD1, LMF2, LNPEP, LPCAT1, LRP1, LRPPRC, LRRC40, LRRK1, LSM4, LTBP1, LYPLA2, MACROH2A1, MAGED1, MAN1A1, MAN1A2, MANBA, MAP2K6, MAPK1, MAPK14, MAPK8, MARCHF5, MARS1, MASTL, MAVS, MBD3, MCM2, METAP1, MFGE8, MGAT2, MICAL1, MIPEP, MLH1, MMAB, MME, MOCS2, MOGS, MRPS27, MRPS9, MSN, MTA3, MTMR14, MTMR6, MVB12A, MVD, MYADM, MYDGF, MYH9, MYO1C, NCL, NDC1, NDRG1, NEDD4, NEK7, NFKB1, NIBAN1, NLE1, NLN, NMT2, NNT, NPM1, NRP2, NSF, NSFL1C, NSUN5, NUDT16, NUP107, NUP133, NUP160, NUP210, NUP35, NUP50, NXN, OCLN, OGT, ORC3, OSBP, OSTC, OTUD6B, P3H1, P3H3, P3H4, P4HA1, P4HA2, P4HB, P4HTM, PAK1, PALD1, PARD3, PARN, PARP1, PARVA, PASK, PAWR, PAXBP1, PCBP2, PCOLCE, PDCD2, PDGFRB, PDIA3, PDIA6, PDZRN3, PEA15, PGAM5, PGM3, PHC1, PHIP, PHPT1, PIAS4, PICALM, PIGS, PIGT, PIK3CA, PIK3R4, PITRM1, PKM, PLK1, PLOD1, PLOD2, PLOD3, PLPP3, PLXNB2, PM20D2, PNPT1, POFUT1, POFUT2, POGLUT2, POGLUT3, POLB, POLR2G, POR, PPIB, PPIC, PPID, PPM1B, PPM1F, PPM1G, PPP1R7, PPP1R9B, PPP2R5A, PPP4R1, PRDX4, PREPL, PRKAA1, PRKAB1, PRKACB, PRKACG, PRKAG1, PRKAR1A, PRKAR2A, PRKAR2B, PRKCA, PRKCSH, PRKDC, PRMT3, PRPF8, PRRC1, PSMC6, PSMD1, PSMD2, PSMD9, PSME3, PTCD3, PTK7, PTPMT1, PTPN1, PTPN12, PTPN2, PTPRD, PURA, PUS7, PXK, PYCARD, QRSL1, RAB1A, RAB2A, RAB8A, RAD51, RANBP2, RANGAP1, RAP2A, RAP2B, RAP2C, RBBP5, RBM3, RBPMS, RCN1, RDX, RELA, RIC1, RIF1, RIMKLB, RIOX1,	

					RNF170, RNPEP, ROCK2, RPL13A, RPL22, RPL27A, RPL4, RPL5, RPL7A, RPN1, RPRD1A, RPS2, RPS21, RPS6KA1, RPS6KA3, RPS6KA4, RPS8, RRBP1, SAMD4B, SARS1, SARS2, SART3, SBF1, SCPEP1, SCRN1, SCRN3, SCYL1, SDF2L1, SEC22B, SEH1L, SEL1L, SEPHS1, SERPINB6, SERPINB9, SERPINH1, SESN2, SET, SETD7, SETDB1, SIN3A, SIRT1, SIRT2, SKP2, SLC2A10, SLK, SMARCAD1, SMARCC1, SNX3, SNX6, SNX9, SORD, SPAG9, SPON1, SPTBN1, SRC, SRPK1, STAM2, STK38, STT3A, STT3B, STX5, STYX, SUMF2, SUPT6H, SYMPK, TARS2, TELO2, TF, TGFB1I1, TGM2, THBS1, TIA1, TIMM50, TIMP3, TIPRL, TLK1, TMED10, TMED2, TMF1, TMX3, TOLLIP, TPP2, TPST1, TPX2, TRAP1, TRIM28, TRIM33, TRIM71, TRIP12, TRMT10C, TRPT1, TTK, TTLL12, TWF1, UBA6, UBE2G1, UBE2H, UBE2O, UBR4, UBR5, UFL1, UGDH, UGGT1, UGGT2, UPF3B, USE1, USP15, USP19, USP47, USP5, USP7, USP9X, UTP25, VIM, VLDLR, VPS25, VPS35, VPS36, VRK1, WARS1, XPNPEP1, XRCC5, XRCC6, XRN1, YARS2, YOD1, ZC3H15, ZC3HAV1, ZDHHC17, ZFP36L2]	
regulation of cellular metabolic process	GO_BiologicalProcess-EBI-UniProt-GOA-ACAP-ARAP_13.05.2021_00h00	1.49E-64	8.841941	565	[AAAS, AASS, AATF, ABAT, ABCB10, ABCB7, ABCF1, ABHD14B, ABI1, ACACA, ACLY, ACO1, ACSL1, ACTN1, ACTN4, ACTR2, ADAR, ADARB1, AEBP2, AGO1, AGO2, AGTPBP1, AIMP2, AK4, AKT1, ANTXR1, ANXA1, ANXA2, ANXA3, ANXA4, AP2M1, AP3B1, APAF1, APOE, APPL2, ARAP1, ARF4, ARFGEF1, ARHGEF11, ARL6IP5, ASPH, ATF7IP, ATP1A1, ATP2B4, ATP6V1A, ATR, AURKA, AURKB, BAG2, BAG3, BAZ1B, BCCIP, BIN1, BIRC6, BPTF, BRAT1, BRD3, BRD4, BRMS1, BRWD1, C1QBP, CACYBP, CALCOCO2, CALR, CAMK1, CAMK2D, CAPN1, CAPN2, CAPNS1, CAPRIN1, CARHSP1, CASK, CASP3,	No change

					CAST, CAT, CAV1, CBL, CBR1, CBX2, CBX5, CCAR1, CCAR2, CCDC22, CCNB1, CCND2, CCNH, CCNL2, CCNY, CCT2, CCT3, CCT4, CCT5, CCT6A, CCT7, CCT8, CD44, CD81, CDC123, CDC42, CDC73, CDH3, CDK5RAP1, CDK5RAP3, CDK7, CEBPZ, CELF1, CHD1, CHD4, CHEK1, CHEK2, CHTF18, CIRBP, CNOT11, COL1A1, COL6A3, COPS2, COPS5, CORO1C, CRLF3, CRTAP, CSDE1, CSPG4, CTBP2, CTCF, CTNNB1, CTSC, CUL4A, CYFIP1, DAGLB, DAPK1, DAXX, DAZAP1, DBNL, DCAF1, DCXR, DDRGK1, DDX20, DDX39B, DDX49, DDX5, DDX54, DDX6, DHX33, DHX8, DHX9, DICER1, DIPK2A, DLG1, DLGAP5, DNMT1, DNMT3A, DNMT3B, DOCK7, DPH1, DPH2, DPH6, DUSP3, ECE1, ECT2, EDRF1, EEF2, EFL1, EGFR, EHD4, EHMT1, EIF3A, EIF4A3, EIF5B, ELP3, EMSY, EPCAM, EPHB3, EPM2AIP1, ERBIN, ERCC2, ERLIN2, ETFA, EXOSC10, EXOSC2, EXOSC5, EXOSC7, FASTKD2, FBXO2, FBXW8, FERMT2, FKBP8, FLT1, FN1, FOXK1, FXR1, G3BP2, GARS1, GATA6, GATAD2A, GCLC, GEMIN5, GIPC1, GLA, GLMN, GNL3, GOLGA2, GPC3, GPD1L, GPI, GPSM1, GSN, GTF2I, GTPBP1, GUF1, H6PD, HAT1, HCFC1, HDAC2, HDAC4, HEATR1, HELLS, HK2, HMOX1, HNRNPF, HNRNPL, HNRNPLL, HNRNPM, HP1BP3, HSD17B4, HSP90AB1, HSPA2, HSPA5, HSPB1, HSPD1, HSPE1, HTT, HUWE1, IGF2BP3, ILF2, ILF3, ILK, INCENP, IPO5, IQGAP1, IQGAP3, IRF2BPL, IRF3, IST1, ITCH, ITGA5, ITGA6, ITGAV, IWS1, JARID2, KANK2, KAT7, KDM1A, KDM2A, KDM3B, KEAP1, LANCL2, LCMT1, LIG3, LIMS1, LIN28A, LMCD1, LPCAT1, LPGAT1, LRP1, LRPPRC, LRRK1, LSM4, LYAR, MACROH2A1, MAGED1, MAP2K6, MAPK1, MAPK14, MAPK8, MARS1, MASTL, MAVS,	

					MBD3, MBNL1, MCM2, MCM7, METAP1, MICAL1, MLH1, MMAB, MRPS27, MSH2, MSH3, MSH6, MSN, MTA3, MTDH, MYADM, MYBBP1A, MYD88, MYDGF, MYEF2, MYH9, MYO1C, NBAS, NCL, NDC1, NEDD4, NEK7, NFKB1, NIBAN1, NIBAN2, NIF3L1, NLE1, NLN, NNT, NOL11, NPM1, NQO2, NSF, NSUN2, NSUN5, NUDT16, NUP107, NUP133, NUP160, NUP210, NUP35, NUP50, NVL, NXN, OCLN, OGT, OPTN, ORC2, ORC3, ORC5, OSBP, P3H1, PABPN1, PAK1, PARD3, PARN, PARP1, PARVA, PASK, PATZ1, PAWR, PAXBP1, PCBP2, PCOLCE, PDCD2, PDGFRB, PDS5A, PEA15, PELP1, PFKM, PHC1, PHIP, PIAS4, PICALM, PIK3C2A, PIK3CA, PIK3R4, PIP4K2B, PIP4K2C, PKM, PLK1, PLPP3, PLXNB2, PM20D2, PNPT1, POFUT1, POGZ, POLA1, POLR1B, POLR2A, POLR2B, POLR2G, POLR3C, POLR3F, POR, PPIB, PPID, PPM1F, PPP1R7, PPP1R9B, PPP2R5A, PPP4R1, PRKAA1, PRKAB1, PRKACB, PRKACG, PRKAG1, PRKAR1A, PRKAR2A, PRKAR2B, PRKCA, PRKCSH, PRKDC, PRMT3, PRRC1, PSIP1, PSMC6, PSMD1, PSMD2, PSMD9, PSME3, PSPC1, PTBP2, PTCD3, PTGIS, PTPN1, PTPN2, PURA, PUS7, PXDN, PYCARD, RAB1A, RAB8A, RAD51, RANBP2, RAP2A, RAP2B, RAP2C, RBM15, RBM25, RBM3, RBPJ, RBPMS, RDH10, RDX, RELA, RFC1, RFC2, RFC3, RFC5, RHEB, RIC1, RIF1, RIOX1, ROCK2, RPA2, RPL13A, RPL22, RPL5, RPRD1A, RPS2, RPS6KA1, RPS6KA3, RPS6KA4, S100A11, SALL2, SAMD4B, SAP30BP, SARS1, SART3, SCARB2, SCFD1, SEC13, SEC22B, SEH1L, SERBP1, SERPINB6, SERPINB9, SERPINH1, SESN2, SET, SETD7, SETDB1, SH3GLB1, SIN3A, SIRT1, SIRT2, SKP2, SLC25A12,	

					SLC25A5, SLC2A10, SLC5A3, SLIRP, SLTM, SMARCA4, SMARCA5, SMARCAD1, SMARCC1, SMARCD1, SMARCD2, SNX6, SNX9, SORBS1, SORBS3, SORD, SPAG9, SPON1, SRC, SRPK1, SRSF1, SRSF7, SSBP1, STK38, STXBP2, STYX, SUPT16H, SUPT5H, SUPT6H, SYMPK, TAX1BP1, TBRG4, TCP1, TDP2, TELO2, TF, TFB1M, TGFB1I1, THBS1, TIA1, TIGAR, TIMP3, TIPRL, TMED10, TMED2, TMF1, TOLLIP, TOP2A, TPX2, TRAP1, TRIM28, TRIM33, TRIM71, TRIP12, TRIP13, TRMT10C, TRPT1, TTK, TTLL12, TWF1, UBE2O, UBR5, UFL1, UPF3B, USP19, USP47, USP5, USP7, USP9X, UTP15, UTP4, VAMP3, VIM, VLDLR, VPS25, VPS26A, VPS26B, VPS35, VRTN, WAPL, WARS1, WDR18, WDR43, WRAP53, XRCC5, XRCC6, XRN1, YAP1, ZC3HAV1, ZFP36L2, ZMYND8, ZNF217, ZNF281, ZNF462]	
cellular macromolecule metabolic process	GO_BiologicalProcess-EBI-UniProt-GOA-ACAP-ARAP_13.05.2021_00h00	1.49E-64	9.149864	776	[AAAS, AASS, AATF, ABCB10, ABCF1, ABHD10, ABHD12, ABHD14B, ABI1, ACO1, ACSL1, ACTN1, ACTN4, ACTR2, ADAM10, ADAR, ADARB1, AEBP2, AGL, AGO1, AGO2, AGTPBP1, AIMP2, AKT1, ALG11, ALG2, ALG5, ALG9, ANKRD28, ANKZF1, ANTXR1, ANXA1, ANXA2, ANXA3, ANXA4, AP2A1, AP2A2, AP2B1, AP2M1, AP2S1, AP3B1, APAF1, APEH, APOBEC3C, APOE, ARAP1, ARF4, ARFGEF1, ARHGEF11, ARL6IP5, ARSB, ASPH, ATF7IP, ATP2B4, ATR, AUP1, AURKA, AURKB, B3GLCT, B4GALT1, BAG2, BAG3, BAZ1B, BCCIP, BIN1, BIRC6, BPNT2, BPTF, BRAT1, BRD3, BRD4, BRMS1, BRWD1, BUB1B, C1QBP, CACYBP, CALR, CALU, CAMK1, CAMK2D, CAPN1, CAPN2, CAPRIN1, CARHSP1, CASK, CASP3, CAST, CAT, CAV1, CAVIN1, CBL, CBX2, CBX5, CCAR1, CCAR2, CCDC22, CCNB1, CCND2, CCNH, CCNL2,	No change

					CCNY, CCT2, CCT3, CCT4, CCT5, CCT6A, CCT7, CCT8, CD2AP, CD44, CD81, CDC123, CDC42, CDC42BPA, CDC42BPB, CDC73, CDH2, CDK5RAP1, CDK5RAP3, CDK7, CEBPZ, CELF1, CFAP20, CHAF1A, CHD1, CHD4, CHEK1, CHEK2, CHPF, CHST14, CHTF18, CIRBP, CKAP4, CLASP1, CNOT11, COG7, COL1A1, COL4A2, COL6A3, COPS2, COPS5, COPS7A, CORO1C, CPSF2, CPSF3, CRLF3, CRTAP, CSDE1, CSPG4, CSTF1, CSTF3, CTBP2, CTCF, CTNNB1, CTSC, CUL4A, CUL4B, CUL7, CWC27, CYFIP1, DAPK1, DARS2, DAXX, DBNL, DCAF1, DCAF13, DDRGK1, DDX20, DDX21, DDX39B, DDX49, DDX5, DDX54, DDX6, DHPS, DHX33, DHX9, DICER1, DIPK2A, DLG1, DLGAP5, DNMT1, DNMT3A, DNMT3B, DOCK7, DPH1, DPH2, DPH6, DRG1, DSP, DUSP3, ECE1, ECPAS, ECT2, EDEM3, EDRF1, EEF1E1, EEF2, EFL1, EGFR, EHD4, EHMT1, EIF3A, EIF4A3, EIF5B, ELP3, EMSY, EPCAM, EPHB3, EPM2AIP1, ERBIN, ERCC2, ERLEC1, ERLIN2, ERO1A, ERP44, ETFA, EXD2, EXOG, EXOSC10, EXOSC2, EXOSC5, EXOSC7, FAF2, FANCD2, FANCI, FASTKD2, FBXO2, FBXO30, FBXW8, FDXR, FERMT2, FGG, FH, FKBP10, FKBP11, FKBP14, FKBP15, FKBP4, FKBP5, FKBP7, FKBP8, FKBP9, FLT1, FN1, FOXK1, FSCN1, FTSJ3, FUT11, FXR1, G3BP2, GALNT1, GALNT10, GALNT2, GARS1, GATA6, GATAD2A, GBE1, GCLC, GEMIN5, GET4, GFPT2, GINS3, GIPC1, GLMN, GLUL, GNL3, GOLGA2, GPC1, GPC3, GPD1L, GPHN, GPI, GSN, GTF2E1, GTF2I, GTF3C1, GTF3C2, GTF3C3, GTF3C4, GTPBP1, GUF1, GXYLT1, GYS1, HAT1, HCFC1, HDAC2, HDAC4, HEATR1, HELLS, HERC2, HEXA, HLA-A, HMBS, HMOX1, HNRNPM, HP1BP3, HSD17B10, HSD17B4, HSP90AB1,	

					HSP90B1, HSPA2, HSPA5, HSPB1, HSPD1, HSPE1, HTT, HUWE1, IGF2BP3, ILF2, ILF3, ILK, INCENP, INO80C, IPO5, IQGAP1, IQGAP3, IRF2BPL, IRF3, IST1, ITCH, ITGA5, ITGA6, ITGB1, IWS1, JADE1, JARID2, KANK2, KAT7, KDM1A, KDM2A, KDM3B, KEAP1, KIF22, KTN1, L2HGDH, LAMB1, LAMC1, LANCL2, LARS1, LARS2, LCMT1, LGALS1, LIG1, LIG3, LIMS1, LIN28A, LMAN1, LMCD1, LNPEP, LRP1, LRPPRC, LRRC40, LRRK1, LRWD1, LSM4, LTBP1, LYAR, LYPLA2, MACROH2A1, MAGED1, MAN1A1, MAN1A2, MANBA, MAP1S, MAP2K6, MAPK1, MAPK14, MAPK8, MARCHF5, MARS1, MASTL, MAVS, MBD3, MCM2, MCM3, MCM4, MCM5, MCM6, MCM7, MCMBP, MDC1, METAP1, METTL1, MFGE8, MGAT2, MICAL1, MIPEP, MLH1, MMAB, MOCS2, MOGS, MRPS27, MRPS9, MSH2, MSH3, MSH6, MSN, MTA3, MTDH, MTMR14, MTMR6, MTREX, MVB12A, MVD, MYADM, MYBBP1A, MYD88, MYDGF, MYEF2, MYH9, MYO1C, NBAS, NCAPG2, NCL, NDC1, NDRG1, NEDD4, NEK7, NFKB1, NIBAN1, NIBAN2, NIF3L1, NLE1, NMT2, NNT, NOL11, NPM1, NPM3, NRP2, NSF, NSFL1C, NSUN2, NSUN5, NTHL1, NUDT16, NUP107, NUP133, NUP160, NUP210, NUP35, NUP50, NVL, NXN, OCLN, OGT, OPTN, ORC2, ORC3, ORC4, ORC5, OSBP, OSTC, OTUD6B, P3H1, P3H3, P3H4, P4HA1, P4HA2, P4HB, P4HTM, PABPN1, PAK1, PALD1, PARD3, PARN, PARP1, PARVA, PASK, PATZ1, PAWR, PAXBP1, PCBP2, PCOLCE, PDCD2, PDGFRB, PDIA6, PDS5A, PDZRN3, PEA15, PELP1, PFKM, PGAM5, PGM2L1, PGM3, PHC1, PHIP, PHPT1, PIAS4, PICALM, PIGS, PIGT, PIK3CA, PIK3R4, PKM, PLD3, PLK1, PLOD1, PLOD2, PLOD3, PLPP3,	

					PLXNB2, PM20D2, PNPT1, POFUT1, POFUT2, POGLUT2, POGLUT3, POGZ, POLA1, POLA2, POLB, POLD1, POLE, POLR1B, POLR2A, POLR2B, POLR2G, POLR3A, POLR3C, POLR3F, POR, PPIB, PPIC, PPID, PPM1B, PPM1F, PPM1G, PPP1R7, PPP1R9B, PPP2R5A, PPP4R1, PRDX4, PRIM1, PRIM2, PRKAA1, PRKAB1, PRKACB, PRKACG, PRKAG1, PRKAR1A, PRKAR2A, PRKAR2B, PRKCA, PRKCSH, PRKDC, PRMT3, PRORP, PRRC1, PSIP1, PSMC6, PSMD1, PSMD2, PSMD9, PSME3, PSPC1, PTCD3, PTGIS, PTK7, PTPMT1, PTPN1, PTPN12, PTPN2, PTPRD, PURA, PUS7, PXDN, PXK, PYCARD, QRSL1, RAB1A, RAB2A, RAB8A, RAD51, RAN, RANBP2, RANGAP1, RAP2A, RAP2B, RAP2C, RBBP5, RBM15, RBM3, RBPJ, RBPMS, RCN1, RDX, RELA, RFC1, RFC2, RFC3, RFC5, RIC1, RIF1, RIMKLB, RIOX1, RNF170, RNH1, ROCK2, RPA1, RPA2, RPA3, RPL13A, RPL22, RPL27A, RPL4, RPL5, RPL7A, RPN1, RPRD1A, RPS2, RPS21, RPS6KA1, RPS6KA3, RPS6KA4, RPS8, RRBP1, S100A11, SALL2, SAMD4B, SAMHD1, SAP30BP, SARS1, SARS2, SART3, SBF1, SCYL1, SDF2L1, SEH1L, SEL1L, SEPHS1, SERBP1, SERPINB6, SERPINB9, SERPINH1, SESN2, SET, SETD7, SETDB1, SH3GLB1, SIN3A, SIRT1, SIRT2, SKP2, SLC2A10, SLIRP, SLK, SLTM, SMARCA4, SMARCA5, SMARCAD1, SMARCC1, SMARCD1, SMARCD2, SNRPD3, SNX3, SNX6, SNX9, SORBS1, SORBS3, SORD, SPAG9, SPON1, SPTBN1, SRC, SRPK1, SSB, SSBP1, SSRP1, STAM2, STK38, STT3A, STT3B, STXBP2, STYX, SUMF2, SUPT16H, SUPT5H, SUPT6H, SYMPK, TARS2, TAX1BP1, TBPL1, TBRG4, TCP1, TDP1, TDP2, TELO2, TF, TFB1M, TGFB1I1, TGM2, THBS1,	

					THUMPD3, TIA1, TIGAR, TIMM50, TIMP3, TIPRL, TLK1, TMED10, TMED2, TMF1, TMX3, TOLLIP, TOP2A, TPP2, TPST1, TPX2, TRAP1, TRIM28, TRIM33, TRIM71, TRIP12, TRIP13, TRMT1, TRMT10C, TRMT1L, TRMT5, TRPT1, TTF2, TTK, TTLL12, TUT1, TWF1, UBA6, UBE2G1, UBE2H, UBE2O, UBR4, UBR5, UFL1, UGDH, UGGT1, UGGT2, UGP2, UNG, UPF2, UPF3B, USP15, USP19, USP47, USP5, USP7, USP9X, UTP15, UTP4, VAMP3, VIM, VLDLR, VPS25, VPS35, VPS36, VRK1, VRTN, WAPL, WARS1, WDHD1, WDR18, WDR43, WRAP53, XRCC5, XRCC6, XRN1, XRN2, YAP1, YARS2, YOD1, ZC3H15, ZC3HAV1, ZDHHC17, ZFP36L2, ZMYND8, ZNF217, ZNF281, ZNF462]	
regulation of macromolecule metabolic process	GO_BiologicalProcess-EBI-UniProt-GOA-ACAP-ARAP_13.05.2021_00h00	1.49E-64	8.683134	573	[AAAS, AASS, AATF, ABCB10, ABCF1, ABHD14B, ABI1, ACO1, ACSL1, ACTN1, ACTN4, ACTR2, ADAM10, ADAR, ADARB1, AEBP2, AGO1, AGO2, AGTPBP1, AIMP2, AKT1, ANK2, ANK3, ANTXR1, ANXA1, ANXA2, ANXA3, ANXA4, AP2A1, AP2M1, AP3B1, APAF1, APOE, APPL2, ARAP1, ARF4, ARFGEF1, ARHGEF11, ARL6IP5, ASPH, ATF7IP, ATP2B1, ATP2B4, ATR, AURKA, AURKB, BAG2, BAG3, BAZ1B, BCCIP, BIN1, BIRC6, BPTF, BRAT1, BRD3, BRD4, BRMS1, BRWD1, C1QBP, CACYBP, CALR, CAMK1, CAMK2D, CAPN2, CAPRIN1, CARHSP1, CASK, CASP3, CAST, CAT, CAV1, CBL, CBX2, CBX5, CCAR1, CCAR2, CCDC22, CCNB1, CCND2, CCNH, CCNL2, CCNY, CCT2, CCT3, CCT4, CCT5, CCT6A, CCT7, CCT8, CD2AP, CD44, CD81, CDC123, CDC42, CDC73, CDH3, CDK5RAP1, CDK5RAP3, CDK7, CEBPZ, CELF1, CHD1, CHD4, CHEK1, CHEK2, CHID1, CHTF18, CIRBP, CNOT11, CNRIP1, COL1A1, COL6A3, COPS2, COPS5, CORO1C, CRLF3, CRTAP, CSDE1, CSPG4, CTBP2, CTCF, CTNNB1, CTSC,	Upregulation

					CUL4A, CYFIP1, CYP51A1, DAPK1, DAXX, DAZAP1, DBNL, DCAF1, DDRGK1, DDX20, DDX21, DDX39B, DDX41, DDX49, DDX5, DDX54, DDX6, DHX33, DHX9, DICER1, DIPK2A, DLG1, DLGAP5, DNMT1, DNMT3A, DNMT3B, DOCK7, DPH1, DPH2, DPH6, DUSP3, ECE1, ECT2, EDRF1, EEF2, EFL1, EGFR, EHD4, EHMT1, EIF3A, EIF4A3, EIF5B, ELP3, EMSY, EPB41L5, EPCAM, EPM2AIP1, ERBIN, ERCC2, ETFA, EXOSC10, EXOSC2, EXOSC5, EXOSC7, F11R, FASTKD2, FBXO2, FBXW8, FERMT2, FKBP8, FLT1, FN1, FOXK1, FXR1, G3BP1, G3BP2, GARS1, GATA6, GATAD2A, GCLC, GEMIN5, GIPC1, GLMN, GNL3, GOLGA2, GPC3, GPD1L, GPI, GSN, GTF2I, GTPBP1, GUF1, HAT1, HCFC1, HDAC2, HDAC4, HEATR1, HELLS, HK1, HLA-A, HMOX1, HNRNPF, HNRNPL, HNRNPLL, HNRNPM, HP1BP3, HSD17B4, HSP90AB1, HSPA2, HSPA5, HSPB1, HSPD1, HSPE1, HSPH1, HTT, HUWE1, IGF2BP3, ILF2, ILF3, ILK, INCENP, IPO5, IQGAP1, IQGAP3, IRF2BPL, IRF3, IST1, ITCH, ITGA5, ITGA6, ITGAV, IWS1, JARID2, KANK2, KAT7, KDM1A, KDM2A, KDM3B, KEAP1, LANCL2, LIG3, LIMS1, LIN28A, LMCD1, LPCAT1, LRP1, LRPPRC, LRRK1, LSM4, LTBP1, LYAR, MACROH2A1, MAGED1, MAP2K6, MAPK1, MAPK14, MAPK8, MARS1, MASTL, MATR3, MAVS, MBD3, MBNL1, MCM2, METAP1, MICAL1, MLH1, MMAB, MRPS27, MSH2, MSH3, MSH6, MSN, MTA3, MTDH, MYADM, MYBBP1A, MYD88, MYDGF, MYEF2, MYH9, MYO1C, MYO1E, NBAS, NCL, NDC1, NDRG2, NEDD4, NEK7, NFKB1, NIBAN1, NIBAN2, NIF3L1, NLE1, NNT, NOL11, NPM1, NSF, NSUN2, NSUN5, NUDT16, NUP107, NUP133, NUP160, NUP210, NUP35,	

					NUP50, NVL, NXN, OCLN, OGT, OPTN, ORC2, ORC3, ORC5, OSBP, P3H1, PABPN1, PAK1, PARD3, PARN, PARP1, PARVA, PASK, PATZ1, PAWR, PAXBP1, PC, PCBP2, PCOLCE, PDCD2, PDCL3, PDGFRB, PDS5A, PEA15, PELP1, PFKM, PHC1, PHIP, PIAS4, PICALM, PIK3CA, PKM, PLD3, PLK1, PLPP3, PLXNB2, PM20D2, PNP, PNPT1, POFUT1, POFUT2, POGZ, POLA1, POLR1B, POLR2A, POLR2B, POLR2G, POLR3A, POLR3C, POLR3F, POR, PPIB, PPID, PPM1B, PPM1F, PPP1R7, PPP1R9B, PPP2R5A, PPP4R1, PRKAA1, PRKAB1, PRKACB, PRKACG, PRKAG1, PRKAR1A, PRKAR2A, PRKAR2B, PRKCA, PRKCSH, PRKDC, PRMT3, PRRC1, PSIP1, PSMC6, PSMD1, PSMD2, PSMD9, PSME3, PSPC1, PTBP2, PTCD3, PTGIS, PTPN1, PTPN2, PURA, PUS7, PXDN, PYCARD, RAB1A, RAD51, RAN, RANBP2, RAP2A, RAP2B, RAP2C, RBM15, RBM25, RBM3, RBPJ, RBPMS, RDX, RELA, RFC1, RFC2, RFC3, RFC5, RFTN1, RIC1, RIF1, RIOX1, RNH1, ROCK2, RPA2, RPL13A, RPL22, RPL27A, RPL4, RPL5, RPL7A, RPRD1A, RPS2, RPS21, RPS6KA1, RPS6KA3, RPS6KA4, RPS8, S100A11, SALL2, SAMD4B, SAP30BP, SARS1, SART3, SCARB2, SEC13, SEC22B, SEH1L, SERBP1, SERPINB6, SERPINB9, SERPINH1, SESN2, SET, SETD7, SETDB1, SF3B1, SIN3A, SIRT1, SIRT2, SKP2, SLC2A10, SLIRP, SLTM, SMARCA4, SMARCA5, SMARCAD1, SMARCC1, SMARCD1, SMARCD2, SNX3, SNX6, SNX9, SORBS1, SORBS3, SORD, SPAG9, SPON1, SPOUT1, SPTBN1, SRC, SRPK1, SRSF1, SRSF7, SSB, SSBP1, STK38, STX5, STXBP2, STYX, SUPT16H, SUPT5H, SUPT6H, SYMPK, TAX1BP1, TBRG4, TCP1, TDP2, TELO2, TF, TFB1M, TGFB1I1, THBS1, TIA1, TIGAR,	

					TIMP3, TIPRL, TMED10, TMED2, TMF1, TOLLIP, TOP2A, TPX2, TRAP1, TRIM28, TRIM33, TRIM71, TRIP12, TRIP13, TRMT10C, TRPT1, TTK, TTLL12, TUT1, TWF1, UBE2O, UBR5, UFL1, UPF2, UPF3B, USP19, USP47, USP5, USP7, USP9X, UTP15, UTP4, VAMP3, VIM, VLDLR, VPS25, VPS35, VRTN, WAPL, WARS1, WDR18, WDR43, WRAP53, XPO5, XRCC5, XRCC6, XRN1, XRN2, YAP1, ZC3H7B, ZC3HAV1, ZFP36L2, ZMYND8, ZNF217, ZNF281, ZNF462]	
regulation of primary metabolic process	GO_BiologicalProcess-EBI-UniProt-GOA-ACAP-ARAP_13.05.2021_00h00	1.49E-64	8.740693	540	[AAAS, AASS, AATF, ABAT, ABCB10, ABCF1, ABHD14B, ABI1, ACACA, ACO1, ACSL1, ACTN1, ACTN4, ACTR2, ADAR, ADARB1, AEBP2, AGO1, AGO2, AGTPBP1, AIMP2, AK4, AKT1, ANTXR1, ANXA1, ANXA2, ANXA3, ANXA4, AP2A1, AP3B1, APAF1, APOE, APPL2, ARF4, ARFGEF1, ARHGEF11, ARL6IP5, ASPH, ATF7IP, ATP1A1, ATP2B4, ATR, AURKA, AURKB, BAG2, BAG3, BAZ1B, BCCIP, BIN1, BIRC6, BPTF, BRAT1, BRD3, BRD4, BRMS1, BRWD1, C1QBP, CALR, CAMK1, CAMK2D, CAPN2, CAPRIN1, CARHSP1, CASK, CASP3, CAST, CAT, CAV1, CBL, CBX2, CBX5, CCAR1, CCAR2, CCDC22, CCNB1, CCND2, CCNH, CCNL2, CCNY, CCT2, CCT3, CCT4, CCT5, CCT6A, CCT7, CCT8, CD44, CD81, CDC123, CDC42, CDC73, CDK5RAP1, CDK5RAP3, CDK7, CEBPZ, CELF1, CHD1, CHD4, CHEK1, CHEK2, CHTF18, CIRBP, CNOT11, COL1A1, COL6A3, COPS2, COPS5, CORO1C, CRLF3, CRTAP, CSDE1, CSPG4, CTBP2, CTCF, CTNNB1, CTSC, CUL4A, CYFIP1, CYP51A1, DAGLB, DAPK1, DAXX, DAZAP1, DBNL, DCAF1, DDRGK1, DDX20, DDX39B, DDX49, DDX5, DDX54, DDX6, DHX33, DHX8, DHX9, DICER1, DIPK2A, DLG1, DLGAP5, DNMT1, DNMT3A, DNMT3B, DOCK7, DPH1, DPH2,	No change

					DPH6, DUSP3, ECT2, EDRF1, EEF2, EFL1, EGFR, EHD4, EHMT1, EIF3A, EIF4A3, EIF5B, ELP3, EMSY, EPCAM, EPM2AIP1, ERBIN, ERCC2, ERLIN2, ETFA, EXOSC10, EXOSC2, EXOSC5, EXOSC7, FASTKD2, FBXO2, FBXW8, FDPS, FERMT2, FKBP8, FLNB, FLT1, FN1, FOXK1, FXR1, G3BP2, GARS1, GATA6, GATAD2A, GCLC, GEMIN5, GIPC1, GLMN, GNL3, GOLGA2, GPC3, GPD1L, GPI, GSN, GTF2I, GTPBP1, GUF1, H6PD, HAT1, HCFC1, HDAC2, HDAC4, HEATR1, HELLS, HMOX1, HNRNPF, HNRNPL, HNRNPLL, HNRNPM, HP1BP3, HSD17B4, HSP90AB1, HSPA2, HSPA5, HSPB1, HSPD1, HSPE1, HTT, HUWE1, IDI1, IGF2BP3, ILF2, ILF3, ILK, INCENP, IPO5, IQGAP1, IQGAP3, IRF2BPL, IRF3, IST1, ITCH, ITGA5, ITGA6, ITGAV, IWS1, JARID2, KANK2, KAT7, KDM1A, KDM2A, KDM3B, KEAP1, LANCL2, LCMT1, LIMS1, LIN28A, LMCD1, LPCAT1, LPGAT1, LRP1, LRPPRC, LRRK1, LSM4, LSS, LYAR, MACROH2A1, MAGED1, MAP2K6, MAPK1, MAPK14, MAPK8, MARS1, MASTL, MAVS, MBD3, MBNL1, MCM2, METAP1, MICAL1, MLH1, MMAB, MRPS27, MSH2, MSH3, MSH6, MSN, MTA3, MTDH, MVD, MVK, MYADM, MYBBP1A, MYD88, MYDGF, MYEF2, MYH9, MYO1C, NBAS, NCL, NDC1, NEDD4, NEK7, NFKB1, NIBAN1, NIBAN2, NIF3L1, NLE1, NLN, NNT, NOL11, NPM1, NSF, NSUN2, NSUN5, NUDT16, NUP107, NUP133, NUP160, NUP210, NUP35, NUP50, NVL, NXN, OCLN, OGT, ORC2, ORC3, OSBP, P3H1, PABPN1, PAK1, PARD3, PARN, PARP1, PARVA, PASK, PATZ1, PAWR, PAXBP1, PCBP2, PCOLCE, PDCD2, PDGFRB, PEA15, PELP1, PFKM, PHC1, PHIP, PIAS4, PICALM, PIK3CA, PIK3R4, PIP4K2B,	

					PIP4K2C, PKM, PLK1, PLPP3, PLXNB2, PM20D2, PNPT1, POFUT1, POGZ, POLA1, POLR1B, POLR2A, POLR2B, POLR2G, POLR3C, POLR3F, POR, PPIB, PPID, PPM1F, PPP1R7, PPP1R9B, PPP2R5A, PPP4R1, PRKAA1, PRKAB1, PRKACB, PRKACG, PRKAG1, PRKAR1A, PRKAR2A, PRKAR2B, PRKCA, PRKCSH, PRKDC, PRMT3, PRRC1, PSIP1, PSMC6, PSMD1, PSMD2, PSMD9, PSME3, PSPC1, PTBP2, PTCD3, PTGIS, PTPN1, PTPN2, PURA, PUS7, PXDN, PYCARD, RAB1A, RAD51, RAN, RANBP2, RAP2A, RAP2B, RAP2C, RBM15, RBM25, RBM3, RBPJ, RBPMS, RDH10, RDX, RELA, RFC1, RFC2, RFC3, RFC5, RIC1, RIF1, RIOX1, ROCK2, RPA2, RPL13A, RPL22, RPL5, RPRD1A, RPS2, RPS6KA1, RPS6KA3, RPS6KA4, SALL2, SAMD4B, SAP30BP, SARS1, SART3, SCARB2, SEC13, SEC22B, SEH1L, SERBP1, SERPINB6, SERPINB9, SERPINH1, SESN2, SET, SETD7, SETDB1, SIN3A, SIRT1, SIRT2, SKP2, SLC25A12, SLC2A10, SLIRP, SLTM, SMARCA4, SMARCA5, SMARCAD1, SMARCC1, SMARCD1, SMARCD2, SNX3, SNX6, SNX9, SORBS1, SORBS3, SORD, SPAG9, SPON1, SRC, SRPK1, SRSF1, SRSF7, STK38, STX5, STXBP2, STYX, SUPT16H, SUPT5H, SUPT6H, SYMPK, TAX1BP1, TBRG4, TCP1, TDP2, TELO2, TF, TFB1M, TGFB1I1, THBS1, TIA1, TIGAR, TIMP3, TIPRL, TMED10, TMED2, TMF1, TOLLIP, TOP2A, TPX2, TRAP1, TRIM28, TRIM33, TRIM71, TRIP12, TRIP13, TRMT10C, TRPT1, TTK, TTLL12, TWF1, UBE2O, UBR5, UFL1, UPF3B, USP19, USP47, USP5, USP7, USP9X, UTP15, UTP4, VIM, VLDLR, VPS25, VPS35, VRTN, WARS1, WDR18, WDR43, WRAP53, XRCC5, XRCC6, XRN1, YAP1, ZC3HAV1, ZFP36L2,	

					ZMYND8, ZNF217, ZNF281, ZNF462]	
RNA binding	GO_MolecularFunction-EBI-UniProt-GOA-ACAP-ARAP_13.05.2021_00h00	1.49E-64	18.96848	331	[AATF, ABCF1, ACIN1, ACO1, ACTN4, ADAR, ADARB1, AGO1, AGO2, ANXA1, ANXA11, ANXA2, APEH, API5, APOBEC3C, ARCN1, ARHGEF1, BCCIP, BMS1, BOP1, C1QBP, CALR, CANX, CAPRIN1, CARHSP1, CAST, CAVIN1, CCAR1, CCAR2, CCT3, CCT4, CCT5, CCT6A, CEBPZ, CELF1, CFAP20, CIRBP, CKAP4, CLUH, COPS5, CPSF2, CPSF3, CSDE1, CSTF1, CSTF3, CYFIP1, DARS2, DAZAP1, DCAF13, DDX10, DDX18, DDX20, DDX21, DDX24, DDX28, DDX31, DDX39B, DDX41, DDX42, DDX46, DDX47, DDX49, DDX5, DDX52, DDX54, DDX6, DHX15, DHX30, DHX33, DHX37, DHX57, DHX8, DHX9, DIAPH1, DICER1, DNMT1, DNTTIP2, DSP, EEF2, EFL1, EFTUD2, EIF3A, EIF4A3, EIF5B, ELAC2, ELP1, ELP3, ESF1, EXOSC10, EXOSC2, EXOSC5, EXOSC7, FAM120A, FASTKD2, FDPS, FKBP4, FLNB, FNDC3A, FNDC3B, FSCN1, FTSJ3, FXR1, G3BP1, G3BP2, GARS1, GEMIN5, GNL2, GNL3, GOT2, GTPBP1, HADHB, HDAC2, HDLBP, HEATR1, HLA-A, HNRNPF, HNRNPL, HNRNPLL, HNRNPM, HSD17B10, HSP90AB1, HSP90B1, HSPA9, HSPB1, HSPD1, HSPE1, HUWE1, IGF2BP3, ILF2, ILF3, IMP3, IMPDH1, IMPDH2, IPO5, KCTD12, KDM1A, KRT18, KTN1, L1TD1, LAS1L, LBR, LGALS1, LIN28A, LRP1, LRPPRC, LRRC59, LSM4, LYAR, MACF1, MAP4, MARS1, MATR3, MBNL1, METTL1, MEX3A, MKI67, MPHOSPH10, MRPS27, MRPS9, MSI1, MTDH, MTREX, MYBBP1A, MYEF2, MYH10, MYH9, MYO5A, NCL, NEDD4, NOL10, NOL11, NOL6, NOL9, NOP14, NOP56, NOP58, NOP9, NPM1, NPM3, NSUN2, NSUN5, NUDT16, NUSAP1, NVL, P4HB, PABPN1, PARN, PARP1, PCBP2, PDCD11, PDIA3, PDIA4, PELP1, PES1, PKM, PLEC, PNO1, PNPT1, POLR2A,	Upregulation

					POLR2B, POLR2G, PPIB, PRDX1, PRKDC, PRPF38A, PRPF38B, PRPF40A, PRPF8, PSIP1, PSMD2, PSPC1, PTBP2, PTCD1, PTCD3, PTPN1, PURA, PUS1, PUS3, PUS7, PWP2, RAN, RANBP2, RANGAP1, RBM15, RBM25, RBM26, RBM3, RBM6, RBMS2, RBPMS, RBPMS2, RCC2, RDX, ROCK2, RPF2, RPL13A, RPL22, RPL27A, RPL4, RPL5, RPL7A, RPN1, RPS2, RPS21, RPS8, RRBP1, RRP12, RRP7A, RRS1, RTCA, SAMD4B, SAMHD1, SARS1, SARS2, SART3, SBDS, SERBP1, SERPINH1, SF3A3, SF3B1, SIN3A, SLC25A5, SLIRP, SLTM, SMARCA4, SNRNP200, SNRPA1, SNRPD3, SNTB2, SPATS2L, SPOUT1, SPTBN1, SRP14, SRPK1, SRPRA, SRSF1, SRSF11, SRSF7, SSB, SSBP1, SSRP1, SUCLG1, SUPT16H, SUPT5H, SUPT6H, TBL2, TBL3, TBRG4, TCP1, TFB1M, THUMPD3, TIA1, TIMM50, TNS1, TOP2A, TPD52L2, TRAP1, TRIM28, TRIM71, TRMT1, TRMT10C, TRMT1L, TSR1, TUT1, UBE2O, UBFD1, UBR5, UPF2, UPF3B, UTP15, UTP18, UTP20, UTP25, UTP4, WDR3, WDR36, WDR43, WRAP53, XPO5, XRCC5, XRCC6, XRN1, XRN2, YARS2, YRDC, YTHDC2, ZC3H15, ZC3H7B, ZC3HAV1, ZFP36L2, ZNF638]	
macromolecule biosynthetic process	GO_BiologicalProcess-EBI-UniProt-GOA-ACAP-ARAP_13.05.2021_00h00	1.49E-64	9.076923	472	[AASS, AATF, ABCB10, ABCF1, ABHD14B, ACO1, ACTN1, ACTN4, ACTR2, AEBP2, AGL, AGO1, AGO2, AIMP2, AKT1, ALG11, ALG2, ALG5, ALG9, ANKRD28, ANTXR1, ANXA3, ANXA4, AP2A1, AP3B1, APEH, APOE, ARF4, ARFGEF1, ARHGEF11, ATF7IP, ATP2B4, ATR, AURKB, B3GLCT, B4GALT1, BAG3, BAZ1B, BPTF, BRD3, BRD4, BRMS1, BRWD1, C1QBP, CACYBP, CALR, CAMK1, CAMK2D, CAPRIN1, CASK, CAT, CAV1, CAVIN1, CBX2, CBX5, CCAR1, CCAR2, CCDC22, CCNB1, CCNH, CCNL2, CCT2, CCT3, CCT4, CCT5, CCT6A,	Upregulation

					CCT7, CCT8, CD81, CDC123, CDC42, CDC73, CDK5RAP1, CDK5RAP3, CDK7, CEBPZ, CELF1, CHAF1A, CHD1, CHD4, CHEK1, CHEK2, CHPF, CHST14, CHTF18, CIRBP, CNOT11, COG7, COL1A1, COL4A2, COL5A1, COPS2, COPS5, CPSF2, CPSF3, CRLF3, CSDE1, CSPG4, CSTF1, CSTF3, CTBP2, CTCF, CTNNB1, CYFIP1, DAPK1, DARS2, DAXX, DCAF1, DDRGK1, DDX20, DDX21, DDX39B, DDX5, DDX54, DDX6, DHPS, DHX33, DHX9, DICER1, DLG1, DLGAP5, DNMT1, DNMT3A, DNMT3B, DPH1, DPH2, DPH6, DRG1, EDEM3, EDRF1, EEF1E1, EEF2, EFL1, EGFR, EHMT1, EIF3A, EIF4A3, EIF5B, ELP3, EMSY, EPCAM, EPM2AIP1, ERBIN, ERCC2, EXD2, EXOSC10, EXOSC2, EXOSC5, EXOSC7, FASTKD2, FOXK1, FUT11, FXR1, G3BP2, GALNT1, GALNT10, GALNT2, GARS1, GATA6, GATAD2A, GBE1, GCLC, GEMIN5, GFPT2, GINS3, GLUL, GNL3, GOLGA2, GPC1, GPC3, GPC6, GPI, GTF2E1, GTF2I, GTF3C1, GTF3C2, GTF3C3, GTF3C4, GTPBP1, GUF1, GXYLT1, GYS1, HAT1, HCFC1, HDAC2, HDAC4, HEATR1, HELLS, HEXA, HMOX1, HP1BP3, HSD17B4, HSP90AB1, HSPA5, HSPB1, HSPD1, HSPH1, IGF2BP3, ILF2, ILF3, ILK, IRF2BPL, IRF3, ITCH, ITGA6, IWS1, JARID2, KANK2, KAT7, KDM1A, KDM2A, KDM3B, KEAP1, LANCL2, LARS1, LARS2, LIG1, LIG3, LIMS1, LIN28A, LMAN1, LMCD1, LRPPRC, LRWD1, LSM4, LYAR, MACROH2A1, MAGED1, MAN1A1, MAN1A2, MAP2K6, MAPK1, MAPK14, MAPK8, MARS1, MAVS, MBD3, MCM2, MCM3, MCM4, MCM5, MCM6, MCM7, MCMBP, METAP1, MGAT2, MLH1, MMAB, MOGS, MRPS27, MRPS9, MSH3, MTA3, MTDH, MVD, MYBBP1A, MYD88, MYDGF, MYEF2, MYO1C, NCAPG2, NCL,	

					NDRG1, NEDD4, NEK7, NFKB1, NIBAN1, NIBAN2, NIF3L1, NMT2, NOL11, NPM1, NPM3, NSUN5, NUP107, NUP35, NVL, OGT, ORC2, ORC3, ORC4, ORC5, OSTC, PABPN1, PARN, PARP1, PASK, PATZ1, PAWR, PAXBP1, PDGFRB, PDS5A, PELP1, PFKM, PGM2L1, PGM3, PHC1, PHIP, PIAS4, PICALM, PIGS, PIGT, PKM, PLK1, PLOD1, PLOD2, PLOD3, PLPP3, PLXNB2, PNPT1, POFUT1, POFUT2, POGLUT2, POGLUT3, POGZ, POLA1, POLA2, POLB, POLD1, POLE, POLR1B, POLR2A, POLR2B, POLR2G, POLR3A, POLR3C, POLR3F, PPID, PPM1B, PPM1F, PRIM1, PRIM2, PRKAA1, PRKAR1A, PRKCSH, PRKDC, PSIP1, PSMC6, PSMD1, PSMD2, PSMD9, PSME3, PSPC1, PTCD3, PTGIS, PTPN2, PURA, PUS7, PXDN, PYCARD, QRSL1, RAD51, RAP2C, RBM15, RBM3, RBPJ, RBPMS, RELA, RFC1, RFC2, RFC3, RFC5, RIF1, RIOX1, ROCK2, RPA1, RPA2, RPA3, RPL13A, RPL22, RPL27A, RPL4, RPL5, RPL7A, RPN1, RPRD1A, RPS2, RPS21, RPS6KA1, RPS6KA3, RPS6KA4, RPS8, RRBP1, S100A11, SALL2, SAMD4B, SAMHD1, SAP30BP, SARS1, SARS2, SDF2L1, SESN2, SET, SETD7, SETDB1, SIN3A, SIRT1, SIRT2, SLC2A10, SLTM, SMARCA4, SMARCA5, SMARCC1, SMARCD1, SMARCD2, SNRPD3, SNX6, SORBS1, SORBS3, SRC, SSBP1, SSRP1, STT3A, STT3B, STXBP2, SUPT16H, SUPT5H, SUPT6H, SYMPK, TARS2, TAX1BP1, TBPL1, TCP1, TDP2, TELO2, TF, TFB1M, TGFB1I1, THBS1, TIA1, TMED2, TMF1, TOP2A, TRAP1, TRIM28, TRIM33, TRIM71, TRIP13, TRMT10C, TTF2, UFL1, UGDH, UGGT1, UGGT2, UGP2, UPF3B, USP47, USP7, USP9X, UTP15, UTP4, VIM, VRTN, WAPL, WARS1, WDHD1, WDR18, WDR43, WRAP53, XRCC5,	

					XRCC6, XRN1, XRN2, YAP1, YARS2, ZC3H15, ZDHHC17, ZFP36L2, ZMYND8, ZNF217, ZNF281, ZNF462]	
gene expression	GO_BiologicalProcess-EBI-UniProt-GOA-ACAP-ARAP_13.05.2021_00h00	1.49E-64	9.040196	551	[AAAS, AASS, AATF, ABCF1, ABHD14B, ACIN1, ACO1, ACTN1, ACTN4, ACTR2, ADAM10, ADAR, ADARB1, AEBP2, AGO1, AGO2, AIMP2, AKT1, ANK2, ANK3, ANXA1, ANXA3, ANXA4, AP3B1, APEH, APOE, APPL2, ARF4, ARHGEF11, ASPH, ATF7IP, ATP2B1, ATP2B4, AURKA, AURKB, BAG2, BAG3, BAZ1B, BMS1, BOP1, BPTF, BRD3, BRD4, BRMS1, BRWD1, C1QBP, CALR, CAMK1, CAMK2D, CAPN2, CAPRIN1, CARHSP1, CASK, CASP3, CAST, CAT, CAV1, CAVIN1, CBX2, CBX5, CCAR1, CCAR2, CCDC22, CCNB1, CCNH, CCNL2, CD2AP, CD81, CDC123, CDC73, CDH3, CDK5RAP1, CDK5RAP3, CDK7, CEBPZ, CELF1, CHD1, CHD4, CHEK1, CHEK2, CHID1, CIRBP, CNOT11, CNRIP1, COL1A1, COL4A2, COPS2, COPS5, CPD, CPSF2, CPSF3, CPZ, CRLF3, CSDE1, CSTF1, CSTF3, CTBP2, CTCF, CTNNB1, CTNNBL1, CWC27, CYFIP1, DAPK1, DARS2, DAXX, DAZAP1, DCAF1, DCAF13, DDRGK1, DDX10, DDX18, DDX20, DDX21, DDX39B, DDX41, DDX42, DDX46, DDX47, DDX49, DDX5, DDX52, DDX54, DDX6, DHPS, DHX15, DHX33, DHX37, DHX8, DHX9, DICER1, DLG1, DLGAP5, DNMT1, DNMT3A, DNMT3B, DPH1, DPH2, DPH6, DRG1, ECE1, EDRF1, EEF1E1, EEF2, EFL1, EFTUD2, EGFR, EHMT1, EIF3A, EIF4A3, EIF5B, ELAC2, ELP1, ELP3, EMSY, EPB41L5, EPCAM, ERBIN, ERCC2, ERO1A, ESF1, EXOSC10, EXOSC2, EXOSC5, EXOSC7, F11R, FASTKD2, FGG, FKBP8, FN1, FOXK1, FTSJ3, FXR1, G3BP1, G3BP2, GALNT2, GARS1, GATA6, GATAD2A, GCLC, GEMIN4, GEMIN5, GLMN, GNL3, GPI, GSN, GTF2E1,	Upregulation

					GTF2I, GTF3C1, GTF3C2, GTF3C3, GTF3C4, GTPBP1, GUF1, HAT1, HCFC1, HDAC2, HDAC4, HEATR1, HELLS, HK1, HLA-A, HMOX1, HNRNPF, HNRNPL, HNRNPLL, HNRNPM, HP1BP3, HSD17B10, HSD17B4, HSP90AB1, HSPA5, HSPB1, HSPD1, HUWE1, IGF2BP3, ILF2, ILF3, ILK, IMP3, IQGAP3, IRF2BPL, IRF3, ITCH, ITGA6, ITGAV, IWS1, JARID2, KANK2, KAT7, KDM1A, KDM2A, KDM3B, KEAP1, LANCL2, LARS1, LARS2, LAS1L, LIMS1, LIN28A, LMCD1, LMF2, LRP1, LRPPRC, LSM4, LTBP1, LYAR, MACROH2A1, MAGED1, MAPK1, MAPK14, MAPK8, MARS1, MATR3, MAVS, MBD3, MBNL1, MCM2, MDN1, METAP1, METTL1, MIPEP, MLH1, MME, MPHOSPH10, MRPS27, MRPS9, MSH2, MSH3, MSH6, MSN, MTA3, MTDH, MTREX, MYADM, MYBBP1A, MYD88, MYDGF, MYEF2, MYH9, MYO1C, MYO1E, NBAS, NCAPG2, NCL, NDC1, NDRG1, NDRG2, NEDD4, NFKB1, NIBAN1, NIBAN2, NIF3L1, NOL10, NOL11, NOL6, NOL9, NOP14, NOP56, NOP58, NOP9, NPM1, NPM3, NSUN2, NSUN5, NUDT16, NUP107, NUP133, NUP160, NUP210, NUP35, NUP50, NVL, OCLN, OGT, ORC2, ORC3, PABPN1, PARN, PARP1, PASK, PATZ1, PAWR, PAXBP1, PC, PCBP2, PDCD11, PDCL3, PELP1, PES1, PFKM, PHC1, PHIP, PIAS4, PICALM, PIK3CA, PITRM1, PKM, PLD3, PLK1, PLPP3, PLXNB2, PNP, PNPT1, POFUT1, POFUT2, POGZ, POLA1, POLB, POLR1B, POLR2A, POLR2B, POLR2G, POLR3A, POLR3C, POLR3F, PPID, PPM1B, PPM1F, PPP1R9B, PRDX4, PRKAA1, PRKAB1, PRKACB, PRKAG1, PRKAR1A, PRKCA, PRKCSH, PRKDC, PRORP, PRPF38A, PRPF38B, PRPF40A, PRPF8, PSIP1, PSMC6, PSMD1, PSMD2, PSMD9, PSME3, PSPC1,	

					PTBP2, PTCD1, PTCD3, PTGIS, PTPN2, PURA, PUS1, PUS3, PUS7, PWP2, PXDN, PYCARD, QRSL1, RAB1A, RAN, RANBP2, RAP2C, RBM15, RBM25, RBM26, RBM3, RBM6, RBMS2, RBPJ, RBPMS, RDX, RELA, RFC1, RFTN1, RIF1, RIOX1, RNH1, ROCK2, RPF2, RPL13A, RPL22, RPL27A, RPL4, RPL5, RPL7A, RPRD1A, RPS2, RPS21, RPS6KA1, RPS6KA3, RPS6KA4, RPS8, RRBP1, RRP12, RRP7A, RRS1, RTCA, SALL2, SAMD4B, SAMHD1, SAP30BP, SARS1, SARS2, SART3, SBDS, SEC13, SEH1L, SERBP1, SERPINB9, SERPINH1, SESN2, SET, SETD7, SETDB1, SF3A3, SF3B1, SIN3A, SIRT1, SIRT2, SLC2A10, SLTM, SMARCA4, SMARCA5, SMARCC1, SMARCD1, SMARCD2, SNRNP200, SNRPA1, SNRPD3, SNX6, SORBS3, SORD, SPON1, SPOUT1, SPTBN1, SRC, SRPK1, SRSF1, SRSF11, SRSF7, SSB, SSRP1, STXBP2, SUPT16H, SUPT5H, SUPT6H, SYMPK, TARS2, TAX1BP1, TBL3, TBPL1, TBRG4, TDP2, TELO2, TEX10, TF, TFB1M, TGFB1I1, THBS1, THUMPD3, TIA1, TMED10, TMED2, TMF1, TOP2A, TRAP1, TRIM28, TRIM33, TRIM71, TRIP13, TRMT1, TRMT10C, TRMT1L, TRMT5, TRPT1, TSEN34, TSR1, TTF2, TUT1, UFL1, UNG, UPF2, UPF3B, USP47, USP7, USP9X, UTP15, UTP18, UTP20, UTP25, UTP4, VAMP3, VIM, VPS35, VRTN, WARS1, WDR18, WDR3, WDR36, WDR43, XPO5, XRCC5, XRCC6, XRN1, XRN2, YAP1, YARS2, ZC3H15, ZC3H7B, ZC3HAV1, ZFP36L2, ZMYND8, ZNF217, ZNF281, ZNF462, ZNF638]	
positive regulation of macromolecule metabolic process	GO_BiologicalProcess-EBI-UniProt-GOA-ACAP-ARAP_13.05.2021_00h00	1.49E-64	9.748771	357	[AATF, ABCB10, ABHD14B, ABI1, ACSL1, ACTN1, ACTN4, ACTR2, ADAR, AGO1, AGO2, AGTPBP1, AIMP2, AKT1, ANK2, ANK3, ANTXR1, ANXA1, ANXA2, ANXA3, AP2M1, AP3B1, APAF1, APOE, ARAP1, ARF4,	No change

					ARFGEF1, ARHGEF11, ARL6IP5, ASPH, ATF7IP, ATP2B4, ATR, AURKA, AURKB, BAG2, BAZ1B, BPTF, BRAT1, BRD4, BRMS1, C1QBP, CACYBP, CALR, CAMK1, CASK, CASP3, CAV1, CCAR1, CCAR2, CCDC22, CCNB1, CCND2, CCNY, CCT2, CCT3, CCT4, CCT5, CCT6A, CCT7, CCT8, CD44, CD81, CDC123, CDC42, CDC73, CDH3, CDK5RAP1, CDK5RAP3, CDK7, CEBPZ, CELF1, CHEK1, CHEK2, CHTF18, CIRBP, CNOT11, COL1A1, COPS5, CRLF3, CSPG4, CTBP2, CTCF, CTNNB1, CTSC, CYFIP1, DAPK1, DAXX, DAZAP1, DBNL, DDRGK1, DDX21, DDX39B, DDX41, DDX5, DDX6, DHX33, DHX9, DIPK2A, DLG1, DLGAP5, DNMT1, DNMT3B, DOCK7, ECE1, ECT2, EDRF1, EEF2, EGFR, EHD4, EIF4A3, EPCAM, EPM2AIP1, ERCC2, ETFA, EXOSC2, EXOSC5, EXOSC7, FASTKD2, FBXW8, FERMT2, FLT1, FN1, FOXK1, FXR1, G3BP1, GARS1, GATA6, GCLC, GLMN, GNL3, GOLGA2, GPC3, GPI, GSN, GTF2I, GTPBP1, GUF1, HCFC1, HDAC2, HDAC4, HEATR1, HK1, HLA-A, HMOX1, HNRNPLL, HSD17B4, HSP90AB1, HSPA2, HSPA5, HSPB1, HSPD1, HSPE1, HSPH1, HUWE1, ILF2, ILF3, ILK, INCENP, IQGAP1, IQGAP3, IRF2BPL, IRF3, IST1, ITCH, ITGA5, ITGA6, JARID2, KAT7, KDM1A, KEAP1, LIMS1, LIN28A, LPCAT1, LRP1, LRRK1, LSM4, LYAR, MACROH2A1, MAGED1, MAP2K6, MAPK1, MAPK14, MAPK8, MARS1, MAVS, MLH1, MMAB, MRPS27, MSH2, MSN, MTA3, MTDH, MYBBP1A, MYD88, MYDGF, MYH9, MYO1C, MYO1E, NBAS, NCL, NEDD4, NEK7, NFKB1, NIBAN1, NIBAN2, NIF3L1, NOL11, NPM1, NSF, NSUN5, NVL, OCLN, OGT, ORC3, OSBP, PAK1, PARN, PARP1, PASK, PATZ1, PAWR, PAXBP1, PCOLCE, PDCD2, PDCL3, PDGFRB, PEA15, PELP1,	

					PFKM, PHIP, PIAS4, PICALM, PIK3CA, PKM, PLK1, PLXNB2, PNP, PNPT1, POGZ, POLR1B, POLR2A, POLR2G, POLR3A, POLR3C, POLR3F, PPM1F, PPP2R5A, PRKAA1, PRKAB1, PRKACB, PRKACG, PRKAG1, PRKAR1A, PRKAR2A, PRKAR2B, PRKCA, PRKCSH, PRKDC, PRRC1, PSMC6, PSMD9, PSME3, PTPN1, PYCARD, RAB1A, RAD51, RAP2A, RAP2B, RAP2C, RBM15, RBM3, RBPJ, RBPMS, RDX, RELA, RFC1, RFC2, RFC3, RFC5, RFTN1, RIF1, ROCK2, RPL5, RPS2, RPS6KA1, RPS6KA3, RPS6KA4, SALL2, SAMD4B, SART3, SEC22B, SERPINB9, SESN2, SETD7, SETDB1, SF3B1, SIN3A, SIRT1, SIRT2, SKP2, SLC2A10, SMARCA4, SMARCA5, SMARCC1, SMARCD1, SMARCD2, SNX9, SORBS1, SORD, SPAG9, SPON1, SPTBN1, SRC, SRSF1, SSBP1, STX5, STXBP2, SUPT16H, SUPT5H, SUPT6H, SYMPK, TCP1, TELO2, TF, TGFB1I1, THBS1, TIGAR, TMED10, TMED2, TMF1, TOLLIP, TOP2A, TPX2, TRIM28, TRIM71, TRMT10C, TTK, UPF3B, USP5, USP7, USP9X, UTP15, VAMP3, VIM, VLDLR, VPS35, WARS1, WDR43, WRAP53, XRCC5, XRCC6, YAP1, ZC3HAV1, ZFP36L2, ZNF281, ZNF462]	
negative regulation of macromolecule metabolic process	GO_BiologicalProcess-EBI-UniProt-GOA-ACAP-ARAP_13.05.2021_00h00	1.49E-64	10.21851	318	[AAAS, AASS, AATF, ADAM10, ADAR, ADARB1, AEBP2, AGO1, AGO2, AKT1, ANTXR1, ANXA1, ANXA2, ANXA4, AP2A1, APOE, APPL2, ATF7IP, ATP2B1, ATP2B4, ATR, AURKA, AURKB, BAG2, BAG3, BIN1, BIRC6, BRMS1, C1QBP, CALR, CAPRIN1, CARHSP1, CASP3, CAST, CAV1, CBL, CBX2, CBX5, CCAR1, CCAR2, CCNB1, CD2AP, CD44, CDC73, CDH3, CDK5RAP1, CDK5RAP3, CELF1, CHID1, CIRBP, CNOT11, COL6A3, COPS2, CORO1C, CRTAP, CSDE1, CTBP2, CTCF, CTNNB1, CYP51A1, DAPK1, DAXX, DCAF1, DDRGK1, DDX20, DDX5,	Upregulation

					DDX54, DDX6, DHX9, DICER1, DLG1, DNMT1, DNMT3A, DNMT3B, DUSP3, EGFR, EHMT1, EIF4A3, ERBIN, EXOSC10, EXOSC2, EXOSC5, EXOSC7, FASTKD2, FKBP8, FN1, FOXK1, FXR1, GARS1, GATA6, GATAD2A, GCLC, GIPC1, GLMN, GPC3, GPD1L, GPI, GTPBP1, HAT1, HCFC1, HDAC2, HDAC4, HELLS, HMOX1, HNRNPM, HSP90AB1, HSPB1, IGF2BP3, ILF3, ILK, IPO5, IQGAP3, IRF2BPL, IRF3, ITCH, ITGAV, JARID2, KANK2, KAT7, KDM1A, KDM2A, LANCL2, LIG3, LIMS1, LIN28A, LMCD1, LRP1, LRPPRC, LRRK1, LSM4, LYAR, MACROH2A1, MAGED1, MAPK14, MASTL, MAVS, MBD3, METAP1, MICAL1, MLH1, MMAB, MSH2, MSH3, MSH6, MTA3, MTDH, MYADM, MYBBP1A, MYD88, MYEF2, MYO1C, NBAS, NCL, NDC1, NDRG2, NEDD4, NFKB1, NIBAN1, NIBAN2, NIF3L1, NLE1, NNT, NPM1, NSUN2, NUDT16, NUP107, NUP133, NUP160, NUP210, NUP35, NUP50, NXN, OCLN, OGT, OPTN, ORC2, P3H1, PARD3, PARN, PARP1, PARVA, PASK, PATZ1, PAWR, PC, PCBP2, PDS5A, PHC1, PIAS4, PICALM, PLK1, PLPP3, PNPT1, POLR2A, POLR2B, POLR2G, POR, PPID, PPM1B, PPM1F, PPP1R9B, PRKAA1, PRKAR1A, PRKAR2A, PRKAR2B, PRKCA, PRKDC, PRMT3, PSMC6, PSMD1, PSMD2, PSMD9, PSME3, PSPC1, PTBP2, PTPN1, PTPN2, PURA, PUS7, PYCARD, RAN, RANBP2, RBM15, RBPJ, RELA, RFC1, RIC1, RIF1, RIOX1, RNH1, ROCK2, RPL13A, RPL22, RPL27A, RPL4, RPL5, RPL7A, RPS2, RPS21, RPS6KA1, RPS6KA3, RPS6KA4, RPS8, S100A11, SALL2, SAMD4B, SARS1, SEC13, SEH1L, SERBP1, SERPINB6, SERPINB9, SERPINH1, SESN2, SET, SETDB1, SIN3A, SIRT1, SIRT2, SLC2A10, SLIRP, SMARCA4, SMARCA5,	

					SMARCC1, SNX3, SNX6, SORBS3, SORD, SPAG9, SPON1, SPOUT1, SRC, SRSF7, SSB, STK38, STYX, SUPT5H, SUPT6H, TAX1BP1, TBRG4, TDP2, THBS1, TIA1, TIMP3, TIPRL, TMED10, TMED2, TMF1, TRIM28, TRIM33, TRIM71, TRIP12, TUT1, UBE2O, UBR5, UFL1, UPF2, UPF3B, USP19, USP47, USP7, USP9X, VIM, VPS25, WAPL, WARS1, XPO5, XRCC5, XRCC6, XRN1, XRN2, YAP1, ZC3H7B, ZC3HAV1, ZFP36L2, ZMYND8, ZNF217, ZNF281]	
phosphotransferase activity, alcohol group as acceptor	GO_MolecularFunction-EBI-UniProt-GOA-ACAP-ARAP_13.05.2021_00h00	1.49E-64	11.04856	157	[ABI1, ACSL1, ADAR, ADARB1, ADPGK, AKT1, ANTXR1, APOE, ATP2B4, ATR, AURKA, AURKB, BAZ1B, BCCIP, BUB1B, CAMK1, CAMK2D, CASK, CASP3, CAV1, CBL, CCNB1, CCND2, CCNH, CCNL2, CCNY, CD81, CDC42BPA, CDC42BPB, CDK5RAP1, CDK5RAP3, CDK7, CHEK1, CHEK2, CLASP1, CORO1C, CSPG4, DAPK1, DAXX, DBNL, DCAF1, DGKA, DIPK2A, DLG1, DUSP3, ECT2, EGFR, ELP3, EPHB3, ETFA, ETNK1, FERMT2, FLT1, GALK1, GNE, GPI, HK1, HK2, HOOK3, HSP90AB1, HSPB1, HTT, ILK, INCENP, IPO5, IQGAP1, IQGAP3, ITPK1, LRRK1, LTBP1, MACROH2A1, MAGED1, MAP2K6, MAPK1, MAPK14, MAPK8, MASTL, MCM2, MMAB, MVK, NEK7, NOL9, NPM1, NRP2, ORC3, PAK1, PAPSS2, PARVA, PASK, PDGFRB, PDXK, PEA15, PFKL, PFKM, PFKP, PGM2L1, PI4KA, PIK3C2A, PIK3CA, PIK3R4, PIP4K2B, PIP4K2C, PKM, PLK1, PPM1F, PPP1R9B, PRKAA1, PRKAB1, PRKACB, PRKACG, PRKAG1, PRKAR1A, PRKAR2A, PRKAR2B, PRKCA, PRKDC, PRRC1, PTK7, PTPN1, PTPN2, PXK, PYCARD, RANBP2, RAP2B, RAP2C, ROCK2, RPS6KA1, RPS6KA3, RPS6KA4, SCYL1, SESN2, SHPK, SIRT1, SLK, SNX6, SNX9, SPAG9, SRC, SRPK1, STK38, TELO2, TF, THBS1, TIGAR, TKFC, TLK1, TPX2, TRIM28, TRPT1, TTK, TWF1,	No change

					VLDLR, VPS25, VRK1, WARS1, XRCC5, XRCC6]	
negative regulation of cellular metabolic process	GO_BiologicalProcess-EBI-UniProt-GOA-ACAP-ARAP_13.05.2021_00h00	1.49E-64	9.68444	267	[AASS, AATF, ABCB7, ADAR, ADARB1, AEBP2, AGO1, AGO2, AKT1, ANTXR1, ANXA2, APOE, APPL2, ATF7IP, ATP1A1, ATP2B4, ATR, AURKB, BAG2, BAG3, BIN1, BIRC6, BRMS1, C1QBP, CALR, CAPRIN1, CASP3, CAST, CAV1, CBL, CBX2, CBX5, CCAR1, CCAR2, CCNB1, CD44, CDC73, CDK5RAP1, CDK5RAP3, CELF1, CIRBP, CNOT11, COL6A3, COPS2, CORO1C, CRTAP, CTBP2, CTCF, CTNNB1, DAPK1, DAXX, DCAF1, DDRGK1, DDX20, DDX5, DDX54, DDX6, DHX9, DICER1, DLG1, DNMT1, DNMT3A, DNMT3B, DUSP3, EHMT1, EIF4A3, ERLIN2, EXOSC10, EXOSC2, EXOSC5, EXOSC7, FKBP8, FOXK1, FXR1, GATA6, GATAD2A, GCLC, GIPC1, GLA, GLMN, GOLGA2, GPC3, GPD1L, GPI, HAT1, HCFC1, HDAC2, HDAC4, HELLS, HK2, HMOX1, HSP90AB1, HSPB1, HSPD1, IGF2BP3, ILF3, ILK, IPO5, IRF2BPL, IRF3, ITGAV, JARID2, KANK2, KAT7, KDM1A, KDM2A, LANCL2, LIG3, LIMS1, LIN28A, LMCD1, LPCAT1, LRP1, LRPPRC, LRRK1, LSM4, LYAR, MACROH2A1, MAGED1, MAPK14, MASTL, MBD3, METAP1, MICAL1, MLH1, MMAB, MSH2, MSH3, MSH6, MTA3, MTDH, MYADM, MYBBP1A, MYD88, NBAS, NCL, NEDD4, NFKB1, NIBAN1, NIBAN2, NIF3L1, NLE1, NNT, NPM1, NSUN2, NUDT16, NXN, OCLN, OGT, OPTN, ORC2, P3H1, PARD3, PARN, PARP1, PARVA, PASK, PATZ1, PAWR, PDS5A, PHC1, PIAS4, PICALM, PIK3CA, PIP4K2B, PIP4K2C, PLK1, PLPP3, PNPT1, POLR2A, POLR2B, POLR2G, POR, PPID, PPM1F, PPP1R9B, PPP2R5A, PRKAA1, PRKAR1A, PRKAR2A, PRKAR2B, PRKDC, PRMT3, PSPC1, PTBP2, PTGIS, PTPN1, PTPN2, PURA, PUS7, PYCARD, RBM15, RBPJ, RELA, RFC1, RIC1, RIF1, RIOX1, ROCK2, RPL13A, RPL5,	Upregulation

					RPS6KA1, RPS6KA3, S100A11, SALL2, SAMD4B, SARS1, SCFD1, SEC22B, SERPINB6, SERPINB9, SERPINH1, SESN2, SET, SETDB1, SIN3A, SIRT1, SIRT2, SLC25A12, SLC2A10, SLIRP, SMARCA4, SMARCA5, SMARCC1, SNX6, SORBS3, SORD, SPAG9, SPON1, SRC, SRSF7, STK38, STYX, SUPT5H, SUPT6H, TDP2, TELO2, THBS1, TIA1, TIGAR, TIMP3, TIPRL, TMED10, TMED2, TRAP1, TRIM28, TRIM33, TRIM71, TRIP12, UBE2O, UBR5, UFL1, USP19, USP47, USP7, USP9X, VPS25, WAPL, WARS1, XRCC5, XRCC6, XRN1, YAP1, ZFP36L2, ZMYND8, ZNF217, ZNF281]	
positive regulation of cellular metabolic process	GO_BiologicalProcess-EBI-UniProt-GOA-ACAP-ARAP_13.05.2021_00h00	1.49E-64	10.1779	349	[AATF, ABAT, ABCB10, ABCB7, ABHD14B, ABI1, ACACA, ACLY, ACSL1, ACTN1, ACTN4, ACTR2, AGO1, AGO2, AGTPBP1, AIMP2, AKT1, ANTXR1, ANXA1, ANXA2, ANXA3, AP2M1, AP3B1, APAF1, APOE, ARAP1, ARF4, ARFGEF1, ARHGEF11, ARL6IP5, ASPH, ATF7IP, ATP2B4, ATR, AURKA, AURKB, BAG2, BAG3, BPTF, BRAT1, BRD4, BRMS1, C1QBP, CACYBP, CALCOCO2, CAMK1, CAPN2, CASK, CASP3, CAV1, CBR1, CCAR1, CCAR2, CCDC22, CCNB1, CCND2, CCNY, CCT2, CCT3, CCT4, CCT5, CCT6A, CCT7, CCT8, CD44, CD81, CDC123, CDC42, CDC73, CDH3, CDK5RAP1, CDK5RAP3, CDK7, CEBPZ, CELF1, CHEK1, CHEK2, CHTF18, CIRBP, CNOT11, COL1A1, COPS5, CRLF3, CSPG4, CTBP2, CTCF, CTNNB1, CTSC, CYFIP1, DAGLB, DAPK1, DAXX, DAZAP1, DBNL, DCXR, DDRGK1, DDX39B, DDX5, DDX6, DHX33, DHX8, DHX9, DIPK2A, DLG1, DLGAP5, DNMT1, DNMT3B, DOCK7, ECE1, ECT2, EDRF1, EEF2, EGFR, EHD4, EIF4A3, EPCAM, EPHB3, EPM2AIP1, ERCC2, ETFA, EXOSC2, EXOSC5, EXOSC7, FASTKD2, FBXW8, FERMT2, FLT1, FN1, FOXK1, FXR1, GARS1, GATA6, GCLC,	No change

					GIPC1, GLMN, GNL3, GOLGA2, GPSM1, GSN, GTF2I, GTPBP1, GUF1, HCFC1, HDAC2, HDAC4, HEATR1, HMOX1, HNRNPLL, HSD17B4, HSP90AB1, HSPA2, HSPA5, HSPD1, HSPE1, HTT, HUWE1, ILF2, ILF3, ILK, INCENP, IQGAP1, IQGAP3, IRF2BPL, IRF3, IST1, ITCH, ITGA5, ITGA6, JARID2, KAT7, KDM1A, KEAP1, LIN28A, LPGAT1, LRP1, LRRK1, LSM4, LYAR, MAGED1, MAP2K6, MAPK1, MAPK14, MARS1, MAVS, MLH1, MMAB, MRPS27, MSH2, MSN, MTA3, MTDH, MYDGF, MYH9, MYO1C, NCL, NEK7, NFKB1, NIBAN1, NIBAN2, NIF3L1, NNT, NOL11, NPM1, NQO2, NSF, NSUN5, NVL, OGT, OPTN, ORC3, OSBP, PAK1, PARD3, PARN, PARP1, PASK, PATZ1, PAWR, PAXBP1, PCOLCE, PDCD2, PDGFRB, PEA15, PELP1, PFKM, PHIP, PIAS4, PICALM, PIK3C2A, PIK3CA, PIK3R4, PIP4K2B, PIP4K2C, PKM, PLK1, PLXNB2, PNPT1, POGZ, POLR2A, POLR2G, POR, PPM1F, PPP2R5A, PRKAA1, PRKACB, PRKACG, PRKAG1, PRKAR1A, PRKAR2A, PRKAR2B, PRKCA, PRKCSH, PRKDC, PRRC1, PSMC6, PSMD9, PSME3, PTPN1, PTPN2, PYCARD, RAB1A, RAD51, RANBP2, RAP2A, RAP2B, RAP2C, RBM15, RBM3, RBPJ, RBPMS, RDH10, RDX, RELA, RFC1, RFC2, RFC3, RFC5, RIF1, ROCK2, RPL5, RPS2, RPS6KA1, RPS6KA3, RPS6KA4, SALL2, SAMD4B, SART3, SESN2, SETD7, SH3GLB1, SIN3A, SIRT1, SIRT2, SKP2, SLC25A12, SLC25A5, SLC2A10, SLC5A3, SMARCA4, SMARCA5, SMARCC1, SMARCD1, SMARCD2, SNX9, SORBS1, SORD, SPAG9, SPON1, SRC, SRSF1, SSBP1, STXBP2, SUPT16H, SUPT5H, SUPT6H, SYMPK, TCP1, TELO2, TF, TGFB1I1, THBS1, TIGAR, TMF1, TOLLIP, TOP2A, TPX2, TRIM28, TRIM71, TRMT10C, TTK, UFL1, UPF3B, USP5, USP7,	

					USP9X, UTP15, VAMP3, VIM, VLDLR, VPS35, WDR43, WRAP53, XRCC5, XRCC6, YAP1, ZC3HAV1, ZFP36L2, ZNF281, ZNF462]	
cellular nitrogen compound biosynthetic process	GO_BiologicalProcess-EBI-UniProt-GOA-ACAP-ARAP_13.05.2021_00h00	1.49E-64	8.94706	458	[AASS, AATF, ABCB10, ABCB7, ABCF1, ABHD14B, ACACA, ACAT1, ACLY, ACO1, ACSL1, ACSL4, ACSS2, ACTN1, ACTN4, ACTR2, ADSL, AEBP2, AGO1, AGO2, AIMP2, AK1, AK4, AKT1, ALDH7A1, AMDHD2, AMPD2, ANKRD28, ANTXR1, ANXA3, ANXA4, AP3B1, APEH, APOE, APRT, ARF4, ARHGEF11, ASL, ASNS, ATF7IP, ATIC, ATP2B4, ATP6V1A, ATR, AURKB, BAG3, BAZ1B, BDH2, BPTF, BRD3, BRD4, BRMS1, BRWD1, C1QBP, CALR, CAMK1, CAMK2D, CAPRIN1, CASK, CAT, CAV1, CAVIN1, CBX2, CBX5, CCAR1, CCAR2, CCDC22, CCNB1, CCNH, CCNL2, CCT2, CCT3, CCT4, CCT5, CCT6A, CCT7, CCT8, CD81, CDC123, CDC73, CDK5RAP1, CDK5RAP3, CDK7, CEBPZ, CELF1, CHD1, CHD4, CHEK1, CHEK2, CHTF18, CIRBP, CMPK1, CNDP2, CNOT11, COL1A1, COL4A2, COPS2, COPS5, CPOX, CPSF2, CPSF3, CRLF3, CSDE1, CSTF1, CSTF3, CTBP2, CTCF, CTNNB1, CTPS1, CYB5R3, CYFIP1, DAPK1, DARS2, DAXX, DCAF1, DCTD, DDRGK1, DDX20, DDX21, DDX39B, DDX5, DDX54, DDX6, DEGS1, DHPS, DHX33, DHX9, DICER1, DLG1, DLGAP5, DNMT1, DNMT3A, DNMT3B, DPH1, DPH2, DPH6, DRG1, EDRF1, EEF1E1, EEF2, EFL1, EGFR, EHMT1, EIF3A, EIF4A3, EIF5B, ELP3, EMSY, EPCAM, ERBIN, ERCC2, EXOSC10, EXOSC2, EXOSC5, EXOSC7, FASTKD2, FECH, FLAD1, FOXK1, FXR1, G3BP2, GARS1, GART, GATA6, GATAD2A, GCDH, GCLC, GCLM, GEMIN5, GFPT2, GLA, GMPPB, GMPS, GNL3, GNPDA2, GPI, GSS, GTF2E1, GTF2I, GTF3C1, GTF3C2, GTF3C3, GTF3C4, GTPBP1, GUF1, HAGH, HAT1, HCFC1, HDAC2,	Upregulation

					HDAC4, HEATR1, HELLS, HMBS, HMOX1, HP1BP3, HSD17B4, HSP90AB1, HSPA5, HSPB1, HSPD1, IDH2, IGF2BP3, ILF2, ILF3, ILK, IMPDH1, IMPDH2, IRF2BPL, IRF3, ITCH, ITGA6, IWS1, JARID2, KANK2, KAT7, KDM1A, KDM2A, KDM3B, KEAP1, LANCL2, LARS1, LARS2, LIG1, LIG3, LIMS1, LIN28A, LMCD1, LRPPRC, LSM4, LYAR, MACROH2A1, MAGED1, MAPK1, MAPK14, MAPK8, MARS1, MAVS, MBD3, MCM2, ME1, METAP1, MLH1, MMAB, MOXD1, MPC2, MRPS27, MRPS9, MTA3, MTAP, MTDH, MTHFD1, MYBBP1A, MYD88, MYDGF, MYEF2, MYO1C, NCAPG2, NCL, NDRG1, NEDD4, NEK7, NFKB1, NIBAN1, NIBAN2, NIF3L1, NOL11, NPM1, NPM3, NSUN5, NT5C2, NTHL1, NUDT16, NUP107, NUP35, NVL, OGT, OPLAH, ORC2, PABPN1, PAICS, PAPSS2, PARN, PARP1, PASK, PATZ1, PAWR, PAXBP1, PDGFRB, PDXK, PELP1, PFAS, PFKM, PGM3, PHC1, PHIP, PIAS4, PICALM, PKM, PLK1, PLPP3, PLXNB2, PNP, PNPT1, POFUT1, POGZ, POLA1, POLA2, POLB, POLD1, POLE, POLR1B, POLR2A, POLR2B, POLR2G, POLR3A, POLR3C, POLR3F, PPID, PPM1F, PRIM1, PRIM2, PRKAA1, PRKAR1A, PRKCSH, PRKDC, PRPSAP1, PSIP1, PSMC6, PSMD1, PSMD2, PSMD9, PSME3, PSPC1, PTCD3, PTGIS, PTPN2, PURA, PUS7, PXDN, PYCARD, QDPR, QRSL1, RAP2C, RBM15, RBM3, RBPJ, RBPMS, RELA, RFC1, RFC2, RFC3, RFC5, RIF1, RIOX1, ROCK2, RPA1, RPA2, RPA3, RPL13A, RPL22, RPL27A, RPL4, RPL5, RPL7A, RPRD1A, RPS2, RPS21, RPS6KA1, RPS6KA3, RPS6KA4, RPS8, RRBP1, SALL2, SAMD4B, SAMHD1, SAP30BP, SARM1, SARS1, SARS2, SESN2, SET, SETD7, SETDB1, SIN3A, SIRT1, SIRT2, SLC25A12, SLTM, SMARCA4, SMARCA5,	

					SMARCC1, SMARCD1, SMARCD2, SMS, SNRPD3, SNX6, SORBS3, SORD, SPR, SPTLC2, SRC, SRM, SSRP1, STXBP2, SUPT16H, SUPT5H, SUPT6H, SYMPK, TARS2, TAX1BP1, TBPL1, TCP1, TDP2, TELO2, TF, TFB1M, TGFB1I1, THBS1, TIA1, TJP2, TMED2, TMF1, TOP2A, TRAP1, TRIM28, TRIM33, TRIM71, TRIP13, TRMT10C, TTF2, UAP1, UFL1, UGDH, UGP2, UNG, UPF3B, USP47, USP7, USP9X, UTP15, UTP4, VIM, VRTN, WARS1, WDR43, WRAP53, XRCC5, XRCC6, XRN1, XRN2, YAP1, YARS2, ZC3H15, ZFP36L2, ZMYND8, ZNF217, ZNF281, ZNF462]	
positive regulation of nitrogen compound metabolic process	GO_BiologicalProcess-EBI-UniProt-GOA-ACAP-ARAP_13.05.2021_00h00	1.49E-64	9.675425	313	[AATF, ABAT, ABCB10, ABCB7, ABHD14B, ABI1, ACSL1, ACTN1, ACTN4, ACTR2, AGO1, AGO2, AGTPBP1, AIMP2, AKT1, ANTXR1, ANXA2, ANXA3, AP3B1, APAF1, APOE, ARF4, ARFGEF1, ARHGEF11, ARL6IP5, ASPH, ATF7IP, ATP2B4, ATR, AURKA, AURKB, BAG2, BPTF, BRAT1, BRD4, BRMS1, C1QBP, CAMK1, CAPN2, CASK, CASP3, CAV1, CCAR1, CCAR2, CCDC22, CCNB1, CCND2, CCNY, CCT2, CCT3, CCT4, CCT5, CCT6A, CCT7, CCT8, CD44, CD81, CDC123, CDC73, CDK5RAP1, CDK5RAP3, CDK7, CEBPZ, CELF1, CHEK1, CHEK2, CHTF18, CIRBP, CNOT11, COL1A1, COPS5, CRLF3, CSPG4, CTBP2, CTCF, CTNNB1, CTSC, CYFIP1, DAPK1, DAXX, DAZAP1, DBNL, DDRGK1, DDX39B, DDX5, DDX6, DHX33, DHX9, DIPK2A, DLG1, DLGAP5, DNMT1, DNMT3B, DOCK7, ECT2, EDRF1, EEF2, EGFR, EHD4, EIF4A3, EPCAM, ERCC2, ETFA, EXOSC2, EXOSC5, EXOSC7, FASTKD2, FBXW8, FERMT2, FLT1, FN1, FOXK1, FXR1, GARS1, GATA6, GCLC, GNL3, GOLGA2, GPC3, GSN, GTF2I, GTPBP1, GUF1, HCFC1, HDAC2, HDAC4, HEATR1, HNRNPLL, HSD17B4, HSP90AB1, HSPA2, HSPA5, HSPD1,	No change

					HSPE1, HUWE1, ILF2, ILF3, ILK, INCENP, IQGAP1, IQGAP3, IRF2BPL, IRF3, IST1, ITCH, ITGA5, ITGA6, JARID2, KAT7, KDM1A, KEAP1, LIN28A, LPCAT1, LRP1, LRRK1, LSM4, LYAR, MAGED1, MAP2K6, MAPK1, MAPK14, MAPK8, MARS1, MAVS, MLH1, MMAB, MRPS27, MSH2, MSN, MTA3, MTDH, MYDGF, MYH9, MYO1C, NCL, NEDD4, NEK7, NFKB1, NIBAN1, NIBAN2, NIF3L1, NOL11, NPM1, NSF, NSUN5, NVL, OGT, ORC3, OSBP, PAK1, PARN, PARP1, PASK, PATZ1, PAWR, PAXBP1, PCOLCE, PDCD2, PDGFRB, PEA15, PELP1, PFKM, PHIP, PIAS4, PICALM, PIK3CA, PKM, PLK1, PLXNB2, PNPT1, POGZ, POLR2A, POLR2G, PPM1F, PPP2R5A, PRKAA1, PRKACB, PRKACG, PRKAG1, PRKAR1A, PRKAR2A, PRKAR2B, PRKCA, PRKCSH, PRKDC, PRRC1, PSMC6, PSMD9, PSME3, PTPN1, PYCARD, RAB1A, RAD51, RAP2A, RAP2B, RAP2C, RBM15, RBM3, RBPJ, RBPMS, RDX, RELA, RFC1, RFC2, RFC3, RFC5, RIF1, ROCK2, RPL5, RPS2, RPS6KA1, RPS6KA3, RPS6KA4, SALL2, SAMD4B, SART3, SEC22B, SESN2, SETD7, SIN3A, SIRT1, SIRT2, SKP2, SLC25A12, SLC2A10, SMARCA4, SMARCA5, SMARCC1, SMARCD1, SMARCD2, SNX9, SORD, SPAG9, SPON1, SRC, SRSF1, STX5, STXBP2, SUPT16H, SUPT5H, SUPT6H, SYMPK, TCP1, TELO2, TF, TGFB1I1, THBS1, TIGAR, TMF1, TOLLIP, TOP2A, TPX2, TRIM28, TRIM71, TRMT10C, TTK, UPF3B, USP5, USP7, USP9X, UTP15, VIM, VLDLR, VPS35, WDR43, WRAP53, XRCC5, XRCC6, YAP1, ZC3HAV1, ZFP36L2, ZNF281, ZNF462]	
intracellular organelle lumen	GO_CellularComponent-EBI-UniProt-GOA-	1.49E-64	13.14335	761	[AAAS, AASS, AATF, ABAT, ABCF1, ABHD10, ABHD14B, ACAA1, ACACA, ACAD8, ACAT1, ACIN1, ACLY, ACOT9,	Upregulation

	ACAP-ARAP_13.05.2021_00h00				ACOX3, ACSS2, ACSS3, ACTBL2, ACTN1, ACTN4, ACTR10, ACTR1B, ACTR2, ADAM10, ADAR, ADARB1, ADD3, AEBP2, AGL, AGO1, AGO2, AGTPBP1, AK4, AKT1, ALDH1L2, ALDH2, ALDH7A1, ALDOC, ANKRD28, ANKS1A, ANP32E, ANTXR1, ANXA1, ANXA11, ANXA2, APAF1, APEH, API5, APOE, APOOL, APRT, ARAP1, ARFGEF1, ARL2, ARL3, ARSA, ARSB, ATF7IP, ATP2B1, ATP6V1A, ATR, AURKA, AURKB, BAIAP2, BAZ1B, BCAT2, BCCIP, BLVRB, BMS1, BOP1, BPNT2, BPTF, BRAT1, BRD4, BRMS1, BRWD1, C1QBP, CACYBP, CALCOCO2, CALR, CALU, CAMK2D, CANX, CAP1, CAPN1, CAPN2, CASK, CASP3, CAT, CAVIN1, CBX2, CBX5, CCAR1, CCAR2, CCDC22, CCNB1, CCND2, CCNH, CCNL2, CCT2, CCT4, CCT8, CD2AP, CDC73, CDCA8, CDH2, CDK5RAP3, CDK7, CELF1, CERCAM, CFAP20, CFL2, CHD1, CHD4, CHEK1, CHEK2, CHID1, CHPF, CHTF18, CIRBP, CKAP4, CKAP5, CMPK1, CNDP2, COG7, COL1A1, COL1A2, COL4A1, COL4A2, COL5A1, COL5A2, COL6A1, COL6A2, COL6A3, COLGALT1, COPE, COPS2, COPS5, COPS7A, CPOX, CPSF2, CPSF3, CRTAP, CSE1L, CSPG4, CSTF1, CSTF3, CTCF, CTNNB1, CTNNBL1, CTSC, CUL4A, CUL4B, CWC27, CXADR, CYB5R3, CYFIP1, DAGLB, DARS2, DAXX, DAZAP1, DBNL, DCAF1, DCAF13, DDRGK1, DDX18, DDX20, DDX21, DDX24, DDX28, DDX31, DDX39B, DDX42, DDX46, DDX47, DDX49, DDX5, DDX52, DDX54, DDX6, DECR1, DHX15, DHX30, DHX33, DHX37, DHX8, DHX9, DNMT1, DNMT3A, DNMT3B, DNTTIP2, DPH1, DPH6, DPPA4, DRG1, DUSP3, ECPAS, ECT2, EDEM3, EEF1E1, EEF2, EFTUD2, EGFR, EHMT1, EIF3A, EIF4A3, ELAC2, ELP3, EMD, EMSY, EPB41, EPB41L5, ERAP1, ERBIN, ERCC2, ERGIC1, ERGIC2,	

					ERLEC1, ERO1A, ERP44, ESD, ESF1, ETFA, ETHE1, EXD2, EXOSC10, EXOSC2, EXOSC5, EXOSC7, FAF2, FAHD1, FAM114A1, FAM118B, FANCD2, FANCI, FASTKD2, FDPS, FDXR, FECH, FERMT2, FGG, FH, FKBP10, FKBP14, FKBP4, FKBP5, FKBP7, FLAD1, FN1, FOXK1, FSCN1, FTH1, FTSJ3, FXR1, GARS1, GATA6, GATAD2A, GBF1, GCDH, GEMIN4, GEMIN5, GET3, GET4, GINS3, GLA, GLS, GLUD1, GNL2, GNL3, GNS, GOLGA3, GOLIM4, GOLT1B, GOT2, GPC1, GPC3, GPC6, GPI, GPSM1, GPX7, GPX8, GSN, GSTK1, GTF2E1, GTF2I, GTF3C1, GTF3C2, GTF3C3, GTF3C4, GUF1, H6PD, HACL1, HADHA, HADHB, HAGH, HAT1, HCFC1, HDAC2, HDAC4, HEATR1, HERC2, HEXA, HIBADH, HMGCL, HMOX1, HNRNPF, HNRNPL, HNRNPM, HOOK3, HP1BP3, HSD17B10, HSD17B4, HSP90AB1, HSP90B1, HSPA2, HSPA5, HSPA9, HSPD1, HSPE1, HSPH1, HTT, HUWE1, IDH2, IGF2BP3, ILF2, ILF3, ILK, IMP3, IMPDH1, IMPDH2, INCENP, INO80C, IPO11, IPO5, IRF2BPL, IRF3, IST1, ITCH, IWS1, JADE1, JARID2, KAT7, KDM1A, KDM2A, KDM3B, KEAP1, KIF20A, KIF22, KIF23, KRT18, KRT8, KTN1, LAMB1, LAMC1, LANCL2, LARS1, LARS2, LAS1L, LCMT1, LGALS1, LIG1, LIG3, LIN28A, LMCD1, LNPEP, LNPK, LRPPRC, LRRC59, LRWD1, LSG1, LSM4, LTBP1, LYAR, MACROH2A1, MAGED2, MAIP1, MANBA, MAP1S, MAP2K6, MAPK1, MAPK14, MAPK8, MARS1, MASTL, MATR3, MBD3, MBNL1, MCC, MCCC2, MCM2, MCM3, MCM4, MCM5, MCM6, MCM7, MCMBP, MDC1, MDN1, MEAK7, METTL1, MFGE8, MICAL3, MICOS13, MIPEP, MKI67, MLH1, MMAB, MOCS2, MPHOSPH10, MRPS27, MRPS9, MSH2, MSH3, MSH6, MTA3, MTAP, MTDH,	

					MTREX, MVB12A, MYBBP1A, MYDGF, MYO1C, MYO1E, NCAPD2, NCAPG, NCAPG2, NCL, NDC80, NDRG1, NEDD4, NEK7, NFKB1, NHLRC2, NIBAN2, NIT2, NLE1, NLN, NOL10, NOL11, NOL6, NOL9, NOP14, NOP56, NOP58, NOP9, NPM1, NPM3, NQO2, NSFL1C, NSUN2, NSUN5, NTHL1, NUDCD1, NUDT16, NUP107, NUP35, NUP50, NUSAP1, NVL, OGT, OPTN, ORC2, ORC3, ORC4, ORC5, OSBP, OSTF1, OXCT1, P3H1, P3H4, P4HA1, P4HA2, P4HB, PABPN1, PAK1, PARN, PARP1, PATZ1, PC, PCBP2, PCCA, PCCB, PDCD11, PDCL3, PDGFRB, PDIA3, PDIA4, PDIA6, PDLIM4, PDLIM7, PDS5A, PDXK, PEA15, PELP1, PES1, PFKL, PHC1, PHPT1, PIAS4, PIK3C2A, PIP4K2B, PIP4K2C, PITRM1, PKM, PKP2, PLD3, PLEKHA5, PLEKHA7, PLK1, PLOD3, PM20D2, PNO1, PNP, PNPT1, POGLUT2, POGLUT3, POGZ, POLA1, POLA2, POLB, POLD1, POLE, POLR1B, POLR2A, POLR2B, POLR2G, POLR3A, POLR3C, POLR3F, PPIB, PPID, PPM1B, PPM1G, PPP1R9B, PRDX4, PRIM1, PRIM2, PRKAA1, PRKAB1, PRKACB, PRKACG, PRKAG1, PRKCA, PRKCSH, PRKDC, PRORP, PRPF38A, PRPF40A, PRPF8, PSIP1, PSMC6, PSMD1, PSMD2, PSMD9, PSME3, PSPC1, PTCD1, PTCD3, PTPMT1, PTPN1, PTPN12, PTPN2, PUS1, PWP2, PXDN, PYCARD, PYCR1, PYCR2, RAB32, RAD51, RAI14, RAN, RANBP2, RANGAP1, RBBP5, RBM15, RBM25, RBM3, RBP1, RBPJ, RBPMS, RCC2, RCN1, RELA, RFC1, RFC2, RFC3, RFC5, RIF1, RIOX1, RNH1, RP2, RPA1, RPA2, RPA3, RPF2, RPL13A, RPL4, RPL5, RPL7A, RPRD1A, RPS2, RPS21, RPS6KA1, RPS6KA3, RPS6KA4, RPS8, RRP12, RRP7A, RRS1, RTCA, S100A11, SAAL1, SAMHD1,	

					SAP30BP, SARS2, SART3, SBDS, SBF1, SCARB2, SDAD1, SDF2L1, SDF4, SEC13, SELENBP1, SEPTIN2, SERPINB9, SERPINH1, SET, SETD7, SETDB1, SF3A3, SF3B1, SIL1, SIN3A, SIRT1, SIRT2, SKP2, SLC25A5, SLC44A1, SLTM, SMARCA4, SMARCA5, SMARCAD1, SMARCC1, SMARCD1, SMARCD2, SMC2, SNRNP200, SNRPA1, SNRPD3, SNTB2, SORBS1, SPARC, SPATS2L, SPON1, SPR, SPTAN1, SPTBN1, SRC, SRP14, SRPK1, SRSF1, SRSF11, SRSF7, SSBP1, SSRP1, STAM2, STX12, STYX, SUCLG1, SUMF2, SUN2, SUPT16H, SUPT5H, SUPT6H, SYMPK, TARS2, TBL3, TBPL1, TBRG4, TDP1, TDP2, TELO2, TEX10, TF, TFB1M, TGFB1I1, THBS1, THUMPD3, TIA1, TIMM50, TIMMDC1, TIMP3, TJP2, TKT, TLK1, TMA16, TMEM192, TMEM43, TOLLIP, TOP2A, TPP2, TPX2, TRAP1, TRIM28, TRIM33, TRIP12, TRMT1, TRMT10C, TRMT5, TSEN34, TSR1, TTF2, TUT1, TWF1, TXNDC5, TXNRD1, UAP1, UBE2O, UBN2, UBR4, UBR5, UGDH, UGGT1, UGGT2, UNG, UPF3B, USP47, USP7, UTP15, UTP18, UTP20, UTP25, UTP4, UTRN, VAT1, VIM, VPS25, VRK1, VWA5A, WAPL, WDHD1, WDR18, WDR3, WDR36, WDR43, WRAP53, XPO5, XRCC5, XRCC6, XRN2, YAP1, YARS2, ZFP36L2, ZMYM2, ZMYM3, ZMYND8, ZNF217, ZNF281, ZNF638]	
regulation of cellular amide metabolic process	GO_BiologicalProcess-EBI-UniProt-GOA-ACAP-ARAP_13.05.2021_00h00	1.49E-64	16.18182	89	[ABCF1, ACO1, AGO1, AGO2, AKT1, APOE, BIN1, C1QBP, CALR, CAPRIN1, CASP3, CDC123, CDK5RAP1, CELF1, CIRBP, CNOT11, COPS5, CSDE1, CYFIP1, DAPK1, DDX39B, DDX6, DHX9, DPH1, DPH2, DPH6, EEF2, EFL1, EIF3A, EIF4A3, EIF5B, EXOSC2, EXOSC5, EXOSC7, FASTKD2, FXR1, GEMIN5, GTPBP1, GUF1, HSPB1, IGF2BP3, ILF3, LIN28A, LRPPRC, LSM4, MAPK1,	Upregulation

					METAP1, MLH1, MRPS27, NCL, NIBAN1, NPM1, NSUN5, PARN, PASK, PICALM, PKM, PLXNB2, PNPT1, POLR2G, PRKAA1, PRKCSH, PRKDC, PTCD3, PURA, PUS7, RBM3, RELA, ROCK2, RPL13A, RPL22, RPL5, RPS6KA1, RPS6KA3, SAMD4B, SESN2, SPON1, THBS1, TIA1, TIGAR, TMED10, TMED2, TRAP1, TRIM71, TRMT10C, UPF3B, VIM, XRN1, ZFP36L2]	
cellular macromolecule biosynthetic process	GO_BiologicalProcess-EBI-UniProt-GOA-ACAP-ARAP_13.05.2021_00h00	1.49E-64	9.028994	464	[AASS, AATF, ABCB10, ABCF1, ABHD14B, ACO1, ACTN1, ACTN4, ACTR2, AEBP2, AGL, AGO1, AGO2, AIMP2, AKT1, ALG11, ALG2, ALG5, ALG9, ANKRD28, ANTXR1, ANXA3, ANXA4, AP3B1, APEH, APOE, ARF4, ARFGEF1, ARHGEF11, ATF7IP, ATP2B4, ATR, AURKB, B3GLCT, B4GALT1, BAG3, BAZ1B, BPTF, BRD3, BRD4, BRMS1, BRWD1, C1QBP, CACYBP, CALR, CAMK1, CAMK2D, CAPRIN1, CASK, CAT, CAV1, CAVIN1, CBX2, CBX5, CCAR1, CCAR2, CCDC22, CCNB1, CCNH, CCNL2, CCT2, CCT3, CCT4, CCT5, CCT6A, CCT7, CCT8, CD81, CDC123, CDC42, CDC73, CDK5RAP1, CDK5RAP3, CDK7, CEBPZ, CELF1, CHAF1A, CHD1, CHD4, CHEK1, CHEK2, CHPF, CHST14, CHTF18, CIRBP, CNOT11, COG7, COL1A1, COL4A2, COPS2, COPS5, CPSF2, CPSF3, CRLF3, CSDE1, CSPG4, CSTF1, CSTF3, CTBP2, CTCF, CTNNB1, CYFIP1, DAPK1, DARS2, DAXX, DCAF1, DDRGK1, DDX20, DDX21, DDX39B, DDX5, DDX54, DDX6, DHPS, DHX33, DHX9, DICER1, DLG1, DLGAP5, DNMT1, DNMT3A, DNMT3B, DPH1, DPH2, DPH6, DRG1, EDEM3, EDRF1, EEF1E1, EEF2, EFL1, EGFR, EHMT1, EIF3A, EIF4A3, EIF5B, ELP3, EMSY, EPCAM, EPM2AIP1, ERBIN, ERCC2, EXD2, EXOSC10, EXOSC2, EXOSC5, EXOSC7, FASTKD2, FOXK1, FUT11, FXR1, G3BP2, GALNT1, GALNT10, GALNT2,	Upregulation

					GARS1, GATA6, GATAD2A, GBE1, GCLC, GEMIN5, GFPT2, GINS3, GLUL, GNL3, GOLGA2, GPI, GTF2E1, GTF2I, GTF3C1, GTF3C2, GTF3C3, GTF3C4, GTPBP1, GUF1, GXYLT1, GYS1, HAT1, HCFC1, HDAC2, HDAC4, HEATR1, HELLS, HMOX1, HP1BP3, HSD17B4, HSP90AB1, HSPA5, HSPB1, HSPD1, IGF2BP3, ILF2, ILF3, ILK, IRF2BPL, IRF3, ITCH, ITGA6, IWS1, JARID2, KANK2, KAT7, KDM1A, KDM2A, KDM3B, KEAP1, LANCL2, LARS1, LARS2, LIG1, LIG3, LIMS1, LIN28A, LMAN1, LMCD1, LRPPRC, LRWD1, LSM4, LYAR, MACROH2A1, MAGED1, MAN1A1, MAN1A2, MAPK1, MAPK14, MAPK8, MARS1, MAVS, MBD3, MCM2, MCM3, MCM4, MCM5, MCM6, MCM7, MCMBP, METAP1, MGAT2, MLH1, MMAB, MOGS, MRPS27, MRPS9, MSH3, MTA3, MTDH, MVD, MYBBP1A, MYD88, MYDGF, MYEF2, MYO1C, NCAPG2, NCL, NDRG1, NEDD4, NEK7, NFKB1, NIBAN1, NIBAN2, NIF3L1, NMT2, NOL11, NPM1, NPM3, NSUN5, NUP107, NUP35, NVL, OGT, ORC2, ORC3, ORC4, ORC5, OSTC, PABPN1, PARN, PARP1, PASK, PATZ1, PAWR, PAXBP1, PDGFRB, PDS5A, PELP1, PFKM, PGM2L1, PGM3, PHC1, PHIP, PIAS4, PICALM, PIGS, PIGT, PKM, PLK1, PLOD1, PLOD2, PLOD3, PLPP3, PLXNB2, PNPT1, POFUT1, POFUT2, POGLUT2, POGLUT3, POGZ, POLA1, POLA2, POLB, POLD1, POLE, POLR1B, POLR2A, POLR2B, POLR2G, POLR3A, POLR3C, POLR3F, PPID, PPM1B, PPM1F, PRIM1, PRIM2, PRKAA1, PRKAR1A, PRKCSH, PRKDC, PSIP1, PSMC6, PSMD1, PSMD2, PSMD9, PSME3, PSPC1, PTCD3, PTGIS, PTPN2, PURA, PUS7, PXDN, PYCARD, QRSL1, RAD51, RAP2C, RBM15, RBM3, RBPJ, RBPMS, RELA, RFC1, RFC2, RFC3, RFC5, RIF1,	

					RIOX1, ROCK2, RPA1, RPA2, RPA3, RPL13A, RPL22, RPL27A, RPL4, RPL5, RPL7A, RPN1, RPRD1A, RPS2, RPS21, RPS6KA1, RPS6KA3, RPS6KA4, RPS8, RRBP1, S100A11, SALL2, SAMD4B, SAMHD1, SAP30BP, SARS1, SARS2, SDF2L1, SESN2, SET, SETD7, SETDB1, SIN3A, SIRT1, SIRT2, SLC2A10, SLTM, SMARCA4, SMARCA5, SMARCC1, SMARCD1, SMARCD2, SNRPD3, SNX6, SORBS1, SORBS3, SRC, SSBP1, SSRP1, STT3A, STT3B, STXBP2, SUPT16H, SUPT5H, SUPT6H, SYMPK, TARS2, TAX1BP1, TBPL1, TCP1, TDP2, TELO2, TF, TFB1M, TGFB1I1, THBS1, TIA1, TMED2, TMF1, TOP2A, TRAP1, TRIM28, TRIM33, TRIM71, TRIP13, TRMT10C, TTF2, UFL1, UGDH, UGGT1, UGGT2, UGP2, UPF3B, USP47, USP7, USP9X, UTP15, UTP4, VIM, VRTN, WAPL, WARS1, WDHD1, WDR18, WDR43, WRAP53, XRCC5, XRCC6, XRN1, XRN2, YAP1, YARS2, ZC3H15, ZDHHC17, ZFP36L2, ZMYND8, ZNF217, ZNF281, ZNF462]	
cellular protein metabolic process	GO_BiologicalProcess-EBI-UniProt-GOA-ACAP-ARAP_13.05.2021_00h00	1.49E-64	10.26415	544	[AAAS, AASS, ABCF1, ABHD10, ABHD12, ABI1, ACO1, ACSL1, ADAM10, ADAR, ADARB1, AGO1, AGO2, AGTPBP1, AIMP2, AKT1, ALG11, ALG2, ALG5, ALG9, ANKZF1, ANTXR1, ANXA1, ANXA2, AP2A1, AP2A2, AP2B1, AP2M1, AP2S1, AP3B1, APAF1, APEH, APOE, ARF4, ARFGEF1, ARL6IP5, ASPH, ATP2B4, ATR, AUP1, AURKA, AURKB, B3GLCT, B4GALT1, BAG2, BAZ1B, BCCIP, BIN1, BIRC6, BRAT1, BRD4, BRMS1, BUB1B, C1QBP, CALR, CALU, CAMK1, CAMK2D, CAPN2, CAPRIN1, CASK, CASP3, CAST, CAV1, CBL, CCAR2, CCDC22, CCNB1, CCND2, CCNH, CCNL2, CCNY, CD2AP, CD44, CD81, CDC123, CDC42, CDC42BPA, CDC42BPB, CDC73, CDH2, CDK5RAP1, CDK5RAP3, CDK7, CELF1, CFAP20, CHEK1,	No change

					CHEK2, CIRBP, CKAP4, CLASP1, CNOT11, COG7, COL6A3, COPS2, COPS5, COPS7A, CORO1C, CRTAP, CSDE1, CSPG4, CTCF, CTNNB1, CTSC, CUL4A, CUL4B, CUL7, CWC27, CYFIP1, DAPK1, DARS2, DAXX, DBNL, DCAF1, DCAF13, DDRGK1, DDX39B, DDX6, DHPS, DHX33, DHX9, DIPK2A, DLG1, DNMT1, DNMT3B, DOCK7, DPH1, DPH2, DPH6, DRG1, DSP, DUSP3, ECPAS, ECT2, EDEM3, EEF1E1, EEF2, EFL1, EGFR, EHD4, EHMT1, EIF3A, EIF4A3, EIF5B, ELP3, EPHB3, ERLEC1, ERLIN2, ERO1A, ETFA, EXOSC2, EXOSC5, EXOSC7, FAF2, FASTKD2, FBXO2, FBXO30, FBXW8, FDXR, FERMT2, FGG, FKBP10, FKBP11, FKBP14, FKBP15, FKBP4, FKBP5, FKBP7, FKBP8, FKBP9, FLT1, FN1, FOXK1, FUT11, FXR1, GALNT1, GALNT10, GALNT2, GARS1, GATA6, GCLC, GEMIN5, GET4, GFPT2, GIPC1, GLMN, GLUL, GNL3, GOLGA2, GPC3, GPD1L, GPHN, GPI, GSN, GTF3C4, GTPBP1, GUF1, GXYLT1, HAT1, HCFC1, HDAC2, HDAC4, HERC2, HLA-A, HMBS, HSP90AB1, HSP90B1, HSPA2, HSPA5, HSPB1, HSPD1, HSPE1, HTT, HUWE1, IGF2BP3, ILF3, ILK, INCENP, INO80C, IPO5, IQGAP1, IQGAP3, IRF2BPL, IST1, ITCH, ITGA5, IWS1, JADE1, JARID2, KAT7, KDM1A, KDM2A, KDM3B, KEAP1, KTN1, L2HGDH, LAMB1, LAMC1, LARS1, LARS2, LCMT1, LGALS1, LIN28A, LMAN1, LMCD1, LNPEP, LRP1, LRPPRC, LRRC40, LRRK1, LSM4, LTBP1, LYPLA2, MACROH2A1, MAGED1, MAN1A1, MAN1A2, MANBA, MAP2K6, MAPK1, MAPK14, MAPK8, MARCHF5, MARS1, MASTL, MAVS, MBD3, MCM2, METAP1, MFGE8, MGAT2, MICAL1, MIPEP, MLH1, MMAB, MOCS2, MOGS, MRPS27, MRPS9, MSN, MTA3, MTMR14, MTMR6, MVB12A, MVD, MYADM, MYDGF,	

					MYH9, MYO1C, NCL, NDC1, NDRG1, NEDD4, NEK7, NFKB1, NIBAN1, NLE1, NMT2, NNT, NPM1, NRP2, NSFL1C, NSUN5, NUP107, NUP133, NUP160, NUP210, NUP35, NUP50, NXN, OCLN, OGT, ORC3, OSBP, OSTC, OTUD6B, P3H1, P3H3, P3H4, P4HA1, P4HA2, P4HB, P4HTM, PAK1, PALD1, PARD3, PARN, PARP1, PARVA, PASK, PAXBP1, PCBP2, PCOLCE, PDCD2, PDGFRB, PDIA6, PDZRN3, PEA15, PGAM5, PGM3, PHC1, PHIP, PHPT1, PIAS4, PICALM, PIGS, PIGT, PIK3CA, PIK3R4, PKM, PLK1, PLOD1, PLOD2, PLOD3, PLPP3, PLXNB2, PM20D2, PNPT1, POFUT1, POFUT2, POGLUT2, POGLUT3, POLB, POLR2G, POR, PPIB, PPIC, PPID, PPM1B, PPM1F, PPM1G, PPP1R7, PPP1R9B, PPP2R5A, PPP4R1, PRDX4, PRKAA1, PRKAB1, PRKACB, PRKACG, PRKAG1, PRKAR1A, PRKAR2A, PRKAR2B, PRKCA, PRKCSH, PRKDC, PRMT3, PRRC1, PSMC6, PSMD1, PSMD2, PSMD9, PSME3, PTCD3, PTK7, PTPMT1, PTPN1, PTPN12, PTPN2, PTPRD, PURA, PUS7, PXK, PYCARD, QRSL1, RAB1A, RAB2A, RAB8A, RAD51, RANBP2, RANGAP1, RAP2A, RAP2B, RAP2C, RBBP5, RBM3, RBPMS, RCN1, RDX, RELA, RIC1, RIF1, RIMKLB, RIOX1, RNF170, ROCK2, RPL13A, RPL22, RPL27A, RPL4, RPL5, RPL7A, RPN1, RPRD1A, RPS2, RPS21, RPS6KA1, RPS6KA3, RPS6KA4, RPS8, RRBP1, SAMD4B, SARS1, SARS2, SART3, SBF1, SCYL1, SDF2L1, SEH1L, SEL1L, SEPHS1, SERPINB6, SERPINB9, SERPINH1, SESN2, SET, SETD7, SETDB1, SIN3A, SIRT1, SIRT2, SKP2, SLK, SMARCAD1, SMARCC1, SNX3, SNX6, SNX9, SORD, SPAG9, SPON1, SPTBN1, SRC, SRPK1, STAM2, STK38, STT3A, STT3B, STYX, SUMF2, SUPT6H, SYMPK, TARS2, TELO2, TF,	

					TGFB1I1, TGM2, THBS1, TIA1, TIMM50, TIMP3, TIPRL, TLK1, TMED10, TMED2, TMF1, TMX3, TOLLIP, TPP2, TPST1, TPX2, TRAP1, TRIM28, TRIM33, TRIM71, TRIP12, TRMT10C, TRPT1, TTK, TTLL12, TWF1, UBA6, UBE2G1, UBE2H, UBE2O, UBR4, UBR5, UFL1, UGGT1, UGGT2, UPF3B, USP15, USP19, USP47, USP5, USP7, USP9X, VIM, VLDLR, VPS25, VPS35, VPS36, VRK1, WARS1, XRCC5, XRCC6, XRN1, YARS2, YOD1, ZC3H15, ZC3HAV1, ZDHHC17, ZFP36L2]	
regulation of protein metabolic process	GO_BiologicalProcess-EBI-UniProt-GOA-ACAP-ARAP_13.05.2021_00h00	1.49E-64	11.29722	317	[AASS, AATF, ABCB10, ABCF1, ABI1, ACO1, ACSL1, ADAR, ADARB1, AGO1, AGO2, AGTPBP1, AIMP2, AKT1, ANTXR1, ANXA2, APAF1, APOE, ARFGEF1, ARL6IP5, ASPH, ATP2B4, AURKA, BAG2, BCCIP, BIN1, BIRC6, BRAT1, BRD4, BRMS1, C1QBP, CALR, CAMK1, CAMK2D, CAPRIN1, CASP3, CAST, CAV1, CBL, CCAR2, CCDC22, CCNB1, CCND2, CCNH, CCNL2, CCNY, CD44, CD81, CDC123, CDK5RAP1, CDK5RAP3, CDK7, CELF1, CHEK1, CHEK2, CIRBP, CNOT11, COL6A3, COPS2, COPS5, CORO1C, CRTAP, CSDE1, CSPG4, CTCF, CTNNB1, CTSC, CUL4A, CYFIP1, CYP51A1, DAPK1, DAXX, DBNL, DDRGK1, DDX39B, DDX6, DHX9, DIPK2A, DLG1, DNMT1, DNMT3B, DOCK7, DPH1, DPH2, DPH6, DUSP3, ECT2, EEF2, EFL1, EGFR, EHD4, EIF3A, EIF4A3, EIF5B, ELP3, ETFA, EXOSC2, EXOSC5, EXOSC7, FASTKD2, FBXO2, FBXW8, FERMT2, FKBP8, FLT1, FN1, FXR1, GCLC, GEMIN5, GIPC1, GLMN, GNL3, GOLGA2, GPC3, GPD1L, GPI, GSN, GTPBP1, GUF1, HDAC2, HDAC4, HSP90AB1, HSPA2, HSPA5, HSPB1, HSPD1, HSPE1, HTT, HUWE1, IGF2BP3, ILF3, ILK, INCENP, IPO5, IQGAP1, IQGAP3, IST1, ITCH, ITGA5, ITGAV, IWS1, JARID2, KAT7, KDM1A, KEAP1, LIN28A, LPCAT1, LRP1, LRPPRC,	No change

					LRRK1, LSM4, MACROH2A1, MAGED1, MAP2K6, MAPK1, MAPK14, MAPK8, MASTL, MAVS, MCM2, METAP1, MICAL1, MLH1, MRPS27, MSN, MYADM, MYDGF, MYH9, MYO1C, NCL, NEDD4, NFKB1, NIBAN1, NLE1, NNT, NPM1, NSF, NSUN5, NXN, OCLN, OGT, ORC3, OSBP, P3H1, PAK1, PARD3, PARN, PARVA, PASK, PAWR, PAXBP1, PCOLCE, PDCD2, PDGFRB, PEA15, PHIP, PIAS4, PICALM, PIK3CA, PKM, PLK1, PLPP3, PLXNB2, PM20D2, PNPT1, POLR2G, POR, PPIB, PPM1F, PPP1R7, PPP1R9B, PPP2R5A, PPP4R1, PRKAA1, PRKACB, PRKACG, PRKAG1, PRKAR1A, PRKAR2A, PRKAR2B, PRKCA, PRKCSH, PRKDC, PRMT3, PRRC1, PSMC6, PSMD1, PSMD2, PSME3, PTCD3, PTPN1, PTPN2, PURA, PUS7, PYCARD, RAB1A, RAD51, RAP2A, RAP2B, RAP2C, RBM3, RBPMS, RDX, RELA, RIC1, RIF1, ROCK2, RPL13A, RPL22, RPL5, RPS2, RPS6KA1, RPS6KA3, RPS6KA4, SAMD4B, SART3, SEC22B, SERPINB6, SERPINB9, SERPINH1, SESN2, SET, SETD7, SIN3A, SIRT1, SIRT2, SKP2, SLC2A10, SMARCC1, SNX3, SNX6, SNX9, SORD, SPAG9, SPON1, SRC, STK38, STX5, STYX, SUPT6H, SYMPK, TELO2, TF, THBS1, TIA1, TIMP3, TIPRL, TMED10, TMED2, TMF1, TOLLIP, TPX2, TRAP1, TRIM71, TRIP12, TRMT10C, TRPT1, TTK, TTLL12, TWF1, UBE2O, UBR5, UFL1, UPF3B, USP19, USP47, USP5, USP7, VIM, VLDLR, VPS25, VPS35, WARS1, XRCC5, XRCC6, XRN1, ZFP36L2]	
nucleic acid metabolic process	GO_BiologicalProcess-EBI-UniProt-GOA-ACAP-ARAP_13.05.2021_00h00	1.49E-64	9.653234	515	[AASS, AATF, ABHD14B, ACIN1, ACTN1, ACTN4, ACTR2, ADAR, ADARB1, AEBP2, AGO1, AGO2, AIMP2, AKT1, ANKRD28, ANTXR1, ANXA3, ANXA4, AP3B1, APOBEC3C, APOE, ARF4, ARHGEF11, ATF7IP, ATP2B4, ATR, AURKB, BAG3, BAZ1B, BCCIP, BMS1, BOP1,	Upregulation

					BPTF, BRD3, BRD4, BRMS1, BRWD1, C1QBP, CALR, CAMK1, CAMK2D, CARHSP1, CASK, CASP3, CAT, CAV1, CAVIN1, CBX2, CBX5, CCAR1, CCAR2, CCDC22, CCNB1, CCNH, CCNL2, CCT2, CCT3, CCT4, CCT5, CCT6A, CCT7, CCT8, CD81, CDC73, CDK5RAP1, CDK5RAP3, CDK7, CEBPZ, CELF1, CHAF1A, CHD1, CHD4, CHEK1, CHEK2, CHTF18, CIRBP, CNOT11, COL1A1, COL4A2, COPS2, COPS5, COPS7A, CPSF2, CPSF3, CRLF3, CSDE1, CSTF1, CSTF3, CTBP2, CTCF, CTNNB1, CTNNBL1, CUL4A, CUL4B, CWC27, CYFIP1, DARS2, DAXX, DAZAP1, DCAF1, DCAF13, DDRGK1, DDX10, DDX18, DDX20, DDX21, DDX24, DDX39B, DDX41, DDX42, DDX46, DDX47, DDX49, DDX5, DDX52, DDX54, DDX6, DHX15, DHX33, DHX37, DHX8, DHX9, DICER1, DLG1, DLGAP5, DNMT1, DNMT3A, DNMT3B, DRG1, EDRF1, EEF1E1, EFTUD2, EGFR, EHMT1, EIF4A3, ELAC2, ELP1, ELP3, EMSY, ENDOD1, EPCAM, ERBIN, ERCC2, ESF1, EXD2, EXOG, EXOSC10, EXOSC2, EXOSC5, EXOSC7, FANCD2, FANCI, FASTKD2, FH, FOXK1, FSCN1, FTSJ3, FXR1, G3BP1, G3BP2, GARS1, GATA6, GATAD2A, GCLC, GEMIN4, GEMIN5, GINS3, GNL3, GPI, GTF2E1, GTF2I, GTF3C1, GTF3C2, GTF3C3, GTF3C4, GTPBP1, HAT1, HCFC1, HDAC2, HDAC4, HEATR1, HELLS, HERC2, HMOX1, HNRNPF, HNRNPL, HNRNPLL, HNRNPM, HP1BP3, HSD17B10, HSD17B4, HSP90AB1, HSPA5, HSPB1, HSPD1, HUWE1, IGF2BP3, ILF2, ILF3, ILK, IMP3, INO80C, IRF2BPL, IRF3, ITCH, ITGA6, IWS1, JARID2, KANK2, KAT7, KDM1A, KDM2A, KDM3B, KEAP1, KIF22, LANCL2, LARS1, LARS2, LAS1L, LIG1, LIG3, LIMS1, LIN28A, LMCD1, LRPPRC, LRWD1, LSM4, LYAR, MACROH2A1, MAGED1, MAP1S,	

					MAPK1, MAPK14, MAPK8, MARS1, MAVS, MBD3, MBNL1, MCM2, MCM3, MCM4, MCM5, MCM6, MCM7, MDC1, MDN1, METTL1, MLH1, MMAB, MPHOSPH10, MSH2, MSH3, MSH6, MTA3, MTDH, MTREX, MYBBP1A, MYD88, MYDGF, MYEF2, MYO1C, NBAS, NCAPG2, NCL, NDRG1, NEDD4, NEK7, NFKB1, NIBAN2, NIF3L1, NOL10, NOL11, NOL6, NOL9, NOP14, NOP56, NOP58, NOP9, NPM1, NPM3, NSUN2, NSUN5, NTHL1, NUDT16, NUP107, NUP35, NVL, OGT, ORC2, ORC3, ORC4, ORC5, PABPN1, PARN, PARP1, PATZ1, PAWR, PAXBP1, PCBP2, PDCD11, PDGFRB, PDS5A, PELP1, PES1, PFKM, PHC1, PHIP, PIAS4, PICALM, PIK3CA, PLD3, PLK1, PLPP3, PNPT1, POFUT1, POGZ, POLA1, POLA2, POLB, POLD1, POLE, POLR1B, POLR2A, POLR2B, POLR2G, POLR3A, POLR3C, POLR3F, PPID, PPM1F, PPP1R9B, PRIM1, PRIM2, PRKAA1, PRKAR1A, PRKCA, PRKDC, PRORP, PRPF38A, PRPF38B, PRPF40A, PRPF8, PSIP1, PSMC6, PSMD1, PSMD2, PSMD9, PSME3, PSPC1, PTBP2, PTCD1, PTGIS, PTPN2, PURA, PUS1, PUS3, PUS7, PWP2, PXDN, PYCARD, QRSL1, RAD51, RAN, RAP2C, RBM15, RBM25, RBM26, RBM3, RBM6, RBMS2, RBPJ, RBPMS, RELA, RFC1, RFC2, RFC3, RFC5, RIF1, RIOX1, RNH1, ROCK2, RPA1, RPA2, RPA3, RPF2, RPL13A, RPL22, RPL27A, RPL4, RPL5, RPL7A, RPRD1A, RPS2, RPS21, RPS6KA1, RPS6KA3, RPS6KA4, RPS8, RRP12, RRP7A, RRS1, RTCA, SALL2, SAMD4B, SAMHD1, SAP30BP, SARS1, SARS2, SART3, SBDS, SERBP1, SESN2, SET, SETD7, SETDB1, SF3A3, SF3B1, SIN3A, SIRT1, SIRT2, SLIRP, SLTM, SMARCA4, SMARCA5, SMARCAD1, SMARCC1, SMARCD1, SMARCD2, SNRNP200, SNRPA1,	

					SNRPD3, SNX6, SORBS3, SPOUT1, SRC, SRPK1, SRSF1, SRSF11, SRSF7, SSB, SSRP1, STXBP2, SUPT16H, SUPT5H, SUPT6H, SYMPK, TARS2, TAX1BP1, TBL3, TBPL1, TBRG4, TCP1, TDP1, TDP2, TELO2, TEX10, TF, TFB1M, TGFB1I1, THUMPD3, TIA1, TIGAR, TMF1, TOP2A, TRIM28, TRIM33, TRIM71, TRIP12, TRIP13, TRMT1, TRMT10C, TRMT1L, TRMT5, TRPT1, TSEN34, TSR1, TTF2, TUT1, UBR5, UFL1, UNG, UPF2, UPF3B, USP47, USP7, USP9X, UTP15, UTP18, UTP20, UTP25, UTP4, VIM, VRTN, WARS1, WDHD1, WDR18, WDR3, WDR36, WDR43, WRAP53, XPO5, XRCC5, XRCC6, XRN1, XRN2, YAP1, YARS2, ZC3H7B, ZC3HAV1, ZFP36L2, ZMYND8, ZNF217, ZNF281, ZNF462, ZNF638]	
nucleus	GO_CellularComponent-EBI-UniProt-GOA-ACAP-ARAP_13.05.2021_00h00	1.49E-64	10.37556	815	[AAAS, AASS, AATF, ABCF1, ABHD14B, ABI1, ACACA, ACAD9, ACIN1, ACLY, ACOT13, ACSS2, ACTBL2, ACTN4, ACTR2, ADAM10, ADAR, ADARB1, ADD3, AEBP2, AGL, AGO1, AGO2, AGTPBP1, AIMP2, AKT1, ALDH1L2, ALDH7A1, ALG2, AMDHD2, ANKRD28, ANKS1A, ANP32E, ANTXR1, ANXA1, ANXA11, ANXA2, ANXA4, APEH, API5, APOBEC3C, APOE, APPL2, APRT, ARAP1, ARFGEF1, ARHGDIA, ARL2, ARL3, ARPC1A, ARVCF, ATF7IP, ATP2B1, ATP6V1A, ATR, AURKA, AURKB, BAG1, BAG3, BAIAP2, BAZ1B, BCCIP, BIN1, BIRC6, BLVRB, BMS1, BOP1, BPNT2, BPTF, BRAT1, BRD3, BRD4, BRMS1, BRWD1, BUB1B, C1QBP, CACYBP, CALCOCO2, CALR, CAMK1, CAMK2D, CAPN2, CARNMT1, CASK, CASP3, CAVIN1, CBX2, CBX5, CCAR1, CCAR2, CCDC22, CCNB1, CCND2, CCNH, CCNL2, CCNY, CCT4, CCT8, CD2AP, CDC73, CDCA8, CDK5RAP3, CDK7, CEBPZ, CELF1, CFAP20, CFAP298, CFL2, CHAF1A, CHD1,	Upregulation

					CHD4, CHEK1, CHEK2, CHID1, CHTF18, CIRBP, CKAP4, CKAP5, CMPK1, CNDP2, CNOT11, COG7, COMMD4, COPE, COPS2, COPS5, COPS7A, CPNE2, CPSF2, CPSF3, CRLF3, CRMP1, CSE1L, CSPG4, CSRP2, CSTF1, CSTF3, CTBP2, CTCF, CTNNB1, CTNNBL1, CTSC, CUL4A, CUL4B, CUL7, CWC27, CXADR, CYFIP1, DAGLB, DAPK1, DARS2, DAXX, DAZAP1, DCAF1, DCAF13, DCTN1, DCTN4, DCXR, DDRGK1, DDX10, DDX18, DDX20, DDX21, DDX24, DDX28, DDX31, DDX39B, DDX41, DDX42, DDX46, DDX47, DDX49, DDX5, DDX52, DDX54, DDX6, DECR1, DHX15, DHX33, DHX37, DHX8, DHX9, DIAPH1, DICER1, DLG1, DLGAP5, DNMT1, DNMT3A, DNMT3B, DNTTIP2, DOCK7, DPH1, DPH6, DPPA4, DRG1, DSP, DUSP3, ECPAS, ECT2, EDRF1, EEF1E1, EEF2, EFTUD2, EGFR, EHD2, EHD4, EHMT1, EIF3A, EIF4A3, EIF5B, ELAC2, ELP1, ELP3, EMD, EMSY, ENOPH1, EPB41, EPB41L5, EPM2AIP1, ERBIN, ERCC2, ERGIC1, ERGIC2, ESF1, ETHE1, EXD2, EXOG, EXOSC10, EXOSC2, EXOSC5, EXOSC7, FAHD1, FAM114A1, FAM118B, FAM120A, FANCD2, FANCI, FAT1, FDPS, FERMT2, FH, FHL1, FKBP4, FKBP5, FLNB, FOXK1, FSCN1, FSD1, FTH1, FTSJ3, FXR1, G3BP1, GARS1, GATA6, GATAD2A, GDAP1, GEMIN4, GEMIN5, GET3, GET4, GINS3, GLUL, GNL2, GNL3, GNPDA2, GOLGA3, GOLT1B, GPC1, GPC6, GPI, GPSM1, GSN, GSTM3, GTF2E1, GTF2I, GTF3C1, GTF3C2, GTF3C3, GTF3C4, HACL1, HADHA, HAT1, HCFC1, HDAC2, HDAC4, HDLBP, HEATR1, HELLS, HERC2, HMOX1, HNRNPF, HNRNPL, HNRNPLL, HNRNPM, HP1BP3, HSD17B4, HSP90AB1, HSP90B1, HSPA14, HSPA2, HSPA4, HSPA5, HSPA9, HSPB1, HSPH1, HTT, HUWE1, IGF2BP3, ILF2, ILF3, ILK, IMP3, IMPDH1,	

					IMPDH2, INCENP, INO80C, IPO11, IPO4, IPO5, IQGAP1, IRF2BPL, IRF3, IST1, ITCH, IWS1, JADE1, JARID2, KAT7, KDM1A, KDM2A, KDM3B, KEAP1, KIF11, KIF20A, KIF22, KIF23, KIF2C, KNTC1, KRT18, KRT8, LANCL2, LARS1, LAS1L, LBR, LCMT1, LGALS1, LIG1, LIG3, LIN28A, LMCD1, LNPK, LPP, LRP1, LRPPRC, LRRC59, LRWD1, LSG1, LSM4, LYAR, MACROH2A1, MAGED1, MAGED2, MAP1S, MAP2K6, MAPK1, MAPK14, MAPK8, MARS1, MASTL, MATR3, MBD3, MBNL1, MCAM, MCC, MCM2, MCM3, MCM4, MCM5, MCM6, MCM7, MCMBP, MDC1, MDN1, MEAK7, MEMO1, METTL1, MEX3A, MFSD10, MGST1, MICAL3, MICOS13, MKI67, MLH1, MMAB, MOCS2, MPC2, MPHOSPH10, MRPS27, MRPS9, MSH2, MSH3, MSH6, MSI1, MSN, MTA3, MTAP, MTDH, MTMR6, MTREX, MVB12A, MYBBP1A, MYD88, MYEF2, MYH10, MYH9, MYO1C, MYO1E, NCAPD2, NCAPG, NCAPG2, NCL, NDC1, NDC80, NDRG1, NDRG2, NEDD4, NEK7, NFKB1, NIBAN2, NIF3L1, NLE1, NOL10, NOL11, NOL6, NOL9, NOP14, NOP56, NOP58, NOP9, NPM1, NPM3, NQO2, NSFL1C, NSUN2, NSUN5, NTHL1, NUCB2, NUDCD1, NUDT16, NUP107, NUP133, NUP160, NUP210, NUP35, NUP50, NUSAP1, NVL, NXN, OGT, OPTN, ORC2, ORC3, ORC4, ORC5, OSBP, P3H4, P4HA2, PABPN1, PAK1, PALLD, PARD6B, PARN, PARP1, PARVA, PASK, PATZ1, PAWR, PAXBP1, PCBP2, PCYT1A, PDCD11, PDCD2, PDCL3, PDGFRB, PDIA3, PDLIM2, PDLIM4, PDLIM7, PDS5A, PDXK, PEA15, PELP1, PES1, PFKM, PFKP, PGRMC2, PHC1, PHIP, PHPT1, PIAS4, PICALM, PIK3C2A, PIP4K2B, PIP4K2C, PKM, PKP2, PLCB3, PLCG1,	

					PLEKHA5, PLEKHA7, PLK1, PLSCR3, PM20D2, PNO1, PNP, PNPT1, POGLUT2, POGZ, POLA1, POLA2, POLB, POLD1, POLE, POLR1B, POLR2A, POLR2B, POLR2G, POLR3A, POLR3C, POLR3F, PON2, PPIB, PPID, PPM1B, PPM1F, PPM1G, PPP1R7, PPP1R9B, PPP2R5A, PRDX1, PRDX4, PREPL, PRIM1, PRIM2, PRKAA1, PRKAB1, PRKACB, PRKACG, PRKAG1, PRKCA, PRKDC, PRORP, PRPF38A, PRPF38B, PRPF40A, PRPF8, PRUNE1, PSIP1, PSMC6, PSMD1, PSMD2, PSMD9, PSME3, PSPC1, PTBP2, PTCD3, PTGIS, PTPMT1, PTPN12, PTPN2, PURA, PUS1, PUS3, PUS7, PWP2, PYCARD, RAB2A, RABL6, RAD51, RAD54L2, RAI14, RAN, RANBP2, RANGAP1, RBBP5, RBM15, RBM25, RBM26, RBM3, RBM6, RBMS2, RBP1, RBPJ, RBPMS, RCC2, RDH10, RELA, RETSAT, RFC1, RFC2, RFC3, RFC5, RHEB, RIF1, RIOX1, RNH1, ROCK2, RP2, RPA1, RPA2, RPA3, RPF2, RPL13A, RPL22, RPL4, RPL5, RPL7A, RPRD1A, RPS2, RPS21, RPS6KA1, RPS6KA3, RPS6KA4, RPS8, RRP12, RRP7A, RRS1, RTCA, S100A11, SAAL1, SALL2, SAMD4B, SAMHD1, SAP30BP, SARS1, SART3, SBDS, SBF1, SCRN1, SCYL1, SDAD1, SEC13, SEH1L, SELENBP1, SEPHS1, SEPTIN2, SEPTIN7, SERBP1, SERPINB6, SERPINB9, SESN2, SET, SETD7, SETDB1, SF3A3, SF3B1, SIN3A, SIRT1, SIRT2, SKP2, SLC25A5, SLC2A1, SLC44A1, SLC9A3R2, SLIRP, SLTM, SMARCA4, SMARCA5, SMARCAD1, SMARCC1, SMARCD1, SMARCD2, SMC2, SNRNP200, SNRPA1, SNRPD3, SNTB2, SNX6, SORBS1, SORBS3, SPARC, SPATS2L, SPR, SPTBN1, SRC, SRP14, SRPK1, SRSF1, SRSF11, SRSF7, SSB, SSBP1, SSRP1, STAM2, STK38, STX12, STXBP2, STYX, SUN2, SUPT16H, SUPT5H,	

					SUPT6H, SYMPK, TBL3, TBPL1, TDP1, TDP2, TELO2, TEX10, TEX2, TGFB1I1, TGM2, THUMPD3, TIA1, TIGAR, TIMM50, TIMMDC1, TIMP3, TJP2, TKFC, TKT, TLK1, TMA16, TMEM115, TMEM192, TMEM43, TMEM97, TMF1, TMX4, TNPO3, TOLLIP, TOMM34, TOP2A, TPP2, TPX2, TRAP1, TRIM28, TRIM33, TRIP12, TRIP13, TRMT1, TRMT10C, TRMT1L, TRMT5, TSEN34, TSR1, TTF2, TTI1, TTK, TTLL12, TUBB6, TUT1, TWF1, TXNL1, TXNRD1, UAP1, UBA6, UBE2H, UBE2O, UBN2, UBR4, UBR5, UFL1, UGDH, UGP2, UNG, UPF2, UPF3B, USP15, USP47, USP5, USP7, USP9X, UTP15, UTP18, UTP20, UTP25, UTP4, UTRN, VIM, VPS25, VPS26C, VPS36, VRK1, VWA5A, WAPL, WARS1, WDHD1, WDR18, WDR3, WDR36, WDR37, WDR43, WRAP53, XPO5, XPO7, XRCC5, XRCC6, XRN1, XRN2, YAP1, YARS2, YTHDC2, ZC3H15, ZC3H7B, ZC3HAV1, ZFP36L2, ZMYM2, ZMYM3, ZMYND8, ZNF217, ZNF281, ZNF462, ZNF532, ZNF638]	
nucleoplasm	GO_CellularComponent-EBI-UniProt-GOA-ACAP-ARAP_13.05.2021_00h00	1.49E-64	12.38586	529	[AAAS, AATF, ABCF1, ABHD14B, ACIN1, ACLY, ACSS2, ACTBL2, ACTN4, ADAR, ADARB1, AEBP2, AGO1, AGO2, AKT1, ALDH1L2, ANKRD28, ANKS1A, ANP32E, ANTXR1, ANXA1, ANXA11, API5, APRT, ARAP1, ARFGEF1, ARL2, ARL3, ATF7IP, ATP2B1, ATP6V1A, ATR, AURKA, AURKB, BAIAP2, BAZ1B, BCCIP, BLVRB, BMS1, BOP1, BPNT2, BPTF, BRAT1, BRD4, BRMS1, BRWD1, CACYBP, CALCOCO2, CAMK2D, CASP3, CAVIN1, CBX2, CBX5, CCAR1, CCAR2, CCDC22, CCNB1, CCND2, CCNH, CCNL2, CCT4, CCT8, CDC73, CDCA8, CDK7, CELF1, CFAP20, CHD1, CHD4, CHEK1, CHEK2, CHTF18, CIRBP, CKAP4, CMPK1, CNDP2, COPE, COPS2, COPS5, COPS7A, CPSF2, CPSF3, CSE1L, CSPG4, CSTF1, CSTF3, CTCF,	Upregulation

					CTNNB1, CTNNBL1, CTSC, CUL4A, CUL4B, CWC27, CXADR, CYFIP1, DAGLB, DARS2, DAXX, DAZAP1, DCAF1, DCAF13, DDX20, DDX21, DDX28, DDX39B, DDX42, DDX46, DDX47, DDX49, DDX5, DDX52, DDX54, DECR1, DHX15, DHX33, DHX37, DHX8, DHX9, DNMT1, DNMT3A, DNMT3B, DNTTIP2, DPH1, DPH6, DPPA4, DRG1, DUSP3, ECPAS, ECT2, EEF1E1, EFTUD2, EHMT1, EIF3A, EIF4A3, ELAC2, EMD, EMSY, EPB41, EPB41L5, ERBIN, ERCC2, ERGIC1, ESF1, ETHE1, EXOSC10, EXOSC2, EXOSC5, EXOSC7, FAHD1, FAM114A1, FAM118B, FANCD2, FANCI, FDPS, FERMT2, FKBP4, FKBP5, FOXK1, FSCN1, FTSJ3, GARS1, GATA6, GATAD2A, GEMIN4, GEMIN5, GET3, GET4, GINS3, GNL3, GOLGA3, GOLT1B, GPC1, GPI, GPSM1, GTF2E1, GTF2I, GTF3C1, GTF3C2, GTF3C3, GTF3C4, HACL1, HADHA, HAT1, HCFC1, HDAC2, HDAC4, HEATR1, HERC2, HNRNPF, HNRNPL, HNRNPM, HP1BP3, HSP90AB1, HSPH1, HTT, HUWE1, IGF2BP3, ILF2, ILF3, ILK, IMP3, INCENP, INO80C, IPO11, IRF2BPL, IRF3, ITCH, IWS1, JADE1, JARID2, KAT7, KDM1A, KDM2A, KDM3B, KEAP1, KIF20A, KIF22, KIF23, KRT8, LANCL2, LARS1, LAS1L, LCMT1, LIG1, LIG3, LMCD1, LNPK, LRPPRC, LRWD1, LSG1, LSM4, LYAR, MACROH2A1, MAGED2, MAP1S, MAP2K6, MAPK1, MAPK14, MAPK8, MASTL, MBD3, MBNL1, MCC, MCM2, MCM3, MCM4, MCM5, MCM6, MCM7, MCMBP, MDC1, MDN1, MEAK7, METTL1, MICAL3, MICOS13, MKI67, MLH1, MMAB, MOCS2, MPHOSPH10, MSH2, MSH3, MSH6, MTA3, MTAP, MTDH, MTREX, MVB12A, MYBBP1A, MYO1C, MYO1E, NCAPD2, NCAPG, NCAPG2, NCL, NDC80, NDRG1, NEK7, NFKB1, NIBAN2, NLE1, NOL11,	

					NOL6, NOL9, NOP14, NOP56, NOP58, NPM1, NPM3, NQO2, NSFL1C, NSUN2, NSUN5, NTHL1, NUDCD1, NUDT16, NUP35, NUP50, NVL, OGT, OPTN, ORC2, ORC3, ORC4, ORC5, OSBP, P4HA2, PABPN1, PAK1, PARN, PARP1, PATZ1, PCBP2, PDCD11, PDCL3, PDLIM7, PDS5A, PDXK, PEA15, PELP1, PES1, PHC1, PHPT1, PIAS4, PIK3C2A, PIP4K2B, PIP4K2C, PKP2, PLEKHA5, PLEKHA7, PLK1, PM20D2, PNO1, POGLUT2, POGZ, POLA1, POLA2, POLB, POLD1, POLE, POLR1B, POLR2A, POLR2B, POLR2G, POLR3A, POLR3C, POLR3F, PPIB, PPID, PPM1G, PPP1R9B, PRIM1, PRIM2, PRKAA1, PRKAB1, PRKACB, PRKACG, PRKAG1, PRKCA, PRKDC, PRORP, PRPF38A, PRPF40A, PRPF8, PSIP1, PSMC6, PSMD1, PSMD2, PSMD9, PSME3, PSPC1, PTCD3, PTPMT1, PTPN12, PTPN2, PUS1, PWP2, PYCARD, RAD51, RAI14, RAN, RANBP2, RANGAP1, RBBP5, RBM15, RBM25, RBM3, RBP1, RBPJ, RBPMS, RELA, RFC1, RFC2, RFC3, RFC5, RIF1, RIOX1, RNH1, RP2, RPA1, RPA2, RPA3, RPF2, RPL5, RPRD1A, RPS2, RPS21, RPS6KA1, RPS6KA3, RPS6KA4, RPS8, RRP7A, RRS1, RTCA, SAAL1, SAMHD1, SAP30BP, SART3, SBDS, SBF1, SDAD1, SEC13, SEPTIN2, SERPINB9, SET, SETD7, SETDB1, SF3A3, SF3B1, SIN3A, SIRT1, SKP2, SLC44A1, SLTM, SMARCA4, SMARCA5, SMARCAD1, SMARCC1, SMARCD1, SMARCD2, SMC2, SNRNP200, SNRPA1, SNRPD3, SNTB2, SPATS2L, SPR, SRC, SRPK1, SRSF1, SRSF11, SRSF7, SSRP1, STAM2, STX12, STYX, SUPT16H, SUPT5H, SUPT6H, SYMPK, TBL3, TBPL1, TDP1, TDP2, TELO2, TEX10, TIA1, TIMM50, TIMMDC1, TJP2, TKT, TLK1, TMA16, TMEM192, TOLLIP, TOP2A, TPP2, TPX2, TRAP1, TRIM28,	

					TRIM33, TRIP12, TRMT1, TRMT10C, TSEN34, TSR1, TTF2, TUT1, TXNRD1, UAP1, UBE2O, UBN2, UBR4, UBR5, UGDH, UNG, UPF3B, USP47, USP7, UTP15, UTP18, UTP20, UTP25, UTP4, UTRN, VPS25, VRK1, VWA5A, WAPL, WDHD1, WDR18, WDR3, WDR36, WDR43, WRAP53, XPO5, XRCC5, XRCC6, XRN2, YAP1, YARS2, ZFP36L2, ZMYM2, ZMYM3, ZMYND8, ZNF217, ZNF281, ZNF638]	
negative regulation of gene expression	GO_BiologicalProcess-EBI-UniProt-GOA-ACAP-ARAP_13.05.2021_00h00	1.49E-64	12.60904	159	[AAAS, ADAM10, ADAR, AEBP2, AGO1, AGO2, AKT1, ANXA1, ANXA4, APOE, APPL2, ATP2B1, ATP2B4, AURKA, C1QBP, CALR, CAPRIN1, CARHSP1, CAST, CCNB1, CD2AP, CDH3, CELF1, CHID1, CIRBP, CNOT11, CSDE1, CTCF, CTNNB1, DAPK1, DDRGK1, DDX5, DDX6, DHX9, DICER1, DNMT1, DNMT3A, DNMT3B, EGFR, EIF4A3, ERBIN, EXOSC10, EXOSC2, EXOSC5, EXOSC7, FASTKD2, FN1, FXR1, GARS1, GATA6, GTPBP1, HAT1, HDAC2, HELLS, HMOX1, HNRNPM, HSP90AB1, HSPB1, IGF2BP3, ILF3, IQGAP3, IRF3, ITCH, JARID2, LIN28A, LSM4, MACROH2A1, MAPK14, MAVS, MLH1, MYADM, MYD88, MYEF2, MYO1C, NBAS, NCL, NDC1, NDRG2, NFKB1, NPM1, NUDT16, NUP107, NUP133, NUP160, NUP210, NUP35, NUP50, OCLN, PARN, PAWR, PC, PCBP2, PICALM, PNPT1, POLR2A, POLR2B, POLR2G, PPM1B, PRKAA1, PRKCA, PRKDC, PSMC6, PSMD1, PSMD2, PSMD9, PSME3, PTBP2, PURA, PUS7, PYCARD, RAN, RANBP2, RIF1, RNH1, ROCK2, RPL13A, RPL22, RPL27A, RPL4, RPL5, RPL7A, RPS2, RPS21, RPS6KA4, RPS8, SAMD4B, SARS1, SEC13, SEH1L, SERBP1, SESN2, SET, SETDB1, SIN3A, SIRT1, SIRT2, SLC2A10, SORD, SPOUT1, SRSF7, SSB, TAX1BP1, TBRG4, THBS1, TIA1, TMF1, TRIM71, TUT1, UPF2, UPF3B, VIM, XPO5,	Upregulation

					XRN1, XRN2, YAP1, ZC3H7B, ZC3HAV1, ZFP36L2, ZNF281]	
phosphorylation	GO_BiologicalProcess-EBI-UniProt-GOA-ACAP-ARAP_13.05.2021_00h00	1.49E-64	10.91703	225	[AAAS, ABI1, ACSL1, ADAM10, ADAR, ADARB1, ADPGK, AK1, AK4, AKT1, ALDOC, ANTXR1, ANXA2, APOE, ATP2B4, ATR, AURKA, AURKB, BAZ1B, BCCIP, BIRC6, BRAT1, BRD4, BUB1B, CAMK1, CAMK2D, CASK, CASP3, CAV1, CBL, CCNB1, CCND2, CCNH, CCNL2, CCNY, CD44, CD81, CDC42, CDC42BPA, CDC42BPB, CDK5RAP1, CDK5RAP3, CDK7, CHEK1, CHEK2, CLASP1, CMPK1, COPS2, CORO1C, CSPG4, DAPK1, DAXX, DBNL, DCAF1, DDRGK1, DGKA, DIPK2A, DLG1, DOCK7, DUSP3, ECT2, EGFR, EHD4, ELP3, ENO2, EPHB3, ETFA, ETNK1, FERMT2, FKBP8, FLT1, FN1, FOXK1, FXR1, GALK1, GARS1, GLMN, GNE, GPD1L, GPI, HDAC2, HDAC4, HK1, HK2, HOOK3, HSP90AB1, HSPA2, HSPB1, HTT, ILF3, ILK, INCENP, IPO5, IQGAP1, IQGAP3, ITGA5, ITGA6, ITPK1, LRRK1, LTBP1, MACROH2A1, MAGED1, MAP2K6, MAPK1, MAPK14, MAPK8, MASTL, MAVS, MCM2, MCM7, MICAL1, MMAB, MVK, MYADM, MYDGF, NDC1, NEK7, NIBAN1, NNT, NOL9, NPM1, NRP2, NUP107, NUP133, NUP160, NUP210, NUP35, NUP50, OCLN, OGT, ORC3, OSBP, PAK1, PAPSS2, PARD3, PARVA, PASK, PDGFRB, PDXK, PEA15, PFKL, PFKM, PFKP, PGM2L1, PHIP, PI4KA, PIK3C2A, PIK3CA, PIK3R4, PIP4K2B, PIP4K2C, PKM, PLK1, PLPP3, PLXNB2, PPM1F, PPP1R9B, PPP2R5A, PPP4R1, PRDX4, PRKAA1, PRKAB1, PRKACB, PRKACG, PRKAG1, PRKAR1A, PRKAR2A, PRKAR2B, PRKCA, PRKDC, PRRC1, PTK7, PTPN1, PTPN2, PXK, PYCARD, RAD51, RANBP2, RAP2A, RAP2B, RAP2C, RBPMS, ROCK2, RPS6KA1, RPS6KA3, RPS6KA4, SCYL1, SEC13, SEH1L,	No change

					SEPHS1, SESN2, SHPK, SIRT1, SIRT2, SKP2, SLK, SNX6, SNX9, SORD, SPAG9, SPTBN1, SRC, SRPK1, STK38, TELO2, TF, THBS1, TIGAR, TJP2, TKFC, TLK1, TMED2, TOLLIP, TPX2, TRIM28, TRPT1, TTK, TWF1, USP15, VLDLR, VPS25, VRK1, WARS1, XRCC5, XRCC6]	
regulation of cellular protein metabolic process	GO_BiologicalProcess-EBI-UniProt-GOA-ACAP-ARAP_13.05.2021_00h00	1.49E-64	11.45675	302	[AASS, ABCF1, ABI1, ACO1, ACSL1, ADAR, ADARB1, AGO1, AGO2, AGTPBP1, AIMP2, AKT1, ANTXR1, ANXA2, APAF1, APOE, ARFGEF1, ARL6IP5, ASPH, ATP2B4, AURKA, BAG2, BCCIP, BIN1, BIRC6, BRAT1, BRD4, BRMS1, C1QBP, CALR, CAMK1, CAMK2D, CAPRIN1, CASP3, CAST, CAV1, CBL, CCAR2, CCDC22, CCNB1, CCND2, CCNH, CCNL2, CCNY, CD44, CD81, CDC123, CDK5RAP1, CDK5RAP3, CDK7, CELF1, CHEK1, CHEK2, CIRBP, CNOT11, COL6A3, COPS2, COPS5, CORO1C, CRTAP, CSDE1, CSPG4, CTCF, CTNNB1, CTSC, CYFIP1, DAPK1, DAXX, DBNL, DDRGK1, DDX39B, DDX6, DHX9, DIPK2A, DLG1, DNMT1, DNMT3B, DOCK7, DPH1, DPH2, DPH6, DUSP3, ECT2, EEF2, EFL1, EGFR, EHD4, EIF3A, EIF4A3, EIF5B, ELP3, ETFA, EXOSC2, EXOSC5, EXOSC7, FASTKD2, FBXO2, FBXW8, FERMT2, FKBP8, FLT1, FN1, FXR1, GCLC, GEMIN5, GIPC1, GLMN, GNL3, GOLGA2, GPC3, GPD1L, GPI, GSN, GTPBP1, GUF1, HDAC2, HDAC4, HSP90AB1, HSPA2, HSPA5, HSPB1, HSPD1, HSPE1, HTT, HUWE1, IGF2BP3, ILF3, ILK, INCENP, IPO5, IQGAP1, IQGAP3, IST1, ITCH, ITGA5, IWS1, JARID2, KAT7, KDM1A, KEAP1, LIN28A, LRP1, LRPPRC, LRRK1, LSM4, MACROH2A1, MAGED1, MAP2K6, MAPK1, MAPK14, MAPK8, MASTL, MAVS, MCM2, METAP1, MICAL1, MLH1, MRPS27, MSN, MYADM, MYDGF, MYH9, MYO1C, NCL, NFKB1, NIBAN1, NLE1, NNT, NPM1, NSUN5, NXN, OCLN, OGT, ORC3,	No change

					OSBP, P3H1, PAK1, PARD3, PARN, PARVA, PASK, PAXBP1, PCOLCE, PDCD2, PDGFRB, PEA15, PHIP, PIAS4, PICALM, PIK3CA, PKM, PLK1, PLPP3, PLXNB2, PM20D2, PNPT1, POLR2G, POR, PPIB, PPM1F, PPP1R7, PPP1R9B, PPP2R5A, PPP4R1, PRKAA1, PRKACB, PRKACG, PRKAG1, PRKAR1A, PRKAR2A, PRKAR2B, PRKCA, PRKCSH, PRKDC, PRMT3, PRRC1, PSMC6, PSME3, PTCD3, PTPN1, PTPN2, PURA, PUS7, PYCARD, RAB1A, RAD51, RAP2A, RAP2B, RAP2C, RBM3, RBPMS, RDX, RELA, RIC1, RIF1, ROCK2, RPL13A, RPL22, RPL5, RPS2, RPS6KA1, RPS6KA3, RPS6KA4, SAMD4B, SART3, SERPINB6, SERPINB9, SERPINH1, SESN2, SET, SETD7, SIN3A, SIRT1, SIRT2, SKP2, SMARCC1, SNX6, SNX9, SORD, SPAG9, SPON1, SRC, STK38, STYX, SUPT6H, SYMPK, TELO2, TF, THBS1, TIA1, TIMP3, TIPRL, TMED10, TMED2, TMF1, TOLLIP, TPX2, TRAP1, TRIM71, TRIP12, TRMT10C, TRPT1, TTK, TTLL12, TWF1, UBE2O, UBR5, UFL1, UPF3B, USP19, USP47, USP5, USP7, VIM, VLDLR, VPS25, VPS35, WARS1, XRCC5, XRCC6, XRN1, ZFP36L2]	
positive regulation of protein metabolic process	GO_BiologicalProcess-EBI-UniProt-GOA-ACAP-ARAP_13.05.2021_00h00	1.49E-64	11.01591	180	[ABCB10, ABI1, ACSL1, AGO2, AGTPBP1, AIMP2, AKT1, ANTXR1, ANXA2, APAF1, APOE, ARFGEF1, ARL6IP5, ASPH, ATP2B4, AURKA, BAG2, BRAT1, BRD4, BRMS1, C1QBP, CAMK1, CASP3, CAV1, CCDC22, CCNB1, CCND2, CCNY, CD44, CD81, CDC123, CDK5RAP1, CDK5RAP3, CHEK2, CIRBP, CSPG4, CTNNB1, CTSC, DAPK1, DAXX, DBNL, DDRGK1, DDX39B, DHX9, DIPK2A, DLG1, DNMT1, DNMT3B, DOCK7, ECT2, EEF2, EGFR, EHD4, EIF4A3, ETFA, FASTKD2, FBXW8, FERMT2, FLT1, FN1, FXR1, GCLC, GNL3, GOLGA2, GPC3, GSN, GUF1, HDAC2, HDAC4, HSP90AB1, HSPA2, HSPA5, HSPD1, HSPE1, HUWE1, ILK, INCENP,	No change

					IQGAP1, IQGAP3, IST1, ITCH, ITGA5, JARID2, KAT7, KEAP1, LIN28A, LPCAT1, LRP1, LRRK1, MAGED1, MAP2K6, MAPK1, MAPK14, MAPK8, MAVS, MRPS27, MSN, MYDGF, MYH9, MYO1C, NEDD4, NIBAN1, NPM1, NSF, NSUN5, OGT, ORC3, OSBP, PAK1, PASK, PAWR, PAXBP1, PCOLCE, PDCD2, PDGFRB, PEA15, PIAS4, PICALM, PIK3CA, PKM, PLK1, PLXNB2, POLR2G, PPM1F, PPP2R5A, PRKAA1, PRKACB, PRKACG, PRKAG1, PRKAR1A, PRKAR2A, PRKAR2B, PRKCA, PRKCSH, PRKDC, PRRC1, PSMC6, PSME3, PTPN1, PYCARD, RAB1A, RAP2A, RAP2B, RAP2C, RBM3, RBPMS, RDX, RELA, RIF1, ROCK2, RPL5, RPS2, RPS6KA4, SART3, SEC22B, SIRT1, SIRT2, SKP2, SLC2A10, SNX9, SORD, SPAG9, SPON1, SRC, STX5, SYMPK, TELO2, TF, THBS1, TOLLIP, TPX2, TRMT10C, TTK, UPF3B, USP5, VIM, VLDLR, VPS35, XRCC5, XRCC6]	
negative regulation of protein metabolic process	GO_BiologicalProcess-EBI-UniProt-GOA-ACAP-ARAP_13.05.2021_00h00	1.49E-64	12.80069	149	[AASS, AATF, ADAR, ADARB1, AGO1, AGO2, AKT1, ANXA2, APOE, ATP2B4, BAG2, BIN1, BIRC6, CALR, CAPRIN1, CASP3, CAST, CAV1, CBL, CCAR2, CCNB1, CD44, CDK5RAP1, CDK5RAP3, CELF1, CIRBP, CNOT11, COL6A3, COPS2, CORO1C, CRTAP, CTNNB1, CYP51A1, DAPK1, DDRGK1, DDX6, DNMT1, DNMT3B, DUSP3, EGFR, EIF4A3, EXOSC2, EXOSC5, EXOSC7, FKBP8, FXR1, GCLC, GIPC1, GLMN, GPC3, GPD1L, GPI, HDAC2, HSP90AB1, HSPB1, IGF2BP3, ILF3, ILK, IPO5, ITGAV, JARID2, KDM1A, LIN28A, LRP1, LRRK1, LSM4, MACROH2A1, MASTL, METAP1, MICAL1, MLH1, MYADM, NCL, NFKB1, NIBAN1, NLE1, NNT, NPM1, NXN, OCLN, OGT, P3H1, PARD3, PARN, PARVA, PICALM, PLK1, PLPP3, PNPT1, POLR2G, POR, PPM1F, PPP1R9B, PRKAA1, PRKAR1A, PRKAR2A,	No change

					PRKAR2B, PRKDC, PRMT3, PTPN1, PTPN2, PURA, PUS7, PYCARD, RELA, RIC1, ROCK2, RPL13A, RPL5, RPS6KA1, RPS6KA3, SAMD4B, SERPINB6, SERPINB9, SERPINH1, SESN2, SET, SIN3A, SIRT1, SIRT2, SLC2A10, SMARCC1, SNX3, SNX6, SORD, SPAG9, SPON1, SRC, STK38, STYX, SUPT6H, THBS1, TIA1, TIMP3, TIPRL, TMED10, TMED2, TRIM71, TRIP12, UBE2O, UBR5, UFL1, USP19, USP47, USP7, VPS25, WARS1, XRN1, ZFP36L2]	
RNA metabolic process	GO_BiologicalProcess-EBI-UniProt-GOA-ACAP-ARAP_13.05.2021_00h00	1.49E-64	9.244223	444	[AASS, AATF, ABHD14B, ACIN1, ACTN1, ACTN4, ACTR2, ADAR, ADARB1, AEBP2, AGO1, AGO2, AIMP2, AKT1, ANXA3, ANXA4, AP3B1, APOBEC3C, APOE, ARF4, ARHGEF11, ATF7IP, ATP2B4, AURKB, BAG3, BAZ1B, BMS1, BOP1, BPTF, BRD3, BRD4, BRMS1, BRWD1, C1QBP, CALR, CAMK1, CAMK2D, CARHSP1, CASK, CAT, CAV1, CAVIN1, CBX2, CBX5, CCAR1, CCAR2, CCDC22, CCNB1, CCNH, CCNL2, CD81, CDC73, CDK5RAP1, CDK5RAP3, CDK7, CEBPZ, CELF1, CHD1, CHD4, CHEK1, CHEK2, CIRBP, CNOT11, COL1A1, COL4A2, COPS2, COPS5, CPSF2, CPSF3, CRLF3, CSDE1, CSTF1, CSTF3, CTBP2, CTCF, CTNNB1, CTNNBL1, CWC27, CYFIP1, DARS2, DAXX, DAZAP1, DCAF1, DCAF13, DDRGK1, DDX10, DDX18, DDX20, DDX21, DDX24, DDX39B, DDX41, DDX42, DDX46, DDX47, DDX49, DDX5, DDX52, DDX54, DDX6, DHX15, DHX33, DHX37, DHX8, DHX9, DICER1, DLG1, DLGAP5, DNMT1, DNMT3A, DNMT3B, DRG1, EDRF1, EEF1E1, EFTUD2, EGFR, EHMT1, EIF4A3, ELAC2, ELP1, ELP3, EMSY, EPCAM, ERBIN, ERCC2, ESF1, EXD2, EXOG, EXOSC10, EXOSC2, EXOSC5, EXOSC7, FASTKD2, FOXK1, FTSJ3, FXR1, G3BP2, GARS1, GATA6, GATAD2A, GCLC, GEMIN4, GEMIN5, GNL3, GPI, GTF2E1,	Upregulation

					GTF2I, GTF3C1, GTF3C2, GTF3C3, GTF3C4, GTPBP1, HAT1, HCFC1, HDAC2, HDAC4, HEATR1, HELLS, HMOX1, HNRNPF, HNRNPL, HNRNPLL, HNRNPM, HP1BP3, HSD17B10, HSD17B4, HSPA5, HSPB1, HSPD1, IGF2BP3, ILF2, ILF3, ILK, IMP3, IRF2BPL, IRF3, ITCH, ITGA6, IWS1, JARID2, KANK2, KAT7, KDM1A, KDM2A, KDM3B, KEAP1, LANCL2, LARS1, LARS2, LAS1L, LIMS1, LIN28A, LMCD1, LRPPRC, LSM4, LYAR, MACROH2A1, MAGED1, MAPK1, MAPK14, MAPK8, MARS1, MAVS, MBD3, MBNL1, MCM2, MDN1, METTL1, MLH1, MPHOSPH10, MTA3, MTDH, MTREX, MYBBP1A, MYD88, MYDGF, MYEF2, MYO1C, NBAS, NCAPG2, NCL, NDRG1, NEDD4, NFKB1, NIBAN2, NIF3L1, NOL10, NOL11, NOL6, NOL9, NOP14, NOP56, NOP58, NOP9, NPM1, NPM3, NSUN2, NSUN5, NUDT16, NUP107, NUP35, NVL, OGT, ORC2, PABPN1, PARN, PARP1, PATZ1, PAWR, PAXBP1, PCBP2, PDCD11, PELP1, PES1, PFKM, PHC1, PHIP, PIAS4, PICALM, PLK1, PLPP3, PNPT1, POFUT1, POGZ, POLA1, POLA2, POLR1B, POLR2A, POLR2B, POLR2G, POLR3A, POLR3C, POLR3F, PPID, PPM1F, PPP1R9B, PRIM1, PRIM2, PRKAA1, PRKAR1A, PRKCA, PRKDC, PRORP, PRPF38A, PRPF38B, PRPF40A, PRPF8, PSIP1, PSMC6, PSMD1, PSMD2, PSMD9, PSME3, PSPC1, PTBP2, PTCD1, PTGIS, PTPN2, PURA, PUS1, PUS3, PUS7, PWP2, PXDN, PYCARD, QRSL1, RAN, RAP2C, RBM15, RBM25, RBM26, RBM3, RBM6, RBMS2, RBPJ, RBPMS, RELA, RFC1, RIF1, RIOX1, RNH1, ROCK2, RPF2, RPL13A, RPL22, RPL27A, RPL4, RPL5, RPL7A, RPRD1A, RPS2, RPS21, RPS6KA1, RPS6KA3, RPS6KA4, RPS8, RRP12, RRP7A,	

					RRS1, RTCA, SALL2, SAMD4B, SAMHD1, SAP30BP, SARS1, SARS2, SART3, SBDS, SERBP1, SESN2, SET, SETD7, SETDB1, SF3A3, SF3B1, SIN3A, SIRT1, SIRT2, SLIRP, SLTM, SMARCA4, SMARCA5, SMARCC1, SMARCD1, SMARCD2, SNRNP200, SNRPA1, SNRPD3, SNX6, SORBS3, SPOUT1, SRC, SRPK1, SRSF1, SRSF11, SRSF7, SSB, SSRP1, STXBP2, SUPT16H, SUPT5H, SUPT6H, SYMPK, TARS2, TAX1BP1, TBL3, TBPL1, TBRG4, TDP2, TELO2, TEX10, TF, TFB1M, TGFB1I1, THUMPD3, TIA1, TMF1, TOP2A, TRIM28, TRIM33, TRIM71, TRIP13, TRMT1, TRMT10C, TRMT1L, TRMT5, TRPT1, TSEN34, TSR1, TTF2, TUT1, UFL1, UPF2, UPF3B, USP47, USP7, USP9X, UTP15, UTP18, UTP20, UTP25, UTP4, VIM, VRTN, WARS1, WDR18, WDR3, WDR36, WDR43, XPO5, XRCC5, XRCC6, XRN1, XRN2, YAP1, YARS2, ZC3H7B, ZC3HAV1, ZFP36L2, ZMYND8, ZNF217, ZNF281, ZNF462, ZNF638]	
nuclear lumen	GO_CellularComponent-EBI-UniProt-GOA-ACAP-ARAP_13.05.2021_00h00	1.49E-64	12.54325	580	[AAAS, AATF, ABCF1, ABHD14B, ACACA, ACIN1, ACLY, ACSS2, ACTBL2, ACTN4, ADAR, ADARB1, ADD3, AEBP2, AGO1, AGO2, AGTPBP1, AKT1, ALDH1L2, ANKRD28, ANKS1A, ANP32E, ANTXR1, ANXA1, ANXA11, API5, APRT, ARAP1, ARFGEF1, ARL2, ARL3, ATF7IP, ATP2B1, ATP6V1A, ATR, AURKA, AURKB, BAIAP2, BAZ1B, BCCIP, BLVRB, BMS1, BOP1, BPNT2, BPTF, BRAT1, BRD4, BRMS1, BRWD1, C1QBP, CACYBP, CALCOCO2, CAMK2D, CASK, CASP3, CAVIN1, CBX2, CBX5, CCAR1, CCAR2, CCDC22, CCNB1, CCND2, CCNH, CCNL2, CCT4, CCT8, CD2AP, CDC73, CDCA8, CDK5RAP3, CDK7, CELF1, CFAP20, CFL2, CHD1, CHD4, CHEK1, CHEK2, CHTF18, CIRBP, CKAP4, CKAP5, CMPK1, CNDP2, COG7, COPE, COPS2, COPS5, COPS7A, CPSF2, CPSF3, CSE1L,	Upregulation

					CSPG4, CSTF1, CSTF3, CTCF, CTNNB1, CTNNBL1, CTSC, CUL4A, CUL4B, CWC27, CXADR, CYFIP1, DAGLB, DARS2, DAXX, DAZAP1, DCAF1, DCAF13, DDRGK1, DDX18, DDX20, DDX21, DDX24, DDX28, DDX31, DDX39B, DDX42, DDX46, DDX47, DDX49, DDX5, DDX52, DDX54, DDX6, DECR1, DHX15, DHX33, DHX37, DHX8, DHX9, DNMT1, DNMT3A, DNMT3B, DNTTIP2, DPH1, DPH6, DPPA4, DRG1, DUSP3, ECPAS, ECT2, EEF1E1, EFTUD2, EHMT1, EIF3A, EIF4A3, ELAC2, ELP3, EMD, EMSY, EPB41, EPB41L5, ERBIN, ERCC2, ERGIC1, ERGIC2, ESF1, ETHE1, EXOSC10, EXOSC2, EXOSC5, EXOSC7, FAHD1, FAM114A1, FAM118B, FANCD2, FANCI, FDPS, FERMT2, FKBP4, FKBP5, FOXK1, FSCN1, FTSJ3, FXR1, GARS1, GATA6, GATAD2A, GEMIN4, GEMIN5, GET3, GET4, GINS3, GNL2, GNL3, GOLGA3, GOLT1B, GPC1, GPI, GPSM1, GTF2E1, GTF2I, GTF3C1, GTF3C2, GTF3C3, GTF3C4, HACL1, HADHA, HAT1, HCFC1, HDAC2, HDAC4, HEATR1, HERC2, HMOX1, HNRNPF, HNRNPL, HNRNPM, HP1BP3, HSP90AB1, HSPA2, HSPA9, HSPH1, HTT, HUWE1, IGF2BP3, ILF2, ILF3, ILK, IMP3, INCENP, INO80C, IPO11, IPO5, IRF2BPL, IRF3, ITCH, IWS1, JADE1, JARID2, KAT7, KDM1A, KDM2A, KDM3B, KEAP1, KIF20A, KIF22, KIF23, KRT18, KRT8, LANCL2, LARS1, LAS1L, LCMT1, LIG1, LIG3, LIN28A, LMCD1, LNPK, LRPPRC, LRWD1, LSG1, LSM4, LYAR, MACROH2A1, MAGED2, MAP1S, MAP2K6, MAPK1, MAPK14, MAPK8, MARS1, MASTL, MATR3, MBD3, MBNL1, MCC, MCM2, MCM3, MCM4, MCM5, MCM6, MCM7, MCMBP, MDC1, MDN1, MEAK7, METTL1, MICAL3, MICOS13, MKI67, MLH1, MMAB, MOCS2, MPHOSPH10, MRPS27, MRPS9,	

					MSH2, MSH3, MSH6, MTA3, MTAP, MTDH, MTREX, MVB12A, MYBBP1A, MYO1C, MYO1E, NCAPD2, NCAPG, NCAPG2, NCL, NDC80, NDRG1, NEDD4, NEK7, NFKB1, NIBAN2, NLE1, NOL10, NOL11, NOL6, NOL9, NOP14, NOP56, NOP58, NOP9, NPM1, NPM3, NQO2, NSFL1C, NSUN2, NSUN5, NTHL1, NUDCD1, NUDT16, NUP107, NUP35, NUP50, NUSAP1, NVL, OGT, OPTN, ORC2, ORC3, ORC4, ORC5, OSBP, P3H4, P4HA2, PABPN1, PAK1, PARN, PARP1, PATZ1, PCBP2, PDCD11, PDCL3, PDLIM7, PDS5A, PDXK, PEA15, PELP1, PES1, PHC1, PHPT1, PIAS4, PIK3C2A, PIP4K2B, PIP4K2C, PKP2, PLEKHA5, PLEKHA7, PLK1, PM20D2, PNO1, POGLUT2, POGZ, POLA1, POLA2, POLB, POLD1, POLE, POLR1B, POLR2A, POLR2B, POLR2G, POLR3A, POLR3C, POLR3F, PPIB, PPID, PPM1B, PPM1G, PPP1R9B, PRIM1, PRIM2, PRKAA1, PRKAB1, PRKACB, PRKACG, PRKAG1, PRKCA, PRKDC, PRORP, PRPF38A, PRPF40A, PRPF8, PSIP1, PSMC6, PSMD1, PSMD2, PSMD9, PSME3, PSPC1, PTCD3, PTPMT1, PTPN12, PTPN2, PUS1, PWP2, PYCARD, RAD51, RAI14, RAN, RANBP2, RANGAP1, RBBP5, RBM15, RBM25, RBM3, RBP1, RBPJ, RBPMS, RCC2, RELA, RFC1, RFC2, RFC3, RFC5, RIF1, RIOX1, RNH1, RP2, RPA1, RPA2, RPA3, RPF2, RPL13A, RPL4, RPL5, RPL7A, RPRD1A, RPS2, RPS21, RPS6KA1, RPS6KA3, RPS6KA4, RPS8, RRP12, RRP7A, RRS1, RTCA, SAAL1, SAMHD1, SAP30BP, SART3, SBDS, SBF1, SDAD1, SEC13, SELENBP1, SEPTIN2, SERPINB9, SET, SETD7, SETDB1, SF3A3, SF3B1, SIN3A, SIRT1, SIRT2, SKP2, SLC44A1, SLTM, SMARCA4, SMARCA5, SMARCAD1, SMARCC1, SMARCD1, SMARCD2, SMC2, SNRNP200,	

					SNRPA1, SNRPD3, SNTB2, SORBS1, SPARC, SPATS2L, SPR, SPTBN1, SRC, SRPK1, SRSF1, SRSF11, SRSF7, SSRP1, STAM2, STX12, STYX, SUN2, SUPT16H, SUPT5H, SUPT6H, SYMPK, TBL3, TBPL1, TDP1, TDP2, TELO2, TEX10, TGFB1I1, THUMPD3, TIA1, TIMM50, TIMMDC1, TJP2, TKT, TLK1, TMA16, TMEM192, TOLLIP, TOP2A, TPP2, TPX2, TRAP1, TRIM28, TRIM33, TRIP12, TRMT1, TRMT10C, TSEN34, TSR1, TTF2, TUT1, TWF1, TXNRD1, UAP1, UBE2O, UBN2, UBR4, UBR5, UGDH, UNG, UPF3B, USP47, USP7, UTP15, UTP18, UTP20, UTP25, UTP4, UTRN, VIM, VPS25, VRK1, VWA5A, WAPL, WDHD1, WDR18, WDR3, WDR36, WDR43, WRAP53, XPO5, XRCC5, XRCC6, XRN2, YAP1, YARS2, ZFP36L2, ZMYM2, ZMYM3, ZMYND8, ZNF217, ZNF281, ZNF638]	
negative regulation of cellular protein metabolic process	GO_BiologicalProcess-EBI-UniProt-GOA-ACAP-ARAP_13.05.2021_00h00	1.49E-64	13.02752	142	[AASS, ADAR, ADARB1, AGO1, AGO2, AKT1, ANXA2, APOE, ATP2B4, BAG2, BIN1, BIRC6, CALR, CAPRIN1, CASP3, CAST, CAV1, CBL, CCAR2, CCNB1, CD44, CDK5RAP1, CDK5RAP3, CELF1, CIRBP, CNOT11, COL6A3, COPS2, CORO1C, CRTAP, CTNNB1, DAPK1, DDRGK1, DDX6, DNMT1, DNMT3B, DUSP3, EIF4A3, EXOSC2, EXOSC5, EXOSC7, FKBP8, FXR1, GCLC, GIPC1, GLMN, GPC3, GPD1L, GPI, HDAC2, HSP90AB1, HSPB1, IGF2BP3, ILF3, ILK, IPO5, JARID2, KDM1A, LIN28A, LRP1, LRRK1, LSM4, MACROH2A1, MASTL, METAP1, MICAL1, MLH1, MYADM, NCL, NFKB1, NIBAN1, NLE1, NNT, NPM1, NXN, OCLN, OGT, P3H1, PARD3, PARN, PARVA, PICALM, PLK1, PLPP3, PNPT1, POLR2G, POR, PPM1F, PPP1R9B, PRKAA1, PRKAR1A, PRKAR2A, PRKAR2B, PRKDC, PRMT3, PTPN1, PTPN2, PURA, PUS7,	No change

					PYCARD, RELA, RIC1, ROCK2, RPL13A, RPL5, RPS6KA1, RPS6KA3, SAMD4B, SERPINB6, SERPINB9, SERPINH1, SESN2, SET, SIN3A, SIRT1, SIRT2, SMARCC1, SNX6, SORD, SPAG9, SRC, STK38, STYX, SUPT6H, THBS1, TIA1, TIMP3, TIPRL, TMED10, TMED2, TRIM71, TRIP12, UBE2O, UBR5, UFL1, USP19, USP47, USP7, VPS25, WARS1, XRN1, ZFP36L2]	
positive regulation of cellular protein metabolic process	GO_BiologicalProcess-EBI-UniProt-GOA-ACAP-ARAP_13.05.2021_00h00	1.49E-64	10.8512	167	[ABI1, ACSL1, AGO2, AGTPBP1, AIMP2, AKT1, ANTXR1, ANXA2, APAF1, APOE, ARFGEF1, ARL6IP5, ASPH, ATP2B4, AURKA, BAG2, BRAT1, BRD4, BRMS1, C1QBP, CAMK1, CAV1, CCDC22, CCNB1, CCND2, CCNY, CD44, CD81, CDC123, CDK5RAP1, CDK5RAP3, CHEK2, CIRBP, CSPG4, CTNNB1, CTSC, DAPK1, DAXX, DBNL, DDRGK1, DDX39B, DHX9, DIPK2A, DLG1, DNMT1, DNMT3B, DOCK7, ECT2, EEF2, EGFR, EHD4, EIF4A3, ETFA, FASTKD2, FBXW8, FERMT2, FLT1, FN1, FXR1, GCLC, GNL3, GOLGA2, GSN, GUF1, HDAC2, HDAC4, HSP90AB1, HSPA2, HSPA5, HSPD1, HSPE1, HUWE1, ILK, INCENP, IQGAP1, IQGAP3, IST1, ITGA5, JARID2, KAT7, KEAP1, LIN28A, LRP1, LRRK1, MAGED1, MAP2K6, MAPK1, MAPK14, MAVS, MRPS27, MSN, MYDGF, MYH9, MYO1C, NIBAN1, NPM1, NSUN5, OGT, ORC3, OSBP, PAK1, PASK, PAXBP1, PCOLCE, PDCD2, PDGFRB, PEA15, PIAS4, PICALM, PIK3CA, PKM, PLK1, PLXNB2, POLR2G, PPM1F, PPP2R5A, PRKAA1, PRKACB, PRKACG, PRKAG1, PRKAR1A, PRKAR2A, PRKAR2B, PRKCA, PRKCSH, PRKDC, PRRC1, PSMC6, PSME3, PTPN1, PYCARD, RAB1A, RAP2A, RAP2B, RAP2C, RBM3, RBPMS, RDX, RIF1, ROCK2, RPL5, RPS2, RPS6KA4, SART3, SIRT1, SIRT2, SKP2, SNX9, SORD, SPAG9, SPON1, SRC, SYMPK, TELO2, TF, THBS1, TOLLIP, TPX2,	No change

					TRMT10C, TTK, UPF3B, USP5, VIM, VLDLR, VPS35, XRCC5, XRCC6]	
RNA processing	GO_BiologicalProcess-EBI-UniProt-GOA-ACAP-ARAP_13.05.2021_00h00	1.49E-64	18.40607	194	[ACIN1, ADAR, ADARB1, AGO1, AGO2, BMS1, BOP1, C1QBP, CCAR1, CCAR2, CCNB1, CCNH, CDC73, CDK5RAP1, CDK7, CELF1, CIRBP, CPSF2, CPSF3, CSTF1, CSTF3, CTNNBL1, CWC27, DAZAP1, DCAF13, DDX10, DDX18, DDX20, DDX21, DDX39B, DDX41, DDX42, DDX46, DDX47, DDX49, DDX5, DDX52, DDX54, DHX15, DHX37, DHX8, DHX9, DICER1, EFTUD2, EGFR, EIF4A3, ELAC2, ELP1, ELP3, ERCC2, ESF1, EXOSC10, EXOSC2, EXOSC5, EXOSC7, FASTKD2, FTSJ3, FXR1, GARS1, GEMIN4, GEMIN5, HEATR1, HNRNPF, HNRNPL, HNRNPLL, HNRNPM, HSD17B10, IGF2BP3, IMP3, IWS1, KDM1A, LAS1L, LIN28A, LSM4, LYAR, MBNL1, MCM2, MDN1, METTL1, MPHOSPH10, MTREX, NCL, NEDD4, NOL10, NOL11, NOL6, NOL9, NOP14, NOP56, NOP58, NOP9, NPM1, NPM3, NSUN2, NSUN5, NUDT16, NVL, PABPN1, PARN, PAXBP1, PCBP2, PDCD11, PELP1, PES1, PNPT1, POLR2A, POLR2B, POLR2G, PPP1R9B, PRKCA, PRKDC, PRORP, PRPF38A, PRPF38B, PRPF40A, PRPF8, PSIP1, PSPC1, PTBP2, PTCD1, PUS1, PUS3, PUS7, PWP2, RBM15, RBM25, RBM26, RBM3, RBM6, RBMS2, RBPMS, RPF2, RPL13A, RPL22, RPL27A, RPL4, RPL5, RPL7A, RPRD1A, RPS2, RPS21, RPS8, RRP12, RRP7A, RRS1, RTCA, SARS1, SART3, SBDS, SF3A3, SF3B1, SLTM, SNRNP200, SNRPA1, SNRPD3, SPOUT1, SRPK1, SRSF1, SRSF11, SRSF7, SSB, SUPT5H, SUPT6H, SYMPK, TBL3, TBRG4, TELO2, TEX10, TFB1M, THUMPD3, TIA1, TRIM71, TRMT1, TRMT10C, TRMT1L, TRMT5, TRPT1, TSEN34, TSR1, TTF2, TUT1, UPF3B, UTP15, UTP18, UTP20, UTP25, UTP4, WDR18, WDR3,	Upregulation

					WDR36, WDR43, XRN2, ZC3H7B, ZNF638]	
kinase activity	GO_BiologicalProcess-EBI-UniProt-GOA-ACAP-ARAP_13.05.2021_00h00	1.49E-64	10.72351	166	[ABI1, ACSL1, ADAR, ADARB1, ADPGK, AK1, AK4, AKT1, ANTXR1, APOE, ATP2B4, ATR, AURKA, AURKB, BAZ1B, BCCIP, BUB1B, CAMK1, CAMK2D, CASK, CASP3, CAV1, CBL, CCNB1, CCND2, CCNH, CCNL2, CCNY, CD44, CD81, CDC42, CDC42BPA, CDC42BPB, CDK5RAP1, CDK5RAP3, CDK7, CHEK1, CHEK2, CLASP1, CMPK1, CORO1C, CSPG4, DAPK1, DAXX, DBNL, DCAF1, DGKA, DIPK2A, DLG1, DUSP3, ECT2, EGFR, ELP3, EPHB3, ETFA, ETNK1, FERMT2, FLT1, GALK1, GNE, GPI, HK1, HK2, HOOK3, HSP90AB1, HSPB1, HTT, ILK, INCENP, IPO5, IQGAP1, IQGAP3, ITPK1, LRRK1, LTBP1, MACROH2A1, MAGED1, MAP2K6, MAPK1, MAPK14, MAPK8, MASTL, MCM2, MMAB, MVK, NEK7, NOL9, NPM1, NRP2, ORC3, PAK1, PAPSS2, PARVA, PASK, PDGFRB, PDXK, PEA15, PFKL, PFKM, PFKP, PGM2L1, PI4KA, PIK3C2A, PIK3CA, PIK3R4, PIP4K2B, PIP4K2C, PKM, PLK1, PPM1F, PPP1R9B, PPP2R5A, PRKAA1, PRKAB1, PRKACB, PRKACG, PRKAG1, PRKAR1A, PRKAR2A, PRKAR2B, PRKCA, PRKDC, PRRC1, PTK7, PTPN1, PTPN2, PXK, PYCARD, RANBP2, RAP2B, RAP2C, ROCK2, RPS6KA1, RPS6KA3, RPS6KA4, SCYL1, SEPHS1, SESN2, SHPK, SIRT1, SKP2, SLK, SNX6, SNX9, SPAG9, SRC, SRPK1, STK38, TELO2, TF, THBS1, TIGAR, TJP2, TKFC, TLK1, TPX2, TRIM28, TRPT1, TTK, TWF1, VLDLR, VPS25, VRK1, WARS1, XRCC5, XRCC6]	No change
regulation of protein modification process	GO_BiologicalProcess-EBI-UniProt-GOA-ACAP-ARAP_13.05.2021_00h00	1.49E-64	10.71222	185	[AASS, ABI1, ACSL1, ADAR, ADARB1, AIMP2, AKT1, ANXA2, APOE, ARFGEF1, ATP2B4, BAG2, BCCIP, BRAT1, BRD4, BRMS1, CAMK1, CAMK2D, CASP3, CAV1, CBL, CCAR2, CCNB1, CCND2, CCNH, CCNL2, CCNY, CD44, CD81, CDK5RAP1, CDK5RAP3, CDK7, CHEK1, CHEK2,	No change

					CORO1C, CRTAP, CSPG4, CTCF, CTNNB1, DAXX, DBNL, DDRGK1, DIPK2A, DLG1, DNMT1, DNMT3B, DOCK7, DUSP3, ECT2, EGFR, EHD4, ELP3, ETFA, FBXO2, FERMT2, FKBP8, FLT1, FN1, FXR1, GCLC, GLMN, GNL3, GOLGA2, GPD1L, HDAC2, HDAC4, HSP90AB1, HSPA2, HSPA5, HSPB1, HTT, HUWE1, ILK, INCENP, IPO5, IQGAP1, IQGAP3, ITCH, ITGA5, IWS1, JARID2, KAT7, KDM1A, LRRK1, MACROH2A1, MAGED1, MAP2K6, MAPK1, MAPK14, MAPK8, MASTL, MAVS, MCM2, MICAL1, MYADM, MYDGF, MYO1C, NIBAN1, NNT, NPM1, NXN, OCLN, OGT, ORC3, OSBP, P3H1, PAK1, PARD3, PARVA, PAXBP1, PDGFRB, PEA15, PHIP, PIAS4, PIK3CA, PLK1, PLPP3, PLXNB2, PPIB, PPM1F, PPP1R7, PPP1R9B, PPP2R5A, PPP4R1, PRKAA1, PRKACB, PRKACG, PRKAG1, PRKAR1A, PRKAR2A, PRKAR2B, PRKCA, PRKDC, PRMT3, PRRC1, PTPN1, PTPN2, PYCARD, RAB1A, RAD51, RAP2A, RAP2B, RAP2C, RBPMS, RELA, RIF1, ROCK2, RPL5, RPS2, RPS6KA4, SART3, SESN2, SET, SETD7, SIN3A, SIRT1, SIRT2, SKP2, SNX6, SNX9, SORD, SPAG9, SRC, STK38, SUPT6H, SYMPK, TELO2, TF, THBS1, TIPRL, TMED2, TOLLIP, TPX2, TRIP12, TRPT1, TTK, TTLL12, TWF1, UBR5, UFL1, VLDLR, VPS25, WARS1, XRCC5, XRCC6]	
negative regulation of protein modification process	GO_BiologicalProcess-EBI-UniProt-GOA-ACAP-ARAP_13.05.2021_00h00	1.49E-64	12.97578	75	[AASS, ADAR, ADARB1, AKT1, APOE, ATP2B4, BAG2, CASP3, CAV1, CBL, CCNB1, CDK5RAP1, CDK5RAP3, CORO1C, CRTAP, CTNNB1, DNMT1, DNMT3B, DUSP3, FKBP8, GCLC, GLMN, GPD1L, HDAC2, HSPB1, ILK, IPO5, JARID2, KDM1A, LRRK1, MACROH2A1, MASTL, MICAL1, MYADM, NIBAN1, NNT, NPM1, NXN, OCLN, OGT, P3H1, PARD3, PARVA, PLK1, PLPP3, PPM1F, PPP1R9B, PRKAA1, PRKAR1A, PRKAR2A,	No change

					PRKAR2B, PRKDC, PRMT3, PTPN1, PTPN2, PYCARD, RELA, ROCK2, RPL5, SET, SIN3A, SIRT1, SIRT2, SNX6, SORD, SPAG9, STK38, SUPT6H, TIPRL, TMED2, TRIP12, UBR5, UFL1, VPS25, WARS1]	
preribosome	GO_CellularComponent-EBI-UniProt-GOA-ACAP-ARAP_13.05.2021_00h00	5.72E-57	39.28571	33	[BMS1, BOP1, DCAF13, FTSJ3, HEATR1, IGF2BP3, IMP3, LAS1L, MDN1, MPHOSPH10, NEDD4, NOL10, NOL6, NOP14, NOP56, NOP58, NOP9, PDCD11, PES1, PRKDC, PWP2, RRP7A, RRS1, TBL3, TSR1, UTP18, UTP20, UTP25, UTP4, WDR3, WDR36, WDR37, XRCC5]	Upregulation
cellular catabolic process	GO_BiologicalProcess-EBI-UniProt-GOA-ACAP-ARAP_13.05.2021_00h00	5.72E-57	12.53538	310	[AASS, ABAT, ABCC1, ABHD10, ABHD12, ACAA1, ACAD8, ACAT1, ACAT2, ACOX3, ACSL1, AGL, AGO1, AGO2, AGTPBP1, AKT1, ALDH1L2, ALDH7A1, AMDHD2, ANKZF1, ANXA2, AP2A1, AP2A2, AP2B1, AP2M1, AP2S1, APOBEC3C, APOE, ARSA, ARSB, ATP2B4, ATP6V1A, AUP1, AURKA, AURKB, BAG2, BAG3, BCAT2, BDH2, BIRC6, BLVRB, BUB1B, CALCOCO2, CAPN1, CAPN2, CAPNS1, CARHSP1, CARNMT1, CASP3, CAT, CAV1, CBL, CCAR2, CCDC22, CCNB1, CD2AP, CD81, CDK5RAP3, CELF1, CHEK2, CIRBP, CNOT11, CRMP1, CSDE1, CSPG4, CTNNB1, CTSC, CUL4A, CUL4B, CUL7, CYP2S1, DAGLB, DAPK1, DCXR, DDRGK1, DDX49, DDX5, DDX6, DECR1, DHX9, DICER1, DPYSL2, DPYSL3, DYNC1LI2, ECE1, ECHDC1, ECPAS, EDEM3, EIF4A3, ERAP1, ERLEC1, ERLIN2, ESD, ETFA, EXOG, EXOSC10, EXOSC2, EXOSC5, EXOSC7, FAF2, FASTKD2, FBXO2, FBXW8, FNBP1L, FOXK1, FXR1, GCDH, GCLC, GET4, GIPC1, GLA, GLMN, GLS, GLUD1, GLUL, GNPDA2, GNS, GOLGA2, GOLT1B, GOT2, GPC1, GPSM1, GSTM2, GSTM3, GTPBP1, HACL1, HADHA, HADHB, HAGH, HEXA, HIBADH, HK2, HMGCL, HMOX1, HMOX2, HNRNPM, HSD17B10, HSD17B4, HSP90AB1, HSP90B1,	No change

					HSPA5, HSPB1, HTT, HUWE1, IGF2BP3, ILVBL, ITCH, KEAP1, LIN28A, LNPEP, LRP1, LRPPRC, LSM4, LYPLA2, MAN1A1, MANBA, MAP1LC3A, MAP1S, MAPK14, MAPK8, MCCC2, METAP1, MLH1, MME, MOXD1, MSN, MTAP, MTDH, MTMR14, MTREX, MVB12A, MYD88, MYEF2, NBAS, NEDD4, NNT, NPM1, NQO2, NSFL1C, NSUN2, NT5C2, NTHL1, NUDT16, OGT, OPTN, OXCT1, PARN, PCBP2, PCCA, PCCB, PDXDC1, PFKM, PGAM5, PGM2L1, PIK3C2A, PIK3CA, PIK3R4, PIP4K2B, PIP4K2C, PLCG1, PLK1, PM20D2, PNP, PNPT1, POLR2G, PON2, POR, PRDX1, PRKAA1, PRKAB1, PRKAG1, PRKCA, PRUNE1, PSMC6, PSMD1, PSMD2, PSMD9, PSME3, PTPN1, PXDN, PYCARD, QDPR, RAB1A, RAB23, RAB8A, RDX, RHEB, RIC1, RNH1, ROCK2, RPL13A, RPL22, RPL27A, RPL4, RPL5, RPL7A, RPS2, RPS21, RPS8, SAMD4B, SAMHD1, SARM1, SBDS, SCARB2, SCFD1, SCLY, SEC16A, SEC22B, SEL1L, SERBP1, SESN2, SET, SH3GLB1, SIRT1, SIRT2, SKP2, SLC25A5, SLIRP, SMARCC1, SMPDL3B, SNX6, SNX9, SORD, SRC, SSB, STAM2, STT3B, STX12, STYX, SUPT5H, TBC1D17, TBRG4, TGFB1I1, TIGAR, TIMP3, TKFC, TMF1, TOLLIP, TRIM71, TRIP12, TUT1, UBA6, UBE2G1, UBE2H, UBR4, UFL1, UGGT1, UGGT2, UNG, UPF2, UPF3B, USP15, USP19, USP47, USP5, USP7, USP9X, VIM, VLDLR, VPS25, VPS26A, VPS26B, VPS35, VPS36, XPNPEP1, XRN1, XRN2, YOD1, ZC3HAV1, ZFP36L2]	
organic substance catabolic process	GO_BiologicalProcess-EBI-UniProt-GOA-ACAP-ARAP_13.05.2021_00h00	5.72E-57	12.96453	307	[AAAS, AASS, ABAT, ABCC1, ABHD10, ABHD12, ACAA1, ACAD8, ACAT1, ACAT2, ACOX3, ADPGK, AGL, AGO1, AGO2, AGTPBP1, AKT1, ALDH1L2, ALDH2, ALDH7A1, ALDOC, AMDHD2, ANKZF1, ANXA2, AP2A1, AP2A2,	No change

					AP2B1, AP2M1, AP2S1, APOBEC3C, APOE, ARSB, ATP2B4, AUP1, AURKA, AURKB, BAG2, BAG3, BCAT2, BDH2, BIRC6, BLVRB, BPNT2, BUB1B, CAPN1, CAPN2, CARHSP1, CARNMT1, CASP3, CAV1, CBL, CCAR2, CCDC22, CCNB1, CD2AP, CD44, CD81, CDK5RAP3, CELF1, CHEK2, CIRBP, CNOT11, CRMP1, CSDE1, CSPG4, CTNNB1, CTSC, CUL4A, CUL4B, CUL7, CYP51A1, DAGLB, DCXR, DDRGK1, DDX49, DDX5, DDX6, DECR1, DHX9, DICER1, DPYSL2, DPYSL3, ECE1, ECHDC1, ECPAS, EDEM3, EGFR, EIF4A3, ENO2, ERAP1, ERLEC1, ERLIN2, ESD, ETFA, EXOG, EXOSC10, EXOSC2, EXOSC5, EXOSC7, FAF2, FASTKD2, FBXO2, FBXW8, FLNB, FOXK1, FXR1, GALE, GALK1, GALM, GCDH, GCLC, GET4, GIPC1, GLA, GLMN, GLS, GLUD1, GLUL, GNPDA2, GNS, GOLT1B, GOT2, GPC1, GPC3, GPC6, GPD1L, GPI, GTPBP1, HACL1, HADHA, HADHB, HAGH, HDAC4, HEXA, HIBADH, HK1, HK2, HMGCL, HMMR, HMOX1, HMOX2, HNRNPM, HOOK3, HSD17B10, HSD17B4, HSP90AB1, HSP90B1, HSPA5, HSPB1, HUWE1, IGF2BP3, ILVBL, ITCH, KEAP1, LIN28A, LNPEP, LPCAT1, LRP1, LRPPRC, LSM4, LYPLA2, MAN1A1, MANBA, MAPK14, MCCC2, METAP1, MLH1, MOXD1, MSN, MTAP, MTREX, MVB12A, MYD88, MYEF2, NBAS, NDC1, NEDD4, NPM1, NQO2, NSF, NSFL1C, NSUN2, NT5C2, NTHL1, NUDT16, NUP107, NUP133, NUP160, NUP210, NUP35, NUP50, OGT, OXCT1, PARN, PCBP2, PCCA, PCCB, PDXDC1, PFKL, PFKM, PFKP, PGM2L1, PIK3R4, PKM, PLCB3, PLCG1, PLD3, PLK1, PM20D2, PNP, PNPT1, POLR2G, PRKAA1, PRKAG1, PRKCA, PRKCSH, PSMC6, PSMD1, PSMD2, PSMD9, PSME3, PTPN1, QDPR, RANBP2, RDX,	

					RELA, RIC1, RNH1, ROCK2, RPL13A, RPL22, RPL27A, RPL4, RPL5, RPL7A, RPS2, RPS21, RPS8, SAMD4B, SAMHD1, SARM1, SBDS, SCARB2, SCLY, SEC13, SEC22B, SEH1L, SEL1L, SERBP1, SESN2, SET, SH3GLB1, SIRT1, SIRT2, SKP2, SLC25A12, SLC44A1, SLIRP, SMARCC1, SMPDL3B, SNX17, SNX3, SNX9, SORD, SSB, STT3B, STX5, STYX, TBRG4, TGFB1I1, TIGAR, TIMP3, TKFC, TMF1, TOLLIP, TRIM71, TRIP12, TUT1, UBA6, UBE2G1, UBE2H, UBR4, UFL1, UGGT1, UGGT2, UNG, UPF2, UPF3B, USE1, USP15, USP19, USP47, USP5, USP7, USP9X, UTP25, VIM, VLDLR, VPS25, VPS35, VPS36, XPNPEP1, XRN1, XRN2, YOD1, ZC3HAV1, ZFP36L2]	
regulation of catabolic process	GO_BiologicalProcess-EBI-UniProt-GOA-ACAP-ARAP_13.05.2021_00h00	5.72E-57	13.81818	152	[AAAS, AGO2, AGTPBP1, AKT1, ANXA2, APOE, ATP2B4, ATP6V1A, AURKA, BAG2, BAG3, CALCOCO2, CAPN1, CAPNS1, CARHSP1, CASP3, CAV1, CCAR2, CCDC22, CD81, CDK5RAP3, CELF1, CHEK2, CIRBP, CNOT11, CTSC, CYP51A1, DAGLB, DAPK1, DDRGK1, DDX49, DDX6, DHX9, EGFR, EXOSC2, EXOSC5, EXOSC7, FASTKD2, FBXW8, FLNB, FOXK1, FXR1, GCLC, GIPC1, GLMN, GOLGA2, GPC3, GPSM1, GTPBP1, HDAC4, HMOX1, HNRNPM, HSP90AB1, HSPB1, HTT, HUWE1, IGF2BP3, ITCH, ITGB1, KEAP1, LPCAT1, LRP1, LRPPRC, LSM4, MAPK14, MAPK8, METAP1, MLH1, MSN, MTDH, MYD88, MYEF2, NBAS, NDC1, NEDD4, NNT, NPM1, NSF, NSUN2, NUP107, NUP133, NUP160, NUP210, NUP35, NUP50, OGT, OPTN, PARN, PIK3C2A, PIK3CA, PIP4K2B, PIP4K2C, PLK1, PNPT1, POLR2G, PRKAA1, PRKAB1, PRKAG1, PRKCA, PSMC6, PSMD1, PSMD2, PSMD9, PSME3, PTPN1, PYCARD, RAB8A, RANBP2, RDX, RELA,	No change

					RHEB, RIC1, ROCK2, RPL5, SAMD4B, SCARB2, SCFD1, SEC13, SEC22B, SEH1L, SERBP1, SESN2, SET, SH3GLB1, SIRT1, SIRT2, SLC25A12, SLC25A5, SLIRP, SMARCC1, SNX3, SNX6, SNX9, STX5, STYX, SUPT5H, TBRG4, TIGAR, TIMP3, TMF1, TRIM71, UFL1, USP19, USP5, USP7, VIM, VPS26A, VPS26B, VPS35, XRN1, ZC3HAV1, ZFP36L2]	
macromolecule catabolic process	GO_BiologicalProcess-EBI-UniProt-GOA-ACAP-ARAP_13.05.2021_00h00	5.72E-57	13.08725	195	[ABHD10, ABHD12, AGL, AGO1, AGO2, AGTPBP1, AKT1, ANKZF1, ANXA2, AP2A1, AP2A2, AP2B1, AP2M1, AP2S1, APOE, ARSB, AUP1, AURKA, AURKB, BAG2, BAG3, BIRC6, BUB1B, CAPN1, CAPN2, CARHSP1, CASP3, CAV1, CBL, CCAR2, CCDC22, CCNB1, CD2AP, CD44, CD81, CDK5RAP3, CELF1, CHEK2, CIRBP, CNOT11, CSDE1, CSPG4, CTNNB1, CTSC, CUL4A, CUL4B, CUL7, CYP51A1, DDRGK1, DDX49, DDX5, DDX6, DHX9, DICER1, ECPAS, EDEM3, EGFR, EIF4A3, ERLEC1, ERLIN2, EXOG, EXOSC10, EXOSC2, EXOSC5, EXOSC7, FAF2, FASTKD2, FBXO2, FBXW8, FXR1, GCLC, GET4, GIPC1, GLMN, GNS, GPC1, GPC3, GPC6, GTPBP1, HEXA, HMMR, HNRNPM, HSP90AB1, HSP90B1, HSPA5, HSPB1, HUWE1, IGF2BP3, ITCH, KEAP1, LIN28A, LPCAT1, LRP1, LRPPRC, LSM4, LYPLA2, MAN1A1, MANBA, MAPK14, METAP1, MLH1, MSN, MTREX, MVB12A, MYD88, MYEF2, NBAS, NEDD4, NPM1, NSF, NSFL1C, NSUN2, NUDT16, OGT, PARN, PCBP2, PFKM, PGM2L1, PIK3R4, PLK1, PNPT1, POLR2G, PRKCA, PSMC6, PSMD1, PSMD2, PSMD9, PSME3, PTPN1, RDX, RELA, RIC1, RNH1, ROCK2, RPL13A, RPL22, RPL27A, RPL4, RPL5, RPL7A, RPS2, RPS21, RPS8, SAMD4B, SEC22B, SEL1L, SERBP1, SET, SH3GLB1, SIRT1, SIRT2, SKP2, SLIRP, SMARCC1, SNX3, SNX9, SSB, STT3B, STX5, STYX, TBRG4, TGFB1I1,	No change

					TIMP3, TMF1, TOLLIP, TRIM71, TRIP12, TUT1, UBA6, UBE2G1, UBE2H, UBR4, UFL1, UGGT1, UGGT2, UPF2, UPF3B, USE1, USP15, USP19, USP47, USP5, USP7, USP9X, UTP25, VIM, VLDLR, VPS25, VPS35, VPS36, XRN1, XRN2, YOD1, ZC3HAV1, ZFP36L2]	
positive regulation of catabolic process	GO_BiologicalProcess-EBI-UniProt-GOA-ACAP-ARAP_13.05.2021_00h00	5.72E-57	13.42155	71	[AGO2, AGTPBP1, AKT1, APOE, AURKA, BAG2, BAG3, CALCOCO2, CAV1, CCDC22, CD81, CDK5RAP3, CELF1, CNOT11, CTSC, DAGLB, DAPK1, DDRGK1, DDX6, EXOSC2, EXOSC5, EXOSC7, FBXW8, FLNB, GCLC, GPC3, GPSM1, GTPBP1, HMOX1, HTT, HUWE1, ITCH, KEAP1, LPCAT1, LRP1, LSM4, MLH1, MSN, MTDH, NEDD4, NNT, NSF, OPTN, PARN, PIK3C2A, PIP4K2B, PIP4K2C, PLK1, PNPT1, POLR2G, PRKAA1, PSMC6, PTPN1, RDX, ROCK2, SAMD4B, SEC22B, SESN2, SH3GLB1, SIRT1, SIRT2, SLC25A5, SNX9, STX5, SUPT5H, TRIM71, UFL1, USP5, VPS35, ZC3HAV1, ZFP36L2]	No change
aromatic compound catabolic process	GO_BiologicalProcess-EBI-UniProt-GOA-ACAP-ARAP_13.05.2021_00h00	5.72E-57	14.00602	93	[ABCC1, ACAA1, ACAT1, AGO1, AGO2, AKT1, ALDH1L2, APOBEC3C, BLVRB, CARHSP1, CASP3, CELF1, CIRBP, CNOT11, CSDE1, DDX49, DDX5, DDX6, DHX9, DICER1, EIF4A3, EXOG, EXOSC10, EXOSC2, EXOSC5, EXOSC7, FASTKD2, FXR1, GTPBP1, HMOX1, HMOX2, HNRNPM, HSPB1, IGF2BP3, LIN28A, LRPPRC, LSM4, MAPK14, MLH1, MOXD1, MTAP, MTREX, MYD88, MYEF2, NBAS, NPM1, NSUN2, NT5C2, NTHL1, NUDT16, PARN, PM20D2, PNP, PNPT1, POLR2G, PON2, PRKCA, PSMC6, PSMD1, PSMD2, PSMD9, PSME3, QDPR, RNH1, ROCK2, RPL13A, RPL22, RPL27A, RPL4, RPL5, RPL7A, RPS2, RPS21, RPS8, SAMD4B, SAMHD1, SARM1, SERBP1, SET, SLIRP, SORD, SSB, TBRG4, TRIM71, TUT1, UNG, UPF2, UPF3B, VIM, XRN1, XRN2, ZC3HAV1, ZFP36L2]	Upregulation

90S preribosome	GO_CellularComponent-EBI-UniProt-GOA-ACAP-ARAP_13.05.2021_00h00	5.72E-57	54.54546	18	[BMS1, BOP1, HEATR1, IGF2BP3, IMP3, MPHOSPH10, NOL6, NOP14, NOP9, PES1, PWP2, RRP7A, TBL3, UTP18, UTP20, UTP4, WDR3, WDR36]	Upregulation
small-subunit processome	GO_CellularComponent-EBI-UniProt-GOA-ACAP-ARAP_13.05.2021_00h00	5.72E-57	50	21	[DCAF13, HEATR1, IGF2BP3, IMP3, MPHOSPH10, NOL10, NOL6, NOP14, NOP56, NOP58, PDCD11, PRKDC, PWP2, TBL3, UTP18, UTP20, UTP25, UTP4, WDR3, WDR36, XRCC5]	Upregulation
cellular amide metabolic process	GO_BiologicalProcess-EBI-UniProt-GOA-ACAP-ARAP_13.05.2021_00h00	5.72E-57	14.11387	176	[AASS, ABCF1, ACACA, ACAT1, ACLY, ACO1, ACOT13, ACOT9, ACSL1, ACSL4, ACSS2, ADAM10, ADSL, AGO1, AGO2, AIMP2, AKT1, ALDH1L2, AMDHD2, APEH, APOE, ARL6IP5, ASL, ASNS, BDH2, BIN1, C1QBP, CALR, CAPRIN1, CARNMT1, CASP3, CDC123, CDK5RAP1, CELF1, CIRBP, CNDP2, CNOT11, COPS5, CPD, CPZ, CSDE1, CYFIP1, DAPK1, DARS2, DDX39B, DDX6, DEGS1, DHPS, DHX33, DHX9, DPH1, DPH2, DPH6, DRG1, ECE1, EEF1E1, EEF2, EFL1, EGFR, EIF3A, EIF4A3, EIF5B, ERAP1, ETHE1, EXOSC2, EXOSC5, EXOSC7, FASTKD2, FXR1, GARS1, GCDH, GCLC, GCLM, GDAP1, GEMIN5, GLA, GNE, GNPDA2, GSS, GSTK1, GSTM2, GSTM3, GTPBP1, GUF1, HAGH, HEXA, HMGCL, HSD17B4, HSPB1, IGF2BP3, ILF3, LARS1, LARS2, LIN28A, LNPEP, LRPPRC, LSM4, MAPK1, MARS1, MCCC2, METAP1, MIPEP, MLH1, MME, MPC2, MRPS27, MRPS9, MTHFD1, MVD, MVK, NCL, NDRG1, NIBAN1, NLN, NPM1, NSUN5, OPLAH, PARN, PASK, PC, PCCA, PCCB, PICALM, PKM, PLPP3, PLXNB2, PM20D2, PNPT1, POLR2G, PRKAA1, PRKCSH, PRKDC, PTCD3, PURA, PUS7, QRSL1, RBM3, RELA, ROCK2, RPL13A, RPL22, RPL27A, RPL4, RPL5, RPL7A, RPS2, RPS21, RPS6KA1, RPS6KA3, RPS8, RRBP1, SAMD4B, SARS1, SARS2, SESN2, SLC25A32, SMPDL3B,	No change

					SPON1, SPTLC2, TARS2, THBS1, TIA1, TIGAR, TMED10, TMED2, TRAP1, TRIM71, TRMT10C, UPF3B, VIM, WARS1, XPNPEP1, XRN1, YARS2, ZC3H15, ZFP36L2]	
cellular nitrogen compound catabolic process	GO_BiologicalProcess-EBI-UniProt-GOA-ACAP-ARAP_13.05.2021_00h00	5.72E-57	14.17683	93	[ABCC1, ACAT1, AGO1, AGO2, AKT1, ALDH1L2, APOBEC3C, BLVRB, CARHSP1, CASP3, CELF1, CIRBP, CNOT11, CRMP1, CSDE1, DDX49, DDX5, DDX6, DHX9, DICER1, DPYSL2, DPYSL3, EIF4A3, EXOG, EXOSC10, EXOSC2, EXOSC5, EXOSC7, FASTKD2, FXR1, GTPBP1, HMOX1, HMOX2, HNRNPM, HSPB1, IGF2BP3, LIN28A, LRPPRC, LSM4, MAPK14, MLH1, MTAP, MTREX, MYD88, MYEF2, NBAS, NPM1, NSUN2, NT5C2, NTHL1, NUDT16, PARN, PM20D2, PNP, PNPT1, POLR2G, POR, PRKCA, PSMC6, PSMD1, PSMD2, PSMD9, PSME3, RNH1, ROCK2, RPL13A, RPL22, RPL27A, RPL4, RPL5, RPL7A, RPS2, RPS21, RPS8, SAMD4B, SAMHD1, SARM1, SERBP1, SET, SLIRP, SORD, SSB, TBRG4, TRIM71, TUT1, UNG, UPF2, UPF3B, VIM, XRN1, XRN2, ZC3HAV1, ZFP36L2]	Upregulation
heterocycle catabolic process	GO_BiologicalProcess-EBI-UniProt-GOA-ACAP-ARAP_13.05.2021_00h00	5.72E-57	14.24149	92	[ABCC1, ACAT1, AGO1, AGO2, AKT1, ALDH1L2, APOBEC3C, BLVRB, CARHSP1, CASP3, CELF1, CIRBP, CNOT11, CRMP1, CSDE1, DDX49, DDX5, DDX6, DHX9, DICER1, DPYSL2, DPYSL3, EIF4A3, EXOG, EXOSC10, EXOSC2, EXOSC5, EXOSC7, FASTKD2, FXR1, GTPBP1, HMOX1, HMOX2, HNRNPM, HSPB1, IGF2BP3, LIN28A, LRPPRC, LSM4, MAPK14, MLH1, MTAP, MTREX, MYD88, MYEF2, NBAS, NPM1, NSUN2, NT5C2, NTHL1, NUDT16, PARN, PM20D2, PNP, PNPT1, POLR2G, PRKCA, PSMC6, PSMD1, PSMD2, PSMD9, PSME3, RNH1, ROCK2, RPL13A, RPL22, RPL27A, RPL4, RPL5, RPL7A, RPS2, RPS21, RPS8, SAMD4B,	Upregulation

					SAMHD1, SARM1, SERBP1, SET, SLIRP, SORD, SSB, TBRG4, TRIM71, TUT1, UNG, UPF2, UPF3B, VIM, XRN1, XRN2, ZC3HAV1, ZFP36L2]	
organic cyclic compound catabolic process	GO_BiologicalProcess-EBI-UniProt-GOA-ACAP-ARAP_13.05.2021_00h00	5.72E-57	13.89685	97	[ABCC1, ACAA1, ACAT1, AGO1, AGO2, AKT1, ALDH1L2, APOBEC3C, APOE, BLVRB, CARHSP1, CASP3, CELF1, CIRBP, CNOT11, CRMP1, CSDE1, DDX49, DDX5, DDX6, DHX9, DICER1, DPYSL2, DPYSL3, EIF4A3, EXOG, EXOSC10, EXOSC2, EXOSC5, EXOSC7, FASTKD2, FXR1, GTPBP1, HMOX1, HMOX2, HNRNPM, HSPB1, IGF2BP3, LIN28A, LRPPRC, LSM4, MAPK14, MLH1, MOXD1, MTAP, MTREX, MYD88, MYEF2, NBAS, NPM1, NSUN2, NT5C2, NTHL1, NUDT16, PARN, PM20D2, PNP, PNPT1, POLR2G, PRKCA, PSMC6, PSMD1, PSMD2, PSMD9, PSME3, QDPR, RNH1, ROCK2, RPL13A, RPL22, RPL27A, RPL4, RPL5, RPL7A, RPS2, RPS21, RPS8, SAMD4B, SAMHD1, SARM1, SERBP1, SET, SLIRP, SNX17, SORD, SSB, TBRG4, TRIM71, TUT1, UNG, UPF2, UPF3B, VIM, XRN1, XRN2, ZC3HAV1, ZFP36L2]	Upregulation
organonitrogen compound catabolic process	GO_BiologicalProcess-EBI-UniProt-GOA-ACAP-ARAP_13.05.2021_00h00	5.72E-57	12.23776	175	[AASS, ABAT, ABCC1, ABHD10, ABHD12, ACAD8, ACAT1, AGTPBP1, AKT1, ALDH1L2, ALDH7A1, AMDHD2, ANKZF1, ANXA2, AP2A1, AP2A2, AP2B1, AP2M1, AP2S1, APOBEC3C, APOE, ARSB, ATP2B4, AUP1, AURKA, AURKB, BAG2, BAG3, BCAT2, BIRC6, BLVRB, BUB1B, CAPN2, CARNMT1, CAV1, CBL, CCAR2, CCDC22, CCNB1, CD2AP, CD44, CD81, CDK5RAP3, CHEK2, CRMP1, CSPG4, CTNNB1, CTSC, CUL4A, CUL4B, CUL7, CYP51A1, DDRGK1, DPYSL2, DPYSL3, ECE1, ECPAS, EDEM3, EGFR, ERAP1, ERLEC1, ERLIN2, FAF2, FBXO2, FBXW8, GCDH, GCLC, GET4, GIPC1, GLA, GLMN, GLS, GLUD1, GLUL, GNPDA2, GNS, GOLT1B, GOT2, GPC1, GPC3, GPC6,	No change

					HEXA, HIBADH, HMGCL, HMMR, HMOX1, HMOX2, HSD17B10, HSP90AB1, HSP90B1, HSPA5, HUWE1, ITCH, KEAP1, LNPEP, LPCAT1, LRP1, LYPLA2, MAN1A1, MANBA, MCCC2, METAP1, MOXD1, MSN, MTAP, MVB12A, NEDD4, NSF, NSFL1C, NT5C2, NTHL1, NUDT16, OGT, PCBP2, PDXDC1, PLK1, PM20D2, PNP, PRKCA, PSMC6, PSMD1, PSMD2, PSMD9, PSME3, QDPR, RDX, RELA, RIC1, RPL5, SAMHD1, SARM1, SBDS, SCLY, SEC22B, SEL1L, SIRT1, SIRT2, SKP2, SLC44A1, SMARCC1, SMPDL3B, SNX3, SNX9, SORD, STT3B, STX5, STYX, TGFB1I1, TIMP3, TMF1, TOLLIP, TRIM71, TRIP12, UBA6, UBE2G1, UBE2H, UBR4, UFL1, UGGT1, UGGT2, UNG, USE1, USP15, USP19, USP47, USP5, USP7, USP9X, UTP25, VLDLR, VPS25, VPS35, VPS36, XPNPEP1, YOD1]	
peptide metabolic process	GO_BiologicalProcess-EBI-UniProt-GOA-ACAP-ARAP_13.05.2021_00h00	5.72E-57	14.78723	139	[ABCF1, ACO1, ADAM10, AGO1, AGO2, AIMP2, AKT1, APEH, APOE, ARL6IP5, BDH2, BIN1, C1QBP, CALR, CAPRIN1, CARNMT1, CASP3, CDC123, CDK5RAP1, CELF1, CIRBP, CNDP2, CNOT11, COPS5, CPD, CPZ, CSDE1, CYFIP1, DAPK1, DARS2, DDX39B, DDX6, DHPS, DHX33, DHX9, DPH1, DPH2, DPH6, DRG1, ECE1, EEF1E1, EEF2, EFL1, EGFR, EIF3A, EIF4A3, EIF5B, ERAP1, ETHE1, EXOSC2, EXOSC5, EXOSC7, FASTKD2, FXR1, GARS1, GCLC, GCLM, GDAP1, GEMIN5, GSS, GSTK1, GSTM2, GSTM3, GTPBP1, GUF1, HAGH, HSPB1, IGF2BP3, ILF3, LARS1, LARS2, LIN28A, LNPEP, LRPPRC, LSM4, MAPK1, MARS1, METAP1, MIPEP, MLH1, MME, MRPS27, MRPS9, NCL, NDRG1, NIBAN1, NLN, NPM1, NSUN5, OPLAH, PARN, PASK, PICALM, PKM, PLXNB2, PNPT1, POLR2G, PRKCSH, PRKDC, PTCD3, PURA, PUS7, QRSL1, RBM3, RELA, ROCK2, RPL13A, RPL22,	Upregulation

					RPL27A, RPL4, RPL5, RPL7A, RPS2, RPS21, RPS6KA1, RPS6KA3, RPS8, RRBP1, SAMD4B, SARS1, SARS2, SESN2, SPON1, TARS2, THBS1, TIA1, TMED10, TMED2, TRAP1, TRIM71, TRMT10C, UPF3B, VIM, WARS1, XPNPEP1, XRN1, YARS2, ZC3H15, ZFP36L2]	
protein catabolic process	GO_BiologicalProcess-EBI-UniProt-GOA-ACAP-ARAP_13.05.2021_00h00	5.72E-57	11.80626	117	[ABHD10, ABHD12, AGTPBP1, AKT1, ANKZF1, ANXA2, AP2A1, AP2A2, AP2B1, AP2M1, AP2S1, APOE, AUP1, AURKA, AURKB, BAG2, BAG3, BIRC6, BUB1B, CAPN2, CAV1, CBL, CCAR2, CCDC22, CCNB1, CD2AP, CD81, CDK5RAP3, CHEK2, CTNNB1, CTSC, CUL4A, CUL4B, CUL7, CYP51A1, DDRGK1, ECPAS, EDEM3, EGFR, ERLEC1, ERLIN2, FAF2, FBXO2, FBXW8, GCLC, GET4, GIPC1, GLMN, GPC1, GPC3, HSP90AB1, HSP90B1, HSPA5, HUWE1, ITCH, KEAP1, LPCAT1, LRP1, LYPLA2, MAN1A1, MANBA, METAP1, MSN, MVB12A, NEDD4, NSF, NSFL1C, OGT, PCBP2, PLK1, PRKCA, PSMC6, PSMD1, PSMD2, PSMD9, PSME3, RDX, RELA, RIC1, RPL5, SEC22B, SEL1L, SIRT1, SIRT2, SKP2, SMARCC1, SNX3, SNX9, STT3B, STX5, STYX, TGFB1I1, TIMP3, TMF1, TOLLIP, TRIM71, TRIP12, UBA6, UBE2G1, UBE2H, UBR4, UFL1, UGGT1, UGGT2, USE1, USP15, USP19, USP47, USP5, USP7, USP9X, UTP25, VLDLR, VPS25, VPS35, VPS36, YOD1]	No change
regulation of cellular catabolic process	GO_BiologicalProcess-EBI-UniProt-GOA-ACAP-ARAP_13.05.2021_00h00	5.72E-57	13.59223	126	[AGO2, AGTPBP1, AKT1, ANXA2, APOE, ATP2B4, ATP6V1A, AURKA, BAG2, BAG3, CALCOCO2, CAPN1, CAPNS1, CARHSP1, CASP3, CAV1, CCAR2, CCDC22, CD81, CDK5RAP3, CELF1, CIRBP, CNOT11, CTSC, DAGLB, DAPK1, DDRGK1, DDX49, DDX6, DHX9, EXOSC2, EXOSC5, EXOSC7, FASTKD2, FBXW8, FOXK1, FXR1, GCLC, GIPC1, GOLGA2, GPSM1, GTPBP1, HMOX1, HNRNPM, HSP90AB1, HSPB1, HTT, IGF2BP3, ITCH, KEAP1,	No change

					LRP1, LRPPRC, LSM4, MAPK14, MAPK8, METAP1, MLH1, MSN, MTDH, MYD88, MYEF2, NBAS, NEDD4, NNT, NPM1, NSUN2, OGT, OPTN, PARN, PIK3C2A, PIK3CA, PIP4K2B, PIP4K2C, PLK1, PNPT1, POLR2G, PRKAA1, PRKAB1, PRKAG1, PRKCA, PSMC6, PSMD1, PSMD2, PSMD9, PSME3, PTPN1, PYCARD, RAB8A, RDX, RHEB, RIC1, ROCK2, RPL5, SAMD4B, SCARB2, SCFD1, SEC22B, SERBP1, SESN2, SET, SH3GLB1, SIRT1, SIRT2, SLC25A5, SLIRP, SMARCC1, SNX6, SNX9, STYX, SUPT5H, TBRG4, TIGAR, TIMP3, TMF1, TRIM71, UFL1, USP19, USP5, USP7, VIM, VPS26A, VPS26B, VPS35, XRN1, ZC3HAV1, ZFP36L2]	
nucleobase-containing compound catabolic process	GO_BiologicalProcess-EBI-UniProt-GOA-ACAP-ARAP_13.05.2021_00h00	5.72E-57	14.04399	83	[ACAT1, AGO1, AGO2, AKT1, APOBEC3C, CARHSP1, CASP3, CELF1, CIRBP, CNOT11, CSDE1, DDX49, DDX5, DDX6, DHX9, DICER1, EIF4A3, EXOG, EXOSC10, EXOSC2, EXOSC5, EXOSC7, FASTKD2, FXR1, GTPBP1, HNRNPM, HSPB1, IGF2BP3, LIN28A, LRPPRC, LSM4, MAPK14, MLH1, MTAP, MTREX, MYD88, MYEF2, NBAS, NPM1, NSUN2, NT5C2, NTHL1, NUDT16, PARN, PNP, PNPT1, POLR2G, PRKCA, PSMC6, PSMD1, PSMD2, PSMD9, PSME3, RNH1, ROCK2, RPL13A, RPL22, RPL27A, RPL4, RPL5, RPL7A, RPS2, RPS21, RPS8, SAMD4B, SAMHD1, SARM1, SERBP1, SET, SLIRP, SORD, SSB, TBRG4, TRIM71, TUT1, UNG, UPF2, UPF3B, VIM, XRN1, XRN2, ZC3HAV1, ZFP36L2]	Upregulation
cellular macromolecule catabolic process	GO_BiologicalProcess-EBI-UniProt-GOA-ACAP-ARAP_13.05.2021_00h00	5.72E-57	13.06773	164	[AGO1, AGO2, AGTPBP1, AKT1, ANKZF1, ANXA2, AP2A1, AP2A2, AP2B1, AP2M1, AP2S1, APOE, AUP1, AURKA, AURKB, BAG2, BIRC6, BUB1B, CAPN2, CARHSP1, CASP3, CAV1, CBL, CCAR2, CCDC22, CCNB1, CD2AP, CD81, CDK5RAP3, CELF1, CHEK2, CIRBP, CNOT11,	No change

					CSDE1, CTNNB1, CTSC, CUL4A, CUL4B, CUL7, DDRGK1, DDX49, DDX5, DDX6, DHX9, DICER1, ECPAS, EDEM3, EIF4A3, ERLEC1, ERLIN2, EXOG, EXOSC10, EXOSC2, EXOSC5, EXOSC7, FAF2, FASTKD2, FBXO2, FBXW8, FXR1, GCLC, GET4, GIPC1, GLMN, GPC1, GTPBP1, HNRNPM, HSP90AB1, HSP90B1, HSPA5, HSPB1, HUWE1, IGF2BP3, ITCH, KEAP1, LIN28A, LRP1, LRPPRC, LSM4, MAN1A1, MANBA, MAPK14, METAP1, MLH1, MSN, MTREX, MVB12A, MYD88, MYEF2, NBAS, NEDD4, NPM1, NSFL1C, NSUN2, NUDT16, OGT, PARN, PCBP2, PLK1, PNPT1, POLR2G, PRKCA, PSMC6, PSMD1, PSMD2, PSMD9, PSME3, RDX, RIC1, RNH1, ROCK2, RPL13A, RPL22, RPL27A, RPL4, RPL5, RPL7A, RPS2, RPS21, RPS8, SAMD4B, SEL1L, SERBP1, SET, SIRT1, SIRT2, SKP2, SLIRP, SMARCC1, SSB, STT3B, STYX, TBRG4, TGFB1I1, TMF1, TOLLIP, TRIM71, TRIP12, TUT1, UBA6, UBE2G1, UBE2H, UBR4, UFL1, UGGT1, UGGT2, UPF2, UPF3B, USP15, USP19, USP47, USP5, USP7, USP9X, VIM, VLDLR, VPS25, VPS35, VPS36, XRN1, XRN2, YOD1, ZC3HAV1, ZFP36L2]	
snoRNA binding	GO_MolecularFunction-EBI-UniProt-GOA-ACAP-ARAP_13.05.2021_00h00	5.72E-57	43.24324	16	[BMS1, DDX21, DHX37, HEATR1, IGF2BP3, IMP3, NOP14, NOP56, NOP58, NUDT16, PRKDC, TBL3, TSR1, UTP25, WDR3, XRCC5]	Upregulation
positive regulation of cellular catabolic process	GO_BiologicalProcess-EBI-UniProt-GOA-ACAP-ARAP_13.05.2021_00h00	5.72E-57	13.72549	63	[AGO2, AGTPBP1, AKT1, APOE, AURKA, BAG2, BAG3, CALCOCO2, CAV1, CCDC22, CD81, CDK5RAP3, CELF1, CNOT11, CTSC, DAGLB, DAPK1, DDRGK1, DDX6, EXOSC2, EXOSC5, EXOSC7, FBXW8, GCLC, GPSM1, GTPBP1, HMOX1, HTT, ITCH, KEAP1, LRP1, LSM4, MLH1, MSN, MTDH, NNT, OPTN, PARN, PIK3C2A, PIP4K2B, PIP4K2C, PLK1, PNPT1, POLR2G, PRKAA1, PSMC6, PTPN1, RDX,	No change

					ROCK2, SAMD4B, SESN2, SH3GLB1, SIRT1, SIRT2, SLC25A5, SNX9, SUPT5H, TRIM71, UFL1, USP5, VPS35, ZC3HAV1, ZFP36L2]	
negative regulation of cellular amide metabolic process	GO_BiologicalProcess-EBI-UniProt-GOA-ACAP-ARAP_13.05.2021_00h00	5.72E-57	16.59193	37	[AGO1, AGO2, APOE, BIN1, CALR, CAPRIN1, CELF1, CIRBP, CNOT11, DAPK1, DDX6, EIF4A3, EXOSC2, EXOSC5, EXOSC7, FXR1, IGF2BP3, ILF3, LIN28A, LSM4, MLH1, NCL, PARN, PNPT1, POLR2G, PRKAA1, PURA, PUS7, ROCK2, RPL13A, SAMD4B, SESN2, SPON1, TIA1, TRIM71, XRN1, ZFP36L2]	Upregulation
positive regulation of cellular amide metabolic process	GO_BiologicalProcess-EBI-UniProt-GOA-ACAP-ARAP_13.05.2021_00h00	5.72E-57	19.54023	34	[AGO2, C1QBP, CASP3, CDC123, CDK5RAP1, CIRBP, DDX39B, DHX9, EEF2, EIF4A3, FASTKD2, FXR1, GUF1, LIN28A, MAPK1, MRPS27, NIBAN1, NPM1, NSUN5, PASK, PICALM, PKM, PLXNB2, POLR2G, PRKCSH, PRKDC, RBM3, RELA, ROCK2, RPL5, THBS1, TRMT10C, UPF3B, VIM]	Upregulation
regulation of protein catabolic process	GO_BiologicalProcess-EBI-UniProt-GOA-ACAP-ARAP_13.05.2021_00h00	5.72E-57	13.58025	55	[AGTPBP1, AKT1, ANXA2, APOE, AURKA, BAG2, CAV1, CCAR2, CCDC22, CD81, CDK5RAP3, CHEK2, CTSC, CYP51A1, DDRGK1, EGFR, FBXW8, GCLC, GIPC1, GPC3, HSP90AB1, HUWE1, ITCH, KEAP1, LPCAT1, LRP1, METAP1, MSN, NEDD4, NSF, OGT, PLK1, PRKCA, PSMC6, PSMD1, PSMD2, PSME3, RDX, RELA, RIC1, RPL5, SEC22B, SIRT2, SMARCC1, SNX3, SNX9, STX5, STYX, TIMP3, TMF1, UFL1, USP19, USP5, USP7, VPS35]	No change
ribosome biogenesis	GO_BiologicalProcess-EBI-UniProt-GOA-ACAP-ARAP_13.05.2021_00h00	5.72E-57	25.9887	92	[AATF, BMS1, BOP1, C1QBP, CUL4A, CUL4B, DCAF13, DDX10, DDX18, DDX21, DDX28, DDX31, DDX47, DDX49, DDX52, DDX54, DHX30, DHX37, EFL1, EIF4A3, ERCC2, ESF1, EXOSC10, EXOSC2, EXOSC5, EXOSC7, FASTKD2, FTSJ3, GEMIN4, GLUL, GNL2, HEATR1, IGF2BP3, IMP3, LAS1L, LSG1, LYAR, MDN1, MPHOSPH10, MTREX, MYBBP1A, NEDD4, NLE1, NOL10, NOL11, NOL6, NOL9, NOP14, NOP56, NOP58, NOP9, NPM1, NPM3, NSUN5, NUDT16, NVL, PDCD11,	Upregulation

					PELP1, PES1, PRKDC, PWP2, RAN, RPF2, RPL13A, RPL22, RPL27A, RPL4, RPL5, RPL7A, RPS2, RPS21, RPS8, RRP12, RRP7A, RRS1, SBDS, SDAD1, TBL3, TEX10, TFB1M, TSR1, UTP15, UTP18, UTP20, UTP25, UTP4, WDR18, WDR3, WDR36, WDR43, XRCC5, XRN2]	
amide biosynthetic process	GO_BiologicalProcess-EBI-UniProt-GOA-ACAP-ARAP_13.05.2021_00h00	5.72E-57	14.00652	129	[ABCF1, ACACA, ACAT1, ACLY, ACO1, ACSL1, ACSL4, ACSS2, ADSL, AGO1, AGO2, AIMP2, AKT1, APEH, ASL, ASNS, BDH2, C1QBP, CALR, CAPRIN1, CDC123, CDK5RAP1, CELF1, CIRBP, CNDP2, CNOT11, COPS5, CSDE1, CYFIP1, DAPK1, DARS2, DDX39B, DDX6, DEGS1, DHPS, DHX33, DHX9, DPH1, DPH2, DPH6, DRG1, EEF1E1, EEF2, EFL1, EGFR, EIF3A, EIF4A3, EIF5B, EXOSC2, EXOSC5, EXOSC7, FASTKD2, FXR1, GARS1, GCDH, GCLC, GCLM, GEMIN5, GSS, GTPBP1, GUF1, HAGH, HSPB1, IGF2BP3, ILF3, LARS1, LARS2, LIN28A, LRPPRC, LSM4, MAPK1, MARS1, METAP1, MLH1, MPC2, MRPS27, MRPS9, NCL, NDRG1, NIBAN1, NPM1, NSUN5, OPLAH, PARN, PASK, PKM, PLXNB2, PNPT1, POLR2G, PRKAA1, PRKCSH, PRKDC, PTCD3, PURA, PUS7, QRSL1, RBM3, ROCK2, RPL13A, RPL22, RPL27A, RPL4, RPL5, RPL7A, RPS2, RPS21, RPS6KA1, RPS6KA3, RPS8, RRBP1, SAMD4B, SARS1, SARS2, SESN2, SPTLC2, TARS2, THBS1, TIA1, TMED2, TRAP1, TRIM71, TRMT10C, UPF3B, VIM, WARS1, XRN1, YARS2, ZC3H15, ZFP36L2]	Upregulation
posttranscriptional regulation of gene expression	GO_BiologicalProcess-EBI-UniProt-GOA-ACAP-ARAP_13.05.2021_00h00	5.72E-57	16.48501	121	[AAAS, ABCF1, ACO1, ADAR, AGO1, AGO2, AKT1, C1QBP, CALR, CAPRIN1, CARHSP1, CAV1, CDC123, CDK5RAP1, CELF1, CIRBP, CNOT11, COPS5, CSDE1, CYFIP1, DAPK1, DDX39B, DDX49, DDX5, DDX6, DHX9, DICER1, DPH1, DPH2, DPH6, EEF2, EFL1, EGFR, EIF3A, EIF4A3, EIF5B, EPB41L5, EXOSC2, EXOSC5, EXOSC7,	Upregulation

					FASTKD2, FXR1, GARS1, GEMIN5, GTPBP1, GUF1, HNRNPM, HSPB1, IGF2BP3, ILF3, LIN28A, LRPPRC, LSM4, MAPK1, MAPK14, MATR3, METAP1, MLH1, MRPS27, MYD88, MYEF2, NCL, NDC1, NIBAN1, NPM1, NSUN2, NSUN5, NUP107, NUP133, NUP160, NUP210, NUP35, NUP50, PARN, PASK, PKM, PLXNB2, PNPT1, POLR2A, POLR2B, POLR2G, PRKCA, PRKCSH, PRKDC, PSMC6, PSMD1, PSMD2, PSMD9, PSME3, PTCD3, PURA, PUS7, RAN, RANBP2, RBM3, ROCK2, RPL13A, RPL22, RPL5, RPS6KA1, RPS6KA3, SAMD4B, SEC13, SEH1L, SERBP1, SESN2, SET, SPOUT1, TBRG4, THBS1, TIA1, TMED2, TRAP1, TRIM71, TRMT10C, UPF3B, VIM, XPO5, XRN1, ZC3H7B, ZFP36L2]	
ribosomal small subunit biogenesis	GO_BiologicalProcess-EBI-UniProt-GOA-ACAP-ARAP_13.05.2021_00h00	5.72E-57	32.43243	24	[BMS1, DCAF13, DDX52, DHX37, ERCC2, HEATR1, NOL10, NOL11, NOP14, NOP9, NPM1, PRKDC, PWP2, RPS21, RPS8, RRP7A, RRS1, TBL3, TSR1, UTP20, UTP25, UTP4, WDR3, XRCC5]	Upregulation
peptide biosynthetic process	GO_BiologicalProcess-EBI-UniProt-GOA-ACAP-ARAP_13.05.2021_00h00	5.72E-57	15.01306	115	[ABCF1, ACO1, AGO1, AGO2, AIMP2, AKT1, APEH, BDH2, C1QBP, CALR, CAPRIN1, CDC123, CDK5RAP1, CELF1, CIRBP, CNDP2, CNOT11, COPS5, CSDE1, CYFIP1, DAPK1, DARS2, DDX39B, DDX6, DHPS, DHX33, DHX9, DPH1, DPH2, DPH6, DRG1, EEF1E1, EEF2, EFL1, EGFR, EIF3A, EIF4A3, EIF5B, EXOSC2, EXOSC5, EXOSC7, FASTKD2, FXR1, GARS1, GCLC, GCLM, GEMIN5, GSS, GTPBP1, GUF1, HAGH, HSPB1, IGF2BP3, ILF3, LARS1, LARS2, LIN28A, LRPPRC, LSM4, MAPK1, MARS1, METAP1, MLH1, MRPS27, MRPS9, NCL, NDRG1, NIBAN1, NPM1, NSUN5, OPLAH, PARN, PASK, PKM, PLXNB2, PNPT1, POLR2G, PRKCSH, PRKDC, PTCD3, PURA, PUS7, QRSL1, RBM3, ROCK2, RPL13A, RPL22, RPL27A, RPL4, RPL5,	Upregulation

					RPL7A, RPS2, RPS21, RPS6KA1, RPS6KA3, RPS8, RRBP1, SAMD4B, SARS1, SARS2, SESN2, TARS2, THBS1, TIA1, TMED2, TRAP1, TRIM71, TRMT10C, UPF3B, VIM, WARS1, XRN1, YARS2, ZC3H15, ZFP36L2]	
cellular protein catabolic process	GO_BiologicalProcess-EBI-UniProt-GOA-ACAP-ARAP_13.05.2021_00h00	5.72E-57	11.76471	98	[AGTPBP1, AKT1, ANKZF1, ANXA2, AP2A1, AP2A2, AP2B1, AP2M1, AP2S1, APOE, AUP1, AURKA, AURKB, BAG2, BIRC6, BUB1B, CAPN2, CAV1, CBL, CCAR2, CCDC22, CCNB1, CD2AP, CD81, CDK5RAP3, CHEK2, CTNNB1, CTSC, CUL4A, CUL4B, CUL7, DDRGK1, ECPAS, EDEM3, ERLEC1, ERLIN2, FAF2, FBXO2, FBXW8, GCLC, GET4, GIPC1, GLMN, HSP90AB1, HSP90B1, HSPA5, HUWE1, ITCH, KEAP1, LRP1, MAN1A1, METAP1, MSN, MVB12A, NEDD4, NSFL1C, OGT, PCBP2, PLK1, PRKCA, PSMC6, PSMD1, PSMD2, PSMD9, PSME3, RDX, RIC1, RPL5, SEL1L, SIRT1, SIRT2, SKP2, SMARCC1, STT3B, STYX, TGFB1I1, TMF1, TOLLIP, TRIM71, TRIP12, UBA6, UBE2G1, UBE2H, UBR4, UFL1, UGGT1, UGGT2, USP15, USP19, USP47, USP5, USP7, USP9X, VLDLR, VPS25, VPS35, VPS36, YOD1]	No change
nucleic acid phosphodiester bond hydrolysis	GO_BiologicalProcess-EBI-UniProt-GOA-ACAP-ARAP_13.05.2021_00h00	5.72E-57	16.94915	60	[AGO1, AGO2, AKT1, BMS1, BOP1, CASP3, CPSF2, CPSF3, CSTF3, CUL4A, CUL4B, DICER1, ELAC2, ENDOD1, ERCC2, EXD2, EXOG, EXOSC10, EXOSC2, EXOSC5, EXOSC7, FSCN1, G3BP1, LAS1L, MAP1S, NOL9, NOP14, NOP9, NPM1, NTHL1, NUDT16, PARN, PARP1, PLD3, PNPT1, POLD1, POLE, POLR1B, PRORP, RFC1, RFC2, RFC3, RFC5, RNH1, RPA1, RPA2, RPA3, RPS21, RRS1, SAMHD1, SIRT1, TBL3, TDP1, TDP2, TSEN34, TSR1, TUT1, UTP20, XRN1, XRN2]	Upregulation
regulation of RNA stability	GO_BiologicalProcess-EBI-UniProt-GOA-ACAP-ARAP_13.0	5.72E-57	16.09195	42	[AGO2, AKT1, CARHSP1, CELF1, CIRBP, CNOT11, DDX49, DDX6, DHX9, EXOSC2, EXOSC5, EXOSC7, FASTKD2, FXR1, HNRNPM, HSPB1, IGF2BP3, LSM4, MAPK14, MLH1,	Upregulation

	5.2021_00h00				MYD88, MYEF2, NPM1, NSUN2, PARN, PNPT1, POLR2G, PRKCA, PSMC6, PSMD1, PSMD2, PSMD9, PSME3, ROCK2, SAMD4B, SERBP1, SET, TBRG4, TRIM71, VIM, XRN1, ZFP36L2]	
nucleolus	GO_CellularComponent-EBI-UniProt-GOA-ACAP-ARAP_13.05.2021_00h00	5.72E-57	18.64734	193	[AATF, ABHD14B, ACACA, ACIN1, ADAR, ADARB1, AGTPBP1, ARFGEF1, ARL2, BMS1, BOP1, BRWD1, C1QBP, CASK, CBX5, CCND2, CD2AP, CDCA8, CDK5RAP3, CDK7, CKAP5, CMPK1, COG7, CTCF, DAXX, DCAF1, DCAF13, DDRGK1, DDX18, DDX21, DDX24, DDX28, DDX31, DDX46, DDX47, DDX5, DDX52, DDX54, DDX6, DHX15, DHX33, DHX37, DHX9, DNTTIP2, DPH6, EEF1E1, EIF3A, EIF4A3, ELP3, ERGIC2, ESF1, EXOSC10, EXOSC2, EXOSC5, EXOSC7, FANCD2, FTSJ3, FXR1, GEMIN4, GET3, GET4, GNL2, GNL3, GOLGA3, GTF3C1, GTF3C3, HEATR1, HMOX1, HNRNPM, HSPA9, IGF2BP3, ILF2, ILF3, IMP3, IPO5, KAT7, KRT18, LAS1L, LIN28A, LRWD1, LYAR, MACROH2A1, MAGED2, MAP1S, MARS1, MCM2, MCM3, MCM7, MDN1, MEAK7, METTL1, MKI67, MPHOSPH10, MRPS27, MRPS9, MTDH, MTREX, MYBBP1A, MYO1C, MYO1E, NCAPG, NCL, NEDD4, NLE1, NOL10, NOL11, NOL6, NOL9, NOP14, NOP56, NOP58, NOP9, NPM1, NPM3, NSUN2, NSUN5, NUDT16, NUSAP1, NVL, ORC4, OSBP, P3H4, PARN, PARP1, PDCD11, PELP1, PES1, PNO1, POLA1, POLR1B, PPID, PPM1B, PRKDC, PSPC1, PWP2, PYCARD, RAI14, RAN, RBBP5, RBPJ, RCC2, RELA, RFC1, RIOX1, RPF2, RPL13A, RPL4, RPL5, RPL7A, RPS2, RPS6KA3, RRP12, RRP7A, RRS1, SBDS, SDAD1, SELENBP1, SETD7, SIN3A, SIRT1, SIRT2, SKP2, SMARCA4, SMARCA5, SMC2, SPATS2L, SPTBN1, SSRP1, TBL3, TDP2, TEX10, THUMPD3, TMA16, TOP2A,	Upregulation

					TSEN34, TSR1, TUT1, TWF1, TXNRD1, UPF3B, UTP15, UTP18, UTP20, UTP25, UTP4, VRK1, WDR18, WDR3, WDR36, WDR43, XRCC5, XRCC6, XRN2]	
RNA catabolic process	GO_BiologicalProcess-EBI-UniProt-GOA-ACAP-ARAP_13.05.2021_00h00	5.72E-57	15.73034	70	[AGO1, AGO2, AKT1, CARHSP1, CELF1, CIRBP, CNOT11, CSDE1, DDX49, DDX5, DDX6, DHX9, EIF4A3, EXOSC10, EXOSC2, EXOSC5, EXOSC7, FASTKD2, FXR1, GTPBP1, HNRNPM, HSPB1, IGF2BP3, LIN28A, LRPPRC, LSM4, MAPK14, MLH1, MTREX, MYD88, MYEF2, NBAS, NPM1, NSUN2, NUDT16, PARN, PNPT1, POLR2G, PRKCA, PSMC6, PSMD1, PSMD2, PSMD9, PSME3, RNH1, ROCK2, RPL13A, RPL22, RPL27A, RPL4, RPL5, RPL7A, RPS2, RPS21, RPS8, SAMD4B, SERBP1, SET, SLIRP, SSB, TBRG4, TRIM71, TUT1, UPF2, UPF3B, VIM, XRN1, XRN2, ZC3HAV1, ZFP36L2]	Upregulation
translation	GO_BiologicalProcess-EBI-UniProt-GOA-ACAP-ARAP_13.05.2021_00h00	5.72E-57	14.7139	108	[ABCF1, ACO1, AGO1, AGO2, AIMP2, AKT1, APEH, C1QBP, CALR, CAPRIN1, CDC123, CDK5RAP1, CELF1, CIRBP, CNOT11, COPS5, CSDE1, CYFIP1, DAPK1, DARS2, DDX39B, DDX6, DHPS, DHX33, DHX9, DPH1, DPH2, DPH6, DRG1, EEF1E1, EEF2, EFL1, EGFR, EIF3A, EIF4A3, EIF5B, EXOSC2, EXOSC5, EXOSC7, FASTKD2, FXR1, GARS1, GEMIN5, GTPBP1, GUF1, HSPB1, IGF2BP3, ILF3, LARS1, LARS2, LIN28A, LRPPRC, LSM4, MAPK1, MARS1, METAP1, MLH1, MRPS27, MRPS9, NCL, NDRG1, NIBAN1, NPM1, NSUN5, PARN, PASK, PKM, PLXNB2, PNPT1, POLR2G, PRKCSH, PRKDC, PTCD3, PURA, PUS7, QRSL1, RBM3, ROCK2, RPL13A, RPL22, RPL27A, RPL4, RPL5, RPL7A, RPS2, RPS21, RPS6KA1, RPS6KA3, RPS8, RRBP1, SAMD4B, SARS1, SARS2, SESN2, TARS2, THBS1, TIA1, TMED2, TRAP1, TRIM71, TRMT10C, UPF3B, VIM, WARS1, XRN1, YARS2, ZC3H15, ZFP36L2]	Upregulation

U3 snoRNA binding	GO_MolecularFunction-EBI-UniProt-GOA-ACAP-ARAP_13.05.2021_00h00	5.72E-57	70	7	[BMS1, DHX37, PRKDC, TBL3, TSR1, UTP25, XRCC5]	Upregulation
mRNA catabolic process	GO_BiologicalProcess-EBI-UniProt-GOA-ACAP-ARAP_13.05.2021_00h00	5.72E-57	16	64	[AGO1, AGO2, AKT1, CARHSP1, CELF1, CIRBP, CNOT11, CSDE1, DDX5, DDX6, DHX9, EIF4A3, EXOSC10, EXOSC2, EXOSC5, EXOSC7, FASTKD2, FXR1, GTPBP1, HNRNPM, HSPB1, IGF2BP3, LSM4, MAPK14, MLH1, MYD88, MYEF2, NBAS, NPM1, NUDT16, PARN, PNPT1, POLR2G, PRKCA, PSMC6, PSMD1, PSMD2, PSMD9, PSME3, RNH1, ROCK2, RPL13A, RPL22, RPL27A, RPL4, RPL5, RPL7A, RPS2, RPS21, RPS8, SAMD4B, SERBP1, SET, SSB, TBRG4, TRIM71, TUT1, UPF2, UPF3B, VIM, XRN1, XRN2, ZC3HAV1, ZFP36L2]	Upregulation
regulation of translation	GO_BiologicalProcess-EBI-UniProt-GOA-ACAP-ARAP_13.05.2021_00h00	5.72E-57	16.84211	80	[ABCF1, ACO1, AGO1, AGO2, AKT1, C1QBP, CALR, CAPRIN1, CDC123, CDK5RAP1, CELF1, CIRBP, CNOT11, COPS5, CSDE1, CYFIP1, DAPK1, DDX39B, DDX6, DHX9, DPH1, DPH2, DPH6, EEF2, EFL1, EIF3A, EIF4A3, EIF5B, EXOSC2, EXOSC5, EXOSC7, FASTKD2, FXR1, GEMIN5, GTPBP1, GUF1, HSPB1, IGF2BP3, ILF3, LIN28A, LRPPRC, LSM4, MAPK1, METAP1, MLH1, MRPS27, NCL, NIBAN1, NPM1, NSUN5, PARN, PASK, PKM, PLXNB2, PNPT1, POLR2G, PRKCSH, PRKDC, PTCD3, PURA, PUS7, RBM3, ROCK2, RPL13A, RPL22, RPL5, RPS6KA1, RPS6KA3, SAMD4B, SESN2, THBS1, TIA1, TMED2, TRAP1, TRIM71, TRMT10C, UPF3B, VIM, XRN1, ZFP36L2]	Upregulation
mRNA metabolic process	GO_BiologicalProcess-EBI-UniProt-GOA-ACAP-ARAP_13.05.2021_00h00	5.72E-57	15.8011	143	[ACIN1, ADAR, ADARB1, AGO1, AGO2, AKT1, AP3B1, C1QBP, CARHSP1, CCAR1, CCAR2, CCNB1, CCNH, CDC73, CDK7, CELF1, CIRBP, CNOT11, CPSF2, CPSF3, CSDE1, CSTF1, CSTF3, CTNNBL1, CWC27, DAZAP1,	Upregulation

					DDX20, DDX39B, DDX41, DDX42, DDX46, DDX47, DDX5, DDX6, DHX15, DHX8, DHX9, EFTUD2, EIF4A3, ERCC2, EXOSC10, EXOSC2, EXOSC5, EXOSC7, FASTKD2, FXR1, GEMIN4, GEMIN5, GTPBP1, HNRNPF, HNRNPL, HNRNPLL, HNRNPM, HSPB1, IGF2BP3, IWS1, KDM1A, LSM4, MAPK14, MBNL1, MLH1, MTREX, MYD88, MYEF2, NBAS, NCL, NPM1, NSUN2, NUDT16, PABPN1, PARN, PAXBP1, PCBP2, PDCD11, PNPT1, POLR2A, POLR2B, POLR2G, PRKCA, PRPF38A, PRPF38B, PRPF40A, PRPF8, PSIP1, PSMC6, PSMD1, PSMD2, PSMD9, PSME3, PSPC1, PTBP2, PUS1, PUS3, PUS7, RBM15, RBM25, RBM26, RBM3, RBM6, RNH1, ROCK2, RPL13A, RPL22, RPL27A, RPL4, RPL5, RPL7A, RPRD1A, RPS2, RPS21, RPS8, SAMD4B, SART3, SERBP1, SET, SF3A3, SF3B1, SLTM, SNRNP200, SNRPA1, SNRPD3, SRPK1, SRSF1, SRSF11, SRSF7, SSB, SUPT5H, SUPT6H, SYMPK, TBRG4, TIA1, TRIM71, TRMT10C, TSEN34, TTF2, TUT1, UPF2, UPF3B, VIM, XRN1, XRN2, ZC3HAV1, ZFP36L2]	
RNA phosphodiester bond hydrolysis	GO_BiologicalProcess-EBI-UniProt-GOA-ACAP-ARAP_13.05.2021_00h00	5.72E-57	18.57924	34	[AGO1, AGO2, BMS1, BOP1, CPSF2, CPSF3, CSTF3, DICER1, ELAC2, EXD2, EXOG, EXOSC10, EXOSC2, EXOSC5, EXOSC7, NOL9, NOP14, NOP9, NPM1, NUDT16, PARN, PNPT1, PRORP, RNH1, RPS21, RRS1, SAMHD1, TBL3, TSEN34, TSR1, TUT1, UTP20, XRN1, XRN2]	Upregulation
regulation of mRNA metabolic process	GO_BiologicalProcess-EBI-UniProt-GOA-ACAP-ARAP_13.05.2021_00h00	5.72E-57	16.14583	62	[AGO2, AKT1, C1QBP, CARHSP1, CCNB1, CDC73, CELF1, CIRBP, CNOT11, DAZAP1, DDX5, DDX6, DHX9, EXOSC2, EXOSC5, EXOSC7, FASTKD2, FXR1, GTPBP1, HNRNPL, HNRNPM, HSPB1, IGF2BP3, IWS1, LSM4, MAPK14, MBNL1, MLH1, MYD88, MYEF2, NBAS, NCL, NPM1, PABPN1, PARN, PNPT1, POLR2G, PRKCA, PSMC6, PSMD1,	Upregulation

					PSMD2, PSMD9, PSME3, RBM15, RBM25, RBM3, ROCK2, SAMD4B, SERBP1, SET, SLTM, SRPK1, SRSF7, SUPT5H, SUPT6H, TBRG4, TIA1, TRIM71, VIM, XRN1, ZC3HAV1, ZFP36L2]	
mRNA processing	GO_BiologicalProcess-EBI-UniProt-GOA-ACAP-ARAP_13.05.2021_00h00	5.72E-57	16.24088	89	[ACIN1, ADAR, ADARB1, C1QBP, CCAR1, CCAR2, CCNB1, CCNH, CDC73, CDK7, CELF1, CIRBP, CPSF2, CPSF3, CSTF1, CSTF3, CTNNBL1, CWC27, DAZAP1, DDX20, DDX39B, DDX41, DDX42, DDX46, DDX47, DDX5, DHX15, DHX8, DHX9, EFTUD2, EIF4A3, ERCC2, FXR1, GEMIN4, GEMIN5, HNRNPF, HNRNPL, HNRNPLL, HNRNPM, IWS1, KDM1A, LSM4, MBNL1, MTREX, NCL, NPM1, PABPN1, PAXBP1, PCBP2, PDCD11, PNPT1, POLR2A, POLR2B, POLR2G, PRKCA, PRPF38A, PRPF38B, PRPF40A, PRPF8, PSIP1, PSPC1, PTBP2, RBM15, RBM25, RBM26, RBM3, RBM6, RPRD1A, SART3, SF3A3, SF3B1, SLTM, SNRNP200, SNRPA1, SNRPD3, SRPK1, SRSF1, SRSF11, SRSF7, SUPT5H, SUPT6H, SYMPK, TBRG4, TIA1, TSEN34, TTF2, TUT1, UPF3B, XRN2]	Upregulation
RNA splicing	GO_BiologicalProcess-EBI-UniProt-GOA-ACAP-ARAP_13.05.2021_00h00	5.72E-57	16.17647	77	[ACIN1, C1QBP, CCAR1, CCAR2, CELF1, CIRBP, CPSF2, CPSF3, CSTF1, CSTF3, CTNNBL1, CWC27, DAZAP1, DDX20, DDX39B, DDX41, DDX42, DDX46, DDX47, DDX5, DHX15, DHX8, DHX9, EFTUD2, EIF4A3, FXR1, GEMIN4, GEMIN5, HNRNPF, HNRNPL, HNRNPLL, HNRNPM, IWS1, KDM1A, LSM4, MBNL1, MPHOSPH10, MTREX, NCL, NPM1, PABPN1, PAXBP1, PCBP2, POLR2A, POLR2B, POLR2G, PPP1R9B, PRPF38A, PRPF38B, PRPF40A, PRPF8, PSIP1, PSPC1, PTBP2, RBM15, RBM25, RBM3, RBM6, SART3, SF3A3, SF3B1, SNRNP200, SNRPA1, SNRPD3, SRPK1, SRSF1, SRSF11, SRSF7, SUPT6H, SYMPK, TELO2, TIA1, TRPT1, TSEN34, TTF2, UPF3B, ZNF638]	Upregulation

negative regulation of translation	GO_BiologicalProcess-EBI-UniProt-GOA-ACAP-ARAP_13.05.2021_00h00	5.72E-57	16.66667	33	[AGO1, AGO2, CALR, CAPRIN1, CELF1, CIRBP, CNOT11, DAPK1, DDX6, EIF4A3, EXOSC2, EXOSC5, EXOSC7, FXR1, IGF2BP3, ILF3, LIN28A, LSM4, MLH1, NCL, PARN, PNPT1, POLR2G, PURA, PUS7, ROCK2, RPL13A, SAMD4B, SESN2, TIA1, TRIM71, XRN1, ZFP36L2]	Upregulation
ncRNA processing	GO_BiologicalProcess-EBI-UniProt-GOA-ACAP-ARAP_13.05.2021_00h00	5.72E-57	22.03024	102	[AGO1, AGO2, BMS1, BOP1, CDK5RAP1, DCAF13, DDX10, DDX18, DDX21, DDX47, DDX49, DDX52, DDX54, DHX37, DICER1, EIF4A3, ELAC2, ELP1, ELP3, ERCC2, ESF1, EXOSC10, EXOSC2, EXOSC5, EXOSC7, FTSJ3, GARS1, GEMIN4, HEATR1, HSD17B10, IGF2BP3, IMP3, LAS1L, LIN28A, LYAR, MDN1, METTL1, MPHOSPH10, MTREX, NEDD4, NOL10, NOL11, NOL6, NOL9, NOP14, NOP56, NOP58, NOP9, NPM3, NSUN2, NSUN5, NUDT16, NVL, PARN, PDCD11, PELP1, PES1, PRKDC, PRORP, PTCD1, PUS1, PUS3, PUS7, PWP2, RPF2, RPL13A, RPL22, RPL27A, RPL4, RPL5, RPL7A, RPS2, RPS21, RPS8, RRP12, RRP7A, RRS1, SARS1, SBDS, SSB, TBL3, TEX10, TFB1M, THUMPD3, TRMT1, TRMT10C, TRMT1L, TRMT5, TRPT1, TSEN34, TSR1, TUT1, UTP15, UTP18, UTP20, UTP25, UTP4, WDR18, WDR3, WDR36, WDR43, XRN2]	Upregulation
positive regulation of translation	GO_BiologicalProcess-EBI-UniProt-GOA-ACAP-ARAP_13.05.2021_00h00	5.72E-57	21.58273	30	[AGO2, C1QBP, CDC123, CDK5RAP1, CIRBP, DDX39B, DHX9, EEF2, EIF4A3, FASTKD2, FXR1, GUF1, LIN28A, MAPK1, MRPS27, NIBAN1, NPM1, NSUN5, PASK, PKM, PLXNB2, POLR2G, PRKCSH, PRKDC, RBM3, RPL5, THBS1, TRMT10C, UPF3B, VIM]	Upregulation
regulation of mRNA catabolic process	GO_BiologicalProcess-EBI-UniProt-GOA-ACAP-ARAP_13.05.2021_00h00	5.72E-57	16.86275	43	[AGO2, AKT1, CARHSP1, CELF1, CIRBP, CNOT11, DDX6, DHX9, EXOSC2, EXOSC5, EXOSC7, FASTKD2, FXR1, GTPBP1, HNRNPM, HSPB1, IGF2BP3, LSM4, MAPK14, MLH1, MYD88, MYEF2, NBAS, NPM1, PARN, PNPT1, POLR2G, PRKCA, PSMC6, PSMD1, PSMD2, PSMD9, PSME3, ROCK2,	Upregulation

					SAMD4B, SERBP1, SET, TBRG4, TRIM71, VIM, XRN1, ZC3HAV1, ZFP36L2]	
regulation of mRNA stability	GO_BiologicalProcess-EBI-UniProt-GOA-ACAP-ARAP_13.05.2021_00h00	5.72E-57	16.19433	40	[AGO2, AKT1, CARHSP1, CELF1, CIRBP, CNOT11, DDX6, DHX9, EXOSC2, EXOSC5, EXOSC7, FASTKD2, FXR1, HNRNPM, HSPB1, IGF2BP3, LSM4, MAPK14, MLH1, MYD88, MYEF2, NPM1, PARN, PNPT1, POLR2G, PRKCA, PSMC6, PSMD1, PSMD2, PSMD9, PSME3, ROCK2, SAMD4B, SERBP1, SET, TBRG4, TRIM71, VIM, XRN1, ZFP36L2]	Upregulation
rRNA metabolic process	GO_BiologicalProcess-EBI-UniProt-GOA-ACAP-ARAP_13.05.2021_00h00	5.72E-57	25.68493	75	[BMS1, BOP1, DCAF13, DDX10, DDX18, DDX21, DDX47, DDX49, DDX52, DDX54, DHX37, EIF4A3, ERCC2, ESF1, EXOSC10, EXOSC2, EXOSC5, EXOSC7, FTSJ3, GEMIN4, HEATR1, IGF2BP3, IMP3, LAS1L, LYAR, MDN1, MPHOSPH10, MTREX, NEDD4, NOL10, NOL11, NOL6, NOL9, NOP14, NOP56, NOP58, NOP9, NPM3, NSUN5, NUDT16, NVL, PDCD11, PELP1, PES1, PRKDC, PWP2, RPF2, RPL13A, RPL22, RPL27A, RPL4, RPL5, RPL7A, RPS2, RPS21, RPS8, RRP12, RRP7A, RRS1, SBDS, TBL3, TEX10, TFB1M, TSR1, UTP15, UTP18, UTP20, UTP25, UTP4, WDR18, WDR3, WDR36, WDR43, XRN1, XRN2]	Upregulation
RNA splicing, via transesterification reactions	GO_BiologicalProcess-EBI-UniProt-GOA-ACAP-ARAP_13.05.2021_00h00	5.72E-57	17.34417	64	[C1QBP, CCAR1, CELF1, CIRBP, CPSF2, CPSF3, CSTF1, CSTF3, CTNNBL1, CWC27, DAZAP1, DDX20, DDX39B, DDX41, DDX42, DDX46, DDX5, DHX15, DHX8, DHX9, EFTUD2, EIF4A3, FXR1, GEMIN4, GEMIN5, HNRNPF, HNRNPL, HNRNPM, KDM1A, LSM4, MBNL1, MPHOSPH10, MTREX, NCL, NPM1, PABPN1, PAXBP1, PCBP2, POLR2A, POLR2B, POLR2G, PRPF38A, PRPF38B, PRPF40A, PRPF8, PSIP1, PSPC1, RBM15, RBM25, RBM3, RBM6, SART3, SF3A3, SF3B1, SNRNP200, SNRPA1, SNRPD3, SRPK1, SRSF1, SRSF11, SRSF7, SYMPK, TIA1, UPF3B]	Upregulation

maturation of SSU-rRNA	GO_BiologicalProcess-EBI-UniProt-GOA-ACAP-ARAP_13.05.2021_00h00	5.72E-57	39.21569	20	[BMS1, DCAF13, DDX52, DHX37, ERCC2, HEATR1, NOL10, NOL11, NOP14, NOP9, PWP2, RPS21, RPS8, RRS1, TBL3, TSR1, UTP20, UTP25, UTP4, WDR3]	Upregulation
maturation of 5.8S rRNA	GO_BiologicalProcess-EBI-UniProt-GOA-ACAP-ARAP_13.05.2021_00h00	5.72E-57	43.24324	16	[BOP1, EXOSC10, EXOSC2, EXOSC7, FTSJ3, LAS1L, MTREX, NEDD4, NOL9, NOP14, NOP9, PES1, PRKDC, RPS21, RRS1, UTP20]	Upregulation
cleavage involved in rRNA processing	GO_BiologicalProcess-EBI-UniProt-GOA-ACAP-ARAP_13.05.2021_00h00	5.72E-57	46.42857	13	[BMS1, BOP1, EXOSC10, EXOSC2, EXOSC7, NOL9, NOP14, NOP9, RPS21, RRS1, TBL3, TSR1, UTP20]	Upregulation
mRNA splicing, via spliceosome	GO_BiologicalProcess-EBI-UniProt-GOA-ACAP-ARAP_13.05.2021_00h00	5.72E-57	17.21312	63	[C1QBP, CCAR1, CELF1, CIRBP, CPSF2, CPSF3, CSTF1, CSTF3, CTNNBL1, CWC27, DAZAP1, DDX20, DDX39B, DDX41, DDX42, DDX46, DDX5, DHX15, DHX8, DHX9, EFTUD2, EIF4A3, FXR1, GEMIN4, GEMIN5, HNRNPF, HNRNPL, HNRNPM, KDM1A, LSM4, MBNL1, MTREX, NCL, NPM1, PABPN1, PAXBP1, PCBP2, POLR2A, POLR2B, POLR2G, PRPF38A, PRPF38B, PRPF40A, PRPF8, PSIP1, PSPC1, RBM15, RBM25, RBM3, RBM6, SART3, SF3A3, SF3B1, SNRNP200, SNRPA1, SNRPD3, SRPK1, SRSF1, SRSF11, SRSF7, SYMPK, TIA1, UPF3B]	Upregulation
maturation of SSU-rRNA from tricistronic rRNA transcript (SSU-rRNA, 5.8S rRNA, LSU-rRNA)	GO_BiologicalProcess-EBI-UniProt-GOA-ACAP-ARAP_13.05.2021_00h00	5.72E-57	44.73684	17	[BMS1, DCAF13, DHX37, ERCC2, HEATR1, NOL10, NOP14, NOP9, PWP2, RPS21, RPS8, RRS1, TBL3, TSR1, UTP20, UTP25, UTP4]	Upregulation
maturation of 5.8S rRNA from tricistronic rRNA transcript (SSU-rRNA, 5.8S rRNA, LSU-rRNA)	GO_BiologicalProcess-EBI-UniProt-GOA-ACAP-ARAP_13.05.2021_00h00	5.72E-57	48	12	[BOP1, EXOSC10, EXOSC2, EXOSC7, FTSJ3, NOL9, NOP14, NOP9, PES1, RPS21, RRS1, UTP20]	Upregulation
endonucleolytic cleavage involved in rRNA processing	GO_BiologicalProcess-EBI-UniProt-	5.72E-57	62.5	10	[BMS1, BOP1, NOL9, NOP14, NOP9, RPS21, RRS1, TBL3, TSR1, UTP20]	Upregulation

	GOA-ACAP-ARAP_13.05.2021_00h00					
extracellular space	GO_CellularComponent-EBI-UniProt-GOA-ACAP-ARAP_13.05.2021_00h00	5.68E-42	11.36237	407	[ABCC1, ABHD14B, ABI1, ACAT1, ACAT2, ACLY, ACO1, ACSL4, ACTBL2, ACTN1, ACTN4, ACTR1B, ACTR2, ADAM10, ADSL, AGO2, AK1, AKR7A2, ALCAM, ALDH1L2, ALDH2, ALDH7A1, ALDOC, ALPL, ANKFY1, ANO6, ANXA1, ANXA11, ANXA2, ANXA3, ANXA4, ANXA5, ANXA6, AP1M1, AP2M1, APAF1, APEH, APOE, APPL2, APRT, ARF4, ARF6, ARHGAP1, ARHGDIA, ARHGEF12, ARL3, ARPC1A, ARSA, ARSB, ASL, ATIC, ATP1A1, ATP2B1, ATP2B2, ATP2B4, ATP6V1A, AUP1, B4GALT1, BAIAP2, BCAT2, BDH2, BLVRB, BPTF, C1QBP, CACYBP, CALR, CANX, CAP1, CAPN1, CAPN2, CAPNS1, CAPZA2, CAT, CBR1, CCT2, CCT3, CCT4, CCT5, CCT6A, CCT7, CCT8, CD2AP, CD44, CD81, CDC42, CDC42BPA, CDC42BPB, CFAP20, CFL2, CHEK1, CHID1, CHST14, CKAP4, CMBL, CMPK1, CNDP2, CNTFR, COBLL1, COL1A1, COL1A2, COL4A1, COL4A2, COL5A1, COL5A2, COL6A1, COL6A2, COL6A3, COPA, COPS2, CPD, CPNE2, CPOX, CPZ, CRTAP, CSE1L, CSPG4, CTNNB1, CTSC, CUL4B, CYB5R1, CYFIP1, DBNL, DCXR, DDX5, DICER1, DIPK2A, DLG1, DPP3, DPYSL2, DPYSL3, DSP, DSTN, ECE1, EEA1, EEF2, EGFL6, EGFR, EHD1, EHD2, EHD4, ENDOD1, ENO2, EPCAM, EPS8, EPS8L2, ERAP1, ERP44, ESD, ESF1, F11R, FAT1, FGG, FH, FKBP4, FKBP5, FLNB, FLT1, FN1, FSCN1, FTH1, GALK1, GALM, GARS1, GART, GATD1, GBE1, GEMIN4, GET3, GIPC1, GIPC2, GLA, GLMN, GLUL, GMPPA, GNB2, GNG12, GNL3, GNS, GOLT1B, GOT2, GPC1, GPC6, GPD1L, GPI, GSN, GSS, GSTK1, GSTM2, GSTM3, HDLBP, HEXA, HLA-A, HMOX1, HNRNPL,	Downregulation

					HNRNPM, HSP90AB1, HSP90B1, HSPA13, HSPA2, HSPA4, HSPA5, HSPA9, HSPB1, HSPD1, HSPE1, HSPH1, HTRA1, HUWE1, IDH2, IMPDH2, IQGAP1, IQGAP2, IRF2BPL, IST1, ITCH, ITGAV, ITGB1, JAM3, KRT18, KRT19, KRT8, LAMB1, LAMC1, LDHB, LGALS1, LMAN1, LRRC57, LSM4, LYPLA2, MACROH2A1, MAN1A1, MAN1A2, MARS1, MBLAC2, MCAM, MEST, MFGE8, MME, MOGS, MON2, MOXD1, MSN, MTAP, MTHFD1, MVB12A, MYDGF, MYH10, MYH9, MYO1C, MYO1E, MYO5A, NCAPG, NCKAP1, NCL, NDRG1, NDRG2, NECTIN2, NEDD4, NIBAN1, NIBAN2, NID1, NIT2, NQO2, NSUN2, NUCB2, P3H1, P4HB, PAICS, PARD3, PARD6B, PCBP2, PCOLCE, PDCD2, PDIA3, PDIA4, PDIA6, PDXK, PFAS, PFKL, PFKP, PHPT1, PI4KA, PIK3C2A, PIP4K2C, PKM, PLD3, PLEC, PLEKHA7, PLOD1, PLOD2, PLOD3, PLXNB2, PNP, PPIB, PPIC, PPP1R7, PRDX1, PRDX4, PRKACB, PRKAR2A, PRKAR2B, PRKCA, PSMC6, PSMD2, PTGIS, PTPRD, PXDN, QDPR, RAB14, RAB1A, RAB2A, RAB5B, RAB5C, RAB8A, RAC3, RAN, RAP1GDS1, RAP2B, RAP2C, RBM15, RDX, RFC1, RFTN1, RHEB, RNH1, RNPEP, RP2, RPL22, RPL4, RPL5, RPS2, RPS8, RRAS, S100A10, S100A11, SARS1, SCAMP2, SCARB2, SCPEP1, SDF4, SEC13, SELENBP1, SEPTIN2, SEPTIN7, SERBP1, SERPINB6, SERPINB9, SERPINH1, SIL1, SLC25A3, SLC2A1, SLC44A1, SLC44A2, SLC9A3R2, SLK, SMARCA4, SMC2, SMPDL3B, SMS, SNX3, SNX9, SORD, SPAG9, SPARC, SPON1, SPR, SPTAN1, SPTBN1, SPTBN2, SRC, SRPRA, SRSF7, SSBP1, SSR4, STXBP2, TAGLN2, TAX1BP1, TCP1, TF, TGM2, THBS1, TIMP3, TKFC, TKT, TLN1, TMED9, TOLLIP, TPM4, TTC38, TUBA4A, TUBB6, TXNDC5, TXNRD1, UBE2G1, UBN2, UGDH,	

					UGGT1, UGP2, UTRN, VAT1, VIM, VLDLR, VPS25, VPS35, VPS36, WARS1, XPNPEP1]	
regulation of localization	GO_BiologicalProcess-EBI-UniProt-GOA-ACAP-ARAP_13.05.2021_00h00	5.68E-42	9.926342	283	[AAAS, ABAT, ACSL1, ACSL4, ACTN1, ACTN4, ADAM10, ADARB1, AGO2, AKT1, ANK2, ANK3, ANKFY1, ANO6, ANXA1, ANXA2, ANXA3, ANXA6, AP2A1, AP2B1, AP2M1, AP2S1, APOE, APPL2, ARAP1, ARF6, ARFGAP1, ARFGAP3, ARHGAP1, ARHGDIA, ARL6IP5, ARSB, ATP1A1, ATP2B1, ATP2B4, AURKB, BAG3, BIN1, C1QBP, CALR, CAMK1, CAMK2D, CAPN1, CASK, CAV1, CAVIN1, CBL, CCAR1, CCT2, CCT3, CCT4, CCT5, CCT6A, CCT7, CCT8, CD2AP, CD81, CDC42, CDH2, CDH3, CDK5RAP3, CFAP20, CFAP298, CLASP1, COL1A1, CORO1C, CTNNB1, CYFIP1, CYP51A1, DAPK1, DDRGK1, DHX9, DIAPH1, DLG1, DOCK7, DPYSL2, DPYSL3, DSP, DUSP3, ECT2, EGFR, EHD1, EHD2, EHD4, ELP3, EMD, EPB41, EPB41L5, EPCAM, ERLEC1, ERO1A, EXOSC10, FERMT2, FGG, FHL1, FLNB, FLT1, FN1, GAB1, GBF1, GCLC, GDI1, GIPC1, GLUD1, GLUL, GNL3, GOPC, GPC1, GPC3, GPC6, GPD1L, GPI, GSN, GSTM2, HADHA, HDAC4, HK2, HMOX1, HSP90AB1, HSPA2, HSPA5, HSPB1, HTT, HUWE1, IDH2, ILK, IPO5, IQGAP1, ITGA6, ITGAV, ITGB1, IWS1, JAM3, KAT7, KIF5B, LAMB1, LMAN1, LRP1, LRRC8A, LSM4, LYAR, MACF1, MACROH2A1, MAGED1, MAP1B, MAP2K6, MAPK1, MAPK14, MAPK8, MAVS, MCAM, MCC, MCU, MEAK7, MEMO1, MESD, MEST, METAP1, MFGE8, MICAL1, MOSPD2, MPC2, MSN, MYADM, MYD88, MYH10, MYO1C, MYO1E, NDC80, NEDD4, NFKB1, NPM1, NRP2, NSF, NSFL1C, NSUN2, NVL, OCLN, OSBP, OXCT1, P3H1, PAK1, PARD3, PARD6B, PARN, PARP1, PASK, PATZ1, PDGFRB, PEA15,	Downregulation

					PFKL, PFKM, PHPT1, PICALM, PIK3C2A, PKP2, PLCG1, PLIN3, PLK1, PLPP3, PLXNB2, POFUT2, PPID, PPM1F, PPP1R9B, PPP2R5A, PREPL, PRKAA1, PRKCA, PRKCSH, PSMD9, PTPN1, PTPN2, PXK, PYCARD, RAB14, RAB23, RAB5B, RAB5C, RAB8A, RAN, RANGAP1, RAP2A, RAP2B, RAP2C, RCC2, RDX, RIC1, ROCK2, RRAS, S100A11, SAR1A, SCFD1, SEC16A, SEC24B, SEPTIN2, SESN2, SESTD1, SH3GL3, SH3GLB1, SHISA8, SIN3A, SIRT1, SLC25A5, SLC2A1, SLC5A3, SLK, SNX17, SNX3, SNX4, SNX6, SORBS1, SPAG9, SPARC, SPTBN1, SRC, STIM1, STXBP2, SUN2, SUPT6H, TBC1D4, TCAF1, TCP1, TF, TGM2, THBS1, TM9SF4, TMED10, TMF1, TPM1, TRIM28, UBR5, USP7, UTRN, VAMP2, VAMP3, VPS35, WAPL, WRAP53, YAP1, YOD1, ZFYVE16, ZMYND8]	
leukocyte activation	GO_BiologicalProcess-EBI-UniProt-GOA-ACAP-ARAP_13.05.2021_00h00	5.68E-42	10.69445	154	[ACAA1, ACLY, ACTR10, ACTR1B, ACTR2, ADAM10, AGL, AKT1, ALDOC, ANO6, ANXA1, ANXA2, ANXA3, AP1M1, AP2A2, AP3B1, APAF1, APEH, APRT, ARSA, ARSB, B4GALT1, CAP1, CAPN1, CASP3, CAT, CAV1, CBL, CCT2, CCT8, CD44, CD81, CDC42, CKAP4, COPB1, CTNNB1, CTPS1, CTSC, CXADR, CYB5R3, CYFIP1, DBNL, DCAF1, DEGS1, DIAPH1, DLG1, DSP, DUSP3, EEF2, ERP44, FAF2, FTH1, GLA, GLMN, GNS, GPI, GSN, HDAC4, HELLS, HLA-A, HMOX1, HMOX2, HOOK3, HSP90AB1, HSPD1, HSPH1, HUWE1, ILF2, IMPDH1, IMPDH2, IQGAP1, IQGAP2, IST1, ITCH, ITGAV, ITGB1, KAT7, KDELR1, LGALS1, LPCAT1, LRRC8A, MANBA, MAPK1, MAPK14, MGST1, MLH1, MME, MOSPD2, MSH2, MSH6, MSN, MYD88, MYDGF, MYH9, MYO1C, NDRG1, NECTIN2, NFKB1, NIT2, ORC3, OSTF1, PAK1, PATZ1, PAWR, PDXK, PFKL, PIK3CA, PKM, PNP, PRDX1, PRDX4,	No change

					PRKAR1A, PRKCSH, PRKDC, PSMD1, PSMD2, PTPN2, PURA, PXDN, PYCARD, RAB14, RAB18, RAB5B, RAB5C, RAP2B, RAP2C, RBPJ, RIF1, RPL22, S100A11, SERPINB6, SHPK, SLC25A5, SLC44A2, SNX4, SNX6, SPTAN1, SRC, SRP14, STXBP2, SUPT6H, SURF4, THBS1, TOLLIP, TXNDC5, UBR4, UNG, VAMP2, VAMP3, VAT1, VPS35L, XRCC5, XRCC6, ZFP36L2]	
cell activation involved in immune response	GO_BiologicalProcess-EBI-UniProt-GOA-ACAP-ARAP_13.05.2021_00h00	5.68E-42	14.44724	115	[ACAA1, ACLY, ACTR10, ACTR1B, ACTR2, ADAM10, AGL, ALDOC, ANO6, ANXA1, ANXA2, ANXA3, AP1M1, AP2A2, APAF1, APEH, APRT, ARSA, ARSB, B4GALT1, CAP1, CAPN1, CAT, CBL, CCT2, CCT8, CD44, CD81, CKAP4, COPB1, CTSC, CYB5R3, CYFIP1, DBNL, DEGS1, DIAPH1, DSP, EEF2, ERP44, FAF2, FTH1, GBF1, GLA, GNS, GPI, GSN, HMOX1, HMOX2, HOOK3, HSP90AB1, HSPD1, HUWE1, ILF2, IMPDH1, IMPDH2, IQGAP1, IQGAP2, IST1, ITGAV, LGALS1, LPCAT1, LRP1, MANBA, MAPK1, MAPK14, MGST1, MLH1, MME, MOSPD2, MSH2, MSH6, MYD88, MYDGF, MYO1C, NFKB1, NIT2, ORC3, OSTF1, PDXK, PFKL, PKM, PNP, PRDX4, PRKCSH, PSMD1, PSMD2, PXDN, PYCARD, RAB14, RAB18, RAB5B, RAB5C, RAP2B, RAP2C, RIF1, S100A11, SERPINB6, SLC44A2, SNX4, SNX6, SPTAN1, SRP14, STXBP2, SUPT6H, SURF4, TOLLIP, TXNDC5, UBR4, UNG, VAMP2, VAMP3, VAT1, VPS35L, XRCC5, XRCC6]	Downregulation
leukocyte mediated immunity	GO_BiologicalProcess-EBI-UniProt-GOA-ACAP-ARAP_13.05.2021_00h00	5.68E-42	11.8593	118	[ACAA1, ACLY, ACTR10, ACTR1B, ACTR2, ADAM10, AGL, ALDOC, ANO6, ANXA2, ANXA3, AP1M1, AP2A2, APAF1, APEH, APRT, ARSA, ARSB, B4GALT1, C1QBP, CAP1, CAPN1, CAT, CBL, CCT2, CCT8, CD44, CD81, CKAP4, COPB1, CTSC, CYB5R3, CYFIP1, DBNL, DDX21, DEGS1, DIAPH1, DSP, EEF2, ERP44, FAF2, FTH1, GLA, GNS, GPI, GSN, HLA-A, HMOX1, HMOX2, HOOK3, HSP90AB1, HSPD1,	Downregulation

					HUWE1, ILF2, IMPDH1, IMPDH2, IQGAP1, IQGAP2, IST1, ITGAV, KIF5B, LPCAT1, MANBA, MAPK1, MAPK14, MAVS, MGST1, MLH1, MME, MOSPD2, MSH2, MSH6, MYD88, NECTIN2, NFKB1, NIT2, ORC3, OSTF1, PDXK, PFKL, PKM, PNP, PRDX1, PRDX4, PRKCSH, PSMD1, PSMD2, PXDN, PYCARD, RAB14, RAB18, RAB5B, RAB5C, RAP2B, RAP2C, RFTN1, RIF1, S100A11, SERPINB6, SERPINB9, SLC44A2, SNX4, SNX6, SPTAN1, SRP14, STXBP2, SUPT6H, SURF4, TOLLIP, TXNDC5, UBR4, UNG, VAMP2, VAMP3, VAT1, VPS35L, XRCC5, XRCC6]	
transport	GO_BiologicalProcess-EBI-UniProt-GOA-ACAP-ARAP_13.05.2021_00h00	5.68E-42	10.28263	553	[AAAS, ABAT, ABCB10, ABCB7, ABCC1, ABCF1, ABI1, ACAA1, ACACA, ACBD3, ACLY, ACO1, ACSL1, ACSL4, ACTN1, ACTN4, ACTR10, ACTR1B, ACTR2, ADAM10, ADD2, ADD3, AGL, AGTPBP1, AKT1, ALDOC, ANK2, ANK3, ANKFY1, ANKRD28, ANO6, ANTXR1, ANXA1, ANXA11, ANXA2, ANXA3, ANXA5, ANXA6, AP1M1, AP1S1, AP2A1, AP2A2, AP2B1, AP2M1, AP2S1, AP3B1, APAF1, APEH, APOE, APOOL, APPL2, APRT, ARAP3, ARCN1, ARF4, ARF6, ARFGAP1, ARFGAP3, ARFGEF1, ARHGAP1, ARL1, ARL3, ARL6IP5, ARPC1A, ARSA, ARSB, ASPH, ATL2, ATL3, ATP1A1, ATP2B1, ATP2B2, ATP2B4, ATP6V1A, AUP1, B4GALT1, BAG3, BAIAP2, BET1, BIN1, CALR, CAMK1, CAMK2D, CANX, CAP1, CAPN1, CAPZA2, CASK, CAT, CAV1, CAVIN1, CBL, CCDC22, CCT2, CCT3, CCT4, CCT5, CCT7, CCT8, CD2AP, CD44, CD81, CDC42, CDH2, CDH3, CHID1, CKAP4, CKAP5, CLASP1, COG1, COG6, COG7, COG8, COL1A1, COPA, COPB1, COPB2, COPE, COPG1, COPG2, COPS2, COPS5, COPZ1, CORO1C, CPSF2, CPSF3, CSE1L, CTBP2, CTNNB1, CTSC, CXADR, CYB5R1, CYB5R3, CYFIP1, CYP51A1, DAPK1, DBNL,	Downregulation

					DCTN1, DCTN4, DDX20, DDX39B, DEGS1, DHX15, DHX9, DIAPH1, DLG1, DOCK7, DPYSL2, DSP, DYNC1LI2, ECT2, EEA1, EEF2, EGFR, EHD1, EHD2, EHD4, EIF4A3, EMD, ENDOD1, EPB41, EPN2, ERBIN, ERGIC1, ERGIC2, ERLEC1, ERLIN2, ERO1A, ERP44, ESYT2, F11R, FAF2, FGG, FHL1, FKBP15, FKBP4, FLNB, FN1, FNBP1L, FTH1, G3BP2, GBF1, GCLC, GDAP1, GDI1, GEMIN4, GEMIN5, GET3, GIPC1, GLA, GLS, GLUD1, GLUL, GNB2, GNS, GOLGA2, GOLT1B, GOPC, GOSR2, GOT2, GPC3, GPD1L, GPI, GSN, GSTM2, HACL1, HADHA, HDLBP, HEATR5A, HERC2, HK2, HMOX1, HMOX2, HOOK3, HSP90AB1, HSP90B1, HSPA14, HSPA2, HSPA4, HSPA5, HSPA9, HSPB1, HSPB11, HSPD1, HSPH1, HTT, HUWE1, IDH2, IGF2BP3, ILF2, IMPDH1, IMPDH2, IPO11, IPO4, IPO5, IQGAP1, IQGAP2, IST1, ITGAV, ITGB1, IWS1, KDELR1, KDELR3, KIF11, KIF20A, KIF22, KIF23, KIF2C, KIF5B, KLC1, KRT18, KTN1, LASP1, LMAN1, LPCAT1, LRP1, LRPPRC, LRRC8A, LSG1, LSM4, LYAR, MACF1, MAGED2, MAIP1, MAN1A1, MANBA, MAP1B, MAP1S, MAP2K6, MAPK1, MAPK14, MAPK8, MAVS, MCU, MESD, METAP1, MFGE8, MFSD10, MGST1, MICAL1, MICAL3, MIPEP, MME, MON2, MOSPD2, MPC2, MRC2, MSN, MTMR6, MVB12A, MYD88, MYH10, MYH9, MYO1C, MYO1E, MYO5A, NBAS, NCKAP1, NDC1, NECAP2, NEDD4, NFKB1, NHLRC2, NIBAN2, NIT2, NLGN4X, NMT2, NNT, NOL6, NOP9, NPM1, NSF, NSUN2, NUP107, NUP133, NUP160, NUP210, NUP35, NUP50, OCLN, OPTN, ORC3, OSBP, OSTF1, OXCT1, P3H1, PABPN1, PAK1, PARD3, PASK, PDGFRB, PDIA4, PDLIM7, PDXK, PEA15, PFKL, PFKM, PGRMC2, PHIP, PHPT1, PICALM,	

					PIK3C2A, PIK3CA, PIK3R4, PIP4K2B, PITRM1, PKM, PKP2, PLCG1, PLIN3, PLPP3, PLSCR3, PNP, PNPT1, POFUT2, PPID, PPM1F, PPP1R7, PRAF2, PRDX4, PREPL, PRKAA1, PRKAG1, PRKCA, PRKCSH, PSIP1, PSMC6, PSMD1, PSMD2, PSMD9, PSME3, PTPN1, PURA, PXDN, PXK, PXMP2, PYCARD, RAB14, RAB18, RAB1A, RAB23, RAB2A, RAB32, RAB5B, RAB5C, RAB8A, RABGAP1L, RAN, RANBP2, RANGAP1, RAP2B, RAP2C, RDX, RFTN1, RIC1, RP2, RPL13A, RPL22, RPL27A, RPL4, RPL5, RPL7A, RPS2, RPS21, RPS8, RRBP1, RRS1, S100A10, S100A11, SAR1A, SCAMP2, SCARB2, SCFD1, SCFD2, SCRN1, SCYL1, SDAD1, SDF4, SEC13, SEC16A, SEC22B, SEC23A, SEC23B, SEC24A, SEC24B, SEC24C, SEC24D, SEC31A, SEC61G, SEH1L, SEL1L, SELENBP1, SEPTIN2, SEPTIN8, SERPINB6, SESN2, SESTD1, SH3GL3, SH3GLB1, SHISA8, SIL1, SIRT1, SLC12A4, SLC1A3, SLC25A12, SLC25A24, SLC25A3, SLC25A32, SLC25A5, SLC2A1, SLC2A10, SLC33A1, SLC44A1, SLC44A2, SLC4A7, SLC5A3, SLC7A3, SLC7A6, SNRPD3, SNX17, SNX3, SNX30, SNX4, SNX6, SNX9, SORBS1, SORD, SPAG9, SPARC, SPTAN1, SPTBN1, SPTBN2, SRC, SRP14, SRPRA, SRPRB, SRSF1, SRSF11, SRSF7, SSB, SSR1, STAM2, STIM1, STX12, STX5, STXBP2, STYX, SUN2, SUPT6H, SURF4, SYMPK, TAGLN2, TBC1D17, TBC1D22B, TBC1D4, TCAF1, TCP1, TEX2, TF, TFG, TGM2, THBS1, TIMM50, TIMP3, TJP2, TLK1, TLN1, TM9SF4, TMED10, TMED2, TMED5, TMED7, TMED9, TMEM115, TMEM167A, TMF1, TMX3, TNPO3, TOLLIP, TOMM34, TRAM1, TRIM28, TUBA4A, TXNDC5, UBE2O, UBR4, UBR5, UPF2, UPF3B,	

					USE1, USP7, UTRN, VAMP2, VAMP3, VAT1, VLDLR, VPS25, VPS26A, VPS26B, VPS26C, VPS35, VPS35L, VPS36, VPS53, XPO5, XPO7, XRCC5, XRCC6, YIF1A, YKT6, YOD1, ZDHHC17, ZFYVE16]	
protein localization	GO_BiologicalProcess-EBI-UniProt-GOA-ACAP-ARAP_13.05.2021_00h00	5.68E-42	14.03061	385	[AAAS, ABAT, ACAA1, ACOX3, ACSL4, ACTN4, ADAM10, AKT1, ANK2, ANK3, ANTXR1, ANXA1, ANXA2, AP1M1, AP1S1, AP2A1, AP2A2, AP2B1, AP2M1, AP2S1, AP3B1, APOE, APPL2, ARCN1, ARF4, ARF6, ARFGAP1, ARFGAP3, ARFGEF1, ARHGAP1, ARHGDIA, ARL1, ARL2, ARL3, ARL6IP5, ATP2B4, ATP6V1A, ATR, AUP1, AURKA, AURKB, BAG3, BAIAP2, BET1, BUB1B, CALR, CAMK1, CANX, CAT, CAV1, CAVIN1, CCDC22, CCT2, CCT3, CCT4, CCT5, CCT6A, CCT7, CCT8, CD2AP, CD81, CDC42, CDH2, CDK5RAP3, COG1, COG6, COG7, COG8, COL1A1, COPA, COPB1, COPB2, COPE, COPG1, COPG2, COPZ1, CORO1C, CSE1L, CTCF, CTNNB1, CYP51A1, DDRGK1, DDX42, DHX9, DIAPH1, DLG1, DSP, ECT2, EGFR, EHD1, EHD2, EHD4, EMD, EPB41, EPB41L3, EPB41L5, ERBIN, ERLEC1, F11R, FAF2, FBN2, FERMT2, FGG, G3BP2, GBF1, GDAP1, GDI1, GET3, GET4, GIPC1, GLUD1, GLUL, GNL3, GOLGA2, GOLT1B, GOPC, GOSR2, GPC1, GPC3, GPC6, GPD1L, GPHN, GSN, GSTK1, HACL1, HADHA, HEATR5A, HERC2, HK1, HK2, HMGCL, HMOX1, HOOK3, HSD17B4, HSP90AB1, HSP90B1, HSPA4, HSPA5, HSPA9, HSPB1, HSPB11, HSPD1, HUWE1, IDH2, ILF3, IPO11, IPO4, IPO5, IQGAP1, IST1, ITGB1, JAM3, KAT7, KDELR1, KDELR3, KEAP1, KIF20A, KIF5B, KRT18, KTN1, LIMS1, LMAN1, LRBA, LRP1, LRRC8A, LRWD1, LSG1, LTBP1, MACF1, MACROH2A1, MAGED1, MAIP1, MAN1A1, MAPK1, MAPK14, MAPK8, MARCHF5, MAVS, MCC, MCU, MEAK7, MESD,	Downregulation

					METAP1, MIPEP, MMAB, MON2, MPC2, MSH2, MSN, MVB12A, MYADM, MYH10, MYH9, MYO1C, MYO1E, MYO5A, NBAS, NDC1, NDC80, NECAP2, NEDD4, NPM1, NSF, NSFL1C, NUP107, NUP133, NUP160, NUP210, NUP35, NUP50, NVL, OCLN, OPTN, OSBP, OXCT1, P3H1, PACS1, PAK1, PARD3, PARP1, PDIA4, PFKL, PFKM, PHIP, PHPT1, PICALM, PIK3R4, PITRM1, PKP2, PLK1, PLOD3, POLR1B, PPID, PPM1F, PPP1R9B, PPP2R5A, PRAF2, PREPL, PRKAA1, PRKCA, PSMD9, PTPN1, PXMP2, RAB14, RAB18, RAB1A, RAB23, RAB2A, RAB32, RAB5B, RAB5C, RAB8A, RABGAP1L, RAN, RANBP2, RANGAP1, RAP2A, RCC2, RDX, RFTN1, RIC1, ROCK2, RP2, RPA1, RPA2, RPF2, RPL13A, RPL22, RPL27A, RPL4, RPL5, RPL7A, RPS2, RPS21, RPS8, RRBP1, RRS1, S100A10, SAR1A, SCAMP2, SCARB2, SCFD1, SCFD2, SCYL1, SDAD1, SEC13, SEC16A, SEC22B, SEC23A, SEC23B, SEC24A, SEC24B, SEC24C, SEC24D, SEC31A, SEC61G, SEH1L, SEL1L, SELENBP1, SEPTIN10, SEPTIN11, SEPTIN2, SEPTIN7, SEPTIN8, SESN2, SH3GLB1, SIL1, SIN3A, SLC2A1, SLC5A3, SLC9A3R2, SNX17, SNX3, SNX30, SNX4, SNX6, SNX9, SORBS1, SPTBN1, SRC, SRP14, SRPRA, SRPRB, SSB, SSR1, STAM2, STX12, STX5, STXBP2, STYX, SUN2, SURF4, TBC1D17, TBC1D22B, TBC1D4, TCAF1, TCP1, TF, TIA1, TIMM50, TJP2, TLK1, TM9SF4, TMED10, TMED2, TMED5, TMED7, TMED9, TMEM115, TMEM167A, TNPO3, TOLLIP, TOMM34, TRAM1, TRIM28, TTK, TWF1, UBR5, USE1, USP9X, UTP25, VAMP2, VAMP3, VLDLR, VPS25, VPS26A, VPS26B, VPS26C, VPS35, VPS35L, VPS36, VPS53, WAPL, WRAP53, XPO5, XPO7, XRCC5, YAP1,	

					YIF1A, YKT6, YOD1, ZDHHC17, ZFYVE16, ZMYND8, ZWILCH]	
cellular response to stress	GO_BiologicalProcess-EBI-UniProt-GOA-ACAP-ARAP_13.05.2021_00h00	5.68E-42	13.129	287	[AAAS, AATF, ABCB10, ACTR2, AK4, AKT1, ANKZF1, ANTXR1, ANXA1, ARFGAP1, ARFGAP3, ARL6IP5, ASNS, ATP6V1A, ATR, AUP1, AURKA, BAG1, BAG2, BAG3, BAZ1B, BCCIP, BRAT1, BRD4, CALR, CAMK2D, CANX, CASP3, CASTOR2, CAT, CAV1, CBL, CBX5, CCAR2, CCNB1, CCNH, CD44, CDC42, CDK5RAP3, CDK7, CHAF1A, CHEK1, CHEK2, CNOT11, CNRIP1, COPS2, COPS5, COPS7A, CTNNB1, CUL4A, CUL4B, CUL7, DAPK1, DAXX, DBNL, DCTN1, DDRGK1, DDX39B, DDX5, DHX9, DLG1, DNMT3A, DNMT3B, DUSP3, ECPAS, ECT2, EDEM3, EEF1E1, EEF2, EGFR, EMSY, ERCC2, ERLEC1, ERLIN2, ERO1A, ERP44, EXD2, FAF2, FANCD2, FANCI, FBXO2, FH, FKBP14, FKBP4, FSCN1, FXR1, GATA6, GET4, GLUL, GOSR2, GPX7, GPX8, GSTM2, GSTM3, GTF2I, HDAC2, HERC2, HK2, HMOX1, HNRNPM, HP1BP3, HSP90AB1, HSP90B1, HSPA13, HSPA14, HSPA2, HSPA5, HSPA9, HSPB1, HSPD1, HSPH1, HUWE1, INO80C, IRF3, ITCH, JAM3, KAT7, KDELR3, KDM1A, KDM2A, KEAP1, KIF22, LAMC1, LARS1, LIG1, LIG3, LRP1, MAN1A1, MAP1B, MAP1LC3A, MAP2K6, MAPK1, MAPK14, MAPK8, MARS1, MASTL, MCM2, MCM3, MCM4, MCM5, MCM6, MCM7, MDC1, MEAK7, MGST1, MLH1, MMAB, MRPS9, MSH2, MSH3, MSH6, MTR, MTREX, MYBBP1A, MYD88, MYDGF, MYEF2, NDC1, NDRG1, NEDD4, NFKB1, NIBAN1, NPM1, NTHL1, NUP107, NUP133, NUP160, NUP210, NUP35, NUP50, OPTN, P4HB, PAK1, PARP1, PAWR, PDCL3, PDIA3, PDIA4, PDIA6, PDS5A, PIAS4, PIK3CA, PIK3R4, PLK1, PNPT1, POLA1, POLB, POLD1, POLE, POLR1B, POLR2A,	No change

					POLR2B, POLR2G, PRDX1, PRKAA1, PRKAG1, PRKCA, PRKDC, PRPF38B, PSMC6, PSMD1, PSMD2, PSMD9, PSME3, PTGIS, PTPN1, PTPN2, PXDN, PYCARD, PYCR1, PYCR2, RAD51, RANBP2, RAP2A, RBPJ, RELA, RFC1, RFC2, RFC3, RFC5, RHEB, RIF1, ROCK2, RPA1, RPA2, RPA3, RPS6KA1, RPS6KA3, SAMHD1, SEC13, SEC16A, SEC31A, SEH1L, SEL1L, SERPINB6, SESN2, SH3GLB1, SIN3A, SIRT1, SIRT2, SLC25A24, SLC2A1, SMARCA5, SMARCAD1, SORD, SPAG9, SRC, SRPRA, SRPRB, SSR1, SSRP1, STT3B, SUPT16H, TBL2, TDP1, TDP2, TF, THBS1, TIGAR, TIPRL, TLK1, TLN1, TMED2, TOP2A, TPM1, TRAP1, TRIM28, TRIP12, TRIP13, TXNRD1, UBA6, UBR5, UFL1, UGGT1, UGGT2, UNG, USP19, USP47, USP7, WDHD1, WRAP53, XRCC5, XRCC6, YAP1, YIF1A, YOD1, ZFP36L2]	
establishment of localization in cell	GO_BiologicalProcess-EBI-UniProt-GOA-ACAP-ARAP_13.05.2021_00h00	5.68E-42	13.71468	398	[AAAS, ACAA1, ACACA, ACLY, ACTN1, ACTN4, ACTR10, ACTR1B, ACTR2, ADAM10, AGL, AGTPBP1, AKT1, ALDOC, ANK2, ANK3, ANKFY1, ANKRD28, ANO6, ANXA2, ANXA3, ANXA5, AP1M1, AP1S1, AP2A1, AP2A2, AP2B1, AP2M1, AP2S1, AP3B1, APAF1, APEH, APOE, APOOL, APPL2, APRT, ARCN1, ARF4, ARF6, ARFGAP1, ARFGAP3, ARHGAP1, ARL1, ARL3, ARSA, ARSB, ATL2, ATL3, ATP1A1, ATP2B4, AUP1, AURKB, B4GALT1, BAG3, BET1, BIN1, CALR, CAMK1, CAMK2D, CANX, CAP1, CAPN1, CAPZA2, CASK, CAT, CAV1, CBL, CCDC22, CCNB1, CCT2, CCT8, CD44, CD81, CDC42, CDCA8, CDH2, CDH3, CHID1, CKAP4, CLASP1, COG1, COG6, COG7, COG8, COPA, COPB1, COPB2, COPE, COPG1, COPG2, COPZ1, CORO1C, CPSF2, CPSF3, CSE1L, CTBP2, CTNNB1, CTSC, CYB5R1, CYB5R3, CYFIP1, DBNL, DCTN1, DCTN4, DDX20, DDX39B, DEGS1, DHX9,	Downregulation

					DIAPH1, DLG1, DLGAP5, DOCK7, DPYSL2, DSP, DYNC1LI2, ECT2, EEA1, EEF2, EHD1, EHD2, EHD4, EIF4A3, EMD, ENDOD1, ERBIN, ERGIC1, ERGIC2, ERLEC1, ERO1A, ERP44, FAF2, FGG, FN1, FNBP1L, FTH1, GBF1, GDAP1, GDI1, GEMIN4, GEMIN5, GET3, GIPC1, GLA, GNS, GOLGA2, GOPC, GOSR2, GPI, GSN, GSTM2, HACL1, HEATR5A, HERC2, HMOX1, HMOX2, HOOK3, HSP90AB1, HSP90B1, HSPA4, HSPA5, HSPA9, HSPB1, HSPB11, HSPD1, HTT, HUWE1, ILF2, IMPDH1, IMPDH2, INCENP, IPO11, IPO4, IPO5, IQGAP1, IQGAP2, IST1, ITGAV, ITGB1, IWS1, KDELR1, KDELR3, KIF22, KIF2C, KIF5B, LMAN1, LPCAT1, LRP1, LRPPRC, LSG1, MAGED2, MAIP1, MAN1A1, MANBA, MAP1B, MAP1S, MAPK1, MAPK14, MAPK8, MAVS, METAP1, MGST1, MIPEP, MLH1, MME, MON2, MOSPD2, MPC2, MSN, MVB12A, MYH9, MYO1C, MYO1E, MYO5A, NDC1, NDC80, NEDD4, NFKB1, NHLRC2, NIT2, NLGN4X, NOL6, NOP9, NPM1, NSF, NSFL1C, NSUN2, NUP107, NUP133, NUP160, NUP210, NUP35, NUP50, NUSAP1, ORC3, OSBP, OSTF1, OXCT1, PABPN1, PAK1, PARD3, PDXK, PFKL, PHIP, PHPT1, PICALM, PIK3R4, PITRM1, PKM, PLCG1, PLK1, PNP, PRDX4, PREPL, PRKAA1, PRKAG1, PRKCA, PRKCSH, PSIP1, PSMD1, PSMD2, PTPN1, PURA, PXDN, PXMP2, PYCARD, RAB14, RAB18, RAB1A, RAB23, RAB2A, RAB32, RAB5B, RAB5C, RAB8A, RABGAP1L, RAN, RANBP2, RANGAP1, RAP2B, RAP2C, RDX, RFTN1, RIC1, RPL13A, RPL22, RPL27A, RPL4, RPL5, RPL7A, RPS2, RPS21, RPS8, RRS1, S100A11, SAR1A, SCARB2, SCFD1, SCFD2, SDAD1, SEC13, SEC16A, SEC22B, SEC23A, SEC23B, SEC24A, SEC24B, SEC24C,	

					SEC24D, SEC31A, SEC61G, SEH1L, SEL1L, SERPINB6, SH3GLB1, SIL1, SLC44A2, SNRPD3, SNX17, SNX3, SNX4, SNX6, SNX9, SPARC, SPTAN1, SPTBN1, SPTBN2, SRC, SRP14, SRPRA, SRPRB, SRSF1, SRSF11, SRSF7, SSB, SSR1, STAM2, STX12, STX5, STXBP2, STYX, SUN2, SUPT6H, SURF4, SYMPK, TAGLN2, TBC1D17, TBC1D22B, TBC1D4, TCAF1, TF, TFG, THBS1, TIMM50, TIMP3, TLK1, TLN1, TM9SF4, TMED10, TMED2, TMED5, TMED7, TMED9, TMEM115, TMEM167A, TMX3, TNPO3, TOLLIP, TOMM34, TRAM1, TRIM28, TUBA4A, TXNDC5, UBE2O, UBR4, UBR5, UPF2, UPF3B, USE1, USP7, VAMP2, VAMP3, VAT1, VPS25, VPS26A, VPS26B, VPS26C, VPS35, VPS35L, VPS36, VPS53, XPO5, XPO7, XRCC5, XRCC6, YIF1A, YKT6, YOD1, ZFYVE16]	
export from cell	GO_BiologicalProcess-EBI-UniProt-GOA-ACAP-ARAP_13.05.2021_00h00	5.68E-42	12.61745	188	[ABAT, ABCC1, ACAA1, ACLY, ACSL4, ACTN1, ACTN4, ACTR10, ACTR1B, ACTR2, ADAM10, AGL, ALDOC, ANO6, ANXA1, ANXA2, ANXA3, ANXA5, AP1M1, AP2A2, APAF1, APEH, APOE, APOOL, APRT, ARF6, ARFGAP3, ARFGEF1, ARSA, ARSB, ATP1A1, ATP2B1, B4GALT1, CANX, CAP1, CAPN1, CASK, CAT, CAVIN1, CBL, CCT2, CCT8, CD2AP, CD44, CHID1, CKAP4, CLASP1, COPB1, COPG1, COPG2, COPS5, CTBP2, CTSC, CYB5R1, CYB5R3, CYFIP1, CYP51A1, DBNL, DEGS1, DIAPH1, DLG1, DPYSL2, DSP, EEF2, EGFR, ENDOD1, ERP44, FAF2, FGG, FLNB, FN1, FTH1, GIPC1, GLA, GLUD1, GLUL, GNS, GPI, GSN, HADHA, HMOX1, HMOX2, HOOK3, HSP90AB1, HUWE1, IDH2, ILF2, IMPDH1, IMPDH2, IQGAP1, IQGAP2, IST1, ITGAV, KIF5B, LPCAT1, LRP1, LRRC8A, LSM4, MAGED2, MANBA, MAP2K6, MAPK1, MAPK14, MCU, MGST1, MICAL1, MICAL3, MME,	Downregulation

					MOSPD2, MPC2, MYH10, MYH9, NFKB1, NHLRC2, NIBAN2, NIT2, NSF, ORC3, OSBP, OSTF1, OXCT1, P3H1, PAK1, PASK, PDIA4, PDXK, PFKL, PFKM, PHPT1, PIK3C2A, PKM, PNP, PPID, PRDX4, PREPL, PRKCA, PRKCSH, PSMD1, PSMD2, PSMD9, PXDN, PYCARD, RAB14, RAB18, RAB1A, RAB5B, RAB5C, RAB8A, RAP2B, RAP2C, RIC1, S100A11, SCFD2, SCRN1, SDF4, SEL1L, SEPTIN2, SERPINB6, SLC2A1, SLC44A2, SNX4, SNX6, SPARC, SPTAN1, SRP14, STXBP2, SURF4, TAGLN2, TF, THBS1, TIMP3, TLN1, TMED10, TMEM167A, TMF1, TMX3, TOLLIP, TUBA4A, TXNDC5, UBR4, USE1, VAMP2, VAMP3, VAT1, VPS35L, XRCC5, XRCC6, YKT6]	
myeloid leukocyte activation	GO_BiologicalProcess-EBI-UniProt-GOA-ACAP-ARAP_13.05.2021_00h00	5.68E-42	14.87603	108	[ACAA1, ACLY, ACTR10, ACTR1B, ACTR2, ADAM10, AGL, ALDOC, ANO6, ANXA2, ANXA3, AP1M1, AP2A2, APAF1, APEH, APRT, ARSA, ARSB, B4GALT1, CAP1, CAPN1, CAT, CBL, CCT2, CCT8, CD44, CKAP4, COPB1, CTSC, CYB5R3, CYFIP1, DBNL, DEGS1, DIAPH1, DSP, EEF2, ERP44, FAF2, FTH1, GLA, GNS, GPI, GSN, HMOX1, HMOX2, HOOK3, HSP90AB1, HSPD1, HUWE1, ILF2, IMPDH1, IMPDH2, IQGAP1, IQGAP2, IST1, ITGAV, LPCAT1, MANBA, MAPK1, MAPK14, MGST1, MME, MOSPD2, MYD88, MYO1C, NDRG1, NECTIN2, NFKB1, NIT2, ORC3, OSTF1, PDXK, PFKL, PKM, PNP, PRDX4, PRKCSH, PSMD1, PSMD2, PXDN, PYCARD, RAB14, RAB18, RAB5B, RAB5C, RAP2B, RAP2C, RBPJ, S100A11, SERPINB6, SHPK, SLC44A2, SNX4, SNX6, SPTAN1, SRP14, STXBP2, SURF4, THBS1, TOLLIP, TXNDC5, UBR4, VAMP2, VAMP3, VAT1, VPS35L, XRCC5, XRCC6]	Downregulation
leukocyte activation involved in immune response	GO_BiologicalProcess-EBI-UniProt-GOA-	5.68E-42	14.28571	113	[ACAA1, ACLY, ACTR10, ACTR1B, ACTR2, ADAM10, AGL, ALDOC, ANO6, ANXA1, ANXA2, ANXA3, AP1M1, AP2A2,	Downregulation

	ACAP-ARAP_13.05.2021_00h00				APAF1, APEH, APRT, ARSA, ARSB, B4GALT1, CAP1, CAPN1, CAT, CBL, CCT2, CCT8, CD44, CD81, CKAP4, COPB1, CTSC, CYB5R3, CYFIP1, DBNL, DEGS1, DIAPH1, DSP, EEF2, ERP44, FAF2, FTH1, GLA, GNS, GPI, GSN, HMOX1, HMOX2, HOOK3, HSP90AB1, HSPD1, HUWE1, ILF2, IMPDH1, IMPDH2, IQGAP1, IQGAP2, IST1, ITGAV, LGALS1, LPCAT1, MANBA, MAPK1, MAPK14, MGST1, MLH1, MME, MOSPD2, MSH2, MSH6, MYD88, MYDGF, MYO1C, NFKB1, NIT2, ORC3, OSTF1, PDXK, PFKL, PKM, PNP, PRDX4, PRKCSH, PSMD1, PSMD2, PXDN, PYCARD, RAB14, RAB18, RAB5B, RAB5C, RAP2B, RAP2C, RIF1, S100A11, SERPINB6, SLC44A2, SNX4, SNX6, SPTAN1, SRP14, STXBP2, SUPT6H, SURF4, TOLLIP, TXNDC5, UBR4, UNG, VAMP2, VAMP3, VAT1, VPS35L, XRCC5, XRCC6]	
regulation of protein localization	GO_BiologicalProcess-EBI-UniProt-GOA-ACAP-ARAP_13.05.2021_00h00	5.68E-42	13.94595	129	[ABAT, ACSL4, ADAM10, AKT1, ANK3, ANXA1, AP2B1, AP2M1, APOE, ARF6, ARHGDIA, ATP2B4, BAG3, CAMK1, CCT2, CCT3, CCT4, CCT5, CCT6A, CCT7, CCT8, CD2AP, CD81, CDC42, CDH2, CDK5RAP3, CTNNB1, CYP51A1, DDRGK1, DLG1, ECT2, EGFR, EMD, EPB41, EPB41L5, ERLEC1, FERMT2, FGG, GBF1, GDI1, GLUD1, GLUL, GNL3, GOPC, GPC1, GPC3, GPC6, GPD1L, GSN, HADHA, HSP90AB1, HUWE1, IDH2, IPO5, IQGAP1, ITGB1, KAT7, KIF5B, LMAN1, LRP1, LRRC8A, MACROH2A1, MAGED1, MAPK1, MAPK14, MAPK8, MAVS, MCU, MEAK7, MESD, METAP1, MPC2, MSN, MYH10, MYO1C, MYO1E, NDC80, NPM1, NSFL1C, NVL, OSBP, OXCT1, P3H1, PAK1, PARP1, PFKL, PFKM, PHPT1, PICALM, PLK1, PPID, PPM1F, PPP1R9B, PPP2R5A, PRKAA1, PRKCA, PSMD9, PTPN1, RAB14, RAB23, RAB8A, RAN, RANGAP1, RDX, ROCK2, SAR1A, SCFD1, SEC16A, SESN2,	Downregulation

					SH3GLB1, SIN3A, SLC2A1, SLC5A3, SNX3, SORBS1, SPTBN1, SRC, TCAF1, TCP1, TM9SF4, TMED10, TRIM28, UBR5, VAMP2, WAPL, WRAP53, YAP1, YOD1, ZMYND8]	
establishment of protein localization	GO_BiologicalProcess-EBI-UniProt-GOA-ACAP-ARAP_13.05.2021_00h00	5.68E-42	14.56816	280	[AAAS, ABAT, ACSL4, ACTN4, AKT1, ANK2, ANK3, ANXA1, AP1M1, AP1S1, AP2A1, AP2A2, AP2B1, AP2M1, AP2S1, AP3B1, APOE, APPL2, ARCN1, ARF4, ARF6, ARFGAP1, ARFGAP3, ARFGEF1, ARHGAP1, ARL1, ARL3, ATP6V1A, AUP1, BAG3, BET1, CALR, CAMK1, CANX, CAV1, CAVIN1, CCDC22, CCT2, CCT3, CCT4, CCT5, CCT6A, CCT7, CCT8, CD2AP, CD81, CDC42, COG1, COG6, COG7, COG8, COL1A1, COPA, COPB1, COPB2, COPE, COPG1, COPG2, COPZ1, CORO1C, CSE1L, CYP51A1, ECT2, EGFR, EHD1, EMD, EPB41L5, ERBIN, ERLEC1, FAF2, FGG, GBF1, GDAP1, GDI1, GET3, GET4, GIPC1, GLUD1, GLUL, GOLGA2, GOLT1B, GOPC, GOSR2, HACL1, HADHA, HERC2, HK1, HK2, HMOX1, HOOK3, HSP90AB1, HSP90B1, HSPA4, HSPA5, HSPA9, HSPB1, HSPB11, HSPD1, HUWE1, IDH2, IPO11, IPO4, IPO5, IST1, KDELR1, KDELR3, KIF20A, KIF5B, KRT18, KTN1, LIMS1, LMAN1, LRP1, LRRC8A, LRWD1, LSG1, MACF1, MACROH2A1, MAIP1, MAN1A1, MAPK1, MAPK14, MAPK8, MAVS, MCC, MCU, METAP1, MIPEP, MON2, MPC2, MVB12A, MYH10, MYH9, MYO1C, MYO1E, MYO5A, NBAS, NDC1, NECAP2, NEDD4, NPM1, NSF, NUP107, NUP133, NUP160, NUP210, NUP35, NUP50, OPTN, OSBP, OXCT1, P3H1, PAK1, PARD3, PDIA4, PFKL, PFKM, PHIP, PHPT1, PIK3R4, PITRM1, PPID, PPM1F, PRAF2, PREPL, PRKAA1, PRKCA, PSMD9, PTPN1, PXMP2, RAB14, RAB18, RAB1A, RAB23, RAB2A, RAB32, RAB5B, RAB5C, RAB8A, RABGAP1L, RAN, RANBP2, RANGAP1,	Downregulation

					RCC2, RDX, RFTN1, RIC1, RP2, RPL13A, RPL22, RPL27A, RPL4, RPL5, RPL7A, RPS2, RPS21, RPS8, RRBP1, SAR1A, SCAMP2, SCARB2, SCFD1, SCFD2, SDAD1, SEC13, SEC16A, SEC22B, SEC23A, SEC23B, SEC24A, SEC24B, SEC24C, SEC24D, SEC31A, SEC61G, SEH1L, SEL1L, SELENBP1, SH3GLB1, SIL1, SLC2A1, SNX17, SNX3, SNX30, SNX4, SNX6, SNX9, SPTBN1, SRC, SRP14, SRPRA, SRPRB, SSR1, STAM2, STX12, STX5, STXBP2, STYX, SURF4, TBC1D17, TBC1D22B, TBC1D4, TCAF1, TCP1, TF, TIMM50, TLK1, TM9SF4, TMED10, TMED2, TMED5, TMED7, TMED9, TMEM115, TMEM167A, TNPO3, TOMM34, TRAM1, TRIM28, UBR5, USE1, VAMP2, VAMP3, VLDLR, VPS25, VPS26A, VPS26B, VPS26C, VPS35, VPS35L, VPS36, VPS53, WRAP53, XPO5, XPO7, YIF1A, YKT6, YOD1, ZDHHC17, ZFYVE16]	
intracellular transport	GO_BiologicalProcess-EBI-UniProt-GOA-ACAP-ARAP_13.05.2021_00h00	5.68E-42	15.13121	271	[AAAS, ACACA, ACTN4, ACTR10, ACTR2, AGTPBP1, AKT1, ANK2, ANK3, ANKFY1, ANKRD28, ANXA2, AP1M1, AP1S1, AP2A1, AP2A2, AP2B1, AP2M1, AP2S1, AP3B1, APOE, APPL2, ARCN1, ARF4, ARF6, ARFGAP1, ARFGAP3, ARHGAP1, ARL1, ARL3, ATL2, ATL3, AUP1, BAG3, BET1, BIN1, CALR, CAMK1, CAPZA2, CCDC22, CD81, CDC42, COG1, COG6, COG7, COG8, COPA, COPB1, COPB2, COPE, COPG1, COPG2, COPZ1, CORO1C, CPSF2, CPSF3, CSE1L, CTSC, DCTN1, DCTN4, DDX20, DDX39B, DHX9, DOCK7, DYNC1LI2, ECT2, EEA1, EHD1, EHD2, EHD4, EIF4A3, EMD, ERBIN, ERGIC1, ERGIC2, ERLEC1, FAF2, FNBP1L, GBF1, GDAP1, GDI1, GEMIN4, GEMIN5, GET3, GIPC1, GOLGA2, GOPC, GOSR2, HACL1, HEATR5A, HERC2, HOOK3, HSP90AB1, HSP90B1, HSPA4, HSPA5, HSPA9, HSPB1, HSPB11, HSPD1, HTT,	Downregulation

					HUWE1, IPO11, IPO4, IPO5, IWS1, KDELR1, KDELR3, KIF5B, LMAN1, LRPPRC, LSG1, MAIP1, MAN1A1, MAP1B, MAP1S, MAPK1, MAPK14, MAPK8, MAVS, METAP1, MIPEP, MON2, MSN, MVB12A, MYO1C, MYO1E, MYO5A, NDC1, NEDD4, NOL6, NOP9, NPM1, NSF, NSUN2, NUP107, NUP133, NUP160, NUP210, NUP35, NUP50, OSBP, PABPN1, PAK1, PARD3, PHIP, PICALM, PIK3R4, PITRM1, PREPL, PRKAA1, PRKAG1, PRKCA, PSIP1, PTPN1, PURA, PXMP2, RAB14, RAB18, RAB1A, RAB23, RAB2A, RAB32, RAB5B, RAB5C, RAB8A, RABGAP1L, RAN, RANBP2, RANGAP1, RDX, RFTN1, RIC1, RPL13A, RPL22, RPL27A, RPL4, RPL5, RPL7A, RPS2, RPS21, RPS8, RRS1, SAR1A, SCARB2, SCFD1, SCFD2, SDAD1, SEC13, SEC16A, SEC22B, SEC23A, SEC23B, SEC24A, SEC24B, SEC24C, SEC24D, SEC31A, SEC61G, SEH1L, SEL1L, SH3GLB1, SIL1, SNRPD3, SNX17, SNX3, SNX4, SNX6, SNX9, SPTAN1, SPTBN1, SPTBN2, SRC, SRP14, SRPRA, SRPRB, SRSF1, SRSF11, SRSF7, SSB, SSR1, STAM2, STX12, STX5, STXBP2, STYX, SUN2, SUPT6H, SURF4, SYMPK, TBC1D17, TBC1D22B, TBC1D4, TCAF1, TFG, TIMM50, TLK1, TM9SF4, TMED10, TMED2, TMED5, TMED7, TMED9, TMEM115, TMEM167A, TNPO3, TOMM34, TRAM1, TRIM28, UBE2O, UBR5, UPF2, UPF3B, USE1, USP7, VAMP2, VAMP3, VPS25, VPS26A, VPS26B, VPS26C, VPS35, VPS35L, VPS36, VPS53, XPO5, XPO7, YIF1A, YKT6, YOD1, ZFYVE16]	
regulation of cellular localization	GO_BiologicalProcess-EBI-UniProt-GOA-ACAP-ARAP_13.0	5.68E-42	15.09648	133	[AAAS, ADAM10, AKT1, ANK2, ANK3, ANXA2, AP2B1, AP2M1, ARF6, ARHGAP1, ATP2B4, AURKB, BAG3, CAMK1, CAMK2D, CAV1, CCT2, CCT3, CCT4, CCT5, CCT6A, CCT7, CCT8,	Downregulation

	5.2021_00h00				CD2AP, CD81, CDC42, CDH2, CDH3, CDK5RAP3, CTNNB1, DDRGK1, DHX9, DIAPH1, DLG1, ECT2, EGFR, EHD1, EHD2, EMD, EPB41, ERLEC1, FERMT2, GBF1, GDI1, GLUL, GNL3, GOPC, GPC1, GPC3, GPC6, GPD1L, GSN, GSTM2, HMOX1, HSP90AB1, HTT, HUWE1, IPO5, IQGAP1, ITGB1, IWS1, KAT7, KIF5B, LMAN1, LRP1, MACROH2A1, MAGED1, MAP1B, MAPK1, MAPK14, MAPK8, MAVS, MEAK7, MESD, METAP1, MPC2, MSN, MYO1C, MYO1E, NDC80, NEDD4, NPM1, NSFL1C, NSUN2, NVL, OSBP, OXCT1, PAK1, PARD3, PARD6B, PARP1, PHPT1, PICALM, PLCG1, PLK1, PPM1F, PPP1R9B, PPP2R5A, PRKAA1, PRKCA, PTPN1, RAB23, RAN, RANGAP1, RDX, ROCK2, SAR1A, SCFD1, SEC16A, SEC24B, SESN2, SH3GLB1, SIN3A, SLC5A3, SNX3, SNX4, SORBS1, SPTBN1, SRC, STXBP2, SUPT6H, TCAF1, TCP1, TM9SF4, TRIM28, UBR5, USP7, VAMP2, WAPL, WRAP53, YAP1, YOD1, ZMYND8]	
myeloid leukocyte mediated immunity	GO_BiologicalProcess-EBI-UniProt-GOA-ACAP-ARAP_13.05.2021_00h00	5.68E-42	16.72026	104	[ACAA1, ACLY, ACTR10, ACTR1B, ACTR2, ADAM10, AGL, ALDOC, ANO6, ANXA2, ANXA3, AP1M1, AP2A2, APAF1, APEH, APRT, ARSA, ARSB, B4GALT1, CAP1, CAPN1, CAT, CBL, CCT2, CCT8, CD44, CKAP4, COPB1, CTSC, CYB5R3, CYFIP1, DBNL, DDX21, DEGS1, DIAPH1, DSP, EEF2, ERP44, FAF2, FTH1, GLA, GNS, GPI, GSN, HMOX1, HMOX2, HOOK3, HSP90AB1, HUWE1, ILF2, IMPDH1, IMPDH2, IQGAP1, IQGAP2, IST1, ITGAV, LPCAT1, MANBA, MAPK1, MAPK14, MAVS, MGST1, MME, MOSPD2, MYD88, NFKB1, NIT2, ORC3, OSTF1, PDXK, PFKL, PKM, PNP, PRDX4, PRKCSH, PSMD1, PSMD2, PXDN, PYCARD, RAB14, RAB18, RAB5B, RAB5C, RAP2B, RAP2C, S100A11, SERPINB6, SERPINB9, SLC44A2, SNX4, SNX6, SPTAN1,	Downregulation

					SRP14, STXBP2, SURF4, TOLLIP, TXNDC5, UBR4, VAMP2, VAMP3, VAT1, VPS35L, XRCC5, XRCC6]	
replication fork	GO_CellularComponent-EBI-UniProt-GOA-ACAP-ARAP_13.05.2021_00h00	5.68E-42	27.77778	20	[BAZ1B, CHEK1, DNMT1, MCM3, POLA1, POLA2, POLD1, POLR1B, PRIM1, PRIM2, RFC1, RFC2, RFC3, RFC5, RPA1, RPA2, RPA3, SMARCA5, SMARCAD1, WDHD1]	Upregulation
vacuole	GO_CellularComponent-EBI-UniProt-GOA-ACAP-ARAP_13.05.2021_00h00	5.68E-42	12.42741	107	[ACLY, ACTR10, ACTR2, ANK2, ANK3, ANKFY1, ANXA1, ANXA11, ANXA2, ANXA6, AP1M1, AP1S1, AP2A1, AP2A2, AP2B1, AP2M1, AP2S1, AP3B1, ARSA, ARSB, ATP6V1A, AUP1, B4GALT1, CALCOCO2, CAP1, CAPN1, CAPN2, CAT, CCT2, CCT8, CDC42, CHID1, CKAP4, COL6A1, CSPG4, CTSC, CYB5R3, DAGLB, DYNC1LI2, ECE1, FAF2, GLA, GLB1L3, GNB2, GNS, GOLT1B, GOPC, GPC1, GPC3, GPC6, HEXA, HSP90AB1, HTT, IMPDH1, IST1, LARS1, LNPEP, LPCAT1, LRBA, LRP1, LRRC8A, MANBA, MAP1LC3A, MAPK1, MEAK7, MGST1, MYO5A, NSF, OCLN, OPTN, PDGFRB, PHIP, PIK3R4, PIP4K2B, PIP4K2C, PLD3, PON2, PRKCSH, PSMD1, PYCARD, RAB14, RAB23, RAB2A, RAB5C, RHEB, SCARB2, SEC13, SEH1L, SH3GLB1, SLC12A4, SLC44A2, SNX6, SPAG9, SRC, STXBP2, SURF4, TBC1D17, TMEM192, TMEM97, TOLLIP, TXNDC5, USE1, USP5, VAT1, VLDLR, VPS26A, VPS35]	Downregulation
chaperonin-containing T-complex	GO_CellularComponent-EBI-UniProt-GOA-ACAP-ARAP_13.05.2021_00h00	5.68E-42	72.72727	8	[CCT2, CCT3, CCT4, CCT5, CCT6A, CCT7, CCT8, TCP1]	Upregulation
cellular response to DNA damage stimulus	GO_BiologicalProcess-EBI-UniProt-GOA-ACAP-ARAP_13.05.2021_00h00	5.68E-42	13.92818	128	[AATF, ACTR2, AKT1, ANTXR1, ATR, AURKA, BAZ1B, BCCIP, BRAT1, BRD4, CASP3, CBL, CBX5, CCAR2, CCNB1, CCNH, CD44, CDK5RAP3, CDK7, CHAF1A, CHEK1, CHEK2, CNOT11, CNRIP1, COPS2, COPS5, COPS7A, CUL4A, CUL4B, DDX39B, DDX5, DHX9,	Upregulation

					EEF1E1, EGFR, EMSY, ERCC2, EXD2, FANCD2, FANCI, FH, FSCN1, FXR1, GTF2I, HERC2, HMOX1, HUWE1, INO80C, IRF3, KAT7, KDM1A, KDM2A, KIF22, LIG1, LIG3, MAP2K6, MAPK1, MAPK14, MAPK8, MASTL, MCM2, MCM3, MCM4, MCM5, MCM6, MCM7, MDC1, MLH1, MMAB, MRPS9, MSH2, MSH3, MSH6, MTREX, NDRG1, NEDD4, NPM1, NTHL1, PAK1, PARP1, PDS5A, PIAS4, PLK1, POLA1, POLB, POLD1, POLE, POLR1B, POLR2A, POLR2B, POLR2G, PRKDC, PYCARD, RAD51, RFC1, RFC2, RFC3, RFC5, RIF1, RPA1, RPA2, RPA3, SAMHD1, SESN2, SIRT1, SMARCA5, SMARCAD1, SSRP1, SUPT16H, TDP1, TDP2, TIGAR, TIPRL, TLK1, TOP2A, TRIM28, TRIP12, TRIP13, UBA6, UBR5, UFL1, UNG, USP47, USP7, WDHD1, WRAP53, XRCC5, XRCC6, YAP1]	
nucleotidyltransferase activity	GO_MolecularFunction-EBI-UniProt-GOA-ACAP-ARAP_13.05.2021_00h00	5.68E-42	21.07843	43	[AURKB, CCT2, CCT4, CHEK2, CHTF18, CTNNB1, FLAD1, GMPPA, GMPPB, GPHN, HSP90AB1, MAPK1, NEK7, NVL, PABPN1, PAPSS2, PARN, PCYT1A, PCYT2, PNPT1, POLA1, POLB, POLD1, POLE, POLR1B, POLR2A, POLR2B, POLR2G, POLR3A, POLR3C, POLR3F, PRIM1, RFC2, RFC3, RFC5, SRC, TCP1, TUT1, UAP1, UGP2, WRAP53, XRCC5, YRDC]	Upregulation
vesicle	GO_CellularComponent-EBI-UniProt-GOA-ACAP-ARAP_13.05.2021_00h00	5.68E-42	12.74345	530	[ABCC1, ABHD14B, ABI1, ACAA1, ACAT1, ACAT2, ACLY, ACO1, ACSL4, ACTBL2, ACTN1, ACTN4, ACTR10, ACTR1B, ACTR2, ADAM10, ADD2, ADSL, AGL, AGO2, AK1, AKR7A2, AKT1, ALCAM, ALDH1L2, ALDH2, ALDH7A1, ALDOC, ALPL, ANK2, ANKFY1, ANO6, ANP32E, ANTXR1, ANXA1, ANXA11, ANXA2, ANXA3, ANXA4, ANXA5, ANXA6, AP1M1, AP1S1, AP2A1, AP2A2, AP2B1, AP2M1, AP2S1, AP3B1, APAF1, APEH, APOE, APOOL, APPL2, APRT, ARAP1, ARCN1, ARF4, ARF6, ARHGAP1, ARHGDIA, ARHGEF12, ARL3,	Downregulation

					ARPC1A, ARSA, ARSB, ASL, ATIC, ATP1A1, ATP2B1, ATP2B2, ATP2B4, ATP6V1A, AUP1, B4GALT1, BAIAP2, BCAT2, BDH2, BET1, BIN1, BIRC6, BLVRB, BPTF, CACYBP, CALCOCO2, CALR, CALU, CAMK2D, CANX, CAP1, CAPN1, CAPN2, CAPNS1, CAPZA2, CASK, CAT, CAV1, CBR1, CCDC22, CCT2, CCT3, CCT4, CCT5, CCT6A, CCT7, CCT8, CD2AP, CD44, CD81, CDC42, CDC42BPA, CDC42BPB, CFAP20, CFL2, CHID1, CHST14, CKAP4, CMBL, CMPK1, CNDP2, COBLL1, COL1A1, COL1A2, COL4A2, COL6A1, COL6A2, COL6A3, COPA, COPB1, COPB2, COPE, COPG1, COPG2, COPS5, COPZ1, CORO1C, CPD, CPNE2, CSE1L, CSPG4, CTNNB1, CTSC, CUL4B, CXADR, CYB5R1, CYB5R3, CYFIP1, DBNL, DCXR, DDX5, DEGS1, DIAPH1, DICER1, DIPK2A, DLG1, DPP3, DPYSL2, DPYSL3, DSP, DSTN, DYNC1LI2, ECE1, ECPAS, EEA1, EEF2, EGFR, EHD1, EHD2, EHD4, ENDOD1, ENO2, EPCAM, EPN2, EPS8, EPS8L2, ERAP1, ERGIC1, ERGIC2, ERP44, ESD, F11R, FAF2, FAT1, FGG, FH, FKBP15, FKBP4, FKBP5, FLNB, FLT1, FN1, FNBP1L, FNDC3A, FSCN1, FTH1, GALK1, GALM, GARS1, GART, GATD1, GBE1, GEMIN4, GET3, GIPC1, GIPC2, GLA, GLUL, GMPPA, GNB2, GNG12, GNS, GOLGA2, GOLIM4, GOLT1B, GOPC, GOSR2, GOT2, GPC1, GPD1L, GPI, GSN, GSS, GSTK1, GSTM2, GSTM3, HEATR5A, HEXA, HLA-A, HMOX2, HNRNPL, HNRNPM, HOOK3, HSP90AB1, HSP90B1, HSPA13, HSPA2, HSPA4, HSPA5, HSPA9, HSPB1, HSPD1, HSPE1, HSPH1, HTRA1, HTT, HUWE1, IDH2, ILF2, IMPDH1, IMPDH2, IQGAP1, IQGAP2, IST1, ITCH, ITGAV, ITGB1, KDELR1, KDELR3, KIF5B, KLC1, KRT18, KRT19, KRT8, LAMB1, LAMC1, LDHB, LGALS1, LMAN1, LNPEP, LPCAT1, LRP1, LRRC57, LSM4, LYPLA2,	

					MACROH2A1, MAGED2, MAN1A1, MAN1A2, MANBA, MAP1LC3A, MAPK1, MAPK14, MARS1, MBLAC2, MEST, MFGE8, MGST1, MME, MOGS, MON2, MOSPD2, MOXD1, MSN, MTAP, MTHFD1, MVB12A, MYD88, MYH10, MYH9, MYO1C, MYO1E, MYO5A, NCAPG, NCKAP1, NCL, NDRG1, NDRG2, NECAP2, NECTIN2, NEDD4, NFKB1, NHLRC2, NIBAN1, NIBAN2, NID1, NIT2, NPM3, NQO2, NSUN2, NUCB2, OCLN, OPTN, OSTF1, P3H1, P4HB, PACS1, PAICS, PARD6B, PCBP2, PCOLCE, PDCD2, PDGFRB, PDIA3, PDIA4, PDIA6, PDLIM4, PDXK, PFAS, PFKL, PFKP, PHIP, PHPT1, PI4KA, PICALM, PIGT, PIK3C2A, PIK3R4, PIP4K2C, PKM, PLD3, PLEC, PLEKHA7, PLIN3, PLOD1, PLOD2, PLOD3, PLXNB2, PNP, PPIB, PPIC, PPP1R7, PRAF2, PRDX1, PRDX4, PRKACB, PRKAR2A, PRKAR2B, PRKCA, PRKCSH, PSMC6, PSMD1, PSMD2, PTPN1, PTPRD, PXDN, PYCARD, QDPR, RAB14, RAB18, RAB1A, RAB23, RAB2A, RAB32, RAB5B, RAB5C, RAB8A, RABGAP1L, RAC3, RAN, RAP1GDS1, RAP2A, RAP2B, RAP2C, RBM15, RCC2, RDX, RFC1, RFTN1, RHEB, RNH1, RNPEP, RP2, RPL22, RPL4, RPL5, RPN1, RPS2, RPS8, RRAS, S100A10, S100A11, SAR1A, SARS1, SCAMP2, SCARB2, SCFD1, SCFD2, SCPEP1, SCYL1, SDF4, SEC13, SEC16A, SEC22B, SEC23A, SEC23B, SEC24A, SEC24B, SEC24C, SEC24D, SEC31A, SELENBP1, SEPTIN2, SEPTIN7, SEPTIN8, SERBP1, SERPINB6, SERPINB9, SH3GL3, SH3GLB1, SLC1A3, SLC25A3, SLC2A1, SLC44A1, SLC44A2, SLC4A7, SLC9A3R2, SLIRP, SLK, SMC2, SMPDL3B, SMS, SNTB2, SNX17, SNX3, SNX4, SNX6, SNX9, SORD, SPAG9, SPARC, SPR, SPTAN1, SPTBN1, SRC, SRP14, SRPRA, SRSF7, SSBP1, SSR4,	

					STAM2, STX12, STX5, STXBP2, SUN2, SURF4, TAGLN2, TAX1BP1, TBC1D17, TBC1D4, TCP1, TF, TGM2, THBS1, TIMP3, TKFC, TKT, TLN1, TM9SF4, TMED10, TMED2, TMED5, TMED7, TMED9, TMEM168, TMEM192, TMX3, TOLLIP, TPM4, TTC38, TUBA4A, TUBB6, TXNDC5, TXNRD1, UBE2G1, UBR4, UGDH, UGGT1, UGP2, USE1, UTRN, VAMP2, VAMP3, VAT1, VIM, VPS25, VPS26A, VPS26B, VPS26C, VPS35, VPS35L, VPS36, VPS53, WARS1, XPNPEP1, XRCC5, XRCC6, YIF1A, YKT6, ZDHHC17, ZFYVE16]	
secretion by cell	GO_BiologicalProcess-EBI-UniProt-GOA-ACAP-ARAP_13.05.2021_00h00	5.68E-42	12.8223	184	[ABAT, ACAA1, ACLY, ACSL4, ACTN1, ACTN4, ACTR10, ACTR1B, ACTR2, ADAM10, AGL, ALDOC, ANO6, ANXA1, ANXA2, ANXA3, ANXA5, AP1M1, AP2A2, APAF1, APEH, APOE, APOOL, APRT, ARF6, ARFGAP3, ARFGEF1, ARSA, ARSB, B4GALT1, CANX, CAP1, CAPN1, CASK, CAT, CAVIN1, CBL, CCT2, CCT8, CD2AP, CD44, CHID1, CKAP4, CLASP1, COPB1, COPG1, COPG2, COPS5, CTBP2, CTSC, CYB5R1, CYB5R3, CYFIP1, CYP51A1, DBNL, DEGS1, DIAPH1, DPYSL2, DSP, EEF2, EGFR, ENDOD1, ERP44, FAF2, FGG, FLNB, FN1, FTH1, GIPC1, GLA, GLUD1, GLUL, GNS, GPI, GSN, HADHA, HMOX1, HMOX2, HOOK3, HSP90AB1, HUWE1, IDH2, ILF2, IMPDH1, IMPDH2, IQGAP1, IQGAP2, IST1, ITGAV, KIF5B, LPCAT1, LRP1, LRRC8A, LSM4, MAGED2, MANBA, MAP2K6, MAPK1, MAPK14, MCU, MGST1, MICAL1, MICAL3, MME, MOSPD2, MPC2, MYH10, MYH9, NFKB1, NHLRC2, NIBAN2, NIT2, NSF, ORC3, OSBP, OSTF1, OXCT1, P3H1, PAK1, PASK, PDIA4, PDXK, PFKL, PFKM, PHPT1, PIK3C2A, PKM, PNP, PPID, PRDX4, PREPL, PRKCA, PRKCSH, PSMD1, PSMD2, PSMD9, PXDN, PYCARD, RAB14, RAB18, RAB1A,	Downregulation

					RAB5B, RAB5C, RAB8A, RAP2B, RAP2C, RIC1, S100A11, SCFD2, SCRN1, SDF4, SEL1L, SEPTIN2, SERPINB6, SLC2A1, SLC44A2, SNX4, SNX6, SPARC, SPTAN1, SRP14, STXBP2, SURF4, TAGLN2, TF, THBS1, TIMP3, TLN1, TMED10, TMEM167A, TMF1, TMX3, TOLLIP, TUBA4A, TXNDC5, UBR4, USE1, VAMP2, VAMP3, VAT1, VPS35L, XRCC5, XRCC6, YKT6]	
DNA damage response, detection of DNA damage	GO_BiologicalProcess-EBI-UniProt-GOA-ACAP-ARAP_13.05.2021_00h00	5.68E-42	29.54545	13	[CUL4A, CUL4B, MRPS9, PARP1, POLD1, POLR1B, RFC1, RFC2, RFC3, RFC5, RPA1, RPA2, RPA3]	Upregulation
secretion	GO_BiologicalProcess-EBI-UniProt-GOA-ACAP-ARAP_13.05.2021_00h00	5.68E-42	12.09068	192	[ABAT, ACAA1, ACLY, ACSL4, ACTN1, ACTN4, ACTR10, ACTR1B, ACTR2, ADAM10, AGL, ALDOC, ANO6, ANXA1, ANXA2, ANXA3, ANXA5, AP1M1, AP2A2, APAF1, APEH, APOE, APOOL, APRT, ARF6, ARFGAP3, ARFGEF1, ARSA, ARSB, B4GALT1, CANX, CAP1, CAPN1, CASK, CAT, CAV1, CAVIN1, CBL, CCT2, CCT8, CD2AP, CD44, CHID1, CKAP4, CLASP1, COPB1, COPG1, COPG2, COPS2, COPS5, CTBP2, CTSC, CYB5R1, CYB5R3, CYFIP1, CYP51A1, DBNL, DEGS1, DHX15, DIAPH1, DPYSL2, DSP, EEF2, EGFR, ENDOD1, ERP44, FAF2, FGG, FLNB, FN1, FTH1, GIPC1, GLA, GLUD1, GLUL, GNS, GOT2, GPI, GSN, HADHA, HK2, HMOX1, HMOX2, HOOK3, HSP90AB1, HUWE1, IDH2, ILF2, IMPDH1, IMPDH2, IQGAP1, IQGAP2, IST1, ITGAV, KIF5B, LPCAT1, LRP1, LRRC8A, LSM4, MAGED2, MANBA, MAP2K6, MAPK1, MAPK14, MCU, MGST1, MICAL1, MICAL3, MME, MOSPD2, MPC2, MYH10, MYH9, NFKB1, NHLRC2, NIBAN2, NIT2, NSF, ORC3, OSBP, OSTF1, OXCT1, P3H1, PAK1, PASK, PDIA4, PDXK, PFKL, PFKM, PHPT1, PIK3C2A, PKM, PNP, POFUT2, PPID, PRDX4, PREPL, PRKAA1,	Downregulation

					PRKCA, PRKCSH, PSMD1, PSMD2, PSMD9, PXDN, PYCARD, RAB14, RAB18, RAB1A, RAB5B, RAB5C, RAB8A, RAP2B, RAP2C, RIC1, S100A11, SCFD2, SCRN1, SDF4, SEL1L, SEPTIN2, SERPINB6, SLC2A1, SLC44A2, SNX4, SNX6, SORD, SPARC, SPTAN1, SRP14, STXBP2, SURF4, TAGLN2, TF, THBS1, TIMP3, TLN1, TMED10, TMEM167A, TMF1, TMX3, TOLLIP, TUBA4A, TXNDC5, UBR4, USE1, VAMP2, VAMP3, VAT1, VPS35L, XRCC5, XRCC6, YKT6]	
anatomical structure homeostasis	GO_BiologicalProcess-EBI-UniProt-GOA-ACAP-ARAP_13.05.2021_00h00	5.68E-42	13.96226	74	[ACACA, ANKRD28, ANTXR1, ATP2B1, ATP2B2, ATP2B4, ATR, AURKB, BAG3, CCT2, CCT3, CCT4, CCT5, CCT6A, CCT7, CCT8, CDH3, CFL2, CHTF18, CTNNB1, CXADR, DCTN1, EGFR, EXOSC10, F11R, FH, GNL3, HSP90AB1, HSPB1, ITGB1, JAM3, LAMC1, LPCAT1, LRRK1, MAPK1, METAP1, MMAB, NEK7, OCLN, PARN, PARP1, PFKM, POLA1, POLA2, POLD1, POLR1B, PRDX1, PRIM1, PRIM2, PRKAA1, PRKCA, PRKDC, RAC3, RAD51, RFC1, RFC2, RFC3, RFC5, RIF1, RPA1, RPA2, RPA3, SLC2A1, SRC, TCP1, TELO2, TF, TJP2, USP7, WRAP53, XRCC5, XRCC6, XRN1, YAP1]	Upregulation
organic substance transport	GO_BiologicalProcess-EBI-UniProt-GOA-ACAP-ARAP_13.05.2021_00h00	5.68E-42	11.97234	329	[AAAS, ABAT, ABCB7, ABCC1, ACACA, ACSL1, ACSL4, ACTN4, AKT1, ANK2, ANK3, ANO6, ANXA1, ANXA2, AP1M1, AP1S1, AP2A1, AP2A2, AP2B1, AP2M1, AP2S1, AP3B1, APOE, APPL2, ARCN1, ARF4, ARF6, ARFGAP1, ARFGAP3, ARFGEF1, ARHGAP1, ARL1, ARL3, ARL6IP5, ATP6V1A, AUP1, BAG3, BET1, CALR, CAMK1, CANX, CAV1, CAVIN1, CCDC22, CD2AP, CD81, CDC42, CKAP5, COG1, COG6, COG7, COG8, COL1A1, COPA, COPB1, COPB2, COPE, COPG1, COPG2, COPZ1, CPSF2, CPSF3, CSE1L, CYB5R1, CYP51A1, DDX39B, DHX9, DPYSL2, ECT2, EGFR, EHD1, EIF4A3, EMD, ERBIN, ERLEC1, ESYT2, FAF2, FGG,	Downregulation

					FLNB, G3BP2, GBF1, GDAP1, GDI1, GET3, GIPC1, GLUD1, GLUL, GOLGA2, GOLT1B, GOPC, GOSR2, GOT2, GPC3, HACL1, HADHA, HDLBP, HERC2, HK2, HOOK3, HSP90AB1, HSP90B1, HSPA4, HSPA5, HSPA9, HSPB1, HSPB11, HSPD1, HUWE1, IDH2, IGF2BP3, IPO11, IPO4, IPO5, IST1, ITGAV, ITGB1, IWS1, KDELR1, KDELR3, KIF20A, KIF5B, KRT18, KTN1, LMAN1, LRP1, LRPPRC, LRRC8A, LSG1, LSM4, MACF1, MAIP1, MAN1A1, MAP2K6, MAPK1, MAPK14, MAPK8, MAVS, MCU, METAP1, MFSD10, MGST1, MIPEP, MON2, MPC2, MVB12A, MYH10, MYH9, MYO1C, MYO1E, MYO5A, NBAS, NDC1, NECAP2, NEDD4, NFKB1, NOL6, NPM1, NSF, NSUN2, NUP107, NUP133, NUP160, NUP210, NUP35, NUP50, OCLN, OPTN, OSBP, OXCT1, P3H1, PABPN1, PAK1, PARD3, PASK, PDIA4, PEA15, PFKL, PFKM, PGRMC2, PHIP, PHPT1, PIK3R4, PITRM1, PLSCR3, PNPT1, PPID, PPM1F, PPP1R7, PRAF2, PREPL, PRKAA1, PRKCA, PSMD9, PTPN1, PXMP2, RAB14, RAB18, RAB1A, RAB23, RAB2A, RAB32, RAB5B, RAB5C, RAB8A, RABGAP1L, RAN, RANBP2, RANGAP1, RFTN1, RIC1, RP2, RPL13A, RPL22, RPL27A, RPL4, RPL5, RPL7A, RPS2, RPS21, RPS8, RRBP1, SAR1A, SCAMP2, SCARB2, SCFD1, SCFD2, SDAD1, SEC13, SEC16A, SEC22B, SEC23A, SEC23B, SEC24A, SEC24B, SEC24C, SEC24D, SEC31A, SEC61G, SEH1L, SEL1L, SELENBP1, SESN2, SH3GLB1, SIL1, SIRT1, SLC1A3, SLC25A12, SLC25A24, SLC25A32, SLC25A5, SLC2A1, SLC2A10, SLC33A1, SLC44A1, SLC44A2, SLC4A7, SLC5A3, SLC7A3, SLC7A6, SNX17, SNX3, SNX30, SNX4, SNX6, SNX9, SORBS1, SPTBN1, SRC, SRP14, SRPRA, SRPRB, SRSF1, SRSF11, SRSF7,	

					SSB, SSR1, STAM2, STX12, STX5, STXBP2, STYX, SUPT6H, SURF4, SYMPK, TBC1D17, TBC1D22B, TBC1D4, TCAF1, TCP1, TEX2, TF, THBS1, TIMM50, TLK1, TM9SF4, TMED10, TMED2, TMED5, TMED7, TMED9, TMEM115, TMEM167A, TMF1, TNPO3, TOMM34, TRAM1, TRIM28, UBR5, UPF2, UPF3B, USE1, VAMP2, VAMP3, VLDLR, VPS25, VPS26A, VPS26B, VPS26C, VPS35, VPS35L, VPS36, VPS53, XPO5, XPO7, YIF1A, YKT6, YOD1, ZDHHC17, ZFYVE16]	
nitrogen compound transport	GO_BiologicalProcess-EBI-UniProt-GOA-ACAP-ARAP_13.05.2021_00h00	5.68E-42	13.12825	303	[AAAS, ABAT, ABCB7, ABCC1, ACSL4, ACTN4, AKT1, ANK2, ANK3, ANXA1, AP1M1, AP1S1, AP2A1, AP2A2, AP2B1, AP2M1, AP2S1, AP3B1, APOE, APPL2, ARCN1, ARF4, ARF6, ARFGAP1, ARFGAP3, ARFGEF1, ARHGAP1, ARL1, ARL3, ARL6IP5, ATP6V1A, AUP1, BAG3, BET1, CALR, CAMK1, CANX, CAV1, CAVIN1, CCDC22, CD2AP, CD81, CDC42, CKAP5, COG1, COG6, COG7, COG8, COL1A1, COPA, COPB1, COPB2, COPE, COPG1, COPG2, COPZ1, CPSF2, CPSF3, CSE1L, CYP51A1, DDX39B, DHX9, DPYSL2, ECT2, EGFR, EHD1, EIF4A3, EMD, ERBIN, ERLEC1, FAF2, FGG, FLNB, G3BP2, GBF1, GDAP1, GDI1, GET3, GIPC1, GLUD1, GLUL, GOLGA2, GOLT1B, GOPC, GOSR2, HACL1, HADHA, HERC2, HOOK3, HSP90AB1, HSP90B1, HSPA4, HSPA5, HSPA9, HSPB1, HSPB11, HSPD1, HUWE1, IDH2, IGF2BP3, IPO11, IPO4, IPO5, IST1, ITGB1, IWS1, KDELR1, KDELR3, KIF20A, KIF5B, KRT18, KTN1, LMAN1, LRP1, LRPPRC, LRRC8A, LSG1, LSM4, MACF1, MAIP1, MAN1A1, MAPK1, MAPK14, MAPK8, MAVS, MCU, METAP1, MGST1, MIPEP, MON2, MPC2, MVB12A, MYH10, MYH9, MYO1C, MYO1E, MYO5A, NBAS, NDC1, NECAP2, NEDD4, NOL6, NPM1, NSF, NSUN2, NUP107, NUP133,	Downregulation

					NUP160, NUP210, NUP35, NUP50, OPTN, OSBP, OXCT1, P3H1, PABPN1, PAK1, PARD3, PASK, PDIA4, PFKL, PFKM, PGRMC2, PHIP, PHPT1, PIK3R4, PITRM1, PNPT1, PPID, PPM1F, PRAF2, PREPL, PRKAA1, PRKCA, PSMD9, PTPN1, PXMP2, RAB14, RAB18, RAB1A, RAB23, RAB2A, RAB32, RAB5B, RAB5C, RAB8A, RABGAP1L, RAN, RANBP2, RANGAP1, RFTN1, RIC1, RP2, RPL13A, RPL22, RPL27A, RPL4, RPL5, RPL7A, RPS2, RPS21, RPS8, RRBP1, SAR1A, SCAMP2, SCARB2, SCFD1, SCFD2, SDAD1, SEC13, SEC16A, SEC22B, SEC23A, SEC23B, SEC24A, SEC24B, SEC24C, SEC24D, SEC31A, SEC61G, SEH1L, SEL1L, SELENBP1, SH3GLB1, SIL1, SLC12A4, SLC1A3, SLC25A12, SLC25A24, SLC25A32, SLC25A5, SLC2A1, SLC33A1, SLC44A1, SLC44A2, SLC7A3, SLC7A6, SNX17, SNX3, SNX30, SNX4, SNX6, SNX9, SPTBN1, SRC, SRP14, SRPRA, SRPRB, SRSF1, SRSF11, SRSF7, SSB, SSR1, STAM2, STX12, STX5, STXBP2, STYX, SUPT6H, SURF4, SYMPK, TBC1D17, TBC1D22B, TBC1D4, TCAF1, TCP1, TF, TIMM50, TLK1, TM9SF4, TMED10, TMED2, TMED5, TMED7, TMED9, TMEM115, TMEM167A, TNPO3, TOMM34, TRAM1, TRIM28, UBR5, UPF2, UPF3B, USE1, VAMP2, VAMP3, VLDLR, VPS25, VPS26A, VPS26B, VPS26C, VPS35, VPS35L, VPS36, VPS53, XPO5, XPO7, YIF1A, YKT6, YOD1, ZDHHC17, ZFYVE16]	
positive regulation of establishment of protein localization	GO_BiologicalProcess-EBI-UniProt-GOA-ACAP-ARAP_13.05.2021_00h00	5.68E-42	16.25344	59	[ABAT, ACSL4, ANK3, ARF6, BAG3, CAMK1, CCT2, CCT3, CCT4, CCT5, CCT6A, CCT7, CCT8, CD2AP, CD81, CDC42, ECT2, EGFR, EMD, FGG, GLUD1, GLUL, HSP90AB1, HUWE1, IPO5, KIF5B, LRP1, LRRC8A, MAPK1, MAPK14, MAPK8, MAVS, MCU, MPC2, MYH10, MYO1C,	No change

					MYO1E, OSBP, OXCT1, PAK1, PFKM, PHPT1, PPID, PRKAA1, PRKCA, PSMD9, RAN, SAR1A, SEC16A, SH3GLB1, SRC, TCAF1, TCP1, TM9SF4, TMED10, TRIM28, UBR5, VAMP2, WRAP53]	
extracellular exosome	GO_CellularComponent-EBI-UniProt-GOA-ACAP-ARAP_13.05.2021_00h00	5.68E-42	16.10355	367	[ABCC1, ABHD14B, ABI1, ACAT1, ACAT2, ACLY, ACO1, ACSL4, ACTBL2, ACTN1, ACTN4, ACTR1B, ACTR2, ADAM10, ADSL, AGO2, AK1, AKR7A2, ALCAM, ALDH1L2, ALDH2, ALDH7A1, ALDOC, ALPL, ANKFY1, ANO6, ANXA1, ANXA11, ANXA2, ANXA3, ANXA4, ANXA5, ANXA6, AP1M1, AP2M1, APAF1, APEH, APOE, APPL2, APRT, ARF4, ARF6, ARHGAP1, ARHGDIA, ARHGEF12, ARL3, ARPC1A, ARSA, ARSB, ASL, ATIC, ATP1A1, ATP2B1, ATP2B2, ATP2B4, ATP6V1A, AUP1, B4GALT1, BAIAP2, BCAT2, BDH2, BLVRB, BPTF, CACYBP, CALR, CANX, CAP1, CAPN1, CAPN2, CAPNS1, CAPZA2, CAT, CBR1, CCT2, CCT3, CCT4, CCT5, CCT6A, CCT7, CCT8, CD2AP, CD44, CD81, CDC42, CDC42BPA, CDC42BPB, CFAP20, CFL2, CHID1, CHST14, CKAP4, CMBL, CMPK1, CNDP2, COBLL1, COL1A2, COL4A2, COL6A1, COL6A2, COL6A3, COPA, CPD, CPNE2, CSE1L, CSPG4, CTNNB1, CTSC, CUL4B, CYB5R1, CYFIP1, DBNL, DCXR, DDX5, DICER1, DLG1, DPP3, DPYSL2, DSP, DSTN, ECE1, EEA1, EEF2, EHD1, EHD2, EHD4, ENDOD1, ENO2, EPCAM, EPS8, EPS8L2, ERAP1, ERP44, ESD, F11R, FAT1, FGG, FH, FKBP4, FKBP5, FLNB, FN1, FSCN1, FTH1, GALK1, GALM, GARS1, GART, GATD1, GBE1, GEMIN4, GET3, GIPC1, GIPC2, GLA, GLUL, GMPPA, GNB2, GNG12, GNS, GOLT1B, GOT2, GPC1, GPD1L, GPI, GSN, GSS, GSTK1, GSTM2, GSTM3, HEXA, HLA-A, HNRNPL, HNRNPM, HSP90AB1, HSP90B1, HSPA13, HSPA2, HSPA4, HSPA5, HSPA9, HSPB1,	Downregulation

					HSPD1, HSPE1, HSPH1, HTRA1, HUWE1, IDH2, IMPDH2, IQGAP1, IQGAP2, IST1, ITCH, ITGAV, ITGB1, KRT18, KRT19, KRT8, LAMB1, LAMC1, LDHB, LGALS1, LMAN1, LRRC57, LYPLA2, MACROH2A1, MAN1A1, MAN1A2, MARS1, MBLAC2, MEST, MFGE8, MME, MOGS, MON2, MSN, MTAP, MTHFD1, MVB12A, MYH10, MYH9, MYO1C, MYO1E, MYO5A, NCAPG, NCKAP1, NCL, NDRG1, NDRG2, NECTIN2, NEDD4, NIBAN1, NIBAN2, NID1, NIT2, NQO2, NSUN2, NUCB2, P3H1, P4HB, PAICS, PARD6B, PCBP2, PCOLCE, PDCD2, PDIA3, PDIA6, PDXK, PFAS, PFKL, PFKP, PHPT1, PI4KA, PIK3C2A, PIP4K2C, PKM, PLD3, PLEC, PLEKHA7, PLOD1, PLOD2, PLOD3, PLXNB2, PNP, PPIB, PPIC, PPP1R7, PRDX1, PRDX4, PRKACB, PRKAR2A, PRKAR2B, PRKCA, PSMC6, PSMD2, PTPRD, PXDN, QDPR, RAB14, RAB1A, RAB2A, RAB5B, RAB5C, RAB8A, RAC3, RAN, RAP1GDS1, RAP2B, RAP2C, RBM15, RDX, RFC1, RFTN1, RHEB, RNH1, RNPEP, RP2, RPL22, RPL4, RPL5, RPS2, RPS8, RRAS, S100A10, S100A11, SARS1, SCAMP2, SCARB2, SCPEP1, SDF4, SEC13, SELENBP1, SEPTIN2, SEPTIN7, SERBP1, SERPINB6, SERPINB9, SLC25A3, SLC2A1, SLC44A1, SLC44A2, SLC9A3R2, SLK, SMC2, SMPDL3B, SMS, SNX3, SNX9, SORD, SPAG9, SPR, SPTAN1, SPTBN1, SRC, SRPRA, SRSF7, SSBP1, SSR4, STXBP2, TAGLN2, TAX1BP1, TCP1, TF, TGM2, THBS1, TKFC, TKT, TLN1, TMED9, TOLLIP, TPM4, TTC38, TUBA4A, TUBB6, TXNDC5, TXNRD1, UBE2G1, UGDH, UGGT1, UGP2, UTRN, VAT1, VIM, VPS25, VPS35, VPS36, WARS1, XPNPEP1]	
myeloid cell activation involved in immune response	GO_BiologicalProcess-EBI-UniProt-	5.68E-42	16.61238	102	[ACAA1, ACLY, ACTR10, ACTR1B, ACTR2, ADAM10, AGL, ALDOC, ANO6, ANXA2, ANXA3,	Downregulation

	GOA-ACAP-ARAP_13.05.2021_00h00				AP1M1, AP2A2, APAF1, APEH, APRT, ARSA, ARSB, B4GALT1, CAP1, CAPN1, CAT, CBL, CCT2, CCT8, CD44, CKAP4, COPB1, CTSC, CYB5R3, CYFIP1, DBNL, DEGS1, DIAPH1, DSP, EEF2, ERP44, FAF2, FTH1, GLA, GNS, GPI, GSN, HMOX1, HMOX2, HOOK3, HSP90AB1, HUWE1, ILF2, IMPDH1, IMPDH2, IQGAP1, IQGAP2, IST1, ITGAV, LPCAT1, MANBA, MAPK1, MAPK14, MGST1, MME, MOSPD2, MYD88, MYO1C, NFKB1, NIT2, ORC3, OSTF1, PDXK, PFKL, PKM, PNP, PRDX4, PRKCSH, PSMD1, PSMD2, PXDN, PYCARD, RAB14, RAB18, RAB5B, RAB5C, RAP2B, RAP2C, S100A11, SERPINB6, SLC44A2, SNX4, SNX6, SPTAN1, SRP14, STXBP2, SURF4, TOLLIP, TXNDC5, UBR4, VAMP2, VAMP3, VAT1, VPS35L, XRCC5, XRCC6]	
exocytosis	GO_BiologicalProcess-EBI-UniProt-GOA-ACAP-ARAP_13.05.2021_00h00	5.68E-42	14.35845	141	[ACAA1, ACLY, ACTN1, ACTN4, ACTR10, ACTR1B, ACTR2, ADAM10, AGL, ALDOC, ANO6, ANXA1, ANXA2, ANXA3, ANXA5, AP1M1, AP2A2, APAF1, APEH, APOOL, APRT, ARFGEF1, ARSA, ARSB, B4GALT1, CAP1, CAPN1, CASK, CAT, CBL, CCT2, CCT8, CD44, CHID1, CKAP4, CLASP1, COPB1, COPS5, CTBP2, CTSC, CYB5R1, CYB5R3, CYFIP1, DBNL, DEGS1, DIAPH1, DSP, EEF2, ENDOD1, ERP44, FAF2, FGG, FN1, FTH1, GIPC1, GLA, GNS, GPI, GSN, HMOX1, HMOX2, HOOK3, HSP90AB1, HUWE1, ILF2, IMPDH1, IMPDH2, IQGAP1, IQGAP2, IST1, ITGAV, LPCAT1, MAGED2, MANBA, MAPK1, MAPK14, MGST1, MICAL1, MICAL3, MME, MOSPD2, MYH9, NFKB1, NHLRC2, NIT2, NSF, ORC3, OSTF1, PAK1, PDXK, PFKL, PIK3C2A, PKM, PNP, PRDX4, PREPL, PRKCSH, PSMD1, PSMD2, PXDN, PYCARD, RAB14, RAB18, RAB5B, RAB5C, RAB8A, RAP2B, RAP2C, S100A11, SCFD2, SCRN1, SDF4, SEPTIN2,	Downregulation

					SERPINB6, SLC44A2, SNX4, SNX6, SPARC, SPTAN1, SRP14, STXBP2, SURF4, TAGLN2, TF, THBS1, TIMP3, TLN1, TMED10, TMEM167A, TMX3, TOLLIP, TUBA4A, TXNDC5, UBR4, VAMP2, VAMP3, VAT1, VPS35L, XRCC5, XRCC6, YKT6]	
protein transport	GO_BiologicalProcess-EBI-UniProt-GOA-ACAP-ARAP_13.05.2021_00h00	5.68E-42	14.38053	260	[AAAS, ABAT, ACSL4, ACTN4, AKT1, ANK2, ANK3, ANXA1, AP1M1, AP1S1, AP2A1, AP2A2, AP2B1, AP2M1, AP2S1, AP3B1, APOE, APPL2, ARCN1, ARF4, ARF6, ARFGAP1, ARFGAP3, ARFGEF1, ARHGAP1, ARL1, ARL3, ATP6V1A, AUP1, BAG3, BET1, CALR, CAMK1, CANX, CAV1, CAVIN1, CCDC22, CD2AP, CD81, CDC42, COG1, COG6, COG7, COG8, COL1A1, COPA, COPB1, COPB2, COPE, COPG1, COPG2, COPZ1, CSE1L, CYP51A1, ECT2, EGFR, EHD1, EMD, ERBIN, ERLEC1, FAF2, FGG, GBF1, GDAP1, GDI1, GET3, GIPC1, GLUD1, GLUL, GOLGA2, GOLT1B, GOPC, GOSR2, HACL1, HADHA, HERC2, HOOK3, HSP90AB1, HSP90B1, HSPA4, HSPA5, HSPA9, HSPB1, HSPB11, HSPD1, HUWE1, IDH2, IPO11, IPO4, IPO5, IST1, KDELR1, KDELR3, KIF20A, KIF5B, KRT18, KTN1, LMAN1, LRP1, LRRC8A, LSG1, MACF1, MAIP1, MAN1A1, MAPK1, MAPK14, MAPK8, MAVS, MCU, METAP1, MIPEP, MON2, MPC2, MVB12A, MYH10, MYH9, MYO1C, MYO1E, MYO5A, NBAS, NDC1, NECAP2, NEDD4, NPM1, NSF, NUP107, NUP133, NUP160, NUP210, NUP35, NUP50, OPTN, OSBP, OXCT1, P3H1, PAK1, PARD3, PDIA4, PFKL, PFKM, PHIP, PHPT1, PIK3R4, PITRM1, PPID, PPM1F, PRAF2, PREPL, PRKAA1, PRKCA, PSMD9, PTPN1, PXMP2, RAB14, RAB18, RAB1A, RAB23, RAB2A, RAB32, RAB5B, RAB5C, RAB8A, RABGAP1L, RAN, RANBP2, RANGAP1, RFTN1, RIC1, RP2, RPL13A, RPL22, RPL27A, RPL4, RPL5,	Downregulation

					RPL7A, RPS2, RPS21, RPS8, RRBP1, SAR1A, SCAMP2, SCARB2, SCFD1, SCFD2, SDAD1, SEC13, SEC16A, SEC22B, SEC23A, SEC23B, SEC24A, SEC24B, SEC24C, SEC24D, SEC31A, SEC61G, SEH1L, SEL1L, SELENBP1, SH3GLB1, SIL1, SLC2A1, SNX17, SNX3, SNX30, SNX4, SNX6, SNX9, SPTBN1, SRC, SRP14, SRPRA, SRPRB, SSR1, STAM2, STX12, STX5, STXBP2, STYX, SURF4, TBC1D17, TBC1D22B, TBC1D4, TCAF1, TCP1, TF, TIMM50, TLK1, TM9SF4, TMED10, TMED2, TMED5, TMED7, TMED9, TMEM115, TMEM167A, TNPO3, TOMM34, TRAM1, TRIM28, UBR5, USE1, VAMP2, VAMP3, VLDLR, VPS25, VPS26A, VPS26B, VPS26C, VPS35, VPS35L, VPS36, VPS53, XPO5, XPO7, YIF1A, YKT6, YOD1, ZDHHC17, ZFYVE16]	
vesicle lumen	GO_CellularComponent-EBI-UniProt-GOA-ACAP-ARAP_13.05.2021_00h00	5.68E-42	18.52861	68	[ACAA1, ACLY, ACTN1, ACTN4, ACTR10, ACTR1B, ACTR2, AGL, ALDOC, ANXA2, APAF1, APOOL, APRT, ARSA, ARSB, CAP1, CAT, CCT2, CCT8, CTSC, CYB5R3, CYFIP1, DBNL, EEF2, EGFR, ERP44, FAF2, FGG, FN1, GLA, GNS, GPI, GSN, HOOK3, HSP90AB1, HUWE1, ILF2, IMPDH1, IMPDH2, IST1, LSM4, MAGED2, MAPK1, MAPK14, NFKB1, NHLRC2, NIT2, OSTF1, PDXK, PFKL, PKM, PNP, PRDX4, PSMD1, PSMD2, PYCARD, S100A11, SPARC, SPTAN1, SRP14, TF, THBS1, TIMP3, TOLLIP, TXNDC5, VAT1, XRCC5, XRCC6]	Downregulation
regulation of intracellular transport	GO_BiologicalProcess-EBI-UniProt-GOA-ACAP-ARAP_13.05.2021_00h00	5.68E-42	13.91753	54	[AAAS, ANK3, ANXA2, ARHGAP1, BAG3, CAMK1, CD81, CDC42, DHX9, ECT2, EHD1, EHD2, EMD, ERLEC1, GDI1, HSP90AB1, HUWE1, IPO5, IWS1, KIF5B, LMAN1, MAP1B, MAPK1, MAPK14, MAPK8, MAVS, MSN, MYO1C, MYO1E, NEDD4, NSUN2, PAK1, PRKAA1, PRKCA, PTPN1, RAB23, RAN, RANGAP1, RDX, SAR1A, SCFD1, SEC16A, SEC24B, SH3GLB1, SNX3, SRC,	Downregulation

					SUPT6H, TCAF1, TM9SF4, TRIM28, UBR5, USP7, VAMP2, YOD1]	
regulation of establishment of protein localization	GO_BiologicalProcess-EBI-UniProt-GOA-ACAP-ARAP_13.05.2021_00h00	5.68E-42	13.31115	80	[ABAT, ACSL4, ANK3, ANXA1, APOE, ARF6, BAG3, CAMK1, CCT2, CCT3, CCT4, CCT5, CCT6A, CCT7, CCT8, CD2AP, CD81, CDC42, CYP51A1, ECT2, EGFR, EMD, EPB41L5, ERLEC1, FGG, GDI1, GLUD1, GLUL, GOPC, HADHA, HSP90AB1, HUWE1, IDH2, IPO5, KIF5B, LMAN1, LRP1, LRRC8A, MAPK1, MAPK14, MAPK8, MAVS, MCU, MPC2, MYH10, MYO1C, MYO1E, OSBP, OXCT1, P3H1, PAK1, PFKL, PFKM, PHPT1, PPID, PPM1F, PRKAA1, PRKCA, PSMD9, PTPN1, RAB23, RAB8A, RAN, RANGAP1, SAR1A, SCFD1, SEC16A, SH3GLB1, SLC2A1, SNX3, SRC, TCAF1, TCP1, TM9SF4, TMED10, TRIM28, UBR5, VAMP2, WRAP53, YOD1]	Downregulation
regulation of cellular protein localization	GO_BiologicalProcess-EBI-UniProt-GOA-ACAP-ARAP_13.05.2021_00h00	5.68E-42	17.15266	100	[ADAM10, AKT1, ANK3, AP2B1, AP2M1, ARF6, ATP2B4, BAG3, CAMK1, CCT2, CCT3, CCT4, CCT5, CCT6A, CCT7, CCT8, CD2AP, CD81, CDC42, CDH2, CDK5RAP3, CTNNB1, DDRGK1, DLG1, ECT2, EGFR, EMD, EPB41, ERLEC1, FERMT2, GBF1, GDI1, GLUL, GNL3, GOPC, GPC1, GPC3, GPC6, GPD1L, GSN, HSP90AB1, HUWE1, IPO5, IQGAP1, ITGB1, KAT7, KIF5B, LMAN1, LRP1, MACROH2A1, MAGED1, MAPK1, MAPK14, MAPK8, MAVS, MEAK7, MESD, METAP1, MSN, MYO1C, MYO1E, NDC80, NPM1, NSFL1C, NVL, PAK1, PARP1, PICALM, PLK1, PPM1F, PPP1R9B, PPP2R5A, PRKAA1, PRKCA, PTPN1, RAB23, RAN, RANGAP1, RDX, ROCK2, SAR1A, SEC16A, SESN2, SH3GLB1, SIN3A, SLC5A3, SORBS1, SPTBN1, SRC, TCAF1, TCP1, TM9SF4, TRIM28, UBR5, VAMP2, WAPL, WRAP53, YAP1, YOD1, ZMYND8]	Downregulation
positive regulation of cellular protein localization	GO_BiologicalProcess-EBI-UniProt-	5.68E-42	20.63492	65	[AKT1, ANK3, AP2B1, ARF6, ATP2B4, BAG3, CCT2, CCT3, CCT4, CCT5, CCT6A, CCT7,	No change

	GOA-ACAP-ARAP_13.05.2021_00h00				CCT8, CD2AP, CD81, CDK5RAP3, DDRGK1, DLG1, ECT2, EGFR, EPB41, FERMT2, GNL3, GPD1L, HSP90AB1, HUWE1, IPO5, IQGAP1, ITGB1, KAT7, KIF5B, LRP1, MAPK1, MAPK14, MAPK8, MAVS, MEAK7, MESD, METAP1, MSN, MYO1C, MYO1E, NDC80, NPM1, PAK1, PARP1, PLK1, PPP1R9B, PRKAA1, RAN, RDX, ROCK2, SESN2, SH3GLB1, SLC5A3, SORBS1, SPTBN1, SRC, TCAF1, TCP1, TM9SF4, TRIM28, UBR5, WRAP53, YAP1]	
neutrophil mediated immunity	GO_BiologicalProcess-EBI-UniProt-GOA-ACAP-ARAP_13.05.2021_00h00	5.68E-42	17.08633	95	[ACAA1, ACLY, ACTR10, ACTR1B, ACTR2, ADAM10, AGL, ALDOC, ANO6, ANXA2, ANXA3, AP1M1, AP2A2, APAF1, APEH, APRT, ARSA, ARSB, B4GALT1, CAP1, CAPN1, CAT, CCT2, CCT8, CD44, CKAP4, COPB1, CTSC, CYB5R3, CYFIP1, DBNL, DEGS1, DIAPH1, DSP, EEF2, ERP44, FAF2, FTH1, GLA, GNS, GPI, GSN, HMOX2, HOOK3, HSP90AB1, HUWE1, ILF2, IMPDH1, IMPDH2, IQGAP1, IQGAP2, IST1, ITGAV, LPCAT1, MANBA, MAPK1, MAPK14, MGST1, MME, MOSPD2, MYD88, NFKB1, NIT2, OSTF1, PDXK, PFKL, PKM, PNP, PRDX4, PRKCSH, PSMD1, PSMD2, PXDN, PYCARD, RAB14, RAB18, RAB5B, RAB5C, RAP2B, RAP2C, S100A11, SERPINB6, SLC44A2, SPTAN1, SRP14, STXBP2, SURF4, TOLLIP, TXNDC5, UBR4, VAMP2, VAT1, VPS35L, XRCC5, XRCC6]	Downregulation
single-stranded DNA binding	GO_MolecularFunction-EBI-UniProt-GOA-ACAP-ARAP_13.05.2021_00h00	5.68E-42	23.4375	30	[ANXA1, DHX9, HSPD1, LRPPRC, MCM2, MCM3, MCM4, MCM5, MCM6, MCM7, MLH1, MSH2, MSH3, MYEF2, NUP35, PCBP2, POLA1, POLR1B, POLR2G, POLR3C, PURA, RAD51, RPA1, RPA2, RPA3, SAMHD1, SMC2, SSBP1, TDP1, TDP2]	Upregulation
intracellular protein transport	GO_BiologicalProcess-EBI-UniProt-GOA-ACAP-ARAP_13.05.2021_00h00	5.68E-42	15.76402	163	[AKT1, ANK3, AP1M1, AP1S1, AP2A1, AP2A2, AP2B1, AP2M1, AP2S1, AP3B1, APPL2, ARCN1, ARF4, ARF6, ARFGAP3, ARL1, AUP1, BAG3, CALR, CAMK1, CD81, CDC42, COG7, COPA, COPB1, COPB2, COPG1,	Downregulation

					COPG2, COPZ1, CSE1L, ECT2, EHD1, EMD, ERBIN, ERLEC1, FAF2, GDAP1, GDI1, GET3, GIPC1, GOSR2, HACL1, HERC2, HSP90AB1, HSP90B1, HSPA4, HSPA5, HSPA9, HSPB1, HSPD1, HUWE1, IPO11, IPO4, IPO5, KIF5B, LMAN1, MAIP1, MAN1A1, MAPK1, MAPK14, MAPK8, MAVS, METAP1, MIPEP, MYO1C, MYO1E, NEDD4, NPM1, NSF, NUP107, NUP133, NUP35, NUP50, PAK1, PARD3, PHIP, PIK3R4, PITRM1, PRKAA1, PRKCA, PTPN1, PXMP2, RAB14, RAB18, RAB1A, RAB23, RAB32, RAB5B, RAB5C, RAB8A, RABGAP1L, RAN, RANBP2, RANGAP1, RFTN1, RIC1, RPL13A, RPL22, RPL27A, RPL4, RPL5, RPL7A, RPS2, RPS21, RPS8, SAR1A, SCARB2, SCFD1, SCFD2, SEC13, SEC16A, SEC23A, SEC23B, SEC24A, SEC24B, SEC24C, SEC24D, SEC31A, SEC61G, SEL1L, SH3GLB1, SIL1, SNX17, SNX6, SNX9, SRP14, SRPRA, SRPRB, SSR1, STAM2, STX12, STX5, STXBP2, STYX, SURF4, TBC1D17, TBC1D22B, TBC1D4, TCAF1, TIMM50, TLK1, TM9SF4, TMED10, TMED2, TMED5, TMED7, TMED9, TNPO3, TOMM34, TRAM1, TRIM28, UBR5, VAMP2, VPS25, VPS26A, VPS26B, VPS26C, VPS35, VPS36, XPO5, XPO7, YOD1, ZFYVE16]	
protein localization to organelle	GO_BiologicalProcess-EBI-UniProt-GOA-ACAP-ARAP_13.05.2021_00h00	5.68E-42	15.3998	156	[ADAM10, AKT1, ANK2, ANTXR1, AP3B1, APPL2, ARF4, ARF6, ARL1, ARL2, ARL3, ATR, AUP1, AURKA, AURKB, BAG3, BUB1B, CALR, CCT2, CCT3, CCT4, CCT5, CCT6A, CCT7, CCT8, CD2AP, CD81, CDK5RAP3, COG7, COL1A1, CSE1L, CTCF, DDRGK1, DHX9, DIAPH1, DLG1, ECT2, EHD1, EMD, FERMT2, GBF1, GDAP1, GET3, GET4, GLUL, GNL3, GOSR2, GPC6, HACL1, HK1, HK2, HMOX1, HOOK3, HSP90AB1, HSPA4, HSPA5, HSPD1, HUWE1, IPO11, IPO4,	No change

					IPO5, KAT7, KDELR1, KDELR3, LMAN1, LRWD1, MACROH2A1, MAGED1, MAIP1, MAN1A1, MAPK1, MAPK14, MAPK8, MARCHF5, MAVS, MEAK7, METAP1, MIPEP, MMAB, MSH2, MSN, NDC80, NEDD4, NPM1, NSFL1C, NUP107, NUP133, NUP35, NUP50, NVL, OPTN, PACS1, PARP1, PIK3R4, PITRM1, PLK1, POLR1B, PPP1R9B, PRKAA1, PXMP2, RAB23, RAN, RANBP2, RANGAP1, RCC2, RDX, ROCK2, RPA1, RPA2, RPF2, RPL13A, RPL22, RPL27A, RPL4, RPL5, RPL7A, RPS2, RPS21, RPS8, RRS1, SCARB2, SEC13, SEC16A, SEC61G, SESN2, SH3GLB1, SIN3A, SRC, SRP14, SRPRA, SRPRB, SSB, STX5, SUN2, TCP1, TIA1, TIMM50, TMED10, TNPO3, TOLLIP, TOMM34, TRAM1, TRIM28, TTK, UBR5, UTP25, VAMP2, VPS25, VPS35, VPS36, WAPL, WRAP53, XRCC5, YAP1, ZFYVE16, ZWILCH]	
positive regulation of protein transport	GO_BiologicalProcess-EBI-UniProt-GOA-ACAP-ARAP_13.05.2021_00h00	5.68E-42	14.36782	50	[ABAT, ACSL4, ANK3, ARF6, BAG3, CAMK1, CD2AP, CD81, CDC42, ECT2, EGFR, EMD, FGG, GLUD1, GLUL, HSP90AB1, HUWE1, IPO5, KIF5B, LRP1, LRRC8A, MAPK1, MAPK14, MAPK8, MAVS, MCU, MPC2, MYH10, MYO1C, MYO1E, OSBP, OXCT1, PAK1, PFKM, PHPT1, PPID, PRKAA1, PRKCA, PSMD9, RAN, SAR1A, SEC16A, SH3GLB1, SRC, TCAF1, TM9SF4, TMED10, TRIM28, UBR5, VAMP2]	Downregulation
establishment of protein localization to organelle	GO_BiologicalProcess-EBI-UniProt-GOA-ACAP-ARAP_13.05.2021_00h00	5.68E-42	13.74322	76	[AKT1, AP3B1, APPL2, BAG3, CCT2, CCT3, CCT4, CCT5, CCT6A, CCT7, CCT8, CSE1L, ECT2, GDAP1, GET3, GOSR2, HACL1, HK1, HK2, HSP90AB1, HSPA4, HSPA5, HSPD1, HUWE1, IPO11, IPO4, IPO5, LMAN1, LRWD1, MACROH2A1, MAIP1, MAN1A1, MAPK1, MAPK14, MAPK8, MAVS, MIPEP, NEDD4, NUP107, NUP133, NUP35, NUP50, PIK3R4, PITRM1, PRKAA1, PXMP2, RAB23, RAN,	Upregulation

					RANBP2, RPL13A, RPL22, RPL27A, RPL4, RPL5, RPL7A, RPS2, RPS21, RPS8, SCARB2, SEC13, SEC61G, SH3GLB1, SRP14, SRPRA, SRPRB, TCP1, TIMM50, TNPO3, TOMM34, TRAM1, TRIM28, UBR5, VPS25, VPS36, WRAP53, ZFYVE16]	
regulation of telomerase RNA localization to Cajal body	GO_BiologicalProcess-EBI-UniProt-GOA-ACAP-ARAP_13.05.2021_00h00	5.68E-42	52.63158	10	[CCT2, CCT3, CCT4, CCT5, CCT6A, CCT7, CCT8, EXOSC10, PARN, TCP1]	Upregulation
positive regulation of telomerase RNA localization to Cajal body	GO_BiologicalProcess-EBI-UniProt-GOA-ACAP-ARAP_13.05.2021_00h00	5.68E-42	50	8	[CCT2, CCT3, CCT4, CCT5, CCT6A, CCT7, CCT8, TCP1]	Upregulation
secretory vesicle	GO_CellularComponent-EBI-UniProt-GOA-ACAP-ARAP_13.05.2021_00h00	5.68E-42	13.23132	147	[ACAA1, ACLY, ACTN1, ACTN4, ACTR10, ACTR1B, ACTR2, ADAM10, AGL, ALDOC, ANO6, ANXA11, ANXA2, ANXA3, AP1M1, AP2A2, APAF1, APEH, APOOL, APRT, ARSA, ARSB, ATP2B1, ATP6V1A, B4GALT1, BAIAP2, BIN1, CALR, CAP1, CAPN1, CAT, CAV1, CCT2, CCT8, CD44, CDC42, CKAP4, COL1A1, COPB1, COPS5, CTSC, CXADR, CYB5R1, CYB5R3, CYFIP1, DBNL, DEGS1, DIAPH1, DPYSL3, DSP, ECE1, EEF2, ERP44, FAF2, FGG, FN1, FNDC3A, FTH1, GARS1, GIPC1, GLA, GNS, GPI, GSN, HEXA, HMOX2, HOOK3, HSP90AB1, HSPD1, HUWE1, ILF2, IMPDH1, IMPDH2, IQGAP1, IQGAP2, IST1, ITGAV, LPCAT1, LRP1, LSM4, MAGED2, MANBA, MAPK1, MAPK14, MFGE8, MGST1, MME, MOSPD2, MOXD1, MYH9, MYO5A, NFKB1, NHLRC2, NIT2, OSTF1, PDXK, PFKL, PICALM, PKM, PNP, PRDX4, PRKCA, PRKCSH, PSMD1, PSMD2, PXDN, PYCARD, RAB14, RAB18, RAB5B, RAB5C, RAB8A, RAP2B, RAP2C, S100A11, SCFD2, SEC22B, SEPTIN2, SEPTIN8, SERPINB6,	Downregulation

					SH3GL3, SLC44A2, SLIRP, SPAG9, SPARC, SPTAN1, SRP14, STX12, STXBP2, SURF4, TCP1, TF, THBS1, TIMP3, TMED10, TMED2, TMED9, TMX3, TOLLIP, TXNDC5, UBR4, VAMP2, VAMP3, VAT1, VPS35L, XRCC5, XRCC6]	
DNA replication	GO_BiologicalProcess-EBI-UniProt-GOA-ACAP-ARAP_13.05.2021_00h00	5.68E-42	17.07989	62	[ANTXR1, ATR, CACYBP, CDC42, CHAF1A, CHEK1, CHTF18, DDX21, DHX9, EGFR, EXD2, GINS3, KAT7, LIG1, LIG3, LRWD1, MCM2, MCM3, MCM4, MCM5, MCM6, MCM7, MCMBP, MMAB, MSH3, ORC2, ORC3, ORC4, ORC5, PDS5A, POLA1, POLA2, POLB, POLD1, POLE, POLR1B, PRIM1, PRIM2, PSMC6, PSMD1, PSMD2, PSMD9, PSME3, PURA, RAD51, RFC1, RFC2, RFC3, RFC5, RPA1, RPA2, RPA3, S100A11, SAMHD1, SET, SIN3A, SSBP1, SSRP1, SUPT16H, WAPL, WDHD1, WDR18]	Upregulation
secretory granule	GO_CellularComponent-EBI-UniProt-GOA-ACAP-ARAP_13.05.2021_00h00	5.68E-42	14.34644	135	[ACAA1, ACLY, ACTN1, ACTN4, ACTR10, ACTR1B, ACTR2, ADAM10, AGL, ALDOC, ANO6, ANXA11, ANXA2, ANXA3, AP1M1, AP2A2, APAF1, APEH, APOOL, APRT, ARSA, ARSB, ATP6V1A, B4GALT1, BAIAP2, CALR, CAP1, CAPN1, CAT, CAV1, CCT2, CCT8, CD44, CDC42, CKAP4, COL1A1, COPB1, CTSC, CXADR, CYB5R1, CYB5R3, CYFIP1, DBNL, DEGS1, DIAPH1, DSP, ECE1, EEF2, ERP44, FAF2, FGG, FN1, FNDC3A, FTH1, GARS1, GLA, GNS, GPI, GSN, HEXA, HMOX2, HOOK3, HSP90AB1, HSPD1, HUWE1, ILF2, IMPDH1, IMPDH2, IQGAP1, IQGAP2, IST1, ITGAV, LPCAT1, LRP1, LSM4, MAGED2, MANBA, MAPK1, MAPK14, MFGE8, MGST1, MME, MOSPD2, MOXD1, MYH9, MYO5A, NFKB1, NHLRC2, NIT2, OSTF1, PDXK, PFKL, PKM, PNP, PRDX4, PRKCA, PRKCSH, PSMD1, PSMD2, PXDN, PYCARD, RAB14, RAB18, RAB5B, RAB5C, RAP2B, RAP2C, S100A11, SCFD2, SERPINB6, SH3GL3, SLC44A2, SLIRP, SPAG9,	Downregulation

					SPARC, SPTAN1, SRP14, STXBP2, SURF4, TCP1, TF, THBS1, TIMP3, TMED10, TMED2, TMX3, TOLLIP, TXNDC5, UBR4, VAMP2, VAMP3, VAT1, VPS35L, XRCC5, XRCC6]	
leukocyte degranulation	GO_BiologicalProcess-EBI-UniProt-GOA-ACAP-ARAP_13.05.2021_00h00	5.68E-42	16.77852	100	[ACAA1, ACLY, ACTR10, ACTR1B, ACTR2, ADAM10, AGL, ALDOC, ANO6, ANXA2, ANXA3, AP1M1, AP2A2, APAF1, APEH, APRT, ARSA, ARSB, B4GALT1, CAP1, CAPN1, CAT, CBL, CCT2, CCT8, CD44, CKAP4, COPB1, CTSC, CYB5R3, CYFIP1, DBNL, DEGS1, DIAPH1, DSP, EEF2, ERP44, FAF2, FTH1, GLA, GNS, GPI, GSN, HMOX1, HMOX2, HOOK3, HSP90AB1, HUWE1, ILF2, IMPDH1, IMPDH2, IQGAP1, IQGAP2, IST1, ITGAV, LPCAT1, MANBA, MAPK1, MAPK14, MGST1, MME, MOSPD2, NFKB1, NIT2, ORC3, OSTF1, PDXK, PFKL, PKM, PNP, PRDX4, PRKCSH, PSMD1, PSMD2, PXDN, PYCARD, RAB14, RAB18, RAB5B, RAB5C, RAP2B, RAP2C, S100A11, SERPINB6, SLC44A2, SNX4, SNX6, SPTAN1, SRP14, STXBP2, SURF4, TOLLIP, TXNDC5, UBR4, VAMP2, VAMP3, VAT1, VPS35L, XRCC5, XRCC6]	Downregulation
regulated exocytosis	GO_BiologicalProcess-EBI-UniProt-GOA-ACAP-ARAP_13.05.2021_00h00	5.68E-42	14.66513	127	[ACAA1, ACLY, ACTN1, ACTN4, ACTR10, ACTR1B, ACTR2, ADAM10, AGL, ALDOC, ANO6, ANXA2, ANXA3, ANXA5, AP1M1, AP2A2, APAF1, APEH, APOOL, APRT, ARSA, ARSB, B4GALT1, CAP1, CAPN1, CASK, CAT, CBL, CCT2, CCT8, CD44, CHID1, CKAP4, COPB1, CTBP2, CTSC, CYB5R1, CYB5R3, CYFIP1, DBNL, DEGS1, DIAPH1, DSP, EEF2, ENDOD1, ERP44, FAF2, FGG, FN1, FTH1, GIPC1, GLA, GNS, GPI, GSN, HMOX1, HMOX2, HOOK3, HSP90AB1, HUWE1, ILF2, IMPDH1, IMPDH2, IQGAP1, IQGAP2, IST1, ITGAV, LPCAT1, MAGED2, MANBA, MAPK1, MAPK14, MGST1, MICAL1, MME, MOSPD2, MYH9, NFKB1, NHLRC2, NIT2, ORC3, OSTF1, PDXK,	Downregulation

					PFKL, PKM, PNP, PRDX4, PREPL, PRKCSH, PSMD1, PSMD2, PXDN, PYCARD, RAB14, RAB18, RAB5B, RAB5C, RAP2B, RAP2C, S100A11, SDF4, SERPINB6, SLC44A2, SNX4, SNX6, SPARC, SPTAN1, SRP14, STXBP2, SURF4, TAGLN2, TF, THBS1, TIMP3, TLN1, TMED10, TMX3, TOLLIP, TUBA4A, TXNDC5, UBR4, VAMP2, VAMP3, VAT1, VPS35L, XRCC5, XRCC6]	
regulation of DNA metabolic process	GO_BiologicalProcess-EBI-UniProt-GOA-ACAP-ARAP_13.05.2021_00h00	5.68E-42	16.62531	67	[ACTR2, AKT1, ANTXR1, ANXA3, ATR, AURKB, CCT2, CCT3, CCT4, CCT5, CCT6A, CCT7, CCT8, CHEK1, CHTF18, CTNNB1, CUL4A, DAXX, DDX39B, DHX9, EGFR, ERCC2, EXOSC10, GNL3, HSP90AB1, KAT7, MAPK1, MBD3, MLH1, MMAB, MSH2, MSH3, MSH6, NEK7, NIBAN2, NPM1, NVL, PARN, PARP1, PDGFRB, PIK3CA, POLR1B, POLR2A, POLR2B, POLR2G, PRKDC, RAD51, RFC2, RFC3, RFC5, RIF1, RPA2, SIRT1, SMARCAD1, SRC, SUPT6H, TCP1, TIGAR, TRIM28, TRIP12, UBR5, USP7, USP9X, WDR18, WRAP53, XRCC5, XRN1]	Upregulation
telomerase RNA localization to Cajal body	GO_BiologicalProcess-EBI-UniProt-GOA-ACAP-ARAP_13.05.2021_00h00	5.68E-42	55	11	[CCT2, CCT3, CCT4, CCT5, CCT6A, CCT7, CCT8, EXOSC10, PARN, TCP1, WRAP53]	Upregulation
regulation of telomere maintenance	GO_BiologicalProcess-EBI-UniProt-GOA-ACAP-ARAP_13.05.2021_00h00	5.68E-42	26.43678	23	[ANTXR1, ATR, AURKB, CCT2, CCT3, CCT4, CCT5, CCT6A, CCT7, CCT8, CTNNB1, EXOSC10, GNL3, MAPK1, MMAB, NEK7, PARN, PARP1, SRC, TCP1, USP7, XRCC5, XRN1]	Upregulation
telomere maintenance	GO_BiologicalProcess-EBI-UniProt-GOA-ACAP-ARAP_13.05.2021_00h00	5.68E-42	24.72528	45	[ANKRD28, ANTXR1, ATR, AURKB, CCT2, CCT3, CCT4, CCT5, CCT6A, CCT7, CCT8, CHTF18, CTNNB1, EXOSC10, GNL3, HSP90AB1, MAPK1, MMAB, NEK7, PARN, PARP1, POLA1, POLA2, POLD1, POLR1B, PRIM1, PRIM2, PRKDC, RAD51, RFC1, RFC2, RFC3, RFC5, RIF1, RPA1, RPA2, RPA3, SRC, TCP1, TELO2, USP7, WRAP53, XRCC5, XRCC6, XRN1]	Upregulation

neutrophil activation involved in immune response	GO_BiologicalProcess-EBI-UniProt-GOA-ACAP-ARAP_13.05.2021_00h00	5.68E-42	17.4954	95	[ACAA1, ACLY, ACTR10, ACTR1B, ACTR2, ADAM10, AGL, ALDOC, ANO6, ANXA2, ANXA3, AP1M1, AP2A2, APAF1, APEH, APRT, ARSA, ARSB, B4GALT1, CAP1, CAPN1, CAT, CCT2, CCT8, CD44, CKAP4, COPB1, CTSC, CYB5R3, CYFIP1, DBNL, DEGS1, DIAPH1, DSP, EEF2, ERP44, FAF2, FTH1, GLA, GNS, GPI, GSN, HMOX2, HOOK3, HSP90AB1, HUWE1, ILF2, IMPDH1, IMPDH2, IQGAP1, IQGAP2, IST1, ITGAV, LPCAT1, MANBA, MAPK1, MAPK14, MGST1, MME, MOSPD2, MYD88, NFKB1, NIT2, OSTF1, PDXK, PFKL, PKM, PNP, PRDX4, PRKCSH, PSMD1, PSMD2, PXDN, PYCARD, RAB14, RAB18, RAB5B, RAB5C, RAP2B, RAP2C, S100A11, SERPINB6, SLC44A2, SPTAN1, SRP14, STXBP2, SURF4, TOLLIP, TXNDC5, UBR4, VAMP2, VAT1, VPS35L, XRCC5, XRCC6]	Downregulation
nuclear origin of replication recognition complex	GO_CellularComponent-EBI-UniProt-GOA-ACAP-ARAP_13.05.2021_00h00	5.68E-42	58.33333	7	[LRWD1, MCM2, MCM7, ORC2, ORC3, ORC4, ORC5]	Upregulation
lysosome	GO_CellularComponent-EBI-UniProt-GOA-ACAP-ARAP_13.05.2021_00h00	5.68E-42	12.41744	94	[ACLY, ACTR10, ACTR2, ANK2, ANK3, ANKFY1, ANXA1, ANXA11, ANXA2, ANXA6, AP1M1, AP1S1, AP2A1, AP2A2, AP2B1, AP2M1, AP2S1, AP3B1, ARSA, ARSB, ATP6V1A, B4GALT1, CAP1, CAPN1, CAPN2, CAT, CCT2, CCT8, CHID1, CKAP4, COL6A1, CSPG4, CTSC, CYB5R3, DAGLB, DYNC1LI2, ECE1, FAF2, GLA, GNB2, GNS, GOLT1B, GOPC, GPC1, GPC3, GPC6, HEXA, HSP90AB1, IMPDH1, IST1, LARS1, LNPEP, LPCAT1, LRBA, LRP1, LRRC8A, MANBA, MAPK1, MEAK7, MGST1, MYO5A, NSF, OCLN, PDGFRB, PHIP, PLD3, PON2, PRKCSH, PSMD1, PYCARD, RAB14, RAB2A, RAB5C, RHEB, SCARB2, SEC13, SEH1L, SLC12A4, SLC44A2, SNX6, SPAG9, SRC, STXBP2, SURF4,	Downregulation

					TMEM192, TMEM97, TOLLIP, TXNDC5, USE1, USP5, VAT1, VLDLR, VPS26A, VPS35]	
DNA repair	GO_BiologicalProcess-EBI-UniProt-GOA-ACAP-ARAP_13.05.2021_00h00	5.68E-42	14.72995	90	[ACTR2, ANTXR1, ATR, BAZ1B, BCCIP, CCNH, CDK7, CHAF1A, CHEK1, CHEK2, COPS2, COPS5, COPS7A, CUL4A, CUL4B, DHX9, EGFR, EMSY, ERCC2, EXD2, FANCD2, FANCI, FH, FSCN1, GTF2I, HERC2, HUWE1, INO80C, KAT7, KDM2A, KIF22, LIG1, LIG3, MAPK8, MCM2, MCM3, MCM4, MCM5, MCM6, MCM7, MDC1, MLH1, MMAB, MSH2, MSH3, MSH6, NPM1, NTHL1, PARP1, PDS5A, PIAS4, POLA1, POLB, POLD1, POLE, POLR1B, POLR2A, POLR2B, POLR2G, PRKDC, RAD51, RFC1, RFC2, RFC3, RFC5, RIF1, RPA1, RPA2, RPA3, SAMHD1, SIRT1, SMARCA5, SMARCAD1, SSRP1, SUPT16H, TDP1, TDP2, TIGAR, TRIM28, TRIP12, TRIP13, UBR5, UFL1, UNG, USP47, USP7, WDHD1, WRAP53, XRCC5, XRCC6]	Upregulation
DNA replication preinitiation complex	GO_CellularComponent-EBI-UniProt-GOA-ACAP-ARAP_13.05.2021_00h00	5.68E-42	66.66666	8	[GINS3, MCM2, MCM3, MCM4, MCM5, MCM6, MCM7, ORC3]	Upregulation
protein localization to chromosome	GO_BiologicalProcess-EBI-UniProt-GOA-ACAP-ARAP_13.05.2021_00h00	5.68E-42	29.29293	29	[ANTXR1, ATR, AURKB, BUB1B, CCT2, CCT3, CCT4, CCT5, CCT6A, CCT7, CCT8, CTCF, GNL3, LRWD1, MACROH2A1, MMAB, MSH2, NDC80, PLK1, POLR1B, RCC2, RPA1, RPA2, TCP1, TTK, WAPL, WRAP53, XRCC5, ZWILCH]	Upregulation
nuclear replication fork	GO_CellularComponent-EBI-UniProt-GOA-ACAP-ARAP_13.05.2021_00h00	5.68E-42	36.84211	14	[BAZ1B, MCM3, POLA1, POLA2, POLD1, POLR1B, PRIM1, PRIM2, RPA1, RPA2, RPA3, SMARCA5, SMARCAD1, WDHD1]	Upregulation
positive regulation of DNA metabolic process	GO_BiologicalProcess-EBI-UniProt-GOA-ACAP-ARAP_13.05.2021_00h00	5.68E-42	21.42857	45	[ACTR2, AKT1, ANTXR1, ANXA3, ATR, AURKB, CCT2, CCT3, CCT4, CCT5, CCT6A, CCT7, CCT8, CHTF18, CTNNB1, DDX39B, DHX9, EGFR, ERCC2, GNL3, HSP90AB1, MAPK1, MLH1, MMAB, MSH2, NEK7, NVL, PARN,	Upregulation

					PARP1, PDGFRB, PRKDC, RAD51, RFC2, RFC3, RFC5, RIF1, SIRT1, SRC, TCP1, TIGAR, TRIM28, USP7, USP9X, WRAP53, XRCC5]	
establishment of protein localization to chromosome	GO_BiologicalProcess-EBI-UniProt-GOA-ACAP-ARAP_13.05.2021_00h00	5.68E-42	44	11	[CCT2, CCT3, CCT4, CCT5, CCT6A, CCT7, CCT8, LRWD1, MACROH2A1, TCP1, WRAP53]	Upregulation
DNA biosynthetic process	GO_BiologicalProcess-EBI-UniProt-GOA-ACAP-ARAP_13.05.2021_00h00	5.68E-42	18.96552	44	[ANKRD28, ANTXR1, ATR, AURKB, CCT2, CCT3, CCT4, CCT5, CCT6A, CCT7, CCT8, CHTF18, CTNNB1, DDX39B, EXOSC10, HSP90AB1, LIG1, LIG3, MAPK1, MMAB, NEK7, NIBAN2, NVL, PARN, PDGFRB, POLA1, POLB, POLD1, POLE, POLR1B, RFC1, RFC2, RFC3, RFC5, RPA1, RPA2, RPA3, SIRT1, SRC, TCP1, TELO2, WRAP53, XRCC5, XRN1]	Upregulation
regulation of protein localization to nucleus	GO_BiologicalProcess-EBI-UniProt-GOA-ACAP-ARAP_13.05.2021_00h00	5.68E-42	23.44828	34	[AKT1, BAG3, CCT2, CCT3, CCT4, CCT5, CCT6A, CCT7, CCT8, CD2AP, CDK5RAP3, ECT2, FERMT2, GLUL, HSP90AB1, IPO5, KAT7, MAGED1, MAPK1, MAPK14, MAVS, NPM1, NVL, PARP1, PLK1, RAB23, RAN, SESN2, SIN3A, SRC, TCP1, TRIM28, UBR5, YAP1]	Upregulation
positive regulation of protein localization to nucleus	GO_BiologicalProcess-EBI-UniProt-GOA-ACAP-ARAP_13.05.2021_00h00	5.68E-42	32.22222	29	[AKT1, BAG3, CCT2, CCT3, CCT4, CCT5, CCT6A, CCT7, CCT8, CD2AP, CDK5RAP3, ECT2, FERMT2, HSP90AB1, IPO5, KAT7, MAPK1, MAPK14, MAVS, NPM1, PARP1, PLK1, RAN, SESN2, SRC, TCP1, TRIM28, UBR5, YAP1]	Upregulation
nuclear replisome	GO_CellularComponent-EBI-UniProt-GOA-ACAP-ARAP_13.05.2021_00h00	5.68E-42	40	10	[MCM3, POLA1, POLA2, POLD1, POLR1B, PRIM1, PRIM2, RPA1, RPA2, RPA3]	Upregulation
regulation of protein localization to chromosome, telomeric region	GO_BiologicalProcess-EBI-UniProt-GOA-ACAP-ARAP_13.05.2021_00h00	5.68E-42	78.57143	11	[CCT2, CCT3, CCT4, CCT5, CCT6A, CCT7, CCT8, GNL3, MACROH2A1, TCP1, WRAP53]	Upregulation
DNA-dependent DNA replication	GO_BiologicalProcess-EBI-	5.68E-42	20.85107	49	[ANTXR1, ATR, CHTF18, DDX21, EXD2, GINS3, KAT7, LIG1, LIG3,	Upregulation

	UniProt-GOA-ACAP-ARAP_13.05.2021_00h00				LRWD1, MCM2, MCM3, MCM4, MCM5, MCM6, MCM7, MCMBP, MMAB, MSH3, ORC2, ORC3, ORC4, ORC5, POLA1, POLA2, POLB, POLD1, POLE, POLR1B, PRIM1, PRIM2, PSMC6, PSMD1, PSMD2, PSMD9, PSME3, PURA, RAD51, RFC1, RFC2, RFC3, RFC5, RPA1, RPA2, RPA3, SAMHD1, SSBP1, WDHD1, WDR18]	
DNA replication initiation	GO_BiologicalProcess-EBI-UniProt-GOA-ACAP-ARAP_13.05.2021_00h00	5.68E-42	44.44444	24	[GINS3, KAT7, LRWD1, MCM2, MCM3, MCM4, MCM5, MCM6, MCM7, ORC2, ORC3, ORC4, ORC5, POLA1, POLA2, POLE, POLR1B, PRIM1, PRIM2, PURA, RPA1, RPA2, RPA3, WDR18]	Upregulation
telomere maintenance via semi-conservative replication	GO_BiologicalProcess-EBI-UniProt-GOA-ACAP-ARAP_13.05.2021_00h00	5.68E-42	32.55814	14	[CHTF18, POLA1, POLA2, POLD1, POLR1B, PRIM1, PRIM2, RFC1, RFC2, RFC3, RFC5, RPA1, RPA2, RPA3]	Upregulation
positive regulation of telomere maintenance	GO_BiologicalProcess-EBI-UniProt-GOA-ACAP-ARAP_13.05.2021_00h00	5.68E-42	32.14286	18	[ANTXR1, ATR, AURKB, CCT2, CCT3, CCT4, CCT5, CCT6A, CCT7, CCT8, CTNNB1, GNL3, MAPK1, MMAB, NEK7, PARN, TCP1, XRCC5]	Upregulation
secretory granule lumen	GO_CellularComponent-EBI-UniProt-GOA-ACAP-ARAP_13.05.2021_00h00	5.68E-42	18.55956	67	[ACAA1, ACLY, ACTN1, ACTN4, ACTR10, ACTR1B, ACTR2, AGL, ALDOC, ANXA2, APAF1, APOOL, APRT, ARSA, ARSB, CAP1, CAT, CCT2, CCT8, CTSC, CYB5R3, CYFIP1, DBNL, EEF2, ERP44, FAF2, FGG, FN1, GLA, GNS, GPI, GSN, HOOK3, HSP90AB1, HUWE1, ILF2, IMPDH1, IMPDH2, IST1, LSM4, MAGED2, MAPK1, MAPK14, NFKB1, NHLRC2, NIT2, OSTF1, PDXK, PFKL, PKM, PNP, PRDX4, PSMD1, PSMD2, PYCARD, S100A11, SPARC, SPTAN1, SRP14, TF, THBS1, TIMP3, TOLLIP, TXNDC5, VAT1, XRCC5, XRCC6]	Downregulation
ficolin-1-rich granule	GO_CellularComponent-EBI-UniProt-GOA-ACAP-ARAP_13.05.2021_00h00	5.68E-42	20.38835	42	[ACLY, ACTR10, ACTR1B, ACTR2, AGL, ALDOC, AP2A2, APAF1, APEH, ARSB, CAPN1, CAT, CCT8, COPB1, DBNL, DIAPH1, DSP, EEF2, FTH1, GNS, GPI, GSN, HOOK3, HSP90AB1, HUWE1, ILF2, IMPDH1, IMPDH2,	No change

					MAPK1, MAPK14, OSTF1, PFKL, PKM, PNP, PRDX4, PSMD2, PXDN, SERPINB6, SRP14, UBR4, VPS35L, XRCC6]	
regulation of DNA biosynthetic process	GO_BiologicalProcess-EBI-UniProt-GOA-ACAP-ARAP_13.05.2021_00h00	5.68E-42	24	30	[ANTXR1, ATR, AURKB, CCT2, CCT3, CCT4, CCT5, CCT6A, CCT7, CCT8, CHTF18, CTNNB1, DDX39B, EXOSC10, HSP90AB1, MAPK1, MMAB, NEK7, NIBAN2, NVL, PARN, PDGFRB, RFC2, RFC3, RFC5, SRC, TCP1, WRAP53, XRCC5, XRN1]	Upregulation
pre-replicative complex assembly involved in nuclear cell cycle DNA replication	GO_BiologicalProcess-EBI-UniProt-GOA-ACAP-ARAP_13.05.2021_00h00	5.68E-42	87.5	7	[MCM2, MCM3, MCM4, MCM5, MCM6, MCM7, ORC3]	Upregulation
azurophil granule	GO_CellularComponent-EBI-UniProt-GOA-ACAP-ARAP_13.05.2021_00h00	5.68E-42	18.3908	32	[ACLY, ACTR10, ACTR2, ANXA11, ANXA2, ARSA, ARSB, B4GALT1, CAP1, CCT2, CCT8, CKAP4, CTSC, CYB5R3, FAF2, GLA, GNS, HEXA, IMPDH1, IST1, LPCAT1, MANBA, MAPK1, MGST1, PSMD1, PYCARD, RAB5C, STXBP2, SURF4, TOLLIP, TXNDC5, VAT1]	Downregulation
mitotic DNA replication	GO_BiologicalProcess-EBI-UniProt-GOA-ACAP-ARAP_13.05.2021_00h00	5.68E-42	52.94118	9	[GINS3, LIG1, MCM2, MCM3, MCM4, MCM6, MCM7, POLA1, RAD51]	Upregulation
regulation of telomere maintenance via telomere lengthening	GO_BiologicalProcess-EBI-UniProt-GOA-ACAP-ARAP_13.05.2021_00h00	5.68E-42	31.34328	21	[ANTXR1, ATR, AURKB, CCT2, CCT3, CCT4, CCT5, CCT6A, CCT7, CCT8, CTNNB1, EXOSC10, MAPK1, MMAB, NEK7, PARN, PARP1, SRC, TCP1, XRCC5, XRN1]	Upregulation
alpha DNA polymerase:primase complex	GO_CellularComponent-EBI-UniProt-GOA-ACAP-ARAP_13.05.2021_00h00	5.68E-42	100	5	[MCM3, POLA1, POLA2, PRIM1, PRIM2]	Upregulation
DNA strand elongation involved in DNA replication	GO_BiologicalProcess-EBI-UniProt-GOA-ACAP-ARAP_13.05.2021_00h00	5.68E-42	42.85714	9	[GINS3, LIG1, LIG3, MCM3, MCM4, MCM7, POLA1, POLE, RFC3]	Upregulation

RNA-dependent DNA biosynthetic process	GO_BiologicalProcess-EBI-UniProt-GOA-ACAP-ARAP_13.05.2021_00h00	5.68E-42	23.89381	27	[ANKRD28, ANTXR1, ATR, AURKB, CCT2, CCT3, CCT4, CCT5, CCT6A, CCT7, CCT8, CTNNB1, EXOSC10, HSP90AB1, MAPK1, MMAB, NEK7, NVL, PARN, RFC1, RPA1, SRC, TCP1, TELO2, WRAP53, XRCC5, XRN1]	Upregulation
base-excision repair	GO_BiologicalProcess-EBI-UniProt-GOA-ACAP-ARAP_13.05.2021_00h00	5.68E-42	22.5	18	[HUWE1, LIG1, LIG3, NTHL1, PARP1, POLB, POLD1, POLE, POLR1B, RFC1, RFC2, RFC3, RFC5, RPA1, RPA2, RPA3, UNG, USP47]	Upregulation
base-excision repair, gap-filling	GO_BiologicalProcess-EBI-UniProt-GOA-ACAP-ARAP_13.05.2021_00h00	5.68E-42	39.02439	16	[LIG1, LIG3, NTHL1, PARP1, POLB, POLD1, POLE, POLR1B, RFC1, RFC2, RFC3, RFC5, RPA1, RPA2, RPA3, UNG]	Upregulation
nucleotide-excision repair	GO_BiologicalProcess-EBI-UniProt-GOA-ACAP-ARAP_13.05.2021_00h00	5.68E-42	25.61984	31	[CCNH, CDK7, COPS2, COPS5, COPS7A, CUL4A, CUL4B, ERCC2, FSCN1, KAT7, LIG1, LIG3, NTHL1, PARP1, POLA1, POLB, POLD1, POLE, POLR1B, POLR2A, POLR2B, POLR2G, RFC1, RFC2, RFC3, RFC5, RPA1, RPA2, RPA3, SIRT1, USP7]	Upregulation
nucleotide-excision repair, DNA gap filling	GO_BiologicalProcess-EBI-UniProt-GOA-ACAP-ARAP_13.05.2021_00h00	5.68E-42	40.625	13	[LIG1, LIG3, POLB, POLD1, POLE, POLR1B, RFC1, RFC2, RFC3, RFC5, RPA1, RPA2, RPA3]	Upregulation
telomere maintenance via telomere lengthening	GO_BiologicalProcess-EBI-UniProt-GOA-ACAP-ARAP_13.05.2021_00h00	5.68E-42	29.78723	28	[ANKRD28, ANTXR1, ATR, AURKB, CCT2, CCT3, CCT4, CCT5, CCT6A, CCT7, CCT8, CTNNB1, EXOSC10, HSP90AB1, MAPK1, MMAB, NEK7, PARN, PARP1, RAD51, RFC1, RPA1, SRC, TCP1, TELO2, WRAP53, XRCC5, XRN1]	Upregulation
protein localization to chromosome, telomeric region	GO_BiologicalProcess-EBI-UniProt-GOA-ACAP-ARAP_13.05.2021_00h00	5.68E-42	46.875	15	[ANTXR1, ATR, CCT2, CCT3, CCT4, CCT5, CCT6A, CCT7, CCT8, GNL3, MACROH2A1, MMAB, TCP1, WRAP53, XRCC5]	Upregulation
establishment of protein localization to telomere	GO_BiologicalProcess-EBI-UniProt-GOA-ACAP-ARAP_13.0	5.68E-42	56.25	9	[CCT2, CCT3, CCT4, CCT5, CCT6A, CCT7, CCT8, TCP1, WRAP53]	Upregulation

	5.2021_00h00					
positive regulation of DNA biosynthetic process	GO_BiologicalProcess-EBI-UniProt-GOA-ACAP-ARAP_13.05.2021_00h00	5.68E-42	36	27	[ANTXR1, ATR, AURKB, CCT2, CCT3, CCT4, CCT5, CCT6A, CCT7, CCT8, CHTF18, CTNNB1, DDX39B, HSP90AB1, MAPK1, MMAB, NEK7, NVL, PARN, PDGFRB, RFC2, RFC3, RFC5, SRC, TCP1, WRAP53, XRCC5]	Upregulation
nucleotide-excision repair, DNA incision	GO_BiologicalProcess-EBI-UniProt-GOA-ACAP-ARAP_13.05.2021_00h00	5.68E-42	34.09091	15	[CUL4A, CUL4B, ERCC2, FSCN1, NTHL1, PARP1, POLD1, POLR1B, RFC1, RFC2, RFC3, RFC5, RPA1, RPA2, RPA3]	Upregulation
azurophil granule lumen	GO_CellularComponent-EBI-UniProt-GOA-ACAP-ARAP_13.05.2021_00h00	5.68E-42	21.35922	22	[ACLY, ACTR10, ACTR2, ANXA2, ARSA, ARSB, CAP1, CCT2, CCT8, CTSC, CYB5R3, FAF2, GLA, GNS, IMPDH1, IST1, MAPK1, PSMD1, PYCARD, TOLLIP, TXNDC5, VAT1]	Downregulation
mitotic DNA replication initiation	GO_BiologicalProcess-EBI-UniProt-GOA-ACAP-ARAP_13.05.2021_00h00	5.68E-42	85.71429	6	[GINS3, MCM2, MCM3, MCM4, MCM7, POLA1]	Upregulation
ficolin-1-rich granule lumen	GO_CellularComponent-EBI-UniProt-GOA-ACAP-ARAP_13.05.2021_00h00	5.68E-42	25	35	[ACLY, ACTR10, ACTR1B, ACTR2, AGL, ALDOC, APAF1, APEH, ARSB, CAPN1, CAT, CCT8, DBNL, EEF2, FTH1, GNS, GPI, GSN, HOOK3, HSP90AB1, HUWE1, ILF2, IMPDH1, IMPDH2, MAPK1, MAPK14, OSTF1, PFKL, PKM, PNP, PRDX4, PSMD2, PXDN, SRP14, XRCC6]	No change
transcription-coupled nucleotide-excision repair	GO_BiologicalProcess-EBI-UniProt-GOA-ACAP-ARAP_13.05.2021_00h00	5.68E-42	28.04878	23	[CCNH, CDK7, COPS2, COPS5, COPS7A, CUL4A, CUL4B, ERCC2, LIG1, LIG3, POLD1, POLR1B, POLR2A, POLR2B, POLR2G, RFC1, RFC2, RFC3, RFC5, RPA1, RPA2, RPA3, USP7]	Upregulation
protein localization to nuclear body	GO_BiologicalProcess-EBI-UniProt-GOA-ACAP-ARAP_13.05.2021_00h00	5.68E-42	75	9	[CCT2, CCT3, CCT4, CCT5, CCT6A, CCT7, CCT8, TCP1, WRAP53]	Upregulation
positive regulation of protein localization to Cajal body	GO_BiologicalProcess-EBI-UniProt-GOA-ACAP-	5.68E-42	72.72727	8	[CCT2, CCT3, CCT4, CCT5, CCT6A, CCT7, CCT8, TCP1]	Upregulation

	ARAP_13.05.2021_00h00					
positive regulation of telomere maintenance via telomerase	GO_BiologicalProcess-EBI-UniProt-GOA-ACAP-ARAP_13.05.2021_00h00	5.68E-42	44.73684	17	[ANTXR1, ATR, AURKB, CCT2, CCT3, CCT4, CCT5, CCT6A, CCT7, CCT8, CTNNB1, MAPK1, MMAB, NEK7, PARN, TCP1, XRCC5]	Upregulation

**Supplementary Table S3.** Gene Set Enrichment Analysis for iVSMCs vs Primary Aortic Smooth Muscle Cells (Upregulation indicates upregulation in iVSMCs)						
**GOTerm**	**Ontology Source**	**Group PValue**	**% Associated Genes**	**Nr. Genes**	**Associated Genes Found**	**Term Status**
heterochromatin	GO_CellularComponent-EBI-UniProt-GOA-ACAP-ARAP_13.05.2021_00h00	9.76E-08	26.829268	22	[BAZ1B, CBX1, CBX2, CBX3, CBX5, CBX8, DNMT1, H1-5, KDM4B, LRWD1, MACROH2A1, MBD3, MPHOSPH8, NOP53, PML, RNF2, RNF40, SIRT1, SMARCAD1, TCP1, TRIM28, UHRF2]	Upregulation
synapse	GO_CellularComponent-EBI-UniProt-GOA-ACAP-ARAP_13.05.2021_00h00	4.34E-07	11.065007	160	[ABI1, ACTB, ACTG1, ACTR2, ADAM10, ADARB1, ADD3, AGRN, AKAP12, ANK3, APBB2, APEX1, APP, ARFGAP1, ARPC5L, ARRB1, ATP1A1, ATP2B1, ATP2B2, BAIAP2, BCR, BIN1, BLVRB, C1QBP, CANX, CAP2, CASK, CDH11, CDK16, CLU, CNGB1, CNN3, CORO1C, CTBP1, CTNNB1, DAGLB, DHX34, DNM1L, DNMBP, DPYSL2, DPYSL3, EFNB2, EIF2S1, EIF3A, EIF3B, EIF6, ETFDH, FARP1, FLRT2, FMR1, GIPC1, GLUL, GOLT1B, GOT1, GPC1, GPC6, GPHN, GSK3B, HDAC4, HIP1, HNRNPA3, HNRNPR, IGF2BP1, INPP4A, ITGA3, ITGB1, ITSN1, KCNMA1, KCTD12, LAMA5, LAMC1, LAMP1, LIN7C, LLGL1, MAP1B, MAP1LC3A, MFF, MME, MPST, MYD88, MYH9, NCDN, NDRG1, NEDD4, NQO1, OBSL1, PAFAH1B1, PALLD, PDZD11, PDZRN3, PIN1, PKN2, PLCB3, PLCG1, PLD1, PPFIBP1, PPP1CA, PPP3CA, PRKCI, PRKCSH, PSMA3, PSMC2, RAB10, RAB11B, RAB3B, RAB3D, RAB5A, RAB8A, RAC3, RGS10, RHEB, RIPK2, RNF40, ROR1, RPL10A, RPL18A, RPL22, RPL24, RPL38, RPL5, RPS13, RPS18, RPS21, RPS23, RPS9, RTN4, SARM1, SCRIB, SEPTIN11, SEPTIN2, SEPTIN8, SEPTIN9, SERPINB8,	No change

					SERPINE2, SH3KBP1, SLC16A3, SLC17A5, SLC1A1, SLC1A4, SLC2A1, SLC4A7, SPARC, SPART, SRC, STON1, STXBP3, SYNJ1, SYNPO, SYPL2, TLN2, TMED9, TNIK, TPBG, TPRG1L, TRAPPC4, TSC2, VDAC1, VDAC2, WFS1, YES1]	
pore complex assembly	GO_BiologicalProcess-EBI-UniProt-GOA-ACAP-ARAP_13.05.2021_00h00	1.49E-05	40.909092	9	[ADAM10, AFDN, AHCTF1, ANO6, CCT3, GSDMD, NUP205, PDZD11, RTN4]	Upregulation
mRNA 5'-UTR binding	GO_MolecularFunction-EBI-UniProt-GOA-ACAP-ARAP_13.05.2021_00h00	3.98E-06	37.931034	11	[DHX36, FMR1, IGF2BP1, IGF2BP2, LARP1, LARP6, MYH10, NCL, RPL5, RPS13, SHMT2]	Upregulation
perinuclear region of cytoplasm	GO_CellularComponent-EBI-UniProt-GOA-ACAP-ARAP_13.05.2021_00h00	3.38E-07	12.580231	98	[ACSL3, ADAM10, AKR1B1, ANKRD13A, APEX1, APP, ARFGEF1, ASPSCR1, ATP6V0A1, ATXN10, CCN2, CDC5L, CLIC1, CLU, CORO1B, CRBN, CTNNB1, DICER1, DNM1L, DOCK6, ECE1, EHD3, EIF2AK2, EML1, ERBB2, EZR, FAT1, FBXW8, FMR1, GALNT1, GALNT2, GAPDH, GOLGA1, HMOX1, HSP90AA1, HSP90B1, IGF2BP1, ITGA3, ITGB1, ITM2C, KIRREL1, KRT18, LAMP1, LIMS1, LRPPRC, M6PR, MAD2L1, MALT1, MAP1B, MAP3K4, MCM3, MGAT1, MSN, MVP, MYO1B, MYO6, NDRG1, NEDD4, NF2, NOCT, OBSL1, PAFAH1B1, PIK3CA, PKN2, PLD1, PLEC, PPIB, PRDX6, PRKCSH, PROCR, PTGES2, RAB10, RAB3B, RAC3, RANGAP1, SEC23B, SEPTIN2, SEPTIN9, SET, SLC11A2, SLC9A1, SNF8, SNX5, SORT1, SRC, STAT1, STOM, SYNJ1, TF, TGM2, THBS3, TMEM192, TRA2B, TSC2, TWF2, VPS33B, WDR44, WRNIP1]	No change
phospholipid binding	GO_MolecularFunction-EBI-UniProt-GOA-ACAP-ARAP_13.05.2021_00h00	3.15E-06	13.496933	66	[ANKFY1, ANXA3, ANXA5, ANXA7, APLP2, APPL2, ARFIP1, BIN1, C2CD2L, CHMP3, CPNE2, ESYT1,	No change

					FERMT2, FLII, GLE1, GSDMD, HIP1, JAG1, KCNQ1, LANCL2, MAP1B, MAP1LC3A, MICALL1, MME, MTCH1, MYO1B, MYO1E, MYOF, NCAPG, NF1, NISCH, NUP35, NUP62, OSBPL10, OSBPL1A, OSBPL2, OSBPL8, PACSIN3, PEBP1, PIGU, PIK3C2A, PLA2G15, PLA2G4A, PLD1, PRKCI, PRKCSH, PTGES2, PXK, SCARB2, SEC14L2, SESTD1, SLC9A1, SNX1, SNX18, SNX21, SNX27, SNX30, SNX5, SVIL, THBS1, TPP1, TWF2, VDAC1, VDAC2, VPS36, WIPI2]	
nervous system process	GO_BiologicalProcess-EBI-UniProt-GOA-ACAP-ARAP_13.05.2021_00h00	3.03E-06	4.684572	75	[AARS1, ABAT, ACTR2, ADARB1, ADNP, ANK3, APP, ATP2B2, B2M, BAIAP2, CASP3, CEMIP, CHD7, CNGB1, COL11A1, COL18A1, CRYZ, CTSC, CXCL12, DHX34, DIAPH1, DICER1, EFEMP1, FEN1, FMR1, GPI, GSK3B, GSTM2, HEXB, ITGA3, ITGB1, JAM3, KCNQ1, LAMC1, LLGL1, LRCH4, LUM, MME, MYO6, NF1, NSUN5, NUDT19, OAT, PAFAH1B1, POMK, PPIP5K2, PPP3CA, RAC3, REEP2, REEP6, RIC8A, RIPK2, ROR1, RPL38, SERPINE2, SLC11A2, SLC1A1, SLC1A4, SLC25A12, SLC9A1, SOD1, SOD2, SPART, SRC, SYNJ1, TGFBI, TJP1, TPBG, TPP1, TYMP, VDAC1, VLDLR, WDR1, WFS1, ZMPSTE24]	No change
NAD binding	GO_MolecularFunction-EBI-UniProt-GOA-ACAP-ARAP_13.05.2021_00h00	9.40E-09	31.343283	21	[ALDH2, BDH2, CRYZ, CTBP1, CYB5R3, FAM3A, GAPDH, GLUD1, GLYR1, GPD1L, HADH, HADHA, IDH2, LDHA, ME1, NNT, PARP1, PHGDH, QDPR, SIRT1, ZC3HAV1]	Upregulation
RNA polymerase binding	GO_MolecularFunction-EBI-UniProt-GOA-ACAP-ARAP_13.05.2021_00h00	9.25E-06	25.806452	16	[AGO2, BIN1, CDC73, ELP4, ERBB2, ERCC5, NEDD4, PKN2, PPIB, RPRD1A, RPRD1B, RPRD2, SMYD3, STOM, TRAPPC8, URI1]	Upregulation

organelle assembly	GO_BiologicalProcess-EBI-UniProt-GOA-ACAP-ARAP_13.05.2021_00h00	8.20E-06	11.334002	113	[ABT1, ACTG1, ACTR3, APEX1, ASPM, ATG7, ATXN10, AUP1, C1QBP, CDK1, CDK2, CETN2, CFAP20, CFAP298, CFL2, CHMP3, CIRBP, CNGB1, CNOT1, CSRP2, DRG1, DYNLL1, EFL1, EHD3, EIF2S1, EIF6, EML3, EXOC2, EZR, FLII, FSCN1, HGS, HSP90AA1, HSPB11, IFT140, IST1, ITGB1, KPNB1, KRT19, KRT8, LAMA5, LMOD1, MAP1LC3A, MLH1, MRTO4, MSN, NCAPG, NDRG1, NECTIN2, NEDD1, NEK1, NLE1, NOCT, NOP53, NPM1, NUDT19, NUP62, OBSL1, OGFOD1, PAFAH1B1, PDCD6IP, PIP4K2B, POGZ, PRKAA1, PTGES2, RAB1A, RAB23, RAB5A, RAB8A, RCC1, RDX, RPF2, RPL24, RPL38, RPL5, RPS23, RPS5, RTTN, SBDS, SCFD1, SEPTIN2, SEPTIN7, SEPTIN9, SMAD4, SMC1A, SMC3, SNF8, SRC, STAG2, SYNE2, SYNPO, TBC1D10A, TBC1D17, TBC1D22B, TBPL1, TPX2, TRAPPC8, TTC39C, TUBA4A, TUBB, TUBGCP2, UBXN2A, UNC119B, VPS25, VPS36, WASHC5, WDR1, WDR11, WDR35, WIPI2, WRAP73, YAP1, ZNF207]	Upregulation
protein localization to vacuole	GO_BiologicalProcess-EBI-UniProt-GOA-ACAP-ARAP_13.05.2021_00h00	1.18E-05	24.285715	17	[CD81, CLU, GBP1, GCC2, GLMP, HGS, M6PR, MEAK7, NEDD4, RTN4, SCARB2, SH3BP4, SNF8, SORT1, VPS25, VPS36, VPS41]	No change
nucleolus	GO_CellularComponent-EBI-UniProt-GOA-ACAP-ARAP_13.05.2021_00h00	2.41E-14	14.202899	147	[ABT1, ACACA, ACADVL, ADARB1, AGPS, AK3, AK6, APEX1, ARFGEF1, ARL14EP, BCAS2, BMS1, C1QBP, CASK, CBX5, CC2D1A, CDK5RAP3, CHD7, CHTOP, CMPK1, CNGB1, COG7, COIL, CSTB, DAB2, DDX47, DIMT1, DNTTIP2, EDF1, EEF1E1, EIF3A, EIF6, ELP3, EMG1, EZR, FBLIM1, FEN1, FMR1, GCFC2, GEMIN4, GET3,	No change

					GLE1, GNL3L, GOLGA3, GRWD1, GTF3C3, H1-10, HERC4, HIRIP3, HMOX1, HNRNPR, HSPB6, ILF3, IPO5, KAT7, KRT18, LARP4B, LIPA, LRWD1, MACROH2A1, MAGED2, MAGI1, MALT1, MCM2, MCM3, MCM7, MEAK7, MPHOSPH8, MRPS31, MRTO4, MTREX, MYO1E, NCAPG, NCL, NEDD4, NF1, NF2, NLE1, NMD3, NOL11, NOM1, NOP53, NPM1, NSUN5, NUB1, NUDT16, ORC4, PAFAH1B2, PARP1, PML, POLR1C, POLR1D, POLR2H, POLR2I, POP1, PPM1B, PPP1CA, PRMT7, PSPC1, PYCARD, RABGEF1, RAI14, RBM4, RCN2, RNF213, RPF2, RPL13, RPL13A, RPL27, RPL36, RPL5, RPS13, RPS9, S100A16, SBDS, SCAF11, SELENBP1, SENP3, SIRT1, SMARCB1, SMC2, SNU13, SPATS2L, SPECC1, SRSF5, SSRP1, STAT1, STK24, STON1, TBCA, TGS1, TMA16, TRA2A, TSEN15, TSEN34, TSPYL1, TUT4, UBTF, URB2, UTP14A, UTP20, UTP3, VRK1, VRK3, WDR12, XPO6, ZNF207]	
transcription regulator activity	GO_BiologicalProcess-EBI-UniProt-GOA-ACAP-ARAP_13.05.2021_00h00	6.12E-09	4.6632123	108	[AASS, ABT1, ADNP, AKR1B1, ANXA3, APEX1, APP, ARID1B, ARRB1, C1QBP, CC2D1A, CCDC22, CD200, CDC5L, CDK5RAP3, CHORDC1, CHP1, CLU, CTBP1, CTNNB1, CUX1, DDX1, DVL2, EDF1, EIF2AK2, ENPP1, ENY2, FGF2, FHL2, GARS1, GTF2I, HDAC2, HDAC4, HDGFL2, HMOX1, HSD17B4, IKBKB, IKBKG, KAT7, KAT8, KDM1A, KDM3B, LMCD1, LRRFIP1, MALT1, MED17, MED23, MED30, MGA, MTA1, MYD88, NCOA5,	Upregulation

					NCOA7, NFKB2, NIBAN2, NPM1, OXSR1, PARP14, PATZ1, PAWR, PBXIP1, PIR, PLPP3, PPP3CA, PRKCI, PSIP1, PTGIS, PXN, PYCARD, RAP2C, RIPK2, RNF2, ROR1, RYBP, SCAI, SF1, SIRT1, SLC30A9, SMAD4, SMARCB1, SMARCC1, SMARCD3, SMARCE1, SNW1, SRA1, SRSF2, STAT1, STAT2, STAT5B, SUFU, TAB2, TAF6L, TAX1BP1, TDRD3, TFAM, TRADD, TRIM21, TRIM25, TRIM28, TRIP13, UBA2, URI1, USP22, USP7, WFS1, WWP2, YAP1, ZNF148]	
cytoplasm	GO_CellularComponent-EBI-UniProt-GOA-ACAP-ARAP_13.05.2021_00h00	1.43E-91	10.32274	1257	[AARS1, AASDHPPT, AASS, ABAT, ABCB7, ABCE1, ABHD12, ABI1, ACACA, ACAD8, ACADM, ACADSB, ACADVL, ACAP2, ACAT2, ACO1, ACOT7, ACOT8, ACOX1, ACSF2, ACSL3, ACSL4, ACSS2, ACTB, ACTG1, ACTR2, ACTR3, ADAM10, ADAMTS9, ADARB1, ADD3, ADK, ADNP, ADSL, AFDN, AGO2, AGPAT2, AGPS, AGRN, AHCTF1, AK1, AK3, AK4, AK6, AKAP12, AKR1B1, ALB, ALDH18A1, ALDH2, ALDH3A2, ALDOA, ALG11, AMOTL1, ANK3, ANKFY1, ANKRD13A, ANKZF1, ANO6, ANXA3, ANXA5, ANXA7, AP3M2, AP5B1, APAF1, APBB2, APEX1, APIP, APLP2, APP, APPL2, APRT, ARAF, ARFGAP1, ARFGEF1, ARFIP1, ARHGAP12, ARHGAP17, ARHGAP5, ARHGEF11, ARHGEF12, ARID1B, ARL14EP, ARPC4, ARPC5L, ARPP19, ARRB1, ASAH1, ASAP2, ASB1, ASNS, ASPM, ASPSCR1, ATE1, ATG4B, ATG7, ATL3, ATP1A1, ATP1B3, ATP2B1, ATP2B2, ATP2C1, ATP6V0A1, ATP6V0C, ATP6V1A, ATP6V1B2, ATPAF1,	No change

					ATXN10, AUP1, B2M, B3GAT3, B4GALT1, B4GALT5, BAG1, BAIAP2, BANF1, BCAT1, BCR, BDH2, BET1, BIN1, BLVRB, BMP1, BNIP1, BOD1, BPNT2, BST1, BZW1, C1GALT1, C1QBP, C1orf198, C2CD2L, CAB39L, CALD1, CAMK2D, CANX, CAP1, CAP2, CAPN5, CAPZA2, CASK, CASP3, CASP6, CBR1, CC2D1A, CCDC22, CCN2, CCT2, CCT3, CCT6A, CD109, CDC34, CDC42BPA, CDC42EP1, CDC5L, CDC73, CDH11, CDIPT, CDK1, CDK16, CDK17, CDK2, CDK5RAP3, CDV3, CELF2, CEMIP, CENPV, CERS6, CETN2, CFAP20, CFAP298, CFL2, CHAF1B, CHAMP1, CHD1L, CHD4, CHID1, CHMP3, CHORDC1, CHP1, CHPF, CHSY3, CHTOP, CIP2A, CIRBP, CKB, CLGN, CLIC1, CLIP2, CLTC, CLTCL1, CLU, CMPK1, CMTR1, CNGB1, CNN3, CNOT1, CNOT10, COG6, COG7, COL11A1, COL12A1, COL15A1, COL18A1, COL4A1, COL5A1, COL5A2, COL6A1, COLEC12, COMMD4, COQ5, CORO1B, CORO1C, CORO2B, CORO7, COX20, COX4I1, COX6C, CPNE2, CRABP2, CRBN, CROT, CRTAP, CRYZ, CRYZL1, CSPG4, CSRP2, CSTB, CTBP1, CTNNB1, CTNNBL1, CTPS1, CTSA, CTSC, CTU1, CUL1, CUL4B, CUX1, CWC22, CXCL12, CYB5R3, CYCS, CYP51A1, CYRIB, DAB2, DAGLB, DAPK1, DARS1, DCN, DCPS, DCXR, DDI2, DDX1, DDX42, DENND2A, DERL1, DGLUCY, DGUOK, DHCR24, DHCR7, DHFR, DHPS, DHRS7B, DHX34, DHX36, DIAPH1, DIAPH3, DICER1, DIMT1, DIPK2A, DLST, DMXL1,	

					DNM1L, DNMBP, DOCK6, DPP3, DPP9, DPYSL2, DPYSL3, DPYSL4, DRG1, DSP, DST, DUSP23, DUSP3, DUT, DVL2, DYM, DYNC1LI1, DYNLL1, EARS2, ECE1, ECI2, EDF1, EED, EEF1E1, EEF1G, EHD3, EIF2AK2, EIF2B1, EIF2D, EIF2S1, EIF3A, EIF3B, EIF3E, EIF3F, EIF4G3, EIF6, ELP3, ELP4, ELP5, EMC1, EMG1, EML1, EML3, EML4, ENO2, ENPP1, ENY2, EOGT, EPB41L2, EPB41L3, EPHA3, EPHB4, EPHX1, EPM2AIP1, EPPK1, EPRS1, ERAP1, ERBB2, ERCC6L, ERLIN2, ERP44, ESYT1, ETFDH, ETHE1, EXD2, EXOC2, EXTL2, EZR, FAHD1, FAM120A, FAM3A, FAM91A1, FANCI, FARP1, FASN, FAT1, FBLIM1, FBXL12, FBXW8, FDXR, FEN1, FERMT1, FERMT2, FGD4, FGF2, FHL1, FHL2, FHOD1, FIBP, FKBP1A, FKBP5, FKBP9, FLII, FLRT2, FMR1, FN1, FN3K, FNBP1L, FSCN1, FSD1, FTH1, FTL, FTO, FUT11, FUT8, G6PD, GAB1, GALE, GALM, GALNT1, GALNT2, GALNT7, GAMT, GAN, GAPDH, GARS1, GATB, GBA2, GBE1, GBP1, GCAT, GCC2, GCDH, GCFC2, GDAP1, GDAP2, GDPGP1, GEMIN4, GET3, GFPT2, GGA2, GGCT, GIPC1, GIPC2, GLA, GLE1, GLMN, GLMP, GLO1, GLRX3, GLUD1, GLUL, GLYR1, GMPS, GNB4, GNE, GNL3L, GOLGA1, GOLGA3, GOLGA7, GOLT1B, GOT1, GPC1, GPC6, GPD1L, GPD2, GPHN, GPI, GPN1, GPR108, GPSM1, GRAMD1A, GRWD1, GSDMD, GSK3B, GSTCD, GSTM2, GSTM3, GSTP1, GTF2E1, GTF2I, GTF3C4, GULP1, GYS1, HACL1, HADH, HADHA, HARS1, HAT1, HDAC2, HDAC4, HEBP1,	

					HELZ, HERC2, HERC4, HEXB, HGS, HHIP, HIBCH, HINT1, HIP1, HK1, HK2, HLA-A, HLA-B, HLA-C, HMBS, HMGCS1, HMOX1, HNRNPA3, HNRNPC, HNRNPH1, HNRNPR, HPX, HSD17B10, HSD17B11, HSD17B4, HSDL2, HSP90AA1, HSP90B1, HSPA14, HSPA4, HSPA5, HSPB6, HSPE1, HSPG2, IARS2, IDH2, IDI1, IFIT5, IFT140, IGF2BP1, IGF2BP2, IGFBP7, IKBKB, IKBKG, ILF3, ILKAP, ILVBL, IMPA1, IMPACT, INPP4A, INTS9, IPO5, IPO9, IQGAP3, IREB2, ISOC1, IST1, ITGA3, ITGAV, ITGB1, ITGB5, ITIH4, ITM2C, ITPK1, ITSN1, IVNS1ABP, JAGN1, JAM3, KAT7, KCNQ1, KDM4B, KDSR, KIF15, KIRREL1, KNTC1, KPNB1, KRT18, KRT19, KRT8, LACTB, LAMC1, LAMP1, LANCL2, LAP3, LARP1, LARP4B, LARP6, LBR, LDHA, LEMD2, LGALS3BP, LIG1, LIG3, LIMS1, LIN7C, LIPA, LLGL1, LMCD1, LMO7, LMOD1, LNPEP, LPCAT2, LPCAT4, LPP, LRCH3, LRPPRC, LRRC1, LRRC8A, LRRC8E, LRRFIP1, LRRK1, LRWD1, LSS, LTBP1, LUM, LYPLA2, M6PR, MACF1, MAD1L1, MAD2L1, MAEA, MAGED2, MAGI1, MALT1, MAN2B2, MAOA, MAP1B, MAP1LC3A, MAP2K3, MAP3K20, MAP3K4, MAP4K4, MARCHF5, MAT2A, MBD3, MBOAT2, MBOAT7, MCM2, MCM3, MCM5, MCM7, MCMBP, ME1, MEAK7, MEST, MFF, MFN1, MGAT1, MGST1, MICAL3, MICALL1, MLF2, MMAB, MME, MMUT, MOCS2, MOGS, MOV10, MPHOSPH8, MPST, MRM3, MRPL10, MRPS17, MRPS21, MRPS31, MRRF, MRTO4, MSH6,	

					MSI2, MSN, MTA1, MTAP, MTCH1, MTHFD2, MTR, MTRR, MVD, MVK, MVP, MYADM, MYD88, MYH10, MYH9, MYO18A, MYO1B, MYO1D, MYO1E, MYO6, MYOF, N6AMT1, NCAPD2, NCAPG, NCAPH, NCDN, NCK2, NCKAP1, NCL, NCLN, NDRG1, NDUFA13, NDUFA4, NDUFAF7, NDUFS3, NEDD1, NEDD4, NEDD8, NEK1, NENF, NES, NF1, NF2, NFATC2IP, NFKB2, NHLRC2, NIBAN1, NIBAN2, NISCH, NMD3, NMT1, NNT, NOCT, NOP53, NPLOC4, NPM1, NQO1, NSDHL, NSMCE3, NT5C, NT5C3B, NUB1, NUBP2, NUBPL, NUCB2, NUDT15, NUDT16, NUDT19, NUF2, NUP205, NUP35, NUP62, NUTF2, NXN, OAT, OBSL1, OGFOD1, OPLAH, ORC4, ORC5, OSBPL10, OSBPL11, OSBPL1A, OSBPL2, OSBPL8, OTUD6B, OXA1L, OXSR1, P3H1, P3H3, PACS1, PACSIN3, PAFAH1B1, PAFAH1B2, PAFAH1B3, PAICS, PAIP1, PALLD, PANK4, PANX1, PAPSS2, PARG, PARL, PARP1, PARP14, PARP4, PAWR, PBXIP1, PC, PCBP3, PCDH7, PCK2, PCYT2, PDAP1, PDCD6IP, PDE5A, PDK1, PDS5B, PDXDC1, PDZD11, PDZRN3, PEBP1, PELO, PFAS, PFKM, PGAM5, PGGHG, PGM2L1, PGM3, PGP, PHGDH, PHKB, PHLDB1, PHPT1, PI4K2B, PIEZO1, PIGO, PIGT, PIGU, PIK3C2A, PIK3CA, PIN1, PIP4K2B, PIR, PITHD1, PITRM1, PKLR, PKM, PKN2, PLA2G15, PLA2G4A, PLBD2, PLCB3, PLCG1, PLD1, PLD3, PLEC, PLOD2, PLPBP, PLPP3, PML, PMM2, PNP, PNPLA4, PNPLA6, POFUT1, POGLUT3, POGZ,	

					POLD1, POLR1C, POLR1D, POLR2A, POLR2H, POLR3A, POLR3C, POMGNT1, POMGNT2, POMK, PPFIBP1, PPIB, PPIE, PPIG, PPIP5K2, PPM1B, PPP1CA, PPP1R14A, PPP1R7, PPP2R5A, PPP3CA, PPP4R3A, PPP5C, PRDX1, PRDX6, PRKAA1, PRKAB2, PRKCI, PRKCSH, PRKG1, PRMT7, PROCR, PRPF19, PRPSAP1, PRPSAP2, PRUNE1, PRXL2B, PSAT1, PSIP1, PSMA3, PSMB10, PSMB2, PSMC2, PSMC3, PSMC6, PSMD1, PSMD12, PSMD14, PSMD2, PSMD6, PSMD7, PSMD8, PSME1, PSME2, PSME4, PSMG1, PSPC1, PTGES2, PTGIS, PTGR1, PTPA, PTPMT1, PTPN12, PTRH2, PXK, PXN, PYCARD, PYCR1, PYCR3, PYGB, QARS1, QDPR, QSOX1, QTRT1, QTRT2, RAB10, RAB11B, RAB1A, RAB23, RAB3B, RAB3D, RAB5A, RAB8A, RABGEF1, RABL6, RAC3, RAD21, RAI14, RALA, RANBP1, RANGAP1, RAP1GDS1, RAP2B, RAP2C, RARS2, RBBP4, RBM22, RBM4, RBMS2, RBP1, RCC1, RCN1, RCN2, RCN3, RDH10, RDH11, RDX, RECQL, REEP2, REEP6, RELCH, RETSAT, RGS10, RHEB, RHOB, RHOC, RIC8A, RIC8B, RIF1, RIN1, RIOK3, RIPK2, RNF114, RNF2, RNF213, RNF40, RNPS1, ROR1, RPL10A, RPL13, RPL13A, RPL18A, RPL22, RPL24, RPL27, RPL32, RPL36, RPL38, RPL5, RPLP1, RPLP2, RPS13, RPS15A, RPS16, RPS18, RPS21, RPS23, RPS4Y1, RPS5, RPS8, RPS9, RRAS2, RRM1, RSPRY1, RTN4, RTTN, RYBP, S100A10, S100A16, SACS, SAE1, SARM1,	

					SARNP, SARS1, SBDS, SCAI, SCARB2, SCFD1, SCLY, SCP2, SCPEP1, SCRIB, SCRN1, SDHC, SEC11C, SEC14L2, SEC23B, SEC61A1, SEC61B, SELENBP1, SELENOT, SENP3, SEPTIN10, SEPTIN11, SEPTIN2, SEPTIN7, SEPTIN8, SEPTIN9, SERPINB1, SERPINB2, SERPINB8, SERPINB9, SERPINE2, SESN2, SET, SEZ6L2, SFXN2, SFXN3, SGSH, SH3BGRL3, SH3BP4, SH3KBP1, SHFL, SHMT2, SIAE, SIRT1, SLC11A2, SLC12A2, SLC17A5, SLC1A1, SLC1A4, SLC1A5, SLC25A10, SLC25A12, SLC25A13, SLC25A15, SLC25A19, SLC25A22, SLC25A24, SLC25A26, SLC25A4, SLC25A5, SLC25A6, SLC2A1, SLC30A9, SLC31A1, SLC44A1, SLC44A2, SLC4A7, SLC9A1, SLIT2, SMAD4, SMAP2, SMARCC1, SMC1A, SMC2, SMC3, SMC4, SMNDC1, SMPD1, SMU1, SMYD3, SNF8, SNRPD3, SNRPE, SNW1, SNX1, SNX18, SNX21, SNX27, SNX5, SOAT1, SOD1, SOD2, SORBS3, SORT1, SPARC, SPART, SPATS2, SPATS2L, SPECC1, SPHK2, SPTLC1, SPTLC2, SQSTM1, SRA1, SRC, SRD5A3, SRPRA, SRR, SRSF10, SRSF2, SRSF5, SRXN1, SSB, SSR3, STAG2, STARD5, STAT1, STAT2, STAT5B, STK24, STOM, STON1, STRN3, STXBP3, SUFU, SUGT1, SULT1E1, SURF1, SVIL, SYNE2, SYNJ1, SYNPO, SYPL2, TAB2, TAGLN, TAGLN2, TALDO1, TAP1, TAX1BP1, TBC1D10A, TBC1D17, TBC1D4, TBC1D8B, TBCA, TBCE, TBCEL, TBK1, TBPL1, TCP1, TDRD3, TF, TFAM, TGFBI,	

					TGFBR3, TGM2, TGS1, THADA, THBS1, THBS3, THEM4, TIGAR, TIMM17B, TIMMDC1, TIPRL, TJP1, TJP2, TKT, TLN2, TM9SF4, TMED9, TMEM119, TMEM168, TMEM192, TMEM65, TMEM97, TMX3, TNIK, TNPO2, TNS3, TOM1, TOM1L2, TOMM34, TOMM7, TOR1AIP2, TOR1B, TPBG, TPI1, TPM3, TPMT, TPP1, TPP2, TPRG1L, TPST2, TPX2, TRA2B, TRADD, TRAM1, TRAM2, TRAPPC4, TRAPPC8, TRIM21, TRIM25, TRIM56, TRIM59, TRIO, TRIP12, TRIP6, TRMT10C, TRMT5, TSC2, TSPO, TTC1, TTC28, TTC5, TTLL12, TUBA4A, TUBA8, TUBB, TUBB3, TUBGCP2, TUT4, TWF2, TXN, TXNDC17, TYMP, UACA, UAP1, UBA2, UBA3, UBAC2, UBE2L6, UBE2Q1, UBR2, UBR4, UBXN2A, UFD1, UGP2, UMPS, UNC119B, UNC93B1, UQCR10, UQCR11, UQCRC2, URI1, URM1, USP11, USP28, USP4, USP48, USP5, USP7, USP9X, USP9Y, UST, UTP14A, UTP20, VARS1, VASP, VAT1, VDAC1, VDAC2, VLDLR, VPS25, VPS26B, VPS33B, VPS36, VPS41, VRK1, VRK3, VTN, VWA8, WASHC5, WDR1, WDR11, WDR35, WDR41, WDR44, WFS1, WIPI2, WRAP73, WRNIP1, WWC2, WWP2, XIAP, XPNPEP3, XPO4, XPO6, XPOT, XYLB, XYLT2, YAP1, YARS1, YBX1, YES1, YKT6, YOD1, YRDC, ZC3HAV1, ZCCHC3, ZDHHC13, ZDHHC21, ZFAND6, ZFP36L2, ZMPSTE24, ZNF148, ZNF207]	
nuclear protein-containing complex	GO_CellularComponent-EBI-UniProt-GOA-ACAP-	1.70E-16	13.949843	178	[AAR2, ACTB, ADNP, AHCTF1, AMOTL1, AQR, ARFGEF1, ARID1B, ASH2L, BCAS2, CBX2, CBX3,	Upregulation

	ARAP_13.05.2021_00h00				CBX5, CBX8, CCNH, CCNK, CDC5L, CDC73, CECR2, CETN2, CHD4, CHTOP, CIRBP, CNGB1, COLEC12, CPSF2, CTNNB1, CTNNBL1, CWC22, DICER1, DYNLL1, EED, ELP4, ENY2, EPB41L2, ERCC5, GARS1, GATAD2A, GCFC2, GEMIN4, GLE1, GNL3L, GPKOW, GTF2E1, GTF2F1, GTF2F2, HDAC2, HDAC4, HINT1, HNRNPA3, HNRNPC, HNRNPH1, HNRNPR, HSPA5, HTATSF1, INTS1, INTS9, IPO5, IVNS1ABP, KAT7, KAT8, KDM3B, KPNB1, LIG3, LRWD1, MAD1L1, MAD2L1, MAGI1, MBD3, MCM2, MCM3, MCM4, MCM5, MCM6, MCM7, MED17, MED23, MED30, MGA, MLH1, MORF4L1, MSH2, MSH6, MTA1, MTREX, MVP, MYH9, MYO1E, MYO6, NCL, NELFCD, NOL11, NUP205, NUP35, NUP62, NUTF2, ORC4, ORC5, PLCG1, POLD1, POLD2, POLE2, POLR1C, POLR1D, POLR2A, POLR2B, POLR2H, POLR2I, POLR3A, POLR3C, POP1, PPIE, PPWD1, PRIM2, PRPF19, PSMD12, RAD21, RAD9A, RANBP1, RANGAP1, RBBP4, RBM22, RHEB, RNF2, RNPS1, RPA1, RPA2, RPRD1A, RPRD1B, RPRD2, RYBP, SAE1, SARNP, SENP3, SF1, SF3A3, SF3B6, SHMT2, SIRT1, SLC25A15, SMAD4, SMARCA1, SMARCB1, SMARCC1, SMARCD3, SMARCE1, SMC3, SMNDC1, SMU1, SNRNP200, SNRPA, SNRPD3, SNRPE, SNU13, SNW1, SRSF2, SSRP1, STON1, SUPT16H, SYNE2, TAF6L, TBPL1, TDRD3, TGS1, THOC2, TRA2A, TRA2B, TRIM28,	

					TSEN34, UBA2, USP22, WDR12, WDR5, XPO4, XPOT, YAP1, YBX1, YEATS4]	
peroxisome	GO_CellularComponent-EBI-UniProt-GOA-ACAP-ARAP_13.05.2021_00h00	1.81E-05	17.948717	28	[ACOT8, ACOX1, ACSL3, ACSL4, AGPS, ALDH3A2, CROT, DHRS7B, DNM1L, ECI2, GDAP1, HACL1, HSD17B4, HSDL2, IDH2, IDI1, ISOC1, MFF, MGST1, MVD, MVK, NUDT19, PTGES2, SCP2, SOD1, TKT, TTC1, VWA8]	Downregulation
magnesium ion binding	GO_MolecularFunction-EBI-UniProt-GOA-ACAP-ARAP_13.05.2021_00h00	1.57E-05	15.74468	37	[AASDHPPT, CDC42BPA, DHX36, DUT, ENO2, EXD2, FEN1, FSCN1, GLUL, GPI, HACL1, IDH2, ILVBL, IMPA1, ITPK1, MAP3K20, MSH2, MSH6, MTHFD2, MVK, NT5C3B, NUDT16, NUDT19, OXSR1, PGM3, PGP, PKLR, PKM, PPM1B, PRPSAP1, PRPSAP2, RHEB, SRR, STK24, TKT, UBA2, XYLT2]	No change
cytosol	GO_CellularComponent-EBI-UniProt-GOA-ACAP-ARAP_13.05.2021_00h00	1.80E-83	13.566584	760	[AARS1, AASDHPPT, AASS, ABCE1, ABI1, ACACA, ACAT2, ACO1, ACOT7, ACOT8, ACOX1, ACSS2, ACTB, ACTG1, ACTR2, ACTR3, ADARB1, ADD3, ADK, ADSL, AFDN, AGO2, AGPS, AHCTF1, AK1, AK3, AK6, AKAP12, AKR1B1, ALDH3A2, ALDOA, AMOTL1, ANK3, ANKFY1, ANO6, ANXA5, APAF1, APEX1, APIP, APP, APRT, ARAF, ARFGAP1, ARFGEF1, ARHGAP12, ARHGAP17, ARHGAP5, ARHGEF11, ARHGEF12, ARID1B, ARL14EP, ARPC4, ARRB1, ASB1, ASNS, ASPSCR1, ATG4B, ATG7, ATP6V0A1, ATP6V1A, ATP6V1B2, ATXN10, B2M, BAG1, BAIAP2, BANF1, BCAT1, BCR, BDH2, BIN1, BLVRB, BPNT2, C1QBP, C1orf198, CAB39L, CALD1, CAMK2D, CAPZA2, CASK, CASP3, CASP6, CBR1, CC2D1A, CCDC22, CCN2, CCT2, CCT3, CCT6A, CD109, CDC34, CDC73, CDK1, CDK16, CDK2, CDK5RAP3, CDV3, CENPV, CETN2,	No change

					CFAP298, CHAF1B, CHD1L, CHMP3, CHORDC1, CHP1, CHPF, CIP2A, CKB, CLTC, CLTCL1, CLU, CMPK1, CNN3, CNOT1, CNOT10, COLEC12, COMMD4, CORO1B, CORO7, COX4I1, CRABP2, CRBN, CROT, CRYZ, CRYZL1, CSTB, CTNNB1, CTNNBL1, CTPS1, CTU1, CUL1, CUL4B, CUX1, CWC22, CYB5R3, CYCS, DAB2, DARS1, DCPS, DDI2, DDX1, DDX42, DENND2A, DGUOK, DHCR24, DHFR, DHPS, DHX34, DHX36, DIAPH1, DIAPH3, DICER1, DIMT1, DLST, DNM1L, DOCK6, DPP3, DPP9, DPYSL2, DPYSL3, DPYSL4, DRG1, DST, DUSP23, DUSP3, DVL2, DYNC1LI1, DYNLL1, ECI2, EDF1, EED, EEF1E1, EEF1G, EHD3, EIF2AK2, EIF2B1, EIF2D, EIF2S1, EIF3A, EIF3B, EIF3E, EIF3F, EIF4G3, EIF6, ELP3, ELP5, EML1, EML4, ENO2, EPB41L2, EPB41L3, EPHA3, EPHB4, EPRS1, ERAP1, ERBB2, ERCC6L, ERLIN2, ETFDH, EXOC2, EXTL2, EZR, FAHD1, FAM120A, FANCI, FARP1, FASN, FBLIM1, FBXL12, FBXW8, FERMT1, FERMT2, FGD4, FHL1, FHOD1, FKBP1A, FKBP5, FLII, FMR1, FN3K, FNBP1L, FSCN1, FTH1, FTL, FTO, G6PD, GAB1, GALE, GAMT, GAN, GAPDH, GARS1, GBA2, GBE1, GBP1, GCC2, GCFC2, GDAP1, GEMIN4, GFPT2, GGCT, GIPC1, GLE1, GLMP, GLO1, GLRX3, GLUL, GLYR1, GMPS, GNB4, GNE, GNL3L, GOLGA1, GOLGA3, GOLT1B, GOT1, GPC1, GPD1L, GPHN, GPI, GPN1, GPSM1, GRAMD1A, GRWD1, GSDMD, GSK3B, GSTM2, GSTM3, GSTP1, GTF2E1, GYS1, HACL1, HARS1, HDAC4, HELZ, HERC2, HERC4, HGS,	

					HINT1, HIP1, HK1, HK2, HMBS, HMGCS1, HMOX1, HNRNPC, HNRNPH1, HSD17B11, HSD17B4, HSP90AA1, HSP90B1, HSPA14, HSPA4, HSPA5, HSPB6, IDH2, IDI1, IFIT5, IGF2BP1, IGF2BP2, IKBKB, IKBKG, ILF3, ILKAP, IMPA1, INPP4A, INTS9, IPO9, IQGAP3, IREB2, IST1, ITGAV, ITPK1, ITSN1, IVNS1ABP, KAT7, KDM4B, KIF15, KNTC1, KPNB1, KRT18, KRT19, KRT8, LACTB, LAMP1, LANCL2, LAP3, LARP4B, LDHA, LIMS1, LIPA, LLGL1, LMO7, LMOD1, LNPEP, LPP, LRCH3, LRRC1, LRRFIP1, LRRK1, LYPLA2, MAD1L1, MAD2L1, MAGED2, MAGI1, MALT1, MAOA, MAP1B, MAP1LC3A, MAP2K3, MAP3K20, MAT2A, MCM5, MCM7, MCMBP, ME1, MEAK7, MICAL3, MOCS2, MOV10, MPHOSPH8, MPST, MSH6, MSI2, MSN, MTA1, MTAP, MTR, MTRR, MVD, MVK, MVP, MYD88, MYH10, MYH9, MYO1D, MYO1E, MYO6, N6AMT1, NCAPD2, NCAPG, NCAPH, NCDN, NCK2, NCKAP1, NDRG1, NEDD1, NEDD4, NEDD8, NEK1, NF1, NF2, NFKB2, NHLRC2, NIBAN1, NIBAN2, NISCH, NMT1, NOP53, NPLOC4, NPM1, NQO1, NT5C, NT5C3B, NUB1, NUBP2, NUCB2, NUDT15, NUDT19, NUF2, NUP205, NUP35, NUTF2, NXN, OBSL1, OGFOD1, OPLAH, ORC4, ORC5, OSBPL10, OSBPL11, OSBPL1A, OSBPL2, OSBPL8, OXSR1, PACS1, PACSIN3, PAFAH1B1, PAFAH1B2, PAFAH1B3, PAICS, PAIP1, PALLD, PANK4, PAPSS2, PARG, PARP14,	

					PARP4, PBXIP1, PC, PCBP3, PCK2, PDAP1, PDCD6IP, PDE5A, PDS5B, PDZD11, PEBP1, PFAS, PFKM, PGGHG, PGM2L1, PGM3, PHGDH, PHKB, PHPT1, PI4K2B, PIGU, PIK3C2A, PIK3CA, PIN1, PIP4K2B, PIR, PKLR, PKM, PKN2, PLA2G4A, PLCB3, PLCG1, PLEC, PLPBP, PML, PMM2, PNP, PNPLA4, PNPLA6, POGZ, POLD1, POLR1C, POLR1D, POLR2H, POLR3A, POLR3C, PPFIBP1, PPIE, PPIG, PPIP5K2, PPM1B, PPP1CA, PPP1R14A, PPP2R5A, PPP3CA, PPP4R3A, PPP5C, PRDX1, PRDX6, PRKAA1, PRKAB2, PRKCI, PRKG1, PRMT7, PRUNE1, PRXL2B, PSAT1, PSIP1, PSMA3, PSMB10, PSMB2, PSMC2, PSMC3, PSMC6, PSMD1, PSMD12, PSMD14, PSMD2, PSMD6, PSMD7, PSMD8, PSME1, PSME2, PSME4, PSMG1, PTGES2, PTPMT1, PTPN12, PTRH2, PXK, PXN, PYCARD, PYCR3, QARS1, QDPR, RAB10, RAB11B, RAB1A, RAB23, RAB3B, RAB5A, RAB8A, RABGEF1, RABL6, RAC3, RAD21, RAI14, RANBP1, RANGAP1, RAP1GDS1, RAP2B, RAP2C, RBBP4, RBM4, RBMS2, RBP1, RGS10, RHEB, RHOB, RHOC, RIC8B, RIN1, RIOK3, RIPK2, RNF114, RNF2, RNF213, RNF40, RNPS1, RPL10A, RPL13, RPL13A, RPL18A, RPL22, RPL24, RPL27, RPL32, RPL36, RPL38, RPL5, RPLP1, RPLP2, RPS13, RPS15A, RPS16, RPS18, RPS21, RPS23, RPS4Y1, RPS5, RPS8, RPS9, RRM1, S100A16, SARM1, SARS1, SBDS, SCFD1, SCLY, SCP2, SCPEP1, SEC14L2, SEC23B,	

					SEC61B, SELENBP1, SEPTIN7, SERPINB8, SERPINB9, SERPINE2, SESN2, SET, SH3KBP1, SHFL, SHMT2, SIRT1, SLC12A2, SLC17A5, SLC25A15, SLC2A1, SLC44A1, SMAD4, SMC1A, SMC2, SMC3, SMC4, SMYD3, SNF8, SNRPD3, SNRPE, SNX1, SNX27, SNX5, SOD1, SORBS3, SORT1, SPART, SPATS2, SPATS2L, SPECC1, SPHK2, SQSTM1, SRA1, SRC, SRR, SRSF10, SRSF2, SRSF5, SRXN1, STAG2, STARD5, STAT1, STAT2, STAT5B, STK24, STXBP3, SUFU, SUGT1, SULT1E1, SVIL, SYNJ1, SYNPO, TAB2, TAGLN2, TALDO1, TAX1BP1, TBC1D10A, TBC1D17, TBC1D4, TBC1D8B, TBCA, TBK1, TCP1, TDRD3, TFAM, TGM2, TGS1, THADA, THEM4, TIGAR, TIPRL, TJP1, TJP2, TKT, TMED9, TNIK, TNS3, TOM1, TOMM34, TPI1, TPM3, TPMT, TPP2, TPX2, TRADD, TRAPPC4, TRAPPC8, TRIM21, TRIM25, TRIM56, TRIO, TRIP12, TRIP6, TSC2, TSPO, TTC1, TUBA4A, TUBB, TUBGCP2, TUT4, TXN, TXNDC17, TYMP, UACA, UAP1, UBA3, UBE2L6, UBE2Q1, UBR2, UBR4, UBXN2A, UFD1, UGP2, UMPS, UNC119B, URI1, URM1, USP11, USP28, USP4, USP48, USP5, USP7, USP9X, USP9Y, UTP14A, VARS1, VASP, VDAC2, VPS25, VPS26B, VPS33B, VPS36, VRK1, WASHC5, WDR1, WDR11, WDR44, WIPI2, WWC2, WWP2, XIAP, XPNPEP3, XPO4, XPO6, XPOT, XYLB, YAP1, YARS1, YBX1, YES1, YKT6, YOD1, ZC3HAV1, ZFAND6]	
cell cortex	GO_CellularComponent-EBI-	1.76E-08	16.717325	55	[ACTB, ACTR2, ADD3, AKAP12,	Upregulation

	UniProt-GOA-ACAP-ARAP_13.05.2021_00h00				C2CD2L, CALD1, CAP1, CAPZA2, CCN2, CHAF1B, CORO1C, CTBP1, CTNNB1, DST, DYNLL1, EPB41L2, EXOC2, EZR, FERMT2, FNBP1L, FSCN1, GIPC1, GLRX3, GPSM1, KRT19, LANCL2, LLGL1, MAEA, MICAL3, MYADM, MYH10, MYH9, MYO1D, MYO6, NCL, NEDD4, NF2, PAFAH1B1, PHLDB1, PXN, RAB10, RAC3, RAI14, RDX, RHOB, RHOC, RIC8B, SEPTIN10, SEPTIN11, SEPTIN2, SEPTIN7, SEPTIN8, SEPTIN9, SLC2A1, WDR1]	
C4-dicarboxylate transport	GO_BiologicalProcess-EBI-UniProt-GOA-ACAP-ARAP_13.05.2021_00h00	2.65E-06	39.285713	11	[ABAT, LRRC8A, LRRC8E, SLC12A2, SLC1A1, SLC1A4, SLC1A5, SLC25A10, SLC25A12, SLC25A13, SLC25A22]	No change
oxidoreductase activity, acting on the CH-CH group of donors	GO_MolecularFunction-EBI-UniProt-GOA-ACAP-ARAP_13.05.2021_00h00	4.02E-06	26.153847	17	[ACAD8, ACADM, ACADSB, ACADVL, ACOX1, BDH2, BLVRB, DHCR24, DHCR7, FASN, GCDH, LBR, PTGES2, PTGR1, RETSAT, SDHC, SRD5A3]	No change
pentosyltransferase activity	GO_MolecularFunction-EBI-UniProt-GOA-ACAP-ARAP_13.05.2021_00h00	9.25E-06	25.806452	16	[APRT, GXYLT1, GXYLT2, MTAP, PARP1, PARP14, PARP4, PNP, POGLUT3, QTRT1, QTRT2, SIRT1, TYMP, UMPS, XYLT2, ZC3HAV1]	No change
enzyme binding	GO_MolecularFunction-EBI-UniProt-GOA-ACAP-ARAP_13.05.2021_00h00	5.33E-19	12.68868	269	[ABAT, ACSL3, ACTB, ACTG1, ADAM10, AFDN, AGO2, AKAP12, AKR1B1, ANKFY1, APP, ARMT1, ARPC4, ARPP19, ARRB1, ATP1A1, ATP1B3, ATP6V0A1, ATP6V0C, ATXN10, AUP1, BAG1, BANF1, BCR, BIN1, BOD1, C1QBP, CAP1, CAP2, CASP3, CBX1, CBX3, CCT2, CDC5L, CDC73, CDK5RAP3, CHD4, CHMP3, CHORDC1, CHP1, CKB, CLTC, CLU, CORO1C, CSPG4, CSTB, CTNNB1, CTNNBL1, CTSC, CUL1, CUL4B, CYRIB, DERL1, DHCR24, DHX36, DIAPH1, DIAPH3, DNM1L, DPYSL2, DSP, DUSP3, DVL2, DYM, DYNLL1, EIF3A,	No change

					EIF6, ELP4, EPRS1, ERBB2, ERCC5, ERLIN2, EXOC2, EZR, FANCI, FARP1, FASN, FERMT2, FGD4, FN1, FNBP1L, GARS1, GBP1, GCC2, GGA2, GLMN, GLRX3, GPI, GSK3B, GSTM2, GSTM3, GSTP1, GTF2F1, GYS1, H1-5, HDAC2, HDAC4, HERC2, HGS, HINT1, HMOX1, HNRNPA0, HNRNPUL1, HSP90AA1, HSPA5, IKBKB, IKBKG, IPO5, IPO9, IQGAP3, ITGA3, ITGAV, ITGB1, KAT8, KCNQ1, KDM1A, KPNB1, LAMP1, LDHA, LIMS1, LLGL1, LMNB1, LRPPRC, MACROH2A1, MALT1, MAP1LC3A, MAP2K3, MARCHF5, MCM2, MCM7, MICAL3, MICALL1, MLH1, MSH2, MSH6, MSN, MTA1, MVP, MYO1E, NCKAP1, NCL, NDRG1, NDUFAF7, NEDD4, NEDD8, NISCH, NPLOC4, NPM1, NUTF2, OXSR1, PAFAH1B1, PANX1, PARP1, PARP14, PATZ1, PAWR, PEBP1, PFKM, PIN1, PKN2, PLCG1, PML, POLD1, POLR2A, PPIB, PPP1CA, PPP2R5A, PPP3CA, PRDX6, PRKAB2, PRKCSH, PSMA3, PSMD1, PTPA, PUS7, PXN, PYCARD, QARS1, RAB8A, RABGEF1, RAC3, RAD9A, RALA, RANBP1, RANGAP1, RBBP4, RCC1, RDX, RFC2, RFC5, RHEB, RHOB, RHOC, RIOK3, RIPK2, RNF40, RPA2, RPL5, RPRD1A, RPRD1B, RPRD2, RTN4, SAE1, SARS1, SCARB2, SERPINB8, SERPINB9, SH3BP4, SIRT1, SLC12A2, SLC25A5, SLC2A1, SLC9A1, SMYD3, SNRPD3, SNU13, SNW1, SOAT1, SOD1, SOD2, SORT1, SPART, SPHK2, SQSTM1, SRC, SRSF2, SRSF5, STAT1, STAT2, STOM, STRN3, SUFU, TAX1BP1, TBK1, TCP1, TJP2, TNPO2,	

					TOM1L2, TOR1AIP1, TOR1AIP2, TPI1, TPX2, TRADD, TRAPPC8, TRIM21, TRIM28, TRIP6, TSC2, TSPYL1, TTC28, TUBA4A, TUBB, TWF2, UBA2, UBE2L6, UFD1, URI1, USP22, USP7, VDAC1, VDAC2, VRK3, WDR70, WFS1, WWC2, XPO6, XPOT, YBX1, YES1, YOD1]	
cellular component disassembly	GO_BiologicalProcess-EBI-UniProt-GOA-ACAP-ARAP_13.05.2021_00h00	9.45E-06	12.561983	76	[ABCE1, ADAM10, ADD3, ATG7, BMP1, CAPZA2, CASP3, CDK1, CDK5RAP3, CECR2, CFL2, CHMP3, CTNNB1, DENR, DICER1, DNM1L, DUSP3, DVL2, EIF2D, FLII, FSCN1, GLE1, GLMN, GRWD1, GSK3B, HK2, IST1, KPNB1, LAMC1, LMOD1, MAP1B, MAP1LC3A, MAP4K4, MICAL3, MRPL10, MRPS17, MRPS21, MRPS31, MRRF, N6AMT1, NCAPG, NCK2, NES, OGFOD1, OXA1L, PAFAH1B1, PELO, PIK3CA, PPP1CA, PTGES2, RDX, SEPTIN9, SET, SHFL, SLC25A4, SLC25A5, SMARCB1, SMARCC1, SMARCD3, SMARCE1, SQSTM1, SUPT16H, SVIL, SYNJ1, TIGAR, TOMM7, TPX2, TSC2, TSPO, TWF2, VDAC1, VPS33B, VRK1, WDR1, WIPI2, ZMPSTE24]	No change
manganese ion binding	GO_MolecularFunction-EBI-UniProt-GOA-ACAP-ARAP_13.05.2021_00h00	1.20E-06	26.027397	19	[B4GALT1, CDIPT, EXD2, EXTL2, FEN1, GALNT1, GALNT2, GLUL, IMPA1, LAP3, ME1, MGAT1, NUDT16, PCK2, POMGNT1, PPM1B, SOD2, XPNPEP3, XYLT2]	Downregulation
histone binding	GO_MolecularFunction-EBI-UniProt-GOA-ACAP-ARAP_13.05.2021_00h00	1.00E-05	15.748032	40	[AASS, BAZ1B, BRD3, CBX2, CBX5, CBX8, CHAF1B, CHD2, FMR1, GLYR1, GRWD1, HAT1, IPO9, KAT7, KAT8, LRWD1, MCM2, MCM7, MPHOSPH8, MSH6, NCAPD2, NPM1, PRMT7, PSME4, PWWP2A, RBBP4, RCC1, SET, SIRT1, SMARCC1, SPHK2, SSRP1, STAT1, TDRD3, TSPYL1,	Upregulation

					TTLL12, UHRF2, VRK1, WDR5, YEATS4]	
melanosome	GO_CellularComponent-EBI-UniProt-GOA-ACAP-ARAP_13.05.2021_00h00	6.02E-06	20.338984	24	[ATP1A1, ATP1B3, ATP6V0A1, ATP6V1B2, CANX, CLTC, FASN, HSP90AA1, HSP90B1, HSPA5, ITGB1, LAMP1, NCAPG, PDCD6IP, PPIB, PRDX1, PSMD2, RAB1A, RAB5A, SLC1A4, SLC1A5, SLC2A1, STOM, TPP1]	Downregulation
actomyosin	GO_CellularComponent-EBI-UniProt-GOA-ACAP-ARAP_13.05.2021_00h00	5.55E-07	25.301205	21	[CDC42BPA, CORO1B, FBLIM1, FERMT2, FHOD1, FSCN1, HDAC4, LPP, MYH10, MYH9, MYO18A, MYO1E, PALLD, PDCD6IP, PXN, ROR1, SEPTIN11, SEPTIN7, SEPTIN9, SYNPO, TRIP6]	Upregulation
tRNA aminoacylation	GO_BiologicalProcess-EBI-UniProt-GOA-ACAP-ARAP_13.05.2021_00h00	1.20E-05	28	14	[AARS1, DARS1, EARS2, EEF1E1, EPRS1, GARS1, GATB, HARS1, IARS2, QARS1, RARS2, SARS1, VARS1, YARS1]	No change
ADP binding	GO_MolecularFunction-EBI-UniProt-GOA-ACAP-ARAP_13.05.2021_00h00	1.31E-05	31.578947	12	[APAF1, ATP1A1, CHORDC1, CYB5R3, GLUD1, ME1, MSH2, MSH6, MYO18A, MYO6, SEC14L2, TAP1]	No change
cell-cell contact zone	GO_CellularComponent-EBI-UniProt-GOA-ACAP-ARAP_13.05.2021_00h00	6.38E-06	23.45679	19	[AFDN, ANK3, ATP1A1, CTNNB1, DSP, FHOD1, ITGB1, JAM3, NECTIN2, OBSL1, PIK3CA, RAP2B, RAP2C, SCRIB, SLC2A1, SLC9A1, TJP1, TJP2, TMEM65]	Upregulation
integrin binding	GO_MolecularFunction-EBI-UniProt-GOA-ACAP-ARAP_13.05.2021_00h00	1.69E-05	18.064516	28	[ADAM10, ANXA7, CCN2, CD81, COL5A1, CXCL12, DST, FERMT1, FERMT2, FGF2, FN1, GLMN, ITGA3, ITGAV, ITGB1, ITGB5, JAM3, LAMA5, LAMC1, MYH9, NISCH, PLPP3, PPP3CA, SRC, TGFBI, THBS1, TLN2, VTN]	No change
ubiquitin-like protein ligase binding	GO_MolecularFunction-EBI-UniProt-GOA-ACAP-ARAP_13.05.2021_00h00	8.19E-07	15.407855	51	[ACTG1, ARRB1, ATP6V0C, AUP1, BAG1, CCT2, CDK5RAP3, CKB, CLU, CUL1, CUL4B, DERL1, DNM1L, ERLIN2, GPI, GSK3B, HERC2, HGS, HSP90AA1, HSPA5, IKBKG, LRPPRC, MAP1LC3A, NEDD8, NPLOC4, PATZ1, PML, POLR2A, PRDX6, PSMA3,	Downregulation

					PSMD1, RALA, RANGAP1, RNF40, RPA2, RPL5, RTN4, SLC25A5, SPART, SQSTM1, SRC, STAT1, STAT2, TCP1, TPI1, TRIM28, TUBB, UBE2L6, USP7, WFS1, YOD1]	
ubiquitin protein ligase binding	GO_MolecularFunction-EBI-UniProt-GOA-ACAP-ARAP_13.05.2021_00h00	8.19E-07	15.064102	47	[ACTG1, ARRB1, ATP6V0C, AUP1, BAG1, CCT2, CKB, CLU, CUL1, CUL4B, DERL1, DNM1L, ERLIN2, GPI, GSK3B, HERC2, HSP90AA1, HSPA5, IKBKG, LRPPRC, MAP1LC3A, NEDD8, NPLOC4, PATZ1, PML, POLR2A, PRDX6, PSMA3, PSMD1, RALA, RANGAP1, RNF40, RPA2, RPL5, RTN4, SLC25A5, SPART, SQSTM1, SRC, TCP1, TPI1, TRIM28, TUBB, UBE2L6, USP7, WFS1, YOD1]	Downregulation
regulation of protein stability	GO_BiologicalProcess-EBI-UniProt-GOA-ACAP-ARAP_13.05.2021_00h00	8.93E-09	17.142857	54	[ATP1B3, B4GALT5, BAG1, CASP3, CCNH, CCT2, CCT3, CCT6A, CD81, CDC73, CHORDC1, CHP1, CLU, COG7, CPNE2, CRTAP, CTSA, DDI2, DERL1, GAPDH, GIPC1, GLMP, GNL3L, GOLGA7, GPI, H1-5, HIP1, HSP90AA1, LAMP1, LSS, NCLN, NF2, NOP53, NPM1, P3H1, PIN1, PLPP3, PPIB, PYCARD, RPL5, RTN4, SIRT1, SRC, SUGT1, TCP1, TF, TOMM7, TRIM21, USP28, USP4, USP7, USP9X, WFS1, ZNF207]	No change
protein stabilization	GO_BiologicalProcess-EBI-UniProt-GOA-ACAP-ARAP_13.05.2021_00h00	8.93E-09	16.990292	35	[ATP1B3, BAG1, CCNH, CCT2, CCT3, CCT6A, CHORDC1, CHP1, CLU, COG7, CPNE2, CRTAP, GAPDH, GLMP, GOLGA7, GPI, H1-5, HIP1, HSP90AA1, LAMP1, NCLN, NOP53, NPM1, P3H1, PIN1, PLPP3, PPIB, RPL5, RTN4, SUGT1, TCP1, USP7, USP9X, WFS1, ZNF207]	Downregulation
cell adhesion molecule binding	GO_MolecularFunction-EBI-UniProt-GOA-ACAP-ARAP_13.05.2021_00h00	6.14E-24	20.30981	118	[ABI1, ADAM10, AFDN, ALDOA, ANK3, ANXA7, BAIAP2, BCAM, BZW1, CALD1, CC2D1A, CCN2, CD200, CD81, CDC42EP1, CDH11,	No change

					CHAF1B, CIP2A, CLIC1, CNN3, COBLL1, COL5A1, CORO1B, CTNNB1, CXCL12, DIAPH3, DSP, DST, EEF1G, EIF2S1, EIF3E, EPHA2, EZR, FASN, FERMT1, FERMT2, FGF2, FN1, FNBP1L, FSCN1, GIPC1, GLMN, GOLGA3, H1-10, HSPA5, IST1, ITGA3, ITGAV, ITGB1, ITGB5, JAM3, KIRREL1, KRT18, LAMA5, LAMC1, LARP1, LDHA, LRRFIP1, LYPLA2, MACF1, MB21D2, MICALL1, MSN, MYH9, MYO1B, NCAPG, NDRG1, NECTIN2, NIBAN2, NISCH, PAICS, PALLD, PDXDC1, PKM, PKN2, PLCB3, PLEC, PLPP3, PPFIBP1, PPP1CA, PPP3CA, PRDX1, PRDX6, PTK7, PTPRF, PUF60, RAB10, RAB11B, RAB1A, RANBP1, RANGAP1, RDX, RPL24, RTN4, SCRIB, SEPTIN2, SEPTIN7, SEPTIN9, SNX1, SNX5, SRC, STAT1, STK24, TAGLN2, TBC1D10A, TENM3, TGFBI, THBS1, TJP1, TJP2, TLN2, TRIM25, TWF2, UBFD1, VASP, VTN, YKT6, ZC3HAV1]	
cadherin binding	GO_MolecularFunction-EBI-UniProt-GOA-ACAP-ARAP_13.05.2021_00h00	6.14E-24	23.314608	83	[ABI1, AFDN, ALDOA, ANK3, BAIAP2, BZW1, CALD1, CC2D1A, CDC42EP1, CDH11, CHAF1B, CIP2A, CLIC1, CNN3, COBLL1, CORO1B, CTNNB1, DIAPH3, EEF1G, EIF2S1, EIF3E, EPHA2, EZR, FASN, FNBP1L, FSCN1, GIPC1, GOLGA3, H1-10, HSPA5, IST1, ITGB1, KRT18, LARP1, LDHA, LRRFIP1, LYPLA2, MACF1, MB21D2, MICALL1, MYH9, MYO1B, NCAPG, NDRG1, NIBAN2, PAICS, PDXDC1, PKM, PKN2, PLCB3, PLEC, PPFIBP1, PPP1CA, PRDX1, PRDX6, PUF60, RAB10, RAB11B, RAB1A, RANBP1, RANGAP1, RDX, RPL24, RTN4, SCRIB,	No change

					SEPTIN2, SEPTIN7, SEPTIN9, SNX1, SNX5, SRC, STAT1, STK24, TAGLN2, TBC1D10A, TJP1, TJP2, TRIM25, TWF2, UBFD1, VASP, YKT6, ZC3HAV1]	
endomembrane system organization	GO_BiologicalProcess-EBI-UniProt-GOA-ACAP-ARAP_13.05.2021_00h00	3.11E-09	14.554794	85	[AHCTF1, AKR1B1, ALDH3A2, ANK3, ANO6, APP, ARFGEF1, ATL3, BAIAP2, BANF1, BET1, BIN1, BNIP1, CDK1, CHMP3, CLU, COG7, COL5A1, CORO1C, DNM1L, DYM, EHD3, EMC1, EPB41L3, ESYT1, FBXW8, FNBP1L, GCC2, GET3, HGS, HMOX1, IST1, JAGN1, KPNB1, LBR, LEMD2, MICALL1, MYH9, MYO18A, MYOF, NDRG1, NECTIN2, NPLOC4, NUP205, NUP35, OBSL1, OSBPL2, PACSIN3, PAFAH1B1, PDCD6IP, PI4K2B, PPP1R7, PRDX6, PTGES2, RAB10, RAB1A, RANGAP1, RCC1, REEP2, REEP6, RTN4, SCARB2, SEC61B, SERPINE2, SMPD1, SNF8, SOD1, SQSTM1, SYNJ1, SYPL2, TBPL1, TMED9, TOR1AIP1, TOR1AIP2, TOR1B, TRAM1, TRAM2, TRAPPC8, UBXN2A, VPS25, VPS33B, VPS36, VRK1, WASHC5, ZMPSTE24]	No change
membrane organization	GO_BiologicalProcess-EBI-UniProt-GOA-ACAP-ARAP_13.05.2021_00h00	3.11E-09	12.288931	131	[ABCC1, ACTB, ACTG1, ACTR2, ACTR3, AGRN, AHCTF1, AKR1B1, ALDH3A2, ANK3, ANKFY1, ANO6, ANXA7, APLP2, APP, APPL2, ARHGAP12, ARPC4, ARRB1, ATP2C1, BAIAP2, BANF1, BET1, BIN1, BNIP1, C2CD2L, CDK1, CHMP3, CHORDC1, CHP1, CLTC, CLTCL1, CLU, COL5A1, CORO1C, CTSC, DAB2, DHCR24, DNM1L, DVL2, EMC1, EPB41L3, FNBP1L, GBA2, GET3, GPHN, GRAMD1A, GSK3B, GULP1, HGS, HIP1, HK2, HMOX1, HSP90AA1, HSPA4, IST1, ITSN1, KPNB1, LBR, LEMD2, LLGL1,	No change

					LPCAT2, M6PR, MFN1, MICALL1, MTCH1, MYADM, MYH9, MYO18A, MYOF, NDRG1, NDUFA13, NECTIN2, NMT1, NUP205, NUP35, OSBPL2, OSBPL8, OXA1L, PACSIN3, PAFAH1B1, PDCD6IP, PIK3C2A, PIP4K2B, PPP1R7, PRDX6, PRKCI, PTGES2, RAB1A, RAB5A, RAB8A, RANGAP1, RCC1, REEP2, RTN4, S100A10, SCARB2, SCFD1, SEC14L2, SEC23B, SEC61B, SEPTIN8, SH3KBP1, SLC25A4, SLC25A5, SLC9A1, SMPD1, SNX18, SOD1, STARD5, SYNJ1, SYPL2, TAP1, TBC1D4, TF, THBS1, THEM4, TMED9, TOR1AIP1, TOR1AIP2, TOR1B, TRAM1, TRAM2, TRAPPC4, UBXN2A, VDAC2, VPS33B, VPS41, VRK1, YKT6, ZMPSTE24]	
response to inorganic substance	GO_BiologicalProcess-EBI-UniProt-GOA-ACAP-ARAP_13.05.2021_00h00	3.75E-07	12.898089	81	[ABAT, ACO1, ADNP, AKR1B1, ANK3, ANKZF1, ANXA7, APEX1, APP, ATG7, B2M, CAMK2D, CASP3, CBX8, CDK1, CDK2, CLU, COL18A1, CPNE2, DDI2, DHFR, DHX36, EIF2S1, FMR1, G6PD, GOLT1B, GOT1, GPHN, GPI, HDAC2, HMGCS1, HMOX1, HSPA5, IMPACT, KCNMA1, KRT8, LDHA, LLGL1, MAGI1, MAP1B, MAP1LC3A, MTR, NDRG1, NEDD4, NQO1, NSMCE3, PARP1, PAWR, PIK3CA, PKLR, PPP1CA, PPP3CA, PPP5C, PRDX1, PRKAA1, PTRH2, PYCR1, QDPR, RANBP1, RHOB, RNF2, S100A16, SIRT1, SLC11A2, SLC12A2, SLC1A1, SLC25A12, SLC25A13, SLC25A24, SMPD1, SOD1, SOD2, SPARC, SRC, STAT1, STK24, TF, THBS1, TIGAR, TSPO, TXN]	No change
cellular response to chemical stress	GO_BiologicalProcess-EBI-UniProt-GOA-	3.75E-07	14.441417	53	[AKR1B1, ANKZF1, APEX1, ATG7, CASP3, CBX8, CDK1,	No change

	ACAP-ARAP_13.05.2021_00h00				CLU, CTNNB1, CYCS, DAPK1, DHFR, EIF2S1, FASN, FUT8, G6PD, GSTP1, HDAC2, HMOX1, IMPACT, LRRC8E, MAP1LC3A, MEAK7, MGMT, MGST1, NCOA7, NQO1, OXSR1, PARP1, PAWR, PDK1, PIK3CA, PML, PPP5C, PRDX1, PRDX6, PRKAA1, PXN, PYCR1, RHOB, SESN2, SIRT1, SLC1A1, SLC25A24, SLC2A1, SOD1, SOD2, SRC, SRXN1, STK24, TSPO, TXN, ZFP36L2]	
vitamin binding	GO_MolecularFunction-EBI-UniProt-GOA-ACAP-ARAP_13.05.2021_00h00	9.77E-07	18.934912	32	[ABAT, ALB, CRABP2, DHFR, FASN, GCAT, GOLT1B, GOT1, HACL1, ILVBL, MMAB, MMUT, MTR, OAT, OGFOD1, P3H1, P3H3, PC, PDXDC1, PLOD2, PLPBP, PSAT1, PYGB, RBP1, SBDS, SCLY, SEC14L2, SHMT2, SPTLC1, SPTLC2, SRR, TKT]	Upregulation
vitamin B6 binding	GO_MolecularFunction-EBI-UniProt-GOA-ACAP-ARAP_13.05.2021_00h00	9.77E-07	25.396826	16	[ABAT, ALB, GCAT, GOLT1B, GOT1, OAT, PDXDC1, PLPBP, PSAT1, PYGB, SBDS, SCLY, SHMT2, SPTLC1, SPTLC2, SRR]	Upregulation
transferase complex	GO_CellularComponent-EBI-UniProt-GOA-ACAP-ARAP_13.05.2021_00h00	1.50E-05	11.691543	94	[ABAT, ACTB, ASB1, ASH2L, AUP1, CBX2, CBX5, CBX8, CCNH, CCNK, CDC73, CDK1, CDK2, CRBN, CUL1, CUL4B, DERL1, DLST, EED, ENY2, FAM3A, FBXL12, FBXW8, GAN, GOLGA7, GTF2E1, GTF2F1, GTF2F2, HDAC2, IFIT5, IKBKB, IKBKG, ILVBL, KAT7, KAT8, LMO7, MAEA, MAT2A, MCM3, MED30, METTL14, MGA, MORF4L1, MYO6, N6AMT1, NEDD4, NSMCE3, PFKM, PHKB, PIK3C2A, PIK3CA, POLD1, POLD2, POLE2, POLR1C, POLR1D, POLR2A, POLR2B, POLR2H, POLR2I, POLR3A, POLR3C, PRIM2, PRKAA1, PRKAB2, PRPSAP1, PRPSAP2, PSMD2, PYCARD, RBBP4, RNF2, RNF40, RPRD1A, RPRD1B, RPRD2, SENP3, SESN2,	Upregulation

					SIRT1, SNRPD3, SNRPE, SNW1, SPTLC1, SPTLC2, SUGT1, TAF6L, TBPL1, TRIM21, UBE2L6, UBR2, USP22, WDR41, WDR5, WWP2, YEATS4]	
transferase complex, transferring phosphorus-containing groups	GO_CellularComponent-EBI-UniProt-GOA-ACAP-ARAP_13.05.2021_00h00	1.50E-05	15.969582	42	[CCNH, CCNK, CDC73, CDK1, CDK2, GTF2E1, GTF2F1, GTF2F2, IFIT5, IKBKB, IKBKG, MCM3, MYO6, PFKM, PHKB, PIK3C2A, PIK3CA, POLD1, POLD2, POLE2, POLR1C, POLR1D, POLR2A, POLR2B, POLR2H, POLR2I, POLR3A, POLR3C, PRIM2, PRKAA1, PRKAB2, PRPSAP1, PRPSAP2, PYCARD, RPRD1A, RPRD1B, RPRD2, SESN2, SNW1, TAF6L, TBPL1, WDR41]	Upregulation
lamellipodium	GO_CellularComponent-EBI-UniProt-GOA-ACAP-ARAP_13.05.2021_00h00	1.44E-09	19.36937	43	[ABI1, ACTB, ACTR2, ACTR3, APBB2, APP, ARPIN, BAIAP2, CDC42BPA, CORO1B, CORO1C, CSPG4, CTNNB1, DPYSL3, EPHA2, FAT1, FERMT2, FGD4, FSCN1, GLMN, IGF2BP1, ITGAV, ITGB1, ITSN1, MYH10, NCAPG, NCKAP1, NF2, PALLD, PIEZO1, PIK3CA, PKN2, PLCG1, PXN, RAC3, RDX, SCRIB, SLC9A1, SNX1, SYNE2, TUBB3, TWF2, VASP]	Upregulation
filopodium	GO_CellularComponent-EBI-UniProt-GOA-ACAP-ARAP_13.05.2021_00h00	1.44E-09	21.052631	24	[ABI1, APP, B4GALT1, BAIAP2, EZR, FARP1, FAT1, FGD4, FMR1, FSCN1, IGF2BP1, ITGA3, ITGAV, ITGB1, MSN, MYO1B, MYO6, NF2, RDX, SYNE2, TUBB3, TWF2, UBE2Q1, VASP]	No change
intrinsic component of plasma membrane	GO_CellularComponent-EBI-UniProt-GOA-ACAP-ARAP_13.05.2021_00h00	7.76E-07	4.716981	85	[ABCC1, ABHD12, ALCAM, ANKH, APP, ATP1A1, ATP1B3, ATP2B1, ATP2B2, BCAM, CANX, CD200, CD81, CLU, CSPG4, DCBLD2, DHX34, EFNB2, ENPP1, EPHA2, EPHA3, EPHB4, ERBB2, FAT1, FLRT2, GPC1, GPC6, GPHN, HHIP, HLA-A, HLA-B, HLA-C, IKBKB, ITGA3, ITGAV, ITGB1, ITGB5, JAG1, JAM3,	No change

					KCNMA1, KCNQ1, KIRREL1, LAMP1, LLGL1, LNPEP, LRCH4, LRRC32, LRRC8A, LRRC8E, M6PR, MAEA, MME, NPR2, PACC1, PCDH7, PLPP3, PLXNB2, PROCR, PTK7, PTPRF, PTPRG, REEP2, ROR1, SEMA3C, SLC11A2, SLC12A2, SLC16A3, SLC17A5, SLC1A1, SLC1A4, SLC1A5, SLC25A13, SLC25A4, SLC25A5, SLC26A2, SLC2A1, SLC31A1, SLC7A6, SLC9A1, STOM, TENM3, TGFBR3, TGM2, TPBG, YKT6]	
integral component of plasma membrane	GO_CellularComponent-EBI-UniProt-GOA-ACAP-ARAP_13.05.2021_00h00	7.76E-07	4.7175307	81	[ABCC1, ABHD12, ALCAM, ANKH, APP, ATP1A1, ATP1B3, ATP2B1, ATP2B2, BCAM, CANX, CD200, CD81, CLU, CSPG4, DCBLD2, DHX34, EFNB2, ENPP1, EPHA2, EPHA3, EPHB4, ERBB2, FAT1, FLRT2, HHIP, HLA-A, HLA-B, HLA-C, IKBKB, ITGA3, ITGAV, ITGB1, ITGB5, JAG1, JAM3, KCNMA1, KCNQ1, KIRREL1, LAMP1, LLGL1, LNPEP, LRCH4, LRRC32, LRRC8A, LRRC8E, M6PR, MAEA, MME, NPR2, PACC1, PCDH7, PLPP3, PLXNB2, PROCR, PTK7, PTPRF, PTPRG, REEP2, ROR1, SEMA3C, SLC11A2, SLC12A2, SLC16A3, SLC17A5, SLC1A1, SLC1A4, SLC1A5, SLC25A13, SLC25A4, SLC25A5, SLC26A2, SLC2A1, SLC31A1, SLC7A6, SLC9A1, STOM, TENM3, TGFBR3, TPBG, YKT6]	No change
nucleosome binding	GO_MolecularFunction-EBI-UniProt-GOA-ACAP-ARAP_13.05.2021_00h00	6.59E-08	28.378378	21	[ACTB, ARID1B, CHD4, EED, GLYR1, H1-10, H1-5, HDAC2, HNRNPC, MACROH2A1, MBD3, RBBP4, RCC1, SMARCA1, SMARCB1, SMARCC1, SMARCE1, SPHK2, SSRP1, SUPT16H, VRK1]	Upregulation
nucleosomal DNA binding	GO_MolecularFunction-EBI-UniProt-GOA-ACAP-	6.59E-08	30.232557	13	[ACTB, CHD4, H1-10, H1-5, HDAC2, HNRNPC, MACROH2A1,	Upregulation

	ARAP_13.05.2021_00h00				MBD3, RBBP4, RCC1, SMARCB1, SMARCC1, SMARCE1]	
identical protein binding	GO_MolecularFunction-EBI-UniProt-GOA-ACAP-ARAP_13.05.2021_00h00	5.48E-21	13.024809	273	[ABAT, ABCB7, ACACA, ACADM, ACADSB, ACADVL, ACOT7, ACOX1, ACTB, ACTG1, ADAM10, ADSL, ALB, ALCAM, ALDH18A1, ALDH3A2, ALDOA, APAF1, APIP, APLP2, APP, APPL2, ASNS, ATG7, ATL3, ATXN10, B2M, BAIAP2, BANF1, BCAT1, BIN1, BMP1, CAMK2D, CAP2, CAPN5, CASP6, CBX1, CBX3, CBX5, CDC42BPA, CIP2A, COIL, CORO1B, CRYZ, CTBP1, CTPS1, CTSC, DAPK1, DCPS, DCXR, DDI2, DERL1, DHPS, DNM1L, DPP9, DPYSL2, DPYSL3, DRG1, DVL2, DYNLL1, ECE1, EED, EIF2AK2, EIF2B1, EIF3F, EMG1, ENPP1, EPM2AIP1, EPRS1, ERBB2, ERCC5, ESYT1, ETHE1, EXD2, EZR, FASN, FGF2, FHL2, FHOD1, FMR1, FN1, FSD1, FTL, G6PD, GALE, GAPDH, GARS1, GBP1, GCC2, GGCT, GIPC1, GIPC2, GLA, GLE1, GLMN, GLRX3, GLUD1, GLUL, GPD1L, GPHN, GSTM2, GSTM3, HACL1, HADH, HADHA, HARS1, HDAC4, HEXB, HIP1, HMGCS1, HMOX1, HNRNPC, HNRNPH1, HSD17B4, HSP90AA1, HSPB6, IKBKB, IKBKG, IMPA1, JAM3, KCTD12, KCTD15, LACTB, LDHA, LRRC8A, LRRFIP1, LRRK1, MAD1L1, MAD2L1, MALT1, MAT2A, MATR3, MCM6, ME1, MFF, MFN1, MGST1, MICALL1, MME, MMUT, MPST, MRM3, MSH2, MSH6, MSI2, MTCH1, MVD, MVK, MVP, MYD88, MYH9, NCL, NDRG1, NECTIN2, NISCH, NOP53, NPM1, NPR2, NQO1, NT5C, NUDT16, NUDT16L1, NUP35, NUTF2, OAT, OPLAH, OXA1L,	No change

					OXSR1, PACSIN3, PAFAH1B1, PAFAH1B2, PAFAH1B3, PAICS, PARP1, PC, PDCD6IP, PFKM, PIP4K2B, PKN2, PML, PNP, PPP5C, PRDX1, PRDX6, PRKAB2, PRPF19, PRPSAP1, PRPSAP2, PSAT1, PSMC3, PSMC6, PSMD7, PSME2, PTPA, PTPRG, PUF60, PYCARD, PYCR1, QDPR, QTRT1, QTRT2, RIPK2, RNF40, RPL22, RPRD1B, RRM1, S100A10, S100A16, SAMHD1, SARS1, SBDS, SCARB2, SCLY, SEC14L2, SEPTIN7, SERPINB8, SF1, SH3BP4, SHMT2, SIRT1, SLC1A1, SLC25A12, SLC25A13, SLC2A1, SLC31A1, SLIT2, SMAD4, SMCHD1, SNF8, SNRNP200, SNRPA, SNX1, SOAT1, SOD1, SOD2, SQSTM1, SRR, STAT1, STAT2, STAT5B, STK24, STOM, TAP1, TBK1, TENM3, TGFBI, TGM2, THBS1, TKT, TMEM192, TOR1B, TPI1, TPP2, TPST2, TRA2B, TRADD, TRIM21, TRIP13, TRMT10C, TSC2, TSPO, TXN, TYMP, UAP1, UBA3, UGP2, USP4, VDAC1, VPS25, VPS41, WRNIP1, XIAP, XPNPEP3]	
protein homodimerization activity	GO_MolecularFunction-EBI-UniProt-GOA-ACAP-ARAP_13.05.2021_00h00	5.48E-21	12.94278	95	[ABCB7, ACOT7, ACOX1, ADAM10, ALDH3A2, APLP2, APPL2, ATG7, B2M, BANF1, CAMK2D, CIP2A, DNM1L, ECE1, EIF2AK2, ENPP1, EPRS1, ERCC5, EXD2, FMR1, G6PD, GALE, GBP1, GGCT, GLA, GLMN, GPD1L, GSTM2, GSTM3, HARS1, HIP1, HMGCS1, HMOX1, HNRNPC, HSD17B4, HSP90AA1, HSPB6, IKBKB, IKBKG, JAM3, LRRFIP1, MAD2L1, MFF, MME, MMUT, MSH2, MSH6, MTCH1, MVD, MYH9, NECTIN2, NPM1, NUDT16,	No change

					NUDT16L1, OXA1L, PAFAH1B2, PAFAH1B3, PDCD6IP, PIP4K2B, PML, PSMD7, PTPA, PYCARD, QTRT1, QTRT2, RIPK2, RNF40, S100A10, S100A16, SARS1, SBDS, SCARB2, SCLY, SEC14L2, SHMT2, SLIT2, SMAD4, SMCHD1, SNF8, SNX1, SRR, STAT1, STOM, TAP1, TENM3, TKT, TMEM192, TPI1, TPST2, TRIM21, TSC2, TXN, TYMP, VPS25, XPNPEP3]	
endothelial cell development	GO_BiologicalProcess-EBI-UniProt-GOA-ACAP-ARAP_13.05.2021_00h00	9.62E-06	23.68421	18	[AFDN, COL15A1, COL18A1, CTNNB1, EZR, FASN, GSTM3, IKBKB, MSN, MYADM, MYD88, RAB1A, RAP2B, RAP2C, RDX, TJP1, TJP2, ZDHHC21]	Upregulation
establishment of endothelial barrier	GO_BiologicalProcess-EBI-UniProt-GOA-ACAP-ARAP_13.05.2021_00h00	9.62E-06	26.785715	15	[AFDN, CTNNB1, EZR, FASN, IKBKB, MSN, MYADM, MYD88, RAB1A, RAP2B, RAP2C, RDX, TJP1, TJP2, ZDHHC21]	Upregulation
establishment of endothelial intestinal barrier	GO_BiologicalProcess-EBI-UniProt-GOA-ACAP-ARAP_13.05.2021_00h00	9.62E-06	47.058823	8	[AFDN, FASN, MYD88, RAB1A, RAP2B, RAP2C, TJP1, TJP2]	Upregulation
ribonucleoprotein complex biogenesis	GO_BiologicalProcess-EBI-UniProt-GOA-ACAP-ARAP_13.05.2021_00h00	1.16E-16	18.75	93	[AAR2, ABCE1, ABT1, AGO2, BMS1, C1QBP, CDC73, CELF2, CHD7, CNGB1, COIL, COLEC12, CUL4B, DDX1, DDX47, DENR, DICER1, DIMT1, EFL1, EIF2D, EIF2S1, EIF3A, EIF3B, EIF3E, EIF3F, EIF6, EMG1, GCFC2, GEMIN4, GLUL, GNL3L, HEATR3, HNRNPC, HSP90AA1, MMAB, MRM3, MRPL10, MRTO4, MTREX, NEDD4, NLE1, NMD3, NOL11, NOM1, NOP53, NPM1, NSUN5, NUDT16, PRMT7, PRPF19, PSIP1, PUF60, RIOK3, RPF2, RPL10A, RPL13, RPL13A, RPL18A, RPL22, RPL24, RPL27, RPL32, RPL36, RPL38, RPL5, RPLP1, RPLP2, RPS16, RPS21, RPS5, RPS8, RPS9, SBDS, SCAF11, SENP3, SF1, SF3A3, SNRNP200,	No change

					SNRPD3, SNRPE, SNU13, SRSF10, SRSF5, TGS1, TSR2, URB2, USP4, UTP14A, UTP20, UTP3, WDR12, YBEY, YTHDC1]	
ribonucleoprotein complex assembly	GO_BiologicalProcess-EBI-UniProt-GOA-ACAP-ARAP_13.05.2021_00h00	1.16E-16	22.660099	46	[AAR2, ABT1, AGO2, CDC73, CELF2, COIL, COLEC12, DDX1, DENR, DICER1, EIF2D, EIF3A, EIF3B, EIF3E, EIF3F, EIF6, GCFC2, GEMIN4, HNRNPC, HSP90AA1, MMAB, MRTO4, NLE1, NOP53, PRMT7, PRPF19, PSIP1, PUF60, RPF2, RPL13A, RPL24, RPL38, RPL5, RPS5, SCAF11, SF1, SF3A3, SNRNP200, SNRPD3, SNRPE, SNU13, SRSF10, SRSF5, TGS1, USP4, YTHDC1]	Upregulation
spliceosomal complex assembly	GO_BiologicalProcess-EBI-UniProt-GOA-ACAP-ARAP_13.05.2021_00h00	1.16E-16	25.423729	15	[CELF2, DDX1, GCFC2, HNRNPC, PRPF19, PSIP1, PUF60, SCAF11, SF1, SF3A3, SNRNP200, SNRPE, SRSF10, SRSF5, YTHDC1]	Upregulation
type I interferon production	GO_BiologicalProcess-EBI-UniProt-GOA-ACAP-ARAP_13.05.2021_00h00	9.42E-07	20.437956	28	[CTNNB1, DHX36, GAPDH, HSP90AA1, IGF2BP1, LRRFIP1, MYD88, NFKB2, NPLOC4, PIN1, POLR1C, POLR1D, POLR2H, POLR3A, POLR3C, PPM1B, PYCARD, RIOK3, RIPK2, STAT1, TAX1BP1, TBK1, TRIM21, TRIM25, TRIM56, UBE2L6, UFD1, ZCCHC3]	Downregulation
regulation of type I interferon production	GO_BiologicalProcess-EBI-UniProt-GOA-ACAP-ARAP_13.05.2021_00h00	9.42E-07	20.437956	28	[CTNNB1, DHX36, GAPDH, HSP90AA1, IGF2BP1, LRRFIP1, MYD88, NFKB2, NPLOC4, PIN1, POLR1C, POLR1D, POLR2H, POLR3A, POLR3C, PPM1B, PYCARD, RIOK3, RIPK2, STAT1, TAX1BP1, TBK1, TRIM21, TRIM25, TRIM56, UBE2L6, UFD1, ZCCHC3]	Downregulation
positive regulation of type I interferon production	GO_BiologicalProcess-EBI-UniProt-GOA-ACAP-ARAP_13.05.2021_00h00	9.42E-07	21.83908	19	[CTNNB1, DHX36, GAPDH, HSP90AA1, IGF2BP1, LRRFIP1, MYD88, NFKB2, POLR1C, POLR1D, POLR2H, POLR3A, POLR3C, RIOK3, RIPK2, STAT1, TBK1, TRIM56, ZCCHC3]	No change
anchoring junction	GO_CellularComponent-EBI-UniProt-GOA-ACAP-	3.00E-26	18.029116	161	[ACTB, ACTG1, ACTR2, ACTR3, ADAM10, ADD3, AFDN, AKAP12,	No change

	ARAP_13.05.2021_00h00				AKR1B1, ALCAM, AMOTL1, ANK3, ANXA5, APP, ARHGAP17, ARL14EP, ARPC5L, ATP1A1, ATP6V0C, B2M, B4GALT1, BAIAP2, CAP1, CAPN5, CASK, CD81, CDC42BPA, CDC42EP1, CDH11, CHAF1B, CHORDC1, CHP1, CLTC, CNN1, CNN3, CORO1B, CORO1C, CORO2B, CSPG4, CSRP2, CTNNB1, DAB2, DNMBP, DSP, DST, DYNLL1, EFNB2, EHD3, EPB41L2, EPB41L3, EPHA2, EPPK1, EZR, FAT1, FBLIM1, FERMT1, FERMT2, FHL1, FHL2, FHOD1, FLII, FLRT2, FSCN1, GAB1, GLMN, HSP90B1, HSPA5, HSPG2, ILF3, IQGAP3, ITGA3, ITGAV, ITGB1, ITGB5, JAG1, JAM3, KIRREL1, KRT18, KRT8, LAP3, LIMS1, LIN7C, LMO7, LPP, MAGI1, MAP4K4, MCAM, MDC1, MME, MSN, MYADM, MYH9, MYO1E, NCKAP1, NDRG1, NECTIN2, NF2, NIBAN2, NPM1, OBSL1, PALLD, PANX1, PDCD6IP, PDZD11, PIGU, PIK3CA, PKN2, PLCG1, PLEC, PLPP3, PPFIBP1, PPIB, PPP1CA, PPP3CA, PRKCI, PROCR, PRUNE1, PTK7, PTPN12, PXN, RAB10, RALA, RAP2B, RAP2C, RDX, RHOB, RPL10A, RPL13A, RPL22, RPL27, RPL38, RPL5, RPLP1, RPLP2, RPS13, RPS16, RPS18, RPS5, RPS8, RPS9, RRAS2, SCARB2, SCRIB, SH3KBP1, SLC2A1, SLC9A1, SORBS3, SRC, SVIL, SYNE2, SYNPO, TGM2, TJP1, TJP2, TLN2, TMEM65, TNS3, TRIP6, VASP, WDR1, YES1]	
cell-cell junction	GO_CellularComponent-EBI-UniProt-GOA-ACAP-ARAP_13.05.2021_00h00	3.00E-26	15.32567	80	[ACTB, ACTG1, ACTR3, ADAM10, ADD3, AFDN, AKR1B1, AMOTL1, ANK3, APP, ARHGAP17, ATP1A1,	Upregulation

					B4GALT1, BAIAP2, CASK, CDC42BPA, CDC42EP1, CDH11, CHAF1B, CNN3, CTNNB1, DNMBP, DSP, EFNB2, EPB41L3, EPHA2, EPPK1, FAT1, FERMT2, FHOD1, FLRT2, FSCN1, GAB1, ILF3, IQGAP3, ITGB1, JAG1, JAM3, KIRREL1, KRT18, KRT8, LIMS1, LIN7C, LMO7, MAGI1, MYADM, MYH9, MYO1E, NDRG1, NECTIN2, NF2, NIBAN2, OBSL1, PANX1, PDCD6IP, PDZD11, PIGU, PIK3CA, PKN2, PLCG1, PLPP3, PPP1CA, PPP3CA, PRKCI, PTK7, PXN, RAB10, RAP2B, RAP2C, RDX, SCRIB, SH3KBP1, SLC2A1, SLC9A1, SYNPO, TJP1, TJP2, TMEM65, VASP, WDR1]	
cell-substrate junction	GO_CellularComponent-EBI-UniProt-GOA-ACAP-ARAP_13.05.2021_00h00	3.00E-26	23.47826	108	[ACTB, ACTG1, ACTR2, ACTR3, ADAM10, AKAP12, ALCAM, ANXA5, ARL14EP, ARPC5L, ATP6V0C, B2M, CAP1, CAPN5, CASK, CD81, CDC42EP1, CHORDC1, CHP1, CLTC, CNN1, CNN3, CORO1B, CORO1C, CORO2B, CSPG4, CSRP2, CTNNB1, DAB2, DST, DYNLL1, EFNB2, EHD3, EPB41L2, EPHA2, EPPK1, EZR, FAT1, FBLIM1, FERMT1, FERMT2, FHL1, FHL2, FLII, FLRT2, GLMN, HSP90B1, HSPA5, HSPG2, ITGA3, ITGAV, ITGB1, ITGB5, LAP3, LIMS1, LMO7, LPP, MAP4K4, MCAM, MDC1, MME, MSN, MYH9, NCKAP1, NECTIN2, NPM1, PALLD, PDCD6IP, PLEC, PPFIBP1, PPIB, PROCR, PRUNE1, PTK7, PTPN12, PXN, RAB10, RALA, RDX, RHOB, RPL10A, RPL13A, RPL22, RPL27, RPL38, RPL5, RPLP1, RPLP2, RPS13, RPS16, RPS18, RPS5, RPS8, RPS9, RRAS2, SCARB2, SH3KBP1, SLC9A1, SORBS3, SRC, SVIL, SYNE2,	No change

					TGM2, TLN2, TNS3, TRIP6, VASP, YES1]	
adherens junction	GO_CellularComponent-EBI-UniProt-GOA-ACAP-ARAP_13.05.2021_00h00	3.00E-26	17.222221	31	[ACTB, ADAM10, AFDN, BAIAP2, CDC42EP1, CDH11, CHAF1B, CNN3, CTNNB1, EFNB2, FERMT2, ILF3, JAG1, KRT18, LIN7C, LMO7, MAGI1, MYH9, MYO1E, NDRG1, NECTIN2, NF2, NIBAN2, PDZD11, PLPP3, PPP1CA, RAB10, RDX, SCRIB, TJP1, TJP2]	Upregulation
vacuole	GO_CellularComponent-EBI-UniProt-GOA-ACAP-ARAP_13.05.2021_00h00	9.73E-08	12.543554	108	[ACTR2, AGRN, AKR1B1, ANK3, ANKFY1, AP5B1, APEX1, ARRB1, ASAH1, ATP6V0A1, ATP6V0C, ATP6V1A, ATP6V1B2, AUP1, B4GALT1, CAP1, CCT2, CHID1, CLTC, CLU, COL6A1, CSPG4, CTSA, CTSC, CYB5R3, DAB2, DAGLB, DCN, ECE1, ENPP1, FAM3A, FTL, GDAP2, GLA, GLMP, GNB4, GOLT1B, GOT1, GPC1, GPC6, GRAMD1A, HEXB, HGS, HSP90AA1, HSPG2, IST1, ITM2C, KCNQ1, LAMP1, LIPA, LNPEP, LRRC8A, LRRC8E, LUM, M6PR, MAN2B2, MAP1LC3A, MEAK7, MGST1, MTCH1, MYO6, PIP4K2B, PLA2G15, PLBD2, PLD1, PLD3, PRDX6, PRKCSH, PSMD1, PTGES2, PYCARD, PYGB, RAB23, RAB3D, RHEB, SCARB2, SEPTIN9, SGSH, SIAE, SLC11A2, SLC17A5, SLC44A2, SMPD1, SNX1, SOD1, SORT1, SPHK2, SQSTM1, SRC, STOM, TBC1D17, TMEM192, TMEM97, TOM1, TPP1, TRIM21, TSC2, TUBB, UNC93B1, USP4, USP5, VAT1, VLDLR, VPS33B, VPS41, WDR11, WDR41, WIPI2]	Downregulation
vacuolar lumen	GO_CellularComponent-EBI-UniProt-GOA-ACAP-ARAP_13.05.2021_00h00	9.73E-08	18.461538	36	[ACTR2, AGRN, ASAH1, CAP1, CCT2, CHID1, CSPG4, CTSA, CTSC, CYB5R3, DCN, FTL, GLA, GPC1, GPC6, HEXB, HSP90AA1, HSPG2, IST1, LIPA, LUM, MAN2B2, MTCH1,	Downregulation

					PLBD2, PLD3, PRDX6, PSMD1, PTGES2, PYCARD, PYGB, SCARB2, SGSH, SMPD1, TPP1, TUBB, VAT1]	
lysosome	GO_CellularComponent-EBI-UniProt-GOA-ACAP-ARAP_13.05.2021_00h00	9.73E-08	12.945839	98	[ACTR2, AGRN, AKR1B1, ANK3, ANKFY1, AP5B1, APEX1, ARRB1, ASAH1, ATP6V0A1, ATP6V0C, ATP6V1A, ATP6V1B2, B4GALT1, CAP1, CCT2, CHID1, CLTC, CLU, COL6A1, CSPG4, CTSA, CTSC, CYB5R3, DAB2, DAGLB, DCN, ECE1, ENPP1, FTL, GDAP2, GLA, GLMP, GNB4, GOLT1B, GOT1, GPC1, GPC6, HEXB, HGS, HSP90AA1, HSPG2, IST1, ITM2C, KCNQ1, LAMP1, LIPA, LNPEP, LRRC8A, LRRC8E, LUM, M6PR, MAN2B2, MEAK7, MGST1, MTCH1, MYO6, PLA2G15, PLBD2, PLD1, PLD3, PRDX6, PRKCSH, PSMD1, PTGES2, PYCARD, PYGB, RAB3D, RHEB, SCARB2, SGSH, SIAE, SLC11A2, SLC17A5, SLC44A2, SMPD1, SNX1, SOD1, SORT1, SPHK2, SQSTM1, SRC, STOM, TMEM192, TMEM97, TOM1, TPP1, TSC2, TUBB, UNC93B1, USP4, USP5, VAT1, VLDLR, VPS33B, VPS41, WDR11, WDR41]	Downregulation
lysosomal lumen	GO_CellularComponent-EBI-UniProt-GOA-ACAP-ARAP_13.05.2021_00h00	9.73E-08	20.560747	22	[AGRN, ASAH1, CHID1, CSPG4, CTSA, DCN, GLA, GPC1, GPC6, HEXB, HSP90AA1, HSPG2, LIPA, LUM, MAN2B2, MTCH1, PLBD2, PLD3, SCARB2, SGSH, SMPD1, TPP1]	Downregulation
ATPase, acting on DNA	GO_MolecularFunction-EBI-UniProt-GOA-ACAP-ARAP_13.05.2021_00h00	2.58E-08	18.04878	37	[AQR, CHD1L, CHD2, CHD4, CHD7, CHTOP, DDX1, DDX42, DDX47, DHX34, DHX36, DICER1, ERCC6L, GTF2F2, HELZ, MCM2, MCM3, MCM4, MCM5, MCM6, MCM7, MOV10, MSH2, MSH6, MTREX, MYO18A, RBBP4, RECQL, RFC2, RFC3, RFC5, SHPRH, SIRT1, SMARCA1,	Upregulation

					SMARCAD1, SNRNP200, WRNIP1]	
helicase activity	GO_MolecularFunction-EBI-UniProt-GOA-ACAP-ARAP_13.05.2021_00h00	2.58E-08	19.444445	35	[AQR, CHD1L, CHD2, CHD4, CHD7, CHTOP, DDX1, DDX42, DDX47, DHX34, DHX36, DICER1, ERCC6L, GTF2F2, HELZ, MCM2, MCM3, MCM4, MCM5, MCM6, MCM7, MOV10, MSH2, MSH6, MTREX, RECQL, RFC2, RFC3, RFC5, SHPRH, SIRT1, SMARCA1, SMARCAD1, SNRNP200, WRNIP1]	Upregulation
nuclear DNA replication	GO_BiologicalProcess-EBI-UniProt-GOA-ACAP-ARAP_13.05.2021_00h00	2.58E-08	23.68421	18	[FEN1, LIG1, MCM2, MCM3, MCM4, MCM5, MCM6, MCM7, POLD1, POLD2, PRIM2, RFC2, RFC3, RFC5, RPA1, RPA2, STON1, ZMPSTE24]	Upregulation
DNA duplex unwinding	GO_BiologicalProcess-EBI-UniProt-GOA-ACAP-ARAP_13.05.2021_00h00	2.58E-08	20.869566	24	[CETN2, CHD1L, CHD2, CHD4, CHD7, CUL4B, DDX1, DHX36, ERCC6L, GTF2F2, MCM2, MCM3, MCM4, MCM5, MCM6, MCM7, PARP1, RECQL, RFC2, RFC3, RFC5, RPA1, SMARCAD1, WRNIP1]	Upregulation
organelle envelope	GO_CellularComponent-EBI-UniProt-GOA-ACAP-ARAP_13.05.2021_00h00	1.09E-18	11.928738	154	[ABCB7, ACADM, ACADVL, ACSL3, ACSL4, AHCTF1, ALDH18A1, APP, BANF1, BIN1, BNIP1, CBX3, CBX5, CENPV, CETN2, CLIC1, CLU, COQ5, COX20, COX4I1, COX6C, CYB5R3, CYCS, DHCR7, DNM1L, DST, ENY2, EPHA3, ETFDH, EXD2, FDXR, GAPDH, GARS1, GCAT, GDAP1, GLE1, GPD2, GTF3C3, HADHA, HK1, HK2, INPP4A, INTS1, IPO5, IPO9, IST1, ITSN1, KPNB1, LBR, LEMD2, LMNB1, LMO7, LRPPRC, MAD1L1, MAD2L1, MAOA, MARCHF5, MATR3, MFF, MFN1, MFSD10, MGST1, MRPL10, MRPS17, MRPS21, MRPS31, MTA1, MTCH1, MVP, MYO1E, MYO6, MYOF, NDUFA13, NDUFA4, NDUFS3, NNT, NUCB2, NUP205, NUP35, NUP62, NUTF2, OSBPL8, OXA1L,	No change

					PAFAH1B1, PARL, PARP1, PGAM5, PLA2G4A, PTPMT1, QTRT1, QTRT2, RANBP1, RANGAP1, RCC1, RETSAT, RIF1, RRM1, RTN4, SARM1, SCAI, SCRN1, SDHC, SFXN2, SFXN3, SH3BGRL2, SHMT2, SIRT1, SLC11A2, SLC16A3, SLC25A10, SLC25A12, SLC25A13, SLC25A15, SLC25A19, SLC25A22, SLC25A24, SLC25A26, SLC25A4, SLC25A5, SLC25A6, SLC44A1, SOD1, SORT1, SPHK2, SRC, SULT1E1, SURF1, SYNE2, THEM4, TIGAR, TIMM17B, TIMMDC1, TMEM65, TMEM97, TMX4, TOMM34, TOMM7, TOR1AIP1, TOR1AIP2, TOR1B, TRA2B, TSPO, TUBB, UQCR10, UQCR11, UQCRC2, VAT1, VDAC1, VDAC2, XPO4, XPOT, YBX1, YEATS4, ZMPSTE24]	
mitochondrion	GO_CellularComponent-EBI-UniProt-GOA-ACAP-ARAP_13.05.2021_00h00	1.09E-18	11.848884	207	[AARS1, AASS, ABAT, ABCB7, ABCE1, ACAD8, ACADM, ACADSB, ACADVL, ACAT2, ACO1, ACOT7, ACSF2, ACSL3, ACSL4, ACSS2, AGPS, AK3, AK4, ALDH18A1, ALDH2, APEX1, ARAF, ATPAF1, BCAT1, BDH2, BNIP1, C1QBP, CDK1, CHPF, CLIC1, CLU, COQ5, COX20, COX4I1, COX6C, CYB5R3, CYCS, CYRIB, DCPS, DDX1, DGLUCY, DGUOK, DHFR, DHX36, DIMT1, DLST, DNM1L, DUT, DYNLL1, EARS2, ECI2, ENY2, ETFDH, ETHE1, EXD2, FAHD1, FAM3A, FDXR, FEN1, FIBP, GARS1, GATB, GCAT, GCDH, GDAP1, GLUD1, GLUL, GPD2, GPN1, GSK3B, GSTP1, GTF3C4, HADH, HADHA, HARS1, HAT1, HIBCH, HK1, HK2, HSD17B10, HSDL2, HSP90AA1, HSPA4, HSPA5, HSPE1,	No change

					IARS2, IDH2, ILF3, IREB2, LACTB, LIG1, LIG3, LRPPRC, LRRK1, MAOA, MARCHF5, ME1, MFF, MFN1, MGST1, MMAB, MMUT, MPST, MRM3, MRPL10, MRPS17, MRPS21, MRPS31, MRRF, MTCH1, MTHFD2, NDUFA13, NDUFA4, NDUFAF7, NDUFS3, NENF, NMT1, NNT, NOCT, NUBPL, OAT, OXA1L, PARG, PARL, PC, PCK2, PDK1, PGAM5, PIN1, PITRM1, PKM, PLA2G4A, PNPLA4, PPP3CA, PRDX6, PTGES2, PTPMT1, PTRH2, PYCARD, PYCR1, QARS1, QTRT1, QTRT2, RALA, RAP1GDS1, RARS2, SACS, SARM1, SBDS, SCP2, SDHC, SESN2, SFXN2, SFXN3, SHMT2, SIRT1, SLC11A2, SLC25A10, SLC25A12, SLC25A13, SLC25A15, SLC25A19, SLC25A22, SLC25A24, SLC25A26, SLC25A4, SLC25A5, SLC25A6, SLC44A1, SLC44A2, SLC9A1, SOAT1, SOD1, SOD2, SPARC, SPHK2, SQSTM1, SRC, STOM, SURF1, SYNE2, TFAM, TGM2, THEM4, TIGAR, TIMM17B, TIMMDC1, TMEM65, TOMM34, TOMM7, TRMT10C, TRMT5, TSPO, UQCR10, UQCR11, UQCRC2, URI1, USP48, VARS1, VAT1, VDAC1, VDAC2, VWA8, XPNPEP3, YKT6, YRDC]	
nuclear envelope	GO_CellularComponent-EBI-UniProt-GOA-ACAP-ARAP_13.05.2021_00h00	1.09E-18	15.182186	75	[AHCTF1, APP, BANF1, BIN1, BNIP1, CBX3, CBX5, CENPV, CETN2, CLIC1, DHCR7, DST, ENY2, EPHA3, GAPDH, GARS1, GLE1, GTF3C3, INPP4A, INTS1, IPO5, IPO9, IST1, ITSN1, KPNB1, LBR, LEMD2, LMNB1, LMO7, LRPPRC, MAD1L1, MAD2L1, MATR3, MFSD10, MTA1, MVP, MYO1E, MYO6, MYOF, NUCB2, NUP205,	Upregulation

					NUP35, NUP62, NUTF2, OSBPL8, PAFAH1B1, PARP1, PLA2G4A, RANBP1, RANGAP1, RCC1, RETSAT, RIF1, RRM1, RTN4, SCAI, SCRN1, SH3BGRL2, SIRT1, SLC16A3, SORT1, SULT1E1, SYNE2, TMEM97, TMX4, TOR1AIP1, TOR1AIP2, TOR1B, TRA2B, TUBB, XPO4, XPOT, YBX1, YEATS4, ZMPSTE24]	
mitochondrial matrix	GO_CellularComponent-EBI-UniProt-GOA-ACAP-ARAP_13.05.2021_00h00	1.09E-18	14.257425	72	[AASS, ABAT, ABCE1, ACAD8, ACADM, ACADSB, ACADVL, ACSF2, ACSS2, AK3, AK4, ALDH2, C1QBP, CDK1, CHPF, COQ5, DGLUCY, DGUOK, DIMT1, DLST, EARS2, ETFDH, ETHE1, EXD2, FAHD1, FAM3A, FDXR, GARS1, GCDH, GLUD1, HADH, HADHA, HIBCH, HSD17B10, HSPE1, IARS2, IDH2, LRPPRC, MMAB, MMUT, MPST, MRM3, MRPL10, MRPS17, MRPS21, MRPS31, MRRF, MTHFD2, NDUFAF7, NDUFS3, NUBPL, OAT, OXA1L, PARG, PC, PCK2, PDK1, PITRM1, PYCR1, QARS1, RARS2, SHMT2, SLC25A5, SOAT1, SOD1, SOD2, TFAM, THEM4, TRMT10C, TRMT5, VDAC1, VDAC2]	No change
nuclear membrane	GO_CellularComponent-EBI-UniProt-GOA-ACAP-ARAP_13.05.2021_00h00	1.09E-18	15.047022	48	[AHCTF1, CBX3, CENPV, CLIC1, DHCR7, EPHA3, GAPDH, GARS1, GLE1, GTF3C3, INPP4A, INTS1, KPNB1, LBR, LEMD2, LMNB1, LRPPRC, MATR3, MFSD10, MYO6, MYOF, NUP205, NUP35, NUP62, NUTF2, OSBPL8, PAFAH1B1, RANGAP1, RCC1, RETSAT, RIF1, SCAI, SCRN1, SH3BGRL2, SIRT1, SLC16A3, SORT1, SULT1E1, SYNE2, TMEM97, TMX4, TOR1AIP1, TOR1AIP2, TOR1B, TRA2B, YBX1, YEATS4, ZMPSTE24]	Upregulation
autophagy	GO_BiologicalProcess-EBI-UniProt-GOA-ACAP-	7.41E-13	13.752122	81	[ANXA7, APEX1, ATG4B, ATG7, ATP6V0A1, ATP6V0C, ATP6V1A,	No change

	ARAP_13.05.2021_00h00				ATP6V1B2, AUP1, CASP3, CHMP3, CLTC, CLU, CTSA, DAPK1, DCN, DNM1L, DYNC1LI1, DYNLL1, EIF4G3, FNBP1L, GAPDH, GPSM1, GRAMD1A, GSK3B, HGS, HK2, HMOX1, HSP90AA1, IKBKG, LARP1, MAP1LC3A, MFN1, MTCH1, NEDD4, PAFAH1B2, PGAM5, PIK3C2A, PIK3CA, PIP4K2B, PRKAA1, PRKAB2, PYCARD, QSOX1, RAB1A, RAB23, RAB5A, RAB8A, RHEB, RIPK2, SCFD1, SESN2, SH3BP4, SIRT1, SLC25A4, SLC25A5, SNF8, SNX5, SQSTM1, SRC, TAB2, TBC1D17, TBK1, TIGAR, TOMM7, TRAPPC4, TRAPPC8, TRIM21, TSC2, TSPO, UBXN2A, VDAC1, VPS25, VPS26B, VPS33B, VPS36, VPS41, WDR41, WIPI2, YOD1, ZMPSTE24]	
regulation of catabolic process	GO_BiologicalProcess-EBI-UniProt-GOA-ACAP-ARAP_13.05.2021_00h00	7.41E-13	13	143	[AGO2, APEX1, APP, ARAF, ATG4B, ATG7, ATP6V0A1, ATP6V0C, ATP6V1A, ATP6V1B2, CASP3, CCDC22, CD81, CDK5RAP3, CIRBP, CLU, CNOT1, CNOT10, CTSA, CTSC, CYP51A1, DAB2, DAGLB, DAPK1, DCN, DCPS, DHX34, DHX36, DNM1L, EIF4G3, EIF6, EZR, FBXW8, FMR1, FTO, GAPDH, GIPC1, GLMN, GPSM1, GSK3B, HDAC4, HGS, HMOX1, HNRNPA0, HNRNPC, HNRNPR, HSP90AA1, IGF2BP1, IGF2BP2, IKBKG, ITGB1, LARP1, LARP4B, LRPPRC, MAD2L1, METTL14, MLH1, MOV10, MSN, MTCH1, MYD88, NDUFA13, NEDD4, NNT, NOCT, NOP53, NPM1, NT5C3B, NUB1, NUDT15, NUP205, NUP35, NUP62, PACSIN3, PAFAH1B2, PAIP1, PIK3C2A, PIK3CA, PIN1, PIP4K2B, PITHD1, PML, PPP1CA, PRKAA1, PRKAB2,	No change

					PSMA3, PSMB10, PSMB2, PSMC2, PSMC3, PSMC6, PSMD1, PSMD12, PSMD14, PSMD2, PSMD6, PSMD7, PSMD8, PSME1, PSME2, PSME4, PYCARD, QSOX1, RAB8A, RDX, RHEB, RIPK2, RNF40, RPL5, RYBP, SCARB2, SCFD1, SERPINE2, SESN2, SET, SH3BP4, SIRT1, SLC25A12, SLC25A4, SLC25A5, SMARCC1, SNF8, SNX1, SNX5, SUFU, TAB2, TBK1, TIGAR, TRIM21, TSC2, TSPO, TUT4, UBXN2A, USP5, USP7, VDAC1, VPS26B, WDR41, WWP2, YBX1, ZC3HAV1, ZFP36L2, ZMPSTE24]	
macroautophagy	GO_BiologicalProcess-EBI-UniProt-GOA-ACAP-ARAP_13.05.2021_00h00	7.41E-13	15.759313	55	[ATG4B, ATG7, ATP6V0A1, ATP6V0C, ATP6V1A, ATP6V1B2, AUP1, CASP3, CHMP3, DYNC1LI1, DYNLL1, GAPDH, GPSM1, HGS, HMOX1, IKBKG, LARP1, MAP1LC3A, MFN1, NEDD4, PAFAH1B2, PGAM5, PIK3CA, PIP4K2B, PRKAA1, PRKAB2, QSOX1, RAB1A, RAB23, RAB5A, RHEB, RIPK2, SCFD1, SESN2, SIRT1, SLC25A4, SLC25A5, SNF8, SNX5, SQSTM1, SRC, TBK1, TIGAR, TOMM7, TRAPPC8, TSC2, UBXN2A, VDAC1, VPS25, VPS26B, VPS33B, VPS36, VPS41, WIPI2, YOD1]	No change
regulation of cellular catabolic process	GO_BiologicalProcess-EBI-UniProt-GOA-ACAP-ARAP_13.05.2021_00h00	7.41E-13	13.376483	124	[AGO2, APEX1, APP, ARAF, ATG7, ATP6V0A1, ATP6V0C, ATP6V1A, ATP6V1B2, CASP3, CCDC22, CD81, CDK5RAP3, CIRBP, CLU, CNOT1, CNOT10, CTSA, CTSC, DAB2, DAGLB, DAPK1, DCN, DCPS, DHX34, DHX36, DNM1L, EIF4G3, EZR, FBXW8, FMR1, FTO, GAPDH, GIPC1, GPSM1, GSK3B, HMOX1, HNRNPA0, HNRNPC, HNRNPR, HSP90AA1, IGF2BP1, IGF2BP2, IKBKG, LARP1, LARP4B, LRPPRC, METTL14, MLH1, MOV10,	No change

					MSN, MTCH1, MYD88, NEDD4, NNT, NOCT, NOP53, NPM1, NT5C3B, NUB1, NUDT15, PACSIN3, PAFAH1B2, PAIP1, PIK3C2A, PIK3CA, PIP4K2B, PITHD1, PML, PPP1CA, PRKAA1, PRKAB2, PSMA3, PSMB10, PSMB2, PSMC2, PSMC3, PSMC6, PSMD1, PSMD12, PSMD14, PSMD2, PSMD6, PSMD7, PSMD8, PSME1, PSME2, PSME4, PYCARD, QSOX1, RAB8A, RDX, RHEB, RIPK2, RNF40, RPL5, RYBP, SCARB2, SCFD1, SESN2, SET, SH3BP4, SIRT1, SLC25A4, SLC25A5, SMARCC1, SNX5, SUFU, TAB2, TBK1, TIGAR, TRIM21, TSC2, TSPO, TUT4, USP5, USP7, VDAC1, VPS26B, WDR41, YBX1, ZC3HAV1, ZFP36L2, ZMPSTE24]	
regulation of macroautophagy	GO_BiologicalProcess-EBI-UniProt-GOA-ACAP-ARAP_13.05.2021_00h00	7.41E-13	18.404907	30	[ATP6V0A1, ATP6V0C, ATP6V1A, ATP6V1B2, CASP3, GAPDH, GPSM1, HMOX1, IKBKG, LARP1, NEDD4, PAFAH1B2, PIK3CA, PIP4K2B, PRKAA1, PRKAB2, QSOX1, RHEB, RIPK2, SCFD1, SESN2, SIRT1, SLC25A4, SLC25A5, SNX5, TBK1, TIGAR, TSC2, VDAC1, VPS26B]	No change
extracellular space	GO_CellularComponent-EBI-UniProt-GOA-ACAP-ARAP_13.05.2021_00h00	1.21E-20	12.060302	432	[AARS1, AASDHPPT, ABCC1, ABI1, ACAT2, ACO1, ACOT7, ACSL4, ACTB, ACTG1, ACTR2, ACTR3, ADAM10, ADNP, ADSL, AGO2, AGRN, AHCTF1, AK1, AKR1B1, ALB, ALCAM, ALDH2, ALDOA, ANKFY1, ANO6, ANXA3, ANXA5, ANXA7, APAF1, APLP2, APP, APPL2, APRT, ARHGEF12, ARPC4, ARPC5L, ASAH1, ATP1A1, ATP1B3, ATP2B1, ATP2B2, ATP6V0A1, ATP6V0C, ATP6V1A, ATP6V1B2, ATXN10, AUP1, B2M, B3GAT3, B4GALT1, BAIAP2, BCAM, BCR, BDH2, BLVRB, BMP1, BST1, C1QBP, CANX,	No change

					CAP1, CAPN5, CAPZA2, CBR1, CC2D1A, CCN2, CCT2, CCT3, CCT6A, CD109, CD81, CDC42BPA, CDH11, CDK1, CFAP20, CFL2, CHAMP1, CHID1, CHMP3, CHORDC1, CHP1, CKB, CLIC1, CLTC, CLTCL1, CLU, CMPK1, CNOT1, COBLL1, COL11A1, COL12A1, COL15A1, COL18A1, COL4A1, COL5A1, COL5A2, COL6A1, COLEC12, CORO1B, CPD, CPNE2, CRABP2, CRTAP, CRYZ, CSPG4, CSTB, CTNNB1, CTSA, CTSC, CUL4B, CXCL12, CYRIB, DARS1, DCN, DCXR, DHX36, DICER1, DIPK2A, DPP3, DPYSL2, DPYSL3, DSP, DUT, ECE1, EEF1G, EFEMP1, EIF2S1, EIF3B, EIF3E, EIF6, ENO2, ENPP1, EPB41L2, EPHB4, ERAP1, ERP44, EZR, FAM3A, FASN, FAT1, FGF2, FKBP5, FLRT2, FN1, FSCN1, FTH1, FTL, FUT8, G6PD, GALM, GALNT7, GAPDH, GARS1, GBE1, GEMIN4, GET3, GGCT, GIPC1, GIPC2, GLA, GLE1, GLMN, GLO1, GLUL, GNB4, GOLGA7, GOLT1B, GOT1, GPC1, GPC6, GPD1L, GPI, GSDMD, GSTCD, GSTM2, GSTM3, GSTP1, HEBP1, HEXB, HGS, HINT1, HLA-A, HLA-B, HLA-C, HMOX1, HNRNPC, HPX, HSP90AA1, HSP90B1, HSPA4, HSPA5, HSPE1, HSPG2, IDH2, IGFBP7, IST1, ITGA3, ITGAV, ITGB1, ITGB5, ITIH4, ITM2C, JAM3, KDSR, KPNB1, KRT18, KRT19, KRT8, LAMA5, LAMC1, LAMP1, LAP3, LDHA, LGALS3BP, LRCH4, LRRC32, LRRC57, LUM, LYPLA2, MACROH2A1, MAN2B2, MCAM, MEST, MGAT1, MME, MOGS, MOV10, MPST, MSN, MTAP, MTCH1, MTHFD2, MVP, MXRA5, MYH10, MYH9, MYO1B,	

					MYO1D, MYO1E, MYO6, MYOF, NCAPG, NCKAP1, NCL, NCOA5, NDRG1, NDUFAF7, NECTIN2, NEDD4, NEDD8, NENF, NIBAN1, NIBAN2, NT5C, NUCB2, NUDT19, NUTF2, OSBPL1A, OXSR1, P3H1, PACSIN3, PAFAH1B1, PAFAH1B2, PAICS, PARP4, PCBP3, PDCD6IP, PEBP1, PFAS, PHGDH, PHPT1, PIK3C2A, PKLR, PKM, PLA2G15, PLBD2, PLD3, PLEC, PLOD2, PLXNB2, PNP, POP1, PPIB, PPP1CA, PPP1R7, PRDX1, PRDX6, PRKCI, PROCR, PRXL2B, PSAT1, PSMA3, PSMB2, PSMC6, PSMD12, PSMD2, PSMD7, PSME1, PSME2, PTGIS, PTGR1, PTPA, PTPRF, PTPRG, PTRH2, PYGB, QDPR, QSOX1, RAB10, RAB11B, RAB1A, RAB3B, RAB3D, RAB5A, RAB8A, RAC3, RALA, RAP1GDS1, RAP2B, RAP2C, RDX, RHEB, RHOB, RHOC, RIPK2, RPL10A, RPL22, RPL24, RPL27, RPL5, RPLP2, RPS13, RPS15A, RPS16, RPS18, RPS5, RPS8, RPS9, RRAS2, S100A10, S100A16, SARS1, SCARB2, SCPEP1, SCRIB, SEC14L2, SELENBP1, SEMA3C, SEPTIN2, SEPTIN7, SEPTIN9, SERPINB1, SERPINB2, SERPINB8, SERPINB9, SERPINE2, SGSH, SH3BGRL3, SH3BP4, SHMT2, SIAE, SLC12A2, SLC1A1, SLC1A4, SLC1A5, SLC26A2, SLC2A1, SLC44A1, SLC44A2, SLC9A1, SLIT2, SMC2, SMPD1, SNF8, SNX18, SOAT1, SOD1, SOD2, SPARC, SQSTM1, SRC, SRPRA, STK24, STOM, STXBP3, SYNE2, TAF6L, TAGLN2, TALDO1, TAX1BP1, TBC1D10A, TCP1, TF,	

					TGFBI, TGFBR3, TGM2, TGS1, THBS1, TKT, TMED9, TNIK, TOM1, TOM1L2, TOR1B, TPI1, TPM3, TPP1, TPRG1L, TSPO, TUBA4A, TUBB, TUBB3, TUT4, TWF2, TXN, TXNDC17, UACA, UGP2, VASP, VAT1, VDAC1, VLDLR, VPS25, VPS36, VTN, WDR1, WWP2, XYLT2, YARS1, YBX1, YES1, ZMPSTE24]	
vesicle-mediated transport	GO_BiologicalProcess-EBI-UniProt-GOA-ACAP-ARAP_13.05.2021_00h00	1.21E-20	11.466897	265	[ABI1, ACAP2, ACSL3, ACTB, ACTG1, ACTR2, ACTR3, ADAM10, ADAMTS9, AGPAT2, ALB, ALDOA, ANK3, ANKFY1, ANKRD13A, ANO6, ANXA3, ANXA5, AP3M2, AP5B1, APAF1, APEX1, APLP2, APP, APPL2, APRT, ARFGAP1, ARFGEF1, ARFIP1, ARHGAP12, ARHGAP17, ARPC4, ARRB1, ASAH1, ATG7, ATL3, ATP2C1, ATP6V0A1, ATP6V0C, B2M, B4GALT1, BAIAP2, BET1, BIN1, BNIP1, BST1, CANX, CAP1, CAPZA2, CASK, CCDC22, CCT2, CD109, CD81, CDK16, CECR2, CHAMP1, CHID1, CHMP3, CHORDC1, CHP1, CLTC, CLTCL1, CLU, COG6, COG7, COLEC12, CORO1C, CORO7, CSTB, CTNNB1, CTSA, CTSC, CUX1, CYB5R3, CYRIB, DAB2, DENND2A, DIAPH1, DNM1L, DPYSL2, DSP, DYNC1LI1, DYNLL1, EHD3, EIF2AK2, ENPP1, EPHA3, ERP44, EXOC2, EZR, FAM91A1, FMR1, FN1, FNBP1L, FTH1, FTL, GCC2, GGA2, GIPC1, GLA, GOLGA7, GOLT1B, GPI, GSDMD, GSK3B, GSTP1, GULP1, HEXB, HGS, HIP1, HLA-B, HLA-C, HMOX1, HNRNPC, HPX, HSP90AA1, HSP90B1, HSPA14, HSPG2, IST1, ITGAV, ITGB1, ITIH4, ITM2C, ITSN1, JAGN1, KIF15, KPNB1, KRT18, LAMP1, LGALS3BP,	No change

					LIN7C, LLGL1, LRP10, M6PR, MACF1, MAGED2, MGST1, MICAL3, MICALL1, MME, MSN, MTCH1, MVP, MYD88, MYH9, MYO18A, MYO1B, MYO1D, MYO1E, MYO6, NCKAP1, NEDD4, NHLRC2, NUDT19, OSBPL1A, PACSIN3, PAFAH1B2, PCDH7, PDAP1, PDCD6IP, PIK3C2A, PIK3CA, PIP4K2B, PKM, PLCG1, PLD1, PLPP3, PNP, PPIE, PPP3CA, PRDX6, PRKAA1, PRKCI, PRKCSH, PSMC2, PSMC3, PSMD1, PSMD12, PSMD14, PSMD2, PSMD6, PSMD7, PTGES2, PYCARD, PYGB, QSOX1, RAB10, RAB11B, RAB1A, RAB3B, RAB3D, RAB5A, RAB8A, RABGEF1, RALA, RAP2B, RAP2C, RDX, RHOB, RIN1, S100A10, SCARB2, SCFD1, SCRIB, SCRN1, SEC14L2, SEC23B, SEPTIN2, SEPTIN8, SEPTIN9, SERPINB1, SH3BP4, SH3KBP1, SLC1A1, SLC44A2, SMPD1, SNF8, SNX1, SNX18, SNX27, SNX5, SOD1, SORT1, SPARC, SPHK2, SQSTM1, SRC, STOM, STON1, STXBP3, SYNJ1, TAGLN2, TAP1, TBC1D10A, TBC1D17, TBC1D4, TBC1D8B, TF, TGM2, THBS1, TM9SF4, TMED9, TMX3, TOM1, TRAPPC4, TRAPPC8, TSC2, TUBA4A, TUBB, UBR4, USP7, VAT1, VLDLR, VPS25, VPS26B, VPS33B, VPS36, VPS41, VTN, WDR1, WDR11, YES1, YKT6]	
vesicle	GO_CellularComponent-EBI-UniProt-GOA-ACAP-ARAP_13.05.2021_00h00	1.21E-20	12.430873	517	[AARS1, AASDHPPT, ABCC1, ABI1, ACAP2, ACAT2, ACO1, ACOT7, ACSL4, ACTB, ACTG1, ACTR2, ACTR3, ADAM10, ADSL, AGO2, AGPAT2, AGRN, AHCTF1, AK1, AKR1B1, ALB, ALCAM, ALDH2, ALDOA, AMOTL1, ANKFY1, ANKRD13A,	No change

					ANO6, ANXA3, ANXA5, ANXA7, AP3M2, APAF1, APEX1, APLP2, APP, APPL2, APRT, ARHGEF12, ARPC4, ARPC5L, ARRB1, ASAH1, ASPSCR1, ATG7, ATP1A1, ATP1B3, ATP2B1, ATP2B2, ATP6V0A1, ATP6V0C, ATP6V1A, ATP6V1B2, AUP1, B2M, B3GAT3, B4GALT1, BAIAP2, BCAM, BCR, BDH2, BET1, BIN1, BLVRB, BMP1, BNIP1, BST1, CAMK2D, CANX, CAP1, CAPN5, CAPZA2, CASK, CBR1, CC2D1A, CCDC22, CCT2, CCT3, CCT6A, CD109, CD81, CDC42BPA, CDH11, CDK1, CDK16, CDK2, CEMIP, CFAP20, CFL2, CHAMP1, CHID1, CHMP3, CHORDC1, CHP1, CKB, CLIC1, CLTC, CLTCL1, CLU, CMPK1, CNGB1, COBLL1, COL12A1, COL15A1, COL18A1, COL6A1, COLEC12, CORO1B, CORO1C, CORO7, CPD, CPNE2, CRABP2, CRYZ, CSPG4, CSTB, CTNNB1, CTSA, CTSC, CUL4B, CXCL12, CYB5R3, CYRIB, DAB2, DARS1, DCXR, DERL1, DHX36, DIAPH1, DICER1, DIPK2A, DNM1L, DPP3, DPYSL2, DPYSL3, DSP, DUT, DVL2, DYNC1LI1, DYNLL1, ECE1, EEF1G, EFEMP1, EHD3, EIF2S1, EIF3B, EIF3E, EIF6, ENO2, EPB41L2, EPHA3, EPHB4, ERAP1, ERBB2, ERP44, EZR, FAM3A, FAM91A1, FASN, FAT1, FBLN2, FKBP5, FLRT2, FN1, FNBP1L, FSCN1, FTH1, FTL, FUT8, G6PD, GALM, GALNT7, GAPDH, GARS1, GBE1, GBP1, GEMIN4, GET3, GGA2, GGCT, GIPC1, GIPC2, GLA, GLO1, GLUL, GNB4, GOLGA1, GOLGA7, GOLT1B, GOT1, GPC1, GPD1L, GPI, GRAMD1A, GSDMD,	

					GSTCD, GSTM2, GSTM3, GSTP1, HEBP1, HEXB, HGS, HINT1, HIP1, HLA-A, HLA-B, HLA-C, HNRNPC, HPX, HSP90AA1, HSP90B1, HSPA4, HSPA5, HSPE1, HSPG2, IDH2, IGFBP7, INPP4A, IST1, ITGA3, ITGAV, ITGB1, ITGB5, ITIH4, ITM2C, ITSN1, KCNQ1, KPNB1, KRT18, KRT19, KRT8, LAMA5, LAMC1, LAMP1, LAP3, LDHA, LGALS3BP, LLGL1, LNPEP, LRCH4, LRRC57, LUM, LYPLA2, M6PR, MACROH2A1, MAGED2, MAN2B2, MAP1LC3A, MEST, MFF, MGAT1, MGST1, MICALL1, MME, MOGS, MPST, MSN, MTAP, MTCH1, MVP, MXRA5, MYD88, MYH10, MYH9, MYO1B, MYO1D, MYO1E, MYO6, MYOF, NCAPG, NCDN, NCK2, NCKAP1, NCL, NDRG1, NECTIN2, NEDD4, NEDD8, NF2, NHLRC2, NIBAN1, NIBAN2, NISCH, NT5C, NUCB2, NUDT19, NUTF2, OSBPL11, OSBPL1A, OXSR1, P3H1, PACS1, PACSIN3, PAFAH1B1, PAFAH1B2, PAICS, PARP4, PCBP3, PCDH7, PDAP1, PDCD6IP, PEBP1, PFAS, PHGDH, PHPT1, PI4K2B, PIGT, PIK3C2A, PITHD1, PKLR, PKM, PLA2G15, PLBD2, PLD1, PLD3, PLEC, PLOD2, PLXNB2, PML, PNP, PPIB, PPIE, PPP1CA, PPP1R7, PRDX1, PRDX6, PRKCI, PRKCSH, PROCR, PRXL2B, PSAT1, PSMA3, PSMB2, PSMC2, PSMC3, PSMC6, PSMD1, PSMD12, PSMD14, PSMD2, PSMD6, PSMD7, PSME1, PSME2, PTGES2, PTGR1, PTPA, PTPRF, PTPRG, PYCARD, PYGB, QDPR, QSOX1, RAB10, RAB11B,	

					RAB1A, RAB23, RAB3B, RAB3D, RAB5A, RAB8A, RABGEF1, RAC3, RALA, RAP1GDS1, RAP2B, RAP2C, RDX, REEP6, RELCH, RHEB, RHOB, RHOC, RIPK2, RPL10A, RPL22, RPL24, RPL27, RPL5, RPLP2, RPS13, RPS15A, RPS16, RPS18, RPS5, RPS8, RPS9, RRAS2, S100A10, S100A16, SARS1, SCARB2, SCFD1, SCPEP1, SCRIB, SEC14L2, SEC23B, SELENBP1, SEMA3C, SEPTIN2, SEPTIN7, SEPTIN8, SEPTIN9, SERPINB1, SERPINB8, SERPINB9, SERPINE2, SGSH, SH3BGRL3, SH3BP4, SH3KBP1, SHMT2, SIAE, SLC11A2, SLC12A2, SLC17A5, SLC1A1, SLC1A4, SLC1A5, SLC26A2, SLC2A1, SLC30A9, SLC31A1, SLC44A1, SLC44A2, SLC4A7, SLC9A1, SLIT2, SMC2, SMPD1, SNF8, SNX1, SNX18, SNX21, SNX27, SNX5, SOAT1, SOD1, SOD2, SORT1, SPARC, SQSTM1, SRC, SRPRA, STK24, STOM, STON1, STXBP3, SYNE2, SYNJ1, SYPL2, TAB2, TAF6L, TAGLN2, TALDO1, TAP1, TAX1BP1, TBC1D10A, TBC1D17, TBC1D4, TCP1, TF, TGFBI, TGFBR3, TGM2, THBS1, TKT, TM9SF4, TMED9, TMEM168, TMEM192, TMX3, TNIK, TOM1, TOM1L2, TOR1B, TPI1, TPM3, TPP1, TPRG1L, TRAPPC4, TRAPPC8, TRIM21, TSPO, TUBA4A, TUBB, TUBB3, TUT4, TWF2, TXN, TXNDC17, UACA, UBR4, UGP2, UNC93B1, VASP, VAT1, VDAC1, VDAC2, VPS25, VPS26B, VPS33B, VPS36, VPS41, VTN, WASHC5, WDR1, WDR11, WDR44, WFS1, WWP2, YBX1, YES1, YKT6,	

					ZDHHC13, ZMPSTE24]	
extracellular exosome	GO_CellularComponent-EBI-UniProt-GOA-ACAP-ARAP_13.05.2021_00h00	1.21E-20	17.025011	388	[AARS1, AASDHPPT, ABCC1, ABI1, ACAT2, ACO1, ACOT7, ACSL4, ACTB, ACTG1, ACTR2, ACTR3, ADAM10, ADSL, AGO2, AGRN, AHCTF1, AK1, AKR1B1, ALB, ALCAM, ALDH2, ALDOA, ANKFY1, ANO6, ANXA3, ANXA5, ANXA7, APAF1, APLP2, APP, APPL2, APRT, ARHGEF12, ARPC4, ARPC5L, ASAH1, ATP1A1, ATP1B3, ATP2B1, ATP2B2, ATP6V0A1, ATP6V0C, ATP6V1A, ATP6V1B2, AUP1, B2M, B3GAT3, B4GALT1, BAIAP2, BCAM, BCR, BDH2, BLVRB, BST1, CANX, CAP1, CAPN5, CAPZA2, CBR1, CC2D1A, CCT2, CCT3, CCT6A, CD81, CDC42BPA, CDH11, CDK1, CFAP20, CFL2, CHAMP1, CHID1, CHMP3, CHORDC1, CHP1, CKB, CLIC1, CLTC, CLTCL1, CLU, CMPK1, COBLL1, COL12A1, COL15A1, COL18A1, COL6A1, CORO1B, CPD, CPNE2, CRABP2, CRYZ, CSPG4, CSTB, CTNNB1, CTSA, CTSC, CUL4B, CXCL12, CYRIB, DARS1, DCXR, DHX36, DICER1, DPP3, DPYSL2, DSP, DUT, ECE1, EEF1G, EFEMP1, EIF2S1, EIF3B, EIF3E, EIF6, ENO2, EPB41L2, EPHB4, ERAP1, ERP44, EZR, FASN, FAT1, FKBP5, FLRT2, FN1, FSCN1, FTH1, FTL, FUT8, G6PD, GALM, GALNT7, GAPDH, GARS1, GBE1, GEMIN4, GET3, GGCT, GIPC1, GIPC2, GLA, GLO1, GLUL, GNB4, GOLGA7, GOLT1B, GOT1, GPC1, GPD1L, GPI, GSTCD, GSTM2, GSTM3, GSTP1, HEBP1, HEXB, HGS, HINT1, HLA-A, HLA-B, HLA-C, HNRNPC, HPX, HSP90AA1, HSP90B1, HSPA4, HSPA5, HSPE1,	No change

					HSPG2, IDH2, IGFBP7, IST1, ITGA3, ITGAV, ITGB1, ITGB5, ITIH4, ITM2C, KPNB1, KRT18, KRT19, KRT8, LAMA5, LAMC1, LAMP1, LAP3, LDHA, LGALS3BP, LRCH4, LRRC57, LUM, LYPLA2, MACROH2A1, MAN2B2, MEST, MGAT1, MME, MOGS, MPST, MSN, MTAP, MTCH1, MVP, MXRA5, MYH10, MYH9, MYO1B, MYO1D, MYO1E, MYO6, MYOF, NCAPG, NCKAP1, NCL, NDRG1, NECTIN2, NEDD4, NEDD8, NIBAN1, NIBAN2, NT5C, NUCB2, NUDT19, NUTF2, OSBPL1A, OXSR1, P3H1, PACSIN3, PAFAH1B1, PAFAH1B2, PAICS, PARP4, PCBP3, PDCD6IP, PEBP1, PFAS, PHGDH, PHPT1, PIK3C2A, PKLR, PKM, PLA2G15, PLBD2, PLD3, PLEC, PLOD2, PLXNB2, PNP, PPIB, PPP1CA, PPP1R7, PRDX1, PRDX6, PRKCI, PROCR, PRXL2B, PSAT1, PSMA3, PSMB2, PSMC6, PSMD12, PSMD2, PSMD7, PSME1, PSME2, PTGR1, PTPA, PTPRF, PTPRG, PYGB, QDPR, QSOX1, RAB10, RAB11B, RAB1A, RAB3B, RAB3D, RAB5A, RAB8A, RAC3, RALA, RAP1GDS1, RAP2B, RAP2C, RDX, RHEB, RHOB, RHOC, RPL10A, RPL22, RPL24, RPL27, RPL5, RPLP2, RPS13, RPS15A, RPS16, RPS18, RPS5, RPS8, RPS9, RRAS2, S100A10, S100A16, SARS1, SCARB2, SCPEP1, SCRIB, SEC14L2, SELENBP1, SEMA3C, SEPTIN2, SEPTIN7, SEPTIN9, SERPINB1, SERPINB8, SERPINB9, SGSH, SH3BGRL3, SH3BP4, SHMT2, SIAE, SLC12A2, SLC1A1,	

					SLC1A4, SLC1A5, SLC26A2, SLC2A1, SLC44A1, SLC44A2, SLC9A1, SLIT2, SMC2, SMPD1, SNF8, SNX18, SOAT1, SOD1, SOD2, SQSTM1, SRC, SRPRA, STK24, STOM, STXBP3, SYNE2, TAF6L, TAGLN2, TALDO1, TAX1BP1, TBC1D10A, TCP1, TF, TGFBI, TGFBR3, TGM2, THBS1, TKT, TMED9, TNIK, TOM1, TOM1L2, TOR1B, TPI1, TPM3, TPP1, TPRG1L, TSPO, TUBA4A, TUBB, TUBB3, TUT4, TWF2, TXN, TXNDC17, UACA, UGP2, VASP, VAT1, VDAC1, VPS25, VPS36, VTN, WDR1, WWP2, YBX1, YES1, ZMPSTE24]	
intracellular vesicle	GO_CellularComponent-EBI-UniProt-GOA-ACAP-ARAP_13.05.2021_00h00	1.21E-20	10.53453	270	[ACAP2, ACTG1, ACTR2, ADAM10, AGPAT2, ALB, ALDOA, AMOTL1, ANKFY1, ANKRD13A, ANO6, ANXA3, ANXA7, AP3M2, APAF1, APEX1, APLP2, APP, APPL2, APRT, ARRB1, ASAH1, ATG7, ATP1A1, ATP1B3, ATP2B1, ATP6V0A1, ATP6V0C, ATP6V1A, ATP6V1B2, AUP1, B2M, B4GALT1, BAIAP2, BET1, BIN1, BNIP1, BST1, CAMK2D, CANX, CAP1, CCDC22, CCT2, CD109, CDK16, CDK2, CEMIP, CHAMP1, CHID1, CHMP3, CHORDC1, CHP1, CLTC, CLTCL1, CLU, CNGB1, COLEC12, CORO1C, CORO7, CSTB, CTSA, CTSC, CYB5R3, CYRIB, DAB2, DERL1, DIAPH1, DIPK2A, DNM1L, DPYSL3, DSP, DVL2, DYNC1LI1, DYNLL1, ECE1, EHD3, EPHA3, ERBB2, ERP44, EZR, FAM3A, FAM91A1, FASN, FN1, FNBP1L, FTH1, FTL, GARS1, GBP1, GGA2, GIPC1, GLA, GOLGA1, GOLGA7, GPC1, GPI, GRAMD1A, GSDMD, GSTP1, HEXB, HGS, HIP1, HLA-A, HLA-B,	No change

					HLA-C, HPX, HSP90AA1, HSP90B1, HSPA5, INPP4A, IST1, ITGAV, ITGB1, ITGB5, ITIH4, ITM2C, ITSN1, KCNQ1, KPNB1, LAMP1, LGALS3BP, LLGL1, LNPEP, M6PR, MAGED2, MAP1LC3A, MFF, MGST1, MICALL1, MME, MTCH1, MVP, MYD88, MYH9, MYO1B, MYO1D, MYO1E, MYO6, MYOF, NCAPG, NCDN, NDRG1, NF2, NHLRC2, NISCH, OSBPL11, OSBPL1A, PACS1, PACSIN3, PAFAH1B2, PCDH7, PDAP1, PDCD6IP, PI4K2B, PIGT, PIK3C2A, PITHD1, PKM, PLD1, PLD3, PML, PNP, PPIB, PPIE, PRDX1, PRDX6, PRKCI, PRKCSH, PSMC2, PSMC3, PSMD1, PSMD12, PSMD14, PSMD2, PSMD6, PSMD7, PTGES2, PYCARD, PYGB, QSOX1, RAB10, RAB11B, RAB1A, RAB23, RAB3B, RAB3D, RAB5A, RAB8A, RABGEF1, RAC3, RALA, RAP2B, RAP2C, REEP6, RELCH, RHOB, RHOC, SCARB2, SCFD1, SEC14L2, SEC23B, SEPTIN2, SEPTIN8, SERPINB1, SERPINE2, SH3BP4, SH3KBP1, SLC11A2, SLC12A2, SLC17A5, SLC1A1, SLC1A4, SLC1A5, SLC2A1, SLC30A9, SLC31A1, SLC44A2, SLC4A7, SMPD1, SNF8, SNX1, SNX18, SNX21, SNX27, SNX5, SOD1, SORT1, SPARC, SQSTM1, SRC, STOM, STON1, STXBP3, SYPL2, TAB2, TAP1, TBC1D17, TCP1, TF, THBS1, TM9SF4, TMED9, TMEM168, TMEM192, TMX3, TNIK, TOM1, TOM1L2, TPP1, TPRG1L, TRAPPC4, TRAPPC8, TRIM21, TUBB, UBR4, UNC93B1, VAT1, VDAC1, VDAC2, VPS25, VPS26B, VPS33B, VPS36,	

					VPS41, WASHC5, WDR11, WDR44, WFS1, YKT6, ZDHHC13]	
extracellular vesicle	GO_CellularComponent-EBI-UniProt-GOA-ACAP-ARAP_13.05.2021_00h00	1.21E-20	17	391	[AARS1, AASDHPPT, ABCC1, ABI1, ACAT2, ACO1, ACOT7, ACSL4, ACTB, ACTG1, ACTR2, ACTR3, ADAM10, ADSL, AGO2, AGRN, AHCTF1, AK1, AKR1B1, ALB, ALCAM, ALDH2, ALDOA, ANKFY1, ANO6, ANXA3, ANXA5, ANXA7, APAF1, APLP2, APP, APPL2, APRT, ARHGEF12, ARPC4, ARPC5L, ASAH1, ATP1A1, ATP1B3, ATP2B1, ATP2B2, ATP6V0A1, ATP6V0C, ATP6V1A, ATP6V1B2, AUP1, B2M, B3GAT3, B4GALT1, BAIAP2, BCAM, BCR, BDH2, BLVRB, BST1, CANX, CAP1, CAPN5, CAPZA2, CBR1, CC2D1A, CCT2, CCT3, CCT6A, CD81, CDC42BPA, CDH11, CDK1, CFAP20, CFL2, CHAMP1, CHID1, CHMP3, CHORDC1, CHP1, CKB, CLIC1, CLTC, CLTCL1, CLU, CMPK1, COBLL1, COL12A1, COL15A1, COL18A1, COL6A1, CORO1B, CPD, CPNE2, CRABP2, CRYZ, CSPG4, CSTB, CTNNB1, CTSA, CTSC, CUL4B, CXCL12, CYRIB, DARS1, DCXR, DHX36, DICER1, DPP3, DPYSL2, DSP, DUT, ECE1, EEF1G, EFEMP1, EIF2S1, EIF3B, EIF3E, EIF6, ENO2, EPB41L2, EPHB4, ERAP1, ERP44, EZR, FASN, FAT1, FBLN2, FKBP5, FLRT2, FN1, FSCN1, FTH1, FTL, FUT8, G6PD, GALM, GALNT7, GAPDH, GARS1, GBE1, GEMIN4, GET3, GGCT, GIPC1, GIPC2, GLA, GLO1, GLUL, GNB4, GOLGA7, GOLT1B, GOT1, GPC1, GPD1L, GPI, GSTCD, GSTM2, GSTM3, GSTP1, HEBP1, HEXB, HGS, HINT1, HLA-A, HLA-B, HLA-C, HNRNPC,	No change

					HPX, HSP90AA1, HSP90B1, HSPA4, HSPA5, HSPE1, HSPG2, IDH2, IGFBP7, IST1, ITGA3, ITGAV, ITGB1, ITGB5, ITIH4, ITM2C, KPNB1, KRT18, KRT19, KRT8, LAMA5, LAMC1, LAMP1, LAP3, LDHA, LGALS3BP, LRCH4, LRRC57, LUM, LYPLA2, MACROH2A1, MAN2B2, MEST, MGAT1, MME, MOGS, MPST, MSN, MTAP, MTCH1, MVP, MXRA5, MYH10, MYH9, MYO1B, MYO1D, MYO1E, MYO6, MYOF, NCAPG, NCKAP1, NCL, NDRG1, NECTIN2, NEDD4, NEDD8, NIBAN1, NIBAN2, NT5C, NUCB2, NUDT19, NUTF2, OSBPL1A, OXSR1, P3H1, PACSIN3, PAFAH1B1, PAFAH1B2, PAICS, PARP4, PCBP3, PDCD6IP, PEBP1, PFAS, PHGDH, PHPT1, PIK3C2A, PKLR, PKM, PLA2G15, PLBD2, PLD3, PLEC, PLOD2, PLXNB2, PNP, PPIB, PPP1CA, PPP1R7, PRDX1, PRDX6, PRKCI, PROCR, PRXL2B, PSAT1, PSMA3, PSMB2, PSMC6, PSMD12, PSMD2, PSMD7, PSME1, PSME2, PTGR1, PTPA, PTPRF, PTPRG, PYGB, QDPR, QSOX1, RAB10, RAB11B, RAB1A, RAB3B, RAB3D, RAB5A, RAB8A, RAC3, RALA, RAP1GDS1, RAP2B, RAP2C, RDX, RHEB, RHOB, RHOC, RPL10A, RPL22, RPL24, RPL27, RPL5, RPLP2, RPS13, RPS15A, RPS16, RPS18, RPS5, RPS8, RPS9, RRAS2, S100A10, S100A16, SARS1, SCARB2, SCPEP1, SCRIB, SEC14L2, SELENBP1, SEMA3C, SEPTIN2, SEPTIN7, SEPTIN9, SERPINB1, SERPINB8, SERPINB9,	

					SERPINE2, SGSH, SH3BGRL3, SH3BP4, SHMT2, SIAE, SLC11A2, SLC12A2, SLC1A1, SLC1A4, SLC1A5, SLC26A2, SLC2A1, SLC44A1, SLC44A2, SLC9A1, SLIT2, SMC2, SMPD1, SNF8, SNX18, SOAT1, SOD1, SOD2, SQSTM1, SRC, SRPRA, STK24, STOM, STXBP3, SYNE2, TAF6L, TAGLN2, TALDO1, TAX1BP1, TBC1D10A, TCP1, TF, TGFBI, TGFBR3, TGM2, THBS1, TKT, TMED9, TNIK, TOM1, TOM1L2, TOR1B, TPI1, TPM3, TPP1, TPRG1L, TSPO, TUBA4A, TUBB, TUBB3, TUT4, TWF2, TXN, TXNDC17, UACA, UGP2, VASP, VAT1, VDAC1, VPS25, VPS36, VTN, WDR1, WWP2, YBX1, YES1, ZMPSTE24]	
programmed cell death	GO_BiologicalProcess-EBI-UniProt-GOA-ACAP-ARAP_13.05.2021_00h00	9.65E-08	10.217983	225	[AARS1, ADAM10, ADARB1, ADNP, AKAP12, AKR1B1, ALB, ANO6, ANXA5, APAF1, APEX1, APIP, APP, ARAF, ARHGEF11, ARHGEF12, ARRB1, ASAH1, ASNS, ATG7, B4GALT1, BAG1, BIN1, BNIP1, C1QBP, C3orf38, CAMK2D, CASP3, CASP6, CCN2, CDC34, CDC73, CDK1, CDK5RAP3, CECR2, CFDP1, CHMP3, CLPTM1L, CLU, CTNNB1, CTNNBL1, CTSC, CUL1, CXCL12, CYCS, DAB2, DAPK1, DCPS, DDX42, DDX47, DHCR24, DICER1, DIPK2A, DNM1L, DNMT1, DSP, DYNLL1, EEF1E1, EIF2AK2, EIF2S1, EPB41L3, EPHA2, ERCC5, ETFDH, FASN, FGD4, FGF2, FHL2, G6PD, GAPDH, GBE1, GGCT, GLMN, GLO1, GPI, GSDMD, GSK3B, GSTP1, GULP1, HDAC2, HDAC4, HHIP, HINT1, HIP1, HK2, HMOX1, HSP90AA1, HSP90B1, HSPA5, HSPB6, HSPE1,	No change

					IGF2BP1, IKBKB, IKBKG, ITGAV, ITGB1, ITM2C, ITPK1, ITSN1, IVNS1ABP, KCNMA1, KDM1A, KPNB1, KRT18, KRT19, KRT8, LAMP1, LDHA, MAD2L1, MAEA, MALT1, MAP3K20, MAP4K4, MCM2, MCM7, MFF, MFSD10, MGMT, MLH1, MSH2, MSH6, MTCH1, MYD88, MYO18A, NCK2, NCKAP1, NDRG1, NDUFA13, NDUFS3, NES, NF1, NF2, NIBAN2, NISCH, NLE1, NMT1, NNT, NOP53, NPM1, NQO1, NUP62, OXSR1, PARP1, PAWR, PDCD6IP, PDK1, PGAM5, PIGT, PIK3CA, PIN1, PKM, PKN2, PLCG1, PML, PPP1CA, PPP3CA, PRKAA1, PRKCI, PTGIS, PTPA, PTPMT1, PTRH2, PUF60, PYCARD, QARS1, RAD21, RAI14, RHOB, RIPK2, RNF213, RNPS1, RTN4, RYBP, SAP30BP, SARM1, SCRIB, SERPINB2, SERPINB9, SH3KBP1, SIRT1, SLC1A1, SLC25A4, SLC25A5, SLC25A6, SLC9A1, SLIT2, SMNDC1, SMPD1, SNW1, SOD1, SOD2, SON, SORT1, SPHK2, SPTLC1, SQSTM1, SRA1, SRC, STAT1, STAT5B, STK24, TAX1BP1, TCP1, TGM2, THBS1, THEM4, TIGAR, TJP1, TPX2, TRADD, TRIO, TSC2, TSPO, UACA, URI1, USP28, VDAC1, VDAC2, WDR35, WFS1, XIAP, YAP1, YARS1, ZFAND6, ZMPSTE24]	
regulation of cell death	GO_BiologicalProcess-EBI-UniProt-GOA-ACAP-ARAP_13.05.2021_00h00	9.65E-08	10.773639	188	[AARS1, ADAM10, ADNP, AKAP12, AKR1B1, ALB, ANO6, ANXA5, APAF1, APEX1, APIP, ARAF, ARHGEF11, ARHGEF12, ARRB1, ASAH1, ASNS, ATG7, B4GALT1, BAG1, BIN1, BNIP1, C1QBP, C3orf38, CAMK2D, CASP3, CASP6, CCN2, CD200, CDC34, CDC73, CDK1, CFDP1,	No change

					CHORDC1, CHP1, CLU, CTNNB1, CTNNBL1, CTSC, CXCL12, DAB2, DAPK1, DCPS, DDX42, DHCR24, DIPK2A, DNM1L, DNMT1, DYNLL1, EEF1E1, EFNB2, EIF2AK2, EIF2S1, ERCC5, ETFDH, FASN, FERMT2, FGD4, FGF2, FHL2, G6PD, GBE1, GLMN, GLO1, GPI, GSK3B, GSTP1, HDAC2, HDAC4, HHIP, HMOX1, HSP90AA1, HSP90B1, HSPA5, HSPB6, IGF2BP1, IKBKB, IKBKG, IMPACT, ITGAV, ITGB1, ITM2C, ITSN1, IVNS1ABP, KCNMA1, KDM1A, KRT18, LDHA, MAD2L1, MAEA, MALT1, MAP3K20, MAP4K4, MEAK7, MGMT, MSH2, MTCH1, MYD88, MYO18A, NCK2, NCOA7, NDRG1, NDUFA13, NDUFS3, NENF, NES, NF1, NF2, NIBAN2, NLE1, NNT, NOP53, NPM1, NQO1, NUP62, OXSR1, PARP1, PAWR, PDCD6IP, PIK3CA, PIN1, PKN2, PLCG1, PML, PPP1CA, PPP3CA, PPP5C, PRKAA1, PRKCI, PTGIS, PTPA, PTPMT1, PTRH2, PYCARD, PYCR1, QARS1, RHOB, RIPK2, RNF213, RNPS1, RRAS2, RTN4, RYBP, SAP30BP, SARM1, SCRIB, SERPINB2, SERPINB9, SIRT1, SLC1A1, SLC25A24, SLC25A4, SLC25A5, SLC9A1, SLIT2, SMAD4, SMPD1, SOD1, SOD2, SON, SORT1, SPHK2, SPTLC1, SQSTM1, SRA1, SRC, STAT1, STAT5B, STK24, TAX1BP1, TBK1, TCP1, TGM2, THBS1, TIGAR, TJP1, TRADD, TRIO, TSPO, TXN, URI1, VDAC1, VDAC2, WDR35, WFS1, XIAP, YAP1, ZFAND6]	
apoptotic process	GO_BiologicalProcess-EBI-UniProt-GOA-ACAP-	9.65E-08	10.544385	215	[AARS1, ADAM10, ADARB1, ADNP, AKAP12, AKR1B1, ALB, ANO6, ANXA5,	No change

	ARAP_13.05.2021_00h00				APAF1, APEX1, APIP, APP, ARAF, ARHGEF11, ARHGEF12, ARRB1, ASNS, ATG7, B4GALT1, BAG1, BIN1, BNIP1, C1QBP, C3orf38, CAMK2D, CASP3, CASP6, CCN2, CDC34, CDC73, CDK1, CDK5RAP3, CECR2, CFDP1, CHMP3, CLPTM1L, CLU, CTNNB1, CTNNBL1, CTSC, CUL1, CXCL12, CYCS, DAB2, DAPK1, DDX42, DDX47, DHCR24, DICER1, DIPK2A, DNM1L, DNMT1, DYNLL1, EEF1E1, EIF2AK2, EIF2S1, EPB41L3, EPHA2, ERCC5, ETFDH, FASN, FGD4, FHL2, G6PD, GAPDH, GBE1, GGCT, GLMN, GLO1, GPI, GSK3B, GSTP1, GULP1, HDAC2, HDAC4, HHIP, HINT1, HIP1, HK2, HMOX1, HSP90AA1, HSP90B1, HSPA5, HSPB6, HSPE1, IGF2BP1, IKBKB, IKBKG, ITGAV, ITGB1, ITM2C, ITSN1, IVNS1ABP, KCNMA1, KDM1A, KPNB1, KRT18, KRT8, LDHA, MAD2L1, MAEA, MALT1, MAP3K20, MAP4K4, MCM2, MCM7, MFF, MFSD10, MGMT, MLH1, MSH2, MSH6, MTCH1, MYD88, MYO18A, NCK2, NCKAP1, NDRG1, NDUFA13, NDUFS3, NES, NF1, NF2, NIBAN2, NISCH, NLE1, NMT1, NNT, NOP53, NPM1, NQO1, NUP62, OXSR1, PARP1, PAWR, PDCD6IP, PDK1, PIGT, PIK3CA, PIN1, PKN2, PLCG1, PML, PPP1CA, PPP3CA, PRKAA1, PRKCI, PTGIS, PTPA, PTPMT1, PTRH2, PUF60, PYCARD, QARS1, RAD21, RAI14, RHOB, RIPK2, RNF213, RNPS1, RTN4, RYBP, SAP30BP, SARM1, SCRIB, SERPINB2, SERPINB9, SH3KBP1, SIRT1, SLC1A1, SLC25A4, SLC25A5, SLC25A6, SLC9A1,	

					SLIT2, SMNDC1, SMPD1, SNW1, SOD1, SOD2, SON, SORT1, SPHK2, SPTLC1, SQSTM1, SRA1, SRC, STAT1, STAT5B, STK24, TAX1BP1, TCP1, TGM2, THBS1, THEM4, TIGAR, TJP1, TPX2, TRADD, TRIO, TSC2, TSPO, UACA, URI1, USP28, VDAC1, VDAC2, WDR35, WFS1, XIAP, YAP1, YARS1, ZFAND6, ZMPSTE24]	
positive regulation of cell death	GO_BiologicalProcess-EBI-UniProt-GOA-ACAP-ARAP_13.05.2021_00h00	9.65E-08	12.1396055	80	[ADAM10, AKAP12, ANO6, APAF1, ARHGEF11, ARHGEF12, ARRB1, ATG7, B4GALT1, BIN1, C1QBP, C3orf38, CAMK2D, CASP3, CCN2, CDC34, CLU, CTNNB1, CTNNBL1, CTSC, DAPK1, DNM1L, DYNLL1, EEF1E1, EFNB2, EIF2S1, FASN, FGD4, GLMN, GSK3B, HDAC4, HMOX1, IGF2BP1, ITM2C, ITSN1, KCNMA1, LDHA, MAP3K20, MTCH1, NCK2, NF1, NQO1, PARP1, PAWR, PIN1, PKN2, PLCG1, PML, PPP1CA, PPP3CA, PRKCI, PTGIS, PTPA, PTRH2, PYCARD, RHOB, RIPK2, RNPS1, RYBP, SAP30BP, SARM1, SCRIB, SIRT1, SLC9A1, SLIT2, SMPD1, SOD1, SOD2, SORT1, SPHK2, SQSTM1, SRC, STK24, TGM2, THBS1, TIGAR, TRADD, TRIO, TSPO, WDR35]	No change
regulation of programmed cell death	GO_BiologicalProcess-EBI-UniProt-GOA-ACAP-ARAP_13.05.2021_00h00	9.65E-08	10.5625	169	[AARS1, ADAM10, ADNP, AKAP12, AKR1B1, ALB, ANO6, ANXA5, APAF1, APEX1, APIP, ARAF, ARHGEF11, ARHGEF12, ARRB1, ASAH1, ASNS, ATG7, B4GALT1, BAG1, BIN1, BNIP1, C1QBP, C3orf38, CAMK2D, CASP3, CASP6, CDC34, CDC73, CDK1, CFDP1, CLU, CTNNB1, CTNNBL1, CTSC, CXCL12, DAB2, DAPK1, DCPS, DDX42, DHCR24, DIPK2A, DNM1L, DNMT1, DYNLL1,	No change

					EEF1E1, EIF2AK2, EIF2S1, ERCC5, ETFDH, FASN, FGD4, FGF2, FHL2, G6PD, GBE1, GLMN, GLO1, GPI, GSK3B, GSTP1, HDAC2, HDAC4, HHIP, HMOX1, HSP90AA1, HSP90B1, HSPA5, HSPB6, IGF2BP1, IKBKB, ITGAV, ITGB1, ITM2C, ITSN1, IVNS1ABP, KCNMA1, KDM1A, KRT18, LDHA, MAD2L1, MAEA, MALT1, MAP3K20, MAP4K4, MGMT, MSH2, MTCH1, MYD88, MYO18A, NCK2, NDRG1, NDUFA13, NDUFS3, NES, NF1, NF2, NIBAN2, NLE1, NNT, NOP53, NPM1, NQO1, NUP62, OXSR1, PARP1, PAWR, PDCD6IP, PIK3CA, PIN1, PKN2, PLCG1, PML, PPP1CA, PPP3CA, PRKAA1, PRKCI, PTGIS, PTPA, PTPMT1, PTRH2, PYCARD, QARS1, RHOB, RIPK2, RNF213, RNPS1, RTN4, RYBP, SARM1, SCRIB, SERPINB2, SERPINB9, SIRT1, SLC1A1, SLC25A4, SLC25A5, SLC9A1, SLIT2, SMPD1, SOD1, SOD2, SON, SORT1, SPHK2, SPTLC1, SQSTM1, SRA1, SRC, STAT1, STAT5B, STK24, TAX1BP1, TCP1, TGM2, THBS1, TIGAR, TJP1, TRADD, TRIO, TSPO, URI1, VDAC1, VDAC2, WDR35, WFS1, XIAP, YAP1, ZFAND6]	
regulation of apoptotic process	GO_BiologicalProcess-EBI-UniProt-GOA-ACAP-ARAP_13.05.2021_00h00	9.65E-08	10.533076	164	[AARS1, ADAM10, ADNP, AKAP12, AKR1B1, ALB, ANO6, ANXA5, APAF1, APEX1, APIP, ARAF, ARHGEF11, ARHGEF12, ARRB1, ASNS, ATG7, B4GALT1, BAG1, BIN1, BNIP1, C1QBP, C3orf38, CAMK2D, CASP3, CASP6, CDC34, CDC73, CDK1, CFDP1, CLU, CTNNB1, CTNNBL1, CTSC, CXCL12, DAB2, DAPK1, DDX42, DHCR24, DIPK2A, DNM1L, DNMT1, DYNLL1, EEF1E1, EIF2AK2, EIF2S1,	No change

					ERCC5, ETFDH, FASN, FGD4, FHL2, G6PD, GBE1, GLMN, GLO1, GPI, GSK3B, GSTP1, HDAC2, HDAC4, HHIP, HMOX1, HSP90AA1, HSP90B1, HSPA5, HSPB6, IGF2BP1, IKBKB, ITGAV, ITGB1, ITM2C, ITSN1, IVNS1ABP, KCNMA1, KDM1A, KRT18, LDHA, MAD2L1, MAEA, MALT1, MAP3K20, MAP4K4, MGMT, MSH2, MTCH1, MYD88, MYO18A, NCK2, NDRG1, NDUFA13, NDUFS3, NES, NF1, NF2, NIBAN2, NLE1, NNT, NOP53, NPM1, NQO1, NUP62, OXSR1, PARP1, PAWR, PIK3CA, PIN1, PKN2, PLCG1, PML, PPP1CA, PPP3CA, PRKAA1, PRKCI, PTGIS, PTPA, PTPMT1, PTRH2, PYCARD, QARS1, RHOB, RIPK2, RNF213, RNPS1, RTN4, RYBP, SARM1, SCRIB, SERPINB2, SERPINB9, SIRT1, SLC1A1, SLC25A5, SLC9A1, SLIT2, SMPD1, SOD1, SOD2, SON, SORT1, SPHK2, SPTLC1, SQSTM1, SRA1, SRC, STAT1, STAT5B, STK24, TAX1BP1, TCP1, TGM2, THBS1, TIGAR, TJP1, TRADD, TRIO, TSPO, URI1, VDAC1, VDAC2, WDR35, WFS1, XIAP, YAP1, ZFAND6]	
positive regulation of apoptotic process	GO_BiologicalProcess-EBI-UniProt-GOA-ACAP-ARAP_13.05.2021_00h00	9.65E-08	12.824957	74	[ADAM10, AKAP12, ANO6, APAF1, ARHGEF11, ARHGEF12, ARRB1, ATG7, B4GALT1, BIN1, C1QBP, C3orf38, CAMK2D, CASP3, CDC34, CLU, CTNNB1, CTNNBL1, CTSC, DAPK1, DNM1L, DYNLL1, EEF1E1, EIF2S1, FASN, FGD4, GLMN, HDAC4, HMOX1, IGF2BP1, ITM2C, ITSN1, KCNMA1, LDHA, MAP3K20, MTCH1, NCK2, NF1, NQO1, PAWR, PIN1, PKN2, PLCG1, PML, PPP1CA, PPP3CA, PRKCI, PTGIS, PTPA, PTRH2, PYCARD, RHOB, RIPK2,	No change

					RNPS1, RYBP, SCRIB, SIRT1, SLC9A1, SLIT2, SMPD1, SOD1, SOD2, SORT1, SPHK2, SQSTM1, SRC, STK24, TGM2, THBS1, TIGAR, TRADD, TRIO, TSPO, WDR35]	
membrane-bounded organelle	GO_CellularComponent-EBI-UniProt-GOA-ACAP-ARAP_13.05.2021_00h00	3.39E-73	9.659048	1289	[AAR2, AARS1, AASDHPPT, AASS, ABAT, ABCB7, ABCC1, ABCE1, ABHD12, ABI1, ABT1, ACACA, ACAD8, ACADM, ACADSB, ACADVL, ACAP2, ACAT2, ACO1, ACOT7, ACOT8, ACOX1, ACSF2, ACSL3, ACSL4, ACSS2, ACTB, ACTG1, ACTR2, ACTR3, ADAM10, ADAMTS9, ADARB1, ADD3, ADK, ADNP, ADSL, AFDN, AGO2, AGPAT2, AGPS, AGRN, AHCTF1, AK1, AK3, AK4, AK6, AKR1B1, ALB, ALCAM, ALDH18A1, ALDH2, ALDH3A2, ALDOA, ALG11, AMOTL1, ANK3, ANKFY1, ANKRD13A, ANO6, ANXA3, ANXA5, ANXA7, AP3M2, AP5B1, APAF1, APBB2, APEX1, APLP2, APP, APPL2, APRT, AQR, ARAF, ARFGAP1, ARFGEF1, ARFIP1, ARHGAP17, ARHGAP5, ARHGEF12, ARID1B, ARL14EP, ARPC4, ARPC5L, ARPP19, ARRB1, ASAH1, ASAP2, ASH2L, ASPM, ASPSCR1, ATE1, ATG7, ATL3, ATP1A1, ATP1B3, ATP2B1, ATP2B2, ATP2C1, ATP6V0A1, ATP6V0C, ATP6V1A, ATP6V1B2, ATPAF1, AUP1, B2M, B3GAT3, B4GALT1, B4GALT5, BAG1, BAIAP2, BANF1, BAZ1B, BCAM, BCAS2, BCAT1, BCR, BDH2, BET1, BIN1, BLVRB, BMP1, BMS1, BNIP1, BPNT2, BRD3, BST1, C1GALT1, C1QBP, C2CD2L, C3orf38, CAMK2D, CANX, CAP1, CAPN5, CAPZA2, CASK, CASP3, CASP6, CBR1, CBX1, CBX2, CBX3,	No change

					CBX5, CBX8, CC2D1A, CCDC22, CCN2, CCNH, CCNK, CCNL1, CCT2, CCT3, CCT6A, CD109, CD81, CDC34, CDC42BPA, CDC5L, CDC73, CDH11, CDIPT, CDK1, CDK16, CDK17, CDK2, CDK5RAP3, CECR2, CELF2, CEMIP, CENPV, CERS6, CETN2, CFAP20, CFAP298, CFL2, CHAF1B, CHAMP1, CHD1L, CHD2, CHD4, CHD7, CHID1, CHMP3, CHORDC1, CHP1, CHPF, CHSY3, CHTOP, CIRBP, CKB, CLGN, CLIC1, CLIP2, CLTC, CLTCL1, CLU, CMPK1, CMTR1, CNGB1, CNOT1, CNOT10, COBLL1, COG6, COG7, COIL, COL11A1, COL12A1, COL15A1, COL18A1, COL4A1, COL5A1, COL5A2, COL6A1, COLEC12, COMMD4, COQ5, CORO1B, CORO1C, CORO7, COX20, COX4I1, COX6C, CPD, CPNE2, CPSF2, CRABP2, CRBN, CROT, CRTAP, CRYZ, CSPG4, CSRP2, CSTB, CSTF3, CTBP1, CTNNB1, CTNNBL1, CTSA, CTSC, CUL1, CUL4B, CUX1, CWC22, CXCL12, CYB5R3, CYCS, CYP51A1, CYRIB, DAB2, DAGLB, DAPK1, DARS1, DCN, DCPS, DCXR, DDI2, DDX1, DDX42, DDX47, DERL1, DGLUCY, DGUOK, DHCR24, DHCR7, DHFR, DHRS7B, DHX36, DIAPH1, DIAPH3, DICER1, DIMT1, DIPK2A, DLST, DNM1L, DNMBP, DNMT1, DNTTIP2, DPCD, DPP3, DPP9, DPYSL2, DPYSL3, DRG1, DSP, DST, DUSP23, DUSP3, DUT, DVL2, DYM, DYNC1LI1, DYNLL1, EARS2, ECE1, ECI2, EDF1, EED, EEF1E1, EEF1G, EFEMP1, EHD3, EIF2AK2, EIF2D, EIF2S1, EIF3A, EIF3B, EIF3E, EIF6, ELP3, ELP4, ELP5, EMC1, EMG1, EML3, ENO2, ENPP1, ENY2, EOGT,	

					EPB41L2, EPHA3, EPHB4, EPHX1, EPM2AIP1, EPPK1, ERAP1, ERBB2, ERCC5, ERLIN2, ERP44, ESYT1, ETFDH, ETHE1, EXD2, EXTL2, EZR, FAHD1, FAM120A, FAM3A, FAM91A1, FANCI, FASN, FAT1, FBLIM1, FBLN2, FBXL12, FBXW8, FDXR, FEN1, FERMT2, FGD4, FGF2, FHL1, FHL2, FHOD1, FIBP, FKBP1A, FKBP5, FKBP9, FLII, FLRT2, FMR1, FN1, FNBP1L, FSCN1, FSD1, FTH1, FTL, FTO, FUT11, FUT8, G6PD, GALM, GALNT1, GALNT2, GALNT7, GAMT, GAPDH, GARS1, GATAD2A, GATB, GBA2, GBE1, GBP1, GCAT, GCC2, GCDH, GCFC2, GDAP1, GDAP2, GEMIN4, GET3, GGA2, GGCT, GIPC1, GIPC2, GLA, GLE1, GLMP, GLO1, GLRX3, GLUD1, GLUL, GLYR1, GNB4, GNL3L, GOLGA1, GOLGA3, GOLGA7, GOLT1B, GOT1, GPC1, GPC6, GPD1L, GPD2, GPI, GPKOW, GPN1, GPR108, GPSM1, GRAMD1A, GRWD1, GSDMD, GSK3B, GSTCD, GSTM2, GSTM3, GSTP1, GTF2E1, GTF2F1, GTF2F2, GTF2I, GTF3C3, GTF3C4, H1-10, H1-5, HACL1, HADH, HADHA, HARS1, HAT1, HDAC2, HDAC4, HDGFL2, HEBP1, HELZ, HERC2, HERC4, HEXB, HGS, HHIP, HIBCH, HINT1, HIP1, HIRIP3, HK1, HK2, HLA-A, HLA-B, HLA-C, HMOX1, HNRNPA0, HNRNPA3, HNRNPC, HNRNPH1, HNRNPLL, HNRNPR, HNRNPUL1, HPX, HSD17B10, HSD17B11, HSD17B4, HSDL2, HSP90AA1, HSP90B1, HSPA14, HSPA4, HSPA5, HSPB11, HSPB6, HSPE1, HSPG2, HTATSF1, IARS2, IDH2, IDI1, IFRD2,	

					IFT140, IGF2BP1, IGF2BP2, IGFBP7, IKBKB, IKBKG, ILF3, ILKAP, ILVBL, INPP4A, INTS1, INTS9, IPO5, IPO9, IREB2, ISOC1, IST1, ITGA3, ITGAV, ITGB1, ITGB5, ITIH4, ITM2C, ITSN1, IVNS1ABP, JAGN1, JAM3, KAT7, KAT8, KCNQ1, KDM1A, KDM3B, KDM4B, KDSR, KNTC1, KPNB1, KRT18, KRT19, KRT8, L1TD1, LACTB, LAMA5, LAMC1, LAMP1, LANCL2, LAP3, LARP4B, LARP6, LBR, LDHA, LEMD2, LGALS3BP, LIG1, LIG3, LIPA, LLGL1, LMCD1, LMNB1, LMO7, LNPEP, LPCAT2, LPCAT4, LPP, LRCH4, LRPPRC, LRRC32, LRRC57, LRRC8A, LRRC8E, LRRFIP1, LRRK1, LRWD1, LSS, LTBP1, LUM, LYPLA2, M6PR, MACF1, MACROD1, MACROH2A1, MAD1L1, MAD2L1, MAEA, MAGED2, MAGI1, MALT1, MAN2B2, MAOA, MAP1B, MAP1LC3A, MAP2K3, MAP3K20, MARCHF5, MATR3, MBD3, MBOAT2, MBOAT7, MCAM, MCM2, MCM3, MCM4, MCM5, MCM6, MCM7, MCMBP, MDC1, ME1, MEAK7, MED17, MED23, MED30, MEST, METTL14, MFF, MFN1, MFSD10, MGA, MGAT1, MGMT, MGST1, MICAL3, MICALL1, MLF2, MLH1, MMAB, MME, MMUT, MOCS2, MOGS, MORF4L1, MOV10, MPHOSPH8, MPST, MRM3, MRPL10, MRPS17, MRPS21, MRPS31, MRRF, MRTO4, MSH2, MSH6, MSI2, MSN, MTA1, MTAP, MTCH1, MTHFD2, MTREX, MTRR, MVD, MVK, MVP, MXRA5, MYD88, MYH10, MYH9, MYO18A, MYO1B, MYO1D, MYO1E, MYO6,	

					MYOF, N6AMT1, NCAPD2, NCAPG, NCAPH, NCDN, NCK2, NCKAP1, NCL, NCLN, NCOA5, NCOA7, NDRG1, NDUFA13, NDUFA4, NDUFAF7, NDUFS3, NECTIN2, NEDD1, NEDD4, NEDD8, NEK1, NELFCD, NENF, NF1, NF2, NFATC2IP, NFKB2, NHLRC2, NIBAN1, NIBAN2, NISCH, NLE1, NMD3, NMT1, NNT, NOCT, NOL11, NOM1, NOP53, NPLOC4, NPM1, NSDHL, NSMCE3, NSUN5, NT5C, NUB1, NUBP2, NUBPL, NUCB2, NUDT16, NUDT16L1, NUDT19, NUF2, NUP205, NUP35, NUP62, NUTF2, NXN, OAT, OBSL1, OGFOD1, ORC4, ORC5, OSBPL10, OSBPL11, OSBPL1A, OSBPL2, OSBPL8, OXA1L, OXSR1, P3H1, P3H3, PACS1, PACSIN3, PAFAH1B1, PAFAH1B2, PAICS, PALLD, PANK4, PANX1, PARG, PARL, PARP1, PARP14, PARP4, PATZ1, PAWR, PBXIP1, PC, PCBP3, PCDH7, PCK2, PCYT2, PDAP1, PDCD6IP, PDK1, PDS5B, PDXDC1, PEBP1, PELO, PFAS, PFKM, PGAM5, PHGDH, PHPT1, PI4K2B, PIEZO1, PIGO, PIGT, PIGU, PIK3C2A, PIN1, PIP4K2B, PIR, PITHD1, PITRM1, PKLR, PKM, PKN2, PLA2G15, PLA2G4A, PLBD2, PLCB3, PLCG1, PLD1, PLD3, PLEC, PLOD2, PLPP3, PLXNB2, PML, PMM2, PNP, PNPLA4, PNPLA6, POFUT1, POGLUT3, POGZ, POLD1, POLD2, POLE2, POLR1C, POLR1D, POLR2A, POLR2B, POLR2H, POLR2I, POLR3A, POLR3C, POMGNT1, POMGNT2, POMK, POP1, PPIB, PPIE, PPIG, PPM1B, PPP1CA, PPP1R7, PPP2R5A, PPP3CA, PPP4R3A, PPP5C,	

					PPWD1, PRDX1, PRDX6, PRIM2, PRKAA1, PRKAB2, PRKCI, PRKCSH, PRKG1, PRMT7, PROCR, PRPF19, PRUNE1, PRXL2B, PSAT1, PSIP1, PSMA3, PSMB10, PSMB2, PSMC2, PSMC3, PSMC6, PSMD1, PSMD12, PSMD14, PSMD2, PSMD6, PSMD7, PSMD8, PSME1, PSME2, PSME4, PSMG1, PSPC1, PTBP2, PTBP3, PTGES2, PTGIS, PTGR1, PTPA, PTPMT1, PTPN12, PTPRF, PTPRG, PTRH2, PUF60, PUS7, PWWP2A, PYCARD, PYCR1, PYGB, QARS1, QDPR, QSOX1, QTRT1, QTRT2, RAB10, RAB11B, RAB1A, RAB23, RAB3B, RAB3D, RAB5A, RAB8A, RABGEF1, RABL6, RAC3, RAD21, RAD9A, RAI14, RALA, RANBP1, RANGAP1, RAP1GDS1, RAP2B, RAP2C, RARS2, RBBP4, RBM22, RBM26, RBM4, RBMS2, RBP1, RCC1, RCN1, RCN2, RCN3, RDH10, RDH11, RDX, RECQL, REEP2, REEP6, RELCH, RETSAT, RFC2, RFC3, RFC5, RGS10, RHEB, RHOB, RHOC, RIF1, RIPK2, RNF114, RNF2, RNF213, RNF40, RNGTT, RNPS1, RPA1, RPA2, RPF2, RPL10A, RPL13, RPL13A, RPL22, RPL24, RPL27, RPL36, RPL5, RPLP2, RPRD1A, RPRD1B, RPRD2, RPS13, RPS15A, RPS16, RPS18, RPS21, RPS23, RPS4Y1, RPS5, RPS8, RPS9, RRAS2, RRM1, RTN4, RTTN, RYBP, S100A10, S100A16, SAAL1, SACS, SAE1, SAMHD1, SAP30BP, SARM1, SARNP, SARS1, SBDS, SCAF11, SCAI, SCARB2, SCFD1, SCLY, SCP2, SCPEP1, SCRIB, SCRN1, SDHC, SEC11C, SEC14L2, SEC23B, SEC61A1,	

					SEC61B, SELENBP1, SELENOT, SEMA3C, SENP3, SEPTIN2, SEPTIN7, SEPTIN8, SEPTIN9, SERPINB1, SERPINB8, SERPINB9, SERPINE2, SESN2, SET, SEZ6L2, SF1, SF3A3, SF3B6, SFXN2, SFXN3, SGSH, SH3BGRL2, SH3BGRL3, SH3BP4, SH3KBP1, SHFL, SHMT2, SHPRH, SIAE, SIRT1, SLC11A2, SLC12A2, SLC16A3, SLC17A5, SLC1A1, SLC1A4, SLC1A5, SLC25A10, SLC25A12, SLC25A13, SLC25A15, SLC25A19, SLC25A22, SLC25A24, SLC25A26, SLC25A4, SLC25A5, SLC25A6, SLC26A2, SLC2A1, SLC30A9, SLC31A1, SLC44A1, SLC44A2, SLC4A7, SLC7A6, SLC9A1, SLIT2, SMAD4, SMARCA1, SMARCAD1, SMARCB1, SMARCC1, SMARCD3, SMARCE1, SMC1A, SMC2, SMC3, SMC4, SMCHD1, SMNDC1, SMPD1, SMU1, SMYD3, SNF8, SNRNP200, SNRPA, SNRPD3, SNRPE, SNU13, SNW1, SNX1, SNX18, SNX21, SNX27, SNX5, SOAT1, SOD1, SOD2, SON, SORBS3, SORT1, SPAG7, SPARC, SPATS2L, SPECC1, SPHK2, SPTLC1, SPTLC2, SQSTM1, SRA1, SRC, SRD5A3, SRPRA, SRSF10, SRSF2, SRSF5, SSB, SSR3, SSRP1, STAG2, STAT1, STAT2, STAT5B, STK24, STOM, STON1, STRN3, STXBP3, SUFU, SUGT1, SULT1E1, SUPT16H, SURF1, SVIL, SYNE2, SYNJ1, SYNPO, SYPL2, TAB2, TAF6L, TAGLN2, TALDO1, TAP1, TAX1BP1, TBC1D10A, TBC1D17, TBC1D4, TBCA, TBK1, TBPL1, TCP1, TDRD3, TF, TFAM, TGFBI,	

					TGFBR3, TGM2, TGS1, THADA, THBS1, THEM4, THOC2, TIGAR, TIMM17B, TIMMDC1, TJP1, TJP2, TKT, TM9SF4, TMA16, TMED9, TMEM119, TMEM168, TMEM192, TMEM65, TMEM97, TMX3, TMX4, TNIK, TNPO2, TOM1, TOM1L2, TOMM34, TOMM7, TOR1AIP1, TOR1AIP2, TOR1B, TPBG, TPI1, TPM3, TPP1, TPP2, TPRG1L, TPST2, TPX2, TRA2A, TRA2B, TRADD, TRAM1, TRAM2, TRAPPC4, TRAPPC8, TRIM21, TRIM25, TRIM28, TRIM56, TRIM59, TRIP12, TRIP13, TRIP6, TRMT10C, TRMT5, TSC2, TSEN15, TSEN34, TSPO, TSPYL1, TTC1, TTC5, TTLL12, TUBA4A, TUBB, TUBB3, TUBGCP2, TUT4, TWF2, TXN, TXNDC17, U2SURP, UACA, UAP1, UBA2, UBA3, UBAC2, UBE2L6, UBE2Q1, UBR2, UBR4, UBTF, UBXN2A, UFD1, UGP2, UHRF2, UMPS, UNC119B, UNC93B1, UQCR10, UQCR11, UQCRC2, URB2, URI1, URM1, USP11, USP22, USP28, USP4, USP48, USP5, USP7, USP9X, USP9Y, UST, UTP14A, UTP20, UTP3, VARS1, VASP, VAT1, VDAC1, VDAC2, VLDLR, VPS25, VPS26B, VPS33B, VPS36, VPS41, VRK1, VRK3, VTN, VWA5A, VWA8, WASHC5, WDR1, WDR11, WDR12, WDR35, WDR41, WDR44, WDR5, WDR70, WFS1, WIPI2, WRAP73, WRNIP1, WWP2, XIAP, XPNPEP3, XPO4, XPO6, XPOT, XYLT2, YAP1, YARS1, YBEY, YBX1, YEATS4, YES1, YKT6, YRDC, YTHDC1, ZC3HAV1, ZDHHC13, ZDHHC21, ZFP36L2, ZMPSTE24, ZMYM3, ZNF148, ZNF207, ZNF512]	

intracellular organelle	GO_CellularComponent-EBI-UniProt-GOA-ACAP-ARAP_13.05.2021_00h00	3.39E-73	9.550899	1276	[AAR2, AARS1, AASS, ABAT, ABCB7, ABCE1, ABHD12, ABI1, ABT1, ACACA, ACAD8, ACADM, ACADSB, ACADVL, ACAP2, ACAT2, ACO1, ACOT7, ACOT8, ACOX1, ACSF2, ACSL3, ACSL4, ACSS2, ACTB, ACTG1, ACTR2, ACTR3, ADAM10, ADAMTS9, ADARB1, ADD3, ADK, ADNP, AFDN, AGO2, AGPAT2, AGPS, AGRN, AHCTF1, AK1, AK3, AK4, AK6, AKAP12, AKR1B1, ALB, ALDH18A1, ALDH2, ALDH3A2, ALDOA, ALG11, AMOTL1, ANK3, ANKFY1, ANKRD13A, ANO6, ANXA3, ANXA7, AP3M2, AP5B1, APAF1, APBB2, APEX1, APLP2, APP, APPL2, APRT, AQR, ARAF, ARFGAP1, ARFGEF1, ARFIP1, ARHGAP17, ARHGAP5, ARID1B, ARL14EP, ARPC4, ARPC5L, ARPP19, ARRB1, ASAH1, ASAP2, ASH2L, ASPM, ASPSCR1, ATE1, ATG7, ATL3, ATP1A1, ATP1B3, ATP2B1, ATP2B2, ATP2C1, ATP6V0A1, ATP6V0C, ATP6V1A, ATP6V1B2, ATPAF1, AUP1, B2M, B3GAT3, B4GALT1, B4GALT5, BAG1, BAIAP2, BANF1, BAZ1B, BCAS2, BCAT1, BDH2, BET1, BIN1, BLVRB, BMP1, BMS1, BNIP1, BOD1, BPNT2, BRD3, BST1, C1GALT1, C1QBP, C2CD2L, C3orf38, CALD1, CAMK2D, CANX, CAP1, CAPZA2, CASK, CASP3, CASP6, CBX1, CBX2, CBX3, CBX5, CBX8, CC2D1A, CCDC102A, CCDC22, CCN2, CCNH, CCNK, CCNL1, CCT2, CCT3, CCT6A, CD109, CDC34, CDC42BPA, CDC42EP1, CDC5L, CDC73, CDIPT, CDK1, CDK16, CDK17, CDK2, CDK5RAP3, CECR2, CELF2, CEMIP, CENPV, CERS6, CETN2, CFAP20, CFAP298,	No change

					CFDP1, CFL2, CHAF1B, CHAMP1, CHD1L, CHD2, CHD4, CHD7, CHID1, CHMP3, CHORDC1, CHP1, CHPF, CHSY3, CHTOP, CIRBP, CKB, CLGN, CLIC1, CLIP2, CLTC, CLTCL1, CLU, CMPK1, CMTR1, CNGB1, CNN3, CNOT1, CNOT10, COG6, COG7, COIL, COL11A1, COL12A1, COL15A1, COL18A1, COL4A1, COL5A1, COL5A2, COL6A1, COLEC12, COMMD4, COQ5, CORO1B, CORO1C, CORO2B, CORO7, COX20, COX4I1, COX6C, CPNE2, CPSF2, CRABP2, CRBN, CROT, CRTAP, CSPG4, CSRP2, CSTB, CSTF3, CTBP1, CTNNB1, CTNNBL1, CTSA, CTSC, CUL1, CUL4B, CUX1, CWC22, CXCL12, CYB5R3, CYCS, CYP51A1, CYRIB, DAB2, DAGLB, DAPK1, DCN, DCPS, DCXR, DDI2, DDX1, DDX42, DDX47, DENND2A, DERL1, DGLUCY, DGUOK, DHCR24, DHCR7, DHFR, DHRS7B, DHX36, DIAPH1, DIAPH3, DICER1, DIMT1, DIPK2A, DLST, DNM1L, DNMBP, DNMT1, DNTTIP2, DPCD, DPP9, DPYSL2, DPYSL3, DRG1, DSP, DST, DUSP23, DUSP3, DUT, DVL2, DYM, DYNC1LI1, DYNLL1, EARS2, ECE1, ECI2, EDF1, EED, EEF1E1, EEF1G, EHD3, EIF2AK2, EIF2D, EIF2S1, EIF3A, EIF3E, EIF6, ELP3, ELP4, ELP5, EMC1, EMG1, EML1, EML3, EML4, ENPP1, ENY2, EOGT, EPB41L2, EPB41L3, EPHA3, EPHX1, EPM2AIP1, EPPK1, ERAP1, ERBB2, ERCC5, ERCC6L, ERLIN2, ERP44, ESYT1, ETFDH, ETHE1, EXD2, EXTL2, EZR, FAHD1, FAM120A, FAM3A, FAM91A1, FANCI, FARP1, FASN, FAT1, FBLIM1, FBXL12, FBXW8,	

					FDXR, FEN1, FERMT1, FERMT2, FGD4, FGF2, FHL1, FHL2, FHOD1, FIBP, FKBP1A, FKBP5, FKBP9, FLII, FLRT2, FMR1, FN1, FNBP1L, FSCN1, FSD1, FTH1, FTL, FTO, FUT11, FUT8, G6PD, GALNT1, GALNT2, GALNT7, GAMT, GAN, GAPDH, GARS1, GATAD2A, GATB, GBA2, GBP1, GCAT, GCC2, GCDH, GCFC2, GDAP1, GDAP2, GEMIN4, GET3, GGA2, GIPC1, GLA, GLE1, GLMP, GLO1, GLRX3, GLUD1, GLUL, GLYR1, GNB4, GNL3L, GOLGA1, GOLGA3, GOLGA7, GOLT1B, GOT1, GPC1, GPC6, GPD2, GPHN, GPI, GPKOW, GPN1, GPR108, GPSM1, GRAMD1A, GRWD1, GSDMD, GSK3B, GSTM2, GSTM3, GSTP1, GTF2E1, GTF2F1, GTF2F2, GTF2I, GTF3C3, GTF3C4, H1-10, H1-5, HACL1, HADH, HADHA, HARS1, HAT1, HDAC2, HDAC4, HDGFL2, HELZ, HERC2, HERC4, HEXB, HGS, HHIP, HIBCH, HINT1, HIP1, HIRIP3, HK1, HK2, HLA-A, HLA-B, HLA-C, HMCES, HMOX1, HNRNPA0, HNRNPA3, HNRNPC, HNRNPH1, HNRNPLL, HNRNPR, HNRNPUL1, HPX, HSD17B10, HSD17B11, HSD17B4, HSDL2, HSP90AA1, HSP90B1, HSPA14, HSPA4, HSPA5, HSPB11, HSPB6, HSPE1, HSPG2, HTATSF1, IARS2, IDH2, IDI1, IFIT5, IFRD2, IFT140, IGF2BP1, IGF2BP2, IGFBP7, IKBKB, IKBKG, ILF3, ILKAP, ILVBL, INPP4A, INTS1, INTS9, IPO5, IPO9, IREB2, ISOC1, IST1, ITGAV, ITGB1, ITGB5, ITIH4, ITM2C, ITSN1, IVNS1ABP, JAGN1, JAM3, KAT7, KAT8, KCNQ1, KDM1A, KDM3B,	

					KDM4B, KDSR, KIF15, KNTC1, KPNB1, KRT18, KRT19, KRT8, L1TD1, LACTB, LAMA5, LAMC1, LAMP1, LANCL2, LAP3, LARP1, LARP4B, LARP6, LBR, LDHA, LEMD2, LGALS3BP, LIG1, LIG3, LIPA, LLGL1, LMCD1, LMNB1, LMO7, LMOD1, LNPEP, LPCAT2, LPCAT4, LPP, LRCH4, LRPPRC, LRRC32, LRRC8A, LRRC8E, LRRFIP1, LRRK1, LRWD1, LSS, LTBP1, LUM, LYPLA2, M6PR, MACF1, MACROD1, MACROH2A1, MAD1L1, MAD2L1, MAEA, MAGED2, MAGI1, MALT1, MAN2B2, MAOA, MAP1B, MAP1LC3A, MAP2K3, MAP3K20, MARCHF5, MATR3, MBD3, MBOAT2, MBOAT7, MCAM, MCM2, MCM3, MCM4, MCM5, MCM6, MCM7, MCMBP, MDC1, ME1, MEAK7, MED17, MED23, MED30, MEST, METTL14, MFF, MFN1, MFSD10, MGA, MGAT1, MGMT, MGST1, MICAL3, MICALL1, MLF2, MLH1, MMAB, MME, MMUT, MOCS2, MOGS, MORF4L1, MOV10, MPHOSPH8, MPST, MRM3, MRPL10, MRPS17, MRPS21, MRPS31, MRRF, MRTO4, MSH2, MSH6, MSI2, MSN, MTA1, MTAP, MTCH1, MTHFD2, MTREX, MTRR, MVD, MVK, MVP, MYADM, MYD88, MYH10, MYH9, MYO18A, MYO1B, MYO1D, MYO1E, MYO6, MYOF, N6AMT1, NCAPD2, NCAPG, NCAPH, NCDN, NCK2, NCKAP1, NCL, NCLN, NCOA5, NCOA7, NDRG1, NDUFA13, NDUFA4, NDUFAF7, NDUFS3, NEDD1, NEDD4, NEDD8, NEK1, NELFCD, NENF, NES, NF1, NF2, NFATC2IP,	

					NFKB2, NHLRC2, NIBAN2, NISCH, NLE1, NMD3, NMT1, NNT, NOCT, NOL11, NOM1, NOP53, NPLOC4, NPM1, NSDHL, NSMCE3, NSUN5, NT5C, NUB1, NUBP2, NUBPL, NUCB2, NUDT16, NUDT16L1, NUDT19, NUF2, NUP205, NUP35, NUP62, NUTF2, NXN, OAT, OBSL1, OGFOD1, ORC4, ORC5, OSBPL10, OSBPL11, OSBPL1A, OSBPL2, OSBPL8, OXA1L, OXSR1, P3H1, P3H3, PACS1, PACSIN3, PAFAH1B1, PAFAH1B2, PALLD, PANK4, PANX1, PARG, PARL, PARP1, PARP14, PARP4, PATZ1, PAWR, PBXIP1, PC, PCBP3, PCDH7, PCK2, PCYT2, PDAP1, PDCD6IP, PDK1, PDS5B, PDXDC1, PEBP1, PELO, PFKM, PGAM5, PHGDH, PHPT1, PI4K2B, PIEZO1, PIGO, PIGT, PIGU, PIK3C2A, PIN1, PIP4K2B, PIR, PITHD1, PITRM1, PKM, PKN2, PLA2G15, PLA2G4A, PLBD2, PLCB3, PLCG1, PLD1, PLD3, PLEC, PLOD2, PLPP3, PML, PMM2, PNP, PNPLA4, PNPLA6, POFUT1, POGLUT3, POGZ, POLD1, POLD2, POLE2, POLR1C, POLR1D, POLR2A, POLR2B, POLR2H, POLR2I, POLR3A, POLR3C, POMGNT1, POMGNT2, POMK, POP1, PPIB, PPIE, PPIG, PPM1B, PPP1CA, PPP1R7, PPP2R5A, PPP3CA, PPP4R3A, PPP5C, PPWD1, PRDX1, PRDX6, PRIM2, PRKAA1, PRKAB2, PRKCI, PRKCSH, PRKG1, PRMT7, PROCR, PRPF19, PRUNE1, PRXL2B, PSIP1, PSMA3, PSMB10, PSMB2, PSMC2, PSMC3, PSMC6, PSMD1, PSMD12, PSMD14, PSMD2, PSMD6, PSMD7, PSMD8, PSME1, PSME2,	

					PSME4, PSMG1, PSPC1, PTBP2, PTBP3, PTGES2, PTGIS, PTPA, PTPMT1, PTPN12, PTRH2, PUF60, PUS7, PWWP2A, PXK, PXN, PYCARD, PYCR1, PYCR3, PYGB, QARS1, QSOX1, QTRT1, QTRT2, RAB10, RAB11B, RAB1A, RAB23, RAB3B, RAB3D, RAB5A, RAB8A, RABGEF1, RABL6, RAC3, RAD21, RAD9A, RAI14, RALA, RANBP1, RANGAP1, RAP1GDS1, RAP2B, RAP2C, RARS2, RBBP4, RBM22, RBM26, RBM4, RBMS2, RBP1, RCC1, RCN1, RCN2, RCN3, RDH10, RDH11, RDX, RECQL, REEP2, REEP6, RELCH, RETSAT, RFC2, RFC3, RFC5, RGS10, RHEB, RHOB, RHOC, RIF1, RIN1, RIPK2, RNF114, RNF2, RNF213, RNF40, RNGTT, RNPS1, ROR1, RPA1, RPA2, RPF2, RPL10A, RPL13, RPL13A, RPL18A, RPL22, RPL24, RPL27, RPL32, RPL36, RPL38, RPL5, RPLP1, RPLP2, RPRD1A, RPRD1B, RPRD2, RPS13, RPS15A, RPS16, RPS18, RPS21, RPS23, RPS4Y1, RPS5, RPS8, RPS9, RRAS2, RRM1, RTN4, RTTN, RYBP, S100A10, S100A16, SAAL1, SACS, SAE1, SAMHD1, SAP30BP, SARM1, SARNP, SARS1, SBDS, SCAF11, SCAI, SCARB2, SCFD1, SCLY, SCP2, SCRIB, SCRN1, SDHC, SEC11C, SEC14L2, SEC23B, SEC61A1, SEC61B, SELENBP1, SELENOT, SENP3, SEPTIN10, SEPTIN11, SEPTIN2, SEPTIN7, SEPTIN8, SEPTIN9, SERPINB1, SERPINB9, SERPINE2, SESN2, SESTD1, SET, SEZ6L2, SF1, SF3A3, SF3B6, SFXN2, SFXN3, SGSH, SH3BGRL2, SH3BGRL3, SH3BP4, SH3KBP1, SHFL,	

					SHMT2, SHPRH, SIAE, SIRT1, SLC11A2, SLC12A2, SLC16A3, SLC17A5, SLC1A1, SLC1A4, SLC1A5, SLC25A10, SLC25A12, SLC25A13, SLC25A15, SLC25A19, SLC25A22, SLC25A24, SLC25A26, SLC25A4, SLC25A5, SLC25A6, SLC2A1, SLC30A9, SLC31A1, SLC44A1, SLC44A2, SLC4A7, SLC7A6, SLC9A1, SMAD4, SMARCA1, SMARCAD1, SMARCB1, SMARCC1, SMARCD3, SMARCE1, SMC1A, SMC2, SMC3, SMC4, SMCHD1, SMNDC1, SMPD1, SMU1, SMYD3, SNF8, SNRNP200, SNRPA, SNRPD3, SNRPE, SNU13, SNW1, SNX1, SNX18, SNX21, SNX27, SNX5, SOAT1, SOD1, SOD2, SON, SORBS3, SORT1, SPAG7, SPARC, SPART, SPATS2L, SPECC1, SPHK2, SPTLC1, SPTLC2, SQSTM1, SRA1, SRC, SRD5A3, SRPRA, SRSF10, SRSF2, SRSF5, SSB, SSR3, SSRP1, STAG2, STAT1, STAT2, STAT5B, STK24, STOM, STON1, STRN3, STXBP3, SUFU, SUGT1, SULT1E1, SUPT16H, SURF1, SVIL, SYNE2, SYNJ1, SYNPO, SYPL2, TAB2, TAF6L, TALDO1, TAP1, TBC1D17, TBCA, TBCE, TBCEL, TBK1, TBPL1, TCP1, TDRD3, TF, TFAM, TGFBI, TGM2, TGS1, THADA, THBS1, THEM4, THOC2, TIGAR, TIMM17B, TIMMDC1, TJP1, TJP2, TKT, TLN2, TM9SF4, TMA16, TMED9, TMEM119, TMEM168, TMEM192, TMEM65, TMEM97, TMX3, TMX4, TNIK, TNPO2, TOM1, TOM1L2, TOMM34, TOMM7, TOR1AIP1, TOR1AIP2, TOR1B, TPBG, TPI1, TPM3,	

					TPP1, TPP2, TPRG1L, TPST2, TPX2, TRA2A, TRA2B, TRADD, TRAM1, TRAM2, TRAPPC4, TRAPPC8, TRIM21, TRIM25, TRIM28, TRIM56, TRIM59, TRIP12, TRIP13, TRIP6, TRMT10C, TRMT5, TSC2, TSEN15, TSEN34, TSPO, TSPYL1, TTC1, TTC28, TTC5, TTLL12, TUBA4A, TUBA8, TUBB, TUBB3, TUBGCP2, TUT4, TWF2, TXN, U2SURP, UACA, UAP1, UBA2, UBA3, UBAC2, UBE2L6, UBE2Q1, UBR2, UBR4, UBTF, UBXN2A, UFD1, UGP2, UHRF2, UMPS, UNC93B1, UQCR10, UQCR11, UQCRC2, URB2, URI1, URM1, USP11, USP22, USP28, USP4, USP48, USP5, USP7, USP9X, USP9Y, UST, UTP14A, UTP20, UTP3, VARS1, VASP, VAT1, VDAC1, VDAC2, VLDLR, VPS25, VPS26B, VPS33B, VPS36, VPS41, VRK1, VRK3, VTN, VWA5A, VWA8, WASHC5, WDR1, WDR11, WDR12, WDR35, WDR41, WDR44, WDR5, WDR70, WFS1, WIPI2, WRAP73, WRNIP1, WWP2, XIAP, XPNPEP3, XPO4, XPO6, XPOT, XYLT2, YAP1, YARS1, YBEY, YBX1, YEATS4, YES1, YKT6, YRDC, YTHDC1, ZC3HAV1, ZDHHC13, ZDHHC21, ZFP36L2, ZMPSTE24, ZMYM3, ZNF148, ZNF207, ZNF512]	
intracellular membrane-bounded organelle	GO_CellularComponent-EBI-UniProt-GOA-ACAP-ARAP_13.05.2021_00h00	3.39E-73	9.810616	1207	[AAR2, AARS1, AASS, ABAT, ABCB7, ABCE1, ABHD12, ABI1, ABT1, ACACA, ACAD8, ACADM, ACADSB, ACADVL, ACAP2, ACAT2, ACO1, ACOT7, ACOT8, ACOX1, ACSF2, ACSL3, ACSL4, ACSS2, ACTB, ACTG1, ACTR2, ACTR3, ADAM10, ADAMTS9, ADARB1, ADD3, ADK, ADNP, AFDN, AGO2, AGPAT2, AGPS, AGRN, AHCTF1, AK3,	No change

					AK4, AK6, AKR1B1, ALB, ALDH18A1, ALDH2, ALDH3A2, ALDOA, ALG11, AMOTL1, ANK3, ANKFY1, ANKRD13A, ANO6, ANXA3, ANXA7, AP3M2, AP5B1, APAF1, APBB2, APEX1, APLP2, APP, APPL2, APRT, AQR, ARAF, ARFGAP1, ARFGEF1, ARFIP1, ARHGAP17, ARHGAP5, ARID1B, ARL14EP, ARPC4, ARPP19, ARRB1, ASAH1, ASAP2, ASH2L, ASPM, ASPSCR1, ATE1, ATG7, ATL3, ATP1A1, ATP1B3, ATP2B1, ATP2B2, ATP2C1, ATP6V0A1, ATP6V0C, ATP6V1A, ATP6V1B2, ATPAF1, AUP1, B2M, B3GAT3, B4GALT1, B4GALT5, BAG1, BAIAP2, BANF1, BAZ1B, BCAS2, BCAT1, BDH2, BET1, BIN1, BLVRB, BMP1, BMS1, BNIP1, BPNT2, BRD3, BST1, C1GALT1, C1QBP, C2CD2L, C3orf38, CAMK2D, CANX, CAP1, CASK, CASP3, CASP6, CBX1, CBX2, CBX3, CBX5, CBX8, CC2D1A, CCDC22, CCN2, CCNH, CCNK, CCNL1, CCT2, CD109, CDC34, CDC5L, CDC73, CDIPT, CDK1, CDK16, CDK17, CDK2, CDK5RAP3, CECR2, CELF2, CEMIP, CENPV, CERS6, CETN2, CFAP20, CFAP298, CFL2, CHAF1B, CHAMP1, CHD1L, CHD2, CHD4, CHD7, CHID1, CHMP3, CHORDC1, CHP1, CHPF, CHSY3, CHTOP, CIRBP, CKB, CLGN, CLIC1, CLIP2, CLTC, CLTCL1, CLU, CMPK1, CMTR1, CNGB1, CNOT1, CNOT10, COG6, COG7, COIL, COL11A1, COL12A1, COL15A1, COL18A1, COL4A1, COL5A1, COL5A2, COL6A1, COLEC12, COMMD4, COQ5, CORO1C, CORO7, COX20, COX4I1, COX6C, CPNE2, CPSF2, CRABP2, CRBN,	

					CROT, CRTAP, CSPG4, CSRP2, CSTB, CSTF3, CTBP1, CTNNB1, CTNNBL1, CTSA, CTSC, CUL1, CUL4B, CUX1, CWC22, CXCL12, CYB5R3, CYCS, CYP51A1, CYRIB, DAB2, DAGLB, DAPK1, DCN, DCPS, DCXR, DDI2, DDX1, DDX42, DDX47, DERL1, DGLUCY, DGUOK, DHCR24, DHCR7, DHFR, DHRS7B, DHX36, DIAPH1, DIAPH3, DICER1, DIMT1, DIPK2A, DLST, DNM1L, DNMBP, DNMT1, DNTTIP2, DPCD, DPP9, DPYSL3, DRG1, DSP, DST, DUSP23, DUSP3, DUT, DVL2, DYM, DYNC1LI1, DYNLL1, EARS2, ECE1, ECI2, EDF1, EED, EEF1E1, EEF1G, EHD3, EIF2AK2, EIF2D, EIF2S1, EIF3A, EIF3E, EIF6, ELP3, ELP4, ELP5, EMC1, EMG1, EML3, ENPP1, ENY2, EOGT, EPB41L2, EPHA3, EPHX1, EPM2AIP1, EPPK1, ERAP1, ERBB2, ERCC5, ERLIN2, ERP44, ESYT1, ETFDH, ETHE1, EXD2, EXTL2, EZR, FAHD1, FAM120A, FAM3A, FAM91A1, FANCI, FASN, FAT1, FBLIM1, FBXL12, FBXW8, FDXR, FEN1, FERMT2, FGD4, FGF2, FHL1, FHL2, FHOD1, FIBP, FKBP1A, FKBP5, FKBP9, FLII, FLRT2, FMR1, FN1, FNBP1L, FSCN1, FSD1, FTH1, FTL, FTO, FUT11, FUT8, G6PD, GALNT1, GALNT2, GALNT7, GAMT, GAPDH, GARS1, GATAD2A, GATB, GBA2, GBP1, GCAT, GCC2, GCDH, GCFC2, GDAP1, GDAP2, GEMIN4, GET3, GGA2, GIPC1, GLA, GLE1, GLMP, GLO1, GLRX3, GLUD1, GLUL, GLYR1, GNB4, GNL3L, GOLGA1, GOLGA3, GOLGA7, GOLT1B, GOT1, GPC1, GPC6, GPD2, GPI, GPKOW, GPN1,	

					GPR108, GPSM1, GRAMD1A, GRWD1, GSDMD, GSK3B, GSTM2, GSTM3, GSTP1, GTF2E1, GTF2F1, GTF2F2, GTF2I, GTF3C3, GTF3C4, H1-10, H1-5, HACL1, HADH, HADHA, HARS1, HAT1, HDAC2, HDAC4, HDGFL2, HELZ, HERC2, HERC4, HEXB, HGS, HHIP, HIBCH, HINT1, HIP1, HIRIP3, HK1, HK2, HLA-A, HLA-B, HLA-C, HMOX1, HNRNPA0, HNRNPA3, HNRNPC, HNRNPH1, HNRNPLL, HNRNPR, HNRNPUL1, HPX, HSD17B10, HSD17B11, HSD17B4, HSDL2, HSP90AA1, HSP90B1, HSPA14, HSPA4, HSPA5, HSPB6, HSPE1, HSPG2, HTATSF1, IARS2, IDH2, IDI1, IFRD2, IGF2BP1, IGF2BP2, IGFBP7, IKBKB, IKBKG, ILF3, ILKAP, ILVBL, INPP4A, INTS1, INTS9, IPO5, IPO9, IREB2, ISOC1, IST1, ITGAV, ITGB1, ITGB5, ITIH4, ITM2C, ITSN1, IVNS1ABP, JAGN1, JAM3, KAT7, KAT8, KCNQ1, KDM1A, KDM3B, KDM4B, KDSR, KNTC1, KPNB1, KRT18, KRT8, L1TD1, LACTB, LAMA5, LAMC1, LAMP1, LANCL2, LAP3, LARP4B, LARP6, LBR, LDHA, LEMD2, LGALS3BP, LIG1, LIG3, LIPA, LLGL1, LMCD1, LMNB1, LMO7, LNPEP, LPCAT2, LPCAT4, LPP, LRCH4, LRPPRC, LRRC32, LRRC8A, LRRC8E, LRRFIP1, LRRK1, LRWD1, LSS, LTBP1, LUM, LYPLA2, M6PR, MACF1, MACROD1, MACROH2A1, MAD1L1, MAD2L1, MAEA, MAGED2, MAGI1, MALT1, MAN2B2, MAOA, MAP1LC3A, MAP2K3, MAP3K20, MARCHF5, MATR3, MBD3, MBOAT2, MBOAT7, MCAM, MCM2, MCM3,	

					MCM4, MCM5, MCM6, MCM7, MCMBP, MDC1, ME1, MEAK7, MED17, MED23, MED30, MEST, METTL14, MFF, MFN1, MFSD10, MGA, MGAT1, MGMT, MGST1, MICAL3, MICALL1, MLF2, MLH1, MMAB, MME, MMUT, MOCS2, MOGS, MORF4L1, MOV10, MPHOSPH8, MPST, MRM3, MRPL10, MRPS17, MRPS21, MRPS31, MRRF, MRTO4, MSH2, MSH6, MSI2, MSN, MTA1, MTAP, MTCH1, MTHFD2, MTREX, MTRR, MVD, MVK, MVP, MYD88, MYH10, MYH9, MYO18A, MYO1B, MYO1D, MYO1E, MYO6, MYOF, N6AMT1, NCAPD2, NCAPG, NCAPH, NCDN, NCK2, NCL, NCLN, NCOA5, NCOA7, NDRG1, NDUFA13, NDUFA4, NDUFAF7, NDUFS3, NEDD1, NEDD4, NEDD8, NEK1, NELFCD, NENF, NF1, NF2, NFATC2IP, NFKB2, NHLRC2, NIBAN2, NISCH, NLE1, NMD3, NMT1, NNT, NOCT, NOL11, NOM1, NOP53, NPLOC4, NPM1, NSDHL, NSMCE3, NSUN5, NT5C, NUB1, NUBP2, NUBPL, NUCB2, NUDT16, NUDT16L1, NUDT19, NUF2, NUP205, NUP35, NUP62, NUTF2, NXN, OAT, OBSL1, OGFOD1, ORC4, ORC5, OSBPL10, OSBPL11, OSBPL1A, OSBPL2, OSBPL8, OXA1L, OXSR1, P3H1, P3H3, PACS1, PACSIN3, PAFAH1B1, PAFAH1B2, PALLD, PANK4, PANX1, PARG, PARL, PARP1, PARP14, PARP4, PATZ1, PAWR, PBXIP1, PC, PCBP3, PCDH7, PCK2, PCYT2, PDAP1, PDCD6IP, PDK1, PDS5B, PDXDC1, PEBP1, PELO, PFKM, PGAM5, PHGDH,	

					PHPT1, PI4K2B, PIEZO1, PIGO, PIGT, PIGU, PIK3C2A, PIN1, PIP4K2B, PIR, PITHD1, PITRM1, PKM, PKN2, PLA2G15, PLA2G4A, PLBD2, PLCB3, PLCG1, PLD1, PLD3, PLOD2, PLPP3, PML, PMM2, PNP, PNPLA4, PNPLA6, POFUT1, POGLUT3, POGZ, POLD1, POLD2, POLE2, POLR1C, POLR1D, POLR2A, POLR2B, POLR2H, POLR2I, POLR3A, POLR3C, POMGNT1, POMGNT2, POMK, POP1, PPIB, PPIE, PPIG, PPM1B, PPP1CA, PPP1R7, PPP2R5A, PPP3CA, PPP4R3A, PPP5C, PPWD1, PRDX1, PRDX6, PRIM2, PRKAA1, PRKAB2, PRKCI, PRKCSH, PRKG1, PRMT7, PRPF19, PRUNE1, PRXL2B, PSIP1, PSMA3, PSMB10, PSMB2, PSMC2, PSMC3, PSMC6, PSMD1, PSMD12, PSMD14, PSMD2, PSMD6, PSMD7, PSMD8, PSME1, PSME2, PSME4, PSMG1, PSPC1, PTBP2, PTBP3, PTGES2, PTGIS, PTPA, PTPMT1, PTPN12, PTRH2, PUF60, PUS7, PWWP2A, PYCARD, PYCR1, PYGB, QARS1, QSOX1, QTRT1, QTRT2, RAB10, RAB11B, RAB1A, RAB23, RAB3B, RAB3D, RAB5A, RAB8A, RABGEF1, RABL6, RAC3, RAD21, RAD9A, RAI14, RALA, RANBP1, RANGAP1, RAP1GDS1, RAP2B, RAP2C, RARS2, RBBP4, RBM22, RBM26, RBM4, RBMS2, RBP1, RCC1, RCN1, RCN2, RCN3, RDH10, RDH11, RECQL, REEP2, REEP6, RELCH, RETSAT, RFC2, RFC3, RFC5, RGS10, RHEB, RHOB, RHOC, RIF1, RNF114, RNF2, RNF213, RNF40, RNGTT, RNPS1, RPA1, RPA2, RPF2,	

					RPL10A, RPL13, RPL13A, RPL22, RPL24, RPL27, RPL36, RPL5, RPRD1A, RPRD1B, RPRD2, RPS13, RPS15A, RPS16, RPS18, RPS21, RPS23, RPS4Y1, RPS5, RPS8, RPS9, RRAS2, RRM1, RTN4, RYBP, S100A10, S100A16, SAAL1, SACS, SAE1, SAMHD1, SAP30BP, SARM1, SARNP, SARS1, SBDS, SCAF11, SCAI, SCARB2, SCFD1, SCLY, SCP2, SCRIB, SCRN1, SDHC, SEC11C, SEC14L2, SEC23B, SEC61A1, SEC61B, SELENBP1, SELENOT, SENP3, SEPTIN2, SEPTIN7, SEPTIN8, SEPTIN9, SERPINB1, SERPINB9, SERPINE2, SESN2, SET, SEZ6L2, SF1, SF3A3, SF3B6, SFXN2, SFXN3, SGSH, SH3BGRL2, SH3BGRL3, SH3BP4, SH3KBP1, SHFL, SHMT2, SHPRH, SIAE, SIRT1, SLC11A2, SLC12A2, SLC16A3, SLC17A5, SLC1A1, SLC1A4, SLC1A5, SLC25A10, SLC25A12, SLC25A13, SLC25A15, SLC25A19, SLC25A22, SLC25A24, SLC25A26, SLC25A4, SLC25A5, SLC25A6, SLC2A1, SLC30A9, SLC31A1, SLC44A1, SLC44A2, SLC4A7, SLC7A6, SLC9A1, SMAD4, SMARCA1, SMARCAD1, SMARCB1, SMARCC1, SMARCD3, SMARCE1, SMC1A, SMC2, SMC3, SMC4, SMCHD1, SMNDC1, SMPD1, SMU1, SMYD3, SNF8, SNRNP200, SNRPA, SNRPD3, SNRPE, SNU13, SNW1, SNX1, SNX18, SNX21, SNX27, SNX5, SOAT1, SOD1, SOD2, SON, SORBS3, SORT1, SPAG7, SPARC, SPATS2L, SPECC1, SPHK2, SPTLC1, SPTLC2,	

					SQSTM1, SRA1, SRC, SRD5A3, SRPRA, SRSF10, SRSF2, SRSF5, SSB, SSR3, SSRP1, STAG2, STAT1, STAT2, STAT5B, STK24, STOM, STON1, STRN3, STXBP3, SUFU, SUGT1, SULT1E1, SUPT16H, SURF1, SVIL, SYNE2, SYNPO, SYPL2, TAB2, TAF6L, TALDO1, TAP1, TBC1D17, TBCA, TBK1, TBPL1, TCP1, TDRD3, TF, TFAM, TGFBI, TGM2, TGS1, THADA, THBS1, THEM4, THOC2, TIGAR, TIMM17B, TIMMDC1, TJP1, TJP2, TKT, TM9SF4, TMA16, TMED9, TMEM119, TMEM168, TMEM192, TMEM65, TMEM97, TMX3, TMX4, TNIK, TNPO2, TOM1, TOM1L2, TOMM34, TOMM7, TOR1AIP1, TOR1AIP2, TOR1B, TPBG, TPI1, TPP1, TPP2, TPRG1L, TPST2, TPX2, TRA2A, TRA2B, TRADD, TRAM1, TRAM2, TRAPPC4, TRAPPC8, TRIM21, TRIM25, TRIM28, TRIM56, TRIM59, TRIP12, TRIP13, TRIP6, TRMT10C, TRMT5, TSC2, TSEN15, TSEN34, TSPO, TSPYL1, TTC1, TTC5, TTLL12, TUBB, TUBB3, TUBGCP2, TUT4, TXN, U2SURP, UACA, UAP1, UBA2, UBA3, UBAC2, UBE2L6, UBE2Q1, UBR2, UBR4, UBTF, UBXN2A, UFD1, UGP2, UHRF2, UMPS, UNC93B1, UQCR10, UQCR11, UQCRC2, URB2, URI1, URM1, USP11, USP22, USP28, USP4, USP48, USP5, USP7, USP9X, USP9Y, UST, UTP14A, UTP20, UTP3, VARS1, VAT1, VDAC1, VDAC2, VLDLR, VPS25, VPS26B, VPS33B, VPS36, VPS41, VRK1, VRK3, VTN, VWA5A, VWA8, WASHC5, WDR11, WDR12, WDR41, WDR44,	

					WDR5, WDR70, WFS1, WIPI2, WRNIP1, WWP2, XIAP, XPNPEP3, XPO4, XPO6, XPOT, XYLT2, YAP1, YARS1, YBEY, YBX1, YEATS4, YES1, YKT6, YRDC, YTHDC1, ZC3HAV1, ZDHHC13, ZDHHC21, ZFP36L2, ZMPSTE24, ZMYM3, ZNF148, ZNF207, ZNF512]	
intracellular organelle lumen	GO_CellularComponent-EBI-UniProt-GOA-ACAP-ARAP_13.05.2021_00h00	3.39E-73	12.08981	700	[AASS, ABAT, ABCE1, ABT1, ACACA, ACAD8, ACADM, ACADSB, ACADVL, ACOT7, ACOT8, ACOX1, ACSF2, ACSS2, ACTB, ACTR2, ADAM10, ADARB1, ADD3, ADK, AFDN, AGO2, AGPS, AGRN, AHCTF1, AK3, AK4, AK6, AKR1B1, ALB, ALDH2, ALDOA, APAF1, APEX1, APLP2, APP, APRT, AQR, ARFGEF1, ARHGAP17, ARID1B, ARL14EP, ARPP19, ARRB1, ASAH1, ASH2L, ASPSCR1, ATG7, ATP2B1, ATP6V0A1, ATP6V1A, B2M, BAIAP2, BANF1, BAZ1B, BCAS2, BLVRB, BMS1, BPNT2, C1QBP, CAMK2D, CANX, CAP1, CASK, CASP3, CASP6, CBX1, CBX2, CBX3, CBX5, CBX8, CC2D1A, CCDC22, CCNH, CCNK, CCNL1, CCT2, CDC34, CDC5L, CDC73, CDK1, CDK2, CDK5RAP3, CELF2, CENPV, CETN2, CFAP20, CFL2, CHAF1B, CHAMP1, CHD1L, CHD4, CHD7, CHID1, CHPF, CHTOP, CIRBP, CLU, CMPK1, CMTR1, CNGB1, COG7, COIL, COL11A1, COL12A1, COL15A1, COL18A1, COL4A1, COL5A1, COL5A2, COL6A1, COLEC12, COQ5, COX4I1, CPSF2, CRABP2, CROT, CRTAP, CSPG4, CSTB, CSTF3, CTBP1, CTNNB1, CTNNBL1, CTSA, CTSC, CUL1, CUL4B, CUX1, CWC22, CYB5R3, CYCS, CYRIB, DAB2, DAGLB, DCN, DCPS, DDI2, DDX1, DDX42, DDX47, DGLUCY, DGUOK, DHX36,	Upregulation

					DIMT1, DLST, DNMT1, DNTTIP2, DRG1, DST, DUSP23, DUSP3, DUT, DVL2, EARS2, ECI2, EDF1, EED, EEF1E1, EIF2D, EIF3A, EIF3E, EIF6, ELP3, ELP4, ELP5, EMG1, ENY2, EOGT, EPB41L2, EPHA3, EPPK1, ERAP1, ERCC5, ERP44, ETFDH, ETHE1, EXD2, EXTL2, EZR, FAHD1, FAM3A, FANCI, FASN, FBLIM1, FDXR, FEN1, FERMT2, FHL2, FHOD1, FIBP, FKBP5, FLII, FMR1, FN1, FSCN1, FTH1, FTL, FTO, GARS1, GATAD2A, GCAT, GCC2, GCDH, GCFC2, GEMIN4, GET3, GLA, GLE1, GLO1, GLUD1, GLYR1, GNL3L, GOLGA3, GOLGA7, GOLT1B, GOT1, GPC1, GPC6, GPI, GPKOW, GPN1, GPSM1, GRWD1, GSDMD, GSK3B, GSTP1, GTF2E1, GTF2F1, GTF2F2, GTF2I, GTF3C3, GTF3C4, H1-10, HACL1, HADH, HADHA, HAT1, HDAC2, HDAC4, HERC2, HERC4, HEXB, HGS, HIBCH, HINT1, HIRIP3, HMOX1, HNRNPA0, HNRNPA3, HNRNPC, HNRNPH1, HNRNPR, HNRNPUL1, HPX, HSD17B10, HSD17B4, HSP90AA1, HSP90B1, HSPA5, HSPB6, HSPE1, HSPG2, HTATSF1, IARS2, IDH2, IGF2BP1, IGFBP7, IKBKG, ILF3, ILKAP, INPP4A, INTS1, INTS9, IPO5, IST1, ITIH4, IVNS1ABP, KAT7, KAT8, KDM1A, KDM3B, KDM4B, KPNB1, KRT18, KRT8, LAMC1, LANCL2, LAP3, LARP4B, LGALS3BP, LIG1, LIG3, LIPA, LMCD1, LMNB1, LMO7, LNPEP, LRCH4, LRPPRC, LRRC32, LRWD1, LTBP1, LUM, MACROD1, MACROH2A1, MAD2L1, MAEA, MAGED2, MAGI1, MALT1, MAN2B2,	

					MAP2K3, MATR3, MBD3, MCM2, MCM3, MCM4, MCM5, MCM6, MCM7, MCMBP, MDC1, MEAK7, MED17, MED23, MED30, METTL14, MGA, MGMT, MICAL3, MLH1, MMAB, MMUT, MOCS2, MORF4L1, MPHOSPH8, MPST, MRM3, MRPL10, MRPS17, MRPS21, MRPS31, MRRF, MRTO4, MSH2, MSH6, MTA1, MTAP, MTCH1, MTHFD2, MTREX, MTRR, MVP, MYO1E, MYO6, NCAPD2, NCAPG, NCAPH, NCL, NCOA5, NDRG1, NDUFA13, NDUFAF7, NDUFS3, NEDD1, NEDD4, NEDD8, NEK1, NELFCD, NF1, NF2, NFKB2, NHLRC2, NIBAN2, NISCH, NLE1, NMD3, NOCT, NOL11, NOM1, NOP53, NPLOC4, NPM1, NSMCE3, NSUN5, NUB1, NUBPL, NUDT16, NUDT19, NUF2, NUP205, NUP35, NUP62, NUTF2, OAT, OGFOD1, ORC4, ORC5, OSBPL11, OXA1L, P3H1, PAFAH1B2, PARG, PARP1, PATZ1, PC, PCK2, PDAP1, PDK1, PDS5B, PHPT1, PIK3C2A, PIN1, PIP4K2B, PIR, PITRM1, PKM, PKN2, PLA2G15, PLBD2, PLD3, PML, PMM2, PNP, POGLUT3, POGZ, POLD1, POLD2, POLE2, POLR1C, POLR1D, POLR2A, POLR2B, POLR2H, POLR2I, POLR3A, POLR3C, POP1, PPIB, PPIE, PPIG, PPM1B, PPP1CA, PPP3CA, PPP4R3A, PPP5C, PPWD1, PRDX6, PRIM2, PRKAA1, PRKAB2, PRKCI, PRKCSH, PRMT7, PRPF19, PSIP1, PSMA3, PSMB10, PSMB2, PSMC2, PSMC3, PSMC6, PSMD1, PSMD12, PSMD14, PSMD2, PSMD6, PSMD7, PSMD8, PSME1,	

					PSME2, PSME4, PSMG1, PSPC1, PTGES2, PTPA, PTPMT1, PTPN12, PUF60, PYCARD, PYCR1, PYGB, QARS1, QSOX1, RAB5A, RABGEF1, RAD21, RAD9A, RAI14, RANGAP1, RARS2, RBBP4, RBM22, RBM4, RBP1, RCC1, RCN1, RCN2, RCN3, RECQL, RFC2, RFC3, RFC5, RIF1, RNF2, RNF213, RNF40, RNGTT, RNPS1, RPA1, RPA2, RPF2, RPL13, RPL13A, RPL27, RPL36, RPL5, RPRD1A, RPRD1B, RPRD2, RPS13, RPS15A, RPS16, RPS18, RPS21, RPS23, RPS4Y1, RPS5, RPS8, RPS9, RYBP, S100A16, SAAL1, SAE1, SAMHD1, SAP30BP, SARNP, SBDS, SCAF11, SCAI, SCARB2, SCP2, SCRIB, SEC14L2, SELENBP1, SENP3, SEPTIN2, SERPINB1, SERPINB9, SET, SF1, SF3A3, SF3B6, SGSH, SH3BGRL2, SH3BGRL3, SHFL, SHMT2, SHPRH, SIRT1, SLC25A10, SLC25A15, SLC25A5, SLC44A1, SLC9A1, SMAD4, SMARCA1, SMARCAD1, SMARCB1, SMARCC1, SMARCD3, SMARCE1, SMC1A, SMC2, SMC3, SMC4, SMCHD1, SMNDC1, SMPD1, SMU1, SMYD3, SNF8, SNRNP200, SNRPA, SNRPD3, SNRPE, SNU13, SNW1, SNX27, SOAT1, SOD1, SOD2, SON, SPARC, SPATS2L, SPECC1, SPHK2, SQSTM1, SRA1, SRC, SRSF10, SRSF2, SRSF5, SSRP1, STAG2, STAT1, STAT2, STAT5B, STK24, STON1, STRN3, SUFU, SUPT16H, SYNE2, TAB2, TAF6L, TBCA, TBK1, TBPL1, TDRD3, TF, TFAM, TGS1, THBS1, THEM4, THOC2, TIMMDC1, TJP2, TKT, TMA16,	

					TMEM192, TNIK, TOR1B, TPP1, TPP2, TPX2, TRA2A, TRA2B, TRIM21, TRIM25, TRIM28, TRIM56, TRIP12, TRMT10C, TRMT5, TSEN15, TSEN34, TSPO, TSPYL1, TTC5, TUBB, TUBGCP2, TUT4, TXN, U2SURP, UACA, UAP1, UBA2, UBE2L6, UBR4, UBTF, UFD1, UHRF2, UQCRC2, URB2, URI1, USP11, USP22, USP28, USP48, USP7, UTP14A, UTP20, UTP3, VAT1, VDAC1, VDAC2, VPS25, VRK1, VRK3, VWA5A, WASHC5, WDR12, WDR5, WFS1, WIPI2, XIAP, XPO4, XPO6, XPOT, YAP1, YARS1, YBX1, YEATS4, YTHDC1, ZFP36L2, ZMYM3, ZNF148, ZNF207]	
nucleic acid metabolic process	GO_BiologicalProcess-EBI-UniProt-GOA-ACAP-ARAP_13.05.2021_00h00	3.39E-73	9.522024	508	[AAR2, AARS1, AASS, ABCE1, ABT1, ACTR2, ACTR3, ADARB1, ADNP, AGO2, AGRN, AKR1B1, ANXA3, APBB2, APEX1, APP, AQR, ARHGEF11, ARID1B, ARRB1, BAZ1B, BCAS2, BMS1, BRD3, C1QBP, CAMK2D, CASK, CASP3, CBX1, CBX2, CBX3, CBX5, CBX8, CC2D1A, CCDC22, CCN2, CCNH, CCNK, CCNL1, CCT2, CCT3, CCT6A, CD200, CD81, CDC34, CDC5L, CDC73, CDK1, CDK16, CDK17, CDK2, CDK5RAP3, CECR2, CELF2, CENPV, CETN2, CHAF1B, CHD1L, CHD2, CHD4, CHD7, CHORDC1, CHP1, CHTOP, CIRBP, CLU, CMTR1, CNGB1, CNOT1, CNOT10, COIL, COLEC12, CPSF2, CRABP2, CSTF3, CTBP1, CTNNB1, CTNNBL1, CTU1, CUL4B, CUX1, CWC22, DAB2, DARS1, DCPS, DDX1, DDX42, DDX47, DHFR, DHX34, DHX36, DICER1, DIMT1, DNMT1, DRG1, DVL2, EARS2, EDF1, EED, EEF1E1, EFEMP1, EIF2AK2, EIF3E, EIF6, ELP3,	Upregulation

					ELP4, ELP5, EMG1, ENPP1, ENY2, EPRS1, ERBB2, ERCC5, EXD2, EZR, FANCI, FEN1, FGF2, FHL2, FHOD1, FMR1, FSCN1, FTO, GARS1, GATAD2A, GATB, GCFC2, GEMIN4, GLMP, GLO1, GLYR1, GNL3L, GPI, GPKOW, GTF2E1, GTF2F1, GTF2F2, GTF2I, GTF3C3, GTF3C4, H1-10, H1-5, HARS1, HAT1, HDAC2, HDAC4, HDGFL2, HERC2, HEXB, HINT1, HMCES, HMOX1, HNRNPA0, HNRNPA3, HNRNPC, HNRNPH1, HNRNPLL, HNRNPR, HNRNPUL1, HSD17B10, HSD17B4, HSP90AA1, HSPA5, HTATSF1, IARS2, IGF2BP1, IGF2BP2, IKBKB, IKBKG, ILF3, IMPACT, INTS1, INTS9, IVNS1ABP, JAG1, KAT7, KAT8, KDM1A, KDM3B, KDM4B, KPNB1, LANCL2, LARP1, LARP4B, LARP6, LIG1, LIG3, LIMS1, LMCD1, LMO7, LRPPRC, LRRFIP1, LRWD1, LUM, MACROH2A1, MAGI1, MALT1, MAP2K3, MAP3K4, MBD3, MCM2, MCM3, MCM4, MCM5, MCM6, MCM7, MDC1, MED17, MED23, MED30, METTL14, MGA, MGMT, MLF2, MLH1, MMAB, MORF4L1, MOV10, MPHOSPH8, MRM3, MRTO4, MSH2, MSH6, MTA1, MTREX, MTRR, MYD88, MYO18A, MYO6, N6AMT1, NCK2, NCL, NCOA5, NCOA7, NDRG1, NDUFA13, NEDD4, NEDD8, NELFCD, NFATC2IP, NFKB2, NIBAN2, NMD3, NOCT, NOL11, NOP53, NPLOC4, NPM1, NSMCE3, NSUN5, NT5C3B, NUDT15, NUDT16, NUDT16L1, NUP35, NUP62, ORC4, ORC5, OXSR1, PAIP1, PARG, PARP1, PARP14, PARP4, PATZ1,	

					PAWR, PBXIP1, PCBP3, PDS5B, PELO, PFKM, PIK3CA, PIN1, PIR, PITHD1, PLD3, PLPP3, PML, POFUT1, POGZ, POLD1, POLD2, POLE2, POLR1C, POLR1D, POLR2A, POLR2B, POLR2H, POLR2I, POLR3A, POLR3C, POP1, PPIE, PPIG, PPP3CA, PPP5C, PPWD1, PRDX6, PRIM2, PRKAA1, PRKCI, PRMT7, PRPF19, PSIP1, PSMA3, PSMB10, PSMB2, PSMC2, PSMC3, PSMC6, PSMD1, PSMD12, PSMD14, PSMD2, PSMD6, PSMD7, PSMD8, PSME1, PSME2, PSME4, PSPC1, PTBP2, PTBP3, PTGES2, PTGIS, PTRH2, PUF60, PUS7, PXN, PYCARD, QARS1, QTRT1, QTRT2, RAD21, RAD9A, RAP2C, RARS2, RBM22, RBM26, RBM4, RBMS2, RECQL, RFC2, RFC3, RFC5, RIF1, RIOK3, RIPK2, RNF2, RNF40, RNGTT, RNPS1, ROR1, RPA1, RPA2, RPF2, RPL10A, RPL13, RPL13A, RPL18A, RPL22, RPL24, RPL27, RPL32, RPL36, RPL38, RPL5, RPLP1, RPLP2, RPRD1A, RPRD1B, RPRD2, RPS13, RPS15A, RPS16, RPS18, RPS21, RPS23, RPS4Y1, RPS5, RPS8, RPS9, RYBP, SAMHD1, SAP30BP, SARNP, SARS1, SBDS, SCAF11, SCAI, SEC14L2, SENP3, SESN2, SET, SF1, SF3A3, SF3B6, SHPRH, SIRT1, SLC25A15, SLC30A9, SLC9A1, SMAD4, SMARCA1, SMARCAD1, SMARCB1, SMARCC1, SMARCD3, SMARCE1, SMC1A, SMC3, SMCHD1, SMNDC1, SMU1, SMYD3, SNF8, SNRNP200, SNRPA, SNRPD3, SNRPE, SNU13, SNW1,	

					SNX5, SOD2, SON, SORBS3, SQSTM1, SRA1, SRC, SRSF10, SRSF2, SRSF5, SSB, SSRP1, STAT1, STAT2, STAT5B, STON1, STRN3, SUFU, SUPT16H, TAB2, TAF6L, TAX1BP1, TBK1, TBPL1, TCP1, TDRD3, TF, TFAM, TGS1, THADA, THOC2, TIGAR, TRA2A, TRA2B, TRADD, TRIM21, TRIM25, TRIM28, TRIM56, TRIP12, TRIP13, TRMT10C, TRMT2A, TRMT5, TSEN15, TSEN34, TSR2, TTC5, TUT4, TXN, U2SURP, UBA2, UBA3, UBE2L6, UBR2, UBTF, UFD1, UHRF2, URI1, URM1, USP22, USP28, USP4, USP7, USP9X, UTP14A, UTP20, UTP3, VARS1, WDR12, WDR70, WFS1, WRNIP1, WWC2, WWP2, YAP1, YARS1, YBEY, YBX1, YEATS4, YES1, YTHDC1, ZC3HAV1, ZFP36L2, ZMPSTE24, ZNF148]	
nucleus	GO_CellularComponent-EBI-UniProt-GOA-ACAP-ARAP_13.05.2021_00h00	3.39E-73	10.286442	808	[AAR2, AASS, ABI1, ABT1, ACACA, ACADM, ACADVL, ACOT7, ACSS2, ACTB, ACTG1, ACTR2, ACTR3, ADAM10, ADARB1, ADD3, ADK, ADNP, AFDN, AGO2, AGPS, AHCTF1, AK3, AK6, AKR1B1, ALB, ALDOA, AMOTL1, ANXA7, APBB2, APEX1, APLP2, APP, APPL2, APRT, AQR, ARFGEF1, ARHGAP17, ARID1B, ARL14EP, ARPC4, ARPP19, ARRB1, ASAH1, ASH2L, ASPM, ASPSCR1, ATE1, ATP2B1, ATP6V0A1, ATP6V1A, BAG1, BAIAP2, BANF1, BAZ1B, BCAS2, BIN1, BLVRB, BMS1, BNIP1, BPNT2, BRD3, C1QBP, C3orf38, CAMK2D, CASK, CASP3, CASP6, CBX1, CBX2, CBX3, CBX5, CBX8, CC2D1A, CCDC22, CCNH, CCNK, CCNL1, CDC34, CDC5L, CDC73, CDK1,	Upregulation

					CDK16, CDK17, CDK2, CDK5RAP3, CECR2, CELF2, CEMIP, CENPV, CERS6, CETN2, CFAP20, CFAP298, CFL2, CHAF1B, CHAMP1, CHD1L, CHD2, CHD4, CHD7, CHID1, CHORDC1, CHP1, CHTOP, CIRBP, CKB, CLIC1, CLIP2, CLU, CMPK1, CMTR1, CNGB1, CNOT1, CNOT10, COG7, COIL, COLEC12, COMMD4, COX4I1, CPNE2, CPSF2, CRABP2, CRBN, CSPG4, CSRP2, CSTB, CSTF3, CTBP1, CTNNB1, CTNNBL1, CTSC, CUL1, CUL4B, CUX1, CWC22, CXCL12, CYCS, DAB2, DAGLB, DAPK1, DCPS, DCXR, DDI2, DDX1, DDX42, DDX47, DGUOK, DHCR24, DHCR7, DHX36, DIAPH1, DIAPH3, DICER1, DIMT1, DLST, DNMT1, DNTTIP2, DPCD, DPP9, DRG1, DSP, DST, DUSP23, DUSP3, DUT, DVL2, DYNLL1, EDF1, EED, EEF1E1, EEF1G, EHD3, EIF2AK2, EIF2D, EIF2S1, EIF3A, EIF3E, EIF6, ELP3, ELP4, ELP5, EMG1, EML3, ENY2, EPB41L2, EPHA3, EPM2AIP1, EPPK1, ERBB2, ERCC5, ETHE1, EXD2, EXTL2, EZR, FAHD1, FAM120A, FAM3A, FANCI, FASN, FAT1, FBLIM1, FBXL12, FEN1, FERMT2, FGF2, FHL1, FHL2, FHOD1, FIBP, FKBP5, FLII, FMR1, FSCN1, FSD1, FTH1, FTO, G6PD, GAMT, GAPDH, GARS1, GATAD2A, GCAT, GCC2, GCFC2, GDAP1, GEMIN4, GET3, GLE1, GLMP, GLO1, GLRX3, GLUL, GLYR1, GNL3L, GOLGA3, GOLT1B, GOT1, GPC1, GPC6, GPI, GPKOW, GPN1, GPSM1, GRWD1, GSDMD, GSK3B, GSTM3, GSTP1, GTF2E1, GTF2F1, GTF2F2, GTF2I, GTF3C3, GTF3C4, H1-10, H1-5, HACL1,	

					HADH, HADHA, HAT1, HDAC2, HDAC4, HDGFL2, HELZ, HERC2, HERC4, HHIP, HINT1, HIP1, HIRIP3, HMOX1, HNRNPA0, HNRNPA3, HNRNPC, HNRNPH1, HNRNPLL, HNRNPR, HNRNPUL1, HSD17B4, HSP90AA1, HSP90B1, HSPA14, HSPA4, HSPA5, HSPB6, HTATSF1, IFRD2, IGF2BP1, IGF2BP2, IKBKB, IKBKG, ILF3, ILKAP, INPP4A, INTS1, INTS9, IPO5, IPO9, IST1, ITM2C, ITSN1, IVNS1ABP, KAT7, KAT8, KDM1A, KDM3B, KDM4B, KNTC1, KPNB1, KRT18, KRT8, LAMA5, LANCL2, LAP3, LARP4B, LARP6, LBR, LDHA, LEMD2, LIG1, LIG3, LIPA, LMCD1, LMNB1, LMO7, LPP, LRCH4, LRPPRC, LRRC32, LRRFIP1, LRWD1, MACROD1, MACROH2A1, MAD1L1, MAD2L1, MAEA, MAGED2, MAGI1, MALT1, MAP2K3, MAP3K20, MATR3, MBD3, MCAM, MCM2, MCM3, MCM4, MCM5, MCM6, MCM7, MCMBP, MDC1, MEAK7, MED17, MED23, MED30, METTL14, MFSD10, MGA, MGMT, MGST1, MICAL3, MLF2, MLH1, MMAB, MOCS2, MORF4L1, MOV10, MPHOSPH8, MRPL10, MRPS31, MRTO4, MSH2, MSH6, MSN, MTA1, MTAP, MTREX, MTRR, MVP, MYD88, MYH10, MYH9, MYO1E, MYO6, MYOF, N6AMT1, NCAPD2, NCAPG, NCAPH, NCDN, NCL, NCOA5, NCOA7, NDRG1, NDUFA13, NDUFS3, NEDD1, NEDD4, NEDD8, NEK1, NELFCD, NF1, NF2, NFATC2IP, NFKB2, NIBAN2, NISCH, NLE1, NMD3, NOCT, NOL11,	

					NOM1, NOP53, NPLOC4, NPM1, NSMCE3, NSUN5, NT5C, NUB1, NUBP2, NUCB2, NUDT16, NUDT16L1, NUDT19, NUF2, NUP205, NUP35, NUP62, NUTF2, NXN, OAT, OGFOD1, ORC4, ORC5, OSBPL11, OSBPL8, OXSR1, PAFAH1B1, PAFAH1B2, PALLD, PANK4, PARG, PARL, PARP1, PARP14, PARP4, PATZ1, PAWR, PBXIP1, PCBP3, PDS5B, PEBP1, PELO, PFKM, PHGDH, PHPT1, PIK3C2A, PIN1, PIP4K2B, PIR, PITHD1, PKM, PKN2, PLA2G15, PLA2G4A, PLCB3, PLCG1, PML, PMM2, PNP, POGZ, POLD1, POLD2, POLE2, POLR1C, POLR1D, POLR2A, POLR2B, POLR2H, POLR2I, POLR3A, POLR3C, POP1, PPIB, PPIE, PPIG, PPM1B, PPP1CA, PPP1R7, PPP2R5A, PPP3CA, PPP4R3A, PPP5C, PPWD1, PRDX1, PRDX6, PRIM2, PRKAA1, PRKAB2, PRKCI, PRMT7, PRPF19, PRUNE1, PSIP1, PSMA3, PSMB10, PSMB2, PSMC2, PSMC3, PSMC6, PSMD1, PSMD12, PSMD14, PSMD2, PSMD6, PSMD7, PSMD8, PSME1, PSME2, PSME4, PSMG1, PSPC1, PTBP2, PTBP3, PTGES2, PTGIS, PTPA, PTPMT1, PTPN12, PUF60, PUS7, PWWP2A, PYCARD, QTRT1, RAB5A, RABGEF1, RABL6, RAD21, RAD9A, RAI14, RANBP1, RANGAP1, RBBP4, RBM22, RBM26, RBM4, RBMS2, RBP1, RCC1, RCN2, RDH10, RECQL, REEP6, RETSAT, RFC2, RFC3, RFC5, RGS10, RHEB, RHOB, RHOC, RIF1, RNF114, RNF2, RNF213, RNF40, RNGTT, RNPS1, RPA1, RPA2, RPF2, RPL10A, RPL13, RPL13A,	

					RPL22, RPL27, RPL36, RPL5, RPRD1A, RPRD1B, RPRD2, RPS13, RPS15A, RPS16, RPS18, RPS21, RPS23, RPS4Y1, RPS5, RPS8, RPS9, RRM1, RTN4, RYBP, S100A16, SAAL1, SACS, SAE1, SAMHD1, SAP30BP, SARNP, SARS1, SBDS, SCAF11, SCAI, SCP2, SCRIB, SCRN1, SEC14L2, SELENBP1, SENP3, SEPTIN2, SEPTIN7, SERPINB9, SESN2, SET, SF1, SF3A3, SF3B6, SH3BGRL2, SH3BGRL3, SH3BP4, SHFL, SHMT2, SHPRH, SIRT1, SLC11A2, SLC16A3, SLC25A10, SLC25A15, SLC25A19, SLC25A5, SLC25A6, SLC2A1, SLC30A9, SLC44A1, SLC9A1, SMAD4, SMARCA1, SMARCAD1, SMARCB1, SMARCC1, SMARCD3, SMARCE1, SMC1A, SMC2, SMC3, SMC4, SMCHD1, SMNDC1, SMU1, SMYD3, SNF8, SNRNP200, SNRPA, SNRPD3, SNRPE, SNU13, SNW1, SNX27, SOD1, SON, SORBS3, SORT1, SPAG7, SPARC, SPATS2L, SPECC1, SPHK2, SQSTM1, SRA1, SRC, SRSF10, SRSF2, SRSF5, SSB, SSRP1, STAG2, STAT1, STAT2, STAT5B, STK24, STON1, STRN3, SUFU, SUGT1, SULT1E1, SUPT16H, SVIL, SYNE2, SYNPO, TAB2, TAF6L, TALDO1, TBCA, TBK1, TBPL1, TDRD3, TFAM, TGM2, TGS1, THOC2, TIGAR, TIMMDC1, TJP1, TJP2, TKT, TMA16, TMEM192, TMEM97, TMX4, TNIK, TNPO2, TOMM34, TOR1AIP1, TOR1AIP2, TOR1B, TPI1, TPP2, TPX2, TRA2A, TRA2B, TRADD, TRIM21, TRIM25, TRIM28,	

					TRIM56, TRIP12, TRIP13, TRIP6, TRMT10C, TRMT5, TSC2, TSEN15, TSEN34, TSPYL1, TTC5, TTLL12, TUBB, TUBB3, TUBGCP2, TUT4, TXN, U2SURP, UACA, UAP1, UBA2, UBA3, UBE2L6, UBE2Q1, UBR2, UBR4, UBTF, UBXN2A, UFD1, UGP2, UHRF2, UMPS, UQCRC2, URB2, URI1, URM1, USP11, USP22, USP28, USP4, USP48, USP5, USP7, USP9X, USP9Y, UTP14A, UTP20, UTP3, VDAC1, VDAC2, VPS25, VPS36, VRK1, VRK3, VWA5A, WASHC5, WDR11, WDR12, WDR5, WDR70, WIPI2, WRNIP1, WWP2, XIAP, XPO4, XPO6, XPOT, YAP1, YARS1, YBEY, YBX1, YEATS4, YTHDC1, ZC3HAV1, ZFP36L2, ZMPSTE24, ZMYM3, ZNF148, ZNF207, ZNF512]	
nucleoplasm	GO_CellularComponent-EBI-UniProt-GOA-ACAP-ARAP_13.05.2021_00h00	3.39E-73	12.011239	513	[ACADVL, ACOT7, ACSS2, ACTB, ADARB1, ADK, AFDN, AGO2, AHCTF1, AK3, AK6, AKR1B1, APEX1, APRT, AQR, ARFGEF1, ARHGAP17, ARID1B, ARL14EP, ARPP19, ARRB1, ASH2L, ASPSCR1, ATP2B1, ATP6V0A1, ATP6V1A, BAIAP2, BANF1, BAZ1B, BCAS2, BLVRB, BMS1, BPNT2, CAMK2D, CASP3, CASP6, CBX1, CBX2, CBX3, CBX5, CBX8, CCDC22, CCNH, CCNK, CCNL1, CDC34, CDC5L, CDC73, CDK1, CDK2, CELF2, CENPV, CETN2, CFAP20, CHAF1B, CHAMP1, CHD1L, CHD4, CHD7, CHTOP, CIRBP, CMPK1, CMTR1, CNGB1, COIL, COLEC12, COX4I1, CPSF2, CRABP2, CSPG4, CSTF3, CTBP1, CTNNB1, CTNNBL1, CTSC, CUL1, CUL4B, CUX1, CWC22, DAGLB, DCPS, DDI2, DDX1,	Upregulation

					DDX42, DDX47, DHX36, DIMT1, DLST, DNMT1, DNTTIP2, DRG1, DST, DUSP23, DUSP3, DUT, DVL2, EDF1, EED, EEF1E1, EIF2D, EIF3A, EIF3E, EIF6, ELP4, ELP5, EMG1, ENY2, EPB41L2, EPHA3, ERCC5, ETHE1, EXTL2, FAHD1, FANCI, FASN, FEN1, FERMT2, FHL2, FHOD1, FIBP, FKBP5, FLII, FMR1, FSCN1, FTO, GARS1, GATAD2A, GCAT, GCC2, GCFC2, GEMIN4, GET3, GLO1, GLYR1, GNL3L, GOLGA3, GOLT1B, GOT1, GPC1, GPI, GPKOW, GPN1, GPSM1, GRWD1, GSDMD, GSK3B, GTF2E1, GTF2F1, GTF2F2, GTF2I, GTF3C3, GTF3C4, H1-10, HACL1, HADH, HADHA, HAT1, HDAC2, HDAC4, HERC2, HINT1, HIRIP3, HNRNPA0, HNRNPA3, HNRNPC, HNRNPH1, HNRNPR, HNRNPUL1, HSP90AA1, HTATSF1, IGF2BP1, IKBKG, ILF3, ILKAP, INPP4A, INTS1, INTS9, IVNS1ABP, KAT7, KAT8, KDM1A, KDM3B, KDM4B, KPNB1, KRT8, LANCL2, LAP3, LIG1, LIG3, LIPA, LMCD1, LMNB1, LMO7, LRCH4, LRPPRC, LRRC32, LRWD1, MACROD1, MACROH2A1, MAD2L1, MAEA, MAGED2, MAGI1, MAP2K3, MBD3, MCM2, MCM3, MCM4, MCM5, MCM6, MCM7, MCMBP, MDC1, MEAK7, MED17, MED23, MED30, METTL14, MGA, MGMT, MICAL3, MLH1, MMAB, MOCS2, MORF4L1, MPHOSPH8, MRPL10, MSH2, MSH6, MTA1, MTAP, MTREX, MTRR, MYO1E, MYO6, NCAPD2, NCAPG, NCAPH, NCL, NCOA5, NDRG1,	

					NDUFA13, NDUFS3, NEDD1, NEDD8, NEK1, NELFCD, NFKB2, NIBAN2, NISCH, NLE1, NMD3, NOCT, NOL11, NOP53, NPLOC4, NPM1, NSMCE3, NSUN5, NUB1, NUDT16, NUDT19, NUF2, NUP35, NUP62, NUTF2, OAT, OGFOD1, ORC4, ORC5, OSBPL11, PARG, PARP1, PATZ1, PDS5B, PHPT1, PIK3C2A, PIN1, PIP4K2B, PIR, PKN2, PLA2G15, PML, PMM2, POGZ, POLD1, POLD2, POLE2, POLR1C, POLR1D, POLR2A, POLR2B, POLR2H, POLR2I, POLR3A, POLR3C, POP1, PPIB, PPIE, PPIG, PPP1CA, PPP3CA, PPP4R3A, PPP5C, PPWD1, PRIM2, PRKAA1, PRKAB2, PRKCI, PRMT7, PRPF19, PSIP1, PSMA3, PSMB10, PSMB2, PSMC2, PSMC3, PSMC6, PSMD1, PSMD12, PSMD14, PSMD2, PSMD6, PSMD7, PSMD8, PSME1, PSME2, PSME4, PSMG1, PSPC1, PTPA, PTPMT1, PTPN12, PUF60, PYCARD, RAB5A, RAD21, RAD9A, RAI14, RANGAP1, RBBP4, RBM22, RBM4, RBP1, RCC1, RECQL, RFC2, RFC3, RFC5, RIF1, RNF2, RNF40, RNGTT, RNPS1, RPA1, RPA2, RPF2, RPL27, RPL5, RPRD1A, RPRD1B, RPRD2, RPS13, RPS15A, RPS16, RPS18, RPS21, RPS23, RPS4Y1, RPS5, RPS8, RPS9, RYBP, SAAL1, SAE1, SAMHD1, SAP30BP, SARNP, SBDS, SCAF11, SCAI, SCP2, SCRIB, SEC14L2, SENP3, SEPTIN2, SERPINB9, SET, SF1, SF3A3, SF3B6, SH3BGRL2, SH3BGRL3, SHFL, SHPRH, SIRT1, SLC25A10, SLC25A15, SLC44A1, SLC9A1, SMAD4, SMARCA1,	

					SMARCAD1, SMARCB1, SMARCC1, SMARCD3, SMARCE1, SMC1A, SMC2, SMC3, SMC4, SMNDC1, SMU1, SMYD3, SNF8, SNRNP200, SNRPA, SNRPD3, SNRPE, SNU13, SNW1, SNX27, SOD1, SON, SPATS2L, SPECC1, SPHK2, SQSTM1, SRA1, SRC, SRSF10, SRSF2, SRSF5, SSRP1, STAG2, STAT1, STAT2, STAT5B, STK24, STON1, STRN3, SUFU, SUPT16H, SYNE2, TAB2, TAF6L, TBK1, TBPL1, TDRD3, TGS1, THOC2, TIMMDC1, TJP2, TKT, TMA16, TMEM192, TNIK, TPP2, TPX2, TRA2A, TRA2B, TRIM21, TRIM25, TRIM28, TRIM56, TRIP12, TRMT10C, TSEN15, TSEN34, TSPYL1, TTC5, TUBGCP2, TXN, U2SURP, UACA, UAP1, UBA2, UBE2L6, UBR4, UBTF, UFD1, UHRF2, UQCRC2, URI1, USP11, USP22, USP28, USP48, USP7, UTP14A, UTP20, UTP3, VPS25, VRK1, VRK3, VWA5A, WASHC5, WDR12, WDR5, WIPI2, XIAP, XPO4, XPO6, XPOT, YAP1, YARS1, YBX1, YEATS4, YTHDC1, ZFP36L2, ZMYM3, ZNF148, ZNF207]	
nuclear lumen	GO_CellularComponent-EBI-UniProt-GOA-ACAP-ARAP_13.05.2021_00h00	3.39E-73	12.110726	560	[ABT1, ACACA, ACADVL, ACOT7, ACSS2, ACTB, ADARB1, ADD3, ADK, AFDN, AGO2, AGPS, AHCTF1, AK3, AK6, AKR1B1, APEX1, APRT, AQR, ARFGEF1, ARHGAP17, ARID1B, ARL14EP, ARPP19, ARRB1, ASH2L, ASPSCR1, ATP2B1, ATP6V0A1, ATP6V1A, BAIAP2, BANF1, BAZ1B, BCAS2, BLVRB, BMS1, BPNT2, C1QBP, CAMK2D, CASK, CASP3, CASP6, CBX1, CBX2, CBX3, CBX5, CBX8, CC2D1A, CCDC22, CCNH, CCNK, CCNL1,	Upregulation

					CDC34, CDC5L, CDC73, CDK1, CDK2, CDK5RAP3, CELF2, CENPV, CETN2, CFAP20, CFL2, CHAF1B, CHAMP1, CHD1L, CHD4, CHD7, CHTOP, CIRBP, CMPK1, CMTR1, CNGB1, COG7, COIL, COLEC12, COX4I1, CPSF2, CRABP2, CSPG4, CSTB, CSTF3, CTBP1, CTNNB1, CTNNBL1, CTSC, CUL1, CUL4B, CUX1, CWC22, DAB2, DAGLB, DCPS, DDI2, DDX1, DDX42, DDX47, DHX36, DIMT1, DLST, DNMT1, DNTTIP2, DRG1, DST, DUSP23, DUSP3, DUT, DVL2, EDF1, EED, EEF1E1, EIF2D, EIF3A, EIF3E, EIF6, ELP3, ELP4, ELP5, EMG1, ENY2, EPB41L2, EPHA3, EPPK1, ERCC5, ETHE1, EXTL2, EZR, FAHD1, FANCI, FASN, FBLIM1, FEN1, FERMT2, FHL2, FHOD1, FIBP, FKBP5, FLII, FMR1, FSCN1, FTO, GARS1, GATAD2A, GCAT, GCC2, GCFC2, GEMIN4, GET3, GLE1, GLO1, GLYR1, GNL3L, GOLGA3, GOLT1B, GOT1, GPC1, GPI, GPKOW, GPN1, GPSM1, GRWD1, GSDMD, GSK3B, GTF2E1, GTF2F1, GTF2F2, GTF2I, GTF3C3, GTF3C4, H1-10, HACL1, HADH, HADHA, HAT1, HDAC2, HDAC4, HERC2, HERC4, HINT1, HIRIP3, HMOX1, HNRNPA0, HNRNPA3, HNRNPC, HNRNPH1, HNRNPR, HNRNPUL1, HSP90AA1, HSPB6, HTATSF1, IGF2BP1, IKBKG, ILF3, ILKAP, INPP4A, INTS1, INTS9, IPO5, IVNS1ABP, KAT7, KAT8, KDM1A, KDM3B, KDM4B, KPNB1, KRT18, KRT8, LANCL2, LAP3, LARP4B, LIG1, LIG3, LIPA, LMCD1, LMNB1, LMO7, LRCH4, LRPPRC, LRRC32, LRWD1, MACROD1,	

					MACROH2A1, MAD2L1, MAEA, MAGED2, MAGI1, MALT1, MAP2K3, MATR3, MBD3, MCM2, MCM3, MCM4, MCM5, MCM6, MCM7, MCMBP, MDC1, MEAK7, MED17, MED23, MED30, METTL14, MGA, MGMT, MICAL3, MLH1, MMAB, MOCS2, MORF4L1, MPHOSPH8, MRPL10, MRPS31, MRTO4, MSH2, MSH6, MTA1, MTAP, MTREX, MTRR, MYO1E, MYO6, NCAPD2, NCAPG, NCAPH, NCL, NCOA5, NDRG1, NDUFA13, NDUFS3, NEDD1, NEDD4, NEDD8, NEK1, NELFCD, NF1, NF2, NFKB2, NIBAN2, NISCH, NLE1, NMD3, NOCT, NOL11, NOM1, NOP53, NPLOC4, NPM1, NSMCE3, NSUN5, NUB1, NUDT16, NUDT19, NUF2, NUP205, NUP35, NUP62, NUTF2, OAT, OGFOD1, ORC4, ORC5, OSBPL11, PAFAH1B2, PARG, PARP1, PATZ1, PDS5B, PHPT1, PIK3C2A, PIN1, PIP4K2B, PIR, PKN2, PLA2G15, PML, PMM2, POGZ, POLD1, POLD2, POLE2, POLR1C, POLR1D, POLR2A, POLR2B, POLR2H, POLR2I, POLR3A, POLR3C, POP1, PPIB, PPIE, PPIG, PPM1B, PPP1CA, PPP3CA, PPP4R3A, PPP5C, PPWD1, PRIM2, PRKAA1, PRKAB2, PRKCI, PRMT7, PRPF19, PSIP1, PSMA3, PSMB10, PSMB2, PSMC2, PSMC3, PSMC6, PSMD1, PSMD12, PSMD14, PSMD2, PSMD6, PSMD7, PSMD8, PSME1, PSME2, PSME4, PSMG1, PSPC1, PTPA, PTPMT1, PTPN12, PUF60, PYCARD, RAB5A, RABGEF1, RAD21, RAD9A, RAI14, RANGAP1, RBBP4,	

					RBM22, RBM4, RBP1, RCC1, RCN2, RECQL, RFC2, RFC3, RFC5, RIF1, RNF2, RNF213, RNF40, RNGTT, RNPS1, RPA1, RPA2, RPF2, RPL13, RPL13A, RPL27, RPL36, RPL5, RPRD1A, RPRD1B, RPRD2, RPS13, RPS15A, RPS16, RPS18, RPS21, RPS23, RPS4Y1, RPS5, RPS8, RPS9, RYBP, S100A16, SAAL1, SAE1, SAMHD1, SAP30BP, SARNP, SBDS, SCAF11, SCAI, SCP2, SCRIB, SEC14L2, SELENBP1, SENP3, SEPTIN2, SERPINB9, SET, SF1, SF3A3, SF3B6, SH3BGRL2, SH3BGRL3, SHFL, SHPRH, SIRT1, SLC25A10, SLC25A15, SLC44A1, SLC9A1, SMAD4, SMARCA1, SMARCAD1, SMARCB1, SMARCC1, SMARCD3, SMARCE1, SMC1A, SMC2, SMC3, SMC4, SMCHD1, SMNDC1, SMU1, SMYD3, SNF8, SNRNP200, SNRPA, SNRPD3, SNRPE, SNU13, SNW1, SNX27, SOD1, SON, SPARC, SPATS2L, SPECC1, SPHK2, SQSTM1, SRA1, SRC, SRSF10, SRSF2, SRSF5, SSRP1, STAG2, STAT1, STAT2, STAT5B, STK24, STON1, STRN3, SUFU, SUPT16H, SYNE2, TAB2, TAF6L, TBCA, TBK1, TBPL1, TDRD3, TGS1, THOC2, TIMMDC1, TJP2, TKT, TMA16, TMEM192, TNIK, TPP2, TPX2, TRA2A, TRA2B, TRIM21, TRIM25, TRIM28, TRIM56, TRIP12, TRMT10C, TSEN15, TSEN34, TSPYL1, TTC5, TUBGCP2, TUT4, TXN, U2SURP, UACA, UAP1, UBA2, UBE2L6, UBR4, UBTF, UFD1, UHRF2, UQCRC2, URB2, URI1, USP11, USP22, USP28, USP48, USP7, UTP14A, UTP20, UTP3, VPS25,	

					VRK1, VRK3, VWA5A, WASHC5, WDR12, WDR5, WIPI2, XIAP, XPO4, XPO6, XPOT, YAP1, YARS1, YBX1, YEATS4, YTHDC1, ZFP36L2, ZMYM3, ZNF148, ZNF207]	
intrinsic component of membrane	GO_CellularComponent-EBI-UniProt-GOA-ACAP-ARAP_13.05.2021_00h00	0.663628	5.5942893	337	[ABCB7, ABCC1, ABHD12, ABHD16A, ACSL3, ACSL4, ADAM10, AFDN, AGPAT2, AGRN, ALCAM, ALDH3A2, ALG11, ANKH, ANO6, APLP2, APP, ASAH1, ATL3, ATP1A1, ATP1B3, ATP2B1, ATP2B2, ATP2C1, ATP6V0A1, ATP6V0C, ATP6V1B2, AUP1, B2M, B3GAT3, B4GALT1, B4GALT5, BCAM, BET1, BNIP1, BPNT2, BST1, C1GALT1, C2CD2L, CANX, CAPZA2, CASK, CD109, CD200, CD276, CD81, CDH11, CDIPT, CERS6, CHPF, CHSY3, CIP2A, CLGN, CLIC1, CLPTM1L, CLU, CNGB1, COL15A1, COLEC12, CORO7, COX20, COX4I1, COX6C, CPD, CSPG4, CUX1, CYP51A1, DAGLB, DCBLD2, DERL1, DHCR24, DHCR7, DHRS7B, DHX34, DHX36, DST, ECE1, EEF1E1, EFNB2, EMC1, ENPP1, EOGT, EPB41L3, EPHA2, EPHA3, EPHB4, EPHX1, ERAP1, ERBB2, ERLIN2, ESYT1, ETFDH, EXD2, EXTL2, FAM171A1, FAM3A, FASN, FAT1, FKBP1A, FLRT2, FTH1, FTO, FUT11, FUT8, GALNT1, GALNT2, GALNT7, GARS1, GBA2, GDAP1, GLMN, GLMP, GOLGA7, GOLT1B, GPC1, GPC6, GPHN, GPR108, GRAMD1A, GSDMD, GSK3B, GTF3C3, GXYLT1, GXYLT2, HDAC2, HECTD4, HELZ, HHIP, HLA-A, HLA-B, HLA-C, HSPA5, IKBKB, ILVBL, INTS1, ITGA3, ITGAV, ITGB1, ITGB5, ITM2C, ITPRIPL2, ITSN1, JAG1, JAGN1, JAM3, KCNMA1,	No change

					KCNQ1, KDSR, KIRREL1, LAMP1, LBR, LDHA, LEMD2, LLGL1, LMO7, LNPEP, LPCAT2, LPCAT4, LRCH4, LRP10, LRRC32, LRRC8A, LRRC8E, M6PR, MACF1, MAEA, MAOA, MARCHF5, MBOAT2, MBOAT7, MCAM, MEST, MFF, MFN1, MFSD10, MGAT1, MGST1, MME, MOGS, MTCH1, MYADM, MYOF, NCKAP1, NCLN, NDUFA13, NDUFA4, NECTIN2, NF1, NNT, NPR2, NSDHL, OSBPL8, OXA1L, PACC1, PANX1, PARL, PCDH7, PDZD11, PGAM5, PIEZO1, PIGO, PIGT, PIGU, PKN2, PLD3, PLPP3, PLXNB2, PNPLA6, POMGNT1, POMGNT2, POMK, PPP1R7, PROCR, PSMD2, PTBP2, PTGES2, PTGIS, PTK7, PTPA, PTPRF, PTPRG, PTRH2, QARS1, QSOX1, RAB11B, RAB3B, RAB5A, RDH10, RDH11, REEP2, REEP6, RELCH, RETREG2, ROR1, RRM1, RTN4, RTTN, SCAI, SCARB2, SCFD1, SDHC, SEC11C, SEC14L2, SEC61A1, SEC61B, SELENOT, SEMA3C, SESTD1, SEZ6L2, SFXN2, SFXN3, SLC11A2, SLC12A2, SLC16A3, SLC17A5, SLC1A1, SLC1A4, SLC1A5, SLC25A10, SLC25A12, SLC25A13, SLC25A15, SLC25A19, SLC25A22, SLC25A24, SLC25A26, SLC25A4, SLC25A5, SLC25A6, SLC26A2, SLC2A1, SLC30A9, SLC31A1, SLC44A1, SLC44A2, SLC4A7, SLC7A6, SLC9A1, SMPD1, SOAT1, SORT1, SPTLC1, SPTLC2, SRD5A3, SSR3, STOM, SURF1, SYNE2, SYPL2, TAP1, TENM3, TGFBR3, TGM2, THADA, TIMM17B, TIMMDC1, TM9SF4,	

					TMED9, TMEM119, TMEM168, TMEM192, TMEM65, TMEM68, TMEM97, TMPPE, TMX3, TMX4, TOMM34, TOMM7, TOR1AIP1, TOR1AIP2, TPBG, TPST2, TRAM1, TRAM2, TRAPPC8, TRIM59, TSPO, TUBA8, UBAC2, UBR4, UNC93B1, UQCR10, UQCR11, UST, VAT1, VDAC1, VDAC2, VLDLR, VPS26B, VRK1, WFS1, XYLT2, YKT6, ZDHHC13, ZDHHC21, ZMPSTE24]	
organelle subcompartment	GO_CellularComponent-EBI-UniProt-GOA-ACAP-ARAP_13.05.2021_00h00	0.663628	10.720087	198	[ABHD12, ACSL3, ACSL4, ADAM10, AGPAT2, ALDH3A2, ALG11, ANXA7, APP, ARFGAP1, ARFGEF1, ARFIP1, ARRB1, ASAP2, ATL3, ATP2C1, AUP1, B2M, B3GAT3, B4GALT1, B4GALT5, BET1, BNIP1, BPNT2, C1GALT1, C2CD2L, CAMK2D, CANX, CDIPT, CDK1, CERS6, CHID1, CHORDC1, CHP1, CHPF, CHSY3, CLGN, CLTC, CLTCL1, COG6, COG7, CORO7, CUX1, CYB5R3, CYP51A1, DERL1, DHCR24, DHCR7, DHRS7B, DNM1L, DNMBP, DST, EMC1, EPHX1, EPM2AIP1, ERAP1, ERLIN2, ERP44, ESYT1, EXTL2, FAM91A1, FKBP1A, FLRT2, FUT11, FUT8, GALNT1, GALNT2, GALNT7, GBA2, GBP1, GCC2, GGA2, GOLGA1, GOLGA3, GOLGA7, GOLT1B, GPR108, GPSM1, GRAMD1A, HLA-A, HLA-B, HLA-C, HMOX1, HSP90B1, HSPA5, ILVBL, JAGN1, KDSR, KPNB1, LBR, LLGL1, LPCAT2, LPCAT4, LRRC8E, LSS, LYPLA2, M6PR, MARCHF5, MBOAT2, MBOAT7, MEST, MGAT1, MGST1, MICALL1, MME, MOGS, MYO18A, MYO1B, NCLN, NPLOC4, NSDHL, OSBPL8, PANX1, PCYT2, PI4K2B, PIEZO1, PIGO, PIGT, PIGU,	No change

					PIK3C2A, PIP4K2B, PLA2G4A, PLD1, PLD3, PLOD2, PLPP3, PML, PNPLA6, POMGNT1, POMGNT2, POMK, PRKCI, PRKCSH, PSMD2, PTGES2, PTGIS, PYCARD, QSOX1, RAB10, RAB1A, RAB8A, RDH10, RDH11, REEP2, REEP6, RELCH, RETSAT, RHEB, RPL27, RRAS2, RTN4, SCARB2, SCFD1, SEC11C, SEC14L2, SEC23B, SEC61A1, SEC61B, SELENOT, SEZ6L2, SLC2A1, SLC9A1, SOAT1, SORT1, SPTLC1, SPTLC2, SRD5A3, SRPRA, SSR3, SYNE2, TAP1, TGFBI, THADA, TKT, TMED9, TMEM119, TMEM97, TMX3, TOR1AIP2, TPST2, TRAM1, TRAM2, TRAPPC4, TRAPPC8, TRIM59, UBAC2, UFD1, UNC93B1, UST, VDAC2, VRK1, WDR11, WFS1, XYLT2, YKT6, ZDHHC13, ZDHHC21, ZMPSTE24]	
nuclear outer membrane-endoplasmic reticulum membrane network	GO_CellularComponent-EBI-UniProt-GOA-ACAP-ARAP_13.05.2021_00h00	0.663628	10.9375	133	[ABHD12, ACSL3, ACSL4, AGPAT2, ALDH3A2, ALG11, ANXA7, ATL3, AUP1, B2M, BET1, BNIP1, C2CD2L, CAMK2D, CANX, CDIPT, CDK1, CERS6, CLGN, CYB5R3, CYP51A1, DERL1, DHCR24, DHCR7, DHRS7B, DNM1L, DST, EMC1, EPHX1, EPM2AIP1, ERAP1, ERLIN2, ERP44, ESYT1, EXTL2, FKBP1A, FLRT2, GALNT1, GALNT2, GBA2, GPSM1, GRAMD1A, HLA-A, HLA-B, HLA-C, HMOX1, HSP90B1, HSPA5, ILVBL, JAGN1, KDSR, LBR, LPCAT2, LPCAT4, LRPPRC, LRRC8E, LSS, MARCHF5, MBOAT2, MBOAT7, MEST, MGST1, MOGS, NCLN, NPLOC4, NSDHL, NUP62, NUTF2, OSBPL8, PANX1, PCYT2, PIEZO1, PIGO, PIGT, PIGU, PIP4K2B, PLA2G4A, PLD1, PLD3, PLOD2, PLPP3, PML,	No change

					PNPLA6, POMGNT2, POMK, PRKCSH, PSMD2, PTGIS, RAB10, RDH10, RDH11, REEP2, REEP6, RETSAT, RHEB, RPL27, RTN4, SCARB2, SCFD1, SEC11C, SEC14L2, SEC23B, SEC61A1, SEC61B, SELENOT, SEZ6L2, SLC9A1, SOAT1, SORT1, SPTLC1, SPTLC2, SRD5A3, SRPRA, SSR3, SYNE2, TAP1, THADA, TKT, TMED9, TMEM119, TMEM97, TMX3, TOR1AIP2, TRAM1, TRAM2, TRAPPC8, TRIM59, UBAC2, UFD1, UNC93B1, VDAC2, WFS1, ZMPSTE24]	
integral component of membrane	GO_CellularComponent-EBI-UniProt-GOA-ACAP-ARAP_13.05.2021_00h00	0.663628	5.5754476	327	[ABCB7, ABCC1, ABHD12, ABHD16A, ACSL3, ACSL4, ADAM10, AFDN, AGPAT2, AGRN, ALCAM, ALDH3A2, ALG11, ANKH, ANO6, APLP2, APP, ASAH1, ATL3, ATP1A1, ATP1B3, ATP2B1, ATP2B2, ATP2C1, ATP6V0A1, ATP6V0C, ATP6V1B2, AUP1, B2M, B3GAT3, B4GALT1, B4GALT5, BCAM, BET1, BNIP1, BPNT2, C1GALT1, C2CD2L, CANX, CAPZA2, CASK, CD200, CD276, CD81, CDH11, CDIPT, CERS6, CHPF, CHSY3, CIP2A, CLGN, CLIC1, CLPTM1L, CLU, CNGB1, COL15A1, COLEC12, CORO7, COX20, COX4I1, COX6C, CPD, CSPG4, CUX1, CYP51A1, DAGLB, DCBLD2, DERL1, DHCR24, DHCR7, DHRS7B, DHX34, DHX36, DST, ECE1, EEF1E1, EFNB2, EMC1, ENPP1, EOGT, EPB41L3, EPHA2, EPHA3, EPHB4, EPHX1, ERAP1, ERBB2, ERLIN2, ESYT1, ETFDH, EXD2, EXTL2, FAM171A1, FAM3A, FASN, FAT1, FKBP1A, FLRT2, FTH1, FTO, FUT11, FUT8, GALNT1, GALNT2, GALNT7, GARS1, GBA2, GDAP1, GLMN,	No change

					GLMP, GOLT1B, GPR108, GRAMD1A, GSDMD, GSK3B, GTF3C3, GXYLT1, GXYLT2, HDAC2, HECTD4, HELZ, HHIP, HLA-A, HLA-B, HLA-C, HSPA5, IKBKB, ILVBL, INTS1, ITGA3, ITGAV, ITGB1, ITGB5, ITM2C, ITPRIPL2, ITSN1, JAG1, JAGN1, JAM3, KCNMA1, KCNQ1, KDSR, KIRREL1, LAMP1, LBR, LDHA, LEMD2, LLGL1, LMO7, LNPEP, LPCAT2, LPCAT4, LRCH4, LRP10, LRRC32, LRRC8A, LRRC8E, M6PR, MACF1, MAEA, MAOA, MARCHF5, MBOAT2, MBOAT7, MCAM, MEST, MFF, MFN1, MFSD10, MGAT1, MGST1, MME, MOGS, MTCH1, MYADM, MYOF, NCKAP1, NCLN, NDUFA13, NDUFA4, NECTIN2, NF1, NNT, NPR2, NSDHL, OSBPL8, OXA1L, PACC1, PANX1, PARL, PCDH7, PDZD11, PGAM5, PIEZO1, PIGO, PIGT, PIGU, PKN2, PLD3, PLPP3, PLXNB2, PNPLA6, POMGNT1, POMGNT2, POMK, PPP1R7, PROCR, PSMD2, PTBP2, PTGES2, PTGIS, PTK7, PTPA, PTPRF, PTPRG, PTRH2, QARS1, QSOX1, RDH10, RDH11, REEP2, REEP6, RELCH, RETREG2, ROR1, RRM1, RTN4, RTTN, SCAI, SCARB2, SCFD1, SDHC, SEC11C, SEC14L2, SEC61A1, SEC61B, SELENOT, SEMA3C, SESTD1, SEZ6L2, SFXN2, SFXN3, SLC11A2, SLC12A2, SLC16A3, SLC17A5, SLC1A1, SLC1A4, SLC1A5, SLC25A10, SLC25A12, SLC25A13, SLC25A15, SLC25A19, SLC25A22, SLC25A24, SLC25A26, SLC25A4, SLC25A5, SLC25A6, SLC26A2, SLC2A1, SLC30A9, SLC31A1, SLC44A1, SLC44A2,	

					SLC4A7, SLC7A6, SLC9A1, SMPD1, SOAT1, SORT1, SPTLC1, SPTLC2, SRD5A3, SSR3, STOM, SURF1, SYNE2, SYPL2, TAP1, TENM3, TGFBR3, THADA, TIMM17B, TIMMDC1, TM9SF4, TMED9, TMEM119, TMEM168, TMEM192, TMEM65, TMEM68, TMEM97, TMPPE, TMX3, TMX4, TOMM34, TOMM7, TOR1AIP1, TOR1AIP2, TPBG, TPST2, TRAM1, TRAM2, TRAPPC8, TRIM59, TSPO, TUBA8, UBAC2, UBR4, UNC93B1, UQCR10, UQCR11, UST, VAT1, VDAC1, VDAC2, VLDLR, VPS26B, VRK1, WFS1, XYLT2, YKT6, ZDHHC13, ZDHHC21, ZMPSTE24]	
organelle membrane	GO_CellularComponent-EBI-UniProt-GOA-ACAP-ARAP_13.05.2021_00h00	0.663628	10.639979	404	[ABCB7, ABHD12, ACADM, ACADVL, ACAP2, ACOX1, ACSL3, ACSL4, ADAM10, AGPAT2, AGPS, AHCTF1, ALDH18A1, ALDH3A2, ALG11, ANKFY1, ANO6, ANXA3, ANXA7, AP3M2, AP5B1, APLP2, APP, APPL2, ARFGAP1, ARFGEF1, ARFIP1, ARRB1, ASAP2, ASPSCR1, ATL3, ATP1A1, ATP2B1, ATP2C1, ATP6V0A1, ATP6V0C, ATP6V1A, ATP6V1B2, AUP1, B2M, B3GAT3, B4GALT1, B4GALT5, BET1, BNIP1, BPNT2, BST1, C1GALT1, C2CD2L, CAMK2D, CANX, CASK, CBX3, CD109, CDIPT, CDK1, CEMIP, CENPV, CERS6, CHMP3, CHORDC1, CHP1, CHPF, CHSY3, CLGN, CLIC1, CLTC, CLTCL1, CLU, CNGB1, COG6, COG7, COL6A1, COLEC12, COQ5, CORO1C, CORO7, COX20, COX4I1, COX6C, CTSA, CTSC, CUX1, CYB5R3, CYCS, CYP51A1, DAB2, DAGLB, DERL1, DHCR24, DHCR7, DHRS7B, DIAPH1, DIPK2A, DNM1L,	No change

					DSP, DST, DYNC1LI1, DYNLL1, ECE1, EHD3, EMC1, ENPP1, EPHA3, EPHX1, EPM2AIP1, ERAP1, ERBB2, ERLIN2, ERP44, ESYT1, ETFDH, EXD2, EXTL2, FDXR, FKBP1A, FLRT2, FUT11, FUT8, GALNT1, GALNT2, GALNT7, GAPDH, GARS1, GBA2, GBP1, GCAT, GDAP1, GDAP2, GGA2, GIPC1, GLE1, GLMP, GNB4, GOLGA1, GOLGA3, GOLGA7, GOLT1B, GPD2, GPI, GPR108, GPSM1, GRAMD1A, GTF3C3, HADHA, HGS, HHIP, HIP1, HK1, HK2, HLA-A, HLA-B, HLA-C, HMOX1, HSD17B4, HSP90B1, HSPA5, ILVBL, INPP4A, INTS1, ITGAV, ITGB1, ITM2C, JAGN1, KCNQ1, KDSR, KPNB1, LAMP1, LBR, LEMD2, LLGL1, LMNB1, LNPEP, LPCAT2, LPCAT4, LRPPRC, LRRC8A, LRRC8E, LSS, M6PR, MAN2B2, MAOA, MAP1LC3A, MARCHF5, MATR3, MBOAT2, MBOAT7, MEAK7, MEST, MFF, MFN1, MFSD10, MGAT1, MGST1, MICALL1, MME, MOGS, MRPL10, MRPS17, MRPS21, MRPS31, MTCH1, MYD88, MYO18A, MYO1B, MYO1E, MYO6, MYOF, NCDN, NCK2, NCLN, NDRG1, NDUFA13, NDUFA4, NDUFS3, NNT, NPLOC4, NSDHL, NUP205, NUP35, NUP62, NUTF2, OSBPL11, OSBPL8, OXA1L, PAFAH1B1, PANX1, PARL, PCDH7, PCYT2, PGAM5, PIEZO1, PIGO, PIGT, PIGU, PIP4K2B, PLA2G4A, PLD1, PLD3, PLOD2, PLPP3, PML, PNPLA6, POMGNT1, POMGNT2, POMK, PRKCI, PRKCSH, PSMD2, PTGES2, PTGIS, PTPMT1, PYCARD, QSOX1, QTRT1, QTRT2, RAB10, RAB11B, RAB1A, RAB23,	

					RAB3B, RAB3D, RAB5A, RAB8A, RABGEF1, RALA, RANGAP1, RAP2B, RAP2C, RCC1, RDH10, RDH11, REEP2, REEP6, RETSAT, RHEB, RHOB, RIF1, RPL27, RRAS2, RTN4, SARM1, SCAI, SCARB2, SCFD1, SCRN1, SDHC, SEC11C, SEC14L2, SEC23B, SEC61A1, SEC61B, SELENOT, SEPTIN2, SEPTIN8, SEPTIN9, SEZ6L2, SFXN2, SFXN3, SH3BGRL2, SH3KBP1, SHMT2, SIRT1, SLC11A2, SLC12A2, SLC16A3, SLC17A5, SLC1A1, SLC25A10, SLC25A12, SLC25A13, SLC25A15, SLC25A19, SLC25A22, SLC25A24, SLC25A26, SLC25A4, SLC25A5, SLC25A6, SLC2A1, SLC44A1, SLC44A2, SLC9A1, SNF8, SNX1, SNX18, SNX21, SNX27, SNX5, SOAT1, SORT1, SPARC, SPHK2, SPTLC1, SPTLC2, SRC, SRD5A3, SRPRA, SSR3, STOM, SULT1E1, SURF1, SYNE2, SYNJ1, SYPL2, TAB2, TAP1, TF, THADA, THEM4, TIGAR, TIMM17B, TIMMDC1, TKT, TMED9, TMEM119, TMEM192, TMEM65, TMEM97, TMX3, TMX4, TOM1, TOMM34, TOMM7, TOR1AIP1, TOR1AIP2, TOR1B, TPRG1L, TPST2, TRA2B, TRAM1, TRAM2, TRAPPC4, TRAPPC8, TRIM59, TSPO, TTC1, UBAC2, UBR4, UFD1, UNC93B1, UQCR10, UQCR11, UQCRC2, UST, VAT1, VDAC1, VDAC2, VLDLR, VPS25, VPS33B, VPS36, VPS41, WDR11, WDR41, WDR44, WFS1, XYLT2, YBX1, YEATS4, YKT6, ZDHHC13, ZDHHC21, ZMPSTE24]	

endoplasmic reticulum	GO_CellularComponent-EBI-UniProt-GOA-ACAP-ARAP_13.05.2021_00h00	0.663628	11.031519	231	[ABHD12, ABI1, ACSL3, ACSL4, ADAM10, ADAMTS9, AGPAT2, ALB, ALDH3A2, ALG11, ANK3, ANXA7, APEX1, APLP2, APP, ARHGAP5, ASAH1, ATL3, ATP1A1, ATP2C1, AUP1, B2M, BAIAP2, BET1, BNIP1, C2CD2L, CAMK2D, CANX, CDIPT, CDK1, CEMIP, CERS6, CHORDC1, CHP1, CLGN, CLU, COL11A1, COL12A1, COL15A1, COL18A1, COL4A1, COL5A1, COL5A2, COL6A1, CRABP2, CRTAP, CTSA, CTSC, CYB5R3, CYP51A1, DERL1, DHCR24, DHCR7, DHRS7B, DNM1L, DST, EEF1G, EMC1, EOGT, EPHX1, EPM2AIP1, ERAP1, ERLIN2, ERP44, ESYT1, EXTL2, FKBP1A, FKBP9, FLRT2, FN1, GALNT1, GALNT2, GBA2, GET3, GLUD1, GLUL, GOLT1B, GPSM1, GRAMD1A, GSTM2, HK2, HLA-A, HLA-B, HLA-C, HMOX1, HNRNPR, HSD17B11, HSP90B1, HSPA5, IGFBP7, ILVBL, JAGN1, KCNQ1, KDSR, KPNB1, LAMC1, LBR, LEMD2, LLGL1, LPCAT2, LPCAT4, LRRC8E, LSS, LTBP1, MARCHF5, MBOAT2, MBOAT7, MEST, MGST1, MOGS, MYO1D, NCK2, NCLN, NENF, NPLOC4, NSDHL, NUCB2, OSBPL8, P3H1, P3H3, PANX1, PCYT2, PDCD6IP, PDXDC1, PIEZO1, PIGO, PIGT, PIGU, PIP4K2B, PKM, PLA2G4A, PLD1, PLD3, PLOD2, PLPP3, PML, PNPLA6, POFUT1, POGLUT3, POMGNT2, POMK, PPIB, PPP3CA, PRKCSH, PRXL2B, PSMD2, PSMG1, PTGES2, PTGIS, PYCARD, QSOX1, RAB10, RAB1A, RAP1GDS1, RCN1, RCN2, RCN3, RDH10, RDH11, REEP2, REEP6, RETSAT, RHEB, RPL13, RPL24,	No change

					RPL27, RPL5, RPS21, RPS23, RPS8, RRAS2, RTN4, S100A10, SCARB2, SCFD1, SCP2, SEC11C, SEC14L2, SEC23B, SEC61A1, SEC61B, SELENOT, SET, SEZ6L2, SLC1A1, SLC30A9, SLC9A1, SOAT1, SORT1, SPHK2, SPTLC1, SPTLC2, SQSTM1, SRD5A3, SRPRA, SSR3, STOM, SYNE2, TAP1, TF, TGM2, THADA, THBS1, TKT, TMED9, TMEM119, TMEM192, TMEM97, TMX3, TOR1AIP2, TOR1B, TPBG, TPST2, TRAM1, TRAM2, TRAPPC4, TRAPPC8, TRIM59, TSPO, UBAC2, UBXN2A, UFD1, UNC93B1, VDAC2, VTN, WASHC5, WFS1, YKT6, ZDHHC13, ZDHHC21, ZMPSTE24]	
bounding membrane of organelle	GO_CellularComponent-EBI-UniProt-GOA-ACAP-ARAP_13.05.2021_00h00	0.663628	10.299295	234	[ACAP2, ACOX1, ACSL3, ACSL4, ADAM10, AGPAT2, AGPS, ALDH3A2, ANKFY1, ANO6, ANXA3, ANXA7, AP5B1, APLP2, APP, APPL2, ARFGAP1, ARFGEF1, ARFIP1, ARRB1, ASAP2, ASPSCR1, ATP2B1, ATP2C1, ATP6V0A1, ATP6V0C, ATP6V1A, ATP6V1B2, B2M, B3GAT3, B4GALT1, B4GALT5, BET1, BPNT2, BST1, C1GALT1, CAMK2D, CASK, CD109, CEMIP, CHMP3, CHORDC1, CHP1, CHPF, CHSY3, CLTC, CLTCL1, CLU, CNGB1, COG6, COG7, COL6A1, COLEC12, CORO1C, CORO7, CTSA, CTSC, CUX1, CYB5R3, DAB2, DAGLB, DHCR24, DHCR7, DHRS7B, DIAPH1, DIPK2A, DNM1L, DSP, DYNC1LI1, DYNLL1, ECE1, EHD3, ENPP1, ERBB2, EXD2, FKBP1A, FUT11, FUT8, GALNT1, GALNT2, GALNT7, GBA2, GBP1, GDAP1, GDAP2, GGA2, GLMP, GNB4, GOLGA1, GOLGA3, GOLGA7, GOLT1B,	No change

					GPI, GPR108, GPSM1, HGS, HHIP, HIP1, HK1, HK2, HLA-A, HLA-B, HLA-C, HSD17B4, INPP4A, ITGAV, ITGB1, ITM2C, LAMP1, LLGL1, LNPEP, LPCAT2, LRPPRC, LRRC8A, LRRC8E, M6PR, MAN2B2, MAOA, MAP1LC3A, MARCHF5, MEAK7, MFF, MFN1, MGAT1, MGST1, MICALL1, MME, MTCH1, MYD88, MYO18A, MYO1B, MYO6, NCDN, NDRG1, NUTF2, OSBPL11, PCDH7, PGAM5, PIEZO1, PLA2G4A, PLD1, PLD3, PLOD2, PLPP3, PML, POMGNT1, PRKCI, PRKCSH, PTGES2, PYCARD, QSOX1, QTRT1, QTRT2, RAB10, RAB11B, RAB1A, RAB23, RAB3B, RAB3D, RAB5A, RAB8A, RABGEF1, RAP2B, RAP2C, REEP6, RETSAT, RHEB, RHOB, RPL27, RRAS2, SARM1, SCARB2, SCFD1, SEC14L2, SEC23B, SEC61A1, SEC61B, SEPTIN2, SEPTIN8, SEPTIN9, SLC11A2, SLC17A5, SLC1A1, SLC2A1, SLC44A1, SLC44A2, SNF8, SNX1, SNX18, SNX21, SNX27, SNX5, SORT1, SPARC, SPHK2, SRPRA, STOM, SYNE2, SYPL2, TAB2, TAP1, TF, TIGAR, TMED9, TMEM192, TMEM97, TMX3, TOM1, TOMM34, TOMM7, TPRG1L, TPST2, TRAPPC4, TSPO, TTC1, UBR4, UNC93B1, UST, VAT1, VDAC1, VDAC2, VLDLR, VPS25, VPS33B, VPS36, VPS41, WDR11, WDR41, WDR44, WFS1, XYLT2, YKT6, ZDHHC13, ZDHHC21]	
endoplasmic reticulum subcompartment	GO_CellularComponent-EBI-UniProt-GOA-ACAP-ARAP_13.05.2021_00h00	0.663628	10.934892	131	[ABHD12, ACSL3, ACSL4, AGPAT2, ALDH3A2, ALG11, ANXA7, ATL3, AUP1, B2M, BET1, BNIP1, C2CD2L, CAMK2D, CANX, CDIPT, CDK1, CERS6, CLGN,	No change

					CYB5R3, CYP51A1, DERL1, DHCR24, DHCR7, DHRS7B, DNM1L, DST, EMC1, EPHX1, EPM2AIP1, ERAP1, ERLIN2, ERP44, ESYT1, EXTL2, FKBP1A, FLRT2, GALNT1, GALNT2, GBA2, GPSM1, GRAMD1A, HLA-A, HLA-B, HLA-C, HMOX1, HSP90B1, HSPA5, ILVBL, JAGN1, KDSR, KPNB1, LBR, LPCAT2, LPCAT4, LRRC8E, LSS, MARCHF5, MBOAT2, MBOAT7, MEST, MGST1, MOGS, NCLN, NPLOC4, NSDHL, OSBPL8, PANX1, PCYT2, PIEZO1, PIGO, PIGT, PIGU, PIP4K2B, PLA2G4A, PLD1, PLD3, PLOD2, PLPP3, PML, PNPLA6, POMGNT2, POMK, PRKCSH, PSMD2, PTGIS, RAB10, RDH10, RDH11, REEP2, REEP6, RETSAT, RHEB, RPL27, RTN4, SCARB2, SCFD1, SEC11C, SEC14L2, SEC23B, SEC61A1, SEC61B, SELENOT, SEZ6L2, SLC9A1, SOAT1, SORT1, SPTLC1, SPTLC2, SRD5A3, SRPRA, SSR3, SYNE2, TAP1, THADA, TKT, TMED9, TMEM119, TMEM97, TMX3, TOR1AIP2, TRAM1, TRAM2, TRAPPC8, TRIM59, UBAC2, UFD1, UNC93B1, VDAC2, WFS1, ZMPSTE24]	
carbohydrate metabolic process	GO_BiologicalProcess-EBI-UniProt-GOA-ACAP-ARAP_13.05.2021_00h00	1.35E-12	14.766082	101	[ACADM, AKR1B1, ALDH2, ALDOA, APP, ARPP19, B3GAT3, B4GALT1, B4GALT5, BPNT2, CHID1, CLTC, CYB5R3, DCXR, DHX34, EIF6, ENO2, ENPP1, EPM2AIP1, FAM3A, FUT8, G6PD, GALE, GALM, GALNT7, GAPDH, GBA2, GBE1, GDPGP1, GLA, GLO1, GNE, GOLT1B, GOT1, GPD1L, GPD2, GPI, GSK3B, GYS1, HDAC4, HECTD4, HEXB, HK1, HK2, IDH2, IMPA1, ITPK1, KCNQ1, LANCL2, LDHA, MAEA, MAN2B2, ME1,	No change

					MOGS, NISCH, NUP205, NUP35, NUP62, PARG, PC, PCK2, PDK1, PFKM, PGGHG, PGM2L1, PGM3, PGP, PHGDH, PHKB, PIK3CA, PKLR, PKM, PLA2G4A, PMM2, POFUT1, POMK, PPIP5K2, PPP1CA, PRKAA1, PTRH2, PYGB, SBDS, SCARB2, SESN2, SIAE, SIRT1, SLC25A10, SLC25A12, SLC25A13, SLC2A1, SRC, SYNJ1, TALDO1, TIGAR, TKT, TPI1, UGP2, USP7, WDR5, XYLB, ZMPSTE24]	
generation of precursor metabolites and energy	GO_BiologicalProcess-EBI-UniProt-GOA-ACAP-ARAP_13.05.2021_00h00	1.35E-12	14.188034	83	[ACADM, ACADVL, ACO1, ACOX1, ACSS2, ADSL, AK4, AKR1B1, ALDH2, ALDOA, APP, BDH2, CDK1, COX4I1, COX6C, CROT, CYCS, DGUOK, DLST, EIF6, ENO2, ENPP1, EPM2AIP1, ETFDH, FAHD1, FAM3A, FDXR, G6PD, GAPDH, GBE1, GFPT2, GPD2, GPI, GSK3B, GYS1, HDAC4, HK1, HK2, IDH2, IREB2, LDHA, ME1, MSH2, NDUFA13, NDUFA4, NDUFS3, NNT, NOP53, NUP205, NUP35, NUP62, OXA1L, PFKM, PGM2L1, PHGDH, PHKB, PIK3CA, PKLR, PKM, PPP1CA, PRKAA1, PTRH2, PYGB, QDPR, SDHC, SHMT2, SLC25A12, SLC25A13, SLC25A22, SLC25A4, SOAT1, SPHK2, SURF1, TALDO1, TIGAR, TKT, TPI1, UGP2, UQCR10, UQCR11, UQCRC2, VDAC2, XYLB]	No change
monosaccharide metabolic process	GO_BiologicalProcess-EBI-UniProt-GOA-ACAP-ARAP_13.05.2021_00h00	1.35E-12	18.910257	59	[ACADM, AKR1B1, ALDOA, ARPP19, B4GALT1, CYB5R3, DCXR, ENO2, ENPP1, EPM2AIP1, FAM3A, FUT8, G6PD, GALE, GALM, GAPDH, GDPGP1, GOLT1B, GOT1, GPI, GSK3B, HECTD4, HK1, HK2, KCNQ1, MAEA, MAN2B2, NISCH, PC, PCK2, PDK1, PFKM, PGM2L1, PGP, PHGDH, PIK3CA, PKLR, PKM, PMM2, POFUT1, PPP1CA, PRKAA1, PTRH2,	No change

					SBDS, SESN2, SIRT1, SLC25A10, SLC25A12, SLC25A13, SLC2A1, SRC, TALDO1, TIGAR, TKT, TPI1, USP7, WDR5, XYLB, ZMPSTE24]	
cellular carbohydrate metabolic process	GO_BiologicalProcess-EBI-UniProt-GOA-ACAP-ARAP_13.05.2021_00h00	1.35E-12	15.454545	51	[ACADM, AKR1B1, ARPP19, B4GALT1, B4GALT5, BPNT2, DHX34, ENPP1, EPM2AIP1, FAM3A, GBE1, GNE, GOLT1B, GOT1, GPD2, GSK3B, GYS1, HDAC4, HK1, HK2, IDH2, IMPA1, ITPK1, MAEA, PCK2, PDK1, PFKM, PGM2L1, PGP, PHGDH, PHKB, PLA2G4A, POMK, PPIP5K2, PPP1CA, PTRH2, PYGB, SCARB2, SESN2, SIRT1, SLC25A12, SLC2A1, SRC, SYNJ1, TIGAR, TPI1, UGP2, USP7, WDR5, XYLB, ZMPSTE24]	No change
carbohydrate biosynthetic process	GO_BiologicalProcess-EBI-UniProt-GOA-ACAP-ARAP_13.05.2021_00h00	1.35E-12	16.929134	43	[ACADM, AKR1B1, ALDOA, ARPP19, B4GALT1, B4GALT5, CLTC, DHX34, ENO2, ENPP1, EPM2AIP1, FAM3A, FUT8, G6PD, GAPDH, GBE1, GOLT1B, GOT1, GPI, GSK3B, GYS1, IMPA1, MAEA, PC, PCK2, PGM2L1, PGP, PHGDH, PPP1CA, PTRH2, SBDS, SESN2, SIRT1, SLC25A10, SLC25A12, SLC25A13, SLC2A1, TALDO1, TKT, TPI1, UGP2, USP7, WDR5]	No change
hexose metabolic process	GO_BiologicalProcess-EBI-UniProt-GOA-ACAP-ARAP_13.05.2021_00h00	1.35E-12	18.59649	53	[ACADM, AKR1B1, ALDOA, ARPP19, B4GALT1, DCXR, ENO2, ENPP1, EPM2AIP1, FAM3A, FUT8, G6PD, GALE, GALM, GAPDH, GDPGP1, GOLT1B, GOT1, GPI, GSK3B, HECTD4, HK1, HK2, KCNQ1, MAEA, MAN2B2, NISCH, PC, PCK2, PDK1, PFKM, PGM2L1, PGP, PIK3CA, PKLR, PKM, PMM2, POFUT1, PPP1CA, PRKAA1, PTRH2, SBDS, SESN2, SIRT1, SLC25A10, SLC25A12, SLC25A13, SRC, TIGAR, TPI1, USP7, WDR5, ZMPSTE24]	No change
monosaccharide biosynthetic process	GO_BiologicalProcess-EBI-UniProt-GOA-ACAP-	1.35E-12	22.689075	27	[ACADM, AKR1B1, ALDOA, ARPP19, ENO2, FAM3A, G6PD, GAPDH,	No change

	ARAP_13.05.2021_00h00				GOLT1B, GOT1, GPI, MAEA, PC, PCK2, PGP, PHGDH, SBDS, SESN2, SIRT1, SLC25A10, SLC25A12, SLC25A13, TALDO1, TKT, TPI1, USP7, WDR5]	
monosaccharide catabolic process	GO_BiologicalProcess-EBI-UniProt-GOA-ACAP-ARAP_13.05.2021_00h00	1.35E-12	23.943663	17	[ALDOA, ENO2, FUT8, GALE, GALM, GAPDH, GPI, HK1, HK2, PFKM, PGM2L1, PKLR, PKM, SLC25A12, TIGAR, TPI1, XYLB]	Downregulation
glucose metabolic process	GO_BiologicalProcess-EBI-UniProt-GOA-ACAP-ARAP_13.05.2021_00h00	1.35E-12	18.852459	46	[ACADM, ALDOA, ARPP19, DCXR, ENO2, ENPP1, EPM2AIP1, FAM3A, G6PD, GALM, GAPDH, GDPGP1, GOLT1B, GOT1, GPI, GSK3B, HECTD4, HK1, HK2, KCNQ1, MAEA, NISCH, PC, PCK2, PDK1, PFKM, PGM2L1, PGP, PIK3CA, PKLR, PKM, PPP1CA, PRKAA1, PTRH2, SBDS, SESN2, SIRT1, SLC25A10, SLC25A12, SLC25A13, SRC, TIGAR, TPI1, USP7, WDR5, ZMPSTE24]	No change
hexose biosynthetic process	GO_BiologicalProcess-EBI-UniProt-GOA-ACAP-ARAP_13.05.2021_00h00	1.35E-12	20.90909	23	[ACADM, AKR1B1, ALDOA, ARPP19, ENO2, FAM3A, GAPDH, GOLT1B, GOT1, GPI, MAEA, PC, PCK2, PGP, SBDS, SESN2, SIRT1, SLC25A10, SLC25A12, SLC25A13, TPI1, USP7, WDR5]	Upregulation
cellular response to stress	GO_BiologicalProcess-EBI-UniProt-GOA-ACAP-ARAP_13.05.2021_00h00	1.27E-18	11.89387	260	[ACADVL, ACTR2, AK4, AKR1B1, ALB, ANKZF1, APEX1, APP, AQR, ARFGAP1, ARMT1, ASNS, ATG7, ATP6V0C, ATP6V1A, ATP6V1B2, AUP1, B2M, BAG1, BAZ1B, CAMK2D, CANX, CASP3, CBX1, CBX3, CBX5, CBX8, CCN2, CCNH, CDC5L, CDK1, CDK2, CDK5RAP3, CETN2, CHAF1B, CHD1L, CHD2, CHORDC1, CLGN, CLU, CNOT1, CNOT10, CTNNB1, CUL1, CUL4B, CXCL12, CYCS, DAPK1, DDX1, DERL1, DHFR, DHX36, DUSP3, DVL2, EEF1E1, EIF2AK2, EIF2S1, EPHA2, ERCC5, ERLIN2, ERP44, EXD2, EXTL2, EZR,	No change

					FANCI, FASN, FEN1, FMR1, FSCN1, FTO, FUT8, G6PD, GLUL, GSK3B, GSTM2, GSTM3, GSTP1, GTF2I, HDAC2, HERC2, HK2, HMCES, HMOX1, HSP90AA1, HSP90B1, HSPA14, HSPA5, IGF2BP1, IKBKB, IKBKG, IMPACT, JAM3, KAT7, KDM1A, KDM4B, LAMC1, LIG1, LIG3, LRRC8E, MACROD1, MAGI1, MAP1B, MAP1LC3A, MAP2K3, MAP3K20, MAP3K4, MAP4K4, MCM2, MCM3, MCM4, MCM5, MCM6, MCM7, MDC1, MEAK7, MGMT, MGST1, MLH1, MMAB, MORF4L1, MSH2, MSH6, MTA1, MTR, MTREX, MYD88, MYO6, NCK2, NCOA7, NDRG1, NEDD4, NEK1, NIBAN1, NOP53, NPLOC4, NPM1, NQO1, NSMCE3, NUDT15, NUDT16L1, NUP205, NUP35, NUP62, OXSR1, PAFAH1B1, PARG, PARP1, PARP4, PAWR, PDK1, PDS5B, PIK3CA, PML, POLD1, POLD2, POLE2, POLR2A, POLR2B, POLR2H, POLR2I, PPIE, PPP1CA, PPP5C, PRDX1, PRDX6, PRKAA1, PRPF19, PSMA3, PSMB10, PSMB2, PSMC2, PSMC3, PSMC6, PSMD1, PSMD12, PSMD14, PSMD2, PSMD6, PSMD7, PSMD8, PSME1, PSME2, PSME4, PTGIS, PTPRF, PXN, PYCARD, PYCR1, QARS1, RAD21, RAD9A, RBM4, RCN3, RECQL, RFC2, RFC3, RFC5, RHEB, RHOB, RIF1, RIPK2, RNF2, RPA1, RPA2, SAMHD1, SEC61B, SESN2, SHPRH, SIRT1, SLC1A1, SLC25A24, SLC2A1, SLC30A9, SLC9A1, SMARCAD1, SMC1A, SMC3, SMCHD1, SNW1, SOD1, SOD2, SQSTM1, SRC, SRPRA, SRXN1, SSRP1, STK24,	

					SUPT16H, TAB2, TF, TGFBR3, THBS1, TIGAR, TIPRL, TNIK, TOMM7, TPP1, TRIM25, TRIM28, TRIP12, TRIP13, TSPO, TTC5, TXN, UBAC2, UBE2L6, UFD1, USP28, USP7, VPS41, WDR70, WFS1, WIPI2, WRNIP1, XIAP, YAP1, YBX1, YOD1, ZFP36L2, ZMPSTE24]	
cellular response to DNA damage stimulus	GO_BiologicalProcess-EBI-UniProt-GOA-ACAP-ARAP_13.05.2021_00h00	1.27E-18	13.819369	127	[ACTR2, APEX1, AQR, ARMT1, BAZ1B, CASP3, CBX1, CBX3, CBX5, CBX8, CCNH, CDC5L, CDK1, CDK2, CDK5RAP3, CETN2, CHAF1B, CHD1L, CHD2, CLU, CNOT1, CNOT10, CUL4B, CXCL12, DDX1, EEF1E1, EPHA2, ERCC5, EXD2, FANCI, FEN1, FMR1, FSCN1, FTO, GTF2I, HERC2, HMCES, HMOX1, IKBKG, KAT7, KDM1A, KDM4B, LIG1, LIG3, MACROD1, MAGI1, MAP3K20, MCM2, MCM3, MCM4, MCM5, MCM6, MCM7, MDC1, MGMT, MLH1, MMAB, MORF4L1, MSH2, MSH6, MTA1, MTREX, MYO6, NDRG1, NEDD4, NEK1, NOP53, NPLOC4, NPM1, NSMCE3, NUDT16L1, PARG, PARP1, PARP4, PDS5B, PML, POLD1, POLD2, POLE2, POLR2A, POLR2B, POLR2H, POLR2I, PPIE, PPP5C, PRPF19, PSMD14, PSME4, PYCARD, RAD21, RAD9A, RECQL, RFC2, RFC3, RFC5, RIF1, RNF2, RPA1, RPA2, SAMHD1, SESN2, SHPRH, SIRT1, SLC30A9, SMARCAD1, SMC1A, SMC3, SMCHD1, SNW1, SSRP1, SUPT16H, TIGAR, TIPRL, TRIM25, TRIM28, TRIP12, TRIP13, TTC5, UBE2L6, UFD1, USP28, USP7, WDR70, WRNIP1, XIAP, YAP1, ZMPSTE24]	Upregulation
chromosomal region	GO_CellularComponent-EBI-UniProt-GOA-	1.27E-18	19.30295	72	[AHCTF1, APEX1, BAZ1B, BOD1, CBX1, CBX3, CBX5, CDC73,	Upregulation

	ACAP-ARAP_13.05.2021_00h00				CDK1, CENPV, CFDP1, CHAMP1, CNGB1, DHX36, DNMT1, DYNC1LI1, DYNLL1, ERCC6L, FEN1, FMR1, HAT1, KAT7, KAT8, KDM1A, KDM4B, KNTC1, LRWD1, MACROH2A1, MAD1L1, MAD2L1, MCM2, MCM3, MCM4, MCM5, MCM6, MCM7, MMAB, MSH2, NCAPD2, NCAPG, NSMCE3, NUF2, ORC4, ORC5, PAFAH1B1, PARP1, PDS5B, PML, POLD1, POLR2B, PPP1CA, PPP2R5A, RAD21, RANGAP1, RIF1, RPA1, RPA2, SEPTIN2, SEPTIN7, SLC25A15, SMC1A, SMC3, SMC4, SMCHD1, SSB, STAG2, STON1, SUGT1, THOC2, UHRF2, WRNIP1, ZNF207]	
chromosome, centromeric region	GO_CellularComponent-EBI-UniProt-GOA-ACAP-ARAP_13.05.2021_00h00	1.27E-18	18.660288	39	[AHCTF1, BAZ1B, BOD1, CBX1, CBX3, CBX5, CENPV, CFDP1, CHAMP1, DNMT1, DYNC1LI1, DYNLL1, ERCC6L, FMR1, KAT7, KAT8, KDM4B, KNTC1, LRWD1, MACROH2A1, MAD1L1, MAD2L1, NCAPD2, NCAPG, NUF2, PAFAH1B1, PDS5B, PPP2R5A, RAD21, RANGAP1, SEPTIN2, SEPTIN7, SMC1A, SMC3, SMC4, STAG2, SUGT1, UHRF2, ZNF207]	Upregulation
chromosome, telomeric region	GO_CellularComponent-EBI-UniProt-GOA-ACAP-ARAP_13.05.2021_00h00	1.27E-18	21.468927	38	[APEX1, CBX1, CBX3, CBX5, CDC73, CDK1, CNGB1, DHX36, FEN1, HAT1, KDM1A, LRWD1, MACROH2A1, MCM2, MCM3, MCM4, MCM5, MCM6, MCM7, MMAB, MSH2, NSMCE3, ORC4, ORC5, PARP1, PML, POLD1, POLR2B, PPP1CA, RIF1, RPA1, RPA2, SLC25A15, SMCHD1, SSB, STON1, THOC2, WRNIP1]	Upregulation
DNA metabolic process	GO_BiologicalProcess-EBI-UniProt-GOA-ACAP-	1.27E-18	13.826679	142	[ACTR2, ANXA3, APEX1, AQR, BAZ1B, CASP3, CBX8, CCN2, CCNH, CCT2, CCT3, CCT6A, CDC34,	Upregulation

	ARAP_13.05.2021_00h00				CDC5L, CDK1, CDK2, CECR2, CETN2, CHAF1B, CHD1L, CNGB1, CTNNB1, CUL4B, DDX1, DHX36, DICER1, DNMT1, ERCC5, EXD2, FANCI, FEN1, FGF2, FSCN1, FTO, GATAD2A, GNL3L, GTF2I, H1-10, H1-5, HERC2, HMCES, HNRNPC, HSP90AA1, KAT7, KDM4B, KPNB1, LIG1, LIG3, LRWD1, MAGI1, MAP3K4, MBD3, MCM2, MCM3, MCM4, MCM5, MCM6, MCM7, MDC1, MGMT, MLH1, MMAB, MORF4L1, MPHOSPH8, MSH2, MSH6, MTA1, MTRR, MYO18A, N6AMT1, NIBAN2, NOP53, NPLOC4, NPM1, NSMCE3, NUDT15, NUDT16L1, ORC4, ORC5, PARG, PARP1, PARP4, PDS5B, PIK3CA, PLD3, PML, POLD1, POLD2, POLE2, POLR2A, POLR2B, POLR2H, POLR2I, PPIE, PPP5C, PRIM2, PRMT7, PRPF19, PSMD14, PSME4, RAD21, RAD9A, RECQL, RFC2, RFC3, RFC5, RIF1, RNF2, RPA1, RPA2, SAMHD1, SESN2, SHPRH, SIRT1, SLC25A15, SLC30A9, SMARCAD1, SMARCB1, SMC1A, SMC3, SMCHD1, SRC, SSRP1, STON1, SUPT16H, TCP1, TIGAR, TRIM25, TRIM28, TRIP12, TRIP13, TTC5, UBE2L6, UBR2, UFD1, UHRF2, USP28, USP7, USP9X, WDR70, WRNIP1, ZMPSTE24]	
chromosome organization	GO_BiologicalProcess-EBI-UniProt-GOA-ACAP-ARAP_13.05.2021_00h00	1.27E-18	12.237237	163	[AASS, ACTB, APEX1, ARID1B, ARRB1, ASH2L, BANF1, BAZ1B, BOD1, BRD3, CAMK2D, CBX2, CBX3, CBX8, CCT2, CCT3, CCT6A, CDC73, CDK1, CDK2, CECR2, CENPV, CETN2, CHAF1B, CHAMP1, CHD1L, CHD2, CHD4, CHD7, CHMP3, CHTOP, CNGB1, CTBP1, CTNNB1, CUL4B,	Upregulation

					DDX1, DHX36, DNMT1, DYNC1LI1, EED, EML3, EML4, ENY2, ERCC6L, FEN1, FMR1, GLYR1, GNL3L, GRWD1, GTF2F2, GTF3C4, H1-10, H1-5, HAT1, HDAC2, HDAC4, HIRIP3, HNRNPC, HSP90AA1, KAT7, KAT8, KDM1A, KDM3B, KDM4B, KNTC1, KPNB1, LRWD1, MACROH2A1, MAD1L1, MAD2L1, MAGI1, MAP3K4, MBD3, MCM2, MCM3, MCM4, MCM5, MCM6, MCM7, MCMBP, MLH1, MMAB, MORF4L1, MPHOSPH8, MSH2, MSH6, MTA1, N6AMT1, NCAPD2, NCAPG, NCAPH, NPM1, NUF2, NUP62, PARG, PARP1, PDCD6IP, PDS5B, PELO, PML, POGZ, POLD1, POLD2, PPP5C, PRIM2, PRKAA1, PRMT7, PSME4, RAD21, RBBP4, RECQL, RFC2, RFC3, RFC5, RIF1, RNF2, RNF40, RPA1, RPA2, RYBP, SET, SHPRH, SIRT1, SMAD4, SMARCA1, SMARCAD1, SMARCB1, SMARCC1, SMARCD3, SMARCE1, SMC1A, SMC2, SMC3, SMC4, SMCHD1, SMYD3, SNW1, SPHK2, SRC, SSRP1, STAG2, STON1, SUPT16H, TAF6L, TCP1, TDRD3, TRIM28, TRIP12, TRIP13, TSPYL1, TTLL12, UBR2, USP22, USP7, UTP3, VRK1, WDR5, WDR70, WRAP73, WRNIP1, YEATS4, ZMPSTE24, ZNF207]	
regulation of DNA metabolic process	GO_BiologicalProcess-EBI-UniProt-GOA-ACAP-ARAP_13.05.2021_00h00	1.27E-18	13.647643	55	[ACTR2, ANXA3, CBX8, CCT2, CCT3, CCT6A, CTNNB1, DHX36, FGF2, GNL3L, H1-10, H1-5, HMCES, HNRNPC, HSP90AA1, KAT7, MAP3K4, MBD3, MGMT, MLH1, MMAB, MPHOSPH8, MSH2, MSH6, NIBAN2, NPM1, NUDT16L1, PARG,	Upregulation

					PARP1, PARP4, PIK3CA, PML, POLR2A, POLR2B, POLR2H, POLR2I, RFC2, RFC3, RFC5, RIF1, RPA2, SIRT1, SMARCAD1, SMCHD1, SRC, STON1, TCP1, TIGAR, TRIM28, TRIP12, USP7, USP9X, WDR70, WRNIP1, ZMPSTE24]	
DNA repair	GO_BiologicalProcess-EBI-UniProt-GOA-ACAP-ARAP_13.05.2021_00h00	1.27E-18	15.875614	97	[ACTR2, APEX1, AQR, BAZ1B, CBX8, CCNH, CDC5L, CDK1, CDK2, CETN2, CHAF1B, CHD1L, CUL4B, DDX1, ERCC5, EXD2, FANCI, FEN1, FSCN1, FTO, GTF2I, HERC2, HMCES, KAT7, KDM4B, LIG1, LIG3, MAGI1, MCM2, MCM3, MCM4, MCM5, MCM6, MCM7, MDC1, MGMT, MLH1, MMAB, MORF4L1, MSH2, MSH6, MTA1, NOP53, NPLOC4, NPM1, NSMCE3, NUDT16L1, PARG, PARP1, PARP4, PDS5B, PML, POLD1, POLD2, POLE2, POLR2A, POLR2B, POLR2H, POLR2I, PPIE, PPP5C, PRPF19, PSMD14, PSME4, RAD21, RAD9A, RECQL, RFC2, RFC3, RFC5, RIF1, RNF2, RPA1, RPA2, SAMHD1, SHPRH, SIRT1, SLC30A9, SMARCAD1, SMC1A, SMC3, SMCHD1, SSRP1, SUPT16H, TIGAR, TRIM25, TRIM28, TRIP12, TRIP13, TTC5, UBE2L6, UFD1, USP28, USP7, WDR70, WRNIP1, ZMPSTE24]	Upregulation
DNA replication initiation	GO_BiologicalProcess-EBI-UniProt-GOA-ACAP-ARAP_13.05.2021_00h00	1.27E-18	33.333332	18	[CDC34, CDK2, KAT7, LRWD1, MCM2, MCM3, MCM4, MCM5, MCM6, MCM7, ORC4, ORC5, POLE2, PRIM2, RPA1, RPA2, SLC25A15, WRNIP1]	Upregulation
nucleotide-excision repair	GO_BiologicalProcess-EBI-UniProt-GOA-ACAP-ARAP_13.05.2021_00h00	1.27E-18	22.314049	27	[AQR, CCNH, CETN2, CHD1L, CUL4B, ERCC5, FSCN1, KAT7, LIG1, LIG3, PARP1, POLD1, POLD2, POLR2A, POLR2B, POLR2H, POLR2I, PPIE, PRPF19, RFC2, RFC3, RFC5, RPA1,	Upregulation

					RPA2, SIRT1, SLC30A9, USP7]	
anion binding	GO_MolecularFunction-EBI-UniProt-GOA-ACAP-ARAP_13.05.2021_00h00	6.96E-39	13.488035	372	[AARS1, ABAT, ABCB7, ABCC1, ABCE1, ACACA, ACAD8, ACADM, ACADSB, ACADVL, ACOX1, ACSF2, ACSL3, ACSL4, ACSS2, ACTB, ACTG1, ACTR2, ACTR3, ADK, AGPS, AGRN, AK1, AK3, AK4, AK6, ALB, ALDH18A1, APAF1, APRT, AQR, ARAF, ARFGEF1, ARHGAP5, ARHGEF11, ARHGEF12, ASNS, ATL3, ATP1A1, ATP2B1, ATP2B2, ATP2C1, ATP6V1A, ATP6V1B2, BAZ1B, BCR, BMP1, BMS1, CAMK2D, CASK, CCT2, CCT3, CCT6A, CDC34, CDC42BPA, CDK1, CDK16, CDK17, CDK2, CHD1L, CHD2, CHD4, CHD7, CHMP3, CHORDC1, CKB, CLU, CMPK1, CNGB1, COLEC12, CRABP2, CRYZ, CTPS1, CTSC, CYB5R3, DAPK1, DARS1, DCAKD, DDX1, DDX42, DDX47, DENND2A, DGUOK, DHCR24, DHFR, DHX34, DHX36, DICER1, DNM1L, DNMBP, DOCK6, DRG1, DYNC1LI1, EARS2, EFL1, EHD3, EIF2AK2, EIF2B1, EIF2S1, ENPP1, EPHA2, EPHA3, EPHB4, EPRS1, ERBB2, ERCC6L, ETFDH, FAM3A, FARP1, FERMT2, FGD4, FLII, FN3K, GARS1, GATB, GBP1, GCAT, GCDH, GDPGP1, GET3, GLE1, GLUD1, GLUL, GMPS, GNE, GNL3L, GOLT1B, GOT1, GPHN, GPI, GPN1, GPN3, GPSM1, GSDMD, GSK3B, GSTM2, GSTM3, GSTP1, GTF2F2, HACL1, HADH, HADHA, HARS1, HELZ, HERC2, HIP1, HK1, HK2, HSP90AA1, HSP90B1, HSPA14, HSPA4, HSPA5, HSPE1, IARS2, IKBKB, ILVBL, IRGQ, ITM2C, ITPK1, ITSN1,	No change

					KCNQ1, KDM1A, KIF15, LANCL2, LBR, LIG1, LIG3, LRRK1, MAGI1, MAP2K3, MAP3K20, MAP3K4, MAP4K4, MAT2A, MCM2, MCM3, MCM4, MCM5, MCM6, MCM7, ME1, MFN1, MGST1, MICAL3, MICALL1, MLH1, MMAB, MME, MOV10, MSH2, MSH6, MTCH1, MTHFD2, MTREX, MTRR, MVD, MVK, MYH10, MYH9, MYO18A, MYO1B, MYO1D, MYO1E, MYO6, NCAPG, NDRG1, NDUFA13, NEK1, NPR2, NUBP2, NUBPL, NUCB2, NUDT16, NUDT19, OAT, OGFOD1, OPLAH, ORC4, ORC5, OSBPL2, OXSR1, P3H1, P3H3, PAICS, PANK4, PAPSS2, PBXIP1, PC, PCK2, PDE5A, PDK1, PDXDC1, PEBP1, PFAS, PFKM, PI4K2B, PIK3C2A, PIK3CA, PIP4K2B, PKLR, PKM, PKN2, PLA2G4A, PLOD2, PLPBP, PNP, POMK, PPIP5K2, PRKAA1, PRKCI, PRKG1, PSAT1, PSMC2, PSMC3, PSMC6, PTGES2, PTK7, PTPA, PXK, PYGB, QARS1, QDPR, QSOX1, RAB10, RAB11B, RAB1A, RAB23, RAB3B, RAB3D, RAB5A, RAB8A, RABGEF1, RABL6, RAC3, RALA, RANBP1, RAP2B, RAP2C, RARS2, RCC1, RECQL, RFC2, RFC5, RHEB, RHOB, RHOC, RIC8A, RIC8B, RIOK3, RIPK2, RNGTT, ROR1, RRAS2, RRM1, SAMHD1, SARS1, SBDS, SCP2, SEC14L2, SEPTIN10, SEPTIN11, SEPTIN2, SEPTIN7, SEPTIN8, SEPTIN9, SESN2, SESTD1, SH3BP4, SHMT2, SHPRH, SIRT1, SLC1A1, SLC25A15, SLC9A1, SMAD4, SMARCA1, SMARCAD1, SMC1A, SMC2, SMC3, SMC4, SMCHD1, SNRNP200, SNX18, SNX21, SNX5,	

					SOAT1, SPHK2, SPTLC1, SPTLC2, SRC, SRPRA, SRR, SRXN1, STARD5, STK24, SVIL, TAP1, TBC1D10A, TBK1, TCP1, TGM2, TKT, TNIK, TOR1B, TPP1, TRIO, TRIP13, TTLL12, TUBA4A, TUBA8, TUBB, TUBB3, TWF2, UBA2, UBA3, UBE2L6, UBE2Q1, UBR2, VARS1, VDAC2, VRK1, VRK3, VWA8, WDR41, WIPI2, WRNIP1, XYLB, YARS1, YES1, ZC3HAV1]	
nucleotide binding	GO_MolecularFunction-EBI-UniProt-GOA-ACAP-ARAP_13.05.2021_00h00	6.96E-39	13.863364	347	[AARS1, ABCB7, ABCC1, ABCE1, ACACA, ACAD8, ACADM, ACADSB, ACADVL, ACOT7, ACOX1, ACSF2, ACSL3, ACSL4, ACSS2, ACTB, ACTG1, ACTR2, ACTR3, ADK, AGPS, AK1, AK3, AK4, AK6, ALDH18A1, ALDH2, APAF1, APRT, AQR, ARAF, ARFGEF1, ARHGAP5, ARHGEF11, ARHGEF12, ASNS, ATL3, ATP1A1, ATP2B1, ATP2B2, ATP2C1, ATP6V1A, ATP6V1B2, BAG1, BAZ1B, BCR, BDH2, BMP1, BMS1, CAMK2D, CASK, CCT2, CCT3, CCT6A, CDC34, CDC42BPA, CDK1, CDK16, CDK17, CDK2, CHD1L, CHD2, CHD4, CHD7, CHORDC1, CHTOP, CKB, CMPK1, CNGB1, COLEC12, CRYZ, CRYZL1, CTBP1, CTPS1, CYB5R3, DAPK1, DARS1, DCAKD, DDX1, DDX42, DDX47, DENND2A, DGUOK, DHCR24, DHCR7, DHFR, DHX34, DHX36, DICER1, DNM1L, DNMBP, DNMT1, DOCK6, DRG1, DYNC1LI1, EARS2, ECI2, EFL1, EHD3, EIF2AK2, EIF2B1, EIF2S1, ENPP1, EPHA2, EPHA3, EPHB4, EPRS1, ERBB2, ERCC6L, ETFDH, FAM114A2, FAM3A, FARP1, FGD4, FN3K, G6PD, GAPDH, GARS1,	No change

					GATB, GBP1, GCDH, GDPGP1, GET3, GLUD1, GLUL, GLYR1, GMPS, GNE, GNL3L, GPD1L, GPHN, GPI, GPN1, GPN3, GPSM1, GSK3B, GTF2F2, HACL1, HADH, HADHA, HARS1, HELZ, HERC2, HINT1, HK1, HK2, HSP90AA1, HSP90B1, HSPA14, HSPA4, HSPA5, HSPE1, IARS2, IDH2, IKBKB, ILVBL, IRGQ, ITM2C, ITPK1, ITSN1, KDM1A, KIF15, LANCL2, LBR, LDHA, LIG1, LIG3, LRRK1, LRWD1, MAGI1, MAP2K3, MAP3K20, MAP3K4, MAP4K4, MAT2A, MBD3, MCM2, MCM3, MCM4, MCM5, MCM6, MCM7, ME1, MFN1, MICAL3, MLH1, MMAB, MOCS2, MOV10, MSH2, MSH6, MTREX, MTRR, MVD, MVK, MYH10, MYH9, MYO18A, MYO1B, MYO1D, MYO1E, MYO6, NDRG1, NDUFA13, NEK1, NNT, NPR2, NT5C, NT5C3B, NUBP2, NUBPL, NUCB2, NUDT16, NUDT19, OPLAH, ORC4, ORC5, OXSR1, PAICS, PANK4, PAPSS2, PARP1, PBXIP1, PC, PCK2, PDE5A, PDK1, PEBP1, PFAS, PFKM, PHGDH, PI4K2B, PIK3C2A, PIK3CA, PIP4K2B, PKLR, PKM, PKN2, POLD1, POMK, PPIP5K2, PRKAA1, PRKCI, PRKG1, PSMC2, PSMC3, PSMC6, PTGES2, PTK7, PTPA, PXK, QARS1, QDPR, QSOX1, RAB10, RAB11B, RAB1A, RAB23, RAB3B, RAB3D, RAB5A, RAB8A, RABGEF1, RABL6, RAC3, RALA, RANBP1, RAP2B, RAP2C, RARS2, RCC1, RECQL, RFC2, RFC5, RHEB, RHOB, RHOC, RIC8A, RIC8B, RIOK3, RIPK2, RNGTT, ROR1, RRAS2, RRM1, SAMHD1, SARS1, SCP2, SEC14L2,	

					SEPTIN10, SEPTIN11, SEPTIN2, SEPTIN7, SEPTIN8, SEPTIN9, SESN2, SH3BP4, SHPRH, SIRT1, SLC25A15, SMARCA1, SMARCAD1, SMC1A, SMC2, SMC3, SMC4, SMCHD1, SNRNP200, SOAT1, SPHK2, SRC, SRPRA, SRR, SRXN1, STK24, TAP1, TBC1D10A, TBK1, TCP1, TGM2, TNIK, TOR1B, TRIO, TRIP13, TSPO, TTLL12, TUBA4A, TUBA8, TUBB, TUBB3, TWF2, UBA2, UBA3, UBE2L6, UBE2Q1, UGP2, VARS1, VDAC2, VRK1, VRK3, VWA8, WDR41, WRNIP1, XYLB, YARS1, YES1, ZC3HAV1]	
hydrolase activity, acting on acid anhydrides, in phosphorus-containing anhydrides	GO_MolecularFunction-EBI-UniProt-GOA-ACAP-ARAP_13.05.2021_00h00	6.96E-39	12.0550165	149	[ACAP2, AFDN, AGRN, ARFGAP1, ARFGEF1, ARHGAP12, ARHGAP17, ARHGAP5, ARHGEF11, ARHGEF12, ARRB1, ASAP2, ATL3, ATP1B3, ATP2B1, BAG1, BCR, BMP1, BMS1, CHD1L, CLU, CORO1C, DCPS, DENND2A, DNM1L, DNMBP, DOCK6, DRG1, DUT, DVL2, DYNLL1, EFL1, EIF2B1, EIF2S1, ENPP1, EPHA2, EPHA3, ERBB2, ETFDH, FARP1, FERMT2, FGD4, GARS1, GBP1, GDPGP1, GMPS, GPN1, GPN3, GPSM1, GSK3B, HERC2, HSP90AA1, IPO5, IQGAP3, ITGB1, ITSN1, KIF15, LIMS1, LLGL1, MAP4K4, MFN1, MMUT, MYH10, MYH9, MYO18A, MYO1B, MYO1D, MYO1E, MYO6, NDRG1, NF1, NUCB2, NUDT15, NUDT16, NUDT16L1, NUDT19, PAFAH1B1, PBXIP1, PGAM5, PIN1, PLXNB2, PRKG1, PRUNE1, PTGES2, PYCARD, RAB10, RAB11B, RAB1A, RAB23, RAB3B, RAB3D, RAB5A, RAB8A, RABGEF1, RAC3,	No change

					RALA, RANBP1, RANGAP1, RAP1GDS1, RAP2B, RAP2C, RCC1, RDX, RGS10, RHEB, RHOB, RHOC, RIC8A, RIC8B, RIN1, RNF213, RNGTT, RRAS2, S100A10, SCRIB, SEC23B, SEPTIN10, SEPTIN11, SEPTIN2, SEPTIN7, SEPTIN8, SEPTIN9, SESN2, SH3BP4, SLIT2, SMAP2, SMARCA1, SMC3, SMCHD1, SNX18, SOD1, SRPRA, TBC1D10A, TBC1D17, TBC1D22B, TBC1D4, TBC1D8B, TGM2, TOR1AIP1, TOR1AIP2, TOR1B, TRIO, TSC2, TUBA4A, TUBA8, TUBB, TUBB3, VWA8, WDR41]	
ribonucleotide binding	GO_MolecularFunction-EBI-UniProt-GOA-ACAP-ARAP_13.05.2021_00h00	6.96E-39	13.52915	304	[AARS1, ABCB7, ABCC1, ABCE1, ACACA, ACADVL, ACOT7, ACSF2, ACSL3, ACSL4, ACSS2, ACTB, ACTG1, ACTR2, ACTR3, ADK, AK1, AK3, AK4, AK6, ALDH18A1, APAF1, APRT, AQR, ARAF, ARFGEF1, ARHGAP5, ARHGEF11, ARHGEF12, ASNS, ATL3, ATP1A1, ATP2B1, ATP2B2, ATP2C1, ATP6V1A, ATP6V1B2, BAZ1B, BCR, BMP1, BMS1, CAMK2D, CASK, CCT2, CCT3, CCT6A, CDC34, CDC42BPA, CDK1, CDK16, CDK17, CDK2, CHD1L, CHD2, CHD4, CHD7, CHORDC1, CKB, CMPK1, CNGB1, COLEC12, CTPS1, CYB5R3, DAPK1, DARS1, DCAKD, DDX1, DDX42, DDX47, DENND2A, DGUOK, DHX34, DHX36, DICER1, DNM1L, DNMBP, DOCK6, DRG1, DYNC1LI1, EARS2, ECI2, EFL1, EHD3, EIF2AK2, EIF2B1, EIF2S1, ENPP1, EPHA2, EPHA3, EPHB4, EPRS1, ERBB2, ERCC6L, ETFDH, FARP1, FGD4, FN3K, GARS1, GATB, GBP1, GCDH, GDPGP1, GET3, GLUD1, GLUL,	No change

					GMPS, GNE, GNL3L, GPHN, GPI, GPN1, GPN3, GPSM1, GSK3B, GTF2F2, HACL1, HADHA, HARS1, HELZ, HERC2, HK1, HK2, HSP90AA1, HSP90B1, HSPA14, HSPA4, HSPA5, HSPE1, IARS2, IKBKB, IRGQ, ITM2C, ITPK1, ITSN1, KIF15, LANCL2, LIG1, LIG3, LRRK1, MAGI1, MAP2K3, MAP3K20, MAP3K4, MAP4K4, MAT2A, MCM2, MCM3, MCM4, MCM5, MCM6, MCM7, ME1, MFN1, MLH1, MMAB, MOV10, MSH2, MSH6, MTREX, MTRR, MVD, MVK, MYH10, MYH9, MYO18A, MYO1B, MYO1D, MYO1E, MYO6, NDRG1, NDUFA13, NEK1, NPR2, NUBP2, NUBPL, NUCB2, NUDT16, NUDT19, OPLAH, ORC4, ORC5, OXSR1, PAICS, PANK4, PAPSS2, PBXIP1, PC, PCK2, PDE5A, PDK1, PEBP1, PFAS, PFKM, PI4K2B, PIK3C2A, PIK3CA, PIP4K2B, PKLR, PKM, PKN2, POMK, PPIP5K2, PRKAA1, PRKCI, PRKG1, PSMC2, PSMC3, PSMC6, PTGES2, PTK7, PTPA, PXK, QARS1, RAB10, RAB11B, RAB1A, RAB23, RAB3B, RAB3D, RAB5A, RAB8A, RABGEF1, RABL6, RAC3, RALA, RANBP1, RAP2B, RAP2C, RARS2, RCC1, RECQL, RFC2, RFC5, RHEB, RHOB, RHOC, RIC8A, RIC8B, RIOK3, RIPK2, RNGTT, ROR1, RRAS2, RRM1, SAMHD1, SARS1, SCP2, SEC14L2, SEPTIN10, SEPTIN11, SEPTIN2, SEPTIN7, SEPTIN8, SEPTIN9, SESN2, SH3BP4, SHPRH, SLC25A15, SMARCA1, SMARCAD1, SMC1A, SMC2, SMC3, SMC4, SMCHD1, SNRNP200, SOAT1, SPHK2, SRC, SRPRA, SRR, SRXN1, STK24,	

					TAP1, TBC1D10A, TBK1, TCP1, TGM2, TNIK, TOR1B, TRIO, TRIP13, TSPO, TTLL12, TUBA4A, TUBA8, TUBB, TUBB3, TWF2, UBA2, UBA3, UBE2L6, UBE2Q1, UGP2, VARS1, VDAC2, VRK1, VRK3, VWA8, WDR41, WRNIP1, XYLB, YARS1, YES1]	
purine ribonucleoside triphosphate binding	GO_MolecularFunction-EBI-UniProt-GOA-ACAP-ARAP_13.05.2021_00h00	6.96E-39	13.305322	285	[AARS1, ABCB7, ABCC1, ABCE1, ACACA, ACSF2, ACSL3, ACSL4, ACSS2, ACTB, ACTG1, ACTR2, ACTR3, ADK, AK1, AK3, AK4, AK6, ALDH18A1, APAF1, AQR, ARAF, ARFGEF1, ARHGAP5, ARHGEF11, ARHGEF12, ASNS, ATL3, ATP1A1, ATP2B1, ATP2B2, ATP2C1, ATP6V1A, ATP6V1B2, BAZ1B, BCR, BMP1, BMS1, CAMK2D, CASK, CCT2, CCT3, CCT6A, CDC34, CDC42BPA, CDK1, CDK16, CDK17, CDK2, CHD1L, CHD2, CHD4, CHD7, CHORDC1, CKB, CMPK1, COLEC12, CTPS1, DAPK1, DARS1, DCAKD, DDX1, DDX42, DDX47, DENND2A, DGUOK, DHX34, DHX36, DICER1, DNM1L, DNMBP, DOCK6, DRG1, DYNC1LI1, EARS2, EFL1, EHD3, EIF2AK2, EIF2B1, EIF2S1, ENPP1, EPHA2, EPHA3, EPHB4, EPRS1, ERBB2, ERCC6L, ETFDH, FARP1, FGD4, FN3K, GARS1, GATB, GBP1, GDPGP1, GET3, GLUD1, GLUL, GMPS, GNE, GNL3L, GPHN, GPI, GPN1, GPN3, GPSM1, GSK3B, GTF2F2, HACL1, HARS1, HELZ, HERC2, HK1, HK2, HSP90AA1, HSP90B1, HSPA14, HSPA4, HSPA5, HSPE1, IARS2, IKBKB, IRGQ, ITM2C, ITPK1, ITSN1, KIF15, LANCL2, LIG1, LIG3, LRRK1, MAGI1, MAP2K3, MAP3K20, MAP3K4, MAP4K4,	No change

					MAT2A, MCM2, MCM3, MCM4, MCM5, MCM6, MCM7, MFN1, MLH1, MMAB, MOV10, MSH2, MSH6, MTREX, MVD, MVK, MYH10, MYH9, MYO18A, MYO1B, MYO1D, MYO1E, MYO6, NDRG1, NDUFA13, NEK1, NPR2, NUBP2, NUBPL, NUCB2, NUDT16, NUDT19, OPLAH, ORC4, ORC5, OXSR1, PAICS, PANK4, PAPSS2, PBXIP1, PC, PCK2, PDK1, PEBP1, PFAS, PFKM, PI4K2B, PIK3C2A, PIK3CA, PIP4K2B, PKLR, PKM, PKN2, POMK, PPIP5K2, PRKAA1, PRKCI, PRKG1, PSMC2, PSMC3, PSMC6, PTGES2, PTK7, PTPA, PXK, QARS1, RAB10, RAB11B, RAB1A, RAB23, RAB3B, RAB3D, RAB5A, RAB8A, RABGEF1, RABL6, RAC3, RALA, RAP2B, RAP2C, RARS2, RCC1, RECQL, RFC2, RFC5, RHEB, RHOB, RHOC, RIC8A, RIC8B, RIOK3, RIPK2, RNGTT, ROR1, RRAS2, RRM1, SAMHD1, SARS1, SEC14L2, SEPTIN10, SEPTIN11, SEPTIN2, SEPTIN7, SEPTIN8, SEPTIN9, SHPRH, SLC25A15, SMARCA1, SMARCAD1, SMC1A, SMC2, SMC3, SMC4, SMCHD1, SNRNP200, SPHK2, SRC, SRPRA, SRR, SRXN1, STK24, TAP1, TBC1D10A, TBK1, TCP1, TGM2, TNIK, TOR1B, TRIO, TRIP13, TTLL12, TUBA4A, TUBA8, TUBB, TUBB3, TWF2, UBA2, UBA3, UBE2L6, UBE2Q1, VARS1, VRK1, VRK3, VWA8, WDR41, WRNIP1, XYLB, YARS1, YES1]	
purine nucleotide binding	GO_MolecularFunction-EBI-UniProt-GOA-ACAP-ARAP_13.05.2021_00h00	6.96E-39	13.52075	303	[AARS1, ABCB7, ABCC1, ABCE1, ACACA, ACADVL, ACOT7, ACSF2, ACSL3, ACSL4, ACSS2, ACTB, ACTG1, ACTR2, ACTR3, ADK, AK1,	No change

					AK3, AK4, AK6, ALDH18A1, APAF1, APRT, AQR, ARAF, ARFGEF1, ARHGAP5, ARHGEF11, ARHGEF12, ASNS, ATL3, ATP1A1, ATP2B1, ATP2B2, ATP2C1, ATP6V1A, ATP6V1B2, BAG1, BAZ1B, BCR, BMP1, BMS1, CAMK2D, CASK, CCT2, CCT3, CCT6A, CDC34, CDC42BPA, CDK1, CDK16, CDK17, CDK2, CHD1L, CHD2, CHD4, CHD7, CHORDC1, CKB, CMPK1, CNGB1, COLEC12, CTPS1, CYB5R3, DAPK1, DARS1, DCAKD, DDX1, DDX42, DDX47, DENND2A, DGUOK, DHX34, DHX36, DICER1, DNM1L, DNMBP, DOCK6, DRG1, DYNC1LI1, EARS2, ECI2, EFL1, EHD3, EIF2AK2, EIF2B1, EIF2S1, ENPP1, EPHA2, EPHA3, EPHB4, EPRS1, ERBB2, ERCC6L, ETFDH, FAM114A2, FARP1, FGD4, FN3K, GARS1, GATB, GBP1, GCDH, GDPGP1, GET3, GLUD1, GLUL, GMPS, GNE, GNL3L, GPHN, GPI, GPN1, GPN3, GPSM1, GSK3B, GTF2F2, HACL1, HADHA, HARS1, HELZ, HERC2, HK1, HK2, HSP90AA1, HSP90B1, HSPA14, HSPA4, HSPA5, HSPE1, IARS2, IKBKB, IRGQ, ITM2C, ITPK1, ITSN1, KIF15, LANCL2, LIG1, LIG3, LRRK1, MAGI1, MAP2K3, MAP3K20, MAP3K4, MAP4K4, MAT2A, MCM2, MCM3, MCM4, MCM5, MCM6, MCM7, ME1, MFN1, MLH1, MMAB, MOV10, MSH2, MSH6, MTREX, MVD, MVK, MYH10, MYH9, MYO18A, MYO1B, MYO1D, MYO1E, MYO6, NDRG1, NDUFA13, NEK1, NPR2, NUBP2, NUBPL, NUCB2, NUDT16, NUDT19, OPLAH, ORC4, ORC5, OXSR1, PAICS,	

					PANK4, PAPSS2, PBXIP1, PC, PCK2, PDE5A, PDK1, PEBP1, PFAS, PFKM, PI4K2B, PIK3C2A, PIK3CA, PIP4K2B, PKLR, PKM, PKN2, POMK, PPIP5K2, PRKAA1, PRKCI, PRKG1, PSMC2, PSMC3, PSMC6, PTGES2, PTK7, PTPA, PXK, QARS1, RAB10, RAB11B, RAB1A, RAB23, RAB3B, RAB3D, RAB5A, RAB8A, RABGEF1, RABL6, RAC3, RALA, RANBP1, RAP2B, RAP2C, RARS2, RCC1, RECQL, RFC2, RFC5, RHEB, RHOB, RHOC, RIC8A, RIC8B, RIOK3, RIPK2, RNGTT, ROR1, RRAS2, RRM1, SAMHD1, SARS1, SCP2, SEC14L2, SEPTIN10, SEPTIN11, SEPTIN2, SEPTIN7, SEPTIN8, SEPTIN9, SESN2, SH3BP4, SHPRH, SLC25A15, SMARCA1, SMARCAD1, SMC1A, SMC2, SMC3, SMC4, SMCHD1, SNRNP200, SOAT1, SPHK2, SRC, SRPRA, SRR, SRXN1, STK24, TAP1, TBC1D10A, TBK1, TCP1, TGM2, TNIK, TOR1B, TRIO, TRIP13, TSPO, TTLL12, TUBA4A, TUBA8, TUBB, TUBB3, TWF2, UBA2, UBA3, UBE2L6, UBE2Q1, VARS1, VRK1, VRK3, VWA8, WDR41, WRNIP1, XYLB, YARS1, YES1]	
purine ribonucleotide binding	GO_MolecularFunction-EBI-UniProt-GOA-ACAP-ARAP_13.05.2021_00h00	6.96E-39	13.522013	301	[AARS1, ABCB7, ABCC1, ABCE1, ACACA, ACADVL, ACOT7, ACSF2, ACSL3, ACSL4, ACSS2, ACTB, ACTG1, ACTR2, ACTR3, ADK, AK1, AK3, AK4, AK6, ALDH18A1, APAF1, APRT, AQR, ARAF, ARFGEF1, ARHGAP5, ARHGEF11, ARHGEF12, ASNS, ATL3, ATP1A1, ATP2B1, ATP2B2, ATP2C1, ATP6V1A, ATP6V1B2, BAZ1B, BCR, BMP1, BMS1, CAMK2D, CASK, CCT2, CCT3, CCT6A, CDC34, CDC42BPA, CDK1, CDK16,	No change

					CDK17, CDK2, CHD1L, CHD2, CHD4, CHD7, CHORDC1, CKB, CMPK1, CNGB1, COLEC12, CTPS1, CYB5R3, DAPK1, DARS1, DCAKD, DDX1, DDX42, DDX47, DENND2A, DGUOK, DHX34, DHX36, DICER1, DNM1L, DNMBP, DOCK6, DRG1, DYNC1LI1, EARS2, ECI2, EFL1, EHD3, EIF2AK2, EIF2B1, EIF2S1, ENPP1, EPHA2, EPHA3, EPHB4, EPRS1, ERBB2, ERCC6L, ETFDH, FARP1, FGD4, FN3K, GARS1, GATB, GBP1, GCDH, GDPGP1, GET3, GLUD1, GLUL, GMPS, GNE, GNL3L, GPHN, GPI, GPN1, GPN3, GPSM1, GSK3B, GTF2F2, HACL1, HADHA, HARS1, HELZ, HERC2, HK1, HK2, HSP90AA1, HSP90B1, HSPA14, HSPA4, HSPA5, HSPE1, IARS2, IKBKB, IRGQ, ITM2C, ITPK1, ITSN1, KIF15, LANCL2, LIG1, LIG3, LRRK1, MAGI1, MAP2K3, MAP3K20, MAP3K4, MAP4K4, MAT2A, MCM2, MCM3, MCM4, MCM5, MCM6, MCM7, ME1, MFN1, MLH1, MMAB, MOV10, MSH2, MSH6, MTREX, MVD, MVK, MYH10, MYH9, MYO18A, MYO1B, MYO1D, MYO1E, MYO6, NDRG1, NDUFA13, NEK1, NPR2, NUBP2, NUBPL, NUCB2, NUDT16, NUDT19, OPLAH, ORC4, ORC5, OXSR1, PAICS, PANK4, PAPSS2, PBXIP1, PC, PCK2, PDE5A, PDK1, PEBP1, PFAS, PFKM, PI4K2B, PIK3C2A, PIK3CA, PIP4K2B, PKLR, PKM, PKN2, POMK, PPIP5K2, PRKAA1, PRKCI, PRKG1, PSMC2, PSMC3, PSMC6, PTGES2, PTK7, PTPA, PXK, QARS1, RAB10, RAB11B, RAB1A, RAB23, RAB3B, RAB3D, RAB5A,	

					RAB8A, RABGEF1, RABL6, RAC3, RALA, RANBP1, RAP2B, RAP2C, RARS2, RCC1, RECQL, RFC2, RFC5, RHEB, RHOB, RHOC, RIC8A, RIC8B, RIOK3, RIPK2, RNGTT, ROR1, RRAS2, RRM1, SAMHD1, SARS1, SCP2, SEC14L2, SEPTIN10, SEPTIN11, SEPTIN2, SEPTIN7, SEPTIN8, SEPTIN9, SESN2, SH3BP4, SHPRH, SLC25A15, SMARCA1, SMARCAD1, SMC1A, SMC2, SMC3, SMC4, SMCHD1, SNRNP200, SOAT1, SPHK2, SRC, SRPRA, SRR, SRXN1, STK24, TAP1, TBC1D10A, TBK1, TCP1, TGM2, TNIK, TOR1B, TRIO, TRIP13, TSPO, TTLL12, TUBA4A, TUBA8, TUBB, TUBB3, TWF2, UBA2, UBA3, UBE2L6, UBE2Q1, VARS1, VRK1, VRK3, VWA8, WDR41, WRNIP1, XYLB, YARS1, YES1]	
adenyl nucleotide binding	GO_MolecularFunction-EBI-UniProt-GOA-ACAP-ARAP_13.05.2021_00h00	6.96E-39	13.917842	227	[AARS1, ABCB7, ABCC1, ABCE1, ACACA, ACADVL, ACOT7, ACSF2, ACSL3, ACSL4, ACSS2, ACTB, ACTG1, ACTR2, ACTR3, ADK, AK1, AK3, AK4, AK6, ALDH18A1, APAF1, APRT, AQR, ARAF, ASNS, ATP1A1, ATP2B1, ATP2B2, ATP2C1, ATP6V1A, ATP6V1B2, BAG1, BAZ1B, BCR, BMS1, CAMK2D, CASK, CCT2, CCT3, CCT6A, CDC34, CDC42BPA, CDK1, CDK16, CDK17, CDK2, CHD1L, CHD2, CHD4, CHD7, CHORDC1, CKB, CMPK1, CNGB1, COLEC12, CTPS1, CYB5R3, DAPK1, DARS1, DCAKD, DDX1, DDX42, DDX47, DGUOK, DHX34, DHX36, DICER1, DYNC1LI1, EARS2, ECI2, EHD3, EIF2AK2, ENPP1, EPHA2, EPHA3, EPHB4, EPRS1, ERBB2, ERCC6L, FN3K, GARS1, GATB, GCDH, GET3, GLUD1, GLUL,	No change

					GMPS, GNE, GPHN, GPI, GSK3B, GTF2F2, HACL1, HADHA, HARS1, HELZ, HK1, HK2, HSP90AA1, HSP90B1, HSPA14, HSPA4, HSPA5, HSPE1, IARS2, IKBKB, ITM2C, ITPK1, KIF15, LANCL2, LIG1, LIG3, LRRK1, MAGI1, MAP2K3, MAP3K20, MAP3K4, MAP4K4, MAT2A, MCM2, MCM3, MCM4, MCM5, MCM6, MCM7, ME1, MLH1, MMAB, MOV10, MSH2, MSH6, MTREX, MVD, MVK, MYH10, MYH9, MYO18A, MYO1B, MYO1D, MYO1E, MYO6, NDUFA13, NEK1, NPR2, NUBP2, NUBPL, OPLAH, ORC4, ORC5, OXSR1, PAICS, PANK4, PAPSS2, PC, PDK1, PEBP1, PFAS, PFKM, PI4K2B, PIK3C2A, PIK3CA, PIP4K2B, PKLR, PKM, PKN2, POMK, PPIP5K2, PRKAA1, PRKCI, PRKG1, PSMC2, PSMC3, PSMC6, PTK7, PTPA, PXK, QARS1, RARS2, RECQL, RFC2, RFC5, RIOK3, RIPK2, ROR1, RRM1, SARS1, SCP2, SEC14L2, SHPRH, SLC25A15, SMARCA1, SMARCAD1, SMC1A, SMC2, SMC3, SMC4, SMCHD1, SNRNP200, SOAT1, SPHK2, SRC, SRR, SRXN1, STK24, TAP1, TBK1, TCP1, TNIK, TOR1B, TRIO, TRIP13, TSPO, TTLL12, TWF2, UBA2, UBA3, UBE2L6, UBE2Q1, VARS1, VRK1, VRK3, VWA8, WRNIP1, XYLB, YARS1, YES1]	
nucleoside-triphosphatase activity	GO_MolecularFunction-EBI-UniProt-GOA-ACAP-ARAP_13.05.2021_00h00	6.96E-39	11.845493	138	[ACAP2, AFDN, AGRN, ARFGAP1, ARFGEF1, ARHGAP12, ARHGAP17, ARHGAP5, ARHGEF11, ARHGEF12, ARRB1, ASAP2, ATL3, ATP1B3, ATP2B1, BAG1, BCR, BMP1, BMS1, CHD1L, CLU, CORO1C, DENND2A, DNM1L, DNMBP, DOCK6, DRG1, DVL2,	No change

					DYNLL1, EFL1, EIF2B1, EIF2S1, EPHA2, EPHA3, ERBB2, ETFDH, FARP1, FERMT2, FGD4, GBP1, GDPGP1, GPN1, GPN3, GPSM1, GSK3B, HERC2, HSP90AA1, IPO5, IQGAP3, ITGB1, ITSN1, KIF15, LIMS1, LLGL1, MAP4K4, MFN1, MMUT, MYH10, MYH9, MYO18A, MYO1B, MYO1D, MYO1E, MYO6, NDRG1, NF1, NUCB2, NUDT19, PAFAH1B1, PBXIP1, PGAM5, PIN1, PLXNB2, PRKG1, PTGES2, PYCARD, RAB10, RAB11B, RAB1A, RAB23, RAB3B, RAB3D, RAB5A, RAB8A, RABGEF1, RAC3, RALA, RANBP1, RANGAP1, RAP1GDS1, RAP2B, RAP2C, RCC1, RDX, RGS10, RHEB, RHOB, RHOC, RIC8A, RIC8B, RIN1, RNF213, RRAS2, S100A10, SCRIB, SEC23B, SEPTIN10, SEPTIN11, SEPTIN2, SEPTIN7, SEPTIN8, SEPTIN9, SESN2, SH3BP4, SLIT2, SMAP2, SMC3, SMCHD1, SNX18, SOD1, SRPRA, TBC1D10A, TBC1D17, TBC1D22B, TBC1D4, TBC1D8B, TGM2, TOR1AIP1, TOR1AIP2, TOR1B, TRIO, TSC2, TUBA4A, TUBA8, TUBB, TUBB3, VWA8, WDR41]	
guanyl ribonucleotide binding	GO_MolecularFunction-EBI-UniProt-GOA-ACAP-ARAP_13.05.2021_00h00	6.96E-39	13.412817	90	[AK3, AK4, ARFGEF1, ARHGAP5, ARHGEF11, ARHGEF12, ATL3, BCR, BMP1, BMS1, CNGB1, DAPK1, DENND2A, DNM1L, DNMBP, DOCK6, DRG1, DYNC1LI1, EFL1, EHD3, EIF2B1, EIF2S1, ETFDH, FARP1, FGD4, GBP1, GDPGP1, GLUD1, GNL3L, GPN1, GPN3, GPSM1, HERC2, IRGQ, ITSN1, LANCL2, LRRK1, MFN1, NDRG1, NPR2, NUCB2, NUDT16, NUDT19, PBXIP1, PCK2,	No change

					PDE5A, PIP4K2B, PRKG1, PTGES2, RAB10, RAB11B, RAB1A, RAB23, RAB3B, RAB3D, RAB5A, RAB8A, RABGEF1, RABL6, RAC3, RALA, RANBP1, RAP2B, RAP2C, RCC1, RHEB, RHOB, RHOC, RIC8A, RIC8B, RNGTT, RRAS2, SAMHD1, SEPTIN10, SEPTIN11, SEPTIN2, SEPTIN7, SEPTIN8, SEPTIN9, SESN2, SH3BP4, SRPRA, TBC1D10A, TGM2, TRIO, TUBA4A, TUBA8, TUBB, TUBB3, WDR41]	
ATP binding	GO_MolecularFunction-EBI-UniProt-GOA-ACAP-ARAP_13.05.2021_00h00	6.96E-39	13.78866	214	[AARS1, ABCB7, ABCC1, ABCE1, ACACA, ACSF2, ACSL3, ACSL4, ACSS2, ACTB, ACTG1, ACTR2, ACTR3, ADK, AK1, AK3, AK4, AK6, ALDH18A1, APAF1, AQR, ARAF, ASNS, ATP1A1, ATP2B1, ATP2B2, ATP2C1, ATP6V1A, ATP6V1B2, BAZ1B, BCR, BMS1, CAMK2D, CASK, CCT2, CCT3, CCT6A, CDC34, CDC42BPA, CDK1, CDK16, CDK17, CDK2, CHD1L, CHD2, CHD4, CHD7, CHORDC1, CKB, CMPK1, COLEC12, CTPS1, DAPK1, DARS1, DCAKD, DDX1, DDX42, DDX47, DGUOK, DHX34, DHX36, DICER1, DYNC1LI1, EARS2, EHD3, EIF2AK2, ENPP1, EPHA2, EPHA3, EPHB4, EPRS1, ERBB2, ERCC6L, FN3K, GARS1, GATB, GET3, GLUD1, GLUL, GMPS, GNE, GPHN, GPI, GSK3B, GTF2F2, HACL1, HARS1, HELZ, HK1, HK2, HSP90AA1, HSP90B1, HSPA14, HSPA4, HSPA5, HSPE1, IARS2, IKBKB, ITM2C, ITPK1, KIF15, LANCL2, LIG1, LIG3, LRRK1, MAGI1, MAP2K3, MAP3K20, MAP3K4, MAP4K4, MAT2A, MCM2, MCM3, MCM4, MCM5, MCM6,	No change

					MCM7, MLH1, MMAB, MOV10, MSH2, MSH6, MTREX, MVD, MVK, MYH10, MYH9, MYO18A, MYO1B, MYO1D, MYO1E, MYO6, NDUFA13, NEK1, NPR2, NUBP2, NUBPL, OPLAH, ORC4, ORC5, OXSR1, PAICS, PANK4, PAPSS2, PC, PDK1, PEBP1, PFAS, PFKM, PI4K2B, PIK3C2A, PIK3CA, PIP4K2B, PKLR, PKM, PKN2, POMK, PPIP5K2, PRKAA1, PRKCI, PRKG1, PSMC2, PSMC3, PSMC6, PTK7, PTPA, PXK, QARS1, RARS2, RECQL, RFC2, RFC5, RIOK3, RIPK2, ROR1, RRM1, SARS1, SEC14L2, SHPRH, SLC25A15, SMARCA1, SMARCAD1, SMC1A, SMC2, SMC3, SMC4, SMCHD1, SNRNP200, SPHK2, SRC, SRR, SRXN1, STK24, TAP1, TBK1, TCP1, TNIK, TOR1B, TRIO, TRIP13, TTLL12, TWF2, UBA2, UBA3, UBE2L6, UBE2Q1, VARS1, VRK1, VRK3, VWA8, WRNIP1, XYLB, YARS1, YES1]	
GTP binding	GO_MolecularFunction-EBI-UniProt-GOA-ACAP-ARAP_13.05.2021_00h00	6.96E-39	13.166144	84	[AK3, AK4, ARFGEF1, ARHGAP5, ARHGEF11, ARHGEF12, ATL3, BCR, BMP1, BMS1, DAPK1, DENND2A, DNM1L, DNMBP, DOCK6, DRG1, DYNC1LI1, EFL1, EHD3, EIF2B1, EIF2S1, ETFDH, FARP1, FGD4, GBP1, GDPGP1, GLUD1, GNL3L, GPN1, GPN3, GPSM1, HERC2, IRGQ, ITSN1, LANCL2, LRRK1, MFN1, NDRG1, NPR2, NUCB2, NUDT16, NUDT19, PBXIP1, PCK2, PIP4K2B, PTGES2, RAB10, RAB11B, RAB1A, RAB23, RAB3B, RAB3D, RAB5A, RAB8A, RABGEF1, RABL6, RAC3, RALA, RAP2B, RAP2C, RCC1, RHEB, RHOB, RHOC, RIC8A, RIC8B, RNGTT, RRAS2, SAMHD1,	No change

					SEPTIN10, SEPTIN11, SEPTIN2, SEPTIN7, SEPTIN8, SEPTIN9, SRPRA, TBC1D10A, TGM2, TRIO, TUBA4A, TUBA8, TUBB, TUBB3, WDR41]	
GTPase activity	GO_MolecularFunction-EBI-UniProt-GOA-ACAP-ARAP_13.05.2021_00h00	6.96E-39	12.124352	117	[ACAP2, AFDN, AGRN, ARFGAP1, ARFGEF1, ARHGAP12, ARHGAP17, ARHGAP5, ARHGEF11, ARHGEF12, ARRB1, ASAP2, ATL3, BCR, BMP1, BMS1, CORO1C, DENND2A, DNM1L, DNMBP, DOCK6, DRG1, DVL2, DYNLL1, EFL1, EIF2B1, EIF2S1, EPHA2, EPHA3, ERBB2, ETFDH, FARP1, FERMT2, FGD4, GBP1, GDPGP1, GPN1, GPN3, GPSM1, GSK3B, HERC2, IPO5, IQGAP3, ITGB1, ITSN1, LIMS1, LLGL1, MAP4K4, MFN1, MMUT, NDRG1, NF1, NUCB2, NUDT19, PAFAH1B1, PBXIP1, PGAM5, PIN1, PLXNB2, PRKG1, PTGES2, PYCARD, RAB10, RAB11B, RAB1A, RAB23, RAB3B, RAB3D, RAB5A, RAB8A, RABGEF1, RAC3, RALA, RANBP1, RANGAP1, RAP1GDS1, RAP2B, RAP2C, RCC1, RDX, RGS10, RHEB, RHOB, RHOC, RIC8A, RIC8B, RIN1, RRAS2, S100A10, SCRIB, SEC23B, SEPTIN10, SEPTIN11, SEPTIN2, SEPTIN7, SEPTIN8, SEPTIN9, SESN2, SH3BP4, SLIT2, SMAP2, SNX18, SOD1, SRPRA, TBC1D10A, TBC1D17, TBC1D22B, TBC1D4, TBC1D8B, TGM2, TRIO, TSC2, TUBA4A, TUBA8, TUBB, TUBB3, WDR41]	No change
replication fork	GO_CellularComponent-EBI-UniProt-GOA-ACAP-ARAP_13.05.2021_00h00	7.51E-18	23.61111	17	[BAZ1B, BCAS2, CDC5L, DNMT1, ERCC5, HMCES, MCM3, POLD1, POLD2, PRIM2, PRPF19, RFC2, RFC3, RFC5, RPA1, RPA2, SMARCAD1]	Upregulation

DNA replication	GO_BiologicalProcess-EBI-UniProt-GOA-ACAP-ARAP_13.05.2021_00h00	7.51E-18	16.804407	61	[ATG7, CDC34, CDK1, CDK2, CHAF1B, DUT, EXD2, FEN1, GRWD1, KAT7, LIG1, LIG3, LRWD1, MCM2, MCM3, MCM4, MCM5, MCM6, MCM7, MCMBP, MMAB, ORC4, ORC5, POLD1, POLD2, POLE2, PRIM2, PSMA3, PSMB10, PSMB2, PSMC2, PSMC3, PSMC6, PSMD1, PSMD12, PSMD14, PSMD2, PSMD6, PSMD7, PSMD8, PSME1, PSME2, PSME4, RAD9A, RBBP4, RECQL, RFC2, RFC3, RFC5, RPA1, RPA2, RRM1, SAMHD1, SET, SLC25A15, SMC3, SSRP1, STON1, SUPT16H, WRNIP1, ZMPSTE24]	No change
nuclear replication fork	GO_CellularComponent-EBI-UniProt-GOA-ACAP-ARAP_13.05.2021_00h00	7.51E-18	31.578947	12	[BAZ1B, BCAS2, CDC5L, ERCC5, MCM3, POLD1, POLD2, PRIM2, PRPF19, RPA1, RPA2, SMARCAD1]	Upregulation
nuclear replisome	GO_CellularComponent-EBI-UniProt-GOA-ACAP-ARAP_13.05.2021_00h00	7.51E-18	40	10	[BCAS2, CDC5L, ERCC5, MCM3, POLD1, POLD2, PRIM2, PRPF19, RPA1, RPA2]	Upregulation
nuclear chromosome	GO_CellularComponent-EBI-UniProt-GOA-ACAP-ARAP_13.05.2021_00h00	7.51E-18	15.789474	39	[ADD3, BAZ1B, BCAS2, CDC5L, ERCC5, HDAC2, LRPPRC, LRWD1, MACROH2A1, MCM2, MCM3, MCM4, MCM5, MCM6, MCM7, MLH1, MORF4L1, NCAPD2, ORC4, ORC5, POLD1, POLD2, POLE2, PRIM2, PRPF19, RAD21, RAD9A, RBBP4, RCC1, RPA1, RPA2, SLC25A15, SMARCAD1, SMARCB1, SMARCE1, SMC1A, SMC2, SMC3, SMCHD1]	Upregulation
DNA-dependent DNA replication	GO_BiologicalProcess-EBI-UniProt-GOA-ACAP-ARAP_13.05.2021_00h00	7.51E-18	21.276596	50	[ATG7, CDC34, CDK2, EXD2, FEN1, KAT7, LIG1, LIG3, LRWD1, MCM2, MCM3, MCM4, MCM5, MCM6, MCM7, MCMBP, MMAB, ORC4, ORC5, POLD1, POLD2, POLE2, PRIM2, PSMA3, PSMB10, PSMB2, PSMC2, PSMC3, PSMC6, PSMD1, PSMD12,	No change

					PSMD14, PSMD2, PSMD6, PSMD7, PSMD8, PSME1, PSME2, PSME4, RECQL, RFC2, RFC3, RFC5, RPA1, RPA2, SAMHD1, SLC25A15, STON1, WRNIP1, ZMPSTE24]	
pre-replicative complex assembly involved in nuclear cell cycle DNA replication	GO_BiologicalProcess-EBI-UniProt-GOA-ACAP-ARAP_13.05.2021_00h00	7.51E-18	75	6	[MCM2, MCM3, MCM4, MCM5, MCM6, MCM7]	Upregulation
pre-replicative complex assembly	GO_BiologicalProcess-EBI-UniProt-GOA-ACAP-ARAP_13.05.2021_00h00	7.51E-18	36.231884	25	[MCM2, MCM3, MCM4, MCM5, MCM6, MCM7, ORC4, ORC5, PSMA3, PSMB10, PSMB2, PSMC2, PSMC3, PSMC6, PSMD1, PSMD12, PSMD14, PSMD2, PSMD6, PSMD7, PSMD8, PSME1, PSME2, PSME4, SLC25A15]	Downregulation
transcription-coupled nucleotide-excision repair	GO_BiologicalProcess-EBI-UniProt-GOA-ACAP-ARAP_13.05.2021_00h00	7.51E-18	24.390244	20	[AQR, CCNH, CUL4B, ERCC5, LIG1, LIG3, POLD1, POLD2, POLR2A, POLR2B, POLR2H, POLR2I, PPIE, PRPF19, RFC2, RFC3, RFC5, RPA1, RPA2, USP7]	Upregulation
regulation of signaling	GO_BiologicalProcess-EBI-UniProt-GOA-ACAP-ARAP_13.05.2021_00h00	2.40E-18	9.252286	344	[ABAT, ABCE1, ACSL4, ADAM10, ADNP, AGPAT2, AKAP12, AKR1B1, APEX1, APIP, APLP2, APP, APPL2, ARAF, ARFGAP1, ARFGEF1, ARHGAP12, ARHGAP17, ARHGAP5, ARHGEF11, ARHGEF12, ARPP19, ARRB1, ASPM, ATP1A1, ATP1B3, ATP2B1, ATP2B2, ATP2C1, ATP6V0C, BAIAP2, BCR, BST1, C1QBP, C2CD2L, CAPN5, CASK, CC2D1A, CCDC22, CCN2, CD109, CD81, CDC34, CDC73, CDH11, CDK1, CDK16, CDK2, CDK5RAP3, CHD4, CHD7, CHORDC1, CHP1, CLU, CNGB1, CNOT1, CSPG4, CTNNB1, CTSC, CXCL12, CYRIB, DAB2, DAPK1, DDX1, DHX34, DHX36, DIPK2A, DNM1L, DUSP3, DVL2, DYNC1LI1, DYNLL1, ECE1, EEF1E1, EHD3, EIF2AK2, EIF2S1, EIF3A, EIF6, ENPP1, ENY2, EPHA2,	No change

					ERBB2, ETFDH, EZR, FASN, FERMT1, FERMT2, FGD4, FGF2, FHL2, FKBP1A, FMR1, FN1, GAB1, GAPDH, GARS1, GBP1, GIPC1, GLUD1, GLUL, GOLT1B, GOT1, GPC1, GPC6, GPD1L, GPI, GPR108, GSK3B, GSTP1, HADH, HADHA, HDAC2, HERC4, HGS, HHIP, HIP1, HMOX1, HPX, HSP90AA1, HSPA5, IFIT5, IFT140, IGF2BP1, IGFBP7, IKBKB, IKBKG, IQGAP3, ITGA3, ITGAV, ITGB1, ITM2C, ITSN1, IVNS1ABP, JAG1, JAGN1, KDM1A, LANCL2, LEMD2, LIMS1, LLGL1, LMCD1, LMO7, LRRC8A, LRRK1, LTBP1, MACF1, MAD2L1, MALT1, MAOA, MAP1B, MAP2K3, MAP3K20, MAP3K4, MAP4K4, MBD3, MMAB, MME, MTCH1, MVP, MYADM, MYD88, MYO1E, NCDN, NCK2, NCLN, NCOA5, NDUFA13, NDUFS3, NECTIN2, NEDD4, NENF, NF1, NF2, NIBAN2, NISCH, NLE1, NMT1, NOP53, NPLOC4, NPM1, NPR2, NUP62, NXN, OSBPL8, PAFAH1B1, PARP1, PARP14, PAWR, PCK2, PEBP1, PELO, PFKM, PHPT1, PIGU, PIK3CA, PIN1, PIP4K2B, PKN2, PLCG1, PLPP3, PML, POFUT1, PPM1B, PPP1CA, PPP3CA, PPP5C, PRDX1, PRKAA1, PRKAB2, PRKCI, PSMA3, PSMB10, PSMB2, PSMC2, PSMC3, PSMC6, PSMD1, PSMD12, PSMD14, PSMD2, PSMD6, PSMD7, PSMD8, PSME1, PSME2, PSME4, PTGES2, PTGIS, PTK7, PTPMT1, PTPN12, PTRH2, PXK, PXN, PYCARD, QARS1, RAB5A, RAB8A, RAC3, RAD9A, RAI14, RBBP4, RCN3, RDX, RFC2, RFC3, RFC5, RGS10, RHEB, RHOB,	

					RHOC, RIOK3, RIPK2, RNF213, ROR1, RPA1, RPA2, RPF2, RPL22, RPL5, RTN4, SAMHD1, SARM1, SCAI, SCRIB, SELENOT, SERPINE2, SESN2, SEZ6L2, SH3BP4, SH3KBP1, SIRT1, SLC12A2, SLC1A1, SLC25A5, SLC2A1, SLC44A2, SLC9A1, SLIT2, SMAD4, SMARCB1, SMPD1, SNW1, SNX5, SOD1, SOD2, SORBS3, SPART, SPHK2, SQSTM1, SRC, SSRP1, STAT1, STAT2, STK24, STRN3, SUFU, SUPT16H, TAB2, TAX1BP1, TBK1, TF, TGFBR3, TGM2, THBS1, THEM4, TMEM65, TNIK, TPBG, TPRG1L, TPX2, TRADD, TRIM21, TRIM25, TRIM59, TRIO, TRIP6, TSC2, TTC5, TTLL12, TXN, TYMP, UACA, UBAC2, UBR2, UBTF, UFD1, URI1, USP7, VDAC2, VPS25, VRK3, VTN, WDR11, WFS1, WWC2, WWP2, XIAP, YAP1, ZCCHC3, ZDHHC13, ZFAND6, ZMPSTE24]	
response to organic substance	GO_BiologicalProcess-EBI-UniProt-GOA-ACAP-ARAP_13.05.2021_00h00	2.40E-18	10.985277	388	[ABAT, ABCC1, ABCE1, ACACA, ACADVL, ACAP2, ACSL3, ACSL4, ACTB, ACTG1, ACTR2, ACTR3, ADAM10, ADNP, AGPAT2, AGRN, AKAP12, AKR1B1, ANKZF1, ANXA3, ANXA7, APAF1, APEX1, APLP2, APP, APPL2, APRT, ARFGAP1, ARID1B, ARPP19, ASAH1, ASH2L, ASNS, ATG7, ATP1A1, ATP2B1, ATP6V0A1, ATP6V0C, ATP6V1A, ATP6V1B2, AUP1, B2M, BAIAP2, C2CD2L, CAMK2D, CANX, CAPN5, CASK, CASP3, CASP6, CCN2, CD109, CD81, CDC34, CDC5L, CDC73, CDK1, CDK16, CDK5RAP3, CHAMP1, CLGN, CLU, CNOT1, COL4A1, COL5A2, COL6A1, COLEC12, CORO1B, CTNNB1, CTSC, CUL1, CXCL12, CYRIB, DAB2, DAPK1,	No change

					DCN, DCPS, DDI2, DDX1, DERL1, DHCR24, DHFR, DHX34, DHX36, DIAPH1, DNM1L, DNMT1, DPYSL2, DPYSL3, DUSP3, DYNLL1, EIF2AK2, EIF2B1, EIF2S1, EIF6, ENPP1, ENY2, EPHA2, EPHA3, EPHX1, EPM2AIP1, EPRS1, ERBB2, ERLIN2, ERP44, ETFDH, EXTL2, EZR, FASN, FERMT1, FERMT2, FGF2, FHL2, FIBP, FKBP1A, FLRT2, FMR1, FN1, FSCN1, FUT8, G6PD, GAB1, GAPDH, GARS1, GBP1, GDAP1, GDAP2, GFPT2, GIPC1, GLUL, GOLT1B, GOT1, GPC1, GPI, GRAMD1A, GSK3B, GSTM2, GSTM3, GSTP1, GTF2F1, GTF2F2, HADH, HADHA, HDAC2, HDAC4, HGS, HHIP, HK2, HLA-A, HLA-B, HLA-C, HMGCS1, HMOX1, HNRNPA0, HNRNPH1, HPX, HSP90AA1, HSP90B1, HSPA14, HSPA4, HSPA5, HSPE1, IFIT5, IGF2BP1, IGFBP7, IKBKB, IKBKG, IMPACT, IPO5, IQGAP3, ITGA3, ITGB1, ITGB5, ITIH4, JAG1, JAGN1, KAT7, KCNMA1, KCNQ1, KDM1A, KRT18, KRT19, KRT8, LAMA5, LANCL2, LARP1, LDHA, LEMD2, LIMS1, LMNB1, LRRC32, LTBP1, LUM, MALT1, MAOA, MAP1B, MAP1LC3A, MAP2K3, MAP4K4, MAT2A, MBD3, MCM2, MCM7, ME1, MEAK7, MGMT, MGST1, MICALL1, MME, MSN, MTAP, MXRA5, MYD88, MYO1E, NCK2, NCL, NCOA5, NDUFA13, NEDD4, NEDD8, NF1, NFKB2, NIBAN2, NOCT, NPLOC4, NPM1, NPR2, NQO1, NSMCE3, NUB1, NUP35, OSBPL8, OXSR1, PAFAH1B1, PANX1, PARP1, PARP14, PCK2, PELO, PHPT1, PIGU,	

					PIK3C2A, PIK3CA, PIN1, PIP4K2B, PKLR, PKM, PKN2, PLCG1, PML, POLR2A, POLR2B, POLR2H, POLR2I, PPP3CA, PPP5C, PRKAA1, PRKCI, PSMA3, PSMB10, PSMB2, PSMC2, PSMC3, PSMC6, PSMD1, PSMD12, PSMD14, PSMD2, PSMD6, PSMD7, PSMD8, PSME1, PSME2, PSME4, PTGIS, PTK7, PTPN12, PTRH2, PXN, PYCARD, QDPR, RAB10, RAB11B, RAB8A, RALA, RANBP1, RANGAP1, RCN3, RDH11, RDX, RFC3, RGS10, RIF1, RIOK3, RIPK2, RNF40, RPL10A, RPL13A, RPL27, RPS16, SAMHD1, SARM1, SEC61A1, SEC61B, SELENOT, SERPINB2, SERPINB9, SESN2, SH3BP4, SHFL, SHMT2, SIRT1, SLC12A2, SLC1A1, SLC25A5, SLC2A1, SLC9A1, SLIT2, SMAD4, SMARCB1, SMARCC1, SMPD1, SMYD3, SNW1, SNX5, SOAT1, SOD1, SOD2, SORT1, SPARC, SPART, SPHK2, SQSTM1, SRA1, SRC, SRPRA, SRR, SRSF2, SRSF5, STAT1, STAT2, STAT5B, STRN3, STXBP3, SYNJ1, TAB2, TALDO1, TAX1BP1, TBC1D4, TBK1, TCP1, TGFBR3, TGM2, THBS1, TJP1, TOR1B, TPP1, TRA2B, TRADD, TRIM21, TRIM25, TRIM56, TSC2, TSPO, TTLL12, TWF2, TXNDC17, UBAC2, UBR2, UBTF, UBXN2A, UFD1, URI1, USP9X, USP9Y, VDAC2, VPS26B, VTN, WDR35, WFS1, XIAP, YAP1, YBX1, YES1, YOD1, ZCCHC3, ZFAND6, ZFP36L2]	
positive regulation of cellular process	GO_BiologicalProcess-EBI-UniProt-GOA-ACAP-ARAP_13.05.2021_00h00	2.40E-18	9.420901	558	[ABAT, ABCB7, ABI1, ABT1, ACACA, ACSL3, ACSL4, ACTG1, ACTR2, ACTR3, ADAM10, ADNP, AFDN, AGO2, AGPAT2, AGRN, AKAP12, AKR1B1,	No change

					ANK3, ANKFY1, ANO6, ANXA3, APAF1, APEX1, APLP2, APP, APPL2, ARAF, ARFGEF1, ARFIP1, ARHGEF11, ARHGEF12, ARID1B, ARPC4, ARPC5L, ARPIN, ARPP19, ARRB1, ASH2L, ASNS, ASPM, ASPSCR1, ATG7, ATL3, ATP1B3, ATP2C1, ATP6V0C, B2M, B3GAT3, B4GALT1, BAIAP2, BIN1, BST1, C1QBP, C2CD2L, C3orf38, CAB39L, CAMK2D, CASK, CASP3, CBR1, CBX8, CC2D1A, CCDC22, CCN2, CCNK, CCT2, CCT3, CCT6A, CD276, CD81, CDC34, CDC42EP1, CDC5L, CDC73, CDK1, CDK2, CDK5RAP3, CEMIP, CENPV, CFAP20, CFL2, CHAF1B, CHAMP1, CHD1L, CHD7, CHMP3, CHORDC1, CHP1, CHTOP, CIRBP, CLIC1, CLTCL1, CLU, CNOT1, CNOT10, COLEC12, CORO1B, CORO1C, CRABP2, CRBN, CSPG4, CTBP1, CTNNB1, CTNNBL1, CTSC, CUL4B, CUX1, CXCL12, CYCS, CYRIB, DAB2, DAGLB, DAPK1, DCN, DCPS, DCXR, DDX1, DERL1, DHPS, DHX34, DHX36, DIAPH1, DICER1, DIMT1, DIPK2A, DNM1L, DNMT1, DPYSL2, DPYSL3, DRG1, DUSP3, DVL2, DYNC1LI1, DYNLL1, ECE1, EDF1, EEF1E1, EFNB2, EHD3, EIF2AK2, EIF2S1, EIF3E, EIF6, ELP3, ELP5, ENY2, EPB41L2, EPHA2, EPHA3, EPHB4, EPM2AIP1, ERBB2, ERCC5, ETFDH, EZR, FASN, FBLN2, FBXW8, FEN1, FERMT1, FERMT2, FGD4, FGF2, FHOD1, FKBP1A, FLRT2, FMR1, FN1, FNBP1L, FSCN1, FTO, G6PD, GAB1, GAPDH, GARS1, GIPC1, GLMN, GLMP, GLUD1, GLUL, GLYR1, GNL3L,	

					GOLT1B, GOT1, GPC1, GPD1L, GPI, GPSM1, GSK3B, GSTM2, GSTP1, GTF2F1, GTF2I, H1-10, H1-5, HDAC2, HDAC4, HDGFL2, HEXB, HGS, HIP1, HLA-A, HLA-B, HMCES, HMOX1, HNRNPC, HNRNPLL, HNRNPR, HPX, HSD17B4, HSP90AA1, HSPA5, HSPE1, IFIT5, IGF2BP1, IKBKB, IKBKG, ILF3, IMPACT, IPO5, IQGAP3, IST1, ITGA3, ITGAV, ITGB1, ITM2C, ITSN1, JAG1, JAM3, KAT7, KAT8, KCNMA1, KCNQ1, KDM1A, LAMC1, LAMP1, LANCL2, LARP1, LARP4B, LARP6, LDHA, LIMS1, LLGL1, LMCD1, LMO7, LMOD1, LRCH4, LRRC8A, LRRK1, LUM, MACF1, MACROH2A1, MAD2L1, MAGI1, MALT1, MAOA, MAP1B, MAP2K3, MAP3K20, MAP3K4, MAP4K4, MARCHF5, MCAM, MEAK7, MED17, MED23, MED30, METTL14, MFF, MGMT, MLH1, MMAB, MME, MOV10, MPHOSPH8, MSH2, MSN, MTA1, MTCH1, MVD, MYADM, MYD88, MYH10, MYH9, MYO18A, MYO1E, MYO6, NCAPG, NCK2, NCKAP1, NCL, NCOA7, NDRG1, NDUFA13, NDUFA4, NECTIN2, NEDD4, NENF, NES, NF1, NF2, NFATC2IP, NFKB2, NIBAN1, NIBAN2, NLE1, NMD3, NMT1, NNT, NOCT, NOL11, NOP53, NPM1, NPR2, NQO1, NSMCE3, NSUN5, NT5C3B, NUB1, NUDT16, NUP62, NUTF2, OBSL1, OSBPL8, OXSR1, P3H1, PACSIN3, PAFAH1B1, PAFAH1B2, PAIP1, PARP1, PARP14, PATZ1, PAWR, PCK2, PDCD6IP, PELO, PFKM, PHLDB1,	

					PHPT1, PIEZO1, PIGU, PIK3C2A, PIK3CA, PIN1, PIP4K2B, PITHD1, PKM, PKN2, PLA2G4A, PLCG1, PLD1, PLPP3, PLXNB2, PML, POGZ, POLR2A, PPM1B, PPP1CA, PPP2R5A, PPP3CA, PPP5C, PRDX6, PRKAA1, PRKCI, PRKCSH, PRPF19, PSMA3, PSMB10, PSMB2, PSMC2, PSMC3, PSMC6, PSMD1, PSMD12, PSMD14, PSMD2, PSMD6, PSMD7, PSMD8, PSME1, PSME2, PSME4, PTGES2, PTGIS, PTK7, PTPA, PTRH2, PXN, PYCARD, RAB1A, RAB3D, RAB5A, RAC3, RAD21, RALA, RANBP1, RAP2B, RAP2C, RBM22, RBM4, RCN3, RDH10, RDX, RFC2, RFC3, RFC5, RHEB, RHOB, RHOC, RIF1, RIPK2, RNF2, RNF40, RNPS1, ROR1, RPA1, RPA2, RPL5, RPLP1, RPRD1B, RPS15A, RPS9, RRAS2, RTN4, RYBP, S100A10, SAAL1, SAE1, SAP30BP, SARM1, SCARB2, SCP2, SCRIB, SEC14L2, SELENOT, SEMA3C, SEPTIN7, SEPTIN9, SERPINE2, SESN2, SH3BP4, SH3KBP1, SHMT2, SIRT1, SLC12A2, SLC1A1, SLC25A12, SLC25A4, SLC25A5, SLC30A9, SLC44A2, SLC9A1, SLIT2, SMAD4, SMARCA1, SMARCB1, SMARCC1, SMARCD3, SMARCE1, SMCHD1, SMPD1, SMYD3, SNF8, SNW1, SNX5, SOAT1, SOD1, SOD2, SORBS3, SORT1, SPARC, SPHK2, SQSTM1, SRA1, SRC, SRSF5, STAT1, STAT5B, STK24, STOM, STON1, SULT1E1, SUPT16H, SVIL, SYNE2, SYNJ1, SYNPO, TAB2, TAF6L, TBC1D10A, TBK1, TCAF1, TCP1, TDRD3, TENM3, TF, TFAM, TGFBR3, TGM2, THBS1, THEM4,	

					TIGAR, TJP1, TM9SF4, TMED9, TMEM119, TNIK, TOMM7, TPBG, TPX2, TRA2A, TRA2B, TRADD, TRIM21, TRIM25, TRIM28, TRIM56, TRIO, TRIP6, TRMT10C, TSC2, TSPO, TTC5, TUT4, TWF2, TXN, UACA, UBA2, UBTF, USP22, USP5, USP7, USP9X, VASP, VDAC1, VDAC2, VLDLR, VRK3, VTN, WASHC5, WDR1, WDR35, WDR5, WFS1, WRAP73, XIAP, XPO4, YAP1, YBX1, YEATS4, YES1, ZC3HAV1, ZCCHC3, ZDHHC13, ZFP36L2, ZNF148]	
negative regulation of cellular process	GO_BiologicalProcess-EBI-UniProt-GOA-ACAP-ARAP_13.05.2021_00h00	2.40E-18	9.737099	500	[AARS1, AASS, ABAT, ABCB7, ABCE1, ABI1, ACADVL, ACOT8, ADAM10, ADAMTS9, ADARB1, ADD3, ADNP, AFDN, AGO2, AKR1B1, ALB, ANK3, ANKRD13A, ANXA5, APAF1, APBB2, APEX1, APIP, APLP2, APP, APPL2, ARAF, ARFGEF1, ARFIP1, ARHGAP12, ARPIN, ARPP19, ARRB1, ASNS, ASPM, ATG7, ATP1A1, B2M, B4GALT1, BAG1, BIN1, BNIP1, BOD1, C1QBP, CAMK2D, CAPN5, CAPZA2, CASK, CASP3, CBX1, CBX2, CBX3, CBX5, CBX8, CC2D1A, CCDC22, CCN2, CD109, CD200, CDC34, CDC5L, CDC73, CDK1, CDK2, CDK5RAP3, CEMIP, CENPV, CFDP1, CHD7, CHORDC1, CHP1, CIRBP, CLTC, CLU, CNN1, CNOT1, CNOT10, COL18A1, COL5A1, COL5A2, CORO1B, CORO1C, CORO2B, CRBN, CRTAP, CSTB, CTBP1, CTNNB1, CTSA, CTSC, CUL1, CUX1, CXCL12, CYP51A1, CYRIB, DAB2, DAPK1, DCBLD2, DCPS, DGUOK, DHCR24, DHFR, DHX34, DHX36, DIAPH1, DICER1, DIPK2A, DNM1L, DNMT1, DPYSL3, DUSP3, DVL2, DYNC1LI1, DYNLL1, EED,	No change

					EEF1E1, EFEMP1, EFNB2, EIF2AK2, EIF2S1, EIF3A, EIF3E, EIF4G3, EIF6, ENPP1, ENY2, EPHA2, EPHA3, EPPK1, EPRS1, ERBB2, ERCC5, ERLIN2, EZR, FAM3A, FASN, FERMT1, FERMT2, FGF2, FHL1, FHL2, FKBP1A, FLII, FMR1, FTH1, FTO, G6PD, GAPDH, GARS1, GATAD2A, GBE1, GBP1, GCFC2, GIPC1, GLA, GLMN, GLO1, GNL3L, GPC1, GPD1L, GPI, GPR108, GSK3B, GSTM2, GSTP1, H1-10, H1-5, HADH, HADHA, HAT1, HDAC2, HDAC4, HERC4, HGS, HHIP, HK2, HLA-A, HLA-B, HMOX1, HNRNPA0, HNRNPC, HNRNPR, HSP90B1, HSPA5, HSPB6, IDH2, IGF2BP1, IGF2BP2, IGFBP7, IKBKB, IKBKG, ILF3, IMPACT, IPO5, IREB2, ITGA3, ITGAV, ITGB1, ITIH4, ITM2C, IVNS1ABP, JAG1, JAGN1, JAM3, KAT7, KAT8, KDM1A, KDM4B, KNTC1, KRT18, LANCL2, LARP1, LARP4B, LEMD2, LIG3, LIMS1, LLGL1, LMCD1, LMOD1, LRPPRC, LRRC32, LRRFIP1, LRRK1, LTBP1, MACROH2A1, MAD1L1, MAD2L1, MAEA, MAGI1, MALT1, MAP1B, MAP2K3, MAP3K20, MAP4K4, MARCHF5, MBD3, MCM2, MCM7, MDC1, MEAK7, METTL14, MGA, MGMT, MLH1, MMAB, MORF4L1, MOV10, MPHOSPH8, MSH2, MSH6, MTA1, MVP, MYADM, MYD88, MYH9, MYO18A, NCAPG, NCK2, NCL, NCOA5, NCOA7, NDRG1, NDUFA13, NDUFS3, NEDD4, NELFCD, NENF, NES, NF1, NF2, NIBAN1, NIBAN2, NISCH, NLE1, NNT, NOCT, NOP53, NPLOC4, NPM1, NPR2, NQO1, NT5C3B, NUBP2, NUDT16, NUDT16L1, NUP62, NXN, OSBPL8, OXSR1,	

					P3H1, P3H3, PACSIN3, PAFAH1B1, PAIP1, PARP1, PARP14, PATZ1, PAWR, PBXIP1, PDS5B, PEBP1, PGP, PHPT1, PIK3CA, PIN1, PIP4K2B, PKN2, PLCG1, PLPP3, PLXNB2, PML, POLR2A, POLR2B, POLR2H, POLR2I, PPM1B, PPP1R14A, PPP2R5A, PPP3CA, PPP5C, PRKAA1, PRKCI, PRKG1, PRPF19, PSMA3, PSMB10, PSMB2, PSMC2, PSMC3, PSMC6, PSMD1, PSMD12, PSMD14, PSMD2, PSMD6, PSMD7, PSMD8, PSME1, PSME2, PSME4, PSPC1, PTBP2, PTBP3, PTGIS, PTPA, PTPN12, PTPRG, PTRH2, PUS7, PYCARD, PYCR1, QARS1, QSOX1, RAB23, RAD21, RAD9A, RANGAP1, RAP2B, RAP2C, RBBP4, RBM4, RDX, RGS10, RHOB, RIF1, RIOK3, RIPK2, RNF2, RNF213, RNF40, RNPS1, RPA2, RPL13A, RPL5, RPS13, RTN4, RYBP, SACS, SAMHD1, SARM1, SARS1, SCAI, SCFD1, SCRIB, SEMA3C, SERPINB1, SERPINB2, SERPINB8, SERPINB9, SERPINE2, SESN2, SESTD1, SET, SF1, SH3BP4, SH3KBP1, SHFL, SHMT2, SIRT1, SLC1A1, SLC25A12, SLC25A15, SLC25A4, SLC25A5, SLC9A1, SLIT2, SMAD4, SMARCB1, SMARCC1, SMARCE1, SMCHD1, SMPD1, SMYD3, SNW1, SNX5, SOD1, SOD2, SON, SORBS3, SORT1, SPARC, SPART, SPHK2, SQSTM1, SRA1, SRC, SRSF10, SRSF2, STAT1, STAT2, STAT5B, STK24, STON1, STRN3, STXBP3, SUFU, SVIL, TAB2, TAX1BP1, TBC1D4, TCAF1, TGFBI, TGFBR3, THADA, THBS1, THEM4, THOC2,	

					TIGAR, TIPRL, TJP1, TMEM119, TOM1L2, TPBG, TPX2, TRADD, TRIM21, TRIM28, TRIM59, TRIO, TRIP12, TRIP13, TSC2, TSPO, TTLL12, TUT4, TWF2, TXN, UACA, UBA2, UBA3, UBAC2, UBR2, UFD1, URI1, USP28, USP4, USP7, USP9X, UTP20, VAT1, VDAC1, VDAC2, VPS25, VRK3, VTN, WDR35, WDR5, WFS1, WWC2, WWP2, XIAP, YAP1, YBX1, YOD1, ZFAND6, ZFP36L2, ZMPSTE24, ZNF148, ZNF207]	
cellular response to chemical stimulus	GO_BiologicalProcess-EBI-UniProt-GOA-ACAP-ARAP_13.05.2021_00h00	2.40E-18	10.553191	372	[ABCC1, ABCE1, ABTB2, ACACA, ACADVL, ACAP2, ACTB, ACTG1, ACTR2, ACTR3, ADAM10, AGPAT2, AGRN, AK4, AKAP12, AKR1B1, ALB, ANK3, ANKZF1, ANO6, APAF1, APEX1, APLP2, APP, APPL2, APRT, ARFGAP1, ARID1B, ARPP19, ASAH1, ASNS, ATG7, ATP1A1, ATP2B1, ATP6V0A1, ATP6V0C, ATP6V1A, ATP6V1B2, AUP1, B2M, BAIAP2, BST1, C1QBP, C2CD2L, CAMK2D, CANX, CAPN5, CASK, CASP3, CASP6, CBX8, CCN2, CD109, CDC34, CDC5L, CDC73, CDK1, CDK16, CDK2, CDK5RAP3, CHAMP1, CLGN, CLU, CNOT1, COL4A1, COL5A2, COL6A1, COLEC12, CORO1B, CPNE2, CRYZ, CTNNB1, CUL1, CXCL12, CYB5R3, CYCS, DAB2, DAPK1, DCPS, DDI2, DERL1, DHFR, DHX34, DHX36, DIAPH1, DNM1L, DNMT1, DPYSL3, DUSP3, DYNLL1, EIF2AK2, EIF2S1, ENPP1, ENY2, EPHA2, EPHA3, EPHX1, EPRS1, ERBB2, ETFDH, EXTL2, EZR, FASN, FERMT1, FERMT2, FGF2, FIBP, FKBP1A, FLRT2, FMR1, FN1, FSCN1, FUT8, G6PD, GAB1, GAPDH,	No change

					GARS1, GBP1, GDAP1, GFPT2, GIPC1, GOLT1B, GOT1, GPC1, GPI, GRAMD1A, GSK3B, GSTM2, GSTM3, GSTP1, GTF2F1, GTF2F2, HDAC2, HDAC4, HGS, HHIP, HK2, HLA-A, HLA-B, HLA-C, HMGCS1, HMOX1, HNRNPH1, HPX, HSP90AA1, HSP90B1, HSPA14, HSPA5, IFIT5, IGF2BP1, IGFBP7, IKBKB, IKBKG, IMPACT, IPO5, IQGAP3, ITGA3, ITGB1, ITGB5, JAGN1, JAM3, KCNQ1, KDM1A, KDM3B, KRT18, KRT8, LAMA5, LANCL2, LARP1, LEMD2, LIMS1, LLGL1, LMNB1, LRRC32, LRRC8E, LTBP1, MALT1, MAOA, MAP1B, MAP1LC3A, MAP2K3, MAP4K4, MAT2A, MCM2, MCM7, MEAK7, MGMT, MGST1, MICALL1, MME, MSN, MTAP, MTR, MYD88, MYO1E, N6AMT1, NCK2, NCL, NCOA5, NCOA7, NDRG1, NDUFA13, NEDD4, NF1, NIBAN2, NNT, NOP53, NPM1, NPR2, NQO1, NSMCE3, NUP35, NXN, OSBPL8, OXSR1, PAFAH1B1, PARP1, PARP14, PAWR, PCK2, PDK1, PELO, PHPT1, PIGU, PIK3C2A, PIK3CA, PIN1, PIP4K2B, PKLR, PKM, PKN2, PLA2G4A, PLCG1, PML, POLR2A, POLR2B, POLR2H, POLR2I, PPIB, PPP3CA, PPP5C, PRDX1, PRDX6, PRKAA1, PRKCI, PRXL2B, PSMA3, PSMB10, PSMB2, PSMC2, PSMC3, PSMC6, PSMD1, PSMD12, PSMD14, PSMD2, PSMD6, PSMD7, PSMD8, PSME1, PSME2, PSME4, PTGES2, PTGIS, PTK7, PTPN12, PTRH2, PXN, PYCARD, PYCR1, QDPR, RAB10, RAB11B,	

					RAB8A, RALA, RANBP1, RANGAP1, RBM22, RDH11, RDX, RGS10, RHOB, RIF1, RIOK3, RIPK2, RPL13A, RPS16, SAMHD1, SBDS, SELENOT, SERPINB2, SERPINB9, SESN2, SH3BP4, SIRT1, SLC12A2, SLC1A1, SLC25A24, SLC25A5, SLC2A1, SLC31A1, SLC9A1, SLIT2, SMAD4, SMARCB1, SMARCC1, SMPD1, SMYD3, SNW1, SNX5, SOD1, SOD2, SORT1, SPART, SPHK2, SQSTM1, SRA1, SRC, SRPRA, SRSF5, SRXN1, STAT1, STAT2, STAT5B, STK24, STRN3, STXBP3, TAB2, TALDO1, TAX1BP1, TBC1D4, TBK1, TCP1, TF, TGFBR3, TGM2, THBS1, TIGAR, TJP1, TPBG, TPP1, TRA2B, TRADD, TRIM21, TRIM25, TSC2, TSPO, TTLL12, TWF2, TXN, TXNDC17, UBR2, UBTF, UBXN2A, UFD1, UMPS, URI1, USP9X, USP9Y, VDAC2, VPS26B, VTN, WDR35, WFS1, XIAP, YAP1, YBX1, YES1, YOD1, ZCCHC3, ZFAND6, ZFP36L2]	
cellular response to endogenous stimulus	GO_BiologicalProcess-EBI-UniProt-GOA-ACAP-ARAP_13.05.2021_00h00	2.40E-18	10.536779	159	[ABCC1, ACACA, ACAP2, ACTB, ACTR2, AKR1B1, APAF1, APEX1, APLP2, APP, APPL2, APRT, ARID1B, ARPP19, ASNS, ATG7, ATP1A1, ATP2B1, ATP6V0A1, ATP6V0C, ATP6V1A, ATP6V1B2, BAIAP2, CAPN5, CASP3, CASP6, CCN2, CD109, CDC5L, CHAMP1, CLU, CNOT1, COL4A1, COL5A2, COL6A1, CORO1B, CTNNB1, DAB2, DHX34, DIAPH1, DNMT1, DUSP3, ENPP1, EPRS1, ERBB2, EZR, FERMT1, FERMT2, FGF2, FIBP, FKBP1A, FLRT2, FUT8, GAB1, GARS1, GIPC1, GOLT1B, GOT1, GPC1, GPI, GSK3B, GSTM2, GSTP1, GTF2F1, GTF2F2, HDAC2, HDAC4,	Upregulation

					HHIP, HMGCS1, HNRNPH1, HSPA5, IGFBP7, IMPACT, IPO5, ITGA3, ITGB1, ITGB5, KCNQ1, KDM1A, LANCL2, LARP1, LEMD2, LIMS1, LRRC32, LTBP1, MAP1B, MCM7, MICALL1, MYO1E, NCL, NCOA5, NEDD4, OSBPL8, PARP1, PCK2, PELO, PIGU, PIK3C2A, PIK3CA, PIN1, PIP4K2B, PKLR, PKM, PLCG1, PML, POLR2A, POLR2B, POLR2H, POLR2I, PPP3CA, PPP5C, PRKAA1, PRKCI, PTPN12, PTRH2, PXN, RAB10, RAB8A, RANGAP1, RDX, RIPK2, SERPINB9, SESN2, SH3BP4, SIRT1, SLC1A1, SLC9A1, SLIT2, SMAD4, SMARCC1, SMC1A, SMYD3, SNW1, SNX5, SOD1, SORT1, SPART, SRA1, SRC, SRSF5, STAT1, STAT5B, STRN3, TBC1D4, TGFBR3, TGM2, THBS1, TIGAR, TSC2, UBR2, URI1, USP9X, USP9Y, VDAC2, XIAP, YAP1, YES1, ZFP36L2, ZMPSTE24]	
response to nitrogen compound	GO_BiologicalProcess-EBI-UniProt-GOA-ACAP-ARAP_13.05.2021_00h00	2.40E-18	12.418301	152	[ABAT, ABCC1, ACTB, ACTR2, AGRN, AKR1B1, ANKZF1, APEX1, APLP2, APP, APPL2, APRT, ARID1B, ARPP19, ASNS, ATG7, ATP2B1, ATP6V0A1, ATP6V0C, ATP6V1A, ATP6V1B2, AUP1, BAIAP2, CASP3, CASP6, CCN2, CD81, CDC5L, CDK1, CDK2, CHAMP1, CLU, COL4A1, COL5A2, COL6A1, COLEC12, CTNNB1, CXCL12, DDI2, DDX1, DERL1, DHFR, DHX34, DHX36, DIAPH1, DNMT1, DPYSL2, EIF2B1, EIF6, ENPP1, EPM2AIP1, EPRS1, ERLIN2, EZR, FMR1, GAB1, GOLT1B, GOT1, GPI, GSK3B, GSTM2, GSTM3, GSTP1, HADH, HADHA, HDAC2, HMGCS1, HSP90B1, HSPA5, IMPACT, IPO5, ITGB1, JAG1, KAT7, KCNQ1, KDM1A, LARP1,	Upregulation

					LDHA, MAP1B, MAP1LC3A, MEAK7, MGMT, MGST1, MTR, MYD88, MYO1E, NCOA5, NPLOC4, NPM1, NQO1, NSMCE3, OSBPL8, PANX1, PARP1, PCK2, PIGU, PIK3C2A, PIK3CA, PIP4K2B, PKLR, PKM, PPP3CA, PPP5C, PRKAA1, PRKCI, PSMB2, PSMC6, PXN, QDPR, RAB10, RAB8A, RANGAP1, RCN3, RGS10, RIOK3, RIPK2, RNF40, SEC61B, SESN2, SH3BP4, SIRT1, SLC1A1, SLC2A1, SLC9A1, SLIT2, SMARCC1, SMPD1, SNX5, SOD1, SORT1, SPARC, SRC, SRR, SRSF5, STAT1, STAT2, STAT5B, STXBP3, TBC1D4, TGFBR3, TGM2, TRIM25, TSC2, TSPO, TXN, UBAC2, UBR2, UFD1, VDAC2, WFS1, YOD1, ZCCHC3]	
response to oxygen-containing compound	GO_BiologicalProcess-EBI-UniProt-GOA-ACAP-ARAP_13.05.2021_00h00	2.40E-18	10.9349375	200	[ABAT, ABCC1, ACACA, ACTB, ACTR2, ADNP, AGRN, AKAP12, AKR1B1, ANKZF1, APEX1, APLP2, APP, APPL2, APRT, ARID1B, ARPP19, ASNS, ATG7, ATP1A1, ATP2B1, ATP6V0A1, ATP6V0C, ATP6V1A, ATP6V1B2, BAIAP2, C2CD2L, CASP3, CBX8, CCN2, CDC5L, CDC73, CDK1, CDK16, CDK2, CHAMP1, CLU, COL18A1, COL4A1, COL5A2, COL6A1, COLEC12, CTNNB1, CXCL12, DAPK1, DCN, DCPS, DHFR, DHX34, DHX36, DNM1L, DNMT1, DPYSL2, DYNLL1, EIF2B1, EIF6, ENPP1, ENY2, EPHA3, EPM2AIP1, EPRS1, EZR, G6PD, GAB1, GDAP1, GDAP2, GLUL, GOLT1B, GOT1, GPI, GRAMD1A, GSK3B, GSTP1, HADH, HADHA, HDAC2, HMGCS1, HMOX1, HNRNPA0, HSPA5, IGFBP7, IMPACT, IPO5, JAG1, JAGN1, KAT7, KCNMA1, KCNQ1, KDM1A,	No change

					KRT18, KRT8, LANCL2, LARP1, LDHA, MALT1, MAP1B, MAP1LC3A, MAP4K4, MBD3, ME1, MEAK7, MGMT, MGST1, MSN, MTAP, MTR, MYD88, MYO1E, NCOA5, NDUFA13, NFKB2, NOCT, NQO1, NUDT15, OSBPL8, OXSR1, PANX1, PARP1, PAWR, PCK2, PHPT1, PIGU, PIK3C2A, PIK3CA, PIP4K2B, PKLR, PKM, PPP3CA, PPP5C, PRDX1, PRKAA1, PRKCI, PSMD14, PTK7, PTRH2, PXN, PYCARD, PYCR1, QDPR, RAB10, RAB11B, RAB8A, RANBP1, RANGAP1, RDH11, RGS10, RHOB, RIPK2, RNF40, RPL10A, RPL27, SARM1, SELENOT, SESN2, SH3BP4, SIRT1, SLC1A1, SLC2A1, SLC9A1, SLIT2, SMARCB1, SMARCC1, SMPD1, SMYD3, SNW1, SNX5, SOD1, SOD2, SORT1, SPARC, SPHK2, SRC, SRR, SRSF2, SRSF5, STAT1, STAT2, STAT5B, STK24, STRN3, STXBP3, SYNJ1, TAB2, TBC1D4, TGFBR3, TGM2, THBS1, TJP1, TRA2B, TRIM25, TSC2, TSPO, TWF2, TXN, UBR2, UBTF, VDAC2, WDR35, YAP1, YES1]	
response to organonitrogen compound	GO_BiologicalProcess-EBI-UniProt-GOA-ACAP-ARAP_13.05.2021_00h00	2.40E-18	12.147887	138	[ABAT, ABCC1, ACTB, ACTR2, AKR1B1, ANKZF1, APEX1, APLP2, APP, APPL2, APRT, ARID1B, ARPP19, ASNS, ATG7, ATP2B1, ATP6V0A1, ATP6V0C, ATP6V1A, ATP6V1B2, AUP1, BAIAP2, CASP3, CASP6, CCN2, CD81, CDC5L, CDK1, CHAMP1, CLU, COL4A1, COL5A2, COL6A1, CTNNB1, CXCL12, DERL1, DHFR, DHX34, DIAPH1, DNMT1, DPYSL2, EIF2B1, EIF6, ENPP1, EPM2AIP1, EPRS1, ERLIN2, EZR, GAB1, GOLT1B, GOT1, GPI, GSK3B, GSTM2,	No change

					GSTP1, HADH, HADHA, HDAC2, HMGCS1, HSP90B1, HSPA5, IMPACT, IPO5, ITGB1, JAG1, KAT7, KCNQ1, KDM1A, LARP1, LDHA, MAP1B, MAP1LC3A, MEAK7, MGMT, MGST1, MYD88, MYO1E, NCOA5, NPLOC4, NQO1, OSBPL8, PANX1, PARP1, PCK2, PIGU, PIK3C2A, PIK3CA, PIP4K2B, PKLR, PKM, PPP3CA, PPP5C, PRKAA1, PRKCI, PSMB2, PSMC6, PXN, QDPR, RAB10, RAB8A, RANGAP1, RCN3, RGS10, RIPK2, RNF40, SEC61B, SESN2, SH3BP4, SIRT1, SLC1A1, SLC2A1, SLC9A1, SLIT2, SMARCC1, SMPD1, SNX5, SOD1, SORT1, SPARC, SRC, SRR, SRSF5, STAT1, STAT2, STAT5B, STXBP3, TBC1D4, TGFBR3, TGM2, TRIM25, TSC2, TSPO, UBAC2, UBR2, UFD1, VDAC2, WFS1, YOD1]	
response to cytokine	GO_BiologicalProcess-EBI-UniProt-GOA-ACAP-ARAP_13.05.2021_00h00	2.40E-18	11.40625	146	[ABCE1, ACSL4, ACTG1, ACTR2, ACTR3, ADAM10, AGPAT2, AKAP12, APLP2, APP, APPL2, ASAH1, B2M, CAMK2D, CANX, CASP3, CDC34, CDC5L, CHAMP1, CUL1, CXCL12, DAPK1, DPYSL3, EIF2AK2, EPRS1, ETFDH, FASN, FGF2, FN1, FSCN1, GAPDH, GBP1, GFPT2, GIPC1, GSK3B, GSTP1, HDAC4, HK2, HLA-A, HLA-B, HLA-C, HMOX1, HPX, HSP90AA1, HSP90B1, HSPA5, IFIT5, IGF2BP1, IKBKB, IKBKG, ITGB1, ITIH4, JAGN1, KRT18, KRT8, LAMA5, LIMS1, LMNB1, MAOA, MAT2A, MCM2, MCM7, MME, MSN, MTAP, MYD88, MYO1E, NCL, NDUFA13, NFKB2, NPR2, NUB1, NUP35, OXSR1, PAFAH1B1, PARP14, PCK2, PIK3CA, PKN2, PML, PRKCI, PSMA3, PSMB10, PSMB2,	Downregulation

					PSMC2, PSMC3, PSMC6, PSMD1, PSMD12, PSMD14, PSMD2, PSMD6, PSMD7, PSMD8, PSME1, PSME2, PSME4, PTGIS, PTPN12, PYCARD, RALA, RIF1, RIPK2, RPL13A, RPS16, SAMHD1, SEC61A1, SERPINB2, SHFL, SHMT2, SIRT1, SLC12A2, SLC1A1, SLC25A5, SLIT2, SMAD4, SMPD1, SOD1, SOD2, SPARC, SQSTM1, SRC, STAT1, STAT2, STAT5B, STXBP3, SYNJ1, TAB2, TALDO1, TAX1BP1, TBK1, TCP1, THBS1, TRADD, TRIM21, TRIM25, TRIM56, TTLL12, TXNDC17, UBXN2A, VPS26B, WDR35, YAP1, YBX1, ZFAND6, ZFP36L2]	
intracellular signal transduction	GO_BiologicalProcess-EBI-UniProt-GOA-ACAP-ARAP_13.05.2021_00h00	2.40E-18	9.748744	291	[ADNP, AKAP12, AKR1B1, AMOTL1, APAF1, APBB2, APEX1, APIP, APP, ARAF, ARFGAP1, ARFGEF1, ARHGAP12, ARHGAP17, ARHGAP5, ARHGEF11, ARHGEF12, ARPP19, ARRB1, ASB1, ATP2C1, BCR, BST1, C1QBP, CAB39L, CAMK2D, CAP1, CAP2, CASP3, CC2D1A, CCDC22, CCN2, CD81, CDC34, CDC42BPA, CDC42EP1, CDC5L, CDK1, CDK2, CDK5RAP3, CHD4, CHORDC1, CHP1, CLU, CNOT1, CNOT10, CSPG4, CTNNB1, CUL1, CXCL12, CYRIB, DAPK1, DDX1, DHCR24, DHX34, DHX36, DICER1, DIPK2A, DNM1L, DNMBP, DNMT1, DOCK6, DUSP3, DVL2, DYNC1LI1, DYNLL1, EEF1E1, EIF2AK2, EIF3A, EIF6, EPHA2, ERBB2, ETFDH, EZR, FASN, FERMT2, FGD4, FGF2, FHL2, FKBP1A, FN1, GAB1, GAPDH, GARS1, GBP1, GOLGA7, GOLT1B, GPD1L, GPI, GSK3B, GSTM2, GSTP1, HDAC2, HDAC4,	No change

					HGS, HINT1, HIP1, HMOX1, HSP90AA1, IFIT5, IKBKB, IKBKG, IQGAP3, ITGA3, ITGAV, ITGB1, ITSN1, IVNS1ABP, KAT7, KDM1A, KNTC1, KPNB1, LARP1, LEMD2, LIMS1, LLGL1, LMCD1, LRRK1, MAD1L1, MAD2L1, MALT1, MAP2K3, MAP3K20, MAP3K4, MAP4K4, MBD3, MDC1, MEAK7, MLH1, MMAB, MSH2, MSH6, MYADM, MYD88, MYO6, NCK2, NCKAP1, NDRG1, NDUFA13, NDUFS3, NEDD4, NENF, NF1, NF2, NFKB2, NISCH, NOP53, NPLOC4, NPM1, NPR2, NUCB2, NUP62, OSBPL8, OXSR1, PAFAH1B1, PARP1, PDK1, PEBP1, PIGU, PIK3C2A, PIK3CA, PIN1, PIP4K2B, PKN2, PLCB3, PLCG1, PLD1, PML, PPM1B, PPP3CA, PPP5C, PRDX1, PRKAA1, PRKAB2, PRKCI, PRKCSH, PRKG1, PRPF19, PSMA3, PSMB10, PSMB2, PSMC2, PSMC3, PSMC6, PSMD1, PSMD12, PSMD14, PSMD2, PSMD6, PSMD7, PSMD8, PSME1, PSME2, PSME4, PTGES2, PTPMT1, PTRH2, PYCARD, QARS1, RAC3, RAD9A, RAI14, RALA, RAP2B, RAP2C, RBBP4, RCN3, RDX, RFC2, RFC3, RFC5, RHEB, RHOB, RHOC, RIOK3, RIPK2, ROR1, RPA1, RPA2, RPF2, RPL5, RRAS2, RTN4, SCAI, SERPINB8, SERPINE2, SESN2, SEZ6L2, SH3BP4, SIRT1, SLC25A15, SLC44A2, SLC9A1, SLIT2, SMAD4, SMPD1, SNF8, SNW1, SOD1, SOD2, SORBS3, SPHK2, SQSTM1, SRC, SSRP1, STAT1, STK24, SUPT16H, TAB2, TBK1, TF, TGFBR3, TGM2, THBS1, THEM4, TIPRL, TJP1, TJP2, TNIK, TNS3, TPBG,	

					TPX2, TRADD, TRIM21, TRIM25, TRIM28, TRIM59, TRIO, TRIP13, TRIP6, TSC2, TTC5, TXN, UACA, UBR2, UFD1, URI1, USP28, USP7, VDAC2, VRK3, WFS1, WWC2, XIAP, YAP1, ZCCHC3, ZDHHC13, ZFAND6, ZFP36L2, ZMPSTE24, ZNF207]	
response to growth factor	GO_BiologicalProcess-EBI-UniProt-GOA-ACAP-ARAP_13.05.2021_00h00	2.40E-18	12.097812	94	[ACAP2, ANXA3, APAF1, APEX1, APLP2, APP, APPL2, BAIAP2, CAPN5, CASK, CCN2, CD109, CDC34, CDC5L, CORO1B, CTNNB1, DAB2, DUSP3, EPHA2, ERBB2, FERMT1, FERMT2, FGF2, FIBP, FKBP1A, FLRT2, FUT8, GAB1, GARS1, GIPC1, GOLT1B, GOT1, GPC1, GPI, GSTP1, GTF2F1, GTF2F2, HDAC2, HGS, HHIP, HNRNPH1, HSPA5, ITGA3, ITGB1, ITGB5, LEMD2, LIMS1, LRRC32, LTBP1, LUM, MAP1B, MAP2K3, MCM7, MICALL1, MXRA5, MYO1E, NCL, NEDD4, NF1, NIBAN2, PARP1, PELO, PIGU, PIK3CA, PIN1, PLCG1, PML, POLR2A, POLR2B, POLR2H, POLR2I, PPP3CA, PTPN12, PTRH2, PXN, RDX, SIRT1, SLIT2, SMAD4, SNW1, SORT1, SPART, SRC, STAT5B, TGFBR3, THBS1, TWF2, URI1, USP9X, USP9Y, VTN, XIAP, YES1, ZFP36L2]	Upregulation
cellular response to organic substance	GO_BiologicalProcess-EBI-UniProt-GOA-ACAP-ARAP_13.05.2021_00h00	2.40E-18	10.6065855	306	[ABCC1, ABCE1, ACACA, ACADVL, ACAP2, ACTB, ACTG1, ACTR2, ACTR3, AGPAT2, AKAP12, AKR1B1, APAF1, APEX1, APLP2, APP, APPL2, APRT, ARFGAP1, ARID1B, ARPP19, ASAH1, ASNS, ATG7, ATP1A1, ATP2B1, ATP6V0A1, ATP6V0C, ATP6V1A, ATP6V1B2, AUP1, B2M, BAIAP2, C2CD2L, CAMK2D, CANX, CAPN5, CASK, CASP3, CASP6, CCN2, CD109, CDC34, CDC5L, CDC73, CDK16, CDK5RAP3,	No change

					CHAMP1, CLGN, CLU, CNOT1, COL4A1, COL5A2, COL6A1, COLEC12, CORO1B, CTNNB1, CUL1, CXCL12, DAB2, DAPK1, DCPS, DERL1, DHX34, DIAPH1, DNM1L, DNMT1, DPYSL3, DUSP3, DYNLL1, EIF2AK2, EIF2S1, ENPP1, ENY2, EPHA3, EPRS1, ERBB2, ETFDH, EXTL2, EZR, FASN, FERMT1, FERMT2, FGF2, FIBP, FKBP1A, FLRT2, FN1, FSCN1, FUT8, GAB1, GAPDH, GARS1, GBP1, GDAP1, GFPT2, GIPC1, GOLT1B, GOT1, GPC1, GPI, GRAMD1A, GSK3B, GSTM2, GSTP1, GTF2F1, GTF2F2, HDAC2, HDAC4, HGS, HHIP, HK2, HLA-A, HLA-B, HLA-C, HMGCS1, HMOX1, HNRNPH1, HPX, HSP90AA1, HSP90B1, HSPA14, HSPA5, IFIT5, IGF2BP1, IGFBP7, IKBKB, IKBKG, IMPACT, IPO5, IQGAP3, ITGA3, ITGB1, ITGB5, JAGN1, KCNQ1, KDM1A, KRT18, KRT8, LAMA5, LANCL2, LARP1, LEMD2, LIMS1, LMNB1, LRRC32, LTBP1, MALT1, MAOA, MAP1B, MAP2K3, MAP4K4, MAT2A, MCM2, MCM7, MGMT, MGST1, MICALL1, MME, MSN, MTAP, MYD88, MYO1E, NCK2, NCL, NCOA5, NDUFA13, NEDD4, NF1, NIBAN2, NPM1, NPR2, NUP35, OSBPL8, OXSR1, PAFAH1B1, PARP1, PARP14, PCK2, PELO, PHPT1, PIGU, PIK3C2A, PIK3CA, PIN1, PIP4K2B, PKLR, PKM, PKN2, PLCG1, PML, POLR2A, POLR2B, POLR2H, POLR2I, PPP3CA, PPP5C, PRKAA1, PRKCI, PSMA3, PSMB10, PSMB2, PSMC2, PSMC3, PSMC6, PSMD1, PSMD12, PSMD14, PSMD2, PSMD6,	

					PSMD7, PSMD8, PSME1, PSME2, PSME4, PTGIS, PTK7, PTPN12, PTRH2, PXN, PYCARD, RAB10, RAB11B, RAB8A, RALA, RANBP1, RANGAP1, RDX, RIF1, RIOK3, RIPK2, RPL13A, RPS16, SAMHD1, SELENOT, SERPINB2, SERPINB9, SESN2, SH3BP4, SIRT1, SLC12A2, SLC1A1, SLC25A5, SLC9A1, SLIT2, SMAD4, SMARCB1, SMARCC1, SMYD3, SNW1, SNX5, SOD1, SOD2, SORT1, SPART, SPHK2, SQSTM1, SRA1, SRC, SRPRA, SRSF5, STAT1, STAT2, STAT5B, STRN3, STXBP3, TAB2, TALDO1, TAX1BP1, TBC1D4, TBK1, TCP1, TGFBR3, TGM2, THBS1, TJP1, TPP1, TRA2B, TRADD, TRIM21, TRIM25, TSC2, TSPO, TTLL12, TWF2, TXNDC17, UBR2, UBTF, UBXN2A, UFD1, URI1, USP9X, USP9Y, VDAC2, VPS26B, VTN, WDR35, WFS1, XIAP, YAP1, YBX1, YES1, YOD1, ZCCHC3, ZFAND6, ZFP36L2]	
cellular response to nitrogen compound	GO_BiologicalProcess-EBI-UniProt-GOA-ACAP-ARAP_13.05.2021_00h00	2.40E-18	12.287581	94	[ABCC1, ACTB, ACTR2, AGRN, AKR1B1, APEX1, APLP2, APP, APPL2, APRT, ARID1B, ARPP19, ATG7, ATP2B1, ATP6V0A1, ATP6V0C, ATP6V1A, ATP6V1B2, BAIAP2, CASP3, CASP6, CDC5L, CDK2, CHAMP1, CLU, COL4A1, COL5A2, COL6A1, COLEC12, CTNNB1, DDI2, DHX34, DIAPH1, DNMT1, ENPP1, EPRS1, EZR, FMR1, GAB1, GOLT1B, GOT1, GSK3B, GSTM2, GSTP1, HDAC2, HSPA5, IMPACT, IPO5, KCNQ1, KDM1A, LARP1, MAP1B, MTR, MYO1E, NCOA5, NPM1, NSMCE3, OSBPL8, PARP1, PCK2, PIGU, PIK3C2A, PIK3CA, PIP4K2B, PKLR, PKM,	Upregulation

					PRKAA1, PRKCI, PXN, RAB10, RAB8A, RANGAP1, RGS10, RIOK3, RIPK2, SESN2, SH3BP4, SIRT1, SLC1A1, SLC9A1, SLIT2, SMARCC1, SNX5, SOD1, SRC, SRSF5, STAT1, STAT5B, TBC1D4, TGM2, TSC2, UBR2, VDAC2, ZCCHC3]	
cellular response to oxygen-containing compound	GO_BiologicalProcess-EBI-UniProt-GOA-ACAP-ARAP_13.05.2021_00h00	2.40E-18	10.783553	139	[ABCC1, ACACA, ACTB, ACTR2, AGRN, AKR1B1, ANKZF1, APEX1, APLP2, APP, APPL2, APRT, ARID1B, ARPP19, ATP2B1, ATP6V0A1, ATP6V0C, ATP6V1A, ATP6V1B2, BAIAP2, C2CD2L, CBX8, CDC5L, CDC73, CDK1, CDK16, CDK2, CHAMP1, CLU, COL4A1, COL5A2, COL6A1, COLEC12, CTNNB1, DAPK1, DCPS, DHFR, DHX34, DHX36, DNM1L, DNMT1, DYNLL1, ENPP1, ENY2, EPHA3, EPRS1, EZR, GAB1, GDAP1, GOLT1B, GOT1, GRAMD1A, GSK3B, GSTP1, HDAC2, HMGCS1, HSPA5, IMPACT, IPO5, JAGN1, KCNQ1, KDM1A, LANCL2, LARP1, MALT1, MAP1B, MAP1LC3A, MAP4K4, MGST1, MSN, MTR, MYD88, MYO1E, NCOA5, NDUFA13, NQO1, OSBPL8, OXSR1, PARP1, PAWR, PCK2, PHPT1, PIGU, PIK3C2A, PIK3CA, PIP4K2B, PKLR, PKM, PPP3CA, PPP5C, PRDX1, PRKAA1, PRKCI, PTK7, PXN, PYCARD, PYCR1, RAB10, RAB11B, RAB8A, RANBP1, RANGAP1, RDH11, RGS10, RHOB, RIPK2, SELENOT, SESN2, SH3BP4, SIRT1, SLC1A1, SLC9A1, SLIT2, SMARCB1, SMARCC1, SMYD3, SNW1, SNX5, SOD1, SOD2, SPHK2, SRC, SRSF5, STAT1, STAT5B, TBC1D4, TGM2, TJP1, TRA2B, TSC2, TSPO, TWF2, TXN, UBR2, UBTF, VDAC2, WDR35, YAP1, YES1]	Upregulation

response to peptide	GO_BiologicalProcess-EBI-UniProt-GOA-ACAP-ARAP_13.05.2021_00h00	2.40E-18	12.478336	72	[ABCC1, AKR1B1, APEX1, APLP2, APP, APPL2, APRT, ARID1B, ATP2B1, ATP6V0A1, ATP6V0C, ATP6V1A, ATP6V1B2, BAIAP2, CCN2, CDC5L, CXCL12, EIF2B1, EIF6, ENPP1, EPM2AIP1, EPRS1, GAB1, GOLT1B, GOT1, GSK3B, GSTP1, HADH, HADHA, JAG1, KAT7, KCNQ1, MAP1B, MEAK7, MYO1E, NCOA5, OSBPL8, PARP1, PCK2, PIGU, PIK3C2A, PIK3CA, PIP4K2B, PKLR, PKM, PRKAA1, PRKCI, PXN, QDPR, RAB10, RAB8A, RANGAP1, RIPK2, RNF40, SESN2, SIRT1, SLC2A1, SLC9A1, SMARCC1, SNX5, SORT1, SPARC, SRC, SRSF5, STAT1, STAT2, STAT5B, STXBP3, TBC1D4, TGFBR3, TSC2, VDAC2]	Upregulation
response to peptide hormone	GO_BiologicalProcess-EBI-UniProt-GOA-ACAP-ARAP_13.05.2021_00h00	2.40E-18	13.5245905	66	[APEX1, APLP2, APPL2, APRT, ARID1B, ATP2B1, ATP6V0A1, ATP6V0C, ATP6V1A, ATP6V1B2, BAIAP2, CCN2, CDC5L, CXCL12, EIF2B1, EIF6, ENPP1, EPM2AIP1, EPRS1, GAB1, GOLT1B, GOT1, GSK3B, GSTP1, HADH, HADHA, KCNQ1, MAP1B, MEAK7, MYO1E, NCOA5, OSBPL8, PARP1, PCK2, PIGU, PIK3C2A, PIK3CA, PIP4K2B, PKLR, PKM, PRKAA1, PRKCI, PXN, QDPR, RAB10, RAB8A, RANGAP1, RNF40, SESN2, SIRT1, SLC2A1, SLC9A1, SMARCC1, SNX5, SORT1, SPARC, SRC, SRSF5, STAT1, STAT2, STAT5B, STXBP3, TBC1D4, TGFBR3, TSC2, VDAC2]	Upregulation
cellular response to cytokine stimulus	GO_BiologicalProcess-EBI-UniProt-GOA-ACAP-ARAP_13.05.2021_00h00	2.40E-18	11.036225	131	[ABCE1, ACTG1, ACTR2, ACTR3, AGPAT2, AKAP12, APLP2, APPL2, ASAH1, B2M, CAMK2D, CANX, CASP3, CDC34, CDC5L, CHAMP1, CUL1, CXCL12,	Downregulation

					DAPK1, DPYSL3, EPRS1, ETFDH, FASN, FGF2, FN1, FSCN1, GAPDH, GBP1, GFPT2, GIPC1, GSK3B, GSTP1, HDAC4, HK2, HLA-A, HLA-B, HLA-C, HMOX1, HPX, HSP90AA1, HSP90B1, HSPA5, IFIT5, IGF2BP1, IKBKB, IKBKG, ITGB1, JAGN1, KRT18, KRT8, LAMA5, LIMS1, LMNB1, MAOA, MAT2A, MCM2, MCM7, MME, MSN, MTAP, MYD88, MYO1E, NCL, NDUFA13, NPR2, NUP35, OXSR1, PAFAH1B1, PARP14, PCK2, PIK3CA, PKN2, PML, PSMA3, PSMB10, PSMB2, PSMC2, PSMC3, PSMC6, PSMD1, PSMD12, PSMD14, PSMD2, PSMD6, PSMD7, PSMD8, PSME1, PSME2, PSME4, PTGIS, PTPN12, PYCARD, RALA, RIF1, RIPK2, RPL13A, RPS16, SAMHD1, SERPINB2, SIRT1, SLC12A2, SLC1A1, SLC25A5, SLIT2, SMAD4, SOD1, SOD2, SQSTM1, SRC, STAT1, STAT2, STAT5B, STXBP3, TAB2, TALDO1, TAX1BP1, TBK1, TCP1, THBS1, TRADD, TRIM21, TRIM25, TTLL12, TXNDC17, UBXN2A, VPS26B, WDR35, YAP1, YBX1, ZFAND6, ZFP36L2]	
cellular response to growth factor stimulus	GO_BiologicalProcess-EBI-UniProt-GOA-ACAP-ARAP_13.05.2021_00h00	2.40E-18	11.81208	88	[ACAP2, APAF1, APEX1, APLP2, APP, APPL2, BAIAP2, CAPN5, CASK, CCN2, CD109, CDC5L, CORO1B, CTNNB1, DAB2, DUSP3, ERBB2, FERMT1, FERMT2, FGF2, FIBP, FKBP1A, FLRT2, FUT8, GAB1, GARS1, GIPC1, GOLT1B, GOT1, GPC1, GPI, GSTP1, GTF2F1, GTF2F2, HDAC2, HGS, HHIP, HNRNPH1, HSPA5, ITGA3, ITGB1, ITGB5, LEMD2, LIMS1, LRRC32, LTBP1, MAP1B, MAP2K3, MCM7, MICALL1,	Upregulation

					MYO1E, NCL, NEDD4, NF1, NIBAN2, PARP1, PELO, PIGU, PIK3CA, PIN1, PLCG1, PML, POLR2A, POLR2B, POLR2H, POLR2I, PPP3CA, PTPN12, PXN, RDX, SIRT1, SLIT2, SMAD4, SNW1, SORT1, SPART, SRC, STAT5B, TGFBR3, THBS1, TWF2, URI1, USP9X, USP9Y, VTN, XIAP, YES1, ZFP36L2]	
regulation of intracellular signal transduction	GO_BiologicalProcess-EBI-UniProt-GOA-ACAP-ARAP_13.05.2021_00h00	2.40E-18	10.280374	198	[AKAP12, AKR1B1, APIP, APP, ARAF, ARFGAP1, ARFGEF1, ARHGAP12, ARHGAP17, ARHGAP5, ARHGEF11, ARHGEF12, ARPP19, ARRB1, ATP2C1, BCR, BST1, C1QBP, CC2D1A, CCDC22, CCN2, CD81, CDC34, CDK1, CDK2, CDK5RAP3, CHD4, CHORDC1, CHP1, CLU, CSPG4, CTNNB1, CXCL12, CYRIB, DDX1, DHX36, DIPK2A, DNM1L, DUSP3, DVL2, DYNC1LI1, DYNLL1, EEF1E1, EIF2AK2, EIF3A, EIF6, EPHA2, ERBB2, ETFDH, EZR, FASN, FERMT2, FGD4, FGF2, FHL2, FKBP1A, FN1, GAB1, GAPDH, GBP1, GOLT1B, GPD1L, GSK3B, GSTP1, HDAC2, HIP1, HMOX1, HSP90AA1, IFIT5, IKBKB, IKBKG, IQGAP3, ITGA3, ITGAV, ITGB1, ITSN1, IVNS1ABP, KDM1A, LEMD2, LIMS1, LMCD1, LRRK1, MAD2L1, MALT1, MAP2K3, MAP3K20, MAP3K4, MAP4K4, MBD3, MMAB, MYADM, MYD88, NCK2, NDUFA13, NDUFS3, NEDD4, NENF, NF1, NF2, NOP53, NPLOC4, NPM1, NPR2, NUP62, OSBPL8, PAFAH1B1, PARP1, PEBP1, PIGU, PIK3CA, PIN1, PIP4K2B, PKN2, PML, PPM1B, PPP3CA, PPP5C, PRDX1, PRKAA1, PRKAB2, PTGES2, PTPMT1, PYCARD, QARS1, RAC3, RAD9A, RAI14,	No change

					RBBP4, RCN3, RDX, RFC2, RFC3, RFC5, RHEB, RHOB, RHOC, RIOK3, RIPK2, ROR1, RPA1, RPA2, RPF2, RPL5, RTN4, SCAI, SERPINE2, SESN2, SEZ6L2, SH3BP4, SIRT1, SLC44A2, SLC9A1, SLIT2, SMAD4, SMPD1, SOD1, SOD2, SORBS3, SPHK2, SQSTM1, SRC, SSRP1, STAT1, STK24, SUPT16H, TAB2, TBK1, TF, TGFBR3, TGM2, THBS1, THEM4, TNIK, TPBG, TPX2, TRADD, TRIM21, TRIM25, TRIM59, TRIO, TRIP6, TSC2, TTC5, TXN, UACA, UBR2, UFD1, URI1, USP7, VDAC2, VRK3, WFS1, WWC2, XIAP, ZCCHC3, ZDHHC13, ZFAND6, ZMPSTE24]	
response to insulin	GO_BiologicalProcess-EBI-UniProt-GOA-ACAP-ARAP_13.05.2021_00h00	2.40E-18	15.873015	50	[APLP2, APPL2, APRT, ATP2B1, ATP6V0A1, ATP6V0C, ATP6V1A, ATP6V1B2, BAIAP2, EIF6, ENPP1, EPM2AIP1, EPRS1, GAB1, GOLT1B, GOT1, GSK3B, GSTP1, HADH, HADHA, KCNQ1, MEAK7, MYO1E, NCOA5, OSBPL8, PARP1, PCK2, PIGU, PIK3C2A, PIK3CA, PIP4K2B, PKLR, PKM, PRKAA1, PRKCI, RAB10, RAB8A, SESN2, SIRT1, SLC2A1, SLC9A1, SMARCC1, SNX5, SORT1, SRC, SRSF5, STAT1, STXBP3, TBC1D4, TSC2]	Upregulation
cellular response to insulin stimulus	GO_BiologicalProcess-EBI-UniProt-GOA-ACAP-ARAP_13.05.2021_00h00	2.40E-18	16.194332	40	[APLP2, APPL2, APRT, ATP2B1, ATP6V0A1, ATP6V0C, ATP6V1A, ATP6V1B2, BAIAP2, ENPP1, EPRS1, GAB1, GOLT1B, GOT1, GSK3B, GSTP1, MYO1E, NCOA5, OSBPL8, PARP1, PCK2, PIGU, PIK3C2A, PIK3CA, PIP4K2B, PKLR, PKM, PRKAA1, PRKCI, RAB10, RAB8A, SIRT1, SLC9A1, SMARCC1, SNX5, SRC, SRSF5, STAT1, TBC1D4, TSC2]	Upregulation
multicellular organism development	GO_BiologicalProcess-EBI-UniProt-GOA-	2.32E-09	8.7545595	504	[AARS1, ABAT, ABI1, ACADM, ACO1, ACSL3, ACSL4, ACTB,	No change

	ACAP-ARAP_13.05.2021_00h00				ACTG1, ACTR2, ADAM10, ADAMTS9, ADARB1, ADNP, AGO2, AGRN, AK4, AKR1B1, ALCAM, ALDH3A2, AMOTL1, ANK3, ANKH, ANO6, ANXA3, APAF1, APEX1, APLP2, APP, APPL2, APRT, ARFGEF1, ARHGAP5, ARID1B, ARRB1, ASAH1, ASB1, ASH2L, ASNS, ASPM, ATP2B1, ATP2B2, ATXN10, B2M, B4GALT1, B4GALT5, BAIAP2, BCR, BIN1, BMP1, BPNT2, C1GALT1, C1QBP, CALD1, CASP3, CBX2, CCN2, CD109, CD81, CDC73, CDH11, CDK1, CDK16, CDK2, CDK5RAP3, CECR2, CFAP20, CFDP1, CFL2, CHAMP1, CHD2, CHD7, CHTOP, CKB, CLTC, CLU, CNGB1, CNOT1, COL11A1, COL12A1, COL15A1, COL18A1, COL4A1, COL5A1, COL5A2, COL6A1, COLEC12, CORO1C, CRABP2, CSPG4, CSRP2, CTNNB1, CTNNBL1, CTSC, CUL1, CUL4B, CUX1, CXCL12, CYP51A1, DAB2, DAGLB, DCN, DDX1, DGUOK, DHCR24, DHFR, DHX36, DICER1, DIPK2A, DNM1L, DNMT1, DPCD, DPYSL2, DPYSL3, DPYSL4, DRG1, DSP, DVL2, DYM, DYNLL1, ECE1, EDF1, EED, EFEMP1, EFNB2, EIF2AK2, EIF2B1, EIF6, ELP3, EMG1, EML1, ENPP1, EPB41L3, EPHA2, EPHA3, EPHB4, ERAP1, ERBB2, EZR, FAM3A, FARP1, FASN, FAT1, FBXW8, FERMT1, FERMT2, FGF2, FHL1, FHL2, FKBP1A, FLII, FLRT2, FMR1, FN1, FTO, FUT8, G6PD, GAB1, GAMT, GARS1, GBA2, GLMN, GLO1, GLUD1, GLUL, GNB4, GPC1, GPC6, GPI, GPSM1, GSK3B, GSTM3, GSTP1, GYS1, HDAC2, HDAC4, HEXB, HGS, HHIP, HK2, HLA-B, HMCES, HMGCS1, HMOX1, HSD17B4,	

					HSP90AA1, HSPA5, HSPB11, HSPB6, HSPG2, IDH2, IFT140, IGF2BP1, IGF2BP2, IGFBP7, IKBKB, IMPACT, IQGAP3, IREB2, IST1, ITGA3, ITGAV, ITGB1, ITGB5, ITM2C, ITPK1, ITSN1, JAG1, JAGN1, JAM3, KAT7, KAT8, KCNQ1, KCTD15, KDM1A, KRT18, KRT19, KRT8, LAMA5, LAMC1, LBR, LDHA, LEMD2, LIG1, LIG3, LIMS1, LIPA, LLGL1, LRCH4, LRP10, LRRC8A, LRRK1, LUM, LYPLA2, MACF1, MACROH2A1, MAD1L1, MAEA, MALT1, MAP1B, MAP3K20, MAP3K4, MAP4K4, MATR3, MBD3, MBOAT2, MBOAT7, MCAM, MCM2, MCM7, METTL14, MGAT1, MGMT, MGST1, MICALL1, MLF2, MLH1, MMAB, MME, MMUT, MOV10, MPST, MSH2, MSH6, MSN, MTCH1, MTR, MYADM, MYH9, MYO1D, MYO1E, MYO6, NCAPG, NCDN, NCKAP1, NCL, NDRG1, NDUFS3, NEDD4, NES, NF1, NF2, NFKB2, NIBAN2, NLE1, NMT1, NOCT, NOM1, NPR2, NSDHL, NSUN5, NXN, OBSL1, OXSR1, P3H1, PAFAH1B1, PAFAH1B2, PAFAH1B3, PALLD, PAPSS2, PARP1, PATZ1, PBXIP1, PCK2, PELO, PGM3, PHGDH, PHLDB1, PIGT, PIGU, PIK3C2A, PIK3CA, PIN1, PIR, PITHD1, PKM, PKN2, PLCG1, PLXNB2, PML, POFUT1, POMGNT2, POMK, PPIB, PPP1CA, PPP3CA, PRDX1, PRDX6, PRKAA1, PRKCI, PRKCSH, PRKG1, PRPF19, PRUNE1, PSMA3, PSMB10, PSMB2, PSMC2, PSMC3, PSMC6, PSMD1, PSMD12, PSMD14, PSMD2, PSMD6, PSMD7, PSMD8, PSME1, PSME2,	

					PSME4, PSMG1, PTBP3, PTGIS, PTK7, PTPRF, PTPRG, PTRH2, PUS7, QARS1, QDPR, RAB10, RAB23, RAB8A, RAC3, RAD21, RALA, RANBP1, RCN3, RDH10, RHEB, RHOB, RIC8A, RIF1, RIPK2, RNF114, RNF2, RNF213, ROR1, RPL13, RPL22, RPL24, RPL38, RPS16, RPS4Y1, RRM1, RTN4, RTTN, RYBP, SAMHD1, SARM1, SARS1, SBDS, SCARB2, SCRIB, SEC61A1, SELENOT, SEMA3C, SEPTIN2, SEPTIN7, SERPINE2, SEZ6L2, SF1, SF3B6, SH3KBP1, SIRT1, SLC11A2, SLC12A2, SLC1A1, SLC25A12, SLC25A5, SLC2A1, SLC4A7, SLC9A1, SLIT2, SMAD4, SMARCA1, SMARCB1, SMARCC1, SMARCD3, SMARCE1, SMCHD1, SMPD1, SNW1, SOAT1, SOD1, SOD2, SORT1, SPARC, SPART, SPHK2, SPTLC2, SRC, SRR, SRSF5, STAT1, STAT5B, STK24, STXBP3, SUFU, SVIL, SYNE2, SYNJ1, SYPL2, TAB2, TAGLN, TENM3, TF, TGFBI, TGFBR3, TGM2, THBS1, THBS3, THOC2, TIGAR, TJP1, TMEM119, TMEM65, TNIK, TPBG, TPI1, TPP1, TRA2B, TRADD, TRAPPC4, TRIM28, TRIO, TRIP12, TRIP6, TSC2, TSPO, TTC39C, TUBB3, TUBGCP2, TWF2, TYMP, UBA2, UBE2Q1, UFD1, UGP2, UMPS, UNC119B, USP22, USP7, USP9X, UST, UTP3, VASP, VDAC2, VLDLR, VTN, WASHC5, WDR1, WDR11, WDR35, WDR5, WFS1, YAP1, YBX1, YTHDC1, ZDHHC21, ZFP36L2, ZMPSTE24, ZMYM3, ZNF148]	
anatomical structure morphogenesis	GO_BiologicalProcess-EBI-UniProt-GOA-	2.32E-09	10.242872	291	[ABI1, ACTB, ACTG1, ACTR2, ADAM10, ADAMTS9, ADARB1,	No change

	ACAP-ARAP_13.05.2021_00h00				ADNP, AGO2, AGRN, AKR1B1, ALCAM, ALDOA, AMOTL1, ANK3, ANXA3, APP, ARHGAP12, ARPIN, B4GALT1, B4GALT5, BAIAP2, BMP1, BPNT2, C1GALT1, C1QBP, CALD1, CAP1, CAP2, CASP3, CCN2, CD109, CD81, CDC42EP1, CDC73, CDH11, CECR2, CFDP1, CFL2, CHAMP1, CHD7, CLTC, CLTCL1, CLU, CNOT1, COL11A1, COL12A1, COL15A1, COL18A1, COL4A1, COL5A1, COL5A2, COL6A1, CORO1B, CORO1C, CRABP2, CSPG4, CSRP2, CSTB, CTNNB1, CUL1, CUX1, CXCL12, CYRIB, DAB2, DCN, DHX36, DIAPH1, DICER1, DNM1L, DNMBP, DPYSL2, DPYSL4, DSP, DVL2, DYNLL1, ECE1, EFEMP1, EFNB2, EPB41L3, EPHA2, EPHA3, EPHB4, ERAP1, ERBB2, EZR, FAM171A1, FAM3A, FARP1, FAT1, FBLIM1, FBXW8, FERMT1, FERMT2, FGD4, FGF2, FHL1, FHL2, FKBP1A, FLII, FLRT2, FN1, GAB1, GAMT, GARS1, GBP1, GLMN, GLUL, GPC1, GPC6, GPI, GSK3B, HDAC2, HEXB, HGS, HHIP, HK2, HMOX1, HSP90AA1, HSPA5, HSPB6, HSPG2, IFT140, IGF2BP1, IGF2BP2, IMPACT, IST1, ITGA3, ITGAV, ITGB1, ITGB5, JAG1, JAM3, KRT18, KRT19, KRT8, LAMA5, LAMC1, LEMD2, LIG1, LIMS1, LIN7C, LIPA, LLGL1, LMOD1, LYPLA2, MACF1, MACROH2A1, MAP1B, MAP3K20, MAP4K4, MARCHF5, MBOAT7, MCAM, MFF, MLF2, MOV10, MSN, MYADM, MYH10, MYH9, MYO1E, MYO6, MYOF, NCKAP1, NCL, NECTIN2, NEDD4, NEDD8, NES, NF1, NF2, NFKB2, NIBAN2, NLE1, NUBPL, NUDT19,	

					OBSL1, OXSR1, PAFAH1B1, PALLD, PHLDB1, PIGU, PIK3C2A, PIK3CA, PKM, PKN2, PLCG1, PLXNB2, PML, POFUT1, PPP1CA, PPP3CA, PRKCI, PSMA3, PSMB10, PSMB2, PSMC2, PSMC3, PSMC6, PSMD1, PSMD12, PSMD14, PSMD2, PSMD6, PSMD7, PSMD8, PSME1, PSME2, PSME4, PTBP3, PTGIS, PTK7, PXN, RAB10, RAB1A, RAB23, RAB8A, RAC3, RALA, RDH10, RDX, RHOB, RHOC, RIC8A, RIPK2, RNF2, RNF213, ROR1, RPL24, RPL38, RTN4, S100A10, SARM1, SARS1, SCFD1, SCRIB, SEMA3C, SEPTIN7, SERPINB8, SERPINE2, SF3B6, SH3KBP1, SIRT1, SLC11A2, SLC12A2, SLC1A1, SLIT2, SMAD4, SMARCC1, SMARCD3, SNX1, SOD1, SPARC, SPART, SRC, STAT1, STAT2, STK24, SUFU, TBPL1, TENM3, TGFBI, TGFBR3, TGM2, THBS1, THBS3, TJP1, TMEM119, TNIK, TPBG, TRIM28, TRIO, TSC2, TTC39C, TUBB3, TWF2, TYMP, UBA2, USP9X, UST, VASP, VAT1, VDAC2, VLDLR, VTN, WDR1, YAP1, YBX1, ZMPSTE24, ZMYM3]	
regulation of developmental process	GO_BiologicalProcess-EBI-UniProt-GOA-ACAP-ARAP_13.05.2021_00h00	2.32E-09	9.721147	251	[ACTR2, ADAM10, ADAMTS9, ADNP, AGO2, AGRN, AKR1B1, ALDOA, ANKH, ANO6, ANXA3, APEX1, APLP2, APP, APPL2, ARPIN, ASH2L, ASPM, ATP2B1, B2M, B4GALT5, BAIAP2, BIN1, C1QBP, CCN2, CD109, CDC42EP1, CDC73, CDK1, CDK5RAP3, CFDP1, CHAMP1, CHD7, CLIC1, CLTC, CNOT1, COL5A1, COL5A2, CORO1B, CORO1C, CRABP2, CTNNB1, CTSC, CUX1, CXCL12, CYRIB, DAB2, DHX36, DIAPH1, DICER1, DNM1L, DNMBP, DNMT1, DPYSL2,	No change

					DVL2, DYNLL1, EEF1E1, EFEMP1, EFNB2, EIF2AK2, EIF6, ENPP1, EPB41L3, EPHA2, EPHA3, EPPK1, ERAP1, ERBB2, EZR, FAM171A1, FBLIM1, FBXW8, FERMT1, FERMT2, FGD4, FGF2, FLRT2, FMR1, FN1, FTO, G6PD, GAB1, GAMT, GARS1, GBP1, GLUL, GPC1, GPC6, GSK3B, HDAC2, HDAC4, HEXB, HGS, HK2, HLA-B, HMCES, HMOX1, HSPB6, HSPG2, IDH2, IKBKB, IMPACT, IQGAP3, IST1, ITGAV, ITGB1, ITSN1, JAG1, KAT7, KDM1A, LAMA5, LIMS1, LLGL1, LRCH4, LRRC8A, MACF1, MACROH2A1, MALT1, MAP1B, MAP4K4, MARCHF5, MBOAT2, METTL14, MFF, MLF2, MLH1, MME, MOV10, MSH2, MSN, MYADM, MYH10, MYH9, NEDD4, NF1, NF2, NIBAN2, NOCT, NPR2, NSUN5, OBSL1, OXSR1, PAFAH1B1, PARP1, PAWR, PHLDB1, PIEZO1, PIGU, PIK3CA, PIN1, PITHD1, PKM, PKN2, PLCG1, PLXNB2, PML, PPIB, PPP3CA, PRKAA1, PRKCI, PRPF19, PRUNE1, PSMA3, PSMB10, PSMB2, PSMC2, PSMC3, PSMC6, PSMD1, PSMD12, PSMD14, PSMD2, PSMD6, PSMD7, PSMD8, PSME1, PSME2, PSME4, PTBP3, PTGIS, PTK7, PTPRF, PTRH2, PUS7, RAC3, RALA, RBM4, RDX, RHEB, RHOB, RHOC, RIF1, RIPK2, ROR1, RTN4, S100A10, SARM1, SARS1, SEMA3C, SEPTIN7, SERPINE2, SH3KBP1, SIRT1, SLC12A2, SLC25A12, SLIT2, SMAD4, SMARCD3, SNW1, SOD1, SOD2, SORT1, SPARC, SPART, SRA1, SRC, STAT1, STAT2, STAT5B, STK24, SUFU, SULT1E1, SYNJ1, TAF6L,	

					TGFBR3, TGM2, THBS1, TJP1, TMEM119, TNIK, TPBG, TRADD, TRIO, TRIP12, TSPO, TWF2, TYMP, UBA2, UST, VAT1, VDAC2, VLDLR, WDR1, WDR5, WWC2, YAP1, YBX1, ZDHHC21, ZFP36L2, ZMPSTE24, ZMYM3]	
tissue development	GO_BiologicalProcess-EBI-UniProt-GOA-ACAP-ARAP_13.05.2021_00h00	2.32E-09	10.234742	218	[ABI1, ACADM, ACADVL, ACTB, ACTG1, ADAMTS9, ADARB1, AFDN, AGPAT2, AGRN, AKR1B1, ALDH3A2, ANKH, ANO6, ANXA7, ARHGAP12, ASAH1, ATP2B1, ATP2C1, B4GALT1, BCR, BDH2, BMP1, BPNT2, CASP3, CASP6, CBR1, CCN2, CD109, CDC73, CDK1, CECR2, CFL2, CHD7, CLTC, CNN3, COL11A1, COL12A1, COL15A1, COL18A1, COL4A1, COL5A1, COL5A2, COL6A1, CORO1C, CRABP2, CSRP2, CSTB, CTNNB1, DAB2, DCN, DHCR24, DIPK2A, DSP, DVL2, DYNLL1, EDF1, EFEMP1, EFNB2, ENPP1, EPHA2, EPHA3, EPPK1, EZR, FASN, FAT1, FBXW8, FERMT1, FERMT2, FGF2, FHL2, FKBP1A, FN1, FN3K, FTO, G6PD, GARS1, GLMN, GPC1, GPC6, GPI, GSK3B, GSTM3, HDAC2, HDAC4, HHIP, HSD17B4, HSPG2, IFT140, IKBKB, IQGAP3, ITGA3, ITGAV, ITGB1, ITGB5, ITPK1, JAG1, KCNQ1, KRT18, KRT19, KRT8, LAMA5, LAMC1, LEMD2, LIMS1, LIN7C, LUM, MACROH2A1, MBD3, MBOAT2, MEST, MGMT, MLF2, MSN, MTCH1, MYADM, MYD88, MYO1E, MYO6, NF1, NF2, NIBAN2, NLE1, NOM1, NSDHL, OBSL1, OXSR1, PAFAH1B1, PALLD, PCK2, PELO, PHGDH, PHLDB1, PIK3CA, PIN1, PLXNB2, PML, POFUT1, PPP1CA, PPP3CA, PRKAA1, PSMA3, PSMB10,	No change

					PSMB2, PSMC2, PSMC3, PSMC6, PSMD1, PSMD12, PSMD14, PSMD2, PSMD6, PSMD7, PSMD8, PSME1, PSME2, PSME4, PTK7, PTRH2, PUS7, RAB10, RAB1A, RALA, RAP2B, RAP2C, RCN3, RDH10, RDX, RHEB, RHOB, RHOC, ROR1, RPL38, RTN4, SBDS, SCRIB, SEMA3C, SERPINE2, SIRT1, SLC4A7, SLC9A1, SLIT2, SMAD4, SOAT1, SOD1, SPART, SPTLC2, SRC, STAT1, STAT5B, STK24, SUFU, SVIL, TAGLN, TAGLN2, TGFBI, TGFBR3, TGM2, THBS3, TIGAR, TJP1, TJP2, TMEM119, TPP1, TRADD, TRIM28, TSC2, TUBB3, UBA2, VASP, VDAC1, VDAC2, VTN, WDR1, YAP1, YBX1, ZDHHC21, ZMPSTE24]	
cell morphogenesis	GO_BiologicalProcess-EBI-UniProt-GOA-ACAP-ARAP_13.05.2021_00h00	2.32E-09	12.5	138	[ABI1, ACTB, ACTR2, ADAM10, ADARB1, ADNP, AKR1B1, ALCAM, ALDOA, ANK3, APP, ARPIN, B4GALT5, BAIAP2, C1QBP, CAP1, CAP2, CASP3, CDC42EP1, CDH11, CFDP1, CLU, COL15A1, COL18A1, CORO1B, CORO1C, CRABP2, CTNNB1, CUX1, CXCL12, DHX36, DIAPH1, DICER1, DNM1L, DNMBP, DPYSL2, DPYSL4, DVL2, DYNLL1, EFNB2, EPB41L3, EPHA2, EPHA3, EPHB4, ERBB2, EZR, FAM171A1, FARP1, FAT1, FBLIM1, FBXW8, FERMT2, FGD4, FLRT2, FN1, GAB1, GBP1, GPC1, GSK3B, HEXB, HSP90AA1, IGF2BP1, IMPACT, IST1, ITGAV, ITGB1, LAMA5, LAMC1, LIMS1, LIPA, LLGL1, LYPLA2, MACF1, MAP1B, MAP4K4, MLF2, MOV10, MSN, MYADM, MYH10, MYH9, NCKAP1, NEDD4, NES, NIBAN2, NUDT19, OBSL1, PAFAH1B1, PALLD, PIGU,	No change

					PIK3CA, PKN2, PLCG1, PLXNB2, PPP3CA, PSMB10, PTK7, PXN, RAB10, RAB1A, RAB8A, RAC3, RDX, RHOB, RHOC, RIPK2, RPL24, RTN4, S100A10, SARM1, SCFD1, SCRIB, SEMA3C, SEPTIN7, SERPINB8, SH3KBP1, SLC11A2, SLIT2, SMAD4, SNX1, SOD1, SPARC, SPART, SRC, TNIK, TPBG, TRIO, TUBB3, TWF2, UBA2, USP9X, UST, VASP, VLDLR, WDR1, YAP1, ZMPSTE24, ZMYM3]	
regulation of anatomical structure morphogenesis	GO_BiologicalProcess-EBI-UniProt-GOA-ACAP-ARAP_13.05.2021_00h00	2.32E-09	12.759644	129	[ACTR2, ADAM10, ADAMTS9, ADNP, AGO2, AKR1B1, ALDOA, ANXA3, ARPIN, BAIAP2, C1QBP, CDC42EP1, CFDP1, CHAMP1, CLTC, CORO1B, CORO1C, CRABP2, CTNNB1, CUX1, CXCL12, CYRIB, DAB2, DHX36, DIAPH1, DNM1L, DNMBP, DVL2, DYNLL1, EPB41L3, EPHA2, ERAP1, ERBB2, EZR, FAM171A1, FBLIM1, FBXW8, FERMT2, FGD4, FGF2, FN1, GAB1, GARS1, GBP1, GLUL, GPC6, GSK3B, HEXB, HGS, HK2, HMOX1, HSPB6, HSPG2, IST1, ITGB1, LIMS1, LLGL1, MACF1, MAP1B, MARCHF5, MFF, MLF2, MOV10, MSN, MYADM, MYH10, MYH9, NEDD4, NF1, NIBAN2, OBSL1, OXSR1, PAFAH1B1, PHLDB1, PIGU, PKM, PKN2, PLCG1, PLXNB2, PML, PPP3CA, PSMA3, PSMB10, PSMB2, PSMC2, PSMC3, PSMC6, PSMD1, PSMD12, PSMD14, PSMD2, PSMD6, PSMD7, PSMD8, PSME1, PSME2, PSME4, PTGIS, PTK7, RAC3, RALA, RDX, RHOB, RHOC, ROR1, RTN4, S100A10, SARM1, SARS1, SEMA3C, SEPTIN7, SH3KBP1, SIRT1, SLC12A2, SLIT2, SPARC, SPART, SRC, STAT1, STAT2, THBS1, TJP1, TNIK,	No change

					TWF2, UST, VAT1, WDR1, ZMPSTE24, ZMYM3]	
cellular component morphogenesis	GO_BiologicalProcess-EBI-UniProt-GOA-ACAP-ARAP_13.05.2021_00h00	2.32E-09	12.305854	103	[ABI1, ACTB, ACTG1, ACTR2, ADAM10, ADARB1, ADNP, ALCAM, ANK3, APP, ARPIN, B4GALT5, BAIAP2, CDH11, CFL2, CORO1B, CORO1C, CRABP2, CSRP2, CTNNB1, CUX1, CXCL12, DHX36, DICER1, DNM1L, DPYSL2, DPYSL4, DVL2, EFNB2, EPB41L3, EPHA2, EPHA3, EPHB4, ERBB2, EZR, FARP1, FBXW8, FLII, FLRT2, FN1, GAB1, GPC1, GSK3B, HSP90AA1, IGF2BP1, IMPACT, IST1, ITGB1, KRT19, KRT8, LAMA5, LAMC1, LLGL1, LMOD1, LYPLA2, MACF1, MAP1B, MAP4K4, MLF2, MOV10, NCKAP1, NECTIN2, NEDD4, NES, NIBAN2, NUBPL, OBSL1, PAFAH1B1, PALLD, PHLDB1, PIGU, PIK3CA, PKN2, PLCG1, PLXNB2, PPP3CA, PTK7, RAB10, RAB8A, RIPK2, RPL24, RTN4, SARM1, SEMA3C, SH3KBP1, SLC11A2, SLIT2, SMAD4, SNX1, SPART, SRC, TBPL1, TNIK, TPBG, TRIO, TUBB3, TWF2, UBA2, USP9X, UST, VASP, VLDLR, WDR1]	Upregulation
cell development	GO_BiologicalProcess-EBI-UniProt-GOA-ACAP-ARAP_13.05.2021_00h00	2.32E-09	10.619872	233	[ABI1, ACSL4, ACTB, ACTG1, ACTR2, ADAM10, ADARB1, ADNP, AFDN, AGRN, AKR1B1, ALCAM, ANK3, APEX1, APLP2, APP, APPL2, ARFGEF1, ASPM, ATXN10, B2M, B4GALT1, B4GALT5, BAIAP2, BIN1, BPNT2, C1QBP, CASP3, CDH11, CDK1, CDK16, CECR2, CFL2, CHD7, CLU, CNGB1, COL11A1, COL15A1, COL18A1, CORO1C, CRABP2, CSRP2, CTNNB1, CUL4B, CUX1, CXCL12, DGUOK, DHFR, DHX36, DICER1, DNM1L, DPYSL2, DPYSL3, DPYSL4, DVL2, DYNLL1, EFNB2, EIF2B1,	Upregulation

					EPB41L3, EPHA2, EPHA3, EPHB4, ERBB2, EZR, FAM3A, FARP1, FASN, FAT1, FBXW8, FERMT2, FGF2, FHL2, FLII, FLRT2, FMR1, FN1, G6PD, GAB1, GBA2, GBP1, GPC1, GSK3B, GSTM3, GSTP1, HDAC2, HDAC4, HEXB, HSD17B4, HSP90AA1, HSPA5, IDH2, IFT140, IGF2BP1, IKBKB, IMPACT, IST1, ITGA3, ITGAV, ITGB1, ITM2C, ITSN1, JAG1, JAM3, KCNQ1, KDM1A, KRT19, KRT8, LAMA5, LAMC1, LIMS1, LLGL1, LMOD1, LRRK1, LYPLA2, MACF1, MAEA, MAP1B, MAP4K4, MICALL1, MLF2, MLH1, MME, MOV10, MSH2, MSI2, MSN, MTCH1, MTR, MYADM, MYD88, MYH9, MYO1E, MYOF, NCDN, NCKAP1, NDRG1, NECTIN2, NEDD4, NF1, NF2, NIBAN2, NPR2, NSUN5, OBSL1, OXSR1, P3H1, PAFAH1B1, PALLD, PANX1, PHGDH, PIGU, PIK3CA, PIN1, PITHD1, PKN2, PLCG1, PLXNB2, PPP3CA, PRDX6, PRKCI, PRKG1, PRPF19, PRUNE1, PSME4, PTBP3, PTK7, PTPRF, PTPRG, PXN, RAB10, RAB1A, RAB8A, RAC3, RANBP1, RAP2B, RAP2C, RDH10, RDX, RHEB, RIPK2, RNF2, ROR1, RPL24, RTN4, S100A10, SARM1, SCARB2, SCRIB, SEMA3C, SEPTIN2, SERPINE2, SH3KBP1, SLC11A2, SLC4A7, SLIT2, SMAD4, SMARCD3, SMYD3, SNW1, SOD1, SPART, SRC, STK24, SYNJ1, SYPL2, TBPL1, TENM3, TGFBR3, TGM2, THOC2, TIGAR, TJP1, TJP2, TNIK, TPBG, TRAPPC4, TRIO, TRIP13, TSPO, TUBB3, TUT4, TWF2, UBA2, USP9X, UST, VASP, VLDLR, WASHC5, WDR1,	

					WDR5, YAP1, YTHDC1, ZDHHC21]	
cell projection organization	GO_BiologicalProcess-EBI-UniProt-GOA-ACAP-ARAP_13.05.2021_00h00	2.32E-09	10.790537	187	[ABI1, ACSL4, ACTB, ACTR2, ACTR3, ADAM10, ADARB1, ADNP, AGRN, ALCAM, ANK3, ANO6, APEX1, APP, ARFGEF1, ARPIN, ATXN10, B2M, B4GALT5, BAIAP2, CASP3, CDC42EP1, CDH11, CDK1, CDK16, CECR2, CETN2, CFAP20, CFAP298, CLU, CNGB1, CORO1B, CORO1C, CRABP2, CSPG4, CTNNB1, CUL4B, CUX1, CXCL12, DGUOK, DHFR, DHX36, DICER1, DNM1L, DPYSL2, DPYSL3, DPYSL4, DVL2, DYNLL1, EFNB2, EHD3, EPB41L3, EPHA2, EPHA3, EPHB4, ERBB2, EXOC2, EZR, FARP1, FBXW8, FGD4, FLRT2, FMR1, FN1, FNBP1L, FSCN1, GAB1, GPC1, GSK3B, HDAC2, HDAC4, HSP90AA1, HSPA5, HSPB11, IFT140, IGF2BP1, IMPACT, IST1, ITGA3, ITGB1, ITM2C, ITSN1, JAM3, KDM1A, LAMA5, LAMC1, LLGL1, LYPLA2, MACF1, MAP1B, MAP4K4, MICALL1, MLF2, MOV10, MTR, MYH9, NCAPG, NCDN, NCK2, NCKAP1, NECTIN2, NEDD1, NEDD4, NEK1, NES, NIBAN2, NUBP2, NUDT19, OBSL1, P3H1, PAFAH1B1, PALLD, PHGDH, PIGU, PIK3CA, PKN2, PLCG1, PLD1, PLXNB2, PPP3CA, PRKCI, PRKCSH, PRKG1, PTGES2, PTK7, PTPRF, PTPRG, RAB10, RAB1A, RAB23, RAB5A, RAB8A, RAC3, RALA, RANBP1, RAP2B, RAP2C, RDX, RIPK2, ROR1, RPL24, RTN4, RTTN, SARM1, SCARB2, SCRIB, SEMA3C, SEPTIN2, SEPTIN7, SEPTIN9, SH3KBP1, SLC11A2, SLIT2, SMAD4, SNX1, SOD1, SPART, SRC, STK24, SYNE2,	Upregulation

					TBC1D10A, TBC1D17, TBC1D22B, TENM3, THOC2, TNIK, TPBG, TRAPPC4, TRIO, TSPO, TTC39C, TUBA4A, TUBB, TUBB3, TWF2, UBA2, UNC119B, USP9X, UST, VASP, VLDLR, WASHC5, WDR11, WDR35, WDR5, WRAP73, YAP1]	
positive regulation of cellular component organization	GO_BiologicalProcess-EBI-UniProt-GOA-ACAP-ARAP_13.05.2021_00h00	2.32E-09	13.096201	162	[ABCB7, ACTR2, ACTR3, ADNP, AGRN, AKR1B1, ANKFY1, ANO6, APEX1, APLP2, APP, APPL2, ARFIP1, ARPC4, ARPC5L, ARPIN, ARRB1, ATL3, B2M, BAIAP2, BIN1, CCN2, CCT2, CCT3, CCT6A, CDC34, CDC42EP1, CFL2, CHAF1B, CHD1L, CHTOP, CLU, CNOT1, CORO1B, CORO1C, CRABP2, CRBN, CTBP1, CTNNB1, CUL4B, CUX1, CXCL12, DAB2, DHX36, DNM1L, DNMT1, DPYSL3, DRG1, DUSP3, DVL2, EPHA2, EPHA3, ERCC5, FBXW8, FEN1, FERMT1, FERMT2, FHOD1, FLRT2, FMR1, FN1, FNBP1L, FSCN1, GLYR1, GNL3L, GSK3B, H1-5, HDAC4, HIP1, HSP90AA1, HSPA5, IST1, ITGA3, ITSN1, KAT7, KDM1A, LIMS1, LLGL1, LMOD1, LRCH4, MACF1, MACROH2A1, MAP1B, MAP3K4, MAP4K4, MARCHF5, MFF, MMAB, MPHOSPH8, MSN, MYD88, MYO1E, NCAPG, NCK2, NCKAP1, NDRG1, NES, NF2, NMT1, NUP62, OBSL1, P3H1, PAFAH1B1, PARP1, PDCD6IP, PHLDB1, PIEZO1, PIK3CA, PIP4K2B, PLXNB2, PML, PPP3CA, PRKCI, PSMC2, PSMC3, PSMC6, PTK7, PXN, PYCARD, RAD21, RALA, RANBP1, RHOC, RIF1, RIPK2, RNF40, ROR1, RPA1, RPA2, S100A10, SCARB2, SEPTIN7, SEPTIN9, SIRT1,	No change

					SLC25A4, SLC25A5, SLIT2, SMAD4, SMARCB1, SMPD1, SNW1, SORBS3, SPHK2, SRC, SYNJ1, SYNPO, TCP1, TENM3, TF, TMED9, TPBG, TRIM28, TWF2, VASP, VDAC1, VLDLR, VTN, WASHC5, WDR1, WDR35, WDR5, WRAP73]	
cell morphogenesis involved in differentiation	GO_BiologicalProcess-EBI-UniProt-GOA-ACAP-ARAP_13.05.2021_00h00	2.32E-09	12.373737	98	[ABI1, ACTB, ACTR2, ADAM10, ADARB1, ADNP, AKR1B1, ALCAM, ANK3, APP, B4GALT5, BAIAP2, C1QBP, CASP3, CDH11, COL15A1, COL18A1, CORO1C, CRABP2, CTNNB1, CUX1, CXCL12, DHX36, DNM1L, DPYSL2, EFNB2, EPHA2, EPHA3, EPHB4, ERBB2, EZR, FARP1, FAT1, FBXW8, FERMT2, FLRT2, FN1, GAB1, GBP1, GPC1, GSK3B, HSP90AA1, IGF2BP1, IST1, ITGAV, ITGB1, LAMA5, LAMC1, LIMS1, LLGL1, LYPLA2, MACF1, MAP1B, MLF2, MYADM, MYH9, NEDD4, NIBAN2, OBSL1, PAFAH1B1, PALLD, PIGU, PIK3CA, PKN2, PLCG1, PLXNB2, PPP3CA, PTK7, PXN, RAB10, RAB1A, RAB8A, RAC3, RIPK2, RPL24, RTN4, S100A10, SARM1, SCRIB, SEMA3C, SH3KBP1, SLC11A2, SLIT2, SMAD4, SOD1, SPART, SRC, TNIK, TPBG, TRIO, TUBB3, TWF2, UBA2, USP9X, UST, VASP, VLDLR, WDR1]	No change
regulation of cell morphogenesis	GO_BiologicalProcess-EBI-UniProt-GOA-ACAP-ARAP_13.05.2021_00h00	2.32E-09	14.98471	49	[ACTR2, ALDOA, ARPIN, BAIAP2, C1QBP, CDC42EP1, CFDP1, CORO1B, CORO1C, CUX1, DHX36, DIAPH1, DNM1L, DNMBP, DVL2, DYNLL1, EPB41L3, EZR, FAM171A1, FBLIM1, FBXW8, FERMT2, FGD4, FN1, GBP1, HEXB, LIMS1, LLGL1, MACF1, MOV10, MSN, MYADM, MYH10, MYH9, OBSL1, PAFAH1B1, PLXNB2, RAC3, RDX, RHOB, RHOC,	No change

					S100A10, SEPTIN7, SH3KBP1, SPARC, SRC, WDR1, ZMPSTE24, ZMYM3]	
regulation of cell projection organization	GO_BiologicalProcess-EBI-UniProt-GOA-ACAP-ARAP_13.05.2021_00h00	2.32E-09	12.256268	88	[ACTR2, ACTR3, ADAM10, ADNP, AGRN, APEX1, ARPIN, B2M, BAIAP2, CDC42EP1, CLU, CORO1B, CORO1C, CRABP2, CUX1, CXCL12, DGUOK, DHX36, DNM1L, DPYSL2, DPYSL3, DVL2, EFNB2, EPHA2, EPHA3, EZR, FBXW8, FMR1, FN1, FNBP1L, FSCN1, GSK3B, HDAC2, HDAC4, HSPA5, IFT140, IST1, ITGA3, ITM2C, KDM1A, LLGL1, MACF1, MAP1B, MAP4K4, MOV10, NCKAP1, NEDD4, OBSL1, P3H1, PAFAH1B1, PIK3CA, PKN2, PLD1, PLXNB2, PPP3CA, PRKCI, PRKCSH, PTK7, PTPRF, PTPRG, RAB5A, RALA, RANBP1, RDX, ROR1, RTN4, SARM1, SCARB2, SEMA3C, SEPTIN7, SEPTIN9, SLIT2, SPART, SRC, STK24, SYNE2, TBC1D10A, TBC1D17, TBC1D22B, TENM3, THOC2, TNIK, TWF2, UST, VLDLR, WASHC5, WRAP73, YAP1]	Upregulation
cell part morphogenesis	GO_BiologicalProcess-EBI-UniProt-GOA-ACAP-ARAP_13.05.2021_00h00	2.32E-09	12.5	93	[ABI1, ACTB, ACTR2, ADAM10, ADARB1, ADNP, ALCAM, ANK3, APP, ARPIN, B4GALT5, BAIAP2, CDH11, CORO1B, CORO1C, CRABP2, CTNNB1, CUX1, CXCL12, DHX36, DICER1, DNM1L, DPYSL2, DPYSL4, DVL2, EFNB2, EPB41L3, EPHA2, EPHA3, EPHB4, ERBB2, EZR, FARP1, FBXW8, FLRT2, FN1, GAB1, GPC1, GSK3B, HSP90AA1, IGF2BP1, IMPACT, IST1, ITGB1, LAMA5, LAMC1, LLGL1, LYPLA2, MACF1, MAP1B, MAP4K4, MLF2, MOV10, NCKAP1, NECTIN2, NEDD4, NES, NIBAN2, NUBPL, OBSL1, PAFAH1B1, PALLD, PIGU, PIK3CA, PKN2,	Upregulation

					PLCG1, PLXNB2, PPP3CA, PTK7, RAB10, RAB8A, RIPK2, RPL24, RTN4, SARM1, SEMA3C, SH3KBP1, SLC11A2, SLIT2, SMAD4, SNX1, SPART, SRC, TNIK, TPBG, TRIO, TUBB3, TWF2, UBA2, USP9X, UST, VASP, VLDLR]	
neurogenesis	GO_BiologicalProcess-EBI-UniProt-GOA-ACAP-ARAP_13.05.2021_00h00	2.32E-09	10.565392	185	[ABI1, ACSL3, ACSL4, ACTB, ACTR2, ADAM10, ADARB1, ADNP, AGRN, ALCAM, ANK3, APEX1, APLP2, APP, APPL2, ARFGEF1, ASPM, ATP2B2, ATXN10, B2M, B4GALT5, BAIAP2, BIN1, CASP3, CDH11, CDK1, CDK16, CDK5RAP3, CECR2, CHD7, CLU, CNGB1, CRABP2, CSPG4, CTNNB1, CUL4B, CUX1, CXCL12, DAGLB, DGUOK, DHFR, DHX36, DICER1, DNM1L, DPYSL2, DPYSL3, DPYSL4, DVL2, EFNB2, EIF2B1, ELP3, EML1, EPB41L3, EPHA2, EPHA3, EPHB4, ERBB2, EZR, FARP1, FBXW8, FGF2, FLRT2, FMR1, FN1, GAB1, GBA2, GPC1, GSK3B, GSTP1, HDAC2, HEXB, HHIP, HSP90AA1, HSPA5, IDH2, IFT140, IGF2BP1, IMPACT, IST1, ITGA3, ITGB1, ITM2C, ITSN1, JAG1, JAM3, KCNQ1, KDM1A, LAMA5, LAMC1, LEMD2, LLGL1, LYPLA2, MACF1, MAP1B, MAP4K4, MBOAT7, METTL14, MICALL1, MLF2, MME, MOV10, MTCH1, MTR, MYO6, NCDN, NCKAP1, NDRG1, NEDD4, NF1, NF2, NIBAN2, NSUN5, OBSL1, P3H1, PAFAH1B1, PALLD, PHGDH, PIGT, PIGU, PIK3CA, PIN1, PKN2, PLCG1, PLXNB2, POMGNT2, POMK, PPP3CA, PRDX6, PRKCI, PRKG1, PRPF19, PRUNE1, PTK7, PTPRF, PTPRG, RAB10, RAB8A, RAC3, RANBP1, RHEB, RIPK2, ROR1, RPL24, RTN4,	Upregulation

					SARM1, SCARB2, SCRIB, SEMA3C, SEPTIN2, SERPINE2, SH3KBP1, SLC11A2, SLC1A1, SLC4A7, SLIT2, SMAD4, SMARCA1, SMARCD3, SMARCE1, SNW1, SOD1, SPART, SRC, STK24, SUFU, SYNE2, SYNJ1, TENM3, TGM2, THOC2, TNIK, TPBG, TRAPPC4, TRIO, TSPO, TUBB3, TUBGCP2, TWF2, UBA2, USP9X, UST, VASP, VLDLR, WASHC5, WDR5]	
cell projection morphogenesis	GO_BiologicalProcess-EBI-UniProt-GOA-ACAP-ARAP_13.05.2021_00h00	2.32E-09	12.517194	91	[ABI1, ACTB, ACTR2, ADAM10, ADARB1, ADNP, ALCAM, ANK3, APP, ARPIN, B4GALT5, BAIAP2, CDH11, CORO1B, CORO1C, CRABP2, CTNNB1, CUX1, CXCL12, DHX36, DICER1, DNM1L, DPYSL2, DPYSL4, DVL2, EFNB2, EPB41L3, EPHA2, EPHA3, EPHB4, ERBB2, EZR, FARP1, FBXW8, FLRT2, FN1, GAB1, GPC1, GSK3B, HSP90AA1, IGF2BP1, IMPACT, IST1, ITGB1, LAMA5, LAMC1, LLGL1, LYPLA2, MACF1, MAP1B, MAP4K4, MLF2, MOV10, NCKAP1, NEDD4, NES, NIBAN2, OBSL1, PAFAH1B1, PALLD, PIGU, PIK3CA, PKN2, PLCG1, PLXNB2, PPP3CA, PTK7, RAB10, RAB8A, RIPK2, RPL24, RTN4, SARM1, SEMA3C, SH3KBP1, SLC11A2, SLIT2, SMAD4, SNX1, SPART, SRC, TNIK, TPBG, TRIO, TUBB3, TWF2, UBA2, USP9X, UST, VASP, VLDLR]	Upregulation
plasma membrane bounded cell projection organization	GO_BiologicalProcess-EBI-UniProt-GOA-ACAP-ARAP_13.05.2021_00h00	2.32E-09	10.855457	184	[ABI1, ACSL4, ACTB, ACTR2, ACTR3, ADAM10, ADARB1, ADNP, AGRN, ALCAM, ANK3, ANO6, APEX1, APP, ARFGEF1, ARPIN, ATXN10, B2M, B4GALT5, BAIAP2, CASP3, CDC42EP1, CDH11, CDK1, CDK16, CECR2, CETN2, CFAP20, CFAP298, CLU, CNGB1, CORO1B, CORO1C, CRABP2, CSPG4, CTNNB1,	Upregulation

					CUL4B, CUX1, CXCL12, DGUOK, DHFR, DHX36, DICER1, DNM1L, DPYSL2, DPYSL3, DPYSL4, DVL2, DYNLL1, EFNB2, EHD3, EPB41L3, EPHA2, EPHA3, EPHB4, ERBB2, EXOC2, EZR, FARP1, FBXW8, FGD4, FLRT2, FMR1, FN1, FNBP1L, FSCN1, GAB1, GPC1, GSK3B, HDAC2, HDAC4, HSP90AA1, HSPA5, HSPB11, IFT140, IGF2BP1, IMPACT, IST1, ITGA3, ITGB1, ITM2C, ITSN1, JAM3, KDM1A, LAMA5, LAMC1, LLGL1, LYPLA2, MACF1, MAP1B, MAP4K4, MICALL1, MLF2, MOV10, MTR, MYH9, NCDN, NCK2, NCKAP1, NECTIN2, NEDD1, NEDD4, NEK1, NIBAN2, NUDT19, OBSL1, P3H1, PAFAH1B1, PALLD, PHGDH, PIGU, PIK3CA, PKN2, PLCG1, PLD1, PLXNB2, PPP3CA, PRKCI, PRKCSH, PRKG1, PTGES2, PTK7, PTPRF, PTPRG, RAB10, RAB1A, RAB23, RAB5A, RAB8A, RAC3, RALA, RANBP1, RAP2B, RAP2C, RDX, RIPK2, ROR1, RPL24, RTN4, RTTN, SARM1, SCARB2, SCRIB, SEMA3C, SEPTIN2, SEPTIN7, SEPTIN9, SH3KBP1, SLC11A2, SLIT2, SMAD4, SNX1, SOD1, SPART, SRC, STK24, SYNE2, TBC1D10A, TBC1D17, TBC1D22B, TENM3, THOC2, TNIK, TPBG, TRAPPC4, TRIO, TSPO, TTC39C, TUBA4A, TUBB, TUBB3, TWF2, UBA2, UNC119B, USP9X, UST, VASP, VLDLR, WASHC5, WDR11, WDR35, WDR5, WRAP73, YAP1]	
regulation of plasma membrane bounded cell projection organization	GO_BiologicalProcess-EBI-UniProt-GOA-ACAP-ARAP_13.05.2021_00h00	2.32E-09	12.17765	85	[ACTR2, ACTR3, ADAM10, ADNP, AGRN, APEX1, ARPIN, B2M, BAIAP2, CDC42EP1, CLU, CORO1B, CORO1C, CRABP2, CUX1, CXCL12,	Upregulation

					DGUOK, DHX36, DNM1L, DPYSL2, DPYSL3, EFNB2, EPHA2, EPHA3, EZR, FBXW8, FMR1, FN1, FNBP1L, FSCN1, GSK3B, HDAC2, HDAC4, HSPA5, IFT140, IST1, ITGA3, ITM2C, KDM1A, MACF1, MAP1B, MAP4K4, NCKAP1, NEDD4, OBSL1, P3H1, PAFAH1B1, PIK3CA, PKN2, PLD1, PLXNB2, PPP3CA, PRKCI, PRKCSH, PTK7, PTPRF, PTPRG, RAB5A, RALA, RANBP1, RDX, ROR1, RTN4, SARM1, SCARB2, SEMA3C, SEPTIN7, SEPTIN9, SLIT2, SPART, SRC, STK24, SYNE2, TBC1D10A, TBC1D17, TBC1D22B, TENM3, THOC2, TNIK, TWF2, UST, VLDLR, WASHC5, WRAP73, YAP1]	
generation of neurons	GO_BiologicalProcess-EBI-UniProt-GOA-ACAP-ARAP_13.05.2021_00h00	2.32E-09	10.416667	170	[ABI1, ACSL3, ACSL4, ACTB, ACTR2, ADAM10, ADARB1, ADNP, AGRN, ALCAM, ANK3, APEX1, APLP2, APP, APPL2, ARFGEF1, ASPM, ATP2B2, ATXN10, B2M, B4GALT5, BAIAP2, BIN1, CASP3, CDH11, CDK16, CDK5RAP3, CECR2, CHD7, CNGB1, CRABP2, CTNNB1, CUL4B, CUX1, CXCL12, DAGLB, DGUOK, DHFR, DHX36, DICER1, DNM1L, DPYSL2, DPYSL3, DPYSL4, DVL2, EFNB2, ELP3, EML1, EPB41L3, EPHA2, EPHA3, EPHB4, ERBB2, EZR, FARP1, FBXW8, FLRT2, FMR1, FN1, GAB1, GBA2, GPC1, GSK3B, HDAC2, HHIP, HSP90AA1, HSPA5, IDH2, IFT140, IGF2BP1, IMPACT, IST1, ITGA3, ITGB1, ITM2C, ITSN1, JAG1, JAM3, KCNQ1, KDM1A, LAMA5, LAMC1, LLGL1, LYPLA2, MACF1, MAP1B, MAP4K4, METTL14, MICALL1, MLF2, MME, MOV10, MTCH1, MTR, MYO6, NCDN,	Upregulation

					NCKAP1, NEDD4, NF1, NF2, NIBAN2, OBSL1, P3H1, PAFAH1B1, PALLD, PHGDH, PIGT, PIGU, PIK3CA, PIN1, PKN2, PLCG1, PLXNB2, POMGNT2, POMK, PPP3CA, PRKCI, PRKG1, PRPF19, PRUNE1, PTK7, PTPRF, PTPRG, RAB10, RAB8A, RAC3, RANBP1, RHEB, RIPK2, ROR1, RPL24, RTN4, SARM1, SCARB2, SCRIB, SEMA3C, SEPTIN2, SERPINE2, SH3KBP1, SLC11A2, SLC4A7, SLIT2, SMAD4, SMARCA1, SMARCD3, SNW1, SOD1, SPART, SRC, STK24, SUFU, SYNJ1, TENM3, TGM2, THOC2, TNIK, TPBG, TRAPPC4, TRIO, TSPO, TUBB3, TUBGCP2, TWF2, UBA2, USP9X, UST, VASP, VLDLR, WASHC5, WDR5]	
neuron development	GO_BiologicalProcess-EBI-UniProt-GOA-ACAP-ARAP_13.05.2021_00h00	2.32E-09	11.083744	135	[ABI1, ACSL4, ACTB, ACTR2, ADAM10, ADARB1, ADNP, AGRN, ALCAM, ANK3, APP, ARFGEF1, ATXN10, B2M, B4GALT5, BAIAP2, CASP3, CDH11, CDK16, CECR2, CNGB1, CRABP2, CTNNB1, CUL4B, CUX1, CXCL12, DGUOK, DHFR, DHX36, DICER1, DNM1L, DPYSL2, DPYSL3, DPYSL4, DVL2, EFNB2, EPB41L3, EPHA2, EPHA3, EPHB4, ERBB2, EZR, FARP1, FBXW8, FLRT2, FMR1, FN1, GAB1, GBA2, GPC1, GSK3B, HDAC2, HSP90AA1, HSPA5, IFT140, IGF2BP1, IMPACT, IST1, ITGA3, ITGB1, ITM2C, ITSN1, JAM3, KCNQ1, KDM1A, LAMA5, LAMC1, LLGL1, LYPLA2, MACF1, MAP1B, MAP4K4, MICALL1, MLF2, MOV10, MTCH1, MTR, NCDN, NCKAP1, NEDD4, NIBAN2, OBSL1, P3H1, PAFAH1B1, PALLD, PHGDH, PIGU, PIK3CA, PKN2, PLCG1, PLXNB2,	Upregulation

					PPP3CA, PRKCI, PRKG1, PTK7, PTPRF, PTPRG, RAB10, RAB8A, RAC3, RANBP1, RIPK2, ROR1, RPL24, RTN4, SARM1, SCARB2, SCRIB, SEMA3C, SEPTIN2, SH3KBP1, SLC11A2, SLC4A7, SLIT2, SMAD4, SOD1, SPART, SRC, STK24, TENM3, THOC2, TNIK, TPBG, TRAPPC4, TRIO, TSPO, TUBB3, TWF2, UBA2, USP9X, UST, VASP, VLDLR, WASHC5, WDR5]	
neuron projection development	GO_BiologicalProcess-EBI-UniProt-GOA-ACAP-ARAP_13.05.2021_00h00	2.32E-09	11.9514475	128	[ABI1, ACSL4, ACTB, ACTR2, ADAM10, ADARB1, ADNP, ALCAM, ANK3, APP, ARFGEF1, ATXN10, B2M, B4GALT5, BAIAP2, CASP3, CDH11, CDK16, CECR2, CRABP2, CTNNB1, CUL4B, CUX1, CXCL12, DGUOK, DHFR, DHX36, DICER1, DNM1L, DPYSL2, DPYSL3, DPYSL4, DVL2, EFNB2, EPB41L3, EPHA2, EPHA3, EPHB4, ERBB2, EZR, FARP1, FBXW8, FLRT2, FMR1, FN1, GAB1, GPC1, GSK3B, HDAC2, HSP90AA1, HSPA5, IGF2BP1, IMPACT, IST1, ITGA3, ITGB1, ITM2C, ITSN1, JAM3, KDM1A, LAMA5, LAMC1, LLGL1, LYPLA2, MACF1, MAP1B, MAP4K4, MICALL1, MLF2, MOV10, MTR, NCDN, NCKAP1, NEDD4, NIBAN2, OBSL1, P3H1, PAFAH1B1, PALLD, PHGDH, PIGU, PIK3CA, PKN2, PLCG1, PLXNB2, PPP3CA, PRKCI, PRKG1, PTK7, PTPRF, PTPRG, RAB10, RAB8A, RAC3, RANBP1, RIPK2, ROR1, RPL24, RTN4, SARM1, SCARB2, SCRIB, SEMA3C, SEPTIN2, SH3KBP1, SLC11A2, SLIT2, SMAD4, SOD1, SPART, SRC, STK24, TENM3, THOC2, TNIK, TPBG, TRAPPC4, TRIO, TSPO, TUBB3, TWF2,	Upregulation

					UBA2, USP9X, UST, VASP, VLDLR, WASHC5, WDR5]	
cell morphogenesis involved in neuron differentiation	GO_BiologicalProcess-EBI-UniProt-GOA-ACAP-ARAP_13.05.2021_00h00	2.32E-09	12.34375	79	[ABI1, ACTB, ACTR2, ADAM10, ADARB1, ADNP, ALCAM, ANK3, APP, B4GALT5, BAIAP2, CDH11, CRABP2, CUX1, CXCL12, DHX36, DNM1L, DPYSL2, EFNB2, EPHA2, EPHA3, EPHB4, ERBB2, EZR, FARP1, FBXW8, FLRT2, FN1, GAB1, GPC1, GSK3B, HSP90AA1, IGF2BP1, IST1, ITGB1, LAMA5, LAMC1, LLGL1, LYPLA2, MACF1, MAP1B, MLF2, NEDD4, NIBAN2, OBSL1, PAFAH1B1, PALLD, PIGU, PIK3CA, PKN2, PLCG1, PLXNB2, PPP3CA, PTK7, RAB10, RAB8A, RIPK2, RPL24, RTN4, SARM1, SCRIB, SEMA3C, SH3KBP1, SLC11A2, SLIT2, SMAD4, SOD1, SPART, SRC, TNIK, TPBG, TRIO, TUBB3, TWF2, UBA2, USP9X, UST, VASP, VLDLR]	Upregulation
neuron projection morphogenesis	GO_BiologicalProcess-EBI-UniProt-GOA-ACAP-ARAP_13.05.2021_00h00	2.32E-09	12.146893	86	[ABI1, ACTB, ACTR2, ADAM10, ADARB1, ADNP, ALCAM, ANK3, APP, B4GALT5, BAIAP2, CDH11, CRABP2, CTNNB1, CUX1, CXCL12, DHX36, DICER1, DNM1L, DPYSL2, DPYSL4, DVL2, EFNB2, EPB41L3, EPHA2, EPHA3, EPHB4, ERBB2, EZR, FARP1, FBXW8, FLRT2, FN1, GAB1, GPC1, GSK3B, HSP90AA1, IGF2BP1, IMPACT, IST1, ITGB1, LAMA5, LAMC1, LLGL1, LYPLA2, MACF1, MAP1B, MAP4K4, MLF2, MOV10, NCKAP1, NEDD4, NIBAN2, OBSL1, PAFAH1B1, PALLD, PIGU, PIK3CA, PKN2, PLCG1, PLXNB2, PPP3CA, PTK7, RAB10, RAB8A, RIPK2, RPL24, RTN4, SARM1, SEMA3C, SH3KBP1, SLC11A2, SLIT2, SMAD4, SPART, SRC, TNIK, TPBG, TRIO, TUBB3,	Upregulation

					TWF2, UBA2, USP9X, UST, VASP, VLDLR]	
sulfur compound metabolic process	GO_BiologicalProcess-EBI-UniProt-GOA-ACAP-ARAP_13.05.2021_00h00	1.69E-38	16.625917	68	[AASS, ABCB7, ACACA, ACOT7, ACOT8, ACSF2, ACSL3, ACSL4, ACSS2, APIP, B4GALT1, B4GALT5, BPNT2, CHPF, CHSY3, CLIC1, CSPG4, DCN, DLST, EEF1G, ENPP1, ETHE1, FAM3A, FASN, G6PD, GCDH, GDAP1, GGCT, GLO1, GLRX3, GSTM2, GSTM3, GSTP1, HEXB, HMGCS1, HSD17B4, LUM, MAT2A, MGST1, MMUT, MPST, MTAP, MTR, MTRR, MVD, MVK, NUBP2, NUBPL, OPLAH, PAPSS2, PC, PDK1, PDZD11, PHGDH, PTGES2, SLC1A1, SLC25A10, SLC25A19, SLC26A2, SOAT1, SOD1, STAT5B, SULT1E1, THEM4, TPST2, TSPO, UST, XYLT2]	No change
phosphorus metabolic process	GO_BiologicalProcess-EBI-UniProt-GOA-ACAP-ARAP_13.05.2021_00h00	1.69E-38	10.977199	337	[AASS, ABHD12, ABHD16A, ABI1, ACACA, ACOT7, ACOT8, ACSF2, ACSL3, ACSL4, ACSS2, ACTB, ADAM10, ADARB1, ADK, ADNP, ADSL, AGPAT2, AK1, AK3, AK4, AK6, AKR1B1, ALDH18A1, ALDOA, APEX1, APP, APRT, ARAF, ARMT1, ARPP19, ARRB1, ATP1A1, ATP6V0C, ATP6V1A, B3GAT3, BAZ1B, BCR, BOD1, BPNT2, BST1, CAB39L, CAMK2D, CASK, CASP3, CCN2, CCNH, CCNK, CCNL1, CD109, CD81, CDC42BPA, CDIPT, CDK1, CDK16, CDK17, CDK2, CDK5RAP3, CEMIP, CHAMP1, CHORDC1, CHP1, CKB, CLU, CMPK1, COLEC12, CORO1C, CROT, CSPG4, CTBP1, CTPS1, DAB2, DAPK1, DCAKD, DCXR, DGUOK, DHX34, DIPK2A, DLST, DUSP23, DUSP3, DUT, DVL2, DYNLL1, EFEMP1, EFL1, EIF2AK2, EIF2S1, EIF6, ELP3, ELP4, ENO2, ENPP1, EPHA2, EPHA3,	No change

					EPHB4, ERBB2, ETFDH, EXTL2, FAM3A, FASN, FERMT2, FGF2, FKBP1A, FMR1, FN1, FN3K, FUT8, G6PD, GAPDH, GARS1, GCDH, GFPT2, GLMN, GMPS, GNE, GPD1L, GPD2, GPHN, GPI, GSK3B, GSTP1, GTF2F1, HADHA, HDAC2, HDAC4, HEXB, HGS, HINT1, HK1, HK2, HMGCS1, HPX, HSD17B4, HSP90AA1, IDH2, IDI1, IKBKB, IKBKG, ILF3, ILKAP, IMPA1, IMPACT, INPP4A, IPO5, IQGAP3, ITPK1, LDHA, LLGL1, LPCAT2, LPCAT4, LRRK1, LTBP1, MACROH2A1, MALT1, MAP2K3, MAP3K20, MAP3K4, MAP4K4, MBOAT2, MBOAT7, MCM2, MCM7, ME1, MFN1, MGAT1, MMAB, MOCS2, MVD, MVK, MVP, MYADM, NCK2, NEK1, NF1, NF2, NIBAN1, NNT, NOCT, NOP53, NPM1, NPR2, NT5C, NT5C3B, NT5DC3, NUDT15, NUDT16, NUDT16L1, NUP205, NUP35, NUP62, OSBPL10, OSBPL8, OXSR1, PAFAH1B1, PAICS, PANK4, PAPSS2, PARP1, PARP14, PCYT2, PDE5A, PDK1, PFAS, PFKM, PGAM5, PGM2L1, PGM3, PGP, PHGDH, PHKB, PHPT1, PI4K2B, PIGO, PIGT, PIGU, PIK3C2A, PIK3CA, PIN1, PIP4K2B, PKLR, PKM, PKN2, PLA2G15, PLA2G4A, PLBD2, PLCB3, PLCG1, PLD1, PLPP3, PLXNB2, PML, PNP, PNPLA6, POMK, PPIP5K2, PPM1B, PPP1CA, PPP1R14A, PPP1R7, PPP2R5A, PPP3CA, PPP5C, PRDX6, PRKAA1, PRKAB2, PRKCI, PRKCSH, PRKG1, PRPSAP1, PRPSAP2, PRUNE1, PTGIS, PTK7, PTPA, PTPMT1, PTPN12, PTPRF, PTPRG, PTRH2, PXK, PYCARD, QARS1, RAB23, RAB5A,	

					RAP2B, RAP2C, RIOK3, RIPK2, RNGTT, ROR1, RPLP1, RPRD1A, RPRD1B, RRM1, SAMHD1, SARM1, SESN2, SET, SIRT1, SLC1A1, SLC25A12, SLC25A13, SLC26A2, SLC44A1, SLC44A2, SLIT2, SMAD4, SMPD1, SMYD3, SNF8, SOAT1, SOD1, SPHK2, SPTLC2, SQSTM1, SRC, SRD5A3, STAT2, STK24, SULT1E1, SYNJ1, TAB2, TALDO1, TBK1, TBPL1, TF, TGFBR3, THBS1, THEM4, TIGAR, TIPRL, TJP2, TKT, TMEM119, TNIK, TPI1, TPST2, TPX2, TRIM28, TRIO, TSC2, TSPO, TXN, TYMP, UAP1, UGP2, UMPS, URI1, VLDLR, VPS25, VRK1, VRK3, VTN, XYLB, YES1, ZMPSTE24]	
carbohydrate derivative metabolic process	GO_BiologicalProcess-EBI-UniProt-GOA-ACAP-ARAP_13.05.2021_00h00	1.69E-38	14.053615	173	[AASS, ACACA, ACOT7, ACOT8, ACSF2, ACSL3, ACSL4, ACSS2, ADAMTS9, ADK, ADSL, AGO2, AGRN, AK1, AK3, AK4, AKR1B1, ALDOA, ALG11, APP, APRT, ARFGEF1, ASAH1, ATP6V0C, ATP6V1A, B3GAT3, B4GALT1, B4GALT5, BPNT2, C1GALT1, CASK, CEMIP, CHORDC1, CHP1, CHPF, CHSY3, CLTC, CMPK1, COG7, COL11A1, CROT, CSPG4, CTNNB1, CTPS1, CTSA, DCAKD, DCN, DCXR, DGUOK, DLST, DUT, EFL1, EIF6, ENO2, ENPP1, EOGT, EPHA2, ERP44, ESYT1, EXTL2, FAM3A, FASN, FGF2, FN3K, FUT11, FUT8, G6PD, GALNT1, GALNT2, GALNT7, GAPDH, GBA2, GCDH, GFPT2, GLA, GMPS, GNE, GPC1, GPC6, GPD1L, GPD2, GPI, GXYLT1, GXYLT2, HDAC4, HEXB, HINT1, HK1, HK2, HMGCS1, HSD17B4, HSPG2, ITIH4, ITM2C, LDHA, LUM, MACROD1, MFN1, MGAT1, MOGS, MTAP, MTCH1, MVD, MVK,	No change

					NPR2, NT5C, NUDT15, NUDT16, NUP205, NUP35, NUP62, PAICS, PANK4, PAPSS2, PARG, PARP1, PAWR, PDE5A, PDK1, PFAS, PFKM, PGM2L1, PGM3, PHGDH, PIGO, PIGT, PIGU, PKLR, PKM, PLOD2, PMM2, PNP, POFUT1, POGLUT3, POMGNT1, POMGNT2, POMK, PRKAA1, PRKCSH, PRPSAP1, PRPSAP2, PSMD2, PTRH2, RAB1A, RAB23, RRM1, SAMHD1, SGSH, SLC25A12, SLC25A13, SLC26A2, SLC9A1, SMPD1, SOAT1, SPHK2, SRD5A3, SULT1E1, TALDO1, TBPL1, THEM4, TIGAR, TJP2, TKT, TPI1, TPST2, TSPO, TYMP, UAP1, UGP2, UMPS, UST, XYLB, XYLT2]	
organophosphate metabolic process	GO_BiologicalProcess-EBI-UniProt-GOA-ACAP-ARAP_13.05.2021_00h00	1.69E-38	14.073426	161	[AASS, ABHD12, ABHD16A, ACACA, ACOT7, ACOT8, ACSF2, ACSL3, ACSL4, ACSS2, ADK, ADSL, AGPAT2, AK1, AK3, AK4, AK6, ALDOA, APP, APRT, ATP6V0C, ATP6V1A, BPNT2, CASK, CD81, CDIPT, CHORDC1, CHP1, CKB, CMPK1, CROT, CTPS1, DCAKD, DCXR, DGUOK, DHX34, DLST, DUT, EFL1, EIF6, ENO2, ENPP1, EPHA2, FAM3A, FASN, FGF2, G6PD, GAPDH, GARS1, GCDH, GFPT2, GMPS, GPD1L, GPD2, GPHN, GPI, HADHA, HDAC4, HEXB, HINT1, HK1, HK2, HMGCS1, HSD17B4, IDH2, IDI1, IMPA1, INPP4A, ITPK1, LDHA, LPCAT2, LPCAT4, MBOAT2, MBOAT7, ME1, MFN1, MOCS2, MVD, MVK, NPR2, NT5C, NT5C3B, NUDT15, NUDT16, NUP205, NUP35, NUP62, OSBPL10, OSBPL8, PAFAH1B1, PAICS, PANK4, PAPSS2, PARP1, PCYT2, PDE5A, PDK1, PFAS, PFKM, PGM2L1, PGM3, PGP, PHGDH,	No change

					PI4K2B, PIGO, PIGT, PIGU, PIK3C2A, PIK3CA, PIP4K2B, PKLR, PKM, PLA2G15, PLA2G4A, PLBD2, PLCB3, PLCG1, PLD1, PLPP3, PNP, PNPLA6, PPIP5K2, PRDX6, PRKAA1, PRKCSH, PRPSAP1, PRPSAP2, PTGIS, PTPMT1, PTRH2, RAB23, RAB5A, RRM1, SAMHD1, SARM1, SLC25A12, SLC25A13, SLC26A2, SLC44A1, SLC44A2, SMPD1, SOAT1, SPHK2, SPTLC2, SRC, SRD5A3, SULT1E1, SYNJ1, TALDO1, TBPL1, THEM4, TIGAR, TJP2, TKT, TPI1, TPST2, TSPO, TYMP, UGP2, UMPS, XYLB]	
cellular lipid metabolic process	GO_BiologicalProcess-EBI-UniProt-GOA-ACAP-ARAP_13.05.2021_00h00	1.69E-38	11.580727	137	[ABHD12, ABHD16A, ACACA, ACADM, ACADSB, ACADVL, ACAT2, ACOT7, ACOT8, ACOX1, ACSF2, ACSL3, ACSL4, ACSS2, AGPAT2, AGPS, AGRN, AKR1B1, ALDH3A2, ALG11, APLP2, APPL2, ASAH1, B4GALT5, BDH2, BPNT2, CBR1, CD81, CDIPT, CERS6, CHORDC1, CHP1, CRABP2, CROT, CTSA, DAGLB, DHRS7B, ECI2, EIF6, EPHX1, ERLIN2, ESYT1, ETFDH, FAM3A, FASN, FGF2, GBA2, GCDH, GLA, GPC1, GPC6, GPD1L, GSTM2, GSTP1, HACL1, HADH, HADHA, HEXB, HMGCS1, HSD17B10, HSD17B4, HSPG2, IDI1, ILVBL, IMPA1, INPP4A, KDSR, LPCAT2, LPCAT4, LSS, LYPLA2, MBOAT2, MBOAT7, MMUT, MTCH1, MVD, MVK, NUDT19, OSBPL10, OSBPL8, PAFAH1B1, PCK2, PCYT2, PDK1, PDXDC1, PGP, PI4K2B, PIGO, PIGT, PIGU, PIK3C2A, PIK3CA, PIP4K2B, PLA2G15, PLA2G4A, PLBD2, PLCB3, PLCG1, PLD1, PLPP3, PNPLA4, PNPLA6, PPP2R5A, PPP3CA,	No change

					PRDX6, PRKAA1, PRKAB2, PRKCSH, PRXL2B, PTGES2, PTGIS, PTGR1, PTPMT1, RAB5A, RBP1, RDH10, RDH11, RETSAT, SCP2, SCPEP1, SESN2, SIRT1, SLC44A1, SLC44A2, SMPD1, SOAT1, SPHK2, SPTLC1, SPTLC2, SRC, SRD5A3, SYNJ1, TBCE, THEM4, TSPO, VDAC2, ZMPSTE24]	
phosphate-containing compound metabolic process	GO_BiologicalProcess-EBI-UniProt-GOA-ACAP-ARAP_13.05.2021_00h00	1.69E-38	10.943149	333	[AASS, ABHD12, ABHD16A, ABI1, ACACA, ACOT7, ACOT8, ACSF2, ACSL3, ACSL4, ACSS2, ACTB, ADAM10, ADARB1, ADK, ADNP, ADSL, AGPAT2, AK1, AK3, AK4, AK6, AKR1B1, ALDH18A1, ALDOA, APEX1, APP, APRT, ARAF, ARMT1, ARPP19, ARRB1, ATP1A1, ATP6V0C, ATP6V1A, B3GAT3, BAZ1B, BCR, BOD1, BPNT2, BST1, CAB39L, CAMK2D, CASK, CASP3, CCN2, CCNH, CCNK, CCNL1, CD109, CD81, CDC42BPA, CDIPT, CDK1, CDK16, CDK17, CDK2, CDK5RAP3, CEMIP, CHAMP1, CHORDC1, CHP1, CKB, CLU, CMPK1, COLEC12, CORO1C, CROT, CSPG4, CTBP1, CTPS1, DAB2, DAPK1, DCAKD, DCXR, DGUOK, DHX34, DIPK2A, DLST, DUSP23, DUSP3, DUT, DVL2, DYNLL1, EFEMP1, EFL1, EIF2AK2, EIF2S1, EIF6, ELP3, ELP4, ENO2, ENPP1, EPHA2, EPHA3, EPHB4, ERBB2, ETFDH, FAM3A, FASN, FERMT2, FGF2, FKBP1A, FMR1, FN1, FN3K, G6PD, GAPDH, GARS1, GCDH, GFPT2, GLMN, GMPS, GNE, GPD1L, GPD2, GPHN, GPI, GSK3B, GSTP1, GTF2F1, HADHA, HDAC2, HDAC4, HEXB, HGS, HINT1, HK1, HK2, HMGCS1, HPX, HSD17B4, HSP90AA1, IDH2,	No change

					IDI1, IKBKB, IKBKG, ILF3, ILKAP, IMPA1, IMPACT, INPP4A, IPO5, IQGAP3, ITPK1, LDHA, LLGL1, LPCAT2, LPCAT4, LRRK1, LTBP1, MACROH2A1, MALT1, MAP2K3, MAP3K20, MAP3K4, MAP4K4, MBOAT2, MBOAT7, MCM2, MCM7, ME1, MFN1, MMAB, MOCS2, MVD, MVK, MVP, MYADM, NCK2, NEK1, NF1, NF2, NIBAN1, NNT, NOCT, NOP53, NPM1, NPR2, NT5C, NT5C3B, NT5DC3, NUDT15, NUDT16, NUDT16L1, NUP205, NUP35, NUP62, OSBPL10, OSBPL8, OXSR1, PAFAH1B1, PAICS, PANK4, PAPSS2, PARP1, PARP14, PCYT2, PDE5A, PDK1, PFAS, PFKM, PGAM5, PGM2L1, PGM3, PGP, PHGDH, PHKB, PHPT1, PI4K2B, PIGO, PIGT, PIGU, PIK3C2A, PIK3CA, PIN1, PIP4K2B, PKLR, PKM, PKN2, PLA2G15, PLA2G4A, PLBD2, PLCB3, PLCG1, PLD1, PLPP3, PLXNB2, PML, PNP, PNPLA6, POMK, PPIP5K2, PPM1B, PPP1CA, PPP1R14A, PPP1R7, PPP2R5A, PPP3CA, PPP5C, PRDX6, PRKAA1, PRKAB2, PRKCI, PRKCSH, PRKG1, PRPSAP1, PRPSAP2, PRUNE1, PTGIS, PTK7, PTPA, PTPMT1, PTPN12, PTPRF, PTPRG, PTRH2, PXK, PYCARD, QARS1, RAB23, RAB5A, RAP2B, RAP2C, RIOK3, RIPK2, RNGTT, ROR1, RPLP1, RPRD1A, RPRD1B, RRM1, SAMHD1, SARM1, SESN2, SET, SIRT1, SLC1A1, SLC25A12, SLC25A13, SLC26A2, SLC44A1, SLC44A2, SLIT2, SMAD4, SMPD1, SMYD3, SNF8, SOAT1, SOD1, SPHK2, SPTLC2, SQSTM1, SRC, SRD5A3, STAT2, STK24, SULT1E1,	

					SYNJ1, TAB2, TALDO1, TBK1, TBPL1, TF, TGFBR3, THBS1, THEM4, TIGAR, TIPRL, TJP2, TKT, TMEM119, TNIK, TPI1, TPST2, TPX2, TRIM28, TRIO, TSC2, TSPO, TXN, TYMP, UGP2, UMPS, URI1, VLDLR, VPS25, VRK1, VRK3, VTN, XYLB, YES1, ZMPSTE24]	
lipid biosynthetic process	GO_BiologicalProcess-EBI-UniProt-GOA-ACAP-ARAP_13.05.2021_00h00	1.69E-38	11.971831	102	[ACACA, ACADVL, ACAT2, ACOT7, ACOT8, ACSL3, ACSL4, ACSS2, AGPAT2, AGPS, AKR1B1, ALDH3A2, ASAH1, ATP1A1, B4GALT5, BPNT2, CBR1, CD81, CDIPT, CERS6, CHORDC1, CHP1, CYB5R3, CYP51A1, DAGLB, DHCR24, DHCR7, DHRS7B, EIF6, ERLIN2, FAM3A, FASN, FDXR, FGF2, G6PD, GPD1L, GSTM2, GSTP1, HEXB, HMGCS1, HSD17B10, HSD17B11, HSD17B4, IDI1, IGFBP7, IMPA1, INPP4A, KDSR, KPNB1, LBR, LPCAT2, LPCAT4, LSS, MBOAT2, MBOAT7, MVD, MVK, NSDHL, OSBPL1A, OSBPL2, PCK2, PCYT2, PI4K2B, PIGO, PIGT, PIGU, PIK3C2A, PIK3CA, PIP4K2B, PLA2G15, PLA2G4A, PLD1, PLPP3, PPP3CA, PRKAA1, PRKAB2, PRKCSH, PRPF19, PRXL2B, PTGES2, PTGIS, PTPMT1, RAB5A, RBP1, RDH10, SCP2, SEC14L2, SF1, SIRT1, SLC44A1, SLC44A2, SMPD1, SOD1, SPHK2, SPTLC1, SPTLC2, SRC, SRD5A3, SYNJ1, TBCE, TSPO, VDAC2]	No change
nucleobase-containing small molecule metabolic process	GO_BiologicalProcess-EBI-UniProt-GOA-ACAP-ARAP_13.05.2021_00h00	1.69E-38	16.592592	112	[AASS, ACACA, ACOT7, ACOT8, ACSF2, ACSL3, ACSL4, ACSS2, ADK, ADSL, AK1, AK3, AK4, AK6, ALDOA, APP, APRT, ATP6V0C, ATP6V1A, BPNT2, CASK, CMPK1, CROT, CTPS1, DCAKD, DGUOK, DLST, DPYSL2, DPYSL3, DPYSL4, DUT, EFL1,	No change

					EIF6, ENO2, ENPP1, EPHA2, EXTL2, FAM3A, FASN, FUT8, GAPDH, GARS1, GCDH, GFPT2, GMPS, GNE, GPI, HDAC4, HINT1, HK1, HK2, HMGCS1, HSD17B4, IDH2, KDM1A, LDHA, MACROD1, ME1, MFN1, MGAT1, MTAP, MVD, MVK, NPR2, NT5C, NT5C3B, NUDT15, NUDT16, NUP205, NUP35, NUP62, PAICS, PANK4, PAPSS2, PARG, PARP1, PDE5A, PDK1, PFAS, PFKM, PGM3, PKLR, PKM, PMM2, PNP, PRKAA1, PRPSAP1, PRPSAP2, PTGIS, PTRH2, RAB23, RRM1, SAMHD1, SARM1, SHMT2, SLC25A12, SLC25A13, SLC26A2, SOAT1, SPHK2, SULT1E1, TBPL1, THEM4, TIGAR, TJP2, TPI1, TPST2, TSPO, TYMP, UAP1, UGP2, UMPS]	
purine-containing compound metabolic process	GO_BiologicalProcess-EBI-UniProt-GOA-ACAP-ARAP_13.05.2021_00h00	1.69E-38	16.831684	85	[AASS, ACACA, ACOT7, ACOT8, ACSF2, ACSL3, ACSL4, ACSS2, ADK, ADSL, AK1, AK3, AK4, ALDOA, APP, APRT, ATP6V0C, ATP6V1A, BPNT2, CASK, CROT, DCAKD, DGUOK, DLST, EFL1, EIF6, ENO2, ENPP1, EPHA2, FAM3A, FASN, GAPDH, GCDH, GMPS, GPI, HDAC4, HINT1, HK1, HK2, HMGCS1, HSD17B4, KDM1A, LDHA, MACROD1, MFN1, MTAP, MVD, MVK, NPR2, NT5C, NUDT15, NUDT16, NUP205, NUP35, NUP62, PAICS, PANK4, PAPSS2, PARP1, PDE5A, PDK1, PFAS, PFKM, PKLR, PKM, PNP, PRKAA1, PRPSAP1, PRPSAP2, PTRH2, RAB23, SAMHD1, SHMT2, SLC25A12, SLC25A13, SLC26A2, SOAT1, SPHK2, SULT1E1, THEM4, TIGAR, TJP2, TPI1, TPST2, TSPO]	No change
carbohydrate derivative biosynthetic process	GO_BiologicalProcess-EBI-UniProt-GOA-	1.69E-38	14.540466	106	[ACACA, ACOT7, ACOT8, ACSL3, ACSL4, ACSS2, ADK,	No change

	ACAP-ARAP_13.05.2021_00h00				ADSL, AGO2, AGRN, AK1, AK3, AK4, ALDOA, ALG11, APRT, ARFGEF1, ATP6V0C, ATP6V1A, B3GAT3, B4GALT1, B4GALT5, C1GALT1, CEMIP, CHORDC1, CHP1, CHPF, CHSY3, CLTC, CMPK1, COG7, CSPG4, CTNNB1, CTPS1, DCAKD, DCN, DCXR, DGUOK, DUT, EOGT, EXTL2, FAM3A, FASN, FUT11, FUT8, G6PD, GALNT1, GALNT2, GALNT7, GCDH, GFPT2, GMPS, GNE, GPC1, GPC6, GXYLT1, GXYLT2, HSPG2, ITM2C, LUM, MGAT1, MOGS, MTAP, MVD, NPR2, PAICS, PANK4, PAPSS2, PARP1, PAWR, PDK1, PFAS, PGM3, PIGO, PIGT, PIGU, PKLR, PLOD2, PMM2, PNP, POFUT1, POGLUT3, POMGNT1, POMGNT2, POMK, PRKAA1, PRPSAP1, PRPSAP2, PSMD2, RRM1, SLC25A12, SLC25A13, SLC26A2, SOAT1, SPHK2, SRD5A3, TBPL1, TKT, TPI1, TYMP, UAP1, UGP2, UMPS, UST, XYLB, XYLT2]	
organonitrogen compound biosynthetic process	GO_BiologicalProcess-EBI-UniProt-GOA-ACAP-ARAP_13.05.2021_00h00	1.69E-38	14.787234	278	[AARS1, AASDHPPT, AASS, ABAT, ABCB7, ABCE1, ACACA, ACADM, ACO1, ACOT7, ACOT8, ACSL3, ACSL4, ACSS2, ADK, ADSL, AGO2, AGRN, AK1, AK3, AK4, ALB, ALDH18A1, ALDH3A2, ALDOA, ALG11, APIP, APP, APRT, ARFGEF1, ASAH1, ASNS, ATG7, ATP6V0C, ATP6V1A, B3GAT3, B4GALT1, B4GALT5, BCAT1, BDH2, C1GALT1, C1QBP, CEMIP, CERS6, CHORDC1, CHP1, CHPF, CHSY3, CIRBP, CKB, CLTC, CMPK1, CNOT1, CNOT10, COG7, CSPG4, CTNNB1, CTPS1, DAPK1, DARS1, DCAKD, DCN, DCPS, DDX1, DENR, DGUOK, DHFR, DHPS, DHX36, DRG1, DUT, EARS2, EEF1E1, EEF1G, EFL1, EIF2AK2, EIF2B1,	No change

					EIF2D, EIF2S1, EIF3A, EIF3B, EIF3E, EIF3F, EIF4G3, EIF6, EOGT, EPRS1, ERBB2, EXTL2, FAM3A, FASN, FMR1, FTO, FUT11, FUT8, GALNT1, GALNT2, GALNT7, GAMT, GAPDH, GARS1, GATB, GCDH, GFPT2, GGCT, GLE1, GLUD1, GLUL, GMPS, GOLGA7, GOLT1B, GOT1, GPC1, GPC6, GPHN, GSTM2, GSTM3, GSTP1, GXYLT1, GXYLT2, HARS1, HMBS, HNRNPR, HSPG2, IARS2, IDH2, IGF2BP1, IGF2BP2, ILF3, ILVBL, IMPACT, IREB2, ITM2C, KDSR, LARP1, LARP4B, LARP6, LPCAT2, LRPPRC, LUM, METTL14, MGAT1, MGST1, MLH1, MOCS2, MOGS, MOV10, MRPL10, MRPS17, MRPS21, MRPS31, MRRF, MTAP, MTR, MTRR, MVD, N6AMT1, NCK2, NCL, NDRG1, NIBAN1, NMT1, NOCT, NPM1, NPR2, NSUN5, NT5C3B, OAT, OGFOD1, OPLAH, OXA1L, PAICS, PAIP1, PANK4, PAPSS2, PARP1, PAWR, PDK1, PELO, PFAS, PGM3, PHGDH, PIGO, PIGT, PIGU, PKLR, PKM, PLA2G4A, PLD1, PLOD2, PLPP3, PLXNB2, PMM2, PNP, POFUT1, POGLUT3, POMGNT1, POMGNT2, POMK, PPM1B, PPP1CA, PPP3CA, PRKAA1, PRKCSH, PRPSAP1, PRPSAP2, PSAT1, PSMD2, PTGIS, PUS7, PYCR1, PYCR3, QARS1, QDPR, RARS2, RBM4, RPL10A, RPL13, RPL13A, RPL18A, RPL22, RPL24, RPL27, RPL32, RPL36, RPL38, RPL5, RPLP1, RPLP2, RPS13, RPS15A, RPS16, RPS18, RPS21, RPS23, RPS4Y1, RPS5, RPS8, RPS9, SARNP, SARS1, SBDS, SELENOT, SESN2, SHFL,	

					SHMT2, SLC11A2, SLC1A1, SLC25A12, SLC25A13, SLC25A15, SLC26A2, SLC44A1, SLC44A2, SMPD1, SOAT1, SPHK2, SPTLC1, SPTLC2, SRD5A3, SRR, TBCE, TBPL1, THBS1, TRMT10C, TSPO, TUT4, TYMP, UMPS, UST, VARS1, WIPI2, XYLT2, YARS1, YBX1, ZDHHC13, ZDHHC21, ZFP36L2]	
ribose phosphate metabolic process	GO_BiologicalProcess-EBI-UniProt-GOA-ACAP-ARAP_13.05.2021_00h00	1.69E-38	16.8357	83	[AASS, ACACA, ACOT7, ACOT8, ACSF2, ACSL3, ACSL4, ACSS2, ADK, ADSL, AK1, AK3, AK4, ALDOA, APP, APRT, ATP6V0C, ATP6V1A, BPNT2, CASK, CMPK1, CROT, CTPS1, DCAKD, DGUOK, DLST, EFL1, EIF6, ENO2, ENPP1, EPHA2, FAM3A, FASN, G6PD, GAPDH, GCDH, GMPS, GPI, HDAC4, HINT1, HK1, HK2, HMGCS1, HSD17B4, LDHA, MFN1, MVD, MVK, NPR2, NT5C, NUDT16, NUP205, NUP35, NUP62, PAICS, PANK4, PAPSS2, PARP1, PDE5A, PDK1, PFAS, PFKM, PKLR, PKM, PRKAA1, PRPSAP1, PRPSAP2, PTRH2, RAB23, SLC25A12, SLC25A13, SLC26A2, SOAT1, SPHK2, SULT1E1, THEM4, TIGAR, TJP2, TKT, TPI1, TPST2, TSPO, UMPS]	No change
organophosphate biosynthetic process	GO_BiologicalProcess-EBI-UniProt-GOA-ACAP-ARAP_13.05.2021_00h00	1.69E-38	13.7447405	98	[ACACA, ACOT7, ACSL3, ACSL4, ACSS2, ADK, ADSL, AGPAT2, AK1, AK3, AK4, AK6, ALDOA, APRT, ATP6V0C, ATP6V1A, BPNT2, CASK, CD81, CDIPT, CHORDC1, CHP1, CKB, CMPK1, CTPS1, DCAKD, DCXR, DGUOK, DHX34, DUT, FAM3A, FASN, FGF2, G6PD, GARS1, GCDH, GMPS, GPD1L, GPHN, HEXB, HMGCS1, IDH2, IDI1, IMPA1, INPP4A, LPCAT2, LPCAT4, MBOAT2, MBOAT7, ME1, MOCS2, MVD, MVK, NPR2, PAICS, PANK4, PAPSS2, PARP1, PCYT2, PDK1,	No change

					PFAS, PI4K2B, PIGO, PIGT, PIGU, PIK3C2A, PIK3CA, PIP4K2B, PKLR, PLA2G4A, PLD1, PNP, PPIP5K2, PRKCSH, PRPSAP1, PRPSAP2, PTGIS, PTPMT1, PTRH2, RAB5A, RRM1, SLC25A12, SLC25A13, SLC26A2, SLC44A1, SLC44A2, SOAT1, SPHK2, SPTLC2, SRC, SRD5A3, SYNJ1, TBPL1, TJP2, TKT, TPI1, UMPS, XYLB]	
purine-containing compound biosynthetic process	GO_BiologicalProcess-EBI-UniProt-GOA-ACAP-ARAP_13.05.2021_00h00	1.69E-38	16.666666	38	[ACACA, ACOT7, ACSL3, ACSL4, ACSS2, ADK, ADSL, AK1, AK3, AK4, ALDOA, APRT, ATP6V0C, ATP6V1A, DCAKD, DGUOK, FAM3A, FASN, GCDH, GMPS, MTAP, NPR2, PAICS, PANK4, PAPSS2, PARP1, PDK1, PFAS, PKLR, PNP, PRPSAP1, PRPSAP2, SHMT2, SLC25A12, SLC25A13, SLC26A2, SOAT1, SPHK2]	No change
ribose phosphate biosynthetic process	GO_BiologicalProcess-EBI-UniProt-GOA-ACAP-ARAP_13.05.2021_00h00	1.69E-38	17.316017	40	[ACACA, ACOT7, ACSL3, ACSL4, ACSS2, ADK, ADSL, AK1, AK3, AK4, ALDOA, APRT, ATP6V0C, ATP6V1A, CMPK1, CTPS1, DCAKD, DGUOK, FAM3A, FASN, G6PD, GCDH, GMPS, NPR2, PAICS, PANK4, PAPSS2, PARP1, PDK1, PFAS, PKLR, PRPSAP1, PRPSAP2, SLC25A12, SLC25A13, SLC26A2, SOAT1, SPHK2, TKT, UMPS]	No change
nucleotide metabolic process	GO_BiologicalProcess-EBI-UniProt-GOA-ACAP-ARAP_13.05.2021_00h00	1.69E-38	16.351118	95	[AASS, ACACA, ACOT7, ACOT8, ACSF2, ACSL3, ACSL4, ACSS2, ADK, ADSL, AK1, AK3, AK4, AK6, ALDOA, APP, APRT, ATP6V0C, ATP6V1A, BPNT2, CASK, CMPK1, CROT, CTPS1, DCAKD, DGUOK, DLST, DUT, EFL1, EIF6, ENO2, ENPP1, EPHA2, FAM3A, FASN, GAPDH, GARS1, GCDH, GMPS, GPI, HDAC4, HINT1, HK1, HK2, HMGCS1, HSD17B4, IDH2, LDHA, ME1, MFN1, MVD, MVK, NPR2, NT5C, NT5C3B, NUDT15, NUDT16,	No change

					NUP205, NUP35, NUP62, PAICS, PANK4, PAPSS2, PARP1, PDE5A, PDK1, PFAS, PFKM, PKLR, PKM, PNP, PRKAA1, PRPSAP1, PRPSAP2, PTGIS, PTRH2, RAB23, RRM1, SAMHD1, SARM1, SLC25A12, SLC25A13, SLC26A2, SOAT1, SPHK2, SULT1E1, TBPL1, THEM4, TIGAR, TJP2, TPI1, TPST2, TSPO, TYMP, UMPS]	
nucleoside triphosphate metabolic process	GO_BiologicalProcess-EBI-UniProt-GOA-ACAP-ARAP_13.05.2021_00h00	1.69E-38	20.168068	24	[ADK, AK1, AK3, AK4, ALDOA, ATP6V0C, ATP6V1A, CMPK1, CTPS1, DGUOK, DUT, EFL1, ENPP1, MFN1, NUDT15, NUDT16, PARP1, PKLR, RAB23, SAMHD1, SLC25A12, SLC25A13, SPHK2, TBPL1]	Downregulation
purine nucleotide metabolic process	GO_BiologicalProcess-EBI-UniProt-GOA-ACAP-ARAP_13.05.2021_00h00	1.69E-38	16.735537	81	[AASS, ACACA, ACOT7, ACOT8, ACSF2, ACSL3, ACSL4, ACSS2, ADK, ADSL, AK1, AK3, AK4, ALDOA, APP, APRT, ATP6V0C, ATP6V1A, BPNT2, CASK, CROT, DCAKD, DGUOK, DLST, EFL1, EIF6, ENO2, ENPP1, EPHA2, FAM3A, FASN, GAPDH, GCDH, GMPS, GPI, HDAC4, HINT1, HK1, HK2, HMGCS1, HSD17B4, LDHA, MFN1, MVD, MVK, NPR2, NT5C, NUDT15, NUDT16, NUP205, NUP35, NUP62, PAICS, PANK4, PAPSS2, PARP1, PDE5A, PDK1, PFAS, PFKM, PKLR, PKM, PNP, PRKAA1, PRPSAP1, PRPSAP2, PTRH2, RAB23, SAMHD1, SLC25A12, SLC25A13, SLC26A2, SOAT1, SPHK2, SULT1E1, THEM4, TIGAR, TJP2, TPI1, TPST2, TSPO]	No change
ribonucleotide metabolic process	GO_BiologicalProcess-EBI-UniProt-GOA-ACAP-ARAP_13.05.2021_00h00	1.69E-38	16.458334	79	[AASS, ACACA, ACOT7, ACOT8, ACSF2, ACSL3, ACSL4, ACSS2, ADK, ADSL, AK1, AK3, AK4, ALDOA, APP, APRT, ATP6V0C, ATP6V1A, BPNT2, CASK, CMPK1, CROT, CTPS1, DCAKD, DGUOK, DLST, EFL1, EIF6, ENO2, ENPP1, EPHA2, FAM3A,	No change

					FASN, GAPDH, GCDH, GMPS, GPI, HDAC4, HINT1, HK1, HK2, HMGCS1, HSD17B4, LDHA, MFN1, MVD, MVK, NPR2, NT5C, NUDT16, NUP205, NUP35, NUP62, PAICS, PANK4, PAPSS2, PARP1, PDE5A, PDK1, PFAS, PFKM, PKLR, PKM, PRKAA1, PTRH2, RAB23, SLC25A12, SLC25A13, SLC26A2, SOAT1, SPHK2, SULT1E1, THEM4, TIGAR, TJP2, TPI1, TPST2, TSPO, UMPS]	
purine nucleoside bisphosphate metabolic process	GO_BiologicalProcess-EBI-UniProt-GOA-ACAP-ARAP_13.05.2021_00h00	1.69E-38	19.463087	29	[AASS, ACACA, ACOT7, ACOT8, ACSF2, ACSL3, ACSL4, ACSS2, BPNT2, CROT, DCAKD, DLST, ENPP1, FAM3A, FASN, GCDH, HMGCS1, HSD17B4, MVD, MVK, PANK4, PAPSS2, PDK1, SLC26A2, SOAT1, SULT1E1, THEM4, TPST2, TSPO]	No change
nucleotide biosynthetic process	GO_BiologicalProcess-EBI-UniProt-GOA-ACAP-ARAP_13.05.2021_00h00	1.69E-38	16.498316	49	[ACACA, ACOT7, ACSL3, ACSL4, ACSS2, ADK, ADSL, AK1, AK3, AK4, AK6, ALDOA, APRT, ATP6V0C, ATP6V1A, CASK, CMPK1, CTPS1, DCAKD, DGUOK, DUT, FAM3A, FASN, GARS1, GCDH, GMPS, IDH2, ME1, NPR2, PAICS, PANK4, PAPSS2, PARP1, PDK1, PFAS, PKLR, PNP, PRPSAP1, PRPSAP2, PTGIS, RRM1, SLC25A12, SLC25A13, SLC26A2, SOAT1, SPHK2, TBPL1, TJP2, UMPS]	No change
ribonucleoside bisphosphate metabolic process	GO_BiologicalProcess-EBI-UniProt-GOA-ACAP-ARAP_13.05.2021_00h00	1.69E-38	19.463087	29	[AASS, ACACA, ACOT7, ACOT8, ACSF2, ACSL3, ACSL4, ACSS2, BPNT2, CROT, DCAKD, DLST, ENPP1, FAM3A, FASN, GCDH, HMGCS1, HSD17B4, MVD, MVK, PANK4, PAPSS2, PDK1, SLC26A2, SOAT1, SULT1E1, THEM4, TPST2, TSPO]	No change
purine nucleotide biosynthetic process	GO_BiologicalProcess-EBI-UniProt-GOA-ACAP-ARAP_13.05.2021_00h00	1.69E-38	16.203703	35	[ACACA, ACOT7, ACSL3, ACSL4, ACSS2, ADK, ADSL, AK1, AK3, AK4, ALDOA, APRT, ATP6V0C, ATP6V1A,	No change

					DCAKD, DGUOK, FAM3A, FASN, GCDH, GMPS, NPR2, PAICS, PANK4, PAPSS2, PARP1, PDK1, PFAS, PKLR, PRPSAP1, PRPSAP2, SLC25A12, SLC25A13, SLC26A2, SOAT1, SPHK2]	
purine ribonucleotide metabolic process	GO_BiologicalProcess-EBI-UniProt-GOA-ACAP-ARAP_13.05.2021_00h00	1.69E-38	16.37931	76	[AASS, ACACA, ACOT7, ACOT8, ACSF2, ACSL3, ACSL4, ACSS2, ADK, ADSL, AK1, AK3, AK4, ALDOA, APP, APRT, ATP6V0C, ATP6V1A, BPNT2, CASK, CROT, DCAKD, DGUOK, DLST, EFL1, EIF6, ENO2, ENPP1, EPHA2, FAM3A, FASN, GAPDH, GCDH, GMPS, GPI, HDAC4, HINT1, HK1, HK2, HMGCS1, HSD17B4, LDHA, MFN1, MVD, MVK, NPR2, NT5C, NUDT16, NUP205, NUP35, NUP62, PAICS, PANK4, PAPSS2, PARP1, PDE5A, PDK1, PFAS, PFKM, PKLR, PKM, PRKAA1, PTRH2, RAB23, SLC25A12, SLC25A13, SLC26A2, SOAT1, SPHK2, SULT1E1, THEM4, TIGAR, TJP2, TPI1, TPST2, TSPO]	No change
ribonucleotide biosynthetic process	GO_BiologicalProcess-EBI-UniProt-GOA-ACAP-ARAP_13.05.2021_00h00	1.69E-38	16.289593	36	[ACACA, ACOT7, ACSL3, ACSL4, ACSS2, ADK, ADSL, AK1, AK3, AK4, ALDOA, APRT, ATP6V0C, ATP6V1A, CMPK1, CTPS1, DCAKD, DGUOK, FAM3A, FASN, GCDH, GMPS, NPR2, PAICS, PANK4, PAPSS2, PARP1, PDK1, PFAS, PKLR, SLC25A12, SLC25A13, SLC26A2, SOAT1, SPHK2, UMPS]	No change
organic acid metabolic process	GO_BiologicalProcess-EBI-UniProt-GOA-ACAP-ARAP_13.05.2021_00h00	6.83E-28	15.424165	180	[AARS1, AASDHPPT, AASS, ABAT, ABCC1, ACACA, ACAD8, ACADM, ACADSB, ACADVL, ACAT2, ACO1, ACOT7, ACOT8, ACOX1, ACSF2, ACSL3, ACSL4, ACSS2, ADSL, AKR1B1, ALDH18A1, ALDH3A2, ALDOA, APIP, APLP2, APP, APPL2, ASAH1, ASNS, BCAT1, BDH2, BPNT2, CBR1, CKB, CRABP2, CROT,	No change

					CTPS1, CYB5R3, DAGLB, DARS1, DCXR, DGLUCY, DHFR, DLST, EARS2, ECI2, EEF1E1, EIF6, ENO2, ENPP1, EPHX1, EPRS1, ERLIN2, ETFDH, FAM3A, FASN, FN3K, GAMT, GAPDH, GARS1, GATB, GBA2, GCAT, GCDH, GFPT2, GLO1, GLUD1, GLUL, GMPS, GNE, GOLT1B, GOT1, GPI, GSTM2, GSTP1, HACL1, HADH, HADHA, HARS1, HDAC4, HIBCH, HK1, HK2, HSD17B10, HSD17B4, IARS2, IDH2, ILVBL, IREB2, LDHA, LYPLA2, ME1, MMUT, MPST, MTAP, MTHFD2, MTR, MTRR, N6AMT1, NQO1, NUDT19, NUP205, NUP35, NUP62, OAT, OSBPL1A, OSBPL2, PAPSS2, PC, PCK2, PDK1, PDXDC1, PDZD11, PFAS, PFKM, PHGDH, PKLR, PKM, PLA2G15, PLA2G4A, PLOD2, PRKAA1, PRKAB2, PRUNE1, PRXL2B, PSAT1, PSMA3, PSMB10, PSMB2, PSMC2, PSMC3, PSMC6, PSMD1, PSMD12, PSMD14, PSMD2, PSMD6, PSMD7, PSMD8, PSME1, PSME2, PSME4, PTGES2, PTGIS, PTGR1, PYCR1, PYCR3, QARS1, QDPR, RARS2, RBP1, RDH10, SARS1, SBDS, SCLY, SCP2, SCPEP1, SESN2, SHMT2, SIRT1, SLC16A3, SLC25A12, SLC25A13, SLC26A2, SLC2A1, SOAT1, SRR, STAT5B, SULT1E1, THEM4, TIGAR, TPI1, TPST2, UGP2, VARS1, VDAC1, VDAC2, XYLB, YARS1]	
lipid metabolic process	GO_BiologicalProcess-EBI-UniProt-GOA-ACAP-ARAP_13.05.2021_00h00	6.83E-28	11.417323	174	[ABHD12, ABHD16A, ACACA, ACAD8, ACADM, ACADSB, ACADVL, ACAT2, ACOT7, ACOT8, ACOX1, ACSF2, ACSL3, ACSL4, ACSS2, AGPAT2, AGPS, AGRN, AKR1B1, ALDH3A2, ALG11, APLP2, APP,	No change

					APPL2, ASAH1, ATP1A1, B4GALT5, BDH2, BPNT2, CBR1, CD81, CDIPT, CERS6, CHORDC1, CHP1, CLU, CRABP2, CROT, CTSA, CYB5R3, CYP51A1, DAGLB, DHCR24, DHCR7, DHRS7B, ECI2, EDF1, EIF6, EPHX1, ERLIN2, ESYT1, ETFDH, FAM3A, FASN, FDXR, FGF2, G6PD, GBA2, GCDH, GLA, GPC1, GPC6, GPD1L, GSTM2, GSTP1, HACL1, HADH, HADHA, HEXB, HMGCS1, HSD17B10, HSD17B11, HSD17B4, HSPG2, IDI1, IGFBP7, ILVBL, IMPA1, INPP4A, KDSR, KPNB1, LACTB, LBR, LIPA, LPCAT2, LPCAT4, LRP10, LSS, LYPLA2, MACROH2A1, MBOAT2, MBOAT7, MMUT, MTCH1, MVD, MVK, NSDHL, NUDT19, OSBPL10, OSBPL1A, OSBPL2, OSBPL8, PAFAH1B1, PAFAH1B2, PAFAH1B3, PC, PCK2, PCYT2, PDK1, PDXDC1, PGP, PI4K2B, PIGO, PIGT, PIGU, PIK3C2A, PIK3CA, PIP4K2B, PLA2G15, PLA2G4A, PLBD2, PLCB3, PLCG1, PLD1, PLD3, PLPP3, PNPLA4, PNPLA6, PPP2R5A, PPP3CA, PRDX6, PRKAA1, PRKAB2, PRKCSH, PRPF19, PRXL2B, PTGES2, PTGIS, PTGR1, PTPMT1, RAB5A, RBP1, RDH10, RDH11, RETSAT, SCP2, SCPEP1, SEC14L2, SESN2, SF1, SIRT1, SLC44A1, SLC44A2, SMARCD3, SMPD1, SOAT1, SOD1, SPHK2, SPTLC1, SPTLC2, SRC, SRD5A3, STAT5B, SULT1E1, SYNJ1, TBCE, TGS1, THEM4, TPP1, TSPO, TTC39B, VDAC2, VLDLR, ZMPSTE24]	
small molecule catabolic process	GO_BiologicalProcess-EBI-UniProt-GOA-ACAP-ARAP_13.05.2021_00h00	6.83E-28	16.041666	77	[AASS, ABAT, ACAD8, ACADM, ACADSB, ACADVL, ACAT2, ACOT8, ACOX1, ALDH2, ALDH3A2, ALDOA,	No change

					BCAT1, BDH2, BPNT2, CROT, DCXR, DLST, ECI2, ENO2, ETFDH, FAM3A, FUT8, GALE, GALM, GAPDH, GCAT, GCDH, GLUD1, GLUL, GOLT1B, GOT1, GPD2, GPI, HACL1, HADH, HADHA, HIBCH, HK1, HK2, HSD17B10, HSD17B4, ILVBL, IMPA1, MGAT1, MMUT, MPST, MTAP, MTRR, NT5C, NUDT15, NUDT16, NUDT19, OAT, PCK2, PFKM, PGM2L1, PHGDH, PKLR, PKM, PLA2G15, PNP, QDPR, SBDS, SCLY, SCP2, SESN2, SHMT2, SLC25A12, SOAT1, SRD5A3, SULT1E1, SYNJ1, TIGAR, TPI1, TYMP, XYLB]	
small molecule biosynthetic process	GO_BiologicalProcess-EBI-UniProt-GOA-ACAP-ARAP_13.05.2021_00h00	6.83E-28	16.942148	123	[AASDHPPT, AASS, ABAT, ACACA, ACADM, ACADVL, ACAT2, ACOT7, ACOT8, ACSL3, ACSS2, ADK, ADSL, AKR1B1, ALDH18A1, ALDOA, APIP, APRT, ARPP19, ASAH1, ASNS, BCAT1, BDH2, CBR1, COQ5, CYB5R3, CYP51A1, DAGLB, DHCR24, DHCR7, DHFR, DHX34, DUT, EIF6, ENO2, ENPP1, ERLIN2, FAM3A, FASN, FDXR, FGF2, G6PD, GAMT, GAPDH, GLUD1, GLUL, GOLT1B, GOT1, GPI, GSTM2, GSTP1, HINT1, HMGCS1, HSD17B10, HSD17B4, IDI1, ILVBL, IMPA1, KPNB1, LBR, LSS, MAEA, MTAP, MTR, MTRR, MVD, MVK, NSDHL, NT5C, NUDT15, NUDT16, OAT, OSBPL1A, OSBPL2, PC, PCK2, PDE5A, PGP, PHGDH, PKLR, PLA2G4A, PLOD2, PNP, PPIP5K2, PRKAA1, PRKAB2, PRXL2B, PSAT1, PTGES2, PTGIS, PTRH2, PYCR1, PYCR3, QDPR, RBP1, RDH10, SAMHD1, SARM1, SBDS, SCP2, SEC14L2, SESN2, SHMT2, SIRT1,	No change

					SLC25A10, SLC25A12, SLC25A13, SOAT1, SOD1, SPHK2, SPTLC1, SPTLC2, SRD5A3, SRR, TALDO1, TKT, TPI1, TYMP, UGP2, UMPS, USP7, VDAC2, WDR5]	
organic hydroxy compound metabolic process	GO_BiologicalProcess-EBI-UniProt-GOA-ACAP-ARAP_13.05.2021_00h00	6.83E-28	13.322369	81	[ABAT, ACADVL, ACAT2, ACOT8, ACSS2, AKR1B1, ALDH2, ALDH3A2, APP, ASAH1, BPNT2, CYB5R3, CYP51A1, DHCR24, DHCR7, DHFR, DHX34, ERLIN2, FDXR, FGF2, G6PD, GBA2, GIPC1, GOLT1B, GOT1, GPD2, HMGCS1, HSD17B10, HSD17B4, HSP90AA1, IDH2, IDI1, IMPA1, INPP4A, ITPK1, KPNB1, LBR, LDHA, LIPA, LSS, MAOA, MVD, MVK, NSDHL, OSBPL1A, OSBPL2, PCK2, PGP, PLA2G4A, PLCB3, PLCG1, PLPP3, PNPLA4, PPIP5K2, PRKAA1, PSAT1, PTGR1, PTRH2, QDPR, RDH10, RDH11, RETSAT, SCP2, SEC14L2, SIRT1, SLC1A1, SLC25A12, SMPD1, SOAT1, SOD1, SPHK2, SPTLC1, SPTLC2, SRD5A3, SULT1E1, SYNJ1, TIGAR, TPI1, TTC39B, VDAC2, VLDLR]	No change
alcohol metabolic process	GO_BiologicalProcess-EBI-UniProt-GOA-ACAP-ARAP_13.05.2021_00h00	6.83E-28	15.892421	65	[ACADVL, ACAT2, ACSS2, AKR1B1, ALDH2, ALDH3A2, APP, ASAH1, BPNT2, CYB5R3, CYP51A1, DHCR24, DHCR7, DHFR, DHX34, ERLIN2, FDXR, FGF2, G6PD, GBA2, GOLT1B, GOT1, GPD2, HMGCS1, HSP90AA1, IDH2, IDI1, IMPA1, INPP4A, ITPK1, KPNB1, LBR, LSS, MVD, MVK, NSDHL, OSBPL1A, PCK2, PGP, PLA2G4A, PLCB3, PLCG1, PLPP3, PNPLA4, PPIP5K2, PRKAA1, PTRH2, QDPR, RDH10, RDH11, RETSAT, SEC14L2, SMPD1, SOAT1, SOD1, SPHK2, SPTLC1, SPTLC2, SRD5A3, SULT1E1, SYNJ1,	No change

					TPI1, TTC39B, VDAC2, VLDLR]	
lipid catabolic process	GO_BiologicalProcess-EBI-UniProt-GOA-ACAP-ARAP_13.05.2021_00h00	6.83E-28	14.130435	52	[ABHD12, ABHD16A, ACADM, ACADVL, ACAT2, ACOT8, ACOX1, ALDH3A2, ASAH1, BDH2, CROT, DAGLB, ECI2, ETFDH, GBA2, GCDH, GLA, HACL1, HADH, HADHA, HEXB, HSD17B10, HSD17B11, HSD17B4, ILVBL, LIPA, LYPLA2, MMUT, NUDT19, PAFAH1B1, PAFAH1B2, PAFAH1B3, PCK2, PDXDC1, PLA2G15, PLA2G4A, PLBD2, PLCB3, PLCG1, PLD1, PLD3, PNPLA4, PNPLA6, PRDX6, PRKAA1, PRKCSH, SCP2, SESN2, SMPD1, SOAT1, SRD5A3, SULT1E1]	Downregulation
organic acid catabolic process	GO_BiologicalProcess-EBI-UniProt-GOA-ACAP-ARAP_13.05.2021_00h00	6.83E-28	16.376307	47	[AASS, ABAT, ACAD8, ACADM, ACADSB, ACADVL, ACAT2, ACOT8, ACOX1, ALDH3A2, BCAT1, BDH2, CROT, DCXR, DLST, ECI2, ETFDH, FAM3A, GCAT, GCDH, GLUD1, GLUL, GOLT1B, GOT1, HACL1, HADH, HADHA, HIBCH, HSD17B10, HSD17B4, ILVBL, MMUT, MPST, MTRR, NUDT19, OAT, PCK2, PHGDH, PLA2G15, QDPR, SBDS, SCLY, SCP2, SESN2, SHMT2, SOAT1, XYLB]	No change
oxoacid metabolic process	GO_BiologicalProcess-EBI-UniProt-GOA-ACAP-ARAP_13.05.2021_00h00	6.83E-28	15.669014	178	[AARS1, AASDHPPT, AASS, ABAT, ABCC1, ACACA, ACAD8, ACADM, ACADSB, ACADVL, ACAT2, ACO1, ACOT7, ACOT8, ACOX1, ACSF2, ACSL3, ACSL4, ACSS2, ADSL, AKR1B1, ALDH18A1, ALDH3A2, ALDOA, APIP, APLP2, APP, APPL2, ASAH1, ASNS, BCAT1, BDH2, BPNT2, CBR1, CKB, CRABP2, CROT, CTPS1, CYB5R3, DAGLB, DARS1, DCXR, DGLUCY, DHFR, DLST, EARS2, ECI2, EEF1E1, EIF6, ENO2, ENPP1, EPHX1, EPRS1, ERLIN2, ETFDH, FAM3A, FASN, FN3K,	No change

					GAMT, GAPDH, GARS1, GATB, GBA2, GCAT, GCDH, GFPT2, GLO1, GLUD1, GLUL, GMPS, GNE, GOLT1B, GOT1, GPI, GSTM2, GSTP1, HACL1, HADH, HADHA, HARS1, HDAC4, HIBCH, HK1, HK2, HSD17B10, HSD17B4, IARS2, IDH2, ILVBL, IREB2, LDHA, LYPLA2, ME1, MMUT, MPST, MTAP, MTHFD2, MTR, MTRR, NQO1, NUDT19, NUP205, NUP35, NUP62, OAT, OSBPL1A, OSBPL2, PAPSS2, PC, PCK2, PDK1, PDXDC1, PDZD11, PFAS, PFKM, PHGDH, PKLR, PKM, PLA2G15, PLA2G4A, PLOD2, PRKAA1, PRKAB2, PRUNE1, PRXL2B, PSAT1, PSMA3, PSMB10, PSMB2, PSMC2, PSMC3, PSMC6, PSMD1, PSMD12, PSMD14, PSMD2, PSMD6, PSMD7, PSMD8, PSME1, PSME2, PSME4, PTGES2, PTGIS, PTGR1, PYCR1, PYCR3, QARS1, QDPR, RARS2, RBP1, RDH10, SARS1, SBDS, SCLY, SCP2, SCPEP1, SESN2, SHMT2, SIRT1, SLC16A3, SLC25A12, SLC25A13, SLC26A2, SLC2A1, SOAT1, SRR, SULT1E1, THEM4, TIGAR, TPI1, TPST2, UGP2, VARS1, VDAC1, VDAC2, XYLB, YARS1]	
organic hydroxy compound biosynthetic process	GO_BiologicalProcess-EBI-UniProt-GOA-ACAP-ARAP_13.05.2021_00h00	6.83E-28	16.728624	45	[ACAT2, ACOT8, AKR1B1, ASAH1, CYB5R3, CYP51A1, DHCR24, DHCR7, DHFR, DHX34, ERLIN2, FGF2, G6PD, GIPC1, GOLT1B, GOT1, HMGCS1, HSD17B10, HSD17B4, IDI1, IMPA1, KPNB1, LBR, LSS, MVD, MVK, NSDHL, OSBPL1A, OSBPL2, PCK2, PGP, PPIP5K2, PRKAA1, PSAT1, PTRH2, QDPR, SCP2, SEC14L2, SIRT1, SOD1, SPHK2, SPTLC1, SPTLC2, SRD5A3, VDAC2]	Upregulation

cellular amino acid metabolic process	GO_BiologicalProcess-EBI-UniProt-GOA-ACAP-ARAP_13.05.2021_00h00	6.83E-28	21.02426	78	[AARS1, AASDHPPT, AASS, ABAT, ACAD8, ACADSB, ADSL, ALDH18A1, APIP, ASNS, BCAT1, CTPS1, DARS1, DGLUCY, DHFR, DLST, EARS2, EEF1E1, EPRS1, FAM3A, FN3K, GARS1, GATB, GCAT, GCDH, GFPT2, GLUD1, GLUL, GMPS, GOLT1B, GOT1, HARS1, HIBCH, HSD17B10, IARS2, ILVBL, MMUT, MPST, MTAP, MTR, MTRR, NQO1, OAT, PFAS, PHGDH, PLOD2, PSAT1, PSMA3, PSMB10, PSMB2, PSMC2, PSMC3, PSMC6, PSMD1, PSMD12, PSMD14, PSMD2, PSMD6, PSMD7, PSMD8, PSME1, PSME2, PSME4, PYCR1, PYCR3, QARS1, QDPR, RARS2, SARS1, SBDS, SCLY, SHMT2, SLC25A12, SLC25A13, SOAT1, SRR, VARS1, YARS1]	No change
cellular lipid catabolic process	GO_BiologicalProcess-EBI-UniProt-GOA-ACAP-ARAP_13.05.2021_00h00	6.83E-28	16.334661	41	[ABHD12, ABHD16A, ACADM, ACADVL, ACAT2, ACOT8, ACOX1, ALDH3A2, ASAH1, BDH2, CROT, DAGLB, ECI2, ETFDH, GBA2, GCDH, GLA, HACL1, HADH, HADHA, HEXB, HSD17B10, HSD17B4, ILVBL, LYPLA2, MMUT, NUDT19, PCK2, PDXDC1, PLA2G15, PLA2G4A, PLBD2, PLCG1, PNPLA4, PNPLA6, PRDX6, SCP2, SESN2, SMPD1, SOAT1, SRD5A3]	Downregulation
alcohol biosynthetic process	GO_BiologicalProcess-EBI-UniProt-GOA-ACAP-ARAP_13.05.2021_00h00	6.83E-28	22.08589	36	[ACAT2, AKR1B1, ASAH1, CYB5R3, CYP51A1, DHCR24, DHCR7, DHFR, DHX34, ERLIN2, FGF2, G6PD, GOLT1B, GOT1, HMGCS1, IDI1, IMPA1, KPNB1, LBR, LSS, MVD, MVK, NSDHL, PCK2, PGP, PPIP5K2, PRKAA1, PTRH2, QDPR, SEC14L2, SOD1, SPHK2, SPTLC1, SPTLC2, SRD5A3, VDAC2]	Upregulation
steroid biosynthetic process	GO_BiologicalProcess-EBI-UniProt-GOA-	6.83E-28	17.58794	35	[ACAT2, ACOT8, AKR1B1, ASAH1, ATP1A1, CYB5R3,	No change

	ACAP-ARAP_13.05.2021_00h00				CYP51A1, DHCR24, DHCR7, ERLIN2, FDXR, G6PD, HMGCS1, HSD17B10, HSD17B11, HSD17B4, IDI1, IGFBP7, KPNB1, LBR, LSS, MVD, MVK, NSDHL, OSBPL1A, OSBPL2, PRKAA1, SCP2, SEC14L2, SF1, SIRT1, SOD1, SRD5A3, TSPO, VDAC2]	
carboxylic acid metabolic process	GO_BiologicalProcess-EBI-UniProt-GOA-ACAP-ARAP_13.05.2021_00h00	6.83E-28	15.503173	171	[AARS1, AASDHPPT, AASS, ABAT, ABCC1, ACACA, ACAD8, ACADM, ACADSB, ACADVL, ACAT2, ACO1, ACOT7, ACOT8, ACOX1, ACSF2, ACSL3, ACSL4, ACSS2, ADSL, AKR1B1, ALDH18A1, ALDH3A2, ALDOA, APIP, APLP2, APP, APPL2, ASAH1, ASNS, BCAT1, BDH2, CBR1, CKB, CRABP2, CROT, CTPS1, CYB5R3, DAGLB, DARS1, DCXR, DGLUCY, DHFR, DLST, EARS2, ECI2, EEF1E1, EIF6, ENO2, EPHX1, EPRS1, ERLIN2, ETFDH, FAM3A, FASN, FN3K, GAMT, GAPDH, GARS1, GATB, GBA2, GCAT, GCDH, GFPT2, GLO1, GLUD1, GLUL, GMPS, GNE, GOLT1B, GOT1, GPI, GSTM2, GSTP1, HACL1, HADH, HADHA, HARS1, HDAC4, HIBCH, HK1, HK2, HSD17B10, HSD17B4, IARS2, IDH2, ILVBL, IREB2, LDHA, LYPLA2, ME1, MMUT, MPST, MTAP, MTHFD2, MTR, MTRR, NQO1, NUDT19, NUP205, NUP35, NUP62, OAT, OSBPL1A, OSBPL2, PC, PCK2, PDK1, PDXDC1, PDZD11, PFAS, PFKM, PHGDH, PKLR, PKM, PLA2G15, PLA2G4A, PLOD2, PRKAA1, PRKAB2, PRXL2B, PSAT1, PSMA3, PSMB10, PSMB2, PSMC2, PSMC3, PSMC6, PSMD1, PSMD12, PSMD14, PSMD2, PSMD6, PSMD7, PSMD8, PSME1, PSME2, PSME4, PTGES2,	No change

					PTGIS, PTGR1, PYCR1, PYCR3, QARS1, QDPR, RARS2, RBP1, RDH10, SARS1, SBDS, SCLY, SCP2, SCPEP1, SESN2, SHMT2, SIRT1, SLC16A3, SLC25A12, SLC25A13, SLC2A1, SOAT1, SRR, THEM4, TIGAR, TPI1, UGP2, VARS1, VDAC1, VDAC2, XYLB, YARS1]	
alpha-amino acid metabolic process	GO_BiologicalProcess-EBI-UniProt-GOA-ACAP-ARAP_13.05.2021_00h00	6.83E-28	18.534483	43	[AASDHPPT, AASS, ACADSB, ADSL, ALDH18A1, APIP, ASNS, CTPS1, DGLUCY, DHFR, DLST, FAM3A, GCAT, GCDH, GFPT2, GLUD1, GLUL, GMPS, GOLT1B, GOT1, HSD17B10, ILVBL, MMUT, MPST, MTAP, MTR, MTRR, OAT, PFAS, PHGDH, PLOD2, PSAT1, PYCR1, PYCR3, QDPR, SARS1, SBDS, SCLY, SHMT2, SLC25A12, SLC25A13, SOAT1, SRR]	Upregulation
cellular amino acid biosynthetic process	GO_BiologicalProcess-EBI-UniProt-GOA-ACAP-ARAP_13.05.2021_00h00	6.83E-28	35.61644	26	[AASDHPPT, AASS, ABAT, ADSL, ALDH18A1, APIP, ASNS, BCAT1, DHFR, GLUD1, GLUL, GOLT1B, GOT1, ILVBL, MTAP, MTR, MTRR, OAT, PHGDH, PLOD2, PSAT1, PYCR1, PYCR3, SHMT2, SLC25A12, SRR]	Upregulation
carboxylic acid biosynthetic process	GO_BiologicalProcess-EBI-UniProt-GOA-ACAP-ARAP_13.05.2021_00h00	6.83E-28	16.964285	57	[AASDHPPT, AASS, ABAT, ACACA, ACADVL, ACOT7, ACOT8, ACSL3, ACSS2, ADSL, ALDH18A1, APIP, ASNS, BCAT1, CBR1, DAGLB, DHFR, EIF6, ERLIN2, FASN, GAMT, GLUD1, GLUL, GOLT1B, GOT1, GSTM2, GSTP1, HSD17B10, HSD17B4, ILVBL, MTAP, MTR, MTRR, OAT, OSBPL1A, OSBPL2, PHGDH, PKLR, PLA2G4A, PLOD2, PRKAA1, PRKAB2, PRXL2B, PSAT1, PTGES2, PTGIS, PYCR1, PYCR3, RBP1, RDH10, SBDS, SCP2, SHMT2, SIRT1, SLC25A12, SRR, UGP2]	Upregulation

carboxylic acid catabolic process	GO_BiologicalProcess-EBI-UniProt-GOA-ACAP-ARAP_13.05.2021_00h00	6.83E-28	15.925926	43	[AASS, ABAT, ACAD8, ACADM, ACADVL, ACAT2, ACOT8, ACOX1, ALDH3A2, BDH2, CROT, DCXR, DLST, ECI2, ETFDH, FAM3A, GCAT, GCDH, GLUD1, GLUL, GOLT1B, GOT1, HACL1, HADH, HADHA, HIBCH, HSD17B10, HSD17B4, ILVBL, MMUT, MTRR, NUDT19, OAT, PCK2, PHGDH, PLA2G15, QDPR, SBDS, SCP2, SESN2, SHMT2, SOAT1, XYLB]	No change
monocarboxylic acid metabolic process	GO_BiologicalProcess-EBI-UniProt-GOA-ACAP-ARAP_13.05.2021_00h00	6.83E-28	13.642756	97	[AASDHPPT, ABAT, ACACA, ACADM, ACADSB, ACADVL, ACAT2, ACOT7, ACOT8, ACOX1, ACSF2, ACSL3, ACSL4, ACSS2, AKR1B1, ALDH3A2, ALDOA, APLP2, APP, APPL2, ASAH1, BDH2, CBR1, CKB, CRABP2, CROT, DAGLB, DCXR, ECI2, EIF6, ENO2, EPHX1, ERLIN2, ETFDH, FAM3A, FASN, GAMT, GAPDH, GBA2, GCDH, GLO1, GPI, GSTM2, GSTP1, HACL1, HADH, HADHA, HDAC4, HK1, HK2, HSD17B10, HSD17B4, IDH2, ILVBL, LDHA, LYPLA2, ME1, MMUT, NUDT19, NUP205, NUP35, NUP62, OSBPL1A, OSBPL2, PC, PCK2, PDK1, PDZD11, PFKM, PHGDH, PKLR, PKM, PLA2G15, PLA2G4A, PRKAA1, PRKAB2, PRXL2B, PTGES2, PTGIS, PTGR1, RBP1, RDH10, SBDS, SCP2, SCPEP1, SESN2, SIRT1, SLC16A3, SLC25A12, SOAT1, SRR, THEM4, TIGAR, TPI1, VDAC1, VDAC2, XYLB]	No change
aspartate family amino acid metabolic process	GO_BiologicalProcess-EBI-UniProt-GOA-ACAP-ARAP_13.05.2021_00h00	6.83E-28	27.692308	18	[AASDHPPT, AASS, APIP, ASNS, DLST, FAM3A, GCAT, GCDH, GOLT1B, GOT1, MTAP, MTR, MTRR, PHGDH, PLOD2, SBDS, SLC25A12, SLC25A13]	Upregulation
alpha-amino acid biosynthetic process	GO_BiologicalProcess-EBI-UniProt-GOA-	6.83E-28	32.05128	25	[AASDHPPT, AASS, ADSL, ALDH18A1, APIP, ASNS, DHFR,	Upregulation

	ACAP-ARAP_13.05.2021_00h00				GLUD1, GLUL, GOLT1B, GOT1, ILVBL, MTAP, MTR, MTRR, OAT, PHGDH, PLOD2, PSAT1, PYCR1, PYCR3, SBDS, SHMT2, SLC25A12, SRR]	
cholesterol biosynthetic process	GO_BiologicalProcess-EBI-UniProt-GOA-ACAP-ARAP_13.05.2021_00h00	6.83E-28	28.35821	19	[ACAT2, CYB5R3, CYP51A1, DHCR24, DHCR7, ERLIN2, G6PD, HMGCS1, IDI1, KPNB1, LBR, LSS, MVD, MVK, NSDHL, PRKAA1, SEC14L2, SOD1, VDAC2]	Upregulation
fatty acid catabolic process	GO_BiologicalProcess-EBI-UniProt-GOA-ACAP-ARAP_13.05.2021_00h00	6.83E-28	20.689655	24	[ACADM, ACADVL, ACAT2, ACOT8, ACOX1, ALDH3A2, BDH2, CROT, ECI2, ETFDH, GCDH, HACL1, HADH, HADHA, HSD17B10, HSD17B4, ILVBL, MMUT, NUDT19, PCK2, PLA2G15, SCP2, SESN2, SOAT1]	Downregulation
fatty acid oxidation	GO_BiologicalProcess-EBI-UniProt-GOA-ACAP-ARAP_13.05.2021_00h00	6.83E-28	20.512821	24	[ACADM, ACADVL, ACAT2, ACOT8, ACOX1, ALDH3A2, APLP2, APPL2, BDH2, CROT, ECI2, ETFDH, GCDH, HACL1, HADH, HADHA, HSD17B10, HSD17B4, ILVBL, PRKAA1, SCP2, SESN2, SOAT1, VDAC2]	No change
monocarboxylic acid catabolic process	GO_BiologicalProcess-EBI-UniProt-GOA-ACAP-ARAP_13.05.2021_00h00	6.83E-28	18.881119	27	[ACADM, ACADVL, ACAT2, ACOT8, ACOX1, ALDH3A2, BDH2, CROT, DCXR, ECI2, ETFDH, GCDH, HACL1, HADH, HADHA, HSD17B10, HSD17B4, ILVBL, MMUT, NUDT19, PCK2, PHGDH, PLA2G15, SCP2, SESN2, SOAT1, XYLB]	No change
aspartate family amino acid biosynthetic process	GO_BiologicalProcess-EBI-UniProt-GOA-ACAP-ARAP_13.05.2021_00h00	6.83E-28	41.666668	10	[AASDHPPT, AASS, APIP, ASNS, GOLT1B, GOT1, MTAP, MTR, MTRR, PLOD2]	Upregulation
cytoskeletal protein binding	GO_MolecularFunction-EBI-UniProt-GOA-ACAP-ARAP_13.05.2021_00h00	2.81E-43	13.832853	144	[ABI1, ACTB, ACTR2, ACTR3, ADD3, ADNP, AFDN, ALDOA, ANK3, APEX1, ARFGEF1, ARPC4, ARPC5L, ATP1A1, B4GALT1, BIN1, CALD1, CAMK2D, CAP1, CAP2, CAPZA2, CETN2, CFL2, CHORDC1, CHP1, CLIP2, CLU, CNN1, CNN3, COBLL1, CORO1B, CORO1C, CORO2B, CORO7, CSRP2, DIAPH1, DIAPH3,	No change

					DNM1L, DPYSL2, DPYSL3, DPYSL4, DRG1, DST, DUSP3, EML1, EML3, EML4, EPB41L2, EPB41L3, EZR, FARP1, FBLIM1, FERMT1, FERMT2, FGD4, FHOD1, FLII, FMR1, FSCN1, FSD1, GAPDH, GBP1, GIPC1, GSK3B, HIP1, HSP90AA1, IMPACT, IQGAP3, ITGB1, KCNMA1, KIF15, KIRREL1, LARP6, LIN7C, LLGL1, LMO7, LMOD1, LRPPRC, MACF1, MAEA, MAGI1, MAP1B, MAP1LC3A, MAP4K4, MICAL3, MSN, MYH10, MYH9, MYO18A, MYO1B, MYO1D, MYO1E, MYO6, NCAPG, NCK2, NDRG1, NF2, NUP62, PACSIN3, PAFAH1B1, PALLD, PANX1, PAWR, PIN1, PLEC, PPP5C, PRKAA1, PRUNE1, PXK, PXN, PYCARD, RAB10, RAB11B, RAB3B, RAB3D, RAB8A, RAI14, RALA, RDX, REEP2, SBDS, SERPINB8, SMC3, SNX5, SORBS3, SVIL, SYNE2, SYNPO, TAGLN, TBCA, TBCE, TBCEL, TLN2, TOR1AIP1, TOR1B, TPM3, TPX2, TTLL12, TUBGCP2, TWF2, VASP, VPS41, WDR1, ZNF207]	
cellular component organization	GO_BiologicalProcess-EBI-UniProt-GOA-ACAP-ARAP_13.05.2021_00h00	2.81E-43	10.981818	755	[AAR2, AASS, ABCB7, ABCC1, ABCE1, ABI1, ABT1, ACACA, ACOT8, ACSL3, ACSL4, ACTB, ACTG1, ACTR2, ACTR3, ADAM10, ADAMTS9, ADARB1, ADD3, ADNP, AFDN, AGO2, AGRN, AHCTF1, AKR1B1, ALB, ALCAM, ALDH3A2, ALDOA, AMOTL1, ANK3, ANKFY1, ANKRD13A, ANO6, ANXA7, APBB2, APEX1, APIP, APLP2, APP, APPL2, ARFGAP1, ARFGEF1, ARFIP1, ARHGAP12, ARHGAP17, ARHGEF11, ARID1B, ARPC4, ARPC5L, ARPIN, ARRB1, ASH2L, ASPM, ATG7, ATL3, ATP2C1, ATP6V0A1, ATP6V0C, ATP6V1A,	No change

					ATP6V1B2, ATPAF1, ATXN10, AUP1, B2M, B4GALT1, B4GALT5, BAIAP2, BANF1, BAZ1B, BCR, BET1, BIN1, BMP1, BNIP1, BOD1, BRD3, BST1, C1QBP, C2CD2L, CALD1, CAMK2D, CAP1, CAP2, CAPZA2, CASP3, CBX2, CBX3, CBX8, CCN2, CCNH, CCT2, CCT3, CCT6A, CDC34, CDC42BPA, CDC42EP1, CDC73, CDH11, CDK1, CDK16, CDK2, CDK5RAP3, CECR2, CELF2, CENPV, CETN2, CFAP20, CFAP298, CFL2, CHAF1B, CHAMP1, CHD1L, CHD2, CHD4, CHD7, CHMP3, CHORDC1, CHP1, CHTOP, CIRBP, CLGN, CLIP2, CLTC, CLTCL1, CLU, CNGB1, CNN1, CNN3, CNOT1, COG7, COIL, COL11A1, COL12A1, COL15A1, COL18A1, COL4A1, COL5A1, COL5A2, COL6A1, COLEC12, CORO1B, CORO1C, CORO2B, CORO7, COX20, CRABP2, CRBN, CRTAP, CRYZ, CSPG4, CSRP2, CTBP1, CTNNB1, CTSC, CUL1, CUL4B, CUX1, CXCL12, CYCS, CYRIB, DAB2, DARS1, DCBLD2, DCN, DDX1, DENR, DGUOK, DHCR24, DHFR, DHX36, DIAPH1, DIAPH3, DICER1, DNM1L, DNMT1, DPYSL2, DPYSL3, DPYSL4, DRG1, DSP, DST, DUSP3, DVL2, DYM, DYNC1LI1, DYNLL1, EED, EFL1, EFNB2, EHD3, EIF2AK2, EIF2D, EIF2S1, EIF3A, EIF3B, EIF3E, EIF3F, EIF6, EMC1, EMG1, EML1, EML3, EML4, ENO2, ENPP1, ENY2, EPB41L2, EPB41L3, EPHA2, EPHA3, EPHB4, EPPK1, EPRS1, ERBB2, ERCC5, ERCC6L, ESYT1, EXOC2, EZR, FAM171A1, FAM91A1, FARP1, FASN, FAT1, FBLIM1, FBLN2, FBXW8, FEN1, FERMT1, FERMT2, FGD4,	

					FGF2, FHL1, FHOD1, FKBP1A, FLII, FLRT2, FMR1, FN1, FNBP1L, FSCN1, FSD1, G6PD, GAB1, GAN, GAPDH, GARS1, GBA2, GCC2, GCFC2, GDAP1, GEMIN4, GET3, GGA2, GGCT, GLE1, GLMN, GLRX3, GLYR1, GNL3L, GPC1, GPC6, GPHN, GRAMD1A, GRWD1, GSDMD, GSK3B, GTF2E1, GTF2F2, GTF3C4, GULP1, H1-10, H1-5, HACL1, HAT1, HDAC2, HDAC4, HDGFL2, HEXB, HGS, HIP1, HIRIP3, HK2, HMOX1, HNRNPC, HSD17B10, HSP90AA1, HSPA4, HSPA5, HSPB11, HSPG2, IFT140, IGF2BP1, IGFBP7, IKBKB, IKBKG, IMPACT, IQGAP3, IST1, ITGA3, ITGAV, ITGB1, ITGB5, ITM2C, ITSN1, JAGN1, JAM3, KAT7, KAT8, KCNMA1, KCTD12, KCTD15, KDM1A, KDM3B, KDM4B, KNTC1, KPNB1, KRT18, KRT19, KRT8, LAMA5, LAMC1, LBR, LEMD2, LIG3, LIMS1, LLGL1, LMOD1, LPCAT2, LRCH3, LRCH4, LRRC8A, LRRC8E, LRWD1, LUM, LYPLA2, M6PR, MACF1, MACROH2A1, MAD1L1, MAD2L1, MAEA, MAGI1, MAP1B, MAP1LC3A, MAP3K20, MAP3K4, MAP4K4, MARCHF5, MAT2A, MBD3, MCM2, MCM3, MCM4, MCM5, MCM6, MCM7, MCMBP, MFF, MFN1, MICAL3, MICALL1, MLF2, MLH1, MMAB, MORF4L1, MOV10, MPHOSPH8, MRPL10, MRPS17, MRPS21, MRPS31, MRRF, MRTO4, MSH2, MSH6, MSN, MTA1, MTCH1, MTR, MYADM, MYD88, MYH10, MYH9, MYO18A, MYO1B, MYO1D, MYO1E, MYO6, MYOF, N6AMT1, NCAPD2, NCAPG, NCAPH,	

					NCDN, NCK2, NCKAP1, NCLN, NDRG1, NDUFA13, NDUFAF7, NDUFS3, NECTIN2, NEDD1, NEDD4, NEK1, NES, NF1, NF2, NFKB2, NIBAN2, NISCH, NLE1, NMT1, NOCT, NOP53, NPLOC4, NPM1, NUBP2, NUBPL, NUDT19, NUF2, NUP205, NUP35, NUP62, OBSL1, OGFOD1, ORC4, ORC5, OSBPL2, OSBPL8, OTUD6B, OXA1L, P3H1, PACSIN3, PAFAH1B1, PALLD, PARG, PARL, PARP1, PAWR, PDCD6IP, PDS5B, PDZD11, PDZRN3, PELO, PHGDH, PHLDB1, PHPT1, PI4K2B, PIEZO1, PIGU, PIK3C2A, PIK3CA, PIN1, PIP4K2B, PITRM1, PKN2, PLCG1, PLD1, PLEC, PLOD2, PLXNB2, PML, POGZ, POLD1, POLD2, PPFIBP1, PPIB, PPP1CA, PPP1R7, PPP3CA, PPP5C, PRDX6, PRIM2, PRKAA1, PRKCI, PRKCSH, PRKG1, PRMT7, PRPF19, PRUNE1, PSIP1, PSMA3, PSMB10, PSMB2, PSMC2, PSMC3, PSMC6, PSMD1, PSMD12, PSMD14, PSMD2, PSMD6, PSMD7, PSMD8, PSME1, PSME2, PSME4, PSMG1, PTGES2, PTK7, PTPA, PTPRF, PTPRG, PUF60, PXN, PYCARD, QSOX1, RAB10, RAB1A, RAB23, RAB3B, RAB5A, RAB8A, RAC3, RAD21, RALA, RANBP1, RANGAP1, RAP1GDS1, RAP2B, RAP2C, RBBP4, RCC1, RDX, RECQL, REEP2, REEP6, RFC2, RFC3, RFC5, RHOB, RHOC, RIC8A, RIF1, RIOK3, RIPK2, RNF2, RNF213, RNF40, ROR1, RPA1, RPA2, RPF2, RPL13A, RPL24, RPL38, RPL5, RPS23, RPS5, RRM1, RTN4, RTTN, RYBP, S100A10, SACS, SAE1, SAMHD1, SARM1, SBDS,	

					SCAF11, SCARB2, SCFD1, SCP2, SCRIB, SEC14L2, SEC23B, SEC61B, SEMA3C, SEPTIN11, SEPTIN2, SEPTIN7, SEPTIN8, SEPTIN9, SERPINB8, SERPINE2, SESN2, SET, SEZ6L2, SF1, SF3A3, SH3BP4, SH3KBP1, SHFL, SHMT2, SHPRH, SIRT1, SLC11A2, SLC12A2, SLC1A1, SLC1A5, SLC25A15, SLC25A4, SLC25A5, SLC25A6, SLC2A1, SLC9A1, SLIT2, SMAD4, SMARCA1, SMARCAD1, SMARCB1, SMARCC1, SMARCD3, SMARCE1, SMC1A, SMC2, SMC3, SMC4, SMCHD1, SMPD1, SMYD3, SNF8, SNRNP200, SNRPD3, SNRPE, SNU13, SNW1, SNX1, SNX18, SOAT1, SOD1, SOD2, SON, SORBS3, SORT1, SPARC, SPART, SPECC1, SPHK2, SQSTM1, SRC, SRSF10, SRSF5, SSRP1, STAG2, STARD5, STAT2, STK24, STON1, SUPT16H, SURF1, SVIL, SYNE2, SYNJ1, SYNPO, SYPL2, TAF6L, TAP1, TBC1D10A, TBC1D17, TBC1D22B, TBC1D4, TBCA, TBCE, TBCEL, TBPL1, TCP1, TDRD3, TENM3, TF, TFAM, TGFBI, TGFBR3, TGS1, THBS1, THEM4, THOC2, TIGAR, TIMM17B, TIMMDC1, TJP1, TJP2, TLN2, TM9SF4, TMED9, TMEM97, TNIK, TOM1L2, TOMM34, TOMM7, TOR1AIP1, TOR1AIP2, TOR1B, TPBG, TPM3, TPP1, TPX2, TRADD, TRAM1, TRAM2, TRAPPC4, TRAPPC8, TRIM28, TRIO, TRIP12, TRIP13, TRIP6, TSC2, TSPO, TSPYL1, TTC39C, TTLL12, TUBA4A, TUBA8, TUBB, TUBB3, TUBGCP2, TWF2, TYMP, UBA2, UBE2L6, UBR2, UBTF, UBXN2A, UNC119B, URI1,	

					USP22, USP4, USP7, USP9X, UST, UTP3, VASP, VAT1, VDAC1, VDAC2, VLDLR, VPS25, VPS33B, VPS36, VPS41, VRK1, VTN, WASHC5, WDR1, WDR11, WDR35, WDR5, WDR70, WIPI2, WRAP73, WRNIP1, YAP1, YEATS4, YKT6, YTHDC1, ZFAND6, ZFP36L2, ZMPSTE24, ZMYM3, ZNF148, ZNF207]	
non-membrane-bounded organelle	GO_CellularComponent-EBI-UniProt-GOA-ACAP-ARAP_13.05.2021_00h00	2.81E-43	10.723709	569	[ABI1, ABT1, ACACA, ACADVL, ACSL3, ACSL4, ACTB, ACTG1, ACTR2, ACTR3, ADARB1, ADD3, ADNP, AGO2, AGPS, AHCTF1, AK1, AK3, AK6, AKAP12, AKR1B1, ALDOA, ANK3, APEX1, APP, ARFGEF1, ARID1B, ARL14EP, ARPC4, ARPC5L, ARRB1, ASPM, ATG7, AUP1, BAIAP2, BANF1, BAZ1B, BCAS2, BCR, BIN1, BMS1, BOD1, C1QBP, CALD1, CANX, CAP1, CAPZA2, CASK, CBX1, CBX2, CBX3, CBX5, CBX8, CC2D1A, CCDC102A, CCT2, CCT3, CCT6A, CDC42BPA, CDC42EP1, CDC5L, CDC73, CDK1, CDK16, CDK2, CDK5RAP3, CECR2, CENPV, CETN2, CFAP20, CFAP298, CFDP1, CFL2, CHAF1B, CHAMP1, CHD4, CHD7, CHORDC1, CHP1, CHTOP, CIRBP, CLIP2, CLTC, CLTCL1, CLU, CMPK1, CNGB1, CNN3, CNOT1, COG7, COIL, CORO1B, CORO1C, CORO2B, CSRP2, CSTB, CTNNB1, CTNNBL1, CTSC, CUX1, CYB5R3, DAB2, DAPK1, DCPS, DCXR, DDI2, DDX1, DDX47, DENND2A, DHCR24, DHX36, DIAPH1, DIMT1, DLST, DNM1L, DNMBP, DNMT1, DNTTIP2, DPP9, DPYSL2, DPYSL3, DSP, DST, DYNC1LI1, DYNLL1, EDF1, EED, EEF1E1, EIF2AK2, EIF2D, EIF2S1, EIF3A, EIF3E, EIF6, ELP3,	No change

					EMG1, EML1, EML3, EML4, EPB41L2, EPB41L3, EPHA3, EPPK1, ERCC5, ERCC6L, EXD2, EZR, FARP1, FBLIM1, FEN1, FERMT1, FERMT2, FGD4, FHL2, FHOD1, FKBP1A, FLII, FMR1, FNBP1L, FSCN1, FSD1, G6PD, GAN, GAPDH, GARS1, GATAD2A, GBP1, GCFC2, GEMIN4, GET3, GLE1, GLRX3, GLYR1, GNL3L, GOLGA3, GPHN, GRWD1, GSK3B, GTF2F2, GTF3C3, H1-10, H1-5, HADHA, HAT1, HDAC2, HDAC4, HELZ, HERC2, HERC4, HINT1, HIP1, HIRIP3, HK2, HMCES, HMOX1, HNRNPA3, HNRNPC, HNRNPR, HSD17B10, HSD17B11, HSD17B4, HSPA14, HSPB11, HSPB6, IFIT5, IFT140, IGF2BP1, IGF2BP2, IKBKG, ILF3, IPO5, IST1, ITSN1, IVNS1ABP, JAM3, KAT7, KAT8, KDM1A, KDM3B, KDM4B, KIF15, KNTC1, KPNB1, KRT18, KRT19, KRT8, LANCL2, LARP1, LARP4B, LEMD2, LIPA, LLGL1, LMNB1, LMOD1, LPCAT2, LPP, LRPPRC, LRRFIP1, LRWD1, LSS, MACF1, MACROH2A1, MAD1L1, MAD2L1, MAEA, MAGED2, MAGI1, MALT1, MAP1B, MAP1LC3A, MBD3, MCM2, MCM3, MCM4, MCM5, MCM6, MCM7, MDC1, MEAK7, MGA, MICAL3, MLH1, MMAB, MORF4L1, MOV10, MPHOSPH8, MRPL10, MRPS17, MRPS21, MRPS31, MRTO4, MSH2, MSH6, MSN, MTA1, MTREX, MTRR, MVP, MYADM, MYH10, MYH9, MYO18A, MYO1B, MYO1D, MYO1E, MYO6, NCAPD2, NCAPG, NCAPH, NCKAP1, NCL, NCOA5,	

					NDRG1, NEDD1, NEDD4, NEK1, NES, NF1, NF2, NFKB2, NISCH, NLE1, NMD3, NOCT, NOL11, NOM1, NOP53, NPM1, NSDHL, NSMCE3, NSUN5, NUB1, NUBP2, NUDT16, NUDT19, NUF2, NUP62, OBSL1, OGFOD1, ORC4, ORC5, OSBPL10, OSBPL2, OXSR1, PAFAH1B1, PAFAH1B2, PALLD, PARP1, PARP4, PAWR, PBXIP1, PDCD6IP, PDS5B, PIN1, PKN2, PLEC, PML, PNPLA4, POGZ, POLD1, POLD2, POLE2, POLR1C, POLR1D, POLR2A, POLR2B, POLR2H, POLR2I, POP1, PPM1B, PPP1CA, PPP2R5A, PPP3CA, PPP4R3A, PRIM2, PRKCI, PRMT7, PROCR, PRPF19, PSIP1, PSMC2, PSMC3, PSMD1, PSMD2, PSPC1, PTGES2, PTPN12, PXK, PXN, PYCARD, PYCR3, RAB10, RAB23, RAB3D, RAB5A, RAB8A, RABGEF1, RABL6, RAC3, RAD21, RAD9A, RAI14, RANBP1, RANGAP1, RBBP4, RBM4, RBP1, RCC1, RCN2, RDH10, RDX, RECQL, REEP2, RFC2, RFC3, RFC5, RHOB, RHOC, RIF1, RIN1, RIPK2, RNF2, RNF213, RNF40, ROR1, RPA1, RPA2, RPF2, RPL10A, RPL13, RPL13A, RPL18A, RPL22, RPL24, RPL27, RPL32, RPL36, RPL38, RPL5, RPLP1, RPLP2, RPS13, RPS15A, RPS16, RPS18, RPS21, RPS23, RPS4Y1, RPS5, RPS8, RPS9, RTTN, S100A16, SAMHD1, SAP30BP, SARM1, SARNP, SBDS, SCAF11, SEC14L2, SELENBP1, SENP3, SEPTIN10, SEPTIN11, SEPTIN2, SEPTIN7, SEPTIN8, SEPTIN9, SERPINB1, SESTD1, SET, SF1, SH3KBP1, SHFL, SHMT2, SHPRH, SIRT1, SLC1A4,	

					SLC25A15, SLC25A5, SLC2A1, SLC30A9, SMAD4, SMARCA1, SMARCAD1, SMARCB1, SMARCC1, SMARCD3, SMARCE1, SMC1A, SMC2, SMC3, SMC4, SMCHD1, SMPD1, SNU13, SNW1, SNX27, SOD2, SORBS3, SPART, SPATS2L, SPECC1, SPHK2, SQSTM1, SRA1, SRC, SRSF5, SSB, SSRP1, STAG2, STAT1, STAT2, STAT5B, STK24, STOM, STON1, SUGT1, SUPT16H, SVIL, SYNE2, SYNJ1, SYNPO, TAP1, TBCA, TBCE, TBCEL, TCP1, TFAM, TGM2, TGS1, THOC2, TJP1, TLN2, TMA16, TNIK, TPM3, TPX2, TRA2A, TRADD, TRIM21, TRIM25, TRIM28, TRIM56, TRIP13, TRIP6, TRMT10C, TSEN15, TSEN34, TSPYL1, TTC28, TTLL12, TUBA4A, TUBA8, TUBB, TUBB3, TUBGCP2, TUT4, TWF2, UACA, UBA2, UBR2, UBR4, UBTF, UHRF2, URB2, USP11, USP7, UTP14A, UTP20, UTP3, VASP, VDAC1, VDAC2, VPS41, VRK1, VRK3, WDR1, WDR11, WDR12, WDR35, WDR70, WRAP73, WRNIP1, XIAP, XPO6, YBX1, YES1, ZNF207]	
cellular component biogenesis	GO_BiologicalProcess-EBI-UniProt-GOA-ACAP-ARAP_13.05.2021_00h00	2.81E-43	12.58427	448	[AAR2, ABCB7, ABCE1, ABT1, ACACA, ACSL3, ACTB, ACTG1, ACTR2, ACTR3, ADAM10, ADD3, ADNP, AFDN, AGO2, AGRN, AHCTF1, ALDOA, ANK3, ANO6, APEX1, APIP, APLP2, APP, APPL2, ARFGEF1, ARFIP1, ARPC4, ARPC5L, ARPIN, ARRB1, ASH2L, ASPM, ATG7, ATL3, ATPAF1, ATXN10, AUP1, B2M, BAIAP2, BANF1, BCR, BET1, BIN1, BMP1, BMS1, C1QBP, CALD1, CAPZA2, CCN2, CCNH, CCT3, CDC34, CDC42EP1, CDC73, CDH11, CDK1, CDK2,	No change

					CELF2, CENPV, CETN2, CFAP20, CFAP298, CFL2, CHAF1B, CHD1L, CHD7, CHMP3, CHORDC1, CHP1, CIRBP, CLGN, CLTC, CLU, CNGB1, CNOT1, COIL, COLEC12, CORO1B, CORO1C, CORO2B, CORO7, COX20, CRBN, CRYZ, CSPG4, CSRP2, CTNNB1, CTSC, CUL1, CUL4B, CXCL12, CYRIB, DARS1, DDX1, DDX47, DENR, DIAPH1, DIAPH3, DICER1, DIMT1, DNM1L, DNMT1, DPYSL3, DRG1, DST, DUSP3, DYNLL1, EFL1, EFNB2, EHD3, EIF2AK2, EIF2D, EIF2S1, EIF3A, EIF3B, EIF3E, EIF3F, EIF6, EMG1, EML3, EPB41L3, EPHA2, EPHA3, EPPK1, EPRS1, ERCC5, EXOC2, EZR, FAM171A1, FARP1, FASN, FBLIM1, FERMT1, FERMT2, FGD4, FHOD1, FKBP1A, FLII, FLRT2, FMR1, FN1, FNBP1L, FSCN1, GARS1, GBA2, GCFC2, GEMIN4, GLRX3, GLUL, GNL3L, GPC1, GPC6, GRWD1, GSDMD, GSK3B, H1-10, H1-5, HAT1, HDAC4, HEATR3, HGS, HIP1, HNRNPC, HSD17B10, HSP90AA1, HSPA4, HSPA5, HSPB11, IFT140, IKBKB, IKBKG, IMPACT, IST1, ITGB1, ITGB5, JAM3, KCTD12, KCTD15, KNTC1, KPNB1, KRT19, KRT8, LAMA5, LAMC1, LBR, LEMD2, LIMS1, LLGL1, LMOD1, LRCH4, LRRC8A, MACF1, MACROH2A1, MAGI1, MAP1B, MAP1LC3A, MAP4K4, MAT2A, MBD3, MCM2, MCM3, MCM4, MCM5, MCM6, MCM7, MLF2, MLH1, MMAB, MPHOSPH8, MRM3, MRPL10, MRTO4, MSN, MTREX, MYADM, MYD88, MYO1B, MYO1E,	

					NCAPG, NCK2, NCKAP1, NCLN, NDRG1, NDUFA13, NDUFAF7, NDUFS3, NECTIN2, NEDD1, NEDD4, NEK1, NF2, NLE1, NMD3, NOCT, NOL11, NOM1, NOP53, NPM1, NSUN5, NUBP2, NUBPL, NUDT16, NUDT19, NUP205, NUP35, NUP62, OBSL1, OGFOD1, ORC4, ORC5, OSBPL2, OTUD6B, OXA1L, PAFAH1B1, PARP1, PAWR, PDCD6IP, PDZD11, PHLDB1, PIEZO1, PIK3C2A, PIK3CA, PIN1, PIP4K2B, PKN2, PLD1, PLEC, PLXNB2, PML, POGZ, PPP3CA, PRKAA1, PRKCI, PRKCSH, PRMT7, PRPF19, PRUNE1, PSIP1, PSMA3, PSMB10, PSMB2, PSMC2, PSMC3, PSMC6, PSMD1, PSMD12, PSMD14, PSMD2, PSMD6, PSMD7, PSMD8, PSME1, PSME2, PSME4, PSMG1, PTGES2, PUF60, PXN, PYCARD, QSOX1, RAB1A, RAB23, RAB5A, RAB8A, RAC3, RAD21, RALA, RANGAP1, RAP1GDS1, RAP2B, RAP2C, RBBP4, RCC1, RDX, RHOC, RIOK3, RIPK2, RNF213, RPA1, RPA2, RPF2, RPL10A, RPL13, RPL13A, RPL18A, RPL22, RPL24, RPL27, RPL32, RPL36, RPL38, RPL5, RPLP1, RPLP2, RPS16, RPS21, RPS23, RPS5, RPS8, RPS9, RRM1, RTN4, RTTN, S100A10, SACS, SAMHD1, SBDS, SCAF11, SCFD1, SENP3, SEPTIN2, SEPTIN7, SEPTIN8, SEPTIN9, SET, SF1, SF3A3, SHMT2, SHPRH, SIRT1, SLC1A5, SLC25A15, SLC2A1, SLC9A1, SLIT2, SMAD4, SMARCB1, SMC1A, SMC3, SMCHD1, SMYD3, SNF8, SNRNP200, SNRPD3, SNRPE, SNU13, SOAT1, SOD2,	

					SORBS3, SPHK2, SRC, SRSF10, SRSF5, STAG2, SURF1, SVIL, SYNE2, SYNJ1, SYNPO, TBC1D10A, TBC1D17, TBC1D22B, TBCA, TBCE, TBCEL, TBPL1, TCP1, TFAM, TGFBR3, TGS1, THBS1, TIMMDC1, TJP1, TLN2, TM9SF4, TNIK, TPBG, TPX2, TRADD, TRAPPC4, TRAPPC8, TRIM28, TRIP13, TRIP6, TSPYL1, TSR2, TTC39C, TUBA4A, TUBB, TUBGCP2, TWF2, UBTF, UBXN2A, UNC119B, URB2, USP4, UTP14A, UTP20, UTP3, VASP, VDAC2, VPS25, VPS36, VRK1, WASHC5, WDR1, WDR11, WDR12, WDR35, WIPI2, WRAP73, YAP1, YBEY, YTHDC1, ZFP36L2, ZNF148, ZNF207]	
regulation of actin filament-based process	GO_BiologicalProcess-EBI-UniProt-GOA-ACAP-ARAP_13.05.2021_00h00	2.81E-43	15.238095	64	[ACTG1, ACTR2, ACTR3, ADD3, ARFGEF1, ARFIP1, ARHGAP17, ARPC4, ARPC5L, ARPIN, ATP1A1, BAIAP2, BIN1, BST1, CAMK2D, CAPZA2, CCN2, CDC42EP1, CFL2, CLU, CORO1B, CORO2B, CXCL12, CYRIB, DSP, DYNLL1, EPHA3, EZR, FERMT2, FHOD1, FLII, FSCN1, GBA2, IQGAP3, JAM3, LMOD1, MYADM, MYH9, MYO1E, NCAPG, NCK2, NCKAP1, NF2, PHPT1, PIK3CA, PKN2, PXN, PYCARD, RAC3, RDX, RHOB, RHOC, S100A10, SLC9A1, SLIT2, SMAD4, SORBS3, SVIL, SYNPO, TJP1, TWF2, VASP, WASHC5, WDR1]	No change
regulation of cellular component biogenesis	GO_BiologicalProcess-EBI-UniProt-GOA-ACAP-ARAP_13.05.2021_00h00	2.81E-43	13.42155	142	[ABCB7, ACTG1, ACTR2, ACTR3, ADD3, ADNP, AGRN, APEX1, APP, ARFGEF1, ARFIP1, ARPC4, ARPC5L, ARPIN, BAIAP2, BIN1, CAPZA2, CCN2, CDC34, CDC42EP1, CHAF1B, CHD1L, CHMP3, CLU, CNOT1, CORO1B, CORO1C, CORO2B, CRBN, CUL4B, CYRIB,	No change

					DNMT1, DPYSL3, DRG1, DUSP3, DYNLL1, EIF2AK2, EML3, EPHA2, EPHA3, ERCC5, EZR, FARP1, FBLIM1, FERMT1, FERMT2, FHOD1, FLII, FLRT2, FMR1, FNBP1L, FSCN1, GBA2, GNL3L, GPC6, GSK3B, HDAC4, HGS, HSP90AA1, HSPA5, IFT140, IKBKB, IMPACT, JAM3, LIMS1, LMOD1, LRCH4, MACF1, MACROH2A1, MAP1B, MAP4K4, MMAB, MPHOSPH8, MSN, MYADM, MYD88, MYO1E, NCAPG, NCK2, NCKAP1, NCLN, NDRG1, NF2, NOP53, NPM1, NUDT16, NUP62, PARP1, PDCD6IP, PHLDB1, PIEZO1, PIK3CA, PIP4K2B, PKN2, PLD1, PPP3CA, PRKAA1, PRKCSH, PRUNE1, PSMC2, PSMC3, PSMC6, PXN, PYCARD, RAB5A, RALA, RDX, RHOC, RIOK3, RIPK2, RPA1, RPA2, RPL13A, S100A10, SACS, SCFD1, SEPTIN7, SEPTIN8, SEPTIN9, SLC9A1, SLIT2, SMAD4, SNF8, SORBS3, SPHK2, SRC, SVIL, SYNE2, SYNPO, TBC1D10A, TBC1D17, TBC1D22B, THBS1, TJP1, TPBG, TRIM28, TWF2, VASP, VDAC2, WASHC5, WRAP73, YAP1]	
regulation of cellular component organization	GO_BiologicalProcess-EBI-UniProt-GOA-ACAP-ARAP_13.05.2021_00h00	2.81E-43	12.121212	308	[AASS, ABCB7, ACTG1, ACTR2, ACTR3, ADAM10, ADD3, ADNP, AGRN, AKR1B1, ANKFY1, ANKRD13A, ANO6, APBB2, APEX1, APLP2, APP, APPL2, ARFGAP1, ARFGEF1, ARFIP1, ARHGAP17, ARHGEF11, ARPC4, ARPC5L, ARPIN, ARRB1, ATL3, B2M, BAIAP2, BIN1, BST1, CAMK2D, CAPZA2, CCN2, CCT2, CCT3, CCT6A, CDC34, CDC42EP1, CDC73, CENPV, CFL2, CHAF1B, CHD1L, CHMP3, CHORDC1, CHTOP, CLTC, CLU,	No change

					CNOT1, COL5A1, CORO1B, CORO1C, CORO2B, CRABP2, CRBN, CTBP1, CTNNB1, CUL4B, CUX1, CXCL12, CYRIB, DAB2, DCBLD2, DGUOK, DHX36, DIAPH1, DNM1L, DNMT1, DPYSL2, DPYSL3, DRG1, DUSP3, DVL2, DYNC1LI1, DYNLL1, EFNB2, EHD3, EIF2AK2, EML3, ENO2, ENPP1, EPB41L3, EPHA2, EPHA3, ERBB2, ERCC5, EZR, FARP1, FBLIM1, FBXW8, FEN1, FERMT1, FERMT2, FHL1, FHOD1, FLII, FLRT2, FMR1, FN1, FNBP1L, FSCN1, FSD1, G6PD, GBA2, GLE1, GLMN, GLYR1, GNL3L, GPC6, GSK3B, H1-10, H1-5, HDAC2, HDAC4, HDGFL2, HIP1, HNRNPC, HSP90AA1, HSPA5, IFT140, IGFBP7, IKBKB, IMPACT, IQGAP3, IST1, ITGA3, ITGAV, ITM2C, ITSN1, JAM3, KAT7, KDM1A, KDM4B, KNTC1, LIMS1, LLGL1, LMOD1, LRCH4, MACF1, MACROH2A1, MAD1L1, MAD2L1, MAGI1, MAP1B, MAP3K4, MAP4K4, MARCHF5, MCM2, MCM7, MFF, MMAB, MOV10, MPHOSPH8, MSN, MYADM, MYD88, MYH9, MYO1E, NCAPG, NCK2, NCKAP1, NCLN, NDRG1, NDUFA13, NDUFS3, NEDD4, NES, NF2, NMT1, NOP53, NPM1, NUBP2, NUP62, OBSL1, OGFOD1, P3H1, PACSIN3, PAFAH1B1, PARG, PARL, PARP1, PDCD6IP, PHLDB1, PHPT1, PIEZO1, PIK3CA, PIN1, PIP4K2B, PKN2, PLD1, PLXNB2, PML, PPP3CA, PRKAA1, PRKCI, PRKCSH, PRUNE1, PSMC2, PSMC3, PSMC6, PTK7, PTPRF, PTPRG, PXN, PYCARD, RAB5A, RAC3, RAD21, RALA,	

					RANBP1, RCC1, RDX, RHOB, RHOC, RIF1, RIOK3, RIPK2, RNF2, RNF40, ROR1, RPA1, RPA2, RPL13A, RTN4, S100A10, SACS, SARM1, SCARB2, SCFD1, SCRIB, SEMA3C, SEPTIN11, SEPTIN7, SEPTIN8, SEPTIN9, SERPINE2, SESN2, SET, SH3BP4, SHFL, SIRT1, SLC25A4, SLC25A5, SLC9A1, SLIT2, SMAD4, SMARCB1, SMPD1, SNW1, SORBS3, SPARC, SPART, SPHK2, SQSTM1, SRC, SSRP1, STAT2, STK24, STON1, SVIL, SYNE2, SYNJ1, SYNPO, TBC1D10A, TBC1D17, TBC1D22B, TBC1D4, TCP1, TENM3, TF, THBS1, THOC2, TIGAR, TJP1, TMED9, TMEM97, TNIK, TOM1L2, TPBG, TPX2, TRIM28, TRIP12, TRIP13, TSC2, TSPO, TTLL12, TUBB, TWF2, URI1, USP7, UST, VASP, VAT1, VDAC1, VDAC2, VLDLR, VTN, WASHC5, WDR1, WDR35, WDR5, WDR70, WRAP73, YAP1, ZMPSTE24, ZNF207]	
actin binding	GO_MolecularFunction-EBI-UniProt-GOA-ACAP-ARAP_13.05.2021_00h00	2.81E-43	15.565032	73	[ACTR2, ACTR3, ADD3, AFDN, ALDOA, ARPC4, ARPC5L, BIN1, CALD1, CAP1, CAP2, CAPZA2, CFL2, CNN1, CNN3, COBLL1, CORO1B, CORO1C, CORO2B, CORO7, DIAPH1, DIAPH3, DST, EPB41L2, EPB41L3, EZR, FERMT1, FERMT2, FGD4, FHOD1, FLII, FSCN1, GBP1, GIPC1, HIP1, IMPACT, IQGAP3, ITGB1, KCNMA1, LMOD1, LRPPRC, MACF1, MAEA, MAP1B, MICAL3, MSN, MYH10, MYH9, MYO18A, MYO1B, MYO1D, MYO1E, MYO6, NCAPG, NCK2, NF2, PALLD, PANX1, PAWR, PLEC, PXK, RAI14, RDX, SERPINB8, SVIL, SYNE2, SYNPO,	No change

					TAGLN, TLN2, TPM3, TWF2, VASP, WDR1]	
organelle organization	GO_BiologicalProcess-EBI-UniProt-GOA-ACAP-ARAP_13.05.2021_00h00	2.81E-43	11.613806	498	[AASS, ABI1, ABT1, ACOT8, ACTB, ACTG1, ACTR2, ACTR3, ADD3, AGRN, AHCTF1, ALDH3A2, ALDOA, AMOTL1, ANK3, ANKFY1, APEX1, APP, ARFGEF1, ARFIP1, ARHGAP12, ARHGAP17, ARHGEF11, ARID1B, ARPC4, ARPC5L, ARPIN, ARRB1, ASH2L, ASPM, ATG7, ATL3, ATP2C1, ATP6V0A1, ATP6V0C, ATP6V1A, ATP6V1B2, ATPAF1, ATXN10, AUP1, BAIAP2, BANF1, BAZ1B, BET1, BIN1, BNIP1, BOD1, BRD3, BST1, C1QBP, CALD1, CAMK2D, CAP1, CAP2, CAPZA2, CBX2, CBX3, CBX8, CCN2, CCT2, CCT3, CCT6A, CDC42BPA, CDC42EP1, CDC73, CDK1, CDK2, CECR2, CENPV, CETN2, CFAP20, CFAP298, CFL2, CHAF1B, CHAMP1, CHD1L, CHD2, CHD4, CHD7, CHMP3, CHORDC1, CHP1, CHTOP, CIRBP, CLIP2, CLTC, CLU, CNGB1, CNN1, CNN3, CNOT1, COG7, CORO1B, CORO1C, CORO2B, CORO7, COX20, CSRP2, CTBP1, CTNNB1, CTSC, CUL4B, CXCL12, CYCS, CYRIB, DDX1, DENR, DHX36, DIAPH1, DIAPH3, DNM1L, DNMT1, DPYSL2, DPYSL3, DRG1, DSP, DST, DYM, DYNC1LI1, DYNLL1, EED, EFL1, EHD3, EIF2D, EIF2S1, EIF6, EMC1, EMG1, EML1, EML3, EML4, ENO2, ENY2, EPB41L2, EPB41L3, EPHA3, EPPK1, ERCC6L, ESYT1, EXOC2, EZR, FAM171A1, FARP1, FAT1, FBXW8, FEN1, FERMT2, FGD4, FHOD1, FLII, FMR1, FNBP1L, FSCN1, FSD1, GAB1, GAN, GAPDH, GBA2, GCC2, GDAP1, GET3, GGCT, GLYR1,	Upregulation

					GNL3L, GRWD1, GSK3B, GTF2F2, GTF3C4, H1-10, H1-5, HACL1, HAT1, HDAC2, HDAC4, HEXB, HGS, HIP1, HIRIP3, HK2, HMOX1, HNRNPC, HSD17B10, HSP90AA1, HSPA4, HSPB11, IFT140, IKBKB, IQGAP3, IST1, ITGB1, ITGB5, JAGN1, JAM3, KAT7, KAT8, KDM1A, KDM3B, KDM4B, KNTC1, KPNB1, KRT18, KRT19, KRT8, LAMA5, LBR, LEMD2, LIG3, LLGL1, LMOD1, LRCH3, LRWD1, MACF1, MACROH2A1, MAD1L1, MAD2L1, MAEA, MAGI1, MAP1B, MAP1LC3A, MAP3K20, MAP3K4, MARCHF5, MBD3, MCM2, MCM3, MCM4, MCM5, MCM6, MCM7, MCMBP, MFF, MFN1, MICAL3, MLH1, MMAB, MORF4L1, MPHOSPH8, MRRF, MRTO4, MSH2, MSH6, MSN, MTA1, MYADM, MYH10, MYH9, MYO18A, MYO1B, MYO1D, MYO1E, MYO6, N6AMT1, NCAPD2, NCAPG, NCAPH, NCK2, NCKAP1, NDRG1, NDUFA13, NDUFAF7, NDUFS3, NECTIN2, NEDD1, NEK1, NES, NF1, NF2, NISCH, NLE1, NMT1, NOCT, NOP53, NPLOC4, NPM1, NUBP2, NUBPL, NUDT19, NUF2, NUP205, NUP35, NUP62, OBSL1, OGFOD1, OXA1L, PACSIN3, PAFAH1B1, PALLD, PARG, PARL, PARP1, PAWR, PDCD6IP, PDS5B, PELO, PHLDB1, PHPT1, PI4K2B, PIGU, PIK3CA, PIN1, PIP4K2B, PITRM1, PKN2, PLEC, PML, POGZ, POLD1, POLD2, PPP5C, PRIM2, PRKAA1, PRKCI, PRKG1, PRMT7, PRUNE1, PSME4, PTGES2, PTK7, PTPA, PXN, PYCARD, RAB10,	

					RAB1A, RAB23, RAB5A, RAB8A, RAC3, RAD21, RALA, RANBP1, RANGAP1, RAP1GDS1, RBBP4, RCC1, RDX, RECQL, REEP2, REEP6, RFC2, RFC3, RFC5, RHOB, RHOC, RIF1, RIPK2, RNF2, RNF40, RPA1, RPA2, RPF2, RPL24, RPL38, RPL5, RPS23, RPS5, RTN4, RTTN, RYBP, S100A10, SAE1, SBDS, SCARB2, SCFD1, SCP2, SEC14L2, SEC23B, SEC61B, SEPTIN2, SEPTIN7, SEPTIN8, SEPTIN9, SERPINB8, SERPINE2, SESN2, SET, SF1, SH3KBP1, SHPRH, SIRT1, SLC25A4, SLC25A5, SLC25A6, SLC9A1, SLIT2, SMAD4, SMARCA1, SMARCAD1, SMARCB1, SMARCC1, SMARCD3, SMARCE1, SMC1A, SMC2, SMC3, SMC4, SMCHD1, SMYD3, SNF8, SNW1, SOD1, SOD2, SON, SORBS3, SORT1, SPART, SPECC1, SPHK2, SQSTM1, SRC, SSRP1, STAG2, STAT2, STON1, SUPT16H, SURF1, SVIL, SYNE2, SYNJ1, SYNPO, TAF6L, TAP1, TBC1D10A, TBC1D17, TBC1D22B, TBC1D4, TBCE, TBCEL, TBPL1, TCP1, TDRD3, TF, TFAM, THEM4, TIGAR, TIMM17B, TIMMDC1, TJP1, TLN2, TMED9, TNIK, TOM1L2, TOMM34, TOMM7, TOR1AIP1, TOR1AIP2, TOR1B, TPM3, TPP1, TPX2, TRAM1, TRAM2, TRAPPC4, TRAPPC8, TRIM28, TRIP12, TRIP13, TSC2, TSPO, TSPYL1, TTC39C, TTLL12, TUBA4A, TUBA8, TUBB, TUBB3, TUBGCP2, TWF2, TYMP, UBR2, UBXN2A, UNC119B, USP22, USP7, UTP3, VASP, VAT1, VDAC1, VDAC2, VPS25, VPS33B, VPS36, VPS41, VRK1, WASHC5, WDR1, WDR11, WDR35, WDR5, WDR70,	

					WIPI2, WRAP73, WRNIP1, YAP1, YEATS4, ZFAND6, ZMPSTE24, ZMYM3, ZNF207]	
cellular component assembly	GO_BiologicalProcess-EBI-UniProt-GOA-ACAP-ARAP_13.05.2021_00h00	2.81E-43	12.431444	408	[AAR2, ABCB7, ABT1, ACACA, ACSL3, ACTB, ACTG1, ACTR2, ACTR3, ADAM10, ADD3, ADNP, AFDN, AGO2, AGRN, AHCTF1, ALDOA, ANK3, ANO6, APEX1, APIP, APLP2, APP, APPL2, ARFGEF1, ARFIP1, ARPC4, ARPC5L, ARPIN, ARRB1, ASH2L, ASPM, ATG7, ATL3, ATPAF1, ATXN10, AUP1, B2M, BAIAP2, BANF1, BCR, BET1, BIN1, BMP1, C1QBP, CALD1, CAPZA2, CCN2, CCNH, CCT3, CDC34, CDC42EP1, CDC73, CDH11, CDK1, CDK2, CELF2, CENPV, CETN2, CFAP20, CFAP298, CFL2, CHAF1B, CHD1L, CHMP3, CHORDC1, CHP1, CIRBP, CLGN, CLTC, CLU, CNGB1, CNOT1, COIL, COLEC12, CORO1B, CORO1C, CORO2B, CORO7, COX20, CRBN, CRYZ, CSPG4, CSRP2, CTNNB1, CTSC, CUL1, CUL4B, CXCL12, CYRIB, DARS1, DDX1, DENR, DIAPH1, DIAPH3, DICER1, DNM1L, DNMT1, DPYSL3, DRG1, DST, DUSP3, DYNLL1, EFL1, EFNB2, EHD3, EIF2AK2, EIF2D, EIF2S1, EIF3A, EIF3B, EIF3E, EIF3F, EIF6, EML3, EPB41L3, EPHA2, EPHA3, EPPK1, EPRS1, ERCC5, EXOC2, EZR, FAM171A1, FARP1, FASN, FBLIM1, FERMT1, FERMT2, FGD4, FHOD1, FKBP1A, FLII, FLRT2, FMR1, FN1, FNBP1L, FSCN1, GARS1, GBA2, GCFC2, GEMIN4, GLRX3, GNL3L, GPC1, GPC6, GRWD1, GSDMD, GSK3B, H1-10, H1-5, HAT1, HDAC4, HGS, HIP1, HNRNPC, HSD17B10, HSP90AA1, HSPA4, HSPA5, HSPB11,	No change

					IFT140, IKBKB, IKBKG, IMPACT, IST1, ITGB1, ITGB5, JAM3, KCTD12, KCTD15, KNTC1, KPNB1, KRT19, KRT8, LAMA5, LAMC1, LBR, LEMD2, LIMS1, LLGL1, LMOD1, LRCH4, LRRC8A, MACF1, MACROH2A1, MAGI1, MAP1B, MAP1LC3A, MAP4K4, MAT2A, MBD3, MCM2, MCM3, MCM4, MCM5, MCM6, MCM7, MLF2, MLH1, MMAB, MPHOSPH8, MRTO4, MSN, MYADM, MYD88, MYO1B, MYO1E, NCAPG, NCK2, NCKAP1, NCLN, NDRG1, NDUFA13, NDUFAF7, NDUFS3, NECTIN2, NEDD1, NEK1, NF2, NLE1, NOCT, NOP53, NPM1, NUBP2, NUBPL, NUDT19, NUP205, NUP35, NUP62, OBSL1, OGFOD1, ORC4, ORC5, OSBPL2, OTUD6B, OXA1L, PAFAH1B1, PARP1, PAWR, PDCD6IP, PDZD11, PHLDB1, PIEZO1, PIK3C2A, PIK3CA, PIN1, PIP4K2B, PKN2, PLD1, PLEC, PLXNB2, PML, POGZ, PRKAA1, PRKCI, PRKCSH, PRMT7, PRPF19, PRUNE1, PSIP1, PSMA3, PSMB10, PSMB2, PSMC2, PSMC3, PSMC6, PSMD1, PSMD12, PSMD14, PSMD2, PSMD6, PSMD7, PSMD8, PSME1, PSME2, PSME4, PSMG1, PTGES2, PUF60, PXN, PYCARD, QSOX1, RAB1A, RAB23, RAB5A, RAB8A, RAC3, RAD21, RALA, RANGAP1, RAP1GDS1, RAP2B, RAP2C, RBBP4, RCC1, RDX, RHOC, RIOK3, RIPK2, RNF213, RPA1, RPA2, RPF2, RPL13A, RPL24, RPL38, RPL5, RPS23, RPS5, RRM1, RTN4, RTTN, S100A10, SACS, SAMHD1, SBDS,	

					SCAF11, SCFD1, SEPTIN2, SEPTIN7, SEPTIN8, SEPTIN9, SET, SF1, SF3A3, SHMT2, SHPRH, SIRT1, SLC1A5, SLC25A15, SLC2A1, SLC9A1, SLIT2, SMAD4, SMARCB1, SMC1A, SMC3, SMCHD1, SMYD3, SNF8, SNRNP200, SNRPD3, SNRPE, SNU13, SOAT1, SOD2, SORBS3, SRC, SRSF10, SRSF5, STAG2, SURF1, SVIL, SYNE2, SYNJ1, SYNPO, TBC1D10A, TBC1D17, TBC1D22B, TBCA, TBCE, TBCEL, TBPL1, TCP1, TFAM, TGFBR3, TGS1, THBS1, TIMMDC1, TJP1, TLN2, TM9SF4, TNIK, TPBG, TPX2, TRADD, TRAPPC4, TRAPPC8, TRIM28, TRIP13, TRIP6, TSPYL1, TTC39C, TUBA4A, TUBB, TUBGCP2, TWF2, UBTF, UBXN2A, UNC119B, USP4, VASP, VDAC2, VPS25, VPS36, VRK1, WASHC5, WDR1, WDR11, WDR35, WIPI2, WRAP73, YAP1, YTHDC1, ZFP36L2, ZNF148, ZNF207]	
regulation of cellular component size	GO_BiologicalProcess-EBI-UniProt-GOA-ACAP-ARAP_13.05.2021_00h00	2.81E-43	14.496314	59	[ACTR2, ACTR3, ADD3, ADNP, ANO6, ARFGEF1, ARFIP1, ARPC4, ARPC5L, ARPIN, BAIAP2, BIN1, CAPZA2, CDC42EP1, CFL2, CHMP3, CORO1B, CRABP2, CXCL12, CYRIB, DPYSL2, EZR, FLII, FN1, GBA2, GSK3B, IQGAP3, IST1, KCNMA1, KDM1A, LMOD1, LRRC8A, LRRC8E, MACF1, MAP1B, MSN, MYADM, MYO1E, NCAPG, NCK2, NCKAP1, NPM1, PAFAH1B1, PIK3CA, PPP1CA, PYCARD, RAB3B, RAB5A, RDX, RTN4, SEMA3C, SLC12A2, SLIT2, SPART, SVIL, TWF2, VASP, WASHC5, WDR1]	No change
protein-containing complex subunit organization	GO_BiologicalProcess-EBI-UniProt-GOA-ACAP-	2.81E-43	13.182674	280	[AAR2, ABCE1, ABT1, ACACA, ACSL3, ACTR2, ACTR3, ADAM10, ADD3, AFDN, AGO2,	No change

	ARAP_13.05.2021_00h00				AHCTF1, ALB, ALDOA, ANO6, APIP, APLP2, APPL2, ARFGEF1, ARFIP1, ARPC4, ARPC5L, ARPIN, ASH2L, ATL3, ATPAF1, B2M, BAIAP2, BET1, BIN1, BMP1, CAPZA2, CCNH, CCT3, CDC42EP1, CDC73, CDK1, CELF2, CENPV, CETN2, CFL2, CHAF1B, CHD1L, CHMP3, CHORDC1, CHP1, CLGN, CLTC, CLU, COIL, COLEC12, CORO1B, CORO7, COX20, CRBN, CRYZ, CTNNB1, CTSC, CUL1, CUL4B, CXCL12, CYRIB, DARS1, DDX1, DENR, DIAPH1, DIAPH3, DICER1, DNM1L, DRG1, DVL2, EHD3, EIF2AK2, EIF2D, EIF3A, EIF3B, EIF3E, EIF3F, EIF6, EPRS1, ERCC5, FASN, FBLIM1, FERMT1, FERMT2, FKBP1A, FLII, FN1, FSCN1, GARS1, GBA2, GCFC2, GEMIN4, GLE1, GNL3L, GRWD1, GSDMD, GSK3B, GTF2E1, H1-10, H1-5, HAT1, HIP1, HNRNPC, HSD17B10, HSP90AA1, HSPA4, HSPA5, IKBKG, IMPACT, IST1, JAM3, KCTD12, KCTD15, KNTC1, LAMC1, LLGL1, LMOD1, LRRC8A, MACROH2A1, MAGI1, MAP1B, MAP1LC3A, MAT2A, MCM2, MCM3, MCM4, MCM5, MCM6, MCM7, MICAL3, MMAB, MRPL10, MRPS17, MRPS21, MRPS31, MRRF, MRTO4, MSN, MYADM, MYD88, MYO1E, N6AMT1, NCAPG, NCK2, NCKAP1, NCLN, NDRG1, NDUFA13, NDUFAF7, NDUFS3, NES, NLE1, NOP53, NPM1, NUBPL, NUP205, NUP35, OGFOD1, ORC4, ORC5, OSBPL2, OTUD6B, OXA1L, PARP1, PDCD6IP, PDZD11, PIEZO1, PIK3C2A, PIK3CA, PIN1, PML, POGZ,	

					PPP1CA, PRMT7, PRPF19, PRUNE1, PSIP1, PSMA3, PSMB10, PSMB2, PSMC2, PSMC3, PSMC6, PSMD1, PSMD12, PSMD14, PSMD2, PSMD6, PSMD7, PSMD8, PSME1, PSME2, PSME4, PSMG1, PUF60, PXN, PYCARD, RAB1A, RAP1GDS1, RBBP4, RDX, RHOC, RIOK3, RIPK2, RNF213, RPA1, RPA2, RPF2, RPL13A, RPL24, RPL38, RPL5, RPS5, RRM1, RTN4, SAMHD1, SCAF11, SCFD1, SEPTIN8, SEPTIN9, SET, SF1, SF3A3, SHFL, SHMT2, SHPRH, SLC1A5, SLC25A15, SLC2A1, SLC9A1, SLIT2, SMAD4, SMARCB1, SMARCC1, SMARCD3, SMARCE1, SMYD3, SNRNP200, SNRPD3, SNRPE, SNU13, SOAT1, SOD2, SRC, SRSF10, SRSF5, SUPT16H, SURF1, SVIL, SYNJ1, TBCA, TBCE, TBCEL, TCP1, TFAM, TGFBR3, TGS1, TIMMDC1, TM9SF4, TPX2, TRADD, TRAPPC4, TSPYL1, TUBGCP2, TWF2, UBTF, USP4, VASP, VDAC2, VPS33B, WASHC5, WDR1, WIPI2, YAP1, YTHDC1, ZFP36L2, ZMPSTE24, ZNF148, ZNF207]	
positive regulation of cellular component biogenesis	GO_BiologicalProcess-EBI-UniProt-GOA-ACAP-ARAP_13.05.2021_00h00	2.81E-43	14.409722	83	[ACTR2, ACTR3, ADNP, AGRN, APEX1, ARFIP1, ARPC4, ARPC5L, ARPIN, BAIAP2, BIN1, CCN2, CDC34, CDC42EP1, CHAF1B, CHD1L, CLU, CNOT1, CORO1B, CRBN, CUL4B, DNMT1, DPYSL3, DRG1, EPHA2, ERCC5, FERMT1, FERMT2, FHOD1, FLRT2, FMR1, FNBP1L, FSCN1, GNL3L, GSK3B, HDAC4, HGS, HSP90AA1, LIMS1, LMOD1, LRCH4, MAP1B, MAP4K4, MMAB, MPHOSPH8, MSN, MYD88, MYO1E, NCAPG, NCK2, NCKAP1,	No change

					NDRG1, NF2, NUP62, PARP1, PDCD6IP, PHLDB1, PIEZO1, PIK3CA, PIP4K2B, PSMC2, PSMC3, PSMC6, PXN, PYCARD, RALA, RHOC, RIPK2, RPA1, RPA2, S100A10, SEPTIN7, SEPTIN9, SNF8, SORBS3, SRC, SYNPO, TPBG, TRIM28, TWF2, VASP, WASHC5, WRAP73]	
actin filament binding	GO_MolecularFunction-EBI-UniProt-GOA-ACAP-ARAP_13.05.2021_00h00	2.81E-43	17.333334	39	[ACTR2, ACTR3, ADD3, AFDN, ARPC4, ARPC5L, BIN1, CAPZA2, CFL2, CORO1B, CORO1C, CORO2B, CORO7, EZR, FERMT1, FERMT2, FHOD1, FLII, FSCN1, HIP1, IQGAP3, LRPPRC, MACF1, MYH10, MYH9, MYO18A, MYO1B, MYO1D, MYO1E, MYO6, NCAPG, PANX1, SVIL, SYNE2, TAGLN, TLN2, TPM3, TWF2, WDR1]	No change
negative regulation of cellular component organization	GO_BiologicalProcess-EBI-UniProt-GOA-ACAP-ARAP_13.05.2021_00h00	2.81E-43	12.804097	100	[AASS, ADD3, AKR1B1, ANKRD13A, APP, ARFGEF1, ARPIN, B2M, CAPZA2, CLU, CORO1B, CORO1C, CORO2B, CRBN, CTBP1, CYRIB, DGUOK, DNM1L, DNMT1, DPYSL3, DYNC1LI1, DYNLL1, EFNB2, EPHA3, FLII, GLMN, GNL3L, GSK3B, H1-10, H1-5, HDAC2, HNRNPC, HSPA5, IKBKB, IMPACT, ITGA3, ITGAV, ITM2C, JAM3, KDM1A, KDM4B, KNTC1, LLGL1, LMOD1, MACROH2A1, MAD1L1, MAD2L1, MAP1B, MAP4K4, MCM2, MCM7, MYADM, NCAPG, NCK2, NOP53, NPM1, NUBP2, PACSIN3, PAFAH1B1, PARP1, PIK3CA, PKN2, PPP3CA, PTPRG, RAD21, RDX, RIOK3, RPL13A, RTN4, SACS, SCFD1, SEMA3C, SET, SIRT1, SLC25A5, SLIT2, SMAD4, SMARCB1, SPART, SPHK2, SRC, STON1, SVIL, TBC1D4, THBS1, THOC2, TIGAR, TJP1, TOM1L2, TPX2,	Upregulation

					TRIP12, TRIP13, TSC2, TSPO, TWF2, VAT1, VDAC2, WDR5, YAP1, ZNF207]	
supramolecular fiber organization	GO_BiologicalProcess-EBI-UniProt-GOA-ACAP-ARAP_13.05.2021_00h00	2.81E-43	14.301802	127	[ABI1, ACTG1, ACTR2, ACTR3, ADAM10, ADD3, ALDOA, APP, ARFGEF1, ARFIP1, ARHGAP12, ARHGAP17, ARPC4, ARPC5L, ARPIN, ARRB1, B2M, BAIAP2, BIN1, BMP1, CALD1, CAPZA2, CCN2, CDC42EP1, CFL2, CHORDC1, CHP1, CLIP2, CLU, COL11A1, COL12A1, COL15A1, COL18A1, COL4A1, COL5A1, COL5A2, COL6A1, CORO1B, CORO1C, CORO2B, CORO7, CRTAP, CSRP2, CXCL12, CYRIB, DIAPH1, DIAPH3, DPYSL3, DRG1, DSP, DST, DYNLL1, EPPK1, EZR, FAM171A1, FAT1, FERMT2, FHOD1, FKBP1A, FLII, FSCN1, FSD1, GBA2, GGA2, HIP1, HSPG2, ITGB1, ITGB5, KPNB1, KRT19, KRT8, LMOD1, LUM, MAP1B, MICAL3, MYADM, MYO1B, MYO1D, MYO1E, MYO6, NCAPG, NCK2, NCKAP1, NDRG1, NF1, NF2, OBSL1, P3H1, PAFAH1B1, PAWR, PIK3CA, PIN1, PKN2, PLEC, PLOD2, PPIB, PRKCI, PRUNE1, PXN, PYCARD, RAC3, RAP1GDS1, RDX, RHOB, RHOC, S100A10, SH3KBP1, SLC9A1, SLIT2, SMAD4, SORBS3, SRC, SVIL, SYNPO, TF, TGFBI, TJP1, TPM3, TPX2, TUBGCP2, TWF2, UBE2L6, USP9X, VASP, WASHC5, WDR1, ZNF207]	No change
actin cytoskeleton organization	GO_BiologicalProcess-EBI-UniProt-GOA-ACAP-ARAP_13.05.2021_00h00	2.81E-43	16.168478	119	[ABI1, ACTB, ACTG1, ACTR2, ACTR3, ADD3, ALDOA, AMOTL1, ARFGEF1, ARFIP1, ARHGAP12, ARHGAP17, ARHGEF11, ARPC4, ARPC5L, ARPIN, ARRB1, ATP2C1, BAIAP2, BIN1, BST1, CALD1, CAP1, CAPZA2, CCN2, CDC42BPA,	No change

					CDC42EP1, CFL2, CLU, CNN1, CNN3, CORO1B, CORO1C, CORO2B, CORO7, CSRP2, CXCL12, CYRIB, DIAPH1, DIAPH3, DPYSL3, DYNLL1, EPB41L2, EPB41L3, EPHA3, EZR, FAM171A1, FARP1, FAT1, FERMT2, FGD4, FHOD1, FLII, FSCN1, GAB1, GBA2, HIP1, IKBKB, IQGAP3, ITGB1, ITGB5, JAM3, KRT19, KRT8, LLGL1, LMOD1, MICAL3, MYADM, MYH10, MYH9, MYO18A, MYO1B, MYO1D, MYO1E, MYO6, NCAPG, NCK2, NCKAP1, NF1, NF2, NISCH, OBSL1, PAFAH1B1, PALLD, PAWR, PDCD6IP, PHPT1, PIGU, PIK3CA, PKN2, PRKCI, PRKG1, PTK7, PXN, PYCARD, RAC3, RALA, RAP1GDS1, RDX, RHOB, RHOC, S100A10, SH3KBP1, SLC9A1, SLIT2, SMAD4, SORBS3, SPECC1, SRC, SVIL, SYNPO, TF, TJP1, TNIK, TPM3, TWF2, VASP, WASHC5, WDR1]	
regulation of organelle organization	GO_BiologicalProcess-EBI-UniProt-GOA-ACAP-ARAP_13.05.2021_00h00	2.81E-43	12.605042	165	[ACTG1, ACTR2, ACTR3, ADD3, APEX1, ARFGEF1, ARFIP1, ARHGAP17, ARPC4, ARPC5L, ARPIN, ARRB1, ATL3, BAIAP2, BIN1, BST1, CAPZA2, CCN2, CCT2, CCT3, CCT6A, CDC42EP1, CENPV, CFL2, CHMP3, CHTOP, CLTC, CLU, CNOT1, CORO1B, CORO2B, CTBP1, CTNNB1, CXCL12, CYRIB, DHX36, DIAPH1, DNM1L, DNMT1, DRG1, DYNC1LI1, DYNLL1, EHD3, EML3, ENO2, EPHA3, EZR, FEN1, FERMT2, FHOD1, FLII, FMR1, FSCN1, FSD1, GBA2, GLYR1, GNL3L, GSK3B, H1-5, HNRNPC, IFT140, IQGAP3, JAM3, KAT7, KNTC1, LMOD1, MACROH2A1, MAD1L1, MAD2L1, MAP1B, MAP3K4, MARCHF5, MCM2, MCM7, MFF, MMAB,	No change

					MPHOSPH8, MSN, MYADM, MYO1E, NCAPG, NCK2, NCKAP1, NDRG1, NES, NF2, NMT1, NPM1, NUBP2, NUP62, OBSL1, PAFAH1B1, PARL, PARP1, PDCD6IP, PHLDB1, PHPT1, PIK3CA, PIN1, PIP4K2B, PKN2, PML, PRKAA1, PRUNE1, PXN, PYCARD, RAB5A, RAC3, RAD21, RALA, RCC1, RDX, RHOB, RHOC, RIF1, RNF40, S100A10, SCARB2, SCFD1, SEPTIN7, SEPTIN9, SIRT1, SLC25A4, SLC25A5, SLC9A1, SLIT2, SMAD4, SMARCB1, SNW1, SORBS3, SPHK2, SQSTM1, SRC, STAT2, STON1, SVIL, SYNE2, SYNJ1, SYNPO, TBC1D10A, TBC1D17, TBC1D22B, TBC1D4, TCP1, TIGAR, TJP1, TMED9, TOM1L2, TPX2, TRIM28, TRIP13, TSC2, TSPO, TWF2, USP7, VASP, VAT1, VDAC1, WASHC5, WDR1, WDR35, WDR5, WRAP73, YAP1, ZNF207]	
regulation of supramolecular fiber organization	GO_BiologicalProcess-EBI-UniProt-GOA-ACAP-ARAP_13.05.2021_00h00	2.81E-43	13.965087	56	[ACTG1, ACTR2, ACTR3, ADD3, APP, ARFGEF1, ARFIP1, ARPC4, ARPC5L, ARPIN, BAIAP2, BIN1, CAPZA2, CCN2, CDC42EP1, CFL2, CLU, CORO1B, CORO2B, CXCL12, CYRIB, DRG1, DYNLL1, FERMT2, FHOD1, FLII, GBA2, LMOD1, MAP1B, MYADM, MYO1E, NCAPG, NCK2, NCKAP1, NDRG1, NF2, PIK3CA, PKN2, PRUNE1, PXN, PYCARD, RDX, RHOC, S100A10, SLC9A1, SLIT2, SMAD4, SORBS3, SVIL, SYNPO, TJP1, TPX2, TWF2, VASP, WASHC5, WDR1]	No change
cytoskeleton	GO_CellularComponent-EBI-UniProt-GOA-ACAP-ARAP_13.05.2021_00h00	2.81E-43	11.138614	270	[ABI1, ACACA, ACTB, ACTG1, ACTR2, ACTR3, ADD3, AK1, AK3, AK6, AKAP12, ALDOA, ANK3, APEX1, APP, ARPC4, ARPC5L, ASPM, ATG7, BAIAP2, BCAS2, BIN1, BOD1,	Upregulation

					CALD1, CAP1, CAPZA2, CASK, CBX1, CBX3, CC2D1A, CCDC102A, CCT2, CCT3, CCT6A, CDC42BPA, CDC42EP1, CDK1, CDK16, CDK2, CDK5RAP3, CENPV, CETN2, CFAP20, CFAP298, CFL2, CHAMP1, CHD4, CHORDC1, CHP1, CLIP2, CLTC, CLTCL1, CLU, CNN3, CORO1B, CORO1C, CORO2B, CTNNB1, CTNNBL1, CTSC, DAPK1, DCXR, DENND2A, DHCR24, DIAPH1, DLST, DNM1L, DNMBP, DPP9, DPYSL2, DPYSL3, DSP, DST, DYNC1LI1, DYNLL1, EIF3A, EIF6, EML1, EML3, EML4, EPB41L2, EPB41L3, EPHA3, EPPK1, EXD2, EZR, FARP1, FBLIM1, FERMT1, FERMT2, FGD4, FHOD1, FLII, FNBP1L, FSCN1, FSD1, G6PD, GAN, GAPDH, GBP1, GLE1, GPHN, GSK3B, GTF2F2, HDAC4, HERC2, HINT1, HIP1, HK2, HNRNPC, HSPB11, IFIT5, IFT140, IGF2BP2, IKBKG, ILF3, IST1, ITSN1, IVNS1ABP, JAM3, KIF15, KNTC1, KRT18, KRT19, KRT8, LANCL2, LLGL1, LMNB1, LMOD1, LPP, LRPPRC, LRRFIP1, LRWD1, MACF1, MAD1L1, MAD2L1, MAEA, MAP1B, MAP1LC3A, MCM3, MICAL3, MSN, MTA1, MTRR, MVP, MYADM, MYH10, MYH9, MYO18A, MYO1B, MYO1D, MYO1E, MYO6, NCAPG, NCKAP1, NCOA5, NDRG1, NEDD1, NEK1, NES, NF2, NISCH, NPM1, NUBP2, NUDT19, NUP62, OBSL1, OSBPL10, PAFAH1B1, PALLD, PARP4, PAWR, PBXIP1, PDCD6IP, PIN1, PKN2, PLEC, PPP2R5A, PPP4R3A, PRKCI, PROCR, PRPF19, PTPN12, PXK, PXN, PYCR3, RAB10, RAB23, RAB3D, RAB5A,	

					RAB8A, RABL6, RAC3, RAD21, RAI14, RANBP1, RANGAP1, RDX, REEP2, RHOB, RHOC, RIF1, RIN1, RIPK2, ROR1, RTTN, SAP30BP, SARM1, SBDS, SEC14L2, SEPTIN10, SEPTIN11, SEPTIN2, SEPTIN7, SEPTIN8, SEPTIN9, SESTD1, SH3KBP1, SHMT2, SLC1A4, SLC25A5, SLC2A1, SLC30A9, SMAD4, SMC1A, SMC3, SNX27, SORBS3, SPECC1, SRA1, SRC, STAG2, STOM, STON1, SVIL, SYNE2, SYNJ1, SYNPO, TAP1, TBCA, TBCE, TBCEL, TCP1, TJP1, TLN2, TNIK, TPM3, TPX2, TRADD, TRIP6, TTC28, TTLL12, TUBA4A, TUBA8, TUBB, TUBB3, TUBGCP2, TWF2, UACA, UBR4, VASP, VPS41, VRK1, WDR1, WDR11, WDR35, WRAP73, XIAP, YES1, ZNF207]	
cytoskeleton organization	GO_BiologicalProcess-EBI-UniProt-GOA-ACAP-ARAP_13.05.2021_00h00	2.81E-43	12.967742	201	[ABI1, ACTB, ACTG1, ACTR2, ACTR3, ADD3, AGRN, AHCTF1, ALDOA, AMOTL1, ANK3, ARFGEF1, ARFIP1, ARHGAP12, ARHGAP17, ARHGEF11, ARPC4, ARPC5L, ARPIN, ARRB1, ASPM, ATP2C1, BAIAP2, BIN1, BST1, CALD1, CAP1, CAP2, CAPZA2, CCN2, CDC42BPA, CDC42EP1, CDK1, CDK2, CECR2, CETN2, CFL2, CHMP3, CHORDC1, CHP1, CLIP2, CLTC, CLU, CNN1, CNN3, CORO1B, CORO1C, CORO2B, CORO7, CSRP2, CTNNB1, CXCL12, CYRIB, DIAPH1, DIAPH3, DPYSL2, DPYSL3, DRG1, DSP, DST, DYNC1LI1, DYNLL1, EML1, EML3, EML4, EPB41L2, EPB41L3, EPHA3, EPPK1, ERCC6L, EZR, FAM171A1, FARP1, FAT1, FERMT2, FGD4, FHOD1, FLII, FSCN1, FSD1, GAB1, GAN, GAPDH, GBA2, GCC2, GSK3B, HIP1, IKBKB, IQGAP3,	No change

					ITGB1, ITGB5, JAM3, KNTC1, KPNB1, KRT18, KRT19, KRT8, LLGL1, LMOD1, LRCH3, MACF1, MAD1L1, MAD2L1, MAEA, MAP1B, MAP3K20, MICAL3, MLH1, MSN, MYADM, MYH10, MYH9, MYO18A, MYO1B, MYO1D, MYO1E, MYO6, NCAPG, NCK2, NCKAP1, NDRG1, NECTIN2, NES, NF1, NF2, NISCH, NPM1, NUBP2, NUF2, NUP62, OBSL1, PACSIN3, PAFAH1B1, PALLD, PAWR, PDCD6IP, PHLDB1, PHPT1, PIGU, PIK3CA, PIN1, PKN2, PLEC, PRKAA1, PRKCI, PRKG1, PRUNE1, PTK7, PTPA, PXN, PYCARD, RAC3, RALA, RANBP1, RANGAP1, RAP1GDS1, RCC1, RDX, RHOB, RHOC, RTTN, S100A10, SBDS, SERPINB8, SH3KBP1, SIRT1, SLC9A1, SLIT2, SMAD4, SMC1A, SMC3, SOD1, SON, SORBS3, SPECC1, SRC, STAG2, SVIL, SYNE2, SYNPO, TBCE, TBCEL, TF, TJP1, TLN2, TNIK, TPM3, TPX2, TUBA4A, TUBA8, TUBB, TUBB3, TUBGCP2, TWF2, VASP, WASHC5, WDR1, WRAP73, YEATS4, ZMYM3, ZNF207]	
positive regulation of organelle organization	GO_BiologicalProcess-EBI-UniProt-GOA-ACAP-ARAP_13.05.2021_00h00	2.81E-43	13.201821	87	[ACTR2, ACTR3, APEX1, ARFIP1, ARPC4, ARPC5L, ARPIN, ARRB1, ATL3, BAIAP2, BIN1, CCN2, CCT2, CCT3, CCT6A, CDC42EP1, CFL2, CHTOP, CNOT1, CORO1B, CTBP1, CTNNB1, DHX36, DNM1L, DNMT1, DRG1, FEN1, FERMT2, FHOD1, FMR1, FSCN1, GLYR1, GSK3B, H1-5, KAT7, LMOD1, MACROH2A1, MAP1B, MAP3K4, MARCHF5, MFF, MMAB, MPHOSPH8, MSN, MYO1E, NCAPG, NCK2, NCKAP1, NDRG1, NES, NF2, NMT1,	No change

					NUP62, PDCD6IP, PIP4K2B, PML, PXN, PYCARD, RAD21, RALA, RHOC, RIF1, RNF40, S100A10, SEPTIN7, SEPTIN9, SIRT1, SLC25A4, SLC25A5, SMAD4, SMARCB1, SNW1, SORBS3, SPHK2, SRC, SYNJ1, SYNPO, TCP1, TMED9, TRIM28, VASP, VDAC1, WASHC5, WDR1, WDR35, WDR5, WRAP73]	
regulation of protein-containing complex assembly	GO_BiologicalProcess-EBI-UniProt-GOA-ACAP-ARAP_13.05.2021_00h00	2.81E-43	14.553015	70	[ACTR2, ACTR3, ADD3, ARFGEF1, ARFIP1, ARPC4, ARPC5L, ARPIN, BAIAP2, BIN1, CAPZA2, CDC42EP1, CHAF1B, CHD1L, CLU, CORO1B, CRBN, CUL4B, CYRIB, DRG1, EIF2AK2, ERCC5, FBLIM1, FERMT1, FERMT2, FLII, FSCN1, GBA2, GNL3L, GSK3B, HSP90AA1, HSPA5, IMPACT, JAM3, LMOD1, MAP1B, MMAB, MSN, MYADM, MYD88, MYO1E, NCAPG, NCK2, NCKAP1, NCLN, NDRG1, NOP53, PARP1, PIEZO1, PRUNE1, PSMC2, PSMC3, PSMC6, PYCARD, RDX, RHOC, RIOK3, RIPK2, RPA1, RPA2, RPL13A, SEPTIN8, SEPTIN9, SLIT2, SRC, SVIL, TWF2, VASP, VDAC2, WASHC5]	No change
protein-containing complex assembly	GO_BiologicalProcess-EBI-UniProt-GOA-ACAP-ARAP_13.05.2021_00h00	2.81E-43	13.813979	251	[AAR2, ABT1, ACACA, ACSL3, ACTR2, ACTR3, ADAM10, ADD3, AFDN, AGO2, AHCTF1, ALDOA, ANO6, APIP, APLP2, APPL2, ARFGEF1, ARFIP1, ARPC4, ARPC5L, ARPIN, ASH2L, ATL3, ATPAF1, B2M, BAIAP2, BET1, BIN1, BMP1, CAPZA2, CCNH, CCT3, CDC42EP1, CDC73, CDK1, CELF2, CENPV, CETN2, CHAF1B, CHD1L, CHMP3, CHORDC1, CHP1, CLGN, CLTC, CLU, COIL, COLEC12, CORO1B, CORO7, COX20, CRBN, CRYZ, CTNNB1, CTSC, CUL1, CUL4B, CXCL12, CYRIB,	No change

					DARS1, DDX1, DENR, DIAPH1, DIAPH3, DICER1, DNM1L, DRG1, EHD3, EIF2AK2, EIF2D, EIF3A, EIF3B, EIF3E, EIF3F, EIF6, EPRS1, ERCC5, FASN, FBLIM1, FERMT1, FERMT2, FKBP1A, FLII, FN1, FSCN1, GARS1, GBA2, GCFC2, GEMIN4, GNL3L, GRWD1, GSDMD, GSK3B, H1-10, H1-5, HAT1, HIP1, HNRNPC, HSD17B10, HSP90AA1, HSPA4, HSPA5, IKBKG, IMPACT, JAM3, KCTD12, KCTD15, KNTC1, LAMC1, LLGL1, LMOD1, LRRC8A, MACROH2A1, MAGI1, MAP1B, MAT2A, MCM2, MCM3, MCM4, MCM5, MCM6, MCM7, MMAB, MRTO4, MSN, MYADM, MYD88, MYO1E, NCAPG, NCK2, NCKAP1, NCLN, NDRG1, NDUFA13, NDUFAF7, NDUFS3, NLE1, NOP53, NPM1, NUBPL, NUP205, ORC4, ORC5, OSBPL2, OTUD6B, OXA1L, PARP1, PDCD6IP, PDZD11, PIEZO1, PIK3C2A, PIN1, PML, POGZ, PRMT7, PRPF19, PRUNE1, PSIP1, PSMA3, PSMB10, PSMB2, PSMC2, PSMC3, PSMC6, PSMD1, PSMD12, PSMD14, PSMD2, PSMD6, PSMD7, PSMD8, PSME1, PSME2, PSME4, PSMG1, PUF60, PXN, PYCARD, RAB1A, RAP1GDS1, RBBP4, RDX, RHOC, RIOK3, RIPK2, RNF213, RPA1, RPA2, RPF2, RPL13A, RPL24, RPL38, RPL5, RPS5, RRM1, RTN4, SAMHD1, SCAF11, SCFD1, SEPTIN8, SEPTIN9, SET, SF1, SF3A3, SHMT2, SHPRH, SLC1A5, SLC25A15, SLC2A1, SLC9A1, SLIT2, SMAD4, SMARCB1, SMYD3, SNRNP200,	

					SNRPD3, SNRPE, SNU13, SOAT1, SOD2, SRC, SRSF10, SRSF5, SURF1, SVIL, SYNJ1, TBCA, TBCE, TBCEL, TCP1, TFAM, TGFBR3, TGS1, TIMMDC1, TM9SF4, TPX2, TRADD, TRAPPC4, TSPYL1, TUBGCP2, TWF2, UBTF, USP4, VASP, VDAC2, WASHC5, YAP1, YTHDC1, ZFP36L2, ZNF148, ZNF207]	
actin filament organization	GO_BiologicalProcess-EBI-UniProt-GOA-ACAP-ARAP_13.05.2021_00h00	2.81E-43	16.919739	78	[ABI1, ACTG1, ACTR2, ACTR3, ADD3, ALDOA, ARFGEF1, ARFIP1, ARHGAP12, ARHGAP17, ARPC4, ARPC5L, ARPIN, ARRB1, BAIAP2, BIN1, CALD1, CAPZA2, CCN2, CDC42EP1, CFL2, CORO1B, CORO1C, CORO2B, CORO7, CXCL12, CYRIB, DIAPH1, DIAPH3, DPYSL3, DYNLL1, EZR, FAM171A1, FAT1, FERMT2, FHOD1, FLII, FSCN1, GBA2, HIP1, ITGB1, ITGB5, LMOD1, MICAL3, MYADM, MYO1B, MYO1D, MYO1E, MYO6, NCAPG, NCK2, NCKAP1, NF2, PAWR, PIK3CA, PKN2, PRKCI, PXN, PYCARD, RAC3, RDX, RHOB, RHOC, S100A10, SH3KBP1, SLC9A1, SLIT2, SORBS3, SRC, SVIL, SYNPO, TF, TJP1, TPM3, TWF2, VASP, WASHC5, WDR1]	No change
actomyosin structure organization	GO_BiologicalProcess-EBI-UniProt-GOA-ACAP-ARAP_13.05.2021_00h00	2.81E-43	18.316832	37	[ACTG1, ARRB1, CCN2, CDC42BPA, CFL2, CNN1, CNN3, CORO2B, CSRP2, DYNLL1, EPB41L2, EPB41L3, FAM171A1, FERMT2, FHOD1, FLII, ITGB1, ITGB5, KRT19, KRT8, LMOD1, MYH10, MYH9, MYO18A, NF2, OBSL1, PDCD6IP, PKN2, PXN, RHOC, S100A10, SLC9A1, SMAD4, SORBS3, SRC, TJP1, WDR1]	Upregulation
positive regulation of protein-containing complex assembly	GO_BiologicalProcess-EBI-UniProt-GOA-ACAP-ARAP_13.05.2021_00h00	2.81E-43	16.197184	46	[ACTR2, ACTR3, ARFIP1, ARPC4, ARPC5L, ARPIN, BAIAP2, BIN1, CDC42EP1, CHAF1B, CHD1L, CLU,	No change

					CORO1B, CRBN, CUL4B, DRG1, ERCC5, FERMT1, FERMT2, FSCN1, GNL3L, GSK3B, HSP90AA1, LMOD1, MAP1B, MMAB, MSN, MYD88, MYO1E, NCAPG, NCK2, NCKAP1, NDRG1, PARP1, PIEZO1, PSMC2, PSMC3, PSMC6, PYCARD, RHOC, RIPK2, RPA1, RPA2, SRC, VASP, WASHC5]	
regulation of actin cytoskeleton organization	GO_BiologicalProcess-EBI-UniProt-GOA-ACAP-ARAP_13.05.2021_00h00	2.81E-43	15.873015	60	[ACTG1, ACTR2, ACTR3, ADD3, ARFGEF1, ARFIP1, ARHGAP17, ARPC4, ARPC5L, ARPIN, BAIAP2, BIN1, BST1, CAPZA2, CCN2, CDC42EP1, CFL2, CLU, CORO1B, CORO2B, CXCL12, CYRIB, DYNLL1, EPHA3, EZR, FERMT2, FHOD1, FLII, FSCN1, GBA2, IQGAP3, JAM3, LMOD1, MYADM, MYO1E, NCAPG, NCK2, NCKAP1, NF2, PHPT1, PIK3CA, PKN2, PXN, PYCARD, RAC3, RDX, RHOB, RHOC, S100A10, SLC9A1, SLIT2, SMAD4, SORBS3, SVIL, SYNPO, TJP1, TWF2, VASP, WASHC5, WDR1]	No change
regulation of cytoskeleton organization	GO_BiologicalProcess-EBI-UniProt-GOA-ACAP-ARAP_13.05.2021_00h00	2.81E-43	14.208633	79	[ACTG1, ACTR2, ACTR3, ADD3, ARFGEF1, ARFIP1, ARHGAP17, ARPC4, ARPC5L, ARPIN, BAIAP2, BIN1, BST1, CAPZA2, CCN2, CDC42EP1, CFL2, CHMP3, CLTC, CLU, CORO1B, CORO2B, CXCL12, CYRIB, DIAPH1, DRG1, DYNLL1, EML3, EPHA3, EZR, FERMT2, FHOD1, FLII, FSCN1, FSD1, GBA2, GSK3B, IQGAP3, JAM3, LMOD1, MAP1B, MYADM, MYO1E, NCAPG, NCK2, NCKAP1, NDRG1, NES, NF2, NPM1, NUBP2, NUP62, PAFAH1B1, PDCD6IP, PHLDB1, PHPT1, PIK3CA, PKN2, PRKAA1, PRUNE1, PXN, PYCARD, RAC3, RDX, RHOB, RHOC, S100A10, SLC9A1,	No change

					SLIT2, SMAD4, SORBS3, SVIL, SYNPO, TJP1, TPX2, TWF2, VASP, WASHC5, WDR1]	
actin cytoskeleton	GO_CellularComponent-EBI-UniProt-GOA-ACAP-ARAP_13.05.2021_00h00	2.81E-43	16.044777	86	[ACACA, ACTB, ACTG1, ACTR2, ACTR3, ALDOA, APEX1, ARPC4, ARPC5L, BAIAP2, BIN1, CALD1, CAP1, CAPZA2, CASK, CCDC102A, CDC42BPA, CFL2, CNN3, CORO1B, CORO1C, CORO2B, DAPK1, DENND2A, DPYSL3, DYNLL1, EPB41L2, EPHA3, EZR, FBLIM1, FERMT2, FHOD1, FLII, FSCN1, GBP1, HDAC4, HNRNPC, IFIT5, ILF3, ITSN1, JAM3, KNTC1, KRT19, LANCL2, LLGL1, LMOD1, LPP, MACF1, MAEA, MYADM, MYH10, MYH9, MYO18A, MYO1B, MYO1D, MYO1E, MYO6, NCAPG, NCKAP1, NCOA5, NF2, PALLD, PAWR, PDCD6IP, PTPN12, PXN, RAB5A, RDX, ROR1, SEPTIN11, SEPTIN2, SEPTIN7, SEPTIN9, SLC2A1, SPECC1, SRC, SVIL, SYNPO, TJP1, TLN2, TPM3, TRIP6, TWF2, VASP, WDR1, YES1]	Upregulation
microtubule cytoskeleton	GO_CellularComponent-EBI-UniProt-GOA-ACAP-ARAP_13.05.2021_00h00	2.81E-43	10.99631	149	[AK3, AK6, APEX1, APP, ASPM, BAIAP2, BCAS2, BOD1, CBX1, CBX3, CC2D1A, CCT2, CCT3, CCT6A, CDK1, CDK16, CDK2, CDK5RAP3, CENPV, CETN2, CFAP20, CHAMP1, CHD4, CHORDC1, CHP1, CLIP2, CLTC, CLTCL1, CTNNB1, CTNNBL1, CTSC, DCXR, DIAPH1, DLST, DNM1L, DPP9, DPYSL2, DST, DYNC1LI1, DYNLL1, EIF3A, EIF6, EML1, EML3, EML4, EZR, FLII, FSD1, G6PD, GAPDH, GLE1, GSK3B, GTF2F2, HERC2, HK2, HSPB11, IFT140, IKBKG, IST1, KIF15, KNTC1, KRT18, LRPPRC, LRWD1, MACF1, MAD1L1, MAD2L1, MAEA, MAP1B, MAP1LC3A, MCM3, MICAL3, MTA1, MYH9,	Upregulation

					NCAPG, NDRG1, NEDD1, NEK1, NISCH, NPM1, NUBP2, NUDT19, NUP62, OBSL1, PAFAH1B1, PARP4, PBXIP1, PDCD6IP, PIN1, PKN2, PPP2R5A, PPP4R3A, PRKCI, PROCR, PRPF19, PXK, PXN, PYCR3, RAB23, RAB3D, RAB8A, RABL6, RAD21, RANBP1, RANGAP1, REEP2, RIF1, RTTN, SARM1, SBDS, SEC14L2, SEPTIN10, SEPTIN11, SEPTIN2, SEPTIN7, SEPTIN8, SEPTIN9, SHMT2, SLC1A4, SLC25A5, SMAD4, SMC1A, SMC3, SPECC1, SRA1, STAG2, SVIL, SYNJ1, TAP1, TBCA, TBCE, TCP1, TPX2, TTC28, TTLL12, TUBA4A, TUBA8, TUBB, TUBB3, TUBGCP2, UBR4, VPS41, VRK1, WDR11, WDR35, WRAP73, XIAP, YES1, ZNF207]	
cellular protein-containing complex assembly	GO_BiologicalProcess-EBI-UniProt-GOA-ACAP-ARAP_13.05.2021_00h00	2.81E-43	14.666667	176	[AAR2, ABT1, ACTR2, ACTR3, ADD3, AGO2, AHCTF1, ARFGEF1, ARFIP1, ARPC4, ARPC5L, ARPIN, ATPAF1, BAIAP2, BIN1, CAPZA2, CCNH, CDC42EP1, CDC73, CELF2, CENPV, CETN2, CHAF1B, CHD1L, CHMP3, CLTC, CLU, COIL, COLEC12, CORO1B, CORO7, COX20, CUL4B, CYRIB, DDX1, DENR, DIAPH1, DIAPH3, DICER1, DNM1L, DRG1, EIF2AK2, EIF2D, EIF3A, EIF3B, EIF3E, EIF3F, EIF6, ERCC5, FKBP1A, FLII, GARS1, GBA2, GCFC2, GEMIN4, GRWD1, H1-10, H1-5, HAT1, HIP1, HNRNPC, HSP90AA1, HSPA4, LMOD1, MACROH2A1, MAP1B, MCM2, MCM3, MCM4, MCM5, MCM6, MCM7, MMAB, MRTO4, MYADM, MYD88, MYO1E, NCAPG, NCK2, NCKAP1, NDRG1, NDUFA13, NDUFAF7, NDUFS3, NLE1, NOP53,	No change

					NPM1, NUBPL, NUP205, ORC4, ORC5, OTUD6B, OXA1L, PARP1, PIK3C2A, PIN1, POGZ, PRMT7, PRPF19, PRUNE1, PSIP1, PSMA3, PSMB10, PSMB2, PSMC2, PSMC3, PSMC6, PSMD1, PSMD12, PSMD14, PSMD2, PSMD6, PSMD7, PSMD8, PSME1, PSME2, PSME4, PSMG1, PUF60, PXN, PYCARD, RAP1GDS1, RBBP4, RDX, RPA1, RPA2, RPF2, RPL13A, RPL24, RPL38, RPL5, RPS5, RTN4, SCAF11, SEPTIN8, SEPTIN9, SET, SF1, SF3A3, SHPRH, SLC25A15, SLIT2, SMAD4, SMARCB1, SMYD3, SNRNP200, SNRPD3, SNRPE, SNU13, SRC, SRSF10, SRSF5, SURF1, SVIL, SYNJ1, TBCA, TBCE, TBCEL, TCP1, TFAM, TGFBR3, TGS1, TIMMDC1, TM9SF4, TPX2, TSPYL1, TUBGCP2, TWF2, UBTF, USP4, VASP, VDAC2, WASHC5, YTHDC1, ZFP36L2, ZNF207]	
actin polymerization or depolymerization	GO_BiologicalProcess-EBI-UniProt-GOA-ACAP-ARAP_13.05.2021_00h00	2.81E-43	16.37931	38	[ABI1, ACTR2, ACTR3, ADD3, ARFGEF1, ARFIP1, ARPC4, ARPC5L, ARPIN, BAIAP2, BIN1, CAPZA2, CDC42EP1, CFL2, CORO1B, CORO7, CXCL12, CYRIB, DIAPH1, DIAPH3, FLII, GBA2, LMOD1, MICAL3, MYADM, MYO1E, NCAPG, NCK2, NCKAP1, PIK3CA, PYCARD, RDX, SLIT2, SVIL, TWF2, VASP, WASHC5, WDR1]	No change
protein-DNA complex assembly	GO_BiologicalProcess-EBI-UniProt-GOA-ACAP-ARAP_13.05.2021_00h00	2.81E-43	16.18123	50	[CCNH, CENPV, CETN2, CHAF1B, CHD1L, CUL4B, ERCC5, GRWD1, H1-10, H1-5, HAT1, MACROH2A1, MCM2, MCM3, MCM4, MCM5, MCM6, MCM7, NPM1, ORC4, ORC5, PARP1, POGZ, PSMA3, PSMB10, PSMB2, PSMC2, PSMC3, PSMC6, PSMD1, PSMD12, PSMD14, PSMD2,	No change

					PSMD6, PSMD7, PSMD8, PSME1, PSME2, PSME4, RBBP4, RPA1, RPA2, SET, SHPRH, SLC25A15, SMARCB1, SMYD3, TSPYL1, UBTF, ZFP36L2]	
regulation of actin filament organization	GO_BiologicalProcess-EBI-UniProt-GOA-ACAP-ARAP_13.05.2021_00h00	2.81E-43	16.494844	48	[ACTG1, ACTR2, ACTR3, ADD3, ARFGEF1, ARFIP1, ARPC4, ARPC5L, ARPIN, BAIAP2, BIN1, CAPZA2, CCN2, CDC42EP1, CFL2, CORO1B, CORO2B, CXCL12, CYRIB, DYNLL1, FERMT2, FHOD1, FLII, GBA2, LMOD1, MYADM, MYO1E, NCAPG, NCK2, NCKAP1, NF2, PIK3CA, PKN2, PXN, PYCARD, RDX, RHOC, S100A10, SLC9A1, SLIT2, SORBS3, SVIL, SYNPO, TJP1, TWF2, VASP, WASHC5, WDR1]	No change
actin filament bundle assembly	GO_BiologicalProcess-EBI-UniProt-GOA-ACAP-ARAP_13.05.2021_00h00	2.81E-43	18.072289	30	[ACTG1, ARRB1, BAIAP2, CALD1, CCN2, CORO1B, CORO2B, DPYSL3, DYNLL1, EZR, FAM171A1, FERMT2, FHOD1, FSCN1, ITGB1, ITGB5, MYO1B, NCK2, NF2, PAWR, PKN2, PXN, RDX, RHOC, S100A10, SLC9A1, SORBS3, SRC, SYNPO, TJP1]	No change
regulation of actin polymerization or depolymerization	GO_BiologicalProcess-EBI-UniProt-GOA-ACAP-ARAP_13.05.2021_00h00	2.81E-43	16.582914	33	[ACTR2, ACTR3, ADD3, ARFGEF1, ARFIP1, ARPC4, ARPC5L, ARPIN, BAIAP2, BIN1, CAPZA2, CDC42EP1, CFL2, CORO1B, CXCL12, CYRIB, FLII, GBA2, LMOD1, MYADM, MYO1E, NCAPG, NCK2, NCKAP1, PIK3CA, PYCARD, RDX, SLIT2, SVIL, TWF2, VASP, WASHC5, WDR1]	No change
positive regulation of biological process	GO_BiologicalProcess-EBI-UniProt-GOA-ACAP-ARAP_13.05.2021_00h00	3.67E-55	9.244851	606	[ABAT, ABCB7, ABCC1, ABI1, ABT1, ACACA, ACSL3, ACSL4, ACTB, ACTG1, ACTR2, ACTR3, ADAM10, ADARB1, ADNP, AFDN, AGO2, AGPAT2, AGRN, AKAP12, AKR1B1, ANK3, ANKFY1, ANO6, ANXA3, APAF1, APEX1, APLP2, APP, APPL2, ARAF, ARFGEF1, ARFIP1, ARHGEF11,	No change

					ARHGEF12, ARID1B, ARPC4, ARPC5L, ARPIN, ARPP19, ARRB1, ASH2L, ASNS, ASPM, ASPSCR1, ATG4B, ATG7, ATL3, ATP1A1, ATP1B3, ATP2B1, ATP2C1, ATP6V0C, B2M, B3GAT3, B4GALT1, B4GALT5, BAIAP2, BAZ1B, BIN1, BST1, C1QBP, C2CD2L, C3orf38, CAB39L, CAMK2D, CASK, CASP3, CBR1, CBX8, CC2D1A, CCDC22, CCN2, CCNK, CCT2, CCT3, CCT6A, CD200, CD276, CD81, CDC34, CDC42EP1, CDC5L, CDC73, CDK1, CDK2, CDK5RAP3, CEMIP, CENPV, CFAP20, CFL2, CHAF1B, CHAMP1, CHD1L, CHD7, CHMP3, CHORDC1, CHP1, CHTOP, CIRBP, CLIC1, CLTCL1, CLU, CNOT1, CNOT10, COLEC12, CORO1B, CORO1C, CPNE2, CRABP2, CRBN, CSPG4, CTBP1, CTNNB1, CTNNBL1, CTSC, CUL1, CUL4B, CUX1, CXCL12, CYCS, CYRIB, DAB2, DAGLB, DAPK1, DCN, DCPS, DCXR, DDX1, DERL1, DHPS, DHX34, DHX36, DIAPH1, DICER1, DIMT1, DIPK2A, DNM1L, DNMT1, DPYSL2, DPYSL3, DRG1, DUSP3, DVL2, DYNC1LI1, DYNLL1, ECE1, EDF1, EEF1E1, EFNB2, EHD3, EIF2AK2, EIF2B1, EIF2S1, EIF3E, EIF6, ELP3, ELP5, ENY2, EPB41L2, EPHA2, EPHA3, EPHB4, EPM2AIP1, ERAP1, ERBB2, ERCC5, ETFDH, EZR, FASN, FBLN2, FBXW8, FEN1, FERMT1, FERMT2, FGD4, FGF2, FHL1, FHOD1, FKBP1A, FLRT2, FMR1, FN1, FNBP1L, FSCN1, FTO, G6PD, GAB1, GAPDH, GARS1, GBP1, GIPC1, GLMN, GLMP, GLUD1, GLUL, GLYR1, GNL3L, GOLT1B, GOT1, GPC1, GPD1L, GPI,	

					GPSM1, GSDMD, GSK3B, GSTM2, GSTP1, GTF2F1, GTF2F2, GTF2I, H1-10, H1-5, HADH, HADHA, HDAC2, HDAC4, HDGFL2, HEXB, HGS, HIP1, HK1, HK2, HLA-A, HLA-B, HMCES, HMOX1, HNRNPA0, HNRNPC, HNRNPLL, HNRNPR, HPX, HSD17B4, HSP90AA1, HSPA5, HSPB6, HSPE1, IFIT5, IGF2BP1, IKBKB, IKBKG, ILF3, IMPACT, IPO5, IQGAP3, IST1, ITGA3, ITGAV, ITGB1, ITM2C, ITSN1, JAG1, JAM3, KAT7, KAT8, KCNMA1, KCNQ1, KDM1A, LAMC1, LAMP1, LANCL2, LARP1, LARP4B, LARP6, LDHA, LIMS1, LLGL1, LMCD1, LMO7, LMOD1, LRCH4, LRRC32, LRRC8A, LRRFIP1, LRRK1, LUM, MACF1, MACROH2A1, MAD2L1, MAGI1, MALT1, MAOA, MAP1B, MAP2K3, MAP3K20, MAP3K4, MAP4K4, MARCHF5, MATR3, MCAM, MEAK7, MED17, MED23, MED30, METTL14, MFF, MGMT, MLH1, MMAB, MME, MOV10, MPHOSPH8, MSH2, MSN, MTA1, MTCH1, MVD, MYADM, MYD88, MYH10, MYH9, MYO18A, MYO1E, MYO6, NCAPG, NCK2, NCKAP1, NCL, NCOA7, NDRG1, NDUFA13, NDUFA4, NECTIN2, NEDD4, NELFCD, NENF, NES, NF1, NF2, NFATC2IP, NFKB2, NIBAN1, NIBAN2, NLE1, NMD3, NMT1, NNT, NOCT, NOL11, NOP53, NPM1, NPR2, NQO1, NSMCE3, NSUN5, NT5C3B, NUB1, NUDT16, NUP62, NUTF2, OBSL1, OSBPL11, OSBPL8, OXSR1, P3H1, PACSIN3, PAFAH1B1, PAFAH1B2, PAIP1, PANX1, PARP1,	

					PARP14, PATZ1, PAWR, PC, PCK2, PDCD6IP, PELO, PFKM, PHLDB1, PHPT1, PIEZO1, PIGU, PIK3C2A, PIK3CA, PIN1, PIP4K2B, PITHD1, PKM, PKN2, PLA2G4A, PLCG1, PLD1, PLPP3, PLXNB2, PML, PNP, POGZ, POLR1C, POLR1D, POLR2A, POLR2B, POLR2H, POLR2I, POLR3A, POLR3C, PPIB, PPIE, PPM1B, PPP1CA, PPP2R5A, PPP3CA, PPP5C, PRDX6, PRKAA1, PRKAB2, PRKCI, PRKCSH, PRPF19, PSMA3, PSMB10, PSMB2, PSMC2, PSMC3, PSMC6, PSMD1, PSMD12, PSMD14, PSMD2, PSMD6, PSMD7, PSMD8, PSME1, PSME2, PSME4, PSPC1, PTGES2, PTGIS, PTK7, PTPA, PTRH2, PXN, PYCARD, RAB1A, RAB3B, RAB3D, RAB5A, RAC3, RAD21, RALA, RANBP1, RAP2B, RAP2C, RBM22, RBM4, RCN3, RDH10, RDX, RFC2, RFC3, RFC5, RHEB, RHOB, RHOC, RIF1, RIOK3, RIPK2, RNF2, RNF40, RNPS1, ROR1, RPA1, RPA2, RPL5, RPLP1, RPRD1B, RPS15A, RPS9, RRAS2, RTN4, RYBP, S100A10, SAAL1, SAE1, SAP30BP, SARM1, SCARB2, SCP2, SCRIB, SEC14L2, SELENOT, SEMA3C, SEPTIN7, SEPTIN9, SERPINB9, SERPINE2, SESN2, SH3BP4, SH3KBP1, SHMT2, SIRT1, SLC12A2, SLC1A1, SLC25A12, SLC25A4, SLC25A5, SLC30A9, SLC44A2, SLC9A1, SLIT2, SMAD4, SMARCA1, SMARCB1, SMARCC1, SMARCD3, SMARCE1, SMCHD1, SMPD1, SMYD3, SNF8, SNW1, SNX1, SNX5, SOAT1, SOD1, SOD2, SORBS3, SORT1, SPARC,	

					SPHK2, SQSTM1, SRA1, SRC, SRSF5, STAT1, STAT5B, STK24, STOM, STON1, SULT1E1, SUPT16H, SVIL, SYNE2, SYNJ1, SYNPO, TAB2, TAF6L, TBC1D10A, TBK1, TCAF1, TCP1, TDRD3, TENM3, TF, TFAM, TGFBR3, TGM2, THBS1, THEM4, TIGAR, TJP1, TM9SF4, TMED9, TMEM119, TNIK, TOMM7, TPBG, TPX2, TRA2A, TRA2B, TRADD, TRIM21, TRIM25, TRIM28, TRIM56, TRIO, TRIP6, TRMT10C, TSC2, TSPO, TTC5, TUT4, TWF2, TXN, UACA, UBA2, UBTF, USP22, USP5, USP7, USP9X, VASP, VDAC1, VDAC2, VLDLR, VRK3, VTN, WASHC5, WDR1, WDR35, WDR5, WFS1, WRAP73, WWP2, XIAP, XPO4, YAP1, YBX1, YEATS4, YES1, ZC3HAV1, ZCCHC3, ZDHHC13, ZFP36L2, ZMPSTE24, ZNF148]	
negative regulation of biological process	GO_BiologicalProcess-EBI-UniProt-GOA-ACAP-ARAP_13.05.2021_00h00	3.67E-55	9.647912	559	[AARS1, AASS, ABAT, ABCB7, ABCE1, ABI1, ACADVL, ACOT8, ADAM10, ADAMTS9, ADARB1, ADD3, ADNP, AFDN, AGO2, AKR1B1, ALB, ANK3, ANKRD13A, ANXA5, ANXA7, APAF1, APBB2, APEX1, APIP, APLP2, APP, APPL2, ARAF, ARFGEF1, ARFIP1, ARHGAP12, ARPIN, ARPP19, ARRB1, ASB1, ASNS, ASPM, ATG7, ATP1A1, ATP2B1, B2M, B4GALT1, BAG1, BANF1, BIN1, BNIP1, BOD1, C1QBP, CAMK2D, CAPN5, CAPZA2, CASK, CASP3, CBX1, CBX2, CBX3, CBX5, CBX8, CC2D1A, CCDC22, CCN2, CD109, CD200, CDC34, CDC5L, CDC73, CDK1, CDK2, CDK5RAP3, CEMIP, CENPV, CFDP1, CHD7, CHID1, CHMP3, CHORDC1, CHP1, CIRBP, CLTC, CLU, CNN1, CNOT1, CNOT10, COL18A1,	No change

					COL5A1, COL5A2, COLEC12, CORO1B, CORO1C, CORO2B, CRBN, CRTAP, CSTB, CTBP1, CTNNB1, CTSA, CTSC, CUL1, CUX1, CXCL12, CYP51A1, CYRIB, DAB2, DAPK1, DCBLD2, DCPS, DGUOK, DHCR24, DHFR, DHX34, DHX36, DIAPH1, DICER1, DIPK2A, DNM1L, DNMT1, DPYSL3, DUSP3, DVL2, DYNC1LI1, DYNLL1, EED, EEF1E1, EFEMP1, EFNB2, EIF2AK2, EIF2S1, EIF3A, EIF3E, EIF4G3, EIF6, ENPP1, ENY2, EPHA2, EPHA3, EPPK1, EPRS1, ERBB2, ERCC5, ERLIN2, EZR, FAM3A, FASN, FERMT1, FERMT2, FGF2, FHL1, FHL2, FKBP1A, FLII, FMR1, FN1, FTH1, FTO, G6PD, GAPDH, GARS1, GATAD2A, GBE1, GBP1, GCFC2, GIPC1, GLA, GLMN, GLO1, GLRX3, GNL3L, GOLT1B, GOT1, GPC1, GPD1L, GPI, GPR108, GPSM1, GSK3B, GSTM2, GSTP1, H1-10, H1-5, HADH, HADHA, HAT1, HDAC2, HDAC4, HELZ, HERC4, HGS, HHIP, HK2, HLA-A, HLA-B, HMOX1, HNRNPA0, HNRNPC, HNRNPR, HSP90B1, HSPA5, HSPB6, HSPG2, IDH2, IFIT5, IGF2BP1, IGF2BP2, IGFBP7, IKBKB, IKBKG, ILF3, IMPACT, IPO5, IQGAP3, IREB2, ITGA3, ITGAV, ITGB1, ITIH4, ITM2C, IVNS1ABP, JAG1, JAGN1, JAM3, KAT7, KAT8, KCNMA1, KCNQ1, KDM1A, KDM4B, KNTC1, KRT18, LANCL2, LARP1, LARP4B, LEMD2, LIG3, LIMS1, LLGL1, LMCD1, LMOD1, LNPEP, LRPPRC, LRRC32, LRRFIP1, LRRK1, LTBP1, MACROH2A1, MAD1L1, MAD2L1, MAEA, MAGI1, MALT1, MAP1B, MAP2K3, MAP3K20,	

					MAP4K4, MARCHF5, MBD3, MCM2, MCM7, MDC1, MEAK7, METTL14, MGA, MGMT, MLH1, MMAB, MORF4L1, MOV10, MPHOSPH8, MRTO4, MSH2, MSH6, MTA1, MVK, MVP, MYADM, MYD88, MYH9, MYO18A, NCAPG, NCK2, NCL, NCOA5, NCOA7, NDRG1, NDUFA13, NDUFS3, NEDD4, NELFCD, NENF, NES, NF1, NF2, NIBAN1, NIBAN2, NISCH, NLE1, NNT, NOCT, NOP53, NPLOC4, NPM1, NPR2, NQO1, NT5C3B, NUBP2, NUCB2, NUDT16, NUDT16L1, NUP205, NUP35, NUP62, NUTF2, NXN, OSBPL8, OXSR1, P3H1, P3H3, PACSIN3, PAFAH1B1, PAIP1, PARP1, PARP14, PATZ1, PAWR, PBXIP1, PC, PDS5B, PEBP1, PELO, PGAM5, PGP, PHPT1, PIK3CA, PIN1, PIP4K2B, PKN2, PLCG1, PLPP3, PLXNB2, PML, POLR2A, POLR2B, POLR2H, POLR2I, PPM1B, PPP1R14A, PPP2R5A, PPP3CA, PPP5C, PRKAA1, PRKCI, PRKG1, PROCR, PRPF19, PSMA3, PSMB10, PSMB2, PSMC2, PSMC3, PSMC6, PSMD1, PSMD12, PSMD14, PSMD2, PSMD6, PSMD7, PSMD8, PSME1, PSME2, PSME4, PSPC1, PTBP2, PTBP3, PTGIS, PTPA, PTPN12, PTPRG, PTRH2, PUS7, PXK, PYCARD, PYCR1, QARS1, QSOX1, RAB23, RAD21, RAD9A, RANGAP1, RAP2B, RAP2C, RBBP4, RBM4, RDX, RGS10, RHEB, RHOB, RIF1, RIOK3, RIPK2, RNF2, RNF213, RNF40, RNPS1, RPA2, RPL10A, RPL13, RPL13A, RPL18A, RPL22, RPL24, RPL27, RPL32, RPL36, RPL38, RPL5, RPLP1, RPLP2, RPS13,	

					RPS15A, RPS16, RPS18, RPS21, RPS23, RPS4Y1, RPS5, RPS8, RPS9, RTN4, RYBP, SACS, SAMHD1, SARM1, SARS1, SCAI, SCFD1, SCRIB, SEMA3C, SERPINB1, SERPINB2, SERPINB8, SERPINB9, SERPINE2, SESN2, SESTD1, SET, SF1, SH3BP4, SH3KBP1, SHFL, SHMT2, SIRT1, SLC12A2, SLC1A1, SLC25A12, SLC25A15, SLC25A4, SLC25A5, SLC9A1, SLIT2, SMAD4, SMARCB1, SMARCC1, SMARCE1, SMCHD1, SMPD1, SMYD3, SNW1, SNX5, SOD1, SOD2, SON, SORBS3, SORT1, SPARC, SPART, SPHK2, SQSTM1, SRA1, SRC, SRSF10, SRSF2, SSB, STAT1, STAT2, STAT5B, STK24, STON1, STRN3, STXBP3, SUFU, SVIL, TAB2, TAX1BP1, TBC1D4, TBK1, TCAF1, TGFBI, TGFBR3, THADA, THBS1, THEM4, THOC2, TIGAR, TIPRL, TJP1, TMEM119, TOM1L2, TPBG, TPX2, TRADD, TRIM21, TRIM25, TRIM28, TRIM59, TRIO, TRIP12, TRIP13, TSC2, TSPO, TTC39B, TTLL12, TUT4, TWF2, TXN, UACA, UBA2, UBA3, UBAC2, UBE2L6, UBR2, UFD1, URI1, USP28, USP4, USP7, USP9X, UTP20, VAT1, VDAC1, VDAC2, VPS25, VRK3, VTN, WDR35, WDR5, WFS1, WWC2, WWP2, XIAP, YAP1, YBX1, YES1, YOD1, ZC3HAV1, ZFAND6, ZFP36L2, ZMPSTE24, ZNF148, ZNF207]	
regulation of metabolic process	GO_BiologicalProcess-EBI-UniProt-GOA-ACAP-ARAP_13.05.2021_00h00	3.67E-55	9.076944	650	[AARS1, AASS, ABAT, ABCB7, ABCE1, ABI1, ABT1, ACACA, ACADM, ACADVL, ACO1, ACOT8, ACOX1, ACSL3, ACTB, ACTG1, ACTR2, ACTR3, ADAM10, ADARB1, ADNP, AFDN, AGO2,	No change

					AGPAT2, AGRN, AK4, AKAP12, AKR1B1, ANK3, ANKRD13A, ANXA3, ANXA7, APAF1, APBB2, APEX1, APLP2, APP, APPL2, ARAF, ARFGEF1, ARHGEF11, ARID1B, ARPP19, ARRB1, ASAH1, ASB1, ASPSCR1, ATG4B, ATG7, ATP1A1, ATP2B1, ATP6V0A1, ATP6V0C, ATP6V1A, ATP6V1B2, B2M, BAZ1B, BIN1, BOD1, BRD3, BST1, C1QBP, CAB39L, CAMK2D, CASK, CASP3, CBR1, CBX1, CBX2, CBX3, CBX5, CBX8, CC2D1A, CCDC22, CCN2, CCNH, CCNK, CCNL1, CCT2, CCT3, CCT6A, CD109, CD200, CD276, CD81, CDC5L, CDC73, CDK1, CDK16, CDK17, CDK2, CDK5RAP3, CELF2, CEMIP, CENPV, CHAMP1, CHD2, CHD4, CHD7, CHID1, CHORDC1, CHP1, CHTOP, CIRBP, CLTC, CLU, CNOT1, CNOT10, COLEC12, CORO1C, CRABP2, CRTAP, CSPG4, CSTB, CTBP1, CTNNB1, CTSA, CTSC, CUX1, CWC22, CYCS, CYP51A1, CYRIB, DAB2, DAGLB, DAPK1, DCN, DCPS, DCXR, DDX1, DENR, DERL1, DHCR24, DHCR7, DHFR, DHX34, DHX36, DICER1, DIMT1, DIPK2A, DNM1L, DNMT1, DUSP3, DVL2, DYNLL1, ECE1, EDF1, EED, EEF1G, EFEMP1, EFL1, EIF2AK2, EIF2B1, EIF2D, EIF2S1, EIF3A, EIF3B, EIF3E, EIF3F, EIF4G3, EIF6, ELP3, ELP4, ENPP1, ENY2, EPHA2, EPHA3, EPHB4, EPM2AIP1, EPRS1, ERBB2, ERLIN2, ETFDH, EZR, FAM3A, FASN, FBXW8, FERMT1, FERMT2, FGF2, FHL2, FHOD1, FKBP1A, FMR1, FN1, FTO, FUT8, G6PD, GAPDH, GARS1, GATAD2A, GBP1, GCFC2, GIPC1, GLA,	

					GLE1, GLMN, GLMP, GLO1, GLYR1, GNL3L, GOLT1B, GOT1, GPD1L, GPI, GPSM1, GSDMD, GSK3B, GSTP1, GTF2F1, GTF2I, H1-10, H1-5, HADH, HADHA, HAT1, HDAC2, HDAC4, HDGFL2, HELZ, HEXB, HGS, HINT1, HIP1, HK1, HK2, HLA-A, HLA-B, HMCES, HMGCS1, HMOX1, HNRNPA0, HNRNPC, HNRNPH1, HNRNPLL, HNRNPR, HPX, HSD17B4, HSP90AA1, HSPA5, HSPE1, HTATSF1, IDI1, IGF2BP1, IGF2BP2, IGFBP7, IKBKB, IKBKG, ILF3, IMPACT, IPO5, IQGAP3, IREB2, IST1, ITGA3, ITGAV, ITGB1, ITIH4, ITM2C, IVNS1ABP, JAG1, KAT7, KAT8, KCNQ1, KDM1A, KDM3B, KDM4B, KPNB1, LACTB, LANCL2, LARP1, LARP4B, LARP6, LIG3, LIMS1, LLGL1, LMCD1, LMO7, LNPEP, LRPPRC, LRRC32, LRRFIP1, LRRK1, LSS, LTBP1, LUM, MACROH2A1, MAD2L1, MAEA, MAGI1, MALT1, MAP2K3, MAP3K20, MAP3K4, MAP4K4, MATR3, MBD3, MBOAT7, MCM2, MCM7, ME1, MED17, MED23, MED30, METTL14, MGA, MGMT, MLF2, MLH1, MMAB, MORF4L1, MOV10, MPHOSPH8, MRTO4, MSH2, MSH6, MSN, MTA1, MTCH1, MVD, MVK, MVP, MYADM, MYD88, MYH9, MYO1E, MYO6, NCK2, NCL, NCOA5, NCOA7, NDUFA13, NDUFA4, NEDD4, NEDD8, NELFCD, NF1, NF2, NFATC2IP, NFKB2, NIBAN1, NIBAN2, NLE1, NMD3, NNT, NOCT, NOL11, NOP53, NPLOC4, NPM1, NQO1, NSMCE3, NSUN5, NT5C3B, NUB1, NUDT15, NUDT16, NUDT16L1, NUP205, NUP35, NUP62,	

					NUTF2, NXN, OGFOD1, ORC5, OSBPL8, OXSR1, P3H1, PACSIN3, PAFAH1B2, PAIP1, PANX1, PARG, PARL, PARP1, PARP14, PARP4, PATZ1, PAWR, PBXIP1, PC, PCBP3, PDK1, PEBP1, PELO, PFKM, PGAM5, PGP, PHGDH, PIK3C2A, PIK3CA, PIN1, PIP4K2B, PIR, PITHD1, PKM, PKN2, PLD1, PLD3, PLPP3, PLXNB2, PML, PNP, POFUT1, POGZ, POLR1C, POLR1D, POLR2A, POLR2B, POLR2H, POLR2I, POLR3A, POLR3C, PPIB, PPIE, PPM1B, PPP1CA, PPP1R14A, PPP1R7, PPP2R5A, PPP3CA, PPP5C, PRDX6, PRKAA1, PRKAB2, PRKCI, PRKCSH, PRMT7, PRPF19, PSIP1, PSMA3, PSMB10, PSMB2, PSMC2, PSMC3, PSMC6, PSMD1, PSMD12, PSMD14, PSMD2, PSMD6, PSMD7, PSMD8, PSME1, PSME2, PSME4, PSPC1, PTBP2, PTBP3, PTGES2, PTGIS, PTPA, PTRH2, PUF60, PUS7, PXN, PYCARD, QARS1, QSOX1, RAB1A, RAB8A, RAD21, RAP2B, RAP2C, RBBP4, RBM22, RBM4, RCN3, RDH10, RDX, RFC2, RFC3, RFC5, RHEB, RIF1, RIOK3, RIPK2, RNF2, RNF40, RNPS1, ROR1, RPA2, RPL10A, RPL13, RPL13A, RPL18A, RPL22, RPL24, RPL27, RPL32, RPL36, RPL38, RPL5, RPLP1, RPLP2, RPRD1A, RPRD1B, RPS13, RPS15A, RPS16, RPS18, RPS21, RPS23, RPS4Y1, RPS5, RPS8, RPS9, RTN4, RYBP, SAE1, SAP30BP, SARNP, SARS1, SCAI, SCARB2, SCFD1, SCRIB, SEC14L2, SELENOT, SERPINB1, SERPINB2, SERPINB8, SERPINB9, SERPINE2, SESN2,	

					SET, SF1, SH3BP4, SHFL, SHMT2, SIRT1, SLC1A1, SLC25A12, SLC25A15, SLC25A4, SLC25A5, SLC30A9, SLC9A1, SLIT2, SMAD4, SMARCA1, SMARCAD1, SMARCB1, SMARCC1, SMARCD3, SMARCE1, SMC3, SMCHD1, SMPD1, SMU1, SMYD3, SNF8, SNW1, SNX1, SNX5, SOAT1, SOD1, SOD2, SON, SORBS3, SORT1, SPHK2, SQSTM1, SRA1, SRC, SRSF10, SRSF2, SRSF5, SSB, STAT1, STAT2, STAT5B, STK24, STON1, STRN3, SUFU, SUPT16H, TAB2, TAF6L, TAX1BP1, TBC1D10A, TBK1, TCP1, TDRD3, TF, TFAM, TGS1, THBS1, TIGAR, TIPRL, TMEM119, TNIK, TPX2, TRA2A, TRA2B, TRADD, TRIM21, TRIM25, TRIM28, TRIM56, TRIP12, TRIP13, TRMT10C, TSC2, TSPO, TTC39B, TTC5, TTLL12, TUT4, TXN, UACA, UBA2, UBA3, UBE2L6, UBR2, UBTF, UBXN2A, UFD1, URI1, USP22, USP4, USP5, USP7, USP9X, VDAC1, VDAC2, VLDLR, VPS25, VPS26B, VRK3, VTN, WDR35, WDR41, WDR5, WDR70, WFS1, WRNIP1, WWC2, WWP2, XIAP, YAP1, YBX1, YEATS4, YES1, YTHDC1, ZC3HAV1, ZCCHC3, ZFP36L2, ZMPSTE24, ZNF148]	
nucleic acid binding	GO_MolecularFunction-EBI-UniProt-GOA-ACAP-ARAP_13.05.2021_00h00	3.67E-55	9.468162	397	[AARS1, ABT1, ACO1, ACTB, ADARB1, ADK, ADNP, AGO2, AHCTF1, AKR1B1, ALB, ALDH18A1, ALDOA, ANXA7, APEX1, APLP2, APP, AQR, ARID1B, ASH2L, BANF1, BMS1, BZW1, C1QBP, CANX, CBX2, CBX8, CC2D1A, CCT3, CCT6A, CDC5L, CELF2, CERS6, CFAP20, CHD2, CHD4, CHD7, CHTOP, CIRBP, CLTC, CMTR1, CNGB1, CNOT1, COIL, CPSF2,	Upregulation

					CRYZ, CSTB, CSTF3, CTNNB1, CTU1, CUL4B, CUX1, CWC22, DARS1, DCN, DCPS, DDX1, DDX42, DDX47, DENR, DHFR, DHX34, DHX36, DIAPH1, DICER1, DIMT1, DNMT1, DNTTIP2, DSP, DUT, DYNC1LI1, EARS2, EDF1, EEF1G, EFL1, EHD3, EIF2AK2, EIF2B1, EIF2D, EIF2S1, EIF3A, EIF3B, EIF3E, EIF3F, EIF4G3, EIF6, ELP3, ELP5, EMG1, ENPP1, EPPK1, EPRS1, ERCC5, ERCC6L, EXD2, EZR, FAM120A, FANCI, FASN, FEN1, FMR1, FSCN1, GARS1, GATAD2A, GCFC2, GLRX3, GLYR1, GNL3L, GPKOW, GRWD1, GTF2F1, GTF2F2, GTF2I, GTF3C3, GTF3C4, H1-10, H1-5, HDAC2, HDAC4, HDGFL2, HEATR6, HELZ, HLA-A, HMCES, HMOX1, HNRNPA0, HNRNPA3, HNRNPC, HNRNPH1, HNRNPLL, HNRNPR, HNRNPUL1, HSD17B10, HSD17B4, HSP90AA1, HSP90B1, HSPE1, HTATSF1, IARS2, IFIT5, IGF2BP1, IGF2BP2, ILF3, IPO5, IREB2, KAT7, KCTD12, KDM1A, KDM3B, KPNB1, KRT18, L1TD1, LARP1, LARP4B, LARP6, LBR, LEMD2, LIG1, LIG3, LMNB1, LRPPRC, LRRFIP1, LRWD1, MACF1, MACROH2A1, MAP3K20, MATR3, MBD3, MCM2, MCM3, MCM4, MCM5, MCM6, MCM7, METTL14, MGA, MGMT, MLH1, MMAB, MOV10, MRM3, MRPL10, MRPS17, MRPS21, MRPS31, MRTO4, MSH2, MSH6, MSI2, MTA1, MTREX, MYH10, MYH9, MYO18A, N6AMT1, NCL, NCOA5, NEDD4, NELFCD, NFKB2, NIBAN2, NMD3, NOCT, NOL11, NOM1,	

					NOP53, NPM1, NQO1, NSUN5, NUCB2, NUDT16, NUDT16L1, NUP35, ORC4, ORC5, OXSR1, PAIP1, PARP1, PARP4, PATZ1, PCBP3, PDAP1, PEBP1, PKM, PKN2, PLEC, PML, POGZ, POLD1, POLD2, POLE2, POLR1C, POLR1D, POLR2A, POLR2B, POLR2H, POLR2I, POLR3A, POLR3C, POP1, PPIB, PPIE, PPIG, PPP5C, PRDX1, PRIM2, PSIP1, PSMD2, PSPC1, PTBP2, PTBP3, PTGES2, PUF60, PUS7, RABGEF1, RANGAP1, RARS2, RBBP4, RBM22, RBM26, RBM4, RBMS2, RCC1, RDX, RECQL, RFC2, RFC3, RFC5, RNF40, RNPS1, RPA1, RPA2, RPF2, RPL10A, RPL13, RPL13A, RPL18A, RPL22, RPL24, RPL27, RPL32, RPL36, RPL38, RPL5, RPS13, RPS15A, RPS16, RPS18, RPS21, RPS23, RPS4Y1, RPS5, RPS8, RPS9, RTN4, RYBP, S100A16, SAMHD1, SARNP, SARS1, SBDS, SCAF11, SEC61B, SET, SF1, SF3A3, SF3B6, SHFL, SHMT2, SHPRH, SIRT1, SLC16A3, SLC25A15, SLC25A5, SMAD4, SMARCA1, SMARCAD1, SMARCB1, SMARCC1, SMARCE1, SMC1A, SMC2, SMC4, SMCHD1, SMNDC1, SMYD3, SNRNP200, SNRPA, SNRPD3, SNRPE, SNU13, SNW1, SOD2, SON, SPAG7, SPATS2, SPATS2L, SRA1, SRPRA, SRSF10, SRSF2, SRSF5, SSB, SSRP1, STAT1, STAT2, STAT5B, STON1, SUPT16H, SYNJ1, TAF6L, TBCA, TBK1, TBPL1, TCP1, TDRD3, TFAM, THOC2, TRA2A, TRA2B, TRIM21, TRIM25, TRIM28, TRIM56, TRIP6, TRMT10C, TRMT2A, TSEN15, TSEN34,	

					TTC5, TUT4, TWF2, TXN, U2SURP, UBA2, UBFD1, UBTF, UHRF2, UTP14A, UTP20, UTP3, WRNIP1, XPOT, YAP1, YARS1, YBX1, YRDC, YTHDC1, ZC3HAV1, ZCCHC3, ZFAND6, ZFP36L2, ZMPSTE24, ZMYM3, ZNF148, ZNF207, ZNF512]	
cellular aromatic compound metabolic process	GO_BiologicalProcess-EBI-UniProt-GOA-ACAP-ARAP_13.05.2021_00h00	3.67E-55	10.122649	619	[AAR2, AARS1, AASS, ABAT, ABCB7, ABCC1, ABCE1, ABT1, ACACA, ACOT7, ACOT8, ACSF2, ACSL3, ACSL4, ACSS2, ACTR2, ACTR3, ADARB1, ADK, ADNP, ADSL, AGO2, AGRN, AK1, AK3, AK4, AK6, AKR1B1, ALB, ALDOA, ANXA3, APBB2, APEX1, APP, APRT, AQR, ARHGEF11, ARID1B, ARRB1, ATP6V0C, ATP6V1A, BAZ1B, BCAS2, BDH2, BLVRB, BMS1, BPNT2, BRD3, C1QBP, CAMK2D, CASK, CASP3, CBX1, CBX2, CBX3, CBX5, CBX8, CC2D1A, CCDC22, CCN2, CCNH, CCNK, CCNL1, CCT2, CCT3, CCT6A, CD200, CD81, CDC34, CDC5L, CDC73, CDK1, CDK16, CDK17, CDK2, CDK5RAP3, CECR2, CELF2, CENPV, CETN2, CHAF1B, CHD1L, CHD2, CHD4, CHD7, CHORDC1, CHP1, CHTOP, CIRBP, CLU, CMPK1, CMTR1, CNGB1, CNOT1, CNOT10, COIL, COLEC12, CPSF2, CRABP2, CROT, CSTF3, CTBP1, CTNNB1, CTNNBL1, CTPS1, CTU1, CUL4B, CUX1, CWC22, DAB2, DARS1, DCAKD, DCPS, DDX1, DDX42, DDX47, DGUOK, DHFR, DHX34, DHX36, DICER1, DIMT1, DLST, DNMT1, DPYSL2, DPYSL3, DPYSL4, DRG1, DUT, DVL2, EARS2, EDF1, EED, EEF1E1, EFEMP1, EFL1, EIF2AK2, EIF3E, EIF6, ELP3, ELP4, ELP5,	No change

					EMG1, ENO2, ENPP1, ENY2, EPHA2, EPHX1, EPRS1, ERBB2, ERCC5, EXD2, EXTL2, EZR, FAM3A, FANCI, FASN, FEN1, FGF2, FHL2, FHOD1, FMR1, FSCN1, FTO, FUT8, GAPDH, GARS1, GATAD2A, GATB, GCDH, GCFC2, GEMIN4, GFPT2, GIPC1, GLMP, GLO1, GLYR1, GMPS, GNE, GNL3L, GPI, GPKOW, GSTM2, GSTM3, GTF2E1, GTF2F1, GTF2F2, GTF2I, GTF3C3, GTF3C4, H1-10, H1-5, HARS1, HAT1, HDAC2, HDAC4, HDGFL2, HERC2, HEXB, HINT1, HK1, HK2, HMBS, HMCES, HMGCS1, HMOX1, HNRNPA0, HNRNPA3, HNRNPC, HNRNPH1, HNRNPLL, HNRNPR, HNRNPUL1, HPX, HSD17B10, HSD17B4, HSP90AA1, HSPA5, HTATSF1, IARS2, IDH2, IGF2BP1, IGF2BP2, IKBKB, IKBKG, ILF3, IMPACT, INTS1, INTS9, IREB2, IVNS1ABP, JAG1, KAT7, KAT8, KCNQ1, KDM1A, KDM3B, KDM4B, KPNB1, LANCL2, LARP1, LARP4B, LARP6, LDHA, LIG1, LIG3, LIMS1, LMCD1, LMO7, LRPPRC, LRRFIP1, LRWD1, LUM, MACROD1, MACROH2A1, MAGI1, MALT1, MAOA, MAP2K3, MAP3K4, MBD3, MCM2, MCM3, MCM4, MCM5, MCM6, MCM7, MDC1, ME1, MED17, MED23, MED30, METTL14, MFN1, MGA, MGAT1, MGMT, MLF2, MLH1, MMAB, MMUT, MORF4L1, MOV10, MPHOSPH8, MRM3, MRTO4, MSH2, MSH6, MTA1, MTAP, MTHFD2, MTR, MTREX, MTRR, MVD, MVK, MYD88, MYO18A, MYO6, N6AMT1, NCK2, NCL, NCOA5,	

					NCOA7, NDRG1, NDUFA13, NEDD4, NEDD8, NELFCD, NFATC2IP, NFKB2, NIBAN2, NMD3, NOCT, NOL11, NOP53, NPLOC4, NPM1, NPR2, NSMCE3, NSUN5, NT5C, NT5C3B, NUDT15, NUDT16, NUDT16L1, NUP205, NUP35, NUP62, ORC4, ORC5, OXSR1, PAICS, PAIP1, PANK4, PAPSS2, PARG, PARP1, PARP14, PARP4, PATZ1, PAWR, PBXIP1, PCBP3, PDE5A, PDK1, PDS5B, PELO, PFAS, PFKM, PGM3, PIK3CA, PIN1, PIR, PITHD1, PKLR, PKM, PLD3, PLPP3, PML, PMM2, PNP, POFUT1, POGZ, POLD1, POLD2, POLE2, POLR1C, POLR1D, POLR2A, POLR2B, POLR2H, POLR2I, POLR3A, POLR3C, POP1, PPIE, PPIG, PPP3CA, PPP5C, PPWD1, PRDX6, PRIM2, PRKAA1, PRKCI, PRMT7, PRPF19, PRPSAP1, PRPSAP2, PSAT1, PSIP1, PSMA3, PSMB10, PSMB2, PSMC2, PSMC3, PSMC6, PSMD1, PSMD12, PSMD14, PSMD2, PSMD6, PSMD7, PSMD8, PSME1, PSME2, PSME4, PSPC1, PTBP2, PTBP3, PTGES2, PTGIS, PTRH2, PUF60, PUS7, PXN, PYCARD, QARS1, QDPR, QTRT1, QTRT2, RAB23, RAD21, RAD9A, RAP2C, RARS2, RBM22, RBM26, RBM4, RBMS2, RECQL, RFC2, RFC3, RFC5, RIF1, RIOK3, RIPK2, RNF2, RNF40, RNGTT, RNPS1, ROR1, RPA1, RPA2, RPF2, RPL10A, RPL13, RPL13A, RPL18A, RPL22, RPL24, RPL27, RPL32, RPL36, RPL38, RPL5, RPLP1, RPLP2, RPRD1A, RPRD1B, RPRD2, RPS13, RPS15A, RPS16, RPS18,	

					RPS21, RPS23, RPS4Y1, RPS5, RPS8, RPS9, RRM1, RYBP, SAMHD1, SAP30BP, SARM1, SARNP, SARS1, SBDS, SCAF11, SCAI, SEC14L2, SENP3, SESN2, SET, SF1, SF3A3, SF3B6, SHMT2, SHPRH, SIRT1, SLC11A2, SLC1A1, SLC25A12, SLC25A13, SLC25A15, SLC25A19, SLC26A2, SLC30A9, SLC9A1, SMAD4, SMARCA1, SMARCAD1, SMARCB1, SMARCC1, SMARCD3, SMARCE1, SMC1A, SMC3, SMCHD1, SMNDC1, SMU1, SMYD3, SNF8, SNRNP200, SNRPA, SNRPD3, SNRPE, SNU13, SNW1, SNX5, SOAT1, SOD2, SON, SORBS3, SPHK2, SQSTM1, SRA1, SRC, SRSF10, SRSF2, SRSF5, SSB, SSRP1, STAT1, STAT2, STAT5B, STON1, STRN3, SUFU, SULT1E1, SUPT16H, TAB2, TAF6L, TAX1BP1, TBK1, TBPL1, TCP1, TDRD3, TF, TFAM, TGS1, THADA, THEM4, THOC2, TIGAR, TJP2, TPI1, TPMT, TPST2, TRA2A, TRA2B, TRADD, TRIM21, TRIM25, TRIM28, TRIM56, TRIP12, TRIP13, TRMT10C, TRMT2A, TRMT5, TSEN15, TSEN34, TSPO, TSR2, TTC5, TUT4, TXN, TYMP, U2SURP, UAP1, UBA2, UBA3, UBE2L6, UBR2, UBTF, UFD1, UGP2, UHRF2, UMPS, URI1, URM1, USP22, USP28, USP4, USP7, USP9X, UTP14A, UTP20, UTP3, VARS1, WDR12, WDR70, WFS1, WRNIP1, WWC2, WWP2, YAP1, YARS1, YBEY, YBX1, YEATS4, YES1, YTHDC1, ZC3HAV1, ZFP36L2, ZMPSTE24, ZNF148]	
negative regulation of metabolic process	GO_BiologicalProcess-EBI-	3.67E-55	10.372579	348	[AASS, ABCB7, ABCE1, ACADVL,	No change

	UniProt-GOA-ACAP-ARAP_13.05.2021_00h00				ACOT8, ADAM10, ADARB1, ADNP, AGO2, AKR1B1, ANKRD13A, ANXA7, APEX1, APLP2, APP, APPL2, ARPP19, ARRB1, ASB1, ATG7, ATP1A1, ATP2B1, BIN1, BOD1, C1QBP, CASP3, CBX1, CBX2, CBX3, CBX5, CBX8, CC2D1A, CCN2, CD109, CD200, CDC73, CDK1, CDK2, CDK5RAP3, CENPV, CHID1, CHORDC1, CHP1, CIRBP, CLTC, CLU, CNOT1, CNOT10, COLEC12, CORO1C, CRTAP, CSTB, CTBP1, CTNNB1, CTSA, CUX1, CYP51A1, DAB2, DAPK1, DCPS, DHCR24, DHFR, DHX34, DHX36, DICER1, DNMT1, DUSP3, DYNLL1, EED, EIF2AK2, EIF2S1, EIF3E, EIF4G3, EIF6, ENPP1, EPHA2, EPRS1, ERLIN2, EZR, FAM3A, FERMT1, FGF2, FHL2, FKBP1A, FMR1, FN1, FTO, G6PD, GAPDH, GARS1, GATAD2A, GBP1, GCFC2, GIPC1, GLA, GLMN, GNL3L, GOLT1B, GOT1, GPD1L, GPI, GSK3B, GSTP1, H1-10, H1-5, HAT1, HDAC2, HDAC4, HELZ, HK2, HMOX1, HNRNPA0, HNRNPC, HNRNPR, IGF2BP1, IGF2BP2, IKBKB, ILF3, IMPACT, IPO5, IQGAP3, IREB2, ITGAV, ITIH4, ITM2C, IVNS1ABP, KAT7, KAT8, KCNQ1, KDM1A, KDM4B, LANCL2, LARP1, LARP4B, LIG3, LIMS1, LLGL1, LMCD1, LNPEP, LRPPRC, LRRC32, LRRFIP1, LRRK1, MACROH2A1, MAD2L1, MAEA, MAGI1, MBD3, METTL14, MLH1, MMAB, MORF4L1, MOV10, MPHOSPH8, MRTO4, MSH2, MSH6, MTA1, MVP, MYADM, MYD88, NCK2, NCL, NCOA5, NCOA7, NDUFA13, NEDD4, NELFCD, NF1, NF2, NIBAN1, NIBAN2, NLE1, NNT, NOCT,	

					NOP53, NPLOC4, NPM1, NT5C3B, NUDT16, NUDT16L1, NUP205, NUP35, NUP62, NUTF2, NXN, OXSR1, P3H1, PAIP1, PARP1, PARP14, PATZ1, PAWR, PBXIP1, PC, PEBP1, PELO, PGAM5, PGP, PIK3CA, PIN1, PIP4K2B, PKN2, PLPP3, PML, POLR2A, POLR2B, POLR2H, POLR2I, PPM1B, PPP1R14A, PPP2R5A, PPP3CA, PPP5C, PRKAA1, PSMA3, PSMB10, PSMB2, PSMC2, PSMC3, PSMC6, PSMD1, PSMD12, PSMD14, PSMD2, PSMD6, PSMD7, PSMD8, PSME1, PSME2, PSME4, PSPC1, PTBP2, PTBP3, PTGIS, PTPA, PTRH2, PUS7, PYCARD, QARS1, QSOX1, RBBP4, RBM4, RHEB, RIF1, RNF2, RNF40, RNPS1, RPL10A, RPL13, RPL13A, RPL18A, RPL22, RPL24, RPL27, RPL32, RPL36, RPL38, RPL5, RPLP1, RPLP2, RPS13, RPS15A, RPS16, RPS18, RPS21, RPS23, RPS4Y1, RPS5, RPS8, RPS9, RTN4, RYBP, SARS1, SCAI, SCFD1, SERPINB1, SERPINB2, SERPINB8, SERPINB9, SERPINE2, SESN2, SET, SF1, SHFL, SHMT2, SIRT1, SLC25A12, SLIT2, SMAD4, SMARCB1, SMARCC1, SMARCE1, SMCHD1, SMPD1, SMYD3, SNW1, SOD1, SORBS3, SPHK2, SQSTM1, SRC, SRSF10, SRSF2, SSB, STAT1, STON1, STRN3, SUFU, TAB2, TAX1BP1, TBK1, THBS1, TIGAR, TIPRL, TRIM21, TRIM25, TRIM28, TRIP12, TSC2, TSPO, TUT4, TXN, UBA2, UBA3, UBE2L6, UBR2, UFD1, URI1, USP4, USP7, USP9X, VDAC1, VDAC2, VPS25, VTN, WDR35,	

					WDR5, WFS1, WWC2, WWP2, XIAP, YAP1, YBX1, ZC3HAV1, ZFP36L2, ZMPSTE24, ZNF148]	
positive regulation of metabolic process	GO_BiologicalProcess-EBI-UniProt-GOA-ACAP-ARAP_13.05.2021_00h00	3.67E-55	9.233098	366	[ABAT, ABCB7, ABI1, ABT1, ACACA, ACSL3, ACTB, ACTG1, ACTR2, ACTR3, ADNP, AFDN, AGO2, AGPAT2, AGRN, AKAP12, AKR1B1, ANK3, ANXA3, APAF1, APEX1, APLP2, APP, APPL2, ARAF, ARFGEF1, ARHGEF11, ARID1B, ARPP19, ARRB1, ASPSCR1, ATG4B, ATG7, B2M, BAZ1B, C1QBP, CAB39L, CASK, CASP3, CBR1, CBX8, CCDC22, CCN2, CCNK, CCT2, CCT3, CCT6A, CD200, CD276, CD81, CDC5L, CDC73, CDK1, CDK5RAP3, CEMIP, CHAMP1, CHD7, CHORDC1, CHP1, CHTOP, CIRBP, CLU, CNOT1, CNOT10, CSPG4, CTBP1, CTNNB1, CTSC, CYCS, CYRIB, DAB2, DAGLB, DAPK1, DCN, DCPS, DCXR, DDX1, DERL1, DHX34, DHX36, DIMT1, DIPK2A, DNMT1, DVL2, DYNLL1, ECE1, EDF1, EIF2AK2, EIF3E, EIF6, ENY2, EPHA2, EPHA3, EPHB4, EPM2AIP1, ERBB2, ETFDH, EZR, FASN, FBXW8, FERMT1, FERMT2, FGF2, FHOD1, FKBP1A, FMR1, FN1, FTO, GAPDH, GARS1, GIPC1, GLMN, GLMP, GLYR1, GPI, GPSM1, GSDMD, GSK3B, GSTP1, GTF2F1, GTF2I, H1-10, H1-5, HADH, HADHA, HDAC2, HDAC4, HEXB, HGS, HIP1, HK1, HLA-A, HMCES, HMOX1, HNRNPA0, HNRNPC, HNRNPLL, HNRNPR, HPX, HSD17B4, HSP90AA1, HSPA5, HSPE1, IGF2BP1, IKBKB, IKBKG, ILF3, IMPACT, IQGAP3, IST1, ITGA3, JAG1, KAT7, KAT8, KDM1A, LARP1, LARP4B, LARP6, LIMS1, LLGL1, LMO7,	No change

					LRRC32, LRRFIP1, LRRK1, LUM, MACROH2A1, MALT1, MAP2K3, MAP3K20, MAP3K4, MAP4K4, MED17, MED23, MED30, METTL14, MGMT, MLH1, MMAB, MOV10, MPHOSPH8, MSH2, MSN, MTA1, MTCH1, MYD88, MYH9, MYO1E, MYO6, NCK2, NCL, NCOA7, NDUFA13, NEDD4, NFATC2IP, NFKB2, NIBAN1, NIBAN2, NMD3, NNT, NOCT, NOL11, NOP53, NPM1, NSMCE3, NSUN5, NT5C3B, NUB1, NUP62, OSBPL8, OXSR1, PACSIN3, PAFAH1B2, PAIP1, PANX1, PARP1, PARP14, PATZ1, PAWR, PFKM, PIK3C2A, PIK3CA, PIN1, PIP4K2B, PITHD1, PKM, PKN2, PLD1, PLXNB2, PML, PNP, POGZ, POLR1C, POLR1D, POLR2A, POLR2H, POLR3A, POLR3C, PPP2R5A, PPP3CA, PRDX6, PRKAA1, PRKAB2, PRKCSH, PRPF19, PSMC2, PSMC3, PSMC6, PSMD14, PSME1, PSME2, PSME4, PTGES2, PTPA, PTRH2, PYCARD, RAB1A, RAP2B, RAP2C, RBM22, RCN3, RDH10, RDX, RFC2, RFC3, RFC5, RIF1, RIOK3, RIPK2, RNF40, ROR1, RPL5, RPLP1, RPRD1B, RPS9, RYBP, SAE1, SCRIB, SEC14L2, SERPINB9, SESN2, SH3BP4, SIRT1, SLC1A1, SLC25A12, SLC25A4, SLC25A5, SLC30A9, SLC9A1, SMAD4, SMARCA1, SMARCB1, SMARCC1, SMARCD3, SMARCE1, SMCHD1, SMPD1, SMYD3, SNF8, SNW1, SNX1, SNX5, SOAT1, SOD1, SOD2, SPHK2, SQSTM1, SRA1, SRC, SRSF5, STAT1, STAT5B, STK24, STON1, SUPT16H, TAB2, TAF6L, TBC1D10A, TBK1,	

					TCP1, TDRD3, TF, TFAM, THBS1, TIGAR, TMEM119, TNIK, TPX2, TRA2A, TRA2B, TRADD, TRIM21, TRIM25, TRIM28, TRIM56, TRMT10C, TSC2, TSPO, TTC5, TUT4, TXN, UACA, UBA2, UBTF, USP22, USP5, USP7, USP9X, VDAC1, VDAC2, VLDLR, VRK3, VTN, WDR35, WDR5, WFS1, WWP2, XIAP, YAP1, YBX1, YEATS4, YES1, ZC3HAV1, ZCCHC3, ZFP36L2, ZMPSTE24, ZNF148]	
transferase activity, transferring phosphorus-containing groups	GO_MolecularFunction-EBI-UniProt-GOA-ACAP-ARAP_13.05.2021_00h00	3.67E-55	10.502283	184	[AASDHPPT, ABI1, ACTB, ADARB1, ADK, ADNP, AK1, AK3, AK4, AK6, ALDH18A1, APP, ARAF, ARRB1, BAZ1B, BCR, CAB39L, CAMK2D, CASK, CASP3, CCNH, CCNK, CCNL1, CCT2, CD81, CDC42BPA, CDIPT, CDK1, CDK16, CDK17, CDK2, CDK5RAP3, CEMIP, CHORDC1, CHP1, CKB, CLU, CMPK1, COLEC12, CORO1C, CSPG4, CTNNB1, CTU1, DAPK1, DCAKD, DGUOK, DIPK2A, DUSP3, DVL2, EFEMP1, EIF2AK2, ELP3, ELP4, EPHA2, EPHA3, EPHB4, ERBB2, ETFDH, FERMT2, FGF2, FN3K, GDPGP1, GNE, GPHN, GPI, GSK3B, GSTP1, GYS1, HGS, HK1, HK2, HSP90AA1, IKBKB, IKBKG, ILKAP, IPO5, IQGAP3, ITPK1, LLGL1, LRRK1, LTBP1, MACROH2A1, MALT1, MAP2K3, MAP3K20, MAP3K4, MAP4K4, MCM2, MMAB, MVK, MVP, NCK2, NEK1, NF1, NF2, NPM1, NPR2, NUP62, OSBPL8, OXSR1, PANK4, PAPSS2, PARP4, PCYT2, PDK1, PFKM, PGM2L1, PI4K2B, PIGO, PIK3C2A, PIK3CA, PIP4K2B, PKLR, PKM, PKN2, POLD1, POLE2, POLR1C, POLR1D, POLR2A, POLR2B, POLR2H, POLR2I,	Upregulation

					POLR3A, POLR3C, POMK, PPIP5K2, PPP2R5A, PPP3CA, PRKAA1, PRKAB2, PRKCI, PRKG1, PRPSAP1, PRPSAP2, PTK7, PXK, PYCARD, QARS1, RAP2B, RAP2C, RFC2, RFC3, RFC5, RIOK3, RIPK2, RNGTT, ROR1, RPLP1, SESN2, SIRT1, SLC1A1, SMPD1, SMYD3, SNF8, SOD1, SPHK2, SQSTM1, SRC, STK24, TAB2, TBK1, TCP1, TF, TGFBR3, THBS1, TIGAR, TJP2, TNIK, TPX2, TRIM28, TRIO, TSC2, TUT4, TXN, UAP1, UGP2, VLDLR, VPS25, VRK1, VRK3, XYLB, YES1, YRDC]	
cellular nitrogen compound metabolic process	GO_BiologicalProcess-EBI-UniProt-GOA-ACAP-ARAP_13.05.2021_00h00	3.67E-55	10.234094	682	[AAR2, AARS1, AASDHPPT, AASS, ABAT, ABCB7, ABCC1, ABCE1, ABT1, ACACA, ACADM, ACO1, ACOT7, ACOT8, ACSF2, ACSL3, ACSL4, ACSS2, ACTR2, ACTR3, ADAM10, ADARB1, ADK, ADNP, ADSL, AGO2, AGRN, AK1, AK3, AK4, AK6, AKR1B1, ALB, ALDOA, ANXA3, APBB2, APEX1, APP, APRT, AQR, ARHGEF11, ARID1B, ARRB1, ASAH1, ASNS, ATP6V0C, ATP6V1A, B4GALT5, BAZ1B, BCAS2, BDH2, BIN1, BLVRB, BMS1, BPNT2, BRD3, C1QBP, CAMK2D, CASK, CASP3, CBX1, CBX2, CBX3, CBX5, CBX8, CC2D1A, CCDC22, CCN2, CCNH, CCNK, CCNL1, CCT2, CCT3, CCT6A, CD200, CD81, CDC34, CDC5L, CDC73, CDK1, CDK16, CDK17, CDK2, CDK5RAP3, CECR2, CELF2, CENPV, CERS6, CETN2, CHAF1B, CHD1L, CHD2, CHD4, CHD7, CHORDC1, CHP1, CHTOP, CIRBP, CLIC1, CLU, CMPK1, CMTR1, CNGB1, CNOT1, CNOT10, COIL, COLEC12, CPD, CPSF2, CRABP2, CROT, CSTF3, CTBP1, CTNNB1, CTNNBL1,	No change

					CTPS1, CTU1, CUL4B, CUX1, CWC22, CYB5R3, DAB2, DAPK1, DARS1, DCAKD, DCPS, DDX1, DDX42, DDX47, DENR, DGUOK, DHFR, DHPS, DHX34, DHX36, DICER1, DIMT1, DLST, DNMT1, DPYSL2, DPYSL3, DPYSL4, DRG1, DUT, DVL2, EARS2, ECE1, EDF1, EED, EEF1E1, EEF1G, EFEMP1, EFL1, EIF2AK2, EIF2B1, EIF2D, EIF2S1, EIF3A, EIF3B, EIF3E, EIF3F, EIF4G3, EIF6, ELP3, ELP4, ELP5, EMG1, ENO2, ENPP1, ENY2, EPHA2, EPRS1, ERAP1, ERBB2, ERCC5, ETHE1, EXD2, EXTL2, EZR, FAM3A, FANCI, FASN, FEN1, FGF2, FHL2, FHOD1, FMR1, FSCN1, FTO, FUT8, G6PD, GAPDH, GARS1, GATAD2A, GATB, GBA2, GCDH, GCFC2, GDAP1, GEMIN4, GFPT2, GGCT, GLA, GLE1, GLMP, GLO1, GLYR1, GMPS, GNE, GNL3L, GPI, GPKOW, GSTM2, GSTM3, GSTP1, GTF2E1, GTF2F1, GTF2F2, GTF2I, GTF3C3, GTF3C4, H1-10, H1-5, HARS1, HAT1, HDAC2, HDAC4, HDGFL2, HERC2, HEXB, HINT1, HK1, HK2, HMBS, HMCES, HMGCS1, HMOX1, HNRNPA0, HNRNPA3, HNRNPC, HNRNPH1, HNRNPLL, HNRNPR, HNRNPUL1, HSD17B10, HSD17B4, HSP90AA1, HSPA5, HTATSF1, IARS2, IDH2, IGF2BP1, IGF2BP2, IKBKB, IKBKG, ILF3, IMPACT, INTS1, INTS9, IREB2, IVNS1ABP, JAG1, KAT7, KAT8, KCNQ1, KDM1A, KDM3B, KDM4B, KPNB1, LANCL2, LARP1, LARP4B, LARP6, LDHA, LIG1, LIG3, LIMS1, LMCD1, LMO7, LNPEP, LRPPRC, LRRFIP1, LRWD1, LUM, MACROD1, MACROH2A1,	

					MAGI1, MALT1, MAOA, MAP2K3, MAP3K4, MBD3, MCM2, MCM3, MCM4, MCM5, MCM6, MCM7, MDC1, ME1, MED17, MED23, MED30, METTL14, MFN1, MGA, MGAT1, MGMT, MLF2, MLH1, MMAB, MME, MORF4L1, MOV10, MPHOSPH8, MPST, MRM3, MRPL10, MRPS17, MRPS21, MRPS31, MRRF, MRTO4, MSH2, MSH6, MTA1, MTAP, MTHFD2, MTREX, MTRR, MVD, MVK, MYD88, MYO18A, MYO6, N6AMT1, NCK2, NCL, NCOA5, NCOA7, NDRG1, NDUFA13, NEDD4, NEDD8, NELFCD, NFATC2IP, NFKB2, NIBAN1, NIBAN2, NMD3, NOCT, NOL11, NOP53, NPLOC4, NPM1, NPR2, NQO1, NSMCE3, NSUN5, NT5C, NT5C3B, NUDT15, NUDT16, NUDT16L1, NUP205, NUP35, NUP62, OGFOD1, OPLAH, ORC4, ORC5, OXA1L, OXSR1, PAICS, PAIP1, PANK4, PAPSS2, PARG, PARL, PARP1, PARP14, PARP4, PATZ1, PAWR, PBXIP1, PC, PCBP3, PDE5A, PDK1, PDS5B, PDZD11, PELO, PFAS, PFKM, PGM3, PIK3CA, PIN1, PIR, PITHD1, PKLR, PKM, PLA2G15, PLD1, PLD3, PLPP3, PLXNB2, PML, PMM2, PNP, POFUT1, POGZ, POLD1, POLD2, POLE2, POLR1C, POLR1D, POLR2A, POLR2B, POLR2H, POLR2I, POLR3A, POLR3C, POP1, PPIE, PPIG, PPP1CA, PPP3CA, PPP5C, PPWD1, PRDX6, PRIM2, PRKAA1, PRKCI, PRKCSH, PRMT7, PRPF19, PRPSAP1, PRPSAP2, PSAT1, PSIP1, PSMA3, PSMB10, PSMB2, PSMC2, PSMC3, PSMC6,	

					PSMD1, PSMD12, PSMD14, PSMD2, PSMD6, PSMD7, PSMD8, PSME1, PSME2, PSME4, PSPC1, PTBP2, PTBP3, PTGES2, PTGIS, PTRH2, PUF60, PUS7, PXN, PYCARD, QARS1, QDPR, QTRT1, QTRT2, RAB23, RAD21, RAD9A, RAP2C, RARS2, RBM22, RBM26, RBM4, RBMS2, RECQL, RFC2, RFC3, RFC5, RIF1, RIOK3, RIPK2, RNF2, RNF40, RNGTT, RNPS1, ROR1, RPA1, RPA2, RPF2, RPL10A, RPL13, RPL13A, RPL18A, RPL22, RPL24, RPL27, RPL32, RPL36, RPL38, RPL5, RPLP1, RPLP2, RPRD1A, RPRD1B, RPRD2, RPS13, RPS15A, RPS16, RPS18, RPS21, RPS23, RPS4Y1, RPS5, RPS8, RPS9, RRM1, RTN4, RYBP, SAMHD1, SAP30BP, SARM1, SARNP, SARS1, SBDS, SCAF11, SCAI, SEC11C, SEC14L2, SELENOT, SENP3, SESN2, SET, SF1, SF3A3, SF3B6, SHFL, SHMT2, SHPRH, SIRT1, SLC11A2, SLC1A1, SLC25A12, SLC25A13, SLC25A15, SLC25A19, SLC26A2, SLC30A9, SLC44A1, SLC9A1, SMAD4, SMARCA1, SMARCAD1, SMARCB1, SMARCC1, SMARCD3, SMARCE1, SMC1A, SMC3, SMCHD1, SMNDC1, SMPD1, SMU1, SMYD3, SNF8, SNRNP200, SNRPA, SNRPD3, SNRPE, SNU13, SNW1, SNX5, SOAT1, SOD1, SOD2, SON, SORBS3, SPHK2, SPTLC1, SPTLC2, SQSTM1, SRA1, SRC, SRSF10, SRSF2, SRSF5, SSB, SSRP1, STAT1, STAT2, STAT5B, STON1, STRN3, SUFU, SULT1E1, SUPT16H, TAB2, TAF6L, TAX1BP1, TBCE,	

					TBK1, TBPL1, TCP1, TDRD3, TF, TFAM, TGS1, THADA, THBS1, THEM4, THOC2, TIGAR, TJP2, TPI1, TPMT, TPP1, TPST2, TRA2A, TRA2B, TRADD, TRIM21, TRIM25, TRIM28, TRIM56, TRIP12, TRIP13, TRMT10C, TRMT2A, TRMT5, TSEN15, TSEN34, TSPO, TSR2, TTC5, TUT4, TXN, TYMP, U2SURP, UAP1, UBA2, UBA3, UBE2L6, UBR2, UBTF, UFD1, UGP2, UHRF2, UMPS, URI1, URM1, USP22, USP28, USP4, USP7, USP9X, UTP14A, UTP20, UTP3, VARS1, VDAC2, WDR12, WDR35, WDR70, WFS1, WRNIP1, WWC2, WWP2, YAP1, YARS1, YBEY, YBX1, YEATS4, YES1, YTHDC1, ZC3HAV1, ZFP36L2, ZMPSTE24, ZNF148]	
macromolecule metabolic process	GO_BiologicalProcess-EBI-UniProt-GOA-ACAP-ARAP_13.05.2021_00h00	3.67E-55	9.006338	881	[AAR2, AARS1, AASDHPPT, AASS, ABCE1, ABHD12, ABHD16A, ABI1, ABT1, ACACA, ACADM, ACO1, ACOT8, ACTB, ACTG1, ACTR2, ACTR3, ADAM10, ADAMTS9, ADARB1, ADNP, AFDN, AGO2, AGPAT2, AGRN, AKAP12, AKR1B1, ALB, ALG11, ANK3, ANKRD13A, ANKZF1, ANXA3, ANXA7, APAF1, APBB2, APEX1, APLP2, APP, APPL2, AQR, ARAF, ARFGEF1, ARHGEF11, ARID1B, ARMT1, ARPP19, ARRB1, ASB1, ASH2L, ASPSCR1, ATE1, ATG4B, ATG7, ATP2B1, AUP1, B2M, B3GAT3, B4GALT1, B4GALT5, BAZ1B, BCAS2, BCR, BIN1, BMP1, BMS1, BOD1, BPNT2, BRD3, BST1, C1GALT1, C1QBP, CAB39L, CAMK2D, CAPN5, CASK, CASP3, CASP6, CBX1, CBX2, CBX3, CBX5, CBX8, CC2D1A, CCDC22, CCN2, CCNH, CCNK, CCNL1, CCT2, CCT3, CCT6A, CD109, CD200,	No change

					CD276, CD81, CDC34, CDC42BPA, CDC5L, CDC73, CDK1, CDK16, CDK17, CDK2, CDK5RAP3, CECR2, CELF2, CEMIP, CENPV, CETN2, CFAP20, CHAF1B, CHAMP1, CHD1L, CHD2, CHD4, CHD7, CHID1, CHORDC1, CHP1, CHPF, CHSY3, CHTOP, CIRBP, CLTC, CLU, CMTR1, CNGB1, CNOT1, CNOT10, COG7, COIL, COL11A1, COL5A1, COLEC12, CORO1C, CPD, CPSF2, CRABP2, CRBN, CRTAP, CSPG4, CSTB, CSTF3, CTBP1, CTNNB1, CTNNBL1, CTSA, CTSC, CTU1, CUL1, CUL4B, CUX1, CWC22, CYCS, CYP51A1, CYRIB, DAB2, DAPK1, DARS1, DCN, DCPS, DDI2, DDX1, DDX42, DDX47, DENR, DERL1, DGUOK, DHCR24, DHFR, DHPS, DHX34, DHX36, DICER1, DIMT1, DIPK2A, DLST, DNM1L, DNMT1, DPP3, DPP9, DRG1, DSP, DUSP23, DUSP3, DUT, DVL2, DYNLL1, EARS2, ECE1, EDF1, EED, EEF1E1, EEF1G, EFEMP1, EFL1, EHD3, EIF2AK2, EIF2B1, EIF2D, EIF2S1, EIF3A, EIF3B, EIF3E, EIF3F, EIF4G3, EIF6, ELP3, ELP4, ELP5, EMG1, ENPP1, ENY2, EOGT, EPHA2, EPHA3, EPHB4, EPM2AIP1, EPRS1, ERAP1, ERBB2, ERCC5, ERLIN2, ERP44, ETFDH, EXD2, EXTL2, EZR, FAM3A, FANCI, FASN, FBXL12, FBXW8, FDXR, FEN1, FERMT1, FERMT2, FGF2, FHL2, FHOD1, FKBP1A, FKBP5, FKBP9, FMR1, FN1, FN3K, FSCN1, FTO, FUT11, FUT8, G6PD, GALNT1, GALNT2, GALNT7, GAN, GAPDH, GARS1, GATAD2A, GATB, GBE1, GBP1, GCFC2, GEMIN4, GFPT2, GGA2, GIPC1, GLE1,	

					GLMN, GLMP, GLO1, GLRX3, GLUL, GLYR1, GNL3L, GOLGA7, GPC1, GPC6, GPD1L, GPHN, GPI, GPKOW, GRWD1, GSDMD, GSK3B, GSTP1, GTF2E1, GTF2F1, GTF2F2, GTF2I, GTF3C3, GTF3C4, GXYLT1, GXYLT2, GYS1, H1-10, H1-5, HARS1, HAT1, HDAC2, HDAC4, HDGFL2, HECTD4, HELZ, HERC2, HERC4, HEXB, HGS, HINT1, HIP1, HK1, HLA-A, HLA-B, HMBS, HMCES, HMOX1, HNRNPA0, HNRNPA3, HNRNPC, HNRNPH1, HNRNPLL, HNRNPR, HNRNPUL1, HPX, HSD17B10, HSD17B4, HSP90AA1, HSP90B1, HSPA5, HSPE1, HSPG2, HTATSF1, IARS2, IGF2BP1, IGF2BP2, IGFBP7, IKBKB, IKBKG, ILF3, ILKAP, IMPACT, INTS1, INTS9, IPO5, IQGAP3, IREB2, IST1, ITGA3, ITGAV, ITGB1, ITIH4, ITM2C, IVNS1ABP, JAG1, KAT7, KAT8, KCNQ1, KDM1A, KDM3B, KDM4B, KPNB1, LACTB, LAMC1, LANCL2, LAP3, LARP1, LARP4B, LARP6, LIG1, LIG3, LIMS1, LLGL1, LMCD1, LMO7, LNPEP, LRPPRC, LRRC32, LRRFIP1, LRRK1, LRWD1, LTBP1, LUM, LYPLA2, MACROD1, MACROH2A1, MAD2L1, MAEA, MAGI1, MALT1, MAP2K3, MAP3K20, MAP3K4, MAP4K4, MARCHF5, MATR3, MBD3, MCM2, MCM3, MCM4, MCM5, MCM6, MCM7, MCMBP, MDC1, MED17, MED23, MED30, METTL14, MGA, MGAT1, MGMT, MLF2, MLH1, MMAB, MME, MOCS2, MOGS, MORF4L1, MOV10, MPHOSPH8, MRM3, MRPL10, MRPS17, MRPS21, MRPS31,	

					MRRF, MRTO4, MSH2, MSH6, MSN, MTA1, MTCH1, MTREX, MTRR, MVD, MVP, MYADM, MYD88, MYH9, MYO18A, MYO1E, MYO6, N6AMT1, NCK2, NCL, NCLN, NCOA5, NCOA7, NDRG1, NDUFA13, NDUFAF7, NEDD4, NEDD8, NEK1, NELFCD, NF1, NF2, NFATC2IP, NFKB2, NIBAN1, NIBAN2, NLE1, NMD3, NMT1, NNT, NOCT, NOL11, NOP53, NPLOC4, NPM1, NPR2, NSMCE3, NSUN5, NT5C3B, NUB1, NUDT15, NUDT16, NUDT16L1, NUP205, NUP35, NUP62, NUTF2, NXN, OBSL1, OGFOD1, ORC4, ORC5, OSBPL8, OTUD6B, OXA1L, OXSR1, P3H1, P3H3, PACSIN3, PAIP1, PANX1, PARG, PARL, PARP1, PARP14, PARP4, PATZ1, PAWR, PBXIP1, PC, PCBP3, PDCD6IP, PDK1, PDS5B, PDZRN3, PEBP1, PELO, PFKM, PGAM5, PGM2L1, PGM3, PGP, PHGDH, PHKB, PHPT1, PIGO, PIGT, PIGU, PIK3CA, PIN1, PIR, PITHD1, PITRM1, PKM, PKN2, PLD1, PLD3, PLOD2, PLPP3, PLXNB2, PML, PMM2, PNP, POFUT1, POGLUT3, POGZ, POLD1, POLD2, POLE2, POLR1C, POLR1D, POLR2A, POLR2B, POLR2H, POLR2I, POLR3A, POLR3C, POMGNT1, POMGNT2, POMK, POP1, PPIB, PPIE, PPIG, PPM1B, PPP1CA, PPP1R14A, PPP1R7, PPP2R5A, PPP3CA, PPP5C, PPWD1, PRDX6, PRIM2, PRKAA1, PRKAB2, PRKCI, PRKCSH, PRKG1, PRMT7, PRPF19, PSIP1, PSMA3, PSMB10, PSMB2, PSMC2, PSMC3, PSMC6, PSMD1, PSMD12, PSMD14, PSMD2, PSMD6, PSMD7, PSMD8, PSME1, PSME2,	

					PSME4, PSPC1, PTBP2, PTBP3, PTGES2, PTGIS, PTK7, PTPA, PTPMT1, PTPN12, PTPRF, PTPRG, PTRH2, PUF60, PUS7, PXK, PXN, PYCARD, PYGB, QARS1, QSOX1, QTRT1, QTRT2, RAB11B, RAB1A, RAB3D, RAB5A, RAB8A, RAD21, RAD9A, RANGAP1, RAP2B, RAP2C, RARS2, RBBP4, RBM22, RBM26, RBM4, RBMS2, RCN1, RCN3, RDX, RECQL, RFC2, RFC3, RFC5, RIF1, RIOK3, RIPK2, RNF114, RNF2, RNF213, RNF214, RNF40, RNGTT, RNPS1, ROR1, RPA1, RPA2, RPF2, RPL10A, RPL13, RPL13A, RPL18A, RPL22, RPL24, RPL27, RPL32, RPL36, RPL38, RPL5, RPLP1, RPLP2, RPRD1A, RPRD1B, RPRD2, RPS13, RPS15A, RPS16, RPS18, RPS21, RPS23, RPS4Y1, RPS5, RPS8, RPS9, RRM1, RSPRY1, RTN4, RYBP, SAE1, SAMHD1, SAP30BP, SARNP, SARS1, SBDS, SCAF11, SCAI, SCARB2, SCPEP1, SCRIB, SCRN1, SEC11C, SEC14L2, SEC61B, SELENOT, SENP3, SERPINB1, SERPINB2, SERPINB8, SERPINB9, SERPINE2, SESN2, SET, SF1, SF3A3, SF3B6, SGSH, SHFL, SHMT2, SHPRH, SIRT1, SLC1A1, SLC25A15, SLC30A9, SLC9A1, SLIT2, SMAD4, SMARCA1, SMARCAD1, SMARCB1, SMARCC1, SMARCD3, SMARCE1, SMC1A, SMC3, SMCHD1, SMNDC1, SMPD1, SMU1, SMYD3, SNF8, SNRNP200, SNRPA, SNRPD3, SNRPE, SNU13, SNW1, SNX1, SNX5, SOAT1, SOD1, SOD2, SON, SORBS3,	

					SORT1, SPHK2, SQSTM1, SRA1, SRC, SRD5A3, SRSF10, SRSF2, SRSF5, SSB, SSRP1, STAT1, STAT2, STAT5B, STK24, STON1, STRN3, SUFU, SUPT16H, TAB2, TAF6L, TAX1BP1, TBC1D10A, TBCE, TBK1, TBPL1, TCP1, TDRD3, TF, TFAM, TGFBI, TGFBR3, TGM2, TGS1, THADA, THBS1, THOC2, TIGAR, TIPRL, TMEM119, TMX3, TNIK, TPP1, TPP2, TPST2, TPX2, TRA2A, TRA2B, TRADD, TRIM21, TRIM25, TRIM28, TRIM56, TRIM59, TRIO, TRIP12, TRIP13, TRMT10C, TRMT2A, TRMT5, TSC2, TSEN15, TSEN34, TSPO, TSR2, TTC5, TTLL12, TUT4, TXN, U2SURP, UACA, UBA2, UBA3, UBAC2, UBE2L6, UBE2Q1, UBR2, UBR4, UBTF, UBXN2A, UFD1, UGP2, UHRF2, UQCRC2, URI1, URM1, USP11, USP22, USP28, USP4, USP48, USP5, USP7, USP9X, USP9Y, UST, UTP14A, UTP20, UTP3, VARS1, VDAC2, VLDLR, VPS25, VPS36, VRK1, VRK3, VTN, WDR12, WDR35, WDR5, WDR70, WFS1, WIPI2, WRNIP1, WWC2, WWP2, XIAP, XPNPEP3, XYLT2, YAP1, YARS1, YBEY, YBX1, YEATS4, YES1, YOD1, YTHDC1, ZC3HAV1, ZCCHC3, ZDHHC13, ZDHHC21, ZFP36L2, ZMPSTE24, ZNF148]	
cellular biosynthetic process	GO_BiologicalProcess-EBI-UniProt-GOA-ACAP-ARAP_13.05.2021_00h00	3.67E-55	10.153166	643	[AARS1, AASDHPPT, AASS, ABAT, ABCB7, ABCE1, ABT1, ACACA, ACADM, ACADVL, ACO1, ACOT7, ACOT8, ACOX1, ACSL3, ACSL4, ACSS2, ACTR2, ACTR3, ADK, ADNP, ADSL, AGO2, AGPAT2, AGPS, AGRN, AK1, AK3, AK4, AK6, AKR1B1, ALB, ALDH18A1, ALDH3A2, ALDOA, ALG11, ANXA3,	No change

					APBB2, APEX1, APIP, APP, APRT, ARFGEF1, ARHGEF11, ARID1B, ARPP19, ARRB1, ASAH1, ASNS, ATG7, ATP1A1, ATP6V0C, ATP6V1A, B3GAT3, B4GALT1, B4GALT5, BAZ1B, BCAT1, BDH2, BPNT2, BRD3, C1GALT1, C1QBP, CAMK2D, CASK, CBR1, CBX1, CBX2, CBX3, CBX5, CBX8, CC2D1A, CCDC22, CCN2, CCNH, CCNK, CCNL1, CCT2, CCT3, CCT6A, CD200, CD81, CDC34, CDC5L, CDC73, CDIPT, CDK1, CDK16, CDK17, CDK2, CDK5RAP3, CENPV, CERS6, CHAF1B, CHD2, CHD4, CHD7, CHORDC1, CHP1, CHPF, CHSY3, CIRBP, CLU, CMPK1, CNGB1, CNOT1, CNOT10, COG7, COLEC12, COQ5, CPSF2, CRABP2, CSPG4, CSTF3, CTBP1, CTNNB1, CTPS1, CUX1, CYB5R3, DAB2, DAGLB, DAPK1, DARS1, DCAKD, DCN, DCPS, DDX1, DENR, DGUOK, DHFR, DHPS, DHRS7B, DHX34, DHX36, DICER1, DNMT1, DRG1, DUT, DVL2, EARS2, EDF1, EED, EEF1E1, EEF1G, EFEMP1, EFL1, EIF2AK2, EIF2B1, EIF2D, EIF2S1, EIF3A, EIF3B, EIF3E, EIF3F, EIF4G3, EIF6, ELP3, ELP4, ENPP1, ENY2, EOGT, EPM2AIP1, EPRS1, ERBB2, ERLIN2, EXD2, EXTL2, EZR, FAM3A, FASN, FDXR, FEN1, FGF2, FHL2, FHOD1, FMR1, FTO, FUT11, FUT8, GALNT1, GALNT2, GALNT7, GAMT, GAPDH, GARS1, GATAD2A, GATB, GBE1, GCDH, GCFC2, GFPT2, GGCT, GIPC1, GLA, GLE1, GLMP, GLO1, GLUD1, GLUL, GLYR1, GMPS, GNL3L, GOLGA7, GOLT1B, GOT1, GPD1L, GPHN, GPI, GRWD1, GSK3B, GSTM2, GSTM3,	

					GSTP1, GTF2E1, GTF2F1, GTF2F2, GTF2I, GTF3C3, GTF3C4, GXYLT1, GXYLT2, GYS1, H1-10, H1-5, HARS1, HAT1, HDAC2, HDAC4, HDGFL2, HEXB, HINT1, HMBS, HMGCS1, HMOX1, HNRNPC, HNRNPR, HSD17B10, HSD17B11, HSD17B4, HSP90AA1, HSPA5, HTATSF1, IARS2, IDH2, IDI1, IGF2BP1, IGF2BP2, IKBKB, IKBKG, ILF3, ILVBL, IMPA1, IMPACT, INPP4A, INTS1, INTS9, IREB2, ITM2C, IVNS1ABP, JAG1, KAT7, KAT8, KDM1A, KDM3B, KDSR, LANCL2, LARP1, LARP4B, LARP6, LIG1, LIG3, LIMS1, LMCD1, LMO7, LPCAT2, LPCAT4, LRPPRC, LRRFIP1, LRWD1, LSS, LUM, MACROH2A1, MAEA, MAGI1, MALT1, MAP2K3, MAP3K4, MAT2A, MBD3, MBOAT2, MBOAT7, MCM2, MCM3, MCM4, MCM5, MCM6, MCM7, MCMBP, ME1, MED17, MED23, MED30, METTL14, MGA, MGAT1, MGST1, MLF2, MLH1, MMAB, MOCS2, MOGS, MORF4L1, MOV10, MPHOSPH8, MPST, MRPL10, MRPS17, MRPS21, MRPS31, MRRF, MTA1, MTAP, MTR, MTRR, MVD, MVK, MYD88, MYO6, N6AMT1, NCK2, NCL, NCOA5, NCOA7, NDRG1, NDUFA13, NEDD4, NEDD8, NELFCD, NFATC2IP, NFKB2, NIBAN1, NIBAN2, NMD3, NMT1, NOCT, NOL11, NOP53, NPLOC4, NPM1, NPR2, NQO1, NSUN5, NT5C, NT5C3B, NUDT15, NUDT16, NUP35, NUP62, OAT, OGFOD1, OPLAH, ORC4, ORC5, OSBPL1A, OSBPL2, OXA1L, OXSR1, PAICS, PAIP1,	

					PANK4, PAPSS2, PARP1, PARP14, PARP4, PATZ1, PAWR, PBXIP1, PCK2, PCYT2, PDE5A, PDK1, PELO, PFAS, PFKM, PGM2L1, PGM3, PGP, PHGDH, PI4K2B, PIGO, PIGT, PIGU, PIK3C2A, PIK3CA, PIN1, PIP4K2B, PIR, PITHD1, PKLR, PKM, PLA2G15, PLA2G4A, PLD1, PLOD2, PLPP3, PLXNB2, PML, PMM2, PNP, POFUT1, POGLUT3, POGZ, POLD1, POLD2, POLE2, POLR1C, POLR1D, POLR2A, POLR2B, POLR2H, POLR2I, POLR3A, POLR3C, POMGNT1, POMGNT2, POMK, PPIE, PPM1B, PPP1CA, PPP3CA, PPP5C, PRIM2, PRKAA1, PRKAB2, PRKCI, PRKCSH, PRPSAP1, PRPSAP2, PRXL2B, PSAT1, PSIP1, PSMA3, PSMB10, PSMB2, PSMC2, PSMC3, PSMC6, PSMD1, PSMD12, PSMD14, PSMD2, PSMD6, PSMD7, PSMD8, PSME1, PSME2, PSME4, PSPC1, PTGES2, PTGIS, PTPMT1, PTRH2, PUS7, PXN, PYCARD, PYCR1, PYCR3, QARS1, QDPR, RAB5A, RAD21, RAD9A, RAP2C, RARS2, RBBP4, RBM4, RBP1, RDH10, RECQL, RFC2, RFC3, RFC5, RIF1, RIPK2, RNF2, RNGTT, RNPS1, ROR1, RPA1, RPA2, RPL10A, RPL13, RPL13A, RPL18A, RPL22, RPL24, RPL27, RPL32, RPL36, RPL38, RPL5, RPLP1, RPLP2, RPRD1A, RPRD1B, RPRD2, RPS13, RPS15A, RPS16, RPS18, RPS21, RPS23, RPS4Y1, RPS5, RPS8, RPS9, RRM1, RYBP, SAMHD1, SAP30BP, SARM1, SARNP, SARS1, SBDS, SCAI, SCP2, SEC14L2, SELENOT, SESN2, SET, SF1, SHFL, SHMT2, SIRT1,	

					SLC11A2, SLC1A1, SLC25A12, SLC25A13, SLC25A15, SLC26A2, SLC2A1, SLC30A9, SLC44A1, SLC44A2, SLC9A1, SMAD4, SMARCA1, SMARCB1, SMARCC1, SMARCD3, SMARCE1, SMC3, SMCHD1, SMPD1, SMYD3, SNF8, SNRPD3, SNRPE, SNW1, SNX5, SOAT1, SOD1, SOD2, SORBS3, SPHK2, SPTLC1, SPTLC2, SQSTM1, SRA1, SRC, SRD5A3, SRR, SRSF10, SRSF2, SSRP1, STAT1, STAT2, STAT5B, STON1, STRN3, SUFU, SUPT16H, SYNJ1, TAB2, TAF6L, TAX1BP1, TBCE, TBK1, TBPL1, TCP1, TDRD3, TF, TFAM, THBS1, TJP2, TKT, TPI1, TRADD, TRIM21, TRIM25, TRIM28, TRIM56, TRIP13, TRMT10C, TSPO, TTC5, TUT4, TXN, TYMP, UAP1, UBA2, UBA3, UBE2L6, UBR2, UBTF, UFD1, UGP2, UMPS, URI1, USP22, USP7, USP9X, UST, VARS1, VDAC2, WDR35, WDR5, WFS1, WIPI2, WRNIP1, WWC2, WWP2, XYLT2, YAP1, YARS1, YBX1, YEATS4, YES1, ZDHHC13, ZDHHC21, ZFP36L2, ZMPSTE24, ZNF148]	
heterocycle metabolic process	GO_BiologicalProcess-EBI-UniProt-GOA-ACAP-ARAP_13.05.2021_00h00	3.67E-55	10.265283	623	[AAR2, AARS1, AASS, ABCB7, ABCC1, ABCE1, ABT1, ACACA, ACOT7, ACOT8, ACSF2, ACSL3, ACSL4, ACSS2, ACTR2, ACTR3, ADARB1, ADK, ADNP, ADSL, AGO2, AGRN, AK1, AK3, AK4, AK6, AKR1B1, ALB, ALDH18A1, ALDOA, ANXA3, APBB2, APEX1, APP, APRT, AQR, ARHGEF11, ARID1B, ARRB1, ATP6V0C, ATP6V1A, BAZ1B, BCAS2, BDH2, BLVRB, BMS1, BPNT2, BRD3, C1QBP, CAMK2D, CASK, CASP3, CBX1,	Upregulation

					CBX2, CBX3, CBX5, CBX8, CC2D1A, CCDC22, CCN2, CCNH, CCNK, CCNL1, CCT2, CCT3, CCT6A, CD200, CD81, CDC34, CDC5L, CDC73, CDK1, CDK16, CDK17, CDK2, CDK5RAP3, CECR2, CELF2, CENPV, CETN2, CHAF1B, CHD1L, CHD2, CHD4, CHD7, CHORDC1, CHP1, CHTOP, CIRBP, CLU, CMPK1, CMTR1, CNGB1, CNOT1, CNOT10, COIL, COLEC12, CPSF2, CRABP2, CROT, CSTF3, CTBP1, CTNNB1, CTNNBL1, CTPS1, CTU1, CUL4B, CUX1, CWC22, DAB2, DARS1, DCAKD, DCPS, DDX1, DDX42, DDX47, DGUOK, DHFR, DHX34, DHX36, DICER1, DIMT1, DLST, DNMT1, DPYSL2, DPYSL3, DPYSL4, DRG1, DUT, DVL2, EARS2, EDF1, EED, EEF1E1, EFEMP1, EFL1, EIF2AK2, EIF3E, EIF6, ELP3, ELP4, ELP5, EMG1, ENO2, ENPP1, ENY2, EPHA2, EPHX1, EPRS1, ERBB2, ERCC5, EXD2, EXTL2, EZR, FAM3A, FANCI, FASN, FEN1, FGF2, FHL2, FHOD1, FMR1, FSCN1, FTO, FUT8, GAPDH, GARS1, GATAD2A, GATB, GCDH, GCFC2, GEMIN4, GFPT2, GLMP, GLO1, GLYR1, GMPS, GNE, GNL3L, GPHN, GPI, GPKOW, GTF2E1, GTF2F1, GTF2F2, GTF2I, GTF3C3, GTF3C4, H1-10, H1-5, HARS1, HAT1, HDAC2, HDAC4, HDGFL2, HERC2, HEXB, HINT1, HK1, HK2, HMBS, HMCES, HMGCS1, HMOX1, HNRNPA0, HNRNPA3, HNRNPC, HNRNPH1, HNRNPLL, HNRNPR, HNRNPUL1, HPX, HSD17B10, HSD17B4, HSP90AA1, HSPA5, HTATSF1, IARS2, IDH2, IGF2BP1,	

					IGF2BP2, IKBKB, IKBKG, ILF3, IMPACT, INTS1, INTS9, IREB2, IVNS1ABP, JAG1, KAT7, KAT8, KCNQ1, KDM1A, KDM3B, KDM4B, KPNB1, LANCL2, LARP1, LARP4B, LARP6, LDHA, LIG1, LIG3, LIMS1, LMCD1, LMO7, LRPPRC, LRRFIP1, LRWD1, LUM, MACROD1, MACROH2A1, MAGI1, MALT1, MAP2K3, MAP3K4, MBD3, MCM2, MCM3, MCM4, MCM5, MCM6, MCM7, MDC1, ME1, MED17, MED23, MED30, METTL14, MFN1, MGA, MGAT1, MGMT, MGST1, MLF2, MLH1, MMAB, MME, MMUT, MOCS2, MORF4L1, MOV10, MPHOSPH8, MRM3, MRTO4, MSH2, MSH6, MTA1, MTAP, MTHFD2, MTR, MTREX, MTRR, MVD, MVK, MYD88, MYO18A, MYO6, N6AMT1, NCK2, NCL, NCOA5, NCOA7, NDRG1, NDUFA13, NEDD4, NEDD8, NELFCD, NFATC2IP, NFKB2, NIBAN2, NMD3, NOCT, NOL11, NOP53, NPLOC4, NPM1, NPR2, NSMCE3, NSUN5, NT5C, NT5C3B, NUDT15, NUDT16, NUDT16L1, NUP205, NUP35, NUP62, OAT, ORC4, ORC5, OXSR1, PAICS, PAIP1, PANK4, PAPSS2, PARG, PARP1, PARP14, PARP4, PATZ1, PAWR, PBXIP1, PC, PCBP3, PDE5A, PDK1, PDS5B, PDZD11, PELO, PFAS, PFKM, PGM3, PIK3CA, PIN1, PIR, PITHD1, PKLR, PKM, PLD3, PLPP3, PML, PMM2, PNP, POFUT1, POGZ, POLD1, POLD2, POLE2, POLR1C, POLR1D, POLR2A, POLR2B, POLR2H, POLR2I, POLR3A, POLR3C, POP1, PPIE, PPIG, PPP3CA, PPP5C, PPWD1,	

					PRDX6, PRIM2, PRKAA1, PRKCI, PRMT7, PRPF19, PRPSAP1, PRPSAP2, PSAT1, PSIP1, PSMA3, PSMB10, PSMB2, PSMC2, PSMC3, PSMC6, PSMD1, PSMD12, PSMD14, PSMD2, PSMD6, PSMD7, PSMD8, PSME1, PSME2, PSME4, PSPC1, PTBP2, PTBP3, PTGES2, PTGIS, PTRH2, PUF60, PUS7, PXN, PYCARD, PYCR1, PYCR3, QARS1, QDPR, QTRT1, QTRT2, RAB23, RAD21, RAD9A, RAP2C, RARS2, RBM22, RBM26, RBM4, RBMS2, RECQL, RFC2, RFC3, RFC5, RIF1, RIOK3, RIPK2, RNF2, RNF40, RNGTT, RNPS1, ROR1, RPA1, RPA2, RPF2, RPL10A, RPL13, RPL13A, RPL18A, RPL22, RPL24, RPL27, RPL32, RPL36, RPL38, RPL5, RPLP1, RPLP2, RPRD1A, RPRD1B, RPRD2, RPS13, RPS15A, RPS16, RPS18, RPS21, RPS23, RPS4Y1, RPS5, RPS8, RPS9, RRM1, RYBP, SAMHD1, SAP30BP, SARM1, SARNP, SARS1, SBDS, SCAF11, SCAI, SEC14L2, SENP3, SESN2, SET, SF1, SF3A3, SF3B6, SHMT2, SHPRH, SIRT1, SLC11A2, SLC25A12, SLC25A13, SLC25A15, SLC25A19, SLC26A2, SLC30A9, SLC9A1, SMAD4, SMARCA1, SMARCAD1, SMARCB1, SMARCC1, SMARCD3, SMARCE1, SMC1A, SMC3, SMCHD1, SMNDC1, SMU1, SMYD3, SNF8, SNRNP200, SNRPA, SNRPD3, SNRPE, SNU13, SNW1, SNX5, SOAT1, SOD2, SON, SORBS3, SPHK2, SQSTM1, SRA1, SRC, SRSF10, SRSF2, SRSF5, SSB, SSRP1, STAT1,	

					STAT2, STAT5B, STON1, STRN3, SUFU, SULT1E1, SUPT16H, TAB2, TAF6L, TAX1BP1, TBK1, TBPL1, TCP1, TDRD3, TF, TFAM, TGS1, THADA, THEM4, THOC2, TIGAR, TJP2, TPI1, TPMT, TPST2, TRA2A, TRA2B, TRADD, TRIM21, TRIM25, TRIM28, TRIM56, TRIP12, TRIP13, TRMT10C, TRMT2A, TRMT5, TSEN15, TSEN34, TSPO, TSR2, TTC5, TUT4, TXN, TYMP, U2SURP, UAP1, UBA2, UBA3, UBE2L6, UBR2, UBTF, UFD1, UGP2, UHRF2, UMPS, URI1, URM1, USP22, USP28, USP4, USP7, USP9X, UTP14A, UTP20, UTP3, VARS1, WDR12, WDR70, WFS1, WRNIP1, WWC2, WWP2, YAP1, YARS1, YBEY, YBX1, YEATS4, YES1, YTHDC1, ZC3HAV1, ZFP36L2, ZMPSTE24, ZNF148]	
regulation of catalytic activity	GO_BiologicalProcess-EBI-UniProt-GOA-ACAP-ARAP_13.05.2021_00h00	3.67E-55	10.046729	258	[ABCE1, ABI1, ACAP2, ACTB, ADARB1, ADNP, AFDN, AGRN, ANXA3, ANXA5, APAF1, APLP2, APP, ARAF, ARFGAP1, ARFGEF1, ARHGAP12, ARHGAP17, ARHGAP5, ARHGEF11, ARHGEF12, ARPP19, ARRB1, ASAP2, ATP1B3, AUP1, B3GAT3, BAG1, BCR, BIN1, BMP1, BOD1, CAB39L, CAMK2D, CAP1, CAP2, CASP3, CBX8, CCN2, CCNH, CCNK, CCNL1, CCT2, CD109, CD200, CD81, CDK5RAP3, CEMIP, CHORDC1, CHP1, CHTOP, CLU, CORO1C, CPNE2, CSPG4, CSTB, CTNNB1, CTSC, CYCS, DAPK1, DENND2A, DHCR24, DHFR, DIPK2A, DNM1L, DNMBP, DOCK6, DUSP3, DVL2, DYNLL1, EIF2AK2, EIF2B1, EIF2S1, ELP3, ELP4, EPHA2, EPHA3,	No change

					EPHB4, EPM2AIP1, ERBB2, ERCC5, ETFDH, FARP1, FASN, FERMT2, FGD4, FGF2, FKBP1A, FN1, GAPDH, GDPGP1, GLA, GLMN, GPI, GPSM1, GSK3B, GSTP1, HERC2, HGS, HIP1, HNRNPR, HSP90AA1, HSPE1, IKBKB, IKBKG, IPO5, IQGAP3, ITGB1, ITIH4, ITSN1, LIMS1, LLGL1, MACROH2A1, MAD2L1, MALT1, MAP2K3, MAP3K20, MAP3K4, MAP4K4, MCM2, MCM7, MGMT, MMUT, MSH2, MSH6, MTCH1, MTRR, MVP, NCK2, NDUFA13, NDUFA4, NES, NF1, NF2, NLE1, NPM1, NQO1, NUCB2, NUDT19, NUP62, OSBPL8, OXA1L, PAFAH1B1, PARP1, PARP4, PBXIP1, PEBP1, PGAM5, PHPT1, PIK3CA, PIN1, PIP4K2B, PKN2, PLCG1, PLXNB2, PML, PPP1R14A, PPP1R7, PPP2R5A, PPP3CA, PRKAA1, PRKAB2, PRKG1, PRPSAP1, PRPSAP2, PSMA3, PSMD1, PSMD14, PSMD2, PSMD6, PSME1, PSME2, PSME4, PTGES2, PTPA, PTRH2, PYCARD, QARS1, RAB1A, RABGEF1, RANBP1, RANGAP1, RAP1GDS1, RAP2B, RAP2C, RCC1, RCN3, RDX, RFC2, RFC3, RFC5, RGS10, RHOC, RIC8A, RIC8B, RIN1, RIPK2, ROR1, RPL5, RPLP1, S100A10, SCARB2, SCRIB, SEC23B, SERPINB1, SERPINB2, SERPINB8, SERPINB9, SERPINE2, SESN2, SET, SH3BP4, SIRT1, SLC1A1, SLIT2, SMAP2, SMPD1, SMYD3, SNF8, SNX18, SOD1, SORT1, SPHK2, SRC, STK24, SUGT1, TAB2, TBC1D10A, TBC1D17, TBC1D22B, TBC1D4, TBC1D8B, TCP1, TF, TGM2, THBS1, TIGAR, TIPRL, TNIK,	

					TOR1AIP1, TOR1AIP2, TPX2, TRADD, TRIO, TSC2, TSPO, TXN, UACA, URI1, VDAC2, VLDLR, VPS25, VRK3, VTN, WDR35, WDR41, XIAP, ZDHHC21]	
organic cyclic compound metabolic process	GO_BiologicalProcess-EBI-UniProt-GOA-ACAP-ARAP_13.05.2021_00h00	3.67E-55	10.309116	657	[AAR2, AARS1, AASS, ABAT, ABCB7, ABCC1, ABCE1, ABT1, ACACA, ACADVL, ACAT2, ACOT7, ACOT8, ACSF2, ACSL3, ACSL4, ACSS2, ACTR2, ACTR3, ADARB1, ADK, ADNP, ADSL, AGO2, AGRN, AK1, AK3, AK4, AK6, AKR1B1, ALB, ALDH18A1, ALDOA, ANXA3, APBB2, APEX1, APP, APRT, AQR, ARHGEF11, ARID1B, ARRB1, ASAH1, ATP1A1, ATP6V0C, ATP6V1A, BAZ1B, BCAS2, BDH2, BLVRB, BMS1, BPNT2, BRD3, C1QBP, CAMK2D, CASK, CASP3, CBX1, CBX2, CBX3, CBX5, CBX8, CC2D1A, CCDC22, CCN2, CCNH, CCNK, CCNL1, CCT2, CCT3, CCT6A, CD200, CD81, CDC34, CDC5L, CDC73, CDK1, CDK16, CDK17, CDK2, CDK5RAP3, CECR2, CELF2, CENPV, CETN2, CHAF1B, CHD1L, CHD2, CHD4, CHD7, CHORDC1, CHP1, CHTOP, CIRBP, CLU, CMPK1, CMTR1, CNGB1, CNOT1, CNOT10, COIL, COLEC12, CPSF2, CRABP2, CROT, CSTF3, CTBP1, CTNNB1, CTNNBL1, CTPS1, CTU1, CUL4B, CUX1, CWC22, CYB5R3, CYP51A1, DAB2, DARS1, DCAKD, DCPS, DDX1, DDX42, DDX47, DGUOK, DHCR24, DHCR7, DHFR, DHX34, DHX36, DICER1, DIMT1, DLST, DNMT1, DPYSL2, DPYSL3, DPYSL4, DRG1, DUT, DVL2, EARS2, EDF1, EED, EEF1E1, EFEMP1, EFL1, EIF2AK2, EIF3E, EIF6, ELP3, ELP4, ELP5,	No change

					EMG1, ENO2, ENPP1, ENY2, EPHA2, EPHX1, EPRS1, ERBB2, ERCC5, ERLIN2, EXD2, EXTL2, EZR, FAM3A, FANCI, FASN, FDXR, FEN1, FGF2, FHL2, FHOD1, FMR1, FSCN1, FTO, FUT8, G6PD, GAPDH, GARS1, GATAD2A, GATB, GBA2, GCDH, GCFC2, GEMIN4, GFPT2, GIPC1, GLMP, GLO1, GLYR1, GMPS, GNE, GNL3L, GPHN, GPI, GPKOW, GSTM2, GSTM3, GTF2E1, GTF2F1, GTF2F2, GTF2I, GTF3C3, GTF3C4, H1-10, H1-5, HARS1, HAT1, HDAC2, HDAC4, HDGFL2, HERC2, HEXB, HINT1, HK1, HK2, HMBS, HMCES, HMGCS1, HMOX1, HNRNPA0, HNRNPA3, HNRNPC, HNRNPH1, HNRNPLL, HNRNPR, HNRNPUL1, HPX, HSD17B10, HSD17B11, HSD17B4, HSP90AA1, HSPA5, HTATSF1, IARS2, IDH2, IDI1, IGF2BP1, IGF2BP2, IGFBP7, IKBKB, IKBKG, ILF3, IMPACT, INTS1, INTS9, IREB2, IVNS1ABP, JAG1, KAT7, KAT8, KCNQ1, KDM1A, KDM3B, KDM4B, KPNB1, LANCL2, LARP1, LARP4B, LARP6, LBR, LDHA, LIG1, LIG3, LIMS1, LIPA, LMCD1, LMO7, LRPPRC, LRRFIP1, LRWD1, LSS, LUM, MACROD1, MACROH2A1, MAGI1, MALT1, MAOA, MAP2K3, MAP3K4, MBD3, MCM2, MCM3, MCM4, MCM5, MCM6, MCM7, MDC1, ME1, MED17, MED23, MED30, METTL14, MFN1, MGA, MGAT1, MGMT, MGST1, MLF2, MLH1, MMAB, MME, MMUT, MOCS2, MORF4L1, MOV10, MPHOSPH8, MRM3, MRTO4, MSH2, MSH6, MTA1, MTAP,	

					MTHFD2, MTR, MTREX, MTRR, MVD, MVK, MYD88, MYO18A, MYO6, N6AMT1, NCK2, NCL, NCOA5, NCOA7, NDRG1, NDUFA13, NEDD4, NEDD8, NELFCD, NFATC2IP, NFKB2, NIBAN2, NMD3, NOCT, NOL11, NOP53, NPLOC4, NPM1, NPR2, NSDHL, NSMCE3, NSUN5, NT5C, NT5C3B, NUDT15, NUDT16, NUDT16L1, NUP205, NUP35, NUP62, OAT, ORC4, ORC5, OSBPL1A, OSBPL2, OXSR1, PAICS, PAIP1, PANK4, PAPSS2, PARG, PARP1, PARP14, PARP4, PATZ1, PAWR, PBXIP1, PC, PCBP3, PDE5A, PDK1, PDS5B, PDZD11, PELO, PFAS, PFKM, PGM3, PIK3CA, PIN1, PIR, PITHD1, PKLR, PKM, PLD3, PLPP3, PML, PMM2, PNP, POFUT1, POGZ, POLD1, POLD2, POLE2, POLR1C, POLR1D, POLR2A, POLR2B, POLR2H, POLR2I, POLR3A, POLR3C, POP1, PPIE, PPIG, PPP3CA, PPP5C, PPWD1, PRDX6, PRIM2, PRKAA1, PRKCI, PRMT7, PRPF19, PRPSAP1, PRPSAP2, PSAT1, PSIP1, PSMA3, PSMB10, PSMB2, PSMC2, PSMC3, PSMC6, PSMD1, PSMD12, PSMD14, PSMD2, PSMD6, PSMD7, PSMD8, PSME1, PSME2, PSME4, PSPC1, PTBP2, PTBP3, PTGES2, PTGIS, PTRH2, PUF60, PUS7, PXN, PYCARD, PYCR1, PYCR3, QARS1, QDPR, QTRT1, QTRT2, RAB23, RAD21, RAD9A, RAP2C, RARS2, RBM22, RBM26, RBM4, RBMS2, RECQL, RFC2, RFC3, RFC5, RIF1, RIOK3, RIPK2, RNF2, RNF40, RNGTT, RNPS1, ROR1, RPA1, RPA2, RPF2, RPL10A,	

					RPL13, RPL13A, RPL18A, RPL22, RPL24, RPL27, RPL32, RPL36, RPL38, RPL5, RPLP1, RPLP2, RPRD1A, RPRD1B, RPRD2, RPS13, RPS15A, RPS16, RPS18, RPS21, RPS23, RPS4Y1, RPS5, RPS8, RPS9, RRM1, RYBP, SAMHD1, SAP30BP, SARM1, SARNP, SARS1, SBDS, SCAF11, SCAI, SCP2, SEC14L2, SENP3, SESN2, SET, SF1, SF3A3, SF3B6, SHMT2, SHPRH, SIRT1, SLC11A2, SLC1A1, SLC25A12, SLC25A13, SLC25A15, SLC25A19, SLC26A2, SLC30A9, SLC9A1, SMAD4, SMARCA1, SMARCAD1, SMARCB1, SMARCC1, SMARCD3, SMARCE1, SMC1A, SMC3, SMCHD1, SMNDC1, SMPD1, SMU1, SMYD3, SNF8, SNRNP200, SNRPA, SNRPD3, SNRPE, SNU13, SNW1, SNX5, SOAT1, SOD1, SOD2, SON, SORBS3, SPHK2, SQSTM1, SRA1, SRC, SRD5A3, SRSF10, SRSF2, SRSF5, SSB, SSRP1, STAT1, STAT2, STAT5B, STON1, STRN3, SUFU, SULT1E1, SUPT16H, TAB2, TAF6L, TAX1BP1, TBK1, TBPL1, TCP1, TDRD3, TF, TFAM, TGS1, THADA, THEM4, THOC2, TIGAR, TJP2, TPI1, TPMT, TPST2, TRA2A, TRA2B, TRADD, TRIM21, TRIM25, TRIM28, TRIM56, TRIP12, TRIP13, TRMT10C, TRMT2A, TRMT5, TSEN15, TSEN34, TSPO, TSR2, TTC39B, TTC5, TUT4, TXN, TYMP, U2SURP, UAP1, UBA2, UBA3, UBE2L6, UBR2, UBTF, UFD1, UGP2, UHRF2, UMPS, URI1, URM1, USP22, USP28, USP4, USP7, USP9X, UTP14A, UTP20, UTP3, VARS1, VDAC2,	

					VLDLR, WDR12, WDR70, WFS1, WRNIP1, WWC2, WWP2, YAP1, YARS1, YBEY, YBX1, YEATS4, YES1, YTHDC1, ZC3HAV1, ZFP36L2, ZMPSTE24, ZNF148]	
organonitrogen compound metabolic process	GO_BiologicalProcess-EBI-UniProt-GOA-ACAP-ARAP_13.05.2021_00h00	3.67E-55	10.641965	741	[AARS1, AASDHPPT, AASS, ABAT, ABCB7, ABCC1, ABCE1, ABHD12, ABHD16A, ABI1, ACACA, ACAD8, ACADM, ACADSB, ACO1, ACOT7, ACOT8, ACSF2, ACSL3, ACSL4, ACSS2, ACTB, ADAM10, ADAMTS9, ADARB1, ADK, ADNP, ADSL, AGO2, AGRN, AK1, AK3, AK4, AKR1B1, ALB, ALDH18A1, ALDH3A2, ALDOA, ALG11, ANKZF1, APAF1, APEX1, APIP, APLP2, APP, APRT, ARAF, ARFGEF1, ARMT1, ARPP19, ARRB1, ASAH1, ASB1, ASH2L, ASNS, ASPSCR1, ATE1, ATG4B, ATG7, ATP6V0C, ATP6V1A, AUP1, B2M, B3GAT3, B4GALT1, B4GALT5, BAZ1B, BCAT1, BCR, BDH2, BIN1, BLVRB, BMP1, BOD1, BPNT2, BST1, C1GALT1, C1QBP, CAB39L, CAMK2D, CAPN5, CASK, CASP3, CASP6, CBX8, CCDC22, CCN2, CCNH, CCNK, CCNL1, CD109, CD81, CDC34, CDC42BPA, CDC73, CDK1, CDK16, CDK17, CDK2, CDK5RAP3, CEMIP, CERS6, CFAP20, CHAMP1, CHORDC1, CHP1, CHPF, CHSY3, CHTOP, CIRBP, CKB, CLIC1, CLTC, CLU, CMPK1, CNOT1, CNOT10, COG7, COL11A1, CORO1C, CPD, CRBN, CROT, CRTAP, CSPG4, CSTB, CTBP1, CTNNB1, CTPS1, CTSA, CTSC, CTU1, CUL1, CUL4B, CYCS, CYP51A1, DAB2, DAPK1, DARS1, DCAKD, DCN, DCPS, DDI2, DDX1, DENR, DERL1, DGLUCY, DGUOK, DHCR24, DHFR, DHPS, DHX36,	No change

					DIPK2A, DLST, DNM1L, DNMT1, DPP3, DPP9, DPYSL2, DPYSL3, DPYSL4, DRG1, DSP, DUSP23, DUSP3, DUT, DVL2, DYNLL1, EARS2, ECE1, EED, EEF1E1, EEF1G, EFEMP1, EFL1, EIF2AK2, EIF2B1, EIF2D, EIF2S1, EIF3A, EIF3B, EIF3E, EIF3F, EIF4G3, EIF6, ELP3, ELP4, ENO2, ENPP1, ENY2, EOGT, EPHA2, EPHA3, EPHB4, EPRS1, ERAP1, ERBB2, ERLIN2, ERP44, ESYT1, ETFDH, ETHE1, EXTL2, EZR, FAM3A, FASN, FBXL12, FBXW8, FDXR, FERMT2, FGF2, FKBP1A, FKBP5, FKBP9, FMR1, FN1, FN3K, FTO, FUT11, FUT8, G6PD, GALNT1, GALNT2, GALNT7, GAMT, GAN, GAPDH, GARS1, GATB, GBA2, GCAT, GCDH, GDAP1, GFPT2, GGA2, GGCT, GIPC1, GLA, GLE1, GLMN, GLO1, GLRX3, GLUD1, GLUL, GLYR1, GMPS, GNE, GNL3L, GOLGA7, GOLT1B, GOT1, GPC1, GPC6, GPD1L, GPHN, GPI, GSK3B, GSTM2, GSTM3, GSTP1, GTF3C4, GXYLT1, GXYLT2, H1-5, HARS1, HAT1, HDAC2, HDAC4, HECTD4, HERC2, HERC4, HEXB, HGS, HIBCH, HINT1, HIP1, HK1, HK2, HLA-A, HMBS, HMCES, HMGCS1, HMOX1, HNRNPC, HNRNPR, HPX, HSD17B10, HSD17B4, HSP90AA1, HSP90B1, HSPA5, HSPE1, HSPG2, IARS2, IDH2, IGF2BP1, IGF2BP2, IGFBP7, IKBKB, IKBKG, ILF3, ILKAP, ILVBL, IMPACT, IPO5, IQGAP3, IREB2, IST1, ITGAV, ITIH4, ITM2C, IVNS1ABP, KAT7, KAT8, KDM1A, KDM3B, KDM4B, KDSR, LACTB, LAMC1, LAP3, LARP1, LARP4B, LARP6, LDHA, LLGL1,	

					LMCD1, LMO7, LNPEP, LPCAT2, LPCAT4, LRPPRC, LRRK1, LTBP1, LUM, LYPLA2, MACROD1, MACROH2A1, MAD2L1, MAEA, MAGI1, MALT1, MAOA, MAP2K3, MAP3K20, MAP3K4, MAP4K4, MARCHF5, MBD3, MBOAT2, MBOAT7, MCM2, MED30, METTL14, MFN1, MGAT1, MGMT, MGST1, MLH1, MMAB, MME, MMUT, MOCS2, MOGS, MORF4L1, MOV10, MPST, MRPL10, MRPS17, MRPS21, MRPS31, MRRF, MSN, MTA1, MTAP, MTCH1, MTHFD2, MTR, MTRR, MVD, MVK, MVP, MYADM, MYH9, N6AMT1, NCK2, NCL, NCLN, NCOA7, NDRG1, NDUFA13, NDUFAF7, NEDD4, NEDD8, NEK1, NF1, NF2, NIBAN1, NLE1, NMT1, NNT, NOCT, NOP53, NPLOC4, NPM1, NPR2, NQO1, NSMCE3, NSUN5, NT5C, NT5C3B, NUB1, NUDT15, NUDT16, NUP205, NUP35, NUP62, NXN, OAT, OBSL1, OGFOD1, OPLAH, OSBPL10, OSBPL8, OTUD6B, OXA1L, OXSR1, P3H1, P3H3, PACSIN3, PAFAH1B1, PAICS, PAIP1, PANK4, PAPSS2, PARG, PARL, PARP1, PARP14, PARP4, PAWR, PC, PDCD6IP, PDE5A, PDK1, PDXDC1, PDZD11, PDZRN3, PEBP1, PELO, PFAS, PFKM, PGAM5, PGM3, PGP, PHGDH, PHKB, PHPT1, PIGO, PIGT, PIGU, PIK3CA, PIN1, PITHD1, PITRM1, PKLR, PKM, PKN2, PLA2G15, PLA2G4A, PLD1, PLOD2, PLPP3, PLXNB2, PML, PMM2, PNP, PNPLA6, POFUT1, POGLUT3, POMGNT1, POMGNT2, POMK, PPIB, PPIE, PPIG, PPM1B, PPP1CA, PPP1R14A, PPP1R7, PPP2R5A, PPP3CA,	

					PPP5C, PPWD1, PRKAA1, PRKAB2, PRKCI, PRKCSH, PRKG1, PRMT7, PRPF19, PRPSAP1, PRPSAP2, PSAT1, PSMA3, PSMB10, PSMB2, PSMC2, PSMC3, PSMC6, PSMD1, PSMD12, PSMD14, PSMD2, PSMD6, PSMD7, PSMD8, PSME1, PSME2, PSME4, PTGES2, PTGIS, PTK7, PTPA, PTPMT1, PTPN12, PTPRF, PTPRG, PTRH2, PUS7, PXK, PYCARD, PYCR1, PYCR3, QARS1, QDPR, QSOX1, RAB11B, RAB1A, RAB23, RAB3D, RAB5A, RAB8A, RANGAP1, RAP2B, RAP2C, RARS2, RBM4, RCN1, RCN3, RDX, RIF1, RIOK3, RIPK2, RNF114, RNF2, RNF213, RNF214, RNF40, RNGTT, ROR1, RPL10A, RPL13, RPL13A, RPL18A, RPL22, RPL24, RPL27, RPL32, RPL36, RPL38, RPL5, RPLP1, RPLP2, RPRD1A, RPRD1B, RPS13, RPS15A, RPS16, RPS18, RPS21, RPS23, RPS4Y1, RPS5, RPS8, RPS9, RRM1, RSPRY1, RTN4, RYBP, SAE1, SAMHD1, SARM1, SARNP, SARS1, SBDS, SCLY, SCPEP1, SCRN1, SEC11C, SEC61B, SELENOT, SENP3, SERPINB1, SERPINB2, SERPINB8, SERPINB9, SERPINE2, SESN2, SET, SGSH, SHFL, SHMT2, SHPRH, SIRT1, SLC11A2, SLC1A1, SLC25A12, SLC25A13, SLC25A15, SLC25A19, SLC26A2, SLC44A1, SLC44A2, SLC9A1, SLIT2, SMAD4, SMARCAD1, SMARCB1, SMARCC1, SMPD1, SMYD3, SNF8, SNW1, SNX1, SNX5, SOAT1, SOD1, SPHK2, SPTLC1, SPTLC2, SQSTM1, SRC, SRD5A3, SRR,	

					STAT2, STAT5B, STK24, SUFU, SULT1E1, TAB2, TAF6L, TBC1D10A, TBCE, TBK1, TBPL1, TF, TGFBI, TGFBR3, TGM2, THBS1, THEM4, TIGAR, TIPRL, TJP2, TMEM119, TMX3, TNIK, TPI1, TPP1, TPP2, TPST2, TPX2, TRADD, TRIM21, TRIM25, TRIM28, TRIM56, TRIM59, TRIO, TRIP12, TRMT10C, TSC2, TSPO, TTLL12, TUT4, TXN, TYMP, UACA, UBA2, UBA3, UBAC2, UBE2L6, UBE2Q1, UBR2, UBR4, UBXN2A, UFD1, UHRF2, UMPS, UQCRC2, URI1, URM1, USP11, USP22, USP28, USP4, USP48, USP5, USP7, USP9X, USP9Y, UST, VARS1, VDAC2, VLDLR, VPS25, VPS36, VRK1, VRK3, VTN, WDR35, WDR5, WDR70, WFS1, WIPI2, WWP2, XIAP, XPNPEP3, XYLT2, YARS1, YBEY, YBX1, YEATS4, YES1, YOD1, ZC3HAV1, ZDHHC13, ZDHHC21, ZFP36L2, ZMPSTE24]	
organic substance biosynthetic process	GO_BiologicalProcess-EBI-UniProt-GOA-ACAP-ARAP_13.05.2021_00h00	3.67E-55	10.329807	664	[AARS1, AASDHPPT, AASS, ABAT, ABCB7, ABCE1, ABT1, ACACA, ACADM, ACADVL, ACAT2, ACO1, ACOT7, ACOT8, ACSL3, ACSL4, ACSS2, ACTR2, ACTR3, ADK, ADNP, ADSL, AGO2, AGPAT2, AGPS, AGRN, AK1, AK3, AK4, AK6, AKAP12, AKR1B1, ALB, ALDH18A1, ALDH3A2, ALDOA, ALG11, ANXA3, APBB2, APEX1, APIP, APP, APRT, ARFGEF1, ARHGEF11, ARID1B, ARPP19, ARRB1, ASAH1, ASNS, ATG7, ATP1A1, ATP6V0C, ATP6V1A, B3GAT3, B4GALT1, B4GALT5, BAZ1B, BCAT1, BDH2, BPNT2, BRD3, C1GALT1, C1QBP, CAMK2D, CASK, CBR1, CBX1, CBX2, CBX3, CBX5, CBX8, CC2D1A, CCDC22,	No change

					CCN2, CCNH, CCNK, CCNL1, CCT2, CCT3, CCT6A, CD200, CD81, CDC34, CDC5L, CDC73, CDIPT, CDK1, CDK16, CDK17, CDK2, CDK5RAP3, CEMIP, CENPV, CERS6, CHAF1B, CHD2, CHD4, CHD7, CHORDC1, CHP1, CHPF, CHSY3, CIRBP, CKB, CLTC, CLU, CMPK1, CNGB1, CNOT1, CNOT10, COG7, COL5A1, COLEC12, COQ5, CPSF2, CRABP2, CSPG4, CSTF3, CTBP1, CTNNB1, CTPS1, CUX1, CYB5R3, CYP51A1, DAB2, DAGLB, DAPK1, DARS1, DCAKD, DCN, DCPS, DCXR, DDX1, DENR, DGUOK, DHCR24, DHCR7, DHFR, DHPS, DHRS7B, DHX34, DHX36, DICER1, DNMT1, DRG1, DUT, DVL2, EARS2, EDF1, EED, EEF1E1, EEF1G, EFEMP1, EFL1, EIF2AK2, EIF2B1, EIF2D, EIF2S1, EIF3A, EIF3B, EIF3E, EIF3F, EIF4G3, EIF6, ELP3, ELP4, ENO2, ENPP1, ENY2, EOGT, EPM2AIP1, EPRS1, ERBB2, ERLIN2, EXD2, EXTL2, EZR, FAM3A, FASN, FDXR, FEN1, FGF2, FHL2, FHOD1, FMR1, FTO, FUT11, FUT8, G6PD, GALNT1, GALNT2, GALNT7, GAMT, GAPDH, GARS1, GATAD2A, GATB, GBE1, GCDH, GCFC2, GFPT2, GGCT, GIPC1, GLE1, GLMP, GLO1, GLUD1, GLUL, GLYR1, GMPS, GNE, GNL3L, GOLGA7, GOLT1B, GOT1, GPC1, GPC6, GPD1L, GPHN, GPI, GRWD1, GSK3B, GSTM2, GSTM3, GSTP1, GTF2E1, GTF2F1, GTF2F2, GTF2I, GTF3C3, GTF3C4, GXYLT1, GXYLT2, GYS1, H1-10, H1-5, HARS1, HAT1, HDAC2, HDAC4, HDGFL2, HEXB, HINT1, HMBS, HMGCS1, HMOX1, HNRNPC, HNRNPR, HSD17B10,	

					HSD17B11, HSD17B4, HSP90AA1, HSPA5, HSPG2, HTATSF1, IARS2, IDH2, IDI1, IGF2BP1, IGF2BP2, IGFBP7, IKBKB, IKBKG, ILF3, ILVBL, IMPA1, IMPACT, INPP4A, INTS1, INTS9, IREB2, ITM2C, IVNS1ABP, JAG1, KAT7, KAT8, KDM1A, KDM3B, KDSR, KPNB1, LANCL2, LARP1, LARP4B, LARP6, LBR, LIG1, LIG3, LIMS1, LMCD1, LMO7, LPCAT2, LPCAT4, LRPPRC, LRRFIP1, LRWD1, LSS, LUM, MACROH2A1, MAEA, MAGI1, MALT1, MAP2K3, MAP3K4, MAT2A, MBD3, MBOAT2, MBOAT7, MCM2, MCM3, MCM4, MCM5, MCM6, MCM7, MCMBP, ME1, MED17, MED23, MED30, METTL14, MGA, MGAT1, MGST1, MLF2, MLH1, MMAB, MOCS2, MOGS, MORF4L1, MOV10, MPHOSPH8, MRPL10, MRPS17, MRPS21, MRPS31, MRRF, MTA1, MTAP, MTR, MTRR, MVD, MVK, MYD88, MYO6, N6AMT1, NCK2, NCL, NCOA5, NCOA7, NDRG1, NDUFA13, NEDD4, NEDD8, NELFCD, NFATC2IP, NFKB2, NIBAN1, NIBAN2, NMD3, NMT1, NOCT, NOL11, NOP53, NPLOC4, NPM1, NPR2, NSDHL, NSUN5, NT5C, NT5C3B, NUDT15, NUDT16, NUP35, NUP62, OAT, OGFOD1, OPLAH, ORC4, ORC5, OSBPL1A, OSBPL2, OXA1L, OXSR1, PAICS, PAIP1, PANK4, PAPSS2, PARP1, PARP14, PARP4, PATZ1, PAWR, PBXIP1, PC, PCK2, PCYT2, PDE5A, PDK1, PELO, PFAS, PFKM, PGM2L1, PGM3, PGP, PHGDH, PI4K2B, PIGO, PIGT, PIGU, PIK3C2A,	

					PIK3CA, PIN1, PIP4K2B, PIR, PITHD1, PKLR, PKM, PLA2G15, PLA2G4A, PLD1, PLOD2, PLPP3, PLXNB2, PML, PMM2, PNP, POFUT1, POGLUT3, POGZ, POLD1, POLD2, POLE2, POLR1C, POLR1D, POLR2A, POLR2B, POLR2H, POLR2I, POLR3A, POLR3C, POMGNT1, POMGNT2, POMK, PPIE, PPIP5K2, PPM1B, PPP1CA, PPP3CA, PPP5C, PRIM2, PRKAA1, PRKAB2, PRKCI, PRKCSH, PRPF19, PRPSAP1, PRPSAP2, PRXL2B, PSAT1, PSIP1, PSMA3, PSMB10, PSMB2, PSMC2, PSMC3, PSMC6, PSMD1, PSMD12, PSMD14, PSMD2, PSMD6, PSMD7, PSMD8, PSME1, PSME2, PSME4, PSPC1, PTGES2, PTGIS, PTPMT1, PTRH2, PUS7, PXN, PYCARD, PYCR1, PYCR3, QARS1, QDPR, RAB5A, RAD21, RAD9A, RAP2C, RARS2, RBBP4, RBM4, RBP1, RDH10, RECQL, RFC2, RFC3, RFC5, RIF1, RIPK2, RNF2, RNGTT, RNPS1, ROR1, RPA1, RPA2, RPL10A, RPL13, RPL13A, RPL18A, RPL22, RPL24, RPL27, RPL32, RPL36, RPL38, RPL5, RPLP1, RPLP2, RPRD1A, RPRD1B, RPRD2, RPS13, RPS15A, RPS16, RPS18, RPS21, RPS23, RPS4Y1, RPS5, RPS8, RPS9, RRM1, RYBP, SAMHD1, SAP30BP, SARM1, SARNP, SARS1, SBDS, SCAI, SCP2, SEC14L2, SELENOT, SESN2, SET, SF1, SHFL, SHMT2, SIRT1, SLC11A2, SLC1A1, SLC25A10, SLC25A12, SLC25A13, SLC25A15, SLC26A2, SLC2A1, SLC30A9, SLC44A1, SLC44A2, SLC9A1, SMAD4, SMARCA1,	

					SMARCB1, SMARCC1, SMARCD3, SMARCE1, SMC3, SMCHD1, SMPD1, SMYD3, SNF8, SNRPD3, SNRPE, SNW1, SNX5, SOAT1, SOD1, SOD2, SORBS3, SPHK2, SPTLC1, SPTLC2, SQSTM1, SRA1, SRC, SRD5A3, SRR, SRSF10, SRSF2, SSRP1, STAT1, STAT2, STAT5B, STON1, STRN3, SUFU, SUPT16H, SYNJ1, TAB2, TAF6L, TALDO1, TAX1BP1, TBCE, TBK1, TBPL1, TCP1, TDRD3, TF, TFAM, THBS1, TJP2, TKT, TPI1, TRADD, TRIM21, TRIM25, TRIM28, TRIM56, TRIP13, TRMT10C, TSPO, TTC5, TUT4, TXN, TYMP, UAP1, UBA2, UBA3, UBE2L6, UBR2, UBTF, UFD1, UGP2, UMPS, URI1, USP22, USP7, USP9X, UST, VARS1, VDAC2, WDR5, WFS1, WIPI2, WRNIP1, WWC2, WWP2, XYLB, XYLT2, YAP1, YARS1, YBX1, YEATS4, YES1, ZDHHC13, ZDHHC21, ZFP36L2, ZMPSTE24, ZNF148]	
nucleobase-containing compound metabolic process	GO_BiologicalProcess-EBI-UniProt-GOA-ACAP-ARAP_13.05.2021_00h00	3.67E-55	10.151335	597	[AAR2, AARS1, AASS, ABCE1, ABT1, ACACA, ACOT7, ACOT8, ACSF2, ACSL3, ACSL4, ACSS2, ACTR2, ACTR3, ADARB1, ADK, ADNP, ADSL, AGO2, AGRN, AK1, AK3, AK4, AK6, AKR1B1, ALDOA, ANXA3, APBB2, APEX1, APP, APRT, AQR, ARHGEF11, ARID1B, ARRB1, ATP6V0C, ATP6V1A, BAZ1B, BCAS2, BMS1, BPNT2, BRD3, C1QBP, CAMK2D, CASK, CASP3, CBX1, CBX2, CBX3, CBX5, CBX8, CC2D1A, CCDC22, CCN2, CCNH, CCNK, CCNL1, CCT2, CCT3, CCT6A, CD200, CD81, CDC34, CDC5L, CDC73, CDK1, CDK16, CDK17, CDK2, CDK5RAP3, CECR2, CELF2, CENPV, CETN2,	No change

					CHAF1B, CHD1L, CHD2, CHD4, CHD7, CHORDC1, CHP1, CHTOP, CIRBP, CLU, CMPK1, CMTR1, CNGB1, CNOT1, CNOT10, COIL, COLEC12, CPSF2, CRABP2, CROT, CSTF3, CTBP1, CTNNB1, CTNNBL1, CTPS1, CTU1, CUL4B, CUX1, CWC22, DAB2, DARS1, DCAKD, DCPS, DDX1, DDX42, DDX47, DGUOK, DHFR, DHX34, DHX36, DICER1, DIMT1, DLST, DNMT1, DPYSL2, DPYSL3, DPYSL4, DRG1, DUT, DVL2, EARS2, EDF1, EED, EEF1E1, EFEMP1, EFL1, EIF2AK2, EIF3E, EIF6, ELP3, ELP4, ELP5, EMG1, ENO2, ENPP1, ENY2, EPHA2, EPRS1, ERBB2, ERCC5, EXD2, EXTL2, EZR, FAM3A, FANCI, FASN, FEN1, FGF2, FHL2, FHOD1, FMR1, FSCN1, FTO, FUT8, GAPDH, GARS1, GATAD2A, GATB, GCDH, GCFC2, GEMIN4, GFPT2, GLMP, GLO1, GLYR1, GMPS, GNE, GNL3L, GPI, GPKOW, GTF2E1, GTF2F1, GTF2F2, GTF2I, GTF3C3, GTF3C4, H1-10, H1-5, HARS1, HAT1, HDAC2, HDAC4, HDGFL2, HERC2, HEXB, HINT1, HK1, HK2, HMCES, HMGCS1, HMOX1, HNRNPA0, HNRNPA3, HNRNPC, HNRNPH1, HNRNPLL, HNRNPR, HNRNPUL1, HSD17B10, HSD17B4, HSP90AA1, HSPA5, HTATSF1, IARS2, IDH2, IGF2BP1, IGF2BP2, IKBKB, IKBKG, ILF3, IMPACT, INTS1, INTS9, IVNS1ABP, JAG1, KAT7, KAT8, KCNQ1, KDM1A, KDM3B, KDM4B, KPNB1, LANCL2, LARP1, LARP4B, LARP6, LDHA, LIG1, LIG3, LIMS1, LMCD1, LMO7, LRPPRC, LRRFIP1, LRWD1, LUM, MACROD1,	

					MACROH2A1, MAGI1, MALT1, MAP2K3, MAP3K4, MBD3, MCM2, MCM3, MCM4, MCM5, MCM6, MCM7, MDC1, ME1, MED17, MED23, MED30, METTL14, MFN1, MGA, MGAT1, MGMT, MLF2, MLH1, MMAB, MORF4L1, MOV10, MPHOSPH8, MRM3, MRTO4, MSH2, MSH6, MTA1, MTAP, MTREX, MTRR, MVD, MVK, MYD88, MYO18A, MYO6, N6AMT1, NCK2, NCL, NCOA5, NCOA7, NDRG1, NDUFA13, NEDD4, NEDD8, NELFCD, NFATC2IP, NFKB2, NIBAN2, NMD3, NOCT, NOL11, NOP53, NPLOC4, NPM1, NPR2, NSMCE3, NSUN5, NT5C, NT5C3B, NUDT15, NUDT16, NUDT16L1, NUP205, NUP35, NUP62, ORC4, ORC5, OXSR1, PAICS, PAIP1, PANK4, PAPSS2, PARG, PARP1, PARP14, PARP4, PATZ1, PAWR, PBXIP1, PCBP3, PDE5A, PDK1, PDS5B, PELO, PFAS, PFKM, PGM3, PIK3CA, PIN1, PIR, PITHD1, PKLR, PKM, PLD3, PLPP3, PML, PMM2, PNP, POFUT1, POGZ, POLD1, POLD2, POLE2, POLR1C, POLR1D, POLR2A, POLR2B, POLR2H, POLR2I, POLR3A, POLR3C, POP1, PPIE, PPIG, PPP3CA, PPP5C, PPWD1, PRDX6, PRIM2, PRKAA1, PRKCI, PRMT7, PRPF19, PRPSAP1, PRPSAP2, PSIP1, PSMA3, PSMB10, PSMB2, PSMC2, PSMC3, PSMC6, PSMD1, PSMD12, PSMD14, PSMD2, PSMD6, PSMD7, PSMD8, PSME1, PSME2, PSME4, PSPC1, PTBP2, PTBP3, PTGES2, PTGIS, PTRH2, PUF60, PUS7, PXN, PYCARD,	

					QARS1, QTRT1, QTRT2, RAB23, RAD21, RAD9A, RAP2C, RARS2, RBM22, RBM26, RBM4, RBMS2, RECQL, RFC2, RFC3, RFC5, RIF1, RIOK3, RIPK2, RNF2, RNF40, RNGTT, RNPS1, ROR1, RPA1, RPA2, RPF2, RPL10A, RPL13, RPL13A, RPL18A, RPL22, RPL24, RPL27, RPL32, RPL36, RPL38, RPL5, RPLP1, RPLP2, RPRD1A, RPRD1B, RPRD2, RPS13, RPS15A, RPS16, RPS18, RPS21, RPS23, RPS4Y1, RPS5, RPS8, RPS9, RRM1, RYBP, SAMHD1, SAP30BP, SARM1, SARNP, SARS1, SBDS, SCAF11, SCAI, SEC14L2, SENP3, SESN2, SET, SF1, SF3A3, SF3B6, SHMT2, SHPRH, SIRT1, SLC25A12, SLC25A13, SLC25A15, SLC26A2, SLC30A9, SLC9A1, SMAD4, SMARCA1, SMARCAD1, SMARCB1, SMARCC1, SMARCD3, SMARCE1, SMC1A, SMC3, SMCHD1, SMNDC1, SMU1, SMYD3, SNF8, SNRNP200, SNRPA, SNRPD3, SNRPE, SNU13, SNW1, SNX5, SOAT1, SOD2, SON, SORBS3, SPHK2, SQSTM1, SRA1, SRC, SRSF10, SRSF2, SRSF5, SSB, SSRP1, STAT1, STAT2, STAT5B, STON1, STRN3, SUFU, SULT1E1, SUPT16H, TAB2, TAF6L, TAX1BP1, TBK1, TBPL1, TCP1, TDRD3, TF, TFAM, TGS1, THADA, THEM4, THOC2, TIGAR, TJP2, TPI1, TPMT, TPST2, TRA2A, TRA2B, TRADD, TRIM21, TRIM25, TRIM28, TRIM56, TRIP12, TRIP13, TRMT10C, TRMT2A, TRMT5, TSEN15, TSEN34, TSPO, TSR2, TTC5, TUT4, TXN, TYMP, U2SURP, UAP1,	

					UBA2, UBA3, UBE2L6, UBR2, UBTF, UFD1, UGP2, UHRF2, UMPS, URI1, URM1, USP22, USP28, USP4, USP7, USP9X, UTP14A, UTP20, UTP3, VARS1, WDR12, WDR70, WFS1, WRNIP1, WWC2, WWP2, YAP1, YARS1, YBEY, YBX1, YEATS4, YES1, YTHDC1, ZC3HAV1, ZFP36L2, ZMPSTE24, ZNF148]	
protein metabolic process	GO_BiologicalProcess-EBI-UniProt-GOA-ACAP-ARAP_13.05.2021_00h00	3.67E-55	10.037049	596	[AARS1, AASDHPPT, AASS, ABCE1, ABHD12, ABI1, ACACA, ACO1, ACOT8, ACTB, ADAM10, ADAMTS9, ADARB1, ADNP, AGO2, AKR1B1, ALB, ALG11, ANKZF1, APAF1, APEX1, APLP2, APP, ARAF, ARFGEF1, ARMT1, ARPP19, ARRB1, ASB1, ASH2L, ASPSCR1, ATE1, ATG4B, ATG7, AUP1, B2M, B3GAT3, B4GALT1, B4GALT5, BAZ1B, BCR, BIN1, BMP1, BOD1, BPNT2, BST1, C1GALT1, C1QBP, CAB39L, CAMK2D, CAPN5, CASK, CASP3, CASP6, CBX8, CCDC22, CCN2, CCNH, CCNK, CCNL1, CD109, CD81, CDC34, CDC42BPA, CDC73, CDK1, CDK16, CDK17, CDK2, CDK5RAP3, CEMIP, CFAP20, CHAMP1, CHORDC1, CHP1, CHPF, CHSY3, CHTOP, CIRBP, CLTC, CLU, CNOT1, CNOT10, COG7, COL11A1, CORO1C, CPD, CRBN, CRTAP, CSPG4, CSTB, CTBP1, CTNNB1, CTSA, CTSC, CTU1, CUL1, CUL4B, CYCS, CYP51A1, DAB2, DAPK1, DARS1, DCN, DCPS, DDI2, DDX1, DENR, DERL1, DGUOK, DHCR24, DHFR, DHPS, DHX36, DIPK2A, DLST, DNM1L, DNMT1, DPP3, DPP9, DRG1, DSP, DUSP23, DUSP3, DVL2, DYNLL1, EARS2, ECE1, EED, EEF1E1, EEF1G, EFEMP1,	No change

					EFL1, EIF2AK2, EIF2B1, EIF2D, EIF2S1, EIF3A, EIF3B, EIF3E, EIF3F, EIF4G3, EIF6, ELP3, ELP4, ENPP1, ENY2, EOGT, EPHA2, EPHA3, EPHB4, EPRS1, ERAP1, ERBB2, ERLIN2, ERP44, ETFDH, EXTL2, EZR, FAM3A, FASN, FBXL12, FBXW8, FDXR, FERMT2, FGF2, FKBP1A, FKBP5, FKBP9, FMR1, FN1, FN3K, FTO, FUT11, FUT8, G6PD, GALNT1, GALNT2, GALNT7, GAN, GAPDH, GARS1, GATB, GFPT2, GGA2, GIPC1, GLE1, GLMN, GLRX3, GLUL, GLYR1, GNL3L, GOLGA7, GPC1, GPD1L, GPHN, GPI, GSK3B, GSTP1, GTF3C4, GXYLT1, GXYLT2, H1-5, HARS1, HAT1, HDAC2, HDAC4, HECTD4, HERC2, HERC4, HEXB, HGS, HIP1, HLA-A, HMBS, HMCES, HNRNPC, HNRNPR, HPX, HSP90AA1, HSP90B1, HSPA5, HSPE1, HSPG2, IARS2, IGF2BP1, IGF2BP2, IGFBP7, IKBKB, IKBKG, ILF3, ILKAP, IMPACT, IPO5, IQGAP3, IREB2, IST1, ITGAV, ITIH4, ITM2C, IVNS1ABP, KAT7, KAT8, KDM1A, KDM3B, KDM4B, LACTB, LAMC1, LAP3, LARP1, LARP4B, LARP6, LLGL1, LMCD1, LMO7, LNPEP, LRPPRC, LRRK1, LTBP1, LYPLA2, MACROD1, MACROH2A1, MAD2L1, MAEA, MAGI1, MALT1, MAP2K3, MAP3K20, MAP3K4, MAP4K4, MARCHF5, MBD3, MCM2, MED30, METTL14, MGAT1, MGMT, MLH1, MMAB, MME, MOCS2, MOGS, MORF4L1, MOV10, MRPL10, MRPS17, MRPS21, MRPS31, MRRF, MSN, MTA1, MTCH1, MVD, MVP, MYADM, MYH9,	

					N6AMT1, NCK2, NCL, NCLN, NCOA7, NDRG1, NDUFA13, NDUFAF7, NEDD4, NEDD8, NEK1, NF1, NF2, NIBAN1, NLE1, NMT1, NNT, NOCT, NOP53, NPLOC4, NPM1, NPR2, NSMCE3, NSUN5, NT5C3B, NUB1, NUDT15, NUDT16, NUP205, NUP35, NUP62, NXN, OBSL1, OGFOD1, OSBPL8, OTUD6B, OXA1L, OXSR1, P3H1, P3H3, PACSIN3, PAIP1, PARG, PARL, PARP1, PARP14, PARP4, PAWR, PDCD6IP, PDK1, PDZRN3, PEBP1, PELO, PGAM5, PGM3, PGP, PHKB, PHPT1, PIGO, PIGT, PIGU, PIK3CA, PIN1, PITHD1, PITRM1, PKM, PKN2, PLD1, PLOD2, PLPP3, PLXNB2, PML, PMM2, POFUT1, POGLUT3, POMGNT1, POMGNT2, POMK, PPIB, PPIE, PPIG, PPM1B, PPP1CA, PPP1R14A, PPP1R7, PPP2R5A, PPP3CA, PPP5C, PPWD1, PRKAA1, PRKAB2, PRKCI, PRKCSH, PRKG1, PRMT7, PRPF19, PSMA3, PSMB10, PSMB2, PSMC2, PSMC3, PSMC6, PSMD1, PSMD12, PSMD14, PSMD2, PSMD6, PSMD7, PSMD8, PSME1, PSME2, PSME4, PTK7, PTPA, PTPMT1, PTPN12, PTPRF, PTPRG, PUS7, PXK, PYCARD, QARS1, QSOX1, RAB11B, RAB1A, RAB3D, RAB5A, RAB8A, RANGAP1, RAP2B, RAP2C, RARS2, RBM4, RCN1, RCN3, RDX, RIF1, RIOK3, RIPK2, RNF114, RNF2, RNF213, RNF214, RNF40, RNGTT, ROR1, RPL10A, RPL13, RPL13A, RPL18A, RPL22, RPL24, RPL27, RPL32, RPL36, RPL38, RPL5, RPLP1, RPLP2, RPRD1A, RPRD1B, RPS13, RPS15A, RPS16, RPS18, RPS21,	

					RPS23, RPS4Y1, RPS5, RPS8, RPS9, RSPRY1, RTN4, RYBP, SAE1, SARNP, SARS1, SCPEP1, SCRN1, SEC11C, SEC61B, SELENOT, SENP3, SERPINB1, SERPINB2, SERPINB8, SERPINB9, SERPINE2, SESN2, SET, SGSH, SHFL, SHMT2, SHPRH, SIRT1, SLC1A1, SLIT2, SMAD4, SMARCAD1, SMARCB1, SMARCC1, SMPD1, SMYD3, SNF8, SNW1, SNX1, SNX5, SOAT1, SOD1, SPHK2, SQSTM1, SRC, SRD5A3, STAT2, STK24, SUFU, TAB2, TAF6L, TBC1D10A, TBCE, TBK1, TF, TGFBI, TGFBR3, TGM2, THBS1, TIPRL, TMEM119, TMX3, TNIK, TPP1, TPP2, TPST2, TPX2, TRADD, TRIM21, TRIM25, TRIM28, TRIM56, TRIM59, TRIO, TRIP12, TRMT10C, TSC2, TSPO, TTLL12, TUT4, TXN, UACA, UBA2, UBA3, UBAC2, UBE2L6, UBE2Q1, UBR2, UBR4, UBXN2A, UFD1, UHRF2, UQCRC2, URI1, URM1, USP11, USP22, USP28, USP4, USP48, USP5, USP7, USP9X, USP9Y, UST, VARS1, VDAC2, VLDLR, VPS25, VPS36, VRK1, VRK3, VTN, WDR35, WDR5, WDR70, WFS1, WIPI2, WWP2, XIAP, XPNPEP3, XYLT2, YARS1, YBEY, YBX1, YEATS4, YES1, YOD1, ZC3HAV1, ZDHHC13, ZDHHC21, ZFP36L2, ZMPSTE24]	
regulation of cellular metabolic process	GO_BiologicalProcess-EBI-UniProt-GOA-ACAP-ARAP_13.05.2021_00h00	3.67E-55	8.763693	560	[AARS1, AASS, ABAT, ABCB7, ABCE1, ABI1, ABT1, ACACA, ACADM, ACADVL, ACO1, ACOT8, ACSL3, ACTB, ACTR2, ACTR3, ADARB1, ADNP, AGO2, AGRN, AK4, AKR1B1, ANKRD13A, ANXA3, APAF1, APBB2, APEX1, APLP2, APP, APPL2, ARAF, ARFGEF1, ARHGEF11, ARID1B,	No change

					ARPP19, ARRB1, ASPSCR1, ATG7, ATP1A1, ATP6V0A1, ATP6V0C, ATP6V1A, ATP6V1B2, BAZ1B, BIN1, BOD1, BRD3, BST1, C1QBP, CAB39L, CAMK2D, CASK, CASP3, CBR1, CBX1, CBX2, CBX3, CBX5, CBX8, CC2D1A, CCDC22, CCN2, CCNH, CCNK, CCNL1, CCT2, CCT3, CCT6A, CD109, CD200, CD81, CDC5L, CDC73, CDK1, CDK16, CDK17, CDK5RAP3, CELF2, CEMIP, CENPV, CHAMP1, CHD2, CHD4, CHD7, CHORDC1, CHP1, CHTOP, CIRBP, CLU, CNOT1, CNOT10, CORO1C, CRABP2, CRTAP, CSPG4, CSTB, CTBP1, CTNNB1, CTSA, CTSC, CUX1, CWC22, CYCS, DAB2, DAGLB, DAPK1, DCN, DCPS, DCXR, DDX1, DENR, DERL1, DHCR24, DHFR, DHX34, DHX36, DICER1, DIMT1, DIPK2A, DNM1L, DNMT1, DUSP3, DVL2, DYNLL1, ECE1, EDF1, EED, EEF1G, EFEMP1, EFL1, EIF2AK2, EIF2B1, EIF2D, EIF2S1, EIF3A, EIF3B, EIF3E, EIF3F, EIF4G3, EIF6, ELP3, ELP4, ENPP1, ENY2, EPHA2, EPHA3, EPHB4, EPM2AIP1, EPRS1, ERBB2, ERLIN2, ETFDH, EZR, FAM3A, FASN, FBXW8, FERMT2, FGF2, FHL2, FHOD1, FKBP1A, FMR1, FN1, FTO, G6PD, GAPDH, GARS1, GATAD2A, GCFC2, GIPC1, GLA, GLE1, GLMN, GLMP, GLO1, GLYR1, GNL3L, GPD1L, GPI, GPSM1, GSK3B, GSTP1, GTF2F1, GTF2I, H1-10, H1-5, HAT1, HDAC2, HDAC4, HDGFL2, HEXB, HGS, HINT1, HIP1, HK2, HMCES, HMOX1, HNRNPA0, HNRNPC, HNRNPH1, HNRNPLL, HNRNPR, HPX, HSD17B4, HSP90AA1, HSPA5, HSPE1, HTATSF1, IGF2BP1, IGF2BP2, IKBKB, IKBKG, ILF3,	

					IMPACT, IPO5, IQGAP3, IREB2, IST1, ITGAV, ITIH4, ITM2C, IVNS1ABP, JAG1, KAT7, KAT8, KDM1A, KDM3B, KDM4B, LANCL2, LARP1, LARP4B, LARP6, LIG3, LIMS1, LLGL1, LMCD1, LMO7, LRPPRC, LRRFIP1, LRRK1, LUM, MACROH2A1, MAD2L1, MAEA, MAGI1, MALT1, MAP2K3, MAP3K20, MAP3K4, MAP4K4, MBD3, MBOAT7, MCM2, MCM7, MED17, MED23, MED30, METTL14, MGA, MGMT, MLF2, MLH1, MMAB, MORF4L1, MOV10, MPHOSPH8, MSH2, MSH6, MSN, MTA1, MTCH1, MVP, MYADM, MYD88, MYH9, MYO6, NCK2, NCL, NCOA5, NCOA7, NDUFA13, NDUFA4, NEDD4, NEDD8, NELFCD, NF1, NF2, NFATC2IP, NFKB2, NIBAN1, NIBAN2, NLE1, NMD3, NNT, NOCT, NOL11, NOP53, NPM1, NQO1, NSMCE3, NSUN5, NT5C3B, NUB1, NUDT15, NUDT16, NUDT16L1, NUP205, NUP35, NUP62, NXN, OGFOD1, ORC5, OSBPL8, OXSR1, P3H1, PACSIN3, PAFAH1B2, PAIP1, PARG, PARL, PARP1, PARP14, PARP4, PATZ1, PAWR, PBXIP1, PCBP3, PDK1, PEBP1, PELO, PFKM, PGP, PIK3C2A, PIK3CA, PIN1, PIP4K2B, PIR, PITHD1, PKM, PKN2, PLD1, PLPP3, PLXNB2, PML, POFUT1, POGZ, POLR2A, POLR2B, POLR2H, POLR2I, POLR3C, PPIB, PPIE, PPP1CA, PPP1R14A, PPP1R7, PPP2R5A, PPP3CA, PPP5C, PRDX6, PRKAA1, PRKAB2, PRKCI, PRKCSH, PRPF19, PSIP1, PSMA3, PSMB10, PSMB2, PSMC2, PSMC3, PSMC6, PSMD1, PSMD12, PSMD14, PSMD2, PSMD6,	

					PSMD7, PSMD8, PSME1, PSME2, PSME4, PSPC1, PTBP2, PTBP3, PTGES2, PTGIS, PTPA, PTRH2, PUF60, PUS7, PXN, PYCARD, QARS1, QSOX1, RAB1A, RAB8A, RAD21, RAP2B, RAP2C, RBM22, RBM4, RCN3, RDH10, RDX, RFC2, RFC3, RFC5, RHEB, RIF1, RIPK2, RNF2, RNF40, RNPS1, ROR1, RPA2, RPL13A, RPL22, RPL38, RPL5, RPLP1, RPRD1A, RPRD1B, RPS13, RPS9, RTN4, RYBP, SAE1, SAP30BP, SARNP, SARS1, SCAI, SCARB2, SCFD1, SCRIB, SEC14L2, SELENOT, SERPINB1, SERPINB2, SERPINB8, SERPINB9, SERPINE2, SESN2, SET, SF1, SH3BP4, SHFL, SHMT2, SIRT1, SLC1A1, SLC25A12, SLC25A15, SLC25A4, SLC25A5, SLC30A9, SLC9A1, SLIT2, SMAD4, SMARCA1, SMARCAD1, SMARCB1, SMARCC1, SMARCD3, SMARCE1, SMC3, SMCHD1, SMPD1, SMU1, SMYD3, SNF8, SNW1, SNX5, SOAT1, SOD1, SOD2, SON, SORBS3, SPHK2, SQSTM1, SRA1, SRC, SRSF10, SRSF2, SRSF5, STAT1, STAT2, STAT5B, STK24, STON1, STRN3, SUFU, SUPT16H, TAB2, TAF6L, TAX1BP1, TBC1D10A, TBK1, TCP1, TDRD3, TF, TFAM, THBS1, TIGAR, TIPRL, TMEM119, TNIK, TPX2, TRA2A, TRA2B, TRADD, TRIM21, TRIM25, TRIM28, TRIM56, TRIP12, TRIP13, TRMT10C, TSC2, TSPO, TTC5, TTLL12, TUT4, TXN, UACA, UBA2, UBA3, UBR2, UBTF, UBXN2A, URI1, USP22, USP4, USP5, USP7, USP9X, VDAC1, VDAC2, VLDLR, VPS25,	

					VPS26B, VRK3, VTN, WDR35, WDR41, WDR5, WDR70, WFS1, WRNIP1, WWC2, WWP2, XIAP, YAP1, YBX1, YEATS4, YES1, YTHDC1, ZC3HAV1, ZFP36L2, ZMPSTE24, ZNF148]	
cellular macromolecule metabolic process	GO_BiologicalProcess-EBI-UniProt-GOA-ACAP-ARAP_13.05.2021_00h00	3.67E-55	9.067327	769	[AARS1, AASDHPPT, AASS, ABCE1, ABHD12, ABI1, ABT1, ACADM, ACO1, ACOT8, ACTB, ACTR2, ACTR3, ADAM10, ADAMTS9, ADARB1, ADNP, AGO2, AGRN, AKR1B1, ALB, ALG11, ANKRD13A, ANKZF1, ANXA3, APAF1, APBB2, APEX1, APLP2, APP, AQR, ARAF, ARFGEF1, ARHGEF11, ARID1B, ARMT1, ARPP19, ARRB1, ASB1, ASH2L, ASPSCR1, ATE1, ATG7, AUP1, B3GAT3, B4GALT1, B4GALT5, BAZ1B, BCR, BIN1, BOD1, BPNT2, BRD3, BST1, C1GALT1, C1QBP, CAB39L, CAMK2D, CASK, CASP3, CBX1, CBX2, CBX3, CBX5, CBX8, CC2D1A, CCDC22, CCN2, CCNH, CCNK, CCNL1, CCT2, CCT3, CCT6A, CD109, CD200, CD81, CDC34, CDC42BPA, CDC5L, CDC73, CDK1, CDK16, CDK17, CDK2, CDK5RAP3, CECR2, CEMIP, CENPV, CETN2, CFAP20, CHAF1B, CHAMP1, CHD1L, CHD2, CHD4, CHD7, CHORDC1, CHP1, CHPF, CHSY3, CHTOP, CIRBP, CLTC, CLU, CMTR1, CNGB1, CNOT1, CNOT10, COG7, COL11A1, COLEC12, CORO1C, CPSF2, CRABP2, CRBN, CRTAP, CSPG4, CSTB, CSTF3, CTBP1, CTNNB1, CTSC, CTU1, CUL1, CUL4B, CUX1, CYCS, DAB2, DAPK1, DARS1, DCN, DCPS, DDI2, DDX1, DENR, DERL1, DGUOK, DHCR24, DHFR, DHPS, DHX34, DHX36, DICER1, DIMT1, DIPK2A,	No change

					DLST, DNM1L, DNMT1, DRG1, DSP, DUSP23, DUSP3, DUT, DVL2, DYNLL1, EARS2, ECE1, EDF1, EED, EEF1E1, EEF1G, EFEMP1, EFL1, EHD3, EIF2AK2, EIF2B1, EIF2D, EIF2S1, EIF3A, EIF3B, EIF3E, EIF3F, EIF4G3, EIF6, ELP3, ELP4, EMG1, ENPP1, ENY2, EOGT, EPHA2, EPHA3, EPHB4, EPM2AIP1, EPRS1, ERBB2, ERCC5, ERLIN2, ERP44, ETFDH, EXD2, EXTL2, EZR, FAM3A, FANCI, FASN, FBXL12, FBXW8, FDXR, FEN1, FERMT2, FGF2, FHL2, FHOD1, FKBP1A, FKBP5, FKBP9, FMR1, FN1, FN3K, FSCN1, FTO, FUT11, FUT8, G6PD, GALNT1, GALNT2, GALNT7, GAN, GAPDH, GARS1, GATAD2A, GATB, GBE1, GCFC2, GFPT2, GIPC1, GLE1, GLMN, GLMP, GLO1, GLUL, GLYR1, GNL3L, GOLGA7, GPC1, GPD1L, GPHN, GPI, GRWD1, GSK3B, GSTP1, GTF2E1, GTF2F1, GTF2F2, GTF2I, GTF3C3, GTF3C4, GXYLT1, GXYLT2, GYS1, H1-10, H1-5, HARS1, HAT1, HDAC2, HDAC4, HDGFL2, HECTD4, HERC2, HERC4, HEXB, HGS, HINT1, HIP1, HLA-A, HMBS, HMCES, HMOX1, HNRNPA0, HNRNPC, HNRNPR, HPX, HSD17B10, HSD17B4, HSP90AA1, HSP90B1, HSPA5, HSPE1, HTATSF1, IARS2, IGF2BP1, IGF2BP2, IGFBP7, IKBKB, IKBKG, ILF3, ILKAP, IMPACT, INTS1, INTS9, IPO5, IQGAP3, IREB2, IST1, ITGB1, ITIH4, ITM2C, IVNS1ABP, JAG1, KAT7, KAT8, KDM1A, KDM3B, KDM4B, KPNB1, LAMC1, LANCL2, LARP1, LARP4B, LARP6, LIG1, LIG3, LIMS1, LLGL1, LMCD1, LMO7, LNPEP, LRPPRC, LRRFIP1,	

					LRRK1, LRWD1, LTBP1, LUM, LYPLA2, MACROD1, MACROH2A1, MAD2L1, MAEA, MAGI1, MALT1, MAP2K3, MAP3K20, MAP3K4, MAP4K4, MARCHF5, MBD3, MCM2, MCM3, MCM4, MCM5, MCM6, MCM7, MCMBP, MDC1, MED17, MED23, MED30, METTL14, MGA, MGAT1, MGMT, MLF2, MLH1, MMAB, MOCS2, MOGS, MORF4L1, MOV10, MPHOSPH8, MRM3, MRPL10, MRPS17, MRPS21, MRPS31, MRRF, MRTO4, MSH2, MSH6, MSN, MTA1, MTCH1, MTREX, MTRR, MVD, MVP, MYADM, MYD88, MYH9, MYO18A, MYO6, N6AMT1, NCK2, NCL, NCOA5, NCOA7, NDRG1, NDUFA13, NDUFAF7, NEDD4, NEDD8, NEK1, NELFCD, NF1, NF2, NFATC2IP, NFKB2, NIBAN1, NIBAN2, NLE1, NMT1, NNT, NOCT, NOL11, NOP53, NPLOC4, NPM1, NPR2, NSMCE3, NSUN5, NT5C3B, NUB1, NUDT15, NUDT16, NUDT16L1, NUP205, NUP35, NUP62, NXN, OBSL1, OGFOD1, ORC4, ORC5, OSBPL8, OTUD6B, OXA1L, OXSR1, P3H1, P3H3, PACSIN3, PAIP1, PARG, PARL, PARP1, PARP14, PARP4, PATZ1, PAWR, PBXIP1, PDCD6IP, PDK1, PDS5B, PDZRN3, PEBP1, PELO, PFKM, PGAM5, PGM2L1, PGM3, PGP, PHKB, PHPT1, PIGO, PIGT, PIGU, PIK3CA, PIN1, PIR, PITHD1, PKM, PKN2, PLD1, PLD3, PLOD2, PLPP3, PLXNB2, PML, PMM2, POFUT1, POGLUT3, POGZ, POLD1, POLD2, POLE2, POLR1C, POLR1D, POLR2A, POLR2B, POLR2H,	

					POLR2I, POLR3A, POLR3C, POMGNT1, POMGNT2, POMK, POP1, PPIB, PPIE, PPIG, PPM1B, PPP1CA, PPP1R14A, PPP1R7, PPP2R5A, PPP3CA, PPP5C, PPWD1, PRIM2, PRKAA1, PRKAB2, PRKCI, PRKCSH, PRKG1, PRMT7, PRPF19, PSIP1, PSMA3, PSMB10, PSMB2, PSMC2, PSMC3, PSMC6, PSMD1, PSMD12, PSMD14, PSMD2, PSMD6, PSMD7, PSMD8, PSME1, PSME2, PSME4, PSPC1, PTGES2, PTGIS, PTK7, PTPA, PTPMT1, PTPN12, PTPRF, PTPRG, PTRH2, PUS7, PXK, PXN, PYCARD, PYGB, QARS1, QSOX1, RAB11B, RAB1A, RAB3D, RAB5A, RAB8A, RAD21, RAD9A, RANGAP1, RAP2B, RAP2C, RARS2, RBBP4, RBM4, RCN1, RCN3, RDX, RECQL, RFC2, RFC3, RFC5, RIF1, RIOK3, RIPK2, RNF114, RNF2, RNF213, RNF214, RNF40, RNGTT, RNPS1, ROR1, RPA1, RPA2, RPL10A, RPL13, RPL13A, RPL18A, RPL22, RPL24, RPL27, RPL32, RPL36, RPL38, RPL5, RPLP1, RPLP2, RPRD1A, RPRD1B, RPRD2, RPS13, RPS15A, RPS16, RPS18, RPS21, RPS23, RPS4Y1, RPS5, RPS8, RPS9, RRM1, RSPRY1, RYBP, SAE1, SAMHD1, SAP30BP, SARNP, SARS1, SCAI, SCRIB, SEC11C, SEC14L2, SEC61B, SELENOT, SENP3, SERPINB1, SERPINB2, SERPINB8, SERPINB9, SERPINE2, SESN2, SET, SF1, SGSH, SHFL, SHMT2, SHPRH, SIRT1, SLC1A1, SLC25A15, SLC30A9, SLC9A1, SLIT2, SMAD4, SMARCA1, SMARCAD1, SMARCB1,	

					SMARCC1, SMARCD3, SMARCE1, SMC1A, SMC3, SMCHD1, SMPD1, SMYD3, SNF8, SNRPD3, SNRPE, SNW1, SNX1, SNX5, SOAT1, SOD1, SOD2, SORBS3, SPHK2, SQSTM1, SRA1, SRC, SRD5A3, SRSF10, SRSF2, SSB, SSRP1, STAT1, STAT2, STAT5B, STK24, STON1, STRN3, SUFU, SUPT16H, TAB2, TAF6L, TAX1BP1, TBC1D10A, TBCE, TBK1, TBPL1, TCP1, TDRD3, TF, TFAM, TGFBR3, TGM2, TGS1, THADA, THBS1, TIGAR, TIPRL, TMEM119, TMX3, TNIK, TPP1, TPP2, TPST2, TPX2, TRADD, TRIM21, TRIM25, TRIM28, TRIM56, TRIM59, TRIO, TRIP12, TRIP13, TRMT10C, TRMT2A, TRMT5, TSC2, TSPO, TTC5, TTLL12, TUT4, TXN, UACA, UBA2, UBA3, UBAC2, UBE2L6, UBE2Q1, UBR2, UBR4, UBTF, UBXN2A, UFD1, UGP2, UHRF2, URI1, URM1, USP11, USP22, USP28, USP4, USP48, USP5, USP7, USP9X, USP9Y, UST, VARS1, VDAC2, VLDLR, VPS25, VPS36, VRK1, VRK3, VTN, WDR35, WDR5, WDR70, WFS1, WIPI2, WRNIP1, WWC2, WWP2, XIAP, XYLT2, YAP1, YARS1, YBX1, YEATS4, YES1, YOD1, ZC3HAV1, ZDHHC13, ZDHHC21, ZFP36L2, ZMPSTE24, ZNF148]	
regulation of macromolecule metabolic process	GO_BiologicalProcess-EBI-UniProt-GOA-ACAP-ARAP_13.05.2021_00h00	3.67E-55	8.834672	583	[AARS1, AASS, ABCE1, ABI1, ABT1, ACO1, ACOT8, ACTB, ACTG1, ACTR2, ACTR3, ADAM10, ADARB1, ADNP, AFDN, AGO2, AGPAT2, AGRN, AKAP12, AKR1B1, ANK3, ANKRD13A, ANXA3, ANXA7, APAF1, APBB2, APEX1, APLP2, APP, APPL2, ARAF, ARFGEF1, ARHGEF11, ARID1B, ARPP19, ARRB1, ASB1, ASPSCR1,	No change

					ATG4B, ATG7, ATP2B1, B2M, BAZ1B, BIN1, BOD1, BRD3, BST1, C1QBP, CAB39L, CAMK2D, CASK, CASP3, CBX1, CBX2, CBX3, CBX5, CBX8, CC2D1A, CCDC22, CCN2, CCNH, CCNK, CCNL1, CCT2, CCT3, CCT6A, CD109, CD200, CD276, CD81, CDC5L, CDC73, CDK1, CDK16, CDK17, CDK2, CDK5RAP3, CELF2, CEMIP, CENPV, CHAMP1, CHD2, CHD4, CHD7, CHID1, CHORDC1, CHP1, CHTOP, CIRBP, CLTC, CLU, CNOT1, CNOT10, COLEC12, CORO1C, CRABP2, CRTAP, CSPG4, CSTB, CTBP1, CTNNB1, CTSA, CTSC, CUX1, CWC22, CYCS, CYP51A1, CYRIB, DAB2, DAPK1, DCPS, DDX1, DENR, DERL1, DHCR24, DHFR, DHX34, DHX36, DICER1, DIMT1, DIPK2A, DNM1L, DNMT1, DUSP3, DVL2, DYNLL1, ECE1, EDF1, EED, EEF1G, EFEMP1, EFL1, EIF2AK2, EIF2B1, EIF2D, EIF2S1, EIF3A, EIF3B, EIF3E, EIF3F, EIF4G3, EIF6, ELP3, ELP4, ENPP1, ENY2, EPHA2, EPM2AIP1, EPRS1, ERBB2, ETFDH, EZR, FASN, FBXW8, FERMT1, FERMT2, FGF2, FHL2, FHOD1, FKBP1A, FMR1, FN1, FTO, FUT8, G6PD, GAPDH, GARS1, GATAD2A, GBP1, GCFC2, GIPC1, GLE1, GLMN, GLMP, GLO1, GLYR1, GNL3L, GPD1L, GPI, GSDMD, GSK3B, GSTP1, GTF2F1, GTF2I, H1-10, H1-5, HAT1, HDAC2, HDAC4, HDGFL2, HELZ, HEXB, HGS, HINT1, HIP1, HK1, HLA-A, HLA-B, HMCES, HMOX1, HNRNPA0, HNRNPC, HNRNPH1, HNRNPLL, HNRNPR, HPX, HSD17B4, HSP90AA1, HSPA5, HSPE1, HTATSF1, IGF2BP1, IGF2BP2, IKBKB, IKBKG, ILF3,	

					IMPACT, IPO5, IQGAP3, IREB2, IST1, ITGA3, ITGAV, ITIH4, ITM2C, IVNS1ABP, JAG1, KAT7, KAT8, KCNQ1, KDM1A, KDM3B, KDM4B, LANCL2, LARP1, LARP4B, LARP6, LIG3, LIMS1, LLGL1, LMCD1, LMO7, LRPPRC, LRRC32, LRRFIP1, LRRK1, LTBP1, LUM, MACROH2A1, MAD2L1, MAGI1, MALT1, MAP2K3, MAP3K20, MAP3K4, MAP4K4, MATR3, MBD3, MCM2, MED17, MED23, MED30, METTL14, MGA, MGMT, MLF2, MLH1, MMAB, MORF4L1, MOV10, MPHOSPH8, MRTO4, MSH2, MSH6, MSN, MTA1, MTCH1, MVP, MYADM, MYD88, MYH9, MYO1E, MYO6, NCK2, NCL, NCOA5, NCOA7, NDUFA13, NEDD4, NEDD8, NELFCD, NF1, NF2, NFATC2IP, NFKB2, NIBAN1, NIBAN2, NLE1, NMD3, NNT, NOCT, NOL11, NOP53, NPLOC4, NPM1, NSMCE3, NSUN5, NT5C3B, NUB1, NUDT15, NUDT16, NUDT16L1, NUP205, NUP35, NUP62, NUTF2, NXN, OGFOD1, ORC5, OSBPL8, OXSR1, P3H1, PACSIN3, PAIP1, PANX1, PARG, PARL, PARP1, PARP14, PARP4, PATZ1, PAWR, PBXIP1, PC, PCBP3, PEBP1, PELO, PFKM, PHGDH, PIK3CA, PIN1, PIR, PITHD1, PKM, PKN2, PLD1, PLD3, PLPP3, PLXNB2, PML, PNP, POFUT1, POGZ, POLR1C, POLR1D, POLR2A, POLR2B, POLR2H, POLR2I, POLR3A, POLR3C, PPIB, PPIE, PPM1B, PPP1CA, PPP1R14A, PPP1R7, PPP2R5A, PPP3CA, PPP5C, PRDX6, PRKAA1, PRKCI, PRKCSH, PRMT7, PRPF19, PSIP1, PSMA3, PSMB10, PSMB2, PSMC2, PSMC3,	

					PSMC6, PSMD1, PSMD12, PSMD14, PSMD2, PSMD6, PSMD7, PSMD8, PSME1, PSME2, PSME4, PSPC1, PTBP2, PTBP3, PTGES2, PTGIS, PTPA, PTRH2, PUF60, PUS7, PXN, PYCARD, QARS1, RAB1A, RAD21, RAP2B, RAP2C, RBBP4, RBM22, RBM4, RCN3, RDX, RFC2, RFC3, RFC5, RIF1, RIOK3, RIPK2, RNF2, RNF40, RNPS1, ROR1, RPA2, RPL10A, RPL13, RPL13A, RPL18A, RPL22, RPL24, RPL27, RPL32, RPL36, RPL38, RPL5, RPLP1, RPLP2, RPRD1A, RPRD1B, RPS13, RPS15A, RPS16, RPS18, RPS21, RPS23, RPS4Y1, RPS5, RPS8, RPS9, RTN4, RYBP, SAE1, SAP30BP, SARNP, SARS1, SCAI, SCARB2, SCRIB, SEC14L2, SELENOT, SERPINB1, SERPINB2, SERPINB8, SERPINB9, SERPINE2, SESN2, SET, SF1, SHFL, SHMT2, SIRT1, SLC1A1, SLC25A15, SLC30A9, SLC9A1, SLIT2, SMAD4, SMARCA1, SMARCAD1, SMARCB1, SMARCC1, SMARCD3, SMARCE1, SMC3, SMCHD1, SMPD1, SMU1, SMYD3, SNF8, SNW1, SNX1, SNX5, SOAT1, SOD1, SOD2, SON, SORBS3, SORT1, SPHK2, SQSTM1, SRA1, SRC, SRSF10, SRSF2, SRSF5, SSB, STAT1, STAT2, STAT5B, STK24, STON1, STRN3, SUFU, SUPT16H, TAB2, TAF6L, TAX1BP1, TBC1D10A, TBK1, TCP1, TDRD3, TF, TFAM, THBS1, TIGAR, TIPRL, TMEM119, TNIK, TPX2, TRA2A, TRA2B, TRADD, TRIM21, TRIM25, TRIM28, TRIM56, TRIP12, TRIP13,	

					TRMT10C, TSC2, TSPO, TTC5, TTLL12, TUT4, TXN, UACA, UBA2, UBA3, UBE2L6, UBR2, UBTF, UBXN2A, UFD1, URI1, USP22, USP4, USP5, USP7, USP9X, VDAC2, VLDLR, VPS25, VRK3, VTN, WDR35, WDR5, WDR70, WFS1, WRNIP1, WWC2, WWP2, XIAP, YAP1, YBX1, YEATS4, YES1, YTHDC1, ZC3HAV1, ZCCHC3, ZFP36L2, ZMPSTE24, ZNF148]	
regulation of primary metabolic process	GO_BiologicalProcess-EBI-UniProt-GOA-ACAP-ARAP_13.05.2021_00h00	3.67E-55	8.756879	541	[AARS1, AASS, ABAT, ABCE1, ABI1, ABT1, ACACA, ACADM, ACADVL, ACO1, ACOT8, ACSL3, ACTB, ACTR2, ACTR3, ADARB1, ADNP, AGO2, AGRN, AK4, AKR1B1, ANXA3, APAF1, APBB2, APEX1, APLP2, APP, APPL2, ARAF, ARFGEF1, ARHGEF11, ARID1B, ARPP19, ARRB1, ASAH1, ASPSCR1, ATG4B, ATG7, ATP1A1, BAZ1B, BIN1, BOD1, BRD3, BST1, C1QBP, CAB39L, CAMK2D, CASK, CASP3, CBX1, CBX2, CBX3, CBX5, CBX8, CC2D1A, CCDC22, CCN2, CCNH, CCNK, CCNL1, CCT2, CCT3, CCT6A, CD109, CD200, CD81, CDC5L, CDC73, CDK1, CDK16, CDK17, CDK5RAP3, CELF2, CEMIP, CENPV, CHAMP1, CHD2, CHD4, CHD7, CHORDC1, CHP1, CHTOP, CIRBP, CLTC, CLU, CNOT1, CNOT10, CORO1C, CRABP2, CRTAP, CSPG4, CSTB, CTBP1, CTNNB1, CTSA, CTSC, CUX1, CWC22, CYCS, CYP51A1, DAB2, DAGLB, DAPK1, DCPS, DDX1, DENR, DERL1, DHCR24, DHCR7, DHFR, DHX34, DHX36, DICER1, DIMT1, DIPK2A, DNMT1, DUSP3, DVL2, DYNLL1, EDF1, EED, EEF1G, EFEMP1, EFL1, EIF2AK2, EIF2B1, EIF2D, EIF2S1, EIF3A,	Upregulation

					EIF3B, EIF3E, EIF3F, EIF4G3, EIF6, ELP3, ELP4, ENPP1, ENY2, EPM2AIP1, EPRS1, ERBB2, ERLIN2, ETFDH, EZR, FAM3A, FASN, FBXW8, FERMT2, FGF2, FHL2, FHOD1, FKBP1A, FMR1, FN1, FTO, G6PD, GAPDH, GARS1, GATAD2A, GCFC2, GIPC1, GLE1, GLMN, GLMP, GLO1, GLYR1, GNL3L, GPD1L, GPI, GSK3B, GSTP1, GTF2F1, GTF2I, H1-10, H1-5, HAT1, HDAC2, HDAC4, HDGFL2, HEXB, HGS, HINT1, HIP1, HMCES, HMGCS1, HMOX1, HNRNPA0, HNRNPC, HNRNPH1, HNRNPLL, HNRNPR, HPX, HSD17B4, HSP90AA1, HSPA5, HSPE1, HTATSF1, IDI1, IGF2BP1, IGF2BP2, IGFBP7, IKBKB, IKBKG, ILF3, IMPACT, IPO5, IQGAP3, IREB2, IST1, ITGAV, ITIH4, ITM2C, IVNS1ABP, JAG1, KAT7, KAT8, KDM1A, KDM3B, KDM4B, KPNB1, LACTB, LANCL2, LARP1, LARP4B, LARP6, LIMS1, LLGL1, LMCD1, LMO7, LRPPRC, LRRFIP1, LRRK1, LSS, LUM, MACROH2A1, MAD2L1, MAEA, MAGI1, MALT1, MAP2K3, MAP3K20, MAP3K4, MAP4K4, MBD3, MBOAT7, MCM2, MED17, MED23, MED30, METTL14, MGA, MGMT, MLF2, MLH1, MMAB, MORF4L1, MOV10, MPHOSPH8, MSH2, MSH6, MSN, MTA1, MTCH1, MVD, MVK, MVP, MYADM, MYD88, MYH9, MYO6, NCK2, NCL, NCOA5, NCOA7, NDUFA13, NEDD4, NEDD8, NELFCD, NF1, NF2, NFATC2IP, NFKB2, NIBAN1, NIBAN2, NLE1, NMD3, NNT, NOCT, NOL11, NOP53, NPM1, NQO1, NSMCE3, NSUN5, NT5C3B, NUB1, NUDT15, NUDT16,	

					NUDT16L1, NUP205, NUP35, NUP62, NXN, OGFOD1, OSBPL8, OXSR1, P3H1, PACSIN3, PAIP1, PARG, PARL, PARP1, PARP14, PARP4, PATZ1, PAWR, PBXIP1, PCBP3, PDK1, PEBP1, PELO, PFKM, PGP, PIK3CA, PIN1, PIP4K2B, PIR, PITHD1, PKM, PKN2, PLD1, PLPP3, PLXNB2, PML, POFUT1, POGZ, POLR2A, POLR2B, POLR2H, POLR2I, POLR3C, PPIB, PPIE, PPP1CA, PPP1R14A, PPP1R7, PPP2R5A, PPP3CA, PPP5C, PRDX6, PRKAA1, PRKAB2, PRKCI, PRKCSH, PRPF19, PSIP1, PSMA3, PSMB10, PSMB2, PSMC2, PSMC3, PSMC6, PSMD1, PSMD12, PSMD14, PSMD2, PSMD6, PSMD7, PSMD8, PSME1, PSME2, PSME4, PSPC1, PTBP2, PTBP3, PTGES2, PTGIS, PTPA, PTRH2, PUF60, PUS7, PXN, PYCARD, QARS1, RAB1A, RAD21, RAP2B, RAP2C, RBM22, RBM4, RCN3, RDH10, RDX, RFC2, RFC3, RFC5, RIF1, RIPK2, RNF2, RNF40, RNPS1, ROR1, RPA2, RPL13A, RPL22, RPL38, RPL5, RPLP1, RPRD1A, RPRD1B, RPS13, RPS9, RTN4, RYBP, SAE1, SAP30BP, SARNP, SARS1, SCAI, SCARB2, SEC14L2, SELENOT, SERPINB1, SERPINB2, SERPINB8, SERPINB9, SERPINE2, SESN2, SET, SF1, SHFL, SHMT2, SIRT1, SLC1A1, SLC25A12, SLC25A15, SLC30A9, SLC9A1, SLIT2, SMAD4, SMARCA1, SMARCAD1, SMARCB1, SMARCC1, SMARCD3, SMARCE1, SMCHD1, SMPD1, SMU1, SMYD3, SNF8, SNW1, SNX1, SNX5,	

					SOAT1, SOD1, SOD2, SON, SORBS3, SPHK2, SQSTM1, SRA1, SRC, SRSF10, SRSF2, SRSF5, STAT1, STAT2, STAT5B, STK24, STON1, STRN3, SUFU, SUPT16H, TAB2, TAF6L, TAX1BP1, TBC1D10A, TBK1, TCP1, TDRD3, TF, TFAM, TGS1, THBS1, TIGAR, TIPRL, TMEM119, TNIK, TPX2, TRA2A, TRA2B, TRADD, TRIM21, TRIM25, TRIM28, TRIM56, TRIP12, TRIP13, TRMT10C, TSC2, TSPO, TTC39B, TTC5, TTLL12, TUT4, TXN, UACA, UBA2, UBA3, UBR2, UBTF, UBXN2A, URI1, USP22, USP4, USP5, USP7, USP9X, VDAC2, VLDLR, VPS25, VRK3, VTN, WDR35, WDR5, WDR70, WFS1, WRNIP1, WWC2, WWP2, XIAP, YAP1, YBX1, YEATS4, YES1, YTHDC1, ZC3HAV1, ZFP36L2, ZMPSTE24, ZNF148]	
macromolecule biosynthetic process	GO_BiologicalProcess-EBI-UniProt-GOA-ACAP-ARAP_13.05.2021_00h00	3.67E-55	9.576923	498	[AARS1, AASS, ABCE1, ABT1, ACADM, ACO1, ACOT8, ACTR2, ACTR3, ADNP, AGO2, AGRN, AKAP12, AKR1B1, ALG11, ANXA3, APBB2, APEX1, APP, ARFGEF1, ARHGEF11, ARID1B, ARRB1, ATG7, B3GAT3, B4GALT1, B4GALT5, BAZ1B, BRD3, C1GALT1, C1QBP, CAMK2D, CASK, CBX1, CBX2, CBX3, CBX5, CBX8, CC2D1A, CCDC22, CCN2, CCNH, CCNK, CCNL1, CCT2, CCT3, CCT6A, CD200, CD81, CDC34, CDC5L, CDC73, CDK1, CDK16, CDK17, CDK2, CDK5RAP3, CEMIP, CENPV, CHAF1B, CHD2, CHD4, CHD7, CHORDC1, CHP1, CHPF, CHSY3, CIRBP, CLTC, CLU, CNGB1, CNOT1, CNOT10, COG7, COL5A1, COLEC12, CPSF2, CRABP2, CSPG4, CSTF3, CTBP1,	No change

					CTNNB1, CUX1, DAB2, DAPK1, DARS1, DCN, DCPS, DDX1, DENR, DHFR, DHPS, DHX36, DICER1, DNMT1, DRG1, DUT, DVL2, EARS2, EDF1, EED, EEF1E1, EEF1G, EFEMP1, EFL1, EIF2AK2, EIF2B1, EIF2D, EIF2S1, EIF3A, EIF3B, EIF3E, EIF3F, EIF4G3, EIF6, ELP3, ELP4, ENPP1, ENY2, EOGT, EPM2AIP1, EPRS1, ERBB2, EXD2, EXTL2, EZR, FEN1, FGF2, FHL2, FHOD1, FMR1, FTO, FUT11, FUT8, GALNT1, GALNT2, GALNT7, GAPDH, GARS1, GATAD2A, GATB, GBE1, GCFC2, GFPT2, GLE1, GLMP, GLO1, GLUL, GLYR1, GNL3L, GOLGA7, GPC1, GPC6, GPI, GRWD1, GSK3B, GSTP1, GTF2E1, GTF2F1, GTF2F2, GTF2I, GTF3C3, GTF3C4, GXYLT1, GXYLT2, GYS1, H1-10, H1-5, HARS1, HAT1, HDAC2, HDAC4, HDGFL2, HEXB, HINT1, HMOX1, HNRNPC, HNRNPR, HSD17B4, HSP90AA1, HSPA5, HSPG2, HTATSF1, IARS2, IGF2BP1, IGF2BP2, IKBKB, IKBKG, ILF3, IMPACT, INTS1, INTS9, IREB2, ITM2C, IVNS1ABP, JAG1, KAT7, KAT8, KDM1A, KDM3B, LANCL2, LARP1, LARP4B, LARP6, LIG1, LIG3, LIMS1, LMCD1, LMO7, LRPPRC, LRRFIP1, LRWD1, LUM, MACROH2A1, MAGI1, MALT1, MAP2K3, MAP3K4, MBD3, MCM2, MCM3, MCM4, MCM5, MCM6, MCM7, MCMBP, MED17, MED23, MED30, METTL14, MGA, MGAT1, MLF2, MLH1, MMAB, MOGS, MORF4L1, MOV10, MPHOSPH8, MRPL10, MRPS17, MRPS21, MRPS31, MRRF, MTA1, MVD, MYD88, MYO6, N6AMT1, NCK2,	

					NCL, NCOA5, NCOA7, NDRG1, NDUFA13, NEDD4, NEDD8, NELFCD, NFATC2IP, NFKB2, NIBAN1, NIBAN2, NMD3, NMT1, NOCT, NOL11, NOP53, NPLOC4, NPM1, NSUN5, NT5C3B, NUP35, NUP62, OGFOD1, ORC4, ORC5, OXA1L, OXSR1, PAIP1, PARP1, PARP14, PARP4, PATZ1, PAWR, PBXIP1, PELO, PFKM, PGM2L1, PGM3, PIGO, PIGT, PIGU, PIN1, PIR, PITHD1, PKM, PLD1, PLOD2, PLPP3, PLXNB2, PML, PMM2, POFUT1, POGLUT3, POGZ, POLD1, POLD2, POLE2, POLR1C, POLR1D, POLR2A, POLR2B, POLR2H, POLR2I, POLR3A, POLR3C, POMGNT1, POMGNT2, POMK, PPIE, PPM1B, PPP1CA, PPP3CA, PPP5C, PRIM2, PRKAA1, PRKCI, PRKCSH, PSIP1, PSMA3, PSMB10, PSMB2, PSMC2, PSMC3, PSMC6, PSMD1, PSMD12, PSMD14, PSMD2, PSMD6, PSMD7, PSMD8, PSME1, PSME2, PSME4, PSPC1, PTGES2, PTGIS, PTRH2, PUS7, PXN, PYCARD, QARS1, RAD21, RAD9A, RAP2C, RARS2, RBBP4, RBM4, RECQL, RFC2, RFC3, RFC5, RIF1, RIPK2, RNF2, RNGTT, RNPS1, ROR1, RPA1, RPA2, RPL10A, RPL13, RPL13A, RPL18A, RPL22, RPL24, RPL27, RPL32, RPL36, RPL38, RPL5, RPLP1, RPLP2, RPRD1A, RPRD1B, RPRD2, RPS13, RPS15A, RPS16, RPS18, RPS21, RPS23, RPS4Y1, RPS5, RPS8, RPS9, RRM1, RYBP, SAMHD1, SAP30BP, SARNP, SARS1, SCAI, SEC14L2, SELENOT, SESN2, SET, SF1, SHFL, SHMT2, SIRT1, SLC25A15, SLC30A9,	

					SLC9A1, SMAD4, SMARCA1, SMARCB1, SMARCC1, SMARCD3, SMARCE1, SMC3, SMCHD1, SMYD3, SNF8, SNRPD3, SNRPE, SNW1, SNX5, SOAT1, SOD2, SORBS3, SPHK2, SQSTM1, SRA1, SRC, SRD5A3, SRSF10, SRSF2, SSRP1, STAT1, STAT2, STAT5B, STON1, STRN3, SUFU, SUPT16H, TAB2, TAF6L, TAX1BP1, TBCE, TBK1, TBPL1, TCP1, TDRD3, TF, TFAM, THBS1, TRADD, TRIM21, TRIM25, TRIM28, TRIM56, TRIP13, TRMT10C, TSPO, TTC5, TUT4, TXN, UBA2, UBA3, UBE2L6, UBR2, UBTF, UFD1, UGP2, URI1, USP22, USP7, USP9X, UST, VARS1, WFS1, WIPI2, WRNIP1, WWC2, WWP2, XYLT2, YAP1, YARS1, YBX1, YEATS4, YES1, ZDHHC13, ZDHHC21, ZFP36L2, ZMPSTE24, ZNF148]	
gene expression	GO_BiologicalProcess-EBI-UniProt-GOA-ACAP-ARAP_13.05.2021_00h00	3.67E-55	9.1550455	558	[AAR2, AARS1, AASS, ABCE1, ABT1, ACO1, ACTB, ACTG1, ACTR2, ACTR3, ADAM10, ADARB1, ADNP, AFDN, AGO2, AGPAT2, AGRN, AKAP12, AKR1B1, ANK3, ANXA3, ANXA7, APBB2, APEX1, APLP2, APP, APPL2, AQR, ARHGEF11, ARID1B, ARRB1, ASB1, ATP2B1, B2M, BAZ1B, BCAS2, BMS1, BRD3, C1QBP, CAMK2D, CASK, CASP3, CBX1, CBX2, CBX3, CBX5, CBX8, CC2D1A, CCDC22, CCN2, CCNH, CCNK, CCNL1, CD200, CD276, CD81, CDC5L, CDC73, CDK1, CDK16, CDK17, CDK2, CDK5RAP3, CELF2, CENPV, CHD2, CHD4, CHD7, CHID1, CHORDC1, CHP1, CHTOP, CIRBP, CLU, CMTR1, CNGB1, CNOT1, CNOT10, COIL, COLEC12, CPD,	No change

					CPSF2, CRABP2, CSTF3, CTBP1, CTNNB1, CTNNBL1, CTU1, CUX1, CWC22, CYRIB, DAB2, DAPK1, DARS1, DCPS, DDI2, DDX1, DDX42, DDX47, DENR, DHCR24, DHFR, DHPS, DHX34, DHX36, DICER1, DIMT1, DNM1L, DNMT1, DRG1, DVL2, EARS2, ECE1, EDF1, EED, EEF1E1, EEF1G, EFEMP1, EFL1, EIF2AK2, EIF2B1, EIF2D, EIF2S1, EIF3A, EIF3B, EIF3E, EIF3F, EIF4G3, EIF6, ELP3, ELP4, ELP5, EMG1, ENPP1, ENY2, EPHA2, EPRS1, ERBB2, EZR, FERMT1, FGF2, FHL2, FHOD1, FKBP1A, FMR1, FN1, FTO, FUT8, GALNT2, GAPDH, GARS1, GATAD2A, GATB, GBP1, GCFC2, GEMIN4, GLE1, GLMN, GLMP, GLO1, GLRX3, GLYR1, GPI, GPKOW, GSDMD, GSK3B, GSTP1, GTF2E1, GTF2F1, GTF2F2, GTF2I, GTF3C3, GTF3C4, H1-10, H1-5, HARS1, HAT1, HDAC2, HDAC4, HDGFL2, HELZ, HEXB, HGS, HINT1, HK1, HLA-A, HLA-B, HMCES, HMOX1, HNRNPA0, HNRNPA3, HNRNPC, HNRNPH1, HNRNPLL, HNRNPR, HNRNPUL1, HPX, HSD17B10, HSD17B4, HSP90AA1, HSPA5, HTATSF1, IARS2, IGF2BP1, IGF2BP2, IKBKB, IKBKG, ILF3, IMPACT, INTS1, INTS9, IQGAP3, IREB2, ITGA3, ITGAV, IVNS1ABP, JAG1, KAT7, KAT8, KCNQ1, KDM1A, KDM3B, LANCL2, LARP1, LARP4B, LARP6, LIMS1, LMCD1, LMO7, LRPPRC, LRRC32, LRRFIP1, LTBP1, LUM, MACROH2A1, MAGI1, MALT1, MAP2K3, MAP3K4, MATR3, MBD3, MCM2, MED17, MED23, MED30, METTL14, MGA,	

					MLF2, MLH1, MME, MORF4L1, MOV10, MPHOSPH8, MRM3, MRPL10, MRPS17, MRPS21, MRPS31, MRRF, MRTO4, MSH2, MSH6, MSN, MTA1, MTREX, MYADM, MYD88, MYH9, MYO1E, MYO6, N6AMT1, NCK2, NCL, NCOA5, NCOA7, NDRG1, NDUFA13, NEDD4, NEDD8, NELFCD, NFATC2IP, NFKB2, NIBAN1, NIBAN2, NOCT, NOL11, NOP53, NPLOC4, NPM1, NSUN5, NT5C3B, NUDT16, NUDT16L1, NUP205, NUP35, NUP62, NUTF2, OGFOD1, OXA1L, OXSR1, PAIP1, PANX1, PARL, PARP1, PARP14, PATZ1, PAWR, PBXIP1, PC, PCBP3, PELO, PFKM, PHGDH, PIK3CA, PIN1, PIR, PITHD1, PITRM1, PKM, PLD1, PLD3, PLPP3, PLXNB2, PML, PNP, POFUT1, POGZ, POLR1C, POLR1D, POLR2A, POLR2B, POLR2H, POLR2I, POLR3A, POLR3C, POP1, PPIE, PPIG, PPM1B, PPP1CA, PPP3CA, PPP5C, PPWD1, PRDX6, PRKAA1, PRKCI, PRKCSH, PRMT7, PRPF19, PSIP1, PSMA3, PSMB10, PSMB2, PSMC2, PSMC3, PSMC6, PSMD1, PSMD12, PSMD14, PSMD2, PSMD6, PSMD7, PSMD8, PSME1, PSME2, PSME4, PSPC1, PTBP2, PTBP3, PTGES2, PTGIS, PTRH2, PUF60, PUS7, PXN, PYCARD, QARS1, QTRT1, QTRT2, RAB1A, RAD21, RAP2C, RARS2, RBBP4, RBM22, RBM26, RBM4, RBMS2, RDX, RIF1, RIOK3, RIPK2, RNF2, RNF40, RNGTT, RNPS1, ROR1, RPF2, RPL10A, RPL13, RPL13A, RPL18A, RPL22, RPL24, RPL27, RPL32, RPL36, RPL38, RPL5, RPLP1, RPLP2,	

					RPRD1A, RPRD1B, RPRD2, RPS13, RPS15A, RPS16, RPS18, RPS21, RPS23, RPS4Y1, RPS5, RPS8, RPS9, RYBP, SAMHD1, SAP30BP, SARNP, SARS1, SBDS, SCAF11, SCAI, SCRIB, SEC11C, SEC14L2, SELENOT, SENP3, SERPINB1, SERPINB9, SERPINE2, SESN2, SET, SF1, SF3A3, SF3B6, SHFL, SHMT2, SIRT1, SLC1A1, SLC25A15, SLC30A9, SLC9A1, SMAD4, SMARCA1, SMARCB1, SMARCC1, SMARCD3, SMARCE1, SMCHD1, SMNDC1, SMU1, SMYD3, SNF8, SNRNP200, SNRPA, SNRPD3, SNRPE, SNU13, SNW1, SNX5, SOD1, SOD2, SON, SORBS3, SORT1, SPHK2, SQSTM1, SRA1, SRC, SRSF10, SRSF2, SRSF5, SSB, SSRP1, STAT1, STAT2, STAT5B, STRN3, SUFU, SUPT16H, TAB2, TAF6L, TAX1BP1, TBC1D10A, TBCE, TBK1, TBPL1, TDRD3, TF, TFAM, TGS1, THADA, THBS1, THOC2, TMEM119, TRA2A, TRA2B, TRADD, TRIM21, TRIM25, TRIM28, TRIM56, TRIP13, TRMT10C, TRMT2A, TRMT5, TSEN15, TSEN34, TSPO, TSR2, TTC5, TUT4, TXN, U2SURP, UBA2, UBA3, UBE2L6, UBR2, UBTF, UFD1, URI1, URM1, USP22, USP4, USP7, USP9X, UTP14A, UTP20, UTP3, VARS1, WDR12, WDR35, WFS1, WWC2, WWP2, XIAP, YAP1, YARS1, YBEY, YBX1, YEATS4, YES1, YTHDC1, ZC3HAV1, ZCCHC3, ZFP36L2, ZMPSTE24, ZNF148]	
negative regulation of macromolecule metabolic process	GO_BiologicalProcess-EBI-UniProt-GOA-ACAP-ARAP_13.05.2021_00h00	3.67E-55	10.31491	321	[AASS, ABCE1, ACOT8, ADAM10, ADARB1, ADNP, AGO2, AKR1B1, ANKRD13A, ANXA7, APEX1, APLP2, APP,	No change

					APPL2, ARPP19, ARRB1, ASB1, ATG7, ATP2B1, BIN1, BOD1, C1QBP, CASP3, CBX1, CBX2, CBX3, CBX5, CBX8, CC2D1A, CCN2, CD109, CD200, CDC73, CDK1, CDK2, CDK5RAP3, CENPV, CHID1, CHORDC1, CHP1, CIRBP, CLTC, CLU, CNOT1, CNOT10, COLEC12, CORO1C, CRTAP, CSTB, CTBP1, CTNNB1, CTSA, CUX1, CYP51A1, DAB2, DAPK1, DCPS, DHCR24, DHFR, DHX34, DHX36, DICER1, DNMT1, DUSP3, EED, EIF2AK2, EIF2S1, EIF3E, EIF6, ENPP1, EPHA2, EPRS1, EZR, FERMT1, FGF2, FHL2, FKBP1A, FMR1, FN1, FTO, G6PD, GAPDH, GARS1, GATAD2A, GBP1, GCFC2, GIPC1, GLMN, GNL3L, GPD1L, GPI, GSK3B, GSTP1, H1-10, H1-5, HAT1, HDAC2, HDAC4, HELZ, HMOX1, HNRNPA0, HNRNPC, HNRNPR, IGF2BP1, IGF2BP2, IKBKB, ILF3, IMPACT, IPO5, IQGAP3, IREB2, ITGAV, ITIH4, ITM2C, IVNS1ABP, KAT7, KAT8, KCNQ1, KDM1A, KDM4B, LANCL2, LARP1, LARP4B, LIG3, LIMS1, LLGL1, LMCD1, LRPPRC, LRRC32, LRRFIP1, LRRK1, MACROH2A1, MAD2L1, MAGI1, MBD3, METTL14, MLH1, MMAB, MORF4L1, MOV10, MPHOSPH8, MRTO4, MSH2, MSH6, MTA1, MVP, MYADM, MYD88, NCK2, NCL, NCOA5, NCOA7, NDUFA13, NEDD4, NELFCD, NF1, NF2, NIBAN1, NIBAN2, NLE1, NNT, NOCT, NOP53, NPLOC4, NPM1, NT5C3B, NUDT16, NUDT16L1, NUP205, NUP35, NUP62, NUTF2, NXN, OXSR1, P3H1, PAIP1, PARP1, PARP14, PATZ1, PAWR, PBXIP1, PC, PEBP1,	

					PELO, PIN1, PKN2, PLPP3, PML, POLR2A, POLR2B, POLR2H, POLR2I, PPM1B, PPP1R14A, PPP3CA, PPP5C, PRKAA1, PSMA3, PSMB10, PSMB2, PSMC2, PSMC3, PSMC6, PSMD1, PSMD12, PSMD14, PSMD2, PSMD6, PSMD7, PSMD8, PSME1, PSME2, PSME4, PSPC1, PTBP2, PTBP3, PTPA, PTRH2, PUS7, PYCARD, QARS1, RBBP4, RBM4, RIF1, RNF2, RNF40, RNPS1, RPL10A, RPL13, RPL13A, RPL18A, RPL22, RPL24, RPL27, RPL32, RPL36, RPL38, RPL5, RPLP1, RPLP2, RPS13, RPS15A, RPS16, RPS18, RPS21, RPS23, RPS4Y1, RPS5, RPS8, RPS9, RTN4, RYBP, SARS1, SCAI, SERPINB1, SERPINB2, SERPINB8, SERPINB9, SERPINE2, SESN2, SET, SF1, SHFL, SHMT2, SIRT1, SLIT2, SMAD4, SMARCB1, SMARCC1, SMARCE1, SMCHD1, SMPD1, SMYD3, SNW1, SORBS3, SPHK2, SQSTM1, SRC, SRSF10, SRSF2, SSB, STAT1, STON1, STRN3, SUFU, TAX1BP1, TBK1, THBS1, TIPRL, TRIM21, TRIM25, TRIM28, TRIP12, TSC2, TSPO, TUT4, TXN, UBA2, UBA3, UBE2L6, UBR2, UFD1, URI1, USP4, USP7, USP9X, VDAC2, VPS25, VTN, WDR35, WDR5, WFS1, WWC2, WWP2, XIAP, YAP1, YBX1, ZC3HAV1, ZFP36L2, ZMPSTE24, ZNF148]	
negative regulation of cellular metabolic process	GO_BiologicalProcess-EBI-UniProt-GOA-ACAP-ARAP_13.05.2021_00h00	3.67E-55	9.648169	266	[AASS, ABCB7, ABCE1, ACADVL, ACOT8, ADARB1, AGO2, AKR1B1, ANKRD13A, APEX1, APLP2, APP, APPL2, ARPP19, ARRB1, ATG7, ATP1A1, BIN1, BOD1, C1QBP, CASP3, CBX1, CBX2, CBX3, CBX5, CBX8,	Upregulation

					CC2D1A, CD109, CDC73, CDK5RAP3, CENPV, CHORDC1, CHP1, CIRBP, CLU, CNOT1, CNOT10, CORO1C, CRTAP, CSTB, CTBP1, CTNNB1, CTSA, CUX1, DAB2, DAPK1, DCPS, DHCR24, DHFR, DHX34, DHX36, DICER1, DNMT1, DUSP3, DYNLL1, EED, EIF2AK2, EIF2S1, EIF3E, EIF4G3, EIF6, ENPP1, EPRS1, ERLIN2, EZR, FAM3A, FHL2, FKBP1A, FMR1, FTO, G6PD, GAPDH, GATAD2A, GCFC2, GIPC1, GLA, GLMN, GNL3L, GPD1L, GPI, GSK3B, GSTP1, H1-10, H1-5, HAT1, HDAC2, HDAC4, HK2, HMOX1, HNRNPA0, HNRNPC, HNRNPR, IGF2BP1, IGF2BP2, IKBKB, ILF3, IMPACT, IPO5, IREB2, ITGAV, ITIH4, ITM2C, IVNS1ABP, KAT7, KAT8, KDM1A, KDM4B, LANCL2, LARP1, LARP4B, LIG3, LIMS1, LLGL1, LMCD1, LRPPRC, LRRFIP1, LRRK1, MACROH2A1, MAD2L1, MAEA, MAGI1, MBD3, METTL14, MLH1, MMAB, MORF4L1, MOV10, MPHOSPH8, MSH2, MSH6, MTA1, MVP, MYADM, MYD88, NCK2, NCL, NCOA5, NCOA7, NDUFA13, NEDD4, NELFCD, NF1, NF2, NIBAN1, NIBAN2, NLE1, NNT, NOCT, NOP53, NPM1, NT5C3B, NUDT16, NUDT16L1, NUP62, NXN, OXSR1, P3H1, PAIP1, PARP1, PARP14, PATZ1, PAWR, PBXIP1, PEBP1, PGP, PIK3CA, PIN1, PIP4K2B, PKN2, PLPP3, PML, POLR2A, POLR2B, POLR2H, POLR2I, PPP1R14A, PPP2R5A, PPP3CA, PPP5C, PRKAA1, PSPC1, PTBP2, PTBP3, PTGIS, PTPA, PUS7, PYCARD, QARS1, QSOX1, RBM4, RIF1, RNF2, RNF40, RNPS1,	

					RPL13A, RPL5, RPS13, RTN4, RYBP, SARS1, SCAI, SCFD1, SERPINB1, SERPINB2, SERPINB8, SERPINB9, SERPINE2, SESN2, SET, SF1, SHFL, SHMT2, SIRT1, SLC25A12, SLIT2, SMAD4, SMARCB1, SMARCC1, SMARCE1, SMCHD1, SMPD1, SMYD3, SNW1, SORBS3, SPHK2, SQSTM1, SRC, SRSF10, SRSF2, STAT1, STON1, STRN3, SUFU, TAB2, THBS1, TIGAR, TIPRL, TRIM21, TRIM28, TRIP12, TSC2, TSPO, TUT4, TXN, UBA2, UBA3, UBR2, URI1, USP4, USP7, USP9X, VDAC1, VDAC2, VPS25, VTN, WDR35, WDR5, WFS1, WWC2, WWP2, XIAP, YAP1, YBX1, ZFP36L2, ZMPSTE24, ZNF148]	
macromolecule modification	GO_BiologicalProcess-EBI-UniProt-GOA-ACAP-ARAP_13.05.2021_00h00	3.67E-55	9.787522	433	[AARS1, AASDHPPT, AASS, ABHD12, ABHD16A, ABI1, ACTB, ADAM10, ADARB1, ADNP, AKR1B1, ALB, ALG11, APEX1, APLP2, APP, ARAF, ARFGEF1, ARMT1, ARPP19, ARRB1, ASB1, ASH2L, ASPSCR1, ATE1, ATG7, AUP1, B3GAT3, B4GALT1, B4GALT5, BAZ1B, BCR, BOD1, BST1, C1GALT1, CAB39L, CAMK2D, CASK, CASP3, CBX8, CCDC22, CCN2, CCNH, CCNK, CCNL1, CD109, CD81, CDC34, CDC42BPA, CDC73, CDK1, CDK16, CDK17, CDK2, CDK5RAP3, CEMIP, CFAP20, CHAMP1, CHORDC1, CHP1, CHTOP, CLU, CMTR1, CNGB1, COG7, CORO1C, CRBN, CRTAP, CSPG4, CTBP1, CTNNB1, CTU1, CUL1, CUL4B, DAB2, DAPK1, DCN, DERL1, DGUOK, DHPS, DIMT1, DIPK2A, DLST, DNM1L, DNMT1, DSP, DUSP23, DUSP3, DVL2, DYNLL1, EED,	Upregulation

					EFEMP1, EIF2AK2, EIF2S1, EIF3F, ELP3, ELP4, ELP5, EMG1, ENPP1, ENY2, EOGT, EPHA2, EPHA3, EPHB4, ERBB2, ETFDH, EXTL2, FAM3A, FASN, FBXL12, FBXW8, FERMT2, FGF2, FKBP1A, FKBP5, FKBP9, FMR1, FN1, FN3K, FTO, FUT11, FUT8, G6PD, GALNT1, GALNT2, GALNT7, GAN, GAPDH, GARS1, GATAD2A, GFPT2, GLMN, GLUL, GLYR1, GNL3L, GOLGA7, GPD1L, GPHN, GPI, GSK3B, GSTP1, GTF3C4, GXYLT1, GXYLT2, H1-5, HAT1, HDAC2, HDAC4, HECTD4, HERC2, HERC4, HGS, HLA-A, HMBS, HMCES, HPX, HSD17B10, HSP90AA1, HSP90B1, HSPA5, IGFBP7, IKBKB, IKBKG, ILF3, ILKAP, IMPACT, IPO5, IQGAP3, IVNS1ABP, KAT7, KAT8, KDM1A, KDM3B, KDM4B, LAMC1, LLGL1, LMO7, LNPEP, LRRK1, LTBP1, LYPLA2, MACROD1, MACROH2A1, MAD2L1, MAEA, MAGI1, MALT1, MAP2K3, MAP3K20, MAP3K4, MAP4K4, MARCHF5, MBD3, MCM2, MED30, METTL14, MGAT1, MGMT, MMAB, MOGS, MORF4L1, MPHOSPH8, MRM3, MTA1, MTRR, MVD, MVP, MYADM, N6AMT1, NCK2, NCOA7, NDUFAF7, NEDD4, NEDD8, NEK1, NF1, NF2, NIBAN1, NIBAN2, NMT1, NNT, NOP53, NPM1, NPR2, NSMCE3, NSUN5, NUB1, NUDT16, NUP205, NUP35, NUP62, NXN, OBSL1, OGFOD1, OSBPL8, OTUD6B, OXSR1, P3H1, P3H3, PARG, PARP1, PARP14, PARP4, PDK1, PDZRN3, PGAM5, PGM3, PGP, PHKB, PHPT1, PIGO, PIGT, PIGU, PIK3CA, PIN1, PKN2, PLOD2,	

					PLPP3, PLXNB2, PML, PMM2, POFUT1, POGLUT3, POMGNT1, POMGNT2, POMK, PPIB, PPIE, PPIG, PPM1B, PPP1CA, PPP1R14A, PPP1R7, PPP2R5A, PPP3CA, PPP5C, PPWD1, PRKAA1, PRKAB2, PRKCI, PRKCSH, PRKG1, PRMT7, PRPF19, PSMA3, PSMB10, PSMB2, PSMC2, PSMC3, PSMC6, PSMD1, PSMD12, PSMD14, PSMD2, PSMD6, PSMD7, PSMD8, PSME1, PSME2, PSME4, PTK7, PTPA, PTPMT1, PTPN12, PTPRF, PTPRG, PUS7, PXK, PYCARD, QARS1, QSOX1, QTRT1, QTRT2, RAB11B, RAB1A, RAB3D, RAB5A, RAB8A, RANGAP1, RAP2B, RAP2C, RCN1, RIF1, RIOK3, RIPK2, RNF114, RNF2, RNF213, RNF214, RNF40, RNGTT, ROR1, RPL5, RPLP1, RPRD1A, RPRD1B, RSPRY1, RYBP, SAE1, SENP3, SESN2, SET, SHMT2, SHPRH, SIRT1, SLC1A1, SLIT2, SMAD4, SMARCAD1, SMARCB1, SMPD1, SMYD3, SNF8, SNW1, SOD1, SPHK2, SQSTM1, SRC, SRD5A3, SSB, STAT2, STK24, TAB2, TAF6L, TBK1, TF, TGFBR3, TGM2, TGS1, THADA, THBS1, TIPRL, TMEM119, TMX3, TNIK, TPP2, TPST2, TPX2, TRIM21, TRIM25, TRIM28, TRIM56, TRIM59, TRIO, TRIP12, TRMT10C, TRMT2A, TRMT5, TSC2, TSPO, TTLL12, TXN, UBA2, UBA3, UBE2L6, UBE2Q1, UBR2, UBR4, UBXN2A, UFD1, UHRF2, URI1, URM1, USP11, USP22, USP28, USP4, USP48, USP5, USP7, USP9X, USP9Y, UST, VDAC2, VLDLR, VPS25, VRK1, VRK3, VTN, WDR5, WDR70, WFS1, WIPI2, WWP2, XIAP,	

					YEATS4, YES1, YOD1, ZC3HAV1, ZDHHC13, ZDHHC21, ZMPSTE24]	
cellular nitrogen compound biosynthetic process	GO_BiologicalProcess-EBI-UniProt-GOA-ACAP-ARAP_13.05.2021_00h00	3.67E-55	9.611252	492	[AARS1, AASS, ABCB7, ABCE1, ABT1, ACACA, ACADM, ACO1, ACOT7, ACSL3, ACSL4, ACSS2, ACTR2, ACTR3, ADK, ADNP, ADSL, AGO2, AGRN, AK1, AK3, AK4, AK6, AKR1B1, ALB, ALDOA, ANXA3, APBB2, APEX1, APP, APRT, ARHGEF11, ARID1B, ARRB1, ASAH1, ASNS, ATP6V0C, ATP6V1A, B4GALT5, BAZ1B, BDH2, BRD3, C1QBP, CAMK2D, CASK, CBX1, CBX2, CBX3, CBX5, CBX8, CC2D1A, CCDC22, CCN2, CCNH, CCNK, CCNL1, CCT2, CCT3, CCT6A, CD200, CD81, CDC5L, CDC73, CDK1, CDK16, CDK17, CDK2, CDK5RAP3, CENPV, CERS6, CHD2, CHD4, CHD7, CHORDC1, CHP1, CIRBP, CLU, CMPK1, CNGB1, CNOT1, CNOT10, COLEC12, CPSF2, CRABP2, CSTF3, CTBP1, CTNNB1, CTPS1, CUX1, CYB5R3, DAB2, DAPK1, DARS1, DCAKD, DCPS, DDX1, DENR, DGUOK, DHFR, DHPS, DHX36, DICER1, DNMT1, DRG1, DUT, DVL2, EARS2, EDF1, EED, EEF1E1, EEF1G, EFEMP1, EFL1, EIF2AK2, EIF2B1, EIF2D, EIF2S1, EIF3A, EIF3B, EIF3E, EIF3F, EIF4G3, EIF6, ELP3, ELP4, ENPP1, ENY2, EPRS1, ERBB2, EZR, FAM3A, FASN, FGF2, FHL2, FHOD1, FMR1, FTO, GAPDH, GARS1, GATAD2A, GATB, GCDH, GCFC2, GFPT2, GGCT, GLA, GLE1, GLMP, GLO1, GLYR1, GMPS, GNL3L, GPI, GTF2E1, GTF2F1, GTF2F2, GTF2I, GTF3C3, GTF3C4, H1-10, H1-5, HARS1, HAT1, HDAC2, HDAC4, HDGFL2, HEXB, HINT1, HMBS,	No change

					HMOX1, HNRNPC, HNRNPR, HSD17B4, HSP90AA1, HSPA5, HTATSF1, IARS2, IDH2, IGF2BP1, IGF2BP2, IKBKB, IKBKG, ILF3, IMPACT, INTS1, INTS9, IREB2, IVNS1ABP, JAG1, KAT7, KAT8, KDM1A, KDM3B, LANCL2, LARP1, LARP4B, LARP6, LIG1, LIG3, LIMS1, LMCD1, LMO7, LRPPRC, LRRFIP1, LUM, MACROH2A1, MAGI1, MALT1, MAP2K3, MAP3K4, MBD3, MCM2, ME1, MED17, MED23, MED30, METTL14, MGA, MLF2, MLH1, MMAB, MORF4L1, MOV10, MPHOSPH8, MRPL10, MRPS17, MRPS21, MRPS31, MRRF, MTA1, MTAP, MYD88, MYO6, N6AMT1, NCK2, NCL, NCOA5, NCOA7, NDRG1, NDUFA13, NEDD4, NEDD8, NELFCD, NFATC2IP, NFKB2, NIBAN1, NIBAN2, NMD3, NOCT, NOL11, NOP53, NPLOC4, NPM1, NPR2, NQO1, NSUN5, NT5C, NT5C3B, NUDT15, NUDT16, NUP35, NUP62, OGFOD1, OPLAH, OXA1L, OXSR1, PAICS, PAIP1, PANK4, PAPSS2, PARP1, PARP14, PARP4, PATZ1, PAWR, PBXIP1, PDE5A, PDK1, PELO, PFAS, PFKM, PGM3, PIN1, PIR, PITHD1, PKLR, PKM, PLD1, PLPP3, PLXNB2, PML, PMM2, PNP, POFUT1, POGZ, POLD1, POLD2, POLE2, POLR1C, POLR1D, POLR2A, POLR2B, POLR2H, POLR2I, POLR3A, POLR3C, PPIE, PPP1CA, PPP3CA, PPP5C, PRIM2, PRKAA1, PRKCI, PRKCSH, PRPSAP1, PRPSAP2, PSAT1, PSIP1, PSMA3, PSMB10, PSMB2, PSMC2, PSMC3, PSMC6, PSMD1, PSMD12, PSMD14,	

					PSMD2, PSMD6, PSMD7, PSMD8, PSME1, PSME2, PSME4, PSPC1, PTGES2, PTGIS, PTRH2, PUS7, PXN, PYCARD, QARS1, QDPR, RAD21, RAP2C, RARS2, RBM4, RFC2, RFC3, RFC5, RIF1, RIPK2, RNF2, RNGTT, RNPS1, ROR1, RPA1, RPA2, RPL10A, RPL13, RPL13A, RPL18A, RPL22, RPL24, RPL27, RPL32, RPL36, RPL38, RPL5, RPLP1, RPLP2, RPRD1A, RPRD1B, RPRD2, RPS13, RPS15A, RPS16, RPS18, RPS21, RPS23, RPS4Y1, RPS5, RPS8, RPS9, RRM1, RYBP, SAMHD1, SAP30BP, SARM1, SARNP, SARS1, SCAI, SEC14L2, SELENOT, SESN2, SET, SF1, SHFL, SHMT2, SIRT1, SLC11A2, SLC1A1, SLC25A12, SLC25A13, SLC25A15, SLC26A2, SLC30A9, SLC9A1, SMAD4, SMARCA1, SMARCB1, SMARCC1, SMARCD3, SMARCE1, SMCHD1, SMPD1, SMYD3, SNF8, SNRPD3, SNRPE, SNW1, SNX5, SOAT1, SOD2, SORBS3, SPHK2, SPTLC1, SPTLC2, SQSTM1, SRA1, SRC, SRSF10, SRSF2, SSRP1, STAT1, STAT2, STAT5B, STON1, STRN3, SUFU, SUPT16H, TAB2, TAF6L, TAX1BP1, TBCE, TBK1, TBPL1, TCP1, TDRD3, TF, TFAM, THBS1, TJP2, TRADD, TRIM21, TRIM25, TRIM28, TRIM56, TRIP13, TRMT10C, TSPO, TTC5, TUT4, TXN, TYMP, UAP1, UBA2, UBA3, UBE2L6, UBR2, UBTF, UFD1, UGP2, UMPS, URI1, USP22, USP7, USP9X, VARS1, WDR35, WFS1, WRNIP1, WWC2, WWP2, YAP1, YARS1, YBX1, YEATS4, YES1,	

					ZFP36L2, ZMPSTE24, ZNF148]	
organic cyclic compound biosynthetic process	GO_BiologicalProcess-EBI-UniProt-GOA-ACAP-ARAP_13.05.2021_00h00	3.67E-55	9.134615	418	[AASS, ABCB7, ABT1, ACACA, ACAT2, ACOT7, ACOT8, ACSL3, ACSL4, ACSS2, ACTR2, ACTR3, ADK, ADNP, ADSL, AGO2, AGRN, AK1, AK3, AK4, AK6, AKR1B1, ALB, ALDH18A1, ALDOA, ANXA3, APBB2, APEX1, APP, APRT, ARHGEF11, ARID1B, ARRB1, ASAH1, ATP1A1, ATP6V0C, ATP6V1A, BAZ1B, BRD3, C1QBP, CAMK2D, CASK, CBX1, CBX2, CBX3, CBX5, CBX8, CC2D1A, CCDC22, CCN2, CCNH, CCNK, CCNL1, CCT2, CCT3, CCT6A, CD200, CD81, CDC5L, CDC73, CDK1, CDK16, CDK17, CDK2, CDK5RAP3, CENPV, CHD2, CHD4, CHD7, CHORDC1, CHP1, CLU, CMPK1, CNGB1, CNOT1, COLEC12, CPSF2, CRABP2, CSTF3, CTBP1, CTNNB1, CTPS1, CUX1, CYB5R3, CYP51A1, DAB2, DCAKD, DDX1, DGUOK, DHCR24, DHCR7, DHFR, DHX36, DICER1, DNMT1, DRG1, DUT, DVL2, EDF1, EED, EFEMP1, EIF2AK2, ELP3, ELP4, ENPP1, ENY2, ERBB2, ERLIN2, EZR, FAM3A, FASN, FDXR, FGF2, FHL2, FHOD1, G6PD, GARS1, GATAD2A, GCDH, GCFC2, GFPT2, GIPC1, GLMP, GLO1, GLYR1, GMPS, GNL3L, GPHN, GPI, GTF2E1, GTF2F1, GTF2F2, GTF2I, GTF3C3, GTF3C4, H1-10, H1-5, HAT1, HDAC2, HDAC4, HDGFL2, HEXB, HINT1, HMBS, HMGCS1, HMOX1, HNRNPC, HSD17B10, HSD17B11, HSD17B4, HSP90AA1, HSPA5, HTATSF1, IDH2, IDI1, IGFBP7, IKBKB, IKBKG, ILF3, IMPACT, INTS1, INTS9, IREB2, IVNS1ABP, JAG1, KAT7, KAT8, KDM1A, KDM3B, KPNB1,	Upregulation

					LANCL2, LBR, LIG1, LIG3, LIMS1, LMCD1, LMO7, LRRFIP1, LSS, LUM, MACROH2A1, MAGI1, MALT1, MAP2K3, MAP3K4, MBD3, MCM2, ME1, MED17, MED23, MED30, MGA, MLF2, MMAB, MOCS2, MORF4L1, MOV10, MPHOSPH8, MTA1, MTAP, MVD, MVK, MYD88, MYO6, NCK2, NCL, NCOA5, NCOA7, NDRG1, NDUFA13, NEDD4, NEDD8, NELFCD, NFATC2IP, NFKB2, NIBAN2, NMD3, NOCT, NOL11, NOP53, NPLOC4, NPM1, NPR2, NSDHL, NT5C, NUDT15, NUDT16, NUP35, NUP62, OAT, OSBPL1A, OSBPL2, OXSR1, PAICS, PANK4, PAPSS2, PARP1, PARP14, PARP4, PATZ1, PAWR, PBXIP1, PDE5A, PDK1, PFAS, PFKM, PGM3, PIN1, PIR, PITHD1, PKLR, PLPP3, PML, PMM2, PNP, POFUT1, POGZ, POLD1, POLD2, POLE2, POLR1C, POLR1D, POLR2A, POLR2B, POLR2H, POLR2I, POLR3A, POLR3C, PPIE, PPP3CA, PPP5C, PRIM2, PRKAA1, PRKCI, PRPSAP1, PRPSAP2, PSAT1, PSIP1, PSMA3, PSMB10, PSMB2, PSMC2, PSMC3, PSMC6, PSMD1, PSMD12, PSMD14, PSMD2, PSMD6, PSMD7, PSMD8, PSME1, PSME2, PSME4, PSPC1, PTGES2, PTGIS, PTRH2, PXN, PYCARD, PYCR1, PYCR3, QARS1, QDPR, RAD21, RAP2C, RFC2, RFC3, RFC5, RIF1, RIPK2, RNF2, RNGTT, RNPS1, ROR1, RPA1, RPA2, RPRD1A, RPRD1B, RPRD2, RRM1, RYBP, SAMHD1, SAP30BP, SARM1, SARS1, SCAI, SCP2, SEC14L2, SESN2, SET, SF1, SHMT2, SIRT1, SLC11A2, SLC25A12, SLC25A13,	

					SLC25A15, SLC26A2, SLC30A9, SLC9A1, SMAD4, SMARCA1, SMARCB1, SMARCC1, SMARCD3, SMARCE1, SMCHD1, SMYD3, SNF8, SNRPD3, SNRPE, SNW1, SNX5, SOAT1, SOD1, SOD2, SORBS3, SPHK2, SQSTM1, SRA1, SRC, SRD5A3, SRSF10, SRSF2, SSRP1, STAT1, STAT2, STAT5B, STON1, STRN3, SUFU, SUPT16H, TAB2, TAF6L, TAX1BP1, TBK1, TBPL1, TCP1, TDRD3, TF, TFAM, TJP2, TRADD, TRIM21, TRIM25, TRIM28, TRIM56, TRIP13, TSPO, TTC5, TXN, TYMP, UAP1, UBA2, UBA3, UBE2L6, UBR2, UBTF, UFD1, UGP2, UMPS, URI1, USP22, USP7, USP9X, VDAC2, WFS1, WRNIP1, WWC2, WWP2, YAP1, YBX1, YEATS4, YES1, ZFP36L2, ZMPSTE24, ZNF148]	
cellular macromolecule biosynthetic process	GO_BiologicalProcess-EBI-UniProt-GOA-ACAP-ARAP_13.05.2021_00h00	3.67E-55	9.496011	488	[AARS1, AASS, ABCE1, ABT1, ACADM, ACO1, ACOT8, ACTR2, ACTR3, ADNP, AGO2, AGRN, AKR1B1, ALG11, ANXA3, APBB2, APEX1, APP, ARFGEF1, ARHGEF11, ARID1B, ARRB1, ATG7, B3GAT3, B4GALT1, B4GALT5, BAZ1B, BRD3, C1GALT1, C1QBP, CAMK2D, CASK, CBX1, CBX2, CBX3, CBX5, CBX8, CC2D1A, CCDC22, CCN2, CCNH, CCNK, CCNL1, CCT2, CCT3, CCT6A, CD200, CD81, CDC34, CDC5L, CDC73, CDK1, CDK16, CDK17, CDK2, CDK5RAP3, CENPV, CHAF1B, CHD2, CHD4, CHD7, CHORDC1, CHP1, CHPF, CHSY3, CIRBP, CLU, CNGB1, CNOT1, CNOT10, COG7, COLEC12, CPSF2, CRABP2, CSPG4, CSTF3, CTBP1, CTNNB1, CUX1,	No change

					DAB2, DAPK1, DARS1, DCN, DCPS, DDX1, DENR, DHFR, DHPS, DHX36, DICER1, DNMT1, DRG1, DUT, DVL2, EARS2, EDF1, EED, EEF1E1, EEF1G, EFEMP1, EFL1, EIF2AK2, EIF2B1, EIF2D, EIF2S1, EIF3A, EIF3B, EIF3E, EIF3F, EIF4G3, EIF6, ELP3, ELP4, ENPP1, ENY2, EOGT, EPM2AIP1, EPRS1, ERBB2, EXD2, EXTL2, EZR, FEN1, FGF2, FHL2, FHOD1, FMR1, FTO, FUT11, FUT8, GALNT1, GALNT2, GALNT7, GAPDH, GARS1, GATAD2A, GATB, GBE1, GCFC2, GFPT2, GLE1, GLMP, GLO1, GLUL, GLYR1, GNL3L, GOLGA7, GPI, GRWD1, GSK3B, GTF2E1, GTF2F1, GTF2F2, GTF2I, GTF3C3, GTF3C4, GXYLT1, GXYLT2, GYS1, H1-10, H1-5, HARS1, HAT1, HDAC2, HDAC4, HDGFL2, HEXB, HINT1, HMOX1, HNRNPC, HNRNPR, HSD17B4, HSP90AA1, HSPA5, HTATSF1, IARS2, IGF2BP1, IGF2BP2, IKBKB, IKBKG, ILF3, IMPACT, INTS1, INTS9, IREB2, ITM2C, IVNS1ABP, JAG1, KAT7, KAT8, KDM1A, KDM3B, LANCL2, LARP1, LARP4B, LARP6, LIG1, LIG3, LIMS1, LMCD1, LMO7, LRPPRC, LRRFIP1, LRWD1, LUM, MACROH2A1, MAGI1, MALT1, MAP2K3, MAP3K4, MBD3, MCM2, MCM3, MCM4, MCM5, MCM6, MCM7, MCMBP, MED17, MED23, MED30, METTL14, MGA, MGAT1, MLF2, MLH1, MMAB, MOGS, MORF4L1, MOV10, MPHOSPH8, MRPL10, MRPS17, MRPS21, MRPS31, MRRF, MTA1, MVD, MYD88, MYO6, N6AMT1, NCK2, NCL, NCOA5, NCOA7, NDRG1, NDUFA13, NEDD4,	

					NEDD8, NELFCD, NFATC2IP, NFKB2, NIBAN1, NIBAN2, NMT1, NOCT, NOL11, NOP53, NPLOC4, NPM1, NSUN5, NT5C3B, NUP35, NUP62, OGFOD1, ORC4, ORC5, OXA1L, OXSR1, PAIP1, PARP1, PARP14, PARP4, PATZ1, PAWR, PBXIP1, PELO, PFKM, PGM2L1, PGM3, PIGO, PIGT, PIGU, PIN1, PIR, PITHD1, PKM, PLD1, PLOD2, PLPP3, PLXNB2, PML, PMM2, POFUT1, POGLUT3, POGZ, POLD1, POLD2, POLE2, POLR1C, POLR1D, POLR2A, POLR2B, POLR2H, POLR2I, POLR3A, POLR3C, POMGNT1, POMGNT2, POMK, PPIE, PPM1B, PPP1CA, PPP3CA, PPP5C, PRIM2, PRKAA1, PRKCI, PRKCSH, PSIP1, PSMA3, PSMB10, PSMB2, PSMC2, PSMC3, PSMC6, PSMD1, PSMD12, PSMD14, PSMD2, PSMD6, PSMD7, PSMD8, PSME1, PSME2, PSME4, PSPC1, PTGES2, PTGIS, PTRH2, PUS7, PXN, PYCARD, QARS1, RAD21, RAD9A, RAP2C, RARS2, RBBP4, RBM4, RECQL, RFC2, RFC3, RFC5, RIF1, RIPK2, RNF2, RNGTT, RNPS1, ROR1, RPA1, RPA2, RPL10A, RPL13, RPL13A, RPL18A, RPL22, RPL24, RPL27, RPL32, RPL36, RPL38, RPL5, RPLP1, RPLP2, RPRD1A, RPRD1B, RPRD2, RPS13, RPS15A, RPS16, RPS18, RPS21, RPS23, RPS4Y1, RPS5, RPS8, RPS9, RRM1, RYBP, SAMHD1, SAP30BP, SARNP, SARS1, SCAI, SEC14L2, SELENOT, SESN2, SET, SF1, SHFL, SHMT2, SIRT1, SLC25A15, SLC30A9, SLC9A1, SMAD4, SMARCA1, SMARCB1,	

					SMARCC1, SMARCD3, SMARCE1, SMC3, SMCHD1, SMYD3, SNF8, SNRPD3, SNRPE, SNW1, SNX5, SOAT1, SOD2, SORBS3, SQSTM1, SRA1, SRC, SRD5A3, SRSF10, SRSF2, SSRP1, STAT1, STAT2, STAT5B, STON1, STRN3, SUFU, SUPT16H, TAB2, TAF6L, TAX1BP1, TBCE, TBK1, TBPL1, TCP1, TDRD3, TF, TFAM, THBS1, TRADD, TRIM21, TRIM25, TRIM28, TRIM56, TRIP13, TRMT10C, TSPO, TTC5, TUT4, TXN, UBA2, UBA3, UBE2L6, UBR2, UBTF, UFD1, UGP2, URI1, USP22, USP7, USP9X, UST, VARS1, WFS1, WIPI2, WRNIP1, WWC2, WWP2, XYLT2, YAP1, YARS1, YBX1, YEATS4, YES1, ZDHHC13, ZDHHC21, ZFP36L2, ZMPSTE24, ZNF148]	
cellular protein metabolic process	GO_BiologicalProcess-EBI-UniProt-GOA-ACAP-ARAP_13.05.2021_00h00	3.67E-55	10.358491	549	[AARS1, AASDHPPT, AASS, ABCE1, ABHD12, ABI1, ACO1, ACTB, ADAM10, ADARB1, ADNP, AGO2, AKR1B1, ALB, ALG11, ANKZF1, APAF1, APLP2, APP, ARAF, ARFGEF1, ARMT1, ARPP19, ARRB1, ASB1, ASH2L, ASPSCR1, ATE1, ATG7, AUP1, B3GAT3, B4GALT1, B4GALT5, BAZ1B, BCR, BIN1, BOD1, BST1, C1GALT1, C1QBP, CAB39L, CAMK2D, CASK, CASP3, CBX8, CCDC22, CCN2, CCNH, CCNK, CCNL1, CD109, CD81, CDC34, CDC42BPA, CDC73, CDK1, CDK16, CDK17, CDK2, CDK5RAP3, CEMIP, CFAP20, CHAMP1, CHORDC1, CHP1, CHTOP, CIRBP, CLTC, CLU, CNOT1, CNOT10, COG7, CORO1C, CRBN, CRTAP, CSPG4, CSTB, CTBP1, CTNNB1, CTSC, CTU1, CUL1, CUL4B, CYCS, DAB2, DAPK1,	No change

					DARS1, DCN, DCPS, DDI2, DDX1, DENR, DERL1, DGUOK, DHCR24, DHFR, DHPS, DHX36, DIPK2A, DLST, DNM1L, DNMT1, DRG1, DSP, DUSP23, DUSP3, DVL2, DYNLL1, EARS2, EED, EEF1E1, EEF1G, EFEMP1, EFL1, EIF2AK2, EIF2B1, EIF2D, EIF2S1, EIF3A, EIF3B, EIF3E, EIF3F, EIF4G3, EIF6, ELP3, ELP4, ENPP1, ENY2, EOGT, EPHA2, EPHA3, EPHB4, EPRS1, ERBB2, ERLIN2, ETFDH, EXTL2, EZR, FAM3A, FASN, FBXL12, FBXW8, FDXR, FERMT2, FGF2, FKBP1A, FKBP5, FKBP9, FMR1, FN1, FN3K, FTO, FUT11, FUT8, G6PD, GALNT1, GALNT2, GALNT7, GAN, GAPDH, GARS1, GATB, GFPT2, GIPC1, GLE1, GLMN, GLUL, GLYR1, GNL3L, GOLGA7, GPD1L, GPHN, GPI, GSK3B, GSTP1, GTF3C4, GXYLT1, GXYLT2, H1-5, HARS1, HAT1, HDAC2, HDAC4, HECTD4, HERC2, HERC4, HEXB, HGS, HIP1, HLA-A, HMBS, HMCES, HNRNPR, HPX, HSP90AA1, HSP90B1, HSPA5, HSPE1, IARS2, IGF2BP1, IGF2BP2, IGFBP7, IKBKB, IKBKG, ILF3, ILKAP, IMPACT, IPO5, IQGAP3, IREB2, IST1, ITIH4, IVNS1ABP, KAT7, KAT8, KDM1A, KDM3B, KDM4B, LAMC1, LARP1, LARP4B, LARP6, LLGL1, LMCD1, LMO7, LNPEP, LRPPRC, LRRK1, LTBP1, LYPLA2, MACROD1, MACROH2A1, MAD2L1, MAEA, MAGI1, MALT1, MAP2K3, MAP3K20, MAP3K4, MAP4K4, MARCHF5, MBD3, MCM2, MED30, METTL14, MGAT1, MLH1, MMAB, MOCS2, MOGS, MORF4L1, MOV10, MRPL10, MRPS17,	

					MRPS21, MRPS31, MRRF, MSN, MTA1, MTCH1, MVD, MVP, MYADM, MYH9, N6AMT1, NCK2, NCL, NCOA7, NDRG1, NDUFA13, NDUFAF7, NEDD4, NEDD8, NEK1, NF1, NF2, NIBAN1, NLE1, NMT1, NNT, NOCT, NOP53, NPLOC4, NPM1, NPR2, NSMCE3, NSUN5, NT5C3B, NUB1, NUDT15, NUP205, NUP35, NUP62, NXN, OBSL1, OGFOD1, OSBPL8, OTUD6B, OXA1L, OXSR1, P3H1, P3H3, PACSIN3, PAIP1, PARG, PARL, PARP1, PARP14, PARP4, PDCD6IP, PDK1, PDZRN3, PEBP1, PELO, PGAM5, PGM3, PGP, PHKB, PHPT1, PIGO, PIGT, PIGU, PIK3CA, PIN1, PITHD1, PKM, PKN2, PLD1, PLOD2, PLPP3, PLXNB2, PML, PMM2, POFUT1, POGLUT3, POMGNT1, POMGNT2, POMK, PPIB, PPIE, PPIG, PPM1B, PPP1CA, PPP1R14A, PPP1R7, PPP2R5A, PPP3CA, PPP5C, PPWD1, PRKAA1, PRKAB2, PRKCI, PRKCSH, PRKG1, PRMT7, PRPF19, PSMA3, PSMB10, PSMB2, PSMC2, PSMC3, PSMC6, PSMD1, PSMD12, PSMD14, PSMD2, PSMD6, PSMD7, PSMD8, PSME1, PSME2, PSME4, PTK7, PTPA, PTPMT1, PTPN12, PTPRF, PTPRG, PUS7, PXK, PYCARD, QARS1, QSOX1, RAB11B, RAB1A, RAB3D, RAB5A, RAB8A, RANGAP1, RAP2B, RAP2C, RARS2, RBM4, RCN1, RCN3, RDX, RIF1, RIOK3, RIPK2, RNF114, RNF2, RNF213, RNF214, RNF40, RNGTT, ROR1, RPL10A, RPL13, RPL13A, RPL18A, RPL22, RPL24, RPL27, RPL32, RPL36, RPL38, RPL5, RPLP1, RPLP2, RPRD1A,	

					RPRD1B, RPS13, RPS15A, RPS16, RPS18, RPS21, RPS23, RPS4Y1, RPS5, RPS8, RPS9, RSPRY1, RYBP, SAE1, SARNP, SARS1, SEC11C, SEC61B, SELENOT, SENP3, SERPINB1, SERPINB2, SERPINB8, SERPINB9, SERPINE2, SESN2, SET, SHFL, SHMT2, SHPRH, SIRT1, SLC1A1, SLIT2, SMAD4, SMARCAD1, SMARCB1, SMARCC1, SMPD1, SMYD3, SNF8, SNW1, SNX5, SOD1, SPHK2, SQSTM1, SRC, SRD5A3, STAT2, STK24, SUFU, TAB2, TAF6L, TBC1D10A, TBCE, TBK1, TF, TGFBR3, TGM2, THBS1, TIPRL, TMEM119, TMX3, TNIK, TPP1, TPP2, TPST2, TPX2, TRADD, TRIM21, TRIM25, TRIM28, TRIM56, TRIM59, TRIO, TRIP12, TRMT10C, TSC2, TSPO, TTLL12, TUT4, TXN, UACA, UBA2, UBA3, UBAC2, UBE2L6, UBE2Q1, UBR2, UBR4, UBXN2A, UFD1, UHRF2, URI1, URM1, USP11, USP22, USP28, USP4, USP48, USP5, USP7, USP9X, USP9Y, UST, VARS1, VDAC2, VLDLR, VPS25, VPS36, VRK1, VRK3, VTN, WDR35, WDR5, WDR70, WFS1, WIPI2, WWP2, XIAP, YARS1, YBX1, YEATS4, YES1, YOD1, ZC3HAV1, ZDHHC13, ZDHHC21, ZFP36L2, ZMPSTE24]	
regulation of protein metabolic process	GO_BiologicalProcess-EBI-UniProt-GOA-ACAP-ARAP_13.05.2021_00h00	3.67E-55	11.047755	310	[AARS1, AASS, ABCE1, ABI1, ACO1, ACOT8, ACTB, ADARB1, ADNP, AGO2, APAF1, APEX1, APLP2, APP, ARAF, ARFGEF1, ARPP19, ARRB1, ASPSCR1, ATG4B, ATG7, BIN1, BOD1, BST1, C1QBP, CAB39L, CAMK2D, CASP3, CCDC22, CCN2, CCNH, CCNK, CCNL1, CD109, CD81, CDK5RAP3, CEMIP, CHAMP1,	No change

					CHORDC1, CHP1, CHTOP, CIRBP, CLU, CNOT1, CNOT10, CORO1C, CRTAP, CSPG4, CSTB, CTBP1, CTNNB1, CTSA, CTSC, CYCS, CYP51A1, DAB2, DAPK1, DCPS, DDX1, DENR, DERL1, DHCR24, DHFR, DHX36, DIPK2A, DNMT1, DUSP3, DVL2, DYNLL1, EEF1G, EFL1, EIF2AK2, EIF2B1, EIF2D, EIF2S1, EIF3A, EIF3B, EIF3E, EIF3F, EIF4G3, EIF6, ELP3, ELP4, ENPP1, EPRS1, ERBB2, ETFDH, EZR, FASN, FBXW8, FERMT2, FGF2, FKBP1A, FMR1, FN1, FTO, G6PD, GAPDH, GIPC1, GLE1, GLMN, GLYR1, GNL3L, GPD1L, GPI, GSK3B, GSTP1, H1-5, HDAC2, HDAC4, HGS, HIP1, HNRNPR, HPX, HSP90AA1, HSPA5, HSPE1, IGF2BP1, IGF2BP2, IKBKB, IKBKG, ILF3, IMPACT, IPO5, IQGAP3, IREB2, IST1, ITGAV, ITIH4, ITM2C, IVNS1ABP, KAT7, KDM1A, KDM4B, LARP1, LARP4B, LARP6, LLGL1, LRPPRC, LRRK1, MACROH2A1, MAD2L1, MALT1, MAP2K3, MAP3K20, MAP3K4, MAP4K4, MCM2, METTL14, MGMT, MLH1, MOV10, MSN, MTA1, MTCH1, MVP, MYADM, MYH9, NCK2, NCL, NCOA7, NDUFA13, NEDD4, NEDD8, NF1, NF2, NIBAN1, NLE1, NNT, NOCT, NOP53, NPM1, NSMCE3, NSUN5, NT5C3B, NUB1, NUDT15, NUP62, NXN, OGFOD1, OSBPL8, P3H1, PACSIN3, PAIP1, PARG, PARL, PARP14, PAWR, PEBP1, PELO, PIK3CA, PIN1, PITHD1, PKM, PKN2, PLD1, PLPP3, PLXNB2, PML, PPIB, PPP1CA, PPP1R14A, PPP1R7, PPP2R5A, PPP3CA, PPP5C, PRKAA1, PRKCSH, PSMA3, PSMC2,	

					PSMC3, PSMC6, PSMD1, PSMD14, PSMD2, PSME1, PSME2, PSME4, PTPA, PUS7, PYCARD, QARS1, RAB1A, RAP2B, RAP2C, RBM4, RCN3, RDX, RIF1, RIPK2, RNF40, RPL13A, RPL22, RPL38, RPL5, RPLP1, RPS9, RTN4, RYBP, SAE1, SARNP, SELENOT, SERPINB1, SERPINB2, SERPINB8, SERPINB9, SERPINE2, SESN2, SET, SHFL, SHMT2, SIRT1, SLC1A1, SLIT2, SMAD4, SMARCB1, SMARCC1, SMPD1, SMYD3, SNF8, SNW1, SNX1, SOAT1, SOD1, SPHK2, SQSTM1, SRC, STAT2, STK24, SUFU, TAB2, TBC1D10A, TBK1, TF, THBS1, TIPRL, TMEM119, TNIK, TPX2, TRADD, TRIM21, TRIP12, TRMT10C, TSC2, TSPO, TTLL12, TUT4, TXN, UACA, UBA2, UBXN2A, URI1, USP4, USP5, USP7, VDAC2, VLDLR, VPS25, VRK3, VTN, WDR35, WDR5, WDR70, WFS1, WWP2, XIAP, YBX1, YES1, ZFP36L2, ZMPSTE24]	
positive regulation of gene expression	GO_BiologicalProcess-EBI-UniProt-GOA-ACAP-ARAP_13.05.2021_00h00	3.67E-55	10.947712	134	[ACTB, ACTG1, AFDN, AGO2, AGPAT2, AKAP12, AKR1B1, ANK3, APP, ARID1B, B2M, BAZ1B, C1QBP, CCN2, CD200, CD276, CD81, CDK1, CIRBP, CLU, CTNNB1, CYRIB, DDX1, DHX34, DHX36, DNMT1, EIF2AK2, EIF3E, EIF6, ERBB2, EZR, FERMT1, FGF2, FMR1, FN1, GAPDH, GARS1, GLMN, GPI, GSDMD, GSK3B, H1-10, H1-5, HDAC2, HGS, HK1, HLA-A, HMCES, HMOX1, HNRNPA0, HNRNPC, HNRNPLL, HPX, HSP90AA1, IGF2BP1, IMPACT, IQGAP3, ITGA3, LARP1, LARP4B, LARP6, LIMS1, LRRC32, LRRFIP1, LUM, MACROH2A1, MALT1, MED23,	Upregulation

					METTL14, MLH1, MOV10, MSH2, MSN, MYD88, MYH9, MYO1E, NCK2, NCL, NFKB2, NIBAN1, NOCT, NPM1, NSUN5, OXSR1, PAIP1, PANX1, PAWR, PKM, PLD1, PLXNB2, PNP, POLR1C, POLR1D, POLR2A, POLR2H, POLR3A, POLR3C, PPP3CA, PRDX6, PRKAA1, PRKCSH, PRPF19, PYCARD, RAB1A, RBM22, RDX, RIF1, RIOK3, RIPK2, RPL5, RPS9, SCRIB, SERPINB9, SIRT1, SNF8, SNW1, SOD1, SPHK2, SRC, SRSF5, STAT1, STAT5B, TBK1, THBS1, TMEM119, TRA2A, TRA2B, TRIM56, TRMT10C, UBA2, YAP1, YBX1, ZCCHC3, ZMPSTE24]	
negative regulation of gene expression	GO_BiologicalProcess-EBI-UniProt-GOA-ACAP-ARAP_13.05.2021_00h00	3.67E-55	14.036479	177	[ADAM10, ADNP, AGO2, ANXA7, APEX1, APLP2, APP, APPL2, ARRB1, ASB1, ATP2B1, C1QBP, CCN2, CD200, CDK1, CDK2, CENPV, CHID1, CIRBP, CNOT1, CNOT10, COLEC12, CTBP1, CTNNB1, DAPK1, DCPS, DHFR, DHX34, DHX36, DICER1, DNMT1, EED, EIF2AK2, EIF2S1, EIF3E, EIF6, EPHA2, EPRS1, EZR, FERMT1, FGF2, FMR1, FN1, FTO, GAPDH, GARS1, GBP1, GSTP1, H1-10, H1-5, HAT1, HDAC2, HELZ, HMOX1, HNRNPA0, HNRNPC, HNRNPR, IGF2BP1, IGF2BP2, ILF3, IQGAP3, IREB2, KCNQ1, LARP1, LARP4B, LRRC32, MACROH2A1, METTL14, MLH1, MORF4L1, MOV10, MPHOSPH8, MRTO4, MYADM, MYD88, NCL, NOCT, NPLOC4, NPM1, NT5C3B, NUDT16, NUDT16L1, NUP205, NUP35, NUP62, NUTF2, PAIP1, PARP14, PAWR, PC, PELO, PIN1, PML, POLR2A, POLR2B, POLR2H, POLR2I, PPM1B, PPP3CA, PRKAA1, PSMA3, PSMB10, PSMB2, PSMC2,	No change

					PSMC3, PSMC6, PSMD1, PSMD12, PSMD14, PSMD2, PSMD6, PSMD7, PSMD8, PSME1, PSME2, PSME4, PTBP2, PTBP3, PTRH2, PUS7, PYCARD, RBBP4, RBM4, RIF1, RNPS1, RPL10A, RPL13, RPL13A, RPL18A, RPL22, RPL24, RPL27, RPL32, RPL36, RPL38, RPL5, RPLP1, RPLP2, RPS13, RPS15A, RPS16, RPS18, RPS21, RPS23, RPS4Y1, RPS5, RPS8, RPS9, SARS1, SERPINB1, SERPINE2, SESN2, SET, SHFL, SHMT2, SIRT1, SMCHD1, SRSF10, SSB, TAX1BP1, TBK1, THBS1, TRIM25, TSPO, TUT4, UBE2L6, UBR2, UFD1, WDR35, WWP2, XIAP, YAP1, YBX1, ZC3HAV1, ZFP36L2, ZMPSTE24, ZNF148]	
regulation of cellular protein metabolic process	GO_BiologicalProcess-EBI-UniProt-GOA-ACAP-ARAP_13.05.2021_00h00	3.67E-55	11.001517	290	[AARS1, AASS, ABCE1, ABI1, ACO1, ACTB, ADARB1, ADNP, AGO2, APAF1, APLP2, APP, ARAF, ARFGEF1, ARPP19, ARRB1, ASPSCR1, BIN1, BOD1, BST1, C1QBP, CAB39L, CAMK2D, CASP3, CCDC22, CCN2, CCNH, CCNK, CCNL1, CD109, CD81, CDK5RAP3, CEMIP, CHAMP1, CHORDC1, CHP1, CHTOP, CIRBP, CLU, CNOT1, CNOT10, CORO1C, CRTAP, CSPG4, CSTB, CTBP1, CTNNB1, CTSC, CYCS, DAB2, DAPK1, DCPS, DDX1, DENR, DERL1, DHCR24, DHFR, DHX36, DIPK2A, DNMT1, DUSP3, DVL2, DYNLL1, EEF1G, EFL1, EIF2AK2, EIF2B1, EIF2D, EIF2S1, EIF3A, EIF3B, EIF3E, EIF3F, EIF4G3, EIF6, ELP3, ELP4, ENPP1, EPRS1, ERBB2, ETFDH, EZR, FASN, FBXW8, FERMT2, FGF2, FKBP1A, FMR1, FN1, FTO, G6PD, GAPDH, GIPC1, GLE1, GLMN, GLYR1, GNL3L, GPD1L, GPI, GSK3B,	No change

					GSTP1, H1-5, HDAC2, HDAC4, HGS, HIP1, HNRNPR, HPX, HSP90AA1, HSPA5, HSPE1, IGF2BP1, IGF2BP2, IKBKB, IKBKG, ILF3, IMPACT, IPO5, IQGAP3, IREB2, IST1, ITIH4, IVNS1ABP, KAT7, KDM1A, KDM4B, LARP1, LARP4B, LARP6, LLGL1, LRPPRC, LRRK1, MACROH2A1, MAD2L1, MALT1, MAP2K3, MAP3K20, MAP3K4, MAP4K4, MCM2, METTL14, MLH1, MOV10, MSN, MTA1, MTCH1, MVP, MYADM, MYH9, NCK2, NCL, NCOA7, NDUFA13, NF1, NF2, NIBAN1, NLE1, NNT, NOCT, NOP53, NPM1, NSMCE3, NSUN5, NT5C3B, NUB1, NUDT15, NUP62, NXN, OGFOD1, OSBPL8, P3H1, PACSIN3, PAIP1, PARG, PARP14, PEBP1, PELO, PIK3CA, PIN1, PITHD1, PKM, PKN2, PLD1, PLPP3, PLXNB2, PML, PPIB, PPP1CA, PPP1R14A, PPP1R7, PPP2R5A, PPP3CA, PPP5C, PRKAA1, PRKCSH, PSMC2, PSMC3, PSMC6, PSMD14, PSME1, PSME2, PSME4, PTPA, PUS7, PYCARD, QARS1, RAB1A, RAP2B, RAP2C, RBM4, RCN3, RDX, RIF1, RIPK2, RNF40, RPL13A, RPL22, RPL38, RPL5, RPLP1, RPS9, RYBP, SAE1, SARNP, SELENOT, SERPINB1, SERPINB2, SERPINB8, SERPINB9, SERPINE2, SESN2, SET, SHFL, SHMT2, SIRT1, SLC1A1, SLIT2, SMAD4, SMARCB1, SMARCC1, SMPD1, SMYD3, SNF8, SNW1, SOD1, SPHK2, SQSTM1, SRC, STAT2, STK24, SUFU, TAB2, TBC1D10A, TBK1, TF, THBS1, TIPRL, TMEM119, TNIK, TPX2, TRADD, TRIM21, TRIP12,	

					TRMT10C, TSC2, TSPO, TTLL12, TUT4, TXN, UACA, UBA2, UBXN2A, URI1, USP4, USP5, USP7, VDAC2, VLDLR, VPS25, VRK3, VTN, WDR35, WDR5, WDR70, WFS1, XIAP, YBX1, YES1, ZFP36L2, ZMPSTE24]	
negative regulation of cellular amide metabolic process	GO_BiologicalProcess-EBI-UniProt-GOA-ACAP-ARAP_13.05.2021_00h00	3.67E-55	19.28251	43	[AGO2, APEX1, BIN1, CIRBP, CLU, CNOT1, CNOT10, DAPK1, DCPS, DHFR, DHX36, EIF2AK2, EIF2S1, EIF3E, EIF6, EPRS1, FMR1, FTO, GAPDH, HNRNPR, IGF2BP1, IGF2BP2, ILF3, IREB2, LARP1, METTL14, MLH1, MOV10, NCL, NOCT, NT5C3B, PIN1, PRKAA1, PUS7, RBM4, RPL13A, RTN4, SESN2, SHFL, SHMT2, TUT4, YBX1, ZFP36L2]	Upregulation
negative regulation of protein metabolic process	GO_BiologicalProcess-EBI-UniProt-GOA-ACAP-ARAP_13.05.2021_00h00	3.67E-55	12.457045	145	[AASS, ACOT8, ADARB1, AGO2, APEX1, APLP2, APP, ARPP19, ARRB1, BIN1, BOD1, CASP3, CD109, CDK5RAP3, CHORDC1, CHP1, CIRBP, CLU, CNOT1, CNOT10, CORO1C, CRTAP, CSTB, CTBP1, CTNNB1, CTSA, CYP51A1, DAPK1, DCPS, DHCR24, DHFR, DHX36, DNMT1, DUSP3, EIF2AK2, EIF2S1, EIF3E, EIF6, ENPP1, EPRS1, FKBP1A, FMR1, FTO, G6PD, GAPDH, GIPC1, GLMN, GNL3L, GPD1L, GPI, GSK3B, GSTP1, HDAC2, HNRNPR, IGF2BP1, IGF2BP2, IKBKB, ILF3, IMPACT, IPO5, IREB2, ITGAV, ITIH4, ITM2C, IVNS1ABP, KDM1A, KDM4B, LARP1, LRRK1, MACROH2A1, MAD2L1, METTL14, MLH1, MOV10, MVP, MYADM, NCK2, NCL, NCOA7, NF1, NF2, NIBAN1, NLE1, NNT, NOCT, NOP53, NPM1, NT5C3B, NUP62, NXN, P3H1, PARP14, PEBP1, PIN1, PKN2, PLPP3, PML, PPP1R14A, PPP5C, PRKAA1, PTPA, PUS7, PYCARD, QARS1, RBM4,	Upregulation

					RPL13A, RPL5, RTN4, RYBP, SERPINB1, SERPINB2, SERPINB8, SERPINB9, SERPINE2, SESN2, SET, SHFL, SHMT2, SIRT1, SLIT2, SMARCB1, SMARCC1, SMPD1, SMYD3, SPHK2, SQSTM1, SRC, SUFU, THBS1, TIPRL, TRIM21, TRIP12, TSC2, TSPO, TUT4, URI1, USP4, USP7, VDAC2, VPS25, VTN, WDR5, XIAP, YBX1, ZFP36L2]	
cellular protein modification process	GO_BiologicalProcess-EBI-UniProt-GOA-ACAP-ARAP_13.05.2021_00h00	3.67E-55	9.686678	405	[AASDHPPT, AASS, ABHD12, ABI1, ACTB, ADAM10, ADARB1, ADNP, AKR1B1, ALB, ALG11, APLP2, APP, ARAF, ARFGEF1, ARMT1, ARPP19, ARRB1, ASB1, ASH2L, ASPSCR1, ATE1, ATG7, AUP1, B3GAT3, B4GALT1, B4GALT5, BAZ1B, BCR, BOD1, BST1, C1GALT1, CAB39L, CAMK2D, CASK, CASP3, CBX8, CCDC22, CCN2, CCNH, CCNK, CCNL1, CD109, CD81, CDC34, CDC42BPA, CDC73, CDK1, CDK16, CDK17, CDK2, CDK5RAP3, CEMIP, CFAP20, CHAMP1, CHORDC1, CHP1, CHTOP, CLU, COG7, CORO1C, CRBN, CRTAP, CSPG4, CTBP1, CTNNB1, CTU1, CUL1, CUL4B, DAB2, DAPK1, DCN, DERL1, DGUOK, DHPS, DIPK2A, DLST, DNM1L, DNMT1, DSP, DUSP23, DUSP3, DVL2, DYNLL1, EED, EFEMP1, EIF2AK2, EIF2S1, EIF3F, ELP3, ELP4, ENPP1, ENY2, EOGT, EPHA2, EPHA3, EPHB4, ERBB2, ETFDH, EXTL2, FAM3A, FASN, FBXL12, FBXW8, FERMT2, FGF2, FKBP1A, FKBP5, FKBP9, FMR1, FN1, FN3K, FUT11, FUT8, G6PD, GALNT1, GALNT2, GALNT7, GAN, GAPDH, GARS1, GFPT2, GLMN, GLUL,	Upregulation

					GLYR1, GNL3L, GOLGA7, GPD1L, GPHN, GPI, GSK3B, GSTP1, GTF3C4, GXYLT1, GXYLT2, H1-5, HAT1, HDAC2, HDAC4, HECTD4, HERC2, HERC4, HGS, HLA-A, HMBS, HMCES, HPX, HSP90AA1, HSP90B1, HSPA5, IGFBP7, IKBKB, IKBKG, ILF3, ILKAP, IMPACT, IPO5, IQGAP3, IVNS1ABP, KAT7, KAT8, KDM1A, KDM3B, KDM4B, LAMC1, LLGL1, LMO7, LNPEP, LRRK1, LTBP1, LYPLA2, MACROD1, MACROH2A1, MAD2L1, MAEA, MAGI1, MALT1, MAP2K3, MAP3K20, MAP3K4, MAP4K4, MARCHF5, MBD3, MCM2, MED30, MGAT1, MMAB, MOGS, MORF4L1, MTA1, MVD, MVP, MYADM, N6AMT1, NCK2, NCOA7, NDUFAF7, NEDD4, NEDD8, NEK1, NF1, NF2, NIBAN1, NMT1, NNT, NOP53, NPM1, NPR2, NSMCE3, NUB1, NUP205, NUP35, NUP62, NXN, OBSL1, OGFOD1, OSBPL8, OTUD6B, OXSR1, P3H1, P3H3, PARG, PARP1, PARP14, PARP4, PDK1, PDZRN3, PGAM5, PGM3, PGP, PHKB, PHPT1, PIGO, PIGT, PIGU, PIK3CA, PIN1, PKN2, PLOD2, PLPP3, PLXNB2, PML, PMM2, POFUT1, POGLUT3, POMGNT1, POMGNT2, POMK, PPIB, PPIE, PPIG, PPM1B, PPP1CA, PPP1R14A, PPP1R7, PPP2R5A, PPP3CA, PPP5C, PPWD1, PRKAA1, PRKAB2, PRKCI, PRKCSH, PRKG1, PRMT7, PRPF19, PSMA3, PSMB10, PSMB2, PSMC2, PSMC3, PSMC6, PSMD1, PSMD12, PSMD14, PSMD2, PSMD6, PSMD7, PSMD8, PSME1, PSME2, PSME4, PTK7, PTPA, PTPMT1, PTPN12,	

					PTPRF, PTPRG, PXK, PYCARD, QARS1, QSOX1, RAB11B, RAB1A, RAB3D, RAB5A, RAB8A, RANGAP1, RAP2B, RAP2C, RCN1, RIF1, RIOK3, RIPK2, RNF114, RNF2, RNF213, RNF214, RNF40, RNGTT, ROR1, RPL5, RPLP1, RPRD1A, RPRD1B, RSPRY1, RYBP, SAE1, SENP3, SESN2, SET, SHMT2, SHPRH, SIRT1, SLC1A1, SLIT2, SMAD4, SMARCAD1, SMARCB1, SMPD1, SMYD3, SNF8, SNW1, SOD1, SPHK2, SQSTM1, SRC, SRD5A3, STAT2, STK24, TAB2, TAF6L, TBK1, TF, TGFBR3, TGM2, THBS1, TIPRL, TMEM119, TMX3, TNIK, TPP2, TPST2, TPX2, TRIM21, TRIM25, TRIM28, TRIM56, TRIM59, TRIO, TRIP12, TSC2, TSPO, TTLL12, TXN, UBA2, UBA3, UBE2L6, UBE2Q1, UBR2, UBR4, UBXN2A, UFD1, UHRF2, URI1, URM1, USP11, USP22, USP28, USP4, USP48, USP5, USP7, USP9X, USP9Y, UST, VDAC2, VLDLR, VPS25, VRK1, VRK3, VTN, WDR5, WDR70, WFS1, WIPI2, WWP2, XIAP, YEATS4, YES1, YOD1, ZC3HAV1, ZDHHC13, ZDHHC21, ZMPSTE24]	
RNA metabolic process	GO_BiologicalProcess-EBI-UniProt-GOA-ACAP-ARAP_13.05.2021_00h00	3.67E-55	9.09848	437	[AAR2, AARS1, AASS, ABCE1, ABT1, ACTR2, ACTR3, ADARB1, ADNP, AGO2, AGRN, AKR1B1, ANXA3, APBB2, APEX1, APP, AQR, ARHGEF11, ARID1B, ARRB1, BAZ1B, BCAS2, BMS1, BRD3, C1QBP, CAMK2D, CASK, CBX1, CBX2, CBX3, CBX5, CBX8, CC2D1A, CCDC22, CCN2, CCNH, CCNK, CCNL1, CD200, CD81, CDC5L, CDC73, CDK1, CDK16, CDK17, CDK5RAP3, CELF2, CENPV, CHD2, CHD4, CHD7, CHORDC1,	Upregulation

					CHP1, CHTOP, CIRBP, CLU, CMTR1, CNGB1, CNOT1, CNOT10, COIL, COLEC12, CPSF2, CRABP2, CSTF3, CTBP1, CTNNB1, CTNNBL1, CTU1, CUX1, CWC22, DAB2, DARS1, DCPS, DDX1, DDX42, DDX47, DHFR, DHX34, DHX36, DICER1, DIMT1, DNMT1, DRG1, DVL2, EARS2, EDF1, EED, EEF1E1, EFEMP1, EIF2AK2, EIF3E, EIF6, ELP3, ELP4, ELP5, EMG1, ENPP1, ENY2, EPRS1, ERBB2, EXD2, EZR, FEN1, FGF2, FHL2, FHOD1, FMR1, FTO, GARS1, GATAD2A, GATB, GCFC2, GEMIN4, GLMP, GLO1, GLYR1, GPI, GPKOW, GTF2E1, GTF2F1, GTF2F2, GTF2I, GTF3C3, GTF3C4, H1-10, H1-5, HARS1, HAT1, HDAC2, HDAC4, HDGFL2, HEXB, HINT1, HMOX1, HNRNPA0, HNRNPA3, HNRNPC, HNRNPH1, HNRNPLL, HNRNPR, HNRNPUL1, HSD17B10, HSD17B4, HSPA5, HTATSF1, IARS2, IGF2BP1, IGF2BP2, IKBKB, IKBKG, ILF3, IMPACT, INTS1, INTS9, IVNS1ABP, JAG1, KAT7, KAT8, KDM1A, KDM3B, LANCL2, LARP1, LARP4B, LARP6, LIMS1, LMCD1, LMO7, LRPPRC, LRRFIP1, LUM, MACROH2A1, MAGI1, MALT1, MAP2K3, MBD3, MCM2, MED17, MED23, MED30, METTL14, MGA, MLF2, MLH1, MORF4L1, MOV10, MPHOSPH8, MRM3, MRTO4, MTA1, MTREX, MYD88, MYO6, NCK2, NCL, NCOA5, NCOA7, NDRG1, NDUFA13, NEDD4, NEDD8, NELFCD, NFATC2IP, NFKB2, NIBAN2, NMD3, NOCT, NOL11, NOP53, NPM1, NSUN5,	

					NT5C3B, NUDT16, NUDT16L1, NUP35, NUP62, OXSR1, PAIP1, PARP1, PARP14, PATZ1, PAWR, PBXIP1, PCBP3, PELO, PFKM, PIN1, PIR, PITHD1, PLPP3, PML, POFUT1, POGZ, POLR1C, POLR1D, POLR2A, POLR2B, POLR2H, POLR2I, POLR3A, POLR3C, POP1, PPIE, PPIG, PPP3CA, PPP5C, PPWD1, PRDX6, PRIM2, PRKAA1, PRKCI, PRMT7, PRPF19, PSIP1, PSMA3, PSMB10, PSMB2, PSMC2, PSMC3, PSMC6, PSMD1, PSMD12, PSMD14, PSMD2, PSMD6, PSMD7, PSMD8, PSME1, PSME2, PSME4, PSPC1, PTBP2, PTBP3, PTGES2, PTGIS, PTRH2, PUF60, PUS7, PXN, PYCARD, QARS1, QTRT1, QTRT2, RAD21, RAP2C, RARS2, RBM22, RBM26, RBM4, RBMS2, RIF1, RIOK3, RIPK2, RNF2, RNF40, RNGTT, RNPS1, ROR1, RPF2, RPL10A, RPL13, RPL13A, RPL18A, RPL22, RPL24, RPL27, RPL32, RPL36, RPL38, RPL5, RPLP1, RPLP2, RPRD1A, RPRD1B, RPRD2, RPS13, RPS15A, RPS16, RPS18, RPS21, RPS23, RPS4Y1, RPS5, RPS8, RPS9, RYBP, SAMHD1, SAP30BP, SARNP, SARS1, SBDS, SCAF11, SCAI, SEC14L2, SENP3, SESN2, SET, SF1, SF3A3, SF3B6, SIRT1, SLC25A15, SLC30A9, SLC9A1, SMAD4, SMARCA1, SMARCB1, SMARCC1, SMARCD3, SMARCE1, SMCHD1, SMNDC1, SMU1, SMYD3, SNF8, SNRNP200, SNRPA, SNRPD3, SNRPE, SNU13, SNW1, SNX5, SOD2, SON, SORBS3, SQSTM1, SRA1, SRC, SRSF10, SRSF2, SRSF5, SSB,	

					SSRP1, STAT1, STAT2, STAT5B, STRN3, SUFU, SUPT16H, TAB2, TAF6L, TAX1BP1, TBK1, TBPL1, TDRD3, TF, TFAM, TGS1, THADA, THOC2, TRA2A, TRA2B, TRADD, TRIM21, TRIM25, TRIM28, TRIM56, TRIP13, TRMT10C, TRMT2A, TRMT5, TSEN15, TSEN34, TSR2, TTC5, TUT4, TXN, U2SURP, UBA2, UBA3, UBR2, UBTF, URI1, URM1, USP22, USP4, USP7, USP9X, UTP14A, UTP20, UTP3, VARS1, WDR12, WFS1, WWC2, WWP2, YAP1, YARS1, YBEY, YBX1, YEATS4, YES1, YTHDC1, ZC3HAV1, ZFP36L2, ZMPSTE24, ZNF148]	
negative regulation of cellular protein metabolic process	GO_BiologicalProcess-EBI-UniProt-GOA-ACAP-ARAP_13.05.2021_00h00	3.67E-55	12.477064	136	[AASS, ADARB1, AGO2, APLP2, APP, ARPP19, ARRB1, BIN1, BOD1, CASP3, CD109, CDK5RAP3, CHORDC1, CHP1, CIRBP, CNOT1, CNOT10, CORO1C, CRTAP, CSTB, CTBP1, CTNNB1, DAPK1, DCPS, DHCR24, DHFR, DHX36, DNMT1, DUSP3, EIF2AK2, EIF2S1, EIF3E, EIF6, ENPP1, EPRS1, FKBP1A, FMR1, FTO, G6PD, GAPDH, GIPC1, GLMN, GNL3L, GPD1L, GPI, GSK3B, GSTP1, HDAC2, HNRNPR, IGF2BP1, IGF2BP2, IKBKB, ILF3, IMPACT, IPO5, IREB2, ITIH4, IVNS1ABP, KDM1A, KDM4B, LARP1, LRRK1, MACROH2A1, MAD2L1, METTL14, MLH1, MOV10, MVP, MYADM, NCK2, NCL, NCOA7, NF1, NF2, NIBAN1, NLE1, NNT, NOCT, NOP53, NPM1, NT5C3B, NUP62, NXN, P3H1, PARP14, PEBP1, PKN2, PLPP3, PML, PPP1R14A, PPP5C, PRKAA1, PTPA, PUS7, PYCARD, QARS1, RBM4, RPL13A, RPL5, RYBP, SERPINB1,	Upregulation

					SERPINB2, SERPINB8, SERPINB9, SERPINE2, SESN2, SET, SHFL, SHMT2, SIRT1, SLIT2, SMARCB1, SMARCC1, SMPD1, SMYD3, SPHK2, SQSTM1, SRC, SUFU, THBS1, TIPRL, TRIM21, TRIP12, TSC2, TSPO, TUT4, URI1, USP4, USP7, VDAC2, VPS25, VTN, WDR5, XIAP, YBX1, ZFP36L2]	
RNA processing	GO_BiologicalProcess-EBI-UniProt-GOA-ACAP-ARAP_13.05.2021_00h00	3.67E-55	15.844402	167	[AAR2, AARS1, ABT1, ADARB1, AGO2, APP, AQR, BCAS2, BMS1, C1QBP, CCNH, CCNL1, CDC5L, CDC73, CELF2, CHD7, CHTOP, CIRBP, CMTR1, CNGB1, COIL, COLEC12, CPSF2, CSTF3, CTNNBL1, CTU1, CWC22, DCPS, DDX1, DDX42, DDX47, DHX36, DICER1, DIMT1, EIF6, ELP3, ELP4, ELP5, EMG1, FMR1, GARS1, GCFC2, GEMIN4, GPKOW, GTF2F1, GTF2F2, HNRNPA0, HNRNPA3, HNRNPC, HNRNPH1, HNRNPLL, HNRNPR, HNRNPUL1, HSD17B10, HTATSF1, INTS1, INTS9, IVNS1ABP, KDM1A, LARP6, MCM2, METTL14, MRM3, MRTO4, MTREX, NCL, NEDD4, NOL11, NOP53, NPM1, NSUN5, NUDT16, PIN1, POLR2A, POLR2B, POLR2H, POLR2I, POP1, PPIE, PPIG, PPP3CA, PPWD1, PRDX6, PRMT7, PRPF19, PSIP1, PSPC1, PTBP2, PTBP3, PUF60, PUS7, QTRT1, QTRT2, RBM22, RBM26, RBM4, RBMS2, RIOK3, RNF40, RNGTT, RNPS1, RPF2, RPL10A, RPL13, RPL13A, RPL18A, RPL22, RPL24, RPL27, RPL32, RPL36, RPL38, RPL5, RPLP1, RPLP2, RPRD1A, RPRD1B, RPRD2, RPS13, RPS16, RPS21, RPS8, RPS9,	Upregulation

					SARNP, SARS1, SBDS, SCAF11, SENP3, SF1, SF3A3, SF3B6, SMNDC1, SMU1, SNRNP200, SNRPA, SNRPD3, SNRPE, SNU13, SNW1, SON, SRSF10, SRSF2, SRSF5, SSB, TGS1, THADA, THOC2, TRA2A, TRA2B, TRMT10C, TRMT2A, TRMT5, TSEN15, TSEN34, TSR2, TUT4, U2SURP, URM1, USP4, UTP14A, UTP20, UTP3, WDR12, YBEY, YBX1, YTHDC1, ZMPSTE24]	
mRNA metabolic process	GO_BiologicalProcess-EBI-UniProt-GOA-ACAP-ARAP_13.05.2021_00h00	3.67E-55	17.900553	162	[AAR2, ADARB1, AGO2, APEX1, APP, AQR, BCAS2, C1QBP, CCNH, CDC5L, CDC73, CELF2, CHTOP, CIRBP, CMTR1, CNOT1, CNOT10, COIL, COLEC12, CPSF2, CSTF3, CTNNBL1, CWC22, DCPS, DDX1, DDX42, DDX47, DHX34, DHX36, EIF3E, FMR1, FTO, GCFC2, GEMIN4, GPKOW, GTF2F1, GTF2F2, HNRNPA0, HNRNPA3, HNRNPC, HNRNPH1, HNRNPLL, HNRNPR, HNRNPUL1, HTATSF1, IGF2BP1, IGF2BP2, KDM1A, LARP1, LARP4B, METTL14, MLH1, MOV10, MRTO4, MTREX, MYD88, NCL, NOCT, NPM1, NT5C3B, NUDT16, NUDT16L1, PAIP1, PCBP3, PELO, PIN1, POLR2A, POLR2B, POLR2H, POLR2I, PPIE, PPWD1, PRDX6, PRMT7, PRPF19, PSIP1, PSMA3, PSMB10, PSMB2, PSMC2, PSMC3, PSMC6, PSMD1, PSMD12, PSMD14, PSMD2, PSMD6, PSMD7, PSMD8, PSME1, PSME2, PSME4, PSPC1, PTBP2, PTBP3, PUF60, PUS7, RBM22, RBM26, RBM4, RNF40, RNGTT, RNPS1, RPL10A, RPL13, RPL13A, RPL18A, RPL22, RPL24, RPL27, RPL32, RPL36, RPL38, RPL5, RPLP1, RPLP2, RPRD1A, RPRD1B,	Upregulation

					RPRD2, RPS13, RPS15A, RPS16, RPS18, RPS21, RPS23, RPS4Y1, RPS5, RPS8, RPS9, SARNP, SCAF11, SET, SF1, SF3A3, SF3B6, SMNDC1, SMU1, SNRNP200, SNRPA, SNRPD3, SNRPE, SNU13, SNW1, SON, SRSF10, SRSF2, SRSF5, SSB, TGS1, THOC2, TRA2A, TRA2B, TRMT10C, TSEN15, TSEN34, TUT4, U2SURP, USP4, YBX1, YTHDC1, ZC3HAV1, ZFP36L2]	
negative regulation of protein modification process	GO_BiologicalProcess-EBI-UniProt-GOA-ACAP-ARAP_13.05.2021_00h00	3.67E-55	12.629758	73	[AASS, ADARB1, ARPP19, ARRB1, BOD1, CASP3, CD109, CDK5RAP3, CHORDC1, CHP1, CORO1C, CRTAP, CTBP1, CTNNB1, DNMT1, DUSP3, ENPP1, FKBP1A, G6PD, GLMN, GNL3L, GPD1L, GSK3B, GSTP1, HDAC2, IKBKB, IMPACT, IPO5, IVNS1ABP, KDM1A, KDM4B, LRRK1, MACROH2A1, MAD2L1, MVP, MYADM, NCK2, NCOA7, NF1, NF2, NIBAN1, NNT, NPM1, NUP62, NXN, P3H1, PARP14, PKN2, PLPP3, PPP1R14A, PPP5C, PRKAA1, PTPA, PYCARD, QARS1, RPL5, SET, SIRT1, SLIT2, SMARCB1, SMPD1, SMYD3, SPHK2, SQSTM1, TIPRL, TRIM21, TRIP12, TSC2, TSPO, URI1, USP4, VPS25, WDR5]	Upregulation
negative regulation of translation	GO_BiologicalProcess-EBI-UniProt-GOA-ACAP-ARAP_13.05.2021_00h00	3.67E-55	18.686869	37	[AGO2, CIRBP, CNOT1, CNOT10, DAPK1, DCPS, DHFR, DHX36, EIF2AK2, EIF2S1, EIF3E, EIF6, EPRS1, FMR1, FTO, GAPDH, HNRNPR, IGF2BP1, IGF2BP2, ILF3, IREB2, LARP1, METTL14, MLH1, MOV10, NCL, NOCT, NT5C3B, PUS7, RBM4, RPL13A, SESN2, SHFL, SHMT2, TUT4, YBX1, ZFP36L2]	Upregulation
cell cycle process	GO_BiologicalProcess-EBI-UniProt-GOA-ACAP-	2.75E-67	12.638889	182	[ACTR2, ACTR3, AHCTF1, ANK3, APAF1, APEX1, APLP2, APP, APPL2,	Upregulation

	ARAP_13.05.2021_00h00				ARPP19, ASPM, BANF1, BCAT1, BOD1, CBX3, CBX5, CCN2, CCNH, CDC34, CDC5L, CDC73, CDK1, CDK16, CDK17, CDK2, CDK5RAP3, CECR2, CENPV, CETN2, CHAMP1, CHMP3, CHORDC1, CLTC, CNOT1, CNOT10, CTNNB1, CUL1, CUL4B, DHFR, DRG1, DYNC1LI1, DYNLL1, EED, EML1, EML3, EML4, ERCC6L, EZR, FBXL12, FEN1, FHL1, FSD1, GIPC1, HSP90AA1, IQGAP3, IST1, ITGB1, KNTC1, KPNB1, LBR, LEMD2, LIG1, LLGL1, MACROH2A1, MAD1L1, MAD2L1, MAP3K20, MCM2, MCM3, MCM4, MCM5, MCM6, MCM7, MCMBP, MDC1, MGA, MLH1, MMAB, MSH2, MSH6, MYH9, NCAPD2, NCAPG, NCAPH, NDRG1, NEDD1, NES, NOP53, NPM1, NUBP2, NUDT16, NUF2, NUP205, NUP35, NUP62, OBSL1, PAFAH1B1, PDCD6IP, PDS5B, PHGDH, PIN1, PKN2, PML, POGZ, POLD1, POLD2, PPP3CA, PRIM2, PRPF19, PSMA3, PSMB10, PSMB2, PSMC2, PSMC3, PSMC6, PSMD1, PSMD12, PSMD14, PSMD2, PSMD6, PSMD7, PSMD8, PSME1, PSME2, PSME4, PTPA, RAD21, RAD9A, RANBP1, RANGAP1, RBBP4, RCC1, RDX, RFC2, RFC3, RFC5, RHOB, RHOC, RNF2, RPA1, RPA2, RPL24, RPRD1B, RTTN, RYBP, SBDS, SEPTIN10, SEPTIN11, SEPTIN2, SEPTIN7, SEPTIN8, SEPTIN9, SET, SIRT1, SLC25A15, SMARCAD1, SMARCD3, SMC1A, SMC2, SMC3, SMC4, SNX18, SON, STAG2, STON1, SVIL, TBCE, TIPRL, TOM1L2, TPX2, TRIP13, TUBA4A, TUBB,	

					TUBGCP2, UBR2, USP28, VRK1, WASHC5, WRAP73, ZFP36L2, ZMPSTE24, ZNF207]	
regulation of localization	GO_BiologicalProcess-EBI-UniProt-GOA-ACAP-ARAP_13.05.2021_00h00	2.75E-67	10.101719	288	[ABAT, ACSL3, ACSL4, ACTB, ACTG1, ADAM10, ADAMTS9, ADARB1, AFDN, AGO2, AKAP12, AKR1B1, AMOTL1, ANK3, ANKFY1, ANKRD13A, ANO6, ANXA3, APEX1, APLP2, APP, APPL2, ARFGAP1, ARFIP1, ARPIN, ARPP19, ARRB1, ASPSCR1, ATP1A1, ATP1B3, ATP2B1, ATP2C1, B2M, B3GAT3, BIN1, BST1, C1QBP, C2CD2L, CAMK2D, CASK, CCT2, CCT3, CCT6A, CD200, CD81, CDK1, CDK16, CDK5RAP3, CEMIP, CFAP20, CFAP298, CHD7, CHMP3, CHORDC1, CHP1, CLIC1, CLTC, CLTCL1, CLU, CORO1B, CORO1C, CRBN, CTNNB1, CXCL12, CYP51A1, CYRIB, DAB2, DAPK1, DHX36, DIAPH1, DNM1L, DPYSL2, DPYSL3, DSP, DUSP3, DYNLL1, EHD3, ELP3, ELP5, ENPP1, ENY2, EPB41L2, EPHA2, EPHA3, EPPK1, EPRS1, ERBB2, EZR, FERMT1, FERMT2, FGF2, FHL1, FKBP1A, FLRT2, FMR1, FN1, FTO, G6PD, GAB1, GBP1, GCC2, GIPC1, GLUD1, GLUL, GNL3L, GPC1, GPC6, GPD1L, GPI, GSK3B, GSTM2, GSTP1, HADH, HADHA, HDAC4, HGS, HIP1, HK2, HMOX1, HNRNPC, HSP90AA1, HSPA5, IDH2, IPO5, ITGA3, ITGAV, ITGB1, ITSN1, JAG1, JAGN1, JAM3, KAT7, KCNMA1, KCNQ1, LAMA5, LAMP1, LLGL1, LRRC8A, MACF1, MACROH2A1, MAD1L1, MAGI1, MAP1B, MAP2K3, MAP4K4, MCAM, MEAK7, MEST, MFF, MSN, MYADM, MYD88, MYH10, MYO18A, MYO1E, MYO6, NDUFA4, NEDD4, NF1, NF2,	No change

					NISCH, NMD3, NMT1, NPM1, NUP62, NUTF2, OSBPL11, OSBPL8, OXSR1, P3H1, PACSIN3, PARL, PARP1, PATZ1, PCK2, PDCD6IP, PFKM, PHPT1, PIGU, PIK3C2A, PIN1, PKN2, PLA2G4A, PLCG1, PLD1, PLPP3, PLXNB2, PML, PPP2R5A, PPP3CA, PRKAA1, PRKCI, PRKCSH, PRKG1, PTGES2, PTPRG, PTRH2, PXK, PYCARD, RAB10, RAB11B, RAB23, RAB3B, RAB3D, RAB5A, RAB8A, RALA, RANGAP1, RAP2B, RAP2C, RBM22, RBM4, RDX, REEP2, REEP6, RHOB, RHOC, RIPK2, RNF2, RRAS2, RTN4, SAE1, SCAI, SCFD1, SCP2, SCRIB, SELENOT, SEMA3C, SEPTIN2, SERPINE2, SESN2, SESTD1, SIRT1, SLC12A2, SLC1A1, SLC25A5, SLC2A1, SLC9A1, SLIT2, SMAD4, SMPD1, SNF8, SNX5, SOD1, SOD2, SPARC, SPHK2, SQSTM1, SRC, STK24, STOM, STON1, STXBP3, SUFU, SYNE2, SYNJ1, TBC1D4, TCAF1, TCP1, TF, TGFBR3, TGM2, THADA, THBS1, TJP1, TM9SF4, TOMM7, TRADD, TRIM21, TRIM25, TRIM28, TRIP6, TSC2, TSPO, TTC39B, TXN, UBAC2, USP7, VDAC1, VTN, WDR1, WFS1, WWP2, XPO4, YAP1, YES1, YOD1, ZMPSTE24]	
mitotic cell cycle	GO_BiologicalProcess-EBI-UniProt-GOA-ACAP-ARAP_13.05.2021_00h00	2.75E-67	13.886384	154	[AHCTF1, ANK3, APEX1, APLP2, APP, APPL2, ARPP19, ASNS, BANF1, BCAT1, BOD1, CCNH, CDC34, CDC5L, CDC73, CDK1, CDK16, CDK17, CDK2, CDK5RAP3, CETN2, CHAMP1, CHMP3, CLTC, CLTCL1, CNOT1, CNOT10, CTNNB1, CUL1, CUL4B, DHFR, DRG1, DUSP3, DYNC1LI1, DYNLL1, EML1, EML3, EML4, ERCC6L, FBXL12,	Upregulation

					FHL1, FSD1, HSP90AA1, IQGAP3, IST1, ITGB1, KIF15, KNTC1, KPNB1, LBR, LEMD2, LIG1, MACROH2A1, MAD1L1, MAD2L1, MAEA, MAP3K20, MCM2, MCM3, MCM4, MCM6, MCM7, MDC1, MSH2, NCAPD2, NCAPG, NCAPH, NDRG1, NEDD1, NES, NLE1, NOP53, NUDT15, NUF2, NUP205, NUP35, NUP62, OBSL1, PAFAH1B1, PDCD6IP, PDS5B, PIN1, PKN2, PML, POGZ, PPP3CA, PPP5C, PSMA3, PSMB10, PSMB2, PSMC2, PSMC3, PSMC6, PSMD1, PSMD12, PSMD14, PSMD2, PSMD6, PSMD7, PSMD8, PSME1, PSME2, PSME4, PTGES2, PTPA, RAD21, RAD9A, RANBP1, RANGAP1, RCC1, RDX, RHOB, RHOC, RNF2, RNF40, RPA2, RPL24, RRM1, SBDS, SCRIB, SET, SIRT1, SLC25A15, SMARCD3, SMC1A, SMC2, SMC3, SMC4, SNX18, SON, SRA1, SRSF2, STAG2, STAT5B, SUGT1, TBCE, TOM1L2, TPX2, TRIP13, TTC28, TTLL12, TUBA4A, TUBA8, TUBB, TUBB3, TUBGCP2, UBA3, USP22, VRK1, WRAP73, YEATS4, ZFP36L2, ZMPSTE24, ZNF207]	
polysome	GO_CellularComponent-EBI-UniProt-GOA-ACAP-ARAP_13.05.2021_00h00	2.75E-67	24.390244	20	[AGO2, DRG1, EIF2S1, FMR1, IMPACT, LARP1, LARP4B, LARP6, MSI2, MYH10, NDRG1, RPL10A, RPL18A, RPL24, RPL32, RPL36, RPL38, RPS21, RPS23, RPS4Y1]	Downregulation
transport	GO_BiologicalProcess-EBI-UniProt-GOA-ACAP-ARAP_13.05.2021_00h00	2.75E-67	10.040908	540	[ABAT, ABCB7, ABCC1, ABCE1, ABI1, ACACA, ACAP2, ACO1, ACSL3, ACSL4, ACTB, ACTG1, ACTR2, ACTR3, ADAM10, ADAMTS9, ADD3, AGPAT2, AHCTF1, ALB, ALDH3A2, ALDOA, ANK3, ANKFY1, ANKH, ANKRD13A,	No change

					ANO6, ANXA3, ANXA5, AP3M2, AP5B1, APAF1, APEX1, APLP2, APP, APPL2, APRT, ARFGAP1, ARFGEF1, ARFIP1, ARHGAP12, ARHGAP17, ARPC4, ARPP19, ARRB1, ASAH1, ASPSCR1, ATG4B, ATG7, ATL3, ATP1A1, ATP1B3, ATP2B1, ATP2B2, ATP2C1, ATP6V0A1, ATP6V0C, ATP6V1A, ATP6V1B2, AUP1, B2M, B3GAT3, B4GALT1, BAIAP2, BANF1, BET1, BIN1, BNIP1, BST1, C2CD2L, CAMK2D, CANX, CAP1, CAPZA2, CASK, CCDC22, CCN2, CCT2, CCT3, CD109, CD200, CD81, CDK1, CDK16, CECR2, CEMIP, CETN2, CHAMP1, CHD7, CHID1, CHMP3, CHORDC1, CHP1, CHTOP, CLIC1, CLTC, CLTCL1, CLU, CNGB1, COG6, COG7, COLEC12, CORO1C, CORO7, COX4I1, COX6C, CPSF2, CRBN, CROT, CSTB, CTNNB1, CTSA, CTSC, CUX1, CXCL12, CYB5R3, CYP51A1, CYRIB, DAB2, DAPK1, DENND2A, DERL1, DIAPH1, DNM1L, DPCD, DPYSL2, DSP, DST, DYNC1LI1, DYNLL1, EHD3, EIF2AK2, EIF2D, EIF2S1, EIF6, ENPP1, ENY2, EPHA3, EPRS1, ERBB2, ERLIN2, ERP44, ESYT1, EXOC2, EZR, FAM91A1, FERMT1, FGF2, FHL1, FHOD1, FKBP1A, FMR1, FN1, FNBP1L, FTH1, FTL, G6PD, GCC2, GDAP1, GEMIN4, GET3, GGA2, GIPC1, GLA, GLE1, GLUD1, GLUL, GOLGA7, GOLT1B, GPD1L, GPI, GRAMD1A, GSDMD, GSK3B, GSTM2, GSTP1, GULP1, HACL1, HADH, HADHA, HEATR3, HERC2, HEXB, HGS, HIP1, HK2, HLA-B, HLA-C, HMOX1, HNRNPA3, HNRNPC, HPX, HSP90AA1, HSP90B1, HSPA14, HSPA4, HSPA5,	

					HSPB11, HSPG2, IDH2, IFT140, IGF2BP1, IGF2BP2, IPO5, IPO9, IREB2, IST1, ITGAV, ITGB1, ITIH4, ITM2C, ITSN1, JAGN1, KCNMA1, KCNQ1, KIF15, KPNB1, KRT18, LAMP1, LGALS3BP, LIN7C, LLGL1, LRP10, LRPPRC, LRRC8A, LRRC8E, M6PR, MACF1, MAGED2, MAGI1, MALT1, MAP1B, MAP4K4, MFF, MFSD10, MGST1, MICAL3, MICALL1, MME, MSN, MTCH1, MVP, MYD88, MYH10, MYH9, MYO18A, MYO1B, MYO1D, MYO1E, MYO6, NCKAP1, NDUFA13, NDUFA4, NEDD4, NHLRC2, NIBAN2, NISCH, NMD3, NMT1, NNT, NPLOC4, NPM1, NUDT19, NUP205, NUP35, NUP62, NUTF2, OSBPL10, OSBPL11, OSBPL1A, OSBPL2, OSBPL8, OXA1L, OXSR1, P3H1, PACC1, PACSIN3, PAFAH1B1, PAFAH1B2, PANX1, PARL, PCDH7, PCK2, PDAP1, PDCD6IP, PDZD11, PFKM, PHPT1, PIEZO1, PIK3C2A, PIK3CA, PIN1, PIP4K2B, PITRM1, PKM, PLA2G4A, PLCG1, PLD1, PLPP3, PML, PNP, PPIE, PPP1R7, PPP3CA, PRDX6, PRKAA1, PRKAB2, PRKCI, PRKCSH, PRKG1, PSIP1, PSMA3, PSMB10, PSMB2, PSMC2, PSMC3, PSMC6, PSMD1, PSMD12, PSMD14, PSMD2, PSMD6, PSMD7, PSMD8, PSME1, PSME2, PSME4, PTGES2, PTRH2, PXK, PYCARD, PYGB, QSOX1, RAB10, RAB11B, RAB1A, RAB23, RAB3B, RAB3D, RAB5A, RAB8A, RABGEF1, RALA, RANBP1, RANGAP1, RAP2B, RAP2C, RBM22, RBM4, RCN3, RDX, REEP2, REEP6, RELCH, RHOB, RIN1, RIPK2, RNF2, RNPS1,	

					RPL10A, RPL13, RPL13A, RPL18A, RPL22, RPL24, RPL27, RPL32, RPL36, RPL38, RPL5, RPLP1, RPLP2, RPS13, RPS15A, RPS16, RPS18, RPS21, RPS23, RPS4Y1, RPS5, RPS8, RPS9, S100A10, SAE1, SARNP, SCARB2, SCFD1, SCP2, SCRIB, SCRN1, SEC14L2, SEC23B, SEC61A1, SEC61B, SELENBP1, SELENOT, SEPTIN2, SEPTIN8, SEPTIN9, SERPINB1, SERPINE2, SESN2, SESTD1, SFXN2, SFXN3, SH3BP4, SH3KBP1, SIRT1, SLC11A2, SLC12A2, SLC16A3, SLC17A5, SLC1A1, SLC1A4, SLC1A5, SLC25A10, SLC25A12, SLC25A13, SLC25A15, SLC25A19, SLC25A22, SLC25A24, SLC25A26, SLC25A4, SLC25A5, SLC25A6, SLC26A2, SLC2A1, SLC30A9, SLC31A1, SLC44A1, SLC44A2, SLC4A7, SLC7A6, SLC9A1, SMAD4, SMPD1, SNF8, SNRPD3, SNRPE, SNX1, SNX18, SNX21, SNX27, SNX30, SNX5, SOAT1, SOD1, SORT1, SPARC, SPHK2, SQSTM1, SRC, SRPRA, SRSF10, SRSF2, SRSF5, SSB, SSR3, STARD5, STAT5B, STOM, STON1, STXBP3, SUFU, SURF1, SYNE2, SYNJ1, TAGLN2, TAP1, TBC1D10A, TBC1D17, TBC1D22B, TBC1D4, TBC1D8B, TCAF1, TCP1, TF, TGM2, THADA, THBS1, THEM4, THOC2, TIMM17B, TJP2, TM9SF4, TMED9, TMX3, TNPO2, TOM1, TOM1L2, TOMM34, TOMM7, TRAM1, TRAM2, TRAPPC4, TRAPPC8, TRIM28, TSC2, TSPO, TTC39B, TUBA4A, TUBB, TXN, UBAC2, UBE2Q1, UBR4, UFD1, UMPS,	

					UNC119B, UNC93B1, USP7, VAT1, VDAC1, VDAC2, VLDLR, VPS25, VPS26B, VPS33B, VPS36, VPS41, VTN, WASHC5, WDR1, WDR11, WDR35, WFS1, WWP2, XPO4, XPO6, XPOT, YBX1, YES1, YKT6, YOD1, YTHDC1, ZDHHC13, ZDHHC21, ZFAND6, ZMPSTE24]	
protein localization	GO_BiologicalProcess-EBI-UniProt-GOA-ACAP-ARAP_13.05.2021_00h00	2.75E-67	13.374636	367	[ABAT, ACOT8, ACOX1, ACSL3, ACSL4, ACTB, ACTG1, ADAM10, ADAMTS9, AFDN, AGPS, AGRN, AHCTF1, AKR1B1, ALDH3A2, ANK3, ANKRD13A, AP3M2, AP5B1, APEX1, APLP2, APP, APPL2, ARFGAP1, ARFGEF1, ARFIP1, ARRB1, ASPSCR1, ATG4B, ATG7, ATP1B3, ATP2C1, ATP6V0A1, ATP6V0C, ATP6V1A, ATP6V1B2, AUP1, B3GAT3, BAIAP2, BET1, BOD1, C2CD2L, CANX, CCDC22, CCT2, CCT3, CCT6A, CD200, CD81, CDK1, CDK16, CDK5RAP3, CEMIP, CETN2, CHAF1B, CHAMP1, CHMP3, CHORDC1, CHP1, CLTC, CLTCL1, CLU, CNGB1, COG6, COG7, CORO1B, CORO1C, CORO7, CROT, CTNNB1, CTSA, CYP51A1, DAB2, DDX1, DDX42, DENND2A, DERL1, DHCR24, DIAPH1, DNM1L, DSP, DVL2, DYNLL1, ECI2, EHD3, EIF2D, EMC1, ENY2, EPB41L2, EPB41L3, EPHA2, EPHA3, ERBB2, EXOC2, EZR, FAM91A1, FERMT1, FERMT2, FKBP1A, GBP1, GCC2, GDAP1, GET3, GGA2, GIPC1, GLE1, GLMP, GLUD1, GLUL, GNL3L, GOLGA7, GOLT1B, GPC1, GPC6, GPD1L, GPHN, GSK3B, H1-5, HACL1, HADH, HADHA, HEATR3, HERC2, HGS, HIP1, HK1, HK2, HMOX1, HSD17B4, HSP90AA1, HSP90B1, HSPA4, HSPA5, HSPB11, IDH2, IFT140, IKBKB,	No change

					ILF3, IPO5, IPO9, IST1, ITGA3, ITGB1, ITSN1, JAGN1, JAM3, KAT7, KPNB1, KRT18, LAMA5, LAMP1, LEMD2, LIMS1, LIN7C, LLGL1, LRRC8A, LRWD1, LTBP1, M6PR, MACF1, MACROH2A1, MAGI1, MAP4K4, MARCHF5, MEAK7, MFF, MICALL1, MMAB, MSH2, MSN, MVP, MYADM, MYH10, MYH9, MYO18A, MYO1D, MYO1E, MYO6, NDUFA13, NEDD1, NEDD4, NEDD8, NF2, NMD3, NMT1, NOP53, NPLOC4, NPM1, NUBP2, NUDT19, NUP205, NUP35, NUP62, NUTF2, OBSL1, OSBPL8, OXA1L, P3H1, PACS1, PAFAH1B1, PARL, PARP1, PCK2, PDCD6IP, PDZD11, PFKM, PHPT1, PIGU, PIN1, PITRM1, PKN2, PML, PPP2R5A, PPP3CA, PRKAA1, PRKCI, PRPF19, PTGES2, RAB10, RAB11B, RAB1A, RAB23, RAB3B, RAB3D, RAB5A, RAB8A, RABGEF1, RAD21, RALA, RANGAP1, RBM22, RCN3, RDX, REEP2, RHOB, RNF2, RPA1, RPA2, RPF2, RPL10A, RPL13, RPL13A, RPL18A, RPL22, RPL24, RPL27, RPL32, RPL36, RPL38, RPL5, RPLP1, RPLP2, RPS13, RPS15A, RPS16, RPS18, RPS21, RPS23, RPS4Y1, RPS5, RPS8, RPS9, RTN4, S100A10, SAE1, SCARB2, SCFD1, SCP2, SCRIB, SEC14L2, SEC23B, SEC61A1, SEC61B, SELENBP1, SELENOT, SEPTIN10, SEPTIN11, SEPTIN2, SEPTIN7, SEPTIN8, SEPTIN9, SESN2, SH3BP4, SLC12A2, SLC1A1, SLC25A6, SLC2A1, SMYD3, SNF8, SNX1, SNX18, SNX21, SNX27, SNX30, SNX5, SORT1, SQSTM1, SRC, SRPRA, SSB, SSR3,	

					STOM, STXBP3, SUFU, TAP1, TBC1D10A, TBC1D17, TBC1D22B, TBC1D4, TBC1D8B, TCAF1, TCP1, TF, TIMM17B, TJP1, TJP2, TM9SF4, TMED9, TNIK, TNPO2, TOM1, TOM1L2, TOMM34, TOMM7, TOR1AIP1, TOR1AIP2, TOR1B, TPBG, TPP1, TRAM1, TRAM2, TRAPPC8, TRIM21, TRIM25, TRIM28, TSC2, TSPO, TWF2, TXN, UBAC2, UBE2Q1, UFD1, UNC119B, UNC93B1, USP4, USP9X, VLDLR, VPS25, VPS26B, VPS33B, VPS36, VPS41, WASHC5, WDR11, WDR35, WIPI2, WRAP73, WWP2, XPO4, XPO6, XPOT, YAP1, YBX1, YKT6, YOD1, ZDHHC21, ZFAND6]	
cellular catabolic process	GO_BiologicalProcess-EBI-UniProt-GOA-ACAP-ARAP_13.05.2021_00h00	2.75E-67	13.869794	343	[AASS, ABAT, ABCC1, ABHD12, ABHD16A, ACAD8, ACADM, ACADSB, ACADVL, ACAT2, ACOT7, ACOT8, ACOX1, ADAMTS9, AGO2, ALB, ALDH3A2, ANKZF1, ANXA7, APEX1, APP, ARAF, ARRB1, ASAH1, ATE1, ATG4B, ATG7, ATP6V0A1, ATP6V0C, ATP6V1A, ATP6V1B2, AUP1, BCAT1, BDH2, BLVRB, CASP3, CCDC22, CD81, CDC34, CDK1, CDK2, CDK5RAP3, CECR2, CHMP3, CIRBP, CLTC, CLU, CNOT1, CNOT10, CRBN, CROT, CRYZ, CSPG4, CTNNB1, CTSA, CTSC, CUL1, CUL4B, DAB2, DAGLB, DAPK1, DCN, DCPS, DCXR, DDI2, DERL1, DHX34, DHX36, DICER1, DLST, DNM1L, DPYSL2, DPYSL3, DPYSL4, DUT, DYNC1LI1, DYNLL1, ECE1, ECI2, EIF3E, EIF4G3, ENPP1, EPHX1, ERAP1, ERLIN2, ETFDH, EZR, FAM3A, FBXL12, FBXW8, FEN1, FMR1, FNBP1L, FTO, GAPDH, GBA2, GCAT, GCDH, GIPC1, GLA, GLMN, GLUD1,	No change

					GLUL, GOLT1B, GOT1, GPC1, GPD2, GPSM1, GRAMD1A, GSK3B, GSTM2, GSTM3, HACL1, HADH, HADHA, HEXB, HGS, HIBCH, HINT1, HK2, HMOX1, HNRNPA0, HNRNPC, HNRNPR, HSD17B10, HSD17B4, HSP90AA1, HSP90B1, HSPA5, IGF2BP1, IGF2BP2, IKBKG, ILVBL, KPNB1, LARP1, LARP4B, LNPEP, LRPPRC, LUM, LYPLA2, MAD2L1, MAEA, MAGI1, MALT1, MAOA, MAP1LC3A, METTL14, MFN1, MGAT1, MLH1, MME, MMUT, MOV10, MPST, MRTO4, MSN, MTA1, MTAP, MTCH1, MTREX, MTRR, MYD88, NEDD4, NEDD8, NNT, NOCT, NOP53, NPLOC4, NPM1, NT5C, NT5C3B, NUB1, NUDT15, NUDT16, NUDT16L1, NUDT19, OAT, PACSIN3, PAFAH1B2, PAIP1, PCK2, PDCD6IP, PDE5A, PDXDC1, PELO, PFKM, PGAM5, PGM2L1, PHGDH, PHKB, PIK3C2A, PIK3CA, PIP4K2B, PITHD1, PLA2G15, PLA2G4A, PLBD2, PLCG1, PML, PNP, PNPLA4, PNPLA6, POP1, PPP1CA, PRDX1, PRDX6, PRKAA1, PRKAB2, PRPF19, PRUNE1, PSMA3, PSMB10, PSMB2, PSMC2, PSMC3, PSMC6, PSMD1, PSMD12, PSMD14, PSMD2, PSMD6, PSMD7, PSMD8, PSME1, PSME2, PSME4, PYCARD, PYGB, QDPR, QSOX1, RAB1A, RAB23, RAB5A, RAB8A, RCN3, RDX, RHEB, RIPK2, RNF114, RNF2, RNF213, RNF40, RNPS1, RPL10A, RPL13, RPL13A, RPL18A, RPL22, RPL24, RPL27, RPL32, RPL36, RPL38, RPL5, RPLP1, RPLP2,	

					RPS13, RPS15A, RPS16, RPS18, RPS21, RPS23, RPS4Y1, RPS5, RPS8, RPS9, RSPRY1, RYBP, SAMHD1, SARM1, SBDS, SCARB2, SCFD1, SCLY, SCP2, SEC61B, SESN2, SET, SGSH, SH3BP4, SHMT2, SIRT1, SLC25A4, SLC25A5, SMARCC1, SMPD1, SNF8, SNX5, SOAT1, SQSTM1, SRC, SRD5A3, SSB, SUFU, TAB2, TBC1D17, TBK1, TIGAR, TOMM7, TPI1, TPP1, TRAPPC4, TRAPPC8, TRIM21, TRIM25, TRIP12, TSC2, TSPO, TUT4, TYMP, UBAC2, UBE2L6, UBR2, UBR4, UBXN2A, UFD1, USP11, USP22, USP28, USP4, USP48, USP5, USP7, USP9X, USP9Y, VDAC1, VDAC2, VLDLR, VPS25, VPS26B, VPS33B, VPS36, VPS41, WDR41, WFS1, WIPI2, WWP2, XYLB, YBX1, YOD1, ZC3HAV1, ZFP36L2, ZMPSTE24]	
antigen processing and presentation of peptide antigen	GO_BiologicalProcess-EBI-UniProt-GOA-ACAP-ARAP_13.05.2021_00h00	2.75E-67	17.73399	36	[B2M, CANX, CAPZA2, CLTC, DYNC1LI1, DYNLL1, ERAP1, HLA-A, HLA-B, HLA-C, IKBKB, IKBKG, ITGAV, ITGB5, KIF15, LNPEP, OSBPL1A, PSMA3, PSMB10, PSMB2, PSMC2, PSMC3, PSMC6, PSMD1, PSMD12, PSMD14, PSMD2, PSMD6, PSMD7, PSMD8, PSME1, PSME2, PSME4, PYCARD, SEC14L2, TAP1]	Downregulation
establishment of localization in cell	GO_BiologicalProcess-EBI-UniProt-GOA-ACAP-ARAP_13.05.2021_00h00	2.75E-67	12.439696	361	[ABCE1, ACACA, ACAP2, ACTB, ACTG1, ACTR2, ACTR3, ADAM10, AGPAT2, ALB, ALDH3A2, ALDOA, ANK3, ANKFY1, ANO6, ANXA3, ANXA5, AP3M2, AP5B1, APAF1, APEX1, APLP2, APP, APPL2, APRT, ARFGAP1, ARFIP1, ASAH1, ASPSCR1, ATG7, ATL3, ATP1A1, ATP6V0A1, ATP6V0C, AUP1, B2M, B3GAT3, B4GALT1, BANF1, BET1, BIN1, BOD1,	No change

					BST1, C2CD2L, CAMK2D, CANX, CAP1, CAPZA2, CASK, CCDC22, CCT2, CD109, CD81, CDK1, CDK16, CEMIP, CHAMP1, CHD7, CHID1, CHMP3, CHORDC1, CHP1, CHTOP, CLTC, CLTCL1, CLU, COG6, COG7, CORO1C, CORO7, CPSF2, CSTB, CTNNB1, CTSA, CTSC, CYB5R3, CYRIB, DAB2, DENND2A, DERL1, DIAPH1, DNM1L, DPYSL2, DSP, DST, DYNC1LI1, DYNLL1, EHD3, EIF2D, EIF6, EML3, EML4, ENY2, ERBB2, ERP44, EZR, FAM91A1, FERMT1, FGF2, FHOD1, FKBP1A, FMR1, FN1, FNBP1L, FTH1, FTL, GCC2, GDAP1, GEMIN4, GET3, GGA2, GIPC1, GLA, GLE1, GOLGA7, GPI, GSDMD, GSK3B, GSTM2, GSTP1, HACL1, HEATR3, HERC2, HEXB, HGS, HLA-B, HLA-C, HMOX1, HSP90AA1, HSP90B1, HSPA4, HSPA5, HSPB11, IFT140, IKBKG, IPO5, IPO9, IST1, ITGAV, ITGB1, ITIH4, ITM2C, ITSN1, JAGN1, KCNQ1, KPNB1, LAMP1, LGALS3BP, LIN7C, LLGL1, LRPPRC, M6PR, MAD1L1, MAD2L1, MAGED2, MAGI1, MALT1, MAP1B, MAP4K4, MFF, MGST1, MICALL1, MLH1, MME, MSN, MTCH1, MVP, MYH9, MYO1B, MYO1D, MYO1E, MYO6, NDUFA13, NEDD4, NHLRC2, NMD3, NMT1, NPLOC4, NPM1, NUF2, NUP205, NUP35, NUP62, NUTF2, OSBPL2, OXA1L, PAFAH1B1, PAFAH1B2, PARL, PCDH7, PDAP1, PDCD6IP, PDZD11, PHPT1, PIN1, PITRM1, PKM, PLCG1, PLD1, PML, PNP, PPIE, PPP3CA, PRDX6, PRKAA1, PRKAB2, PRKCI, PRKCSH, PSIP1, PSMC2, PSMC3,	

					PSMD1, PSMD12, PSMD14, PSMD2, PSMD6, PSMD7, PTGES2, PYCARD, PYGB, QSOX1, RAB10, RAB11B, RAB1A, RAB23, RAB3B, RAB3D, RAB5A, RAB8A, RABGEF1, RANBP1, RANGAP1, RAP2B, RAP2C, RBM22, RBM4, RDX, REEP2, REEP6, RELCH, RHOB, RNF2, RNPS1, RPL10A, RPL13, RPL13A, RPL18A, RPL22, RPL24, RPL27, RPL32, RPL36, RPL38, RPL5, RPLP1, RPLP2, RPS13, RPS15A, RPS16, RPS18, RPS21, RPS23, RPS4Y1, RPS5, RPS8, RPS9, SAE1, SARNP, SCARB2, SCFD1, SCP2, SCRIB, SEC14L2, SEC23B, SEC61A1, SEC61B, SELENOT, SEPTIN9, SERPINB1, SLC11A2, SLC1A1, SLC25A6, SLC44A2, SNF8, SNRPD3, SNRPE, SNX1, SNX18, SNX27, SNX5, SOD1, SORT1, SPARC, SPHK2, SQSTM1, SRC, SRPRA, SRSF10, SRSF2, SRSF5, SSB, SSR3, STOM, STON1, STXBP3, SUFU, SYNE2, SYNJ1, TAGLN2, TAP1, TBC1D10A, TBC1D17, TBC1D22B, TBC1D4, TBC1D8B, TCAF1, TF, THBS1, THOC2, TIMM17B, TM9SF4, TMED9, TMX3, TNPO2, TOM1, TOM1L2, TOMM34, TOMM7, TRAM1, TRAM2, TRAPPC4, TRAPPC8, TRIM28, TSC2, TSPO, TUBA4A, TUBB, TXN, UBAC2, UBR4, UFD1, UNC93B1, USP7, VAT1, VPS25, VPS26B, VPS33B, VPS36, VPS41, WDR1, WDR11, WDR35, XPO4, XPO6, XPOT, YKT6, YOD1, YTHDC1, ZDHHC21, ZFAND6, ZMPSTE24]	
localization within membrane	GO_BiologicalProcess-EBI-UniProt-GOA-ACAP-	2.75E-67	13.861386	112	[ACAP2, ACSL3, ACTB, ADAM10, AFDN, AGRN, AKR1B1, ALDH3A2, ANK3, APP, ATP1B3,	No change

	ARAP_13.05.2021_00h00				ATP2C1, CCDC22, CD81, CEMIP, CHORDC1, CHP1, CLTC, DAB2, EHD3, EMC1, EPB41L3, EPHA2, EPHA3, ERBB2, EXOC2, EZR, FERMT2, GBP1, GCC2, GDAP1, GET3, GGA2, GOLGA7, GPC1, GPC6, GPHN, HGS, HMOX1, HSP90AA1, HSPA4, HSPA5, IKBKB, ITGA3, ITGB1, KRT18, LAMA5, LIN7C, LLGL1, MACF1, MFF, MICALL1, MTCH1, MYADM, MYO1E, NDUFA13, NMT1, OXA1L, PACS1, PDZD11, PPP2R5A, PRKCI, RAB10, RAB11B, RAB3B, RAB3D, RAB8A, RABGEF1, RALA, RDX, REEP2, RPL10A, RPL13, RPL13A, RPL18A, RPL22, RPL24, RPL27, RPL32, RPL36, RPL38, RPL5, RPLP1, RPLP2, RPS13, RPS15A, RPS16, RPS18, RPS21, RPS23, RPS4Y1, RPS5, RPS8, RPS9, S100A10, SCRIB, SEC61A1, SEC61B, SLC1A1, SNF8, SNX27, SQSTM1, SRPRA, SSR3, STOM, TCAF1, TM9SF4, TNIK, TPBG, TRAM1, TRAM2, ZDHHC21]	
response to oxygen levels	GO_BiologicalProcess-EBI-UniProt-GOA-ACAP-ARAP_13.05.2021_00h00	2.75E-67	14.009662	58	[ABAT, ADSL, AK4, APAF1, ATG7, ATP6V1A, BNIP1, CASP3, CCN2, CXCL12, DNM1L, FASN, HDAC2, HK2, HMOX1, HSP90B1, KCNMA1, LDHA, NDRG1, NF1, NOP53, PDK1, PIN1, PKLR, PLOD2, PML, POLR2A, PRKAA1, PSMA3, PSMB10, PSMB2, PSMC2, PSMC3, PSMC6, PSMD1, PSMD12, PSMD14, PSMD2, PSMD6, PSMD7, PSMD8, PSME1, PSME2, PSME4, PTGIS, SCFD1, SIRT1, SLC11A2, SLC1A1, SLC2A1, SLC9A1, SMAD4, SOD2, SRC, TGFBR3, THBS1, TIGAR, TM9SF4]	Downregulation
cellular macromolecule localization	GO_BiologicalProcess-EBI-UniProt-GOA-	2.75E-67	14.181439	272	[ACSL3, ACTB, ADAM10, AFDN, AGRN, AKR1B1,	No change

	ACAP-ARAP_13.05.2021_00h00				ALDH3A2, ANK3, ANKRD13A, AP3M2, APEX1, APLP2, APP, APPL2, ARFIP1, ASPSCR1, ATP1B3, ATP2C1, AUP1, B3GAT3, BOD1, CCT2, CCT3, CCT6A, CD81, CDK1, CDK5RAP3, CEMIP, CHAMP1, CHORDC1, CHP1, CLTC, CLTCL1, CLU, CNGB1, COG7, CORO1B, CTNNB1, CTSA, DAB2, DDX1, DERL1, DHX36, DIAPH1, DNM1L, DVL2, EHD3, EIF2D, EMC1, EPB41L2, EPB41L3, EPHA2, EPHA3, ERBB2, EZR, FAM91A1, FERMT1, FERMT2, GBP1, GCC2, GDAP1, GET3, GGA2, GIPC1, GLMP, GLUL, GNL3L, GOLGA7, GPC1, GPC6, GPD1L, GPHN, GSK3B, H1-5, HACL1, HEATR3, HERC2, HGS, HK1, HK2, HMOX1, HSP90AA1, HSP90B1, HSPA4, HSPA5, IFT140, IKBKB, IPO5, IPO9, ITGA3, ITGB1, ITSN1, JAM3, KAT7, KPNB1, KRT18, LAMA5, LAMP1, LEMD2, LIN7C, LLGL1, LRWD1, M6PR, MACF1, MACROH2A1, MAGI1, MARCHF5, MEAK7, MFF, MICALL1, MMAB, MSH2, MSN, MYADM, MYO1E, MYO6, NDUFA13, NEDD1, NEDD4, NF2, NMD3, NMT1, NOP53, NPLOC4, NPM1, NUBP2, NUP35, NUP62, NUTF2, OBSL1, OSBPL8, OXA1L, PACS1, PARL, PARP1, PDZD11, PIGU, PIN1, PITRM1, PML, PPP2R5A, PPP3CA, PRKAA1, PRKCI, PRPF19, PTGES2, RAB10, RAB11B, RAB1A, RAB23, RAB3B, RAB3D, RAB5A, RAB8A, RABGEF1, RAD21, RALA, RANGAP1, RBM22, RDX, REEP2, RHOB, RNF2, RPA1, RPA2, RPF2, RPL10A, RPL13, RPL13A, RPL18A, RPL22, RPL24, RPL27, RPL32, RPL36,	

					RPL38, RPL5, RPLP1, RPLP2, RPS13, RPS15A, RPS16, RPS18, RPS21, RPS23, RPS4Y1, RPS5, RPS8, RPS9, RTN4, S100A10, SAE1, SCARB2, SCFD1, SCRIB, SEC23B, SEC61A1, SEC61B, SEPTIN10, SEPTIN11, SEPTIN2, SEPTIN7, SEPTIN8, SEPTIN9, SESN2, SH3BP4, SLC1A1, SLC25A6, SNF8, SNX1, SNX27, SNX5, SORT1, SQSTM1, SRC, SRPRA, SSB, SSR3, STOM, STXBP3, SUFU, TBC1D10A, TBC1D17, TBC1D22B, TBC1D4, TBC1D8B, TCAF1, TCP1, TIMM17B, TM9SF4, TMED9, TNIK, TNPO2, TOM1, TOM1L2, TOMM34, TOMM7, TOR1AIP1, TOR1AIP2, TOR1B, TPBG, TPP1, TRAM1, TRAM2, TRAPPC8, TRIM28, TSC2, TSPO, TWF2, TXN, UBAC2, UFD1, UNC93B1, USP4, VPS25, VPS26B, VPS33B, VPS36, VPS41, WASHC5, WDR11, WDR35, WIPI2, WRAP73, XPO4, XPO6, XPOT, YAP1, YBX1, YOD1, ZDHHC21, ZFAND6]	
export from cell	GO_BiologicalProcess-EBI-UniProt-GOA-ACAP-ARAP_13.05.2021_00h00	2.75E-67	12.416107	185	[ABAT, ABCC1, ACSL4, ACTR2, ADAM10, AGPAT2, ALB, ALDOA, ANO6, ANXA3, ANXA5, APAF1, APEX1, APLP2, APP, APRT, ARFGEF1, ARFIP1, ARHGAP17, ASAH1, ATG7, ATP1A1, ATP1B3, ATP2B1, ATP6V0A1, ATP6V0C, B2M, B4GALT1, BST1, C2CD2L, CANX, CAP1, CASK, CCN2, CCT2, CD109, CD200, CDK16, CHAMP1, CHD7, CHID1, CHMP3, CLU, CSTB, CTSA, CTSC, CXCL12, CYB5R3, CYP51A1, CYRIB, DIAPH1, DNM1L, DPYSL2, DSP, DYNC1LI1, DYNLL1, ENY2, ERP44, EXOC2, EZR, FMR1, FN1, FTH1, FTL, GIPC1, GLA, GLUD1,	No change

					GLUL, GOLGA7, GPI, GSDMD, GSK3B, GSTP1, HADH, HADHA, HEXB, HGS, HLA-B, HLA-C, HMOX1, HSP90AA1, IDH2, IST1, ITGAV, ITIH4, ITM2C, ITSN1, JAGN1, KCNQ1, KPNB1, LAMP1, LGALS3BP, LIN7C, LLGL1, LRRC8A, M6PR, MAGED2, MAP4K4, MGST1, MICAL3, MME, MTCH1, MVP, MYH10, MYH9, MYO18A, NHLRC2, NIBAN2, NISCH, P3H1, PAFAH1B1, PAFAH1B2, PCDH7, PCK2, PDAP1, PDCD6IP, PDZD11, PFKM, PHPT1, PIK3C2A, PKM, PLA2G4A, PLD1, PNP, PPIE, PPP3CA, PRDX6, PRKCSH, PSMC2, PSMC3, PSMD1, PSMD12, PSMD14, PSMD2, PSMD6, PSMD7, PTGES2, PYCARD, PYGB, QSOX1, RAB10, RAB11B, RAB1A, RAB3B, RAB3D, RAB5A, RAB8A, RALA, RAP2B, RAP2C, RCN3, RIPK2, SCRIB, SCRN1, SELENOT, SEPTIN2, SEPTIN9, SERPINB1, SERPINE2, SLC12A2, SLC1A5, SLC2A1, SLC44A2, SLC9A1, SMAD4, SNF8, SOD1, SPARC, SPHK2, STOM, STXBP3, SYNJ1, TAGLN2, TF, THBS1, TMX3, TOM1, TUBA4A, TUBB, UBE2Q1, UBR4, VAT1, VPS41, WDR1, YKT6]	
organic substance catabolic process	GO_BiologicalProcess-EBI-UniProt-GOA-ACAP-ARAP_13.05.2021_00h00	2.75E-67	13.851352	328	[AASS, ABAT, ABCC1, ABHD12, ABHD16A, ACAD8, ACADM, ACADSB, ACADVL, ACAT2, ACOT7, ACOT8, ACOX1, ADAMTS9, AGO2, AGRN, ALB, ALDH2, ALDH3A2, ALDOA, ANKZF1, APEX1, APP, ARAF, ARRB1, ASAH1, ATE1, ATG4B, ATG7, AUP1, BCAT1, BDH2, BLVRB, BPNT2, CASP3, CCDC22, CD81, CDC34, CDK1, CDK2, CDK5RAP3, CECR2, CEMIP, CHORDC1, CHP1,	No change

					CIRBP, CLTC, CLU, CNOT1, CNOT10, CRBN, CROT, CSPG4, CTNNB1, CTSA, CTSC, CUL1, CUL4B, CYP51A1, DAB2, DAGLB, DCN, DCPS, DCXR, DDI2, DERL1, DHX34, DHX36, DICER1, DLST, DPYSL2, DPYSL3, DPYSL4, DUT, ECE1, ECI2, EIF3E, EIF6, ENO2, ENPP1, ERAP1, ERLIN2, ETFDH, EZR, FAM3A, FBXL12, FBXW8, FEN1, FGF2, FMR1, FTO, FUT8, GALE, GALM, GAPDH, GBA2, GCAT, GCDH, GIPC1, GLA, GLMN, GLUD1, GLUL, GOLT1B, GOT1, GPC1, GPC6, GPD1L, GPD2, GPI, GSK3B, HACL1, HADH, HADHA, HDAC4, HEXB, HGS, HIBCH, HINT1, HK1, HK2, HMOX1, HNRNPA0, HNRNPC, HNRNPR, HSD17B10, HSD17B11, HSD17B4, HSP90AA1, HSP90B1, HSPA5, HSPG2, IGF2BP1, IGF2BP2, ILVBL, IMPA1, KPNB1, LARP1, LARP4B, LDHA, LIPA, LNPEP, LRPPRC, LUM, LYPLA2, MAD2L1, MAEA, MAGI1, MALT1, MAN2B2, MAOA, METTL14, MGAT1, MLH1, MMUT, MOV10, MPST, MRTO4, MSN, MTA1, MTAP, MTREX, MTRR, MYD88, NDUFA13, NEDD4, NEDD8, NOCT, NOP53, NPLOC4, NPM1, NT5C, NT5C3B, NUB1, NUDT15, NUDT16, NUDT16L1, NUDT19, NUP205, NUP35, NUP62, OAT, PACSIN3, PAFAH1B1, PAFAH1B2, PAFAH1B3, PAIP1, PCK2, PDCD6IP, PDE5A, PDXDC1, PELO, PFKM, PGM2L1, PHGDH, PHKB, PIN1, PITHD1, PKLR, PKM, PLA2G15, PLA2G4A, PLBD2, PLCB3, PLCG1, PLD1, PLD3, PML, PNP, PNPLA4, PNPLA6, POP1,	

					PPP1CA, PRDX6, PRKAA1, PRKCSH, PRPF19, PSMA3, PSMB10, PSMB2, PSMC2, PSMC3, PSMC6, PSMD1, PSMD12, PSMD14, PSMD2, PSMD6, PSMD7, PSMD8, PSME1, PSME2, PSME4, PYGB, QDPR, RCN3, RDX, RNF114, RNF2, RNF213, RNF40, RNPS1, RPL10A, RPL13, RPL13A, RPL18A, RPL22, RPL24, RPL27, RPL32, RPL36, RPL38, RPL5, RPLP1, RPLP2, RPS13, RPS15A, RPS16, RPS18, RPS21, RPS23, RPS4Y1, RPS5, RPS8, RPS9, RSPRY1, RYBP, SAMHD1, SARM1, SBDS, SCARB2, SCLY, SCP2, SEC61B, SERPINE2, SESN2, SET, SGSH, SHMT2, SIRT1, SLC25A12, SLC44A1, SLC9A1, SMARCC1, SMPD1, SNF8, SNX1, SNX5, SOAT1, SQSTM1, SRD5A3, SSB, SUFU, SULT1E1, SYNJ1, TIGAR, TPI1, TPP1, TRIM25, TRIP12, TUT4, TYMP, UBAC2, UBE2L6, UBR2, UBR4, UBXN2A, UFD1, USP11, USP22, USP28, USP4, USP48, USP5, USP7, USP9X, USP9Y, VLDLR, VPS25, VPS36, WFS1, WWP2, XYLB, YBX1, YOD1, ZC3HAV1, ZFP36L2, ZMPSTE24]	
peptidase complex	GO_CellularComponent-EBI-UniProt-GOA-ACAP-ARAP_13.05.2021_00h00	2.75E-67	23	23	[ENY2, GLMN, PIGT, PIGU, PSMA3, PSMB10, PSMB2, PSMC2, PSMC3, PSMC6, PSMD1, PSMD12, PSMD14, PSMD2, PSMD6, PSMD7, PSMD8, PSME1, PSME2, PSME4, SEC11C, TAF6L, USP22]	Downregulation
myeloid leukocyte activation	GO_BiologicalProcess-EBI-UniProt-GOA-ACAP-ARAP_13.05.2021_00h00	2.75E-67	12.396694	90	[ACTR2, ADAM10, AGPAT2, ALDOA, ANO6, ANXA3, APAF1, APP, APRT, ASAH1, ATG7, ATP6V0A1, ATP6V0C, B2M, B4GALT1, BST1, CAP1, CCT2, CD200, CHAMP1, CLU, CSTB, CTSA, CTSC, CYB5R3, DIAPH1, DSP,	Downregulation

					DYNC1LI1, DYNLL1, ERP44, FTH1, FTL, GLA, GOLGA7, GPI, GSDMD, GSTP1, HEXB, HLA-B, HLA-C, HMOX1, HSP90AA1, IST1, ITGAV, ITM2C, KPNB1, LAMP1, MGST1, MME, MTCH1, MVP, MYD88, MYO18A, NDRG1, NECTIN2, PAFAH1B2, PDAP1, PKM, PLA2G4A, PLD1, PNP, PPIE, PRDX6, PRKCSH, PSMC2, PSMC3, PSMD1, PSMD12, PSMD14, PSMD2, PSMD6, PSMD7, PTGES2, PYCARD, PYGB, QSOX1, RAB10, RAB3D, RAP2B, RAP2C, SERPINB1, SLC44A2, SPHK2, STOM, STXBP3, THBS1, TOM1, TUBB, UBR4, VAT1]	
regulation of response to biotic stimulus	GO_BiologicalProcess-EBI-UniProt-GOA-ACAP-ARAP_13.05.2021_00h00	2.75E-67	13.288288	59	[ABCE1, APLP2, APPL2, C1QBP, CUL1, ERAP1, FAM3A, HLA-A, HLA-B, HPX, HSP90AA1, IGF2BP1, IKBKB, IKBKG, LAMP1, MALT1, MATR3, NECTIN2, NOP53, NPLOC4, PARP14, PKN2, POLR3C, PPM1B, PSMA3, PSMB10, PSMB2, PSMC2, PSMC3, PSMC6, PSMD1, PSMD12, PSMD14, PSMD2, PSMD6, PSMD7, PSMD8, PSME1, PSME2, PSME4, PSPC1, PYCARD, RIOK3, RIPK2, SAMHD1, SERPINB9, SRC, STAT1, STAT2, STAT5B, TAB2, TBK1, TIGAR, TRIM21, TTLL12, UFD1, XIAP, ZCCHC3, ZMPSTE24]	Downregulation
cell migration	GO_BiologicalProcess-EBI-UniProt-GOA-ACAP-ARAP_13.05.2021_00h00	2.75E-67	10.549044	171	[ABCC1, ACTG1, ADAM10, ADAMTS9, ADARB1, AFDN, AGO2, AKAP12, AMOTL1, ANO6, ANXA3, APEX1, APLP2, APP, APPL2, ARPC5L, ARPIN, ASPM, ATP1B3, B4GALT1, BST1, C1QBP, CAP1, CCN2, CD200, CD81, CDC42BPA, CDK1, CEMIP, CLU, COL5A1, CORO1B, CORO1C, CSPG4, CTNNB1, CXCL12, CYRIB, DAB2, DHX34,	No change

					DIAPH1, DNM1L, DPYSL3, DUSP3, DYNLL1, EFNB2, ELP3, ELP5, EPHA2, EPHA3, EPHB4, EPPK1, FAT1, FERMT1, FERMT2, FGF2, FLRT2, FN1, FSCN1, FUT8, GAB1, GIPC1, GLMN, GLUL, GPC1, GPC6, GPI, GSTP1, HDAC4, HEXB, HMOX1, HSPA5, IDH2, ITGA3, ITGAV, ITGB1, ITGB5, JAG1, JAGN1, JAM3, LAMA5, LAMC1, MACF1, MAP1B, MAP2K3, MAP4K4, MBOAT7, MCAM, MEAK7, MLF2, MSN, MYADM, MYD88, MYH9, MYO18A, MYO1E, NCK2, NCKAP1, NF1, NF2, NISCH, OSBPL8, OXSR1, PAFAH1B1, PALLD, PATZ1, PDXDC1, PIGU, PIK3C2A, PIK3CA, PKN2, PLCG1, PLPP3, PLXNB2, PML, POMGNT2, POMK, PPIB, PPP3CA, PRKCI, PRKG1, PTGES2, PTK7, PTPRF, PTPRG, PXN, PYCARD, RAB1A, RAP2B, RAP2C, RDX, RHOB, RHOC, RIC8A, RIPK2, RRAS2, RTN4, SBDS, SCAI, SCRIB, SEMA3C, SERPINE2, SH3KBP1, SIRT1, SLC12A2, SLC16A3, SLC7A6, SLC9A1, SLIT2, SOD2, SPARC, SRC, STAT1, STAT5B, STK24, SYNE2, TCAF1, TGFBR3, THBS1, TJP1, TPBG, TRADD, TRIP6, TSPO, TUBGCP2, UBA2, USP9X, USP9Y, VTN, WDR1, WWC2, YES1]	
regulation of protein localization	GO_BiologicalProcess-EBI-UniProt-GOA-ACAP-ARAP_13.05.2021_00h00	2.75E-67	12.756757	118	[ABAT, ACSL3, ACSL4, ACTB, ADAM10, AFDN, AKR1B1, ANK3, ANKRD13A, ARFIP1, ATP2C1, B3GAT3, C2CD2L, CCT2, CCT3, CCT6A, CD200, CD81, CDK1, CDK16, CDK5RAP3, CEMIP, CHORDC1, CHP1, CLTC, CLU, CTNNB1, CYP51A1, DAB2, DNM1L, DYNLL1, ENY2, EPB41L2, EPHA2, EPHA3, ERBB2, EZR, FERMT1, FERMT2, FKBP1A, GBP1, GCC2, GLUD1, GLUL,	No change

					GNL3L, GPC1, GPC6, GPD1L, GSK3B, HADH, HADHA, HSP90AA1, IDH2, IPO5, ITGA3, ITGB1, JAGN1, KAT7, LLGL1, LRRC8A, MACROH2A1, MAGI1, MAP4K4, MEAK7, MFF, MSN, MYH10, MYO18A, MYO1E, NF2, NMD3, NMT1, NPM1, NUP62, NUTF2, P3H1, PARL, PARP1, PCK2, PFKM, PHPT1, PIN1, PPP2R5A, PPP3CA, PRKAA1, PRKCI, PTGES2, RAB11B, RAB23, RAB8A, RANGAP1, RBM22, RDX, RNF2, RTN4, SAE1, SCFD1, SESN2, SLC12A2, SLC1A1, SLC2A1, SQSTM1, SRC, STOM, SUFU, TCAF1, TCP1, TM9SF4, TOMM7, TRIM21, TRIM25, TRIM28, TXN, UBAC2, WWP2, XPO4, YAP1, YOD1]	
establishment of protein localization	GO_BiologicalProcess-EBI-UniProt-GOA-ACAP-ARAP_13.05.2021_00h00	2.75E-67	13.579604	261	[ABAT, ACSL3, ACSL4, ADAMTS9, AFDN, AHCTF1, ALDH3A2, ANK3, AP3M2, AP5B1, APEX1, APLP2, APP, APPL2, ARFGAP1, ARFGEF1, ARFIP1, ARRB1, ASPSCR1, ATG4B, ATG7, ATP2C1, ATP6V0A1, ATP6V0C, ATP6V1A, ATP6V1B2, AUP1, B3GAT3, BET1, C2CD2L, CANX, CCDC22, CCT2, CCT3, CCT6A, CD200, CD81, CDK1, CDK16, CEMIP, CETN2, CHMP3, CHORDC1, CHP1, CLTC, CLTCL1, CLU, COG6, COG7, CORO1C, CORO7, CTSA, CYP51A1, DAB2, DENND2A, DERL1, DNM1L, DYNLL1, EHD3, EIF2D, EMC1, ENY2, ERBB2, EXOC2, EZR, FAM91A1, FERMT1, GCC2, GDAP1, GET3, GGA2, GIPC1, GLE1, GLUD1, GLUL, GOLGA7, GOLT1B, H1-5, HACL1, HADH, HADHA, HEATR3, HERC2, HGS, HIP1, HK1, HK2, HMOX1, HSP90AA1, HSP90B1, HSPA4, HSPA5, HSPB11, IDH2, IPO5, IPO9, IST1, ITSN1, JAGN1,	No change

					KPNB1, KRT18, LAMP1, LIMS1, LIN7C, LLGL1, LRRC8A, LRWD1, M6PR, MACF1, MACROH2A1, MAGI1, MAP4K4, MFF, MICALL1, MVP, MYH10, MYH9, MYO18A, MYO1D, MYO1E, MYO6, NDUFA13, NEDD4, NMD3, NMT1, NPLOC4, NPM1, NUDT19, NUP205, NUP35, NUP62, NUTF2, OXA1L, P3H1, PAFAH1B1, PARL, PCK2, PDCD6IP, PFKM, PHPT1, PIN1, PITRM1, PML, PPP3CA, PRKAA1, PRKCI, PTGES2, RAB10, RAB11B, RAB1A, RAB23, RAB3B, RAB3D, RAB5A, RAB8A, RABGEF1, RALA, RANGAP1, RBM22, RCN3, RDX, REEP2, RHOB, RNF2, RPL10A, RPL13, RPL13A, RPL18A, RPL22, RPL24, RPL27, RPL32, RPL36, RPL38, RPL5, RPLP1, RPLP2, RPS13, RPS15A, RPS16, RPS18, RPS21, RPS23, RPS4Y1, RPS5, RPS8, RPS9, SAE1, SCARB2, SCFD1, SCRIB, SEC14L2, SEC23B, SEC61A1, SEC61B, SELENBP1, SELENOT, SEPTIN9, SLC12A2, SLC1A1, SLC25A6, SLC2A1, SMYD3, SNF8, SNX1, SNX18, SNX21, SNX27, SNX30, SNX5, SORT1, SRC, SRPRA, SSR3, STOM, STXBP3, SUFU, TAP1, TBC1D10A, TBC1D17, TBC1D22B, TBC1D4, TBC1D8B, TCAF1, TCP1, TF, TIMM17B, TM9SF4, TMED9, TNPO2, TOM1, TOM1L2, TOMM34, TOMM7, TRAM1, TRAM2, TRIM28, TSC2, TSPO, TXN, UBAC2, UBE2Q1, UFD1, UNC119B, UNC93B1, VLDLR, VPS25, VPS26B, VPS33B, VPS36, VPS41, WASHC5, WDR11, WWP2, XPO4, XPO6, XPOT,	

					YKT6, YOD1, ZDHHC21, ZFAND6]	
intracellular transport	GO_BiologicalProcess-EBI-UniProt-GOA-ACAP-ARAP_13.05.2021_00h00	2.75E-67	13.176996	236	[ABCE1, ACACA, ACAP2, ACTR2, ALDH3A2, ANK3, ANKFY1, AP3M2, AP5B1, APEX1, APLP2, APP, APPL2, ARFGAP1, ARFIP1, ASPSCR1, ATL3, AUP1, B3GAT3, BANF1, BET1, BIN1, CAPZA2, CCDC22, CD81, CDK1, CEMIP, CHMP3, CHORDC1, CHP1, CHTOP, CLTC, CLTCL1, CLU, COG6, COG7, CORO1C, CORO7, CPSF2, CTSA, CTSC, DAB2, DENND2A, DERL1, DNM1L, DST, DYNC1LI1, DYNLL1, EHD3, EIF2D, EIF6, ENY2, ERBB2, EZR, FAM91A1, FERMT1, FHOD1, FNBP1L, GCC2, GDAP1, GEMIN4, GET3, GGA2, GIPC1, GLE1, GOLGA7, HACL1, HEATR3, HERC2, HGS, HSP90AA1, HSP90B1, HSPA4, HSPA5, HSPB11, IFT140, IPO5, IPO9, ITSN1, KPNB1, LAMP1, LRPPRC, M6PR, MAGI1, MALT1, MAP1B, MFF, MICALL1, MSN, MYO1B, MYO1D, MYO1E, MYO6, NDUFA13, NEDD4, NMD3, NMT1, NPLOC4, NPM1, NUP205, NUP35, NUP62, NUTF2, OSBPL2, OXA1L, PAFAH1B1, PARL, PIN1, PITRM1, PML, PPP3CA, PRKAA1, PRKAB2, PRKCI, PSIP1, PTGES2, RAB10, RAB11B, RAB1A, RAB23, RAB5A, RAB8A, RABGEF1, RANBP1, RANGAP1, RBM22, RBM4, RDX, REEP2, REEP6, RELCH, RHOB, RNF2, RNPS1, RPL10A, RPL13, RPL13A, RPL18A, RPL22, RPL24, RPL27, RPL32, RPL36, RPL38, RPL5, RPLP1, RPLP2, RPS13, RPS15A, RPS16, RPS18, RPS21, RPS23, RPS4Y1, RPS5, RPS8, RPS9, SAE1, SARNP, SCARB2, SCFD1, SCP2, SCRIB,	No change

					SEC14L2, SEC23B, SEC61A1, SEC61B, SEPTIN9, SLC1A1, SLC25A6, SNF8, SNRPD3, SNRPE, SNX1, SNX18, SNX27, SNX5, SOD1, SORT1, SQSTM1, SRC, SRPRA, SRSF10, SRSF2, SRSF5, SSB, SSR3, STOM, STXBP3, SUFU, SYNE2, TAP1, TBC1D10A, TBC1D17, TBC1D22B, TBC1D4, TBC1D8B, TCAF1, THOC2, TIMM17B, TM9SF4, TMED9, TNPO2, TOM1, TOM1L2, TOMM34, TOMM7, TRAM1, TRAM2, TRAPPC4, TRAPPC8, TRIM28, TSC2, TSPO, TUBB, TXN, UBAC2, UFD1, UNC93B1, USP7, VPS25, VPS26B, VPS33B, VPS36, VPS41, WDR11, WDR35, XPO4, XPO6, XPOT, YKT6, YOD1, YTHDC1, ZDHHC21, ZFAND6, ZMPSTE24]	
regulation of transport	GO_BiologicalProcess-EBI-UniProt-GOA-ACAP-ARAP_13.05.2021_00h00	2.75E-67	10.14571	188	[ABAT, ACSL3, ACSL4, ACTB, ACTG1, ANK3, ANKFY1, ANKRD13A, ANO6, APEX1, APLP2, APP, APPL2, ARFGAP1, ARFIP1, ARPP19, ARRB1, ASPSCR1, ATP1A1, ATP1B3, ATP2B1, ATP2C1, B2M, B3GAT3, BIN1, C2CD2L, CAMK2D, CASK, CD200, CD81, CDK1, CDK16, CEMIP, CHD7, CHMP3, CHORDC1, CHP1, CLIC1, CLTCL1, CLU, CRBN, CTNNB1, CXCL12, CYP51A1, DAB2, DAPK1, DIAPH1, DNM1L, DPYSL2, DYNLL1, EHD3, ENPP1, ENY2, EPHA3, EPRS1, ERBB2, EZR, FERMT1, FHL1, FKBP1A, FMR1, G6PD, GCC2, GIPC1, GLUD1, GLUL, GPD1L, GSK3B, GSTM2, HADH, HADHA, HGS, HIP1, HK2, HMOX1, HNRNPC, IDH2, IPO5, ITGAV, ITGB1, ITSN1, JAGN1, KCNMA1, KCNQ1, LAMP1, LLGL1, LRRC8A, MAGI1,	No change

					MAP1B, MAP4K4, MFF, MSN, MYH10, MYO18A, MYO1E, MYO6, NDUFA4, NEDD4, NMT1, NUP62, NUTF2, OSBPL8, OXSR1, P3H1, PACSIN3, PARL, PCK2, PDCD6IP, PFKM, PHPT1, PIN1, PLA2G4A, PLCG1, PLD1, PML, PPP3CA, PRKAA1, PRKCI, PRKCSH, PTRH2, PXK, PYCARD, RAB10, RAB11B, RAB23, RAB3B, RAB3D, RAB5A, RAB8A, RALA, RANGAP1, RBM22, RBM4, RDX, REEP2, REEP6, RIPK2, RNF2, SAE1, SCFD1, SCP2, SCRIB, SELENOT, SEPTIN2, SERPINE2, SESTD1, SIRT1, SLC12A2, SLC1A1, SLC25A5, SLC2A1, SLC9A1, SMAD4, SMPD1, SNF8, SNX5, SOD1, SPHK2, SRC, STOM, STON1, STXBP3, SUFU, SYNJ1, TBC1D4, TCAF1, TF, TGM2, THADA, THBS1, TM9SF4, TOMM7, TRIM28, TSC2, TSPO, TTC39B, TXN, UBAC2, USP7, VDAC1, VTN, WDR1, WFS1, WWP2, XPO4, YES1, YOD1, ZMPSTE24]	
regulation of cell cycle	GO_BiologicalProcess-EBI-UniProt-GOA-ACAP-ARAP_13.05.2021_00h00	2.75E-67	11.846405	145	[ACTB, ADAM10, ADARB1, AHCTF1, APAF1, APBB2, APEX1, APLP2, APP, APPL2, ASNS, ASPM, BIN1, CASP3, CBX3, CBX5, CCN2, CCNH, CCNK, CCNL1, CDC5L, CDC73, CDK1, CDK16, CDK17, CDK2, CDK5RAP3, CENPV, CETN2, CHMP3, CHORDC1, CLIC1, CLTC, CNOT1, CNOT10, CTBP1, CTNNB1, CUL1, DHCR24, DRG1, DUSP3, DYNC1LI1, DYNLL1, EED, EML3, ETFDH, FBXL12, FEN1, FGF2, FHL1, FSD1, GIPC1, GLMN, HSP90AA1, IPO5, ITGB1, KNTC1, MACROH2A1, MAD1L1, MAD2L1, MAEA, MAGI1, MAP3K20, MCM2, MDC1, MGA,	Upregulation

					MMAB, MSH2, NDRG1, NEDD1, NF2, NLE1, NOP53, NPM1, NPR2, NUBP2, NUDT16, NUP62, OBSL1, PAFAH1B1, PDCD6IP, PIN1, PKN2, PML, PRPF19, PSMA3, PSMB10, PSMB2, PSMC2, PSMC3, PSMC6, PSMD1, PSMD12, PSMD14, PSMD2, PSMD6, PSMD7, PSMD8, PSME1, PSME2, PSME4, PTGES2, RAD21, RAD9A, RANBP1, RBBP4, RCC1, RDX, RHOB, RNF2, RNF40, RPA2, RPRD1B, RPS15A, RYBP, SCRIB, SIRT1, SLC25A15, SMARCD3, SON, SRA1, SRC, SRSF5, STAT5B, SVIL, THBS1, TIPRL, TOM1L2, TPX2, TRIM21, TRIP13, TSC2, TTC28, TTLL12, TUBA4A, TUBB, UBA3, UHRF2, USP22, USP28, WDR12, XIAP, ZFP36L2, ZMPSTE24, ZNF207]	
regulation of cellular localization	GO_BiologicalProcess-EBI-UniProt-GOA-ACAP-ARAP_13.05.2021_00h00	2.75E-67	12.826334	113	[ACSL3, ACTB, ADAM10, AKR1B1, ANK3, ANKRD13A, APEX1, ARFIP1, ATP2C1, B3GAT3, C2CD2L, CAMK2D, CCT2, CCT3, CCT6A, CD81, CDK1, CDK16, CDK5RAP3, CEMIP, CHD7, CHMP3, CHORDC1, CHP1, CLTC, CTNNB1, DAB2, DHX36, DIAPH1, DYNLL1, ENY2, EPB41L2, EPHA2, EPHA3, ERBB2, EZR, FERMT1, FERMT2, FKBP1A, GBP1, GCC2, GLUL, GNL3L, GPC1, GPC6, GPD1L, GSK3B, GSTM2, HMOX1, HSP90AA1, IPO5, ITGA3, ITGB1, JAGN1, KAT7, LAMP1, MACROH2A1, MAD1L1, MAGI1, MAP1B, MAP4K4, MEAK7, MFF, MSN, MYO1E, NEDD4, NF2, NMD3, NMT1, NPM1, NUP62, NUTF2, PARL, PARP1, PHPT1, PIN1, PLCG1, PML, PPP2R5A, PRKAA1,	No change

					PRKCI, PTGES2, RAB11B, RAB23, RANGAP1, RBM22, RBM4, RDX, REEP2, REEP6, RNF2, RTN4, SAE1, SCFD1, SCP2, SESN2, SLC1A1, SPHK2, SQSTM1, SRC, STOM, SUFU, TCAF1, TCP1, TM9SF4, TOMM7, TRIM28, TXN, UBAC2, USP7, XPO4, YAP1, YOD1]	
regulation of small molecule metabolic process	GO_BiologicalProcess-EBI-UniProt-GOA-ACAP-ARAP_13.05.2021_00h00	2.75E-67	13.199105	59	[ACADM, ACADVL, AK4, APLP2, APP, APPL2, ARPP19, DHCR7, DHX34, EIF6, ENPP1, EPM2AIP1, ERLIN2, FAM3A, GSK3B, HDAC4, KPNB1, MAEA, NQO1, NUP205, NUP35, NUP62, PARP1, PDK1, PGP, PPP1CA, PRKAA1, PRKAB2, PSMA3, PSMB10, PSMB2, PSMC2, PSMC3, PSMC6, PSMD1, PSMD12, PSMD14, PSMD2, PSMD6, PSMD7, PSMD8, PSME1, PSME2, PSME4, PTRH2, RDH10, SEC14L2, SESN2, SIRT1, SLC25A12, SOD1, SPHK2, SRC, TIGAR, TTC39B, USP7, VDAC2, WDR5, ZMPSTE24]	No change
mitotic cell cycle process	GO_BiologicalProcess-EBI-UniProt-GOA-ACAP-ARAP_13.05.2021_00h00	2.75E-67	13.089005	125	[AHCTF1, ANK3, APEX1, APLP2, APP, APPL2, ARPP19, BANF1, BCAT1, BOD1, CCNH, CDC34, CDC73, CDK1, CDK16, CDK17, CDK2, CDK5RAP3, CETN2, CHAMP1, CHMP3, CLTC, CNOT1, CNOT10, CUL1, CUL4B, DHFR, DRG1, DYNC1LI1, DYNLL1, EML1, EML3, EML4, ERCC6L, FBXL12, FHL1, FSD1, HSP90AA1, IQGAP3, IST1, ITGB1, KNTC1, KPNB1, LBR, LEMD2, LIG1, MACROH2A1, MAD1L1, MAD2L1, MAP3K20, MCM2, MCM3, MCM4, MCM6, MCM7, MDC1, MSH2, NCAPD2, NCAPG, NCAPH, NDRG1, NEDD1, NES, NOP53, NUF2, NUP205, NUP35, NUP62, OBSL1, PAFAH1B1, PDCD6IP, PDS5B,	Upregulation

					PIN1, PML, POGZ, PPP3CA, PSMA3, PSMB10, PSMB2, PSMC2, PSMC3, PSMC6, PSMD1, PSMD12, PSMD14, PSMD2, PSMD6, PSMD7, PSMD8, PSME1, PSME2, PSME4, PTPA, RAD21, RAD9A, RANBP1, RANGAP1, RCC1, RDX, RHOB, RHOC, RPA2, RPL24, SBDS, SET, SLC25A15, SMARCD3, SMC1A, SMC2, SMC3, SMC4, SNX18, SON, STAG2, TBCE, TOM1L2, TPX2, TRIP13, TUBA4A, TUBB, VRK1, WRAP73, ZFP36L2, ZMPSTE24, ZNF207]	
proteasome accessory complex	GO_CellularComponent-EBI-UniProt-GOA-ACAP-ARAP_13.05.2021_00h00	2.75E-67	46.153847	12	[PSMC2, PSMC3, PSMC6, PSMD1, PSMD12, PSMD14, PSMD2, PSMD6, PSMD7, PSMD8, PSME1, PSME2]	Downregulation
proteasome complex	GO_CellularComponent-EBI-UniProt-GOA-ACAP-ARAP_13.05.2021_00h00	2.75E-67	25	16	[PSMA3, PSMB10, PSMB2, PSMC2, PSMC3, PSMC6, PSMD1, PSMD12, PSMD14, PSMD2, PSMD6, PSMD7, PSMD8, PSME1, PSME2, PSME4]	Downregulation
myeloid leukocyte mediated immunity	GO_BiologicalProcess-EBI-UniProt-GOA-ACAP-ARAP_13.05.2021_00h00	2.75E-67	14.14791	88	[ACTR2, ADAM10, AGPAT2, ALDOA, ANO6, ANXA3, APAF1, APRT, ASAH1, ATG7, ATP6V0A1, ATP6V0C, B2M, B4GALT1, BST1, CAP1, CCT2, CHAMP1, CLU, CSTB, CTSA, CTSC, CYB5R3, DDX1, DHX36, DIAPH1, DSP, DYNC1LI1, DYNLL1, ERP44, FTH1, FTL, GLA, GOLGA7, GPI, GSDMD, GSTP1, HEXB, HLA-B, HLA-C, HMOX1, HSP90AA1, IST1, ITGAV, ITM2C, JAGN1, KPNB1, LAMP1, MGST1, MME, MTCH1, MVP, MYD88, PAFAH1B2, PDAP1, PKM, PLD1, PNP, PPIE, PRDX6, PRKCSH, PSMC2, PSMC3, PSMD1, PSMD12, PSMD14, PSMD2, PSMD6, PSMD7, PTGES2, PYCARD, PYGB, QSOX1, RAB10, RAB3D, RAP2B, RAP2C, SERPINB1, SERPINB9, SLC44A2,	Downregulation

					SPHK2, STOM, STXBP3, TOM1, TUBB, UBR4, VAT1, WDR1]	
antigen processing and presentation of peptide antigen via MHC class I	GO_BiologicalProcess-EBI-UniProt-GOA-ACAP-ARAP_13.05.2021_00h00	2.75E-67	28.15534	29	[B2M, CANX, ERAP1, HLA-A, HLA-B, HLA-C, IKBKB, IKBKG, ITGAV, ITGB5, LNPEP, PSMA3, PSMB10, PSMB2, PSMC2, PSMC3, PSMC6, PSMD1, PSMD12, PSMD14, PSMD2, PSMD6, PSMD7, PSMD8, PSME1, PSME2, PSME4, SEC14L2, TAP1]	Downregulation
antigen processing and presentation of exogenous peptide antigen	GO_BiologicalProcess-EBI-UniProt-GOA-ACAP-ARAP_13.05.2021_00h00	2.75E-67	18.27957	34	[B2M, CANX, CAPZA2, CLTC, DYNC1LI1, DYNLL1, HLA-A, HLA-B, HLA-C, IKBKB, IKBKG, ITGAV, ITGB5, KIF15, LNPEP, OSBPL1A, PSMA3, PSMB10, PSMB2, PSMC2, PSMC3, PSMC6, PSMD1, PSMD12, PSMD14, PSMD2, PSMD6, PSMD7, PSMD8, PSME1, PSME2, PSME4, SEC14L2, TAP1]	Downregulation
RNA binding	GO_MolecularFunction-EBI-UniProt-GOA-ACAP-ARAP_13.05.2021_00h00	2.75E-67	15.472779	270	[AARS1, ABT1, ACO1, ADARB1, ADK, AGO2, ALDH18A1, ALDOA, ANXA7, APEX1, AQR, BMS1, BZW1, C1QBP, CANX, CBX8, CCT3, CCT6A, CDC5L, CELF2, CFAP20, CHD2, CHTOP, CIRBP, CLTC, CNGB1, CNOT1, COIL, CPSF2, CRYZ, CSTB, CSTF3, CTU1, CWC22, DARS1, DCN, DCPS, DDX1, DDX42, DDX47, DENR, DHFR, DHX34, DHX36, DIAPH1, DICER1, DIMT1, DNMT1, DNTTIP2, DSP, DUT, DYNC1LI1, EARS2, EDF1, EEF1G, EFL1, EIF2AK2, EIF2B1, EIF2D, EIF2S1, EIF3A, EIF3B, EIF3E, EIF3F, EIF4G3, EIF6, ELP3, ELP5, EMG1, EPPK1, EPRS1, EZR, FAM120A, FASN, FMR1, FSCN1, GARS1, GLRX3, GNL3L, GPKOW, GRWD1, GTF2F1, H1-10, H1-5, HDAC2, HEATR6, HELZ, HLA-A, HNRNPA0, HNRNPA3, HNRNPC, HNRNPH1, HNRNPLL, HNRNPR, HNRNPUL1,	No change

					HSD17B10, HSP90AA1, HSP90B1, HSPE1, HTATSF1, IARS2, IFIT5, IGF2BP1, IGF2BP2, ILF3, IPO5, IREB2, KCTD12, KDM1A, KPNB1, KRT18, L1TD1, LARP1, LARP4B, LARP6, LBR, LRPPRC, LRRFIP1, MACF1, MAP3K20, MATR3, METTL14, MOV10, MRM3, MRPL10, MRPS17, MRPS21, MRPS31, MRTO4, MSI2, MTREX, MYH10, MYH9, MYO18A, NCL, NCOA5, NEDD4, NELFCD, NMD3, NOCT, NOL11, NOM1, NOP53, NPM1, NQO1, NSUN5, NUDT16, NUDT16L1, PAIP1, PARP1, PCBP3, PDAP1, PEBP1, PKM, PKN2, PLEC, POLR2A, POLR2B, POP1, PPIB, PPIE, PPIG, PPP5C, PRDX1, PSIP1, PSMD2, PSPC1, PTBP2, PTBP3, PUF60, PUS7, RANGAP1, RARS2, RBM22, RBM26, RBM4, RBMS2, RDX, RNF40, RNPS1, RPF2, RPL10A, RPL13, RPL13A, RPL18A, RPL22, RPL24, RPL27, RPL32, RPL36, RPL38, RPL5, RPS13, RPS15A, RPS16, RPS18, RPS21, RPS23, RPS4Y1, RPS5, RPS8, RPS9, RTN4, S100A16, SAMHD1, SARNP, SARS1, SBDS, SCAF11, SEC61B, SF1, SF3A3, SF3B6, SHFL, SHMT2, SLC16A3, SLC25A5, SMARCE1, SMC1A, SMNDC1, SNRNP200, SNRPA, SNRPD3, SNRPE, SNU13, SNW1, SON, SPATS2, SPATS2L, SRA1, SRPRA, SRSF10, SRSF2, SRSF5, SSB, SSRP1, SUPT16H, SYNJ1, TBCA, TCP1, TDRD3, TFAM, THOC2, TRA2A, TRA2B, TRIM21, TRIM25, TRIM28, TRIM56, TRIP6, TRMT10C, TRMT2A, TUT4, TWF2, TXN, U2SURP, UBFD1, UBTF,	

					UTP14A, UTP20, UTP3, XPOT, YARS1, YBX1, YRDC, YTHDC1, ZC3HAV1, ZCCHC3, ZFP36L2, ZNF207]	
macromolecule catabolic process	GO_BiologicalProcess-EBI-UniProt-GOA-ACAP-ARAP_13.05.2021_00h00	2.75E-67	13.624161	203	[ABHD12, ADAMTS9, AGO2, AGRN, ANKZF1, APEX1, ARAF, ARRB1, ATE1, ATG4B, ATG7, AUP1, CASP3, CCDC22, CD81, CDC34, CDK1, CDK2, CDK5RAP3, CECR2, CEMIP, CHORDC1, CHP1, CIRBP, CLTC, CLU, CNOT1, CNOT10, CRBN, CSPG4, CTNNB1, CTSA, CTSC, CUL1, CUL4B, CYP51A1, DAB2, DCN, DCPS, DDI2, DERL1, DHX34, DHX36, DICER1, EIF3E, ERLIN2, EZR, FBXL12, FBXW8, FEN1, FGF2, FMR1, FTO, GIPC1, GLMN, GPC1, GPC6, GSK3B, HEXB, HGS, HNRNPA0, HNRNPC, HNRNPR, HSP90AA1, HSP90B1, HSPA5, HSPG2, IGF2BP1, IGF2BP2, KPNB1, LARP1, LARP4B, LRPPRC, LUM, LYPLA2, MAD2L1, MAEA, MAGI1, MALT1, METTL14, MLH1, MOV10, MRTO4, MSN, MTA1, MTREX, MYD88, NDUFA13, NEDD4, NEDD8, NOCT, NOP53, NPLOC4, NPM1, NT5C3B, NUB1, NUDT15, NUDT16, NUDT16L1, PACSIN3, PAIP1, PDCD6IP, PELO, PFKM, PGM2L1, PHKB, PIN1, PITHD1, PML, POP1, PPP1CA, PRPF19, PSMA3, PSMB10, PSMB2, PSMC2, PSMC3, PSMC6, PSMD1, PSMD12, PSMD14, PSMD2, PSMD6, PSMD7, PSMD8, PSME1, PSME2, PSME4, PYGB, RCN3, RDX, RNF114, RNF2, RNF213, RNF40, RNPS1, RPL10A, RPL13, RPL13A, RPL18A, RPL22, RPL24, RPL27, RPL32, RPL36, RPL38, RPL5, RPLP1, RPLP2, RPS13, RPS15A, RPS16,	No change

					RPS18, RPS21, RPS23, RPS4Y1, RPS5, RPS8, RPS9, RSPRY1, RYBP, SEC61B, SERPINE2, SET, SGSH, SIRT1, SLC9A1, SMARCC1, SNF8, SNX1, SNX5, SQSTM1, SSB, SUFU, TPP1, TRIM25, TRIP12, TUT4, UBAC2, UBE2L6, UBR2, UBR4, UBXN2A, UFD1, USP11, USP22, USP28, USP4, USP48, USP5, USP7, USP9X, USP9Y, VLDLR, VPS25, VPS36, WFS1, WWP2, YBX1, YOD1, ZC3HAV1, ZFP36L2, ZMPSTE24]	
regulation of cell cycle process	GO_BiologicalProcess-EBI-UniProt-GOA-ACAP-ARAP_13.05.2021_00h00	2.75E-67	11.636364	96	[AHCTF1, APAF1, APEX1, APLP2, APP, APPL2, CBX3, CBX5, CCN2, CDC5L, CDC73, CDK1, CDK2, CDK5RAP3, CENPV, CETN2, CHMP3, CHORDC1, CLTC, CNOT1, CNOT10, CTNNB1, CUL1, DRG1, DYNC1LI1, DYNLL1, EED, EML3, FEN1, FHL1, FSD1, GIPC1, HSP90AA1, KNTC1, MACROH2A1, MAD1L1, MAD2L1, MAP3K20, MDC1, MGA, MMAB, MSH2, NDRG1, NEDD1, NOP53, NPM1, NUBP2, NUDT16, NUP62, OBSL1, PAFAH1B1, PDCD6IP, PIN1, PKN2, PML, PRPF19, PSMA3, PSMB10, PSMB2, PSMC2, PSMC3, PSMC6, PSMD1, PSMD12, PSMD14, PSMD2, PSMD6, PSMD7, PSMD8, PSME1, PSME2, PSME4, RAD21, RAD9A, RANBP1, RBBP4, RCC1, RDX, RNF2, RPA2, RPRD1B, RYBP, SIRT1, SLC25A15, SMARCD3, SVIL, TIPRL, TOM1L2, TPX2, TRIP13, TUBA4A, TUBB, USP28, ZFP36L2, ZMPSTE24, ZNF207]	Upregulation
aromatic compound catabolic process	GO_BiologicalProcess-EBI-UniProt-GOA-ACAP-ARAP_13.05.2021_00h00	2.75E-67	15.813253	105	[ABCC1, ACOT7, AGO2, ALB, APEX1, BLVRB, CASP3, CECR2, CIRBP, CNOT1, CNOT10, DCPS, DHX34,	Downregulation

					DHX36, DICER1, DUT, EIF3E, ENPP1, EPHX1, FEN1, FMR1, FTO, HINT1, HMOX1, HNRNPA0, HNRNPC, HNRNPR, IGF2BP1, IGF2BP2, KPNB1, LARP1, LARP4B, LRPPRC, MAOA, METTL14, MGAT1, MLH1, MOV10, MRTO4, MTAP, MTREX, MYD88, NOCT, NPM1, NT5C, NT5C3B, NUDT15, NUDT16, NUDT16L1, PAIP1, PDE5A, PELO, PNP, POP1, PSMA3, PSMB10, PSMB2, PSMC2, PSMC3, PSMC6, PSMD1, PSMD12, PSMD14, PSMD2, PSMD6, PSMD7, PSMD8, PSME1, PSME2, PSME4, QDPR, RNPS1, RPL10A, RPL13, RPL13A, RPL18A, RPL22, RPL24, RPL27, RPL32, RPL36, RPL38, RPL5, RPLP1, RPLP2, RPS13, RPS15A, RPS16, RPS18, RPS21, RPS23, RPS4Y1, RPS5, RPS8, RPS9, SAMHD1, SARM1, SET, SOAT1, SSB, TUT4, TYMP, YBX1, ZC3HAV1, ZFP36L2]	
secretion by cell	GO_BiologicalProcess-EBI-UniProt-GOA-ACAP-ARAP_13.05.2021_00h00	2.75E-67	12.473867	179	[ABAT, ACSL4, ACTR2, ADAM10, AGPAT2, ALB, ALDOA, ANO6, ANXA3, ANXA5, APAF1, APEX1, APLP2, APP, APRT, ARFGEF1, ARFIP1, ARHGAP17, ASAH1, ATG7, ATP6V0A1, ATP6V0C, B2M, B4GALT1, BST1, C2CD2L, CANX, CAP1, CASK, CCN2, CCT2, CD109, CD200, CDK16, CHAMP1, CHD7, CHID1, CHMP3, CLU, CSTB, CTSA, CTSC, CXCL12, CYB5R3, CYP51A1, CYRIB, DIAPH1, DNM1L, DPYSL2, DSP, DYNC1LI1, DYNLL1, ENY2, ERP44, EXOC2, EZR, FMR1, FN1, FTH1, FTL, GIPC1, GLA, GLUD1, GLUL, GOLGA7, GPI, GSDMD, GSK3B, GSTP1, HADH, HADHA, HEXB, HGS, HLA-B, HLA-C, HMOX1, HSP90AA1,	No change

					IDH2, IST1, ITGAV, ITIH4, ITM2C, ITSN1, JAGN1, KPNB1, LAMP1, LGALS3BP, LIN7C, LLGL1, LRRC8A, M6PR, MAGED2, MAP4K4, MGST1, MICAL3, MME, MTCH1, MVP, MYH10, MYH9, MYO18A, NHLRC2, NIBAN2, NISCH, P3H1, PAFAH1B1, PAFAH1B2, PCDH7, PCK2, PDAP1, PDCD6IP, PDZD11, PFKM, PHPT1, PIK3C2A, PKM, PLA2G4A, PLD1, PNP, PPIE, PPP3CA, PRDX6, PRKCSH, PSMC2, PSMC3, PSMD1, PSMD12, PSMD14, PSMD2, PSMD6, PSMD7, PTGES2, PYCARD, PYGB, QSOX1, RAB10, RAB11B, RAB1A, RAB3B, RAB3D, RAB5A, RAB8A, RALA, RAP2B, RAP2C, RCN3, RIPK2, SCRIB, SCRN1, SELENOT, SEPTIN2, SEPTIN9, SERPINB1, SERPINE2, SLC12A2, SLC1A5, SLC2A1, SLC44A2, SMAD4, SNF8, SOD1, SPARC, SPHK2, STOM, STXBP3, SYNJ1, TAGLN2, TF, THBS1, TMX3, TOM1, TUBA4A, TUBB, UBE2Q1, UBR4, VAT1, VPS41, WDR1, YKT6]	
response to decreased oxygen levels	GO_BiologicalProcess-EBI-UniProt-GOA-ACAP-ARAP_13.05.2021_00h00	2.75E-67	13.766233	53	[ABAT, ADSL, AK4, APAF1, BNIP1, CASP3, CCN2, CXCL12, DNM1L, HK2, HMOX1, HSP90B1, KCNMA1, LDHA, NDRG1, NF1, NOP53, PDK1, PIN1, PKLR, PLOD2, PML, PRKAA1, PSMA3, PSMB10, PSMB2, PSMC2, PSMC3, PSMC6, PSMD1, PSMD12, PSMD14, PSMD2, PSMD6, PSMD7, PSMD8, PSME1, PSME2, PSME4, PTGIS, SCFD1, SIRT1, SLC11A2, SLC1A1, SLC2A1, SLC9A1, SMAD4, SOD2, SRC, TGFBR3, THBS1, TIGAR, TM9SF4]	Downregulation
cellular amide metabolic process	GO_BiologicalProcess-EBI-UniProt-GOA-	2.75E-67	15.396953	192	[AARS1, AASDHPPT, AASS, ABCE1, ACACA, ACO1,	No change

	ACAP-ARAP_13.05.2021_00h00				ACOT7, ACOT8, ACSF2, ACSL3, ACSL4, ACSS2, ADAM10, ADSL, AGO2, APEX1, APP, ASAH1, ASNS, B4GALT5, BDH2, BIN1, C1QBP, CASP3, CERS6, CIRBP, CLIC1, CLU, CNOT1, CNOT10, CPD, DAPK1, DARS1, DCPS, DDX1, DENR, DHFR, DHPS, DHX36, DLST, DRG1, EARS2, ECE1, EEF1E1, EEF1G, EFL1, EIF2AK2, EIF2B1, EIF2D, EIF2S1, EIF3A, EIF3B, EIF3E, EIF3F, EIF4G3, EIF6, EPRS1, ERAP1, ERBB2, ETHE1, FAM3A, FASN, FMR1, FTO, G6PD, GAPDH, GARS1, GATB, GBA2, GCDH, GDAP1, GGCT, GLA, GLE1, GLO1, GNE, GSTM2, GSTM3, GSTP1, HARS1, HEXB, HMGCS1, HNRNPR, HSD17B4, IARS2, IGF2BP1, IGF2BP2, ILF3, IMPACT, IREB2, LARP1, LARP4B, LARP6, LNPEP, LRPPRC, METTL14, MLH1, MME, MOV10, MRPL10, MRPS17, MRPS21, MRPS31, MRRF, MTHFD2, MTRR, MVD, MVK, N6AMT1, NCK2, NCL, NDRG1, NIBAN1, NOCT, NPM1, NSUN5, NT5C3B, OGFOD1, OPLAH, OXA1L, PAIP1, PARL, PC, PDK1, PDZD11, PELO, PIN1, PKM, PLA2G15, PLD1, PLPP3, PLXNB2, PPP1CA, PPP3CA, PRKAA1, PRKCSH, PTGES2, PUS7, QARS1, RARS2, RBM4, RPL10A, RPL13, RPL13A, RPL18A, RPL22, RPL24, RPL27, RPL32, RPL36, RPL38, RPL5, RPLP1, RPLP2, RPS13, RPS15A, RPS16, RPS18, RPS21, RPS23, RPS4Y1, RPS5, RPS8, RPS9, RTN4, SARNP, SARS1, SEC11C, SELENOT, SESN2, SHFL, SHMT2, SLC1A1, SLC25A15,	

					SMPD1, SOAT1, SOD1, SPHK2, SPTLC1, SPTLC2, TBCE, THBS1, THEM4, TIGAR, TPP1, TRMT10C, TSPO, TUT4, VARS1, YARS1, YBX1, ZFP36L2]	
cellular nitrogen compound catabolic process	GO_BiologicalProcess-EBI-UniProt-GOA-ACAP-ARAP_13.05.2021_00h00	2.75E-67	16.310976	107	[ABCC1, ACOT7, AGO2, ALB, APEX1, BLVRB, CASP3, CECR2, CIRBP, CNOT1, CNOT10, DCPS, DHX34, DHX36, DICER1, DPYSL2, DPYSL3, DPYSL4, DUT, EIF3E, ENPP1, FEN1, FMR1, FTO, HINT1, HMOX1, HNRNPA0, HNRNPC, HNRNPR, IGF2BP1, IGF2BP2, KPNB1, LARP1, LARP4B, LRPPRC, METTL14, MGAT1, MLH1, MOV10, MPST, MRTO4, MTAP, MTREX, MYD88, NOCT, NPM1, NT5C, NT5C3B, NUDT15, NUDT16, NUDT16L1, PAIP1, PDE5A, PELO, PNP, POP1, PSMA3, PSMB10, PSMB2, PSMC2, PSMC3, PSMC6, PSMD1, PSMD12, PSMD14, PSMD2, PSMD6, PSMD7, PSMD8, PSME1, PSME2, PSME4, RNPS1, RPL10A, RPL13, RPL13A, RPL18A, RPL22, RPL24, RPL27, RPL32, RPL36, RPL38, RPL5, RPLP1, RPLP2, RPS13, RPS15A, RPS16, RPS18, RPS21, RPS23, RPS4Y1, RPS5, RPS8, RPS9, SAMHD1, SARM1, SET, SOAT1, SSB, TUT4, TYMP, VDAC2, YBX1, ZC3HAV1, ZFP36L2]	Downregulation
heterocycle catabolic process	GO_BiologicalProcess-EBI-UniProt-GOA-ACAP-ARAP_13.05.2021_00h00	2.75E-67	16.25387	105	[ABCC1, ACOT7, AGO2, ALB, APEX1, BLVRB, CASP3, CECR2, CIRBP, CNOT1, CNOT10, DCPS, DHX34, DHX36, DICER1, DPYSL2, DPYSL3, DPYSL4, DUT, EIF3E, ENPP1, FEN1, FMR1, FTO, HINT1, HMOX1, HNRNPA0, HNRNPC, HNRNPR, IGF2BP1, IGF2BP2, KPNB1, LARP1, LARP4B, LRPPRC, METTL14, MGAT1, MLH1, MOV10, MRTO4,	Downregulation

					MTAP, MTREX, MYD88, NOCT, NPM1, NT5C, NT5C3B, NUDT15, NUDT16, NUDT16L1, PAIP1, PDE5A, PELO, PNP, POP1, PSMA3, PSMB10, PSMB2, PSMC2, PSMC3, PSMC6, PSMD1, PSMD12, PSMD14, PSMD2, PSMD6, PSMD7, PSMD8, PSME1, PSME2, PSME4, RNPS1, RPL10A, RPL13, RPL13A, RPL18A, RPL22, RPL24, RPL27, RPL32, RPL36, RPL38, RPL5, RPLP1, RPLP2, RPS13, RPS15A, RPS16, RPS18, RPS21, RPS23, RPS4Y1, RPS5, RPS8, RPS9, SAMHD1, SARM1, SET, SOAT1, SSB, TUT4, TYMP, YBX1, ZC3HAV1, ZFP36L2]	
secretion	GO_BiologicalProcess-EBI-UniProt-GOA-ACAP-ARAP_13.05.2021_00h00	2.75E-67	11.838791	188	[ABAT, ACSL3, ACSL4, ACTR2, ADAM10, AGPAT2, ALB, ALDOA, ANO6, ANXA3, ANXA5, APAF1, APEX1, APLP2, APP, APRT, ARFGEF1, ARFIP1, ARHGAP17, ASAH1, ATG7, ATP6V0A1, ATP6V0C, B2M, B4GALT1, BST1, C2CD2L, CANX, CAP1, CASK, CCN2, CCT2, CD109, CD200, CDK16, CHAMP1, CHD7, CHID1, CHMP3, CLU, CSTB, CTSA, CTSC, CXCL12, CYB5R3, CYP51A1, CYRIB, DIAPH1, DNM1L, DPYSL2, DSP, DYNC1LI1, DYNLL1, ENY2, ERP44, EXOC2, EZR, FMR1, FN1, FTH1, FTL, GIPC1, GLA, GLUD1, GLUL, GOLGA7, GPI, GSDMD, GSK3B, GSTP1, HADH, HADHA, HEXB, HGS, HK2, HLA-B, HLA-C, HMOX1, HSP90AA1, IDH2, IST1, ITGAV, ITIH4, ITM2C, ITSN1, JAGN1, KCNQ1, KPNB1, LAMP1, LGALS3BP, LIN7C, LLGL1, LRRC8A, M6PR, MAGED2, MAP4K4, MGST1, MICAL3, MME, MTCH1, MVP, MYH10, MYH9,	No change

					MYO18A, MYO6, NHLRC2, NIBAN2, NISCH, P3H1, PAFAH1B1, PAFAH1B2, PCDH7, PCK2, PDAP1, PDCD6IP, PDZD11, PFKM, PHPT1, PIK3C2A, PKM, PLA2G4A, PLD1, PNP, PPIE, PPP3CA, PRDX6, PRKAA1, PRKCI, PRKCSH, PSMC2, PSMC3, PSMD1, PSMD12, PSMD14, PSMD2, PSMD6, PSMD7, PTGES2, PYCARD, PYGB, QSOX1, RAB10, RAB11B, RAB1A, RAB3B, RAB3D, RAB5A, RAB8A, RALA, RAP2B, RAP2C, RCN3, RIPK2, SCRIB, SCRN1, SELENOT, SEPTIN2, SEPTIN9, SERPINB1, SERPINE2, SLC12A2, SLC1A5, SLC2A1, SLC44A2, SMAD4, SNF8, SNX5, SOD1, SPARC, SPHK2, STAT5B, STOM, STXBP3, SYNJ1, TAGLN2, TF, THBS1, TMX3, TOM1, TUBA4A, TUBB, UBE2Q1, UBR4, UMPS, VAT1, VPS41, WDR1, YKT6]	
organic substance transport	GO_BiologicalProcess-EBI-UniProt-GOA-ACAP-ARAP_13.05.2021_00h00	2.75E-67	11.826783	325	[ABAT, ABCB7, ABCC1, ACACA, ACSL3, ACSL4, ACTB, ADAMTS9, AHCTF1, ALDH3A2, ANK3, ANO6, AP3M2, AP5B1, APEX1, APLP2, APPL2, ARFGAP1, ARFGEF1, ARFIP1, ARPP19, ARRB1, ASPSCR1, ATG4B, ATG7, ATP2C1, ATP6V0A1, ATP6V0C, ATP6V1A, ATP6V1B2, AUP1, B3GAT3, BET1, C2CD2L, CANX, CCDC22, CD200, CD81, CDK1, CDK16, CEMIP, CETN2, CHD7, CHMP3, CHORDC1, CHP1, CHTOP, CLTC, CLTCL1, CLU, COG6, COG7, CORO7, CPSF2, CROT, CTSA, CXCL12, CYP51A1, DAB2, DENND2A, DERL1, DNM1L, DPYSL2, DYNLL1, EHD3, EIF2D, ENPP1, ENY2, EPRS1, ERBB2, ESYT1, EXOC2, EZR, FAM91A1, FERMT1,	No change

					FMR1, GCC2, GDAP1, GET3, GGA2, GIPC1, GLE1, GLUD1, GLUL, GOLGA7, GOLT1B, GRAMD1A, GULP1, HACL1, HADH, HADHA, HEATR3, HERC2, HGS, HIP1, HK2, HNRNPA3, HPX, HSP90AA1, HSP90B1, HSPA4, HSPA5, HSPB11, IDH2, IGF2BP1, IGF2BP2, IPO5, IPO9, IST1, ITGAV, ITGB1, ITSN1, JAGN1, KPNB1, KRT18, LIN7C, LLGL1, LRP10, LRPPRC, LRRC8A, LRRC8E, M6PR, MACF1, MAGI1, MAP4K4, MFF, MFSD10, MGST1, MICALL1, MTCH1, MVP, MYH10, MYH9, MYO18A, MYO1D, MYO1E, MYO6, NDUFA13, NEDD4, NISCH, NMD3, NMT1, NPLOC4, NPM1, NUDT19, NUP205, NUP35, NUP62, NUTF2, OSBPL10, OSBPL11, OSBPL1A, OSBPL2, OSBPL8, OXA1L, OXSR1, P3H1, PAFAH1B1, PARL, PCK2, PDCD6IP, PFKM, PHPT1, PIN1, PITRM1, PLA2G4A, PML, PPP1R7, PPP3CA, PRKAA1, PRKAB2, PRKCI, PTGES2, PTRH2, RAB10, RAB11B, RAB1A, RAB23, RAB3B, RAB3D, RAB5A, RAB8A, RABGEF1, RANGAP1, RBM22, RCN3, REEP2, RELCH, RHOB, RIPK2, RNF2, RNPS1, RPL10A, RPL13, RPL13A, RPL18A, RPL22, RPL24, RPL27, RPL32, RPL36, RPL38, RPL5, RPLP1, RPLP2, RPS13, RPS15A, RPS16, RPS18, RPS21, RPS23, RPS4Y1, RPS5, RPS8, RPS9, SAE1, SARNP, SCARB2, SCFD1, SCP2, SCRIB, SEC14L2, SEC23B, SEC61A1, SEC61B, SELENBP1, SELENOT, SEPTIN9, SESN2, SFXN2, SFXN3, SH3BP4, SIRT1,	

					SLC12A2, SLC16A3, SLC17A5, SLC1A1, SLC1A4, SLC1A5, SLC25A10, SLC25A12, SLC25A13, SLC25A15, SLC25A19, SLC25A22, SLC25A24, SLC25A26, SLC25A4, SLC25A5, SLC25A6, SLC26A2, SLC2A1, SLC44A1, SLC44A2, SLC4A7, SLC7A6, SNF8, SNX1, SNX18, SNX21, SNX27, SNX30, SNX5, SOAT1, SORT1, SRC, SRPRA, SRSF2, SRSF5, SSB, SSR3, STARD5, STOM, STXBP3, SUFU, TAP1, TBC1D10A, TBC1D17, TBC1D22B, TBC1D4, TBC1D8B, TCAF1, TCP1, TF, THBS1, THOC2, TIMM17B, TM9SF4, TMED9, TNPO2, TOM1, TOM1L2, TOMM34, TOMM7, TRAM1, TRAM2, TRIM28, TSC2, TSPO, TTC39B, TXN, UBAC2, UBE2Q1, UFD1, UNC119B, UNC93B1, VLDLR, VPS25, VPS26B, VPS33B, VPS36, VPS41, WASHC5, WDR11, WWP2, XPO4, XPO6, XPOT, YBX1, YES1, YKT6, YOD1, YTHDC1, ZDHHC21, ZFAND6]	
nitrogen compound transport	GO_BiologicalProcess-EBI-UniProt-GOA-ACAP-ARAP_13.05.2021_00h00	2.75E-67	12.521664	289	[ABAT, ABCB7, ABCC1, ACSL3, ACSL4, ACTB, ADAMTS9, AHCTF1, ALDH3A2, ANK3, AP3M2, AP5B1, APEX1, APLP2, APPL2, ARFGAP1, ARFGEF1, ARFIP1, ARRB1, ASPSCR1, ATG4B, ATG7, ATP2C1, ATP6V0A1, ATP6V0C, ATP6V1A, ATP6V1B2, AUP1, B3GAT3, BET1, C2CD2L, CANX, CCDC22, CD200, CD81, CDK1, CDK16, CEMIP, CETN2, CHD7, CHMP3, CHORDC1, CHP1, CHTOP, CLTC, CLTCL1, CLU, COG6, COG7, CORO7, CPSF2, CTSA, CXCL12, CYP51A1, DAB2, DENND2A, DERL1, DNM1L,	No change

					DPYSL2, DYNLL1, EHD3, EIF2D, ENY2, ERBB2, EXOC2, EZR, FAM91A1, FERMT1, FMR1, GCC2, GDAP1, GET3, GGA2, GIPC1, GLE1, GLUD1, GLUL, GOLGA7, GOLT1B, HACL1, HADH, HADHA, HEATR3, HERC2, HGS, HIP1, HNRNPA3, HPX, HSP90AA1, HSP90B1, HSPA4, HSPA5, HSPB11, IDH2, IGF2BP1, IGF2BP2, IPO5, IPO9, IST1, ITGB1, ITSN1, JAGN1, KPNB1, KRT18, LIN7C, LLGL1, LRPPRC, LRRC8A, LRRC8E, M6PR, MACF1, MAGI1, MAP4K4, MFF, MGST1, MICALL1, MTCH1, MVP, MYH10, MYH9, MYO18A, MYO1D, MYO1E, MYO6, NDUFA13, NEDD4, NISCH, NMD3, NMT1, NPLOC4, NPM1, NUDT19, NUP205, NUP35, NUP62, NUTF2, OXA1L, OXSR1, P3H1, PAFAH1B1, PARL, PCK2, PDCD6IP, PFKM, PHPT1, PIN1, PITRM1, PML, PPP3CA, PRKAA1, PRKCI, PTGES2, RAB10, RAB11B, RAB1A, RAB23, RAB3B, RAB3D, RAB5A, RAB8A, RABGEF1, RANGAP1, RBM22, RCN3, REEP2, RHOB, RIPK2, RNF2, RNPS1, RPL10A, RPL13, RPL13A, RPL18A, RPL22, RPL24, RPL27, RPL32, RPL36, RPL38, RPL5, RPLP1, RPLP2, RPS13, RPS15A, RPS16, RPS18, RPS21, RPS23, RPS4Y1, RPS5, RPS8, RPS9, SAE1, SARNP, SCARB2, SCFD1, SCRIB, SEC14L2, SEC23B, SEC61A1, SEC61B, SELENBP1, SELENOT, SEPTIN9, SFXN2, SFXN3, SH3BP4, SLC12A2, SLC17A5, SLC1A1, SLC1A4, SLC1A5, SLC25A12, SLC25A13, SLC25A15,	

					SLC25A19, SLC25A22, SLC25A24, SLC25A4, SLC25A5, SLC25A6, SLC2A1, SLC44A1, SLC44A2, SLC7A6, SNF8, SNX1, SNX18, SNX21, SNX27, SNX30, SNX5, SORT1, SRC, SRPRA, SRSF2, SRSF5, SSB, SSR3, STOM, STXBP3, SUFU, TAP1, TBC1D10A, TBC1D17, TBC1D22B, TBC1D4, TBC1D8B, TCAF1, TCP1, TF, THOC2, TIMM17B, TM9SF4, TMED9, TNPO2, TOM1, TOM1L2, TOMM34, TOMM7, TRAM1, TRAM2, TRIM28, TSC2, TSPO, TXN, UBAC2, UBE2Q1, UFD1, UNC119B, UNC93B1, VLDLR, VPS25, VPS26B, VPS33B, VPS36, VPS41, WASHC5, WDR11, WWP2, XPO4, XPO6, XPOT, YBX1, YKT6, YOD1, YTHDC1, ZDHHC21, ZFAND6]	
organic cyclic compound catabolic process	GO_BiologicalProcess-EBI-UniProt-GOA-ACAP-ARAP_13.05.2021_00h00	2.75E-67	15.616046	109	[ABCC1, ACOT7, AGO2, ALB, APEX1, BLVRB, CASP3, CECR2, CIRBP, CNOT1, CNOT10, DCPS, DHX34, DHX36, DICER1, DPYSL2, DPYSL3, DPYSL4, DUT, EIF3E, ENPP1, FEN1, FMR1, FTO, HINT1, HMOX1, HNRNPA0, HNRNPC, HNRNPR, HSD17B11, IGF2BP1, IGF2BP2, KPNB1, LARP1, LARP4B, LRPPRC, MAOA, METTL14, MGAT1, MLH1, MOV10, MRTO4, MTAP, MTREX, MYD88, NOCT, NPM1, NT5C, NT5C3B, NUDT15, NUDT16, NUDT16L1, PAIP1, PDE5A, PELO, PNP, POP1, PSMA3, PSMB10, PSMB2, PSMC2, PSMC3, PSMC6, PSMD1, PSMD12, PSMD14, PSMD2, PSMD6, PSMD7, PSMD8, PSME1, PSME2, PSME4, QDPR, RNPS1, RPL10A, RPL13, RPL13A, RPL18A, RPL22, RPL24, RPL27, RPL32, RPL36, RPL38, RPL5, RPLP1,	Downregulation

					RPLP2, RPS13, RPS15A, RPS16, RPS18, RPS21, RPS23, RPS4Y1, RPS5, RPS8, RPS9, SAMHD1, SARM1, SET, SOAT1, SSB, SULT1E1, TUT4, TYMP, YBX1, ZC3HAV1, ZFP36L2]	
organonitrogen compound catabolic process	GO_BiologicalProcess-EBI-UniProt-GOA-ACAP-ARAP_13.05.2021_00h00	2.75E-67	13.006993	186	[AASS, ABAT, ABCC1, ABHD12, ABHD16A, ACAD8, ACADSB, ACOT7, ADAMTS9, AGRN, ALB, ANKZF1, ARAF, ARRB1, ASAH1, ATE1, ATG4B, ATG7, AUP1, BCAT1, BLVRB, CCDC22, CD81, CDC34, CDK1, CDK2, CDK5RAP3, CEMIP, CHORDC1, CHP1, CLTC, CLU, CRBN, CSPG4, CTNNB1, CTSA, CTSC, CUL1, CUL4B, CYP51A1, DAB2, DCN, DDI2, DERL1, DLST, DPYSL2, DPYSL3, DPYSL4, DUT, ECE1, ERAP1, ERLIN2, EZR, FAM3A, FBXL12, FBXW8, FGF2, FMR1, GBA2, GCAT, GCDH, GIPC1, GLA, GLMN, GLUD1, GLUL, GOLT1B, GOT1, GPC1, GPC6, GSK3B, HEXB, HGS, HIBCH, HINT1, HMOX1, HSD17B10, HSP90AA1, HSP90B1, HSPA5, HSPG2, LNPEP, LUM, LYPLA2, MAD2L1, MAEA, MAGI1, MALT1, MAOA, MPST, MSN, MTA1, MTAP, MTRR, NDUFA13, NEDD4, NEDD8, NOP53, NPLOC4, NT5C, NUB1, NUDT15, NUDT16, OAT, PACSIN3, PDCD6IP, PDE5A, PDXDC1, PIN1, PITHD1, PLA2G15, PLA2G4A, PML, PNP, PRPF19, PSMA3, PSMB10, PSMB2, PSMC2, PSMC3, PSMC6, PSMD1, PSMD12, PSMD14, PSMD2, PSMD6, PSMD7, PSMD8, PSME1, PSME2, PSME4, QDPR, RCN3, RDX, RNF114, RNF2, RNF213, RNF40, RPL5, RSPRY1, RYBP, SAMHD1, SARM1, SBDS, SCLY, SEC61B, SERPINE2, SGSH,	No change

					SHMT2, SIRT1, SLC44A1, SLC9A1, SMARCC1, SMPD1, SNF8, SNX1, SNX5, SOAT1, SQSTM1, SUFU, TPP1, TRIM25, TRIP12, TYMP, UBAC2, UBE2L6, UBR2, UBR4, UBXN2A, UFD1, USP11, USP22, USP28, USP4, USP48, USP5, USP7, USP9X, USP9Y, VLDLR, VPS25, VPS36, WFS1, WWP2, YOD1, ZMPSTE24]	
endopeptidase complex	GO_CellularComponent-EBI-UniProt-GOA-ACAP-ARAP_13.05.2021_00h00	2.75E-67	25.352112	18	[PIGT, PIGU, PSMA3, PSMB10, PSMB2, PSMC2, PSMC3, PSMC6, PSMD1, PSMD12, PSMD14, PSMD2, PSMD6, PSMD7, PSMD8, PSME1, PSME2, PSME4]	Downregulation
proteasome regulatory particle	GO_CellularComponent-EBI-UniProt-GOA-ACAP-ARAP_13.05.2021_00h00	2.75E-67	43.47826	10	[PSMC2, PSMC3, PSMC6, PSMD1, PSMD12, PSMD14, PSMD2, PSMD6, PSMD7, PSMD8]	Downregulation
myeloid cell activation involved in immune response	GO_BiologicalProcess-EBI-UniProt-GOA-ACAP-ARAP_13.05.2021_00h00	2.75E-67	13.517916	83	[ACTR2, ADAM10, AGPAT2, ALDOA, ANO6, ANXA3, APAF1, APRT, ASAH1, ATG7, ATP6V0A1, ATP6V0C, B2M, B4GALT1, BST1, CAP1, CCT2, CHAMP1, CLU, CSTB, CTSA, CTSC, CYB5R3, DIAPH1, DSP, DYNC1LI1, DYNLL1, ERP44, FTH1, FTL, GLA, GOLGA7, GPI, GSDMD, GSTP1, HEXB, HLA-B, HLA-C, HMOX1, HSP90AA1, IST1, ITGAV, ITM2C, KPNB1, LAMP1, MGST1, MME, MTCH1, MVP, MYD88, PAFAH1B2, PDAP1, PKM, PLD1, PNP, PPIE, PRDX6, PRKCSH, PSMC2, PSMC3, PSMD1, PSMD12, PSMD14, PSMD2, PSMD6, PSMD7, PTGES2, PYCARD, PYGB, QSOX1, RAB10, RAB3D, RAP2B, RAP2C, SERPINB1, SLC44A2, SPHK2, STOM, STXBP3, TOM1, TUBB, UBR4, VAT1]	Downregulation
peptide metabolic process	GO_BiologicalProcess-EBI-UniProt-GOA-ACAP-	2.75E-67	15.74468	148	[AARS1, ABCE1, ACO1, ADAM10, AGO2, APEX1, APP, BDH2, BIN1, C1QBP,	No change

	ARAP_13.05.2021_00h00				CASP3, CIRBP, CLIC1, CLU, CNOT1, CNOT10, CPD, DAPK1, DARS1, DCPS, DDX1, DENR, DHFR, DHPS, DHX36, DRG1, EARS2, ECE1, EEF1E1, EEF1G, EFL1, EIF2AK2, EIF2B1, EIF2D, EIF2S1, EIF3A, EIF3B, EIF3E, EIF3F, EIF4G3, EIF6, EPRS1, ERAP1, ERBB2, ETHE1, FMR1, FTO, G6PD, GAPDH, GARS1, GATB, GDAP1, GGCT, GLE1, GLO1, GSTM2, GSTM3, GSTP1, HARS1, HNRNPR, IARS2, IGF2BP1, IGF2BP2, ILF3, IMPACT, IREB2, LARP1, LARP4B, LARP6, LNPEP, LRPPRC, METTL14, MLH1, MME, MOV10, MRPL10, MRPS17, MRPS21, MRPS31, MRRF, N6AMT1, NCK2, NCL, NDRG1, NIBAN1, NOCT, NPM1, NSUN5, NT5C3B, OGFOD1, OPLAH, OXA1L, PAIP1, PARL, PELO, PIN1, PKM, PLD1, PLXNB2, PPP1CA, PRKCSH, PTGES2, PUS7, QARS1, RARS2, RBM4, RPL10A, RPL13, RPL13A, RPL18A, RPL22, RPL24, RPL27, RPL32, RPL36, RPL38, RPL5, RPLP1, RPLP2, RPS13, RPS15A, RPS16, RPS18, RPS21, RPS23, RPS4Y1, RPS5, RPS8, RPS9, RTN4, SARNP, SARS1, SEC11C, SELENOT, SESN2, SHFL, SHMT2, SLC1A1, SOD1, TBCE, THBS1, TPP1, TRMT10C, TUT4, VARS1, YARS1, YBX1, ZFP36L2]	
exocytosis	GO_BiologicalProcess-EBI-UniProt-GOA-ACAP-ARAP_13.05.2021_00h00	2.75E-67	13.849287	136	[ACTR2, ADAM10, AGPAT2, ALB, ALDOA, ANO6, ANXA3, ANXA5, APAF1, APEX1, APLP2, APP, APRT, ARFGEF1, ARHGAP17, ASAH1, ATG7, ATP6V0A1, ATP6V0C, B2M, B4GALT1, BST1, CAP1, CASK, CCT2, CD109, CDK16, CHAMP1, CHID1,	No change

					CHMP3, CLU, CSTB, CTSA, CTSC, CYB5R3, CYRIB, DIAPH1, DNM1L, DSP, DYNC1LI1, DYNLL1, ERP44, EXOC2, FMR1, FN1, FTH1, FTL, GIPC1, GLA, GOLGA7, GPI, GSDMD, GSK3B, GSTP1, HEXB, HGS, HLA-B, HLA-C, HMOX1, HSP90AA1, IST1, ITGAV, ITIH4, ITM2C, ITSN1, JAGN1, KPNB1, LAMP1, LGALS3BP, LIN7C, LLGL1, MAGED2, MGST1, MICAL3, MME, MTCH1, MVP, MYH9, NHLRC2, PAFAH1B2, PCDH7, PDAP1, PDCD6IP, PIK3C2A, PKM, PLD1, PNP, PPIE, PRDX6, PRKCSH, PSMC2, PSMC3, PSMD1, PSMD12, PSMD14, PSMD2, PSMD6, PSMD7, PTGES2, PYCARD, PYGB, QSOX1, RAB10, RAB11B, RAB3B, RAB3D, RAB5A, RAB8A, RALA, RAP2B, RAP2C, SCRIB, SCRN1, SEPTIN2, SEPTIN9, SERPINB1, SLC44A2, SNF8, SOD1, SPARC, SPHK2, STOM, STXBP3, SYNJ1, TAGLN2, TF, THBS1, TMX3, TOM1, TUBA4A, TUBB, UBR4, VAT1, VPS41, WDR1, YKT6]	
regulation of mitotic cell cycle	GO_BiologicalProcess-EBI-UniProt-GOA-ACAP-ARAP_13.05.2021_00h00	2.75E-67	12.355848	75	[APEX1, APLP2, APP, APPL2, ASNS, CDC73, CDK1, CDK2, CDK5RAP3, CETN2, CNOT1, CNOT10, CTNNB1, CUL1, DUSP3, DYNC1LI1, DYNLL1, FHL1, HSP90AA1, KNTC1, MAD1L1, MAD2L1, MAEA, MAP3K20, MDC1, MSH2, NEDD1, NLE1, NOP53, NUP62, OBSL1, PAFAH1B1, PIN1, PKN2, PML, PSMA3, PSMB10, PSMB2, PSMC2, PSMC3, PSMC6, PSMD1, PSMD12, PSMD14, PSMD2, PSMD6, PSMD7, PSMD8, PSME1, PSME2, PSME4, PTGES2, RAD21, RAD9A, RCC1, RDX, RNF40, RPA2, SCRIB,	Upregulation

					SIRT1, SLC25A15, SMARCD3, SRA1, STAT5B, TOM1L2, TPX2, TRIP13, TTC28, TTLL12, TUBA4A, TUBB, USP22, ZFP36L2, ZMPSTE24, ZNF207]	
negative regulation of cell cycle process	GO_BiologicalProcess-EBI-UniProt-GOA-ACAP-ARAP_13.05.2021_00h00	2.75E-67	13.801453	57	[APAF1, CBX3, CBX5, CDC5L, CDC73, CDK1, CDK2, CDK5RAP3, CNOT1, CNOT10, CUL1, DYNC1LI1, EED, FHL1, KNTC1, MACROH2A1, MAD1L1, MAD2L1, MAP3K20, MDC1, MGA, MMAB, MSH2, NOP53, NPM1, NUBP2, PML, PRPF19, PSMA3, PSMB10, PSMB2, PSMC2, PSMC3, PSMC6, PSMD1, PSMD12, PSMD14, PSMD2, PSMD6, PSMD7, PSMD8, PSME1, PSME2, PSME4, RAD21, RAD9A, RBBP4, RNF2, RPA2, RYBP, SLC25A15, TIPRL, TOM1L2, TRIP13, USP28, ZFP36L2, ZNF207]	Upregulation
protein transport	GO_BiologicalProcess-EBI-UniProt-GOA-ACAP-ARAP_13.05.2021_00h00	2.75E-67	13.440266	243	[ABAT, ACSL3, ACSL4, ADAMTS9, AHCTF1, ALDH3A2, ANK3, AP3M2, AP5B1, APEX1, APLP2, APPL2, ARFGAP1, ARFGEF1, ARFIP1, ARRB1, ASPSCR1, ATG4B, ATG7, ATP2C1, ATP6V0A1, ATP6V0C, ATP6V1A, ATP6V1B2, AUP1, B3GAT3, BET1, C2CD2L, CANX, CCDC22, CD200, CD81, CDK1, CDK16, CEMIP, CETN2, CHMP3, CHORDC1, CHP1, CLTC, CLTCL1, CLU, COG6, COG7, CORO7, CTSA, CYP51A1, DAB2, DENND2A, DERL1, DNM1L, DYNLL1, EHD3, EIF2D, ENY2, ERBB2, EXOC2, EZR, FAM91A1, FERMT1, GCC2, GDAP1, GET3, GGA2, GIPC1, GLE1, GLUD1, GLUL, GOLGA7, GOLT1B, HACL1, HADH, HADHA, HEATR3, HERC2, HGS, HIP1, HSP90AA1, HSP90B1, HSPA4, HSPA5, HSPB11, IDH2, IPO5, IPO9,	No change

					IST1, ITSN1, JAGN1, KPNB1, KRT18, LIN7C, LLGL1, LRRC8A, M6PR, MACF1, MAGI1, MAP4K4, MFF, MICALL1, MVP, MYH10, MYH9, MYO18A, MYO1D, MYO1E, MYO6, NDUFA13, NEDD4, NMD3, NMT1, NPLOC4, NPM1, NUDT19, NUP205, NUP35, NUP62, NUTF2, OXA1L, P3H1, PAFAH1B1, PARL, PCK2, PDCD6IP, PFKM, PHPT1, PIN1, PITRM1, PML, PPP3CA, PRKAA1, PRKCI, PTGES2, RAB10, RAB11B, RAB1A, RAB23, RAB3B, RAB3D, RAB5A, RAB8A, RABGEF1, RANGAP1, RBM22, RCN3, REEP2, RHOB, RNF2, RPL10A, RPL13, RPL13A, RPL18A, RPL22, RPL24, RPL27, RPL32, RPL36, RPL38, RPL5, RPLP1, RPLP2, RPS13, RPS15A, RPS16, RPS18, RPS21, RPS23, RPS4Y1, RPS5, RPS8, RPS9, SAE1, SCARB2, SCFD1, SCRIB, SEC14L2, SEC23B, SEC61A1, SEC61B, SELENBP1, SELENOT, SEPTIN9, SLC12A2, SLC1A1, SLC25A6, SLC2A1, SNF8, SNX1, SNX18, SNX21, SNX27, SNX30, SNX5, SORT1, SRC, SRPRA, SSR3, STOM, STXBP3, SUFU, TAP1, TBC1D10A, TBC1D17, TBC1D22B, TBC1D4, TBC1D8B, TCAF1, TCP1, TF, TIMM17B, TM9SF4, TMED9, TNPO2, TOM1, TOM1L2, TOMM34, TOMM7, TRAM1, TRAM2, TRIM28, TSC2, TSPO, TXN, UBAC2, UBE2Q1, UFD1, UNC119B, UNC93B1, VLDLR, VPS25, VPS26B, VPS33B, VPS36, VPS41, WASHC5, WDR11, WWP2, XPO4, XPO6, XPOT, YKT6, YOD1, ZDHHC21, ZFAND6]	

cytosolic ribosome	GO_CellularComponent-EBI-UniProt-GOA-ACAP-ARAP_13.05.2021_00h00	2.75E-67	21.367521	25	[EIF2D, EIF2S1, RPL10A, RPL13, RPL13A, RPL18A, RPL22, RPL24, RPL27, RPL32, RPL36, RPL38, RPL5, RPLP1, RPLP2, RPS13, RPS15A, RPS16, RPS18, RPS21, RPS23, RPS4Y1, RPS5, RPS8, RPS9]	Downregulation
protein catabolic process	GO_BiologicalProcess-EBI-UniProt-GOA-ACAP-ARAP_13.05.2021_00h00	2.75E-67	12.108981	120	[ABHD12, ADAMTS9, ANKZF1, ARAF, ARRB1, ATE1, ATG4B, ATG7, AUP1, CCDC22, CD81, CDC34, CDK1, CDK2, CDK5RAP3, CLTC, CLU, CRBN, CTNNB1, CTSA, CTSC, CUL1, CUL4B, CYP51A1, DAB2, DDI2, DERL1, ERLIN2, EZR, FBXL12, FBXW8, FMR1, GIPC1, GLMN, GPC1, GSK3B, HGS, HSP90AA1, HSP90B1, HSPA5, LYPLA2, MAD2L1, MAEA, MAGI1, MALT1, MSN, MTA1, NDUFA13, NEDD4, NEDD8, NOP53, NPLOC4, NUB1, NUDT15, PACSIN3, PDCD6IP, PIN1, PITHD1, PML, PRPF19, PSMA3, PSMB10, PSMB2, PSMC2, PSMC3, PSMC6, PSMD1, PSMD12, PSMD14, PSMD2, PSMD6, PSMD7, PSMD8, PSME1, PSME2, PSME4, RCN3, RDX, RNF114, RNF2, RNF213, RNF40, RPL5, RSPRY1, RYBP, SEC61B, SERPINE2, SGSH, SIRT1, SMARCC1, SNF8, SNX1, SNX5, SQSTM1, SUFU, TPP1, TRIM25, TRIP12, UBAC2, UBE2L6, UBR2, UBR4, UBXN2A, UFD1, USP11, USP22, USP28, USP4, USP48, USP5, USP7, USP9X, USP9Y, VLDLR, VPS25, VPS36, WFS1, WWP2, YOD1, ZMPSTE24]	No change
regulation of intracellular transport	GO_BiologicalProcess-EBI-UniProt-GOA-ACAP-ARAP_13.05.2021_00h00	2.75E-67	13.917526	54	[ANK3, APEX1, ARFIP1, B3GAT3, CD81, CDK1, CEMIP, CHMP3, CHORDC1, CHP1, DAB2, ERBB2, EZR, FERMT1, GCC2, IPO5, LAMP1, MAGI1, MAP1B,	Upregulation

					MFF, MSN, MYO1E, NEDD4, NMT1, NUP62, NUTF2, PARL, PIN1, PRKAA1, RAB11B, RAB23, RANGAP1, RBM22, RBM4, RDX, REEP2, REEP6, RNF2, SAE1, SCFD1, SCP2, SLC1A1, SRC, STOM, SUFU, TCAF1, TM9SF4, TOMM7, TRIM28, TXN, UBAC2, USP7, XPO4, YOD1]	
regulation of cellular amide metabolic process	GO_BiologicalProcess-EBI-UniProt-GOA-ACAP-ARAP_13.05.2021_00h00	2.75E-67	16.181818	89	[AARS1, ABCE1, ACO1, AGO2, APEX1, APP, BIN1, C1QBP, CASP3, CIRBP, CLU, CNOT1, CNOT10, DAPK1, DCPS, DDX1, DENR, DHFR, DHX36, EEF1G, EFL1, EIF2AK2, EIF2B1, EIF2D, EIF2S1, EIF3A, EIF3B, EIF3E, EIF3F, EIF4G3, EIF6, EPRS1, ERBB2, FMR1, FTO, GAPDH, GLE1, HNRNPR, IGF2BP1, IGF2BP2, ILF3, IMPACT, IREB2, LARP1, LARP4B, LARP6, LRPPRC, METTL14, MLH1, MOV10, NCK2, NCL, NIBAN1, NOCT, NPM1, NSUN5, NT5C3B, OGFOD1, PAIP1, PDK1, PELO, PIN1, PKM, PLD1, PLXNB2, PPP1CA, PPP3CA, PRKAA1, PRKCSH, PUS7, RBM4, RPL13A, RPL22, RPL38, RPL5, RPS9, RTN4, SARNP, SELENOT, SESN2, SHFL, SHMT2, SPHK2, THBS1, TIGAR, TRMT10C, TUT4, YBX1, ZFP36L2]	No change
nucleobase-containing compound catabolic process	GO_BiologicalProcess-EBI-UniProt-GOA-ACAP-ARAP_13.05.2021_00h00	2.75E-67	16.582064	98	[ACOT7, AGO2, APEX1, CASP3, CECR2, CIRBP, CNOT1, CNOT10, DCPS, DHX34, DHX36, DICER1, DUT, EIF3E, ENPP1, FEN1, FMR1, FTO, HINT1, HNRNPA0, HNRNPC, HNRNPR, IGF2BP1, IGF2BP2, KPNB1, LARP1, LARP4B, LRPPRC, METTL14, MGAT1, MLH1, MOV10, MRTO4, MTAP, MTREX, MYD88, NOCT, NPM1, NT5C, NT5C3B, NUDT15, NUDT16, NUDT16L1, PAIP1, PDE5A, PELO, PNP, POP1, PSMA3,	Downregulation

					PSMB10, PSMB2, PSMC2, PSMC3, PSMC6, PSMD1, PSMD12, PSMD14, PSMD2, PSMD6, PSMD7, PSMD8, PSME1, PSME2, PSME4, RNPS1, RPL10A, RPL13, RPL13A, RPL18A, RPL22, RPL24, RPL27, RPL32, RPL36, RPL38, RPL5, RPLP1, RPLP2, RPS13, RPS15A, RPS16, RPS18, RPS21, RPS23, RPS4Y1, RPS5, RPS8, RPS9, SAMHD1, SARM1, SET, SOAT1, SSB, TUT4, TYMP, YBX1, ZC3HAV1, ZFP36L2]	
cellular macromolecule catabolic process	GO_BiologicalProcess-EBI-UniProt-GOA-ACAP-ARAP_13.05.2021_00h00	2.75E-67	13.864542	174	[ADAMTS9, AGO2, ANKZF1, APEX1, ARAF, ARRB1, ATE1, AUP1, CASP3, CCDC22, CD81, CDC34, CDK1, CDK2, CDK5RAP3, CECR2, CIRBP, CLTC, CLU, CNOT1, CNOT10, CRBN, CTNNB1, CTSC, CUL1, CUL4B, DAB2, DCPS, DDI2, DERL1, DHX34, DHX36, DICER1, EIF3E, ERLIN2, EZR, FBXL12, FBXW8, FEN1, FMR1, FTO, GIPC1, GLMN, GPC1, GSK3B, HNRNPA0, HNRNPC, HNRNPR, HSP90AA1, HSP90B1, HSPA5, IGF2BP1, IGF2BP2, KPNB1, LARP1, LARP4B, LRPPRC, MAD2L1, MAEA, MAGI1, MALT1, METTL14, MLH1, MOV10, MRTO4, MSN, MTA1, MTREX, MYD88, NEDD4, NEDD8, NOCT, NOP53, NPLOC4, NPM1, NT5C3B, NUB1, NUDT15, NUDT16, NUDT16L1, PAIP1, PDCD6IP, PELO, PITHD1, PML, POP1, PRPF19, PSMA3, PSMB10, PSMB2, PSMC2, PSMC3, PSMC6, PSMD1, PSMD12, PSMD14, PSMD2, PSMD6, PSMD7, PSMD8, PSME1, PSME2, PSME4, RCN3, RDX, RNF114, RNF2, RNF213, RNF40, RNPS1, RPL10A, RPL13, RPL13A, RPL18A,	No change

					RPL22, RPL24, RPL27, RPL32, RPL36, RPL38, RPL5, RPLP1, RPLP2, RPS13, RPS15A, RPS16, RPS18, RPS21, RPS23, RPS4Y1, RPS5, RPS8, RPS9, RSPRY1, RYBP, SEC61B, SET, SGSH, SIRT1, SMARCC1, SNF8, SNX5, SQSTM1, SSB, SUFU, TPP1, TRIM25, TRIP12, TUT4, UBAC2, UBE2L6, UBR2, UBR4, UBXN2A, UFD1, USP11, USP22, USP28, USP4, USP48, USP5, USP7, USP9X, USP9Y, VLDLR, VPS25, VPS36, WFS1, WWP2, YBX1, YOD1, ZC3HAV1, ZFP36L2, ZMPSTE24]	
regulation of establishment of protein localization	GO_BiologicalProcess-EBI-UniProt-GOA-ACAP-ARAP_13.05.2021_00h00	2.75E-67	12.645591	76	[ABAT, ACSL3, ACSL4, ANK3, ARFIP1, ATP2C1, B3GAT3, C2CD2L, CCT2, CCT3, CCT6A, CD200, CD81, CDK1, CDK16, CEMIP, CHORDC1, CHP1, CLU, CYP51A1, DNM1L, DYNLL1, ENY2, ERBB2, EZR, FERMT1, GCC2, GLUD1, GLUL, HADH, HADHA, IDH2, IPO5, JAGN1, LLGL1, LRRC8A, MAGI1, MAP4K4, MFF, MYH10, MYO18A, MYO1E, NMT1, NUP62, NUTF2, P3H1, PARL, PCK2, PFKM, PHPT1, PIN1, PPP3CA, PRKAA1, RAB23, RAB8A, RANGAP1, RBM22, RNF2, SAE1, SCFD1, SLC12A2, SLC1A1, SLC2A1, SRC, STOM, SUFU, TCAF1, TCP1, TM9SF4, TOMM7, TRIM28, TXN, UBAC2, WWP2, XPO4, YOD1]	Upregulation
protein localization to membrane	GO_BiologicalProcess-EBI-UniProt-GOA-ACAP-ARAP_13.05.2021_00h00	2.75E-67	14.46281	105	[ACSL3, ACTB, ADAM10, AFDN, AGRN, AKR1B1, ALDH3A2, ANK3, APP, ATP1B3, ATP2C1, CD81, CEMIP, CHORDC1, CHP1, CLTC, DAB2, EHD3, EMC1, EPB41L3, EPHA2, EPHA3, ERBB2, EZR, FERMT2, GBP1, GCC2, GDAP1, GET3, GGA2, GOLGA7, GPC1, GPC6, GPHN,	No change

					HGS, HMOX1, HSP90AA1, HSPA4, HSPA5, IKBKB, ITGA3, ITGB1, KRT18, LAMA5, LIN7C, LLGL1, MACF1, MFF, MICALL1, MYADM, MYO1E, NDUFA13, NMT1, OXA1L, PACS1, PDZD11, PPP2R5A, PRKCI, RAB10, RAB11B, RAB3B, RAB3D, RAB8A, RABGEF1, RDX, REEP2, RPL10A, RPL13, RPL13A, RPL18A, RPL22, RPL24, RPL27, RPL32, RPL36, RPL38, RPL5, RPLP1, RPLP2, RPS13, RPS15A, RPS16, RPS18, RPS21, RPS23, RPS4Y1, RPS5, RPS8, RPS9, S100A10, SCRIB, SEC61A1, SEC61B, SLC1A1, SQSTM1, SRPRA, SSR3, STOM, TCAF1, TM9SF4, TNIK, TPBG, TRAM1, TRAM2, ZDHHC21]	
regulation of cellular protein localization	GO_BiologicalProcess-EBI-UniProt-GOA-ACAP-ARAP_13.05.2021_00h00	2.75E-67	14.236707	83	[ACSL3, ACTB, ADAM10, AKR1B1, ANK3, ANKRD13A, ATP2C1, B3GAT3, CCT2, CCT3, CCT6A, CD81, CDK1, CDK5RAP3, CEMIP, CHORDC1, CHP1, CLTC, CTNNB1, DAB2, EPB41L2, EPHA2, EPHA3, ERBB2, EZR, FERMT1, FERMT2, GBP1, GCC2, GLUL, GNL3L, GPC1, GPC6, GPD1L, GSK3B, HSP90AA1, IPO5, ITGA3, ITGB1, KAT7, MACROH2A1, MAGI1, MEAK7, MFF, MSN, MYO1E, NF2, NMD3, NMT1, NPM1, NUP62, NUTF2, PARL, PARP1, PIN1, PPP2R5A, PRKAA1, PRKCI, PTGES2, RAB11B, RAB23, RANGAP1, RBM22, RDX, RNF2, RTN4, SAE1, SESN2, SLC1A1, SQSTM1, SRC, STOM, SUFU, TCAF1, TCP1, TM9SF4, TOMM7, TRIM28, TXN, UBAC2, XPO4, YAP1, YOD1]	Upregulation
positive regulation of cellular protein localization	GO_BiologicalProcess-EBI-UniProt-GOA-ACAP-	2.75E-67	17.142857	54	[ACSL3, ANK3, ATP2C1, CCT2, CCT3, CCT6A, CD81, CDK1, CDK5RAP3, CEMIP,	Upregulation

	ARAP_13.05.2021_00h00				CHORDC1, CHP1, EPB41L2, EPHA2, EPHA3, ERBB2, EZR, FERMT2, GNL3L, GPD1L, GSK3B, IPO5, ITGA3, ITGB1, KAT7, MAGI1, MEAK7, MFF, MSN, MYO1E, NMD3, NMT1, NPM1, NUP62, NUTF2, PARP1, PIN1, PRKAA1, PRKCI, RBM22, RDX, RNF2, RTN4, SAE1, SESN2, SQSTM1, SRC, STOM, TCAF1, TCP1, TM9SF4, TOMM7, TRIM28, YAP1]	
neutrophil mediated immunity	GO_BiologicalProcess-EBI-UniProt-GOA-ACAP-ARAP_13.05.2021_00h00	2.75E-67	14.928058	83	[ACTR2, ADAM10, AGPAT2, ALDOA, ANO6, ANXA3, APAF1, APRT, ASAH1, ATG7, ATP6V0A1, ATP6V0C, B2M, B4GALT1, BST1, CAP1, CCT2, CHAMP1, CLU, CSTB, CTSA, CTSC, CYB5R3, DIAPH1, DSP, DYNC1LI1, DYNLL1, ERP44, FTH1, FTL, GLA, GOLGA7, GPI, GSDMD, GSTP1, HEXB, HLA-B, HLA-C, HSP90AA1, IST1, ITGAV, ITM2C, JAGN1, KPNB1, LAMP1, MGST1, MME, MTCH1, MVP, MYD88, PAFAH1B2, PDAP1, PKM, PLD1, PNP, PPIE, PRDX6, PRKCSH, PSMC2, PSMC3, PSMD1, PSMD12, PSMD14, PSMD2, PSMD6, PSMD7, PTGES2, PYCARD, PYGB, QSOX1, RAB10, RAB3D, RAP2B, RAP2C, SERPINB1, SLC44A2, STOM, STXBP3, TOM1, TUBB, UBR4, VAT1, WDR1]	Downregulation
mRNA binding	GO_MolecularFunction-EBI-UniProt-GOA-ACAP-ARAP_13.05.2021_00h00	2.75E-67	18.971062	59	[ACO1, ADARB1, AGO2, AQR, C1QBP, CELF2, CIRBP, CRYZ, CSTF3, DENR, DHFR, DHX36, EIF2S1, EIF3A, EIF3E, EIF4G3, EIF6, FMR1, HNRNPA0, HNRNPA3, HNRNPC, HNRNPLL, HNRNPR, IGF2BP1, IGF2BP2, ILF3, IREB2, LARP1, LARP4B, LARP6, METTL14, MYH10, NCL, NOCT, NUDT16, PCBP3, PKM, PPIE, PTBP2, PTBP3, RBM4, RBMS2, RNF40, RNPS1,	Upregulation

					RPL13A, RPL5, RPS13, RPS5, SF1, SF3B6, SHFL, SHMT2, SRSF5, SSB, THOC2, TRA2B, YBX1, YTHDC1, ZFP36L2]	
spliceosomal complex	GO_CellularComponent-EBI-UniProt-GOA-ACAP-ARAP_13.05.2021_00h00	2.75E-67	18.781727	37	[AAR2, AQR, BCAS2, CDC5L, CIRBP, CTNNBL1, CWC22, GCFC2, GPKOW, HNRNPA3, HNRNPC, HNRNPH1, HNRNPR, HTATSF1, IVNS1ABP, MTREX, NCL, PPIE, PPWD1, PRPF19, RBM22, RHEB, SF1, SF3A3, SF3B6, SMNDC1, SMU1, SNRNP200, SNRPA, SNRPD3, SNRPE, SNU13, SNW1, SRSF2, TRA2A, TRA2B, YBX1]	Upregulation
ribosome	GO_CellularComponent-EBI-UniProt-GOA-ACAP-ARAP_13.05.2021_00h00	2.75E-67	15.6	39	[APEX1, CANX, EIF2AK2, EIF2D, EIF2S1, FMR1, HSPA14, LARP1, LARP4B, MRPL10, MRPS17, MRPS21, MRPS31, NPM1, RPL10A, RPL13, RPL13A, RPL18A, RPL22, RPL24, RPL27, RPL32, RPL36, RPL38, RPL5, RPLP1, RPLP2, RPS13, RPS15A, RPS16, RPS18, RPS21, RPS23, RPS4Y1, RPS5, RPS8, RPS9, SF1, TBCE]	Downregulation
intracellular protein transport	GO_BiologicalProcess-EBI-UniProt-GOA-ACAP-ARAP_13.05.2021_00h00	2.75E-67	14.700193	152	[ALDH3A2, ANK3, AP3M2, APEX1, APLP2, APPL2, ARFIP1, ASPSCR1, AUP1, B3GAT3, CD81, CDK1, CEMIP, CHORDC1, CHP1, CLTC, CLTCL1, CLU, COG7, CTSA, DERL1, EIF2D, ERBB2, FAM91A1, FERMT1, GCC2, GDAP1, GET3, GGA2, GIPC1, GOLGA7, HACL1, HEATR3, HERC2, HGS, HSP90AA1, HSP90B1, HSPA4, HSPA5, IPO5, IPO9, KPNB1, M6PR, MAGI1, MFF, MICALL1, MYO1E, MYO6, NDUFA13, NEDD4, NMT1, NPLOC4, NPM1, NUP35, NUP62, NUTF2, OXA1L, PARL, PIN1, PITRM1, PML, PPP3CA, PRKAA1, PRKCI, RAB1A, RAB23, RAB5A, RAB8A, RABGEF1, RANGAP1, RBM22, REEP2, RHOB, RNF2,	No change

					RPL10A, RPL13, RPL13A, RPL18A, RPL22, RPL24, RPL27, RPL32, RPL36, RPL38, RPL5, RPLP1, RPLP2, RPS13, RPS15A, RPS16, RPS18, RPS21, RPS23, RPS4Y1, RPS5, RPS8, RPS9, SAE1, SCARB2, SCFD1, SCRIB, SEC23B, SEC61A1, SEC61B, SEPTIN9, SLC1A1, SLC25A6, SNF8, SNX1, SNX27, SNX5, SORT1, SRPRA, SSR3, STOM, STXBP3, SUFU, TBC1D10A, TBC1D17, TBC1D22B, TBC1D4, TBC1D8B, TCAF1, TIMM17B, TM9SF4, TMED9, TNPO2, TOM1, TOM1L2, TOMM34, TOMM7, TRAM1, TRAM2, TRIM28, TSC2, TSPO, TXN, UBAC2, UFD1, UNC93B1, VPS25, VPS26B, VPS33B, VPS36, VPS41, WDR11, XPO4, XPO6, XPOT, YOD1, ZDHHC21, ZFAND6]	
positive regulation of cellular catabolic process	GO_BiologicalProcess-EBI-UniProt-GOA-ACAP-ARAP_13.05.2021_00h00	2.75E-67	13.071896	60	[AGO2, APP, ATG7, CCDC22, CD81, CDK5RAP3, CLU, CNOT1, CNOT10, CTSC, DAB2, DAGLB, DAPK1, DCN, DCPS, DHX36, EZR, FBXW8, FMR1, FTO, GPSM1, GSK3B, HMOX1, HNRNPR, HSP90AA1, IKBKG, LARP1, METTL14, MLH1, MOV10, MSN, NNT, NOCT, NOP53, NT5C3B, NUB1, PACSIN3, PAFAH1B2, PIK3C2A, PIP4K2B, PRKAA1, PSMC2, PSMC3, PSMC6, RDX, RIPK2, RNF40, SESN2, SH3BP4, SIRT1, SLC25A4, SLC25A5, TBK1, TRIM21, TSC2, TUT4, USP5, VDAC1, ZC3HAV1, ZFP36L2]	Upregulation
protein localization to organelle	GO_BiologicalProcess-EBI-UniProt-GOA-ACAP-ARAP_13.05.2021_00h00	2.75E-67	15.893386	161	[ADAM10, ALDH3A2, ANKRD13A, APLP2, APP, APPL2, AUP1, BOD1, CCT2, CCT3, CCT6A, CD81, CDK1, CDK5RAP3, CHAMP1, CHORDC1, CHP1, CLU, CNGB1, COG7, DDX1, DIAPH1, DNM1L, EMC1, EZR, FERMT1, FERMT2, GBP1, GCC2, GDAP1, GET3,	No change

					GLMP, GLUL, GNL3L, GPC6, GSK3B, H1-5, HACL1, HEATR3, HGS, HK1, HK2, HMOX1, HSP90AA1, HSPA4, HSPA5, IFT140, IPO5, IPO9, KAT7, KPNB1, LAMP1, LEMD2, LLGL1, LRWD1, M6PR, MACROH2A1, MAGI1, MARCHF5, MEAK7, MFF, MICALL1, MMAB, MSH2, MSN, NDUFA13, NEDD1, NEDD4, NF2, NMD3, NMT1, NOP53, NPM1, NUP35, NUP62, NUTF2, OBSL1, OSBPL8, OXA1L, PACS1, PARL, PARP1, PIN1, PITRM1, PML, PPP3CA, PRKAA1, PTGES2, RAB10, RAB23, RAD21, RALA, RANGAP1, RBM22, RDX, RNF2, RPA1, RPA2, RPF2, RPL10A, RPL13, RPL13A, RPL18A, RPL22, RPL24, RPL27, RPL32, RPL36, RPL38, RPL5, RPLP1, RPLP2, RPS13, RPS15A, RPS16, RPS18, RPS21, RPS23, RPS4Y1, RPS5, RPS8, RPS9, RTN4, SAE1, SCARB2, SCRIB, SEC61A1, SEC61B, SESN2, SH3BP4, SLC25A6, SNF8, SORT1, SRC, SRPRA, SSB, SSR3, SUFU, TCP1, TIMM17B, TNPO2, TOMM34, TOMM7, TOR1AIP1, TOR1AIP2, TOR1B, TPP1, TRAM1, TRAM2, TRIM28, TSC2, TSPO, TXN, VPS25, VPS36, VPS41, WDR35, WRAP73, YAP1, YBX1, ZFAND6]	
positive regulation of cellular amide metabolic process	GO_BiologicalProcess-EBI-UniProt-GOA-ACAP-ARAP_13.05.2021_00h00	2.75E-67	17.241379	30	[AGO2, C1QBP, CASP3, CIRBP, CLU, DHX36, EIF3E, EIF6, ERBB2, FMR1, IMPACT, LARP1, LARP4B, LARP6, METTL14, NCK2, NIBAN1, NPM1, NSUN5, PAIP1, PKM, PLD1, PLXNB2, PPP3CA, PRKCSH, RPL5, RPS9, SPHK2, THBS1, TRMT10C]	No change
ribosome biogenesis	GO_BiologicalProcess-EBI-UniProt-GOA-ACAP-	2.75E-67	16.949152	60	[ABCE1, ABT1, BMS1, C1QBP, CHD7, CNGB1, CUL4B, DDX47,	No change

	ARAP_13.05.2021_00h00				DIMT1, EFL1, EIF2S1, EIF6, EMG1, GEMIN4, GLUL, GNL3L, HEATR3, MRM3, MRPL10, MRTO4, MTREX, NEDD4, NLE1, NMD3, NOL11, NOM1, NOP53, NPM1, NSUN5, NUDT16, RIOK3, RPF2, RPL10A, RPL13, RPL13A, RPL18A, RPL22, RPL24, RPL27, RPL32, RPL36, RPL38, RPL5, RPLP1, RPLP2, RPS16, RPS21, RPS5, RPS8, RPS9, SBDS, SENP3, SNU13, TSR2, URB2, UTP14A, UTP20, UTP3, WDR12, YBEY]	
antigen processing and presentation of exogenous peptide antigen via MHC class I	GO_BiologicalProcess-EBI-UniProt-GOA-ACAP-ARAP_13.05.2021_00h00	2.75E-67	31.764706	27	[B2M, HLA-A, HLA-B, HLA-C, IKBKB, IKBKG, ITGAV, ITGB5, LNPEP, PSMA3, PSMB10, PSMB2, PSMC2, PSMC3, PSMC6, PSMD1, PSMD12, PSMD14, PSMD2, PSMD6, PSMD7, PSMD8, PSME1, PSME2, PSME4, SEC14L2, TAP1]	Downregulation
amide biosynthetic process	GO_BiologicalProcess-EBI-UniProt-GOA-ACAP-ARAP_13.05.2021_00h00	2.75E-67	15.6351795	144	[AARS1, ABCE1, ACACA, ACO1, ACSL3, ACSL4, ACSS2, ADSL, AGO2, APP, ASAH1, ASNS, B4GALT5, BDH2, C1QBP, CERS6, CIRBP, CNOT1, CNOT10, DAPK1, DARS1, DCPS, DDX1, DENR, DHFR, DHPS, DHX36, DRG1, EARS2, EEF1E1, EEF1G, EFL1, EIF2AK2, EIF2B1, EIF2D, EIF2S1, EIF3A, EIF3B, EIF3E, EIF3F, EIF4G3, EIF6, EPRS1, ERBB2, FAM3A, FASN, FMR1, FTO, GAPDH, GARS1, GATB, GCDH, GGCT, GLE1, HARS1, HNRNPR, IARS2, IGF2BP1, IGF2BP2, ILF3, IMPACT, IREB2, LARP1, LARP4B, LARP6, LRPPRC, METTL14, MLH1, MOV10, MRPL10, MRPS17, MRPS21, MRPS31, MRRF, N6AMT1, NCK2, NCL, NDRG1, NIBAN1, NOCT, NPM1, NSUN5, NT5C3B, OGFOD1, OPLAH, OXA1L, PAIP1, PDK1, PELO,	No change

					PKM, PLD1, PLXNB2, PPP1CA, PPP3CA, PRKAA1, PRKCSH, PUS7, QARS1, RARS2, RBM4, RPL10A, RPL13, RPL13A, RPL18A, RPL22, RPL24, RPL27, RPL32, RPL36, RPL38, RPL5, RPLP1, RPLP2, RPS13, RPS15A, RPS16, RPS18, RPS21, RPS23, RPS4Y1, RPS5, RPS8, RPS9, SARNP, SARS1, SELENOT, SESN2, SHFL, SHMT2, SLC1A1, SLC25A15, SMPD1, SOAT1, SPHK2, SPTLC1, SPTLC2, TBCE, THBS1, TRMT10C, TUT4, VARS1, YARS1, YBX1, ZFP36L2]	
regulation of protein transport	GO_BiologicalProcess-EBI-UniProt-GOA-ACAP-ARAP_13.05.2021_00h00	2.75E-67	12.456747	72	[ABAT, ACSL3, ACSL4, ANK3, ARFIP1, ATP2C1, B3GAT3, C2CD2L, CD200, CD81, CDK1, CDK16, CEMIP, CHORDC1, CHP1, CLU, CYP51A1, DNM1L, DYNLL1, ENY2, ERBB2, EZR, FERMT1, GCC2, GLUD1, GLUL, HADH, HADHA, IDH2, IPO5, JAGN1, LLGL1, LRRC8A, MAGI1, MAP4K4, MFF, MYH10, MYO18A, MYO1E, NMT1, NUP62, NUTF2, P3H1, PARL, PCK2, PFKM, PHPT1, PIN1, PPP3CA, PRKAA1, RAB23, RAB8A, RANGAP1, RBM22, RNF2, SAE1, SCFD1, SLC12A2, SLC1A1, SLC2A1, SRC, STOM, SUFU, TCAF1, TM9SF4, TOMM7, TRIM28, TXN, UBAC2, WWP2, XPO4, YOD1]	Upregulation
catalytic step 2 spliceosome	GO_CellularComponent-EBI-UniProt-GOA-ACAP-ARAP_13.05.2021_00h00	2.75E-67	21.59091	19	[AQR, BCAS2, CDC5L, CWC22, HNRNPA3, HNRNPC, HNRNPH1, HNRNPR, MTREX, PPIE, PPWD1, PRPF19, RBM22, SF3A3, SF3B6, SNRNP200, SNRPD3, SNRPE, SNW1]	Upregulation
protein-DNA complex subunit organization	GO_BiologicalProcess-EBI-UniProt-GOA-ACAP-ARAP_13.05.2021_00h00	2.75E-67	15.320334	55	[CCNH, CENPV, CETN2, CHAF1B, CHD1L, CUL4B, ERCC5, GRWD1, GTF2E1, H1-10, H1-5, HAT1, MACROH2A1, MCM2, MCM3, MCM4, MCM5,	Upregulation

					MCM6, MCM7, NPM1, ORC4, ORC5, PARP1, POGZ, PSMA3, PSMB10, PSMB2, PSMC2, PSMC3, PSMC6, PSMD1, PSMD12, PSMD14, PSMD2, PSMD6, PSMD7, PSMD8, PSME1, PSME2, PSME4, RBBP4, RPA1, RPA2, SET, SHPRH, SLC25A15, SMARCB1, SMARCC1, SMARCD3, SMARCE1, SMYD3, SUPT16H, TSPYL1, UBTF, ZFP36L2]	
establishment of protein localization to organelle	GO_BiologicalProcess-EBI-UniProt-GOA-ACAP-ARAP_13.05.2021_00h00	2.75E-67	17.72152	98	[ALDH3A2, APLP2, APPL2, CCT2, CCT3, CCT6A, CDK1, CHORDC1, CHP1, CLU, FERMT1, GCC2, GDAP1, GET3, H1-5, HACL1, HEATR3, HGS, HK1, HK2, HSP90AA1, HSPA4, HSPA5, IPO5, IPO9, KPNB1, LAMP1, LRWD1, M6PR, MACROH2A1, MAGI1, MFF, NDUFA13, NEDD4, NMT1, NUP35, NUP62, NUTF2, OXA1L, PARL, PIN1, PITRM1, PML, PPP3CA, PRKAA1, RAB10, RAB23, RALA, RBM22, RNF2, RPL10A, RPL13, RPL13A, RPL18A, RPL22, RPL24, RPL27, RPL32, RPL36, RPL38, RPL5, RPLP1, RPLP2, RPS13, RPS15A, RPS16, RPS18, RPS21, RPS23, RPS4Y1, RPS5, RPS8, RPS9, SAE1, SCARB2, SCRIB, SEC61A1, SEC61B, SLC25A6, SNF8, SORT1, SRPRA, SSR3, SUFU, TCP1, TIMM17B, TNPO2, TOMM34, TOMM7, TRAM1, TRAM2, TRIM28, TSC2, TSPO, VPS25, VPS36, VPS41, ZFAND6]	No change
establishment of protein localization to membrane	GO_BiologicalProcess-EBI-UniProt-GOA-ACAP-ARAP_13.05.2021_00h00	2.75E-67	16.87345	68	[ACSL3, AFDN, ALDH3A2, ANK3, APP, ATP2C1, CEMIP, CHORDC1, CHP1, EMC1, ERBB2, GCC2, GDAP1, GET3, GGA2, GOLGA7, HMOX1, HSP90AA1, HSPA4, HSPA5, KRT18, MACF1, MFF, MICALL1, MYO1E, NDUFA13, NMT1,	Downregulation

					OXA1L, PRKCI, RAB10, RAB11B, RAB8A, RABGEF1, REEP2, RPL10A, RPL13, RPL13A, RPL18A, RPL22, RPL24, RPL27, RPL32, RPL36, RPL38, RPL5, RPLP1, RPLP2, RPS13, RPS15A, RPS16, RPS18, RPS21, RPS23, RPS4Y1, RPS5, RPS8, RPS9, SCRIB, SEC61A1, SEC61B, SLC1A1, SRPRA, SSR3, STOM, TCAF1, TRAM1, TRAM2, ZDHHC21]	
secretory vesicle	GO_CellularComponent-EBI-UniProt-GOA-ACAP-ARAP_13.05.2021_00h00	2.75E-67	11.881188	132	[ACTR2, ADAM10, AGPAT2, ALB, ALDOA, ANO6, ANXA3, ANXA7, APAF1, APEX1, APLP2, APP, APRT, ASAH1, ATG7, ATP2B1, ATP6V0A1, ATP6V0C, ATP6V1A, B2M, B4GALT1, BAIAP2, BIN1, BST1, CAP1, CCT2, CD109, CDK16, CHAMP1, CLU, CSTB, CTSA, CTSC, CYB5R3, CYRIB, DIAPH1, DNM1L, DPYSL3, DSP, DYNC1LI1, DYNLL1, ECE1, ERP44, FAM3A, FN1, FTH1, FTL, GARS1, GIPC1, GLA, GOLGA1, GOLGA7, GPI, GSDMD, GSTP1, HEXB, HLA-B, HLA-C, HSP90AA1, IST1, ITGAV, ITIH4, ITM2C, KPNB1, LAMP1, LGALS3BP, LLGL1, MAGED2, MFF, MGST1, MME, MTCH1, MVP, MYH9, NHLRC2, PAFAH1B2, PCDH7, PDAP1, PKM, PLD1, PNP, PPIE, PRDX6, PRKCSH, PSMC2, PSMC3, PSMD1, PSMD12, PSMD14, PSMD2, PSMD6, PSMD7, PTGES2, PYCARD, PYGB, QSOX1, RAB10, RAB11B, RAB3B, RAB3D, RAB5A, RAB8A, RAP2B, RAP2C, SEPTIN2, SEPTIN8, SERPINB1, SERPINE2, SLC17A5, SLC44A2, SMPD1, SOD1, SPARC, STOM, STON1, STXBP3, SYPL2, TCP1, TF, THBS1, TMED9, TMX3, TOM1, TPRG1L, TRAPPC4,	Downregulation

					TUBB, UBR4, VAT1, VDAC1, VDAC2, VPS33B, WFS1]	
morphogenesis of a polarized epithelium	GO_BiologicalProcess-EBI-UniProt-GOA-ACAP-ARAP_13.05.2021_00h00	2.75E-67	19.512196	32	[ACTB, ACTG1, CLTC, DAB2, DVL2, EZR, FAT1, GPC6, LAMA5, MSN, PAFAH1B1, PSMA3, PSMB10, PSMB2, PSMC2, PSMC3, PSMC6, PSMD1, PSMD12, PSMD14, PSMD2, PSMD6, PSMD7, PSMD8, PSME1, PSME2, PSME4, PTK7, RAB10, ROR1, SCRIB, WDR1]	Downregulation
posttranscriptional regulation of gene expression	GO_BiologicalProcess-EBI-UniProt-GOA-ACAP-ARAP_13.05.2021_00h00	2.75E-67	15.531335	114	[AARS1, ABCE1, ACO1, AGO2, APEX1, APP, C1QBP, CIRBP, CNOT1, CNOT10, COLEC12, DAPK1, DCPS, DDX1, DENR, DHFR, DHX36, DICER1, EEF1G, EFL1, EIF2AK2, EIF2B1, EIF2D, EIF2S1, EIF3A, EIF3B, EIF3E, EIF3F, EIF4G3, EIF6, EPRS1, ERBB2, FMR1, FTO, GAPDH, GARS1, GLE1, HELZ, HNRNPA0, HNRNPC, HNRNPR, IGF2BP1, IGF2BP2, ILF3, IMPACT, IREB2, LARP1, LARP4B, LARP6, LRPPRC, MATR3, METTL14, MLH1, MOV10, MYD88, NCK2, NCL, NIBAN1, NOCT, NPM1, NSUN5, NT5C3B, NUP205, NUP35, NUP62, OGFOD1, PAIP1, PELO, PKM, PLD1, PLXNB2, POLR2A, POLR2B, POLR2H, POLR2I, PPP1CA, PPP3CA, PRKCSH, PSMA3, PSMB10, PSMB2, PSMC2, PSMC3, PSMC6, PSMD1, PSMD12, PSMD14, PSMD2, PSMD6, PSMD7, PSMD8, PSME1, PSME2, PSME4, PUS7, RBM4, RPL13A, RPL22, RPL38, RPL5, RPS9, SARNP, SELENOT, SESN2, SET, SHFL, SHMT2, THBS1, TRMT10C, TUT4, YBX1, YTHDC1, ZFP36L2, ZMPSTE24]	No change
secretory granule	GO_CellularComponent-EBI-UniProt-GOA-ACAP-ARAP_13.05.2021_00h00	2.75E-67	11.689692	110	[ACTR2, ADAM10, AGPAT2, ALB, ALDOA, ANO6, ANXA3, ANXA7, APAF1, APLP2, APP, APRT, ASAH1, ATG7, ATP6V0A1,	Downregulation

					ATP6V0C, ATP6V1A, B2M, B4GALT1, BAIAP2, BST1, CAP1, CCT2, CD109, CHAMP1, CLU, CSTB, CTSA, CTSC, CYB5R3, CYRIB, DIAPH1, DSP, DYNC1LI1, DYNLL1, ECE1, ERP44, FAM3A, FN1, FTH1, FTL, GARS1, GLA, GOLGA1, GOLGA7, GPI, GSDMD, GSTP1, HEXB, HLA-B, HLA-C, HSP90AA1, IST1, ITGAV, ITIH4, ITM2C, KPNB1, LAMP1, LGALS3BP, MAGED2, MGST1, MME, MTCH1, MVP, MYH9, NHLRC2, PAFAH1B2, PCDH7, PDAP1, PKM, PLD1, PNP, PPIE, PRDX6, PRKCSH, PSMC2, PSMC3, PSMD1, PSMD12, PSMD14, PSMD2, PSMD6, PSMD7, PTGES2, PYCARD, PYGB, QSOX1, RAB10, RAB3D, RAP2B, RAP2C, SERPINB1, SERPINE2, SLC44A2, SMPD1, SOD1, SPARC, STOM, STXBP3, TCP1, TF, THBS1, TMX3, TOM1, TUBB, UBR4, VAT1, VDAC2, VPS33B, WFS1]	
peptide biosynthetic process	GO_BiologicalProcess-EBI-UniProt-GOA-ACAP-ARAP_13.05.2021_00h00	2.75E-67	16.057442	123	[AARS1, ABCE1, ACO1, AGO2, APP, BDH2, C1QBP, CIRBP, CNOT1, CNOT10, DAPK1, DARS1, DCPS, DDX1, DENR, DHFR, DHPS, DHX36, DRG1, EARS2, EEF1E1, EEF1G, EFL1, EIF2AK2, EIF2B1, EIF2D, EIF2S1, EIF3A, EIF3B, EIF3E, EIF3F, EIF4G3, EIF6, EPRS1, ERBB2, FMR1, FTO, GAPDH, GARS1, GATB, GGCT, GLE1, HARS1, HNRNPR, IARS2, IGF2BP1, IGF2BP2, ILF3, IMPACT, IREB2, LARP1, LARP4B, LARP6, LRPPRC, METTL14, MLH1, MOV10, MRPL10, MRPS17, MRPS21, MRPS31, MRRF, N6AMT1, NCK2, NCL, NDRG1, NIBAN1, NOCT, NPM1, NSUN5, NT5C3B, OGFOD1, OPLAH, OXA1L, PAIP1, PELO, PKM,	No change

					PLD1, PLXNB2, PPP1CA, PRKCSH, PUS7, QARS1, RARS2, RBM4, RPL10A, RPL13, RPL13A, RPL18A, RPL22, RPL24, RPL27, RPL32, RPL36, RPL38, RPL5, RPLP1, RPLP2, RPS13, RPS15A, RPS16, RPS18, RPS21, RPS23, RPS4Y1, RPS5, RPS8, RPS9, SARNP, SARS1, SELENOT, SESN2, SHFL, SHMT2, SLC1A1, TBCE, THBS1, TRMT10C, TUT4, VARS1, YARS1, YBX1, ZFP36L2]	
leukocyte degranulation	GO_BiologicalProcess-EBI-UniProt-GOA-ACAP-ARAP_13.05.2021_00h00	2.75E-67	13.758389	82	[ACTR2, ADAM10, AGPAT2, ALDOA, ANO6, ANXA3, APAF1, APRT, ASAH1, ATG7, ATP6V0A1, ATP6V0C, B2M, B4GALT1, BST1, CAP1, CCT2, CHAMP1, CLU, CSTB, CTSA, CTSC, CYB5R3, DIAPH1, DSP, DYNC1LI1, DYNLL1, ERP44, FTH1, FTL, GLA, GOLGA7, GPI, GSDMD, GSTP1, HEXB, HLA-B, HLA-C, HMOX1, HSP90AA1, IST1, ITGAV, ITM2C, KPNB1, LAMP1, MGST1, MME, MTCH1, MVP, PAFAH1B2, PDAP1, PKM, PLD1, PNP, PPIE, PRDX6, PRKCSH, PSMC2, PSMC3, PSMD1, PSMD12, PSMD14, PSMD2, PSMD6, PSMD7, PTGES2, PYCARD, PYGB, QSOX1, RAB10, RAB3D, RAP2B, RAP2C, SERPINB1, SLC44A2, SPHK2, STOM, STXBP3, TOM1, TUBB, UBR4, VAT1]	Downregulation
modification-dependent macromolecule catabolic process	GO_BiologicalProcess-EBI-UniProt-GOA-ACAP-ARAP_13.05.2021_00h00	2.75E-67	12.390671	85	[ANKZF1, ARAF, ARRB1, ATE1, AUP1, CCDC22, CDC34, CDK1, CDK2, CDK5RAP3, CLU, CRBN, CTNNB1, CUL1, CUL4B, DAB2, DERL1, ERLIN2, FBXL12, FBXW8, GIPC1, GSK3B, HSP90B1, HSPA5, MAD2L1, MAEA, MAGI1, MTA1, NEDD4, NEDD8, NOP53, NPLOC4, NUB1, PML, PSMA3,	No change

					PSMB10, PSMB2, PSMC2, PSMC3, PSMC6, PSMD1, PSMD12, PSMD14, PSMD2, PSMD6, PSMD7, PSMD8, PSME1, PSME2, PSME4, RNF114, RNF2, RNF213, RNF40, RPL5, RYBP, SEC61B, SIRT1, SMARCC1, SNF8, SQSTM1, SUFU, TRIM25, TRIP12, TUT4, UBE2L6, UBR2, UBR4, UBXN2A, UFD1, USP11, USP22, USP28, USP4, USP48, USP5, USP7, USP9X, USP9Y, VPS25, VPS36, WFS1, WWP2, YOD1, ZMPSTE24]	
cellular protein catabolic process	GO_BiologicalProcess-EBI-UniProt-GOA-ACAP-ARAP_13.05.2021_00h00	2.75E-67	12.605042	105	[ANKZF1, ARAF, ARRB1, ATE1, AUP1, CCDC22, CD81, CDC34, CDK1, CDK2, CDK5RAP3, CLTC, CLU, CRBN, CTNNB1, CTSC, CUL1, CUL4B, DAB2, DDI2, DERL1, ERLIN2, EZR, FBXL12, FBXW8, FMR1, GIPC1, GLMN, GSK3B, HSP90AA1, HSP90B1, HSPA5, MAD2L1, MAEA, MAGI1, MALT1, MSN, MTA1, NEDD4, NEDD8, NOP53, NPLOC4, NUB1, NUDT15, PDCD6IP, PITHD1, PML, PRPF19, PSMA3, PSMB10, PSMB2, PSMC2, PSMC3, PSMC6, PSMD1, PSMD12, PSMD14, PSMD2, PSMD6, PSMD7, PSMD8, PSME1, PSME2, PSME4, RCN3, RDX, RNF114, RNF2, RNF213, RNF40, RPL5, RSPRY1, RYBP, SEC61B, SIRT1, SMARCC1, SNF8, SNX5, SQSTM1, SUFU, TPP1, TRIM25, TRIP12, UBAC2, UBE2L6, UBR2, UBR4, UBXN2A, UFD1, USP11, USP22, USP28, USP4, USP48, USP5, USP7, USP9X, USP9Y, VLDLR, VPS25, VPS36, WFS1, WWP2, YOD1, ZMPSTE24]	No change
regulated exocytosis	GO_BiologicalProcess-EBI-UniProt-GOA-	2.75E-67	13.394919	116	[ACTR2, ADAM10, AGPAT2, ALB, ALDOA, ANO6,	Downregulation

	ACAP-ARAP_13.05.2021_00h00				ANXA3, ANXA5, APAF1, APLP2, APP, APRT, ARHGAP17, ASAH1, ATG7, ATP6V0A1, ATP6V0C, B2M, B4GALT1, BST1, CAP1, CASK, CCT2, CD109, CHAMP1, CHID1, CLU, CSTB, CTSA, CTSC, CYB5R3, CYRIB, DIAPH1, DNM1L, DSP, DYNC1LI1, DYNLL1, ERP44, FMR1, FN1, FTH1, FTL, GIPC1, GLA, GOLGA7, GPI, GSDMD, GSK3B, GSTP1, HEXB, HLA-B, HLA-C, HMOX1, HSP90AA1, IST1, ITGAV, ITIH4, ITM2C, KPNB1, LAMP1, LGALS3BP, MAGED2, MGST1, MME, MTCH1, MVP, MYH9, NHLRC2, PAFAH1B2, PCDH7, PDAP1, PKM, PLD1, PNP, PPIE, PRDX6, PRKCSH, PSMC2, PSMC3, PSMD1, PSMD12, PSMD14, PSMD2, PSMD6, PSMD7, PTGES2, PYCARD, PYGB, QSOX1, RAB10, RAB11B, RAB3D, RAB5A, RAP2B, RAP2C, SCRIB, SEPTIN9, SERPINB1, SLC44A2, SOD1, SPARC, SPHK2, STOM, STXBP3, SYNJ1, TAGLN2, TF, THBS1, TMX3, TOM1, TUBA4A, TUBB, UBR4, VAT1, VPS41, WDR1]	
regulation of innate immune response	GO_BiologicalProcess-EBI-UniProt-GOA-ACAP-ARAP_13.05.2021_00h00	2.75E-67	15.269461	51	[ABCE1, APLP2, APPL2, CUL1, ERAP1, FAM3A, HLA-A, HLA-B, HPX, HSP90AA1, IGF2BP1, IKBKB, IKBKG, LAMP1, MALT1, MATR3, NECTIN2, PARP14, PKN2, POLR3C, PSMA3, PSMB10, PSMB2, PSMC2, PSMC3, PSMC6, PSMD1, PSMD12, PSMD14, PSMD2, PSMD6, PSMD7, PSMD8, PSME1, PSME2, PSME4, PSPC1, PYCARD, RIOK3, SAMHD1, SERPINB9, SRC, STAT1, STAT2, STAT5B, TAB2, TBK1, TRIM21, TTLL12, XIAP, ZCCHC3]	No change

neutrophil activation involved in immune response	GO_BiologicalProcess-EBI-UniProt-GOA-ACAP-ARAP_13.05.2021_00h00	2.75E-67	14.917127	81	[ACTR2, ADAM10, AGPAT2, ALDOA, ANO6, ANXA3, APAF1, APRT, ASAH1, ATG7, ATP6V0A1, ATP6V0C, B2M, B4GALT1, BST1, CAP1, CCT2, CHAMP1, CLU, CSTB, CTSA, CTSC, CYB5R3, DIAPH1, DSP, DYNC1LI1, DYNLL1, ERP44, FTH1, FTL, GLA, GOLGA7, GPI, GSDMD, GSTP1, HEXB, HLA-B, HLA-C, HSP90AA1, IST1, ITGAV, ITM2C, KPNB1, LAMP1, MGST1, MME, MTCH1, MVP, MYD88, PAFAH1B2, PDAP1, PKM, PLD1, PNP, PPIE, PRDX6, PRKCSH, PSMC2, PSMC3, PSMD1, PSMD12, PSMD14, PSMD2, PSMD6, PSMD7, PTGES2, PYCARD, PYGB, QSOX1, RAB10, RAB3D, RAP2B, RAP2C, SERPINB1, SLC44A2, STOM, STXBP3, TOM1, TUBB, UBR4, VAT1]	Downregulation
RNA catabolic process	GO_BiologicalProcess-EBI-UniProt-GOA-ACAP-ARAP_13.05.2021_00h00	2.75E-67	17.977528	80	[AGO2, APEX1, CIRBP, CNOT1, CNOT10, DCPS, DHX34, DHX36, EIF3E, FEN1, FMR1, FTO, HNRNPA0, HNRNPC, HNRNPR, IGF2BP1, IGF2BP2, LARP1, LARP4B, LRPPRC, METTL14, MLH1, MOV10, MRTO4, MTREX, MYD88, NOCT, NPM1, NT5C3B, NUDT16, NUDT16L1, PAIP1, PELO, POP1, PSMA3, PSMB10, PSMB2, PSMC2, PSMC3, PSMC6, PSMD1, PSMD12, PSMD14, PSMD2, PSMD6, PSMD7, PSMD8, PSME1, PSME2, PSME4, RNPS1, RPL10A, RPL13, RPL13A, RPL18A, RPL22, RPL24, RPL27, RPL32, RPL36, RPL38, RPL5, RPLP1, RPLP2, RPS13, RPS15A, RPS16, RPS18, RPS21, RPS23, RPS4Y1, RPS5, RPS8, RPS9, SET, SSB, TUT4, YBX1, ZC3HAV1, ZFP36L2]	Downregulation

translation	GO_BiologicalProcess-EBI-UniProt-GOA-ACAP-ARAP_13.05.2021_00h00	2.75E-67	16.212534	119	[AARS1, ABCE1, ACO1, AGO2, APP, C1QBP, CIRBP, CNOT1, CNOT10, DAPK1, DARS1, DCPS, DDX1, DENR, DHFR, DHPS, DHX36, DRG1, EARS2, EEF1E1, EEF1G, EFL1, EIF2AK2, EIF2B1, EIF2D, EIF2S1, EIF3A, EIF3B, EIF3E, EIF3F, EIF4G3, EIF6, EPRS1, ERBB2, FMR1, FTO, GAPDH, GARS1, GATB, GLE1, HARS1, HNRNPR, IARS2, IGF2BP1, IGF2BP2, ILF3, IMPACT, IREB2, LARP1, LARP4B, LARP6, LRPPRC, METTL14, MLH1, MOV10, MRPL10, MRPS17, MRPS21, MRPS31, MRRF, N6AMT1, NCK2, NCL, NDRG1, NIBAN1, NOCT, NPM1, NSUN5, NT5C3B, OGFOD1, OXA1L, PAIP1, PELO, PKM, PLD1, PLXNB2, PPP1CA, PRKCSH, PUS7, QARS1, RARS2, RBM4, RPL10A, RPL13, RPL13A, RPL18A, RPL22, RPL24, RPL27, RPL32, RPL36, RPL38, RPL5, RPLP1, RPLP2, RPS13, RPS15A, RPS16, RPS18, RPS21, RPS23, RPS4Y1, RPS5, RPS8, RPS9, SARNP, SARS1, SELENOT, SESN2, SHFL, SHMT2, TBCE, THBS1, TRMT10C, TUT4, VARS1, YARS1, YBX1, ZFP36L2]	No change
translational initiation	GO_BiologicalProcess-EBI-UniProt-GOA-ACAP-ARAP_13.05.2021_00h00	2.75E-67	22.00957	46	[ABCE1, AGO2, DDX1, DENR, EIF2AK2, EIF2B1, EIF2D, EIF2S1, EIF3A, EIF3B, EIF3E, EIF3F, EIF4G3, EIF6, FMR1, GLE1, IMPACT, LARP1, NCK2, NPM1, PAIP1, PPP1CA, RBM4, RPL10A, RPL13, RPL13A, RPL18A, RPL22, RPL24, RPL27, RPL32, RPL36, RPL38, RPL5, RPLP1, RPLP2, RPS13, RPS15A, RPS16, RPS18, RPS21, RPS23, RPS4Y1, RPS5, RPS8, RPS9]	Downregulation
protein targeting	GO_BiologicalProcess-EBI-UniProt-GOA-	2.75E-67	17.772512	75	[ALDH3A2, ANK3, APEX1, CEMIP, CHORDC1, CHP1,	Downregulation

	ACAP-ARAP_13.05.2021_00h00				CLU, ERBB2, GCC2, GDAP1, GET3, GIPC1, GOLGA7, HACL1, HGS, HSP90AA1, HSPA4, HSPA5, M6PR, MAGI1, MFF, MICALL1, MYO1E, NDUFA13, NEDD4, PARL, PIN1, PITRM1, PRKAA1, PRKCI, RABGEF1, RNF2, RPL10A, RPL13, RPL13A, RPL18A, RPL22, RPL24, RPL27, RPL32, RPL36, RPL38, RPL5, RPLP1, RPLP2, RPS13, RPS15A, RPS16, RPS18, RPS21, RPS23, RPS4Y1, RPS5, RPS8, RPS9, SAE1, SCARB2, SEC61A1, SEC61B, SLC1A1, SLC25A6, SORT1, SRPRA, SSR3, STOM, TCAF1, TIMM17B, TOMM34, TOMM7, TRAM1, TRAM2, TSPO, VPS41, ZDHHC21, ZFAND6]	
protein localization to nucleus	GO_BiologicalProcess-EBI-UniProt-GOA-ACAP-ARAP_13.05.2021_00h00	2.75E-67	15.181519	46	[APLP2, APPL2, CCT2, CCT3, CCT6A, CDK1, CDK5RAP3, CHORDC1, CHP1, FERMT1, FERMT2, GLUL, GSK3B, HEATR3, IPO5, IPO9, KAT7, KPNB1, NF2, NMD3, NOP53, NPM1, NUP35, NUP62, NUTF2, OSBPL8, PARP1, PIN1, PML, PPP3CA, RAB23, RANGAP1, RBM22, RPF2, SESN2, SRC, SUFU, TCP1, TNPO2, TOR1AIP1, TOR1AIP2, TOR1B, TRIM28, TSC2, TXN, YAP1]	Upregulation
proteolysis involved in cellular protein catabolic process	GO_BiologicalProcess-EBI-UniProt-GOA-ACAP-ARAP_13.05.2021_00h00	2.75E-67	12.483746	96	[ANKZF1, ARAF, ARRB1, ATE1, AUP1, CCDC22, CDC34, CDK1, CDK2, CDK5RAP3, CLU, CRBN, CTNNB1, CTSC, CUL1, CUL4B, DAB2, DDI2, DERL1, ERLIN2, FBXL12, FBXW8, FMR1, GIPC1, GLMN, GSK3B, HSP90B1, HSPA5, MAD2L1, MAEA, MAGI1, MALT1, MTA1, NEDD4, NEDD8, NOP53, NPLOC4, NUB1, NUDT15, PDCD6IP, PITHD1, PML, PRPF19, PSMA3, PSMB10, PSMB2, PSMC2,	No change

					PSMC3, PSMC6, PSMD1, PSMD12, PSMD14, PSMD2, PSMD6, PSMD7, PSMD8, PSME1, PSME2, PSME4, RCN3, RNF114, RNF2, RNF213, RNF40, RPL5, RSPRY1, RYBP, SEC61B, SIRT1, SMARCC1, SNF8, SQSTM1, SUFU, TRIM25, TRIP12, UBAC2, UBE2L6, UBR2, UBR4, UBXN2A, UFD1, USP11, USP22, USP28, USP4, USP48, USP5, USP7, USP9X, USP9Y, VPS25, VPS36, WFS1, WWP2, YOD1, ZMPSTE24]	
interleukin-1-mediated signaling pathway	GO_BiologicalProcess-EBI-UniProt-GOA-ACAP-ARAP_13.05.2021_00h00	2.75E-67	23.30097	24	[CUL1, IGF2BP1, IKBKB, IKBKG, MYD88, PSMA3, PSMB10, PSMB2, PSMC2, PSMC3, PSMC6, PSMD1, PSMD12, PSMD14, PSMD2, PSMD6, PSMD7, PSMD8, PSME1, PSME2, PSME4, RIPK2, SQSTM1, TAB2]	Downregulation
protein localization to endoplasmic reticulum	GO_BiologicalProcess-EBI-UniProt-GOA-ACAP-ARAP_13.05.2021_00h00	2.75E-67	22.222221	34	[GET3, HSPA5, PTGES2, RAB10, RPL10A, RPL13, RPL13A, RPL18A, RPL22, RPL24, RPL27, RPL32, RPL36, RPL38, RPL5, RPLP1, RPLP2, RPS13, RPS15A, RPS16, RPS18, RPS21, RPS23, RPS4Y1, RPS5, RPS8, RPS9, RTN4, SEC61A1, SEC61B, SRPRA, SSR3, TRAM1, TRAM2]	Downregulation
establishment of protein localization to endoplasmic reticulum	GO_BiologicalProcess-EBI-UniProt-GOA-ACAP-ARAP_13.05.2021_00h00	2.75E-67	25.396826	32	[GET3, HSPA5, RAB10, RPL10A, RPL13, RPL13A, RPL18A, RPL22, RPL24, RPL27, RPL32, RPL36, RPL38, RPL5, RPLP1, RPLP2, RPS13, RPS15A, RPS16, RPS18, RPS21, RPS23, RPS4Y1, RPS5, RPS8, RPS9, SEC61A1, SEC61B, SRPRA, SSR3, TRAM1, TRAM2]	Downregulation
regulation of protein localization to nucleus	GO_BiologicalProcess-EBI-UniProt-GOA-ACAP-ARAP_13.05.2021_00h00	2.75E-67	19.310345	28	[CCT2, CCT3, CCT6A, CDK1, CDK5RAP3, CHORDC1, CHP1, FERMT1, FERMT2, GLUL, GSK3B, IPO5, KAT7, NF2, NMD3, NPM1, NUP62, NUTF2, PARP1, PIN1,	Upregulation

					RAB23, RBM22, SESN2, SRC, SUFU, TCP1, TRIM28, YAP1]	
activation of innate immune response	GO_BiologicalProcess-EBI-UniProt-GOA-ACAP-ARAP_13.05.2021_00h00	2.75E-67	18.98734	30	[CUL1, HSP90AA1, IGF2BP1, IKBKB, IKBKG, MALT1, MATR3, PKN2, PSMA3, PSMB10, PSMB2, PSMC2, PSMC3, PSMC6, PSMD1, PSMD12, PSMD14, PSMD2, PSMD6, PSMD7, PSMD8, PSME1, PSME2, PSME4, PSPC1, PYCARD, SRC, TAB2, TBK1, ZCCHC3]	Downregulation
mRNA catabolic process	GO_BiologicalProcess-EBI-UniProt-GOA-ACAP-ARAP_13.05.2021_00h00	2.75E-67	19	76	[AGO2, APEX1, CIRBP, CNOT1, CNOT10, DCPS, DHX34, DHX36, EIF3E, FMR1, FTO, HNRNPA0, HNRNPC, HNRNPR, IGF2BP1, IGF2BP2, LARP1, LARP4B, METTL14, MLH1, MOV10, MRTO4, MYD88, NOCT, NPM1, NT5C3B, NUDT16, NUDT16L1, PAIP1, PELO, PSMA3, PSMB10, PSMB2, PSMC2, PSMC3, PSMC6, PSMD1, PSMD12, PSMD14, PSMD2, PSMD6, PSMD7, PSMD8, PSME1, PSME2, PSME4, RNPS1, RPL10A, RPL13, RPL13A, RPL18A, RPL22, RPL24, RPL27, RPL32, RPL36, RPL38, RPL5, RPLP1, RPLP2, RPS13, RPS15A, RPS16, RPS18, RPS21, RPS23, RPS4Y1, RPS5, RPS8, RPS9, SET, SSB, TUT4, YBX1, ZC3HAV1, ZFP36L2]	Downregulation
regulation of translation	GO_BiologicalProcess-EBI-UniProt-GOA-ACAP-ARAP_13.05.2021_00h00	2.75E-67	16.421053	78	[AARS1, ABCE1, ACO1, AGO2, APP, C1QBP, CIRBP, CNOT1, CNOT10, DAPK1, DCPS, DDX1, DENR, DHFR, DHX36, EEF1G, EFL1, EIF2AK2, EIF2B1, EIF2D, EIF2S1, EIF3A, EIF3B, EIF3E, EIF3F, EIF4G3, EIF6, EPRS1, ERBB2, FMR1, FTO, GAPDH, GLE1, HNRNPR, IGF2BP1, IGF2BP2, ILF3, IMPACT, IREB2, LARP1, LARP4B, LARP6, LRPPRC, METTL14, MLH1, MOV10, NCK2, NCL, NIBAN1, NOCT,	No change

					NPM1, NSUN5, NT5C3B, OGFOD1, PAIP1, PELO, PKM, PLD1, PLXNB2, PPP1CA, PRKCSH, PUS7, RBM4, RPL13A, RPL22, RPL38, RPL5, RPS9, SARNP, SELENOT, SESN2, SHFL, SHMT2, THBS1, TRMT10C, TUT4, YBX1, ZFP36L2]	
protein targeting to membrane	GO_BiologicalProcess-EBI-UniProt-GOA-ACAP-ARAP_13.05.2021_00h00	2.75E-67	20.600859	48	[ALDH3A2, ANK3, CEMIP, CHORDC1, CHP1, ERBB2, GDAP1, GET3, GOLGA7, HSPA5, MFF, MICALL1, MYO1E, PRKCI, RABGEF1, RPL10A, RPL13, RPL13A, RPL18A, RPL22, RPL24, RPL27, RPL32, RPL36, RPL38, RPL5, RPLP1, RPLP2, RPS13, RPS15A, RPS16, RPS18, RPS21, RPS23, RPS4Y1, RPS5, RPS8, RPS9, SEC61A1, SEC61B, SLC1A1, SRPRA, SSR3, STOM, TCAF1, TRAM1, TRAM2, ZDHHC21]	Downregulation
proteasomal protein catabolic process	GO_BiologicalProcess-EBI-UniProt-GOA-ACAP-ARAP_13.05.2021_00h00	2.75E-67	13.069307	66	[ANKZF1, ARAF, ARRB1, AUP1, CDC34, CDK1, CDK2, CLU, CRBN, CTNNB1, CUL1, CUL4B, DAB2, DDI2, DERL1, ERLIN2, FBXL12, FBXW8, FMR1, GIPC1, GSK3B, HSP90B1, HSPA5, MAD2L1, MAEA, MTA1, NEDD4, NOP53, NPLOC4, NUB1, NUDT15, PITHD1, PML, PRPF19, PSMA3, PSMB10, PSMB2, PSMC2, PSMC3, PSMC6, PSMD1, PSMD12, PSMD14, PSMD2, PSMD6, PSMD7, PSMD8, PSME1, PSME2, PSME4, RCN3, RNF40, RYBP, SEC61B, SIRT1, SMARCC1, TRIM25, UBAC2, UBR2, UBXN2A, UFD1, USP5, USP7, WFS1, WWP2, YOD1]	No change
ncRNA metabolic process	GO_BiologicalProcess-EBI-UniProt-GOA-ACAP-ARAP_13.05.2021_00h00	2.75E-67	15.08079	84	[AARS1, ABT1, AGO2, BMS1, CHD7, CNGB1, COLEC12, CTU1, DARS1, DDX1, DDX47, DICER1, DIMT1, EARS2, EEF1E1, EIF6, ELP3, ELP4, ELP5, EMG1,	No change

					EPRS1, GARS1, GATB, GEMIN4, HARS1, HSD17B10, IARS2, INTS1, INTS9, MRM3, MRTO4, MTREX, NEDD4, NOL11, NOP53, NSUN5, NUDT16, NUDT16L1, PELO, POP1, PUS7, QARS1, QTRT1, QTRT2, RARS2, RIOK3, RPF2, RPL10A, RPL13, RPL13A, RPL18A, RPL22, RPL24, RPL27, RPL32, RPL36, RPL38, RPL5, RPLP1, RPLP2, RPS16, RPS21, RPS8, RPS9, SARS1, SBDS, SENP3, SNU13, SSB, THADA, TRMT10C, TRMT5, TSEN15, TSEN34, TSR2, TUT4, URM1, UTP14A, UTP20, UTP3, VARS1, WDR12, YARS1, YBEY]	
secretory granule lumen	GO_CellularComponent-EBI-UniProt-GOA-ACAP-ARAP_13.05.2021_00h00	2.75E-67	15.5124655	56	[ACTR2, ALB, ALDOA, APAF1, APP, APRT, ATG7, B2M, CAP1, CCT2, CHAMP1, CLU, CSTB, CTSA, CTSC, CYB5R3, CYRIB, ERP44, FN1, FTL, GLA, GPI, GSDMD, GSTP1, HEXB, HSP90AA1, IST1, ITIH4, KPNB1, LGALS3BP, MAGED2, MVP, NHLRC2, PAFAH1B2, PKM, PNP, PPIE, PRDX6, PSMC2, PSMC3, PSMD1, PSMD12, PSMD14, PSMD2, PSMD6, PSMD7, PTGES2, PYCARD, PYGB, QSOX1, SERPINB1, SPARC, TF, THBS1, TUBB, VAT1]	Downregulation
non-canonical Wnt signaling pathway	GO_BiologicalProcess-EBI-UniProt-GOA-ACAP-ARAP_13.05.2021_00h00	2.75E-67	18.354431	29	[AGO2, CLTC, CTNNB1, DAB2, DVL2, GPC6, GPI, MOV10, PLCB3, PPP3CA, PSMA3, PSMB10, PSMB2, PSMC2, PSMC3, PSMC6, PSMD1, PSMD12, PSMD14, PSMD2, PSMD6, PSMD7, PSMD8, PSME1, PSME2, PSME4, PTK7, RNF213, ROR1]	Downregulation
post-translational protein modification	GO_BiologicalProcess-EBI-UniProt-GOA-ACAP-ARAP_13.05.2021_00h00	2.75E-67	14.0625	54	[ADAM10, ALB, APLP2, APP, ASB1, ATG7, CCDC22, CRTAP, CUL1, CUL4B, FBXL12, FBXW8, FN1, FN3K, GAN, HSP90B1, IGFBP7, LAMC1, LMO7, LTBP1,	No change

					NEDD8, NUB1, OBSL1, P3H1, PPIB, PPP3CA, PRKCSH, PSMA3, PSMB10, PSMB2, PSMC2, PSMC3, PSMC6, PSMD1, PSMD12, PSMD14, PSMD2, PSMD6, PSMD7, PSMD8, PSME1, PSME2, PSME4, QSOX1, RAB11B, RAB1A, RAB5A, RAB8A, RCN1, TF, UBA3, WDR5, WFS1, WIPI2]	
ficolin-1-rich granule	GO_CellularComponent-EBI-UniProt-GOA-ACAP-ARAP_13.05.2021_00h00	2.75E-67	16.504854	34	[ACTR2, ALDOA, APAF1, ASAH1, ATG7, ATP6V0A1, ATP6V0C, CLU, CSTB, DIAPH1, DSP, DYNC1LI1, DYNLL1, FTH1, GPI, GSDMD, GSTP1, HSP90AA1, KPNB1, LAMP1, MVP, PAFAH1B2, PDAP1, PKM, PNP, PPIE, PSMC2, PSMC3, PSMD12, PSMD14, PSMD2, PSMD6, PSMD7, UBR4]	Downregulation
regulation of hematopoietic progenitor cell differentiation	GO_BiologicalProcess-EBI-UniProt-GOA-ACAP-ARAP_13.05.2021_00h00	2.75E-67	21.875	21	[DHX36, EIF2AK2, METTL14, PSMA3, PSMB10, PSMB2, PSMC2, PSMC3, PSMC6, PSMD1, PSMD12, PSMD14, PSMD2, PSMD6, PSMD7, PSMD8, PSME1, PSME2, PSME4, PUS7, YAP1]	Downregulation
negative regulation of cell cycle G2/M phase transition	GO_BiologicalProcess-EBI-UniProt-GOA-ACAP-ARAP_13.05.2021_00h00	2.75E-67	20.168068	24	[CDK1, CDK5RAP3, CUL1, FHL1, MACROH2A1, NOP53, PSMA3, PSMB10, PSMB2, PSMC2, PSMC3, PSMC6, PSMD1, PSMD12, PSMD14, PSMD2, PSMD6, PSMD7, PSMD8, PSME1, PSME2, PSME4, RAD21, SLC25A15]	Downregulation
regulation of mRNA metabolic process	GO_BiologicalProcess-EBI-UniProt-GOA-ACAP-ARAP_13.05.2021_00h00	2.75E-67	17.1875	66	[AGO2, APEX1, C1QBP, CDC73, CELF2, CIRBP, CNOT1, CNOT10, CWC22, DCPS, DHX34, DHX36, FMR1, FTO, HNRNPA0, HNRNPC, HNRNPR, IGF2BP1, IGF2BP2, LARP1, LARP4B, METTL14, MLH1, MOV10, MYD88, NCL, NOCT, NPM1, NT5C3B, PAIP1, PRDX6, PRPF19, PSMA3, PSMB10, PSMB2, PSMC2, PSMC3, PSMC6, PSMD1, PSMD12, PSMD14,	No change

					PSMD2, PSMD6, PSMD7, PSMD8, PSME1, PSME2, PSME4, PUF60, RBM4, RNF40, RNPS1, SET, SF1, SMU1, SNW1, SON, SRSF10, SRSF2, TRA2A, TRA2B, TUT4, YBX1, YTHDC1, ZC3HAV1, ZFP36L2]	
cytoplasmic translation	GO_BiologicalProcess-EBI-UniProt-GOA-ACAP-ARAP_13.05.2021_00h00	2.75E-67	21.95122	36	[AARS1, DENR, DHX36, DRG1, EIF2D, EIF3A, EIF3B, EIF3E, EIF3F, EIF6, FMR1, NDRG1, PKM, RBM4, RPL10A, RPL13, RPL13A, RPL18A, RPL22, RPL24, RPL27, RPL32, RPL36, RPL38, RPL5, RPLP1, RPLP2, RPS13, RPS15A, RPS16, RPS18, RPS21, RPS23, RPS5, RPS8, RPS9]	Downregulation
mRNA processing	GO_BiologicalProcess-EBI-UniProt-GOA-ACAP-ARAP_13.05.2021_00h00	2.75E-67	17.153284	94	[AAR2, ADARB1, APP, AQR, BCAS2, C1QBP, CCNH, CDC5L, CDC73, CELF2, CHTOP, CIRBP, CMTR1, COIL, COLEC12, CPSF2, CSTF3, CTNNBL1, CWC22, DCPS, DDX1, DDX42, DDX47, DHX36, FMR1, GCFC2, GEMIN4, GPKOW, GTF2F1, GTF2F2, HNRNPA0, HNRNPA3, HNRNPC, HNRNPH1, HNRNPLL, HNRNPR, HNRNPUL1, HTATSF1, KDM1A, METTL14, MTREX, NCL, NPM1, PIN1, POLR2A, POLR2B, POLR2H, POLR2I, PPIE, PPWD1, PRDX6, PRMT7, PRPF19, PSIP1, PSPC1, PTBP2, PTBP3, PUF60, RBM22, RBM26, RBM4, RNF40, RNGTT, RNPS1, RPRD1A, RPRD1B, RPRD2, SARNP, SCAF11, SF1, SF3A3, SF3B6, SMNDC1, SMU1, SNRNP200, SNRPA, SNRPD3, SNRPE, SNU13, SNW1, SON, SRSF10, SRSF2, SRSF5, TGS1, THOC2, TRA2A, TRA2B, TSEN15, TSEN34, U2SURP, USP4, YBX1, YTHDC1]	Upregulation
RNA splicing	GO_BiologicalProcess-EBI-	2.75E-67	17.436975	83	[AAR2, AQR, BCAS2, C1QBP, CDC5L,	Upregulation

	UniProt-GOA-ACAP-ARAP_13.05.2021_00h00				CELF2, CIRBP, COIL, COLEC12, CPSF2, CSTF3, CTNNBL1, CWC22, DCPS, DDX1, DDX42, DDX47, FMR1, GCFC2, GEMIN4, GPKOW, GTF2F1, GTF2F2, HNRNPA0, HNRNPA3, HNRNPC, HNRNPH1, HNRNPLL, HNRNPR, HNRNPUL1, HTATSF1, IVNS1ABP, KDM1A, METTL14, MTREX, NCL, NPM1, PIN1, POLR2A, POLR2B, POLR2H, POLR2I, PPIE, PPIG, PPWD1, PRDX6, PRMT7, PRPF19, PSIP1, PSPC1, PTBP2, PTBP3, PUF60, RBM22, RBM4, RNPS1, RPS13, SCAF11, SF1, SF3A3, SF3B6, SMNDC1, SMU1, SNRNP200, SNRPA, SNRPD3, SNRPE, SNU13, SNW1, SON, SRSF10, SRSF2, SRSF5, TGS1, THOC2, TRA2A, TRA2B, TSEN15, TSEN34, U2SURP, USP4, YBX1, YTHDC1]	
ncRNA processing	GO_BiologicalProcess-EBI-UniProt-GOA-ACAP-ARAP_13.05.2021_00h00	2.75E-67	15.334773	71	[AARS1, ABT1, AGO2, BMS1, CHD7, CNGB1, COLEC12, CTU1, DDX1, DDX47, DICER1, DIMT1, EIF6, ELP3, ELP4, ELP5, EMG1, GARS1, GEMIN4, HSD17B10, INTS1, INTS9, MRM3, MRTO4, MTREX, NEDD4, NOL11, NOP53, NSUN5, NUDT16, POP1, PUS7, QTRT1, QTRT2, RIOK3, RPF2, RPL10A, RPL13, RPL13A, RPL18A, RPL22, RPL24, RPL27, RPL32, RPL36, RPL38, RPL5, RPLP1, RPLP2, RPS16, RPS21, RPS8, RPS9, SARS1, SBDS, SENP3, SNU13, SSB, THADA, TRMT10C, TRMT5, TSEN15, TSEN34, TSR2, TUT4, URM1, UTP14A, UTP20, UTP3, WDR12, YBEY]	No change
regulation of RNA splicing	GO_BiologicalProcess-EBI-UniProt-GOA-ACAP-ARAP_13.05.2021_00h00	2.75E-67	18.125	29	[C1QBP, CELF2, CIRBP, CWC22, FMR1, HNRNPH1, HNRNPLL, NCL, NPM1, POLR2A, PRDX6, PRPF19, PTBP2, PTBP3, PUF60, RBM22,	Upregulation

					RBM4, RNPS1, RPS13, SF1, SMU1, SNW1, SON, SRSF10, SRSF2, SRSF5, TRA2A, TRA2B, YTHDC1]	
positive regulation of translation	GO_BiologicalProcess-EBI-UniProt-GOA-ACAP-ARAP_13.05.2021_00h00	2.75E-67	18.705036	26	[AGO2, C1QBP, CIRBP, DHX36, EIF3E, EIF6, ERBB2, FMR1, IMPACT, LARP1, LARP4B, LARP6, METTL14, NCK2, NIBAN1, NPM1, NSUN5, PAIP1, PKM, PLD1, PLXNB2, PRKCSH, RPL5, RPS9, THBS1, TRMT10C]	No change
hematopoietic stem cell differentiation	GO_BiologicalProcess-EBI-UniProt-GOA-ACAP-ARAP_13.05.2021_00h00	2.75E-67	21.276596	20	[CHD2, EIF2AK2, PSMA3, PSMB10, PSMB2, PSMC2, PSMC3, PSMC6, PSMD1, PSMD12, PSMD14, PSMD2, PSMD6, PSMD7, PSMD8, PSME1, PSME2, PSME4, PUS7, YAP1]	Downregulation
regulation of mRNA catabolic process	GO_BiologicalProcess-EBI-UniProt-GOA-ACAP-ARAP_13.05.2021_00h00	2.75E-67	18.039215	46	[AGO2, APEX1, CIRBP, CNOT1, CNOT10, DCPS, DHX34, DHX36, FMR1, FTO, HNRNPA0, HNRNPC, HNRNPR, IGF2BP1, IGF2BP2, LARP1, LARP4B, METTL14, MLH1, MOV10, MYD88, NOCT, NPM1, NT5C3B, PAIP1, PSMA3, PSMB10, PSMB2, PSMC2, PSMC3, PSMC6, PSMD1, PSMD12, PSMD14, PSMD2, PSMD6, PSMD7, PSMD8, PSME1, PSME2, PSME4, SET, TUT4, YBX1, ZC3HAV1, ZFP36L2]	No change
regulation of mRNA stability	GO_BiologicalProcess-EBI-UniProt-GOA-ACAP-ARAP_13.05.2021_00h00	2.75E-67	17.813765	44	[AGO2, APEX1, CIRBP, CNOT1, CNOT10, DCPS, DHX36, FMR1, FTO, HNRNPA0, HNRNPC, HNRNPR, IGF2BP1, IGF2BP2, LARP1, LARP4B, METTL14, MLH1, MOV10, MYD88, NOCT, NPM1, NT5C3B, PAIP1, PSMA3, PSMB10, PSMB2, PSMC2, PSMC3, PSMC6, PSMD1, PSMD12, PSMD14, PSMD2, PSMD6, PSMD7, PSMD8, PSME1, PSME2, PSME4, SET, TUT4, YBX1, ZFP36L2]	No change
nuclear-transcribed mRNA catabolic process	GO_BiologicalProcess-EBI-UniProt-GOA-ACAP-	2.75E-67	17.567568	39	[AGO2, CNOT1, CNOT10, DCPS, DHX34, DHX36, EIF3E, MLH1,	Downregulation

	ARAP_13.05.2021_00h00				MRTO4, NOCT, NT5C3B, PELO, RNPS1, RPL10A, RPL13, RPL13A, RPL18A, RPL22, RPL24, RPL27, RPL32, RPL36, RPL38, RPL5, RPLP1, RPLP2, RPS13, RPS15A, RPS16, RPS18, RPS21, RPS23, RPS4Y1, RPS5, RPS8, RPS9, SSB, TUT4, ZFP36L2]	
rRNA metabolic process	GO_BiologicalProcess-EBI-UniProt-GOA-ACAP-ARAP_13.05.2021_00h00	2.75E-67	15.753425	46	[ABT1, BMS1, CHD7, CNGB1, DDX47, DIMT1, EIF6, EMG1, GEMIN4, MRM3, MRTO4, MTREX, NEDD4, NOL11, NOP53, NSUN5, NUDT16, PELO, RIOK3, RPF2, RPL10A, RPL13, RPL13A, RPL18A, RPL22, RPL24, RPL27, RPL32, RPL36, RPL38, RPL5, RPLP1, RPLP2, RPS16, RPS21, RPS8, RPS9, SBDS, SENP3, SNU13, TSR2, UTP14A, UTP20, UTP3, WDR12, YBEY]	No change
regulation of hematopoietic stem cell differentiation	GO_BiologicalProcess-EBI-UniProt-GOA-ACAP-ARAP_13.05.2021_00h00	2.75E-67	24.358974	19	[EIF2AK2, PSMA3, PSMB10, PSMB2, PSMC2, PSMC3, PSMC6, PSMD1, PSMD12, PSMD14, PSMD2, PSMD6, PSMD7, PSMD8, PSME1, PSME2, PSME4, PUS7, YAP1]	Downregulation
T cell receptor signaling pathway	GO_BiologicalProcess-EBI-UniProt-GOA-ACAP-ARAP_13.05.2021_00h00	2.75E-67	15.981735	35	[CD276, CD81, CUL1, DUSP3, EIF2B1, EZR, GBP1, HLA-A, IKBKB, IKBKG, MALT1, NECTIN2, PAWR, PHPT1, PIK3CA, PKN2, PLCG1, PSMA3, PSMB10, PSMB2, PSMC2, PSMC3, PSMC6, PSMD1, PSMD12, PSMD14, PSMD2, PSMD6, PSMD7, PSMD8, PSME1, PSME2, PSME4, RIPK2, TAB2]	Downregulation
nuclear-transcribed mRNA catabolic process, nonsense-mediated decay	GO_BiologicalProcess-EBI-UniProt-GOA-ACAP-ARAP_13.05.2021_00h00	2.75E-67	19.847328	26	[DHX34, EIF3E, RNPS1, RPL10A, RPL13, RPL13A, RPL18A, RPL22, RPL24, RPL27, RPL32, RPL36, RPL38, RPL5, RPLP1, RPLP2, RPS13, RPS15A, RPS16, RPS18, RPS21, RPS23, RPS4Y1, RPS5, RPS8, RPS9]	Downregulation
SRP-dependent cotranslational protein targeting to membrane	GO_BiologicalProcess-EBI-UniProt-GOA-ACAP-	2.75E-67	27.619047	29	[RPL10A, RPL13, RPL13A, RPL18A, RPL22, RPL24, RPL27, RPL32,	Downregulation

	ARAP_13.05.2021_00h00				RPL36, RPL38, RPL5, RPLP1, RPLP2, RPS13, RPS15A, RPS16, RPS18, RPS21, RPS23, RPS4Y1, RPS5, RPS8, RPS9, SEC61A1, SEC61B, SRPRA, SSR3, TRAM1, TRAM2]	
mRNA splicing, via spliceosome	GO_BiologicalProcess-EBI-UniProt-GOA-ACAP-ARAP_13.05.2021_00h00	2.75E-67	19.945354	73	[AAR2, AQR, BCAS2, C1QBP, CDC5L, CELF2, CIRBP, COIL, COLEC12, CPSF2, CSTF3, CTNNBL1, CWC22, DCPS, DDX1, DDX42, FMR1, GCFC2, GEMIN4, GPKOW, GTF2F1, GTF2F2, HNRNPA0, HNRNPA3, HNRNPC, HNRNPH1, HNRNPR, HNRNPUL1, HTATSF1, KDM1A, METTL14, MTREX, NCL, NPM1, PIN1, POLR2A, POLR2B, POLR2H, POLR2I, PPIE, PPWD1, PRDX6, PRMT7, PRPF19, PSIP1, PSPC1, PUF60, RBM22, RBM4, RNPS1, SCAF11, SF1, SF3A3, SF3B6, SMNDC1, SMU1, SNRNP200, SNRPA, SNRPD3, SNRPE, SNU13, SNW1, SON, SRSF10, SRSF2, SRSF5, TGS1, TRA2A, TRA2B, U2SURP, USP4, YBX1, YTHDC1]	Upregulation
anaphase-promoting complex-dependent catabolic process	GO_BiologicalProcess-EBI-UniProt-GOA-ACAP-ARAP_13.05.2021_00h00	2.75E-67	21.83908	19	[CDK1, CDK2, MAD2L1, PSMA3, PSMB10, PSMB2, PSMC2, PSMC3, PSMC6, PSMD1, PSMD12, PSMD14, PSMD2, PSMD6, PSMD7, PSMD8, PSME1, PSME2, PSME4]	Downregulation

**Supplementary Table S4.** Proteins Upregulated in Male iPSCs and Male iVSMCs					
	**Related to Vascular Function**	**Related to Vascular Disease**	**Related to Vascular Disease in Another Cell Type**	**PMID**	**Source**
LMNB1	no	no	yes	32788068	https://pubmed.ncbi.nlm.nih.gov/32788068/
ALDH1B1	no	no	no		
PGM1	no	no	no		
RBM34	no	no	yes	31881747	https://www.mdpi.com/2218-273X/10/1/35
CEBPZ	no	no	no		
CGNL1	yes	no	no	30809157	https://www.frontiersin.org/journals/physiology/articles/10.3389/fphys.2019.00101/full
SEPTIN6	no	no	no		
APOOL	no	no	no		
PBXIP1	no	no	no		
CPPED1	no	no	no		
PRXL2A	no	no	yes	29212778	https://pubmed.ncbi.nlm.nih.gov/29212778/
SHPK	no	no	no		

Supplementary Table S5. Proteins Upregulated in Female iPSCs and Female iVSMCs					
	**Related to Vascular Function**	**Related to Vascular Disease**	**Related to Vascular Disease in Another Cell Type**	**PMID**	**Source**
ARVCF	no	no	yes	37759455	https://pubmed.ncbi.nlm.nih.gov/37759455/
UQCR11	no	no	yes	32760426	https://pubmed.ncbi.nlm.nih.gov/32760426/
GYG2	no	no	yes	32541024	https://www.pnas.org/doi/pdf/10.1073/pnas.2006348117
DIAPH2	no	no	no		
CPNE3	no	no	yes	29297177	https://pubmed.ncbi.nlm.nih.gov/29297177/
COL4A1	no	no	yes		
PLS3	no	no	yes	31694393	https://doi.org/10.1161/ATVBAHA.119.313440
NQO2	no	no	no		
UBA1	no	no	yes	32258175	https://www.ncbi.nlm.nih.gov/pmc/articles/PMC7109586
CPT2	no	no	no		
PNPLA4	no	no	no		
PRCP	no	no	yes	23744584	https://pubmed.ncbi.nlm.nih.gov/23744584/
USP11	no	no	no		
FPGS	no	no	no		
PUDP	no	yes	yes		https://doi.org/10.1101/2023.02.09.527800
IQGAP2	no	no	no		
PPA1	no	no	no		
SURF1	no	no	no		
RBBP7	no	no	no		
HADH	no	no	yes	36035955	https://pubmed.ncbi.nlm.nih.gov/36035955/
LYRM7	no	yes	no	29876469	https://doi.org/10.1016/j.dib.2018.01.108
PRPF38B	no	no	no		

POGLUT3	no	no	no		
TMEM256	no	no	no		
SYAP1	no	no	no		
MRI1	no	no	no		
PDCL3	no	no	no		
C17orf75	no	no	no		
CTPS2	no	no	yes	36970364	https://pubmed.ncbi.nlm.nih.gov/36970364/
MIOS	no	no	no		
DNPEP	no	no	no		
RAP2c	no	no	yes	33363474	https://www.ncbi.nlm.nih.gov/pmc/articles/PMC7753098/

Supplementary Table S6. Proteins Upregulated in Male iVSMCs									
	Related to Vascular Function (broad, related to any vascular)	Related to Vascular Disease in VSMCs (endothelial,related to	Related to Vascular Disease in Another Cell Type	Up or Down (what about the mutations?)	Protective vs. Risk	Disease	PMID	Source	Sidenotes and More Papers
**Up in Males**									
iVSMCs									
UNC119B	no	no	yes			CAD	35175464	Transcriptome-wide association study of coronary artery disease identifies novel susceptibility genes - PMC (nih.gov)	
PDLIM1	yes	yes	yes	Down	Protective	Diabetic Retinopathy	37037887	PDLIM1 inhibits cell migration and invasion in diabetic retinopathy via negatively regulating Wnt3a - PMC (nih.gov)	
MEN1	no	no	no						
SUPT5H	no	yes	yes	Down		Hypertension	37660920	https://www.ncbi.nlm.nih.gov/pmc/articles/PMC10558802/	

MATN2	yes	maybe	maybe	Down				Nidogen-2 Maintains the Contractile Phenotype of Vascular Smooth Muscle Cells and Prevents Neointima Formation via Bridging Jagged1-Notch3 Signaling | Circulation (ahajournals.org)	Possible regulator of VSMC phenotypic transition, PMID: 33441605 shows possible association with CVD
PSMD14	no	yes	yes	Up	Risk	Vascular Invasion		Deubiquitinase PSMD14 enhances hepatocellular carcinoma growth and metastasis by stabilizing GRB2 - ScienceDirect	
KRIT1	yes	yes	yes	Down/Silenced	Risk	Aortic Endothileal Dysfunction	31590384	KRIT1 Deficiency Promotes Aortic Endothelial Dysfunction - PMC (nih.gov)	

TOR1B	yes	maybe	maybe				27755386	Dissecting the genetic components of a quantitative trait locus for blood pressure and renal pathology on rat chromosome 3 - PMC (nih.gov)	Possible regulator of blood pressure, could be related to many vascular disesase
MGST3	no	no	no						
SPTBN2	maybe	no	no				37779199	Mettl1-mediated internal m7G methylation of Sptbn2 mRNA elicits neurogenesis and anti-alzheimer's disease - PMC (nih.gov)	No definite correlation, but likely related to iPSC differentiation and possibly vascular differentiation
BCAT2	no	no	no						
SLC31A1	no	yes	no	Up	Risk	Atherosclerosis	37442861	Copy of iPSC vs VSMC gene sex comparison_Liam_Male.xlsx (sharepoint.com)	Involved in cuproptosis mechanism
PHGDH	yes	no	yes			Angiogenesis defect related diseases		Serine Synthesis via PHGDH Is Essential for Heme Production in Endothelial Cells - ScienceDirect	

EEF1E1	no	no	yes	Up	Risk	Vascular Invasion	34282404	A Novel HCC Prognosis Predictor EEF1E1 Is Related to Immune Infiltration and May Be Involved in EEF1E1/ATM/p53 Signaling - PMC (nih.gov)	
MYO1B	yes	no	no				36654782	Myo1b Promotes Premature Endothelial Senescence and Dysfunction via Suppressing Autophagy: Implications for Vascular Aging - PMC (nih.gov)	Inovolved in angiogenesis and vascular senescence

STRN	yes	no	yes	Down	Risk	CAD	31821324	RNA-sequencing reveals that STRN, ZNF484 and WNK1 add to the value of mitochondrial MT-COI and COX10 as markers of unstable coronary artery disease - PubMed (nih.gov)	This article shows vascular function https://pubmed.ncbi.nlm.nih.gov/26169051/
PPL	no	no	no						
ABCC9	yes	no	yes			LOF associated with various diseases	35588738	KCNJ8/ABCC9-containing K-ATP channel modulates brain vascular smooth muscle development and neurovascular coupling - PubMed (nih.gov)	This article describes LOF related diseases https://www.ncbi.nlm.nih.gov/pmc/articles/PMC9044080/
SNX2	no	no	maybe				30877150	The emerging role of sorting nexins in cardiovascular diseases - PMC (nih.gov)	Involved in endosomal sorting processes, may regulate various cardiovascular diseases
MACROH2A1	no	no	no						
LRCH4	no	no	no						
RP2	no	no	no						
WDHD1	no	no	no						

SLC25A12	yes	yes	yes			Various diseases associateed with vascular senescence		Capsaicin inhibits A7r5 cell senescence via the mitochondrial carrier protein Slc25a12 - ScienceDirect	
MPDZ	yes	no	yes		Protective	Tumor angiogenesis	29620522	MPDZ promotes DLL4-induced Notch signaling during angiogenesis - PMC (nih.gov)	Involved in angiogenesis
UBXN7	no	yes	no		Risk	Cerebral Atherosclerosis	32424284	Shared proteomic effects of cerebral atherosclerosis and Alzheimer's disease on the human brain - PMC (nih.gov)	
PAPSS2	no	no	maybe	Down			34572333	Long Term Response to Circulating Angiogenic Cells, Unstimulated or Atherosclerotic Pre-Conditioned, in Critical Limb Ischemic Mice - PMC (nih.gov)	Hard to tell if there is a relation, need to look deeper into this article

SGPL1	maybe	no	no			Possibly associated with angiogenesis according to the article	28181337	Deficiency of the sphingosine-1-phosphate lyase SGPL1 is associated with congenital nephrotic syndrome and congenital adrenal calcifications - PMC (nih.gov)	
ASMTL	yes	no	likely			Probably Vascular Invasion		[illegible] acid-downregulated long noncoding RNA ASMTL-AS1 inhibits renal cell carcinoma growth via binding to HuR and reducing vascular endothelial growth factor expression - Cai - 2023 - Journal of Biochemical and Molecular Toxicology - Wiley Online	Regulates VEGF expression
GOT2	no	no	no						

ASS1	likely	no	likely				21106532	Endothelial Argininosuccinate Synthetase 1 Regulates Nitric Oxide Production and Monocyte Adhesion under Static and Laminar Shear Stress Conditions - PMC (nih.gov)	Suggested to be important in pathway for endothileal vascular health
GBA1	no	no	no						
ATP1B1	no	no	yes		Risk	CAD	32591598	Prioritization of causal genes for coronary artery disease based on cumulative evidence from experimental and in silico studies - PMC (nih.gov)	

ALPL	no	yes	no	Up	Risk	Vascular Calcification (hypertension, atherosclerosis, etc.)	37080965	Signaling pathways in vascular function and hypertension: molecular mechanisms and therapeutic interventions - PMC (nih.gov)	
PYGL	yes	yes	no			Hypertension	35611851	miR-155-5p regulates hypoxia-induced pulmonary artery smooth muscle cell function by targeting PYGL - PMC (nih.gov)	Need to dive deeper into this article to find out if it is a risk or protective gene
TPM3	yes	yes	no	Up	Risk	Hypertension	33341060	Physical interaction of tropomyosin 3 and STIM1 regulates vascular smooth muscle contractility and contributes to hypertension - ScienceDirect	
EPRS1	no	no	no						
KRT19	no	no	no						

UCHL1	yes	no	yes		Risk	Hypertension	34130526	Long-term inhibition of UCHL1 decreases hypertension and retinopathy in spontaneously hypertensive rats - PMC (nih.gov)	
ALDOC	no	no	no				35900662	Unbiased proteomic analysis of extracellular vesicles secreted by senescent human vascular smooth muscle cells reveals their ability to modulate immune cell functions - PMC (nih.gov)	Glycolysis related enzyme, upregulated in senescent VSMCs
TXN	no	no	yes		Risk	Tumor Angiogenesis	34433833	Expression of TXNIP is associated with angiogenesis and postoperative relapse of conventional renal cell carcinoma - PMC (nih.gov)	
PC	no	no	no						

CKB	no	no	yes	Up		Ischemic Stroke	6487101	Serum creatine kinase B concentrations in acute cerebrovascular diseases - PubMed (nih.gov)	
MYL4	yes	yes	yes	up	Risk	HCM,IHD, DCM	31481666	Human cardiac myosin light chain 4 (MYL4) mosaic expression patterns vary by sex - PMC (nih.gov)	Study on Expression differences based on sex
JUP	no	yes	yes		Risk	Atherosclerosis, CAD	23110151	Antibody Phage Display Assisted Identification of Junction Plakoglobin as a Potential Biomarker for Atherosclerosis - PMC (nih.gov)	
RPS2	no	no	no						
SMPD1	no	no	no						
IGFBP2	no	no	yes	Up	Risk	PAD	24548188	IGF-I and IGFBP2 in peripheral artery disease: results of a prospective study - PubMed (nih.gov)	
SRM	no	no	no						
LMNB1	no	no	no						
MMUT	no	no	no						

RPL13	Probably	no	yes		Protective	Congenital Heart Disease	31625562	Model system identification of novel congenital heart disease gene candidates: focus on RPL13 - PMC (nih.gov)	
VARS1	no	no	no						
AK4	yes	no	no				34638712	Adenylate Kinase 4—A Key Regulator of Proliferation and Metabolic Shift in Human Pulmonary Arterial Smooth Muscle Cells via Akt and HIF-1α Signaling Pathways - PMC (nih.gov)	Metabolic Regulator for PASMCs
DPP4	yes	yes	yes		Risk	Any disease associated with vascular aging	34489872	https://www.ncbi.nlm.nih.gov/pmc/articles/PMC8416540/#:~:text=DPP4%20inhibitors%20play%20a%20beneficial,AP%2DI%20and%20NF%2DKB	

CAD	yes	no	no				14718356	https://www.ahajournals.org/doi/10.1161/01.HYP.0000112424.06921.52?url_ver=Z39.88-2003&rfr_id=ori:rid:crossref.org&rfr_dat=cr_pub%20%200pubmed	Involved in vasodilation mechanism
TKT	no	no	yes			Congenital Heart Disease	27259054	https://www.ncbi.nlm.nih.gov/pmc/articles/PMC4908149/	Mutation based disease
CCND2	no	no	no				29018036	https://www.ncbi.nlm.nih.gov/pmc/articles/PMC5756126/	Overexpression of genee enhances regenerative potency of cardiomyocyte iPSCs
SLC7A1	no	no	yes		Mutation/Risk	Hypertension	17325243	https://www.ahajournals.org/doi/10.1161/CIRCULATIONAHA.106.665836	
L1CAM	yes	no	yes		Protective	Various Cancers	28134764	https://www.ncbi.nlm.nih.gov/pmc/articles/PMC5343790/	
GALNS	no	no	no						

IRS1	no	no	no					https://www.jbc.org/article/S0021-9258(20)36725-9/pdf#:~:text=During%20normoglycemia%2C%20IGF%2DI%20signaling,vascular%20smooth%20muscle%20cell%20differentiation.	Can affect pathway of VSMC migration and proliferation
DEK	no	no	yes	Up	Risk	Tumor angiogenesis		https://www.ncbi.nlm.nih.gov/pmc/articles/PMC5029660/	
NUDT1	yes	yes	yes		Protective	Pulmonary Arterial Hypertension	33021405	https://pubmed.ncbi.nlm.nih.gov/33021405/	Can be seen as risk for cancer, as it activates PSMC proliferation
LONP1									
COL15A1	no	no	yes	Dysfunction	Risk	Various diseases	36978846	https://www.ncbi.nlm.nih.gov/pmc/articles/PMC10045650/#:~:text=In%20mitochondria%2C%20Lonp1%20(i),iv)%20regulates%20stress%20response%20by	Gene for Cardiac Mitochondria, influences heart development

CAPG	no	no	yes	Up	Risk	Hypoxia-Induced Pulmonary Hypertension	27093378	https://pubmed.ncbi.nlm.nih.gov/27093378/	
MTOR	yes	no	yes			Alzheimers		https://www.sciencedirect.com/science/article/pii/S092544391500349X#s0045	Involved in VSMC proliferation, and various other vascular pathways
GATA4	yes	no	no				17975667	https://pubmed.ncbi.nlm.nih.gov/17975667/#:~:text=Abstract,nodal%20regulator%20of%20cardiac%20angiogenesis.	
MKI67	no	no	no						
NSF	no	no	no						
GNPDA1	no	no	no						
PLA2G4A	no	no	yes		Protective	CAD, Myocardial Infarction	31092728	https://insight.jci.org/articles/view/125638	Gene variant associated with protective influence on CAD, MI
CAPZB	no	no	no						Associated with actin binding, maybe loose VSMC association
GCLC	yes	yes	yes		Protective	Atherosclerosis	18035085	https://www.sciencedirect.com/science/article/pii/S000287030700612 6?via%3Dihub	
ADGRE5	no	no	no						
PSMB2	no	no	no						

VPS41	no	no	no						
NUDT2	no	no	no						
NUP98	no	no	no						
YARS1	no	no	no						
ALDH18A1	no	no	no						
ADK	yes	yes	yes		Risk	Potentially Atherosclerosis and many other heart diseases	32352607	https://www.ncbi.nlm.nih.gov/pmc/articles/PMC7752304/#:~:text=Our%20results%20indicate%20that%20ADK,under%20metabolic%20and%20hemodynamic%20stress.	
RRP1	no	no	yes	Mutation/ LOF	Protective	Lyme Disease	37874144	https://www.ncbi.nlm.nih.gov/pmc/articles/PMC10652863/	Involved in BBB vasculature
RPS3A	no	yes	yes	Down	Risk	CAD, Atherosclerosis	30131868	https://www.nature.com/articles/s41421-018-0041-2	
RPS8	no	no	no				24879686	https://www.ncbi.nlm.nih.gov/pmc/articles/PMC4988620/	Differential Expression in Animal Study on Atherosclerosis, no other vascular relation
RPS18	no	no	no						

RPL7A	no	yes	no	Down	Risk	Aortic Aneurysm	34355024	Frontiers | Bioinformatics Analysis Reveals the Potential Diagnostic Biomarkers for Abdominal Aortic Aneurysm (frontiersin.org)	
RPL32	no	no	no						
MRPS5	yes	no	no					Reduced Mitochondrial Protein Translation Promotes Cardiomyocyte Proliferation and Heart Regeneration | Circulation (ahajournals.org)	Mitochondrial protein, associated with cardiomyocyte proliferation and heart regeneration
RPL24	no	no	no						
RPL19	no	no	no						
ANK2	maybe	no	yes			Various Cardiovascular Diseases		The Evolving Role of Ankyrin-B in Cardiovascular Disease - PMC (nih.gov)	Novel association with vascular function, loosely correlated to cerebral aneursym incedence
LRP1	yes	yes	no		Protective	Atherosclerosis	24504736	LRP1: Role in the regulation of vascular integrity - PMC (nih.gov)	

CRYZ	no	no	no						
FUT2	yes	no	no				23065099	A novel role for inducible Fut2 in angiogenesis - PMC (nih.gov)	Regulates activity of angiogenesis pathway, unknown if related to diseases
FSTL1	yes	no	no				18718903	Follistatin-like 1, a Secreted Muscle Protein, Promotes Endothelial Cell Function and Revascularization in Ischemic Tissue through a Nitric-oxide Synthase-dependent Mechanism - PMC (nih.gov)	Follastin protein associated with vasculature remodeling, therefore likely related to diseases
TRAF2	no	yes	no	Up	Risk	Atherosclerosis	35252400	Frontiers | The Role of Tumor Necrosis Factor Associated Factors (TRAFs) in Vascular Inflammation and Atherosclerosis (frontiersin.org)	

ACACA	no	no	yes			EC migration-related diseases	29208696	Acetyl-CoA carboxylase 1 regulates endothelial cell migration by shifting the phospholipid composition - PMC (nih.gov)	Enzyme that catalyzes the rate limiting step of FA synthesis, associated with
IK	no	no	no						
TUBB3	no	no	no						
ATR	yes	no	no				8213165	[Effects of atropine on contraction of rabbit aortic smooth muscle and cell proliferation] - PubMed (nih.gov)	Kinase associated with proliferation checkpoint. Affects aortic muscle contraction.
ARHGEF7	yes	yes	no		Protective	Atherosclerosis	35047576	Frontiers | The Emerging Role of Rho Guanine Nucleotide Exchange Factors in Cardiovascular Disorders: Insights Into Atherosclerosis: A Mini Review (frontiersin.org)	
TTLL12	no	no	no						
DPYSL3	no	no	no						

ITPR2	yes	yes	yes		Protective	Hypertension	27777977	IP3 receptors regulate vascular smooth muscle contractility and hypertension - PMC (nih.gov)	Receptor family that mediates intracellular Ca release which regulates contractility and hypertension
ITPR1	yes	yes	yes						Same receptor family and links as above
CASP8	no	no	yes	Mutation	Risk	CAD	28633917	Polymorphic variants of Caspase genes (8 & 3) in the risk prediction of Coronary Artery Disease - PubMed (nih.gov)	Caspase gene, mutation associated with CAD
NCAPH	no	no	no						
NCAPD2	no	no	no						
QPRT	no	no	no						
DLGAP5	no	no	no						
TRIP10	no	no	no						
ELAVL1	yes	no	yes			CHD	36477660	Role of hsa_circ_0000280 in regulating vascular smooth muscle cell function and attenuating neointimal hyperplasia via ELAVL1 - PMC (nih.gov)	RNA binding protein linked to vascular remodeling

ETFDH	no	no	no						
DPYSL2	yes	no	no			Neurological Diseases	28413470	Identification of genes associated with the effect of inflammation on the neurotransmission of vascular smooth muscle cell - PubMed (nih.gov)	Collapsin response mediator protein associated with inflammation response in VSMCs
PCK2	yes	yes	no		Protective	Atherosclerosis	35982907	Role of PCK2 in the proliferation of vascular smooth muscle cells in neointimal hyperplasia - PMC (nih.gov)	Gluconeogenesis gene associated with vascular remodeling in VSMCs (associated with atherosclerosis)
LRRFIP1	yes	maybe	no					The microRNA miR-132 targets Lrrfip1 to block vascular smooth muscle cell proliferation and neointimal hyperplasia - ScienceDirect	Transcription inhibitor gene that is associated with blocking VSMC proliferation, not sure if associated with diseases
LRRK1	no	no	no						
TKFC	no	no	no						
PARP14	no	no	no						

PDCD4	yes	likely	no	down	risk	Atherosclerosis	20357187	https://www.ncbi.nlm.nih.gov/pmc/articles/PMC2889634/	Tumor supressor gene that has been studied on VSMCs
NOM1	no	no	no						
CRB2	no	no	no						
NIPBL	no	no	no						
ZCCHC8	no	no	no						
RPL22L1	no	no	no						
PGM2L1	no	no	no						
SKIC3	no	no	no						
ULK3	no	no	no						
CSPG4	no	no	maybe					https://www.nature.com/articles/s41598-021-85335-x	Proteoglycan that is regulated by myocardin trans factors
CD109	yes	yes	no			vascular fibrosis	36909248	https://www.ncbi.nlm.nih.gov/pmc/articles/PMC9998030/	Glycoprotein whose regulation affects TGF-B pathway (inhibits it)
SYNE3	maybe	maybe	maybe			atherosclerosis/hypertension		https://www.ahajournals.org/doi/full/10.1161/ATVBAHA.117.310237	Involved in cytoskeletal organization, linked to arterial stiffness
CFAP47	no	no	no						
CCDC80	maybe	yes	no	Up	risk	atherosclerosis	30439364	https://www.sciencedirect.com/science/article/pii/S0014299183065 99?via%3Dihub	Function of this gene is somewhat unknown, but associated with vasculature remodeling and accelerating atherosclerosis
MAVS	no	no	no						
PATL1	no	no	no						
ANKMY2	no	no	no						

AFAP1L2	no	no	no	Up (in cell proliferation)			33340241	https://www.ncbi.nlm.nih.gov/pmc/articles/PMC7812284/	Gene may just be associated with cell proliferation, but significant fold change in hypoxic PASMC
DIPK2A	no	no	no						Gene associated with cardiac muscle cell proliferation
CYP2W1	no	no	no						Theorized that CYP450 family is associated with vascular cell proliferation
PIP4K2C	no	no	yes	down	risk	cardiac fibrosis, cardiac hypertrophy	34026458	Therapeutic Delivery of Pip4k2c-Modified mRNA Attenuates Cardiac Hypertrophy and Fibrosis in the Failing Heart - PMC (nih.gov)	Possible inhibitor of cardiac fibrosis and hypertrophy

TRPM4	indirectly	no	yes				23443854	https://www.ncbi.nlm.nih.gov/pmc/articles/PMC3686874/	Gene for a Ca2+ ion channel, Ca2+ overload associated with several heart diseases (CAD, heart failure, etc.) as Ca2+ is responsible for contractions
IFT81	yes	no	no						Associated with vascular integrity (tubulin tranport)
NSMAF	no	no	no						
GCDH	no	no	no						
RIT1	no	no	no	mutation	risk	noonan syndrome, various CV diseases	27101134	https://www.sciencedirect.com/science/article/pii/S109836002101412X#:~:text=RIT1%20is%20one%20of%20the,hypertrophic%20cardiomyopathy%2C%20and%20lymphatic%20problems.	GTPase asspcoated with Noonan Syndrome, and some cardiovascular connections
TNPO1	maybe	yes	no	Up	risk	atherosclerosis	32714363	https://www.ncbi.nlm.nih.gov/pmc/articles/PMC7344232/	Nuclear localization signal gene; associated as a biomarker for atherosclerosis

ARHGEF2	no	no	no						Lots of articles referencing vasculature and this gene though, just no direct relation
MYDGF	no	yes	yes	down	risk	atherosclerosis	34020949	https://www.ncbi.nlm.nih.gov/pmc/articles/PMC8139583/	myeloid derived growth factor; deficiency associated with worsened atherosclerosis
ITFG2	no	no	yes	up	risk	MI	29876469	https://www.ncbi.nlm.nih.gov/pmc/articles/PMC5988399/	Integrin involved in AA starvation, upregulated in MI
TAMM41	no	no	no						
GALM	no	no	no						
HMCES	no	no	no						
PGM2	no	no	no						
BORCS6	no	no	no						
MASTL	no	no	no						
VPS39	no	no	no						
PPP1R10	no	sort of	sort of	Telomere length as a potential biomarker of coronary artery disease - PMC (nih.gov)		Gene for protein phosphatase that is associated with preventing cell senescence in VSMC and other vascular cells		https://pubmed.ncbi.nlm.nih.gov/29067974	

HHIP	no	yes	no	HHIPL1, a Gene at the 14q32 Coronary Artery Disease Locus, Positively Regulates Hedgehog Signaling and Promotes Atheroscl erosis - PMC (nih.gov)		Gene associated with hedgehog complex. Postive regulation of atherosclerosis			
USP28	no	no	maybe			Deubiquitinase associated with heart issues, but mostly secondary effecs from other non-heart diseases			
CYP2S1	no	yes	no	Role of retinoic acid metaboliz ing cytochro me P450s, CYP26, in inflammat ion and cancer - PMC (nih.gov)		Inhibition of this C450 enzyme has postive associations with atherosclerosis		https://pu bmed.ncb i.nlm.nih. gov/2623 3912	
VAT1	no	no	no						
TXNDC17	no	no	no						
AARSD1	no	no	no						

STRA6	yes	yes	no	STRA6 is essential for induction of vascular smooth muscle lineages in human embryonic cardiac outflow tract development - PubMed (nih.gov)		Signaling receptor and transporter of retinol, an important pathway for VSMC development		36635482	
MAK16	no	no	yes	Transcriptome alterations of vascular smooth muscle cells in aortic wall of myocardial infarction patients - PMC (nih.gov)		RNA binding protein, downregulated in MI		https://pubmed.ncbi.nlm.nih.gov/29876469	
NLN	maybe	yes	yes	The Interaction between 30b-5p miRNA and MBNL1 mRNA is Involved in Vascular Smooth Muscle Cell Differentiation in Patients with Coronary Atherosclerosis - PMC (nih.gov)		Metallopeptidase protein that cleaves a sequence in neurotensin, associated in several VSMC differentiation/proliferation systems that relate to atherosclerosis and MI		The Interaction between 30b-5p miRNA and MBNL1 mRNA is Involved in Vascular Smooth Muscle Cell Differentiation in Patients with Coronary Atherosclerosis - PMC (nih.gov)	

UACA	no	no	yes	https://www.ncbi.nlm.nih.gov/pmc/articles/PMC5988399/		Regulatory protein associated with apoptosis, upregulated in MI		29876469	
TRIM5	no	yes	yes	https://www.ncbi.nlm.nih.gov/pmc/articles/PMC6232936/		Gene encodes ubiquitin ligase, possible target for CAD		30460244	
TRIM2	no	no	no						
TANGO6	no	no	no						
TNKS1BP1	no	no	yes	Single-cell transcriptomics reveals zinc and copper ions homeostasis in epicardial adipose tissue of heart failure - PMC (nih.gov)		Ankyrin associated gene, altered regulation in heart failure		https://pubmed.ncbi.nlm.nih.gov/37705737	
WDR12	no	yes	yes	Abnormal Ribosome Biogenesis Partly Induced p53-Dependent Aortic Medial Smooth Muscle Cell Apoptosis and Oxidative Stress - PMC (nih.gov)		Gene involved in rRNA maturation, implications on VSMC apoptosis		https://pubmed.ncbi.nlm.nih.gov/31210846	

SRR	no	no	yes	Genetic Insights Into Smooth Muscle Cell Contributions to Coronary Artery Disease - PMC (nih.gov)		Candidate CAD gene, gene associated with lyase and other various proteins		https://pubmed.ncbi.nlm.nih.gov/31043074	
QRSL1	no	yes	no	Abnormal Endothelial Gene Expression Associated With Early Coronary Atherosclerosis - PMC (nih.gov)		Amidotransferase associated gene, differential expression in healthy vs early atherosclerosis subjects		https://pubmed.ncbi.nlm.nih.gov/32673514	
CPVL	no	yes	no	Identification of PTPN22 as a potential genetic biomarker for abdominal aortic aneurysm - PMC (nih.gov)		Carboxypeptidase that is a possible biomarker for abdominal aortic aneurysm		https://pubmed.ncbi.nlm.nih.gov/36588574	
OSGEPL1	no	no	no						
TUT1	no	no	no						
WDR55	no	no	no						
METTL7A	no	no	no						
WDR41	no	no	no						
RIC8A	no	no	no						
SLC17A5	no	no	no						
HEBP1	no	no	yes	https://www.ncbi.nlm.nih.gov/pmc/articles/PMC9262492/		Heme binding protein that promotes endothelial angiogenesis		35796141	
FARSB	no	no	no						
MAN2C1	no	no	no						
SPATS2L	no	no	no						
UFSP2	no	no	no						
ABCF3	no	no	no						

SPTLC3	no	no	yes	https://scholarscompass.vcu.edu/etd/7287/				https://doi.org/10.25772/WJZA-Z415	
BABAM2	no	no	no						
PLEK2	no	no	yes	https://www.ncbi.nlm.nih.gov/pmc/articles/PMC5749534/		Gene associated with cell spreading, downregulated effector in a pathway that results in vascular occlusion		29202466	
SMARCAL1	no	no	maybe			Lots of small references to this gene being associated with elastin production, therefore loosely related to many vascular diseases			
FLRT3	no	maybe	yes	https://www.ncbi.nlm.nih.gov/pmc/articles/PMC6373724/		SNP in this gene is associated with atherosclerotic cerebovascular disease		20425258	
MACROH2A2	no	no	no						
**LARS1**	no	no	no						
GMPR2	no	no	no						
MORF4L1	no	no	yes	down	risk	CAD	36139449	https://www.ncbi.nlm.nih.gov/pmc/articles/PMC9496853/	36139449
TJP2	yes	no	no				https://pubmed.ncbi.nlm.nih.gov/19380416/		19380416
BAZ2A	no	no	no						
FBXO3	no	yes	yes		risk	Atherosclerosis	30448480	https://www.ncbi.nlm.nih.gov/pmc/articles/PMC7425077/	30448480

PCOLCE2	no	yes	yes	down	protective	Atherosclerosis	34551590	https://www.ahajournals.org/doi/10.1161/ATVBAHA.121.316615	
NOB1	no	no	yes	up	risk	endothileal cancer	https://www.ncbi.nlm.nih.gov/pmc/articles/PMC6491816/		
SUFU	no	maybe	maybe			Possibly Atherosclerosis	https://www.ncbi.nlm.nih.gov/pmc/articles/PMC7640405/		33143723
PACSIN2	no	no	function, not disease				https://www.ncbi.nlm.nih.gov/pmc/articles/PMC9285741/		34990060
FAF1	no	no	no						
PROCR	no	maybe	yes	down	protective	CAD	35264566	https://pubmed.ncbi.nlm.nih.gov/35264566/	
FZD1	yes	maybe	maybe				https://pubmed.ncbi.nlm.nih.gov/15923619/	https://academic.oup.com/cardiovascres/article/95/2/233/293764	15923619
AGTPBP1	kind of	no	no				https://www.ncbi.nlm.nih.gov/pmc/articles/PMC3840609/		
FARSA	maybe	no	no				https://www.ncbi.nlm.nih.gov/pmc/articles/PMC7176856/		32155686
MAN2B2	no	no	no						

TDRKH	yes	yes	no	down	risk	CAD	35735005	Effects of Coronary Artery Disease–Associated Variants on Vascular Smooth Muscle Cells | Circulation (ahajournals.org)	Involved in RNA binding activity, associated with CAD and VSMC proliferation
YARS2	yes	no	yes			Various CVDs	The regulatory roles of aminoacyl-tRNA synthetase in cardiovascular disease - ScienceDirect		Catalyzes tyrosine binding to tRNA, possible biomarker for CVDs
FBXO7	no	no	no						Associated with FBOX subunit in ubiquitination pathway
RPL36	no	no	no						Encodes part of the ribosomal 60S subunit
CHST2	no	no	no						Enzyme catalyzes sulfation of a specific sugar, expressed by vascular endothileal cells, could be important in antibody mechanism, associated with non CVD diseases

ASB3	no	no	no						Ankyrin repeat, associated with hepatocellular carcinoma
GTF3C3	no	no	no						
BZW2	no	no	no						

**Supplementary Table S7.** Proteins Upregulated in Female iVSMCs								
	Related to Vascular function	Related to vascular disease	related to vascular disease in another cell type	Up or Down	Protective vs Risk?	Disease?	PMID	source
**Up in female**								
iVSMCs								
SBNO1	no	no	no					
VWA5A	no	no	yes	Upregulated	Risk	Atherosclerosis	38292008	https://www.ncbi.nlm.nih.gov/pmc/articles/PMC10825564/
NME4	no	no	no					
PLPP3	yes	yes	no	Downregulated	Protective	aortic aneurysm and transmural rupture	35383201	https://www.ncbi.nlm.nih.gov/pmc/articles/PMC8983654/#:~:text=Loss%20of%20Plpp3%20in%20smooth,stimulates%20aortic%20aneurysm%20formation27
DCLK1	no	no	yes	Upregulated	Risk	Atherosclerosis	36896602	https://www.embopress.org/doi/full/10.15252/emmm.202217198
ARPC3	no	no	no					
TXNL1	no	no	no					
NMT2	no	no	no					

ICMT	yes	yes	no	Mutation / Downregulated	Protective	HGPS, increase cell proliferation	33526168	https://elifesciences.org/articles/63284
SLIT3	yes	no	no	?	?		19741192	https://www.ncbi.nlm.nih.gov/pmc/articles/PMC2774558/
ECI2	no	no	yes	Upregulated	Risk	aneurysm	37827202	https://www.sciencedirect.com/science/article/pii/S0925443923002818
ERAL1	no	no	no					
CIAO1	no	no	no					
PLPBP	no	no	no					
TNFAIP8	no	no	no					
SVIL	no	no	yes	Upregulated	Risk	Coronary artery/ microvascular dysfunction	38776872	
CAVIN2	no	no	yes	Upregulated	Risk	disturbed flow	34282126	https://www.nature.com/articles/s41420-021-00567-0
EGFR	no	no	no					
FGA	no	no	no					
FGB	no	no	no					https://bmcmedgenet.biomedcentral.com/articles/10.1186/1471-2350-11-28

FGG	no	yes	yes	Upregulated	Risk	Aortic dysfunction, Hypertension	33304927	https://www.frontiersin.org/articles/10.3389/fcvm.2020.581362/full
**ARG1**	no	no	yes	Upregulated; Downregulated	Risk	coronary artery disease	30526064; 36252109	https://pubmed.ncbi.nlm.nih.gov/30526064/
**BCHE**	no	no	yes	Upregulated; Downregulated	Risk	coronary artery disease; hypertension	12387587	https://pubmed.ncbi.nlm.nih.gov/12387587/
EPHX1	no	no	no					
LAMC1	yes	no	no	Upregulated	Risk		33230159	https://www.ncbi.nlm.nih.gov/pmc/articles/PMC7683538/
ALAD	no	no	no					
VCL	yes	no	yes	Downregulated	Risk	Atherosclerosis	38287421	https://www.ncbi.nlm.nih.gov/pmc/articles/PMC10826019/
HK1	no	no	yes	Downregulated	Risk	Coronary Artery disease	26351984	https://www.ncbi.nlm.nih.gov/pmc/articles/PMC4651403/
SLC9A1	yes	no	yes	Upregulated	Risk	Hypertension	34440087	https://www.mdpi.com/2227-9059/9/8/883

TGM2	yes	no	no	Downregulated	Protective	Hypertension	34326316	https://www.ncbi.nlm.nih.gov/pmc/articles/PMC8322091/
ENPP1	yes	no	no	Mutation		Hypertension	36937905	https://www.frontiersin.org/articles/10.3389/fcvm.2023.1105381/full#:~:text=Generalized%20arterial%20calcification%20of%20infancy%20(GACI)%20is%20a%20rare%20genetic,intrauterine%20or%20early%20infancy%20death.
SFPQ	yes	no	no				28743736	https://www.ncbi.nlm.nih.gov/pmc/articles/PMC5548871/
PTPRD	no	no	no					

IGFBP5	no	no	yes	Upregulated	Risk	Coronary artery Disease	32194505	https://www.ncbi.nlm.nih.gov/pmc/articles/PMC7063065/#:~:text=IGFBP%2D5%20may%20play%20a,mortality%20in%20humans%20(136).
MYL9	yes	no	no	Downregulated			34012019	https://www.nature.com/articles/s41598-021-90119-4
CANX	no	yes	no	Upregulated	Risk	Atherosclerosis	22582288	https://www.sciencedirect.com/science/article/pii/S0032579119402496
**SMARCA1**	no	no	no					
CCN2	yes	yes	no	Downregulated	Risk	aneurysm	36625347	https://insight.jci.org/articles/view/162987
EPHA3	no	no	no					
LMOD1	yes	yes	no	Downregulated	Risk	Coronary Artery Disease	30444878	https://pubmed.ncbi.nlm.nih.gov/30444878/

**TSPO**	yes	yes	no	Upregulated	Risk	Atherosclerosis	31515530	https://www.ncbi.nlm.nih.gov/pmc/articles/PMC7471478/#:~:text=These%20results%20suggest%20that%20TSPO,diseases%20including%20atherosclerosis%20and%20restenosis.
HOXA10	no	no	no					
PRDX2	yes	yes	no	Upregulated	Protective	Athersclerosis	33791345	https://www.frontiersin.org/articles/10.3389/fcvm.2021.624796/full#:~:text=10.3389%2Ffcvm.2021.624796-,PRDX2%20Protects%20Against%20Atherosclerosis%20by%20Regulating%20the%20Phenotype%20and,the%20Vascular%20Smooth%20Muscle%20Cell

STAT3	yes	no	no	Upregulated	Risk	Vascular Injury - neointimal lesions	22418922	https://www.ncbi.nlm.nih.gov/pmc/articles/PMC3350628/
CETN2	no	no	no					
PSMC4	no	no	no					
TNNT2	yes	yes	no	Upregulated	Risk	Aortic Aneurysm and Dissection	37555319	https://www.ahajournals.org/doi/10.1161/CIRCULATIONAHA.123.063332
MAPK8	no	yes	no	Downregulated	Risk	Ischemia	27504807	https://elifesciences.org/articles/18414
NES	yes	yes	no				19896481	https://pubmed.ncbi.nlm.nih.gov/19896481/
RPIA	no	no	no					
CEBPD	yes	yes	no	Upregulated	Risk	Atherosclerosis	25614278	https://www.ahajournals.org/doi/10.1161/atvbaha.114.305218
**SULT1E1**	yes	no	no	Upregulated	Risk	Atherosclerosis	23685729	https://www.spandidos-publications.com/10.3892/mmr.2013.1483

MTHFS	no	no	yes	Upregulated	Protective	CVD		https://www.sciencedirect.com/science/article/pii/S0022316622027493
TPMT	no	no	no					
ALDH3A2	yes	yes	no	Upregulated	Risk	Atherosclerosis	36224302	https://www.ncbi.nlm.nih.gov/pmc/articles/PMC9556750/
CNN1	yes	yes	no	Upregulated	Protective?	Healthy vsmcs	36411459	https://biosignaling.biomedcentral.com/articles/10.1186/s12964-022-00993-2
MAP2K6	yes	no	no	Downregulated	Risk?	Atherosclerosis	23175675	https://www.ahajournals.org/doi/full/10.1161/atvbaha.112.300645
AGFG1	no	yes	no	Upregulated	Risk	Aortic Dissection	35178459	https://www.hindawi.com/journals/jir/2022/7585149/
HK2	yes	no	no				36370492	https://pubmed.ncbi.nlm.nih.gov/36370492/

CTSC	yes	yes	no	Mutation/ Downregulation	Protective/reduced risk	Atherosclerosis	31926332	https://www.ncbi.nlm.nih.gov/pmc/articles/PMC7029168/
HCCS	no	no	no					
ATP1B3	no	no	yes	Upregulated	Risk	Aneurysm	36172868	https://www.ahajournals.org/doi/10.1161/ATVBAHA.122.317953
MANF	yes	yes	no	Upregulated	Risk?		38145449	https://assets.researchsquare.com/files/rs-3169400/v1/6cc40fcc-589a-4c7a-9aeb-ea511492b819.pdf?c=1704121529
ATP5MF	no	no	yes	Upregulated	Risk?	Coronary Artery Disease?	6433946	https://www.nature.com/articles/s44161-023-00312-z
RAB15	yes	yes	no	Upregulated	Risk	Atherosclerosis	36231021; 26305474	https://www.ncbi.nlm.nih.gov/pmc/articles/PMC9563587/
PRPS1	no	no	no					

RAB5B	no	no	yes	Upregulated	Protective	Atherosclerosis, Hypertension	30333257	https://www.ncbi.nlm.nih.gov/pmc/articles/PMC6328933/
UBE2M	no	no	no					
VBP1	no	no	yes	Downregulated	Protective	Coronary vascular dysfunction	23685742	https://doi.org/10.1161/circulationaha.112.001238
PSMC1	no	no	yes	Upregulated?	Risk	Coronary artery disease?	26986213	https://www.ncbi.nlm.nih.gov/pmc/articles/PMC4795702/
SNRPD1	no	no	yes	Downregulated	Protective	aneurysm	37827202	https://www.sciencedirect.com/science/article/pii/S0925443923002818
SNRPD2	no	no	yes	Upregulated	Risk	Cornary Artery Disease	32981416	https://www.ncbi.nlm.nih.gov/pmc/articles/PMC7641189/
RPS4X	no	yes	no	Upregulated	Risk	Myocardial Infarction	29876469	https://www.ncbi.nlm.nih.gov/pmc/articles/PMC5988399/
RELN	no	no	yes	Upregulation	Risk	Atherosclerosis	26980442	https://pubmed.ncbi.nlm.nih.gov/26980442/
MRPS36	no	no	no					

TFAM	yes	no	no	Upregulated	Risk	Vascular disease	36834896; 36312244	https://www.ncbi.nlm.nih.gov/pmc/articles/PMC9961025/
CDK16	no	no	no					
XPC	no	no	no					
OCRL	maybe	no	no				33738288	https://www.ncbi.nlm.nih.gov/pmc/articles/PMC7960786/
KMT2A	no	no	no					
SSBP1	no	no	no					
EEF1A2	no	no	no					
KHDRBS1	no	no	no					
TFCP2	no	no	yes	Downregulated	Risk	Coronary Artery Disease	36889372	https://www.sciencedirect.com/science/article/abs/pii/S1567133X23000054
BPTF	no	no	no					
ANK3	no	no	yes					
ILK	yes	yes	no				19262169	https://www.ncbi.nlm.nih.gov/pmc/articles/PMC2679879/
LSAMP	yes	yes	no	Mutation	Risk	Coronary Artery Disease	18318786	https://www.ncbi.nlm.nih.gov/pmc/articles/PMC2652886/
DDX39B	no	no	no					

BLMH	no	yes	no				28413470	https://www.ncbi.nlm.nih.gov/pmc/articles/PMC5377265/
CRMP1	yes	no	no				28413470	https://pubmed.ncbi.nlm.nih.gov/28413470/
PGM5	no	no	yes			Atherosclerosis	28740544	https://www.ncbi.nlm.nih.gov/pmc/articles/PMC5505053/
RSU1	no	no	no					
SF1	no	no	no					
TRIP6	no	no	no					
LAMA4	no	no	no					
ATP6V1F	no	no	no					
**IAH1**	no	no	yes	Downregulated	Protective	Aneurysm	35280433	https://www.ncbi.nlm.nih.gov/pmc/articles/PMC8914561/
FILIP1L	no	no	no					
**KLHL22**	no	no	yes	Mutation	Risk	Coronary artery disease	35959094	https://www.sciencedirect.com/science/article/pii/S2666602222000696
RABL3	no	no	no					
**TJAP1**	no	yes	no	Upregulated, downregulated	Risk	Myocardial infarction	29876469; 28396569	https://www.ncbi.nlm.nih.gov/pmc/articles/PMC5988399/
CEP78	no	no	no					

BRMS1L	no	no	yes			Hypertension?	33568719	https://www.ncbi.nlm.nih.gov/pmc/articles/PMC7875986/
DDX59	no	no	yes	Downregulated	Risk	Coronary artery disease; Abnormal vascular development	30444878; 32591598	https://www.ncbi.nlm.nih.gov/pmc/articles/PMC6268002/
SPOUT1	no	no	no					
CAMSAP1	no	no	no					
LRCH2	no	no	no					
MBNL2	yes	no	no				27317697	https://www.ncbi.nlm.nih.gov/pmc/articles/PMC5062968/
TBC1D9	no	no	yes	*Downregulated*	*Protective*	*Atherosclerosis*	34712662	https://www.ncbi.nlm.nih.gov/pmc/articles/PMC8546328/
ATG9A	yes	yes	no	Upregulated	Protective	Atherosclerosis	35090378	https://pubmed.ncbi.nlm.nih.gov/35090378/
FILIP1	no	no	no					
CENPV	no	no	no					https://www.ncbi.nlm.nih.gov/pmc/articles/PMC9323755/
THOC6	no	no	no					
ERO1B	no	no	no					

ARMC8	no	no	yes			Atherosclerosis	31539914	https://www.ncbi.nlm.nih.gov/pmc/articles/PMC6698662/
GSPT2	no	no	no					
PHACTR4	yes	no	yes		Protective	Atherosclerosis	37225873; 25143458	https://www.ncbi.nlm.nih.gov/pmc/articles/PMC10206587/
NECAP1	no	no	yes	Upregulated	Risk	Coronary Artery Disease	34565095	https://www.imrpress.com/journal/RCM/22/3/10.31083/j.rcm2203103/htm
LPCAT1	no	yes	no		Risk	coronary artery disease	32396387	https://www.ahajournals.org/doi/10.1161/CIRCGEN.119.002806
PCDH19	no	no	no					
KLHDC4	no	no	yes			Cardiac allograft vasculopathy		https://www.amjtransplant.org/article/S1600-6135(24)00069-8/fulltext
URGCP	no	no	no					
PPIL4	no	no	no					
OSCP1	no	no	yes			Peripheral artery disease	34712662	https://pubmed.ncbi.nlm.nih.gov/26690514/
SCFD1	no	no	no					

ARAP3	yes	no	no				35427166	https://www.science.org/doi/10.1126/sciadv.abm3471
RUFY2	no	no	no					
SEPTIN8	no	no	no					
PXDN	yes	no	yes	Downregulated	Protective	Atherosclerosis	35219848	https://www.sciencedirect.com/science/article/pii/S089158492200079X?via%3Dihub
GGH	no	no	yes	Mutation	Protective	Cardiovascular disease	22649255	https://pubmed.ncbi.nlm.nih.gov/22649255/
RBPMS	yes	no	no				31283468	https://www.ncbi.nlm.nih.gov/pmc/articles/PMC6613909/
PHKB	maybe	maybe	no				37476587	
EVI5L	no	no	no					
SNRNP40	no	no	no					
NXPE2	no	no	no					
SEH1L	no	no	no					
CYFIP2	no	no	no					
SMARCD1	yes	yes	no	Upregulated	Risk	Aneurysm	32787523	https://pubmed.ncbi.nlm.nih.gov/32787523/
BTF3L4	no	no	yes					https://www.ncbi.nlm.nih.gov/pmc/articles/PMC8469527/

USP47	yes	no	yes			Atherosclerosis	35509185	https://www.ncbi.nlm.nih.gov/pmc/articles/PMC9090392/
LRATD2	no	no	no					
NSD1	no	no	no					
COL21A1	no	no	yes	Mutation			35922433	https://www.ncbi.nlm.nih.gov/pmc/articles/PMC9349177/
NEDD4L	yes	no	yes	Downregulated	Risk	Atherosclerosis	28029182	https://onlinelibrary.wiley.com/doi/pdf/10.1002/jbmr.3073
ALS2	no	no	no					
CNN2	yes	no	yes	Upregulated	Risk	Atherosclerosis	26970176; 27575021	https://pubmed.ncbi.nlm.nih.gov/26970176/
PFDN5	no	no	yes				33372532	https://doi.org/10.1161/JAHA.120.018020
DPYSL5	no	no	yes		Risk	Hypertension	23118132; 17554300	https://www.ncbi.nlm.nih.gov/pmc/articles/PMC3583256/
MACROD1	no	no	no					
RBM4	yes	no	no			Cardiovascular Disease	36408906	https://academic.oup.com/nar/article/50/20/11895/6835366

PPP1R12C	no	no	no					
MTMR12	no	no	no					
NT5C3A	no	no	no					
PPIL3	no	no	no					
CHMP4B	no	no	yes	Downregulated	Risk	Aneurysm	37827202	https://www.sciencedirect.com/science/article/pii/S0925443923002818
ANTXR1	no	no	yes	Upregulated	Risk	Peripheral artery disease	26785120	https://www.ncbi.nlm.nih.gov/pmc/articles/PMC4718698/
PEAK1	yes	no	no			Cardiovascular disease	35241704	https://doi.org/10.1038%2Fs41598-022-07280-7
KAT8	no	no	yes	Upregulated	Risk	Coronary Artery Disease	30355081; 38334359	https://www.ahajournals.org/doi/10.1161/CIRCRESAHA.118.312497; https://elifesciences.org/articles/85235
QTRT2	no	no	no					
ELP3	no	no	no					
SRA1	no	no	yes			Atherosclerosis	24388105	https://www.ncbi.nlm.nih.gov/pmc/articles/PMC4163848/

DYNLRB1	no	no	yes	Mutation	Risk	Coronary Artery Disease	28530674	https://www.ncbi.nlm.nih.gov/pmc/articles/PMC5555387/
ANLN	yes	no	yes			Aneurysm	36579645	https://www.ahajournals.org/doi/10.1161/ATVBAHA.122.318135
MXRA5	no	no	no					
POLR3E	no	no	no					
POLR3B	no	no	no					
OCIAD1	no	no	no					
MTRES1	no	no	no					
DIP2B	no	no	yes	Mutation	Risk	Coronary Artery Disease	29491472	https://pubmed.ncbi.nlm.nih.gov/29491472/
SLAIN2	no	no	yes	Downregulated		Low wall shear stress regions	23284944	https://www.ncbi.nlm.nih.gov/pmc/articles/PMC3527404/
STK26	no	no	no					
TCF20	no	no	no					
SAP30BP	no	no	no					
AGO2	no	no	yes		Risk	Aneurysm	32151690; 36924230	https://pubmed.ncbi.nlm.nih.gov/32151690/
PRPF19	no	no	no					
NENF	no	no	no					
NDRG2	no	no	yes			Atherosclerosis	34470477; 19815093	https://pubmed.ncbi.nlm.nih.gov/34470477/

TPRKB	no	no	yes		Risk	Atherosclerosis	28902926	https://www.ncbi.nlm.nih.gov/pmc/articles/PMC5597284/
TCAF1	no	no	yes		Risk	Coronary Artery Disease	34121455	https://doi.org/10.1152/physiolgenomics.00020.2021
CLIC4	no	no	yes			Atherosclerosis	33439448	https://link.springer.com/article/10.1007/s11010-020-04035-8

Supplementary Table S8. Regulation by sex in iPSCs

LeadingGOTerm	ID	GOTerm	Ontology Source	Term PValue	Term PValue Corrected with Bonferroni step down	Group PValue	Group PValue Corrected with Bonferroni step down	GOLevels	GOGroups	% Associated Genes	Nr. Genes	Associated Genes Found	% Nr. Genes Changed	Term Status
TRUE	GO:0070013	intracellular organelle lumen	GO_CellularComponent-EBI-UniProt-GOA-ACAP-ARAP_13.05.2021_00h00	1.90E-14	1.50E-11	1.90E-14	4.00E-13	[4]	Group03	4.162349	241	[illegible] AIFM1, AIP, AK2, AK4, AKT2, ALAS1, ALDH1B1, ALDH6A1, ALDH7A1, ANP32E, ANXA2, APBB1, APEH, APOOL, APRT, ATG7, ATP5F1B, BAG6, BASP1, BCAT2, BCKDHA, BCKDHB, BDH1, CAPN2, CASK, CCDC137, CDC25C, CDK4, CDK5RAP3, CDK6, CDK9, CMPK1, COL4A1, COL6A1, CPNE3, CPOX, CPPED1, CPS1, CPT2, CRYM, CSTB, CTSB, CYB5R3, DBNL, DCAF8, DDX50, DHTKD1, DIAPH2, DNM1, DPH1, DUSP12, ECI1, EFTUD2, ELP4, ERP44, ETHE1, EZR, FABP5, FASN, FECH, FERMT2, FH, FHL2, FLAD1, FN1, FPGS, FUCA2, GCAT, GEMIN8, GJA1, GLO1, GLYR1, GNS, GOT1, GRHPR, GRPEL2, GSTK1, GTF2E1, GTF2H4, HADH, HEXB, HGS, HIBADH, HIBCH, HINT1, HIRIP3, HMOX1, HNRNPA3, HSPD1, HUWE1, IARS2, IDH2, IMMT, INTS2, IRF2BP2, IRF2BPL, IRF3, ITPR2, JPT1, KDM4B, KDM5B, KDM5C, LAMP2, LARP4B, LARP7, LGALS1, LIAS, LIPA, LMNB1, LRPPRC, LTA4H, LYRM7, MARS1, MCCC2, MDH2, MICOS13, MIF, MIOS, MLH3, MPHOSPH6, MRI1, MRPL17, MRPS21, MTHFD1L, MYO6, NCBP3, NDUFA8, NDUFS3, NDUFS8, NDUFV2, NELFB, NFAT5, NFS1, NIT2, NME2, NOP53, NQO2, NR6A1, NSA2, NSL1, NUDCD1, NXF1, OTUB1, P4HA1, PAFAH1B2, PAXX, PCYOX1L, PDCL3, PDK1, PDSS2, PEG10, PGM1, PHAX, PIR, PKP2, POGLUT3, POLR3G, PPA2, PPOX, PPP3CB, PRC1, PRDX5, PRKCA, PRKCI, PRODH, PSMB5, PSMD10, PSMD14, PSMD2, PSME2, PTBP1, PTPN11, PTPN14, PXDN, PYCR1, RAB34, RAN, RBBP7, RBFOX2, RBM34, RDH14, RECQL, RMI2, RNF126, RNF168, RPF1, RPP25, RPS11, RPS3, RRP1, SCRIB, SIRT3, SLC3A2, SNRPD3, SOD2, SPTBN1, SRI, SRPK2, STAT1, STK39, SUDS3, SUMF2, SYAP1, TAOK2, TASOR, TGFBRAP1, THBS1, TIMELESS, TKT, TOLLIP, TOP2A, TOR1A, TRIM22, TRIM26, TRMT112,	-76	Downregulation
TRUE	GO:0031982	vesicle	GO_CellularComponent-EBI-UniProt-GOA-ACAP-ARAP_13.05.2021_00h00	1.44E-15	1.14E-12	8.38E-10	1.42E-08	[4]	Group17	4.640539	193	[ABCB6, ACAT1, ACO1, ACP2, ACTR10, ACY1, AK2, AKT2, ALCAM, ALDH1A1, ALDH7A1, ANP32E, ANXA2, APEH, APOOL, APPL1, APRT, ARHGDIA, ARHGEF18, ATG7, ATP2A2, ATP5F1B, ATP6V1H, B3GAT3, BAG6, BASP1, BCAT2, BROX, C17orf75, CA2, CAPN2, CASK, CBR1, CCT6A, CLTCL1, CMBL, CMPK1, CNTN1, COL6A1, CORO7, CPNE3, CPPED1, CRYM, CSTB, CTSB, CYB5R3, DBNL, DCXR, DEGS1, DIAPH2, DICER1, DNM1, DNPH1, EBP, ECE1, EHD4, EPHA4, EPN2, ERP44, EXOC3, EZR, FABP3, FABP5, FASN, FBLN1, FH, FLT1, FN1, FREM2, FUCA2, GATD1, GJA1, GLO1, GNS, GOT1, GRHPR, GSS, GSTK1, GSTM2, HEXB, HGS, HINT1, HPRT1, HSPD1, HUWE1, IDH2, IGFBP2, IQGAP2, ITPR2, KIDINS220, KIF3B, LAMA5, LAMP2, LCP1, LDHA, LGALS1, LLGL1, LTA4H, LYPLA2, MARS1, MDH2, MGRN1, MICALL1, MIF, MOSPD2, MPHOSPH6, MPI, MYO1D, MYO5C, MYO6, NDRG3, NECAP1, NIT2, NME2, NQO2, OSBPL1A, OTUB1, PACS1, PAFAH1B2, PCYOX1L, PEPD, PEX1, PGLS, PGM1, PI4K2A, PLXNB2, PPA1, PRCP, PRDX5, PRKCA, PRKCI, PSMB5, PSMD14, PSMD2, PSME2, PTBP1, PTGR1, PTPRG, PXDN, RAB11FIP5, RAB17, RAB34, RAB3A, RAB8A, RAN, RAP2C, RIPK1, RPL34, RPS11, RPS3, RRAS, SCFD2, SCRIB, SCRN2, SCYL1, SEC24C, SEPTIN6, SLC1A5, SLC3A2, SLC44A2, SLC4A7, SLC7A5, SLC9A7, SNX9, SOD2, SPTBN1, SRI, SYAP1, TAOK2, TBC1D4, TGFBRAP1, THBS1, TKFC, TKT, TMEM256, TOLLIP, TOR1A, TUBA4A, TUBB2A, TUBB3, TUBB6, UBA1, UNC93B1, UXS1, VCL, VPS25, VPS26B, VPS29, VPS53, WDR81, WFS1, YES1, ZDHHC17]	-72	Downregulation
FALSE	GO:0005615	extracellular space	GO_CellularComponent-EBI-UniProt-GOA-ACAP-ARAP_13.05.2021_00h00	3.21E-11	2.51E-08	8.38E-10	1.42E-08	[2, 3]	Group17	4.466778	160	[ABCB6, ACAT1, ACO1, ACP2, ACY1, AK2, ALCAM, ALDH1A1, ALDH7A1, ANOS1, ANXA2, APEH, APPL1, APRT, ARHGDIA, ARHGEF18, ATP5F1B, ATP6V1H, ATXN10, B3GAT3, BAG6, BASP1, BCAT2, BROX, CA2, CAPN2, CBR1, CCN2, CCT6A, CLTCL1, CMBL, CMPK1, CNTN1, COL4A1, COL6A1, CPNE3, CPOX, CPZ, CRYM, CSTB, CTSB, DBNL, DCXR, DICER1, DNM1, DNPEP, DNPH1, ECE1, EHD4, ERP44, EZR, FABP3, FABP5, FASN, FBLN1, FH, FLT1, FN1, FREM2, FUCA2, GATD1, GLO1, GNS, GOT1, GRHPR, GSS, GSTK1, GSTM2, HEXB, HGS, HINT1, HMOX1, HPRT1, HSPD1, HUWE1, IDH2, IGFBP2, IQGAP2, IRF2BPL, KIF3B, LAMA5, LAMP2, LCP1, LDHA, LGALS1, LTA4H, LYPLA2, MARS1, MDH2, MGRN1, MIF, MOV10, MPHOSPH6, MPI, MYO1D, MYO5C, MYO6, NDRG3, NIT2, NME2, NQO2, OSBPL1A, OTUB1, PAFAH1B2, PEPD, PEX1, PGLS, PGM1, PLXNB2, PPA1, PRCP, PRDX5, PRKCA, PRKCI, PSMB5, PSMD2, PSME2, PTBP1, PTGR1, PTPRG, PXDN, RAB17, RAB34, RAB8A, RAN, RAP2C, RPL34, RPS11, RPS3, RRAS, RTN3, SCRIB, SCRN2, SFRP2, SLC1A5, SLC3A2, SLC44A2, SLC7A5, SNX9, SOD2, SPTBN1, SRI, SYAP1, THBS1, TKFC, TKT, TMEM256, TOLLIP, TOR1A, TRIM26, TUBA4A, TUBB2A, TUBB3, TUBB6, UBA1, UXS1, VCL, VPS25, YES1, ZNF649]	-72	Downregulation
TRUE	GO:0031090	organelle membrane	GO_CellularComponent-EBI-UniProt-GOA-ACAP-ARAP_13.05.2021_00h00	1.78E-08	1.37E-05	7.18E-20	1.65E-18	[3, 4]	Group21	4.134843	157	[ABCB6, ACP2, AIFM1, AKAP1, ALG9, ANXA2, APEH, APOOL, APPL1, ARFGAP2, ATP2A2, ATP5F1B, ATP5ME, ATP6V1H, B3GAT3, B3GNT7, BAK1, BDH1, CASK, CDK4, CLTCL1, COL6A1, COQ6, CORO7, COX6C, CPNE3, CPS1, CPT2, CYB5R3, DBNL, DEGS1, DMAC2L, DNM1, EBP, ECE1, EHD4, EMC2, EMC3, EPHA4, EPN2, ERP44, EXOC3, FABP5, FAM162A, FECH, FKBP2, FPGS, GALNT17, GALNT2, GCAT, GJA1, GOLGB1, GPD2, GRPEL2, HGS, HM13, HMOX1, HSPD1, HUWE1, IMMT, INTS2, IQGAP2, ITPR2, JPT1, LAMP2, LARGE2, LLGL1, LMNB1, LRPPRC, LYRM7, MBOAT7, MFSD10, MICALL1, MICOS13, MIOS, MOSPD2, MPC2, MRPL17, MRPS21, MRPS25, MTARC2, MTX1, MYO6, NDUFA5, NDUFA8, NDUFAF4, NDUFB11, NDUFS3, NDUFS8, NDUFV2, NECAP1, NLRX1, NME2, OSBPL8, PCYT1A, PCYT1B, PEX1, PEX10, PI4K2A, PIGS, PLD2, PLPP3, PPOX, PRCP, PRKCA, PRKCI, PRODH, PSMD2, PTDSS1, RAB11FIP5, RAB17, RAB34, RAB3A, RAB8A, RAP2C, RDH14, RIPK1, RPS3, RTN3, SCYL1, SEC24C, SEPHS1, SEZ6L2, SLC25A24, SLC3A2, SLC44A2, SLC7A5, SLC9A7, SNX9, SRD5A3, SRI, STX16, SURF1, TAOK2, TGFBRAP1, THADA, TKT, TMEM147, TMEM97, TOMM20, TOR1A, TRAM1, TYRO3, UBA1, UBXN4, UFD1, UNC93B1, UQCC2, UQCR11, UXS1, VPS25, VPS29, VPS53, WDR81, WFS1, ZBED1, ZDHHC17]	-69	Downregulation

FALSE	GO:1903561	extracellular vesicle	GO_CellularComponent-EBI-UniProt-GOA-ACAP-ARAP_13.05.2021_00h00	2.28E-23	1.81E-20	8.38E-10	1.42E-08	[5]	Group17	6.347826	146	[ABCB6, ACAT1, ACO1, ACP2, ACY1, AK2, ALCAM, ALDH1A1, ALDH7A1, ANXA2, APEH, APPL1, APRT, ARHGDIA, ARHGEF18, ATP5F1B, ATP6V1H, B3GAT3, BAG6, BASP1, BCAT2, BROX, CA2, CAPN2, CBR1, CCT6A, CLTCL1, CMBL, CMPK1, CNTN1, COL6A1, CPNE3, CRYM, CSTB, CTSB, DBNL, DCXR, DICER1, DNM1, DNPH1, ECE1, EHD4, ERP44, EZR, FABP3, FABP5, FASN, FBLN1, FH, FN1, FREM2, FUCA2, GATD1, GLO1, GNS, GOT1, GRHPR, GSS, GSTK1, GSTM2, HEXB, HGS, HINT1, HPRT1, HSPD1, HUWE1, IDH2, IGFBP2, IQGAP2, KIF3B, LAMA5, LAMP2, LCP1, LDHA, LGALS1, LTA4H, LYPLA2, MARS1, MDH2, MGRN1, MIF, MPHOSPH6, MPI, MYO1D, MYO5C, MYO6, NDRG3, NIT2, NME2, NQO2, OSBPL1A, OTUB1, PAFAH1B2, PEPD, PEX1, PGLS, PGM1, PLXNB2, PPA1, PRCP, PRDX5, PRKCA, PRKCI, PSMB5, PSMD2, PSME2, PTBP1, PTGR1, PTPRG, PXDN, RAB17, RAB34, RAB3A, RAB8A, RAN, RAP2C, RPL34, RPS11, RPS3, RRAS, SCRIB, SCRN2, SLC1A5, SLC3A2, SLC44A2, SLC7A5, SNX9, SOD2, SPTBN1, SRI, SYAP1, THBS1, TKFC, TKT, TMEM256, TOLLIP, TOR1A, TUBA4A, TUBB2A, TUBB3, TUBB6, UBA1, UXS1, VCL, VPS25, YES1]	-74	Downregulation
TRUE	GO:0070887	cellular response to chemical stimulus	GO_BiologicalProcess-EBI-UniProt-GOA-ACAP-ARAP_13.05.2021_00h00	6.14E-07	0.000460681	2.53E-07	3.04E-06	[3]	Group18	4.028369	142	[ABCB6, AIFM1, AIP, AK4, AKT2, ANOS1, ANXA2, APPL1, APRT, ARHGDIA, ARHGEF16, ARHGEF18, ATG7, ATP2A2, ATP6V1H, BAG3, BAG6, BAK1, BCAT2, CA2, CAPN2, CASK, CASTOR2, CCN2, CDK4, CDK5RAP3, CDK9, CMBL, CNTN1, COL4A1, COL6A1, CPNE3, CPS1, CTSB, CYB5R3, DUSP12, ECHDC3, EFTUD2, EHD4, EPHA4, EPN2, EZR, FAM162A, FASN, FECH, FERMT2, FLT1, FN1, GJA1, GOT1, GRB7, GSTK1, GSTM2, HGS, HMOX1, HSPD1, IGFBP2, IMPACT, IRF3, ITPR2, KANK1, KDM5B, KIDINS220, LAMA5, LANCL1, LCP1, LGALS1, LLGL1, LMNB1, MARS1, MICALL1, MIF, MOSPD2, MPC2, MTARC2, NFAT5, NGLY1, NME2, NOP53, NQO2, OSBPL8, OTUB1, PDK1, PEG10, PPM1F, PPP3CB, PRCP, PRDX5, PRKAR1A, PRKCA, PRKCI, PRODH, PRPF38B, PRXL2A, PSMB5, PSMD10, PSMD14, PSMD2, PSME2, PTBP1, PTK7, PTPN11, PTPN14, PXDN, PYCR1, RAB11FIP5, RAB8A, RANBP1, RBFOX2, RIPK1, RNF126, RPS3, SESN2, SFRP2, SHPK, SIGIRR, SIRT3, SLC25A24, SLC9A7, SOD2, SPART, SRI, STAT1, STK39, SYAP1, TBC1D4, TGFBRAP1, THBS1, TIMELESS, TOLLIP, TOR1A, TPM1, TRIM22, TRIM26, UFD1, USP9X, VPS26B, WFS1, XIAP, XRCC4, YES1, ZDHHC17]	-70	Downregulation
TRUE	GO:0043168	anion binding	GO_MolecularFunction-EBI-UniProt-GOA-ACAP-ARAP_13.05.2021_00h00	2.05E-12	1.61E-09	4.98E-15	1.10E-13	[3]	Group19	4.931109	136	[AARS2, ABCB6, ACADS, ACAT1, ACOX3, AIFM1, AK2, AK4, AKT2, ALAS1, ANXA2, APRT, ARAF, ARHGAP28, ARHGDIA, ARHGEF16, ARHGEF18, ARHGEF40, ATP2A2, ATP5F1B, CASK, CASTOR2, CCT6A, CDK4, CDK6, CDK9, CHM, CMPK1, COQ6, CPS1, CTPS2, CYB5R3, DDX10, DDX50, DHTKD1, DICER1, DNM1, EFTUD2, EHD4, EPHA4, EXOC7, FABP3, FABP5, FERMT2, FLAD1, FLT1, FPGS, GCAT, GOT1, GPHN, GRHPR, GSS, GSTM2, HADH, HSPD1, IARS2, IQGAP2, ITPR2, KIF3B, KIF5C, KYAT1, LANCL1, MAP3K4, MARS1, MCCC2, MICALL1, MLH3, MOV10, MTARC2, MTHFD1L, MYO1D, MYO5C, MYO6, NFS1, NLGN4X, NLRP2, NLRX1, NME2, NME3, NQO2, OPLAH, P3H3, P4HA1, PAK4, PBXIP1, PDK1, PEX1, PHKG2, PI4K2A, PPOX, PRKAR1A, PRKCA, PRKCI, PRODH, PRPF38B, PTK7, RAB17, RAB34, RAB3A, RAB8A, RAN, RANBP1, RAP2C, RECQL, RIPK1, RRAS, RTKN, SCYL1, SEPHS1, SEPTIN6, SESN2, SHPK, SIRT3, SRPK2, STK39, TAOK2, TGFBRAP1, TKFC, TKT, TOP2A, TOR1A, TP53I3, TRIM26, TUBA4A, TUBB2A, TUBB2B, TUBB3, TUBB6, TWF1, TYRO3, UBA1, UBA6, UBE2C, UXS1, WARS2, YES1]	-71	Downregulation
TRUE	GO:0051649	establishment of localization in cell	GO_BiologicalProcess-EBI-UniProt-GOA-ACAP-ARAP_13.05.2021_00h00	8.51E-11	6.65E-08	5.99E-14	1.08E-12	[3]	Group22	4.686423	136	[ACTR10, AIFM1, AIP, AKT2, ALMS1, ANXA2, APEH, APOOL, APPL1, APRT, ARFGAP2, ATG7, ATP2A2, ATP5F1B, ATP5ME, B3GAT3, BAG3, BAK1, C17orf75, CASK, CHM, CLTCL1, CORO7, CPNE3, CPPED1, CPT2, CSTB, CTSB, CTTN, CYB5R3, DBNL, DEGS1, DMAC2L, DNM1, DYNLL2, EHD4, ERP44, EXOC4, EZR, FABP3, FABP5, FN1, FUCA2, GEMIN8, GJA1, GNS, GOLGB1, GRPEL2, GSTM2, HEXB, HGS, HM13, HMOX1, HSPD1, HUWE1, IFT46, IPO4, IQGAP2, ITPR2, KIF3B, KIF5C, LAMP2, LCP1, LLGL1, LRPPRC, LTA4H, MGRN1, MICALL1, MIF, MOSPD2, MPC2, MYO1D, MYO5C, MYO6, NIT2, NLGN4X, NME2, NXF1, PAFAH1B2, PCYOX1L, PEX1, PEX10, PGM1, PHAX, PI4K2A, PLD2, PPP3CB, PRCP, PRDX5, PRKCA, PRKCI, PSMD14, PSMD2, PTPN11, PTPN14, PXDN, RAB11FIP5, RAB17, RAB3A, RAB8A, RAN, RANBP1, RAP2C, RNF126, RPL34, RPS11, RPS3, SCFD2, SCRIB, SEC24C, SLC44A2, SNRPD3, SNX9, SPTBN1, SRI, STX16, TAOK2, TBC1D4, TGFBRAP1, THBS1, TOLLIP, TOMM20, TOR1A, TRAM1, TTC21B, TUBA4A, TUBB2A, UBASH3B, UFD1, UNC93B1, VCL, VPS25, VPS26B, VPS29, VPS53, WDR81]	-71	Downregulation

TRUE	GO:0006793	phosphorus metabolic process	GO_BiologicalProcess-EBI-UniProt-GOA-ACAP-ARAP_13.05.2021_00h00	7.59E-09	5.89E-06	7.59E-09	1.06E-07	[3]	Group15	4.397394	135	[ABHD5, ACAT1, ACP2, AK2, AK4, AKT2, ALDH1A1, AMPD2, ANP32E, ANXA2, APRT, ARAF, ATP5F1B, ATP5ME, B3GAT3, BAK1, CAPN2, CASK, CCN2, CCNY, CDC25C, CDK4, CDK5RAP3, CDK6, CDK9, CEP43, CMPK1, CNTN1, CPNE3, CPPED1, CPS1, CTPS2, DBNL, DCTD, DCXR, DHTKD1, DMAC2L, DNPH1, DUSP12, EFR3A, EHD4, ELP4, EPHA4, FABP3, FABP5, FASN, FBLN1, FERMT2, FLAD1, FLT1, FN1, GNPDA2, GPD2, GPHN, GTF2H4, HEXB, HGS, HINT1, HPRT1, IDH2, IMPACT, KIDINS220, LCP1, LDHA, LLGL1, LRRC40, MAP3K4, MBOAT7, MCCC2, MIF, MPC2, NFS1, NME2, NME3, NOP53, NT5C2, OSBPL8, PAK4, PCYT1A, PCYT1B, PDK1, PGLS, PGM1, PHKA2, PHKG2, PI4K2A, PIGS, PLD2, PLPP3, PLXNB2, PPA1, PPA2, PPM1F, PPP3CB, PRDX5, PRKAR1A, PRKCA, PRKCI, PSMD10, PTBP1, PTDSS1, PTK7, PTPN11, PTPN14, PTPRG, PUDP, RAN, RAP2C, RIPK1, RPS3, SCYL1, SEPHS1, SESN2, SFRP2, SHPK, SLC44A2, SMG8, SNX9, SPTBN1, SRD5A3, SRPK2, STK39, SYAP1, TAOK2, THBS1, TKFC, TKT, TOLLIP, TWF1, TYRO3, UBASH3B, UXS1, VPS25, WDR81, YES1]	-72	Downregulation
FALSE	GO:0006796	phosphate-containing compound metabolic process	GO_BiologicalProcess-EBI-UniProt-GOA-ACAP-ARAP_13.05.2021_00h00	1.41E-08	1.09E-05	7.59E-09	1.06E-07	[4]	Group15	4.370687	133	[ABHD5, ACAT1, ACP2, AK2, AK4, AKT2, ALDH1A1, AMPD2, ANP32E, ANXA2, APRT, ARAF, ATP5F1B, ATP5ME, B3GAT3, BAK1, CAPN2, CASK, CCN2, CCNY, CDC25C, CDK4, CDK5RAP3, CDK6, CDK9, CEP43, CMPK1, CNTN1, CPNE3, CPPED1, CPS1, CTPS2, DBNL, DCTD, DCXR, DHTKD1, DMAC2L, DNPH1, DUSP12, EFR3A, EHD4, ELP4, EPHA4, FABP3, FABP5, FASN, FBLN1, FERMT2, FLAD1, FLT1, FN1, GPD2, GPHN, GTF2H4, HEXB, HGS, HINT1, HPRT1, IDH2, IMPACT, KIDINS220, LCP1, LDHA, LLGL1, LRRC40, MAP3K4, MBOAT7, MCCC2, MIF, MPC2, NFS1, NME2, NME3, NOP53, NT5C2, OSBPL8, PAK4, PCYT1A, PCYT1B, PDK1, PGLS, PGM1, PHKA2, PHKG2, PI4K2A, PIGS, PLD2, PLPP3, PLXNB2, PPA1, PPA2, PPM1F, PPP3CB, PRDX5, PRKAR1A, PRKCA, PRKCI, PSMD10, PTBP1, PTDSS1, PTK7, PTPN11, PTPN14, PTPRG, PUDP, RAN, RAP2C, RIPK1, RPS3, SCYL1, SEPHS1, SESN2, SFRP2, SHPK, SLC44A2, SMG8, SNX9, SPTBN1, SRD5A3, SRPK2, STK39, SYAP1, TAOK2, THBS1, TKFC, TKT, TOLLIP, TWF1, TYRO3, UBASH3B, VPS25, WDR81, YES1]	-72	Downregulation
FALSE	GO:0000166	nucleotide binding	GO_MolecularFunction-EBI-UniProt-GOA-ACAP-ARAP_13.05.2021_00h00	4.20E-12	3.30E-09	4.98E-15	1.10E-13	[3, 4]	Group19	5.033959	126	[AARS2, ABCB6, ACADS, ACAT1, ACOX3, AIFM1, AK2, AK4, AKT2, ALDH1A1, ALDH1B1, ALDH6A1, APRT, ARAF, ARHGAP28, ARHGDIA, ARHGEF16, ARHGEF18, ARHGEF40, ATP2A2, ATP5F1B, BAG3, CASK, CCT6A, CDK4, CDK6, CDK9, CHM, CMPK1, COQ6, CPS1, CRYM, CTPS2, CYB5R3, DDX10, DDX50, DICER1, DNM1, EFTUD2, EHD4, EPHA4, FLAD1, FLT1, FPGS, GLYR1, GPHN, GRHPR, GRPEL2, GSS, HADH, HIBADH, HINT1, HPRT1, HSPD1, IARS2, IDH2, KIF3B, KIF5C, LDHA, MAP3K4, MARS1, MCCC2, MLH3, MOV10, MTHFD1L, MYO1D, MYO5C, MYO6, NLRP2, NLRX1, NME2, NME3, NQO2, NT5C2, OPLAH, PAK4, PBXIP1, PDK1, PEX1, PHKG2, PI4K2A, PPOX, PRKAR1A, PRKCA, PRKCI, PRODH, PRPF38B, PTK7, RAB17, RAB34, RAB3A, RAB8A, RAN, RANBP1, RAP2C, RECQL, RIPK1, RRAS, RTKN, SCYL1, SEPHS1, SEPTIN6, SESN2, SHPK, SIRT3, SRPK2, STK39, TAOK2, TGFBRAP1, TKFC, TOP2A, TOR1A, TP53I3, TUBA4A, TUBB2A, TUBB2B, TUBB3, TUBB6, TWF1, TYRO3, UBA1, UBA6, UBE2C, UXS1, WARS2, YES1]	-71	Downregulation
FALSE	GO:0008104	protein localization	GO_BiologicalProcess-EBI-UniProt-GOA-ACAP-ARAP_13.05.2021_00h00	4.53E-08	3.47E-05	5.99E-14	1.08E-12	[3]	Group22	4.409621	121	[ACOX3, AIFM1, AIP, AKT2, ANXA2, APBB1, APPL1, ARFGAP2, ARHGAP33, ARHGDIA, ARHGEF16, ARHGEF18, ATG7, ATP6V1H, B3GAT3, BAG3, BAG6, C17orf75, CCT6A, CDK5RAP3, CGNL1, CHM, CLTCL1, CORO7, CTTN, EFR3A, EHD4, EMC2, EMC3, EXOC3, EXOC4, EXOC7, EZR, FERMT2, FRAS1, GJA1, GOLGB1, GPHN, GRPEL2, GSTK1, HADH, HGS, HM13, HMOX1, HSPD1, HUWE1, IDH2, IPO4, ITPR2, KIF3B, KIF5C, LAMA5, LAMP2, LARP7, LCP1, LLGL1, MICALL1, MIOS, MLH3, MPC2, MTX1, MYO1D, MYO6, NECAP1, NOP53, NXF1, OSBPL8, PACS1, PEX1, PEX10, PHAX, PKP2, PPM1F, PPP3CB, PRKCA, PRKCI, PSMD10, PTPN11, PTPN14, RAB11FIP5, RAB17, RAB34, RAB3A, RAB8A, RAN, RPL34, RPS11, RPS3, SCFD2, SCRIB, SCYL1, SEC24C, SEPTIN6, SESN2, SIRT3, SNX9, SPTBN1, SRI, STX16, TAOK2, TASOR, TBC1D4, TGFBRAP1, TOLLIP, TOMM20, TOR1A, TRAM1, TRIM22, TTC21B, TWF1, UFD1, UNC93B1, UQCC2, USP9X, VCL, VPS25, VPS26B, VPS29, VPS53, XRCC4, ZDHHC17]	-63	Downregulation

TRUE	GO:0044248	cellular catabolic process	GO_BiologicalProcess-EBI-UniProt-GOA-ACAP-ARAP_13.05.2021_00h00	8.05E-10	6.26E-07	3.49E-09	5.23E-08	[3]	Group14	4.771532	118	[ABHD5, ACADS, ACAT1, ACOX3, AIFM1, AKT2, ALDH6A1, ALDH7A1, ANXA2, ARAF, ATG7, ATP2A2, ATP6V1H, BAG3, BAG6, BCAT2, BCKDHA, BCKDHB, BDH1, CAPN2, CDK5RAP3, CNOT11, CPS1, CPT2, CRYM, CTSB, CTTN, DCXR, DICER1, DNPEP, DNPH1, DYNLL2, ECE1, ECHDC1, ECI1, EIF4G3, EPHA4, EXOC4, EXOC7, EZR, FABP3, FABP5, GCAT, GNPDA2, GNS, GOT1, GPD2, GSTM2, HADH, HEXB, HGS, HIBADH, HIBCH, HINT1, HM13, HMOX1, HPRT1, HUWE1, KYAT1, LAMP2, LARP4B, LCP1, LRPPRC, LTA4H, LYPLA2, MCCC2, MOV10, NGLY1, NIT1, NOP53, NQO2, NT5C2, PAFAH1B2, PCYOX1L, PHAX, PHKA2, PHKG2, PNPLA4, PRDX5, PRKCA, PRODH, PSMB5, PSMD10, PSMD14, PSMD2, PSME2, PXDN, RAB8A, RDH14, RIPK1, RNF126, RNF168, RPL34, RPS11, RPS3, SESN2, SMG8, SNX9, SRD5A3, TGFBRAP1, TKFC, TMTC3, TOLLIP, TOMM20, TOR1A, TRIM22, UBA1, UBA6, UBE2C, UBXN4, UFD1, USP11, USP9X, VPS25, VPS26B, VPS29, WDR81, WFS1]	-70	Downregulation
FALSE	GO:0005739	mitochondrion	GO_CellularComponent-EBI-UniProt-GOA-ACAP-ARAP_13.05.2021_00h00	2.91E-20	2.31E-17	7.18E-20	1.65E-18	[3, 4, 5]	Group21	6.697195	117	[AARS2, ABCB6, ABHD5, ACADS, ACAT1, ACO1, AIFM1, AK2, AK4, AKAP1, ALAS1, ALDH1B1, ALDH6A1, ALDH7A1, APOOL, ARAF, ATP5F1B, ATP5ME, BAK1, BCAT2, BCKDHA, BCKDHB, BDH1, CAPN2, CDC25C, COQ6, COX6C, CPNE3, CPOX, CPS1, CPT2, CRYM, CYB5R3, DCAF8, DEGS1, DHTKD1, DMAC2L, DNM1, ECHDC3, ECI1, EPHA4, ETHE1, FAM162A, FECH, FH, FLAD1, FPGS, GCAT, GJA1, GPD2, GRPEL2, GSTK1, HADH, HIBADH, HIBCH, HSPD1, IARS2, IDH2, IMMT, IRF3, KYAT1, LIAS, LRPPRC, LYRM7, MCCC2, MDH2, MICOS13, MPC2, MRPL17, MRPS21, MRPS25, MTARC2, MTFR1L, MTHFD1L, MTX1, NDUFA5, NDUFA8, NDUFAF4, NDUFB11, NDUFS3, NDUFS8, NDUFV2, NFS1, NIT1, NLRX1, NME2, P4HA1, PDK1, PDSS2, PI4K2A, PNPLA4, PPA2, PPOX, PRC1, PRDX5, PRKCA, PRODH, PTPN11, PYCR1, RAB11FIP5, RIPK1, RPS3, SESN2, SIRT3, SLC25A24, SLC44A2, SLC9A7, SOD2, SRI, SURF1, TGFBRAP1, TOMM20, UBA1, UQCC2, UQCR11, WARS2, WDR81]	-83	Downregulation
FALSE	GO:0071310	cellular response to organic substance	GO_BiologicalProcess-EBI-UniProt-GOA-ACAP-ARAP_13.05.2021_00h00	1.35E-05	0.009686923	2.53E-07	3.04E-06	[4]	Group18	4.020797	116	[AIFM1, AIP, AKT2, ANOS1, ANXA2, APPL1, APRT, ARHGDIA, ARHGEF18, ATG7, ATP6V1H, BAG3, BAG6, BAK1, BCAT2, CA2, CAPN2, CASK, CASTOR2, CCN2, CDK4, CDK5RAP3, CDK9, CNTN1, COL4A1, COL6A1, CPNE3, CPS1, CTSB, DUSP12, ECHDC3, EHD4, EPHA4, EPN2, EZR, FASN, FECH, FERMT2, FLT1, FN1, GJA1, GOT1, GRB7, GSTM2, HGS, HMOX1, HSPD1, IGFBP2, IMPACT, IRF3, ITPR2, KANK1, KDM5B, KIDINS220, LAMA5, LCP1, LGALS1, LMNB1, MARS1, MICALL1, MIF, MPC2, NFAT5, NGLY1, NME2, OSBPL8, OTUB1, PEG10, PPP3CB, PRCP, PRKAR1A, PRKCA, PRKCI, PSMB5, PSMD10, PSMD14, PSMD2, PSME2, PTBP1, PTK7, PTPN11, PTPN14, PXDN, RAB11FIP5, RAB8A, RANBP1, RBFOX2, RIPK1, RNF126, RPS3, SESN2, SFRP2, SHPK, SIGIRR, SLC9A7, SOD2, SPART, SRI, STAT1, STK39, SYAP1, TBC1D4, TGFBRAP1, THBS1, TIMELESS, TOLLIP, TOR1A, TRIM22, TRIM26, UFD1, USP9X, VPS26B, WFS1, XIAP, YES1, ZDHHC17]	-68	Downregulation
FALSE	GO:1901575	organic substance catabolic process	GO_BiologicalProcess-EBI-UniProt-GOA-ACAP-ARAP_13.05.2021_00h00	7.74E-09	6.00E-06	3.49E-09	5.23E-08	[3]	Group14	4.6875	111	[ABHD5, ACADS, ACAT1, ACOX3, AIFM1, AKT2, ALDH1A1, ALDH1B1, ALDH6A1, ALDH7A1, ANXA2, ARAF, ATG7, BAG3, BAG6, BCAT2, BCKDHA, BCKDHB, BDH1, CAPN2, CDK4, CDK5RAP3, CNOT11, CPS1, CPT2, CRYM, CTSB, DCXR, DHTKD1, DICER1, DNPH1, ECE1, ECHDC1, ECI1, EPHA4, EZR, FABP3, FABP5, FUCA2, GCAT, GJA1, GNPDA2, GNS, GOT1, GPD2, HADH, HEXB, HGS, HIBADH, HIBCH, HINT1, HM13, HMOX1, HPRT1, HUWE1, KYAT1, LAMP2, LARP4B, LCP1, LDHA, LIPA, LRPPRC, LTA4H, LYPLA2, MCCC2, MOV10, NGLY1, NOP53, NQO2, NT5C2, PAFAH1B2, PCYOX1L, PGM1, PHAX, PHKA2, PHKG2, PLD2, PNPLA4, PRDX5, PRKCA, PRODH, PSMB5, PSMD10, PSMD14, PSMD2, PSME2, RDH14, RIPK1, RNF126, RNF168, RPL34, RPS11, RPS3, SESN2, SMG8, SNX9, SRD5A3, TKFC, TMTC3, TOLLIP, TOR1A, UBA1, UBA6, UBE2C, UBXN4, UFD1, USP11, USP9X, VPS25, WDR81, WFS1]	-71	Downregulation

FALSE	GO:0071702	organic substance transport	GO_BiologicalProcess-EBI-UniProt-GOA-ACAP-ARAP_13.05.2021_00h00	2.83E-05	0.020147879	5.99E-14	1.08E-12	[4]	Group22	4.002911	110	[ABCB6, AIFM1, AIP, AKT2, ANXA2, APBB1, APPL1, ARFGAP2, ARHGAP33, ATG7, ATP6V1H, B3GAT3, BAG3, C17orf75, CA2, CHM, CLTCL1, CORO7, CPT2, CTTN, EXOC3, EXOC4, EXOC7, EZR, FABP3, FABP5, FRAS1, GJA1, GRPEL2, HADH, HGS, HM13, HNRNPA3, HSPD1, HUWE1, IDH2, IPO4, ITPR2, KDM5B, KIF3B, KIF5C, LAMP2, LCP1, LLGL1, LRPPRC, MFSD10, MICALL1, MPC2, MTX1, MYO1D, MYO6, NCBP3, NECAP1, NXF1, OSBPL1A, OSBPL8, PEX1, PEX10, PHAX, PLIN2, PPM1F, PPP3CB, PRKCA, PRKCI, PTPN11, PTPN14, RAB11FIP5, RAB17, RAB34, RAB3A, RAB8A, RAN, RIPK1, RPL34, RPS11, RPS3, SCFD2, SCRIB, SEC24C, SESN2, SIRT3, SLC1A5, SLC25A24, SLC3A2, SLC44A2, SLC4A2, SLC4A7, SLC7A3, SLC7A5, SNX9, SPTBN1, SRI, STK39, STX16, TAOK2, TBC1D4, TGFBRAP1, THBS1, TOMM20, TOR1A, TRAM1, UFD1, UNC93B1, UQCC2, VPS25, VPS26B, VPS29, VPS53, YES1, ZDHHC17]	-61	Downregulation
TRUE	GO:1901566	organonitrogen compound biosynthetic process	GO_BiologicalProcess-EBI-UniProt-GOA-ACAP-ARAP_13.05.2021_00h00	9.02E-13	7.10E-10	5.63E-14	1.07E-12	[4]	Group13	5.585106	105	[AARS2, AATF, ABCB6, ACAT1, ACO1, ADI1, AK2, AK4, AKT2, ALAS1, ALDH7A1, ALG9, AMPD2, APEH, APRT, ATG7, ATP5F1B, ATP5ME, B3GAT3, B3GNT7, BCAT2, CAPN2, CDK4, CMPK1, CNOT11, CPOX, CPS1, CTPS2, DCTD, DEGS1, DMAC2L, DPH1, EIF4G3, FABP3, FABP5, FASN, FECH, FLAD1, FPGS, FTSJ1, GALNT17, GALNT2, GAMT, GOT1, GPHN, GRB7, GSS, GSTK1, GSTM2, HPRT1, IARS2, IDH2, IMPACT, KBTBD8, LARGE2, LARP4B, LRPPRC, MARS1, MOV10, MPC2, MPI, MRI1, MRPL17, MRPS21, MRPS25, MTHFD1L, NFS1, NME2, NME3, OPLAH, PCYT1A, PCYT1B, PDK1, PIGS, PLPP3, PLXNB2, POFUT1, POGLUT3, PPA1, PPA2, PPOX, PSMD2, PTDSS1, PUDP, PYCR1, PYCR3, RDH14, RPL22L1, RPL34, RPS11, RPS3, RWDD1, SEPHS1, SESN2, SIRT3, SLC44A2, SRD5A3, STK39, TGFBRAP1, THBS1, TMTC3, TRMT112, UQCC2, WARS2, ZDHHC17]	-77	Downregulation
FALSE	GO:0031410	cytoplasmic vesicle	GO_CellularComponent-EBI-UniProt-GOA-ACAP-ARAP_13.05.2021_00h00	1.57E-05	0.011287821	8.38E-10	1.42E-08	[3, 4, 6]	Group17	4.104769	105	[ABCB6, ACTR10, AKT2, ANP32E, ANXA2, APEH, APOOL, APPL1, APRT, ATG7, C17orf75, CLTCL1, CORO7, CPNE3, CPPED1, CSTB, CTSB, CYB5R3, DBNL, DEGS1, DIAPH2, DNM1, EBP, ECE1, EHD4, EPHA4, EPN2, ERP44, EXOC3, EZR, FABP5, FASN, FLT1, FN1, FUCA2, GJA1, GNS, HEXB, HGS, HSPD1, HUWE1, IGFBP2, IQGAP2, ITPR2, KIDINS220, LAMP2, LLGL1, LTA4H, MGRN1, MICALL1, MIF, MOSPD2, MYO1D, MYO6, NECAP1, NIT2, NME2, OSBPL1A, PACS1, PAFAH1B2, PCYOX1L, PGM1, PI4K2A, PRCP, PRDX5, PRKCA, PRKCI, PSMD14, PSMD2, PXDN, RAB11FIP5, RAB17, RAB34, RAB3A, RAB8A, RAN, RAP2C, RIPK1, SCFD2, SCYL1, SEC24C, SEPTIN6, SLC1A5, SLC3A2, SLC44A2, SLC4A7, SLC9A7, SNX9, SRI, TAOK2, TGFBRAP1, THBS1, TOLLIP, TOR1A, TUBB2A, UBA1, UNC93B1, VCL, VPS25, VPS26B, VPS29, VPS53, WDR81, WFS1, ZDHHC17]	-66	Downregulation
FALSE	GO:0016192	vesicle-mediated transport	GO_BiologicalProcess-EBI-UniProt-GOA-ACAP-ARAP_13.05.2021_00h00	1.99E-07	0.000150859	8.38E-10	1.42E-08	[2, 4]	Group17	4.500217	104	[ACTR10, AKT2, ALMS1, ANXA2, APEH, APOOL, APPL1, APRT, ARFGAP2, ATG7, ATP2A2, ATP6V1H, C17orf75, CASK, CHM, CLTCL1, CORO7, CPNE3, CPPED1, CSTB, CTSB, CTTN, CYB5R3, DBNL, DEGS1, DNM1, DYNLL2, EHD4, EPN2, ERP44, EXOC3, EXOC4, EXOC7, EZR, FABP5, FN1, FUCA2, GNS, GOLGB1, HEXB, HGS, HMOX1, HUWE1, IQGAP2, KIF3B, LAMP2, LLGL1, LTA4H, MGRN1, MICALL1, MIF, MOSPD2, MYO1D, MYO6, NCKIPSD, NECAP1, NIT2, NLGN4X, NME2, OSBPL1A, PAFAH1B2, PCYOX1L, PGM1, PI4K2A, PLD2, PLPP3, PPP3CB, PRCP, PRDX5, PRKCI, PSMD14, PSMD2, PXDN, RAB11FIP5, RAB17, RAB34, RAB3A, RAB8A, RAP2C, RNF126, RTN3, SCFD2, SCRIB, SCYL1, SEC24C, SLC44A2, SNX9, SPTBN1, STX16, TBC1D4, TGFBRAP1, THBS1, TOLLIP, TOR1A, TUBA4A, TUBB2A, TYRO3, VCL, VPS25, VPS26B, VPS29, VPS53, WDR81, YES1]	-71	Downregulation

FALSE	GO:0017076	purine nucleotide binding	GO_MolecularFunction-EBI-UniProt-GOA-ACAP-ARAP_13.05.2021_00h00	4.14E-08	3.18E-05	4.98E-15	1.10E-13	[4, 5]	Group19	4.640785	104	[AARS2, ABCB6, ACAT1, AK2, AK4, AKT2, ALDH6A1, APRT, ARAF, ARHGAP28, ARHGDIA, ARHGEF16, ARHGEF18, ARHGEF40, ATP2A2, ATP5F1B, BAG3, CASK, CCT6A, CDK4, CDK6, CDK9, CHM, CMPK1, CPS1, CTPS2, CYB5R3, DDX10, DDX50, DICER1, DNM1, EFTUD2, EHD4, EPHA4, FLAD1, FLT1, FPGS, GPHN, GRPEL2, GSS, HSPD1, IARS2, KIF3B, KIF5C, MAP3K4, MARS1, MCCC2, MLH3, MOV10, MTHFD1L, MYO1D, MYO5C, MYO6, NLRP2, NLRX1, NME2, NME3, OPLAH, PAK4, PBXIP1, PDK1, PEX1, PHKG2, PI4K2A, PRKAR1A, PRKCA, PRKCI, PRPF38B, PTK7, RAB17, RAB34, RAB3A, RAB8A, RAN, RANBP1, RAP2C, RECQL, RIPK1, RRAS, RTKN, SCYL1, SEPHS1, SEPTIN6, SESN2, SHPK, SRPK2, STK39, TAOK2, TGFBRAP1, TKFC, TOP2A, TOR1A, TUBA4A, TUBB2A, TUBB2B, TUBB3, TUBB6, TWF1, TYRO3, UBA1, UBA6, UBE2C, WARS2, YES1]	-70	Downregulation
FALSE	GO:0032555	purine ribonucleotide binding	GO_MolecularFunction-EBI-UniProt-GOA-ACAP-ARAP_13.05.2021_00h00	1.19E-07	9.01E-05	4.98E-15	1.10E-13	[4, 5, 6]	Group19	4.58221	102	[AARS2, ABCB6, ACAT1, AK2, AK4, AKT2, ALDH6A1, APRT, ARAF, ARHGAP28, ARHGDIA, ARHGEF16, ARHGEF18, ARHGEF40, ATP2A2, ATP5F1B, CASK, CCT6A, CDK4, CDK6, CDK9, CHM, CMPK1, CPS1, CTPS2, CYB5R3, DDX10, DDX50, DICER1, DNM1, EFTUD2, EHD4, EPHA4, FLAD1, FLT1, FPGS, GPHN, GSS, HSPD1, IARS2, KIF3B, KIF5C, MAP3K4, MARS1, MCCC2, MLH3, MOV10, MTHFD1L, MYO1D, MYO5C, MYO6, NLRP2, NLRX1, NME2, NME3, OPLAH, PAK4, PBXIP1, PDK1, PEX1, PHKG2, PI4K2A, PRKAR1A, PRKCA, PRKCI, PRPF38B, PTK7, RAB17, RAB34, RAB3A, RAB8A, RAN, RANBP1, RAP2C, RECQL, RIPK1, RRAS, RTKN, SCYL1, SEPHS1, SEPTIN6, SESN2, SHPK, SRPK2, STK39, TAOK2, TGFBRAP1, TKFC, TOP2A, TOR1A, TUBA4A, TUBB2A, TUBB2B, TUBB3, TUBB6, TWF1, TYRO3, UBA1, UBA6, UBE2C, WARS2, YES1]	-71	Downregulation
FALSE	GO:0071705	nitrogen compound transport	GO_BiologicalProcess-EBI-UniProt-GOA-ACAP-ARAP_13.05.2021_00h00	2.25E-05	0.016069684	5.99E-14	1.08E-12	[4]	Group22	4.159445	96	[ABCB6, AIFM1, AIP, AKT2, APBB1, APPL1, ARFGAP2, ARHGAP33, ATG7, ATP6V1H, B3GAT3, BAG3, C17orf75, CA2, CHM, CLTCL1, CORO7, CTTN, EXOC3, EXOC4, EXOC7, EZR, FRAS1, GJA1, GRPEL2, HADH, HGS, HM13, HNRNPA3, HSPD1, HUWE1, IDH2, IPO4, ITPR2, KIF3B, KIF5C, LAMP2, LCP1, LLGL1, LRPPRC, MICALL1, MPC2, MTX1, MYO1D, MYO6, NCBP3, NECAP1, NXF1, PEX1, PEX10, PHAX, PPM1F, PPP3CB, PRKCA, PRKCI, PTPN11, PTPN14, RAB11FIP5, RAB17, RAB34, RAB3A, RAB8A, RAN, RIPK1, RPL34, RPS11, RPS3, SCFD2, SCRIB, SEC24C, SIRT3, SLC1A5, SLC25A24, SLC3A2, SLC44A2, SLC7A3, SLC7A5, SNX9, SPTBN1, SRI, STK39, STX16, TAOK2, TBC1D4, TGFBRAP1, TOMM20, TOR1A, TRAM1, UFD1, UNC93B1, UQCC2, VPS25, VPS26B, VPS29, VPS53, ZDHHC17]	-62	Downregulation
TRUE	GO:0033554	cellular response to stress	GO_BiologicalProcess-EBI-UniProt-GOA-ACAP-ARAP_13.05.2021_00h00	3.79E-06	0.002784591	3.79E-06	3.79E-05	[3]	Group10	4.345837	95	[AATF, AIFM1, AK4, APBB1, ATG7, ATP2A2, ATP6V1H, BAG3, BAG6, BAK1, CA2, CASTOR2, CCN2, CDC25C, CDK5RAP3, CDK6, CDK9, CNOT11, DBNL, DNPEP, DUSP12, EPHA4, ERP44, EZR, FAM162A, FASN, FH, GSTM2, GTF2H4, HM13, HMOX1, HPF1, HSPD1, HUWE1, IMPACT, IRF3, KDM4B, LAMP2, LANCL1, MAP3K4, MARS1, MIF, MIOS, MLH3, MTARC2, MYO6, NGLY1, NME2, NOP53, OTUB1, PAXX, PDCL3, PDK1, PRDX5, PRKCA, PRODH, PRPF38B, PSMB5, PSMD10, PSMD14, PSMD2, PSME2, PTPN11, PXDN, PYCR1, RBBP7, RECQL, RIPK1, RMI2, RNF126, RNF168, RPS3, SESN2, SFRP2, SIRT3, SLC25A24, SOD2, STK39, TAOK2, TGFBRAP1, THBS1, TIMELESS, TMTC3, TOP2A, TOR1A, TPM1, UBA1, UBA6, UBXN4, UFD1, WDR4, WFS1, XAB2, XIAP, XRCC4]	-71	Downregulation

FALSE	GO:0035639	purine ribonucleoside triphosphate binding	GO_MolecularFunction-EBI-UniProt-GOA-ACAP-ARAP_13.05.2021_00h00	5.93E-06	0.004326972	4.98E-15	1.10E-13	[4]	Group19	4.341737	93	[AARS2, ABCB6, AK2, AK4, AKT2, ARAF, ARHGAP28, ARHGEF16, ARHGEF18, ARHGEF40, ATP2A2, ATP5F1B, CASK, CCT6A, CDK4, CDK6, CDK9, CMPK1, CPS1, CTPS2, DDX10, DDX50, DICER1, DNM1, EFTUD2, EHD4, EPHA4, FLAD1, FLT1, FPGS, GPHN, GSS, HSPD1, IARS2, KIF3B, KIF5C, MAP3K4, MARS1, MCCC2, MLH3, MOV10, MTHFD1L, MYO1D, MYO5C, MYO6, NLRP2, NLRX1, NME2, NME3, OPLAH, PAK4, PBXIP1, PDK1, PEX1, PHKG2, PI4K2A, PRKCA, PRKCI, PRPF38B, PTK7, RAB17, RAB34, RAB3A, RAB8A, RAN, RAP2C, RECQL, RIPK1, RRAS, RTKN, SCYL1, SEPHS1, SEPTIN6, SHPK, SRPK2, STK39, TAOK2, TGFBRAP1, TKFC, TOP2A, TOR1A, TUBA4A, TUBB2A, TUBB2B, TUBB3, TUBB6, TWF1, TYRO3, UBA1, UBA6, UBE2C, WARS2, YES1]	-69	Downregulation
TRUE	GO:0019899	enzyme binding	GO_MolecularFunction-EBI-UniProt-GOA-ACAP-ARAP_13.05.2021_00h00	7.51E-06	0.005460759	7.51E-06	6.01E-05	[3]	Group08	4.339623	92	[AATF, ACAT1, ANXA2, APBB1, APPL1, ARHGAP33, ARHGDIA, ARHGEF16, ATP2A2, ATXN10, BAG6, CAPN2, CCNY, CDC25C, CDK5RAP3, CDK9, CEP43, CHM, CNTN1, CPNE3, CSTB, DIAPH2, DNM1, DUSP12, ELP4, EPHA4, EXOC4, EZR, FASN, FERMT2, FN1, GRB7, GSTM2, HGS, HINT1, HM13, HMOX1, HSPD1, IPO4, IQGAP2, KIF3B, LAMP2, LCP1, LDHA, LLGL1, LMNB1, LRPPRC, MICALL1, NME2, OTUB1, PHKG2, PKP2, PPP3CB, PRC1, PRDX5, PRKAR1A, PRKCA, PTPN11, PTPN14, PXDN, RAB11FIP5, RAB34, RAB3A, RAB8A, RANBP1, RIPK1, RPS3, RTKN, SIRT3, SLC4A2, SNRPD3, SNX9, SOD2, SPART, SPTBN1, SRI, STAT1, STK39, SUDS3, TAOK2, TGFBRAP1, TOLLIP, TOP2A, TRIM22, TUBA4A, TUBB2A, UBASH3B, UBE2C, UFD1, VCL, WFS1, YES1]	-67	Downregulation
TRUE	GO:0042802	identical protein binding	GO_MolecularFunction-EBI-UniProt-GOA-ACAP-ARAP_13.05.2021_00h00	6.91E-06	0.005031978	6.91E-06	6.22E-05	[3]	Group01	4.341603	91	[ACAT1, ACY1, ALAS1, ALCAM, ANXA2, APEH, APPL1, ATG7, ATXN10, BAK1, CASTOR2, CEP43, CPOX, CRYM, CTPS2, DCTD, DCXR, DNM1, DNPEP, DNPH1, DYNLL2, EBP, ECE1, EPHA4, ETHE1, EZR, FABP5, FAM118A, FASN, FBLN1, FECH, FHL2, FLAD1, FN1, GNPDA2, GPHN, GRB7, GRHPR, GRPEL2, GSS, GSTM2, HADH, HEXB, HM13, HMOX1, HPRT1, IRF3, KYAT1, LCP1, LDHA, LGALS1, MICALL1, MIF, MPC2, MRI1, MTHFD1L, NFS1, NLGN4X, NME2, NOP53, NQO2, NR6A1, OPLAH, P4HA1, PAFAH1B2, PAXX, PCYT1A, PRDX5, PSME2, PTPRG, PYCR1, RIPK1, SEPHS1, SNX9, SOD2, STAT1, SUMF2, THBS1, TKT, TOP2A, TOR1A, TP53I3, TPM1, TRIM22, UBASH3B, UXS1, VPS25, XIAP, XRCC4, ZBED1, ZDHHC17]	-66	Downregulation
FALSE	GO:0045184	establishment of protein localization	GO_BiologicalProcess-EBI-UniProt-GOA-ACAP-ARAP_13.05.2021_00h00	2.05E-06	0.001509926	5.99E-14	1.08E-12	[3, 4]	Group22	4.526535	87	[AIFM1, AIP, AKT2, APBB1, APPL1, ARFGAP2, ARHGAP33, ATG7, ATP6V1H, B3GAT3, BAG3, BAG6, C17orf75, CCT6A, CHM, CLTCL1, CORO7, CTTN, EMC2, EMC3, EXOC3, EXOC4, EXOC7, EZR, FRAS1, GJA1, GRPEL2, HADH, HGS, HM13, HMOX1, HSPD1, HUWE1, IDH2, IPO4, ITPR2, KIF3B, KIF5C, LAMP2, LCP1, LLGL1, MICALL1, MPC2, MTX1, MYO1D, MYO6, NECAP1, NXF1, PEX1, PEX10, PHAX, PPM1F, PPP3CB, PRKCA, PRKCI, PTPN11, PTPN14, RAB11FIP5, RAB17, RAB34, RAB3A, RAB8A, RAN, RPL34, RPS11, RPS3, SCFD2, SCRIB, SEC24C, SIRT3, SNX9, SPTBN1, SRI, STX16, TAOK2, TBC1D4, TGFBRAP1, TOMM20, TRAM1, UFD1, UNC93B1, UQCC2, VPS25, VPS26B, VPS29, VPS53, ZDHHC17]	-64	Downregulation

FALSE	GO:0034613	cellular protein localization	GO_BiologicalProcess-EBI-UniProt-GOA-ACAP-ARAP_13.05.2021_00h00	1.76E-06	0.001301673	5.99E-14	1.08E-12	[4]	Group22	4.566929	87	[AIFM1, AIP, AKT2, ANXA2, APPL1, ARHGEF16, B3GAT3, BAG3, BAG6, C17orf75, CCT6A, CDK5RAP3, CHM, CLTCL1, CTTN, EFR3A, EHD4, EMC2, EMC3, EXOC3, EZR, FERMT2, GJA1, GOLGB1, GPHN, GRPEL2, HGS, HM13, HMOX1, HSPD1, HUWE1, IPO4, KIF5C, LAMA5, LAMP2, LARP7, LCP1, LLGL1, MICALL1, MIOS, MYO6, NOP53, OSBPL8, PACS1, PEX1, PEX10, PKP2, PPM1F, PRKCA, PRKCI, PTPN11, PTPN14, RAB11FIP5, RAB17, RAB34, RAB3A, RAB8A, RAN, RPL34, RPS11, RPS3, SCFD2, SCRIB, SCYL1, SEC24C, SEPTIN6, SESN2, SNX9, SPTBN1, STX16, TAOK2, TASOR, TBC1D4, TGFBRAP1, TOLLIP, TOMM20, TOR1A, TRAM1, TTC21B, TWF1, UFD1, UNC93B1, VCL, VPS25, VPS26B, VPS29, XRCC4]	-60	Downregulation
FALSE	GO:0015031	protein transport	GO_BiologicalProcess-EBI-UniProt-GOA-ACAP-ARAP_13.05.2021_00h00	4.56E-06	0.003331067	5.99E-14	1.08E-12	[4, 5]	Group22	4.535398	82	[AIFM1, AIP, AKT2, APBB1, APPL1, ARFGAP2, ARHGAP33, ATG7, ATP6V1H, B3GAT3, BAG3, C17orf75, CHM, CLTCL1, CORO7, CTTN, EXOC3, EXOC4, EXOC7, EZR, FRAS1, GJA1, GRPEL2, HADH, HGS, HM13, HSPD1, HUWE1, IDH2, IPO4, ITPR2, KIF3B, KIF5C, LAMP2, LCP1, LLGL1, MICALL1, MPC2, MTX1, MYO1D, MYO6, NECAP1, NXF1, PEX1, PEX10, PHAX, PPM1F, PPP3CB, PRKCA, PRKCI, PTPN11, PTPN14, RAB11FIP5, RAB17, RAB34, RAB3A, RAB8A, RAN, RPL34, RPS11, RPS3, SCFD2, SCRIB, SEC24C, SIRT3, SNX9, SPTBN1, SRI, STX16, TAOK2, TBC1D4, TGFBRAP1, TOMM20, TRAM1, UFD1, UNC93B1, UQCC2, VPS25, VPS26B, VPS29, VPS53, ZDHHC17]	-66	Downregulation
FALSE	GO:0046907	intracellular transport	GO_BiologicalProcess-EBI-UniProt-GOA-ACAP-ARAP_13.05.2021_00h00	1.23E-05	0.008876458	5.99E-14	1.08E-12	[3, 4]	Group22	4.466778	80	[ACTR10, AIFM1, AIP, AKT2, ALMS1, ANXA2, APPL1, ARFGAP2, ATP2A2, ATP5F1B, ATP5ME, B3GAT3, BAG3, C17orf75, CHM, CLTCL1, CORO7, CPT2, CTTN, DMAC2L, DYNLL2, EHD4, EZR, FABP3, GEMIN8, GOLGB1, GRPEL2, HGS, HM13, HSPD1, HUWE1, IFT46, IPO4, KIF3B, KIF5C, LAMP2, LCP1, LRPPRC, MGRN1, MICALL1, MYO1D, MYO5C, MYO6, NXF1, PEX1, PEX10, PHAX, PRKCA, PRKCI, PTPN11, PTPN14, RAB17, RAB8A, RAN, RANBP1, RNF126, RPL34, RPS11, RPS3, SCFD2, SCRIB, SEC24C, SNRPD3, SNX9, SPTBN1, STX16, TAOK2, TBC1D4, TGFBRAP1, TOMM20, TRAM1, TTC21B, TUBB2A, UFD1, UNC93B1, VPS25, VPS26B, VPS29, VPS53, WDR81]	-69	Downregulation
FALSE	GO:0031967	organelle envelope	GO_CellularComponent-EBI-UniProt-GOA-ACAP-ARAP_13.05.2021_00h00	1.08E-11	8.49E-09	7.18E-20	1.65E-18	[3, 4]	Group21	6.119288	79	[ABCB6, AIFM1, AK2, AKAP1, APEH, APOOL, ATP5F1B, ATP5ME, BAK1, BDH1, CAPN2, CDC25C, CDK4, COQ6, COX6C, CPOX, CPS1, CPT2, CYB5R3, DEGS1, DMAC2L, DNM1, EBP, EPHA4, FAM162A, FECH, FPGS, GCAT, GJA1, GPD2, GRPEL2, HSPD1, IMMT, INTS2, JPT1, LMNB1, LRPPRC, LYRM7, MFSD10, MICOS13, MPC2, MRPL17, MRPS21, MRPS25, MTARC2, MTX1, MYO6, NDUFA5, NDUFA8, NDUFAF4, NDUFB11, NDUFS3, NDUFS8, NDUFV2, NLRX1, NME2, NXF1, OSBPL8, PCYT1A, PPOX, PRKCA, PRODH, RAB11FIP5, RAN, RANBP1, RPS3, SEPHS1, SLC25A24, SURF1, TGFBRAP1, TMEM97, TOMM20, TOR1A, TUBB2A, TYRO3, UBXN4, UQCC2, UQCR11, ZBED1]	-70	Downregulation

FALSE	GO:0030554	adenyl nucleotide binding	GO_MolecularFunction-EBI-UniProt-GOA-ACAP-ARAP_13.05.2021_00h00	1.01E-06	0.000753031	4.98E-15	1.10E-13	[5, 6]	Group19	4.782342	78	[AARS2, ABCB6, ACAT1, AK2, AK4, AKT2, ALDH6A1, APRT, ARAF, ATP2A2, ATP5F1B, BAG3, CASK, CCT6A, CDK4, CDK6, CDK9, CMPK1, CPS1, CTPS2, CYB5R3, DDX10, DDX50, DICER1, EHD4, EPHA4, FLAD1, FLT1, FPGS, GPHN, GRPEL2, GSS, HSPD1, IARS2, KIF3B, KIF5C, MAP3K4, MARS1, MCCC2, MLH3, MOV10, MTHFD1L, MYO1D, MYO5C, MYO6, NLRP2, NLRX1, NME2, NME3, OPLAH, PAK4, PDK1, PEX1, PHKG2, PI4K2A, PRKAR1A, PRKCA, PRKCI, PTK7, RECQL, RIPK1, SCYL1, SEPHS1, SHPK, SRPK2, STK39, TAOK2, TGFBRAP1, TKFC, TOP2A, TOR1A, TWF1, TYRO3, UBA1, UBA6, UBE2C, WARS2, YES1]	-74	Downregulation
TRUE	GO:0006082	organic acid metabolic process	GO_BiologicalProcess-EBI-UniProt-GOA-ACAP-ARAP_13.05.2021_00h00	1.62E-13	1.28E-10	3.92E-14	7.84E-13	[3]	Group20	6.683805	78	[AARS2, ABHD5, ACADS, ACAT1, ACO1, ACOX3, ACY1, ADI1, AKT2, ALDH6A1, ALDH7A1, BCAT2, BCKDHA, BCKDHB, CBR1, CPS1, CPT2, CRYM, CTPS2, CYB5R3, DCXR, DEGS1, DHTKD1, ECHDC1, ECHDC3, ECI1, FABP3, FABP5, FASN, FH, FPGS, GAMT, GCAT, GLO1, GNPDA2, GOT1, GRHPR, GSS, GSTM2, HADH, HIBADH, HIBCH, IARS2, IDH2, KYAT1, LCP1, LDHA, LIAS, LTA4H, LYPLA2, MARS1, MCCC2, MDH2, MIF, MPC2, MRI1, MTARC2, MTHFD1L, NIT2, OSBPL1A, PDK1, PEPD, PGM1, PPA1, PPA2, PRODH, PSMB5, PSMD10, PSMD14, PSMD2, PSME2, PTGR1, PTGR2, PYCR1, PYCR3, SEPHS1, SESN2, WARS2]	-78	Downregulation
FALSE	GO:0046903	secretion	GO_BiologicalProcess-EBI-UniProt-GOA-ACAP-ARAP_13.05.2021_00h00	7.22E-07	0.000541097	5.99E-14	1.08E-12	[4]	Group22	4.848867	77	[ACTR10, ANXA2, APBB1, APEH, APOOL, APRT, ATG7, ATP2A2, CASK, CCN2, CPNE3, CPPED1, CSTB, CTSB, CYB5R3, DBNL, DEGS1, ERP44, EXOC3, EXOC4, EXOC7, EZR, FABP5, FN1, FUCA2, GJA1, GNS, HADH, HEXB, HGS, HMOX1, HUWE1, IDH2, IQGAP2, ITPR2, KDM5B, LAMP2, LLGL1, LTA4H, MIF, MOSPD2, MPC2, MTX1, MYO6, NIT2, NME2, PAFAH1B2, PCYOX1L, PGM1, PI4K2A, PPP3CB, PRCP, PRDX5, PRKCA, PRKCI, PSMD14, PSMD2, PTPN11, PXDN, RAB11FIP5, RAB3A, RAB8A, RAP2C, SCFD2, SCRIB, SIRT3, SLC1A5, SLC44A2, SRI, STK39, THBS1, TOLLIP, TUBA4A, TUBB2A, TYRO3, UQCC2, VCL]	-70	Downregulation
FALSE	GO:0032559	adenyl ribonucleotide binding	GO_MolecularFunction-EBI-UniProt-GOA-ACAP-ARAP_13.05.2021_00h00	3.07E-06	0.00225299	4.98E-15	1.10E-13	[5, 6, 7]	Group19	4.697157	76	[AARS2, ABCB6, ACAT1, AK2, AK4, AKT2, ALDH6A1, APRT, ARAF, ATP2A2, ATP5F1B, CASK, CCT6A, CDK4, CDK6, CDK9, CMPK1, CPS1, CTPS2, CYB5R3, DDX10, DDX50, DICER1, EHD4, EPHA4, FLAD1, FLT1, FPGS, GPHN, GSS, HSPD1, IARS2, KIF3B, KIF5C, MAP3K4, MARS1, MCCC2, MLH3, MOV10, MTHFD1L, MYO1D, MYO5C, MYO6, NLRP2, NLRX1, NME2, NME3, OPLAH, PAK4, PDK1, PEX1, PHKG2, PI4K2A, PRKAR1A, PRKCA, PRKCI, PTK7, RECQL, RIPK1, SCYL1, SEPHS1, SHPK, SRPK2, STK39, TAOK2, TGFBRAP1, TKFC, TOP2A, TOR1A, TWF1, TYRO3, UBA1, UBA6, UBE2C, WARS2, YES1]	-75	Downregulation

FALSE	GO:0043436	oxoacid metabolic process	GO_BiologicalProcess-EBI-UniProt-GOA-ACAP-ARAP_13.05.2021_00h00	3.33E-13	2.62E-10	3.92E-14	7.84E-13	[4]	Group20	6.690141	76	[AARS2, ABHD5, ACADS, ACAT1, ACO1, ACOX3, ACY1, ADI1, AKT2, ALDH6A1, ALDH7A1, BCAT2, CBR1, CPS1, CPT2, CRYM, CTPS2, CYB5R3, DCXR, DEGS1, DHTKD1, ECHDC1, ECHDC3, ECI1, FABP3, FABP5, FASN, FH, FPGS, GAMT, GCAT, GLO1, GNPDA2, GOT1, GRHPR, GSS, GSTM2, HADH, HIBADH, HIBCH, IARS2, IDH2, KYAT1, LCP1, LDHA, LIAS, LTA4H, LYPLA2, MARS1, MCCC2, MDH2, MIF, MPC2, MRI1, MTARC2, MTHFD1L, NIT2, OSBPL1A, PDK1, PEPD, PGM1, PPA1, PPA2, PRODH, PSMB5, PSMD10, PSMD14, PSMD2, PSME2, PTGR1, PTGR2, PYCR1, PYCR3, SEPHS1, SESN2, WARS2]	-78	Downregulation
FALSE	GO:0032940	secretion by cell	GO_BiologicalProcess-EBI-UniProt-GOA-ACAP-ARAP_13.05.2021_00h00	1.19E-07	9.02E-05	5.99E-14	1.08E-12	[3, 5]	Group22	5.156795	74	[ACTR10, ANXA2, APBB1, APEH, APOOL, APRT, ATG7, ATP2A2, CASK, CCN2, CPNE3, CPPED1, CSTB, CTSB, CYB5R3, DBNL, DEGS1, ERP44, EXOC3, EXOC4, EXOC7, EZR, FABP5, FN1, FUCA2, GJA1, GNS, HADH, HEXB, HGS, HMOX1, HUWE1, IDH2, IQGAP2, ITPR2, KDM5B, LAMP2, LLGL1, LTA4H, MIF, MOSPD2, MPC2, NIT2, NME2, PAFAH1B2, PCYOX1L, PGM1, PI4K2A, PPP3CB, PRCP, PRDX5, PRKCA, PSMD14, PSMD2, PTPN11, PXDN, RAB11FIP5, RAB3A, RAB8A, RAP2C, SCFD2, SCRIB, SIRT3, SLC1A5, SLC44A2, SRI, STK39, THBS1, TOLLIP, TUBA4A, TUBB2A, TYRO3, UQCC2, VCL]	-69	Downregulation
FALSE	GO:0005524	ATP binding	GO_MolecularFunction-EBI-UniProt-GOA-ACAP-ARAP_13.05.2021_00h00	1.49E-05	0.010698957	4.98E-15	1.10E-13	[5, 6, 7, 8]	Group19	4.574742	71	[AARS2, ABCB6, AK2, AK4, AKT2, ARAF, ATP2A2, ATP5F1B, CASK, CCT6A, CDK4, CDK6, CDK9, CMPK1, CPS1, CTPS2, DDX10, DDX50, DICER1, EHD4, EPHA4, FLAD1, FLT1, FPGS, GPHN, GSS, HSPD1, IARS2, KIF3B, KIF5C, MAP3K4, MARS1, MCCC2, MLH3, MOV10, MTHFD1L, MYO1D, MYO5C, MYO6, NLRP2, NLRX1, NME2, NME3, OPLAH, PAK4, PDK1, PEX1, PHKG2, PI4K2A, PRKCA, PRKCI, PTK7, RECQL, RIPK1, SCYL1, SEPHS1, SHPK, SRPK2, STK39, TAOK2, TGFBRAP1, TKFC, TOP2A, TOR1A, TWF1, TYRO3, UBA1, UBA6, UBE2C, WARS2, YES1]	-73	Downregulation
FALSE	GO:1901565	organonitrogen compound catabolic process	GO_BiologicalProcess-EBI-UniProt-GOA-ACAP-ARAP_13.05.2021_00h00	3.05E-05	0.021642427	3.49E-09	5.23E-08	[4]	Group14	4.615385	66	[ACAT1, ALDH6A1, ALDH7A1, ANXA2, ARAF, ATG7, BAG3, BAG6, BCAT2, BCKDHA, BCKDHB, CAPN2, CDK5RAP3, CRYM, CTSB, DNPH1, ECE1, EPHA4, EZR, GCAT, GJA1, GNPDA2, GNS, GOT1, HEXB, HGS, HIBADH, HIBCH, HINT1, HM13, HMOX1, HPRT1, HUWE1, KYAT1, LAMP2, LTA4H, LYPLA2, MCCC2, NGLY1, NOP53, NT5C2, PCYOX1L, PRKCA, PRODH, PSMB5, PSMD10, PSMD14, PSMD2, PSME2, RIPK1, RNF126, RNF168, SNX9, TMTC3, TOLLIP, TOR1A, UBA1, UBA6, UBE2C, UBXN4, UFD1, USP11, USP9X, VPS25, WDR81, WFS1]	-74	Downregulation

FALSE	GO:0043603	cellular amide metabolic process	GO_BiologicalProcess-EBI-UniProt-GOA-ACAP-ARAP_13.05.2021_00h00	2.24E-06	0.001651709	5.63E-14	1.07E-12	[4]	Group13	5.052125	63	[AARS2, ACAT1, ACO1, AKT2, APEH, CDK4, CNOT11, CPS1, CPZ, DEGS1, DNPEP, DPH1, ECE1, EIF4G3, EPHA4, ETHE1, FASN, FPGS, FTSJ1, GLO1, GNPDA2, GRB7, GSS, GSTK1, GSTM2, HEXB, HM13, IARS2, IMPACT, KBTBD8, LARP4B, LRPPRC, LTA4H, MARS1, MCCC2, MOV10, MPC2, MRPL17, MRPS21, MRPS25, MTHFD1L, NIT1, OPLAH, PDK1, PLPP3, PLXNB2, PPA1, PPA2, RDH14, RPL22L1, RPL34, RPS11, RPS3, RTN3, RWDD1, SESN2, SIRT3, STK39, TGFBRAP1, THBS1, TRMT112, UQCC2, WARS2]	-68	Downregulation
FALSE	GO:0019637	organophosphate metabolic process	GO_BiologicalProcess-EBI-UniProt-GOA-ACAP-ARAP_13.05.2021_00h00	2.75E-07	0.000207751	7.59E-09	1.06E-07	[3, 4]	Group15	5.419581	62	[ABHD5, ACAT1, AK2, AK4, ALDH1A1, AMPD2, APRT, ATP5F1B, ATP5ME, CAPN2, CASK, CMPK1, CPNE3, CPS1, CTPS2, DCTD, DCXR, DHTKD1, DMAC2L, DNPH1, DUSP12, EFR3A, FABP3, FABP5, FASN, FLAD1, FLT1, GPD2, GPHN, HEXB, HINT1, HPRT1, IDH2, LCP1, LDHA, MBOAT7, MCCC2, MPC2, NFS1, NME2, NME3, NT5C2, OSBPL8, PCYT1A, PCYT1B, PDK1, PGLS, PGM1, PI4K2A, PIGS, PLD2, PLPP3, PRDX5, PTDSS1, PUDP, RAN, SHPK, SLC44A2, SRD5A3, TKFC, TKT, WDR81]	-74	Downregulation
FALSE	GO:1901698	response to nitrogen compound	GO_BiologicalProcess-EBI-UniProt-GOA-ACAP-ARAP_13.05.2021_00h00	2.50E-05	0.017814854	2.53E-07	3.04E-06	[3]	Group18	4.820262	59	[AIFM1, AKT2, APPL1, APRT, ATG7, ATP6V1H, BAG6, CA2, CAPN2, CASTOR2, CCN2, CDK4, COL4A1, COL6A1, CPS1, DNM1, DUSP12, ECHDC3, EFTUD2, EPHA4, EZR, FABP3, GJA1, GOT1, GRB7, GSS, GSTM2, HADH, HM13, HPRT1, HSPD1, IGFBP2, IMPACT, IRF3, ITPR2, KANK1, LCP1, LDHA, MARS1, MTARC2, NGLY1, NME2, OSBPL8, PRKAR1A, PRKCA, PRKCI, PTPN11, PXDN, RAB8A, SESN2, STAT1, SYAP1, TBC1D4, TIMELESS, TOMM20, TOR1A, UBXN4, UFD1, WFS1]	-66	Downregulation
FALSE	GO:0005740	mitochondrial envelope	GO_CellularComponent-EBI-UniProt-GOA-ACAP-ARAP_13.05.2021_00h00	4.61E-11	3.61E-08	7.18E-20	1.65E-18	[4, 5, 6]	Group21	7.004831	58	[ABCB6, AIFM1, AK2, AKAP1, APOOL, ATP5F1B, ATP5ME, BAK1, BDH1, CAPN2, CDC25C, COQ6, COX6C, CPOX, CPS1, CPT2, CYB5R3, DEGS1, DMAC2L, DNM1, EPHA4, FAM162A, FECH, FPGS, GCAT, GJA1, GPD2, GRPEL2, HSPD1, IMMT, LYRM7, MICOS13, MPC2, MRPL17, MRPS21, MRPS25, MTARC2, MTX1, NDUFA5, NDUFA8, NDUFAF4, NDUFB11, NDUFS3, NDUFS8, NDUFV2, NLRX1, NME2, PPOX, PRKCA, PRODH, RAB11FIP5, RPS3, SLC25A24, SURF1, TGFBRAP1, TOMM20, UQCC2, UQCR11]	-78	Downregulation

FALSE	GO:0010243	response to organonitrogen compound	GO_BiologicalProcess-EBI-UniProt-GOA-ACAP-ARAP_13.05.2021_00h00	8.33E-06	0.006046693	2.53E-07	3.04E-06	[4]	Group18	5.017606	57	[AIFM1, AKT2, APPL1, APRT, ATG7, ATP6V1H, BAG6, CA2, CAPN2, CASTOR2, CCN2, CDK4, COL4A1, COL6A1, CPS1, DNM1, DUSP12, ECHDC3, EFTUD2, EPHA4, EZR, FABP3, GJA1, GOT1, GRB7, GSS, GSTM2, HADH, HM13, HPRT1, HSPD1, IGFBP2, IMPACT, ITPR2, KANK1, LCP1, LDHA, MARS1, NGLY1, NME2, OSBPL8, PRKAR1A, PRKCA, PRKCI, PTPN11, PXDN, RAB8A, SESN2, STAT1, SYAP1, TBC1D4, TIMELESS, TOMM20, TOR1A, UBXN4, UFD1, WFS1]	-65	Downregulation
FALSE	GO:0006887	exocytosis	GO_BiologicalProcess-EBI-UniProt-GOA-ACAP-ARAP_13.05.2021_00h00	9.03E-08	6.89E-05	5.99E-14	1.08E-12	[3, 4, 5, 6]	Group22	5.804481	57	[ACTR10, ANXA2, APEH, APOOL, APRT, ATG7, ATP2A2, CASK, CPNE3, CPPED1, CSTB, CTSB, CYB5R3, DBNL, DEGS1, ERP44, EXOC3, EXOC4, EXOC7, FABP5, FN1, FUCA2, GNS, HEXB, HGS, HMOX1, HUWE1, IQGAP2, LAMP2, LLGL1, LTA4H, MIF, MOSPD2, NIT2, NME2, PAFAH1B2, PCYOX1L, PGM1, PI4K2A, PPP3CB, PRCP, PRDX5, PSMD14, PSMD2, PXDN, RAB11FIP5, RAB3A, RAB8A, RAP2C, SCFD2, SCRIB, SLC44A2, THBS1, TOLLIP, TUBA4A, TUBB2A, VCL]	-70	Downregulation
FALSE	GO:0099503	secretory vesicle	GO_CellularComponent-EBI-UniProt-GOA-ACAP-ARAP_13.05.2021_00h00	4.17E-06	0.003050949	5.99E-14	1.08E-12	[4, 5, 7]	Group22	5.130513	57	[ACTR10, ANXA2, APEH, APOOL, APRT, ATG7, CPNE3, CPPED1, CSTB, CTSB, CYB5R3, DBNL, DEGS1, DNM1, ECE1, ERP44, EXOC3, FABP5, FN1, FUCA2, GNS, HEXB, HSPD1, HUWE1, IQGAP2, ITPR2, LAMP2, LLGL1, LTA4H, MIF, MOSPD2, NIT2, NME2, PAFAH1B2, PCYOX1L, PGM1, PI4K2A, PRCP, PRDX5, PRKCA, PSMD14, PSMD2, PXDN, RAB11FIP5, RAB3A, RAB8A, RAP2C, SCFD2, SEPTIN6, SLC44A2, SRI, THBS1, TOLLIP, TOR1A, TUBB2A, VCL, WFS1]	-67	Downregulation
TRUE	GO:0031175	neuron projection development	GO_BiologicalProcess-EBI-UniProt-GOA-ACAP-ARAP_13.05.2021_00h00	1.70E-05	0.012149832	1.70E-05	0.000101814	[5, 6, 8, 9, 10]	Group09	5.042017	54	[ALCAM, ANOS1, APBB1, ARHGAP33, ARHGDIA, ATXN10, CNTN1, CTTN, DBNL, DICER1, EPHA4, EZR, FN1, GJA1, GRB7, HPRT1, IMPACT, KANK1, KIDINS220, KIF5C, LAMA5, LGALS1, LLGL1, LYPLA2, MICALL1, MOV10, NCKIPSD, NME2, PLXNB2, PPP3CB, PRKCA, PRKCI, PTK7, PTPN11, PTPRG, RAB17, RAB3A, RAB8A, RANBP1, RBFOX2, SCRIB, SFRP2, SLC9A7, SPART, SPTBN1, TAOK2, TOR1A, TUBB2B, TUBB3, TWF1, UBA6, USP9X, VCL, ZDHHC17]	-69	Downregulation

FALSE	GO:0031966	mitochondrial membrane	GO_CellularComponent-EBI-UniProt-GOA-ACAP-ARAP_13.05.2021_00h00	3.32E-10	2.59E-07	7.18E-20	1.65E-18	[4, 5, 6, 7]	Group21	6.931964	54	[ABCB6, AIFM1, AKAP1, APOOL, ATP5F1B, ATP5ME, BAK1, BDH1, COQ6, COX6C, CPS1, CPT2, CYB5R3, DEGS1, DMAC2L, DNM1, EPHA4, FAM162A, FECH, FPGS, GCAT, GJA1, GPD2, GRPEL2, HSPD1, IMMT, LYRM7, MICOS13, MPC2, MRPL17, MRPS21, MRPS25, MTARC2, MTX1, NDUFA5, NDUFA8, NDUFAF4, NDUFB11, NDUFS3, NDUFS8, NDUFV2, NLRX1, NME2, PPOX, PRKCA, PRODH, RAB11FIP5, RPS3, SLC25A24, SURF1, TGFBRAP1, TOMM20, UQCC2, UQCR11]	-76	Downregulation
FALSE	GO:0030141	secretory granule	GO_CellularComponent-EBI-UniProt-GOA-ACAP-ARAP_13.05.2021_00h00	1.53E-06	0.00113766	5.99E-14	1.08E-12	[3, 5, 6, 8]	Group22	5.526036	52	[ACTR10, ANXA2, APEH, APOOL, APRT, ATG7, CPNE3, CPPED1, CSTB, CTSB, CYB5R3, DBNL, DEGS1, ECE1, ERP44, EXOC3, FABP5, FN1, FUCA2, GNS, HEXB, HSPD1, HUWE1, IQGAP2, ITPR2, LAMP2, LTA4H, MIF, MOSPD2, NIT2, NME2, PAFAH1B2, PCYOX1L, PGM1, PRCP, PRDX5, PRKCA, PSMD14, PSMD2, PXDN, RAB11FIP5, RAB3A, RAP2C, SCFD2, SLC44A2, SRI, THBS1, TOLLIP, TOR1A, TUBB2A, VCL, WFS1]	-67	Downregulation
FALSE	GO:0005759	mitochondrial matrix	GO_CellularComponent-EBI-UniProt-GOA-ACAP-ARAP_13.05.2021_00h00	7.00E-16	5.54E-13	7.18E-20	1.65E-18	[4, 5, 6]	Group21	10.09901	51	[ACADS, ACAT1, AK4, ALAS1, ALDH1B1, ALDH6A1, ALDH7A1, ATP5F1B, BCAT2, BCKDHA, BCKDHB, BDH1, CPS1, DHTKD1, ECI1, ETHE1, FECH, FH, FLAD1, FPGS, GRPEL2, GSTK1, HADH, HIBADH, HIBCH, HSPD1, IARS2, IDH2, LIAS, LRPPRC, LYRM7, MCCC2, MDH2, MRPL17, MRPS21, MTHFD1L, NDUFS3, NDUFS8, NFS1, PDK1, PDSS2, PPA2, PRDX5, PRODH, PYCR1, RPS3, SIRT3, SOD2, TGFBRAP1, UQCC2, WARS2]	-86	Downregulation
FALSE	GO:0045055	regulated exocytosis	GO_BiologicalProcess-EBI-UniProt-GOA-ACAP-ARAP_13.05.2021_00h00	5.78E-07	0.000434364	5.99E-14	1.08E-12	[4, 5, 6, 7]	Group22	5.773672	50	[ACTR10, ANXA2, APEH, APOOL, APRT, ATG7, ATP2A2, CASK, CPNE3, CPPED1, CSTB, CTSB, CYB5R3, DBNL, DEGS1, ERP44, FABP5, FN1, FUCA2, GNS, HEXB, HMOX1, HUWE1, IQGAP2, LAMP2, LTA4H, MIF, MOSPD2, NIT2, NME2, PAFAH1B2, PCYOX1L, PGM1, PI4K2A, PPP3CB, PRCP, PRDX5, PSMD14, PSMD2, PXDN, RAB11FIP5, RAB3A, RAP2C, SCRIB, SLC44A2, THBS1, TOLLIP, TUBA4A, TUBB2A, VCL]	-72	Downregulation

FALSE	GO:0006518	peptide metabolic process	GO_BiologicalProcess-EBI-UniProt-GOA-ACAP-ARAP_13.05.2021_00h00	1.33E-05	0.009595391	5.63E-14	1.07E-12	[4, 5]	Group13	5.212766	49	[AARS2, ACO1, AKT2, APEH, CDK4, CNOT11, CPZ, DNPEP, DPH1, ECE1, EIF4G3, EPHA4, ETHE1, FTSJ1, GLO1, GRB7, GSS, GSTK1, GSTM2, HM13, IARS2, IMPACT, KBTBD8, LARP4B, LRPPRC, LTA4H, MARS1, MOV10, MRPL17, MRPS21, MRPS25, OPLAH, PLXNB2, PPA1, PPA2, RDH14, RPL22L1, RPL34, RPS11, RPS3, RTN3, RWDD1, SESN2, STK39, TGFBRAP1, THBS1, TRMT112, UQCC2, WARS2]	-67	Downregulation
FALSE	GO:0002366	leukocyte activation involved in immune response	GO_BiologicalProcess-EBI-UniProt-GOA-ACAP-ARAP_13.05.2021_00h00	9.37E-06	0.006792319	5.99E-14	1.08E-12	[3, 4]	Group22	5.562579	44	[ACTR10, ANXA2, APEH, APRT, ATG7, CPNE3, CPPED1, CSTB, CTSB, CYB5R3, DBNL, DEGS1, ERP44, FABP5, FUCA2, GNS, HEXB, HMOX1, HSPD1, HUWE1, IQGAP2, LAMP2, LCP1, LGALS1, LTA4H, MIF, MOSPD2, NIT2, NME2, PAFAH1B2, PGM1, PI4K2A, PRCP, PRDX5, PSMD14, PSMD2, PXDN, RAB3A, RAP2C, RNF168, SLC44A2, TOLLIP, TUBB2A, VCL]	-77	Downregulation
TRUE	GO:0050839	cell adhesion molecule binding	GO_MolecularFunction-EBI-UniProt-GOA-ACAP-ARAP_13.05.2021_00h00	3.39E-09	2.63E-06	3.39E-09	5.42E-08	[3]	Group11	7.401033	43	[ANXA2, ARHGEF16, ARVCF, BAG3, BCAT2, CCN2, CTTN, DBNL, EHD4, EPN2, EXOC3, EZR, FASN, FBLN1, FERMT2, FN1, LAMA5, LCP1, LDHA, LYPLA2, MB21D2, MICALL1, NLGN4X, PAK4, PKP2, PLPP3, PRDX5, PRKCA, PTK7, PTPN11, RAN, RANBP1, RPL34, SCRIB, SCYL1, SFRP2, SLC3A2, SNX9, SPTBN1, STAT1, THBS1, TWF1, VCL]	NA	No change
FALSE	GO:0090407	organophosphate biosynthetic process	GO_BiologicalProcess-EBI-UniProt-GOA-ACAP-ARAP_13.05.2021_00h00	1.52E-06	0.001129748	7.59E-09	1.06E-07	[4, 5]	Group15	6.030856	43	[ABHD5, ACAT1, AK2, AK4, AMPD2, APRT, ATP5F1B, ATP5ME, CAPN2, CASK, CMPK1, CPNE3, CPS1, CTPS2, DCTD, DCXR, DMAC2L, EFR3A, FABP3, FABP5, FASN, FLAD1, FLT1, GPHN, HEXB, HPRT1, IDH2, MBOAT7, MPC2, NFS1, NME2, NME3, PCYT1A, PCYT1B, PDK1, PI4K2A, PIGS, PLD2, PTDSS1, SLC44A2, SRD5A3, TKT, WDR81]	-79	Downregulation

FALSE	GO:0002274	myeloid leukocyte activation	GO_BiologicalProcess-EBI-UniProt-GOA-ACAP-ARAP_13.05.2021_00h00	2.04E-06	0.00150638	5.99E-14	1.08E-12	[3, 4]	Group22	5.922865	43	[ACTR10, ANXA2, APEH, APRT, ATG7, CPNE3, CPPED1, CSTB, CTSB, CYB5R3, DBNL, DEGS1, ERP44, FABP5, FUCA2, GNS, HEXB, HMOX1, HSPD1, HUWE1, IQGAP2, LAMP2, LTA4H, MIF, MOSPD2, NIT2, NME2, PAFAH1B2, PGM1, PI4K2A, PRCP, PRDX5, PSMD14, PSMD2, PXDN, RAB3A, RAP2C, SHPK, SLC44A2, THBS1, TOLLIP, TUBB2A, VCL]	-74	Downregulation
FALSE	GO:0006091	generation of precursor metabolites and energy	GO_BiologicalProcess-EBI-UniProt-GOA-ACAP-ARAP_13.05.2021_00h00	1.25E-08	9.70E-06	7.18E-20	1.65E-18	[3]	Group21	7.179487	42	[ACAT1, ACO1, AK4, AKT2, ALDH1A1, ALDH1B1, ATP5F1B, ATP5ME, BDH1, COX6C, DEGS1, DHTKD1, DMAC2L, FECH, FH, GPD2, GYG2, IDH2, LDHA, LYRM7, MDH2, MTFR1L, NDUFA5, NDUFA8, NDUFB11, NDUFS3, NDUFS8, NDUFV2, NOP53, NQO2, PGLS, PGM1, PHKA2, PHKG2, SHPK, SIRT3, STK39, SURF1, TGFBRAP1, TKT, UQCC2, UQCR11]	-86	Downregulation
FALSE	GO:0019866	organelle inner membrane	GO_CellularComponent-EBI-UniProt-GOA-ACAP-ARAP_13.05.2021_00h00	2.51E-08	1.93E-05	7.18E-20	1.65E-18	[4, 5]	Group21	7.105719	41	[AIFM1, APOOL, ATP5F1B, ATP5ME, BDH1, COQ6, COX6C, CPS1, CPT2, DMAC2L, FECH, FPGS, GCAT, GPD2, GRPEL2, HSPD1, IMMT, LMNB1, LRPPRC, MFSD10, MICOS13, MPC2, MRPL17, MRPS21, MRPS25, MTX1, NDUFA5, NDUFA8, NDUFAF4, NDUFB11, NDUFS3, NDUFS8, NDUFV2, PPOX, PRODH, RPS3, SLC25A24, SURF1, TGFBRAP1, UQCC2, UQCR11]	-80	Downregulation
FALSE	GO:0002444	myeloid leukocyte mediated immunity	GO_BiologicalProcess-EBI-UniProt-GOA-ACAP-ARAP_13.05.2021_00h00	5.45E-07	0.000409736	5.99E-14	1.08E-12	[4]	Group22	6.430868	40	[ACTR10, ANXA2, APEH, APRT, ATG7, CPNE3, CPPED1, CSTB, CTSB, CYB5R3, DBNL, DEGS1, ERP44, FABP5, FUCA2, GNS, HEXB, HMOX1, HUWE1, IQGAP2, LAMP2, LTA4H, MIF, MOSPD2, NIT2, NME2, PAFAH1B2, PGM1, PI4K2A, PRCP, PRDX5, PSMD14, PSMD2, PXDN, RAB3A, RAP2C, SLC44A2, TOLLIP, TUBB2A, VCL]	-78	Downregulation

FALSE	GO:0002275	myeloid cell activation involved in immune response	GO_BiologicalProcess-EBI-UniProt-GOA-ACAP-ARAP_13.05.2021_00h00	4.05E-07	0.000304795	5.99E-14	1.08E-12	[4, 5]	Group22	6.514658	40	[ACTR10, ANXA2, APEH, APRT, ATG7, CPNE3, CPPED1, CSTB, CTSB, CYB5R3, DBNL, DEGS1, ERP44, FABP5, FUCA2, GNS, HEXB, HMOX1, HUWE1, IQGAP2, LAMP2, LTA4H, MIF, MOSPD2, NIT2, NME2, PAFAH1B2, PGM1, PI4K2A, PRCP, PRDX5, PSMD14, PSMD2, PXDN, RAB3A, RAP2C, SLC44A2, TOLLIP, TUBB2A, VCL]	-78	Downregulation
FALSE	GO:0043299	leukocyte degranulation	GO_BiologicalProcess-EBI-UniProt-GOA-ACAP-ARAP_13.05.2021_00h00	2.26E-07	0.000170557	5.99E-14	1.08E-12	[3, 4, 5, 6, 7, 8]	Group22	6.71141	40	[ACTR10, ANXA2, APEH, APRT, ATG7, CPNE3, CPPED1, CSTB, CTSB, CYB5R3, DBNL, DEGS1, ERP44, FABP5, FUCA2, GNS, HEXB, HMOX1, HUWE1, IQGAP2, LAMP2, LTA4H, MIF, MOSPD2, NIT2, NME2, PAFAH1B2, PGM1, PI4K2A, PRCP, PRDX5, PSMD14, PSMD2, PXDN, RAB3A, RAP2C, SLC44A2, TOLLIP, TUBB2A, VCL]	-78	Downregulation
FALSE	GO:0036230	granulocyte activation	GO_BiologicalProcess-EBI-UniProt-GOA-ACAP-ARAP_13.05.2021_00h00	9.91E-08	7.54E-05	5.99E-14	1.08E-12	[4, 5]	Group22	6.951872	39	[ACTR10, ANXA2, APEH, APRT, ATG7, CPNE3, CPPED1, CSTB, CTSB, CYB5R3, DBNL, DEGS1, ERP44, FABP5, FUCA2, GNS, HEXB, HUWE1, IQGAP2, LAMP2, LTA4H, MIF, MOSPD2, NIT2, NME2, PAFAH1B2, PGM1, PI4K2A, PRCP, PRDX5, PSMD14, PSMD2, PXDN, RAB3A, RAP2C, SLC44A2, TOLLIP, TUBB2A, VCL]	-77	Downregulation
FALSE	GO:0007005	mitochondrion organization	GO_BiologicalProcess-EBI-UniProt-GOA-ACAP-ARAP_13.05.2021_00h00	1.20E-06	0.000893938	7.18E-20	1.65E-18	[5]	Group21	6.397306	38	[AIFM1, AIP, ALAS1, APOOL, ATG7, ATP5F1B, ATP5ME, BAG3, BAK1, CLUH, CTTN, DMAC2L, DNM1, FAM162A, GRPEL2, HSPD1, HUWE1, IMMT, LYRM7, MICOS13, MTFR1L, MTX1, NDUFA5, NDUFA8, NDUFAF4, NDUFB11, NDUFS3, NDUFS8, NDUFV2, PSMD10, RAB3A, SESN2, SIRT3, SOD2, SURF1, TOMM20, UQCC2, WDR81]	-84	Downregulation

FALSE	GO:0005743	mitochondrial inner membrane	GO_CellularComponent-EBI-UniProt-GOA-ACAP-ARAP_13.05.2021_00h00	2.94E-08	2.26E-05	7.18E-20	1.65E-18	[5, 6, 7, 8]	Group21	7.392996	38	[AIFM1, APOOL, ATP5F1B, ATP5ME, BDH1, COQ6, COX6C, CPS1, CPT2, DMAC2L, FECH, FPGS, GCAT, GPD2, GRPEL2, HSPD1, IMMT, MICOS13, MPC2, MRPL17, MRPS21, MRPS25, MTX1, NDUFA5, NDUFA8, NDUFAF4, NDUFB11, NDUFS3, NDUFS8, NDUFV2, PPOX, PRODH, RPS3, SLC25A24, SURF1, TGFBRAP1, UQCC2, UQCR11]	-84	Downregulation
FALSE	GO:0043312	neutrophil degranulation	GO_BiologicalProcess-EBI-UniProt-GOA-ACAP-ARAP_13.05.2021_00h00	9.67E-08	7.37E-05	5.99E-14	1.08E-12	[4, 5, 6, 7, 8, 9]	Group22	7.063197	38	[ACTR10, ANXA2, APEH, APRT, ATG7, CPNE3, CPPED1, CSTB, CTSB, CYB5R3, DBNL, DEGS1, ERP44, FABP5, FUCA2, GNS, HEXB, HUWE1, IQGAP2, LAMP2, LTA4H, MIF, MOSPD2, NIT2, NME2, PAFAH1B2, PGM1, PRCP, PRDX5, PSMD14, PSMD2, PXDN, RAB3A, RAP2C, SLC44A2, TOLLIP, TUBB2A, VCL]	-76	Downregulation
TRUE	GO:0010035	response to inorganic substance	GO_BiologicalProcess-EBI-UniProt-GOA-ACAP-ARAP_13.05.2021_00h00	2.85E-05	0.020255408	1.74E-06	1.91E-05	[3]	Group16	5.732484	36	[ABCB6, ACO1, AIFM1, APBB1, ATG7, BAK1, CA2, CAPN2, CDK4, CPNE3, CPS1, DUSP12, FECH, GOT1, GPHN, GSS, HMOX1, HSPD1, IGFBP2, IMPACT, LDHA, LLGL1, PRPF38B, PTPN11, PYCR1, RANBP1, RIPK1, RPS3, SIRT3, SLC25A24, SOD2, STAT1, TGFBRAP1, THBS1, TIMELESS, XRCC4]	-86	Downregulation
FALSE	GO:0006520	cellular amino acid metabolic process	GO_BiologicalProcess-EBI-UniProt-GOA-ACAP-ARAP_13.05.2021_00h00	1.93E-10	1.50E-07	3.92E-14	7.84E-13	[3, 4, 6]	Group20	9.433962	35	[AARS2, ACAT1, ACY1, ADI1, ALDH6A1, ALDH7A1, BCAT2, CPS1, CRYM, CTPS2, FPGS, GCAT, GOT1, GSS, HIBADH, HIBCH, IARS2, KYAT1, MARS1, MCCC2, MRI1, NIT2, PEPD, PPA1, PPA2, PRODH, PSMB5, PSMD10, PSMD14, PSMD2, PSME2, PYCR1, PYCR3, SEPHS1, WARS2]	-83	Downregulation

FALSE	GO:0006979	response to oxidative stress	GO_BiologicalProcess-EBI-UniProt-GOA-ACAP-ARAP_13.05.2021_00h00	1.09E-06	0.00080995	1.74E-06	1.91E-05	[3]	Group16	6.918239	33	[AIFM1, ATG7, ATP2A2, BAK1, CAPN2, DUSP12, GSS, HMOX1, HSPD1, IMPACT, LANCL1, LDHA, LIAS, NDUFS8, NME2, PDK1, PRDX5, PRODH, PRPF38B, PSMB5, PTPN11, PXDN, PYCR1, RIPK1, RPS3, SESN2, SIRT3, SLC25A24, SOD2, STAT1, TGFBRAP1, TOR1A, TPM1]	-70	Downregulation
FALSE	GO:1901652	response to peptide	GO_Biolo	7.03E-05	0.049080863	2.53E-07	3.04E-06	[4, 5]	Group18	5.719237	33	[AKT2, APPL1, APRT, ATP6V1H, CA2, CCN2, CDK4, CPS1, DNM1,	-61	Downregulation
FALSE	GO:0044282	small molecule catabolic process	GO_BiologicalProcess-EBI-UniProt-GOA-ACAP-ARAP_13.05.2021_00h00	1.07E-05	0.00775973	3.92E-14	7.84E-13	[3]	Group20	6.458334	31	[ACADS, ACAT1, ACOX3, AKT2, ALDH1A1, ALDH1B1, ALDH6A1, ALDH7A1, BCAT2, BCKDHA, BCKDHB, BDH1, CPT2, CRYM, DCXR, ECHDC1, ECI1, GCAT, GNPDA2, GOT1, GPD2, HADH, HIBADH, HIBCH, KYAT1, MCCC2, NQO2, PRODH, SESN2, SRD5A3, TKFC]	-81	Downregulation
FALSE	GO:0015980	energy derivation by oxidation of organic compounds	GO_BiologicalProcess-EBI-UniProt-GOA-ACAP-ARAP_13.05.2021_00h00	9.66E-09	7.48E-06	7.18E-20	1.65E-18	[4]	Group21	8.857142	31	[ACO1, AK4, AKT2, ATP5F1B, ATP5ME, COX6C, DHTKD1, DMAC2L, FH, GPD2, GYG2, IDH2, LYRM7, MDH2, MTFR1L, NDUFA5, NDUFA8, NDUFB11, NDUFS3, NDUFS8, NDUFV2, NOP53, PGM1, PHKA2, PHKG2, SIRT3, STK39, SURF1, TGFBRAP1, UQCC2, UQCR11]	-90	Downregulation
FALSE	GO:0045296	cadherin binding	GO_MolecularFunction-EBI-UniProt-GOA-ACAP-ARAP_13.05.2021_00h00	5.15E-08	3.94E-05	3.39E-09	5.42E-08	[4]	Group11	8.426967	30	[ANXA2, ARHGEF16, ARVCF, BAG3, CTTN, DBNL, EHD4, EPN2, EXOC3, EZR, FASN, LDHA, LYPLA2, MB21D2, MICALL1, PAK4, PKP2, PRDX5, PTPN11, RAN, RANBP1, RPL34, SCRIB, SCYL1, SLC3A2, SNX9, SPTBN1, STAT1, TWF1, VCL]	NA	No change

FALSE	GO:0043434	response to peptide hormone	GO_BiologicalProcess-EBI-UniProt-GOA-ACAP-ARAP_13.05.2021_00h00	5.02E-05	0.035424377	2.53E-07	3.04E-06	[4, 5, 6]	Group18	6.147541	30	[AKT2, APPL1, APRT, ATP6V1H, CA2, CCN2, CDK4, CPS1, ECHDC3, FABP3, GJA1, GOT1, GRB7, HADH, IGFBP2, KANK1, LCP1, MARS1, NME2, OSBPL8, PRKAR1A, PRKCA, PRKCI, PTPN11, PXDN, RAB8A, SESN2, STAT1, SYAP1, TBC1D4]	-60	Downregulation
TRUE	GO:0005925	focal adhesion	GO_CellularComponent-EBI-UniProt-GOA-ACAP-ARAP_13.05.2021_00h00	5.56E-05	0.039075519	5.56E-05	0.000111168	[5]	Group00	6.19469	28	[ALCAM, CAPN2, CASK, CPNE3, CTTN, EZR, FERMT2, FHL2, FLNC, FLT1, FOCAD, GJA1, GRB7, LCP1, NME2, PAK4, PARVB, PTK7, PXDN, RPS11, RPS3, RRAS, SLC4A2, SORBS2, STX16, TWF1, VCL, YES1]	NA	No change
FALSE	GO:0034774	secretory granule lumen	GO_CellularComponent-EBI-UniProt-GOA-ACAP-ARAP_13.05.2021_00h00	7.79E-07	0.00058297	5.99E-14	1.08E-12	[4, 5, 6, 7, 8, 9]	Group22	7.756233	28	[ACTR10, ANXA2, APOOL, APRT, ATG7, CPPED1, CSTB, CYB5R3, DBNL, ERP44, FABP5, FN1, FUCA2, GNS, HEXB, HUWE1, MIF, NIT2, NME2, PAFAH1B2, PCYOX1L, PRDX5, PSMD14, PSMD2, THBS1, TOLLIP, TUBB2A, VCL]	-71	Downregulation
FALSE	GO:0045333	cellular respiration	GO_BiologicalProcess-EBI-UniProt-GOA-ACAP-ARAP_13.05.2021_00h00	2.92E-08	2.24E-05	7.18E-20	1.65E-18	[5]	Group21	9.920635	25	[ACO1, AK4, ATP5F1B, ATP5ME, COX6C, DHTKD1, DMAC2L, FH, GPD2, IDH2, LYRM7, MDH2, MTFR1L, NDUFA5, NDUFA8, NDUFB11, NDUFS3, NDUFS8, NDUFV2, NOP53, SIRT3, SURF1, TGFBRAP1, UQCC2, UQCR11]	-92	Downregulation

FALSE	GO:0016054	organic acid catabolic process	GO_BiologicalProcess-EBI-UniProt-GOA-ACAP-ARAP_13.05.2021_00h00	1.29E-06	0.000957732	3.92E-14	7.84E-13	[4]	Group20	8.36237	24	[ACADS, ACAT1, ACOX3, AKT2, ALDH6A1, ALDH7A1, BCAT2, BCKDHA, BCKDHB, CPT2, CRYM, DCXR, ECHDC1, ECI1, GCAT, GNPDA2, GOT1, HADH, HIBADH, HIBCH, KYAT1, MCCC2, PRODH, SESN2]	-83	Downregulation
FALSE	GO:0034599	cellular response to oxidative stress	GO_BiologicalProcess-EBI-UniProt-GOA-ACAP-ARAP_13.05.2021_00h00	5.33E-05	0.03753474	1.74E-06	1.91E-05	[4, 5]	Group16	7.028754	22	[AIFM1, ATG7, ATP2A2, HMOX1, IMPACT, LANCL1, NME2, PDK1, PRDX5, PRODH, PRPF38B, PTPN11, PXDN, PYCR1, RIPK1, RPS3, SESN2, SIRT3, SLC25A24, SOD2, TGFBRAP1, TPM1]	-68	Downregulation
FALSE	GO:0032868	response to insulin	GO_BiologicalProcess-EBI-UniProt-GOA-ACAP-ARAP_13.05.2021_00h00	5.86E-05	0.041083297	2.53E-07	3.04E-06	[5, 6, 7]	Group18	6.984127	22	[AKT2, APPL1, APRT, ATP6V1H, CDK4, ECHDC3, FABP3, GOT1, GRB7, HADH, IGFBP2, KANK1, LCP1, MARS1, OSBPL8, PRKCI, PTPN11, RAB8A, SESN2, STAT1, SYAP1, TBC1D4]	-68	Downregulation
FALSE	GO:0009060	aerobic respiration	GO_BiologicalProcess-EBI-UniProt-GOA-ACAP-ARAP_13.05.2021_00h00	5.83E-08	4.45E-05	7.18E-20	1.65E-18	[6]	Group21	10.62802	22	[ACO1, AK4, ATP5F1B, ATP5ME, COX6C, DHTKD1, DMAC2L, FH, IDH2, MDH2, MTFR1L, NDUFA5, NDUFA8, NDUFB11, NDUFS3, NDUFS8, NDUFV2, NOP53, SIRT3, SURF1, UQCC2, UQCR11]	-95	Downregulation
FALSE	GO:0000302	response to reactive oxygen species	GO_BiologicalProcess-EBI-UniProt-GOA-ACAP-ARAP_13.05.2021_00h00	2.37E-06	0.001743046	1.74E-06	1.91E-05	[4]	Group16	8.86076	21	[AIFM1, BAK1, CAPN2, DUSP12, HMOX1, HSPD1, IMPACT, LDHA, PRDX5, PRPF38B, PTPN11, PXDN, PYCR1, RIPK1, RPS3, SESN2, SIRT3, SOD2, STAT1, TGFBRAP1, TPM1]	-71	Downregulation

FALSE	GO:0046395	carboxylic acid catabolic process	GO_BiologicalProcess-EBI-UniProt-GOA-ACAP-ARAP_13.05.2021_00h00	1.78E-05	0.01275664	3.92E-14	7.84E-13	[5, 6]	Group20	7.777778	21	[ACADS, ACAT1, ACOX3, AKT2, ALDH6A1, ALDH7A1, CPT2, CRYM, DCXR, ECHDC1, ECI1, GCAT, GNPDA2, GOT1, HADH, HIBADH, HIBCH, KYAT1, MCCC2, PRODH, SESN2]	-81	Downregulation
FALSE	GO:0098798	mitochondrial protein-containing complex	GO_CellularComponent-EBI-UniProt-GOA-ACAP-ARAP_13.05.2021_00h00	6.21E-05	0.043468714	7.18E-20	1.65E-18	[2, 4, 5, 6]	Group21	7.352941	20	[APOOL, ATP5F1B, ATP5ME, BCKDHA, BCKDHB, GRPEL2, IMMT, MCCC2, MICOS13, MRPL17, MRPS21, MTX1, NDUFA5, NDUFA8, NDUFB11, NDUFS3, NDUFS8, NDUFV2, PDK1, TOMM20]	-80	Downregulation
FALSE	GO:0044242	cellular lipid catabolic process	GO_BiologicalProcess-EBI-UniProt-GOA-ACAP-ARAP_13.05.2021_00h00	6.40E-05	0.044710408	3.92E-14	7.84E-13	[4, 5]	Group20	7.569721	19	[ABHD5, ACADS, ACAT1, ACOX3, AKT2, CPS1, CPT2, ECHDC1, ECI1, FABP3, FABP5, HADH, HEXB, LCP1, LYPLA2, PNPLA4, PRDX5, SESN2, SRD5A3]	-74	Downregulation
FALSE	GO:0101002	ficolin-1-rich granule	GO_CellularComponent-EBI-UniProt-GOA-ACAP-ARAP_13.05.2021_00h00	3.97E-06	0.002909122	5.99E-14	1.08E-12	[4, 6, 7, 9]	Group22	9.223301	19	[ACTR10, APEH, ATG7, CSTB, CTSB, DBNL, GNS, HUWE1, LAMP2, LTA4H, MIF, NME2, PAFAH1B2, PGM1, PRCP, PSMD14, PSMD2, PXDN, VCL]	-79	Downregulation
TRUE	GO:0010952	positive regulation of peptidase activity	GO_BiologicalProcess-EBI-UniProt-GOA-ACAP-ARAP_13.05.2021_00h00	2.66E-05	0.018941719	2.66E-05	0.000133018	[6, 7, 8, 9]	Group04	8.372093	18	[AIFM1, BAK1, CCN2, CNTN1, DNPEP, EPHA4, FAM162A, FASN, FBLN1, FN1, HSPD1, NLRP2, PPM1F, PSMD14, PSME2, RIPK1, RPS3, SFRP2]	NA	No change
FALSE	GO:1904813	ficolin-1-rich granule lumen	GO_CellularComponent-EBI-UniProt-GOA-ACAP-ARAP_13.05.2021_00h00	2.80E-07	0.000211222	5.99E-14	1.08E-12	[5, 7, 8, 10]	Group22	12.14286	17	[ACTR10, APEH, ATG7, CSTB, CTSB, DBNL, GNS, HUWE1, LTA4H, MIF, NME2, PAFAH1B2, PGM1, PSMD14, PSMD2, PXDN, VCL]	-76	Downregulation

FALSE	GO:0042542	response to hydrogen peroxide	GO_BiologicalProcess-EBI-UniProt-GOA-ACAP-ARAP_13.05.2021_00h00	6.43E-06	0.004688838	1.74E-06	1.91E-05	[4, 5]	Group16	10.19108	16	[AIFM1, BAK1, CAPN2, DUSP12, HMOX1, HSPD1, IMPACT, LDHA, PRPF38B, PTPN11, PYCR1, RIPK1, RPS3, SOD2, STAT1, TGFBRAP1]	-81	Downregulation
TRUE	GO:0016614	oxidoreductase activity, acting on CH-OH group of donors	GO_MolecularFunction-EBI-UniProt-GOA-ACAP-ARAP_13.05.2021_00h00	9.55E-06	0.006911748	7.71E-08	1.00E-06	[3]	Group12	10.41667	15	[BDH1, CBR1, DCXR, FASN, GLYR1, GPD2, GRHPR, HADH, HIBADH, IDH2, LDHA, MDH2, PTGR1, PTGR2, RDH14]	-87	Downregulation
FALSE	GO:0051287	NAD binding	GO_MolecularFunction-EBI-UniProt-GOA-ACAP-ARAP_13.05.2021_00h00	1.81E-06	0.001341817	7.71E-08	1.00E-06	[4, 5]	Group12	16.41791	11	[ALDH1A1, ALDH1B1, CYB5R3, GLYR1, GRHPR, HADH, HIBADH, IDH2, LDHA, SIRT3, UXS1]	-82	Downregulation
TRUE	GO:0016655	oxidoreductase activity, acting on NAD(P)H, quinone or similar compound as acceptor	GO_MolecularFunction-EBI-UniProt-GOA-ACAP-ARAP_13.05.2021_00h00	3.84E-05	0.027138531	3.84E-05	0.000153542	[4]	Group06	14.7541	9	[CBR1, DCXR, NDUFA5, NDUFA8, NDUFS3, NDUFS8, NDUFV2, NQO2, TP53I3]	-89	Downregulation
TRUE	GO:0065002	intracellular protein transmembrane transport	GO_BiologicalProcess-EBI-UniProt-GOA-ACAP-ARAP_13.05.2021_00h00	5.70E-05	0.040004311	5.70E-05	5.70E-05	[4, 5, 6, 7]	Group02	16	8	[AIFM1, AKT2, GRPEL2, HSPD1, PEX1, PEX10, TOMM20, TRAM1]	-75	Downregulation
TRUE	GO:0016903	oxidoreductase activity, acting on the aldehyde or oxo group of donors	GO_MolecularFunction-EBI-UniProt-GOA-ACAP-ARAP_13.05.2021_00h00	4.20E-05	0.029662208	4.20E-05	0.000126043	[3]	Group07	16.66667	8	[ALDH16A1, ALDH1A1, ALDH1B1, ALDH6A1, ALDH7A1, BCKDHA, BCKDHB, DHTKD1]	-88	Downregulation
FALSE	GO:0009083	branched-chain amino acid catabolic process	GO_BiologicalProcess-EBI-UniProt-GOA-ACAP-ARAP_13.05.2021_00h00	9.95E-08	7.56E-05	3.92E-14	7.84E-13	[5]	Group20	34.78261	8	[ACAT1, ALDH6A1, BCAT2, BCKDHA, BCKDHB, HIBADH, HIBCH, MCCC2]	-100	Downregulation
TRUE	GO:0015949	nucleobase-containing small molecule interconversion	GO_BiologicalProcess-EBI-UniProt-GOA-ACAP-ARAP_13.05.2021_00h00	1.00E-05	0.007239313	1.00E-05	7.01E-05	[4, 5, 6]	Group05	24.13793	7	[AK2, AK4, CMPK1, CTPS2, DCTD, NME2, NME3]	-86	Downregulation
FALSE	GO:0006573	valine metabolic process	GO_BiologicalProcess-EBI-UniProt-GOA-ACAP-ARAP_13.05.2021_00h00	3.51E-05	0.024872194	3.92E-14	7.84E-13	[4, 5, 7]	Group20	50	4	[ALDH6A1, BCAT2, HIBADH, HIBCH]	-100	Downregulation

Supplementary Table S9. Regulation by sex in iVSMCs

LeadingGOTerm	ID	GOTerm	Ontology Source	Term PValue	Term PValue Corrected with Bonferroni step down	Group PValue	Group PValue Corrected with Bonferroni step down	GOLevels	GOGroups	% Associated Genes	Nr. Genes	Associated Genes Found	% Nr. Genes Changed	Term Status
FALSE	GO:0016812	hydrolase activity, acting on carbon-nitrogen (but not peptide) bonds, in cyclic amides	GO_MolecularFunction-EBI-UniProt-GOA-ACAP-ARAP_13.05.2021_00h00	6.08E-06	0.003056748	2.82E-21	3.38E-20	[4]	Group12	38.46154	5	[CAD, CRMP1, DPYSL2, DPYSL3, DPYSL5]	60	Upregulation
FALSE	GO:0043034	costamere	GO_CellularComponent-EBI-UniProt-GOA-ACAP-ARAP_13.05.2021_00h00	1.07E-07	5.64E-05	1.78E-12	1.78E-11	[2, 5, 6, 7]	Group11	36.842106	7	[ANK2, ANK3, ILK, KRT19, PGM5, SVIL, VCL]	-71	Downregulation
FALSE	GO:0006206	pyrimidine nucleobase metabolic process	GO_BiologicalProcess-EBI-UniProt-GOA-ACAP-ARAP_13.05.2021_00h00	9.07E-07	0.000471465	2.82E-21	3.38E-20	[5, 6, 7]	Group12	28	7	[CAD, CRMP1, CTPS2, DPYSL2, DPYSL3, DPYSL5, MTOR]	NA	No change
FALSE	GO:0006098	pentose-phosphate shunt	GO_BiologicalProcess-EBI-UniProt-GOA-ACAP-ARAP_13.05.2021_00h00	8.29E-05	0.03878227	2.82E-21	3.38E-20	[4, 5, 6]	Group12	23.809525	5	[PGM2, PHGDH, RPIA, SHPK, TKT]	80	Upregulation
FALSE	GO:0043039	tRNA aminoacylation	GO_BiologicalProcess-EBI-UniProt-GOA-ACAP-ARAP_13.05.2021_00h00	1.06E-08	5.61E-06	2.82E-21	3.38E-20	[5, 6, 8, 9]	Group12	22	11	[AARSD1, EEF1E1, EPRS1, FARSA, FARSB, LARS1, PPA1, QRSL1, VARS1, YARS1, YARS2]	91	Upregulation
FALSE	GO:0009112	nucleobase metabolic process	GO_BiologicalProcess-EBI-UniProt-GOA-ACAP-ARAP_13.05.2021_00h00	1.00E-06	0.000519232	2.82E-21	3.38E-20	[4, 5, 6]	Group12	18.75	9	[CAD, CRMP1, CTPS2, DPYSL2, DPYSL3, DPYSL5, GMPR2, MTOR, PRPS1]	NA	No change
FALSE	GO:0004812	aminoacyl-tRNA ligase activity	GO_MolecularFunction-EBI-UniProt-GOA-ACAP-ARAP_13.05.2021_00h00	6.14E-06	0.003081275	2.82E-21	3.38E-20	[4]	Group12	17.777779	8	[AARSD1, EPRS1, FARSA, FARSB, LARS1, VARS1, YARS1, YARS2]	100	Upregulation
FALSE	GO:0051156	glucose 6-phosphate metabolic process	GO_BiologicalProcess-EBI-UniProt-GOA-ACAP-ARAP_13.05.2021_00h00	1.71E-06	0.000882472	2.82E-21	3.38E-20	[4, 5]	Group12	17.647058	9	[GNPDA1, HK1, HK2, PGM2, PGM2L1, PHGDH, RPIA, SHPK, TKT]	67	Upregulation
FALSE	GO:0019320	hexose catabolic process	GO_BiologicalProcess-EBI-UniProt-GOA-ACAP-ARAP_13.05.2021_00h00	1.46E-06	0.000755606	2.82E-21	3.38E-20	[5, 6]	Group12	15.625	10	[ALDOC, FUT2, GALM, GNPDA1, HK1, HK2, PGM2, PGM2L1, SLC25A12, TKFC]	80	Upregulation

FALSE	GO:0016879	ligase activity, forming carbon-nitrogen bonds	GO_MolecularFunction-EBI-UniProt-GOA-ACAP-ARAP_13.05.2021_00h00	3.72E-05	0.017977053	2.82E-21	3.38E-20	[3]	Group12	14.035088	8	[ASS1, CAD, CTPS2, FPGS, GCLC, MTHFS, QRSL1, TTLL12]	62	Upregulation
TRUE	GO:1902041	regulation of extrinsic apoptotic signaling pathway via death domain receptors	GO_BiologicalProcess-EBI-UniProt-GOA-ACAP-ARAP_13.05.2021_00h00	9.73E-05	0.045233835	9.73E-05	9.73E-05	[6, 7, 8, 9]	Group04	12.307693	8	[ARHGEF2, CASP8, ETFDH, FAF1, FGA, FGB, FGG, TRAF2]	62	Upregulation
FALSE	GO:0072527	pyrimidine-containing compound metabolic process	GO_BiologicalProcess-EBI-UniProt-GOA-ACAP-ARAP_13.05.2021_00h00	2.15E-06	0.001103253	2.82E-21	3.38E-20	[4]	Group12	12.121212	12	[AK4, CAD, CRMP1, CTPS2, DPYSL2, DPYSL3, DPYSL5, MTOR, NME4, NT5C3A, PRPS1, PUDP]	NA	No change
FALSE	GO:0009064	glutamine family amino acid metabolic process	GO_BiologicalProcess-EBI-UniProt-GOA-ACAP-ARAP_13.05.2021_00h00	9.90E-06	0.004922019	2.82E-21	3.38E-20	[5, 6, 8]	Group12	11.458333	11	[ALDH18A1, ARG1, ASS1, CAD, CTPS2, FPGS, GCLC, GOT2, MTHFS, PHGDH, SLC25A12]	64	Upregulation
FALSE	GO:0044291	cell-cell contact zone	GO_CellularComponent-EBI-UniProt-GOA-ACAP-ARAP_13.05.2021_00h00	8.11E-05	0.038058387	1.78E-12	1.78E-11	[5]	Group11	11.111111	9	[ANK2, ANK3, ATP1B1, JUP, PGM5, RAP2C, SLC9A1, TJP2, VCL]	NA	No change
FALSE	GO:0005977	glycogen metabolic process	GO_BiologicalProcess-EBI-UniProt-GOA-ACAP-ARAP_13.05.2021_00h00	9.83E-05	0.045609424	2.82E-21	3.38E-20	[6, 7]	Group12	10.843373	9	[ENPP1, GYG2, IRS1, MTOR, PGM1, PGM2, PGM2L1, PHKB, PYGL]	67	Upregulation
FALSE	GO:0034109	homotypic cell-cell adhesion	GO_BiologicalProcess-EBI-UniProt-GOA-ACAP-ARAP_13.05.2021_00h00	4.79E-05	0.022916803	1.78E-12	1.78E-11	[4]	Group11	10.638298	10	[ANK3, FGA, FGB, FGG, ILK, JUP, MYL9, PLPP3, TJP2, VCL]	-80	Downregulation
FALSE	GO:0045047	protein targeting to ER	GO_BiologicalProcess-EBI-UniProt-GOA-ACAP-ARAP_13.05.2021_00h00	1.90E-05	0.009353515	2.82E-21	3.38E-20	[6, 7, 8]	Group12	9.836065	12	[CHMP4B, RPL13, RPL19, RPL24, RPL32, RPL36, RPL7A, RPS18, RPS2, RPS3A, RPS4X, RPS8]	83	Upregulation
FALSE	GO:0022626	cytosolic ribosome	GO_CellularComponent-EBI-UniProt-GOA-ACAP-ARAP_13.05.2021_00h00	6.40E-05	0.030357256	2.82E-21	3.38E-20	[3, 4, 5, 6]	Group12	9.40171	11	[RPL13, RPL19, RPL24, RPL32, RPL36, RPL7A, RPS18, RPS2, RPS3A, RPS4X, RPS8]	91	Upregulation
FALSE	GO:0000184	nuclear-transcribed mRNA catabolic process, nonsense-mediated decay	GO_BiologicalProcess-EBI-UniProt-GOA-ACAP-ARAP_13.05.2021_00h00	3.89E-05	0.018693303	2.82E-21	3.38E-20	[7, 8, 9, 10]	Group12	9.160305	12	[GSPT2, RPL13, RPL19, RPL24, RPL32, RPL36, RPL7A, RPS18, RPS2, RPS3A, RPS4X, RPS8]	83	Upregulation

FALSE	GO:0002181	cytoplasmic translation	GO_BiologicalProcess-EBI-UniProt-GOA-ACAP-ARAP_13.05.2021_00h00	4.38E-06	0.002223951	2.82E-21	3.38E-20	[6, 7, 8]	Group12	9.146341	15	[GSPT2, MTOR, RBM4, RPL13, RPL19, RPL22L1, RPL24, RPL32, RPL36, RPL7A, RPS18, RPS2, RPS3A, RPS4X, RPS8]	80	Upregulation
TRUE	GO:0060048	cardiac muscle contraction	GO_BiologicalProcess-EBI-UniProt-GOA-ACAP-ARAP_13.05.2021_00h00	2.02E-05	0.009883039	2.02E-05	8.08E-05	[6]	Group07	9.090909	13	[ANK2, ASB3, ATP1B1, CCN2, GATA4, JUP, MAP2K6, MTOR, MYL4, NEDD4L, SLC9A1, TNNT2, TRPM4]	62	Upregulation
FALSE	GO:0016052	carbohydrate catabolic process	GO_BiologicalProcess-EBI-UniProt-GOA-ACAP-ARAP_13.05.2021_00h00	5.72E-07	0.000297795	2.82E-21	3.38E-20	[4]	Group12	8.636364	19	[ALDOC, FUT2, GALM, GNPDA1, HK1, HK2, MAN2B2, MAN2C1, NUP98, PGM1, PGM2, PGM2L1, PHGDH, PHKB, PYGL, SEH1L, SLC25A12, STAT3, TKFC]	74	Upregulation
FALSE	GO:0006520	cellular amino acid metabolic process	GO_BiologicalProcess-EBI-UniProt-GOA-ACAP-ARAP_13.05.2021_00h00	7.78E-11	4.22E-08	2.82E-21	3.38E-20	[3, 4, 6]	Group12	8.625337	32	[AARSD1, ALDH18A1, ARG1, ASS1, BCAT2, BLMH, CAD, CTPS2, EEF1E1, EPRS1, FARSA, FARSB, FPGS, GCDH, GCLC, GOT2, LARS1, MMUT, MRI1, MTHFS, PHGDH, PPA1, PSMB2, PSMC1, PSMC4, PSMD14, QRSL1, SLC25A12, SRR, VARS1, YARS1, YARS2]	72	Upregulation
FALSE	GO:0070972	protein localization to endoplasmic reticulum	GO_BiologicalProcess-EBI-UniProt-GOA-ACAP-ARAP_13.05.2021_00h00	4.13E-05	0.019829202	2.82E-21	3.38E-20	[6]	Group12	8.496732	13	[ANK2, CHMP4B, RPL13, RPL19, RPL24, RPL32, RPL36, RPL7A, RPS18, RPS2, RPS3A, RPS4X, RPS8]	85	Upregulation
FALSE	GO:0072329	monocarboxylic acid catabolic process	GO_BiologicalProcess-EBI-UniProt-GOA-ACAP-ARAP_13.05.2021_00h00	9.15E-05	0.0426245	2.82E-21	3.38E-20	[6, 7]	Group12	8.391608	12	[ALDH3A2, CPT2, CYP2W1, ECI2, ETFDH, GCDH, HADH, IRS1, MMUT, MTOR, PCK2, PHGDH]	67	Upregulation
FALSE	GO:0030027	lamellipodium	GO_CellularComponent-EBI-UniProt-GOA-ACAP-ARAP_13.05.2021_00h00	2.80E-06	0.001432131	1.78E-12	1.78E-11	[3, 4]	Group11	8.1081085	18	[ALS2, ANTXR1, ARAP3, ARHGEF7, ARPC3, CAPG, CAPZB, CSPG4, DPP4, DPYSL3, ILK, IQGAP2, PHACTR4, PLEK2, PXDN, SLC9A1, SNX2, TUBB3]	NA	No change

FALSE	GO:0005996	monosaccharide metabolic process	GO_BiologicalProcess-EBI-UniProt-GOA-ACAP-ARAP_13.05.2021_00h00	4.16E-08	2.20E-05	2.82E-21	3.38E-20	[3, 4]	Group12	8.01282	25	[ALDOC, ENPP1, FUT2, GALM, GCLC, GNPDA1, GOT2, HK1, HK2, IRS1, MAN2B2, MAN2C1, MTOR, NLN, PC, PCK2, PGM1, PGM2, PGM2L1, PGM5, PHGDH, RPIA, SLC25A12, TKFC, TKT]	80	Upregulation
FALSE	GO:0005925	focal adhesion	GO_CellularComponent-EBI-UniProt-GOA-ACAP-ARAP_13.05.2021_00h00	1.93E-10	1.04E-07	1.78E-12	1.78E-11	[5]	Group11	7.743363	35	[ADGRE5, ARHGEF2, ARHGEF7, ARPC3, CNN1, CNN2, CPNE3, CSPG4, DPP4, EGFR, FLRT3, FZD1, ILK, JUP, L1CAM, LRP1, PACSIN2, PDLIM1, PEAK1, PGM5, PROCR, PXDN, RPL19, RPL7A, RPS18, RPS2, RPS3A, RPS4X, RPS8, RSU1, SLC9A1, SVIL, TGM2, TRIP6, VCL]	NA	No change
FALSE	GO:0006399	tRNA metabolic process	GO_BiologicalProcess-EBI-UniProt-GOA-ACAP-ARAP_13.05.2021_00h00	3.49E-05	0.016878698	2.82E-21	3.38E-20	[7, 8]	Group12	7.6923075	15	[AARSD1, EEF1E1, ELP3, EPRS1, FARSA, FARSB, LARS1, OSGEPL1, PPA1, QRSL1, QTRT2, TPRKB, VARS1, YARS1, YARS2]	73	Upregulation
FALSE	GO:0019318	hexose metabolic process	GO_BiologicalProcess-EBI-UniProt-GOA-ACAP-ARAP_13.05.2021_00h00	1.98E-06	0.001015443	2.82E-21	3.38E-20	[4, 5]	Group12	7.368421	21	[ALDOC, ENPP1, FUT2, GALM, GNPDA1, GOT2, HK1, HK2, IRS1, MAN2B2, MAN2C1, MTOR, NLN, PC, PCK2, PGM1, PGM2, PGM2L1, PGM5, SLC25A12, TKFC]	81	Upregulation
FALSE	GO:1901605	alpha-amino acid metabolic process	GO_BiologicalProcess-EBI-UniProt-GOA-ACAP-ARAP_13.05.2021_00h00	2.00E-05	0.009799207	2.82E-21	3.38E-20	[4, 5, 7]	Group12	7.327586	17	[ALDH18A1, ARG1, ASS1, BCAT2, BLMH, CAD, CTPS2, FPGS, GCDH, GCLC, GOT2, MMUT, MRI1, MTHFS, PHGDH, SLC25A12, SRR]	65	Upregulation
FALSE	GO:0000956	nuclear-transcribed mRNA catabolic process	GO_BiologicalProcess-EBI-UniProt-GOA-ACAP-ARAP_13.05.2021_00h00	4.27E-05	0.020431283	2.82E-21	3.38E-20	[6, 7, 8, 9]	Group12	7.207207	16	[AGO2, GSPT2, PATL1, RPL13, RPL19, RPL24, RPL32, RPL36, RPL7A, RPS18, RPS2, RPS3A, RPS4X, RPS8, TNKS1BP1, TUT1]	81	Upregulation

FALSE	GO:0043292	contractile fiber	GO_CellularComponent-EBI-UniProt-GOA-ACAP-ARAP_13.05.2021_00h00	1.45E-05	0.007158129	1.78E-12	1.78E-11	[3, 4, 5]	Group11	7.2	18	[ABCC9, ANK2, ANK3, CAPZB, DEK, ILK, JUP, KRT19, LMOD1, MTMR12, MYL4, MYL9, PDLIM1, PGM5, SVIL, TNNT2, TPM3, VCL]	NA	No change
FALSE	GO:0006413	translational initiation	GO_BiologicalProcess-EBI-UniProt-GOA-ACAP-ARAP_13.05.2021_00h00	7.72E-05	0.036341353	2.82E-21	3.38E-20	[3, 6, 7, 8]	Group12	7.1770334	15	[AGO2, KHDRBS1, MTOR, RBM4, RPL13, RPL19, RPL24, RPL32, RPL36, RPL7A, RPS18, RPS2, RPS3A, RPS4X, RPS8]	73	Upregulation
FALSE	GO:0044282	small molecule catabolic process (eh not informative)	GO_BiologicalProcess-EBI-UniProt-GOA-ACAP-ARAP_13.05.2021_00h00	3.55E-09	1.89E-06	2.82E-21	3.38E-20	[3]	Group12	7.0833335	34	[ALDH1B1, ALDH3A2, ALDOC, ARG1, BCAT2, BLMH, CPT2, CYP2W1, ECI2, ETFDH, FUT2, GALM, GCDH, GNPDA1, GOT2, HADH, HK1, HK2, IRS1, MMUT, MTHFS, MTOR, NQO2, NT5C3A, NUDT1, OCRL, PCK2, PGM2, PGM2L1, PHGDH, QPRT, SLC25A12, SULT1E1, TKFC]	62	Upregulation
FALSE	GO:0006006	glucose metabolic process	GO_BiologicalProcess-EBI-UniProt-GOA-ACAP-ARAP_13.05.2021_00h00	3.80E-05	0.01833562	2.82E-21	3.38E-20	[5, 6]	Group12	6.967213	17	[ALDOC, ENPP1, GALM, GNPDA1, GOT2, HK1, HK2, IRS1, MTOR, NLN, PC, PCK2, PGM1, PGM2, PGM2L1, PGM5, SLC25A12]	76	Upregulation
FALSE	GO:0006612	protein targeting to membrane	GO_BiologicalProcess-EBI-UniProt-GOA-ACAP-ARAP_13.05.2021_00h00	7.60E-05	0.035926589	2.82E-21	3.38E-20	[5, 6, 7, 8]	Group12	6.866953	16	[ALDH3A2, ANK3, CHMP4B, ICMT, RPL13, RPL19, RPL24, RPL32, RPL36, RPL7A, RPS18, RPS2, RPS3A, RPS4X, RPS8, TCAF1]	62	Upregulation
FALSE	GO:0044242	cellular lipid catabolic process	GO_BiologicalProcess-EBI-UniProt-GOA-ACAP-ARAP_13.05.2021_00h00	5.42E-05	0.025866676	2.82E-21	3.38E-20	[4, 5]	Group12	6.772908	17	[ALDH3A2, CPT2, CYP2W1, ECI2, ETFDH, GCDH, HADH, IRS1, LRP1, MMUT, MTOR, OCRL, PCK2, PLA2G4A, PNPLA4, SGPL1, SMPD1]	65	Upregulation
FALSE	GO:0045296	cadherin binding	GO_MolecularFunction-EBI-UniProt-GOA-ACAP-ARAP_13.05.2021_00h00	1.79E-06	0.000923065	3.63E-07	2.90E-06	[4]	Group08	6.741573	24	[ABCF3, ANK3, ANLN, ARVCF, BZW2, CAPG, CAPZB, CHMP4B, CNN2, EGFR, JUP, LRRFIP1, MYO1B, PACSIN2, PDLIM1, PPL, RPL24, RPL7A, RPS2, SNX2, SPTBN2, TJP2, TNKS1BP1, VCL]	71	Upregulation

FALSE	GO:0016051	carbohydrate biosynthetic process	GO_BiologicalProcess-EBI-UniProt-GOA-ACAP-ARAP_13.05.2021_00h00	6.29E-05	0.029854787	2.82E-21	3.38E-20	[4]	Group12	6.6929135	17	[ALDOC, ENPP1, FUT2, GNPDA1, GOT2, GYG2, IRS1, MTOR, NLN, PC, PCK2, PGM1, PGM2, PGM2L1, PHGDH, SLC25A12, TKT]	88	Upregulation
FALSE	GO:0016054	organic acid catabolic process	GO_BiologicalProcess-EBI-UniProt-GOA-ACAP-ARAP_13.05.2021_00h00	2.74E-05	0.013296038	2.82E-21	3.38E-20	[4]	Group12	6.620209	19	[ALDH3A2, ARG1, BCAT2, BLMH, CPT2, CYP2W1, ECI2, ETFDH, GCDH, GNPDA1, GOT2, HADH, IRS1, MMUT, MTHFS, MTOR, PCK2, PHGDH, QPRT]	63	Upregulation
FALSE	GO:0034660	ncRNA metabolic process	GO_BiologicalProcess-EBI-UniProt-GOA-ACAP-ARAP_13.05.2021_00h00	4.11E-07	0.000214556	2.82E-21	3.38E-20	[6, 7]	Group12	5.924596	33	[AARSD1, AGO2, EEF1E1, ELP3, EPRS1, FARSA, FARSB, LARS1, MAK16, NOB1, OSGEPL1, PPA1, QRSL1, QTRT2, RPL13, RPL19, RPL24, RPL32, RPL36, RPL7A, RPS2, RPS8, RRP1, STAT3, TDRKH, TPRKB, TUT1, VARS1, WDR12, WDR55, YARS1, YARS2, ZCCHC8]	82	Upregulation
TRUE	GO:0042493	response to drug	GO_BiologicalProcess-EBI-UniProt-GOA-ACAP-ARAP_13.05.2021_00h00	8.07E-06	0.004027995	8.07E-06	4.84E-05	[3]	Group06	5.882353	26	[AK4, ALAD, ANTXR1, ARG1, ASS1, ATR, BCHE, BLMH, CAD, CYP2S1, CYP2W1, DPYSL2, EGFR, FZD1, GATA4, GGH, HADH, IGFBP2, MAP2K6, SMPD1, SRR, STAT3, TSPO, UCHL1, USP47, XPC]	NA	No change
TRUE	GO:0050839	cell adhesion molecule binding	GO_MolecularFunction-EBI-UniProt-GOA-ACAP-ARAP_13.05.2021_00h00	3.63E-07	0.000190226	3.63E-07	2.90E-06	[3]	Group08	5.8519793	34	[ABCF3, ANK3, ANLN, ARVCF, BCAT2, BZW2, CAPG, CAPZB, CCN2, CHMP4B, CNN2, EGFR, FGA, FGB, FGG, ILK, JUP, LAMA4, LAMC1, LRRFIP1, MYO1B, PACSIN2, PDLIM1, PLPP3, PPL, PTPRD, RPL24, RPL7A, RPS2, SNX2, SPTBN2, TJP2, TNKS1BP1, VCL]	NA	No change

TRUE	GO:0070161	anchoring junction	GO_CellularComponent-EBI-UniProt-GOA-ACAP-ARAP_13.05.2021_00h00	2.80E-10	1.51E-07	1.78E-12	1.78E-11	[3]	Group11	5.823068	52	[illegible], ANK2, ANK3, ARHGEF2, ARHGEF7, ARPC3, ARVCF, ATP1B1, CGNL1, CLIC4, CNN1, CNN2, CPNE3, CSPG4, DPP4, EGFR, FLRT3, FZD1, ILK, JUP, KRIT1, L1CAM, LRP1, MPDZ, PACSIN2, PDLIM1, PEAK1, PGM5, PLPP3, PPL, PROCR, PXDN, RAP2C, RPL19, RPL7A, RPS18, RPS2, RPS3A, RPS4X, RPS8, RSU1, SLC9A1, STRN, SVIL, TGM2, TJAP1, TJP2, TNKS1BP1,	NA	No change
TRUE	GO:0031983	vesicle lumen	GO_CellularComponent-EBI-UniProt-GOA-ACAP-ARAP_13.05.2021_00h00	8.84E-05	0.041284597	8.84E-05	0.000176808	[4, 5]	Group01	5.7220707	21	[ALAD, ALDOC, APOOL, ARG1, ARMC8, CNN2, CPPED1, CTSC, EGFR, FGA, FGB, FGG, GALNS, GGH, GNPDA1, JUP, PGM2, PSMD14, PYGL, VAT1, VCL]	NA	No change
FALSE	GO:0019693	ribose phosphate metabolic process	GO_BiologicalProcess-EBI-UniProt-GOA-ACAP-ARAP_13.05.2021_00h00	7.01E-06	0.003510661	2.82E-21	3.38E-20	[4, 5]	Group12	5.679513	28	[ACACA, ADK, AK4, ALDOC, ATP5MF, CAD, CTPS2, ENPP1, GCDH, GMPR2, GNPDA1, HK1, HK2, NME4, NUDT2, NUP98, PAPSS2, PGM1, PRPS1, PYGL, RPIA, SEH1L, SLC25A12, STAT3, SULT1E1, TJP2, TKT, TSPO]	NA	No change
FALSE	GO:0006401	RNA catabolic process	GO_BiologicalProcess-EBI-UniProt-GOA-ACAP-ARAP_13.05.2021_00h00	2.61E-05	0.012666107	2.82E-21	3.38E-20	[5, 6, 7]	Group12	5.6179776	25	[AGO2, ELAVL1, GSPT2, MTOR, PATL1, PSMB2, PSMC1, PSMC4, PSMD14, RPL13, RPL19, RPL24, RPL32, RPL36, RPL7A, RPS18, RPS2, RPS3A, RPS4X, RPS8, TNKS1BP1, TNPO1, TRAF2, TUT1, ZCCHC8]	80	Upregulation

FALSE	GO:0072594	establishment of protein localization to organelle	GO_BiologicalProcess-EBI-UniProt-GOA-ACAP-ARAP_13.05.2021_00h00	2.94E-06	0.001501122	2.82E-21	3.38E-20	[4, 5, 6]	Group12	5.605787	31	[ALDH3A2, CASP8, CHMP4B, ELAVL1, FBXO7, HK1, HK2, JUP, MACROH2A1, MACROH2A2, MAPK8, MAVS, NIPBL, NUP98, PPP1R10, RPL13, RPL19, RPL24, RPL32, RPL36, RPL7A, RPS18, RPS2, RPS3A, RPS4X, RPS8, STAT3, SUFU, TNPO1, TSPO, VPS41]	74	Upregulation
FALSE	GO:0015629	actin cytoskeleton	GO_CellularComponent-EBI-UniProt-GOA-ACAP-ARAP_13.05.2021_00h00	4.40E-06	0.002229565	1.78E-12	1.78E-11	[6]	Group11	5.597015	30	[ACACA, ANLN, ARPC3, CAPG, CAPZB, CGNL1, CLIC4, DPYSL3, EPHA3, FILIP1, ILK, IQGAP2, JUP, KRT19, LMOD1, MYL4, MYL9, MYO1B, PDLIM1, PEAK1, PGM5, PLS3, PXDN, SPTBN2, SVIL, TNKS1BP1, TNNT2, TPM3, TRIP6, VCL]	NA	No change
TRUE	GO:0010038	response to metal ion	GO_BiologicalProcess-EBI-UniProt-GOA-ACAP-ARAP_13.05.2021_00h00	6.24E-05	0.02968671	6.24E-05	0.000187101	[4]	Group00	5.5690074	23	[ALAD, ANK3, ARG1, ASS1, CASP8, CLIC4, CPNE3, EGFR, FGA, FGB, FGG, GCLC, GGH, GNPDA1, IGFBP2, LONP1, MAPK8, NEDD4L, NUDT1, SLC25A12, SMPD1, TNNT2, TSPO]	-61	Downregulation
FALSE	GO:0005911	cell-cell junction	GO_CellularComponent-EBI-UniProt-GOA-ACAP-ARAP_13.05.2021_00h00	7.33E-06	0.003665262	1.78E-12	1.78E-11	[4]	Group11	5.5555553	29	[ANK2, ANK3, ARHGEF2, ARVCF, ATP1B1, CGNL1, CLIC4, CNN2, DPP4, FLRT3, ILK, JUP, KRIT1, MPDZ, PACSIN2, PDLIM1, PGM5, PLPP3, PPL, PXDN, RAP2C, SLC9A1, STRN, TJAP1, TJP2, TNKS1BP1, TNPO1, UBA1, VCL]	NA	No change

FALSE	GO:0006082	organic acid metabolic process	GO_BiologicalProcess-EBI-UniProt-GOA-ACAP-ARAP_13.05.2021_00h00	1.14E-10	6.16E-08	2.82E-21	3.38E-20	[3]	Group12	5.3984575	63	[AARSD1, ACACA, ALDH18A1, ALDH3A2, ALDOC, ARG1, ASS1, BCAT2, BLMH, CAD, CKB, CPT2, CTPS2, CYP2S1, CYP2W1, ECI2, EEF1E1, ENPP1, EPHX1, EPRS1, ETFDH, FARSA, FARSB, FPGS, GCDH, GCLC, GNPDA1, GOT2, HADH, HK1, HK2, IRS1, LARS1, MGST3, MMUT, MRI1, MRPS36, MTHFS, MTOR, NUP98, PAPSS2, PC, PCK2, PGM1,	65	Upregulation
FALSE	GO:0043043	peptide biosynthetic process	GO_BiologicalProcess-EBI-UniProt-GOA-ACAP-ARAP_13.05.2021_00h00	2.63E-07	0.000138442	2.82E-21	3.38E-20	[5, 6]	Group12	5.3524804	41	[AARSD1, AGO2, DDX39B, EEF1A2, EEF1E1, EGFR, ELAVL1, EPRS1, ERAL1, FARSA, FARSB, GCLC, GSPT2, IGFBP5, KHDRBS1, LARS1, MRPS36, MRPS5, MTOR, MTRES1, PATL1, PPA1, QRSL1, RBM4, RPL13, RPL19, RPL22L1, RPL24, RPL32, RPL36, RPL7A, RPS18, RPS2, RPS3A, RPS4X, RPS8, STAT3, TNKS1BP1, VARS1, YARS1, YARS2]	66	Upregulation
FALSE	GO:0019752	carboxylic acid metabolic process	GO_BiologicalProcess-EBI-UniProt-GOA-ACAP-ARAP_13.05.2021_00h00	6.27E-10	3.36E-07	2.82E-21	3.38E-20	[5]	Group12	5.349048	59	[AARSD1, ACACA, ALDH18A1, ALDH3A2, ALDOC, ARG1, ASS1, BCAT2, BLMH, CAD, CKB, CPT2, CTPS2, CYP2S1, CYP2W1, ECI2, EEF1E1, EPHX1, EPRS1, ETFDH, FARSA, FARSB, FPGS, GCDH, GCLC, GNPDA1, GOT2, HADH, HK1, HK2, IRS1, LARS1, MGST3, MMUT, MRI1, MRPS36, MTHFS, MTOR, NUP98, PC, PCK2, PGM1, PHGDH, PLA2G4A,	68	Upregulation

FALSE	GO:0055086	nucleobase-containing small molecule metabolic process	GO_BiologicalProcess-EBI-UniProt-GOA-ACAP-ARAP_13.05.2021_00h00	1.65E-06	0.000853796	2.82E-21	3.38E-20	[3, 4, 5]	Group12	5.3333335	36	[ACACA, ADK, AK4, ALDOC, ASMTL, ATP5MF, CAD, CRMP1, CTPS2, DPYSL2, DPYSL3, DPYSL5, ENPP1, GCDH, GMPR2, GNPDA1, HK1, HK2, MACROD1, MTOR, NME4, NT5C3A, NUDT1, NUDT2, NUP98, PAPSS2, PGM1, PRPS1, PUDP, QPRT, SEH1L, SLC25A12, STAT3, SULT1E1, TJP2, TSPO]	NA	No change
FALSE	GO:0005975	carbohydrate metabolic process	GO_BiologicalProcess-EBI-UniProt-GOA-ACAP-ARAP_13.05.2021_00h00	2.14E-06	0.001098652	2.82E-21	3.38E-20	[3]	Group12	5.263158	36	[ALDH1B1, ALDOC, CHST2, ENPP1, FUT2, GALM, GCLC, GNPDA1, GOT2, GYG2, HK1, HK2, IRS1, MAN2B2, MAN2C1, MTOR, NLN, NUP98, OCRL, PC, PCK2, PGM1, PGM2, PGM2L1, PGM5, PHGDH, PHKB, PLA2G4A, PYGL, RPIA, SEH1L, SHPK, SLC25A12, STAT3, TKFC, TKT]	72	Upregulation
FALSE	GO:0046700	heterocycle catabolic process	GO_BiologicalProcess-EBI-UniProt-GOA-ACAP-ARAP_13.05.2021_00h00	4.23E-06	0.002152407	2.82E-21	3.38E-20	[4]	Group12	5.263158	34	[AGO2, CRMP1, DPYSL2, DPYSL3, DPYSL5, ELAVL1, ENPP1, GSPT2, MTHFS, MTOR, NT5C3A, NUDT1, PATL1, PSMB2, PSMC1, PSMC4, PSMD14, QPRT, RPL13, RPL19, RPL24, RPL32, RPL36, RPL7A, RPS18, RPS2, RPS3A, RPS4X, RPS8, TNKS1BP1, TNPO1, TRAF2, TUT1, ZCCHC8]	71	Upregulation

FALSE	GO:0043604	amide biosynthetic process	GO_BiologicalProcess-EBI-UniProt-GOA-ACAP-ARAP_13.05.2021_00h00	6.03E-08	3.19E-05	2.82E-21	3.38E-20	[5]	Group12	5.211726	48	[AARSD1, ACACA, AGO2, ARG1, ASS1, DDX39B, EEF1A2, EEF1E1, EGFR, ELAVL1, EPRS1, ERAL1, FARSA, FARSB, GCDH, GCLC, GSPT2, IGFBP5, KHDRBS1, LARS1, MRPS36, MRPS5, MTOR, MTRES1, NSMAF, PATL1, PPA1, QRSL1, RBM4, RPL13, RPL19, RPL22L1, RPL24, RPL32, RPL36, RPL7A, RPS18, RPS2, RPS3A, RPS4X, RPS8, SMPD1, SPTLC3,	69	Upregulation
FALSE	GO:0044270	cellular nitrogen compound catabolic process	GO_BiologicalProcess-EBI-UniProt-GOA-ACAP-ARAP_13.05.2021_00h00	5.67E-06	0.002858485	2.82E-21	3.38E-20	[4]	Group12	5.1829267	34	[AGO2, CRMP1, DPYSL2, DPYSL3, DPYSL5, ELAVL1, ENPP1, GSPT2, MTHFS, MTOR, NT5C3A, NUDT1, PATL1, PSMB2, PSMC1, PSMC4, PSMD14, QPRT, RPL13, RPL19, RPL24, RPL32, RPL36, RPL7A, RPS18, RPS2, RPS3A, RPS4X, RPS8, TNKS1BP1, TNPO1, TRAF2, TUT1, ZCCHC8]	71	Upregulation
FALSE	GO:1901361	organic cyclic compound catabolic process	GO_BiologicalProcess-EBI-UniProt-GOA-ACAP-ARAP_13.05.2021_00h00	9.85E-06	0.004907749	2.82E-21	3.38E-20	[4]	Group12	5.0143266	35	[AGO2, CRMP1, DPYSL2, DPYSL3, DPYSL5, ELAVL1, ENPP1, GSPT2, MTHFS, MTOR, NT5C3A, NUDT1, PATL1, PSMB2, PSMC1, PSMC4, PSMD14, QPRT, RPL13, RPL19, RPL24, RPL32, RPL36, RPL7A, RPS18, RPS2, RPS3A, RPS4X, RPS8, SULT1E1, TNKS1BP1, TNPO1, TRAF2, TUT1, ZCCHC8]	69	Upregulation

FALSE	GO:0009117	nucleotide metabolic process	GO_BiologicalProcess-EBI-UniProt-GOA-ACAP-ARAP_13.05.2021_00h00	7.63E-05	0.035999224	2.82E-21	3.38E-20	[5, 6, 7]	Group12	4.991394	29	[ACACA, ADK, AK4, ALDOC, ASMTL, ATP5MF, CAD, CTPS2, ENPP1, GCDH, GMPR2, GNPDA1, HK1, HK2, NME4, NT5C3A, NUDT2, NUP98, PAPSS2, PGM1, PRPS1, PUDP, QPRT, SEH1L, SLC25A12, STAT3, SULT1E1, TJP2, TSPO]	NA	No change
FALSE	GO:0006091	generation of precursor metabolites and energy	GO_BiologicalProcess-EBI-UniProt-GOA-ACAP-ARAP_13.05.2021_00h00	8.11E-05	0.038127536	2.82E-21	3.38E-20	[3]	Group12	4.957265	29	[AK4, ALDH1B1, ALDOC, ATP5MF, ENPP1, ETFDH, GNPDA1, GYG2, HK1, HK2, IRS1, LYRM7, MTOR, NQO2, NUP98, PGM1, PGM2, PGM2L1, PHGDH, PHKB, PYGL, RPIA, SEH1L, SHPK, SLC25A12, STAT3, SURF1, TKT, UQCR11]	NA	No change
FALSE	GO:0030036	actin cytoskeleton organization	GO_BiologicalProcess-EBI-UniProt-GOA-ACAP-ARAP_13.05.2021_00h00	1.10E-05	0.005469878	1.78E-12	1.78E-11	[3, 6]	Group11	4.8913045	36	[ANLN, ANTXR1, ARHGEF2, ARHGEF7, ARPC3, CAPG, CAPZB, CCN2, CGNL1, CNN1, CNN2, CYFIP2, DIAPH2, DPYSL3, EPHA3, ILK, IQGAP2, KRT19, LMOD1, LRP1, MTOR, MYO1B, PACSIN2, PDCL3, PDLIM1, PGM5, PHACTR4, PLEK2, PLS3, PXDN, SLC9A1, SPTBN2, SVIL, TNNT2, TPM3, TRIP10]	NA	No change

TRUE	GO:0005773	vacuole	GO_CellularComponent-EBI-UniProt-GOA-ACAP-ARAP_13.05.2021_00h00	5.21E-06	0.002631801	5.21E-06	3.65E-05	[3, 4, 5]	Group09	4.7619047	41	[ANK2, ANK3, ARG1, ARHGEF7, ASS1, ATG9A, ATP6V1F, BORCS6, CPNE3, CPPED1, CSPG4, CTSC, DPP4, EEF1A2, ENPP1, GALNS, GGH, ITFG2, KLHL22, LARS1, LPCAT1, LRP1, MAN2B2, MIOS, MTOR, NSF, OCIAD1, OCRL, PIP4K2C, PRCP, SEH1L, SLC17A5, SMPD1, SNX2, TRIP10, UBA1, ULK3, VAT1, VPS39, VPS41, WDR41]	NA	No change
FALSE	GO:0005764	lysosome	GO_CellularComponent-EBI-UniProt-GOA-ACAP-ARAP_13.05.2021_00h00	2.34E-05	0.011418846	5.21E-06	3.65E-05	[5, 6, 7]	Group09	4.7556143	36	[ANK2, ANK3, ARG1, ASS1, BORCS6, CPNE3, CPPED1, CSPG4, CTSC, DPP4, EEF1A2, ENPP1, GALNS, GGH, ITFG2, KLHL22, LARS1, LPCAT1, LRP1, MAN2B2, MIOS, MTOR, NSF, OCIAD1, OCRL, PRCP, SEH1L, SLC17A5, SMPD1, SNX2, TRIP10, UBA1, VAT1, VPS39, VPS41, WDR41]	NA	No change
FALSE	GO:0016071	mRNA metabolic process	GO_BiologicalProcess-EBI-UniProt-GOA-ACAP-ARAP_13.05.2021_00h00	4.41E-06	0.002229715	2.82E-21	3.38E-20	[6, 7]	Group12	4.7513814	43	[AGO2, DDX39B, ELAVL1, GSPT2, IK, KHDRBS1, MBNL2, MTOR, NUP98, PATL1, PPIL3, PRPF19, PRPF38B, PSMB2, PSMC1, PSMC4, PSMD14, RBM4, RPL13, RPL19, RPL24, RPL32, RPL36, RPL7A, RPS18, RPS2, RPS3A, RPS4X, RPS8, SF1, SFPQ, SNRNP40, SNRPD1, SNRPD2, STAT3, SUPT5H, TFCP2, THOC6, TNKS1BP1,	NA	No change

FALSE	GO:0003723	RNA binding	GO_MolecularFunction-EBI-UniProt-GOA-ACAP-ARAP_13.05.2021_00h00	8.40E-11	4.55E-08	2.82E-21	3.38E-20	[4]	Group12	4.6991405	82	[illegible] AGO2, ALDH18A1, ASS1, BAZ2A, CANX, CEBPZ, CPNE3, CRYZ, CYFIP2, DDX39B, DDX59, DEK, EEF1A2, ELAVL1, ELP3, EPRS1, ERAL1, FARSA, FARSB, GOT2, GSPT2, KHDRBS1, LONP1, LRP1, LRRFIP1, MAK16, MANF, MBNL2, MKI67, MRPS5, MTRES1, NOM1, NUDT1, NUP98, PATL1, PDCD4, PPIL4, PPP1R10,	NA	No change
TRUE	GO:0070062	extracellular exosome	GO_CellularComponent-EBI-UniProt-GOA-ACAP-ARAP_13.05.2021_00h00	1.34E-13	7.29E-11	1.34E-13	1.47E-12	[3, 4, 6]	Group02	4.6511626	106	[illegible] AGO2, ALAD, ALDOC, ALPL, ARPC3, ASS1, ATP1B1, ATP1B3, ATP6V1F, BCAT2, BLMH, BPTF, CAD, CANX, CAPG, CAPZB, CHMP4B, CKB, CLIC4, COL15A1, CPNE3, CPVL, CRB2, CRYZ, CSPG4, CTSC, CYFIP2, DIP2B, DPP4, DPYSL2, FGA, FGB, FGG, FSTL1, FUT2, GALM, GALNS, GGH, GNPDA1, GOT2, HEBP1, IGFBP2, IQGAP2, JUP, KRT19, LAMA4,	NA	No change
FALSE	GO:0006518	peptide metabolic process	GO_BiologicalProcess-EBI-UniProt-GOA-ACAP-ARAP_13.05.2021_00h00	1.18E-05	0.005847276	2.82E-21	3.38E-20	[4, 5]	Group12	4.574468	43	[illegible] AGO2, DDX39B, DNPEP, EEF1A2, EEF1E1, EGFR, ELAVL1, EPRS1, ERAL1, FARSA, FARSB, GCLC, GSPT2, IGFBP5, KHDRBS1, LARS1, MRPS36, MRPS5, MTOR, MTRES1, NLN, PATL1, PPA1, QRSL1, RBM4, RPL13, RPL19, RPL22L1, RPL24, RPL32, RPL36, RPL7A, RPS18, RPS2, RPS3A, RPS4X, RPS8, STAT3, TNKS1BP1, VARS1, YARS1, YARS2]	65	Upregulation

FALSE	GO:0043603	cellular amide metabolic process	GO_BiologicalProcess-EBI-UniProt-GOA-ACAP-ARAP_13.05.2021_00h00	2.74E-07	0.000143642	2.82E-21	3.38E-20	[4]		Group12	4.5709705	57	[AARSD1, ACACA, AGO2, ARG1, ASS1, DDX39B, DNPEP, EEF1A2, EEF1E1, EGFR, ELAVL1, EPRS1, ERAL1, FARSA, FARSB, FPGS, GCDH, GCLC, GNPDA1, GSPT2, IGFBP5, KHDRBS1, LARS1, MRPS36, MRPS5, MTHFS, MTOR, MTRES1, NLN, NSMAF, PATL1, PC, PLPP3, PPA1, QRSL1, RBM4, RPL13, RPL19, RPL22L1, RPL24, RPL32,	65	Upregulation
FALSE	GO:1901566	organonitrogen compound biosynthetic process	GO_BiologicalProcess-EBI-UniProt-GOA-ACAP-ARAP_13.05.2021_00h00	3.57E-10	1.92E-07	2.82E-21	3.38E-20	[4]		Group12	4.5212765	85	[AARSD1, ACACA, ADK, AGO2, AK4, ALAD, ALDH18A1, ALDH3A2, ARG1, ASS1, ATP5MF, BCAT2, CAD, CHST2, CKB, CSPG4, CTPS2, DDX39B, EEF1A2, EEF1E1, EGFR, ELAVL1, EPRS1, ERAL1, FARSA, FARSB, FPGS, FUT2, GCDH, GCLC, GMPR2, GOT2, GSPT2, IGFBP5, KHDRBS1, LARS1, LPCAT1, MGST3, MRI1, MRPS36, MRPS5, MTHFS,	64	Upregulation
FALSE	GO:1901575	organic substance catabolic process (eh not informative)	GO_BiologicalProcess-EBI-UniProt-GOA-ACAP-ARAP_13.05.2021_00h00	7.19E-12	3.91E-09	2.82E-21	3.38E-20	[3]		Group12	4.391892	104	[AGO2, AGTPBP1, ALAD, ALDH1B1, ALDH3A2, ALDOC, ARG1, ARMC8, BCAT2, BLMH, CASP8, CHMP4B, CPT2, CRMP1, CSPG4, CTSC, CYP2W1, DPYSL2, DPYSL3, DPYSL5, ECI2, EEF1A2, EGFR, ELAVL1, ENPP1, ETFDH, FAF1, FBXO7, FUT2, GALM, GALNS, GCDH, GCLC, GNPDA1, GOT2, GSPT2, HADH, HK1, HK2, IAH1, IRS1, KLHL22, LONP1,	62	Upregulation

FALSE	GO:0008092	cytoskeletal protein binding	GO_MolecularFunction-EBI-UniProt-GOA-ACAP-ARAP_13.05.2021_00h00	2.19E-05	0.010673971	1.78E-12	1.78E-11	[3]	Group11	4.322767	45	[AGTPBP1, ALDOC, ANK2, ANK3, ANLN, ANTXR1, ARHGEF2, ARHGEF7, ARPC3, CAMSAP1, CAPG, CAPZB, CETN2, CNN1, CNN2, CRMP1, DIAPH2, DIP2B, DLGAP5, DPYSL2, DPYSL3, DPYSL5, EGFR, IFT81, IQGAP2, KRIT1, LMOD1, MYL4, MYL9, MYO1B, PACSIN2, PDLIM1, PHACTR4, PLS3, PXDN, SPTBN2, SVIL, SYNE3, TNNT2,	NA	No change
TRUE	GO:0044248	cellular catabolic process	GO_BiologicalProcess-EBI-UniProt-GOA-ACAP-ARAP_13.05.2021_00h00	1.87E-11	1.02E-08	2.82E-21	3.38E-20	[3]	Group12	4.286292	106	[AGO2, AGTPBP1, ALAD, ALDH3A2, ARG1, ARMC8, ATG9A, BCAT2, BLMH, CASP8, CHMP4B, CPT2, CRMP1, CRYZ, CSPG4, CTSC, CYP2S1, CYP2W1, DNPEP, DPYSL2, DPYSL3, DPYSL5, ECI2, EEF1A2, ELAVL1, ENPP1, EPHX1, ETFDH, FAF1, FBXO7, GALNS, GATA4, GCDH, GCLC, GNPDA1, GOT2, GSPT2, HADH, HK2, IRS1, ITPR1,	61	Upregulation
FALSE	GO:0019637	organophosphate metabolic process	GO_BiologicalProcess-EBI-UniProt-GOA-ACAP-ARAP_13.05.2021_00h00	1.20E-05	0.005912007	2.82E-21	3.38E-20	[3, 4]	Group12	4.283217	49	[ACACA, ADK, AK4, ALDOC, ASMTL, ATP5MF, CAD, CKB, CPNE3, CTPS2, CYP2W1, ENPP1, GCDH, GMPR2, GNPDA1, HK1, HK2, IRS1, LPCAT1, MTMR12, NME4, NT5C3A, NUDT2, NUP98, OCRL, PAPSS2, PGM1, PGM2, PGM2L1, PHGDH, PIP4K2C, PLA2G4A, PLPP3, PRPS1, PUDP, PYGL, QPRT, RPIA, SEH1L, SHPK, SLC25A12, SMPD1,	61	Upregulation

FALSE	GO:0032559	adenyl ribonucleotide binding	GO_MolecularFunction-EBI-UniProt-GOA-ACAP-ARAP_13.05.2021_00h00	2.36E-07	0.000124524	9.78E-11	8.80E-10	[5, 6, 7]	Group10	4.264524	69	[AARSD1, ABCC9, ABCF3, ACACA, ADK, AK4, ALDH18A1, ANTXR1, ARHGEF7, ASS1, ATR, CAD, CDK16, CKB, CTPS2, DCLK1, DDX39B, DDX59, ECI2, EGFR, ENPP1, EPHA3, EPRS1, FARSA, FARSB, FPGS, GCDH, GCLC, HK1, HK2, ILK, LARS1, LONP1, LRRK1, MAP2K6, MAPK8, MASTL, MKI67, MTHFS, MTOR, MYO1B, NME4, NSF,	61	Upregulation
FALSE	GO:0005524	ATP binding	GO_MolecularFunction-EBI-UniProt-GOA-ACAP-ARAP_13.05.2021_00h00	3.86E-07	0.000201874	9.78E-11	8.80E-10	[5, 6, 7, 8]	Group10	4.2525773	66	[AARSD1, ABCC9, ABCF3, ACACA, ADK, AK4, ALDH18A1, ANTXR1, ARHGEF7, ASS1, ATR, CAD, CDK16, CKB, CTPS2, DCLK1, DDX39B, DDX59, EGFR, ENPP1, EPHA3, EPRS1, FARSA, FARSB, FPGS, GCLC, HK1, HK2, ILK, LARS1, LONP1, LRRK1, MAP2K6, MAPK8, MASTL, MKI67, MTHFS, MTOR, MYO1B, NME4, NSF, PAPSS2, PC,	62	Upregulation
TRUE	GO:0034622	cellular protein-containing complex assembly	GO_BiologicalProcess-EBI-UniProt-GOA-ACAP-ARAP_13.05.2021_00h00	1.10E-05	0.005469477	1.10E-05	5.52E-05	[6]	Group05	4.25	51	[AGO2, ANLN, ANTXR1, ARHGEF7, ARPC3, ATR, BAZ2A, CAMSAP1, CAPG, CAPZB, CENPV, CETN2, CHMP4B, CYFIP2, DDX39B, DLGAP5, ERAL1, FGA, FGB, FGG, HCCS, IQGAP2, LMOD1, LONP1, LYRM7, MACROH2A1, MACROH2A2, MTOR, NT5C3A, NUP98, OCRL, PRPF19, PSMB2, PSMC1, PSMC4, PSMD14,	-69	Downregulation

TRUE	GO:0005829	cytosol	GO_CellularComponent-EBI-UniProt-GOA-ACAP-ARAP_13.05.2021_00h00	7.67E-29	4.19E-26	7.67E-29	9.96E-28	[2, 3, 4]	Group03	4.212781	236	[ACACA, ADK, AFAP1L2, AGFG1, AGO2, AGTPBP1, ALAD, ALDH1B1, ALDH3A2, ALDOC, ALS2, ANK2, ANK3, ARAP3, ARG1, ARHGEF2, ARHGEF7, ARMC8, ARPC3, ASB3, ASMTL, ASS1, ATP6V1F, BABAM2, BAZ2A, BLMH, BORCS6, CAD, CAPZB, CASP8, CAVIN2, CCN2, CCND2, CD109, CDK16, CENPV, CEP78, CETN2, CHMP4B, CHST2, CIAO1, CKB, CLIC4,	NA	No change
FALSE	GO:0035639	purine ribonucleoside triphosphate binding	GO_MolecularFunction-EBI-UniProt-GOA-ACAP-ARAP_13.05.2021_00h00	6.47E-09	3.44E-06	9.78E-11	8.80E-10	[4]	Group10	4.1549954	89	[AARSD1, ABCC9, ABCF3, ACACA, ADK, AK4, ALDH18A1, ALS2, ANTXR1, ARHGEF2, ARHGEF7, ASS1, ATR, CAD, CDK16, CKB, CTPS2, DCLK1, DDX39B, DDX59, EEF1A2, EGFR, ENPP1, EPHA3, EPRS1, ERAL1, ETFDH, FARSA, FARSB, FPGS, GCLC, GSPT2, HK1, HK2, ILK, LARS1, LONP1, LRRK1, MAP2K6, MAPK8, MASTL, MKI67, MTHFS,	NA	No change
FALSE	GO:0032555	purine ribonucleotide binding	GO_MolecularFunction-EBI-UniProt-GOA-ACAP-ARAP_13.05.2021_00h00	4.52E-09	2.41E-06	9.78E-11	8.80E-10	[4, 5, 6]	Group10	4.132974	92	[AARSD1, ABCC9, ABCF3, ACACA, ADK, AK4, ALDH18A1, ALS2, ANTXR1, ARHGEF2, ARHGEF7, ASS1, ATR, CAD, CDK16, CKB, CTPS2, DCLK1, DDX39B, DDX59, ECI2, EEF1A2, EGFR, ENPP1, EPHA3, EPRS1, ERAL1, ETFDH, FARSA, FARSB, FPGS, GCDH, GCLC, GSPT2, HK1, HK2, ILK, LARS1, LONP1, LRRK1, MAP2K6, MAPK8, MASTL, MKI67, MTHFS,	NA	No change

TRUE	GO:0043168	anion binding	GO_MolecularFunction-EBI-UniProt-GOA-ACAP-ARAP_13.05.2021_00h00	3.73E-10	2.00E-07	9.78E-11	8.80E-10	[3]		Group10	4.0246553	111	[AARSD1, ABCC9, ABCF3, ACACA, ADK, AK4, ALDH18A1, ALS2, ANTXR1, ARAP3, ARHGEF2, ARHGEF7, ASS1, ATR, CAD, CAPG, CDK16, CKB, CRYZ, CTPS2, CTSC, CYP2W1, DCLK1, DDX39B, DDX59, EEF1A2, EGFR, ENPP1, EPHA3, EPRS1, ERAL1, ETFDH, FARSA, FARSB, FPGS, GCDH, GCLC, GOT2, GSPT2, HADH, HK1, HK2, ILK, IQGAP2, ITPR1, ITPR2,	NA	No change

**Suppl Table S10**. Comparison of xlinked genes in iPSCs and iVSMCS												
CellType	Gene	LogFC	Pval	Chromosome	Related to Vascular function	Related to vascular disease	related to vascular disease in another cell type	Up or Down	Protective vs Risk?	Disease?	PMID	source
iPSC	PIR	-0.71515	0.000459682	X-linked								
iPSC	CASK	-0.34341	0.027179849	X-linked								
iPSC	GYG2	-1.28348	1.36E-08	X-linked								
iPSC	DIAPH2	-1.13907	0.003010035	X-linked								
iPSC	PSMD10	-1.01372	0.009068166	X-linked								
iPSC	AIFM1	-0.28219	0.004570766	X-linked								
iPSC	ZBED1	1.178606	0.000878292	X-linked								
iPSC	HPRT1	-0.38062	0.015338594	X-linked								
iPSC	ARAF	-0.38501	0.033848125	X-linked								
iPSC	LAMP2	-0.36656	0.031955202	X-linked								
iPSC	PLS3	-0.44318	0.01017973	X-linked								
iPSC	UBA1	-0.57339	1.59E-05	X-linked								
iPSC	ANOS1	-1.27034	2.31E-05	X-linked								
iPSC	CHM	-0.83022	0.019417605	X-linked								
iPSC	KDM5C	-0.74932	0.031633893	X-linked								
iPSC	PNPLA4	-0.77025	0.000280674	X-linked								
iPSC	PHKA2	-0.8511	0.001147581	X-linked								
iPSC	USP11	-0.34479	0.043952536	X-linked								
iPSC	XIAP	-0.33552	0.001925248	X-linked								
iPSC	PUDP	-1.27151	2.98E-07	X-linked								
iPSC	SEPTIN6	0.498935	0.012503382	X-linked								
iPSC	EBP	0.688767	0.000530591	X-linked								
iPSC	RBBP7	-1.05087	1.47E-07	X-linked								
iPSC	APOOL	0.578963	0.001376666	X-linked								
iPSC	HUWE1	-0.21311	0.007300765	X-linked								
iPSC	NLGN4X	-1.07229	0.000499559	X-linked								
iPSC	IGSF1	-1.17164	0.007284893	X-linked								
iPSC	MOSPD2	-0.60774	0.004147623	X-linked								
iPSC	SLC7A3	0.388945	0.04857084	X-linked								
iPSC	USP9X	-0.25958	0.016662079	X-linked								
iPSC	TSR2	-0.74635	0.024883858	X-linked								
iPSC	SYAP1	-0.90736	0.000337024	X-linked								
iPSC	SLC9A7	-1.36428	0.000769281	X-linked								
iPSC	CTPS2	-0.79268	0.000408208	X-linked								
iPSC	GEMIN8	-0.59009	0.005457888	X-linked								
iPSC	NDUFB11	-0.81033	0.00804612	X-linked								
iPSC	FTSJ1	-0.52733	0.003401159	X-linked								
iPSC	RAP2C	-0.71431	5.62E-05	X-linked								
iPSC	PCYT1B	-0.47145	0.030882827	X-linked								
iVSMC	GYG2	-0.4331	0.029610169	X-linked	no	yes	yes		Risk	Atherosclerosis, Coronary Artery Diseasey	32541024	https://pubmed.ncbi.nlm.nih.gov/32541024/
iVSMC	DIAPH2	-1.55622	0.000729539	X-linked	no	no	no					
iVSMC	RP2	0.450243	0.035693233	X-linked	no	no	no					
iVSMC	ASMTL	0.810314	0.004344337	X-linked	no	no	yes	Down	Risk	Turner Syndrom, Aortic dissection?	22707402	https://academic.oup.com/edrv/article/33/5/677/2354778
iVSMC	PLS3	-0.61357	0.000686906	X-linked	no	no	yes	Mutation	Risk	Aortic Dissection	31694393	https://pubmed.ncbi.nlm.nih.gov/31694393/
iVSMC	UBA1	-0.57909	1.86E-05	X-linked	no	no	no					
iVSMC	SMARCA1	-0.28461	0.047519799	X-linked	no	no	no					
iVSMC	L1CAM	1.18438	0.00066392	X-linked	yes	no	no				30283356	https://www.ncbi.nlm.nih.gov/pmc/articles/PMC6157320/
iVSMC	CETN2	-0.39297	0.014169143	X-linked	no	no	no					
iVSMC	PNPLA4	-0.6131	0.004319432	X-linked	no	no	yes	Up	Protective	Atherosclerosis	34017580	https://www.ncbi.nlm.nih.gov/pmc/articles/PMC8129354/

iVSMC	USP11	-0.48339	0.007742401	X-linked	no	no	yes			Atherosclerosis	37287426	https://www.ncbi.nlm.nih.gov/pmc/articles/PMC10375200/
iVSMC	HCCS	-0.69638	0.001393364	X-linked	no	no	no					
iVSMC	PRPS1	-0.55846	0.032112605	X-linked	no	yes	no			Atherosclerosis	36073366	https://www.ahajournals.org/doi/10.1161/CIRCULATIONAHA.121.058901
iVSMC	VBP1	-0.27658	0.049282234	X-linked	no	no	yes	Up	Risk	Atherosclerosis	27374120	https://maayanlab.cloud/Harmonizome/gene_set/Atherosclerosis/CTD+Gene-Disease+Associations
iVSMC	RPS4X	-0.50842	0.000902997	X-linked	no	no	yes	Up	Risk	Atherosclerosis	37889192	https://www.biorxiv.org/content/10.1101/2023.02.09.527800v1.full.pdf
iVSMC	CDK16	-0.67091	0.004244774	X-linked	no	no	yes	Down	Protective	Atherosclerosis	35949126	https://www.ncbi.nlm.nih.gov/pmc/articles/PMC9682560/
iVSMC	OCRL	-0.54092	0.043205977	X-linked	maybe	no	no				33738288	https://www.ncbi.nlm.nih.gov/pmc/articles/PMC7960786/
iVSMC	PUDP	-0.89762	0.000190731	X-linked	no	no	yes	Up	Risk	Atherosclerosis	37889192	https://www.biorxiv.org/content/10.1101/2023.02.09.527800v1.full.pdf
iVSMC	**SEPTIN6**	0.946685	1.52E-05	X-linked	no	no	no					
iVSMC	RBBP7	-0.55877	0.002331752	X-linked	no	no	yes	Up	Risk	Peripheral Artery Disease	37154037	https://www.ahajournals.org/doi/10.1161/CIRCRESAHA.122.322325
iVSMC	LRCH2	-0.68797	0.041898157	X-linked	no	no	no					
iVSMC	APOOL	0.406889	0.023347209	X-linked	no	no	no					
iVSMC	CFAP47	0.784783	0.018160328	X-linked	no	no	no					
iVSMC	GSPT2	-0.56477	0.030760832	X-linked	no	no	no					
iVSMC	PCDH19	-1.40581	0.002444432	X-linked	no	no	no					
iVSMC	SYAP1	-1.01159	6.08E-05	X-linked	no	no	yes	Mutation	Risk	Aortic Dissection; Coronary Artery Disease	37813462; 34121455	https://jmg.bmj.com/content/61/3/232

iVSMC	MXRA5	-0.6801	0.030619684	X-linked	no	no	yes	Up	Risk	Atherosclerosis	38292008	https://www.ncbi.nlm.nih.gov/pmc/articles/PMC10825564/
iVSMC	CTPS2	-0.52107	0.017929885	X-linked	no	no	yes	Up	Risk	Coronary Atherosclerosis	38454353	https://www.ncbi.nlm.nih.gov/pmc/articles/PMC10921789/
iVSMC	STK26	-0.96101	1.13E-05	X-linked	no	no	yes			Myocardial Infarction	36036671	https://www.ncbi.nlm.nih.gov/pmc/articles/PMC10129261/
iVSMC	RAP2C	-0.55334	0.001534831	X-linked	no	no	yes	Down	Risk	Atherosclerosis	33921168	https://www.ncbi.nlm.nih.gov/pmc/articles/PMC8071517/
